# Global, regional, and national temporal trends in prevalence, deaths and disability-adjusted life years for chronic pulmonary disease, 1990–2021: an age-period-cohort analysis based on the global burden of disease study 2021

**DOI:** 10.3389/fmed.2025.1554442

**Published:** 2025-03-04

**Authors:** Wubing Cao, Jun Zheng, Qun Li, Dabin Guo, Xianzhi Fan, Guoning Zhu, Xiao Yuan

**Affiliations:** The People’s Hospital of Longyou County, Quzhou, China

**Keywords:** COPD—chronic obstructive pulmonary disease, GBD (global burden disease), age-period-cohort (APC) analysis, SDI: Sociodemographic index, pulmanory disease

## Abstract

**Background:**

Chronic obstructive pulmonary disease (COPD) is a leading cause of morbidity and mortality globally, with significant disparities in disease burden across countries and socioeconomic regions. Despite advancements in public health, the global burden of COPD remains substantial, particularly in low- and middle-income countries. This study aims to provide a comprehensive analysis of global, regional, and national trends in COPD-related prevalence, deaths, and disability-adjusted life years (DALYs) from 1990 to 2021 using an age-period-cohort (APC) model.

**Methods:**

Data from the Global Burden of Disease Study 2021 were analyzed for 204 countries and territories, stratified by five Sociodemographic Index (SDI) levels. An APC model was employed to assess the temporal effects of age, time periods, and birth cohorts on COPD burden. Trends in prevalence, deaths, and DALYs were evaluated through metrics such as Net Drift, Local Drift, and risk ratios.

**Results:**

Globally, from 1990 to 2021, the age-standardized rates of COPD demonstrated a decline of −1.46% (95% UI: −3.36 to 0.39%) in prevalence, −37.12% (95% UI: −43.37% to −27.68%) in deaths, and −36.98% (95% UI: −42.37% to −28.54%) in DALYs. After adjusting for age and cohort effects, the annual changes were −0.35% (95% UI: −0.39% to −0.32%) in prevalence, −3.87% (95% UI: −4.00% to −3.74%) in deaths, and − 2.95% (95% UI: −3.02% to −2.89%) in DALYs. Notably, in middle, low-middle, and low SDI regions, the age-standardized prevalence rates in 2021 showed an increase compared to 1990, with respective changes of 4.03% (95% UI: 2.00–5.89%), 0.13% (95% UI: −2.90 to 2.84%), and 6.71% (95% UI: 4.25–8.91%). However, age-standardized deaths and DALYs significantly decreased across all five SDI regions. From an age effect perspective, globally, over 50% of COPD prevalence is concentrated among individuals aged 65 years and older, particularly in middle, low-middle, and high-middle SDI regions. COPD-related deaths and DALYs have shown a declining trend across all age groups. Globally, the period effect indicates that earlier periods were associated with a higher burden of disease, while cohort effects highlight that birth cohorts around 1920 had a particularly pronounced impact on the COPD burden. Both period and cohort effects exhibited notable heterogeneity across different SDI regions and countries,

**Conclusion:**

The prevalence of COPD remains concerning. Compared to 1990, the global prevalence of COPD in 2021 showed a stable or slightly increasing trend, with over half of the countries experiencing an annual increase in prevalence during the 1990–2021 period. Global mortality and DALYs associated with COPD showed a notable decline in 2021 compared to 1990. However, this trend exhibited significant heterogeneity across countries and regions, likely linked to differences in socioeconomic development. Countries in the high-middle and middle SDI regions were found to be more affected by period effects. From an age effect perspective, population aging has undoubtedly exacerbated the COPD burden. Regarding cohort effects, earlier birth cohorts demonstrated a stronger contribution to the increasing disease burden. While Higher levels of socioeconomic development can mitigate the adverse effects associated with earlier birth cohorts.

## Introduction

Chronic obstructive pulmonary disease (COPD) is a major public health challenge globally, marked by irreversible airflow limitation and a progressive decline in lung function. As one of the leading causes of morbidity and mortality worldwide, COPD was responsible for approximately 3.3 million deaths in 2019, making it the third leading cause of death globally (1).

Beyond its impact on mortality, COPD imposes a significant burden on global health systems through its associated disability-adjusted life years (DALYs), primarily driven by years of life lost (YLLs) and years lived with disability (YLDs) (2).

Economic development is a key factor influencing the burden of chronic obstructive pulmonary disease, potentially reflecting the level of investment in healthcare systems (3). The Global Burden of Disease (GBD) study offers a unique platform for analyzing temporal and spatial trends in COPD prevalence, mortality, and DALYs across 204 countries and territories. Utilizing standardized methodologies, GBD also assesses the Socio-Demographic Index (SDI) for each country and classifies them into five SDI tiers. This allows researchers to explore how socioeconomic factors, demographic shifts, and evolving risk factor exposures contribute to the global burden of COPD, while accounting for the variations in healthcare development and infrastructure across different SDI levels (4–6).

The evolving landscape of COPD burden necessitates analytical approaches that transcend conventional age-standardized metrics. While the GBD studies have meticulously documented temporal trends (3), their traditional methodology obscures three interwoven temporal drivers: the biological imprint of population aging, generational shifts in environmental exposures, and period-specific policy impacts. This limitation is particularly consequential for COPD—a disease where more than half of disability-adjusted life years (DALYs) accumulate after age 65 (7), which means we cannot determine whether this is due to aging itself or an increase in early-life risk exposures among these populations. The age-period-cohort (APC) model addresses this complexity by simultaneously quantifying how aging modulates individual susceptibility, how macroeconomic development reshapes population-wide risks through healthcare access and environmental regulations, and how historical exposures leave enduring signatures across generations (8). Our application of APC modeling to 204 countries will reveal critical synergies by mapping these SDI-stratified temporal dynamics, we identify actionable leverage points—for instance, targeting a certain birth cohort in industrializing regions with tailored smoking cessation programs before irreversible lung function decline. This approach transcends descriptive epidemiology, helping guide the formulation of healthcare policies and the allocation of healthcare expenditures to reduce the global burden of COPD and alleviate its impact on vulnerable populations.

## Methods

### Overview of GBD

The Global Burden of Disease (GBD) 2021, generated by the Institute for Health Metrics and Evaluation (IHME), provides comprehensive and up-to-date estimates on the health loss associated with 371 diseases, injuries, impairments, and 88 risk factors by age and sex across 204 countries and territories. The methodologies applied in GBD 2021, including data extraction, modeling processes, and statistical techniques, have been thoroughly detailed in previous publications (9–11). Detailed estimates for both fatal and non-fatal outcomes, including COPD prevalence, deaths, and disability-adjusted life years (DALYs), are publicly available through the GBD 2021 Results Tool and the Global Health Data Exchange (GHDx).^1^ The GBD study complies with the Guidelines for Accurate and Transparent Health Estimate Reporting (GATHER), ensuring rigorous standards in the transparency and accuracy of its data and findings (12).

### Study design and data sources

This study utilized data from the Global Burden of Disease (GBD) 2021 study, which provides comprehensive estimates for prevalence, deaths, and disability-adjusted life years (DALYs) for chronic obstructive pulmonary disease (COPD) (ICD 10 code: J41-J44.9) across 204 countries and territories from 1990 to 2021. The data were extracted from the GBD Results Tool (see text footnote 1).

### Sociodemographic index classification

Countries and territories were grouped into five SDI regions (low, low-middle, middle, high-middle, high) based on composite indicators of income per capita, educational attainment, and fertility rates. This stratification enabled comparison of disease burden trends across varying levels of socioeconomic development (13).

### Age-period-cohort analysis

An Age-Period-Cohort model was applied to analyze the temporal trends in COPD burden, separating the influences of age, time periods, and birth cohorts. The analysis utilized the APC Web Tool,^2^ developed by the National Cancer Institute, which implements advanced computational methods for age-specific rates over time (14). The APC Web Tool automatically addresses collinearity among age, period, and cohort variables using the intrinsic estimator method. The metric Net Drift, Local Drift, Period Effects, Cohort Effects and age effect were derived from the APC model.

### Uncertainty and statistical considerations

The estimates of prevalence, deaths, and DALYs are presented with 95% uncertainty intervals (UIs), defined by the 2.5th and 97.5th values of the 1,000 ordered estimates generated by the GBD algorithm. These intervals reflect the inherent uncertainty in epidemiological estimates and provide a range for interpretation.

## Results

### Trends in COPD burden from1990 to 2021: prevalence, DALYs and mortality

Table 1 presents absolute values for DALYs, deaths, and prevalence, along with their age-standardized rates (ASRs), percentage changes from 1990 to 2021, and Net Drift values estimated using the APC model. The Net Drift metric represents the annual percentage change adjusted for age, period, and cohort effects. Between 1990 and 2021, as the global population expanded, absolute numbers of COPD-related DALYs, deaths, and prevalence rose significantly. Specifically, DALYs increased from 56.38 million (95% UI: 50.36–61.3) to 78.63 million (95% UI: 71.36–85.76), deaths grew from 2.48 million (95% UI: 2.19–2.7) to 3.62 million (95% UI: 3.21–4.0), and prevalence surged from 98.64 million (95% UI: 84.38–113.04) to 209.32 million (95% UI: 179.9–239.36). Globally, despite increases in absolute numbers, the burden of COPD, as measured by age-standardized DALY, death, and prevalence rates, exhibited consistent declines. Net Drift analyses revealed year-on-year reductions in COPD-related DALY, death, and prevalence rates when accounting for age and cohort effects.

**Table 1:** Trend of COPD related deaths, prevalence and DALYs from 1990 to 2021 by SDI quintiles.

	Global		High SDI		High-middle SDI		Middle SDI		Middle-Low SDI		Low SDI	
	1990	2021	1990	2021	1990	2021	1990	2021	1990	2021	1990	2021
No. (*10^6)												
Population	3074.03 (3013.3 to 3140.23)	5250.06 (5099.17 to 5412.24)	627.66(612.22 to 642.96)	858.55(834.81 to 882.73)	693.18(670.14 to 717.34)	999.56(957.89 to 1043.18)	958.21(925.71 to 988.96)	1698.31(1630.1 to 1765.58)	570.29(549.76 to 590.04)	1156.27(1092.99 to 1221.89)	221.7(215.58 to 227.89)	533.1(508.27 to 558.49)
DALYs	56.38(50.36 to 61.3)	78.63(71.36 to 85.76)	6.56(6.11 to 6.93)	9.92(9.02 to 10.63)	14.04(12.51 to 15.4)	13.19(11.57 to 14.9)	21.26(18.61 to 23.53)	26.35(23.2 to 29.79)	11.03(8.84 to 12.8)	22.52(19.97 to 25.14)	3.47(2.77 to 4.06)	6.6(5.75 to 7.53)
Deaths	2.48(2.19 to 2.7)	3.62(3.21 to 4)	0.29(0.26 to 0.3)	0.44(0.39 to 0.47)	0.67(0.59 to 0.74)	0.66(0.57 to 0.76)	0.95(0.82 to 1.05)	1.26(1.08 to 1.45)	0.44(0.35 to 0.52)	0.99(0.87 to 1.11)	0.13(0.1 to 0.16)	0.26(0.23 to 0.3)
Prevalence	98.64(84.38 to 113.04)	209.32(179.9 to 239.36)	28.11(24.15 to 32.17)	50.96(45.35 to 56.69)	24.24(20.63 to 28)	45(38.18 to 52.12)	25.27(21.41 to 29.12)	62.99(52.73 to 73.47)	15.84(13.54 to 18)	37.94(32.63 to 43.19)	5.09(4.33 to 5.81)	12.27(10.49 to 14.08)
Age-standardized rate (per 100,000)												
Deaths	71.92(64.47 to 77.53)	45.22(40.61 to 49.7)	25.53(23.71 to 26.5)	19.44(17.26 to 20.66)	79.53(70.61 to 86.61)	35.91(30.78 to 40.69)	92.07(74.17 to 107.46)	84.76(75.8 to 93.78)	123.89(109.19 to 134.8)	57.45(49.59 to 65.43)	77.67(61.91 to 91.44)	70.7(63.35 to 79.76)
DALYs	1492.64(1342.46 to 1609.3)	940.66(871.48 to 1014.59)	589.8(557.84 to 616.39)	471.22(437.45 to 498.84)	1511.32(1365.74 to 1635.67)	691.14(621.83 to 772.74)	1963.19(1602.24 to 2252.75)	1707.9(1558.88 to 1865.11)	2332.91(2063.49 to 2546.31)	1076.67(963.62 to 1201.24)	1673.81(1373.37 to 1936.99)	1457.94(1318.76 to 1617.05)
Prevalence	2550.02(2318.34 to 2806.32)	2512.86(2293.93 to 2748.52)	2602.27(2359.28 to 2847.55)	2527.66(2364.4 to 2701.8)	2523.42(2287.88 to 2794.38)	2385.34(2149.82 to 2648.74)	2621.01(2378.69 to 2867.91)	2726.76(2478.22 to 2,979)	2459.97(2217.17 to 2717.88)	2463.19(2208.84 to 2735.96)	2190.49(1969.32 to 2409.53)	2337.52(2119.39 to 2567.85)
Percentage change, 1990–2021 (%)												
Deaths	−37.12(−43.37 to −27.68)		−23.85(−27.38 to −20.63)		−54.85(−61.65 to −46.85)		−7.93(−20.33 to 16.03)		−53.62(−60.31 to −44.06)		−8.96(−20.17 to 11.87)	
DALYs	−36.98(−42.37 to −28.54)		−20.1(−22.48 to −17.87)		−54.27(−60.12 to −47.24)		−13(−23.54 to 7.28)		−53.85(−59.73 to −45.6)		−12.9(−22.5 to 4.71)	
Prevalence	−1.46(−3.36 to 0.39)		−2.87(−5.71 to 0.64)		−5.47(−7.86 to −3.24)		4.03(2 to 5.89)		0.13(−2.9 to 2.84)		6.71(4.25 to 8.91)	
APC model estimate, Net drift (% per year)												
DALYs	−2.95(−3.02 to −2.89)		−0.63(−0.7 to −0.56)		−0.71(−0.79 to −0.63)		−2.71(−2.77 to −2.66)		−0.74(−0.84 to −0.65)		−1.66(−1.71 to −1.62)	
Deaths	−3.87(−4 to −3.74)		−0.82(−1 to −0.65)		−0.96(−1.12 to −0.8)		−2.71(−2.77 to −2.66)		−1.01(−1.2 to −0.82)		−2.18(−2.25 to −2.1)	
Prevalence	−0.35(−0.39 to −0.32)		−0.82(−1 to −0.65)		0(−0.01 to 0.01)		−0.18(−0.21 to −0.16)		0.09(0.07 to 0.11)		−0.17(−0.18 to −0.15)	

DALYs, disability-adjusted life years.

From an absolute value perspective, increases in the numbers of deaths, DALYs, and prevalence were driven by population growth. Compared to 1990, the age-standardized rates of deaths and DALYs decreased across all five SDI regions by 2021. Net Drift analyses further confirmed that, after adjusting for age and cohort effects, COPD-related deaths and DALYs consistently declined annually across all SDI regions.

However, trends in prevalence were less favorable. Except for high-SDI and high-middle-SDI regions, other SDI regions experienced increases in age-standardized prevalence rates between 1990 and 2021. The Net Drift values indicated that while most regions showed decreasing trends, the prevalence in middle-low SDI regions exhibited a gradual year-on-year increase (Net Drift: 0.09, 95% UI: 0.07–0.11). This trend contrasts with the analysis based solely on age-standardized prevalence rates, highlighting differences in methodological insights.

**Figure 1:**
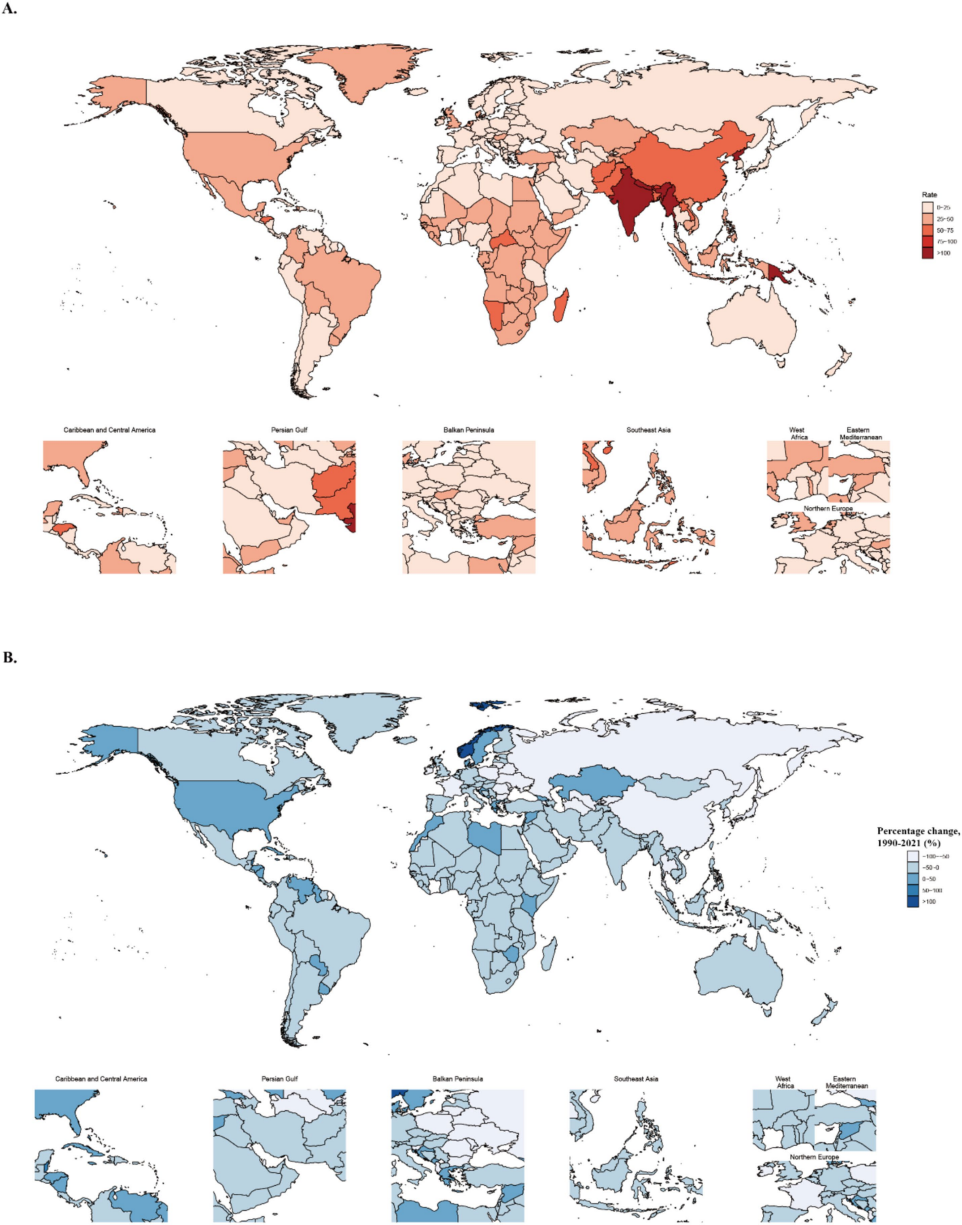
Global disease burden and percentage change of COPD-related deaths. **(A)** Global distribution of COPD-related age-standardized deaths rates in 2021. **(B)** Global distribution of percentage changes in COPD-related age-standardized mortality rates from 1990 to 2021.

At the national level, Figure 1, Supplementary Figures S1–S7, and Supplementary Table S1 summarize the burden of COPD from 1990 to 2021. Globally, the average age-standardized prevalence rate was 2075.72 per 100,000 population (95% UI: 2000.15–2150.30). Notably, three countries—Turkey, the United Kingdom, and the United States—exceeded 1.5 times the global average. Morocco demonstrated the largest relative increase in age-standardized prevalence rate, rising by 35.32% (95% UI: 24.84–45.54) during the study period. APC modeling estimated increasing prevalence trends in 138 countries, decreasing trends in 24 countries, and relatively stable trends in 42 countries. In 2021, the highest COPD-related DALY rates were observed in Papua New Guinea (3004.36, 95% UI: 2404.29–3732.82), Nepal (2836.01, 95% UI: 2275.31–3485.04), India (2171.16, 95% UI: 1953.69–2422.39), the Democratic People’s Republic of Korea (1968.2, 95% UI: 1500.48–2612.25), and Myanmar (1958.92, 95% UI: 1604.09–2,354). The global average age-standardized DALY rate in 2021 declined to 1500.78 per 100,000 (95% UI: 1400.25–1600.50). From 1990 to 2021, Saint Vincent and the Grenadines exhibited the largest relative increase in age-standardized DALY rate (60.06, 95% UI: 42.14–79.78), accompanied by similar increases in 33 other countries. In 2021, the global average age-standardized death rate for COPD was 29.71 per 100,000 (95% UI: 27.50–32.50). Seventeen countries, including India, China, and the Democratic People’s Republic of Korea, reported death rates exceeding twice the global average. Norway exhibited the largest increase in age-standardized death rates, with a rise of 100.73% (95% UI: 87.23–110.72), while most countries demonstrated declines.

**Figure 2:**
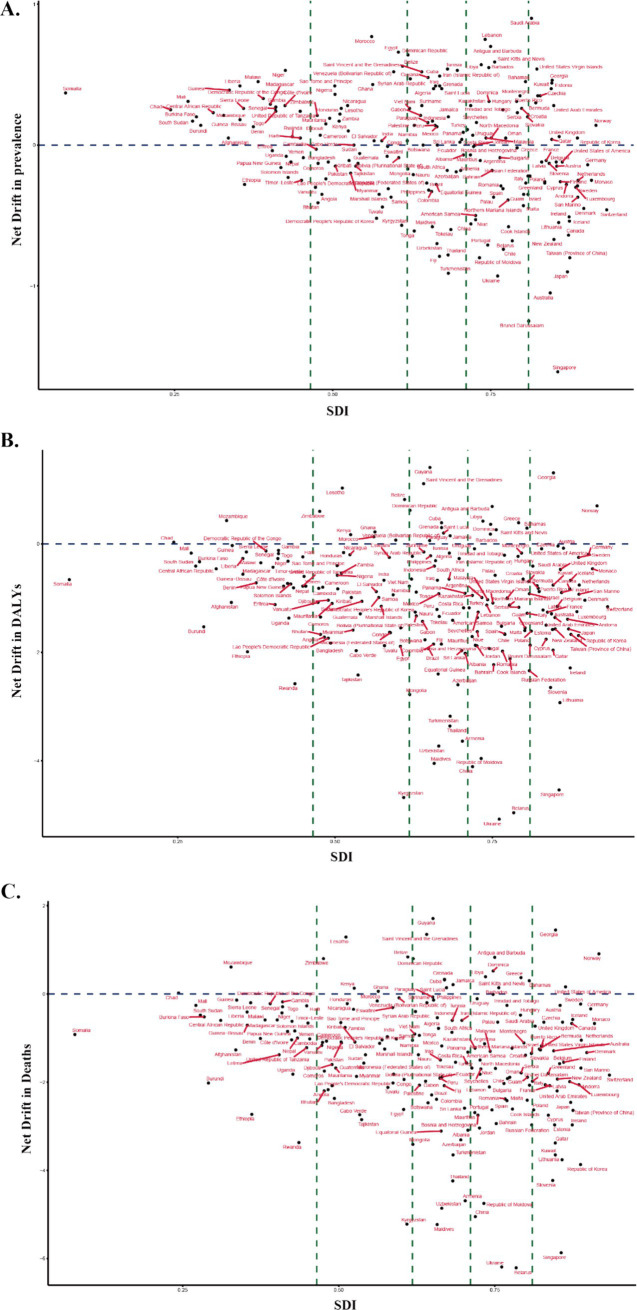
Distribution of Net Drift in COPD burden across SDI at the national level. **(A)** Net Drift in prevalence vs. SDI. **(B)** Net Drift in DALYs vs. SDI. **(C)** Net Drift in mortality vs. SDI. The blue dashed line at Y = 0 indicates countries with an average annual increase in burden from 1990 to 2021 if positioned above the line. The green dashed lines at SDI values of 0.4658, 0.6188, 0.7119, and 0.8102 divide the GBD-defined SDI categories into low, middle-low, middle, high-middle, and high SDI regions.

Using the APC model, national-level Net Drift values were computed and visualized against SDI in Figure 2, focusing on countries where Net Drift was greater than zero. This indicates a year-on-year increase in disease burden, even after adjusting for age and cohort effects. Over half of the countries exhibited positive Net Drift values for prevalence. However, our SDI-stratified analysis revealed that only the middle-low SDI region demonstrated a positive Net Drift overall. This discrepancy may be attributed to the disproportionate influence of several populous countries within this region (e.g., India, Indonesia, Egypt), where persistently elevated Net Drift values skewed the regional trend. Regarding deaths and DALYs, positive Net Drift values were primarily observed in countries and regions with high-middle, middle, or middle-low SDI.

### Temporal trends in COPD prevalence, DALYs, and deaths by age

**Figure 3:**
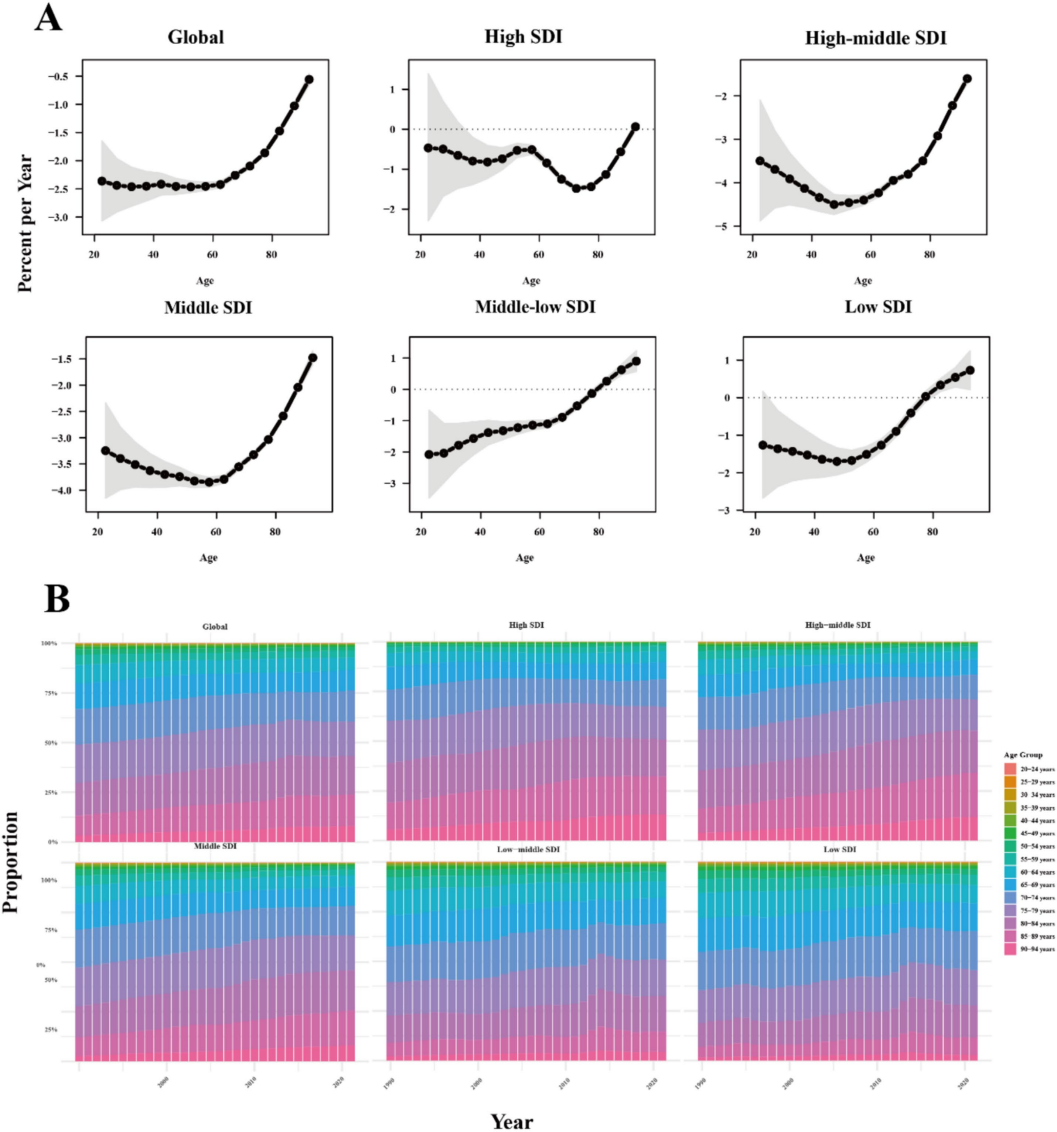
Age stratification analysis of COPD-related deaths. **(A)** The age local drift of COPD related deaths across SDI quintiles. **(B)** Age distribution of COPD related deaths from 1990 to 2021 across SDI quintiles. SDI: Sociodemographic Index.

The annual percentage change (local drift) in COPD prevalence, DALYs, and deaths for each age group, calculated from the APC model, is presented in Figure 3A, Supplementary Figures S8, S9A, and Supplementary Table S2 for Global and five SDI regions, and Supplementary Table S3 for 204 countries and territories. Temporal changes in age distribution of COPD were illustrated in Figure 3B and Supplementary Figures S8, S9B. Globally, COPD prevalence demonstrated declining trends in young and middle-aged groups, particularly from the 45–50 years group (−0.39, 95% CI: −0.42 to −0.36%) to the 60–65 years group (−0.08, 95% CI: −0.1 to −0.06%). The decline was more pronounced in the 45–50 years group and gradually mitigated with increasing age. In contrast, prevalence trends flattened in the 70–75 years group (0, 95% CI: −0.02 to 0.02%) and transitioned to an increasing trend from the 75–80 years group onwards, peaking in older age groups at 90–95 years (0.41, 95% CI: 0.33–0.49%). A gradual shift in the prevalence burden from younger to older age groups was observed globally, with over 50% of prevalence concentrated among individuals older than 65 years, particularly in middle, middle-low, and high-middle SDI regions.

COPD-related DALYs has similar trends observed in deaths. Globally, COPD-related deaths and DALYs have been on a declining trajectory across all age groups. However, in middle-low and low SDI regions, there has been an increase in COPD-related deaths and DALYs among individuals aged 80 and above. From an age-based distribution of disease burden, the proportion of COPD-related burden in the 85–90 and 90–94 age groups have been gradually increasing, while the proportion in other age groups has remained relatively stable.

### Age, period, and birth cohort effects on COPD burden

**Figure 4:**
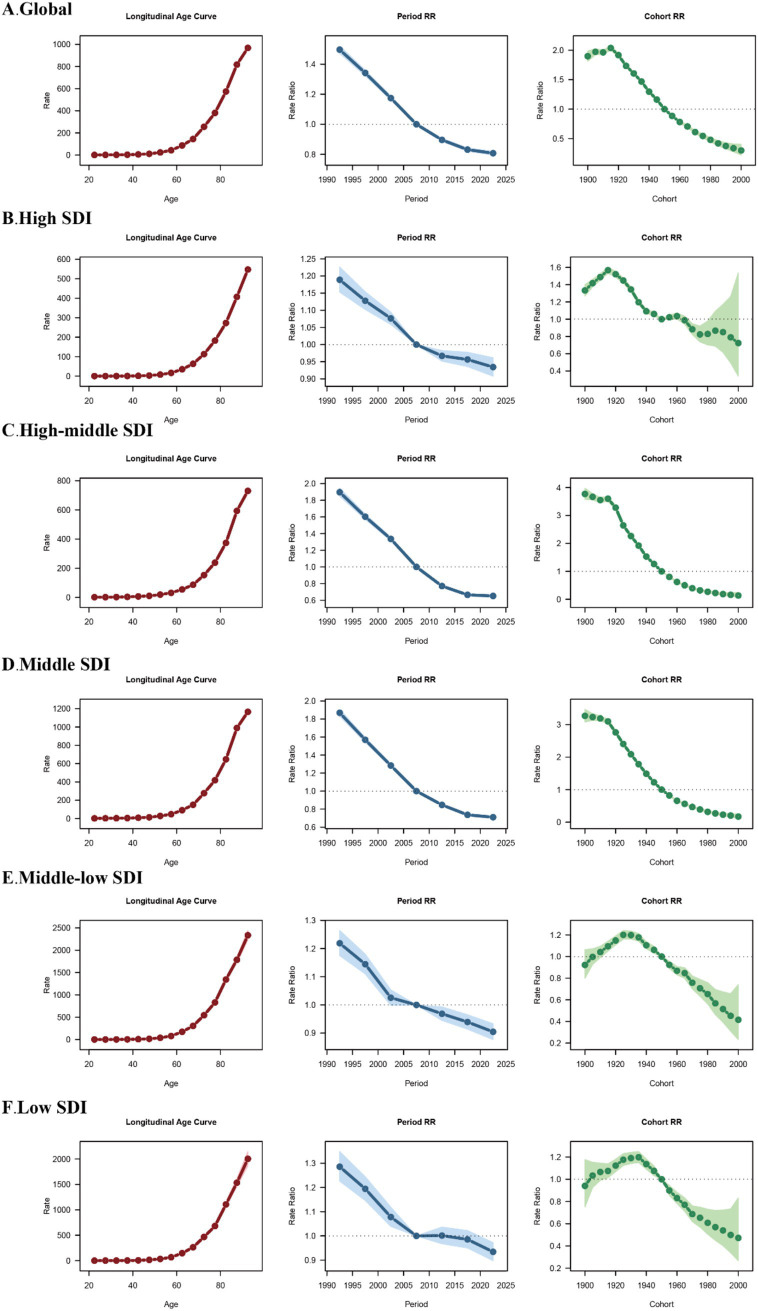
Age, period and birth cohort effects on COPD related deaths across SDI quintiles. Analysis of Age, period and birth cohort effects on COPD related deaths in **(A)** global level, **(B)** high SDI region, **(C)** high-middle SDI region, **(D)** middle SDI region, **(E)** middle-low SDI region, **(F)** low SDI region. RR, rate radio.

The age, period, and birth cohort effects for COPD related Deaths, DALYs and Prevalence derived from the APC model are illustrated in Figure 4, Supplementary Figures S10, S11 and detailed in Supplementary Tables S4–S6. Across all SDI regions, age effects demonstrated a consistent pattern: the DALYs, deaths, and prevalence rates of COPD were lower in younger age groups and progressively increased with advancing age, highlighting the cumulative impact of risk factors and age-related susceptibility to COPD.

Period effects revealed a general decline in DALYs and deaths across all SDI regions. Compared with the 2005–2009 period, rate ratios gradually decreased in subsequent periods. However, the trends in prevalence risk varied significantly among SDI regions: Globally, the relative risk of prevalence declined steadily over time, reflecting the positive impact of improved healthcare and control measures. In high SDI regions, there was a transient increase in prevalence risk during 2004–2005, followed by a consistent decrease in later periods. In middle and high-middle SDI regions, the prevalence risk ratios remained relatively stable throughout the study period. Conversely, low SDI regions showed an increasing prevalence risk ratio in later periods, indicating persistent challenges in mitigating COPD burden. Birth cohort effects showed a general pattern of increasing risk in earlier cohorts, followed by a declining trend in more recent cohorts across all disease burden metrics. Regarding prevalence risk ratios, the highest values were observed around the 1940 birth cohort.

For deaths, although the overall trends were similar across SDI regions, the magnitude of cohort effects varied considerably: Globally, the highest death risk ratio was seen in the 1915–1920 cohort (2.04, 95% UI: 2.00–2.07). In high SDI regions, the peak occurred in the 1915–1920 cohort (1.57, 95% UI: 1.53–1.60), while in high-middle and middle SDI regions, the highest death risk ratios were observed in the 1900–1904 cohort (3.77, 95% UI: 3.58–3.97 and 3.27, 95% UI: 3.08–3.47, respectively). Middle-low SDI regions showed the highest death risk ratio in the 1930–1934 cohort (1.20, 95% UI: 1.16–1.24), while in low SDI regions, the peak was in the 1935–1939 cohort (1.20, 95% UI: 1.15–1.25).

For DALYs, cohort effects also displayed an initial rise followed by a subsequent decline: Globally, the highest DALY risk ratio occurred in the 1915–1920 cohort (1.85, 95% UI: 1.82–1.89), similar with high SDI regions (1.40, 95% UI: 1.37–1.43). The peak DALY risk ratios in high-middle and middle SDI regions were seen in the 1905–1909 cohort (3.15, 95% UI: 2.90 to 3.42 and 2.88, 95% UI: 2.62–3.17, respectively). In middle-low SDI regions, the highest DALY risk ratio appeared in the 1930–1934 cohort (1.18, 95% UI: 1.15–1.21), while in low SDI regions, the peak occurred in the 1935–1939 cohort (1.17, 95% UI: 1.13–1.21).

**Figure 5:**
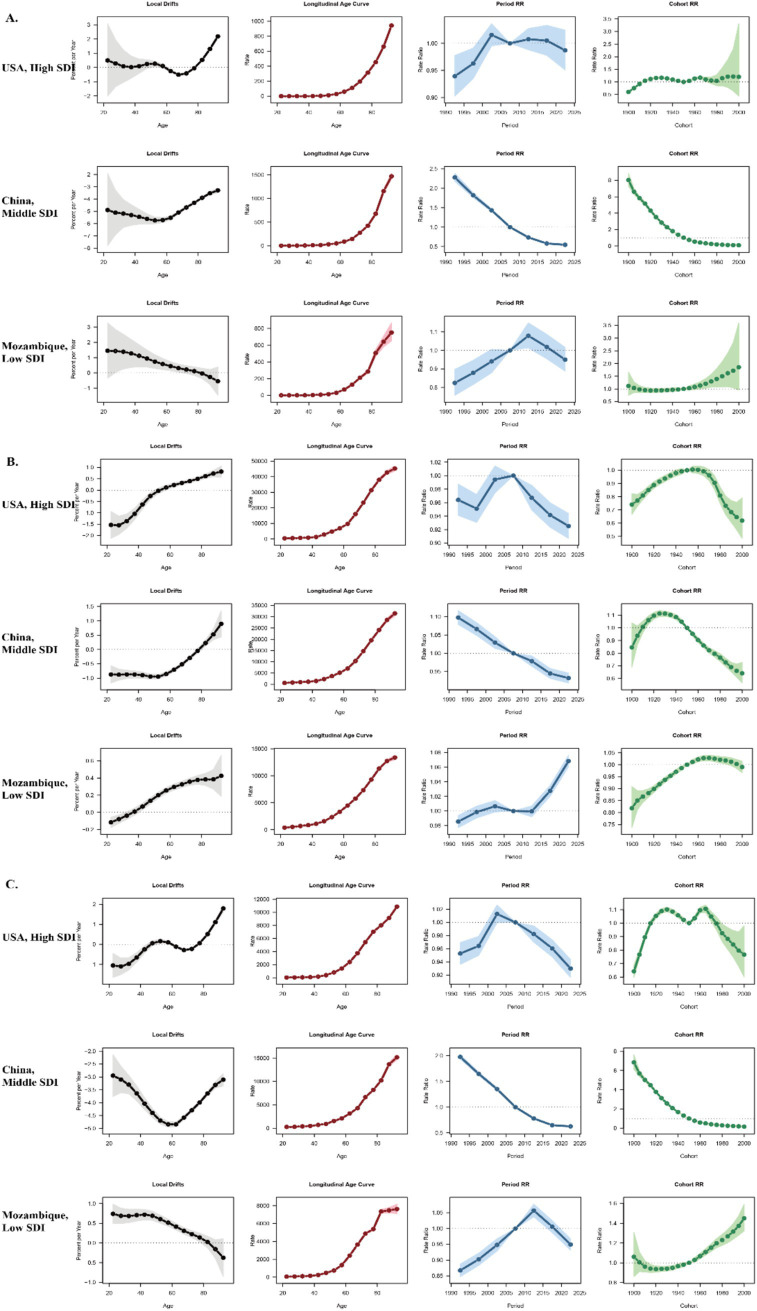
Age, period and birth cohort effects on COPD related deaths, prevalence and DALYs in USA, China and Mozambique. **(A)** Age, period and birth cohort effects on COPD related deaths on three countries. **(B)** Age, period and birth cohort effects on COPD related prevalence on three countries. **(C)** Age, period and birth cohort effects on COPD related DALYs on three countries.

Country-specific results for prevalence, DALYs, and deaths are presented in Supplementary Tables S7–S9. Representative countries from different SDI levels are highlighted in Figure 5:

The USA, representing high SDI countries, exhibited minimal variation in APC indicators, with all metrics remaining within a narrow range.

China, as an example of a middle SDI country, showed a clear shift in COPD burden toward older populations, with prevalence, DALYs, and deaths increasingly concentrated in the elderly. Period effects demonstrated a declining risk in more recent periods, while cohort effects showed significant improvements among newer generations.

Mozambique, representing low SDI countries, showed the highest net drift among all examples, with substantial increases in prevalence, DALYs, and deaths across nearly all age groups. Period and cohort effects both indicated continuous deterioration, emphasizing the persistent challenges faced by low-income regions.

## Discussion

This study provides a comprehensive analysis of the global, regional, and national trends in the burden of chronic obstructive pulmonary disease (COPD) across different age groups, time periods, and birth cohorts, utilizing an age-period-cohort (APC) model.

First, our findings reveal that, after adjusting for age and cohort effects, the prevalence of COPD continued to increase annually from 1990 to 2021 in more than half of the countries analyzed. Second, disparities in the burden of COPD, measured by disability-adjusted life years (DALYs) and mortality, were observed across countries with varying Socio-Demographic Index (SDI) levels. The differences in these burdens, particularly when interpreted alongside APC model results, warrant further investigation.

Building upon these findings, we need to further analyze the underlying connections within objective outcomes to formulate our perspectives for guiding future research and policy development. However, prior to this, it is essential to summarize existing research and understanding of COPD. COPD is primarily caused by long-term exposure to harmful particles and gases, with smoking being the most significant risk factor globally. Tobacco use accounts for up to 70% of COPD cases in high-income countries (15), while in low- and middle-income countries (LMICs), indoor air pollution from solid fuel use is a leading cause (16–20). Occupational exposure to dust, fumes, and chemicals also significantly contributes to COPD risk, particularly in industrialized regions (21–23). Additionally, ambient air pollution has emerged as a critical factor globally, with particulate matter (PM2.5) exposure associated with higher COPD incidence (19, 24, 25). Other emerging risks include climate change, which exacerbates air pollution and increases the frequency of respiratory infections (18, 26), and genetic predisposition, such as Alpha-1 antitrypsin deficiency (27).

Recent research has explored the multifaceted influences of age, geography, and sociopolitical factors on COPD. Age-related studies have emphasized the growing burden among older populations, driven by increased life expectancy and cumulative exposure to risk factors (3, 28). Younger populations, particularly in low-SDI regions, are increasingly exposed to indoor air pollution, setting the stage for early-onset COPD (29, 30).

Geographic and climatic factors significantly influence COPD burden. For instance, high-altitude regions tend to have lower COPD prevalence due to reduced exposure to air pollution, whereas urbanized areas show higher prevalence rates linked to traffic-related pollution (18, 26, 31, 32). Sociopolitical factors, such as healthcare accessibility, economic inequality, and smoking regulations, play a pivotal role. Countries with robust tobacco control policies, such as Australia and Singapore, have seen substantial declines in COPD prevalence (3, 33), while LMICs struggle with rising burdens due to limited healthcare infrastructure and weak enforcement of environmental policies (19, 20). Therefore, based on the above research, it is evident that inhalational factors—including pollutants and particulate matter—constitute the primary drivers of COPD pathogenesis. Factors such as climate, altitude, transportation, and environmental conditions ultimately exert their influence by modulating these inhalational exposures, which we collectively term risk factors due to their close association with pollutant inhalation. Conversely, socioeconomic development—manifested through healthcare expenditure and tobacco control policies—acts as a protective factor that mitigates disease burden. Notably, these two forces exhibit inherent tension: socioeconomic progress often simultaneously enhances healthcare capacity and exacerbates environmental pollution. This duality forms the core of our argument—policymaking must balance the triangular relationship between disease burden, protective factors, and risk factors. Tailoring policies to regional socioeconomic development levels and disease burden profiles represents a critical decision-making challenge for governments and health authorities. A key objective of this study is to provide evidence to inform such decisions.

Returning to our findings: The analysis of Net Drift values at the national level and their distribution across SDI categories reveals a notable pattern: countries with positive annual Net Drift values (greater than 0) between 1990 and 2021 are predominantly located in the high-middle, middle, and middle-low SDI regions. While this observation could be partially explained by the proportion of countries within these SDI categories (as they constitute the majority of the 204 countries and territories included in the GBD database), we propose an alternative hypothesis. During the period from 1990 to 2021, countries within these SDI levels likely experienced significant socioeconomic changes. These include trade-offs between economic development and increased environmental risks, such as air pollution, or the proliferation of tobacco use associated with improved living conditions. Simultaneously, healthcare investments may not have kept pace, and public health awareness may have remained relatively low. These factors could explain why countries in these SDI categories show higher exposure to risk factors for COPD compared to low SDI regions and yet lack the robust healthcare infrastructure observed in high SDI regions. Consequently, nations in these regions may need to place greater emphasis on balancing economic development with health policies.

Our analysis highlights a consistent increase in COPD burden with advancing age, as demonstrated by the age effects across all SDI regions. This trend reflects the cumulative exposure to risk factors such as smoking, occupational hazards, and air pollution, along with age-related declines in lung function. The pronounced burden in older age groups underscores the importance of age-specific interventions, particularly in high- and middle-SDI regions, where aging populations are a growing concern. Therefore, geriatric medicine-related policies—such as regular health screenings, comprehensive geriatric assessments, community-based healthcare initiatives, and tobacco control measures targeting older adults—require heightened attention (34).

Period effects revealed a general decline in COPD-related DALYs and deaths across most SDI regions after 2005–2009, suggesting progress in healthcare access, smoking cessation programs, and pollution control policies (35–38). However, the trends in period effect risk ratios varied significantly, with high-middle and middle SDI regions exhibiting greater fluctuations. This variability is likely associated with socioeconomic development; however, it is notable that many high-middle SDI countries also experienced a year-by-year increase in mortality and DALYs during 1990–2021. This observation underscores the importance of balancing socioeconomic progress with adequate investments in public health. Such findings hold critical implications for policy-making in low SDI regions, where development has been relatively delayed, emphasizing the need for targeted strategies to integrate economic growth with public health investments.

After correcting for period and age effects, birth cohort effects still showed a rising risk in earlier cohorts, followed by a decline in more recent ones, consistent across all COPD burden metrics. This decline in younger cohorts suggests that improvements in living conditions, healthcare, and awareness of risk factors have begun to offset COPD burden globally. However, regional disparities remain prominent. For instance, the highest prevalence risk ratios were observed in the 1940 birth cohort, whereas the highest death risk ratios varied by SDI region, peaking globally in the 1915–1920 cohort. Notably, the cohort with the peak risk ratio in low SDI regions lagged behind that in higher SDI regions. This is a particularly intriguing finding. First and foremost, the association with socioeconomic factors is indisputable, as this pattern emerges from SDI-stratified analyses. The critical question lies in how this phenomenon interacts with socioeconomic determinants. We posit that this linkage stems from the historical sequence of industrial-economic development and advancements in healthcare technology. When analyzed through the lens of birth cohort effects on mortality—given that COPD-related deaths predominantly result from acute exacerbations leading to respiratory failure—the mechanism becomes clearer.

In higher-SDI regions, environmental pollution emerged earlier, accompanied by higher tobacco exposure rates. The 1920 birth cohort in these areas faced dual challenges: heightened exposure to nascent industrial pollutants and limited access to contemporaneous medical interventions, inevitably amplifying disease burden. Conversely, low-SDI regions initially avoided this risk peak due to lower industrialization levels and milder environmental pollution during that era. By the time industrial pollution escalated in low-SDI regions, global medical technologies had advanced substantially (39), thereby attenuating cohort-specific mortality effects.

This rationale explains why the 1920–1930 birth cohorts in high-middle and middle-SDI regions exhibit the highest risk ratios (RR). These regions pursued socioeconomic development trajectories mirroring high-SDI nations but lagged in parallel healthcare investments, resulting in disproportionate health sacrifices during industrialization. For policymakers in these areas, this underscores the urgency of two parallel strategies: (1) prioritizing healthcare system strengthening commensurate with economic growth, and (2) accelerating industrial transition from heavily polluting sectors to tertiary industries or cleaner production models.

Thus, the findings underscore the need for tailored strategies to address COPD burden across different SDI regions. In low SDI regions, the rising prevalence and DALYs highlight the urgent need for interventions targeting modifiable risk factors, such as indoor air pollution and tobacco use, as well as improvements in access to healthcare and early diagnosis.

The observed cohort effects indicate that interventions targeting younger populations could yield long-term benefits. For example, smoking cessation programs, stricter pollution controls, and public health campaigns targeting respiratory health could significantly reduce COPD incidence in future generations. High-SDI countries’ success in reducing COPD burden—such as Norway’s 28% decline in smoking-related mortality through cohort-targeted workplace programs and Brazil’s 70% smoking prevalence drop via synchronized MPOWER policies (40)—provides adaptable frameworks for LMICs, including phased tobacco taxation paired with mobile cessation outreach and clean energy subsidies to offset biomass fuel exposure in high-risk rural cohorts. Additionally, the persistent burden in older populations highlights the importance of improving disease management and providing adequate healthcare resources for COPD-related complications.

This study utilizes the APC model to disentangle the effects of age, period, and birth cohorts on COPD burden, providing robust insights into temporal trends across diverse regions. By incorporating multiple disease burden metrics—prevalence, DALYs, and deaths—it offers a comprehensive understanding of COPD’s global impact. However, several limitations warrant attention. Firstly, the model relies on aggregate data, which may not adequately capture individual-level variability in risk factors, potentially masking heterogeneity within specific populations. Secondly, the accuracy of the estimates may be affected by disparities in data quality and completeness, particularly in low-SDI regions where healthcare reporting and surveillance systems may be less developed. Lastly, the analysis focuses exclusively on interpreting APC model outputs and commonly recognized COPD risk factors, without integrating direct data analysis on exposure to these risk factors. This limits the ability to quantify the contributions of specific exposures, such as smoking, air pollution, and occupational hazards, to the observed trends.

While our analysis highlights the limitations of aggregate data and unmeasured risk factor exposures, future studies could address these gaps by integrating individual-level data sources for examples, electronic health records, biobanks to disentangle how specific risk factors such as smoking intensity, occupational particulate matter exposure, or genetic susceptibility interact with age-period-cohort effects. For instance, linking longitudinal exposure histories to individual COPD outcomes could clarify how cohort-specific exposures drive disparities in disease burden.

## Conclusion

1. With the growth of the global population, the absolute numbers of COPD prevalence, mortality, and DALYs have also increased.
2. The prevalence of COPD remains concerning. Compared to 1990, the global prevalence of COPD in 2021 showed a stable or slightly increasing trend, with over half of the countries experiencing an annual increase in prevalence during the 1990–2021 period.
3. Global mortality and DALYs associated with COPD showed a notable decline in 2021 compared to 1990. However, this trend exhibited significant heterogeneity across countries and regions, likely linked to differences in socioeconomic development.
4. Countries in the high-middle and middle SDI regions were found to be more affected by period effects.
5. From an age effect perspective, population aging has undoubtedly exacerbated the COPD burden.
6. Regarding cohort effects, earlier birth cohorts demonstrated a stronger contribution to the increasing disease burden. While Higher levels of socioeconomic development can mitigate the adverse effects associated with earlier birth cohorts.
7. *Policy implications*: Our findings underscore the need for policymakers to balance socioeconomic development with targeted mitigation of COPD risk factors, such as air pollution and tobacco use. Region-specific strategies are critical:
  a) *High-SDI Regions*:
    Prioritize aging-focused policies, including geriatric healthcare infrastructure and long-term care systems, to address the disproportionate burden among elderly populations.
  b) *Middle-High- and Middle-SDI Regions*:
    Weigh the dual impacts of industrialization and medical advancement. While economic growth enables higher healthcare standards, stricter environmental regulations (e.g., emission controls, occupational safety laws) are essential to counterbalance industrial expansion’s adverse effects.
  c) *Low-Middle- and Low-SDI Regions*:
    Focus on economic development to strengthen healthcare infrastructure while implementing cost-effective preventive measures (e.g., clean energy transitions, tobacco taxation). Strengthening primary care systems and early diagnosis programs could mitigate rising prevalence despite limited resources.

## Data Availability

The original contributions presented in the study are included in the article/Supplementary material, further inquiries can be directed to the corresponding author.

## Footnotes

^1^ https://vizhub.healthdata.org/gbd-results/

^2^ https://analysistools.cancer.gov/apc/
