# Supplementary material for: Global, regional, and national temporal trends in prevalence, deaths and disability-adjusted life years for chronic pulmonary disease, 1990–2021: an age-period-cohort analysis based on the global burden of disease study 2021
Source: Front Med (Lausanne). 2025 Mar 4;12:1554442. doi: 10.3389/fmed.2025.1554442 (PMC11913687; doi:10.3389/fmed.2025.1554442)
Supplement: Supplementary file 1 [file Supplementary_file_1.docx]

**
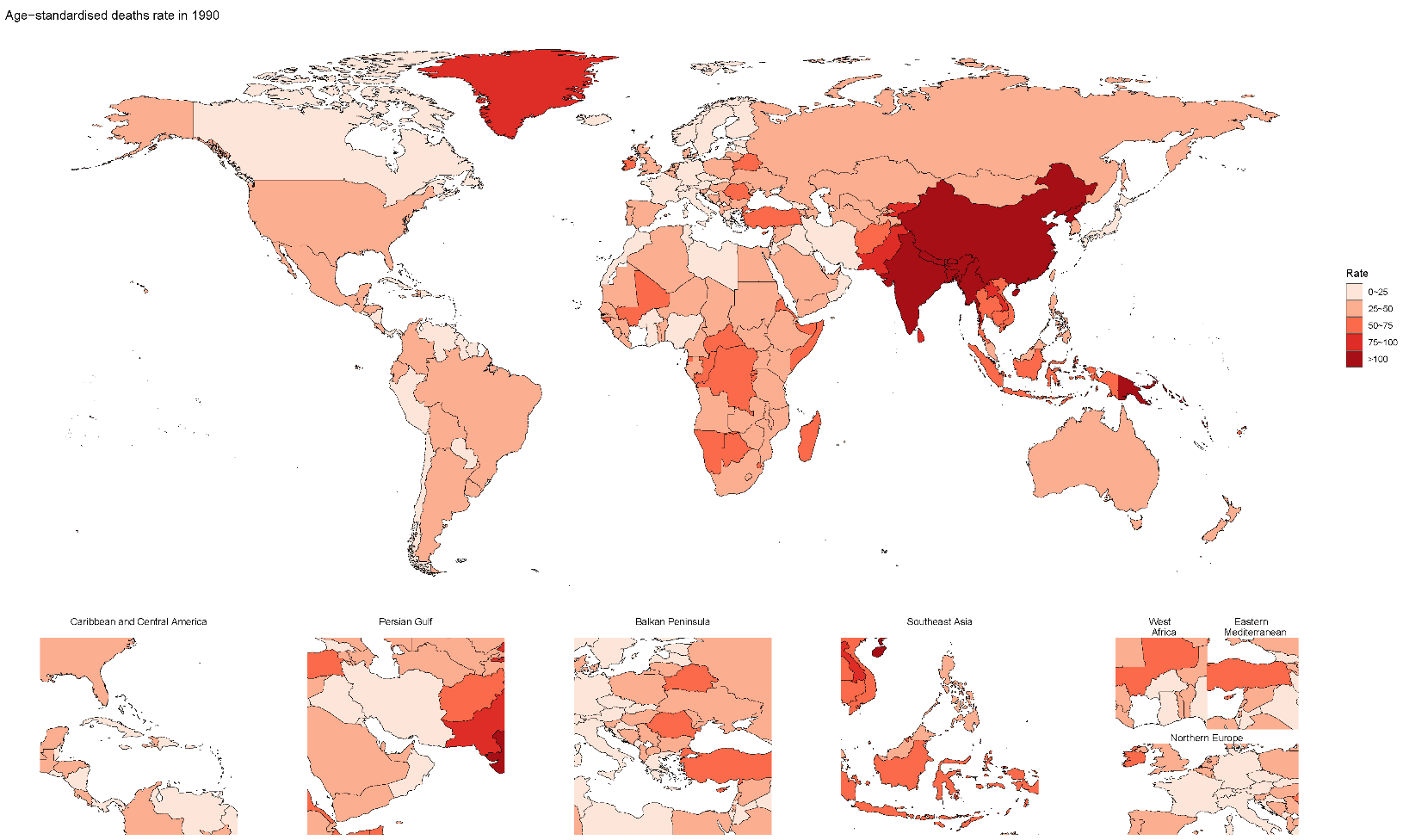
Figure S1 Age-Standardized deaths rate (per 100,000 population) in 1990**

**Figure S2 Age-Standardized prevalence rate (per 100,000 population) in 1990**

**
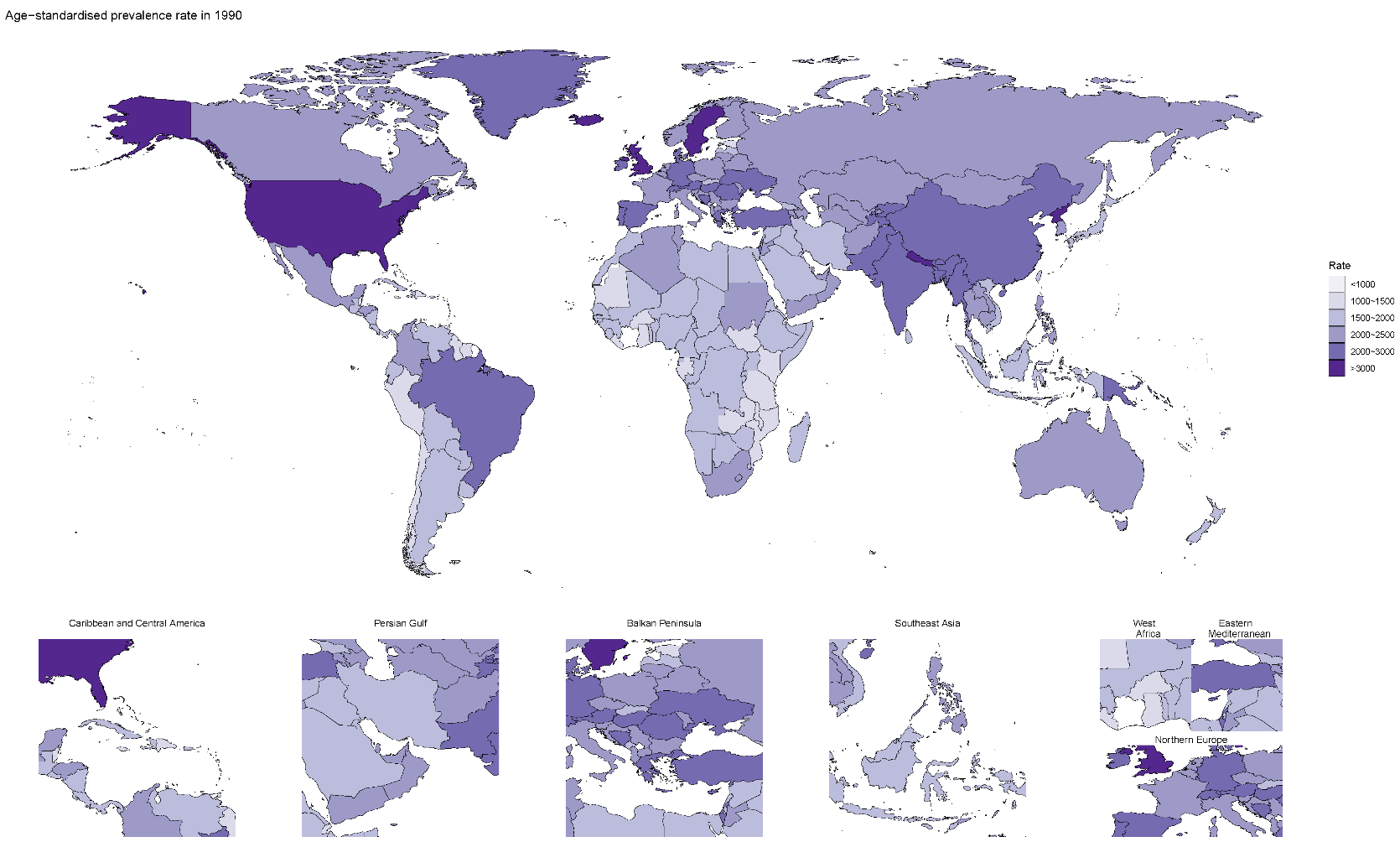
**

**
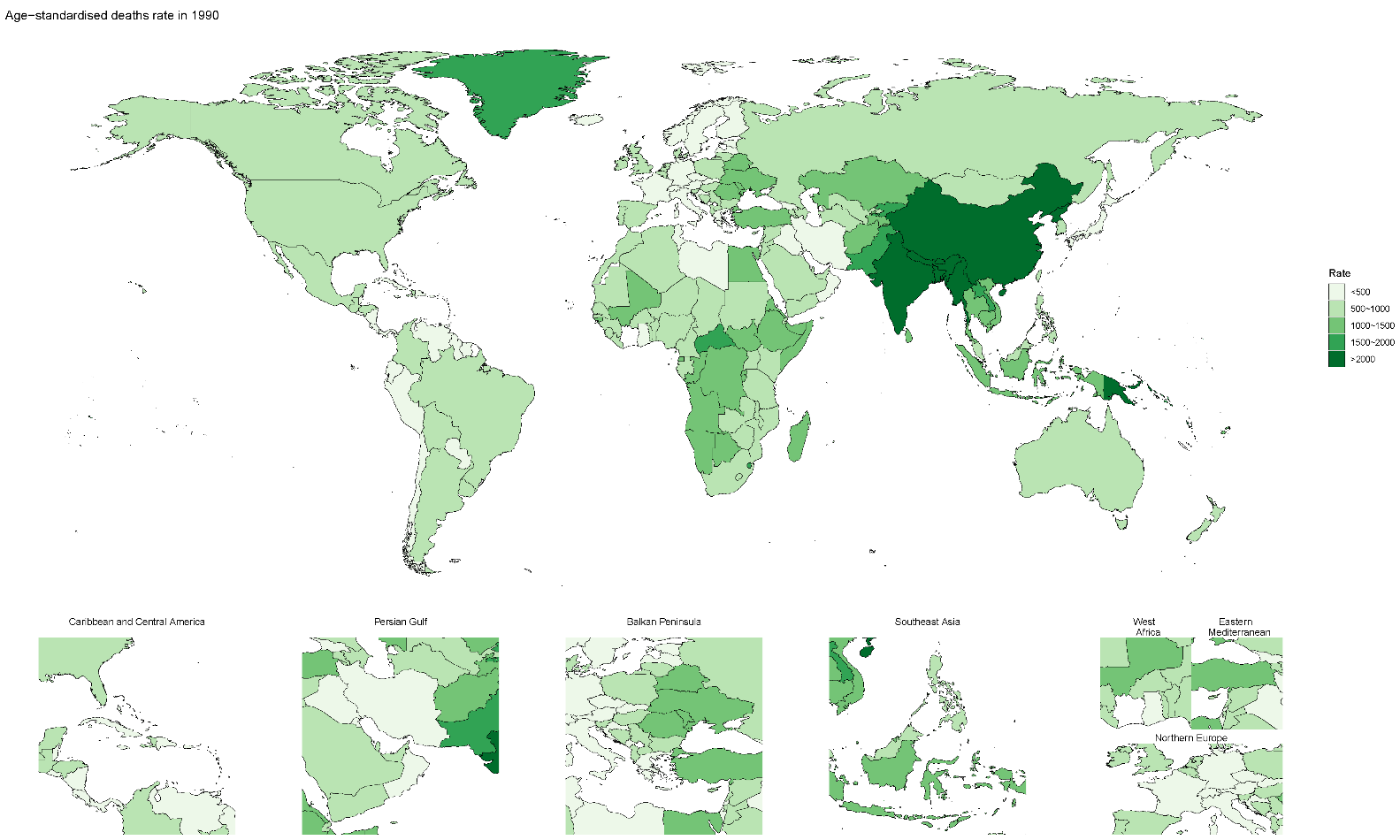
Figure S3 Age-Standardized Disability Adjusted Life Years (DALYs) rate (per 100,000 population) in 1990**

**
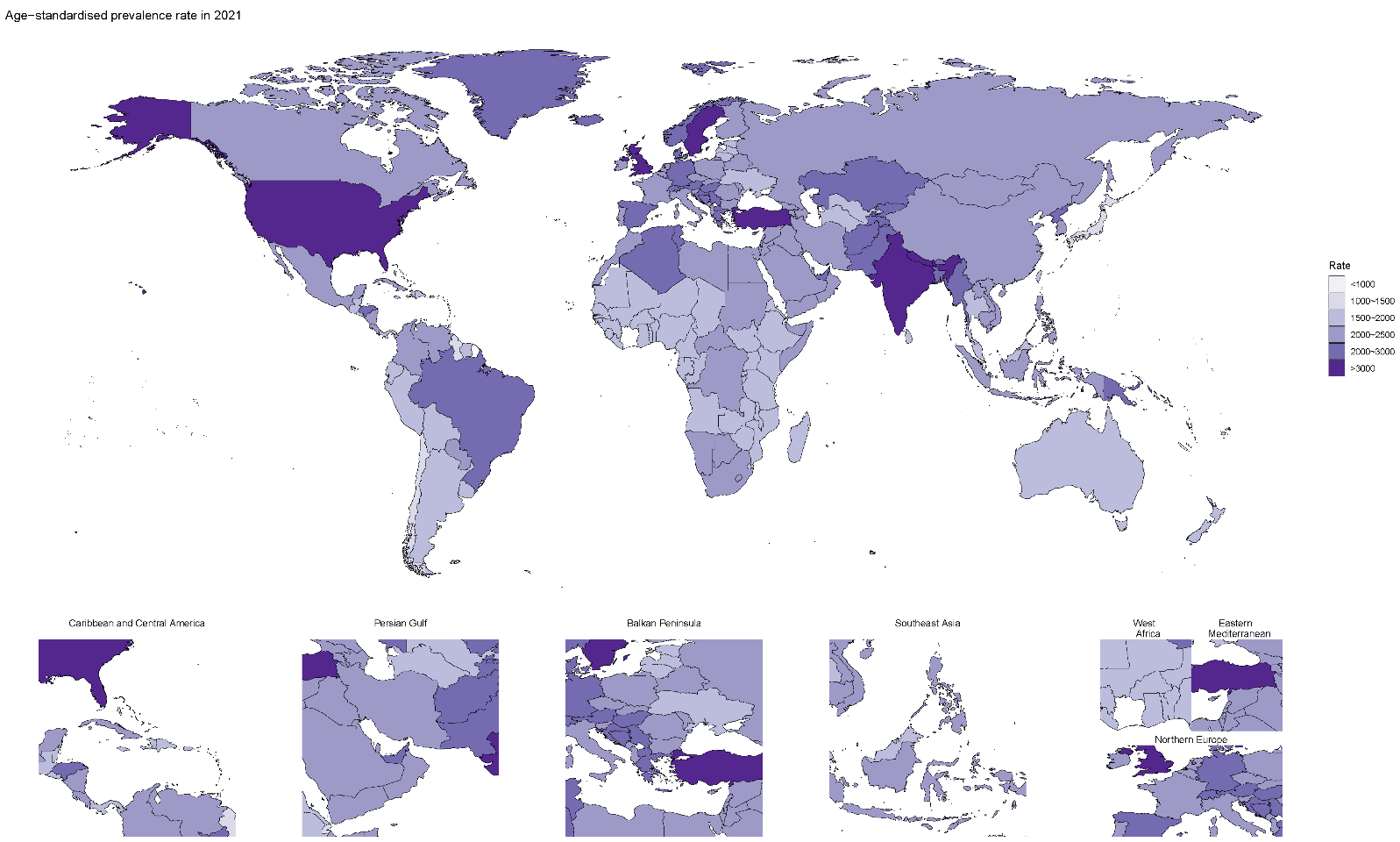
Figure S4 Age-Standardized prevalence rate (per 100,000 population) in 2021**

**
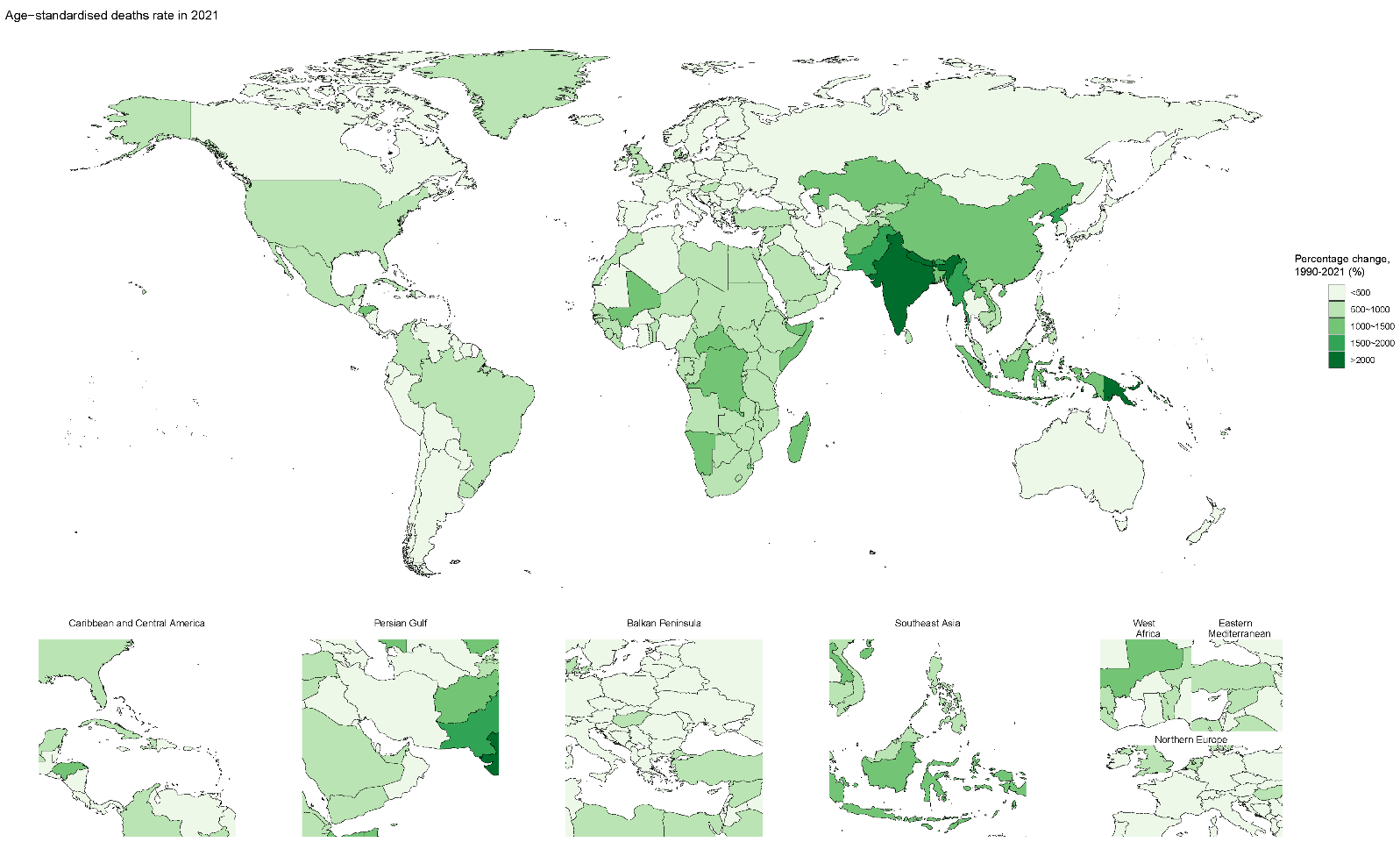
Figure S5 Age-Standardized Disability Adjusted Life Years (DALYs) rate (per 100,000 population) in 2021**

**
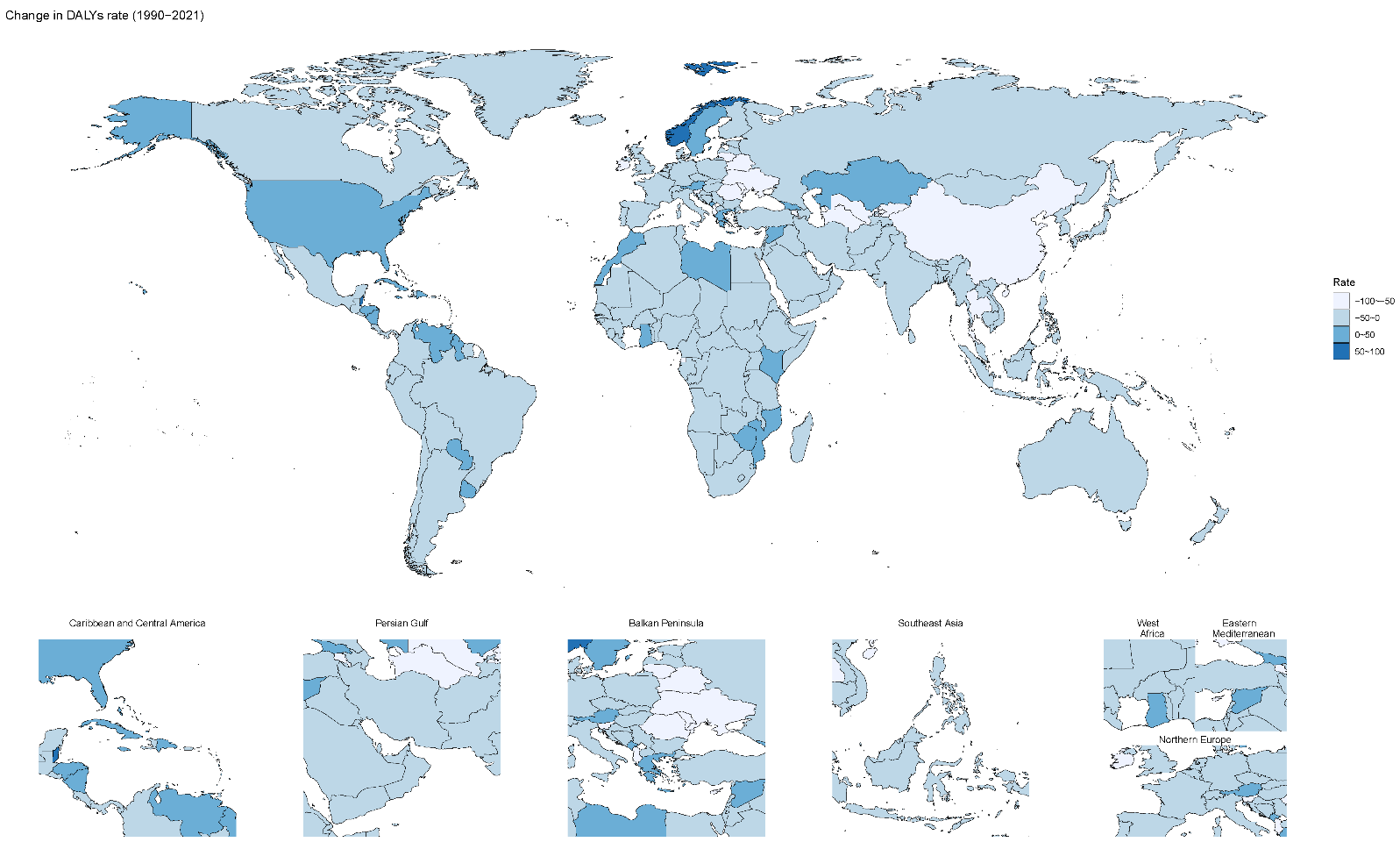
Figure S6 Age-Standardized Disability Adjusted Life Years (DALYs) rate percentage change (%) from 1990 to2021**

**
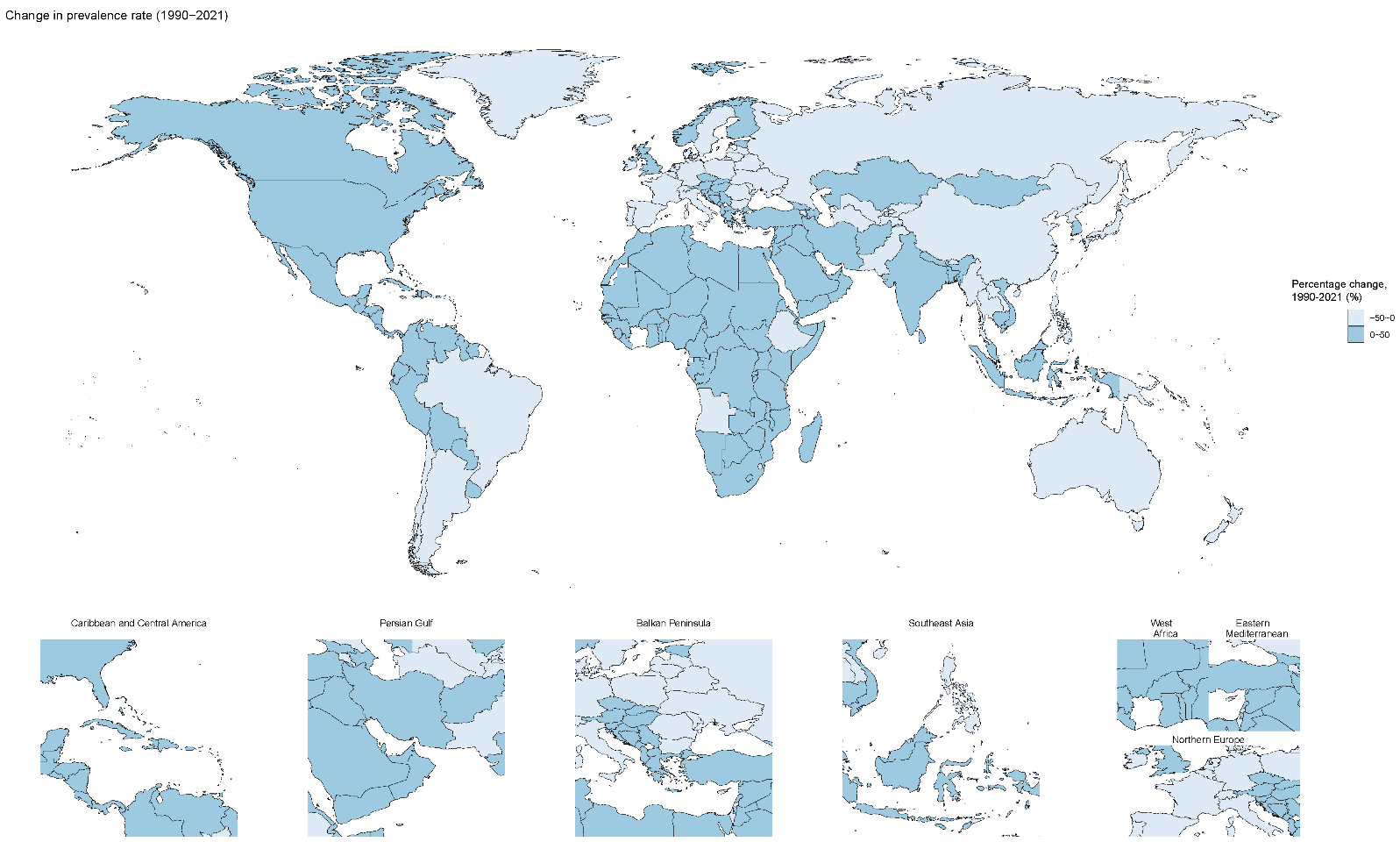
Figure S7 Age-Standardized prevalence rate percentage change (%) from 1990 to2021**

**Figure S8 Local drift and age distribution of prevalence from 1990 to 2021 for COPD across SDI quintiles**

**
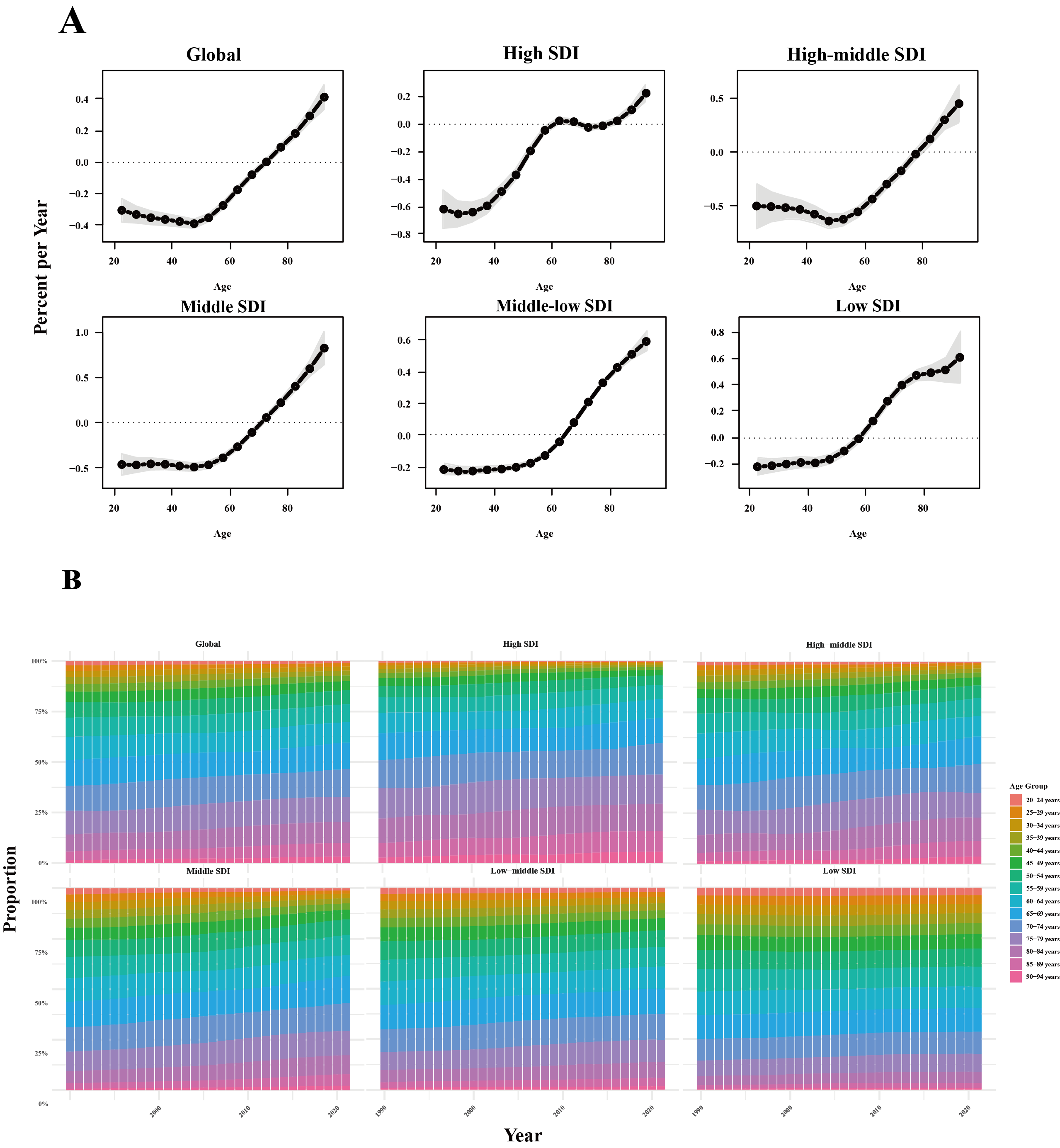
**

**Figure S9 Local drift and age distribution of DALYs from 1990 to 2021 for COPD across SDI quintiles**

**
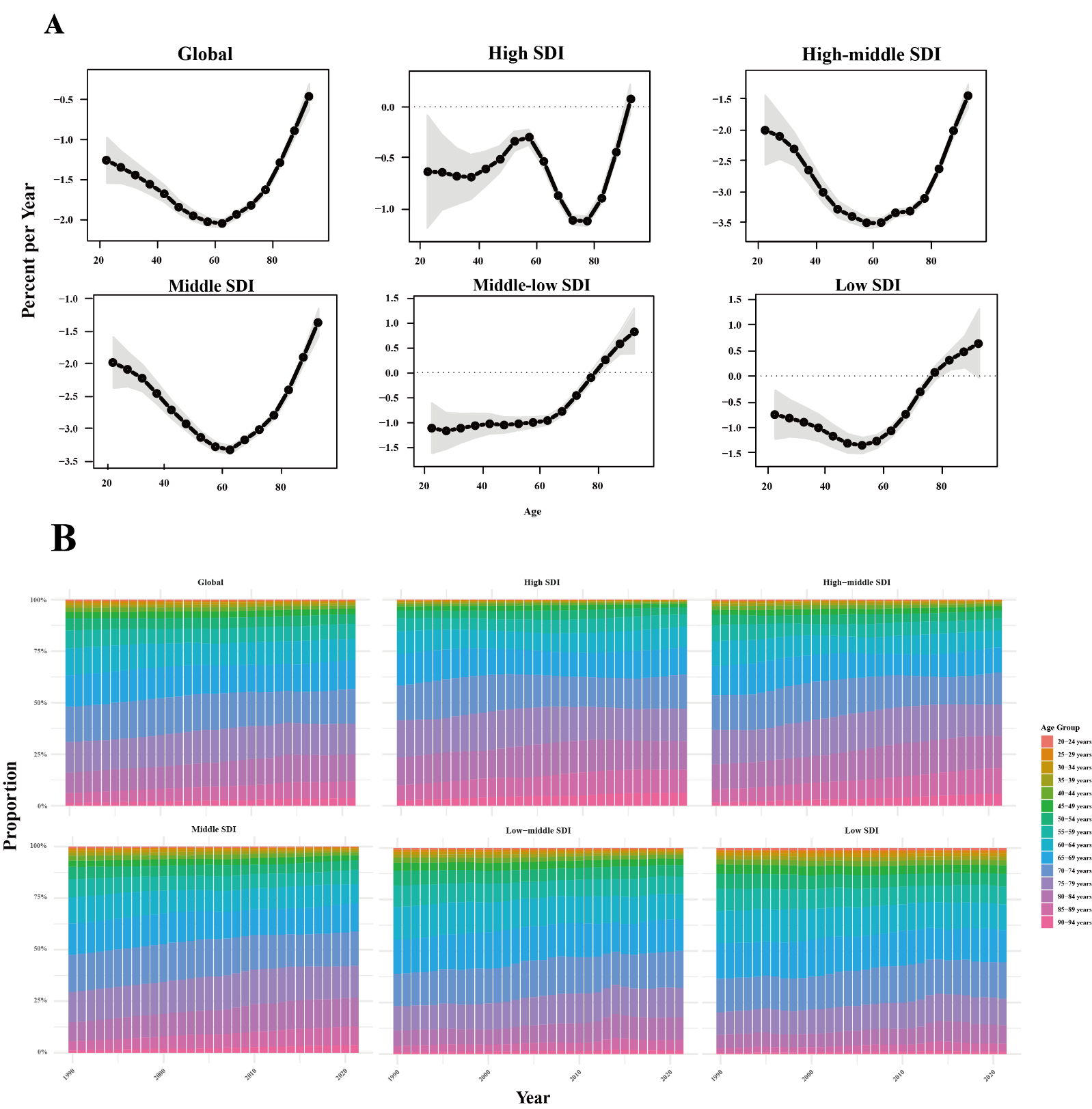
**

**Figure S10 Age, period and birth cohort effects on COPD prevalence across SDI quintiles
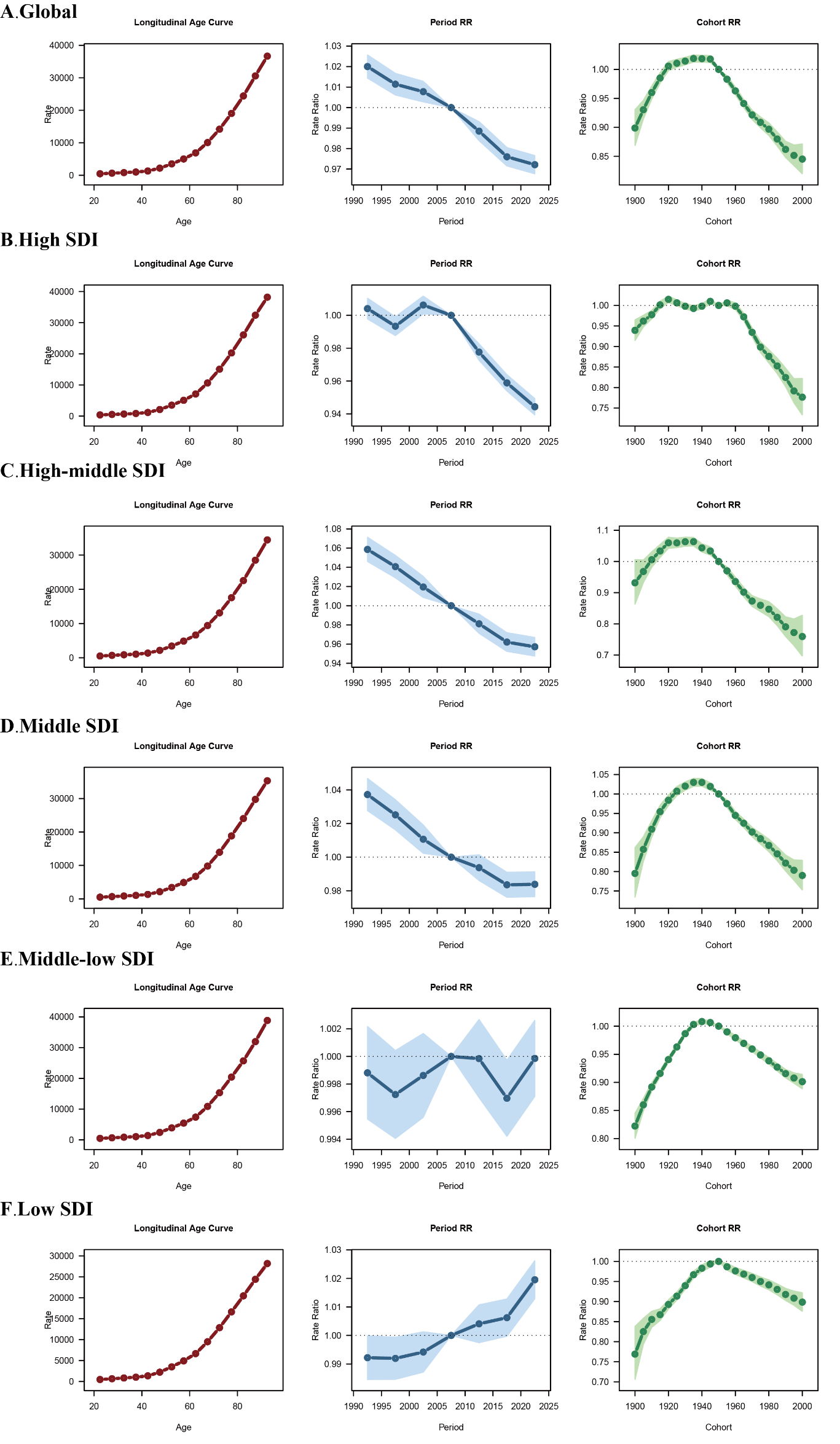
**

**
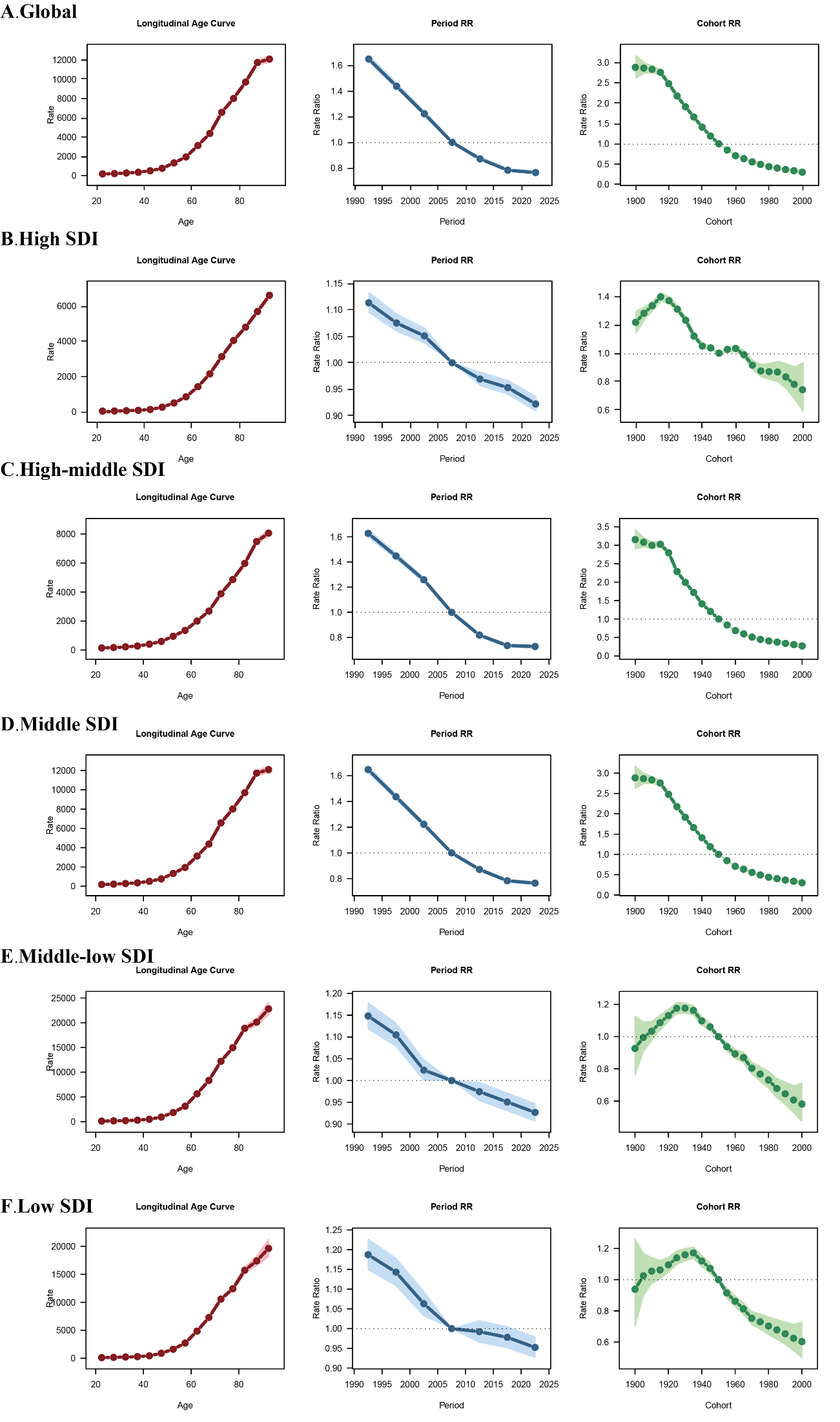
Figure S11 Age, period and birth cohort effects on COPD Disability Adjusted Life Years (DALYs) across SDI quintiles**
