# Supplementary material for: Global, regional, and national temporal trends in prevalence, deaths and disability-adjusted life years for chronic pulmonary disease, 1990–2021: an age-period-cohort analysis based on the global burden of disease study 2021
Source: Front Med (Lausanne). 2025 Mar 4;12:1554442. doi: 10.3389/fmed.2025.1554442 (PMC11913687; doi:10.3389/fmed.2025.1554442)
Supplement: Supplementary file 2 [file Supplementary_file_2.docx]

| **Nomenclature** | **Interpretation** |
| --- | --- |
| Net Drift | APC analogue of the estimated annual percentage change (EAPC) in the age-standardized rate (ASR);  log-linear component of *FTT*(*p* \| *a*0)**,** *PRR*(*p* \| *p*0) and *CRR*(*c* \| *c*0) |
| Age deviations, *AD*(*a*) | Non-linear age effects incorporated into *LongAge*(*a* \| *c*0)**,** *CrossAge*(*a* \| *p*0) and *Long*2*CrossRR*(*a* \| *c*0, *p*0);  orthogonal to the linear trend in age |
| Period deviations, *PD*(*p*) | Non-linear period effects incorporated into *FTT*(*p* \| *a*0) and *PRR*(*p* \| *p*0); orthogonal to the linear trend in period |
| Cohort deviations, *CD*(*c*) | Non-linear cohort effects incorporated into *CRR*(*c* \| *c*0) and  *LocalDrifts* (*a*); orthogonal to the linear trend in cohort (over the entire rate matrix) |
| Longitudinal Age Curve, *LongAge*(*a* \| *c*0) | Fitted longitudinal age-specific rates in reference cohort *c*0 adjusted for period deviations |
| Period Rate Ratios, *PRR*(*p* \| *p*0) | Ratio of age-specific rates in period *p* relative to reference period *p*0 |
| Cohort Rate Ratios, *CRR*(*c* \| *c*0) | Ratio of age-specific rates in cohort *c* relative to reference cohort *c*0 |
| Local Drifts, *LocalDrifts*(*a*) | Estimated annual percentage change over time specific to age group *a* |

*For APC model defined over A age groups and P calendar periods with equal intervals. In the web tool, the central age group, calendar period, and birth cohort define the reference values a0, p0, c0, respectively. When there is an even number of age, period, or cohort categories, the reference value is the lower of the two central values. In the R package, reference values can be set to any observed age, period and cohort = period-age in the rate matrix.

COPD data in put

The GBD 2021 study utilizes a wide range of data sources to synthesize information on morbidity, mortality, and attributable risk for 204 countries and territories. These data sources include surveys, censuses, vital statistics, and other health-related data. To access the specific data input sources used in the GBD study, an interactive citation tool is provided through the GHDx. The Data Input Sources Tool in GHDx, accessible at <https://ghdx.healthdata.org/gbd-2021>, allows users to view and access GHDx records for the input sources. It also enables users to export a comma-separated value (CSV) file containing metadata, citations, and information about where the data were used in the GBD study. This tool helps researchers and stakeholders access the specific sources used in the study and explore the associated metadata. The CoD (Causes of Death) database used in the GBD 2021 study includes various types of data sources, such as vital registration, verbal autopsy, cancer registries, police records, sibling history, surveillance, survey/census data, and minimally invasive tissue sample (MITS) diagnoses. While complete vital registration systems provide comprehensive cause of death information, not all countries have such systems in place. For countries with incomplete vital registration systems, other data types may be used to supplement the vital statistics for causes of death.

Overall, the GBD 2021 study ensures transparency and accessibility of the data sources used through the interactive citation tool and provides a comprehensive range of information on morbidity, mortality, and attributable risk worldwide. To improve the representation of population-level disease incidence for COPD in the GBD 2021 study, several adjustments were made to the data prior to analysis using the DisMod-MR modeling tool. These adjustments were performed using the MR-BRT (Bayesian regularized model with temporal smoothing) modeling tool. The purpose of these adjustments was to enhance the accuracy and reliability of the estimates. Specifically, the study accounted for various study-specific factors when adjusting the data. Adjustments were made to ensure that the analysis appropriately accounted for these factors, resulting in more robust and accurate estimates of disease incidence. Vital registration and surveillance data from the cause of death (COD) database were used to estimate COPD mortality. Verbal autopsy data were not included and were instead mapped to an overall chronic respiratory model. The outlier criteria excluded data points that (1) were implausibly high or low relative to global or regional patterns, (2) substantially conflicted with established age or temporal patterns, or (3) significantly conflicted with other data sources conducted from the same locations or locations with similar characteristics (ie, Socio-demographic Index).
