# Supplementary material for: Global, regional, and national temporal trends in prevalence, deaths and disability-adjusted life years for chronic pulmonary disease, 1990–2021: an age-period-cohort analysis based on the global burden of disease study 2021
Source: Front Med (Lausanne). 2025 Mar 4;12:1554442. doi: 10.3389/fmed.2025.1554442 (PMC11913687; doi:10.3389/fmed.2025.1554442)
Supplement: Supplementary file 3 [file Table_1.docx]

Table S1 The age-standardized Deaths prevalence DALYs rate in 1990 and 2021, as well as Percentage chage and Net Drift from 1990 to 2021· for COPD across countries

| measure_name | location_name | year | Rate | Percentage_chage from 1990 to 2021(95%UI) | NetDrift from 1990 to 2021(95%UI) |
| --- | --- | --- | --- | --- | --- |
| Deaths | Afghanistan | 2021 | 50.6(36.13to65.6) | -18.68(-42.56to40.68) | -1.27(-1.45 to -1.1) |
| Deaths | Afghanistan | 1990 | 62.22(38.16to82.85) | NA | NA |
| DALYs (Disability-Adjusted Life Years) | Afghanistan | 1990 | 1426.64(960.32to1838.69) | NA | NA |
| DALYs (Disability-Adjusted Life Years) | Afghanistan | 2021 | 1155.17(875.13to1480.07) | -19.03(-41.57to33.26) | -1.08(-1.12 to -1.04) |
| Prevalence | Afghanistan | 1990 | 2385.47(2138.28to2655.11) | NA | NA |
| Prevalence | Afghanistan | 2021 | 2590.12(2315.15to2913.83) | 8.58(2.2to16) | 0.05(0.03 to 0.08) |
| Deaths | Albania | 2021 | 15.57(11.92to21.04) | -61.53(-72.08to-45.95) | -3.09(-4.32 to -1.85) |
| Deaths | Albania | 1990 | 40.47(34.68to47.31) | NA | NA |
| DALYs (Disability-Adjusted Life Years) | Albania | 1990 | 759.54(662.9to864.71) | NA | NA |
| DALYs (Disability-Adjusted Life Years) | Albania | 2021 | 342.39(280.66to430.36) | -54.92(-64.36to-42.15) | -2.02(-2.22 to -1.81) |
| Prevalence | Albania | 1990 | 2319.36(2036.62to2638.44) | NA | NA |
| Prevalence | Albania | 2021 | 2389.58(2092.08to2724.78) | 3.03(-6.57to13.31) | -0.07(-0.11 to -0.04) |
| Deaths | Algeria | 2021 | 22.08(17.4to27.31) | -13.76(-35.37to13.84) | -0.78(-1.27 to -0.28) |
| Deaths | Algeria | 1990 | 25.6(20.81to31.74) | NA | NA |
| DALYs (Disability-Adjusted Life Years) | Algeria | 2021 | 488.58(415.38to573.16) | -7.88(-25.22to13.83) | -0.31(-0.51 to -0.12) |
| DALYs (Disability-Adjusted Life Years) | Algeria | 1990 | 530.38(447.94to630.53) | NA | NA |
| Prevalence | Algeria | 1990 | 2184.97(1917.62to2492.49) | NA | NA |
| Prevalence | Algeria | 2021 | 2643.64(2331.69to2954.44) | 20.99(11.93to32.61) | 0.4(0.38 to 0.41) |
| Deaths | American Samoa | 1990 | 78.17(68.92to89.67) | NA | NA |
| Deaths | American Samoa | 2021 | 48.75(41.55to57.11) | -37.64(-49.14to-24.92) | -1.5(-4.52 to 1.63) |
| DALYs (Disability-Adjusted Life Years) | American Samoa | 1990 | 1544.45(1371.26to1739.36) | NA | NA |
| DALYs (Disability-Adjusted Life Years) | American Samoa | 2021 | 993.97(860.8to1150.4) | -35.64(-46.94to-23.42) | -1.36(-1.79 to -0.93) |
| Prevalence | American Samoa | 1990 | 2275.87(2017.61to2565.99) | NA | NA |
| Prevalence | American Samoa | 2021 | 1975.32(1744.82to2226.52) | -13.21(-18.87to-7.49) | -0.5(-0.8 to -0.21) |
| Deaths | Andorra | 2021 | 17.72(12.5to23.07) | -45.27(-63.71to-19.83) | -1.99(-9.09 to 5.67) |
| Deaths | Andorra | 1990 | 32.37(24.01to43.1) | NA | NA |
| DALYs (Disability-Adjusted Life Years) | Andorra | 1990 | 654.03(509.97to838.39) | NA | NA |
| DALYs (Disability-Adjusted Life Years) | Andorra | 2021 | 390.98(303.12to486.99) | -40.22(-56.61to-18.16) | -1.39(-1.98 to -0.79) |
| Prevalence | Andorra | 1990 | 2863.66(2569.41to3171.15) | NA | NA |
| Prevalence | Andorra | 2021 | 2655.23(2387.68to2982.29) | -7.28(-12.67to-1.79) | -0.28(-0.46 to -0.11) |
| Deaths | Angola | 1990 | 49.94(36.19to64.52) | NA | NA |
| Deaths | Angola | 2021 | 29.26(22.6to38.2) | -41.42(-59.06to-5.16) | -2.17(-2.45 to -1.88) |
| DALYs (Disability-Adjusted Life Years) | Angola | 2021 | 692.53(547.4to863.01) | -38.91(-54.98to-10.74) | -1.72(-1.77 to -1.67) |
| DALYs (Disability-Adjusted Life Years) | Angola | 1990 | 1133.66(856.24to1425.55) | NA | NA |
| Prevalence | Angola | 1990 | 1766.25(1567.65to2007.82) | NA | NA |
| Prevalence | Angola | 2021 | 1672.11(1444.04to1899.12) | -5.33(-12.09to1.86) | -0.35(-0.38 to -0.32) |
| Deaths | Antigua and Barbuda | 2021 | 7.71(7.11to8.53) | 40.47(27.18to54.78) | 0.83(-10.6 to 13.72) |
| Deaths | Antigua and Barbuda | 1990 | 5.49(5.03to5.91) | NA | NA |
| DALYs (Disability-Adjusted Life Years) | Antigua and Barbuda | 1990 | 132.15(121.23to143.46) | NA | NA |
| DALYs (Disability-Adjusted Life Years) | Antigua and Barbuda | 2021 | 179.35(165.54to194.02) | 35.71(25.63to47.38) | 0.69(-0.32 to 1.71) |
| Prevalence | Antigua and Barbuda | 1990 | 1011.32(867.97to1165.68) | NA | NA |
| Prevalence | Antigua and Barbuda | 2021 | 1325.13(1134.42to1515.79) | 31.03(20.41to44.89) | 0.7(0.41 to 0.99) |
| Deaths | Argentina | 1990 | 27.2(25.31to29.03) | NA | NA |
| Deaths | Argentina | 2021 | 24.51(22.43to26.4) | -9.88(-16.78to-2.52) | -1.13(-1.31 to -0.95) |
| DALYs (Disability-Adjusted Life Years) | Argentina | 2021 | 487.71(454.18to519.7) | -14.2(-20.45to-7.76) | -0.99(-1.1 to -0.89) |
| DALYs (Disability-Adjusted Life Years) | Argentina | 1990 | 568.44(534.79to605.11) | NA | NA |
| Prevalence | Argentina | 1990 | 1603.99(1386.47to1818.58) | NA | NA |
| Prevalence | Argentina | 2021 | 1601.3(1436.49to1793.4) | -0.17(-9.16to11.21) | -0.11(-0.14 to -0.07) |
| Deaths | Armenia | 1990 | 43.3(40.45to45.79) | NA | NA |
| Deaths | Armenia | 2021 | 15.19(13.08to17.33) | -64.92(-69.01to-59.93) | -4.69(-5.36 to -4.01) |
| DALYs (Disability-Adjusted Life Years) | Armenia | 1990 | 918.66(869.63to967.9) | NA | NA |
| DALYs (Disability-Adjusted Life Years) | Armenia | 2021 | 339.94(305.43to379.96) | -63(-66.73to-58.79) | -3.64(-3.83 to -3.45) |
| Prevalence | Armenia | 1990 | 2390.25(2132.91to2696.99) | NA | NA |
| Prevalence | Armenia | 2021 | 2370.28(2110.39to2680.12) | -0.84(-10.8to10.65) | -0.18(-0.22 to -0.15) |
| Deaths | Australia | 1990 | 28.76(26.87to30.34) | NA | NA |
| Deaths | Australia | 2021 | 18.33(16.16to19.88) | -36.26(-42.29to-30.87) | -1.29(-1.82 to -0.76) |
| DALYs (Disability-Adjusted Life Years) | Australia | 2021 | 365.77(332.29to390.19) | -38.85(-43.19to-34.59) | -1.29(-1.46 to -1.12) |
| DALYs (Disability-Adjusted Life Years) | Australia | 1990 | 598.16(564.83to629.03) | NA | NA |
| Prevalence | Australia | 1990 | 2076.5(1826.07to2292.52) | NA | NA |
| Prevalence | Australia | 2021 | 1562.13(1386.02to1789.86) | -24.77(-31.45to-15.31) | -1.05(-1.09 to -1.01) |
| Deaths | Austria | 2021 | 15.69(13.94to16.79) | -2.88(-9.22to2.83) | -0.26(-1.12 to 0.62) |
| Deaths | Austria | 1990 | 16.16(14.95to16.89) | NA | NA |
| DALYs (Disability-Adjusted Life Years) | Austria | 1990 | 380.49(357.14to406.3) | NA | NA |
| DALYs (Disability-Adjusted Life Years) | Austria | 2021 | 381.09(350.49to411.09) | 0.16(-4.54to4.88) | -0.04(-0.19 to 0.1) |
| Prevalence | Austria | 1990 | 2860.19(2600.49to3116.52) | NA | NA |
| Prevalence | Austria | 2021 | 2891.94(2593.55to3153.57) | 1.11(-4.99to8.55) | -0.15(-0.18 to -0.11) |
| Deaths | Azerbaijan | 1990 | 29.65(24.75to35.61) | NA | NA |
| Deaths | Azerbaijan | 2021 | 15.34(12.08to19.77) | -48.25(-61.3to-29.59) | -3.31(-3.66 to -2.96) |
| DALYs (Disability-Adjusted Life Years) | Azerbaijan | 2021 | 374.49(309.17to479.57) | -43.54(-55.91to-26.11) | -2.6(-2.67 to -2.52) |
| DALYs (Disability-Adjusted Life Years) | Azerbaijan | 1990 | 663.27(573.56to768.07) | NA | NA |
| Prevalence | Azerbaijan | 1990 | 2205.22(1949.45to2500.73) | NA | NA |
| Prevalence | Azerbaijan | 2021 | 2246.65(1967.69to2555.5) | 1.88(-5.46to9.27) | -0.19(-0.23 to -0.15) |
| Deaths | Bahamas | 2021 | 10.34(8.5to12.42) | 18.51(-4.4to45.33) | 0.3(-2.75 to 3.46) |
| Deaths | Bahamas | 1990 | 8.73(7.91to9.56) | NA | NA |
| DALYs (Disability-Adjusted Life Years) | Bahamas | 2021 | 239.25(201.04to280.35) | 13.77(-5.59to37.04) | 0.29(-0.09 to 0.68) |
| DALYs (Disability-Adjusted Life Years) | Bahamas | 1990 | 210.29(191.81to229.66) | NA | NA |
| Prevalence | Bahamas | 1990 | 1107.27(963.84to1280.03) | NA | NA |
| Prevalence | Bahamas | 2021 | 1320.88(1167.46to1488.52) | 19.29(9.19to30.97) | 0.45(0.3 to 0.59) |
| Deaths | Bahrain | 2021 | 35.37(30.06to40.52) | -44.64(-54.62to-34.75) | -2.94(-4.07 to -1.79) |
| Deaths | Bahrain | 1990 | 63.88(56.85to71.22) | NA | NA |
| DALYs (Disability-Adjusted Life Years) | Bahrain | 2021 | 681.46(591.17to765.26) | -43.38(-52.27to-34.28) | -2.23(-2.41 to -2.04) |
| DALYs (Disability-Adjusted Life Years) | Bahrain | 1990 | 1203.52(1091.35to1332.11) | NA | NA |
| Prevalence | Bahrain | 1990 | 2462.86(2192.99to2778.89) | NA | NA |
| Prevalence | Bahrain | 2021 | 2547.63(2251.17to2882.69) | 3.44(-3.47to11.34) | -0.18(-0.28 to -0.07) |
| Deaths | Bangladesh | 2021 | 59.65(46.23to77.22) | -41.49(-55.32to-23.34) | -2.37(-2.61 to -2.12) |
| Deaths | Bangladesh | 1990 | 101.94(77.88to125.49) | NA | NA |
| DALYs (Disability-Adjusted Life Years) | Bangladesh | 2021 | 1301.68(1050.53to1612.22) | -42.94(-54.9to-27.9) | -1.83(-1.96 to -1.7) |
| DALYs (Disability-Adjusted Life Years) | Bangladesh | 1990 | 2281.06(1801.47to2722.49) | NA | NA |
| Prevalence | Bangladesh | 1990 | 2914.65(2671.74to3206.63) | NA | NA |
| Prevalence | Bangladesh | 2021 | 2956.2(2669.12to3284.81) | 1.43(-4.04to7.17) | -0.12(-0.14 to -0.1) |
| Deaths | Barbados | 2021 | 7.52(5.97to9.05) | 11.92(-11.56to37.88) | -0.01(-5.33 to 5.6) |
| Deaths | Barbados | 1990 | 6.72(6.14to7.33) | NA | NA |
| DALYs (Disability-Adjusted Life Years) | Barbados | 2021 | 171.75(142.62to201.16) | 11.93(-7.94to31.21) | 0.18(-0.31 to 0.68) |
| DALYs (Disability-Adjusted Life Years) | Barbados | 1990 | 153.44(141.23to166.25) | NA | NA |
| Prevalence | Barbados | 1990 | 1043.02(904.91to1211.5) | NA | NA |
| Prevalence | Barbados | 2021 | 1305.44(1133.26to1483.27) | 25.16(15.17to35.44) | 0.55(0.41 to 0.7) |
| Deaths | Belarus | 2021 | 7.72(6.39to9.03) | -84.67(-87.25to-81.7) | -6.21(-6.66 to -5.76) |
| Deaths | Belarus | 1990 | 50.32(47.12to53.37) | NA | NA |
| DALYs (Disability-Adjusted Life Years) | Belarus | 1990 | 1022.26(963.46to1084.37) | NA | NA |
| DALYs (Disability-Adjusted Life Years) | Belarus | 2021 | 245.19(212.95to282.99) | -76.02(-79.4to-72.07) | -4.96(-5.08 to -4.83) |
| Prevalence | Belarus | 1990 | 2460.8(2209.37to2744.35) | NA | NA |
| Prevalence | Belarus | 2021 | 2046.92(1814.29to2305.15) | -16.82(-23.55to-10.65) | -0.68(-0.73 to -0.62) |
| Deaths | Belgium | 2021 | 21.42(18.54to22.99) | -37.56(-42.38to-33.89) | -1.58(-2.11 to -1.06) |
| Deaths | Belgium | 1990 | 34.3(31.64to36.26) | NA | NA |
| DALYs (Disability-Adjusted Life Years) | Belgium | 1990 | 691.93(649.94to728.78) | NA | NA |
| DALYs (Disability-Adjusted Life Years) | Belgium | 2021 | 477.58(437.24to512.88) | -30.98(-35.29to-27.12) | -1.15(-1.27 to -1.03) |
| Prevalence | Belgium | 1990 | 2495.23(2254.28to2763.21) | NA | NA |
| Prevalence | Belgium | 2021 | 2469.8(2225.42to2749.37) | -1.02(-7.23to6.39) | -0.12(-0.15 to -0.1) |
| Deaths | Belize | 2021 | 20.61(17.68to23.5) | 58(34.75to83.85) | 0.84(-1.81 to 3.55) |
| Deaths | Belize | 1990 | 13.05(11.9to14.18) | NA | NA |
| DALYs (Disability-Adjusted Life Years) | Belize | 2021 | 444.57(391.58to501.54) | 50.74(31to71.18) | 0.82(0.44 to 1.19) |
| DALYs (Disability-Adjusted Life Years) | Belize | 1990 | 294.92(271.92to318.85) | NA | NA |
| Prevalence | Belize | 1990 | 1454.85(1279.95to1642.28) | NA | NA |
| Prevalence | Belize | 2021 | 1746.82(1525.5to1967.8) | 20.07(8.16to32.96) | 0.56(0.4 to 0.72) |
| Deaths | Benin | 1990 | 36.54(27.58to44.63) | NA | NA |
| Deaths | Benin | 2021 | 24.12(18.28to31.85) | -34(-49.67to-8.55) | -1.01(-1.47 to -0.55) |
| DALYs (Disability-Adjusted Life Years) | Benin | 2021 | 604.11(487.65to760.4) | -28.24(-42.88to-8.45) | -0.72(-0.78 to -0.66) |
| DALYs (Disability-Adjusted Life Years) | Benin | 1990 | 841.84(663.23to993.94) | NA | NA |
| Prevalence | Benin | 1990 | 1626.94(1440.21to1848.18) | NA | NA |
| Prevalence | Benin | 2021 | 1705.2(1495.85to1929.75) | 4.81(-1.2to11.61) | 0.13(0.09 to 0.16) |
| Deaths | Bermuda | 1990 | 11.85(10.92to12.81) | NA | NA |
| Deaths | Bermuda | 2021 | 8.67(7.39to10.36) | -26.84(-38.39to-10.86) | -1.25(-10.67 to 9.16) |
| DALYs (Disability-Adjusted Life Years) | Bermuda | 1990 | 251.73(230.95to273.39) | NA | NA |
| DALYs (Disability-Adjusted Life Years) | Bermuda | 2021 | 198.74(171.34to230.34) | -21.05(-31.46to-8.69) | -0.77(-1.67 to 0.14) |
| Prevalence | Bermuda | 1990 | 1292.2(1115.61to1478.85) | NA | NA |
| Prevalence | Bermuda | 2021 | 1521.79(1309.73to1727.1) | 17.77(8.7to26.84) | 0.3(0.03 to 0.57) |
| Deaths | Bhutan | 1990 | 112.71(77.38to152.83) | NA | NA |
| Deaths | Bhutan | 2021 | 87.61(67.09to116.37) | -22.27(-43.79to21.59) | -2.2(-3.34 to -1.04) |
| DALYs (Disability-Adjusted Life Years) | Bhutan | 2021 | 1678.89(1317.32to2166) | -29.3(-47.29to3.47) | -1.67(-1.79 to -1.54) |
| DALYs (Disability-Adjusted Life Years) | Bhutan | 1990 | 2374.53(1683.6to3107.79) | NA | NA |
| Prevalence | Bhutan | 1990 | 2969.93(2681.69to3264.38) | NA | NA |
| Prevalence | Bhutan | 2021 | 2772.6(2481.15to3092.09) | -6.64(-12.3to-1.59) | -0.41(-0.48 to -0.33) |
| Deaths | Bolivia (Plurinational State of) | 2021 | 26.26(20.41to33.45) | -26.66(-41.62to-3.68) | -1.94(-2.38 to -1.49) |
| Deaths | Bolivia (Plurinational State of) | 1990 | 35.81(28.63to42.98) | NA | NA |
| DALYs (Disability-Adjusted Life Years) | Bolivia (Plurinational State of) | 1990 | 667.98(546.14to789.81) | NA | NA |
| DALYs (Disability-Adjusted Life Years) | Bolivia (Plurinational State of) | 2021 | 470.77(376.96to590.85) | -29.52(-42.97to-9.86) | -1.55(-1.61 to -1.49) |
| Prevalence | Bolivia (Plurinational State of) | 1990 | 1900.83(1672.36to2171.32) | NA | NA |
| Prevalence | Bolivia (Plurinational State of) | 2021 | 1967.35(1724.58to2233.81) | 3.5(-2.81to10.7) | -0.09(-0.12 to -0.06) |
| Deaths | Bosnia and Herzegovina | 2021 | 16.32(12.95to19.8) | -47.2(-58.45to-33.81) | -2.75(-3.61 to -1.88) |
| Deaths | Bosnia and Herzegovina | 1990 | 30.92(27.41to34.53) | NA | NA |
| DALYs (Disability-Adjusted Life Years) | Bosnia and Herzegovina | 2021 | 403.71(338.66to474.43) | -41.21(-50.75to-30.09) | -1.87(-2 to -1.73) |
| DALYs (Disability-Adjusted Life Years) | Bosnia and Herzegovina | 1990 | 686.75(620.88to752.25) | NA | NA |
| Prevalence | Bosnia and Herzegovina | 1990 | 2520.04(2246.7to2839.35) | NA | NA |
| Prevalence | Bosnia and Herzegovina | 2021 | 2584.7(2311.27to2896.99) | 2.57(-4.53to10.07) | -0.01(-0.04 to 0.02) |
| Deaths | Botswana | 2021 | 35.18(28.01to47.73) | -46.04(-59.81to-18.38) | -2.47(-3.28 to -1.66) |
| Deaths | Botswana | 1990 | 65.19(47.59to82.39) | NA | NA |
| DALYs (Disability-Adjusted Life Years) | Botswana | 2021 | 840.65(700.98to1049.95) | -40.5(-53.72to-19.53) | -1.77(-1.87 to -1.68) |
| DALYs (Disability-Adjusted Life Years) | Botswana | 1990 | 1412.78(1065.18to1757.2) | NA | NA |
| Prevalence | Botswana | 1990 | 1976.36(1749.2to2219.02) | NA | NA |
| Prevalence | Botswana | 2021 | 2016.93(1774.24to2287.47) | 2.05(-5.09to9.06) | 0(-0.06 to 0.06) |
| Deaths | Brazil | 1990 | 45.48(41.87to47.78) | NA | NA |
| Deaths | Brazil | 2021 | 26.49(23.59to28.19) | -41.75(-44.74to-39.03) | -2.29(-2.38 to -2.2) |
| DALYs (Disability-Adjusted Life Years) | Brazil | 1990 | 895.31(848.17to935.19) | NA | NA |
| DALYs (Disability-Adjusted Life Years) | Brazil | 2021 | 558.69(517.05to588.99) | -37.6(-40.14to-35.14) | -1.93(-1.98 to -1.87) |
| Prevalence | Brazil | 1990 | 2583.2(2281.62to2885.54) | NA | NA |
| Prevalence | Brazil | 2021 | 2555.99(2259.52to2860.37) | -1.05(-4to1.76) | -0.27(-0.28 to -0.25) |
| Deaths | Brunei Darussalam | 1990 | 63.76(53.82to75.88) | NA | NA |
| Deaths | Brunei Darussalam | 2021 | 33.64(28.55to39.83) | -47.25(-57.77to-33.79) | -1.98(-4.21 to 0.31) |
| DALYs (Disability-Adjusted Life Years) | Brunei Darussalam | 2021 | 595.82(519.54to686.73) | -47.78(-56.55to-35.97) | -1.87(-2.15 to -1.59) |
| DALYs (Disability-Adjusted Life Years) | Brunei Darussalam | 1990 | 1140.97(985.18to1313.3) | NA | NA |
| Prevalence | Brunei Darussalam | 1990 | 2272.34(2023.69to2530.72) | NA | NA |
| Prevalence | Brunei Darussalam | 2021 | 1662.05(1457.5to1879.28) | -26.86(-32.97to-20.83) | -1.25(-1.39 to -1.11) |
| Deaths | Bulgaria | 2021 | 15.24(13.01to17.69) | -51.38(-59.49to-40.75) | -2.03(-2.61 to -1.45) |
| Deaths | Bulgaria | 1990 | 31.35(28.09to34.98) | NA | NA |
| DALYs (Disability-Adjusted Life Years) | Bulgaria | 1990 | 672.25(608.95to740.13) | NA | NA |
| DALYs (Disability-Adjusted Life Years) | Bulgaria | 2021 | 394.48(344.83to447.94) | -41.32(-50.02to-30.19) | -1.52(-1.63 to -1.41) |
| Prevalence | Bulgaria | 1990 | 2475.06(2181.23to2745.96) | NA | NA |
| Prevalence | Bulgaria | 2021 | 2453.21(2164.24to2774.09) | -0.88(-8.11to7.53) | -0.09(-0.12 to -0.07) |
| Deaths | Burkina Faso | 1990 | 22.96(18.59to28.61) | NA | NA |
| Deaths | Burkina Faso | 2021 | 18.51(14.97to23.72) | -19.39(-37.38to3.76) | -0.52(-0.94 to -0.1) |
| DALYs (Disability-Adjusted Life Years) | Burkina Faso | 2021 | 491.7(416.02to586.83) | -13.49(-30.02to5.13) | -0.33(-0.38 to -0.28) |
| DALYs (Disability-Adjusted Life Years) | Burkina Faso | 1990 | 568.38(478.52to665.91) | NA | NA |
| Prevalence | Burkina Faso | 1990 | 1489.91(1304.23to1675.86) | NA | NA |
| Prevalence | Burkina Faso | 2021 | 1608.13(1425.7to1828.58) | 7.93(0.92to15.76) | 0.2(0.17 to 0.23) |
| Deaths | Burundi | 2021 | 37.72(26.31to48.99) | -35.89(-52.57to-11.33) | -2.02(-2.37 to -1.67) |
| Deaths | Burundi | 1990 | 58.84(41.78to73.08) | NA | NA |
| DALYs (Disability-Adjusted Life Years) | Burundi | 2021 | 877.53(646.02to1091.66) | -32.98(-48.45to-10.12) | -1.53(-1.58 to -1.49) |
| DALYs (Disability-Adjusted Life Years) | Burundi | 1990 | 1309.37(961.52to1595.74) | NA | NA |
| Prevalence | Burundi | 1990 | 1715.01(1520.02to1945.59) | NA | NA |
| Prevalence | Burundi | 2021 | 1878.33(1651.01to2110.58) | 9.52(1.51to18.16) | 0.14(0.11 to 0.17) |
| Deaths | Cabo Verde | 2021 | 16.21(12.25to19.86) | -56.75(-66.5to-41.43) | -2.74(-4.62 to -0.83) |
| Deaths | Cabo Verde | 1990 | 37.48(30.01to44.06) | NA | NA |
| DALYs (Disability-Adjusted Life Years) | Cabo Verde | 2021 | 388.2(317.27to462.46) | -51.6(-60.56to-37.84) | -1.97(-2.18 to -1.75) |
| DALYs (Disability-Adjusted Life Years) | Cabo Verde | 1990 | 802.09(669.17to926.73) | NA | NA |
| Prevalence | Cabo Verde | 1990 | 985.59(864.7to1112.81) | NA | NA |
| Prevalence | Cabo Verde | 2021 | 1001.29(872.55to1138.04) | 1.59(-4.79to8.31) | 0(-0.13 to 0.14) |
| Deaths | Cambodia | 2021 | 46.08(36.74to55.7) | -12.62(-35.31to29.01) | -1.1(-1.37 to -0.83) |
| Deaths | Cambodia | 1990 | 52.73(39.6to63.63) | NA | NA |
| DALYs (Disability-Adjusted Life Years) | Cambodia | 2021 | 935.16(771.66to1124.75) | -16.41(-35.39to15.65) | -0.82(-0.86 to -0.78) |
| DALYs (Disability-Adjusted Life Years) | Cambodia | 1990 | 1118.74(869.93to1325.37) | NA | NA |
| Prevalence | Cambodia | 1990 | 2167.17(1904.7to2447.99) | NA | NA |
| Prevalence | Cambodia | 2021 | 2230.67(1969.93to2529.52) | 2.93(-4.54to11.51) | -0.03(-0.05 to -0.01) |
| Deaths | Cameroon | 1990 | 38.6(29.89to47.21) | NA | NA |
| Deaths | Cameroon | 2021 | 24.78(19.62to32.22) | -35.79(-52.02to-9.93) | -1.07(-1.34 to -0.8) |
| DALYs (Disability-Adjusted Life Years) | Cameroon | 1990 | 863.65(704.13to1027.1) | NA | NA |
| DALYs (Disability-Adjusted Life Years) | Cameroon | 2021 | 620.09(506.5to770.63) | -28.2(-43.92to-3.8) | -0.8(-0.84 to -0.75) |
| Prevalence | Cameroon | 1990 | 1512.12(1324.99to1725.19) | NA | NA |
| Prevalence | Cameroon | 2021 | 1574.02(1383.41to1800.29) | 4.09(-1.7to11.44) | 0.07(0.04 to 0.1) |
| Deaths | Canada | 1990 | 24.54(22.47to25.86) | NA | NA |
| Deaths | Canada | 2021 | 18.46(16.12to19.95) | -24.77(-30.22to-19.43) | -0.67(-1.13 to -0.21) |
| DALYs (Disability-Adjusted Life Years) | Canada | 1990 | 511.66(478.41to544.39) | NA | NA |
| DALYs (Disability-Adjusted Life Years) | Canada | 2021 | 393.18(360.94to423.66) | -23.15(-28.25to-18.52) | -0.83(-0.91 to -0.74) |
| Prevalence | Canada | 1990 | 2076.16(1804.27to2371.24) | NA | NA |
| Prevalence | Canada | 2021 | 2098.38(1880.9to2351.54) | 1.07(-8.92to12.6) | -0.64(-0.71 to -0.57) |
| Deaths | Central African Republic | 1990 | 66.96(41.72to88.9) | NA | NA |
| Deaths | Central African Republic | 2021 | 55.87(32.76to82.29) | -16.56(-34.24to7.55) | -0.6(-1.05 to -0.14) |
| DALYs (Disability-Adjusted Life Years) | Central African Republic | 1990 | 1514.36(1022.39to1953.54) | NA | NA |
| DALYs (Disability-Adjusted Life Years) | Central African Republic | 2021 | 1302.89(863.86to1797.34) | -13.96(-29.66to7.04) | -0.46(-0.53 to -0.4) |
| Prevalence | Central African Republic | 1990 | 1941.74(1708.22to2209.27) | NA | NA |
| Prevalence | Central African Republic | 2021 | 2145.27(1877.74to2435.77) | 10.48(4.8to16.43) | 0.23(0.18 to 0.28) |
| Deaths | Chad | 2021 | 30.78(21.93to39.36) | -8.84(-28.71to21.3) | 0.02(-0.38 to 0.41) |
| Deaths | Chad | 1990 | 33.77(23.53to42.38) | NA | NA |
| DALYs (Disability-Adjusted Life Years) | Chad | 2021 | 763.57(583.64to942.36) | -4.92(-22.93to19.06) | 0.03(-0.02 to 0.08) |
| DALYs (Disability-Adjusted Life Years) | Chad | 1990 | 803.11(598.74to972.2) | NA | NA |
| Prevalence | Chad | 1990 | 1700.76(1485.88to1923.74) | NA | NA |
| Prevalence | Chad | 2021 | 1857.59(1625to2113.48) | 9.22(3.24to15.32) | 0.25(0.23 to 0.28) |
| Deaths | Chile | 1990 | 24.93(23.38to26.27) | NA | NA |
| Deaths | Chile | 2021 | 15.97(13.91to17.2) | -35.95(-41.41to-31.31) | -1.92(-2.31 to -1.52) |
| DALYs (Disability-Adjusted Life Years) | Chile | 1990 | 484.47(462.17to508.31) | NA | NA |
| DALYs (Disability-Adjusted Life Years) | Chile | 2021 | 282.02(256.06to301.72) | -41.79(-45.91to-38.11) | -1.67(-1.75 to -1.59) |
| Prevalence | Chile | 1990 | 1425.55(1247.9to1622.34) | NA | NA |
| Prevalence | Chile | 2021 | 1213.7(1060.34to1388.75) | -14.86(-24.01to-3.66) | -0.74(-0.77 to -0.71) |
| Deaths | China | 1990 | 231.78(198.98to257.42) | NA | NA |
| Deaths | China | 2021 | 73.23(59.73to86.85) | -68.4(-74.77to-60.49) | -5.05(-5.3 to -4.8) |
| DALYs (Disability-Adjusted Life Years) | China | 1990 | 3852.57(3349.97to4279.01) | NA | NA |
| DALYs (Disability-Adjusted Life Years) | China | 2021 | 1227.66(1048.45to1442.54) | -68.13(-74.22to-60.78) | -4.11(-4.19 to -4.02) |
| Prevalence | China | 1990 | 2761.81(2498.94to3033.6) | NA | NA |
| Prevalence | China | 2021 | 2499.35(2236.21to2793.29) | -9.5(-14.38to-5.17) | -0.56(-0.61 to -0.51) |
| Deaths | Colombia | 2021 | 30.38(25.16to35.39) | -18.72(-30.43to-6.41) | -2.39(-2.66 to -2.11) |
| Deaths | Colombia | 1990 | 37.37(34.87to39.08) | NA | NA |
| DALYs (Disability-Adjusted Life Years) | Colombia | 2021 | 559.45(477.79to640.18) | -26.7(-35.65to-16.95) | -1.85(-1.99 to -1.71) |
| DALYs (Disability-Adjusted Life Years) | Colombia | 1990 | 763.19(724.42to796.84) | NA | NA |
| Prevalence | Colombia | 1990 | 2273.38(2069.07to2538.12) | NA | NA |
| Prevalence | Colombia | 2021 | 2340.91(2106.1to2604.23) | 2.97(-4.44to10.2) | -0.33(-0.37 to -0.29) |
| Deaths | Comoros | 2021 | 26.32(18.25to34.84) | -38.34(-54.75to-14.31) | -1.75(-3.24 to -0.24) |
| Deaths | Comoros | 1990 | 42.69(29.03to57.27) | NA | NA |
| DALYs (Disability-Adjusted Life Years) | Comoros | 2021 | 615.46(455.41to774.81) | -34.77(-50.14to-12.67) | -1.35(-1.54 to -1.16) |
| DALYs (Disability-Adjusted Life Years) | Comoros | 1990 | 943.55(681.95to1232.71) | NA | NA |
| Prevalence | Comoros | 1990 | 1510.75(1332.76to1690.44) | NA | NA |
| Prevalence | Comoros | 2021 | 1492.5(1299.98to1696.45) | -1.21(-7.47to5.21) | -0.13(-0.25 to -0.01) |
| Deaths | Congo | 2021 | 36.18(28.3to47.33) | -39.04(-52.12to-12.88) | -2.02(-2.55 to -1.5) |
| Deaths | Congo | 1990 | 59.34(38.4to83.54) | NA | NA |
| DALYs (Disability-Adjusted Life Years) | Congo | 1990 | 1293.88(892.44to1768.41) | NA | NA |
| DALYs (Disability-Adjusted Life Years) | Congo | 2021 | 825.18(673.44to1037.16) | -36.22(-49.71to-11.43) | -1.63(-1.7 to -1.55) |
| Prevalence | Congo | 1990 | 1637.74(1442.4to1857.02) | NA | NA |
| Prevalence | Congo | 2021 | 1716.96(1501.89to1954.86) | 4.84(-1.38to12.49) | -0.01(-0.06 to 0.05) |
| Deaths | Cook Islands | 1990 | 53.54(43.81to62.64) | NA | NA |
| Deaths | Cook Islands | 2021 | 24.84(20.09to30.67) | -53.6(-64.76to-34.13) | -2.61(-9.84 to 5.2) |
| DALYs (Disability-Adjusted Life Years) | Cook Islands | 1990 | 1066.61(895.4to1222.27) | NA | NA |
| DALYs (Disability-Adjusted Life Years) | Cook Islands | 2021 | 534.64(449.7to630.68) | -49.88(-60.08to-32.91) | -2.12(-3 to -1.24) |
| Prevalence | Cook Islands | 1990 | 2082.01(1809.15to2349.32) | NA | NA |
| Prevalence | Cook Islands | 2021 | 1821.43(1557.93to2077.04) | -12.52(-21.16to-3.8) | -0.58(-1.04 to -0.13) |
| Deaths | Costa Rica | 2021 | 19.73(16.61to22.08) | -22.97(-31.79to-14.64) | -1.56(-2.39 to -0.72) |
| Deaths | Costa Rica | 1990 | 25.62(23.2to27.42) | NA | NA |
| DALYs (Disability-Adjusted Life Years) | Costa Rica | 2021 | 378.84(336.42to418.37) | -22.31(-30.19to-15.01) | -1.18(-1.29 to -1.07) |
| DALYs (Disability-Adjusted Life Years) | Costa Rica | 1990 | 487.63(451.54to518.91) | NA | NA |
| Prevalence | Costa Rica | 1990 | 1918.16(1683.24to2172.08) | NA | NA |
| Prevalence | Costa Rica | 2021 | 2098.78(1814.84to2368.32) | 9.42(-2.83to23.66) | 0.04(0.01 to 0.08) |
| Deaths | Croatia | 2021 | 19.05(16.72to21.23) | 0.31(-12.48to14.22) | -1.39(-2.42 to -0.35) |
| Deaths | Croatia | 1990 | 18.99(17.35to20.63) | NA | NA |
| DALYs (Disability-Adjusted Life Years) | Croatia | 2021 | 410.22(367.21to456.28) | -7.79(-16.79to2.31) | -0.65(-0.76 to -0.54) |
| DALYs (Disability-Adjusted Life Years) | Croatia | 1990 | 444.88(412.36to478.73) | NA | NA |
| Prevalence | Croatia | 1990 | 2238.98(1958.16to2529.54) | NA | NA |
| Prevalence | Croatia | 2021 | 2553.27(2289.68to2833.69) | 14.04(5.1to26.64) | 0.26(0.23 to 0.28) |
| Deaths | Cuba | 2021 | 22.57(19.78to25.41) | 41.61(23.14to60.85) | 0.32(-0.09 to 0.74) |
| Deaths | Cuba | 1990 | 15.94(14.97to16.8) | NA | NA |
| DALYs (Disability-Adjusted Life Years) | Cuba | 1990 | 356.98(335.91to378.45) | NA | NA |
| DALYs (Disability-Adjusted Life Years) | Cuba | 2021 | 501.11(445.8to556.48) | 40.37(25.05to56.44) | 0.39(0.22 to 0.55) |
| Prevalence | Cuba | 1990 | 1711.73(1497.19to1960.52) | NA | NA |
| Prevalence | Cuba | 2021 | 2139.36(1919.69to2374.51) | 24.98(12.22to41.82) | 0.49(0.47 to 0.52) |
| Deaths | Cyprus | 2021 | 27.02(22.71to31.62) | -55.21(-66.32to-32.65) | -2.75(-4.91 to -0.54) |
| Deaths | Cyprus | 1990 | 60.33(42.38to73.65) | NA | NA |
| DALYs (Disability-Adjusted Life Years) | Cyprus | 2021 | 455.12(393.6to521.74) | -50.59(-60.53to-31.99) | -1.76(-2.08 to -1.44) |
| DALYs (Disability-Adjusted Life Years) | Cyprus | 1990 | 921.04(690.89to1093.42) | NA | NA |
| Prevalence | Cyprus | 1990 | 2671.67(2387.23to3013.31) | NA | NA |
| Prevalence | Cyprus | 2021 | 2496.35(2221.78to2781.09) | -6.56(-13.62to0.94) | -0.27(-0.32 to -0.21) |
| Deaths | Czechia | 2021 | 16.6(14.24to18.8) | -15.34(-27.69to-0.97) | -0.64(-1.34 to 0.05) |
| Deaths | Czechia | 1990 | 19.6(17.99to21.68) | NA | NA |
| DALYs (Disability-Adjusted Life Years) | Czechia | 2021 | 414.71(371.23to462.55) | -14.35(-24.9to-2.46) | -0.09(-0.22 to 0.04) |
| DALYs (Disability-Adjusted Life Years) | Czechia | 1990 | 484.17(443.76to533.14) | NA | NA |
| Prevalence | Czechia | 1990 | 2107.39(1836.26to2393.4) | NA | NA |
| Prevalence | Czechia | 2021 | 2498.69(2264.56to2734.86) | 18.57(6.41to31.38) | 0.35(0.32 to 0.38) |
| Deaths | C么te d'Ivoire | 2021 | 24.43(19.57to30.76) | -33.7(-48.83to-13.57) | -1.04(-1.32 to -0.76) |
| Deaths | C么te d'Ivoire | 1990 | 36.85(29.86to44.76) | NA | NA |
| DALYs (Disability-Adjusted Life Years) | C么te d'Ivoire | 1990 | 842.6(704.68to1004.37) | NA | NA |
| DALYs (Disability-Adjusted Life Years) | C么te d'Ivoire | 2021 | 617.34(510.65to756.38) | -26.73(-40.82to-8.64) | -0.73(-0.77 to -0.68) |
| Prevalence | C么te d'Ivoire | 1990 | 1558.16(1377.48to1762.65) | NA | NA |
| Prevalence | C么te d'Ivoire | 2021 | 1691.67(1489.89to1934.8) | 8.57(1.9to15.17) | 0.28(0.25 to 0.31) |
| Deaths | Democratic People's Republic of Korea | 2021 | 107.68(80.67to156.7) | -31.87(-47.17to-12.61) | -1.04(-1.19 to -0.89) |
| Deaths | Democratic People's Republic of Korea | 1990 | 158.04(111.71to221.04) | NA | NA |
| DALYs (Disability-Adjusted Life Years) | Democratic People's Republic of Korea | 1990 | 2799.28(2073.73to3681.84) | NA | NA |
| DALYs (Disability-Adjusted Life Years) | Democratic People's Republic of Korea | 2021 | 1968.2(1500.48to2612.25) | -29.69(-44.96to-10.2) | -0.99(-1.05 to -0.94) |
| Prevalence | Democratic People's Republic of Korea | 1990 | 3166.45(2930.4to3426.85) | NA | NA |
| Prevalence | Democratic People's Republic of Korea | 2021 | 2757.5(2487.07to3066.78) | -12.92(-18.45to-6.87) | -0.52(-0.55 to -0.49) |
| Deaths | Democratic Republic of the Congo | 2021 | 47.53(29.56to79.56) | -8.24(-35.9to23.47) | -0.22(-0.37 to -0.08) |
| Deaths | Democratic Republic of the Congo | 1990 | 51.79(36.1to74.79) | NA | NA |
| DALYs (Disability-Adjusted Life Years) | Democratic Republic of the Congo | 1990 | 1125.72(811.56to1536.86) | NA | NA |
| DALYs (Disability-Adjusted Life Years) | Democratic Republic of the Congo | 2021 | 1073.41(720.28to1615.37) | -4.65(-28.24to22.83) | -0.13(-0.16 to -0.1) |
| Prevalence | Democratic Republic of the Congo | 1990 | 1756.13(1554.01to1980.06) | NA | NA |
| Prevalence | Democratic Republic of the Congo | 2021 | 2029.81(1800.47to2308.63) | 15.58(8.44to22.59) | 0.33(0.31 to 0.35) |
| Deaths | Denmark | 2021 | 35.43(31.37to38.12) | 2.96(-6.29to10.35) | -1.33(-2 to -0.65) |
| Deaths | Denmark | 1990 | 34.41(32.4to36.25) | NA | NA |
| DALYs (Disability-Adjusted Life Years) | Denmark | 2021 | 682.66(625.85to729.99) | -14.46(-20.41to-9.43) | -1.04(-1.27 to -0.81) |
| DALYs (Disability-Adjusted Life Years) | Denmark | 1990 | 798.05(757.87to841.24) | NA | NA |
| Prevalence | Denmark | 1990 | 2941.77(2648.48to3252.83) | NA | NA |
| Prevalence | Denmark | 2021 | 2732.96(2448.7to3009.8) | -7.1(-14.14to0.53) | -0.45(-0.51 to -0.39) |
| Deaths | Djibouti | 2021 | 20.48(13.05to29.2) | -34.87(-53.81to-5.41) | -1.6(-3.22 to 0.05) |
| Deaths | Djibouti | 1990 | 31.44(22to41.79) | NA | NA |
| DALYs (Disability-Adjusted Life Years) | Djibouti | 1990 | 704.2(516.7to903.3) | NA | NA |
| DALYs (Disability-Adjusted Life Years) | Djibouti | 2021 | 506.44(362.23to680.9) | -28.08(-45.7to-2.31) | -1.04(-1.26 to -0.82) |
| Prevalence | Djibouti | 1990 | 1318.58(1146.93to1488.6) | NA | NA |
| Prevalence | Djibouti | 2021 | 1412.18(1230.01to1602.95) | 7.1(1.13to13.86) | 0.15(0 to 0.3) |
| Deaths | Dominica | 2021 | 17.85(15.3to20.95) | -0.19(-19.25to22.57) | 0.52(-6.39 to 7.94) |
| Deaths | Dominica | 1990 | 17.89(15.79to20.24) | NA | NA |
| DALYs (Disability-Adjusted Life Years) | Dominica | 1990 | 359.73(319.34to400.09) | NA | NA |
| DALYs (Disability-Adjusted Life Years) | Dominica | 2021 | 373.21(326.91to438.9) | 3.75(-13.72to24.97) | 0.36(-0.41 to 1.15) |
| Prevalence | Dominica | 1990 | 1282.53(1121.49to1462.43) | NA | NA |
| Prevalence | Dominica | 2021 | 1451.22(1241.72to1668.9) | 13.15(4.1to20.54) | 0.31(0.02 to 0.59) |
| Deaths | Dominican Republic | 2021 | 13.11(9.55to20.04) | -12.09(-38.83to34.46) | 0.8(0.23 to 1.37) |
| Deaths | Dominican Republic | 1990 | 14.91(12.56to17.47) | NA | NA |
| DALYs (Disability-Adjusted Life Years) | Dominican Republic | 1990 | 288.02(250.75to333.46) | NA | NA |
| DALYs (Disability-Adjusted Life Years) | Dominican Republic | 2021 | 294.88(229.69to409.07) | 2.38(-22.96to44.23) | 0.62(0.44 to 0.8) |
| Prevalence | Dominican Republic | 1990 | 1346.75(1182.26to1524.59) | NA | NA |
| Prevalence | Dominican Republic | 2021 | 1680.84(1452.7to1916.87) | 24.81(15.96to34.51) | 0.64(0.61 to 0.66) |
| Deaths | Ecuador | 2021 | 17.43(14.6to20.63) | -34.65(-45.11to-23.08) | -1.88(-2.31 to -1.45) |
| Deaths | Ecuador | 1990 | 26.68(24.77to28.18) | NA | NA |
| DALYs (Disability-Adjusted Life Years) | Ecuador | 1990 | 454.07(427to478.81) | NA | NA |
| DALYs (Disability-Adjusted Life Years) | Ecuador | 2021 | 296.86(256.18to345.1) | -34.62(-43.94to-24.28) | -1.34(-1.47 to -1.21) |
| Prevalence | Ecuador | 1990 | 1588.31(1376.19to1817.78) | NA | NA |
| Prevalence | Ecuador | 2021 | 1737.48(1521.87to1977.78) | 9.39(-3.62to24.93) | 0(-0.04 to 0.03) |
| Deaths | Egypt | 2021 | 25.87(21.51to31.06) | -46.42(-58.57to-28.03) | -2.62(-2.78 to -2.46) |
| Deaths | Egypt | 1990 | 48.3(40to55.61) | NA | NA |
| DALYs (Disability-Adjusted Life Years) | Egypt | 1990 | 1015.46(857.09to1138.64) | NA | NA |
| DALYs (Disability-Adjusted Life Years) | Egypt | 2021 | 602.17(511.84to700.27) | -40.7(-51.99to-24.56) | -1.89(-1.98 to -1.79) |
| Prevalence | Egypt | 1990 | 1868.22(1644.89to2143.63) | NA | NA |
| Prevalence | Egypt | 2021 | 2472.66(2161.71to2806.85) | 32.35(23.55to41.79) | 0.66(0.64 to 0.67) |
| Deaths | El Salvador | 2021 | 17.68(13.73to22.56) | -26.17(-44.28to-3.06) | -1.12(-1.75 to -0.48) |
| Deaths | El Salvador | 1990 | 23.95(20.99to27.55) | NA | NA |
| DALYs (Disability-Adjusted Life Years) | El Salvador | 2021 | 361.12(294.3to437.05) | -23.58(-40.2to-2.81) | -0.88(-0.96 to -0.81) |
| DALYs (Disability-Adjusted Life Years) | El Salvador | 1990 | 472.52(419.31to539.06) | NA | NA |
| Prevalence | El Salvador | 1990 | 1670.1(1452.44to1901.53) | NA | NA |
| Prevalence | El Salvador | 2021 | 1855.16(1599.69to2125.36) | 11.08(-0.36to26.59) | 0.03(-0.01 to 0.06) |
| Deaths | Equatorial Guinea | 2021 | 25.39(17.97to36.81) | -55.47(-71.33to-14.19) | -3.11(-4.47 to -1.73) |
| Deaths | Equatorial Guinea | 1990 | 57.01(39.01to76.69) | NA | NA |
| DALYs (Disability-Adjusted Life Years) | Equatorial Guinea | 1990 | 1292.92(929.68to1703.85) | NA | NA |
| DALYs (Disability-Adjusted Life Years) | Equatorial Guinea | 2021 | 613.98(458.71to833.3) | -52.51(-67.43to-16.47) | -2.4(-2.57 to -2.23) |
| Prevalence | Equatorial Guinea | 1990 | 1835.02(1614.19to2079.1) | NA | NA |
| Prevalence | Equatorial Guinea | 2021 | 1732.08(1522.72to1979.46) | -5.61(-11.17to0.46) | -0.28(-0.39 to -0.16) |
| Deaths | Eritrea | 1990 | 51.09(32.89to66.81) | NA | NA |
| Deaths | Eritrea | 2021 | 36.51(28.01to46.83) | -28.55(-43.74to3.83) | -1.38(-1.85 to -0.91) |
| DALYs (Disability-Adjusted Life Years) | Eritrea | 2021 | 844.47(676.17to1047.84) | -29.8(-42.52to-7.13) | -1.12(-1.2 to -1.04) |
| DALYs (Disability-Adjusted Life Years) | Eritrea | 1990 | 1202.89(861.07to1497.37) | NA | NA |
| Prevalence | Eritrea | 1990 | 1529.39(1341.13to1714.28) | NA | NA |
| Prevalence | Eritrea | 2021 | 1543.62(1344.22to1764.93) | 0.93(-7.08to8.46) | -0.04(-0.11 to 0.02) |
| Deaths | Estonia | 2021 | 6.13(5.33to6.91) | -36.36(-45.13to-27.8) | -2.97(-5.63 to -0.24) |
| Deaths | Estonia | 1990 | 9.64(8.98to10.33) | NA | NA |
| DALYs (Disability-Adjusted Life Years) | Estonia | 2021 | 170.09(151.14to190.77) | -32.49(-39.82to-25.1) | -1.58(-1.77 to -1.39) |
| DALYs (Disability-Adjusted Life Years) | Estonia | 1990 | 251.93(233.44to271.78) | NA | NA |
| Prevalence | Estonia | 1990 | 1352.47(1200.44to1516.22) | NA | NA |
| Prevalence | Estonia | 2021 | 1532.4(1367.82to1728.88) | 13.3(4.66to24.4) | 0.4(0.35 to 0.46) |
| Deaths | Eswatini | 1990 | 68.8(46.08to89.86) | NA | NA |
| Deaths | Eswatini | 2021 | 52.37(38.51to68.74) | -23.87(-42.17to4.01) | -0.31(-1.2 to 0.58) |
| DALYs (Disability-Adjusted Life Years) | Eswatini | 1990 | 1513.96(1073.79to1893.29) | NA | NA |
| DALYs (Disability-Adjusted Life Years) | Eswatini | 2021 | 1264.8(979.92to1626.08) | -16.46(-35.28to12.89) | -0.25(-0.38 to -0.12) |
| Prevalence | Eswatini | 1990 | 1932.31(1697.17to2193.9) | NA | NA |
| Prevalence | Eswatini | 2021 | 1921.59(1707.84to2173.55) | -0.56(-7.2to6.92) | -0.08(-0.17 to 0.01) |
| Deaths | Ethiopia | 1990 | 44.91(31.46to54.86) | NA | NA |
| Deaths | Ethiopia | 2021 | 25.4(20.27to30.05) | -43.46(-55.47to-27.27) | -2.73(-2.87 to -2.58) |
| DALYs (Disability-Adjusted Life Years) | Ethiopia | 2021 | 619.18(510.02to708.79) | -43.37(-53.21to-30.49) | -1.99(-2.07 to -1.92) |
| DALYs (Disability-Adjusted Life Years) | Ethiopia | 1990 | 1093.38(817.17to1295.81) | NA | NA |
| Prevalence | Ethiopia | 1990 | 1821(1601.44to2053.96) | NA | NA |
| Prevalence | Ethiopia | 2021 | 1715.27(1509.18to1918.44) | -5.81(-9.92to-1.61) | -0.28(-0.34 to -0.22) |
| Deaths | Fiji | 2021 | 38.5(29.31to47.65) | -36.98(-53.99to-15.65) | -2.08(-2.99 to -1.16) |
| Deaths | Fiji | 1990 | 61.09(51.15to72.5) | NA | NA |
| DALYs (Disability-Adjusted Life Years) | Fiji | 2021 | 778.59(613.56to940.77) | -37.9(-53.31to-18.85) | -1.85(-1.98 to -1.73) |
| DALYs (Disability-Adjusted Life Years) | Fiji | 1990 | 1253.86(1066.77to1462.49) | NA | NA |
| Prevalence | Fiji | 1990 | 2100.68(1843.83to2381.55) | NA | NA |
| Prevalence | Fiji | 2021 | 1655.84(1446.42to1890.83) | -21.18(-27.44to-15.11) | -0.79(-0.88 to -0.71) |
| Deaths | Finland | 2021 | 9.9(8.74to10.74) | -20.21(-28.02to-13.25) | -1.55(-2.77 to -0.31) |
| Deaths | Finland | 1990 | 12.4(11.52to13.23) | NA | NA |
| DALYs (Disability-Adjusted Life Years) | Finland | 1990 | 322.94(301.51to347.53) | NA | NA |
| DALYs (Disability-Adjusted Life Years) | Finland | 2021 | 259.79(237.73to283.43) | -19.55(-25.48to-13.97) | -0.9(-1.01 to -0.79) |
| Prevalence | Finland | 1990 | 2125.14(1943.43to2326.77) | NA | NA |
| Prevalence | Finland | 2021 | 2133.03(1886.93to2393.42) | 0.37(-8.12to10.48) | -0.26(-0.31 to -0.22) |
| Deaths | France | 2021 | 8.9(7.7to9.66) | -51.63(-55.23to-47.94) | -2.08(-2.51 to -1.64) |
| Deaths | France | 1990 | 18.4(16.77to19.5) | NA | NA |
| DALYs (Disability-Adjusted Life Years) | France | 2021 | 208.64(188.85to228.62) | -39.49(-43.67to-35.4) | -1.26(-1.44 to -1.09) |
| DALYs (Disability-Adjusted Life Years) | France | 1990 | 344.81(319.26to368.38) | NA | NA |
| Prevalence | France | 1990 | 2255.01(2001.12to2522.03) | NA | NA |
| Prevalence | France | 2021 | 2182.53(1950.2to2438.77) | -3.21(-11.08to5.85) | -0.11(-0.15 to -0.07) |
| Deaths | Gabon | 2021 | 26.25(19.14to37.88) | -41.79(-60.77to-9.47) | -1.98(-3.05 to -0.89) |
| Deaths | Gabon | 1990 | 45.09(35.44to57.11) | NA | NA |
| DALYs (Disability-Adjusted Life Years) | Gabon | 2021 | 616.69(481.4to822.05) | -36.45(-53.47to-8.88) | -1.43(-1.55 to -1.3) |
| DALYs (Disability-Adjusted Life Years) | Gabon | 1990 | 970.41(788.18to1196.51) | NA | NA |
| Prevalence | Gabon | 1990 | 1391.46(1210.2to1575.16) | NA | NA |
| Prevalence | Gabon | 2021 | 1530.9(1330.61to1767.76) | 10.02(3.6to17.75) | 0.21(0.13 to 0.28) |
| Deaths | Gambia | 2021 | 30.68(21.08to41.46) | -14.38(-40.38to17.64) | -0.18(-1.13 to 0.78) |
| Deaths | Gambia | 1990 | 35.83(26.74to45.23) | NA | NA |
| DALYs (Disability-Adjusted Life Years) | Gambia | 1990 | 839.39(665.37to1026.54) | NA | NA |
| DALYs (Disability-Adjusted Life Years) | Gambia | 2021 | 744.46(564.13to962.59) | -11.31(-32.66to15.81) | -0.12(-0.25 to 0.01) |
| Prevalence | Gambia | 1990 | 1629.89(1430.6to1848.06) | NA | NA |
| Prevalence | Gambia | 2021 | 1778.31(1574.41to2005.21) | 9.11(3.23to16.85) | 0.27(0.19 to 0.35) |
| Deaths | Georgia | 1990 | 9.83(8.49to11.24) | NA | NA |
| Deaths | Georgia | 2021 | 10.97(9.36to12.74) | 11.63(-8.78to37.05) | 1.45(0.53 to 2.39) |
| DALYs (Disability-Adjusted Life Years) | Georgia | 2021 | 295.55(261.43to333.88) | 16.46(0.28to35.83) | 1.31(1.12 to 1.49) |
| DALYs (Disability-Adjusted Life Years) | Georgia | 1990 | 253.78(227.99to281.96) | NA | NA |
| Prevalence | Georgia | 1990 | 1782.14(1561.27to2030.01) | NA | NA |
| Prevalence | Georgia | 2021 | 2091.88(1844.96to2352.67) | 17.38(3.52to31.33) | 0.46(0.42 to 0.5) |
| Deaths | Germany | 1990 | 21.49(19.6to22.75) | NA | NA |
| Deaths | Germany | 2021 | 18.28(16.51to19.46) | -14.92(-20.9to-9.5) | -0.34(-0.63 to -0.04) |
| DALYs (Disability-Adjusted Life Years) | Germany | 1990 | 489.59(455.52to521.56) | NA | NA |
| DALYs (Disability-Adjusted Life Years) | Germany | 2021 | 442.86(409.16to475.53) | -9.54(-14.97to-4.24) | -0.19(-0.28 to -0.09) |
| Prevalence | Germany | 1990 | 2873.29(2587.82to3205.01) | NA | NA |
| Prevalence | Germany | 2021 | 2759.62(2508.82to3043.39) | -3.96(-12.19to3.59) | -0.14(-0.18 to -0.1) |
| Deaths | Ghana | 2021 | 13.85(10.97to17.07) | -7.35(-30.82to25.87) | 0.06(-0.27 to 0.39) |
| Deaths | Ghana | 1990 | 14.94(12.03to18.21) | NA | NA |
| DALYs (Disability-Adjusted Life Years) | Ghana | 1990 | 410.5(348.32to481.99) | NA | NA |
| DALYs (Disability-Adjusted Life Years) | Ghana | 2021 | 411.82(344.86to486) | 0.32(-18.17to24.54) | 0.22(0.17 to 0.27) |
| Prevalence | Ghana | 1990 | 1438.67(1260.06to1614.25) | NA | NA |
| Prevalence | Ghana | 2021 | 1672.26(1471.4to1876.37) | 16.24(9.56to23.2) | 0.43(0.41 to 0.45) |
| Deaths | Greece | 1990 | 14.55(13.27to15.42) | NA | NA |
| Deaths | Greece | 2021 | 14.98(13.08to16.14) | 2.96(-3.33to9.08) | 0.37(-0.26 to 1.01) |
| DALYs (Disability-Adjusted Life Years) | Greece | 2021 | 380.35(340.27to424.19) | 4.67(-2.75to12.73) | 0.39(0.23 to 0.56) |
| DALYs (Disability-Adjusted Life Years) | Greece | 1990 | 363.4(330.46to398.37) | NA | NA |
| Prevalence | Greece | 1990 | 2563.04(2260.52to2891.06) | NA | NA |
| Prevalence | Greece | 2021 | 2575.73(2325.26to2864.41) | 0.5(-8.41to11.06) | -0.02(-0.03 to 0) |
| Deaths | Greenland | 1990 | 85.37(69.16to97.02) | NA | NA |
| Deaths | Greenland | 2021 | 42.98(34.48to52.82) | -49.66(-58.7to-37.13) | -1.8(-9.81 to 6.92) |
| DALYs (Disability-Adjusted Life Years) | Greenland | 1990 | 1724.4(1446.21to1936.51) | NA | NA |
| DALYs (Disability-Adjusted Life Years) | Greenland | 2021 | 907.57(779.86to1067.9) | -47.37(-55.33to-36.29) | -1.5(-2.2 to -0.79) |
| Prevalence | Greenland | 1990 | 2728.23(2491.3to3005.96) | NA | NA |
| Prevalence | Greenland | 2021 | 2542.79(2306.06to2822.86) | -6.8(-12.83to0.49) | -0.26(-0.54 to 0.03) |
| Deaths | Grenada | 2021 | 13.62(11.91to15.08) | 20.84(2.5to39.86) | 0.33(-5.22 to 6.21) |
| Deaths | Grenada | 1990 | 11.27(10.25to12.41) | NA | NA |
| DALYs (Disability-Adjusted Life Years) | Grenada | 1990 | 260.65(237.21to286.14) | NA | NA |
| DALYs (Disability-Adjusted Life Years) | Grenada | 2021 | 310.32(274.6to343.63) | 19.06(3to36.08) | 0.3(-0.36 to 0.96) |
| Prevalence | Grenada | 1990 | 1241.39(1076.4to1414.06) | NA | NA |
| Prevalence | Grenada | 2021 | 1482.2(1299.19to1658.69) | 19.4(10.45to30.08) | 0.42(0.17 to 0.66) |
| Deaths | Guam | 1990 | 44.85(39.55to49.83) | NA | NA |
| Deaths | Guam | 2021 | 13.64(11.57to15.55) | -69.58(-74.33to-64.7) | -1.89(-4.53 to 0.81) |
| DALYs (Disability-Adjusted Life Years) | Guam | 2021 | 404.98(359.51to450.33) | -53.03(-59.11to-46.94) | -1.55(-1.88 to -1.22) |
| DALYs (Disability-Adjusted Life Years) | Guam | 1990 | 862.24(786.16to943.49) | NA | NA |
| Prevalence | Guam | 1990 | 1821.17(1564.65to2098.26) | NA | NA |
| Prevalence | Guam | 2021 | 1623.56(1387.66to1836.26) | -10.85(-18.63to-2.75) | -0.35(-0.53 to -0.17) |
| Deaths | Guatemala | 2021 | 17.78(15.57to20.01) | -36.72(-45.51to-26.69) | -1.57(-2.02 to -1.11) |
| Deaths | Guatemala | 1990 | 28.1(25.61to30.19) | NA | NA |
| DALYs (Disability-Adjusted Life Years) | Guatemala | 2021 | 348.63(307.48to392.08) | -31.99(-40.9to-21.79) | -1.29(-1.4 to -1.17) |
| DALYs (Disability-Adjusted Life Years) | Guatemala | 1990 | 512.58(471to552.44) | NA | NA |
| Prevalence | Guatemala | 1990 | 1803.39(1584.05to2040.03) | NA | NA |
| Prevalence | Guatemala | 2021 | 1906.12(1694.12to2164.38) | 5.7(-7.98to21.64) | -0.13(-0.16 to -0.11) |
| Deaths | Guinea | 2021 | 28.71(21.16to37.24) | -17.52(-40.23to28.48) | -0.14(-0.54 to 0.27) |
| Deaths | Guinea | 1990 | 34.81(23.81to44.76) | NA | NA |
| DALYs (Disability-Adjusted Life Years) | Guinea | 1990 | 802.39(581.56to985.96) | NA | NA |
| DALYs (Disability-Adjusted Life Years) | Guinea | 2021 | 713.48(558.98to891.5) | -11.08(-31.48to24.66) | -0.03(-0.08 to 0.02) |
| Prevalence | Guinea | 1990 | 1618.35(1424.4to1837.1) | NA | NA |
| Prevalence | Guinea | 2021 | 1842.2(1625.56to2067.62) | 13.83(5.99to22.55) | 0.39(0.36 to 0.41) |
| Deaths | Guinea-Bissau | 2021 | 36.12(24.36to45.93) | -29.53(-48.24to-6.64) | -0.71(-1.52 to 0.1) |
| Deaths | Guinea-Bissau | 1990 | 51.26(37.48to64.95) | NA | NA |
| DALYs (Disability-Adjusted Life Years) | Guinea-Bissau | 1990 | 1216.2(911.72to1492.77) | NA | NA |
| DALYs (Disability-Adjusted Life Years) | Guinea-Bissau | 2021 | 887.63(660.38to1092.06) | -27.02(-44.19to-5.73) | -0.56(-0.68 to -0.44) |
| Prevalence | Guinea-Bissau | 1990 | 1666.21(1477.1to1877.54) | NA | NA |
| Prevalence | Guinea-Bissau | 2021 | 1757.8(1541.23to2001.48) | 5.5(-0.61to12.15) | 0.18(0.09 to 0.27) |
| Deaths | Guyana | 2021 | 14.2(11.19to17.72) | 26.23(-2.22to58.42) | 1.71(-0.17 to 3.61) |
| Deaths | Guyana | 1990 | 11.25(10.15to12.42) | NA | NA |
| DALYs (Disability-Adjusted Life Years) | Guyana | 2021 | 336.02(271.46to415.63) | 34.81(6.31to67.38) | 1.41(1.17 to 1.66) |
| DALYs (Disability-Adjusted Life Years) | Guyana | 1990 | 249.26(226.01to272.73) | NA | NA |
| Prevalence | Guyana | 1990 | 1163.95(1006.46to1333.4) | NA | NA |
| Prevalence | Guyana | 2021 | 1411.33(1215.33to1605.97) | 21.25(12.92to31.52) | 0.48(0.38 to 0.58) |
| Deaths | Haiti | 1990 | 39.14(17.45to56.79) | NA | NA |
| Deaths | Haiti | 2021 | 35.24(16.08to53.06) | -9.97(-31.11to22.51) | -0.27(-0.63 to 0.09) |
| DALYs (Disability-Adjusted Life Years) | Haiti | 2021 | 728.06(356.16to1071.15) | -11.18(-32.85to19.54) | -0.24(-0.29 to -0.19) |
| DALYs (Disability-Adjusted Life Years) | Haiti | 1990 | 819.75(392.57to1165.65) | NA | NA |
| Prevalence | Haiti | 1990 | 1777.03(1555.84to2034.88) | NA | NA |
| Prevalence | Haiti | 2021 | 1909.4(1687.77to2174.25) | 7.45(1.93to13.94) | 0.05(0.03 to 0.08) |
| Deaths | Honduras | 2021 | 56.63(44.76to70.3) | 25.59(-3.7to56.62) | -0.22(-0.58 to 0.15) |
| Deaths | Honduras | 1990 | 45.09(37.38to56.31) | NA | NA |
| DALYs (Disability-Adjusted Life Years) | Honduras | 1990 | 887.48(746.75to1076.22) | NA | NA |
| DALYs (Disability-Adjusted Life Years) | Honduras | 2021 | 1049.64(846.1to1295.33) | 18.27(-8.92to47.32) | -0.16(-0.31 to -0.01) |
| Prevalence | Honduras | 1990 | 2128.56(1864.34to2401.8) | NA | NA |
| Prevalence | Honduras | 2021 | 2521.81(2240.28to2870.89) | 18.48(11.08to26.21) | 0.26(0.24 to 0.29) |
| Deaths | Hungary | 1990 | 34.89(32.75to37.07) | NA | NA |
| Deaths | Hungary | 2021 | 29.25(25.55to33.28) | -16.17(-26.54to-4.58) | -0.48(-0.9 to -0.05) |
| DALYs (Disability-Adjusted Life Years) | Hungary | 1990 | 776.75(734.04to826.31) | NA | NA |
| DALYs (Disability-Adjusted Life Years) | Hungary | 2021 | 744.21(658.45to834.29) | -4.19(-15.21to7.72) | -0.23(-0.38 to -0.08) |
| Prevalence | Hungary | 1990 | 2561.16(2274.07to2884.03) | NA | NA |
| Prevalence | Hungary | 2021 | 2933.74(2673.56to3185.9) | 14.55(3.69to27.06) | 0.31(0.29 to 0.33) |
| Deaths | Iceland | 1990 | 19.31(17.29to20.73) | NA | NA |
| Deaths | Iceland | 2021 | 16.87(14.11to18.77) | -12.63(-21.74to-3) | -0.58(-4.23 to 3.21) |
| DALYs (Disability-Adjusted Life Years) | Iceland | 2021 | 397.5(353.63to437.69) | -16.82(-23.09to-10.4) | -0.67(-0.97 to -0.37) |
| DALYs (Disability-Adjusted Life Years) | Iceland | 1990 | 477.9(440.4to517.3) | NA | NA |
| Prevalence | Iceland | 1990 | 3092.11(2792.54to3400.82) | NA | NA |
| Prevalence | Iceland | 2021 | 2769.55(2492.45to3053.54) | -10.43(-16.81to-2.43) | -0.54(-0.63 to -0.45) |
| Deaths | India | 1990 | 119.72(92.9to144.99) | NA | NA |
| Deaths | India | 2021 | 108.39(94.73to122.39) | -9.47(-25.48to19.28) | -0.97(-1.23 to -0.7) |
| DALYs (Disability-Adjusted Life Years) | India | 2021 | 2171.16(1953.69to2422.39) | -13.46(-26.7to10.14) | -0.67(-0.79 to -0.55) |
| DALYs (Disability-Adjusted Life Years) | India | 1990 | 2508.84(1975.51to2965.31) | NA | NA |
| Prevalence | India | 1990 | 2967.33(2676to3242.41) | NA | NA |
| Prevalence | India | 2021 | 3067.86(2782.53to3347.91) | 3.39(1.38to5.24) | 0.03(0.02 to 0.04) |
| Deaths | Indonesia | 2021 | 49.53(40.95to58.23) | -6.55(-26.94to42.55) | -0.54(-0.59 to -0.48) |
| Deaths | Indonesia | 1990 | 53(37.97to61.85) | NA | NA |
| DALYs (Disability-Adjusted Life Years) | Indonesia | 1990 | 1140.65(870.7to1312.2) | NA | NA |
| DALYs (Disability-Adjusted Life Years) | Indonesia | 2021 | 1040.41(874.72to1216) | -8.79(-26.58to31.08) | -0.41(-0.44 to -0.37) |
| Prevalence | Indonesia | 1990 | 1972.09(1735.91to2218.39) | NA | NA |
| Prevalence | Indonesia | 2021 | 2204.76(1930.54to2481.68) | 11.8(8.86to14.9) | 0.17(0.15 to 0.19) |
| Deaths | Iran (Islamic Republic of) | 2021 | 17.44(15.06to19.2) | -16.99(-30.32to7.36) | -0.6(-0.75 to -0.45) |
| Deaths | Iran (Islamic Republic of) | 1990 | 21.01(17.12to24.08) | NA | NA |
| DALYs (Disability-Adjusted Life Years) | Iran (Islamic Republic of) | 2021 | 424.08(383.16to462.24) | -11.38(-23.48to8.16) | -0.27(-0.34 to -0.2) |
| DALYs (Disability-Adjusted Life Years) | Iran (Islamic Republic of) | 1990 | 478.53(405.07to540.13) | NA | NA |
| Prevalence | Iran (Islamic Republic of) | 1990 | 1611.84(1415.39to1817.15) | NA | NA |
| Prevalence | Iran (Islamic Republic of) | 2021 | 2054.63(1798.37to2321.91) | 27.47(22.51to32.12) | 0.53(0.5 to 0.56) |
| Deaths | Iraq | 2021 | 11.44(8.67to14.41) | -5.75(-33.01to36.5) | -1.5(-1.81 to -1.18) |
| Deaths | Iraq | 1990 | 12.14(9.33to15.58) | NA | NA |
| DALYs (Disability-Adjusted Life Years) | Iraq | 2021 | 320.73(262.89to381.13) | -6.18(-25.64to22.99) | -0.67(-0.77 to -0.57) |
| DALYs (Disability-Adjusted Life Years) | Iraq | 1990 | 341.87(280.61to408.72) | NA | NA |
| Prevalence | Iraq | 1990 | 1675.15(1466.3to1924.39) | NA | NA |
| Prevalence | Iraq | 2021 | 2003.35(1756.16to2283.54) | 19.59(11.22to29.24) | 0.42(0.39 to 0.45) |
| Deaths | Ireland | 1990 | 51.56(48.4to54.2) | NA | NA |
| Deaths | Ireland | 2021 | 23.26(20.03to25.66) | -54.89(-59.49to-50.92) | -2.97(-3.75 to -2.19) |
| DALYs (Disability-Adjusted Life Years) | Ireland | 2021 | 451.69(406.89to492.35) | -53.68(-57.15to-50.2) | -2.29(-2.51 to -2.08) |
| DALYs (Disability-Adjusted Life Years) | Ireland | 1990 | 975.14(917.99to1023.41) | NA | NA |
| Prevalence | Ireland | 1990 | 2785.8(2495.32to3060.54) | NA | NA |
| Prevalence | Ireland | 2021 | 2466.78(2233.2to2735.65) | -11.45(-18.11to-3.8) | -0.51(-0.55 to -0.46) |
| Deaths | Israel | 1990 | 22.97(20.96to24.39) | NA | NA |
| Deaths | Israel | 2021 | 12.08(10.31to13.15) | -47.44(-51.94to-43.54) | -1.34(-2.34 to -0.32) |
| DALYs (Disability-Adjusted Life Years) | Israel | 2021 | 292.37(263.81to320.7) | -36.65(-40.7to-32.54) | -0.92(-1.03 to -0.81) |
| DALYs (Disability-Adjusted Life Years) | Israel | 1990 | 461.47(423.92to494.02) | NA | NA |
| Prevalence | Israel | 1990 | 2592.27(2345.95to2884.18) | NA | NA |
| Prevalence | Israel | 2021 | 2340.1(2084.01to2602.78) | -9.73(-15.59to-1.66) | -0.35(-0.38 to -0.33) |
| Deaths | Italy | 1990 | 22.31(20.15to23.44) | NA | NA |
| Deaths | Italy | 2021 | 14.18(11.89to15.43) | -36.45(-41.3to-33.35) | -2.07(-2.39 to -1.75) |
| DALYs (Disability-Adjusted Life Years) | Italy | 1990 | 444.09(415.7to469.61) | NA | NA |
| DALYs (Disability-Adjusted Life Years) | Italy | 2021 | 285.38(256.09to309.66) | -35.74(-38.73to-33.19) | -1.28(-1.41 to -1.16) |
| Prevalence | Italy | 1990 | 2255.74(1980.04to2537.61) | NA | NA |
| Prevalence | Italy | 2021 | 2159.42(1891.01to2433.84) | -4.27(-7.28to-1.45) | -0.27(-0.29 to -0.25) |
| Deaths | Jamaica | 1990 | 12.16(11.17to12.99) | NA | NA |
| Deaths | Jamaica | 2021 | 14.7(11.51to18.47) | 20.94(-5.44to51.38) | 0.21(-0.85 to 1.27) |
| DALYs (Disability-Adjusted Life Years) | Jamaica | 2021 | 335.42(271.26to413.45) | 19.03(-4.12to46.49) | 0.15(0.03 to 0.27) |
| DALYs (Disability-Adjusted Life Years) | Jamaica | 1990 | 281.79(259.39to300.73) | NA | NA |
| Prevalence | Jamaica | 1990 | 1481.86(1299.39to1657.33) | NA | NA |
| Prevalence | Jamaica | 2021 | 1653.88(1478.44to1848.05) | 11.61(1.14to21.41) | 0.2(0.15 to 0.24) |
| Deaths | Japan | 1990 | 12.24(10.95to12.87) | NA | NA |
| Deaths | Japan | 2021 | 5.84(4.93to6.33) | -52.32(-55.18to-50.35) | -2.46(-2.81 to -2.11) |
| DALYs (Disability-Adjusted Life Years) | Japan | 1990 | 274.06(251.9to296.49) | NA | NA |
| DALYs (Disability-Adjusted Life Years) | Japan | 2021 | 155.76(137.62to174.26) | -43.17(-45.43to-40.69) | -1.57(-1.7 to -1.44) |
| Prevalence | Japan | 1990 | 1674.2(1455.45to1916.56) | NA | NA |
| Prevalence | Japan | 2021 | 1285.53(1092.96to1492.23) | -23.22(-27.08to-18.81) | -0.9(-0.95 to -0.85) |
| Deaths | Jordan | 2021 | 10.73(8.55to13.2) | -49.45(-63.59to-32.1) | -3.04(-3.63 to -2.44) |
| Deaths | Jordan | 1990 | 21.23(17.18to26.03) | NA | NA |
| DALYs (Disability-Adjusted Life Years) | Jordan | 2021 | 311.01(266.39to360.78) | -39.15(-51.91to-25.21) | -1.88(-1.96 to -1.8) |
| DALYs (Disability-Adjusted Life Years) | Jordan | 1990 | 511.14(436.06to598.7) | NA | NA |
| Prevalence | Jordan | 1990 | 2023.15(1788.86to2299.83) | NA | NA |
| Prevalence | Jordan | 2021 | 2153.03(1877.41to2442.68) | 6.42(-0.94to14.42) | 0(-0.03 to 0.04) |
| Deaths | Kazakhstan | 1990 | 45.23(40.35to50.01) | NA | NA |
| Deaths | Kazakhstan | 2021 | 46.68(40.28to54.1) | 3.22(-13.54to24.07) | -1.23(-1.49 to -0.97) |
| DALYs (Disability-Adjusted Life Years) | Kazakhstan | 2021 | 1023.13(889.04to1171.56) | 1.24(-14.31to19.56) | -0.95(-1.15 to -0.76) |
| DALYs (Disability-Adjusted Life Years) | Kazakhstan | 1990 | 1010.63(908.23to1113.48) | NA | NA |
| Prevalence | Kazakhstan | 1990 | 2494(2232.83to2826.06) | NA | NA |
| Prevalence | Kazakhstan | 2021 | 2778.16(2508.07to3109.01) | 11.39(1.82to20.58) | 0.28(0.23 to 0.32) |
| Deaths | Kenya | 1990 | 32.57(18.94to52.77) | NA | NA |
| Deaths | Kenya | 2021 | 34.58(20.66to64.8) | 6.16(-16.32to36.42) | 0.13(-0.09 to 0.35) |
| DALYs (Disability-Adjusted Life Years) | Kenya | 2021 | 766.86(514.09to1250.53) | 7.2(-11.43to31.58) | 0.17(0.11 to 0.23) |
| DALYs (Disability-Adjusted Life Years) | Kenya | 1990 | 715.37(471.36to1054.73) | NA | NA |
| Prevalence | Kenya | 1990 | 1415.15(1233.59to1594.96) | NA | NA |
| Prevalence | Kenya | 2021 | 1528.53(1330.32to1716.06) | 8.01(5.35to11.04) | 0.1(0.07 to 0.14) |
| Deaths | Kiribati | 2021 | 83.25(62.48to133.85) | -20.9(-40.51to6.4) | -1.09(-2.75 to 0.6) |
| Deaths | Kiribati | 1990 | 105.25(66.66to167.66) | NA | NA |
| DALYs (Disability-Adjusted Life Years) | Kiribati | 2021 | 1684.31(1265.06to2595.52) | -23.36(-41.48to1.72) | -0.99(-1.25 to -0.72) |
| DALYs (Disability-Adjusted Life Years) | Kiribati | 1990 | 2197.67(1410.58to3256.15) | NA | NA |
| Prevalence | Kiribati | 1990 | 2372.05(2093.17to2657.2) | NA | NA |
| Prevalence | Kiribati | 2021 | 2277.89(1999.04to2574.47) | -3.97(-9.78to2.57) | -0.11(-0.35 to 0.13) |
| Deaths | Kuwait | 2021 | 2.74(2.24to3.28) | -53.92(-61.84to-44.81) | -3.65(-5.56 to -1.7) |
| Deaths | Kuwait | 1990 | 5.95(5.22to6.55) | NA | NA |
| DALYs (Disability-Adjusted Life Years) | Kuwait | 2021 | 160.46(138.49to185.65) | -20.95(-28.27to-13.66) | -0.76(-0.92 to -0.6) |
| DALYs (Disability-Adjusted Life Years) | Kuwait | 1990 | 202.99(182.25to226.32) | NA | NA |
| Prevalence | Kuwait | 1990 | 1648.36(1447.33to1890.08) | NA | NA |
| Prevalence | Kuwait | 2021 | 2006.85(1773.08to2282.36) | 21.75(12.96to31.68) | 0.44(0.39 to 0.49) |
| Deaths | Kyrgyzstan | 1990 | 95.46(87.99to103.05) | NA | NA |
| Deaths | Kyrgyzstan | 2021 | 28.25(23.88to32.67) | -70.41(-75.08to-65.35) | -5.22(-5.51 to -4.93) |
| DALYs (Disability-Adjusted Life Years) | Kyrgyzstan | 2021 | 627.15(542.74to714.78) | -68.28(-72.75to-63.11) | -4.68(-4.8 to -4.55) |
| DALYs (Disability-Adjusted Life Years) | Kyrgyzstan | 1990 | 1977.09(1839.47to2129.62) | NA | NA |
| Prevalence | Kyrgyzstan | 1990 | 2970.96(2718.63to3276.68) | NA | NA |
| Prevalence | Kyrgyzstan | 2021 | 2653.88(2332.34to2976.49) | -10.67(-17.75to-4.91) | -0.57(-0.61 to -0.53) |
| Deaths | Lao People's Democratic Republic | 2021 | 56.4(41.34to72.81) | -34.92(-52.43to-7.31) | -2.08(-2.4 to -1.76) |
| Deaths | Lao People's Democratic Republic | 1990 | 86.67(56.3to111.02) | NA | NA |
| DALYs (Disability-Adjusted Life Years) | Lao People's Democratic Republic | 1990 | 1862.13(1252.31to2347.83) | NA | NA |
| DALYs (Disability-Adjusted Life Years) | Lao People's Democratic Republic | 2021 | 1176.83(892.84to1502.07) | -36.8(-52.89to-11.74) | -1.74(-1.8 to -1.68) |
| Prevalence | Lao People's Democratic Republic | 1990 | 2487.21(2219.05to2778.5) | NA | NA |
| Prevalence | Lao People's Democratic Republic | 2021 | 2401.41(2141.97to2705.24) | -3.45(-9.8to2.4) | -0.24(-0.27 to -0.21) |
| Deaths | Latvia | 2021 | 6.94(5.9to8.11) | -44.81(-54.01to-33.98) | -2.06(-3.9 to -0.19) |
| Deaths | Latvia | 1990 | 12.58(11.52to13.69) | NA | NA |
| DALYs (Disability-Adjusted Life Years) | Latvia | 2021 | 214.69(185.89to243.6) | -32.07(-41.29to-21.51) | -1.22(-1.35 to -1.08) |
| DALYs (Disability-Adjusted Life Years) | Latvia | 1990 | 316.05(287.48to345.6) | NA | NA |
| Prevalence | Latvia | 1990 | 1813.04(1591.83to2056.14) | NA | NA |
| Prevalence | Latvia | 2021 | 1801.32(1589.74to2021.76) | -0.65(-9.67to10.59) | -0.12(-0.17 to -0.08) |
| Deaths | Lebanon | 1990 | 31.37(20.45to41.7) | NA | NA |
| Deaths | Lebanon | 2021 | 19.65(16.16to23.51) | -37.37(-54.94to2.12) | -2.11(-2.69 to -1.52) |
| DALYs (Disability-Adjusted Life Years) | Lebanon | 1990 | 700.15(509.11to888.11) | NA | NA |
| DALYs (Disability-Adjusted Life Years) | Lebanon | 2021 | 477.84(416.89to545.89) | -31.75(-46.93to-3.01) | -1.13(-1.19 to -1.06) |
| Prevalence | Lebanon | 1990 | 1898.4(1677.37to2152.02) | NA | NA |
| Prevalence | Lebanon | 2021 | 2481.23(2159.62to2823.22) | 30.7(22.19to39.21) | 0.75(0.72 to 0.78) |
| Deaths | Lesotho | 2021 | 68.03(48.45to89.97) | 12.52(-15.22to49.63) | 1.29(0.61 to 1.99) |
| Deaths | Lesotho | 1990 | 60.46(43.77to83.11) | NA | NA |
| DALYs (Disability-Adjusted Life Years) | Lesotho | 1990 | 1355.16(1050.28to1752.63) | NA | NA |
| DALYs (Disability-Adjusted Life Years) | Lesotho | 2021 | 1616.31(1182.32to2071.61) | 19.27(-6.71to57.42) | 1.03(0.92 to 1.15) |
| Prevalence | Lesotho | 1990 | 2155.66(1913.98to2453.65) | NA | NA |
| Prevalence | Lesotho | 2021 | 2334.74(2076.67to2646.7) | 8.31(1.88to14.44) | 0.22(0.16 to 0.27) |
| Deaths | Liberia | 2021 | 26.55(19.69to35.93) | -23.67(-44.42to9.25) | -0.41(-1.07 to 0.25) |
| Deaths | Liberia | 1990 | 34.79(26.5to41.49) | NA | NA |
| DALYs (Disability-Adjusted Life Years) | Liberia | 2021 | 652.08(513.7to830.56) | -15.55(-34.84to12.77) | -0.27(-0.36 to -0.19) |
| DALYs (Disability-Adjusted Life Years) | Liberia | 1990 | 772.17(618.68to918.39) | NA | NA |
| Prevalence | Liberia | 1990 | 1425.92(1259.63to1604.91) | NA | NA |
| Prevalence | Liberia | 2021 | 1718.18(1498.14to1946.85) | 20.5(12.17to29.4) | 0.42(0.37 to 0.47) |
| Deaths | Libya | 1990 | 19.62(14.31to25.67) | NA | NA |
| Deaths | Libya | 2021 | 19.96(15.43to26.6) | 1.72(-27.3to39.57) | 0.39(-0.1 to 0.89) |
| DALYs (Disability-Adjusted Life Years) | Libya | 1990 | 473.66(369.66to595.71) | NA | NA |
| DALYs (Disability-Adjusted Life Years) | Libya | 2021 | 504.59(410.08to628.23) | 6.53(-17.91to36.53) | 0.42(0.33 to 0.52) |
| Prevalence | Libya | 1990 | 1790.05(1575.96to2024.96) | NA | NA |
| Prevalence | Libya | 2021 | 2224.56(1971.25to2518.3) | 24.27(16.1to33) | 0.57(0.54 to 0.6) |
| Deaths | Lithuania | 2021 | 8.9(7.88to9.82) | -69.02(-72.63to-65.37) | -3.76(-4.72 to -2.8) |
| Deaths | Lithuania | 1990 | 28.73(26.69to30.5) | NA | NA |
| DALYs (Disability-Adjusted Life Years) | Lithuania | 2021 | 249.92(222.79to275.46) | -61.21(-65.32to-57.03) | -2.93(-3.03 to -2.82) |
| DALYs (Disability-Adjusted Life Years) | Lithuania | 1990 | 644.22(605.75to684.04) | NA | NA |
| Prevalence | Lithuania | 1990 | 2137.81(1890.65to2408.07) | NA | NA |
| Prevalence | Lithuania | 2021 | 1875.04(1673.21to2112.08) | -12.29(-20.47to-3.9) | -0.55(-0.6 to -0.5) |
| Deaths | Luxembourg | 2021 | 18.59(16.43to20.77) | -28.29(-35.55to-20.63) | -2.06(-5.09 to 1.06) |
| Deaths | Luxembourg | 1990 | 25.93(24.27to27.25) | NA | NA |
| DALYs (Disability-Adjusted Life Years) | Luxembourg | 1990 | 555.2(521.72to590.12) | NA | NA |
| DALYs (Disability-Adjusted Life Years) | Luxembourg | 2021 | 399.59(360.41to442.07) | -28.03(-33.68to-21.91) | -1.25(-1.48 to -1.01) |
| Prevalence | Luxembourg | 1990 | 2726.23(2474.42to3004.15) | NA | NA |
| Prevalence | Luxembourg | 2021 | 2570.61(2309.6to2854.1) | -5.71(-12.36to2.87) | -0.3(-0.37 to -0.24) |
| Deaths | Madagascar | 1990 | 60.48(48.42to72.17) | NA | NA |
| Deaths | Madagascar | 2021 | 54(40.77to70.83) | -10.73(-31.52to15.15) | -0.59(-0.79 to -0.39) |
| DALYs (Disability-Adjusted Life Years) | Madagascar | 1990 | 1263.53(1025.66to1497.36) | NA | NA |
| DALYs (Disability-Adjusted Life Years) | Madagascar | 2021 | 1147.8(892.85to1466.97) | -9.16(-28.82to15.89) | -0.43(-0.45 to -0.4) |
| Prevalence | Madagascar | 1990 | 1614.63(1408.48to1813.26) | NA | NA |
| Prevalence | Madagascar | 2021 | 1857.98(1648.41to2120.94) | 15.07(6.31to24.55) | 0.29(0.27 to 0.32) |
| Deaths | Malawi | 1990 | 32.87(26.27to40.98) | NA | NA |
| Deaths | Malawi | 2021 | 29.2(23.86to35.62) | -11.18(-29.7to13.01) | -0.59(-0.93 to -0.25) |
| DALYs (Disability-Adjusted Life Years) | Malawi | 1990 | 744.64(606.95to885.9) | NA | NA |
| DALYs (Disability-Adjusted Life Years) | Malawi | 2021 | 704.6(592.39to839.1) | -5.38(-21.28to15.22) | -0.35(-0.4 to -0.3) |
| Prevalence | Malawi | 1990 | 1299.1(1145.82to1461.97) | NA | NA |
| Prevalence | Malawi | 2021 | 1530.87(1352.11to1728.65) | 17.84(7.52to27.5) | 0.45(0.41 to 0.48) |
| Deaths | Malaysia | 2021 | 28.85(24.9to32.9) | -30.87(-45.33to-16.51) | -1.3(-1.53 to -1.06) |
| Deaths | Malaysia | 1990 | 41.73(35.85to48.11) | NA | NA |
| DALYs (Disability-Adjusted Life Years) | Malaysia | 2021 | 628.09(565.4to692.06) | -29.89(-42.25to-17.62) | -1.03(-1.13 to -0.94) |
| DALYs (Disability-Adjusted Life Years) | Malaysia | 1990 | 895.86(789.18to1003.19) | NA | NA |
| Prevalence | Malaysia | 1990 | 1737.55(1518.35to1965.95) | NA | NA |
| Prevalence | Malaysia | 2021 | 1784.36(1540.75to2022.61) | 2.69(-5.56to10.3) | 0.05(0.03 to 0.07) |
| Deaths | Maldives | 2021 | 35.01(29.07to42.49) | -61.89(-71.35to-37.89) | -5.23(-6.79 to -3.64) |
| Deaths | Maldives | 1990 | 91.85(62.27to111.24) | NA | NA |
| DALYs (Disability-Adjusted Life Years) | Maldives | 2021 | 696.35(597.42to814.59) | -63.02(-71.42to-41.25) | -4.05(-4.25 to -3.85) |
| DALYs (Disability-Adjusted Life Years) | Maldives | 1990 | 1882.94(1269.67to2256.62) | NA | NA |
| Prevalence | Maldives | 1990 | 2451.48(2175.41to2751.74) | NA | NA |
| Prevalence | Maldives | 2021 | 2268.24(1999.51to2573.46) | -7.47(-16.17to1.13) | -0.58(-0.71 to -0.45) |
| Deaths | Mali | 2021 | 44.4(35.43to57.08) | -12.93(-34.59to17.12) | -0.26(-0.51 to -0.01) |
| Deaths | Mali | 1990 | 50.99(41.93to61.22) | NA | NA |
| DALYs (Disability-Adjusted Life Years) | Mali | 2021 | 1042.23(837.23to1324.98) | -11.25(-29.85to15.87) | -0.17(-0.21 to -0.14) |
| DALYs (Disability-Adjusted Life Years) | Mali | 1990 | 1174.28(980.64to1375.82) | NA | NA |
| Prevalence | Mali | 1990 | 1656.03(1463.16to1876.02) | NA | NA |
| Prevalence | Mali | 2021 | 1845.71(1626.52to2078.3) | 11.45(5.21to17.98) | 0.31(0.28 to 0.34) |
| Deaths | Malta | 2021 | 9.74(8.48to10.87) | -54.23(-59.25to-48.71) | -2.41(-6.56 to 1.93) |
| Deaths | Malta | 1990 | 21.27(19.62to22.83) | NA | NA |
| DALYs (Disability-Adjusted Life Years) | Malta | 1990 | 450.93(419.92to483.79) | NA | NA |
| DALYs (Disability-Adjusted Life Years) | Malta | 2021 | 244.56(218.62to274.13) | -45.77(-50.82to-40.38) | -1.6(-1.91 to -1.29) |
| Prevalence | Malta | 1990 | 2365.89(2112.34to2631.29) | NA | NA |
| Prevalence | Malta | 2021 | 2133.04(1879.62to2381.1) | -9.84(-16.17to-2.27) | -0.43(-0.51 to -0.35) |
| Deaths | Marshall Islands | 2021 | 71.32(53.51to90.51) | -30.84(-45.88to-12.9) | -1.25(-3.71 to 1.27) |
| Deaths | Marshall Islands | 1990 | 103.12(78.69to122.01) | NA | NA |
| DALYs (Disability-Adjusted Life Years) | Marshall Islands | 1990 | 2150.42(1637.24to2544.26) | NA | NA |
| DALYs (Disability-Adjusted Life Years) | Marshall Islands | 2021 | 1467.49(1126.82to1862.39) | -31.76(-46.14to-14.49) | -1.15(-1.55 to -0.74) |
| Prevalence | Marshall Islands | 1990 | 2301.73(2041.52to2598.64) | NA | NA |
| Prevalence | Marshall Islands | 2021 | 2024.13(1797.43to2296.13) | -12.06(-18.01to-6.28) | -0.35(-0.71 to 0.02) |
| Deaths | Mauritania | 1990 | 33.33(25.36to40.9) | NA | NA |
| Deaths | Mauritania | 2021 | 19.31(14.83to24.42) | -42.08(-57.9to-15.45) | -1.68(-2.53 to -0.82) |
| DALYs (Disability-Adjusted Life Years) | Mauritania | 2021 | 482.86(395.79to574.92) | -36.25(-50.96to-13.52) | -1.1(-1.2 to -1.01) |
| DALYs (Disability-Adjusted Life Years) | Mauritania | 1990 | 757.41(599.92to899.98) | NA | NA |
| Prevalence | Mauritania | 1990 | 1370.61(1208.18to1541.27) | NA | NA |
| Prevalence | Mauritania | 2021 | 1500.83(1316.64to1725.33) | 9.5(2.22to17.7) | 0.19(0.14 to 0.25) |
| Deaths | Mauritius | 1990 | 33.48(31.24to35.45) | NA | NA |
| Deaths | Mauritius | 2021 | 16.88(15.32to18.02) | -49.58(-53.43to-45.61) | -2.69(-4.06 to -1.29) |
| DALYs (Disability-Adjusted Life Years) | Mauritius | 1990 | 683.52(645.06to723.59) | NA | NA |
| DALYs (Disability-Adjusted Life Years) | Mauritius | 2021 | 388.62(361.01to420.1) | -43.14(-46.71to-39.37) | -1.71(-1.86 to -1.55) |
| Prevalence | Mauritius | 1990 | 1631.22(1442.97to1860.08) | NA | NA |
| Prevalence | Mauritius | 2021 | 1687.13(1499.78to1901.07) | 3.43(-3.11to10.38) | -0.07(-0.13 to -0.01) |
| Deaths | Mexico | 1990 | 43.92(41.61to45.07) | NA | NA |
| Deaths | Mexico | 2021 | 27.87(24.9to30.7) | -36.53(-42.52to-30.75) | -1.18(-1.35 to -1.02) |
| DALYs (Disability-Adjusted Life Years) | Mexico | 2021 | 519.67(469.83to570.36) | -29.7(-35.98to-23.65) | -0.92(-0.99 to -0.84) |
| DALYs (Disability-Adjusted Life Years) | Mexico | 1990 | 739.18(708.08to763.46) | NA | NA |
| Prevalence | Mexico | 1990 | 2018.35(1783.92to2302.59) | NA | NA |
| Prevalence | Mexico | 2021 | 2238.95(1970.83to2525.77) | 10.93(6.01to16.37) | 0.13(0.12 to 0.15) |
| Deaths | Micronesia (Federated States of) | 2021 | 67.13(53.27to86.18) | -41.83(-56.8to-17.95) | -1.82(-3.51 to -0.09) |
| Deaths | Micronesia (Federated States of) | 1990 | 115.41(85.11to149.45) | NA | NA |
| DALYs (Disability-Adjusted Life Years) | Micronesia (Federated States of) | 2021 | 1396.63(1121.89to1761.24) | -40.65(-54.93to-17.89) | -1.64(-1.9 to -1.39) |
| DALYs (Disability-Adjusted Life Years) | Micronesia (Federated States of) | 1990 | 2353.05(1717.15to2906.41) | NA | NA |
| Prevalence | Micronesia (Federated States of) | 1990 | 2470.02(2199.02to2765.16) | NA | NA |
| Prevalence | Micronesia (Federated States of) | 2021 | 2210.98(1956.06to2493.15) | -10.49(-17.58to-3.48) | -0.31(-0.52 to -0.1) |
| Deaths | Monaco | 2021 | 12.09(9.45to14.93) | -11.56(-31.7to15.82) | -0.67(-11.02 to 10.89) |
| Deaths | Monaco | 1990 | 13.67(10.63to16.48) | NA | NA |
| DALYs (Disability-Adjusted Life Years) | Monaco | 2021 | 292.09(246.1to339.51) | -12.53(-26.75to4.79) | -0.46(-1.45 to 0.53) |
| DALYs (Disability-Adjusted Life Years) | Monaco | 1990 | 333.93(281.51to385.14) | NA | NA |
| Prevalence | Monaco | 1990 | 2518.13(2208.02to2861.42) | NA | NA |
| Prevalence | Monaco | 2021 | 2386.91(2120.48to2666.93) | -5.21(-12.17to2.16) | -0.25(-0.52 to 0.03) |
| Deaths | Mongolia | 1990 | 40.46(31.83to50.11) | NA | NA |
| Deaths | Mongolia | 2021 | 20.59(16.43to24.89) | -49.12(-61.54to-29.06) | -3.41(-4.04 to -2.77) |
| DALYs (Disability-Adjusted Life Years) | Mongolia | 2021 | 453.68(376.58to532.25) | -46.58(-58.78to-29.25) | -2.78(-2.88 to -2.69) |
| DALYs (Disability-Adjusted Life Years) | Mongolia | 1990 | 849.27(694.29to1029.74) | NA | NA |
| Prevalence | Mongolia | 1990 | 2185.01(1930.39to2489.62) | NA | NA |
| Prevalence | Mongolia | 2021 | 2187.97(1928.74to2468.99) | 0.14(-7.06to7.74) | -0.17(-0.21 to -0.13) |
| Deaths | Montenegro | 1990 | 5.71(4.78to6.88) | NA | NA |
| Deaths | Montenegro | 2021 | 5.87(4.63to7.15) | 2.79(-23.22to33.65) | -1.11(-5.23 to 3.2) |
| DALYs (Disability-Adjusted Life Years) | Montenegro | 1990 | 185.35(161.06to212.82) | NA | NA |
| DALYs (Disability-Adjusted Life Years) | Montenegro | 2021 | 187.53(161.34to217.71) | 1.18(-12.71to16.91) | -0.17(-0.44 to 0.11) |
| Prevalence | Montenegro | 1990 | 1813.51(1590.8to2058.49) | NA | NA |
| Prevalence | Montenegro | 2021 | 2053.55(1782.44to2305.68) | 13.24(6.95to20.92) | 0.35(0.27 to 0.43) |
| Deaths | Morocco | 1990 | 21.45(14.33to27.92) | NA | NA |
| Deaths | Morocco | 2021 | 23.52(18.52to28.25) | 9.63(-17.14to50.97) | -0.16(-0.37 to 0.04) |
| DALYs (Disability-Adjusted Life Years) | Morocco | 1990 | 505.94(361.23to631.38) | NA | NA |
| DALYs (Disability-Adjusted Life Years) | Morocco | 2021 | 557.91(461.15to645.66) | 10.27(-12.22to42.44) | 0.13(0.09 to 0.17) |
| Prevalence | Morocco | 1990 | 1745.14(1534.32to1986.55) | NA | NA |
| Prevalence | Morocco | 2021 | 2361.44(2081.75to2665.44) | 35.32(24.84to45.54) | 0.77(0.74 to 0.79) |
| Deaths | Mozambique | 1990 | 28.34(21.53to35.33) | NA | NA |
| Deaths | Mozambique | 2021 | 27.78(21.01to35.02) | -1.98(-27.19to27.47) | 0.61(0.32 to 0.91) |
| DALYs (Disability-Adjusted Life Years) | Mozambique | 1990 | 656.87(524.85to788.92) | NA | NA |
| DALYs (Disability-Adjusted Life Years) | Mozambique | 2021 | 683.39(549.98to823.52) | 4.04(-18.12to31.04) | 0.43(0.37 to 0.49) |
| Prevalence | Mozambique | 1990 | 1412.61(1233.71to1601.78) | NA | NA |
| Prevalence | Mozambique | 2021 | 1571.19(1375.56to1771.66) | 11.23(5.2to17.74) | 0.21(0.19 to 0.23) |
| Deaths | Myanmar | 2021 | 104.53(82.46to126.42) | -22.87(-42.32to9.41) | -1.87(-1.96 to -1.78) |
| Deaths | Myanmar | 1990 | 135.53(99.61to173.15) | NA | NA |
| DALYs (Disability-Adjusted Life Years) | Myanmar | 1990 | 2710.62(2042.45to3435.53) | NA | NA |
| DALYs (Disability-Adjusted Life Years) | Myanmar | 2021 | 1958.92(1604.09to2354) | -27.73(-45.07to-0.27) | -1.59(-1.64 to -1.54) |
| Prevalence | Myanmar | 1990 | 2779.98(2508.16to3054.35) | NA | NA |
| Prevalence | Myanmar | 2021 | 2719.81(2465.81to3044.72) | -2.16(-9.71to5.54) | -0.2(-0.22 to -0.18) |
| Deaths | Namibia | 2021 | 51.42(40.79to68.23) | -21.57(-39.38to9.69) | -1.05(-1.78 to -0.32) |
| Deaths | Namibia | 1990 | 65.56(49.3to85.47) | NA | NA |
| DALYs (Disability-Adjusted Life Years) | Namibia | 1990 | 1431.13(1088.79to1767.53) | NA | NA |
| DALYs (Disability-Adjusted Life Years) | Namibia | 2021 | 1171.77(951.35to1457.16) | -18.12(-34.34to8.1) | -0.78(-0.87 to -0.69) |
| Prevalence | Namibia | 1990 | 1994.23(1749.44to2267.97) | NA | NA |
| Prevalence | Namibia | 2021 | 2047.18(1801.24to2327.96) | 2.65(-4.02to9.17) | 0.03(-0.03 to 0.09) |
| Deaths | Nauru | 2021 | 79.33(53.83to140.78) | -27.04(-46.94to15.74) | -1.38(-6.53 to 4.05) |
| Deaths | Nauru | 1990 | 108.74(84.73to163.25) | NA | NA |
| DALYs (Disability-Adjusted Life Years) | Nauru | 2021 | 1614.56(1176.72to2426.49) | -27.7(-45.14to2) | -1.26(-2.12 to -0.4) |
| DALYs (Disability-Adjusted Life Years) | Nauru | 1990 | 2233.05(1739.46to3176.44) | NA | NA |
| Prevalence | Nauru | 1990 | 2273.51(2008.22to2555.3) | NA | NA |
| Prevalence | Nauru | 2021 | 2138.45(1864.22to2421) | -5.94(-11.47to0.76) | -0.21(-0.99 to 0.59) |
| Deaths | Nepal | 1990 | 176.12(121.67to222.55) | NA | NA |
| Deaths | Nepal | 2021 | 146.13(116.66to182.46) | -17.03(-37.73to17.87) | -1(-1.17 to -0.84) |
| DALYs (Disability-Adjusted Life Years) | Nepal | 2021 | 2836.01(2275.31to3485.04) | -21.47(-40.3to10.3) | -0.81(-0.86 to -0.76) |
| DALYs (Disability-Adjusted Life Years) | Nepal | 1990 | 3611.26(2493to4552.9) | NA | NA |
| Prevalence | Nepal | 1990 | 3049.02(2802.37to3266.97) | NA | NA |
| Prevalence | Nepal | 2021 | 3051.13(2835.24to3271.74) | 0.07(-5.15to5.13) | -0.12(-0.13 to -0.1) |
| Deaths | Netherlands | 2021 | 26.93(23.51to29.07) | -16.13(-22.3to-10.36) | -1.05(-1.5 to -0.59) |
| Deaths | Netherlands | 1990 | 32.1(29.46to33.82) | NA | NA |
| DALYs (Disability-Adjusted Life Years) | Netherlands | 1990 | 658.81(616.49to692.81) | NA | NA |
| DALYs (Disability-Adjusted Life Years) | Netherlands | 2021 | 537.79(493.18to576.92) | -18.37(-23.36to-13.08) | -0.76(-0.95 to -0.58) |
| Prevalence | Netherlands | 1990 | 2405.38(2200.87to2673.25) | NA | NA |
| Prevalence | Netherlands | 2021 | 2263.34(2048.89to2513.4) | -5.91(-13.59to3.88) | -0.25(-0.29 to -0.21) |
| Deaths | New Zealand | 2021 | 21.97(19.19to23.76) | -32.74(-38.2to-28.08) | -1.93(-3.1 to -0.75) |
| Deaths | New Zealand | 1990 | 32.67(30.31to34.2) | NA | NA |
| DALYs (Disability-Adjusted Life Years) | New Zealand | 2021 | 431.82(393.22to462.12) | -35.71(-39.22to-31.94) | -1.57(-1.71 to -1.42) |
| DALYs (Disability-Adjusted Life Years) | New Zealand | 1990 | 671.68(638.18to703.55) | NA | NA |
| Prevalence | New Zealand | 1990 | 1955.25(1694.11to2252.48) | NA | NA |
| Prevalence | New Zealand | 2021 | 1678.32(1429.32to1958.56) | -14.16(-19.53to-7.7) | -0.67(-0.71 to -0.63) |
| Deaths | Nicaragua | 1990 | 17.75(15.36to20.07) | NA | NA |
| Deaths | Nicaragua | 2021 | 18.66(15.18to22.05) | 5.13(-15.72to27.24) | -0.35(-1.12 to 0.43) |
| DALYs (Disability-Adjusted Life Years) | Nicaragua | 1990 | 374.25(336.46to413.67) | NA | NA |
| DALYs (Disability-Adjusted Life Years) | Nicaragua | 2021 | 381.7(327.58to437.44) | 1.99(-13.55to19.63) | -0.2(-0.31 to -0.08) |
| Prevalence | Nicaragua | 1990 | 1853.52(1618.86to2114.79) | NA | NA |
| Prevalence | Nicaragua | 2021 | 2223.48(1965.05to2482.65) | 19.96(10.08to30.61) | 0.28(0.24 to 0.32) |
| Deaths | Niger | 1990 | 37.3(24.68to48.42) | NA | NA |
| Deaths | Niger | 2021 | 28.5(18.98to37.47) | -23.6(-43.62to8.44) | -0.62(-0.99 to -0.26) |
| DALYs (Disability-Adjusted Life Years) | Niger | 2021 | 702.13(524.46to871.29) | -19.87(-36.83to6.35) | -0.32(-0.37 to -0.27) |
| DALYs (Disability-Adjusted Life Years) | Niger | 1990 | 876.25(635.55to1086.55) | NA | NA |
| Prevalence | Niger | 1990 | 1658.35(1440.53to1881.3) | NA | NA |
| Prevalence | Niger | 2021 | 1945.97(1710.19to2209.98) | 17.34(10.5to25.4) | 0.53(0.5 to 0.56) |
| Deaths | Nigeria | 1990 | 24.21(18.34to30.04) | NA | NA |
| Deaths | Nigeria | 2021 | 18.62(15.79to22.12) | -23.1(-40.82to12.01) | -1.09(-1.23 to -0.95) |
| DALYs (Disability-Adjusted Life Years) | Nigeria | 2021 | 490.54(424.15to569.06) | -17(-33.72to11.41) | -0.58(-0.61 to -0.54) |
| DALYs (Disability-Adjusted Life Years) | Nigeria | 1990 | 590.98(473.38to708.27) | NA | NA |
| Prevalence | Nigeria | 1990 | 1567.56(1359.31to1762.6) | NA | NA |
| Prevalence | Nigeria | 2021 | 1788.63(1562.86to2005.8) | 14.1(11.34to17.64) | 0.36(0.33 to 0.38) |
| Deaths | Niue | 2021 | 48.45(39.22to59.64) | -34.91(-48.82to-15.34) | -1.82(-16.75 to 15.79) |
| Deaths | Niue | 1990 | 74.44(62.89to88.19) | NA | NA |
| DALYs (Disability-Adjusted Life Years) | Niue | 2021 | 985.74(811.57to1191.85) | -34.15(-47.82to-16.37) | -1.62(-3.67 to 0.48) |
| DALYs (Disability-Adjusted Life Years) | Niue | 1990 | 1496.95(1271.72to1761.46) | NA | NA |
| Prevalence | Niue | 1990 | 2195.79(1936.13to2479.02) | NA | NA |
| Prevalence | Niue | 2021 | 1920.92(1678.86to2190.63) | -12.52(-20.01to-4.96) | -0.53(-1.82 to 0.78) |
| Deaths | North Macedonia | 1990 | 27.61(23.87to32.95) | NA | NA |
| Deaths | North Macedonia | 2021 | 21(16.05to28.34) | -23.94(-41.19to0.56) | -1.69(-2.91 to -0.46) |
| DALYs (Disability-Adjusted Life Years) | North Macedonia | 2021 | 454.42(362.35to594.99) | -24.17(-39.04to-2.33) | -1.09(-1.21 to -0.98) |
| DALYs (Disability-Adjusted Life Years) | North Macedonia | 1990 | 599.24(533.68to688.26) | NA | NA |
| Prevalence | North Macedonia | 1990 | 2427.41(2130.3to2742.78) | NA | NA |
| Prevalence | North Macedonia | 2021 | 2624.09(2302.9to3004.59) | 8.1(-1.85to20.4) | 0.05(0.01 to 0.08) |
| Deaths | Northern Mariana Islands | 2021 | 47.04(40.84to53.5) | -31.68(-45.66to-15.3) | -1.23(-4.7 to 2.36) |
| Deaths | Northern Mariana Islands | 1990 | 68.86(57.65to82.74) | NA | NA |
| DALYs (Disability-Adjusted Life Years) | Northern Mariana Islands | 2021 | 940.6(840.4to1055.79) | -30.37(-44.22to-13.84) | -1.13(-1.62 to -0.63) |
| DALYs (Disability-Adjusted Life Years) | Northern Mariana Islands | 1990 | 1350.94(1137.98to1621.71) | NA | NA |
| Prevalence | Northern Mariana Islands | 1990 | 2014.26(1749.2to2296.27) | NA | NA |
| Prevalence | Northern Mariana Islands | 2021 | 1867(1609.47to2151.45) | -7.31(-15.3to0.76) | -0.39(-0.74 to -0.05) |
| Deaths | Norway | 2021 | 24.65(21.65to26.3) | 100.73(87.23to110.72) | 0.91(-0.24 to 2.08) |
| Deaths | Norway | 1990 | 12.28(11.45to12.82) | NA | NA |
| DALYs (Disability-Adjusted Life Years) | Norway | 2021 | 497.3(456.34to532.24) | 57.21(49.36to64.35) | 0.7(0.58 to 0.82) |
| DALYs (Disability-Adjusted Life Years) | Norway | 1990 | 316.34(296.03to338.19) | NA | NA |
| Prevalence | Norway | 1990 | 2480.03(2193.59to2787.31) | NA | NA |
| Prevalence | Norway | 2021 | 2768.83(2464.33to3111.06) | 11.65(8.36to14.96) | 0.14(0.12 to 0.16) |
| Deaths | Oman | 1990 | 18.62(13.38to23.96) | NA | NA |
| Deaths | Oman | 2021 | 14.24(11.62to17.63) | -23.48(-44.16to15.18) | -1.89(-2.84 to -0.94) |
| DALYs (Disability-Adjusted Life Years) | Oman | 2021 | 380.91(326.15to458.22) | -22.02(-38.02to6.08) | -1(-1.13 to -0.87) |
| DALYs (Disability-Adjusted Life Years) | Oman | 1990 | 488.48(388.97to597.76) | NA | NA |
| Prevalence | Oman | 1990 | 2082.83(1848.99to2356.59) | NA | NA |
| Prevalence | Oman | 2021 | 2314.58(2036.61to2644.12) | 11.13(3.4to20.29) | 0.05(0.01 to 0.1) |
| Deaths | Pakistan | 1990 | 83.97(64.44to98.28) | NA | NA |
| Deaths | Pakistan | 2021 | 73.8(60.04to94.55) | -12.12(-31.71to24) | -1.11(-1.18 to -1.03) |
| DALYs (Disability-Adjusted Life Years) | Pakistan | 1990 | 1783.98(1421.94to2048.46) | NA | NA |
| DALYs (Disability-Adjusted Life Years) | Pakistan | 2021 | 1541.67(1287.52to1855.86) | -13.58(-30.23to13.08) | -0.83(-0.86 to -0.81) |
| Prevalence | Pakistan | 1990 | 2643.8(2329.53to2967.91) | NA | NA |
| Prevalence | Pakistan | 2021 | 2606.29(2311.9to2925.99) | -1.42(-4.57to1.93) | -0.15(-0.17 to -0.14) |
| Deaths | Palau | 2021 | 70.54(57.96to84.93) | -15(-34.32to9.59) | -0.64(-4.59 to 3.48) |
| Deaths | Palau | 1990 | 82.99(68.59to101.4) | NA | NA |
| DALYs (Disability-Adjusted Life Years) | Palau | 2021 | 1338.13(1120.27to1589.76) | -18.36(-36.18to3.43) | -0.58(-1.17 to 0.01) |
| DALYs (Disability-Adjusted Life Years) | Palau | 1990 | 1639.14(1348.08to1990.14) | NA | NA |
| Prevalence | Palau | 1990 | 2210.65(1934.41to2501.28) | NA | NA |
| Prevalence | Palau | 2021 | 1988.29(1735.17to2245.21) | -10.06(-18.39to-1.16) | -0.37(-0.82 to 0.09) |
| Deaths | Palestine | 1990 | 27.88(21.05to34.67) | NA | NA |
| Deaths | Palestine | 2021 | 16.34(13.91to19.89) | -41.38(-54.85to-17.13) | -2.07(-2.95 to -1.18) |
| DALYs (Disability-Adjusted Life Years) | Palestine | 2021 | 397.17(353.23to454.73) | -32.5(-45.29to-12.54) | -1.33(-1.43 to -1.23) |
| DALYs (Disability-Adjusted Life Years) | Palestine | 1990 | 588.43(467.45to712.02) | NA | NA |
| Prevalence | Palestine | 1990 | 1951.02(1723.89to2212.09) | NA | NA |
| Prevalence | Palestine | 2021 | 2147.67(1912.25to2436.2) | 10.08(1.94to17.45) | 0.14(0.1 to 0.19) |
| Deaths | Panama | 1990 | 17.72(16.02to19.05) | NA | NA |
| Deaths | Panama | 2021 | 15.53(12.08to18.57) | -12.35(-28.63to4.75) | -1.32(-2.38 to -0.26) |
| DALYs (Disability-Adjusted Life Years) | Panama | 2021 | 313.24(258.1to363.23) | -13.45(-27.02to0.39) | -0.91(-1.08 to -0.75) |
| DALYs (Disability-Adjusted Life Years) | Panama | 1990 | 361.93(336.03to389.55) | NA | NA |
| Prevalence | Panama | 1990 | 1637.43(1432.17to1858.99) | NA | NA |
| Prevalence | Panama | 2021 | 1863.21(1677.28to2075.51) | 13.79(4.08to24.98) | 0.09(0.05 to 0.13) |
| Deaths | Papua New Guinea | 2021 | 156.82(123.55to197.43) | -16.82(-38.08to14.85) | -0.85(-1.04 to -0.65) |
| Deaths | Papua New Guinea | 1990 | 188.53(140.8to243.16) | NA | NA |
| DALYs (Disability-Adjusted Life Years) | Papua New Guinea | 2021 | 3004.36(2404.29to3732.82) | -19.34(-39.95to12.22) | -0.78(-0.81 to -0.74) |
| DALYs (Disability-Adjusted Life Years) | Papua New Guinea | 1990 | 3724.74(2788.16to4811.98) | NA | NA |
| Prevalence | Papua New Guinea | 1990 | 2846.9(2595to3118.55) | NA | NA |
| Prevalence | Papua New Guinea | 2021 | 2711.59(2480.62to2980.45) | -4.75(-9.16to-0.48) | -0.16(-0.19 to -0.13) |
| Deaths | Paraguay | 1990 | 17.04(14.65to19.96) | NA | NA |
| Deaths | Paraguay | 2021 | 18.7(14.91to23.53) | 9.7(-15to36.67) | -0.07(-0.81 to 0.67) |
| DALYs (Disability-Adjusted Life Years) | Paraguay | 1990 | 347.01(304.21to401.38) | NA | NA |
| DALYs (Disability-Adjusted Life Years) | Paraguay | 2021 | 385.23(315.58to470.01) | 11.01(-11.29to36.69) | 0.06(-0.02 to 0.15) |
| Prevalence | Paraguay | 1990 | 1954.9(1709.12to2251.08) | NA | NA |
| Prevalence | Paraguay | 2021 | 2153.44(1889.7to2446.94) | 10.16(-2.1to24.13) | 0.16(0.13 to 0.19) |
| Deaths | Peru | 2021 | 9.44(6.51to12.85) | -27.31(-51.99to11.67) | -1.91(-2.29 to -1.53) |
| Deaths | Peru | 1990 | 12.98(9.86to16.05) | NA | NA |
| DALYs (Disability-Adjusted Life Years) | Peru | 1990 | 271(214.59to328.03) | NA | NA |
| DALYs (Disability-Adjusted Life Years) | Peru | 2021 | 200.04(154.29to255.74) | -26.18(-45.7to3.18) | -1.21(-1.28 to -1.13) |
| Prevalence | Peru | 1990 | 1404.73(1224.87to1625.35) | NA | NA |
| Prevalence | Peru | 2021 | 1538.03(1327.25to1755.86) | 9.49(-3.21to23.37) | 0.14(0.13 to 0.16) |
| Deaths | Philippines | 1990 | 46.58(41.87to51.28) | NA | NA |
| Deaths | Philippines | 2021 | 32.81(27.69to38.24) | -29.56(-42.15to-14.99) | -0.14(-0.27 to -0.01) |
| DALYs (Disability-Adjusted Life Years) | Philippines | 1990 | 961.37(882.15to1056.81) | NA | NA |
| DALYs (Disability-Adjusted Life Years) | Philippines | 2021 | 807.05(707.12to923.78) | -16.05(-28.41to-2.57) | -0.22(-0.28 to -0.15) |
| Prevalence | Philippines | 1990 | 2422.93(2133.86to2712.38) | NA | NA |
| Prevalence | Philippines | 2021 | 2213.41(1960.93to2482.71) | -8.65(-11.07to-6.19) | -0.32(-0.36 to -0.28) |
| Deaths | Poland | 1990 | 25.16(24.09to25.9) | NA | NA |
| Deaths | Poland | 2021 | 11.8(10.69to12.81) | -53.1(-56.75to-49.57) | -2.63(-3.03 to -2.22) |
| DALYs (Disability-Adjusted Life Years) | Poland | 1990 | 597.58(571.96to626.59) | NA | NA |
| DALYs (Disability-Adjusted Life Years) | Poland | 2021 | 327.79(298.61to358.48) | -45.15(-48.6to-41.99) | -1.61(-1.7 to -1.52) |
| Prevalence | Poland | 1990 | 2273.76(2003.6to2550.63) | NA | NA |
| Prevalence | Poland | 2021 | 2262.26(1997.88to2539.22) | -0.51(-3.65to2.63) | -0.22(-0.26 to -0.19) |
| Deaths | Portugal | 2021 | 17.55(14.93to19.18) | -38.02(-44.61to-32.28) | -2.63(-3.22 to -2.03) |
| Deaths | Portugal | 1990 | 28.32(26.42to30.05) | NA | NA |
| DALYs (Disability-Adjusted Life Years) | Portugal | 1990 | 573.56(539.5to611.6) | NA | NA |
| DALYs (Disability-Adjusted Life Years) | Portugal | 2021 | 344.54(312.27to373.1) | -39.93(-44.29to-35.39) | -1.93(-2.03 to -1.83) |
| Prevalence | Portugal | 1990 | 2706.54(2483.56to2951.6) | NA | NA |
| Prevalence | Portugal | 2021 | 2318.03(2088.23to2583.42) | -14.35(-21.31to-6.75) | -0.71(-0.77 to -0.65) |
| Deaths | Puerto Rico | 2021 | 16.98(14.01to19.81) | -24.8(-37.26to-12.2) | -1.18(-1.88 to -0.48) |
| Deaths | Puerto Rico | 1990 | 22.58(21.01to23.97) | NA | NA |
| DALYs (Disability-Adjusted Life Years) | Puerto Rico | 1990 | 422.95(399to448.54) | NA | NA |
| DALYs (Disability-Adjusted Life Years) | Puerto Rico | 2021 | 354.55(305.58to406.86) | -16.17(-27.33to-4.72) | -0.88(-1 to -0.76) |
| Prevalence | Puerto Rico | 1990 | 1530.93(1343.7to1745.27) | NA | NA |
| Prevalence | Puerto Rico | 2021 | 1802.96(1581.24to2026.47) | 17.77(7.19to28.62) | 0.35(0.31 to 0.38) |
| Deaths | Qatar | 1990 | 29.4(24.12to35.54) | NA | NA |
| Deaths | Qatar | 2021 | 12.79(9.99to15.94) | -56.51(-66.97to-42.05) | -3.38(-5.06 to -1.67) |
| DALYs (Disability-Adjusted Life Years) | Qatar | 2021 | 336.21(287.54to395.65) | -44.52(-54.99to-32.01) | -1.96(-2.19 to -1.72) |
| DALYs (Disability-Adjusted Life Years) | Qatar | 1990 | 605.95(516.58to706.51) | NA | NA |
| Prevalence | Qatar | 1990 | 2115.89(1863.92to2403.14) | NA | NA |
| Prevalence | Qatar | 2021 | 2384.48(2104.19to2686.75) | 12.69(4.54to20.24) | 0.04(-0.07 to 0.15) |
| Deaths | Republic of Korea | 1990 | 28.73(22.89to34.09) | NA | NA |
| Deaths | Republic of Korea | 2021 | 12.03(9.74to14.78) | -58.13(-66.81to-41.26) | -3.87(-4.3 to -3.44) |
| DALYs (Disability-Adjusted Life Years) | Republic of Korea | 2021 | 289.13(251.75to335.7) | -47.04(-54.76to-34.1) | -1.69(-1.81 to -1.56) |
| DALYs (Disability-Adjusted Life Years) | Republic of Korea | 1990 | 545.98(472.24to617.55) | NA | NA |
| Prevalence | Republic of Korea | 1990 | 2149.18(1882.65to2433.37) | NA | NA |
| Prevalence | Republic of Korea | 2021 | 2287.85(2091.34to2510.81) | 6.45(-1.82to15.23) | 0.05(0.01 to 0.1) |
| Deaths | Republic of Moldova | 2021 | 11.54(10.25to12.95) | -74.58(-77.6to-71.43) | -4.75(-5.38 to -4.11) |
| Deaths | Republic of Moldova | 1990 | 45.41(42.69to48.18) | NA | NA |
| DALYs (Disability-Adjusted Life Years) | Republic of Moldova | 2021 | 314.75(283.83to348.95) | -68.58(-72.06to-65.05) | -3.96(-4.12 to -3.79) |
| DALYs (Disability-Adjusted Life Years) | Republic of Moldova | 1990 | 1001.85(941.95to1066) | NA | NA |
| Prevalence | Republic of Moldova | 1990 | 2472.46(2220.62to2751.17) | NA | NA |
| Prevalence | Republic of Moldova | 2021 | 2054.31(1805.95to2331.16) | -16.91(-23.17to-10.88) | -0.8(-0.84 to -0.76) |
| Deaths | Romania | 1990 | 54.76(51.27to57.77) | NA | NA |
| Deaths | Romania | 2021 | 17.41(15.34to19.38) | -68.2(-72.22to-64.34) | -2.38(-2.66 to -2.09) |
| DALYs (Disability-Adjusted Life Years) | Romania | 1990 | 1072.31(1010.94to1131.17) | NA | NA |
| DALYs (Disability-Adjusted Life Years) | Romania | 2021 | 459.19(415.1to503.41) | -57.18(-61.94to-52.71) | -1.98(-2.08 to -1.89) |
| Prevalence | Romania | 1990 | 2527.95(2232.71to2842.2) | NA | NA |
| Prevalence | Romania | 2021 | 2367.71(2125.87to2635.41) | -6.34(-16.01to5.56) | -0.29(-0.31 to -0.27) |
| Deaths | Russian Federation | 2021 | 13.04(12to14.13) | -56.25(-59.58to-53.04) | -2.97(-3.23 to -2.7) |
| Deaths | Russian Federation | 1990 | 29.81(28.54to30.43) | NA | NA |
| DALYs (Disability-Adjusted Life Years) | Russian Federation | 1990 | 677.94(654.8to700.48) | NA | NA |
| DALYs (Disability-Adjusted Life Years) | Russian Federation | 2021 | 352.01(328.27to379.88) | -48.08(-51.32to-44.65) | -2.34(-2.49 to -2.19) |
| Prevalence | Russian Federation | 1990 | 2328.53(2055.06to2624.68) | NA | NA |
| Prevalence | Russian Federation | 2021 | 2196.02(1945.53to2463.55) | -5.69(-8.3to-3.42) | -0.22(-0.26 to -0.17) |
| Deaths | Rwanda | 1990 | 64.53(46.29to78.59) | NA | NA |
| Deaths | Rwanda | 2021 | 34.47(23.96to46.31) | -46.59(-62.28to-23.42) | -3.37(-3.68 to -3.07) |
| DALYs (Disability-Adjusted Life Years) | Rwanda | 2021 | 800.01(611.66to1028.73) | -44.07(-57.87to-23.79) | -2.58(-2.63 to -2.54) |
| DALYs (Disability-Adjusted Life Years) | Rwanda | 1990 | 1430.48(1063.03to1702.37) | NA | NA |
| Prevalence | Rwanda | 1990 | 1717.67(1528.15to1944.38) | NA | NA |
| Prevalence | Rwanda | 2021 | 1830.31(1621.65to2058.32) | 6.56(-1.07to15.76) | 0.06(0.03 to 0.09) |
| Deaths | Saint Kitts and Nevis | 2021 | 16.55(14.25to18.64) | 15.49(-2.32to33.99) | 0.14(-8.49 to 9.58) |
| Deaths | Saint Kitts and Nevis | 1990 | 14.33(13.15to15.4) | NA | NA |
| DALYs (Disability-Adjusted Life Years) | Saint Kitts and Nevis | 1990 | 297.98(274.17to321.8) | NA | NA |
| DALYs (Disability-Adjusted Life Years) | Saint Kitts and Nevis | 2021 | 345.5(297.19to389.88) | 15.95(-0.51to35.09) | 0.26(-0.7 to 1.23) |
| Prevalence | Saint Kitts and Nevis | 1990 | 1138.63(989.37to1296.51) | NA | NA |
| Prevalence | Saint Kitts and Nevis | 2021 | 1457.74(1255.85to1677.64) | 28.03(18.4to39.26) | 0.59(0.23 to 0.94) |
| Deaths | Saint Lucia | 1990 | 25.29(23.78to27.15) | NA | NA |
| Deaths | Saint Lucia | 2021 | 23.53(19.22to27.78) | -6.96(-24.29to10.94) | 0.14(-2.8 to 3.16) |
| DALYs (Disability-Adjusted Life Years) | Saint Lucia | 2021 | 487.49(404.87to574.54) | 0.08(-17.52to18.82) | 0.2(-0.19 to 0.59) |
| DALYs (Disability-Adjusted Life Years) | Saint Lucia | 1990 | 487.08(458.95to523.15) | NA | NA |
| Prevalence | Saint Lucia | 1990 | 1520.62(1324.23to1713.84) | NA | NA |
| Prevalence | Saint Lucia | 2021 | 1761.21(1536.13to1974.36) | 15.82(6.86to25.02) | 0.4(0.23 to 0.58) |
| Deaths | Saint Vincent and the Grenadines | 1990 | 6.5(5.96to7.08) | NA | NA |
| Deaths | Saint Vincent and the Grenadines | 2021 | 11.15(9.89to12.49) | 71.62(50.19to96.85) | 1.35(-5.33 to 8.5) |
| DALYs (Disability-Adjusted Life Years) | Saint Vincent and the Grenadines | 1990 | 150.03(137.04to163.29) | NA | NA |
| DALYs (Disability-Adjusted Life Years) | Saint Vincent and the Grenadines | 2021 | 240.14(216.88to267.42) | 60.06(42.14to79.78) | 1.11(0.36 to 1.88) |
| Prevalence | Saint Vincent and the Grenadines | 1990 | 1093.03(950.19to1243.07) | NA | NA |
| Prevalence | Saint Vincent and the Grenadines | 2021 | 1314.21(1156.11to1497.43) | 20.24(9.84to30.59) | 0.52(0.27 to 0.76) |
| Deaths | Samoa | 2021 | 69.57(54.86to85.98) | -31.28(-42.99to-16.35) | -1.02(-2.48 to 0.46) |
| Deaths | Samoa | 1990 | 101.24(75.55to135.45) | NA | NA |
| DALYs (Disability-Adjusted Life Years) | Samoa | 1990 | 1995.61(1503.27to2539.75) | NA | NA |
| DALYs (Disability-Adjusted Life Years) | Samoa | 2021 | 1396.39(1123.6to1732.57) | -30.03(-40.48to-15.07) | -0.94(-1.15 to -0.74) |
| Prevalence | Samoa | 1990 | 2558.6(2284.63to2887.52) | NA | NA |
| Prevalence | Samoa | 2021 | 2293.9(2022.62to2584.23) | -10.35(-17.34to-1.88) | -0.37(-0.52 to -0.22) |
| Deaths | San Marino | 2021 | 6.05(4.27to8.3) | -52.66(-66.41to-33.19) | -1.68(-21.66 to 23.39) |
| Deaths | San Marino | 1990 | 12.78(10.64to15.07) | NA | NA |
| DALYs (Disability-Adjusted Life Years) | San Marino | 1990 | 297.48(259.03to337.09) | NA | NA |
| DALYs (Disability-Adjusted Life Years) | San Marino | 2021 | 183.81(148.11to222.92) | -38.21(-49.34to-24.25) | -0.93(-2.18 to 0.34) |
| Prevalence | San Marino | 1990 | 2393.14(2095.26to2701.08) | NA | NA |
| Prevalence | San Marino | 2021 | 2154.35(1897.78to2430.94) | -9.98(-15.8to-3.9) | -0.37(-0.68 to -0.06) |
| Deaths | Sao Tome and Principe | 1990 | 64.61(53.85to75.99) | NA | NA |
| Deaths | Sao Tome and Principe | 2021 | 58.43(44.21to72.16) | -9.56(-29.4to24.56) | -0.65(-2.68 to 1.43) |
| DALYs (Disability-Adjusted Life Years) | Sao Tome and Principe | 2021 | 1269.66(1005.65to1563.85) | -6.61(-25.67to25.49) | -0.4(-0.67 to -0.13) |
| DALYs (Disability-Adjusted Life Years) | Sao Tome and Principe | 1990 | 1359.56(1157.63to1583.01) | NA | NA |
| Prevalence | Sao Tome and Principe | 1990 | 1771.14(1580.29to2002.84) | NA | NA |
| Prevalence | Sao Tome and Principe | 2021 | 2084.48(1858.31to2339.77) | 17.69(10.67to25.86) | 0.42(0.22 to 0.61) |
| Deaths | Saudi Arabia | 1990 | 33.75(24.89to42.77) | NA | NA |
| Deaths | Saudi Arabia | 2021 | 22.69(18.55to27.31) | -32.77(-49.59to-0.5) | -0.73(-0.95 to -0.51) |
| DALYs (Disability-Adjusted Life Years) | Saudi Arabia | 2021 | 533.08(452.57to618.8) | -23.38(-41.54to7.49) | -0.41(-0.52 to -0.31) |
| DALYs (Disability-Adjusted Life Years) | Saudi Arabia | 1990 | 695.74(529.3to865.83) | NA | NA |
| Prevalence | Saudi Arabia | 1990 | 1500.78(1312.36to1711.53) | NA | NA |
| Prevalence | Saudi Arabia | 2021 | 2050.86(1796.44to2325.25) | 36.65(26.63to46.6) | 0.9(0.88 to 0.92) |
| Deaths | Senegal | 1990 | 33.86(27.28to41.41) | NA | NA |
| Deaths | Senegal | 2021 | 25.94(20.06to33.05) | -23.4(-43.46to1.59) | -0.43(-0.82 to -0.05) |
| DALYs (Disability-Adjusted Life Years) | Senegal | 2021 | 632.07(513.66to758.44) | -19.4(-36to2.44) | -0.27(-0.32 to -0.21) |
| DALYs (Disability-Adjusted Life Years) | Senegal | 1990 | 784.23(657.4to937.47) | NA | NA |
| Prevalence | Senegal | 1990 | 1545.82(1379.87to1731.84) | NA | NA |
| Prevalence | Senegal | 2021 | 1680.25(1479.89to1901.31) | 8.7(1.58to16.75) | 0.26(0.23 to 0.28) |
| Deaths | Serbia | 1990 | 33.2(28.49to40.21) | NA | NA |
| Deaths | Serbia | 2021 | 19.76(16.6to23.28) | -40.48(-52.91to-23.23) | -1.79(-2.37 to -1.21) |
| DALYs (Disability-Adjusted Life Years) | Serbia | 1990 | 679.95(593.41to787.76) | NA | NA |
| DALYs (Disability-Adjusted Life Years) | Serbia | 2021 | 475.15(406.91to548.67) | -30.12(-42.26to-13.95) | -1.17(-1.35 to -0.99) |
| Prevalence | Serbia | 1990 | 2382.85(2122.66to2676.2) | NA | NA |
| Prevalence | Serbia | 2021 | 2692.46(2420.28to2989.34) | 12.99(5.79to21.65) | 0.23(0.21 to 0.25) |
| Deaths | Seychelles | 2021 | 23.68(20.4to27.46) | -32.91(-42.1to-20.19) | -1.7(-5.14 to 1.85) |
| Deaths | Seychelles | 1990 | 35.3(30.23to39.7) | NA | NA |
| DALYs (Disability-Adjusted Life Years) | Seychelles | 2021 | 538.59(475.57to600.26) | -30.31(-38.43to-21.11) | -1.26(-1.67 to -0.84) |
| DALYs (Disability-Adjusted Life Years) | Seychelles | 1990 | 772.86(673.87to857.06) | NA | NA |
| Prevalence | Seychelles | 1990 | 1616.22(1413.06to1858.05) | NA | NA |
| Prevalence | Seychelles | 2021 | 1835.81(1598.8to2078.76) | 13.59(5.36to24.05) | 0.16(-0.06 to 0.37) |
| Deaths | Sierra Leone | 1990 | 34.13(26.77to41.62) | NA | NA |
| Deaths | Sierra Leone | 2021 | 25.64(18.37to34.62) | -24.86(-45.74to6.05) | -0.2(-0.72 to 0.32) |
| DALYs (Disability-Adjusted Life Years) | Sierra Leone | 1990 | 785.46(645.47to927.38) | NA | NA |
| DALYs (Disability-Adjusted Life Years) | Sierra Leone | 2021 | 646.88(505.84to831.93) | -17.64(-35.83to7.64) | -0.15(-0.21 to -0.09) |
| Prevalence | Sierra Leone | 1990 | 1608.64(1403.39to1817.18) | NA | NA |
| Prevalence | Sierra Leone | 2021 | 1770.38(1550.59to1987.35) | 10.05(2.5to16.77) | 0.26(0.23 to 0.3) |
| Deaths | Singapore | 1990 | 40.79(38.24to42.73) | NA | NA |
| Deaths | Singapore | 2021 | 5.93(5.19to6.4) | -85.46(-86.8to-84.4) | -5.87(-6.93 to -4.8) |
| DALYs (Disability-Adjusted Life Years) | Singapore | 1990 | 770.14(729.76to806.94) | NA | NA |
| DALYs (Disability-Adjusted Life Years) | Singapore | 2021 | 146.48(131.42to161.67) | -80.98(-82.63to-79.23) | -4.54(-4.74 to -4.34) |
| Prevalence | Singapore | 1990 | 1459.45(1274.5to1672.62) | NA | NA |
| Prevalence | Singapore | 2021 | 922.56(805.95to1060.47) | -36.79(-44.37to-28.08) | -1.61(-1.66 to -1.55) |
| Deaths | Slovakia | 2021 | 9.62(8.09to11.53) | -27.83(-40.87to-12.11) | -1.35(-2.35 to -0.34) |
| Deaths | Slovakia | 1990 | 13.33(11.79to14.92) | NA | NA |
| DALYs (Disability-Adjusted Life Years) | Slovakia | 2021 | 272.01(238.55to314.65) | -21.5(-31.84to-8.85) | -0.69(-0.77 to -0.61) |
| DALYs (Disability-Adjusted Life Years) | Slovakia | 1990 | 346.5(309.9to378.7) | NA | NA |
| Prevalence | Slovakia | 1990 | 1916.55(1683.25to2175.87) | NA | NA |
| Prevalence | Slovakia | 2021 | 2118.52(1878.43to2394.73) | 10.54(2.24to19.6) | 0.2(0.18 to 0.23) |
| Deaths | Slovenia | 2021 | 11.49(9.92to12.67) | -56.15(-61.29to-51.18) | -4.23(-5.92 to -2.51) |
| Deaths | Slovenia | 1990 | 26.2(24.47to27.77) | NA | NA |
| DALYs (Disability-Adjusted Life Years) | Slovenia | 1990 | 580.81(547.9to617.28) | NA | NA |
| DALYs (Disability-Adjusted Life Years) | Slovenia | 2021 | 277.59(245.36to308.42) | -52.21(-56.6to-47.61) | -2.65(-2.86 to -2.44) |
| Prevalence | Slovenia | 1990 | 2157.07(1915.16to2427.36) | NA | NA |
| Prevalence | Slovenia | 2021 | 2177.73(1923.82to2436.97) | 0.96(-6.79to9.56) | -0.13(-0.17 to -0.09) |
| Deaths | Solomon Islands | 2021 | 66.46(54.6to80.63) | -24.17(-37.98to-5.55) | -0.84(-1.89 to 0.21) |
| Deaths | Solomon Islands | 1990 | 87.65(70.35to106.08) | NA | NA |
| DALYs (Disability-Adjusted Life Years) | Solomon Islands | 1990 | 1791.8(1431.4to2207.36) | NA | NA |
| DALYs (Disability-Adjusted Life Years) | Solomon Islands | 2021 | 1368.16(1142.77to1653.18) | -23.64(-37.91to-3.49) | -0.74(-0.9 to -0.58) |
| Prevalence | Solomon Islands | 1990 | 2689.84(2424.83to2989.04) | NA | NA |
| Prevalence | Solomon Islands | 2021 | 2490.64(2242.06to2783.82) | -7.41(-12.2to-2.18) | -0.23(-0.34 to -0.11) |
| Deaths | Somalia | 2021 | 41.35(23.88to57.88) | -29.01(-45.18to-9.45) | -0.92(-1.21 to -0.63) |
| Deaths | Somalia | 1990 | 58.26(35.7to77.92) | NA | NA |
| DALYs (Disability-Adjusted Life Years) | Somalia | 2021 | 1040.23(683.92to1374.61) | -20.81(-35.52to-2.02) | -0.66(-0.7 to -0.61) |
| DALYs (Disability-Adjusted Life Years) | Somalia | 1990 | 1313.57(868.25to1712.3) | NA | NA |
| Prevalence | Somalia | 1990 | 1818.53(1585.9to2049.83) | NA | NA |
| Prevalence | Somalia | 2021 | 2116.82(1858.64to2404.17) | 16.4(10.02to24.06) | 0.37(0.33 to 0.4) |
| Deaths | South Africa | 1990 | 36.18(31.67to44.58) | NA | NA |
| Deaths | South Africa | 2021 | 34.01(31.2to36.95) | -6(-22.48to6.38) | -0.87(-1.08 to -0.66) |
| DALYs (Disability-Adjusted Life Years) | South Africa | 1990 | 886.2(796.06to1027.79) | NA | NA |
| DALYs (Disability-Adjusted Life Years) | South Africa | 2021 | 853.41(789.28to935.98) | -3.7(-14.23to5.59) | -0.65(-0.78 to -0.51) |
| Prevalence | South Africa | 1990 | 2165.91(1915.93to2430.8) | NA | NA |
| Prevalence | South Africa | 2021 | 2170.53(1917.47to2437.23) | 0.21(-2.94to3.47) | -0.18(-0.22 to -0.15) |
| Deaths | South Sudan | 1990 | 47.35(31.45to62.62) | NA | NA |
| Deaths | South Sudan | 2021 | 37.74(25.3to52.04) | -20.29(-41.82to3.67) | -0.48(-0.9 to -0.05) |
| DALYs (Disability-Adjusted Life Years) | South Sudan | 2021 | 863.67(616.81to1149.37) | -16.35(-36.01to7.85) | -0.41(-0.46 to -0.36) |
| DALYs (Disability-Adjusted Life Years) | South Sudan | 1990 | 1032.44(731.85to1333.2) | NA | NA |
| Prevalence | South Sudan | 1990 | 1462.63(1276.5to1647.56) | NA | NA |
| Prevalence | South Sudan | 2021 | 1601(1404.86to1818.43) | 9.46(0.8to19.76) | 0.17(0.14 to 0.21) |
| Deaths | Spain | 2021 | 21.98(19to23.95) | -37.72(-42.56to-33.28) | -2.42(-2.71 to -2.13) |
| Deaths | Spain | 1990 | 35.29(32.23to37.39) | NA | NA |
| DALYs (Disability-Adjusted Life Years) | Spain | 2021 | 410.16(370.27to444.09) | -38.09(-41.95to-34.38) | -1.66(-1.77 to -1.55) |
| DALYs (Disability-Adjusted Life Years) | Spain | 1990 | 662.52(622.96to704.42) | NA | NA |
| Prevalence | Spain | 1990 | 2803.96(2554.06to3127.78) | NA | NA |
| Prevalence | Spain | 2021 | 2658.24(2409.07to2950.54) | -5.2(-11.51to3.17) | -0.31(-0.34 to -0.27) |
| Deaths | Sri Lanka | 1990 | 76.91(65.98to85.8) | NA | NA |
| Deaths | Sri Lanka | 2021 | 46.21(31.82to60.72) | -39.92(-58.11to-13.76) | -2.59(-2.88 to -2.3) |
| DALYs (Disability-Adjusted Life Years) | Sri Lanka | 1990 | 1428.46(1248.27to1590.9) | NA | NA |
| DALYs (Disability-Adjusted Life Years) | Sri Lanka | 2021 | 864.19(631.53to1103.11) | -39.5(-55.78to-15.78) | -1.97(-2.12 to -1.83) |
| Prevalence | Sri Lanka | 1990 | 1797.97(1578.41to2033.2) | NA | NA |
| Prevalence | Sri Lanka | 2021 | 1923.09(1670.3to2193.97) | 6.96(-0.53to15.51) | 0.02(0.01 to 0.04) |
| Deaths | Sudan | 2021 | 25.51(16.28to36.36) | -26.31(-48.96to25.85) | -1.38(-1.56 to -1.2) |
| Deaths | Sudan | 1990 | 34.62(14.42to57.01) | NA | NA |
| DALYs (Disability-Adjusted Life Years) | Sudan | 1990 | 823.9(425.43to1245.79) | NA | NA |
| DALYs (Disability-Adjusted Life Years) | Sudan | 2021 | 623.17(442.16to841.65) | -24.36(-44.6to14.78) | -1.06(-1.11 to -1.02) |
| Prevalence | Sudan | 1990 | 2319.16(2042.52to2601.73) | NA | NA |
| Prevalence | Sudan | 2021 | 2416.13(2114.44to2730.3) | 4.18(-3.16to12.15) | -0.06(-0.09 to -0.03) |
| Deaths | Suriname | 1990 | 19.56(17.31to21.84) | NA | NA |
| Deaths | Suriname | 2021 | 15.32(11.86to19.65) | -21.69(-41.48to1.63) | -0.2(-2.04 to 1.67) |
| DALYs (Disability-Adjusted Life Years) | Suriname | 2021 | 355.3(284.8to434.64) | -15.2(-33.54to5.22) | -0.15(-0.39 to 0.09) |
| DALYs (Disability-Adjusted Life Years) | Suriname | 1990 | 418.99(379.12to465.36) | NA | NA |
| Prevalence | Suriname | 1990 | 1389.28(1215.73to1605.01) | NA | NA |
| Prevalence | Suriname | 2021 | 1581.99(1391.61to1787.28) | 13.87(7.4to21.09) | 0.28(0.18 to 0.39) |
| Deaths | Sweden | 2021 | 13.51(11.69to14.92) | 31.86(18.07to44.65) | -0.23(-1.2 to 0.75) |
| Deaths | Sweden | 1990 | 10.24(9.52to10.86) | NA | NA |
| DALYs (Disability-Adjusted Life Years) | Sweden | 1990 | 314.04(287.47to343.6) | NA | NA |
| DALYs (Disability-Adjusted Life Years) | Sweden | 2021 | 339.36(304.58to374.57) | 8.06(0.89to15.56) | -0.25(-0.37 to -0.14) |
| Prevalence | Sweden | 1990 | 3209.08(2898.46to3557.98) | NA | NA |
| Prevalence | Sweden | 2021 | 3097.64(2760.32to3448.67) | -3.47(-8.29to1.09) | -0.29(-0.33 to -0.25) |
| Deaths | Switzerland | 2021 | 11.31(9.69to12.43) | -39.98(-46.27to-33.96) | -1.84(-2.81 to -0.87) |
| Deaths | Switzerland | 1990 | 18.85(17.5to20.08) | NA | NA |
| DALYs (Disability-Adjusted Life Years) | Switzerland | 2021 | 289.27(259.16to319.86) | -34.85(-39.27to-30.21) | -1.16(-1.27 to -1.05) |
| DALYs (Disability-Adjusted Life Years) | Switzerland | 1990 | 444.04(412.42to477.44) | NA | NA |
| Prevalence | Switzerland | 1990 | 2766.29(2489.87to3086.66) | NA | NA |
| Prevalence | Switzerland | 2021 | 2548.98(2306.28to2820.47) | -7.86(-14.75to-0.15) | -0.46(-0.5 to -0.43) |
| Deaths | Syrian Arab Republic | 2021 | 25.82(20.35to31.73) | 0.78(-30.77to49.75) | -0.4(-0.68 to -0.12) |
| Deaths | Syrian Arab Republic | 1990 | 25.62(18.9to32.53) | NA | NA |
| DALYs (Disability-Adjusted Life Years) | Syrian Arab Republic | 1990 | 605.31(469.6to746.18) | NA | NA |
| DALYs (Disability-Adjusted Life Years) | Syrian Arab Republic | 2021 | 605.91(498.55to740.44) | 0.1(-26.07to42.49) | -0.23(-0.29 to -0.17) |
| Prevalence | Syrian Arab Republic | 1990 | 1980.98(1754.23to2235.69) | NA | NA |
| Prevalence | Syrian Arab Republic | 2021 | 2446.57(2166.79to2749.06) | 23.5(16.34to31.88) | 0.47(0.45 to 0.49) |
| Deaths | Taiwan (Province of China) | 2021 | 17.06(14.87to18.72) | -41.03(-46.5to-35.68) | -2.64(-3.2 to -2.08) |
| Deaths | Taiwan (Province of China) | 1990 | 28.93(26.59to30.62) | NA | NA |
| DALYs (Disability-Adjusted Life Years) | Taiwan (Province of China) | 2021 | 343.42(311.47to377.54) | -40.52(-44.89to-36.11) | -1.67(-1.78 to -1.56) |
| DALYs (Disability-Adjusted Life Years) | Taiwan (Province of China) | 1990 | 577.34(539.63to617.83) | NA | NA |
| Prevalence | Taiwan (Province of China) | 1990 | 1932.15(1713.87to2155.84) | NA | NA |
| Prevalence | Taiwan (Province of China) | 2021 | 1512.21(1331.26to1725.43) | -21.73(-30.03to-12.74) | -0.8(-0.82 to -0.77) |
| Deaths | Tajikistan | 2021 | 29.1(20.8to38.89) | -39.93(-58.54to-16.65) | -2.85(-3.16 to -2.53) |
| Deaths | Tajikistan | 1990 | 48.45(39.12to59.98) | NA | NA |
| DALYs (Disability-Adjusted Life Years) | Tajikistan | 1990 | 1063.76(900.02to1271.71) | NA | NA |
| DALYs (Disability-Adjusted Life Years) | Tajikistan | 2021 | 637.12(492.25to826.72) | -40.11(-54.29to-20.79) | -2.42(-2.55 to -2.29) |
| Prevalence | Tajikistan | 1990 | 2522.65(2239.46to2841.52) | NA | NA |
| Prevalence | Tajikistan | 2021 | 2519.36(2214.07to2838.91) | -0.13(-6.61to5.23) | -0.15(-0.18 to -0.12) |
| Deaths | Thailand | 1990 | 55.13(45.52to64.14) | NA | NA |
| Deaths | Thailand | 2021 | 19.8(15.22to24.97) | -64.09(-73.21to-51.99) | -4.24(-4.55 to -3.93) |
| DALYs (Disability-Adjusted Life Years) | Thailand | 1990 | 1179.1(1010.81to1352.99) | NA | NA |
| DALYs (Disability-Adjusted Life Years) | Thailand | 2021 | 461.07(381.24to559.88) | -60.9(-69to-50.52) | -3.36(-3.55 to -3.16) |
| Prevalence | Thailand | 1990 | 2128.97(1914.28to2332.72) | NA | NA |
| Prevalence | Thailand | 2021 | 1711(1490.46to1934.21) | -19.63(-26.19to-13.32) | -0.78(-0.82 to -0.74) |
| Deaths | Timor-Leste | 1990 | 61.07(40.32to78.21) | NA | NA |
| Deaths | Timor-Leste | 2021 | 51.35(38.52to65.43) | -15.91(-39.28to17.91) | -0.66(-1.67 to 0.35) |
| DALYs (Disability-Adjusted Life Years) | Timor-Leste | 1990 | 1262.75(875.76to1586.04) | NA | NA |
| DALYs (Disability-Adjusted Life Years) | Timor-Leste | 2021 | 1047.94(814.39to1288.81) | -17.01(-37.74to14.06) | -0.6(-0.73 to -0.47) |
| Prevalence | Timor-Leste | 1990 | 2333.86(2068.8to2654.4) | NA | NA |
| Prevalence | Timor-Leste | 2021 | 2295.43(2041.89to2596.01) | -1.65(-8.22to5.42) | -0.25(-0.33 to -0.18) |
| Deaths | Togo | 1990 | 35.09(26.98to42.06) | NA | NA |
| Deaths | Togo | 2021 | 28.22(20.94to38.12) | -19.6(-38.16to9.22) | -0.29(-0.78 to 0.2) |
| DALYs (Disability-Adjusted Life Years) | Togo | 1990 | 816.2(657.06to956.45) | NA | NA |
| DALYs (Disability-Adjusted Life Years) | Togo | 2021 | 705.07(558.67to907.89) | -13.61(-31.11to11.06) | -0.18(-0.24 to -0.11) |
| Prevalence | Togo | 1990 | 1590.26(1393.99to1796.69) | NA | NA |
| Prevalence | Togo | 2021 | 1725.8(1505.49to1960.26) | 8.52(2.86to15.49) | 0.24(0.2 to 0.29) |
| Deaths | Tokelau | 2021 | 45.39(33.12to66.2) | -34.06(-51.27to-6) | -1.55(-19.46 to 20.34) |
| Deaths | Tokelau | 1990 | 68.84(48.23to91.92) | NA | NA |
| DALYs (Disability-Adjusted Life Years) | Tokelau | 1990 | 1385.25(982.56to1830.78) | NA | NA |
| DALYs (Disability-Adjusted Life Years) | Tokelau | 2021 | 921.21(704.89to1227.57) | -33.5(-48.61to-8.96) | -1.41(-3.97 to 1.22) |
| Prevalence | Tokelau | 1990 | 2176.87(1933.55to2457.95) | NA | NA |
| Prevalence | Tokelau | 2021 | 1887.77(1641.64to2144.29) | -13.28(-19.38to-6.46) | -0.6(-2.19 to 1.02) |
| Deaths | Tonga | 2021 | 48.96(39.77to58.69) | -24.86(-42.94to-1.99) | -0.94(-3.52 to 1.72) |
| Deaths | Tonga | 1990 | 65.16(52.91to76.25) | NA | NA |
| DALYs (Disability-Adjusted Life Years) | Tonga | 1990 | 1330.02(1114.81to1535.57) | NA | NA |
| DALYs (Disability-Adjusted Life Years) | Tonga | 2021 | 988.89(814.24to1161.4) | -25.65(-42.35to-4.52) | -0.89(-1.23 to -0.55) |
| Prevalence | Tonga | 1990 | 2330.29(2045.18to2631.68) | NA | NA |
| Prevalence | Tonga | 2021 | 1951.31(1690.26to2217.16) | -16.26(-23.26to-8.64) | -0.61(-0.82 to -0.4) |
| Deaths | Trinidad and Tobago | 1990 | 14.63(13.77to15.44) | NA | NA |
| Deaths | Trinidad and Tobago | 2021 | 10.78(8.34to13.35) | -26.3(-42.74to-8.8) | -0.26(-1.77 to 1.27) |
| DALYs (Disability-Adjusted Life Years) | Trinidad and Tobago | 1990 | 295.55(277.46to314.49) | NA | NA |
| DALYs (Disability-Adjusted Life Years) | Trinidad and Tobago | 2021 | 256.19(210.35to308.6) | -13.32(-29.8to4.89) | -0.21(-0.39 to -0.03) |
| Prevalence | Trinidad and Tobago | 1990 | 1285.24(1117.94to1448.19) | NA | NA |
| Prevalence | Trinidad and Tobago | 2021 | 1434.75(1240.91to1640.26) | 11.63(1.62to22.35) | 0.25(0.19 to 0.32) |
| Deaths | Tunisia | 2021 | 17.61(12.17to25.98) | -6.13(-36.01to44.52) | -0.6(-1.01 to -0.19) |
| Deaths | Tunisia | 1990 | 18.76(15.24to22.6) | NA | NA |
| DALYs (Disability-Adjusted Life Years) | Tunisia | 1990 | 463.75(400.76to538.26) | NA | NA |
| DALYs (Disability-Adjusted Life Years) | Tunisia | 2021 | 460.89(370.65to585.52) | -0.62(-21.68to33.82) | -0.17(-0.21 to -0.12) |
| Prevalence | Tunisia | 1990 | 2307.32(2031.06to2627.26) | NA | NA |
| Prevalence | Tunisia | 2021 | 2808.2(2494.08to3138.15) | 21.71(15to30.19) | 0.54(0.51 to 0.56) |
| Deaths | Turkey | 2021 | 40.77(33.86to48.52) | -29.14(-44.27to-3.09) | -1.68(-1.92 to -1.43) |
| Deaths | Turkey | 1990 | 57.54(45.02to67.31) | NA | NA |
| DALYs (Disability-Adjusted Life Years) | Turkey | 2021 | 850.19(727.99to983.51) | -29.15(-42.5to-8.65) | -1.18(-1.29 to -1.06) |
| DALYs (Disability-Adjusted Life Years) | Turkey | 1990 | 1199.96(985.75to1388.47) | NA | NA |
| Prevalence | Turkey | 1990 | 2829.81(2507.77to3180.37) | NA | NA |
| Prevalence | Turkey | 2021 | 3146.68(2861.31to3477.82) | 11.2(4.71to18.88) | 0.11(0.08 to 0.15) |
| Deaths | Turkmenistan | 2021 | 11.05(8.76to13.74) | -71.27(-77.18to-63.84) | -3.65(-4.09 to -3.21) |
| Deaths | Turkmenistan | 1990 | 38.45(34.98to41.82) | NA | NA |
| DALYs (Disability-Adjusted Life Years) | Turkmenistan | 1990 | 833.48(757.54to907.96) | NA | NA |
| DALYs (Disability-Adjusted Life Years) | Turkmenistan | 2021 | 307.79(254.46to369.5) | -63.07(-69.57to-54.55) | -3.18(-3.32 to -3.04) |
| Prevalence | Turkmenistan | 1990 | 2117.2(1862.96to2407.51) | NA | NA |
| Prevalence | Turkmenistan | 2021 | 1680.97(1478.82to1909.51) | -20.6(-30.33to-11.07) | -0.91(-0.96 to -0.85) |
| Deaths | Tuvalu | 1990 | 98.16(69.99to119.81) | NA | NA |
| Deaths | Tuvalu | 2021 | 54.85(41.47to70.2) | -44.12(-56.74to-19.92) | -2.12(-7.67 to 3.77) |
| DALYs (Disability-Adjusted Life Years) | Tuvalu | 2021 | 1138.27(890.67to1437.52) | -44.75(-56.11to-24.65) | -1.88(-2.7 to -1.06) |
| DALYs (Disability-Adjusted Life Years) | Tuvalu | 1990 | 2060.07(1495.59to2496.17) | NA | NA |
| Prevalence | Tuvalu | 1990 | 2392.12(2110.52to2684.5) | NA | NA |
| Prevalence | Tuvalu | 2021 | 2094.63(1836.1to2383.5) | -12.44(-18.45to-5.44) | -0.44(-1.05 to 0.19) |
| Deaths | Uganda | 2021 | 28.93(19.57to39.04) | -35.48(-53.63to-7.96) | -1.82(-2.05 to -1.59) |
| Deaths | Uganda | 1990 | 44.84(30.24to59.36) | NA | NA |
| DALYs (Disability-Adjusted Life Years) | Uganda | 1990 | 979.47(704.19to1264.74) | NA | NA |
| DALYs (Disability-Adjusted Life Years) | Uganda | 2021 | 693.4(506.33to880.76) | -29.21(-46.18to-5.09) | -1.37(-1.43 to -1.31) |
| Prevalence | Uganda | 1990 | 1651(1443.19to1871.59) | NA | NA |
| Prevalence | Uganda | 2021 | 1663.08(1467.43to1879.94) | 0.73(-6.44to8.39) | -0.08(-0.1 to -0.06) |
| Deaths | Ukraine | 2021 | 9.39(7.1to11.8) | -80.87(-85.44to-75.78) | -6.19(-6.58 to -5.8) |
| Deaths | Ukraine | 1990 | 49.08(46.19to51.35) | NA | NA |
| DALYs (Disability-Adjusted Life Years) | Ukraine | 1990 | 1055.36(999.59to1107.94) | NA | NA |
| DALYs (Disability-Adjusted Life Years) | Ukraine | 2021 | 274.39(225.05to334.21) | -74(-78.91to-68.16) | -5.08(-5.25 to -4.9) |
| Prevalence | Ukraine | 1990 | 2636.78(2310.89to2998.17) | NA | NA |
| Prevalence | Ukraine | 2021 | 1978.64(1720.66to2252.75) | -24.96(-30.41to-18.81) | -0.93(-0.98 to -0.88) |
| Deaths | United Arab Emirates | 2021 | 26.26(18.3to33.46) | -31.01(-50.67to-9.33) | -2.2(-2.74 to -1.66) |
| Deaths | United Arab Emirates | 1990 | 38.06(28.41to49.81) | NA | NA |
| DALYs (Disability-Adjusted Life Years) | United Arab Emirates | 2021 | 594.58(488.75to713.85) | -33.74(-49.06to-18.98) | -1.36(-1.68 to -1.04) |
| DALYs (Disability-Adjusted Life Years) | United Arab Emirates | 1990 | 897.36(701.58to1161.39) | NA | NA |
| Prevalence | United Arab Emirates | 1990 | 2326.06(2060.22to2605.85) | NA | NA |
| Prevalence | United Arab Emirates | 2021 | 2686.42(2401.97to3008.25) | 15.49(8.04to23.77) | 0.28(0.22 to 0.34) |
| Deaths | United Kingdom | 2021 | 26.22(23.42to27.57) | -21.91(-26.02to-19.51) | -0.64(-1.21 to -0.08) |
| Deaths | United Kingdom | 1990 | 33.57(31.83to34.43) | NA | NA |
| DALYs (Disability-Adjusted Life Years) | United Kingdom | 2021 | 571.45(532.64to601.96) | -21.57(-24.29to-19.78) | -0.47(-0.63 to -0.32) |
| DALYs (Disability-Adjusted Life Years) | United Kingdom | 1990 | 728.64(699.01to754.24) | NA | NA |
| Prevalence | United Kingdom | 1990 | 3170.87(2852.34to3489.52) | NA | NA |
| Prevalence | United Kingdom | 2021 | 3270.26(2957.04to3570.38) | 3.13(1.02to5.31) | 0.06(0.04 to 0.08) |
| Deaths | United Republic of Tanzania | 2021 | 21.64(16.78to27.41) | -26.8(-44.19to-1.41) | -1.22(-1.44 to -0.99) |
| Deaths | United Republic of Tanzania | 1990 | 29.56(23.48to35.93) | NA | NA |
| DALYs (Disability-Adjusted Life Years) | United Republic of Tanzania | 2021 | 543.49(445.23to659.63) | -20.45(-36.17to1.67) | -0.78(-0.82 to -0.74) |
| DALYs (Disability-Adjusted Life Years) | United Republic of Tanzania | 1990 | 683.23(554.36to796.05) | NA | NA |
| Prevalence | United Republic of Tanzania | 1990 | 1381(1206.33to1560.55) | NA | NA |
| Prevalence | United Republic of Tanzania | 2021 | 1538.19(1356.18to1744.38) | 11.38(4.61to18.66) | 0.24(0.22 to 0.26) |
| Deaths | United States Virgin Islands | 1990 | 12.79(10.85to14.92) | NA | NA |
| Deaths | United States Virgin Islands | 2021 | 7.66(5.88to10.2) | -40.06(-55.97to-13.08) | -1.27(-7.51 to 5.39) |
| DALYs (Disability-Adjusted Life Years) | United States Virgin Islands | 2021 | 182.91(149.95to224.07) | -31.41(-45.25to-9.66) | -0.82(-1.52 to -0.11) |
| DALYs (Disability-Adjusted Life Years) | United States Virgin Islands | 1990 | 266.67(230.13to308.34) | NA | NA |
| Prevalence | United States Virgin Islands | 1990 | 1183.32(1031.3to1379.44) | NA | NA |
| Prevalence | United States Virgin Islands | 2021 | 1478.42(1288.27to1679.19) | 24.94(15.33to34.08) | 0.54(0.3 to 0.78) |
| Deaths | United States of America | 1990 | 26.51(24.52to27.53) | NA | NA |
| Deaths | United States of America | 2021 | 31.32(27.47to33.15) | 18.15(11.82to21.67) | 0.16(-0.07 to 0.4) |
| DALYs (Disability-Adjusted Life Years) | United States of America | 2021 | 777.93(725.17to819.94) | 7.46(4.05to10.52) | -0.08(-0.15 to -0.01) |
| DALYs (Disability-Adjusted Life Years) | United States of America | 1990 | 723.91(683.54to765.5) | NA | NA |
| Prevalence | United States of America | 1990 | 3224.12(2925.81to3516.13) | NA | NA |
| Prevalence | United States of America | 2021 | 3445.29(3263.49to3602.42) | 6.86(1.48to13.83) | -0.12(-0.2 to -0.04) |
| Deaths | Uruguay | 1990 | 30.26(28.57to31.65) | NA | NA |
| Deaths | Uruguay | 2021 | 32.36(29.74to34.48) | 6.93(0.1to13.6) | -0.11(-0.67 to 0.44) |
| DALYs (Disability-Adjusted Life Years) | Uruguay | 1990 | 641.69(611.97to670.73) | NA | NA |
| DALYs (Disability-Adjusted Life Years) | Uruguay | 2021 | 642.28(600.48to679.05) | 0.09(-5.52to6.63) | -0.09(-0.19 to 0.01) |
| Prevalence | Uruguay | 1990 | 1539.43(1344.57to1739.48) | NA | NA |
| Prevalence | Uruguay | 2021 | 1553.79(1365.19to1751.77) | 0.93(-8.91to11.69) | 0.07(0.03 to 0.11) |
| Deaths | Uzbekistan | 2021 | 8.75(7.46to10.28) | -65.09(-71.67to-56.77) | -4.86(-5.12 to -4.61) |
| Deaths | Uzbekistan | 1990 | 25.07(21.61to29.76) | NA | NA |
| DALYs (Disability-Adjusted Life Years) | Uzbekistan | 1990 | 583.73(513.5to662.35) | NA | NA |
| DALYs (Disability-Adjusted Life Years) | Uzbekistan | 2021 | 238.39(206.48to271.83) | -59.16(-65.35to-51.56) | -3.73(-3.88 to -3.57) |
| Prevalence | Uzbekistan | 1990 | 2209.57(1939.21to2492.48) | NA | NA |
| Prevalence | Uzbekistan | 2021 | 1880.94(1663.83to2124.79) | -14.87(-21.9to-6.7) | -0.7(-0.74 to -0.66) |
| Deaths | Vanuatu | 2021 | 83.39(54.05to112.14) | -27.82(-41.62to-10.55) | -1.17(-2.32 to 0) |
| Deaths | Vanuatu | 1990 | 115.53(74.14to152.7) | NA | NA |
| DALYs (Disability-Adjusted Life Years) | Vanuatu | 1990 | 2338.24(1539.15to3050.72) | NA | NA |
| DALYs (Disability-Adjusted Life Years) | Vanuatu | 2021 | 1709.11(1132.62to2218.1) | -26.91(-41.14to-9.82) | -1.08(-1.27 to -0.89) |
| Prevalence | Vanuatu | 1990 | 2578.44(2309.33to2883.67) | NA | NA |
| Prevalence | Vanuatu | 2021 | 2322.9(2051.12to2603.16) | -9.91(-14.51to-4.76) | -0.3(-0.47 to -0.13) |
| Deaths | Venezuela (Bolivarian Republic of) | 1990 | 18.86(17.26to19.98) | NA | NA |
| Deaths | Venezuela (Bolivarian Republic of) | 2021 | 22.12(17.16to27.82) | 17.31(-7.49to46.98) | -0.09(-0.38 to 0.21) |
| DALYs (Disability-Adjusted Life Years) | Venezuela (Bolivarian Republic of) | 2021 | 462.77(370.7to574.17) | 15.17(-6.1to41.64) | 0.07(-0.01 to 0.15) |
| DALYs (Disability-Adjusted Life Years) | Venezuela (Bolivarian Republic of) | 1990 | 401.81(373.82to426.23) | NA | NA |
| Prevalence | Venezuela (Bolivarian Republic of) | 1990 | 1679.96(1471.73to1892.15) | NA | NA |
| Prevalence | Venezuela (Bolivarian Republic of) | 2021 | 2120.81(1898.04to2390.93) | 26.24(16.55to37.05) | 0.52(0.51 to 0.54) |
| Deaths | Viet Nam | 1990 | 54.29(39.97to67.85) | NA | NA |
| Deaths | Viet Nam | 2021 | 43.96(35.11to54.17) | -19.01(-41.66to26.28) | -0.91(-1.03 to -0.79) |
| DALYs (Disability-Adjusted Life Years) | Viet Nam | 2021 | 863.32(702.77to1024.54) | -18.15(-38.16to20.31) | -0.65(-0.71 to -0.59) |
| DALYs (Disability-Adjusted Life Years) | Viet Nam | 1990 | 1054.72(810.9to1299.01) | NA | NA |
| Prevalence | Viet Nam | 1990 | 1990.22(1756.48to2275.43) | NA | NA |
| Prevalence | Viet Nam | 2021 | 2291.31(2035.09to2592.2) | 15.13(8.17to21.6) | 0.26(0.24 to 0.27) |
| Deaths | Yemen | 2021 | 31.26(22.66to41.27) | -17.86(-42.49to32.96) | -1.02(-1.25 to -0.79) |
| Deaths | Yemen | 1990 | 38.05(20.06to53.44) | NA | NA |
| DALYs (Disability-Adjusted Life Years) | Yemen | 1990 | 905.15(560.94to1202.82) | NA | NA |
| DALYs (Disability-Adjusted Life Years) | Yemen | 2021 | 730.21(567.09to920.09) | -19.33(-40.06to17.68) | -0.78(-0.85 to -0.71) |
| Prevalence | Yemen | 1990 | 2474.97(2201.61to2791.86) | NA | NA |
| Prevalence | Yemen | 2021 | 2478.52(2169.71to2808.67) | 0.14(-8.76to7.49) | -0.09(-0.12 to -0.06) |
| Deaths | Zambia | 2021 | 29.44(23.12to36.83) | -14.21(-36.53to20.19) | -0.76(-1.1 to -0.4) |
| Deaths | Zambia | 1990 | 34.32(25.83to42.74) | NA | NA |
| DALYs (Disability-Adjusted Life Years) | Zambia | 2021 | 705.51(572.04to851.09) | -9.88(-30.99to23.56) | -0.54(-0.6 to -0.48) |
| DALYs (Disability-Adjusted Life Years) | Zambia | 1990 | 782.84(607.9to948.22) | NA | NA |
| Prevalence | Zambia | 1990 | 1413.24(1237.48to1606.52) | NA | NA |
| Prevalence | Zambia | 2021 | 1547.52(1346.26to1774.17) | 9.5(1.32to18.48) | 0.17(0.14 to 0.2) |
| Deaths | Zimbabwe | 1990 | 27.4(22.18to33.61) | NA | NA |
| Deaths | Zimbabwe | 2021 | 28.99(22.37to35.79) | 5.81(-18.67to38.67) | 0.8(0.37 to 1.24) |
| DALYs (Disability-Adjusted Life Years) | Zimbabwe | 2021 | 721.09(593.96to856.23) | 11.9(-8.44to37.41) | 0.6(0.52 to 0.68) |
| DALYs (Disability-Adjusted Life Years) | Zimbabwe | 1990 | 644.41(540.4to755.08) | NA | NA |
| Prevalence | Zimbabwe | 1990 | 1683.08(1461.65to1912.08) | NA | NA |
| Prevalence | Zimbabwe | 2021 | 1798.65(1573.57to2076.37) | 6.87(0.99to14) | 0.21(0.17 to 0.25) |
