# Supplementary material for: Global, regional, and national temporal trends in prevalence, deaths and disability-adjusted life years for chronic pulmonary disease, 1990–2021: an age-period-cohort analysis based on the global burden of disease study 2021
Source: Front Med (Lausanne). 2025 Mar 4;12:1554442. doi: 10.3389/fmed.2025.1554442 (PMC11913687; doi:10.3389/fmed.2025.1554442)
Supplement: Supplementary file 4 [file Table_2.docx]

Table S2 The local drift of Deaths prevalence DALYs from 1990 to 2021 for COPD across SDI quintiles Location

| **Region** | **measure** | **age** | **LocalDrift(%)** |
| --- | --- | --- | --- |
| global | Prevalence | 20 ~ 25 | -0.31(-0.38 to -0.23) |
| global | Prevalence | 25 ~ 30 | -0.33(-0.39 to -0.28) |
| global | Prevalence | 30 ~ 35 | -0.35(-0.4 to -0.31) |
| global | Prevalence | 35 ~ 40 | -0.36(-0.4 to -0.32) |
| global | Prevalence | 40 ~ 45 | -0.38(-0.41 to -0.34) |
| global | Prevalence | 45 ~ 50 | -0.39(-0.42 to -0.36) |
| global | Prevalence | 50 ~ 55 | -0.35(-0.38 to -0.33) |
| global | Prevalence | 55 ~ 60 | -0.27(-0.3 to -0.25) |
| global | Prevalence | 60 ~ 65 | -0.18(-0.2 to -0.15) |
| global | Prevalence | 65 ~ 70 | -0.08(-0.1 to -0.06) |
| global | Prevalence | 70 ~ 75 | 0(-0.02 to 0.02) |
| global | Prevalence | 75 ~ 80 | 0.09(0.07 to 0.12) |
| global | Prevalence | 80 ~ 85 | 0.18(0.15 to 0.21) |
| global | Prevalence | 85 ~ 90 | 0.29(0.25 to 0.33) |
| global | Prevalence | 90 ~ 95 | 0.41(0.33 to 0.49) |
| global | Prevalence | 20 ~ 25 | -0.31(-0.38 to -0.23) |
| global | Prevalence | 25 ~ 30 | -0.33(-0.39 to -0.28) |
| global | Prevalence | 30 ~ 35 | -0.35(-0.4 to -0.31) |
| global | Prevalence | 35 ~ 40 | -0.36(-0.4 to -0.32) |
| global | Prevalence | 40 ~ 45 | -0.38(-0.41 to -0.34) |
| global | Prevalence | 45 ~ 50 | -0.39(-0.42 to -0.36) |
| global | Prevalence | 50 ~ 55 | -0.35(-0.38 to -0.33) |
| global | Prevalence | 55 ~ 60 | -0.27(-0.3 to -0.25) |
| global | Prevalence | 60 ~ 65 | -0.18(-0.2 to -0.15) |
| global | Prevalence | 65 ~ 70 | -0.08(-0.1 to -0.06) |
| global | Prevalence | 70 ~ 75 | 0(-0.02 to 0.02) |
| global | Prevalence | 75 ~ 80 | 0.09(0.07 to 0.12) |
| global | Prevalence | 80 ~ 85 | 0.18(0.15 to 0.21) |
| global | Prevalence | 85 ~ 90 | 0.29(0.25 to 0.33) |
| global | Prevalence | 90 ~ 95 | 0.41(0.33 to 0.49) |
| global | Prevalence | 20 ~ 25 | -0.31(-0.38 to -0.23) |
| global | Prevalence | 25 ~ 30 | -0.33(-0.39 to -0.28) |
| global | Prevalence | 30 ~ 35 | -0.35(-0.4 to -0.31) |
| global | Prevalence | 35 ~ 40 | -0.36(-0.4 to -0.32) |
| global | Prevalence | 40 ~ 45 | -0.38(-0.41 to -0.34) |
| global | Prevalence | 45 ~ 50 | -0.39(-0.42 to -0.36) |
| global | Prevalence | 50 ~ 55 | -0.35(-0.38 to -0.33) |
| global | Prevalence | 55 ~ 60 | -0.27(-0.3 to -0.25) |
| global | Prevalence | 60 ~ 65 | -0.18(-0.2 to -0.15) |
| global | Prevalence | 65 ~ 70 | -0.08(-0.1 to -0.06) |
| global | Prevalence | 70 ~ 75 | 0(-0.02 to 0.02) |
| global | Prevalence | 75 ~ 80 | 0.09(0.07 to 0.12) |
| global | Prevalence | 80 ~ 85 | 0.18(0.15 to 0.21) |
| global | Prevalence | 85 ~ 90 | 0.29(0.25 to 0.33) |
| global | Prevalence | 90 ~ 95 | 0.41(0.33 to 0.49) |
| global | Prevalence | 20 ~ 25 | -0.31(-0.38 to -0.23) |
| global | Prevalence | 25 ~ 30 | -0.33(-0.39 to -0.28) |
| global | Prevalence | 30 ~ 35 | -0.35(-0.4 to -0.31) |
| global | Prevalence | 35 ~ 40 | -0.36(-0.4 to -0.32) |
| global | Prevalence | 40 ~ 45 | -0.38(-0.41 to -0.34) |
| global | Prevalence | 45 ~ 50 | -0.39(-0.42 to -0.36) |
| global | Prevalence | 50 ~ 55 | -0.35(-0.38 to -0.33) |
| global | Prevalence | 55 ~ 60 | -0.27(-0.3 to -0.25) |
| global | Prevalence | 60 ~ 65 | -0.18(-0.2 to -0.15) |
| global | Prevalence | 65 ~ 70 | -0.08(-0.1 to -0.06) |
| global | Prevalence | 70 ~ 75 | 0(-0.02 to 0.02) |
| global | Prevalence | 75 ~ 80 | 0.09(0.07 to 0.12) |
| global | Prevalence | 80 ~ 85 | 0.18(0.15 to 0.21) |
| global | Prevalence | 85 ~ 90 | 0.29(0.25 to 0.33) |
| global | Prevalence | 90 ~ 95 | 0.41(0.33 to 0.49) |
| global | Deaths | 20 ~ 25 | -2.36(-3.07 to -1.65) |
| global | Deaths | 25 ~ 30 | -2.44(-2.91 to -1.96) |
| global | Deaths | 30 ~ 35 | -2.46(-2.81 to -2.12) |
| global | Deaths | 35 ~ 40 | -2.45(-2.71 to -2.19) |
| global | Deaths | 40 ~ 45 | -2.42(-2.61 to -2.22) |
| global | Deaths | 45 ~ 50 | -2.46(-2.6 to -2.32) |
| global | Deaths | 50 ~ 55 | -2.46(-2.57 to -2.36) |
| global | Deaths | 55 ~ 60 | -2.45(-2.53 to -2.38) |
| global | Deaths | 60 ~ 65 | -2.42(-2.48 to -2.36) |
| global | Deaths | 65 ~ 70 | -2.26(-2.3 to -2.21) |
| global | Deaths | 70 ~ 75 | -2.09(-2.13 to -2.06) |
| global | Deaths | 75 ~ 80 | -1.86(-1.9 to -1.82) |
| global | Deaths | 80 ~ 85 | -1.47(-1.51 to -1.43) |
| global | Deaths | 85 ~ 90 | -1.03(-1.08 to -0.97) |
| global | Deaths | 90 ~ 95 | -0.56(-0.66 to -0.46) |
| global | Deaths | 20 ~ 25 | -2.36(-3.07 to -1.65) |
| global | Deaths | 25 ~ 30 | -2.44(-2.91 to -1.96) |
| global | Deaths | 30 ~ 35 | -2.46(-2.81 to -2.12) |
| global | Deaths | 35 ~ 40 | -2.45(-2.71 to -2.19) |
| global | Deaths | 40 ~ 45 | -2.42(-2.61 to -2.22) |
| global | Deaths | 45 ~ 50 | -2.46(-2.6 to -2.32) |
| global | Deaths | 50 ~ 55 | -2.46(-2.57 to -2.36) |
| global | Deaths | 55 ~ 60 | -2.45(-2.53 to -2.38) |
| global | Deaths | 60 ~ 65 | -2.42(-2.48 to -2.36) |
| global | Deaths | 65 ~ 70 | -2.26(-2.3 to -2.21) |
| global | Deaths | 70 ~ 75 | -2.09(-2.13 to -2.06) |
| global | Deaths | 75 ~ 80 | -1.86(-1.9 to -1.82) |
| global | Deaths | 80 ~ 85 | -1.47(-1.51 to -1.43) |
| global | Deaths | 85 ~ 90 | -1.03(-1.08 to -0.97) |
| global | Deaths | 90 ~ 95 | -0.56(-0.66 to -0.46) |
| global | Deaths | 20 ~ 25 | -2.36(-3.07 to -1.65) |
| global | Deaths | 25 ~ 30 | -2.44(-2.91 to -1.96) |
| global | Deaths | 30 ~ 35 | -2.46(-2.81 to -2.12) |
| global | Deaths | 35 ~ 40 | -2.45(-2.71 to -2.19) |
| global | Deaths | 40 ~ 45 | -2.42(-2.61 to -2.22) |
| global | Deaths | 45 ~ 50 | -2.46(-2.6 to -2.32) |
| global | Deaths | 50 ~ 55 | -2.46(-2.57 to -2.36) |
| global | Deaths | 55 ~ 60 | -2.45(-2.53 to -2.38) |
| global | Deaths | 60 ~ 65 | -2.42(-2.48 to -2.36) |
| global | Deaths | 65 ~ 70 | -2.26(-2.3 to -2.21) |
| global | Deaths | 70 ~ 75 | -2.09(-2.13 to -2.06) |
| global | Deaths | 75 ~ 80 | -1.86(-1.9 to -1.82) |
| global | Deaths | 80 ~ 85 | -1.47(-1.51 to -1.43) |
| global | Deaths | 85 ~ 90 | -1.03(-1.08 to -0.97) |
| global | Deaths | 90 ~ 95 | -0.56(-0.66 to -0.46) |
| global | Deaths | 20 ~ 25 | -2.36(-3.07 to -1.65) |
| global | Deaths | 25 ~ 30 | -2.44(-2.91 to -1.96) |
| global | Deaths | 30 ~ 35 | -2.46(-2.81 to -2.12) |
| global | Deaths | 35 ~ 40 | -2.45(-2.71 to -2.19) |
| global | Deaths | 40 ~ 45 | -2.42(-2.61 to -2.22) |
| global | Deaths | 45 ~ 50 | -2.46(-2.6 to -2.32) |
| global | Deaths | 50 ~ 55 | -2.46(-2.57 to -2.36) |
| global | Deaths | 55 ~ 60 | -2.45(-2.53 to -2.38) |
| global | Deaths | 60 ~ 65 | -2.42(-2.48 to -2.36) |
| global | Deaths | 65 ~ 70 | -2.26(-2.3 to -2.21) |
| global | Deaths | 70 ~ 75 | -2.09(-2.13 to -2.06) |
| global | Deaths | 75 ~ 80 | -1.86(-1.9 to -1.82) |
| global | Deaths | 80 ~ 85 | -1.47(-1.51 to -1.43) |
| global | Deaths | 85 ~ 90 | -1.03(-1.08 to -0.97) |
| global | Deaths | 90 ~ 95 | -0.56(-0.66 to -0.46) |
| global | DALYs | 20 ~ 25 | -1.26(-1.55 to -0.97) |
| global | DALYs | 25 ~ 30 | -1.35(-1.55 to -1.15) |
| global | DALYs | 30 ~ 35 | -1.44(-1.6 to -1.28) |
| global | DALYs | 35 ~ 40 | -1.56(-1.69 to -1.43) |
| global | DALYs | 40 ~ 45 | -1.68(-1.79 to -1.57) |
| global | DALYs | 45 ~ 50 | -1.85(-1.93 to -1.76) |
| global | DALYs | 50 ~ 55 | -1.95(-2.02 to -1.88) |
| global | DALYs | 55 ~ 60 | -2.03(-2.09 to -1.97) |
| global | DALYs | 60 ~ 65 | -2.05(-2.1 to -2) |
| global | DALYs | 65 ~ 70 | -1.94(-1.98 to -1.89) |
| global | DALYs | 70 ~ 75 | -1.82(-1.86 to -1.78) |
| global | DALYs | 75 ~ 80 | -1.63(-1.67 to -1.58) |
| global | DALYs | 80 ~ 85 | -1.29(-1.34 to -1.23) |
| global | DALYs | 85 ~ 90 | -0.89(-0.97 to -0.81) |
| global | DALYs | 90 ~ 95 | -0.46(-0.62 to -0.3) |
| global | DALYs | 20 ~ 25 | -1.26(-1.55 to -0.97) |
| global | DALYs | 25 ~ 30 | -1.35(-1.55 to -1.15) |
| global | DALYs | 30 ~ 35 | -1.44(-1.6 to -1.28) |
| global | DALYs | 35 ~ 40 | -1.56(-1.69 to -1.43) |
| global | DALYs | 40 ~ 45 | -1.68(-1.79 to -1.57) |
| global | DALYs | 45 ~ 50 | -1.85(-1.93 to -1.76) |
| global | DALYs | 50 ~ 55 | -1.95(-2.02 to -1.88) |
| global | DALYs | 55 ~ 60 | -2.03(-2.09 to -1.97) |
| global | DALYs | 60 ~ 65 | -2.05(-2.1 to -2) |
| global | DALYs | 65 ~ 70 | -1.94(-1.98 to -1.89) |
| global | DALYs | 70 ~ 75 | -1.82(-1.86 to -1.78) |
| global | DALYs | 75 ~ 80 | -1.63(-1.67 to -1.58) |
| global | DALYs | 80 ~ 85 | -1.29(-1.34 to -1.23) |
| global | DALYs | 85 ~ 90 | -0.89(-0.97 to -0.81) |
| global | DALYs | 90 ~ 95 | -0.46(-0.62 to -0.3) |
| global | DALYs | 20 ~ 25 | -1.26(-1.55 to -0.97) |
| global | DALYs | 25 ~ 30 | -1.35(-1.55 to -1.15) |
| global | DALYs | 30 ~ 35 | -1.44(-1.6 to -1.28) |
| global | DALYs | 35 ~ 40 | -1.56(-1.69 to -1.43) |
| global | DALYs | 40 ~ 45 | -1.68(-1.79 to -1.57) |
| global | DALYs | 45 ~ 50 | -1.85(-1.93 to -1.76) |
| global | DALYs | 50 ~ 55 | -1.95(-2.02 to -1.88) |
| global | DALYs | 55 ~ 60 | -2.03(-2.09 to -1.97) |
| global | DALYs | 60 ~ 65 | -2.05(-2.1 to -2) |
| global | DALYs | 65 ~ 70 | -1.94(-1.98 to -1.89) |
| global | DALYs | 70 ~ 75 | -1.82(-1.86 to -1.78) |
| global | DALYs | 75 ~ 80 | -1.63(-1.67 to -1.58) |
| global | DALYs | 80 ~ 85 | -1.29(-1.34 to -1.23) |
| global | DALYs | 85 ~ 90 | -0.89(-0.97 to -0.81) |
| global | DALYs | 90 ~ 95 | -0.46(-0.62 to -0.3) |
| global | DALYs | 20 ~ 25 | -1.26(-1.55 to -0.97) |
| global | DALYs | 25 ~ 30 | -1.35(-1.55 to -1.15) |
| global | DALYs | 30 ~ 35 | -1.44(-1.6 to -1.28) |
| global | DALYs | 35 ~ 40 | -1.56(-1.69 to -1.43) |
| global | DALYs | 40 ~ 45 | -1.68(-1.79 to -1.57) |
| global | DALYs | 45 ~ 50 | -1.85(-1.93 to -1.76) |
| global | DALYs | 50 ~ 55 | -1.95(-2.02 to -1.88) |
| global | DALYs | 55 ~ 60 | -2.03(-2.09 to -1.97) |
| global | DALYs | 60 ~ 65 | -2.05(-2.1 to -2) |
| global | DALYs | 65 ~ 70 | -1.94(-1.98 to -1.89) |
| global | DALYs | 70 ~ 75 | -1.82(-1.86 to -1.78) |
| global | DALYs | 75 ~ 80 | -1.63(-1.67 to -1.58) |
| global | DALYs | 80 ~ 85 | -1.29(-1.34 to -1.23) |
| global | DALYs | 85 ~ 90 | -0.89(-0.97 to -0.81) |
| global | DALYs | 90 ~ 95 | -0.46(-0.62 to -0.3) |
| high_sdi | Prevalence | 20 ~ 25 | -0.62(-0.76 to -0.48) |
| high_sdi | Prevalence | 25 ~ 30 | -0.65(-0.75 to -0.56) |
| high_sdi | Prevalence | 30 ~ 35 | -0.64(-0.71 to -0.57) |
| high_sdi | Prevalence | 35 ~ 40 | -0.6(-0.66 to -0.54) |
| high_sdi | Prevalence | 40 ~ 45 | -0.49(-0.54 to -0.44) |
| high_sdi | Prevalence | 45 ~ 50 | -0.37(-0.41 to -0.33) |
| high_sdi | Prevalence | 50 ~ 55 | -0.2(-0.23 to -0.16) |
| high_sdi | Prevalence | 55 ~ 60 | -0.05(-0.08 to -0.02) |
| high_sdi | Prevalence | 60 ~ 65 | 0.02(0 to 0.05) |
| high_sdi | Prevalence | 65 ~ 70 | 0.01(-0.01 to 0.04) |
| high_sdi | Prevalence | 70 ~ 75 | -0.03(-0.05 to -0.01) |
| high_sdi | Prevalence | 75 ~ 80 | -0.02(-0.04 to 0.01) |
| high_sdi | Prevalence | 80 ~ 85 | 0.02(0 to 0.05) |
| high_sdi | Prevalence | 85 ~ 90 | 0.1(0.07 to 0.14) |
| high_sdi | Prevalence | 90 ~ 95 | 0.22(0.16 to 0.28) |
| high_sdi | Prevalence | 20 ~ 25 | -0.62(-0.76 to -0.48) |
| high_sdi | Prevalence | 25 ~ 30 | -0.65(-0.75 to -0.56) |
| high_sdi | Prevalence | 30 ~ 35 | -0.64(-0.71 to -0.57) |
| high_sdi | Prevalence | 35 ~ 40 | -0.6(-0.66 to -0.54) |
| high_sdi | Prevalence | 40 ~ 45 | -0.49(-0.54 to -0.44) |
| high_sdi | Prevalence | 45 ~ 50 | -0.37(-0.41 to -0.33) |
| high_sdi | Prevalence | 50 ~ 55 | -0.2(-0.23 to -0.16) |
| high_sdi | Prevalence | 55 ~ 60 | -0.05(-0.08 to -0.02) |
| high_sdi | Prevalence | 60 ~ 65 | 0.02(0 to 0.05) |
| high_sdi | Prevalence | 65 ~ 70 | 0.01(-0.01 to 0.04) |
| high_sdi | Prevalence | 70 ~ 75 | -0.03(-0.05 to -0.01) |
| high_sdi | Prevalence | 75 ~ 80 | -0.02(-0.04 to 0.01) |
| high_sdi | Prevalence | 80 ~ 85 | 0.02(0 to 0.05) |
| high_sdi | Prevalence | 85 ~ 90 | 0.1(0.07 to 0.14) |
| high_sdi | Prevalence | 90 ~ 95 | 0.22(0.16 to 0.28) |
| high_sdi | Prevalence | 20 ~ 25 | -0.62(-0.76 to -0.48) |
| high_sdi | Prevalence | 25 ~ 30 | -0.65(-0.75 to -0.56) |
| high_sdi | Prevalence | 30 ~ 35 | -0.64(-0.71 to -0.57) |
| high_sdi | Prevalence | 35 ~ 40 | -0.6(-0.66 to -0.54) |
| high_sdi | Prevalence | 40 ~ 45 | -0.49(-0.54 to -0.44) |
| high_sdi | Prevalence | 45 ~ 50 | -0.37(-0.41 to -0.33) |
| high_sdi | Prevalence | 50 ~ 55 | -0.2(-0.23 to -0.16) |
| high_sdi | Prevalence | 55 ~ 60 | -0.05(-0.08 to -0.02) |
| high_sdi | Prevalence | 60 ~ 65 | 0.02(0 to 0.05) |
| high_sdi | Prevalence | 65 ~ 70 | 0.01(-0.01 to 0.04) |
| high_sdi | Prevalence | 70 ~ 75 | -0.03(-0.05 to -0.01) |
| high_sdi | Prevalence | 75 ~ 80 | -0.02(-0.04 to 0.01) |
| high_sdi | Prevalence | 80 ~ 85 | 0.02(0 to 0.05) |
| high_sdi | Prevalence | 85 ~ 90 | 0.1(0.07 to 0.14) |
| high_sdi | Prevalence | 90 ~ 95 | 0.22(0.16 to 0.28) |
| high_sdi | Prevalence | 20 ~ 25 | -0.62(-0.76 to -0.48) |
| high_sdi | Prevalence | 25 ~ 30 | -0.65(-0.75 to -0.56) |
| high_sdi | Prevalence | 30 ~ 35 | -0.64(-0.71 to -0.57) |
| high_sdi | Prevalence | 35 ~ 40 | -0.6(-0.66 to -0.54) |
| high_sdi | Prevalence | 40 ~ 45 | -0.49(-0.54 to -0.44) |
| high_sdi | Prevalence | 45 ~ 50 | -0.37(-0.41 to -0.33) |
| high_sdi | Prevalence | 50 ~ 55 | -0.2(-0.23 to -0.16) |
| high_sdi | Prevalence | 55 ~ 60 | -0.05(-0.08 to -0.02) |
| high_sdi | Prevalence | 60 ~ 65 | 0.02(0 to 0.05) |
| high_sdi | Prevalence | 65 ~ 70 | 0.01(-0.01 to 0.04) |
| high_sdi | Prevalence | 70 ~ 75 | -0.03(-0.05 to -0.01) |
| high_sdi | Prevalence | 75 ~ 80 | -0.02(-0.04 to 0.01) |
| high_sdi | Prevalence | 80 ~ 85 | 0.02(0 to 0.05) |
| high_sdi | Prevalence | 85 ~ 90 | 0.1(0.07 to 0.14) |
| high_sdi | Prevalence | 90 ~ 95 | 0.22(0.16 to 0.28) |
| high_sdi | Deaths | 20 ~ 25 | -0.47(-2.29 to 1.39) |
| high_sdi | Deaths | 25 ~ 30 | -0.5(-1.68 to 0.7) |
| high_sdi | Deaths | 30 ~ 35 | -0.65(-1.48 to 0.18) |
| high_sdi | Deaths | 35 ~ 40 | -0.8(-1.38 to -0.2) |
| high_sdi | Deaths | 40 ~ 45 | -0.82(-1.23 to -0.41) |
| high_sdi | Deaths | 45 ~ 50 | -0.74(-1.01 to -0.47) |
| high_sdi | Deaths | 50 ~ 55 | -0.53(-0.7 to -0.35) |
| high_sdi | Deaths | 55 ~ 60 | -0.51(-0.63 to -0.39) |
| high_sdi | Deaths | 60 ~ 65 | -0.84(-0.93 to -0.76) |
| high_sdi | Deaths | 65 ~ 70 | -1.25(-1.32 to -1.18) |
| high_sdi | Deaths | 70 ~ 75 | -1.48(-1.54 to -1.43) |
| high_sdi | Deaths | 75 ~ 80 | -1.44(-1.49 to -1.39) |
| high_sdi | Deaths | 80 ~ 85 | -1.13(-1.18 to -1.08) |
| high_sdi | Deaths | 85 ~ 90 | -0.56(-0.63 to -0.5) |
| high_sdi | Deaths | 90 ~ 95 | 0.06(-0.04 to 0.17) |
| high_sdi | Deaths | 20 ~ 25 | -0.47(-2.29 to 1.39) |
| high_sdi | Deaths | 25 ~ 30 | -0.5(-1.68 to 0.7) |
| high_sdi | Deaths | 30 ~ 35 | -0.65(-1.48 to 0.18) |
| high_sdi | Deaths | 35 ~ 40 | -0.8(-1.38 to -0.2) |
| high_sdi | Deaths | 40 ~ 45 | -0.82(-1.23 to -0.41) |
| high_sdi | Deaths | 45 ~ 50 | -0.74(-1.01 to -0.47) |
| high_sdi | Deaths | 50 ~ 55 | -0.53(-0.7 to -0.35) |
| high_sdi | Deaths | 55 ~ 60 | -0.51(-0.63 to -0.39) |
| high_sdi | Deaths | 60 ~ 65 | -0.84(-0.93 to -0.76) |
| high_sdi | Deaths | 65 ~ 70 | -1.25(-1.32 to -1.18) |
| high_sdi | Deaths | 70 ~ 75 | -1.48(-1.54 to -1.43) |
| high_sdi | Deaths | 75 ~ 80 | -1.44(-1.49 to -1.39) |
| high_sdi | Deaths | 80 ~ 85 | -1.13(-1.18 to -1.08) |
| high_sdi | Deaths | 85 ~ 90 | -0.56(-0.63 to -0.5) |
| high_sdi | Deaths | 90 ~ 95 | 0.06(-0.04 to 0.17) |
| high_sdi | Deaths | 20 ~ 25 | -0.47(-2.29 to 1.39) |
| high_sdi | Deaths | 25 ~ 30 | -0.5(-1.68 to 0.7) |
| high_sdi | Deaths | 30 ~ 35 | -0.65(-1.48 to 0.18) |
| high_sdi | Deaths | 35 ~ 40 | -0.8(-1.38 to -0.2) |
| high_sdi | Deaths | 40 ~ 45 | -0.82(-1.23 to -0.41) |
| high_sdi | Deaths | 45 ~ 50 | -0.74(-1.01 to -0.47) |
| high_sdi | Deaths | 50 ~ 55 | -0.53(-0.7 to -0.35) |
| high_sdi | Deaths | 55 ~ 60 | -0.51(-0.63 to -0.39) |
| high_sdi | Deaths | 60 ~ 65 | -0.84(-0.93 to -0.76) |
| high_sdi | Deaths | 65 ~ 70 | -1.25(-1.32 to -1.18) |
| high_sdi | Deaths | 70 ~ 75 | -1.48(-1.54 to -1.43) |
| high_sdi | Deaths | 75 ~ 80 | -1.44(-1.49 to -1.39) |
| high_sdi | Deaths | 80 ~ 85 | -1.13(-1.18 to -1.08) |
| high_sdi | Deaths | 85 ~ 90 | -0.56(-0.63 to -0.5) |
| high_sdi | Deaths | 90 ~ 95 | 0.06(-0.04 to 0.17) |
| high_sdi | Deaths | 20 ~ 25 | -0.47(-2.29 to 1.39) |
| high_sdi | Deaths | 25 ~ 30 | -0.5(-1.68 to 0.7) |
| high_sdi | Deaths | 30 ~ 35 | -0.65(-1.48 to 0.18) |
| high_sdi | Deaths | 35 ~ 40 | -0.8(-1.38 to -0.2) |
| high_sdi | Deaths | 40 ~ 45 | -0.82(-1.23 to -0.41) |
| high_sdi | Deaths | 45 ~ 50 | -0.74(-1.01 to -0.47) |
| high_sdi | Deaths | 50 ~ 55 | -0.53(-0.7 to -0.35) |
| high_sdi | Deaths | 55 ~ 60 | -0.51(-0.63 to -0.39) |
| high_sdi | Deaths | 60 ~ 65 | -0.84(-0.93 to -0.76) |
| high_sdi | Deaths | 65 ~ 70 | -1.25(-1.32 to -1.18) |
| high_sdi | Deaths | 70 ~ 75 | -1.48(-1.54 to -1.43) |
| high_sdi | Deaths | 75 ~ 80 | -1.44(-1.49 to -1.39) |
| high_sdi | Deaths | 80 ~ 85 | -1.13(-1.18 to -1.08) |
| high_sdi | Deaths | 85 ~ 90 | -0.56(-0.63 to -0.5) |
| high_sdi | Deaths | 90 ~ 95 | 0.06(-0.04 to 0.17) |
| high_sdi | DALYs | 20 ~ 25 | -0.64(-1.19 to -0.08) |
| high_sdi | DALYs | 25 ~ 30 | -0.64(-1.02 to -0.27) |
| high_sdi | DALYs | 30 ~ 35 | -0.68(-0.96 to -0.4) |
| high_sdi | DALYs | 35 ~ 40 | -0.69(-0.91 to -0.47) |
| high_sdi | DALYs | 40 ~ 45 | -0.61(-0.78 to -0.44) |
| high_sdi | DALYs | 45 ~ 50 | -0.51(-0.65 to -0.38) |
| high_sdi | DALYs | 50 ~ 55 | -0.34(-0.44 to -0.24) |
| high_sdi | DALYs | 55 ~ 60 | -0.3(-0.38 to -0.22) |
| high_sdi | DALYs | 60 ~ 65 | -0.54(-0.6 to -0.47) |
| high_sdi | DALYs | 65 ~ 70 | -0.87(-0.93 to -0.82) |
| high_sdi | DALYs | 70 ~ 75 | -1.12(-1.17 to -1.07) |
| high_sdi | DALYs | 75 ~ 80 | -1.12(-1.17 to -1.07) |
| high_sdi | DALYs | 80 ~ 85 | -0.9(-0.96 to -0.84) |
| high_sdi | DALYs | 85 ~ 90 | -0.44(-0.53 to -0.36) |
| high_sdi | DALYs | 90 ~ 95 | 0.08(-0.07 to 0.22) |
| high_sdi | DALYs | 20 ~ 25 | -0.64(-1.19 to -0.08) |
| high_sdi | DALYs | 25 ~ 30 | -0.64(-1.02 to -0.27) |
| high_sdi | DALYs | 30 ~ 35 | -0.68(-0.96 to -0.4) |
| high_sdi | DALYs | 35 ~ 40 | -0.69(-0.91 to -0.47) |
| high_sdi | DALYs | 40 ~ 45 | -0.61(-0.78 to -0.44) |
| high_sdi | DALYs | 45 ~ 50 | -0.51(-0.65 to -0.38) |
| high_sdi | DALYs | 50 ~ 55 | -0.34(-0.44 to -0.24) |
| high_sdi | DALYs | 55 ~ 60 | -0.3(-0.38 to -0.22) |
| high_sdi | DALYs | 60 ~ 65 | -0.54(-0.6 to -0.47) |
| high_sdi | DALYs | 65 ~ 70 | -0.87(-0.93 to -0.82) |
| high_sdi | DALYs | 70 ~ 75 | -1.12(-1.17 to -1.07) |
| high_sdi | DALYs | 75 ~ 80 | -1.12(-1.17 to -1.07) |
| high_sdi | DALYs | 80 ~ 85 | -0.9(-0.96 to -0.84) |
| high_sdi | DALYs | 85 ~ 90 | -0.44(-0.53 to -0.36) |
| high_sdi | DALYs | 90 ~ 95 | 0.08(-0.07 to 0.22) |
| high_sdi | DALYs | 20 ~ 25 | -0.64(-1.19 to -0.08) |
| high_sdi | DALYs | 25 ~ 30 | -0.64(-1.02 to -0.27) |
| high_sdi | DALYs | 30 ~ 35 | -0.68(-0.96 to -0.4) |
| high_sdi | DALYs | 35 ~ 40 | -0.69(-0.91 to -0.47) |
| high_sdi | DALYs | 40 ~ 45 | -0.61(-0.78 to -0.44) |
| high_sdi | DALYs | 45 ~ 50 | -0.51(-0.65 to -0.38) |
| high_sdi | DALYs | 50 ~ 55 | -0.34(-0.44 to -0.24) |
| high_sdi | DALYs | 55 ~ 60 | -0.3(-0.38 to -0.22) |
| high_sdi | DALYs | 60 ~ 65 | -0.54(-0.6 to -0.47) |
| high_sdi | DALYs | 65 ~ 70 | -0.87(-0.93 to -0.82) |
| high_sdi | DALYs | 70 ~ 75 | -1.12(-1.17 to -1.07) |
| high_sdi | DALYs | 75 ~ 80 | -1.12(-1.17 to -1.07) |
| high_sdi | DALYs | 80 ~ 85 | -0.9(-0.96 to -0.84) |
| high_sdi | DALYs | 85 ~ 90 | -0.44(-0.53 to -0.36) |
| high_sdi | DALYs | 90 ~ 95 | 0.08(-0.07 to 0.22) |
| high_sdi | DALYs | 20 ~ 25 | -0.64(-1.19 to -0.08) |
| high_sdi | DALYs | 25 ~ 30 | -0.64(-1.02 to -0.27) |
| high_sdi | DALYs | 30 ~ 35 | -0.68(-0.96 to -0.4) |
| high_sdi | DALYs | 35 ~ 40 | -0.69(-0.91 to -0.47) |
| high_sdi | DALYs | 40 ~ 45 | -0.61(-0.78 to -0.44) |
| high_sdi | DALYs | 45 ~ 50 | -0.51(-0.65 to -0.38) |
| high_sdi | DALYs | 50 ~ 55 | -0.34(-0.44 to -0.24) |
| high_sdi | DALYs | 55 ~ 60 | -0.3(-0.38 to -0.22) |
| high_sdi | DALYs | 60 ~ 65 | -0.54(-0.6 to -0.47) |
| high_sdi | DALYs | 65 ~ 70 | -0.87(-0.93 to -0.82) |
| high_sdi | DALYs | 70 ~ 75 | -1.12(-1.17 to -1.07) |
| high_sdi | DALYs | 75 ~ 80 | -1.12(-1.17 to -1.07) |
| high_sdi | DALYs | 80 ~ 85 | -0.9(-0.96 to -0.84) |
| high_sdi | DALYs | 85 ~ 90 | -0.44(-0.53 to -0.36) |
| high_sdi | DALYs | 90 ~ 95 | 0.08(-0.07 to 0.22) |
| high-middle_sdi | Prevalence | 20 ~ 25 | -0.5(-0.71 to -0.29) |
| high-middle_sdi | Prevalence | 25 ~ 30 | -0.51(-0.64 to -0.37) |
| high-middle_sdi | Prevalence | 30 ~ 35 | -0.51(-0.62 to -0.41) |
| high-middle_sdi | Prevalence | 35 ~ 40 | -0.53(-0.62 to -0.44) |
| high-middle_sdi | Prevalence | 40 ~ 45 | -0.58(-0.65 to -0.5) |
| high-middle_sdi | Prevalence | 45 ~ 50 | -0.64(-0.71 to -0.57) |
| high-middle_sdi | Prevalence | 50 ~ 55 | -0.62(-0.68 to -0.57) |
| high-middle_sdi | Prevalence | 55 ~ 60 | -0.55(-0.6 to -0.5) |
| high-middle_sdi | Prevalence | 60 ~ 65 | -0.44(-0.48 to -0.39) |
| high-middle_sdi | Prevalence | 65 ~ 70 | -0.3(-0.34 to -0.25) |
| high-middle_sdi | Prevalence | 70 ~ 75 | -0.17(-0.22 to -0.13) |
| high-middle_sdi | Prevalence | 75 ~ 80 | -0.02(-0.07 to 0.03) |
| high-middle_sdi | Prevalence | 80 ~ 85 | 0.12(0.06 to 0.18) |
| high-middle_sdi | Prevalence | 85 ~ 90 | 0.3(0.21 to 0.39) |
| high-middle_sdi | Prevalence | 90 ~ 95 | 0.45(0.28 to 0.63) |
| high-middle_sdi | Prevalence | 20 ~ 25 | -0.5(-0.71 to -0.29) |
| high-middle_sdi | Prevalence | 25 ~ 30 | -0.51(-0.64 to -0.37) |
| high-middle_sdi | Prevalence | 30 ~ 35 | -0.51(-0.62 to -0.41) |
| high-middle_sdi | Prevalence | 35 ~ 40 | -0.53(-0.62 to -0.44) |
| high-middle_sdi | Prevalence | 40 ~ 45 | -0.58(-0.65 to -0.5) |
| high-middle_sdi | Prevalence | 45 ~ 50 | -0.64(-0.71 to -0.57) |
| high-middle_sdi | Prevalence | 50 ~ 55 | -0.62(-0.68 to -0.57) |
| high-middle_sdi | Prevalence | 55 ~ 60 | -0.55(-0.6 to -0.5) |
| high-middle_sdi | Prevalence | 60 ~ 65 | -0.44(-0.48 to -0.39) |
| high-middle_sdi | Prevalence | 65 ~ 70 | -0.3(-0.34 to -0.25) |
| high-middle_sdi | Prevalence | 70 ~ 75 | -0.17(-0.22 to -0.13) |
| high-middle_sdi | Prevalence | 75 ~ 80 | -0.02(-0.07 to 0.03) |
| high-middle_sdi | Prevalence | 80 ~ 85 | 0.12(0.06 to 0.18) |
| high-middle_sdi | Prevalence | 85 ~ 90 | 0.3(0.21 to 0.39) |
| high-middle_sdi | Prevalence | 90 ~ 95 | 0.45(0.28 to 0.63) |
| high-middle_sdi | Prevalence | 20 ~ 25 | -0.5(-0.71 to -0.29) |
| high-middle_sdi | Prevalence | 25 ~ 30 | -0.51(-0.64 to -0.37) |
| high-middle_sdi | Prevalence | 30 ~ 35 | -0.51(-0.62 to -0.41) |
| high-middle_sdi | Prevalence | 35 ~ 40 | -0.53(-0.62 to -0.44) |
| high-middle_sdi | Prevalence | 40 ~ 45 | -0.58(-0.65 to -0.5) |
| high-middle_sdi | Prevalence | 45 ~ 50 | -0.64(-0.71 to -0.57) |
| high-middle_sdi | Prevalence | 50 ~ 55 | -0.62(-0.68 to -0.57) |
| high-middle_sdi | Prevalence | 55 ~ 60 | -0.55(-0.6 to -0.5) |
| high-middle_sdi | Prevalence | 60 ~ 65 | -0.44(-0.48 to -0.39) |
| high-middle_sdi | Prevalence | 65 ~ 70 | -0.3(-0.34 to -0.25) |
| high-middle_sdi | Prevalence | 70 ~ 75 | -0.17(-0.22 to -0.13) |
| high-middle_sdi | Prevalence | 75 ~ 80 | -0.02(-0.07 to 0.03) |
| high-middle_sdi | Prevalence | 80 ~ 85 | 0.12(0.06 to 0.18) |
| high-middle_sdi | Prevalence | 85 ~ 90 | 0.3(0.21 to 0.39) |
| high-middle_sdi | Prevalence | 90 ~ 95 | 0.45(0.28 to 0.63) |
| high-middle_sdi | Prevalence | 20 ~ 25 | -0.5(-0.71 to -0.29) |
| high-middle_sdi | Prevalence | 25 ~ 30 | -0.51(-0.64 to -0.37) |
| high-middle_sdi | Prevalence | 30 ~ 35 | -0.51(-0.62 to -0.41) |
| high-middle_sdi | Prevalence | 35 ~ 40 | -0.53(-0.62 to -0.44) |
| high-middle_sdi | Prevalence | 40 ~ 45 | -0.58(-0.65 to -0.5) |
| high-middle_sdi | Prevalence | 45 ~ 50 | -0.64(-0.71 to -0.57) |
| high-middle_sdi | Prevalence | 50 ~ 55 | -0.62(-0.68 to -0.57) |
| high-middle_sdi | Prevalence | 55 ~ 60 | -0.55(-0.6 to -0.5) |
| high-middle_sdi | Prevalence | 60 ~ 65 | -0.44(-0.48 to -0.39) |
| high-middle_sdi | Prevalence | 65 ~ 70 | -0.3(-0.34 to -0.25) |
| high-middle_sdi | Prevalence | 70 ~ 75 | -0.17(-0.22 to -0.13) |
| high-middle_sdi | Prevalence | 75 ~ 80 | -0.02(-0.07 to 0.03) |
| high-middle_sdi | Prevalence | 80 ~ 85 | 0.12(0.06 to 0.18) |
| high-middle_sdi | Prevalence | 85 ~ 90 | 0.3(0.21 to 0.39) |
| high-middle_sdi | Prevalence | 90 ~ 95 | 0.45(0.28 to 0.63) |
| high-middle_sdi | Deaths | 20 ~ 25 | -3.5(-4.88 to -2.1) |
| high-middle_sdi | Deaths | 25 ~ 30 | -3.69(-4.58 to -2.8) |
| high-middle_sdi | Deaths | 30 ~ 35 | -3.91(-4.51 to -3.31) |
| high-middle_sdi | Deaths | 35 ~ 40 | -4.13(-4.57 to -3.69) |
| high-middle_sdi | Deaths | 40 ~ 45 | -4.34(-4.66 to -4.02) |
| high-middle_sdi | Deaths | 45 ~ 50 | -4.5(-4.73 to -4.27) |
| high-middle_sdi | Deaths | 50 ~ 55 | -4.46(-4.62 to -4.3) |
| high-middle_sdi | Deaths | 55 ~ 60 | -4.4(-4.52 to -4.28) |
| high-middle_sdi | Deaths | 60 ~ 65 | -4.23(-4.32 to -4.15) |
| high-middle_sdi | Deaths | 65 ~ 70 | -3.95(-4.01 to -3.88) |
| high-middle_sdi | Deaths | 70 ~ 75 | -3.8(-3.86 to -3.75) |
| high-middle_sdi | Deaths | 75 ~ 80 | -3.5(-3.55 to -3.45) |
| high-middle_sdi | Deaths | 80 ~ 85 | -2.92(-2.97 to -2.87) |
| high-middle_sdi | Deaths | 85 ~ 90 | -2.22(-2.29 to -2.16) |
| high-middle_sdi | Deaths | 90 ~ 95 | -1.61(-1.72 to -1.49) |
| high-middle_sdi | Deaths | 20 ~ 25 | -3.5(-4.88 to -2.1) |
| high-middle_sdi | Deaths | 25 ~ 30 | -3.69(-4.58 to -2.8) |
| high-middle_sdi | Deaths | 30 ~ 35 | -3.91(-4.51 to -3.31) |
| high-middle_sdi | Deaths | 35 ~ 40 | -4.13(-4.57 to -3.69) |
| high-middle_sdi | Deaths | 40 ~ 45 | -4.34(-4.66 to -4.02) |
| high-middle_sdi | Deaths | 45 ~ 50 | -4.5(-4.73 to -4.27) |
| high-middle_sdi | Deaths | 50 ~ 55 | -4.46(-4.62 to -4.3) |
| high-middle_sdi | Deaths | 55 ~ 60 | -4.4(-4.52 to -4.28) |
| high-middle_sdi | Deaths | 60 ~ 65 | -4.23(-4.32 to -4.15) |
| high-middle_sdi | Deaths | 65 ~ 70 | -3.95(-4.01 to -3.88) |
| high-middle_sdi | Deaths | 70 ~ 75 | -3.8(-3.86 to -3.75) |
| high-middle_sdi | Deaths | 75 ~ 80 | -3.5(-3.55 to -3.45) |
| high-middle_sdi | Deaths | 80 ~ 85 | -2.92(-2.97 to -2.87) |
| high-middle_sdi | Deaths | 85 ~ 90 | -2.22(-2.29 to -2.16) |
| high-middle_sdi | Deaths | 90 ~ 95 | -1.61(-1.72 to -1.49) |
| high-middle_sdi | Deaths | 20 ~ 25 | -3.5(-4.88 to -2.1) |
| high-middle_sdi | Deaths | 25 ~ 30 | -3.69(-4.58 to -2.8) |
| high-middle_sdi | Deaths | 30 ~ 35 | -3.91(-4.51 to -3.31) |
| high-middle_sdi | Deaths | 35 ~ 40 | -4.13(-4.57 to -3.69) |
| high-middle_sdi | Deaths | 40 ~ 45 | -4.34(-4.66 to -4.02) |
| high-middle_sdi | Deaths | 45 ~ 50 | -4.5(-4.73 to -4.27) |
| high-middle_sdi | Deaths | 50 ~ 55 | -4.46(-4.62 to -4.3) |
| high-middle_sdi | Deaths | 55 ~ 60 | -4.4(-4.52 to -4.28) |
| high-middle_sdi | Deaths | 60 ~ 65 | -4.23(-4.32 to -4.15) |
| high-middle_sdi | Deaths | 65 ~ 70 | -3.95(-4.01 to -3.88) |
| high-middle_sdi | Deaths | 70 ~ 75 | -3.8(-3.86 to -3.75) |
| high-middle_sdi | Deaths | 75 ~ 80 | -3.5(-3.55 to -3.45) |
| high-middle_sdi | Deaths | 80 ~ 85 | -2.92(-2.97 to -2.87) |
| high-middle_sdi | Deaths | 85 ~ 90 | -2.22(-2.29 to -2.16) |
| high-middle_sdi | Deaths | 90 ~ 95 | -1.61(-1.72 to -1.49) |
| high-middle_sdi | Deaths | 20 ~ 25 | -3.5(-4.88 to -2.1) |
| high-middle_sdi | Deaths | 25 ~ 30 | -3.69(-4.58 to -2.8) |
| high-middle_sdi | Deaths | 30 ~ 35 | -3.91(-4.51 to -3.31) |
| high-middle_sdi | Deaths | 35 ~ 40 | -4.13(-4.57 to -3.69) |
| high-middle_sdi | Deaths | 40 ~ 45 | -4.34(-4.66 to -4.02) |
| high-middle_sdi | Deaths | 45 ~ 50 | -4.5(-4.73 to -4.27) |
| high-middle_sdi | Deaths | 50 ~ 55 | -4.46(-4.62 to -4.3) |
| high-middle_sdi | Deaths | 55 ~ 60 | -4.4(-4.52 to -4.28) |
| high-middle_sdi | Deaths | 60 ~ 65 | -4.23(-4.32 to -4.15) |
| high-middle_sdi | Deaths | 65 ~ 70 | -3.95(-4.01 to -3.88) |
| high-middle_sdi | Deaths | 70 ~ 75 | -3.8(-3.86 to -3.75) |
| high-middle_sdi | Deaths | 75 ~ 80 | -3.5(-3.55 to -3.45) |
| high-middle_sdi | Deaths | 80 ~ 85 | -2.92(-2.97 to -2.87) |
| high-middle_sdi | Deaths | 85 ~ 90 | -2.22(-2.29 to -2.16) |
| high-middle_sdi | Deaths | 90 ~ 95 | -1.61(-1.72 to -1.49) |
| high-middle_sdi | DALYs | 20 ~ 25 | -2(-2.57 to -1.44) |
| high-middle_sdi | DALYs | 25 ~ 30 | -2.1(-2.47 to -1.73) |
| high-middle_sdi | DALYs | 30 ~ 35 | -2.31(-2.58 to -2.04) |
| high-middle_sdi | DALYs | 35 ~ 40 | -2.65(-2.87 to -2.43) |
| high-middle_sdi | DALYs | 40 ~ 45 | -3(-3.18 to -2.83) |
| high-middle_sdi | DALYs | 45 ~ 50 | -3.28(-3.42 to -3.14) |
| high-middle_sdi | DALYs | 50 ~ 55 | -3.4(-3.5 to -3.29) |
| high-middle_sdi | DALYs | 55 ~ 60 | -3.5(-3.59 to -3.41) |
| high-middle_sdi | DALYs | 60 ~ 65 | -3.5(-3.57 to -3.42) |
| high-middle_sdi | DALYs | 65 ~ 70 | -3.34(-3.4 to -3.27) |
| high-middle_sdi | DALYs | 70 ~ 75 | -3.31(-3.37 to -3.25) |
| high-middle_sdi | DALYs | 75 ~ 80 | -3.1(-3.16 to -3.05) |
| high-middle_sdi | DALYs | 80 ~ 85 | -2.63(-2.69 to -2.56) |
| high-middle_sdi | DALYs | 85 ~ 90 | -2.01(-2.11 to -1.92) |
| high-middle_sdi | DALYs | 90 ~ 95 | -1.45(-1.63 to -1.26) |
| high-middle_sdi | DALYs | 20 ~ 25 | -2(-2.57 to -1.44) |
| high-middle_sdi | DALYs | 25 ~ 30 | -2.1(-2.47 to -1.73) |
| high-middle_sdi | DALYs | 30 ~ 35 | -2.31(-2.58 to -2.04) |
| high-middle_sdi | DALYs | 35 ~ 40 | -2.65(-2.87 to -2.43) |
| high-middle_sdi | DALYs | 40 ~ 45 | -3(-3.18 to -2.83) |
| high-middle_sdi | DALYs | 45 ~ 50 | -3.28(-3.42 to -3.14) |
| high-middle_sdi | DALYs | 50 ~ 55 | -3.4(-3.5 to -3.29) |
| high-middle_sdi | DALYs | 55 ~ 60 | -3.5(-3.59 to -3.41) |
| high-middle_sdi | DALYs | 60 ~ 65 | -3.5(-3.57 to -3.42) |
| high-middle_sdi | DALYs | 65 ~ 70 | -3.34(-3.4 to -3.27) |
| high-middle_sdi | DALYs | 70 ~ 75 | -3.31(-3.37 to -3.25) |
| high-middle_sdi | DALYs | 75 ~ 80 | -3.1(-3.16 to -3.05) |
| high-middle_sdi | DALYs | 80 ~ 85 | -2.63(-2.69 to -2.56) |
| high-middle_sdi | DALYs | 85 ~ 90 | -2.01(-2.11 to -1.92) |
| high-middle_sdi | DALYs | 90 ~ 95 | -1.45(-1.63 to -1.26) |
| high-middle_sdi | DALYs | 20 ~ 25 | -2(-2.57 to -1.44) |
| high-middle_sdi | DALYs | 25 ~ 30 | -2.1(-2.47 to -1.73) |
| high-middle_sdi | DALYs | 30 ~ 35 | -2.31(-2.58 to -2.04) |
| high-middle_sdi | DALYs | 35 ~ 40 | -2.65(-2.87 to -2.43) |
| high-middle_sdi | DALYs | 40 ~ 45 | -3(-3.18 to -2.83) |
| high-middle_sdi | DALYs | 45 ~ 50 | -3.28(-3.42 to -3.14) |
| high-middle_sdi | DALYs | 50 ~ 55 | -3.4(-3.5 to -3.29) |
| high-middle_sdi | DALYs | 55 ~ 60 | -3.5(-3.59 to -3.41) |
| high-middle_sdi | DALYs | 60 ~ 65 | -3.5(-3.57 to -3.42) |
| high-middle_sdi | DALYs | 65 ~ 70 | -3.34(-3.4 to -3.27) |
| high-middle_sdi | DALYs | 70 ~ 75 | -3.31(-3.37 to -3.25) |
| high-middle_sdi | DALYs | 75 ~ 80 | -3.1(-3.16 to -3.05) |
| high-middle_sdi | DALYs | 80 ~ 85 | -2.63(-2.69 to -2.56) |
| high-middle_sdi | DALYs | 85 ~ 90 | -2.01(-2.11 to -1.92) |
| high-middle_sdi | DALYs | 90 ~ 95 | -1.45(-1.63 to -1.26) |
| high-middle_sdi | DALYs | 20 ~ 25 | -2(-2.57 to -1.44) |
| high-middle_sdi | DALYs | 25 ~ 30 | -2.1(-2.47 to -1.73) |
| high-middle_sdi | DALYs | 30 ~ 35 | -2.31(-2.58 to -2.04) |
| high-middle_sdi | DALYs | 35 ~ 40 | -2.65(-2.87 to -2.43) |
| high-middle_sdi | DALYs | 40 ~ 45 | -3(-3.18 to -2.83) |
| high-middle_sdi | DALYs | 45 ~ 50 | -3.28(-3.42 to -3.14) |
| high-middle_sdi | DALYs | 50 ~ 55 | -3.4(-3.5 to -3.29) |
| high-middle_sdi | DALYs | 55 ~ 60 | -3.5(-3.59 to -3.41) |
| high-middle_sdi | DALYs | 60 ~ 65 | -3.5(-3.57 to -3.42) |
| high-middle_sdi | DALYs | 65 ~ 70 | -3.34(-3.4 to -3.27) |
| high-middle_sdi | DALYs | 70 ~ 75 | -3.31(-3.37 to -3.25) |
| high-middle_sdi | DALYs | 75 ~ 80 | -3.1(-3.16 to -3.05) |
| high-middle_sdi | DALYs | 80 ~ 85 | -2.63(-2.69 to -2.56) |
| high-middle_sdi | DALYs | 85 ~ 90 | -2.01(-2.11 to -1.92) |
| high-middle_sdi | DALYs | 90 ~ 95 | -1.45(-1.63 to -1.26) |
| middle_sdi | Prevalence | 20 ~ 25 | -0.46(-0.58 to -0.34) |
| middle_sdi | Prevalence | 25 ~ 30 | -0.46(-0.55 to -0.38) |
| middle_sdi | Prevalence | 30 ~ 35 | -0.45(-0.52 to -0.39) |
| middle_sdi | Prevalence | 35 ~ 40 | -0.46(-0.52 to -0.4) |
| middle_sdi | Prevalence | 40 ~ 45 | -0.48(-0.53 to -0.42) |
| middle_sdi | Prevalence | 45 ~ 50 | -0.49(-0.53 to -0.44) |
| middle_sdi | Prevalence | 50 ~ 55 | -0.46(-0.5 to -0.42) |
| middle_sdi | Prevalence | 55 ~ 60 | -0.39(-0.42 to -0.35) |
| middle_sdi | Prevalence | 60 ~ 65 | -0.26(-0.3 to -0.23) |
| middle_sdi | Prevalence | 65 ~ 70 | -0.11(-0.14 to -0.07) |
| middle_sdi | Prevalence | 70 ~ 75 | 0.06(0.02 to 0.09) |
| middle_sdi | Prevalence | 75 ~ 80 | 0.22(0.18 to 0.26) |
| middle_sdi | Prevalence | 80 ~ 85 | 0.4(0.35 to 0.46) |
| middle_sdi | Prevalence | 85 ~ 90 | 0.6(0.51 to 0.69) |
| middle_sdi | Prevalence | 90 ~ 95 | 0.82(0.64 to 1.01) |
| middle_sdi | Prevalence | 20 ~ 25 | -0.46(-0.58 to -0.34) |
| middle_sdi | Prevalence | 25 ~ 30 | -0.46(-0.55 to -0.38) |
| middle_sdi | Prevalence | 30 ~ 35 | -0.45(-0.52 to -0.39) |
| middle_sdi | Prevalence | 35 ~ 40 | -0.46(-0.52 to -0.4) |
| middle_sdi | Prevalence | 40 ~ 45 | -0.48(-0.53 to -0.42) |
| middle_sdi | Prevalence | 45 ~ 50 | -0.49(-0.53 to -0.44) |
| middle_sdi | Prevalence | 50 ~ 55 | -0.46(-0.5 to -0.42) |
| middle_sdi | Prevalence | 55 ~ 60 | -0.39(-0.42 to -0.35) |
| middle_sdi | Prevalence | 60 ~ 65 | -0.26(-0.3 to -0.23) |
| middle_sdi | Prevalence | 65 ~ 70 | -0.11(-0.14 to -0.07) |
| middle_sdi | Prevalence | 70 ~ 75 | 0.06(0.02 to 0.09) |
| middle_sdi | Prevalence | 75 ~ 80 | 0.22(0.18 to 0.26) |
| middle_sdi | Prevalence | 80 ~ 85 | 0.4(0.35 to 0.46) |
| middle_sdi | Prevalence | 85 ~ 90 | 0.6(0.51 to 0.69) |
| middle_sdi | Prevalence | 90 ~ 95 | 0.82(0.64 to 1.01) |
| middle_sdi | Prevalence | 20 ~ 25 | -0.46(-0.58 to -0.34) |
| middle_sdi | Prevalence | 25 ~ 30 | -0.46(-0.55 to -0.38) |
| middle_sdi | Prevalence | 30 ~ 35 | -0.45(-0.52 to -0.39) |
| middle_sdi | Prevalence | 35 ~ 40 | -0.46(-0.52 to -0.4) |
| middle_sdi | Prevalence | 40 ~ 45 | -0.48(-0.53 to -0.42) |
| middle_sdi | Prevalence | 45 ~ 50 | -0.49(-0.53 to -0.44) |
| middle_sdi | Prevalence | 50 ~ 55 | -0.46(-0.5 to -0.42) |
| middle_sdi | Prevalence | 55 ~ 60 | -0.39(-0.42 to -0.35) |
| middle_sdi | Prevalence | 60 ~ 65 | -0.26(-0.3 to -0.23) |
| middle_sdi | Prevalence | 65 ~ 70 | -0.11(-0.14 to -0.07) |
| middle_sdi | Prevalence | 70 ~ 75 | 0.06(0.02 to 0.09) |
| middle_sdi | Prevalence | 75 ~ 80 | 0.22(0.18 to 0.26) |
| middle_sdi | Prevalence | 80 ~ 85 | 0.4(0.35 to 0.46) |
| middle_sdi | Prevalence | 85 ~ 90 | 0.6(0.51 to 0.69) |
| middle_sdi | Prevalence | 90 ~ 95 | 0.82(0.64 to 1.01) |
| middle_sdi | Prevalence | 20 ~ 25 | -0.46(-0.58 to -0.34) |
| middle_sdi | Prevalence | 25 ~ 30 | -0.46(-0.55 to -0.38) |
| middle_sdi | Prevalence | 30 ~ 35 | -0.45(-0.52 to -0.39) |
| middle_sdi | Prevalence | 35 ~ 40 | -0.46(-0.52 to -0.4) |
| middle_sdi | Prevalence | 40 ~ 45 | -0.48(-0.53 to -0.42) |
| middle_sdi | Prevalence | 45 ~ 50 | -0.49(-0.53 to -0.44) |
| middle_sdi | Prevalence | 50 ~ 55 | -0.46(-0.5 to -0.42) |
| middle_sdi | Prevalence | 55 ~ 60 | -0.39(-0.42 to -0.35) |
| middle_sdi | Prevalence | 60 ~ 65 | -0.26(-0.3 to -0.23) |
| middle_sdi | Prevalence | 65 ~ 70 | -0.11(-0.14 to -0.07) |
| middle_sdi | Prevalence | 70 ~ 75 | 0.06(0.02 to 0.09) |
| middle_sdi | Prevalence | 75 ~ 80 | 0.22(0.18 to 0.26) |
| middle_sdi | Prevalence | 80 ~ 85 | 0.4(0.35 to 0.46) |
| middle_sdi | Prevalence | 85 ~ 90 | 0.6(0.51 to 0.69) |
| middle_sdi | Prevalence | 90 ~ 95 | 0.82(0.64 to 1.01) |
| middle_sdi | Deaths | 20 ~ 25 | -3.25(-4.15 to -2.34) |
| middle_sdi | Deaths | 25 ~ 30 | -3.4(-3.99 to -2.8) |
| middle_sdi | Deaths | 30 ~ 35 | -3.51(-3.94 to -3.09) |
| middle_sdi | Deaths | 35 ~ 40 | -3.63(-3.95 to -3.3) |
| middle_sdi | Deaths | 40 ~ 45 | -3.7(-3.94 to -3.46) |
| middle_sdi | Deaths | 45 ~ 50 | -3.74(-3.92 to -3.56) |
| middle_sdi | Deaths | 50 ~ 55 | -3.82(-3.95 to -3.7) |
| middle_sdi | Deaths | 55 ~ 60 | -3.85(-3.94 to -3.75) |
| middle_sdi | Deaths | 60 ~ 65 | -3.79(-3.87 to -3.72) |
| middle_sdi | Deaths | 65 ~ 70 | -3.55(-3.61 to -3.5) |
| middle_sdi | Deaths | 70 ~ 75 | -3.32(-3.37 to -3.27) |
| middle_sdi | Deaths | 75 ~ 80 | -3.04(-3.08 to -2.99) |
| middle_sdi | Deaths | 80 ~ 85 | -2.59(-2.64 to -2.54) |
| middle_sdi | Deaths | 85 ~ 90 | -2.04(-2.11 to -1.97) |
| middle_sdi | Deaths | 90 ~ 95 | -1.48(-1.61 to -1.35) |
| middle_sdi | Deaths | 20 ~ 25 | -3.25(-4.15 to -2.34) |
| middle_sdi | Deaths | 25 ~ 30 | -3.4(-3.99 to -2.8) |
| middle_sdi | Deaths | 30 ~ 35 | -3.51(-3.94 to -3.09) |
| middle_sdi | Deaths | 35 ~ 40 | -3.63(-3.95 to -3.3) |
| middle_sdi | Deaths | 40 ~ 45 | -3.7(-3.94 to -3.46) |
| middle_sdi | Deaths | 45 ~ 50 | -3.74(-3.92 to -3.56) |
| middle_sdi | Deaths | 50 ~ 55 | -3.82(-3.95 to -3.7) |
| middle_sdi | Deaths | 55 ~ 60 | -3.85(-3.94 to -3.75) |
| middle_sdi | Deaths | 60 ~ 65 | -3.79(-3.87 to -3.72) |
| middle_sdi | Deaths | 65 ~ 70 | -3.55(-3.61 to -3.5) |
| middle_sdi | Deaths | 70 ~ 75 | -3.32(-3.37 to -3.27) |
| middle_sdi | Deaths | 75 ~ 80 | -3.04(-3.08 to -2.99) |
| middle_sdi | Deaths | 80 ~ 85 | -2.59(-2.64 to -2.54) |
| middle_sdi | Deaths | 85 ~ 90 | -2.04(-2.11 to -1.97) |
| middle_sdi | Deaths | 90 ~ 95 | -1.48(-1.61 to -1.35) |
| middle_sdi | Deaths | 20 ~ 25 | -3.25(-4.15 to -2.34) |
| middle_sdi | Deaths | 25 ~ 30 | -3.4(-3.99 to -2.8) |
| middle_sdi | Deaths | 30 ~ 35 | -3.51(-3.94 to -3.09) |
| middle_sdi | Deaths | 35 ~ 40 | -3.63(-3.95 to -3.3) |
| middle_sdi | Deaths | 40 ~ 45 | -3.7(-3.94 to -3.46) |
| middle_sdi | Deaths | 45 ~ 50 | -3.74(-3.92 to -3.56) |
| middle_sdi | Deaths | 50 ~ 55 | -3.82(-3.95 to -3.7) |
| middle_sdi | Deaths | 55 ~ 60 | -3.85(-3.94 to -3.75) |
| middle_sdi | Deaths | 60 ~ 65 | -3.79(-3.87 to -3.72) |
| middle_sdi | Deaths | 65 ~ 70 | -3.55(-3.61 to -3.5) |
| middle_sdi | Deaths | 70 ~ 75 | -3.32(-3.37 to -3.27) |
| middle_sdi | Deaths | 75 ~ 80 | -3.04(-3.08 to -2.99) |
| middle_sdi | Deaths | 80 ~ 85 | -2.59(-2.64 to -2.54) |
| middle_sdi | Deaths | 85 ~ 90 | -2.04(-2.11 to -1.97) |
| middle_sdi | Deaths | 90 ~ 95 | -1.48(-1.61 to -1.35) |
| middle_sdi | Deaths | 20 ~ 25 | -3.25(-4.15 to -2.34) |
| middle_sdi | Deaths | 25 ~ 30 | -3.4(-3.99 to -2.8) |
| middle_sdi | Deaths | 30 ~ 35 | -3.51(-3.94 to -3.09) |
| middle_sdi | Deaths | 35 ~ 40 | -3.63(-3.95 to -3.3) |
| middle_sdi | Deaths | 40 ~ 45 | -3.7(-3.94 to -3.46) |
| middle_sdi | Deaths | 45 ~ 50 | -3.74(-3.92 to -3.56) |
| middle_sdi | Deaths | 50 ~ 55 | -3.82(-3.95 to -3.7) |
| middle_sdi | Deaths | 55 ~ 60 | -3.85(-3.94 to -3.75) |
| middle_sdi | Deaths | 60 ~ 65 | -3.79(-3.87 to -3.72) |
| middle_sdi | Deaths | 65 ~ 70 | -3.55(-3.61 to -3.5) |
| middle_sdi | Deaths | 70 ~ 75 | -3.32(-3.37 to -3.27) |
| middle_sdi | Deaths | 75 ~ 80 | -3.04(-3.08 to -2.99) |
| middle_sdi | Deaths | 80 ~ 85 | -2.59(-2.64 to -2.54) |
| middle_sdi | Deaths | 85 ~ 90 | -2.04(-2.11 to -1.97) |
| middle_sdi | Deaths | 90 ~ 95 | -1.48(-1.61 to -1.35) |
| middle_sdi | DALYs | 20 ~ 25 | -1.96(-2.35 to -1.57) |
| middle_sdi | DALYs | 25 ~ 30 | -2.07(-2.33 to -1.8) |
| middle_sdi | DALYs | 30 ~ 35 | -2.2(-2.41 to -2) |
| middle_sdi | DALYs | 35 ~ 40 | -2.43(-2.6 to -2.26) |
| middle_sdi | DALYs | 40 ~ 45 | -2.68(-2.82 to -2.54) |
| middle_sdi | DALYs | 45 ~ 50 | -2.89(-3 to -2.78) |
| middle_sdi | DALYs | 50 ~ 55 | -3.1(-3.19 to -3.01) |
| middle_sdi | DALYs | 55 ~ 60 | -3.24(-3.31 to -3.16) |
| middle_sdi | DALYs | 60 ~ 65 | -3.28(-3.35 to -3.22) |
| middle_sdi | DALYs | 65 ~ 70 | -3.13(-3.19 to -3.08) |
| middle_sdi | DALYs | 70 ~ 75 | -2.98(-3.03 to -2.93) |
| middle_sdi | DALYs | 75 ~ 80 | -2.76(-2.82 to -2.7) |
| middle_sdi | DALYs | 80 ~ 85 | -2.38(-2.45 to -2.31) |
| middle_sdi | DALYs | 85 ~ 90 | -1.88(-1.99 to -1.78) |
| middle_sdi | DALYs | 90 ~ 95 | -1.36(-1.57 to -1.14) |
| middle_sdi | DALYs | 20 ~ 25 | -1.96(-2.35 to -1.57) |
| middle_sdi | DALYs | 25 ~ 30 | -2.07(-2.33 to -1.8) |
| middle_sdi | DALYs | 30 ~ 35 | -2.2(-2.41 to -2) |
| middle_sdi | DALYs | 35 ~ 40 | -2.43(-2.6 to -2.26) |
| middle_sdi | DALYs | 40 ~ 45 | -2.68(-2.82 to -2.54) |
| middle_sdi | DALYs | 45 ~ 50 | -2.89(-3 to -2.78) |
| middle_sdi | DALYs | 50 ~ 55 | -3.1(-3.19 to -3.01) |
| middle_sdi | DALYs | 55 ~ 60 | -3.24(-3.31 to -3.16) |
| middle_sdi | DALYs | 60 ~ 65 | -3.28(-3.35 to -3.22) |
| middle_sdi | DALYs | 65 ~ 70 | -3.13(-3.19 to -3.08) |
| middle_sdi | DALYs | 70 ~ 75 | -2.98(-3.03 to -2.93) |
| middle_sdi | DALYs | 75 ~ 80 | -2.76(-2.82 to -2.7) |
| middle_sdi | DALYs | 80 ~ 85 | -2.38(-2.45 to -2.31) |
| middle_sdi | DALYs | 85 ~ 90 | -1.88(-1.99 to -1.78) |
| middle_sdi | DALYs | 90 ~ 95 | -1.36(-1.57 to -1.14) |
| middle_sdi | DALYs | 20 ~ 25 | -1.96(-2.35 to -1.57) |
| middle_sdi | DALYs | 25 ~ 30 | -2.07(-2.33 to -1.8) |
| middle_sdi | DALYs | 30 ~ 35 | -2.2(-2.41 to -2) |
| middle_sdi | DALYs | 35 ~ 40 | -2.43(-2.6 to -2.26) |
| middle_sdi | DALYs | 40 ~ 45 | -2.68(-2.82 to -2.54) |
| middle_sdi | DALYs | 45 ~ 50 | -2.89(-3 to -2.78) |
| middle_sdi | DALYs | 50 ~ 55 | -3.1(-3.19 to -3.01) |
| middle_sdi | DALYs | 55 ~ 60 | -3.24(-3.31 to -3.16) |
| middle_sdi | DALYs | 60 ~ 65 | -3.28(-3.35 to -3.22) |
| middle_sdi | DALYs | 65 ~ 70 | -3.13(-3.19 to -3.08) |
| middle_sdi | DALYs | 70 ~ 75 | -2.98(-3.03 to -2.93) |
| middle_sdi | DALYs | 75 ~ 80 | -2.76(-2.82 to -2.7) |
| middle_sdi | DALYs | 80 ~ 85 | -2.38(-2.45 to -2.31) |
| middle_sdi | DALYs | 85 ~ 90 | -1.88(-1.99 to -1.78) |
| middle_sdi | DALYs | 90 ~ 95 | -1.36(-1.57 to -1.14) |
| middle_sdi | DALYs | 20 ~ 25 | -1.96(-2.35 to -1.57) |
| middle_sdi | DALYs | 25 ~ 30 | -2.07(-2.33 to -1.8) |
| middle_sdi | DALYs | 30 ~ 35 | -2.2(-2.41 to -2) |
| middle_sdi | DALYs | 35 ~ 40 | -2.43(-2.6 to -2.26) |
| middle_sdi | DALYs | 40 ~ 45 | -2.68(-2.82 to -2.54) |
| middle_sdi | DALYs | 45 ~ 50 | -2.89(-3 to -2.78) |
| middle_sdi | DALYs | 50 ~ 55 | -3.1(-3.19 to -3.01) |
| middle_sdi | DALYs | 55 ~ 60 | -3.24(-3.31 to -3.16) |
| middle_sdi | DALYs | 60 ~ 65 | -3.28(-3.35 to -3.22) |
| middle_sdi | DALYs | 65 ~ 70 | -3.13(-3.19 to -3.08) |
| middle_sdi | DALYs | 70 ~ 75 | -2.98(-3.03 to -2.93) |
| middle_sdi | DALYs | 75 ~ 80 | -2.76(-2.82 to -2.7) |
| middle_sdi | DALYs | 80 ~ 85 | -2.38(-2.45 to -2.31) |
| middle_sdi | DALYs | 85 ~ 90 | -1.88(-1.99 to -1.78) |
| middle_sdi | DALYs | 90 ~ 95 | -1.36(-1.57 to -1.14) |
| low-middle_sdi | Prevalence | 20 ~ 25 | -0.21(-0.25 to -0.18) |
| low-middle_sdi | Prevalence | 25 ~ 30 | -0.22(-0.25 to -0.2) |
| low-middle_sdi | Prevalence | 30 ~ 35 | -0.22(-0.25 to -0.2) |
| low-middle_sdi | Prevalence | 35 ~ 40 | -0.22(-0.24 to -0.2) |
| low-middle_sdi | Prevalence | 40 ~ 45 | -0.21(-0.23 to -0.19) |
| low-middle_sdi | Prevalence | 45 ~ 50 | -0.2(-0.22 to -0.18) |
| low-middle_sdi | Prevalence | 50 ~ 55 | -0.17(-0.19 to -0.16) |
| low-middle_sdi | Prevalence | 55 ~ 60 | -0.13(-0.14 to -0.11) |
| low-middle_sdi | Prevalence | 60 ~ 65 | -0.04(-0.05 to -0.03) |
| low-middle_sdi | Prevalence | 65 ~ 70 | 0.08(0.07 to 0.09) |
| low-middle_sdi | Prevalence | 70 ~ 75 | 0.21(0.2 to 0.22) |
| low-middle_sdi | Prevalence | 75 ~ 80 | 0.33(0.31 to 0.35) |
| low-middle_sdi | Prevalence | 80 ~ 85 | 0.43(0.41 to 0.45) |
| low-middle_sdi | Prevalence | 85 ~ 90 | 0.51(0.48 to 0.54) |
| low-middle_sdi | Prevalence | 90 ~ 95 | 0.59(0.53 to 0.65) |
| low-middle_sdi | Prevalence | 20 ~ 25 | -0.21(-0.25 to -0.18) |
| low-middle_sdi | Prevalence | 25 ~ 30 | -0.22(-0.25 to -0.2) |
| low-middle_sdi | Prevalence | 30 ~ 35 | -0.22(-0.25 to -0.2) |
| low-middle_sdi | Prevalence | 35 ~ 40 | -0.22(-0.24 to -0.2) |
| low-middle_sdi | Prevalence | 40 ~ 45 | -0.21(-0.23 to -0.19) |
| low-middle_sdi | Prevalence | 45 ~ 50 | -0.2(-0.22 to -0.18) |
| low-middle_sdi | Prevalence | 50 ~ 55 | -0.17(-0.19 to -0.16) |
| low-middle_sdi | Prevalence | 55 ~ 60 | -0.13(-0.14 to -0.11) |
| low-middle_sdi | Prevalence | 60 ~ 65 | -0.04(-0.05 to -0.03) |
| low-middle_sdi | Prevalence | 65 ~ 70 | 0.08(0.07 to 0.09) |
| low-middle_sdi | Prevalence | 70 ~ 75 | 0.21(0.2 to 0.22) |
| low-middle_sdi | Prevalence | 75 ~ 80 | 0.33(0.31 to 0.35) |
| low-middle_sdi | Prevalence | 80 ~ 85 | 0.43(0.41 to 0.45) |
| low-middle_sdi | Prevalence | 85 ~ 90 | 0.51(0.48 to 0.54) |
| low-middle_sdi | Prevalence | 90 ~ 95 | 0.59(0.53 to 0.65) |
| low-middle_sdi | Prevalence | 20 ~ 25 | -0.21(-0.25 to -0.18) |
| low-middle_sdi | Prevalence | 25 ~ 30 | -0.22(-0.25 to -0.2) |
| low-middle_sdi | Prevalence | 30 ~ 35 | -0.22(-0.25 to -0.2) |
| low-middle_sdi | Prevalence | 35 ~ 40 | -0.22(-0.24 to -0.2) |
| low-middle_sdi | Prevalence | 40 ~ 45 | -0.21(-0.23 to -0.19) |
| low-middle_sdi | Prevalence | 45 ~ 50 | -0.2(-0.22 to -0.18) |
| low-middle_sdi | Prevalence | 50 ~ 55 | -0.17(-0.19 to -0.16) |
| low-middle_sdi | Prevalence | 55 ~ 60 | -0.13(-0.14 to -0.11) |
| low-middle_sdi | Prevalence | 60 ~ 65 | -0.04(-0.05 to -0.03) |
| low-middle_sdi | Prevalence | 65 ~ 70 | 0.08(0.07 to 0.09) |
| low-middle_sdi | Prevalence | 70 ~ 75 | 0.21(0.2 to 0.22) |
| low-middle_sdi | Prevalence | 75 ~ 80 | 0.33(0.31 to 0.35) |
| low-middle_sdi | Prevalence | 80 ~ 85 | 0.43(0.41 to 0.45) |
| low-middle_sdi | Prevalence | 85 ~ 90 | 0.51(0.48 to 0.54) |
| low-middle_sdi | Prevalence | 90 ~ 95 | 0.59(0.53 to 0.65) |
| low-middle_sdi | Prevalence | 20 ~ 25 | -0.21(-0.25 to -0.18) |
| low-middle_sdi | Prevalence | 25 ~ 30 | -0.22(-0.25 to -0.2) |
| low-middle_sdi | Prevalence | 30 ~ 35 | -0.22(-0.25 to -0.2) |
| low-middle_sdi | Prevalence | 35 ~ 40 | -0.22(-0.24 to -0.2) |
| low-middle_sdi | Prevalence | 40 ~ 45 | -0.21(-0.23 to -0.19) |
| low-middle_sdi | Prevalence | 45 ~ 50 | -0.2(-0.22 to -0.18) |
| low-middle_sdi | Prevalence | 50 ~ 55 | -0.17(-0.19 to -0.16) |
| low-middle_sdi | Prevalence | 55 ~ 60 | -0.13(-0.14 to -0.11) |
| low-middle_sdi | Prevalence | 60 ~ 65 | -0.04(-0.05 to -0.03) |
| low-middle_sdi | Prevalence | 65 ~ 70 | 0.08(0.07 to 0.09) |
| low-middle_sdi | Prevalence | 70 ~ 75 | 0.21(0.2 to 0.22) |
| low-middle_sdi | Prevalence | 75 ~ 80 | 0.33(0.31 to 0.35) |
| low-middle_sdi | Prevalence | 80 ~ 85 | 0.43(0.41 to 0.45) |
| low-middle_sdi | Prevalence | 85 ~ 90 | 0.51(0.48 to 0.54) |
| low-middle_sdi | Prevalence | 90 ~ 95 | 0.59(0.53 to 0.65) |
| low-middle_sdi | Deaths | 20 ~ 25 | -2.08(-3.47 to -0.67) |
| low-middle_sdi | Deaths | 25 ~ 30 | -2.04(-2.98 to -1.09) |
| low-middle_sdi | Deaths | 30 ~ 35 | -1.78(-2.48 to -1.09) |
| low-middle_sdi | Deaths | 35 ~ 40 | -1.57(-2.09 to -1.04) |
| low-middle_sdi | Deaths | 40 ~ 45 | -1.38(-1.77 to -0.99) |
| low-middle_sdi | Deaths | 45 ~ 50 | -1.32(-1.6 to -1.04) |
| low-middle_sdi | Deaths | 50 ~ 55 | -1.22(-1.43 to -1.02) |
| low-middle_sdi | Deaths | 55 ~ 60 | -1.14(-1.3 to -0.99) |
| low-middle_sdi | Deaths | 60 ~ 65 | -1.1(-1.22 to -0.98) |
| low-middle_sdi | Deaths | 65 ~ 70 | -0.89(-0.99 to -0.79) |
| low-middle_sdi | Deaths | 70 ~ 75 | -0.53(-0.62 to -0.43) |
| low-middle_sdi | Deaths | 75 ~ 80 | -0.14(-0.23 to -0.04) |
| low-middle_sdi | Deaths | 80 ~ 85 | 0.26(0.14 to 0.38) |
| low-middle_sdi | Deaths | 85 ~ 90 | 0.62(0.46 to 0.79) |
| low-middle_sdi | Deaths | 90 ~ 95 | 0.9(0.57 to 1.23) |
| low-middle_sdi | Deaths | 20 ~ 25 | -2.08(-3.47 to -0.67) |
| low-middle_sdi | Deaths | 25 ~ 30 | -2.04(-2.98 to -1.09) |
| low-middle_sdi | Deaths | 30 ~ 35 | -1.78(-2.48 to -1.09) |
| low-middle_sdi | Deaths | 35 ~ 40 | -1.57(-2.09 to -1.04) |
| low-middle_sdi | Deaths | 40 ~ 45 | -1.38(-1.77 to -0.99) |
| low-middle_sdi | Deaths | 45 ~ 50 | -1.32(-1.6 to -1.04) |
| low-middle_sdi | Deaths | 50 ~ 55 | -1.22(-1.43 to -1.02) |
| low-middle_sdi | Deaths | 55 ~ 60 | -1.14(-1.3 to -0.99) |
| low-middle_sdi | Deaths | 60 ~ 65 | -1.1(-1.22 to -0.98) |
| low-middle_sdi | Deaths | 65 ~ 70 | -0.89(-0.99 to -0.79) |
| low-middle_sdi | Deaths | 70 ~ 75 | -0.53(-0.62 to -0.43) |
| low-middle_sdi | Deaths | 75 ~ 80 | -0.14(-0.23 to -0.04) |
| low-middle_sdi | Deaths | 80 ~ 85 | 0.26(0.14 to 0.38) |
| low-middle_sdi | Deaths | 85 ~ 90 | 0.62(0.46 to 0.79) |
| low-middle_sdi | Deaths | 90 ~ 95 | 0.9(0.57 to 1.23) |
| low-middle_sdi | Deaths | 20 ~ 25 | -2.08(-3.47 to -0.67) |
| low-middle_sdi | Deaths | 25 ~ 30 | -2.04(-2.98 to -1.09) |
| low-middle_sdi | Deaths | 30 ~ 35 | -1.78(-2.48 to -1.09) |
| low-middle_sdi | Deaths | 35 ~ 40 | -1.57(-2.09 to -1.04) |
| low-middle_sdi | Deaths | 40 ~ 45 | -1.38(-1.77 to -0.99) |
| low-middle_sdi | Deaths | 45 ~ 50 | -1.32(-1.6 to -1.04) |
| low-middle_sdi | Deaths | 50 ~ 55 | -1.22(-1.43 to -1.02) |
| low-middle_sdi | Deaths | 55 ~ 60 | -1.14(-1.3 to -0.99) |
| low-middle_sdi | Deaths | 60 ~ 65 | -1.1(-1.22 to -0.98) |
| low-middle_sdi | Deaths | 65 ~ 70 | -0.89(-0.99 to -0.79) |
| low-middle_sdi | Deaths | 70 ~ 75 | -0.53(-0.62 to -0.43) |
| low-middle_sdi | Deaths | 75 ~ 80 | -0.14(-0.23 to -0.04) |
| low-middle_sdi | Deaths | 80 ~ 85 | 0.26(0.14 to 0.38) |
| low-middle_sdi | Deaths | 85 ~ 90 | 0.62(0.46 to 0.79) |
| low-middle_sdi | Deaths | 90 ~ 95 | 0.9(0.57 to 1.23) |
| low-middle_sdi | Deaths | 20 ~ 25 | -2.08(-3.47 to -0.67) |
| low-middle_sdi | Deaths | 25 ~ 30 | -2.04(-2.98 to -1.09) |
| low-middle_sdi | Deaths | 30 ~ 35 | -1.78(-2.48 to -1.09) |
| low-middle_sdi | Deaths | 35 ~ 40 | -1.57(-2.09 to -1.04) |
| low-middle_sdi | Deaths | 40 ~ 45 | -1.38(-1.77 to -0.99) |
| low-middle_sdi | Deaths | 45 ~ 50 | -1.32(-1.6 to -1.04) |
| low-middle_sdi | Deaths | 50 ~ 55 | -1.22(-1.43 to -1.02) |
| low-middle_sdi | Deaths | 55 ~ 60 | -1.14(-1.3 to -0.99) |
| low-middle_sdi | Deaths | 60 ~ 65 | -1.1(-1.22 to -0.98) |
| low-middle_sdi | Deaths | 65 ~ 70 | -0.89(-0.99 to -0.79) |
| low-middle_sdi | Deaths | 70 ~ 75 | -0.53(-0.62 to -0.43) |
| low-middle_sdi | Deaths | 75 ~ 80 | -0.14(-0.23 to -0.04) |
| low-middle_sdi | Deaths | 80 ~ 85 | 0.26(0.14 to 0.38) |
| low-middle_sdi | Deaths | 85 ~ 90 | 0.62(0.46 to 0.79) |
| low-middle_sdi | Deaths | 90 ~ 95 | 0.9(0.57 to 1.23) |
| low-middle_sdi | DALYs | 20 ~ 25 | -1.11(-1.61 to -0.61) |
| low-middle_sdi | DALYs | 25 ~ 30 | -1.17(-1.53 to -0.81) |
| low-middle_sdi | DALYs | 30 ~ 35 | -1.11(-1.4 to -0.82) |
| low-middle_sdi | DALYs | 35 ~ 40 | -1.06(-1.31 to -0.82) |
| low-middle_sdi | DALYs | 40 ~ 45 | -1.03(-1.23 to -0.83) |
| low-middle_sdi | DALYs | 45 ~ 50 | -1.05(-1.21 to -0.89) |
| low-middle_sdi | DALYs | 50 ~ 55 | -1.03(-1.16 to -0.9) |
| low-middle_sdi | DALYs | 55 ~ 60 | -1(-1.11 to -0.89) |
| low-middle_sdi | DALYs | 60 ~ 65 | -0.96(-1.05 to -0.87) |
| low-middle_sdi | DALYs | 65 ~ 70 | -0.78(-0.87 to -0.69) |
| low-middle_sdi | DALYs | 70 ~ 75 | -0.46(-0.55 to -0.37) |
| low-middle_sdi | DALYs | 75 ~ 80 | -0.1(-0.21 to 0) |
| low-middle_sdi | DALYs | 80 ~ 85 | 0.25(0.12 to 0.39) |
| low-middle_sdi | DALYs | 85 ~ 90 | 0.58(0.36 to 0.8) |
| low-middle_sdi | DALYs | 90 ~ 95 | 0.82(0.37 to 1.27) |
| low-middle_sdi | DALYs | 20 ~ 25 | -1.11(-1.61 to -0.61) |
| low-middle_sdi | DALYs | 25 ~ 30 | -1.17(-1.53 to -0.81) |
| low-middle_sdi | DALYs | 30 ~ 35 | -1.11(-1.4 to -0.82) |
| low-middle_sdi | DALYs | 35 ~ 40 | -1.06(-1.31 to -0.82) |
| low-middle_sdi | DALYs | 40 ~ 45 | -1.03(-1.23 to -0.83) |
| low-middle_sdi | DALYs | 45 ~ 50 | -1.05(-1.21 to -0.89) |
| low-middle_sdi | DALYs | 50 ~ 55 | -1.03(-1.16 to -0.9) |
| low-middle_sdi | DALYs | 55 ~ 60 | -1(-1.11 to -0.89) |
| low-middle_sdi | DALYs | 60 ~ 65 | -0.96(-1.05 to -0.87) |
| low-middle_sdi | DALYs | 65 ~ 70 | -0.78(-0.87 to -0.69) |
| low-middle_sdi | DALYs | 70 ~ 75 | -0.46(-0.55 to -0.37) |
| low-middle_sdi | DALYs | 75 ~ 80 | -0.1(-0.21 to 0) |
| low-middle_sdi | DALYs | 80 ~ 85 | 0.25(0.12 to 0.39) |
| low-middle_sdi | DALYs | 85 ~ 90 | 0.58(0.36 to 0.8) |
| low-middle_sdi | DALYs | 90 ~ 95 | 0.82(0.37 to 1.27) |
| low-middle_sdi | DALYs | 20 ~ 25 | -1.11(-1.61 to -0.61) |
| low-middle_sdi | DALYs | 25 ~ 30 | -1.17(-1.53 to -0.81) |
| low-middle_sdi | DALYs | 30 ~ 35 | -1.11(-1.4 to -0.82) |
| low-middle_sdi | DALYs | 35 ~ 40 | -1.06(-1.31 to -0.82) |
| low-middle_sdi | DALYs | 40 ~ 45 | -1.03(-1.23 to -0.83) |
| low-middle_sdi | DALYs | 45 ~ 50 | -1.05(-1.21 to -0.89) |
| low-middle_sdi | DALYs | 50 ~ 55 | -1.03(-1.16 to -0.9) |
| low-middle_sdi | DALYs | 55 ~ 60 | -1(-1.11 to -0.89) |
| low-middle_sdi | DALYs | 60 ~ 65 | -0.96(-1.05 to -0.87) |
| low-middle_sdi | DALYs | 65 ~ 70 | -0.78(-0.87 to -0.69) |
| low-middle_sdi | DALYs | 70 ~ 75 | -0.46(-0.55 to -0.37) |
| low-middle_sdi | DALYs | 75 ~ 80 | -0.1(-0.21 to 0) |
| low-middle_sdi | DALYs | 80 ~ 85 | 0.25(0.12 to 0.39) |
| low-middle_sdi | DALYs | 85 ~ 90 | 0.58(0.36 to 0.8) |
| low-middle_sdi | DALYs | 90 ~ 95 | 0.82(0.37 to 1.27) |
| low-middle_sdi | DALYs | 20 ~ 25 | -1.11(-1.61 to -0.61) |
| low-middle_sdi | DALYs | 25 ~ 30 | -1.17(-1.53 to -0.81) |
| low-middle_sdi | DALYs | 30 ~ 35 | -1.11(-1.4 to -0.82) |
| low-middle_sdi | DALYs | 35 ~ 40 | -1.06(-1.31 to -0.82) |
| low-middle_sdi | DALYs | 40 ~ 45 | -1.03(-1.23 to -0.83) |
| low-middle_sdi | DALYs | 45 ~ 50 | -1.05(-1.21 to -0.89) |
| low-middle_sdi | DALYs | 50 ~ 55 | -1.03(-1.16 to -0.9) |
| low-middle_sdi | DALYs | 55 ~ 60 | -1(-1.11 to -0.89) |
| low-middle_sdi | DALYs | 60 ~ 65 | -0.96(-1.05 to -0.87) |
| low-middle_sdi | DALYs | 65 ~ 70 | -0.78(-0.87 to -0.69) |
| low-middle_sdi | DALYs | 70 ~ 75 | -0.46(-0.55 to -0.37) |
| low-middle_sdi | DALYs | 75 ~ 80 | -0.1(-0.21 to 0) |
| low-middle_sdi | DALYs | 80 ~ 85 | 0.25(0.12 to 0.39) |
| low-middle_sdi | DALYs | 85 ~ 90 | 0.58(0.36 to 0.8) |
| low-middle_sdi | DALYs | 90 ~ 95 | 0.82(0.37 to 1.27) |
| low_sdi | Prevalence | 20 ~ 25 | -0.22(-0.29 to -0.16) |
| low_sdi | Prevalence | 25 ~ 30 | -0.22(-0.27 to -0.17) |
| low_sdi | Prevalence | 30 ~ 35 | -0.2(-0.25 to -0.16) |
| low_sdi | Prevalence | 35 ~ 40 | -0.19(-0.23 to -0.15) |
| low_sdi | Prevalence | 40 ~ 45 | -0.2(-0.23 to -0.16) |
| low_sdi | Prevalence | 45 ~ 50 | -0.17(-0.2 to -0.13) |
| low_sdi | Prevalence | 50 ~ 55 | -0.1(-0.14 to -0.07) |
| low_sdi | Prevalence | 55 ~ 60 | -0.01(-0.04 to 0.02) |
| low_sdi | Prevalence | 60 ~ 65 | 0.12(0.09 to 0.15) |
| low_sdi | Prevalence | 65 ~ 70 | 0.27(0.24 to 0.31) |
| low_sdi | Prevalence | 70 ~ 75 | 0.4(0.36 to 0.43) |
| low_sdi | Prevalence | 75 ~ 80 | 0.47(0.43 to 0.51) |
| low_sdi | Prevalence | 80 ~ 85 | 0.49(0.43 to 0.55) |
| low_sdi | Prevalence | 85 ~ 90 | 0.51(0.42 to 0.61) |
| low_sdi | Prevalence | 90 ~ 95 | 0.61(0.41 to 0.8) |
| low_sdi | Prevalence | 20 ~ 25 | -0.22(-0.29 to -0.16) |
| low_sdi | Prevalence | 25 ~ 30 | -0.22(-0.27 to -0.17) |
| low_sdi | Prevalence | 30 ~ 35 | -0.2(-0.25 to -0.16) |
| low_sdi | Prevalence | 35 ~ 40 | -0.19(-0.23 to -0.15) |
| low_sdi | Prevalence | 40 ~ 45 | -0.2(-0.23 to -0.16) |
| low_sdi | Prevalence | 45 ~ 50 | -0.17(-0.2 to -0.13) |
| low_sdi | Prevalence | 50 ~ 55 | -0.1(-0.14 to -0.07) |
| low_sdi | Prevalence | 55 ~ 60 | -0.01(-0.04 to 0.02) |
| low_sdi | Prevalence | 60 ~ 65 | 0.12(0.09 to 0.15) |
| low_sdi | Prevalence | 65 ~ 70 | 0.27(0.24 to 0.31) |
| low_sdi | Prevalence | 70 ~ 75 | 0.4(0.36 to 0.43) |
| low_sdi | Prevalence | 75 ~ 80 | 0.47(0.43 to 0.51) |
| low_sdi | Prevalence | 80 ~ 85 | 0.49(0.43 to 0.55) |
| low_sdi | Prevalence | 85 ~ 90 | 0.51(0.42 to 0.61) |
| low_sdi | Prevalence | 90 ~ 95 | 0.61(0.41 to 0.8) |
| low_sdi | Prevalence | 20 ~ 25 | -0.22(-0.29 to -0.16) |
| low_sdi | Prevalence | 25 ~ 30 | -0.22(-0.27 to -0.17) |
| low_sdi | Prevalence | 30 ~ 35 | -0.2(-0.25 to -0.16) |
| low_sdi | Prevalence | 35 ~ 40 | -0.19(-0.23 to -0.15) |
| low_sdi | Prevalence | 40 ~ 45 | -0.2(-0.23 to -0.16) |
| low_sdi | Prevalence | 45 ~ 50 | -0.17(-0.2 to -0.13) |
| low_sdi | Prevalence | 50 ~ 55 | -0.1(-0.14 to -0.07) |
| low_sdi | Prevalence | 55 ~ 60 | -0.01(-0.04 to 0.02) |
| low_sdi | Prevalence | 60 ~ 65 | 0.12(0.09 to 0.15) |
| low_sdi | Prevalence | 65 ~ 70 | 0.27(0.24 to 0.31) |
| low_sdi | Prevalence | 70 ~ 75 | 0.4(0.36 to 0.43) |
| low_sdi | Prevalence | 75 ~ 80 | 0.47(0.43 to 0.51) |
| low_sdi | Prevalence | 80 ~ 85 | 0.49(0.43 to 0.55) |
| low_sdi | Prevalence | 85 ~ 90 | 0.51(0.42 to 0.61) |
| low_sdi | Prevalence | 90 ~ 95 | 0.61(0.41 to 0.8) |
| low_sdi | Prevalence | 20 ~ 25 | -0.22(-0.29 to -0.16) |
| low_sdi | Prevalence | 25 ~ 30 | -0.22(-0.27 to -0.17) |
| low_sdi | Prevalence | 30 ~ 35 | -0.2(-0.25 to -0.16) |
| low_sdi | Prevalence | 35 ~ 40 | -0.19(-0.23 to -0.15) |
| low_sdi | Prevalence | 40 ~ 45 | -0.2(-0.23 to -0.16) |
| low_sdi | Prevalence | 45 ~ 50 | -0.17(-0.2 to -0.13) |
| low_sdi | Prevalence | 50 ~ 55 | -0.1(-0.14 to -0.07) |
| low_sdi | Prevalence | 55 ~ 60 | -0.01(-0.04 to 0.02) |
| low_sdi | Prevalence | 60 ~ 65 | 0.12(0.09 to 0.15) |
| low_sdi | Prevalence | 65 ~ 70 | 0.27(0.24 to 0.31) |
| low_sdi | Prevalence | 70 ~ 75 | 0.4(0.36 to 0.43) |
| low_sdi | Prevalence | 75 ~ 80 | 0.47(0.43 to 0.51) |
| low_sdi | Prevalence | 80 ~ 85 | 0.49(0.43 to 0.55) |
| low_sdi | Prevalence | 85 ~ 90 | 0.51(0.42 to 0.61) |
| low_sdi | Prevalence | 90 ~ 95 | 0.61(0.41 to 0.8) |
| low_sdi | Deaths | 20 ~ 25 | -1.26(-2.68 to 0.17) |
| low_sdi | Deaths | 25 ~ 30 | -1.36(-2.37 to -0.34) |
| low_sdi | Deaths | 30 ~ 35 | -1.43(-2.22 to -0.64) |
| low_sdi | Deaths | 35 ~ 40 | -1.53(-2.15 to -0.9) |
| low_sdi | Deaths | 40 ~ 45 | -1.64(-2.12 to -1.16) |
| low_sdi | Deaths | 45 ~ 50 | -1.7(-2.06 to -1.34) |
| low_sdi | Deaths | 50 ~ 55 | -1.67(-1.94 to -1.41) |
| low_sdi | Deaths | 55 ~ 60 | -1.51(-1.72 to -1.3) |
| low_sdi | Deaths | 60 ~ 65 | -1.26(-1.43 to -1.1) |
| low_sdi | Deaths | 65 ~ 70 | -0.9(-1.04 to -0.76) |
| low_sdi | Deaths | 70 ~ 75 | -0.41(-0.54 to -0.28) |
| low_sdi | Deaths | 75 ~ 80 | 0.03(-0.11 to 0.17) |
| low_sdi | Deaths | 80 ~ 85 | 0.34(0.17 to 0.51) |
| low_sdi | Deaths | 85 ~ 90 | 0.54(0.28 to 0.8) |
| low_sdi | Deaths | 90 ~ 95 | 0.73(0.22 to 1.25) |
| low_sdi | Deaths | 20 ~ 25 | -1.26(-2.68 to 0.17) |
| low_sdi | Deaths | 25 ~ 30 | -1.36(-2.37 to -0.34) |
| low_sdi | Deaths | 30 ~ 35 | -1.43(-2.22 to -0.64) |
| low_sdi | Deaths | 35 ~ 40 | -1.53(-2.15 to -0.9) |
| low_sdi | Deaths | 40 ~ 45 | -1.64(-2.12 to -1.16) |
| low_sdi | Deaths | 45 ~ 50 | -1.7(-2.06 to -1.34) |
| low_sdi | Deaths | 50 ~ 55 | -1.67(-1.94 to -1.41) |
| low_sdi | Deaths | 55 ~ 60 | -1.51(-1.72 to -1.3) |
| low_sdi | Deaths | 60 ~ 65 | -1.26(-1.43 to -1.1) |
| low_sdi | Deaths | 65 ~ 70 | -0.9(-1.04 to -0.76) |
| low_sdi | Deaths | 70 ~ 75 | -0.41(-0.54 to -0.28) |
| low_sdi | Deaths | 75 ~ 80 | 0.03(-0.11 to 0.17) |
| low_sdi | Deaths | 80 ~ 85 | 0.34(0.17 to 0.51) |
| low_sdi | Deaths | 85 ~ 90 | 0.54(0.28 to 0.8) |
| low_sdi | Deaths | 90 ~ 95 | 0.73(0.22 to 1.25) |
| low_sdi | Deaths | 20 ~ 25 | -1.26(-2.68 to 0.17) |
| low_sdi | Deaths | 25 ~ 30 | -1.36(-2.37 to -0.34) |
| low_sdi | Deaths | 30 ~ 35 | -1.43(-2.22 to -0.64) |
| low_sdi | Deaths | 35 ~ 40 | -1.53(-2.15 to -0.9) |
| low_sdi | Deaths | 40 ~ 45 | -1.64(-2.12 to -1.16) |
| low_sdi | Deaths | 45 ~ 50 | -1.7(-2.06 to -1.34) |
| low_sdi | Deaths | 50 ~ 55 | -1.67(-1.94 to -1.41) |
| low_sdi | Deaths | 55 ~ 60 | -1.51(-1.72 to -1.3) |
| low_sdi | Deaths | 60 ~ 65 | -1.26(-1.43 to -1.1) |
| low_sdi | Deaths | 65 ~ 70 | -0.9(-1.04 to -0.76) |
| low_sdi | Deaths | 70 ~ 75 | -0.41(-0.54 to -0.28) |
| low_sdi | Deaths | 75 ~ 80 | 0.03(-0.11 to 0.17) |
| low_sdi | Deaths | 80 ~ 85 | 0.34(0.17 to 0.51) |
| low_sdi | Deaths | 85 ~ 90 | 0.54(0.28 to 0.8) |
| low_sdi | Deaths | 90 ~ 95 | 0.73(0.22 to 1.25) |
| low_sdi | Deaths | 20 ~ 25 | -1.26(-2.68 to 0.17) |
| low_sdi | Deaths | 25 ~ 30 | -1.36(-2.37 to -0.34) |
| low_sdi | Deaths | 30 ~ 35 | -1.43(-2.22 to -0.64) |
| low_sdi | Deaths | 35 ~ 40 | -1.53(-2.15 to -0.9) |
| low_sdi | Deaths | 40 ~ 45 | -1.64(-2.12 to -1.16) |
| low_sdi | Deaths | 45 ~ 50 | -1.7(-2.06 to -1.34) |
| low_sdi | Deaths | 50 ~ 55 | -1.67(-1.94 to -1.41) |
| low_sdi | Deaths | 55 ~ 60 | -1.51(-1.72 to -1.3) |
| low_sdi | Deaths | 60 ~ 65 | -1.26(-1.43 to -1.1) |
| low_sdi | Deaths | 65 ~ 70 | -0.9(-1.04 to -0.76) |
| low_sdi | Deaths | 70 ~ 75 | -0.41(-0.54 to -0.28) |
| low_sdi | Deaths | 75 ~ 80 | 0.03(-0.11 to 0.17) |
| low_sdi | Deaths | 80 ~ 85 | 0.34(0.17 to 0.51) |
| low_sdi | Deaths | 85 ~ 90 | 0.54(0.28 to 0.8) |
| low_sdi | Deaths | 90 ~ 95 | 0.73(0.22 to 1.25) |
| low_sdi | DALYs | 20 ~ 25 | -0.75(-1.22 to -0.27) |
| low_sdi | DALYs | 25 ~ 30 | -0.82(-1.18 to -0.45) |
| low_sdi | DALYs | 30 ~ 35 | -0.9(-1.2 to -0.59) |
| low_sdi | DALYs | 35 ~ 40 | -1(-1.27 to -0.74) |
| low_sdi | DALYs | 40 ~ 45 | -1.17(-1.39 to -0.94) |
| low_sdi | DALYs | 45 ~ 50 | -1.31(-1.49 to -1.12) |
| low_sdi | DALYs | 50 ~ 55 | -1.35(-1.5 to -1.19) |
| low_sdi | DALYs | 55 ~ 60 | -1.26(-1.4 to -1.13) |
| low_sdi | DALYs | 60 ~ 65 | -1.06(-1.18 to -0.95) |
| low_sdi | DALYs | 65 ~ 70 | -0.74(-0.85 to -0.63) |
| low_sdi | DALYs | 70 ~ 75 | -0.31(-0.42 to -0.19) |
| low_sdi | DALYs | 75 ~ 80 | 0.07(-0.07 to 0.21) |
| low_sdi | DALYs | 80 ~ 85 | 0.31(0.12 to 0.5) |
| low_sdi | DALYs | 85 ~ 90 | 0.47(0.16 to 0.79) |
| low_sdi | DALYs | 90 ~ 95 | 0.63(-0.04 to 1.31) |
| low_sdi | DALYs | 20 ~ 25 | -0.75(-1.22 to -0.27) |
| low_sdi | DALYs | 25 ~ 30 | -0.82(-1.18 to -0.45) |
| low_sdi | DALYs | 30 ~ 35 | -0.9(-1.2 to -0.59) |
| low_sdi | DALYs | 35 ~ 40 | -1(-1.27 to -0.74) |
| low_sdi | DALYs | 40 ~ 45 | -1.17(-1.39 to -0.94) |
| low_sdi | DALYs | 45 ~ 50 | -1.31(-1.49 to -1.12) |
| low_sdi | DALYs | 50 ~ 55 | -1.35(-1.5 to -1.19) |
| low_sdi | DALYs | 55 ~ 60 | -1.26(-1.4 to -1.13) |
| low_sdi | DALYs | 60 ~ 65 | -1.06(-1.18 to -0.95) |
| low_sdi | DALYs | 65 ~ 70 | -0.74(-0.85 to -0.63) |
| low_sdi | DALYs | 70 ~ 75 | -0.31(-0.42 to -0.19) |
| low_sdi | DALYs | 75 ~ 80 | 0.07(-0.07 to 0.21) |
| low_sdi | DALYs | 80 ~ 85 | 0.31(0.12 to 0.5) |
| low_sdi | DALYs | 85 ~ 90 | 0.47(0.16 to 0.79) |
| low_sdi | DALYs | 90 ~ 95 | 0.63(-0.04 to 1.31) |
| low_sdi | DALYs | 20 ~ 25 | -0.75(-1.22 to -0.27) |
| low_sdi | DALYs | 25 ~ 30 | -0.82(-1.18 to -0.45) |
| low_sdi | DALYs | 30 ~ 35 | -0.9(-1.2 to -0.59) |
| low_sdi | DALYs | 35 ~ 40 | -1(-1.27 to -0.74) |
| low_sdi | DALYs | 40 ~ 45 | -1.17(-1.39 to -0.94) |
| low_sdi | DALYs | 45 ~ 50 | -1.31(-1.49 to -1.12) |
| low_sdi | DALYs | 50 ~ 55 | -1.35(-1.5 to -1.19) |
| low_sdi | DALYs | 55 ~ 60 | -1.26(-1.4 to -1.13) |
| low_sdi | DALYs | 60 ~ 65 | -1.06(-1.18 to -0.95) |
| low_sdi | DALYs | 65 ~ 70 | -0.74(-0.85 to -0.63) |
| low_sdi | DALYs | 70 ~ 75 | -0.31(-0.42 to -0.19) |
| low_sdi | DALYs | 75 ~ 80 | 0.07(-0.07 to 0.21) |
| low_sdi | DALYs | 80 ~ 85 | 0.31(0.12 to 0.5) |
| low_sdi | DALYs | 85 ~ 90 | 0.47(0.16 to 0.79) |
| low_sdi | DALYs | 90 ~ 95 | 0.63(-0.04 to 1.31) |
| low_sdi | DALYs | 20 ~ 25 | -0.75(-1.22 to -0.27) |
| low_sdi | DALYs | 25 ~ 30 | -0.82(-1.18 to -0.45) |
| low_sdi | DALYs | 30 ~ 35 | -0.9(-1.2 to -0.59) |
| low_sdi | DALYs | 35 ~ 40 | -1(-1.27 to -0.74) |
| low_sdi | DALYs | 40 ~ 45 | -1.17(-1.39 to -0.94) |
| low_sdi | DALYs | 45 ~ 50 | -1.31(-1.49 to -1.12) |
| low_sdi | DALYs | 50 ~ 55 | -1.35(-1.5 to -1.19) |
| low_sdi | DALYs | 55 ~ 60 | -1.26(-1.4 to -1.13) |
| low_sdi | DALYs | 60 ~ 65 | -1.06(-1.18 to -0.95) |
| low_sdi | DALYs | 65 ~ 70 | -0.74(-0.85 to -0.63) |
| low_sdi | DALYs | 70 ~ 75 | -0.31(-0.42 to -0.19) |
| low_sdi | DALYs | 75 ~ 80 | 0.07(-0.07 to 0.21) |
| low_sdi | DALYs | 80 ~ 85 | 0.31(0.12 to 0.5) |
| low_sdi | DALYs | 85 ~ 90 | 0.47(0.16 to 0.79) |
| low_sdi | DALYs | 90 ~ 95 | 0.63(-0.04 to 1.31) |
