# Supplementary material for: Global, regional, and national temporal trends in prevalence, deaths and disability-adjusted life years for chronic pulmonary disease, 1990–2021: an age-period-cohort analysis based on the global burden of disease study 2021
Source: Front Med (Lausanne). 2025 Mar 4;12:1554442. doi: 10.3389/fmed.2025.1554442 (PMC11913687; doi:10.3389/fmed.2025.1554442)
Supplement: Supplementary file 5 [file Table_3.docx]

**Table S3 The local drift of Deaths, prevalence and DALYs from 1990 to 2021 for COPD across countries"**

**Region, measure, age, Local Drift（95%UI）（%）**

Australia,Prevalence,20 ~ 25,-1 28(-1 56 to -1)

Australia,Prevalence,25 ~ 30,-1 27(-1 47 to -1 08)

Australia,Prevalence,30 ~ 35,-1 23(-1 37 to -1 08)

Australia,Prevalence,35 ~ 40,-1 17(-1 29 to -1 06)

Australia,Prevalence,40 ~ 45,-1 15(-1 25 to -1 06)

Australia,Prevalence,45 ~ 50,-1 18(-1 25 to -1 1)

Australia,Prevalence,50 ~ 55,-1 2(-1 26 to -1 14)

Australia,Prevalence,55 ~ 60,-1 19(-1 24 to -1 14)

Australia,Prevalence,60 ~ 65,-1 14(-1 18 to -1 1)

Australia,Prevalence,65 ~ 70,-1 07(-1 1 to -1 03)

Australia,Prevalence,70 ~ 75,-0 96(-0 99 to -0 93)

Australia,Prevalence,75 ~ 80,-0 82(-0 86 to -0 78)

Australia,Prevalence,80 ~ 85,-0 66(-0 7 to -0 62)

Australia,Prevalence,85 ~ 90,-0 5(-0 57 to -0 44)

Australia,Prevalence,90 ~ 95,-0 37(-0 49 to -0 25)

Australia,Prevalence,20 ~ 25,-1 28(-1 56 to -1)

Australia,Prevalence,25 ~ 30,-1 27(-1 47 to -1 08)

Australia,Prevalence,30 ~ 35,-1 23(-1 37 to -1 08)

Australia,Prevalence,35 ~ 40,-1 17(-1 29 to -1 06)

Australia,Prevalence,40 ~ 45,-1 15(-1 25 to -1 06)

Australia,Prevalence,45 ~ 50,-1 18(-1 25 to -1 1)

Australia,Prevalence,50 ~ 55,-1 2(-1 26 to -1 14)

Australia,Prevalence,55 ~ 60,-1 19(-1 24 to -1 14)

Australia,Prevalence,60 ~ 65,-1 14(-1 18 to -1 1)

Australia,Prevalence,65 ~ 70,-1 07(-1 1 to -1 03)

Australia,Prevalence,70 ~ 75,-0 96(-0 99 to -0 93)

Australia,Prevalence,75 ~ 80,-0 82(-0 86 to -0 78)

Australia,Prevalence,80 ~ 85,-0 66(-0 7 to -0 62)

Australia,Prevalence,85 ~ 90,-0 5(-0 57 to -0 44)

Australia,Prevalence,90 ~ 95,-0 37(-0 49 to -0 25)

Australia,Prevalence,20 ~ 25,-1 28(-1 56 to -1)

Australia,Prevalence,25 ~ 30,-1 27(-1 47 to -1 08)

Australia,Prevalence,30 ~ 35,-1 23(-1 37 to -1 08)

Australia,Prevalence,35 ~ 40,-1 17(-1 29 to -1 06)

Australia,Prevalence,40 ~ 45,-1 15(-1 25 to -1 06)

Australia,Prevalence,45 ~ 50,-1 18(-1 25 to -1 1)

Australia,Prevalence,50 ~ 55,-1 2(-1 26 to -1 14)

Australia,Prevalence,55 ~ 60,-1 19(-1 24 to -1 14)

Australia,Prevalence,60 ~ 65,-1 14(-1 18 to -1 1)

Australia,Prevalence,65 ~ 70,-1 07(-1 1 to -1 03)

Australia,Prevalence,70 ~ 75,-0 96(-0 99 to -0 93)

Australia,Prevalence,75 ~ 80,-0 82(-0 86 to -0 78)

Australia,Prevalence,80 ~ 85,-0 66(-0 7 to -0 62)

Australia,Prevalence,85 ~ 90,-0 5(-0 57 to -0 44)

Australia,Prevalence,90 ~ 95,-0 37(-0 49 to -0 25)

Australia,Prevalence,20 ~ 25,-1 28(-1 56 to -1)

Australia,Prevalence,25 ~ 30,-1 27(-1 47 to -1 08)

Australia,Prevalence,30 ~ 35,-1 23(-1 37 to -1 08)

Australia,Prevalence,35 ~ 40,-1 17(-1 29 to -1 06)

Australia,Prevalence,40 ~ 45,-1 15(-1 25 to -1 06)

Australia,Prevalence,45 ~ 50,-1 18(-1 25 to -1 1)

Australia,Prevalence,50 ~ 55,-1 2(-1 26 to -1 14)

Australia,Prevalence,55 ~ 60,-1 19(-1 24 to -1 14)

Australia,Prevalence,60 ~ 65,-1 14(-1 18 to -1 1)

Australia,Prevalence,65 ~ 70,-1 07(-1 1 to -1 03)

Australia,Prevalence,70 ~ 75,-0 96(-0 99 to -0 93)

Australia,Prevalence,75 ~ 80,-0 82(-0 86 to -0 78)

Australia,Prevalence,80 ~ 85,-0 66(-0 7 to -0 62)

Australia,Prevalence,85 ~ 90,-0 5(-0 57 to -0 44)

Australia,Prevalence,90 ~ 95,-0 37(-0 49 to -0 25)

Australia,Deaths,20 ~ 25,-1 25(-6 5 to 4 29)

Australia,Deaths,25 ~ 30,-0 99(-4 64 to 2 8)

Australia,Deaths,30 ~ 35,-0 69(-3 33 to 2 02)

Australia,Deaths,35 ~ 40,-0 35(-2 17 to 1 5)

Australia,Deaths,40 ~ 45,-0 22(-1 4 to 0 97)

Australia,Deaths,45 ~ 50,-0 38(-1 1 to 0 35)

Australia,Deaths,50 ~ 55,-1 04(-1 51 to -0 56)

Australia,Deaths,55 ~ 60,-1 7(-2 02 to -1 39)

Australia,Deaths,60 ~ 65,-2 35(-2 57 to -2 12)

Australia,Deaths,65 ~ 70,-2 64(-2 81 to -2 47)

Australia,Deaths,70 ~ 75,-2 45(-2 58 to -2 31)

Australia,Deaths,75 ~ 80,-2 03(-2 16 to -1 91)

Australia,Deaths,80 ~ 85,-1 31(-1 44 to -1 18)

Australia,Deaths,85 ~ 90,-0 43(-0 59 to -0 26)

Australia,Deaths,90 ~ 95,0 42(0 14 to 0 71)

Australia,Deaths,20 ~ 25,-1 25(-6 5 to 4 29)

Australia,Deaths,25 ~ 30,-0 99(-4 64 to 2 8)

Australia,Deaths,30 ~ 35,-0 69(-3 33 to 2 02)

Australia,Deaths,35 ~ 40,-0 35(-2 17 to 1 5)

Australia,Deaths,40 ~ 45,-0 22(-1 4 to 0 97)

Australia,Deaths,45 ~ 50,-0 38(-1 1 to 0 35)

Australia,Deaths,50 ~ 55,-1 04(-1 51 to -0 56)

Australia,Deaths,55 ~ 60,-1 7(-2 02 to -1 39)

Australia,Deaths,60 ~ 65,-2 35(-2 57 to -2 12)

Australia,Deaths,65 ~ 70,-2 64(-2 81 to -2 47)

Australia,Deaths,70 ~ 75,-2 45(-2 58 to -2 31)

Australia,Deaths,75 ~ 80,-2 03(-2 16 to -1 91)

Australia,Deaths,80 ~ 85,-1 31(-1 44 to -1 18)

Australia,Deaths,85 ~ 90,-0 43(-0 59 to -0 26)

Australia,Deaths,90 ~ 95,0 42(0 14 to 0 71)

Australia,Deaths,20 ~ 25,-1 25(-6 5 to 4 29)

Australia,Deaths,25 ~ 30,-0 99(-4 64 to 2 8)

Australia,Deaths,30 ~ 35,-0 69(-3 33 to 2 02)

Australia,Deaths,35 ~ 40,-0 35(-2 17 to 1 5)

Australia,Deaths,40 ~ 45,-0 22(-1 4 to 0 97)

Australia,Deaths,45 ~ 50,-0 38(-1 1 to 0 35)

Australia,Deaths,50 ~ 55,-1 04(-1 51 to -0 56)

Australia,Deaths,55 ~ 60,-1 7(-2 02 to -1 39)

Australia,Deaths,60 ~ 65,-2 35(-2 57 to -2 12)

Australia,Deaths,65 ~ 70,-2 64(-2 81 to -2 47)

Australia,Deaths,70 ~ 75,-2 45(-2 58 to -2 31)

Australia,Deaths,75 ~ 80,-2 03(-2 16 to -1 91)

Australia,Deaths,80 ~ 85,-1 31(-1 44 to -1 18)

Australia,Deaths,85 ~ 90,-0 43(-0 59 to -0 26)

Australia,Deaths,90 ~ 95,0 42(0 14 to 0 71)

Australia,Deaths,20 ~ 25,-1 25(-6 5 to 4 29)

Australia,Deaths,25 ~ 30,-0 99(-4 64 to 2 8)

Australia,Deaths,30 ~ 35,-0 69(-3 33 to 2 02)

Australia,Deaths,35 ~ 40,-0 35(-2 17 to 1 5)

Australia,Deaths,40 ~ 45,-0 22(-1 4 to 0 97)

Australia,Deaths,45 ~ 50,-0 38(-1 1 to 0 35)

Australia,Deaths,50 ~ 55,-1 04(-1 51 to -0 56)

Australia,Deaths,55 ~ 60,-1 7(-2 02 to -1 39)

Australia,Deaths,60 ~ 65,-2 35(-2 57 to -2 12)

Australia,Deaths,65 ~ 70,-2 64(-2 81 to -2 47)

Australia,Deaths,70 ~ 75,-2 45(-2 58 to -2 31)

Australia,Deaths,75 ~ 80,-2 03(-2 16 to -1 91)

Australia,Deaths,80 ~ 85,-1 31(-1 44 to -1 18)

Australia,Deaths,85 ~ 90,-0 43(-0 59 to -0 26)

Australia,Deaths,90 ~ 95,0 42(0 14 to 0 71)

Australia,DALYs,20 ~ 25,-1 34(-2 78 to 0 12)

Australia,DALYs,25 ~ 30,-1 2(-2 2 to -0 18)

Australia,DALYs,30 ~ 35,-0 94(-1 7 to -0 17)

Australia,DALYs,35 ~ 40,-0 64(-1 22 to -0 06)

Australia,DALYs,40 ~ 45,-0 51(-0 94 to -0 08)

Australia,DALYs,45 ~ 50,-0 63(-0 93 to -0 32)

Australia,DALYs,50 ~ 55,-1 07(-1 29 to -0 85)

Australia,DALYs,55 ~ 60,-1 56(-1 73 to -1 4)

Australia,DALYs,60 ~ 65,-2 07(-2 19 to -1 94)

Australia,DALYs,65 ~ 70,-2 32(-2 42 to -2 21)

Australia,DALYs,70 ~ 75,-2 18(-2 28 to -2 09)

Australia,DALYs,75 ~ 80,-1 85(-1 94 to -1 75)

Australia,DALYs,80 ~ 85,-1 26(-1 37 to -1 14)

Australia,DALYs,85 ~ 90,-0 52(-0 68 to -0 35)

Australia,DALYs,90 ~ 95,0 22(-0 09 to 0 52)

Australia,DALYs,20 ~ 25,-1 34(-2 78 to 0 12)

Australia,DALYs,25 ~ 30,-1 2(-2 2 to -0 18)

Australia,DALYs,30 ~ 35,-0 94(-1 7 to -0 17)

Australia,DALYs,35 ~ 40,-0 64(-1 22 to -0 06)

Australia,DALYs,40 ~ 45,-0 51(-0 94 to -0 08)

Australia,DALYs,45 ~ 50,-0 63(-0 93 to -0 32)

Australia,DALYs,50 ~ 55,-1 07(-1 29 to -0 85)

Australia,DALYs,55 ~ 60,-1 56(-1 73 to -1 4)

Australia,DALYs,60 ~ 65,-2 07(-2 19 to -1 94)

Australia,DALYs,65 ~ 70,-2 32(-2 42 to -2 21)

Australia,DALYs,70 ~ 75,-2 18(-2 28 to -2 09)

Australia,DALYs,75 ~ 80,-1 85(-1 94 to -1 75)

Australia,DALYs,80 ~ 85,-1 26(-1 37 to -1 14)

Australia,DALYs,85 ~ 90,-0 52(-0 68 to -0 35)

Australia,DALYs,90 ~ 95,0 22(-0 09 to 0 52)

Australia,DALYs,20 ~ 25,-1 34(-2 78 to 0 12)

Australia,DALYs,25 ~ 30,-1 2(-2 2 to -0 18)

Australia,DALYs,30 ~ 35,-0 94(-1 7 to -0 17)

Australia,DALYs,35 ~ 40,-0 64(-1 22 to -0 06)

Australia,DALYs,40 ~ 45,-0 51(-0 94 to -0 08)

Australia,DALYs,45 ~ 50,-0 63(-0 93 to -0 32)

Australia,DALYs,50 ~ 55,-1 07(-1 29 to -0 85)

Australia,DALYs,55 ~ 60,-1 56(-1 73 to -1 4)

Australia,DALYs,60 ~ 65,-2 07(-2 19 to -1 94)

Australia,DALYs,65 ~ 70,-2 32(-2 42 to -2 21)

Australia,DALYs,70 ~ 75,-2 18(-2 28 to -2 09)

Australia,DALYs,75 ~ 80,-1 85(-1 94 to -1 75)

Australia,DALYs,80 ~ 85,-1 26(-1 37 to -1 14)

Australia,DALYs,85 ~ 90,-0 52(-0 68 to -0 35)

Australia,DALYs,90 ~ 95,0 22(-0 09 to 0 52)

Australia,DALYs,20 ~ 25,-1 34(-2 78 to 0 12)

Australia,DALYs,25 ~ 30,-1 2(-2 2 to -0 18)

Australia,DALYs,30 ~ 35,-0 94(-1 7 to -0 17)

Australia,DALYs,35 ~ 40,-0 64(-1 22 to -0 06)

Australia,DALYs,40 ~ 45,-0 51(-0 94 to -0 08)

Australia,DALYs,45 ~ 50,-0 63(-0 93 to -0 32)

Australia,DALYs,50 ~ 55,-1 07(-1 29 to -0 85)

Australia,DALYs,55 ~ 60,-1 56(-1 73 to -1 4)

Australia,DALYs,60 ~ 65,-2 07(-2 19 to -1 94)

Australia,DALYs,65 ~ 70,-2 32(-2 42 to -2 21)

Australia,DALYs,70 ~ 75,-2 18(-2 28 to -2 09)

Australia,DALYs,75 ~ 80,-1 85(-1 94 to -1 75)

Australia,DALYs,80 ~ 85,-1 26(-1 37 to -1 14)

Australia,DALYs,85 ~ 90,-0 52(-0 68 to -0 35)

Australia,DALYs,90 ~ 95,0 22(-0 09 to 0 52)

Austria,Prevalence,20 ~ 25,0 15(-0 07 to 0 37)

Austria,Prevalence,25 ~ 30,0 06(-0 09 to 0 2)

Austria,Prevalence,30 ~ 35,-0 07(-0 19 to 0 04)

Austria,Prevalence,35 ~ 40,-0 21(-0 31 to -0 11)

Austria,Prevalence,40 ~ 45,-0 33(-0 41 to -0 25)

Austria,Prevalence,45 ~ 50,-0 4(-0 47 to -0 34)

Austria,Prevalence,50 ~ 55,-0 37(-0 43 to -0 32)

Austria,Prevalence,55 ~ 60,-0 29(-0 34 to -0 24)

Austria,Prevalence,60 ~ 65,-0 2(-0 24 to -0 16)

Austria,Prevalence,65 ~ 70,-0 11(-0 15 to -0 07)

Austria,Prevalence,70 ~ 75,-0 04(-0 07 to 0)

Austria,Prevalence,75 ~ 80,0 03(-0 01 to 0 07)

Austria,Prevalence,80 ~ 85,0 08(0 04 to 0 13)

Austria,Prevalence,85 ~ 90,0 13(0 08 to 0 18)

Austria,Prevalence,90 ~ 95,0 15(0 05 to 0 24)

Austria,Prevalence,20 ~ 25,0 15(-0 07 to 0 37)

Austria,Prevalence,25 ~ 30,0 06(-0 09 to 0 2)

Austria,Prevalence,30 ~ 35,-0 07(-0 19 to 0 04)

Austria,Prevalence,35 ~ 40,-0 21(-0 31 to -0 11)

Austria,Prevalence,40 ~ 45,-0 33(-0 41 to -0 25)

Austria,Prevalence,45 ~ 50,-0 4(-0 47 to -0 34)

Austria,Prevalence,50 ~ 55,-0 37(-0 43 to -0 32)

Austria,Prevalence,55 ~ 60,-0 29(-0 34 to -0 24)

Austria,Prevalence,60 ~ 65,-0 2(-0 24 to -0 16)

Austria,Prevalence,65 ~ 70,-0 11(-0 15 to -0 07)

Austria,Prevalence,70 ~ 75,-0 04(-0 07 to 0)

Austria,Prevalence,75 ~ 80,0 03(-0 01 to 0 07)

Austria,Prevalence,80 ~ 85,0 08(0 04 to 0 13)

Austria,Prevalence,85 ~ 90,0 13(0 08 to 0 18)

Austria,Prevalence,90 ~ 95,0 15(0 05 to 0 24)

Austria,Prevalence,20 ~ 25,0 15(-0 07 to 0 37)

Austria,Prevalence,25 ~ 30,0 06(-0 09 to 0 2)

Austria,Prevalence,30 ~ 35,-0 07(-0 19 to 0 04)

Austria,Prevalence,35 ~ 40,-0 21(-0 31 to -0 11)

Austria,Prevalence,40 ~ 45,-0 33(-0 41 to -0 25)

Austria,Prevalence,45 ~ 50,-0 4(-0 47 to -0 34)

Austria,Prevalence,50 ~ 55,-0 37(-0 43 to -0 32)

Austria,Prevalence,55 ~ 60,-0 29(-0 34 to -0 24)

Austria,Prevalence,60 ~ 65,-0 2(-0 24 to -0 16)

Austria,Prevalence,65 ~ 70,-0 11(-0 15 to -0 07)

Austria,Prevalence,70 ~ 75,-0 04(-0 07 to 0)

Austria,Prevalence,75 ~ 80,0 03(-0 01 to 0 07)

Austria,Prevalence,80 ~ 85,0 08(0 04 to 0 13)

Austria,Prevalence,85 ~ 90,0 13(0 08 to 0 18)

Austria,Prevalence,90 ~ 95,0 15(0 05 to 0 24)

Austria,Prevalence,20 ~ 25,0 15(-0 07 to 0 37)

Austria,Prevalence,25 ~ 30,0 06(-0 09 to 0 2)

Austria,Prevalence,30 ~ 35,-0 07(-0 19 to 0 04)

Austria,Prevalence,35 ~ 40,-0 21(-0 31 to -0 11)

Austria,Prevalence,40 ~ 45,-0 33(-0 41 to -0 25)

Austria,Prevalence,45 ~ 50,-0 4(-0 47 to -0 34)

Austria,Prevalence,50 ~ 55,-0 37(-0 43 to -0 32)

Austria,Prevalence,55 ~ 60,-0 29(-0 34 to -0 24)

Austria,Prevalence,60 ~ 65,-0 2(-0 24 to -0 16)

Austria,Prevalence,65 ~ 70,-0 11(-0 15 to -0 07)

Austria,Prevalence,70 ~ 75,-0 04(-0 07 to 0)

Austria,Prevalence,75 ~ 80,0 03(-0 01 to 0 07)

Austria,Prevalence,80 ~ 85,0 08(0 04 to 0 13)

Austria,Prevalence,85 ~ 90,0 13(0 08 to 0 18)

Austria,Prevalence,90 ~ 95,0 15(0 05 to 0 24)

Austria,Deaths,20 ~ 25,-0 77(-8 89 to 8 08)

Austria,Deaths,25 ~ 30,-0 77(-6 33 to 5 12)

Austria,Deaths,30 ~ 35,-0 94(-5 04 to 3 33)

Austria,Deaths,35 ~ 40,-1 35(-4 38 to 1 77)

Austria,Deaths,40 ~ 45,-1 67(-3 77 to 0 48)

Austria,Deaths,45 ~ 50,-1 39(-2 71 to -0 05)

Austria,Deaths,50 ~ 55,-0 31(-1 09 to 0 47)

Austria,Deaths,55 ~ 60,0 45(-0 06 to 0 96)

Austria,Deaths,60 ~ 65,0 81(0 44 to 1 18)

Austria,Deaths,65 ~ 70,0 85(0 56 to 1 13)

Austria,Deaths,70 ~ 75,0 81(0 58 to 1 05)

Austria,Deaths,75 ~ 80,0 14(-0 07 to 0 36)

Austria,Deaths,80 ~ 85,-0 4(-0 61 to -0 19)

Austria,Deaths,85 ~ 90,-0 26(-0 51 to 0)

Austria,Deaths,90 ~ 95,-0 05(-0 47 to 0 38)

Austria,Deaths,20 ~ 25,-0 77(-8 89 to 8 08)

Austria,Deaths,25 ~ 30,-0 77(-6 33 to 5 12)

Austria,Deaths,30 ~ 35,-0 94(-5 04 to 3 33)

Austria,Deaths,35 ~ 40,-1 35(-4 38 to 1 77)

Austria,Deaths,40 ~ 45,-1 67(-3 77 to 0 48)

Austria,Deaths,45 ~ 50,-1 39(-2 71 to -0 05)

Austria,Deaths,50 ~ 55,-0 31(-1 09 to 0 47)

Austria,Deaths,55 ~ 60,0 45(-0 06 to 0 96)

Austria,Deaths,60 ~ 65,0 81(0 44 to 1 18)

Austria,Deaths,65 ~ 70,0 85(0 56 to 1 13)

Austria,Deaths,70 ~ 75,0 81(0 58 to 1 05)

Austria,Deaths,75 ~ 80,0 14(-0 07 to 0 36)

Austria,Deaths,80 ~ 85,-0 4(-0 61 to -0 19)

Austria,Deaths,85 ~ 90,-0 26(-0 51 to 0)

Austria,Deaths,90 ~ 95,-0 05(-0 47 to 0 38)

Austria,Deaths,20 ~ 25,-0 77(-8 89 to 8 08)

Austria,Deaths,25 ~ 30,-0 77(-6 33 to 5 12)

Austria,Deaths,30 ~ 35,-0 94(-5 04 to 3 33)

Austria,Deaths,35 ~ 40,-1 35(-4 38 to 1 77)

Austria,Deaths,40 ~ 45,-1 67(-3 77 to 0 48)

Austria,Deaths,45 ~ 50,-1 39(-2 71 to -0 05)

Austria,Deaths,50 ~ 55,-0 31(-1 09 to 0 47)

Austria,Deaths,55 ~ 60,0 45(-0 06 to 0 96)

Austria,Deaths,60 ~ 65,0 81(0 44 to 1 18)

Austria,Deaths,65 ~ 70,0 85(0 56 to 1 13)

Austria,Deaths,70 ~ 75,0 81(0 58 to 1 05)

Austria,Deaths,75 ~ 80,0 14(-0 07 to 0 36)

Austria,Deaths,80 ~ 85,-0 4(-0 61 to -0 19)

Austria,Deaths,85 ~ 90,-0 26(-0 51 to 0)

Austria,Deaths,90 ~ 95,-0 05(-0 47 to 0 38)

Austria,Deaths,20 ~ 25,-0 77(-8 89 to 8 08)

Austria,Deaths,25 ~ 30,-0 77(-6 33 to 5 12)

Austria,Deaths,30 ~ 35,-0 94(-5 04 to 3 33)

Austria,Deaths,35 ~ 40,-1 35(-4 38 to 1 77)

Austria,Deaths,40 ~ 45,-1 67(-3 77 to 0 48)

Austria,Deaths,45 ~ 50,-1 39(-2 71 to -0 05)

Austria,Deaths,50 ~ 55,-0 31(-1 09 to 0 47)

Austria,Deaths,55 ~ 60,0 45(-0 06 to 0 96)

Austria,Deaths,60 ~ 65,0 81(0 44 to 1 18)

Austria,Deaths,65 ~ 70,0 85(0 56 to 1 13)

Austria,Deaths,70 ~ 75,0 81(0 58 to 1 05)

Austria,Deaths,75 ~ 80,0 14(-0 07 to 0 36)

Austria,Deaths,80 ~ 85,-0 4(-0 61 to -0 19)

Austria,Deaths,85 ~ 90,-0 26(-0 51 to 0)

Austria,Deaths,90 ~ 95,-0 05(-0 47 to 0 38)

Austria,DALYs,20 ~ 25,0 04(-1 07 to 1 17)

Austria,DALYs,25 ~ 30,-0 03(-0 78 to 0 74)

Austria,DALYs,30 ~ 35,-0 23(-0 82 to 0 37)

Austria,DALYs,35 ~ 40,-0 51(-1 to -0 03)

Austria,DALYs,40 ~ 45,-0 82(-1 21 to -0 43)

Austria,DALYs,45 ~ 50,-0 81(-1 1 to -0 52)

Austria,DALYs,50 ~ 55,-0 23(-0 44 to -0 02)

Austria,DALYs,55 ~ 60,0 28(0 12 to 0 44)

Austria,DALYs,60 ~ 65,0 54(0 41 to 0 67)

Austria,DALYs,65 ~ 70,0 59(0 48 to 0 71)

Austria,DALYs,70 ~ 75,0 58(0 47 to 0 68)

Austria,DALYs,75 ~ 80,0 13(0 02 to 0 24)

Austria,DALYs,80 ~ 85,-0 26(-0 37 to -0 14)

Austria,DALYs,85 ~ 90,-0 16(-0 32 to -0 01)

Austria,DALYs,90 ~ 95,0 02(-0 26 to 0 29)

Austria,DALYs,20 ~ 25,0 04(-1 07 to 1 17)

Austria,DALYs,25 ~ 30,-0 03(-0 78 to 0 74)

Austria,DALYs,30 ~ 35,-0 23(-0 82 to 0 37)

Austria,DALYs,35 ~ 40,-0 51(-1 to -0 03)

Austria,DALYs,40 ~ 45,-0 82(-1 21 to -0 43)

Austria,DALYs,45 ~ 50,-0 81(-1 1 to -0 52)

Austria,DALYs,50 ~ 55,-0 23(-0 44 to -0 02)

Austria,DALYs,55 ~ 60,0 28(0 12 to 0 44)

Austria,DALYs,60 ~ 65,0 54(0 41 to 0 67)

Austria,DALYs,65 ~ 70,0 59(0 48 to 0 71)

Austria,DALYs,70 ~ 75,0 58(0 47 to 0 68)

Austria,DALYs,75 ~ 80,0 13(0 02 to 0 24)

Austria,DALYs,80 ~ 85,-0 26(-0 37 to -0 14)

Austria,DALYs,85 ~ 90,-0 16(-0 32 to -0 01)

Austria,DALYs,90 ~ 95,0 02(-0 26 to 0 29)

Austria,DALYs,20 ~ 25,0 04(-1 07 to 1 17)

Austria,DALYs,25 ~ 30,-0 03(-0 78 to 0 74)

Austria,DALYs,30 ~ 35,-0 23(-0 82 to 0 37)

Austria,DALYs,35 ~ 40,-0 51(-1 to -0 03)

Austria,DALYs,40 ~ 45,-0 82(-1 21 to -0 43)

Austria,DALYs,45 ~ 50,-0 81(-1 1 to -0 52)

Austria,DALYs,50 ~ 55,-0 23(-0 44 to -0 02)

Austria,DALYs,55 ~ 60,0 28(0 12 to 0 44)

Austria,DALYs,60 ~ 65,0 54(0 41 to 0 67)

Austria,DALYs,65 ~ 70,0 59(0 48 to 0 71)

Austria,DALYs,70 ~ 75,0 58(0 47 to 0 68)

Austria,DALYs,75 ~ 80,0 13(0 02 to 0 24)

Austria,DALYs,80 ~ 85,-0 26(-0 37 to -0 14)

Austria,DALYs,85 ~ 90,-0 16(-0 32 to -0 01)

Austria,DALYs,90 ~ 95,0 02(-0 26 to 0 29)

Austria,DALYs,20 ~ 25,0 04(-1 07 to 1 17)

Austria,DALYs,25 ~ 30,-0 03(-0 78 to 0 74)

Austria,DALYs,30 ~ 35,-0 23(-0 82 to 0 37)

Austria,DALYs,35 ~ 40,-0 51(-1 to -0 03)

Austria,DALYs,40 ~ 45,-0 82(-1 21 to -0 43)

Austria,DALYs,45 ~ 50,-0 81(-1 1 to -0 52)

Austria,DALYs,50 ~ 55,-0 23(-0 44 to -0 02)

Austria,DALYs,55 ~ 60,0 28(0 12 to 0 44)

Austria,DALYs,60 ~ 65,0 54(0 41 to 0 67)

Austria,DALYs,65 ~ 70,0 59(0 48 to 0 71)

Austria,DALYs,70 ~ 75,0 58(0 47 to 0 68)

Austria,DALYs,75 ~ 80,0 13(0 02 to 0 24)

Austria,DALYs,80 ~ 85,-0 26(-0 37 to -0 14)

Austria,DALYs,85 ~ 90,-0 16(-0 32 to -0 01)

Austria,DALYs,90 ~ 95,0 02(-0 26 to 0 29)

Cyprus,Prevalence,20 ~ 25,-0 02(-0 32 to 0 27)

Cyprus,Prevalence,25 ~ 30,-0 04(-0 23 to 0 16)

Cyprus,Prevalence,30 ~ 35,-0 09(-0 24 to 0 07)

Cyprus,Prevalence,35 ~ 40,-0 17(-0 3 to -0 03)

Cyprus,Prevalence,40 ~ 45,-0 27(-0 39 to -0 15)

Cyprus,Prevalence,45 ~ 50,-0 39(-0 49 to -0 28)

Cyprus,Prevalence,50 ~ 55,-0 47(-0 56 to -0 38)

Cyprus,Prevalence,55 ~ 60,-0 5(-0 58 to -0 42)

Cyprus,Prevalence,60 ~ 65,-0 46(-0 53 to -0 39)

Cyprus,Prevalence,65 ~ 70,-0 39(-0 46 to -0 33)

Cyprus,Prevalence,70 ~ 75,-0 31(-0 37 to -0 25)

Cyprus,Prevalence,75 ~ 80,-0 21(-0 28 to -0 15)

Cyprus,Prevalence,80 ~ 85,-0 08(-0 17 to 0 01)

Cyprus,Prevalence,85 ~ 90,0 09(-0 11 to 0 29)

Cyprus,Prevalence,90 ~ 95,0 26(-0 15 to 0 67)

Cyprus,Prevalence,20 ~ 25,-0 02(-0 32 to 0 27)

Cyprus,Prevalence,25 ~ 30,-0 04(-0 23 to 0 16)

Cyprus,Prevalence,30 ~ 35,-0 09(-0 24 to 0 07)

Cyprus,Prevalence,35 ~ 40,-0 17(-0 3 to -0 03)

Cyprus,Prevalence,40 ~ 45,-0 27(-0 39 to -0 15)

Cyprus,Prevalence,45 ~ 50,-0 39(-0 49 to -0 28)

Cyprus,Prevalence,50 ~ 55,-0 47(-0 56 to -0 38)

Cyprus,Prevalence,55 ~ 60,-0 5(-0 58 to -0 42)

Cyprus,Prevalence,60 ~ 65,-0 46(-0 53 to -0 39)

Cyprus,Prevalence,65 ~ 70,-0 39(-0 46 to -0 33)

Cyprus,Prevalence,70 ~ 75,-0 31(-0 37 to -0 25)

Cyprus,Prevalence,75 ~ 80,-0 21(-0 28 to -0 15)

Cyprus,Prevalence,80 ~ 85,-0 08(-0 17 to 0 01)

Cyprus,Prevalence,85 ~ 90,0 09(-0 11 to 0 29)

Cyprus,Prevalence,90 ~ 95,0 26(-0 15 to 0 67)

Cyprus,Prevalence,20 ~ 25,-0 02(-0 32 to 0 27)

Cyprus,Prevalence,25 ~ 30,-0 04(-0 23 to 0 16)

Cyprus,Prevalence,30 ~ 35,-0 09(-0 24 to 0 07)

Cyprus,Prevalence,35 ~ 40,-0 17(-0 3 to -0 03)

Cyprus,Prevalence,40 ~ 45,-0 27(-0 39 to -0 15)

Cyprus,Prevalence,45 ~ 50,-0 39(-0 49 to -0 28)

Cyprus,Prevalence,50 ~ 55,-0 47(-0 56 to -0 38)

Cyprus,Prevalence,55 ~ 60,-0 5(-0 58 to -0 42)

Cyprus,Prevalence,60 ~ 65,-0 46(-0 53 to -0 39)

Cyprus,Prevalence,65 ~ 70,-0 39(-0 46 to -0 33)

Cyprus,Prevalence,70 ~ 75,-0 31(-0 37 to -0 25)

Cyprus,Prevalence,75 ~ 80,-0 21(-0 28 to -0 15)

Cyprus,Prevalence,80 ~ 85,-0 08(-0 17 to 0 01)

Cyprus,Prevalence,85 ~ 90,0 09(-0 11 to 0 29)

Cyprus,Prevalence,90 ~ 95,0 26(-0 15 to 0 67)

Cyprus,Prevalence,20 ~ 25,-0 02(-0 32 to 0 27)

Cyprus,Prevalence,25 ~ 30,-0 04(-0 23 to 0 16)

Cyprus,Prevalence,30 ~ 35,-0 09(-0 24 to 0 07)

Cyprus,Prevalence,35 ~ 40,-0 17(-0 3 to -0 03)

Cyprus,Prevalence,40 ~ 45,-0 27(-0 39 to -0 15)

Cyprus,Prevalence,45 ~ 50,-0 39(-0 49 to -0 28)

Cyprus,Prevalence,50 ~ 55,-0 47(-0 56 to -0 38)

Cyprus,Prevalence,55 ~ 60,-0 5(-0 58 to -0 42)

Cyprus,Prevalence,60 ~ 65,-0 46(-0 53 to -0 39)

Cyprus,Prevalence,65 ~ 70,-0 39(-0 46 to -0 33)

Cyprus,Prevalence,70 ~ 75,-0 31(-0 37 to -0 25)

Cyprus,Prevalence,75 ~ 80,-0 21(-0 28 to -0 15)

Cyprus,Prevalence,80 ~ 85,-0 08(-0 17 to 0 01)

Cyprus,Prevalence,85 ~ 90,0 09(-0 11 to 0 29)

Cyprus,Prevalence,90 ~ 95,0 26(-0 15 to 0 67)

Cyprus,Deaths,20 ~ 25,-3 06(-21 49 to 19 68)

Cyprus,Deaths,25 ~ 30,-3 65(-16 86 to 11 66)

Cyprus,Deaths,30 ~ 35,-3 54(-13 57 to 7 66)

Cyprus,Deaths,35 ~ 40,-2 81(-10 02 to 4 98)

Cyprus,Deaths,40 ~ 45,-2 58(-7 74 to 2 88)

Cyprus,Deaths,45 ~ 50,-2 36(-5 83 to 1 25)

Cyprus,Deaths,50 ~ 55,-2 41(-4 77 to 0)

Cyprus,Deaths,55 ~ 60,-2 31(-3 88 to -0 71)

Cyprus,Deaths,60 ~ 65,-2 59(-3 71 to -1 47)

Cyprus,Deaths,65 ~ 70,-2 78(-3 63 to -1 93)

Cyprus,Deaths,70 ~ 75,-2 49(-3 16 to -1 82)

Cyprus,Deaths,75 ~ 80,-2 69(-3 24 to -2 14)

Cyprus,Deaths,80 ~ 85,-3 32(-3 81 to -2 83)

Cyprus,Deaths,85 ~ 90,-3 74(-4 47 to -3 01)

Cyprus,Deaths,90 ~ 95,-2 19(-3 73 to -0 63)

Cyprus,Deaths,20 ~ 25,-3 06(-21 49 to 19 68)

Cyprus,Deaths,25 ~ 30,-3 65(-16 86 to 11 66)

Cyprus,Deaths,30 ~ 35,-3 54(-13 57 to 7 66)

Cyprus,Deaths,35 ~ 40,-2 81(-10 02 to 4 98)

Cyprus,Deaths,40 ~ 45,-2 58(-7 74 to 2 88)

Cyprus,Deaths,45 ~ 50,-2 36(-5 83 to 1 25)

Cyprus,Deaths,50 ~ 55,-2 41(-4 77 to 0)

Cyprus,Deaths,55 ~ 60,-2 31(-3 88 to -0 71)

Cyprus,Deaths,60 ~ 65,-2 59(-3 71 to -1 47)

Cyprus,Deaths,65 ~ 70,-2 78(-3 63 to -1 93)

Cyprus,Deaths,70 ~ 75,-2 49(-3 16 to -1 82)

Cyprus,Deaths,75 ~ 80,-2 69(-3 24 to -2 14)

Cyprus,Deaths,80 ~ 85,-3 32(-3 81 to -2 83)

Cyprus,Deaths,85 ~ 90,-3 74(-4 47 to -3 01)

Cyprus,Deaths,90 ~ 95,-2 19(-3 73 to -0 63)

Cyprus,Deaths,20 ~ 25,-3 06(-21 49 to 19 68)

Cyprus,Deaths,25 ~ 30,-3 65(-16 86 to 11 66)

Cyprus,Deaths,30 ~ 35,-3 54(-13 57 to 7 66)

Cyprus,Deaths,35 ~ 40,-2 81(-10 02 to 4 98)

Cyprus,Deaths,40 ~ 45,-2 58(-7 74 to 2 88)

Cyprus,Deaths,45 ~ 50,-2 36(-5 83 to 1 25)

Cyprus,Deaths,50 ~ 55,-2 41(-4 77 to 0)

Cyprus,Deaths,55 ~ 60,-2 31(-3 88 to -0 71)

Cyprus,Deaths,60 ~ 65,-2 59(-3 71 to -1 47)

Cyprus,Deaths,65 ~ 70,-2 78(-3 63 to -1 93)

Cyprus,Deaths,70 ~ 75,-2 49(-3 16 to -1 82)

Cyprus,Deaths,75 ~ 80,-2 69(-3 24 to -2 14)

Cyprus,Deaths,80 ~ 85,-3 32(-3 81 to -2 83)

Cyprus,Deaths,85 ~ 90,-3 74(-4 47 to -3 01)

Cyprus,Deaths,90 ~ 95,-2 19(-3 73 to -0 63)

Cyprus,Deaths,20 ~ 25,-3 06(-21 49 to 19 68)

Cyprus,Deaths,25 ~ 30,-3 65(-16 86 to 11 66)

Cyprus,Deaths,30 ~ 35,-3 54(-13 57 to 7 66)

Cyprus,Deaths,35 ~ 40,-2 81(-10 02 to 4 98)

Cyprus,Deaths,40 ~ 45,-2 58(-7 74 to 2 88)

Cyprus,Deaths,45 ~ 50,-2 36(-5 83 to 1 25)

Cyprus,Deaths,50 ~ 55,-2 41(-4 77 to 0)

Cyprus,Deaths,55 ~ 60,-2 31(-3 88 to -0 71)

Cyprus,Deaths,60 ~ 65,-2 59(-3 71 to -1 47)

Cyprus,Deaths,65 ~ 70,-2 78(-3 63 to -1 93)

Cyprus,Deaths,70 ~ 75,-2 49(-3 16 to -1 82)

Cyprus,Deaths,75 ~ 80,-2 69(-3 24 to -2 14)

Cyprus,Deaths,80 ~ 85,-3 32(-3 81 to -2 83)

Cyprus,Deaths,85 ~ 90,-3 74(-4 47 to -3 01)

Cyprus,Deaths,90 ~ 95,-2 19(-3 73 to -0 63)

Cyprus,DALYs,20 ~ 25,-0 75(-3 05 to 1 6)

Cyprus,DALYs,25 ~ 30,-0 92(-2 48 to 0 66)

Cyprus,DALYs,30 ~ 35,-0 97(-2 19 to 0 26)

Cyprus,DALYs,35 ~ 40,-0 98(-1 99 to 0 03)

Cyprus,DALYs,40 ~ 45,-1 18(-2 02 to -0 34)

Cyprus,DALYs,45 ~ 50,-1 37(-2 05 to -0 69)

Cyprus,DALYs,50 ~ 55,-1 59(-2 12 to -1 06)

Cyprus,DALYs,55 ~ 60,-1 69(-2 1 to -1 27)

Cyprus,DALYs,60 ~ 65,-1 97(-2 3 to -1 64)

Cyprus,DALYs,65 ~ 70,-2 18(-2 46 to -1 89)

Cyprus,DALYs,70 ~ 75,-1 98(-2 23 to -1 74)

Cyprus,DALYs,75 ~ 80,-2 22(-2 45 to -1 99)

Cyprus,DALYs,80 ~ 85,-2 98(-3 23 to -2 74)

Cyprus,DALYs,85 ~ 90,-3 58(-4 to -3 17)

Cyprus,DALYs,90 ~ 95,-2 19(-3 13 to -1 25)

Cyprus,DALYs,20 ~ 25,-0 75(-3 05 to 1 6)

Cyprus,DALYs,25 ~ 30,-0 92(-2 48 to 0 66)

Cyprus,DALYs,30 ~ 35,-0 97(-2 19 to 0 26)

Cyprus,DALYs,35 ~ 40,-0 98(-1 99 to 0 03)

Cyprus,DALYs,40 ~ 45,-1 18(-2 02 to -0 34)

Cyprus,DALYs,45 ~ 50,-1 37(-2 05 to -0 69)

Cyprus,DALYs,50 ~ 55,-1 59(-2 12 to -1 06)

Cyprus,DALYs,55 ~ 60,-1 69(-2 1 to -1 27)

Cyprus,DALYs,60 ~ 65,-1 97(-2 3 to -1 64)

Cyprus,DALYs,65 ~ 70,-2 18(-2 46 to -1 89)

Cyprus,DALYs,70 ~ 75,-1 98(-2 23 to -1 74)

Cyprus,DALYs,75 ~ 80,-2 22(-2 45 to -1 99)

Cyprus,DALYs,80 ~ 85,-2 98(-3 23 to -2 74)

Cyprus,DALYs,85 ~ 90,-3 58(-4 to -3 17)

Cyprus,DALYs,90 ~ 95,-2 19(-3 13 to -1 25)

Cyprus,DALYs,20 ~ 25,-0 75(-3 05 to 1 6)

Cyprus,DALYs,25 ~ 30,-0 92(-2 48 to 0 66)

Cyprus,DALYs,30 ~ 35,-0 97(-2 19 to 0 26)

Cyprus,DALYs,35 ~ 40,-0 98(-1 99 to 0 03)

Cyprus,DALYs,40 ~ 45,-1 18(-2 02 to -0 34)

Cyprus,DALYs,45 ~ 50,-1 37(-2 05 to -0 69)

Cyprus,DALYs,50 ~ 55,-1 59(-2 12 to -1 06)

Cyprus,DALYs,55 ~ 60,-1 69(-2 1 to -1 27)

Cyprus,DALYs,60 ~ 65,-1 97(-2 3 to -1 64)

Cyprus,DALYs,65 ~ 70,-2 18(-2 46 to -1 89)

Cyprus,DALYs,70 ~ 75,-1 98(-2 23 to -1 74)

Cyprus,DALYs,75 ~ 80,-2 22(-2 45 to -1 99)

Cyprus,DALYs,80 ~ 85,-2 98(-3 23 to -2 74)

Cyprus,DALYs,85 ~ 90,-3 58(-4 to -3 17)

Cyprus,DALYs,90 ~ 95,-2 19(-3 13 to -1 25)

Cyprus,DALYs,20 ~ 25,-0 75(-3 05 to 1 6)

Cyprus,DALYs,25 ~ 30,-0 92(-2 48 to 0 66)

Cyprus,DALYs,30 ~ 35,-0 97(-2 19 to 0 26)

Cyprus,DALYs,35 ~ 40,-0 98(-1 99 to 0 03)

Cyprus,DALYs,40 ~ 45,-1 18(-2 02 to -0 34)

Cyprus,DALYs,45 ~ 50,-1 37(-2 05 to -0 69)

Cyprus,DALYs,50 ~ 55,-1 59(-2 12 to -1 06)

Cyprus,DALYs,55 ~ 60,-1 69(-2 1 to -1 27)

Cyprus,DALYs,60 ~ 65,-1 97(-2 3 to -1 64)

Cyprus,DALYs,65 ~ 70,-2 18(-2 46 to -1 89)

Cyprus,DALYs,70 ~ 75,-1 98(-2 23 to -1 74)

Cyprus,DALYs,75 ~ 80,-2 22(-2 45 to -1 99)

Cyprus,DALYs,80 ~ 85,-2 98(-3 23 to -2 74)

Cyprus,DALYs,85 ~ 90,-3 58(-4 to -3 17)

Cyprus,DALYs,90 ~ 95,-2 19(-3 13 to -1 25)

Iran_(Islamic_Republic_of),Prevalence,20 ~ 25,-0 08(-0 2 to 0 04)

Iran_(Islamic_Republic_of),Prevalence,25 ~ 30,-0 06(-0 14 to 0 02)

Iran_(Islamic_Republic_of),Prevalence,30 ~ 35,-0 02(-0 08 to 0 05)

Iran_(Islamic_Republic_of),Prevalence,35 ~ 40,0 03(-0 03 to 0 09)

Iran_(Islamic_Republic_of),Prevalence,40 ~ 45,0 1(0 04 to 0 15)

Iran_(Islamic_Republic_of),Prevalence,45 ~ 50,0 19(0 14 to 0 25)

Iran_(Islamic_Republic_of),Prevalence,50 ~ 55,0 32(0 27 to 0 37)

Iran_(Islamic_Republic_of),Prevalence,55 ~ 60,0 47(0 43 to 0 52)

Iran_(Islamic_Republic_of),Prevalence,60 ~ 65,0 63(0 59 to 0 68)

Iran_(Islamic_Republic_of),Prevalence,65 ~ 70,0 79(0 74 to 0 83)

Iran_(Islamic_Republic_of),Prevalence,70 ~ 75,0 94(0 89 to 0 98)

Iran_(Islamic_Republic_of),Prevalence,75 ~ 80,1 07(1 01 to 1 13)

Iran_(Islamic_Republic_of),Prevalence,80 ~ 85,1 18(1 08 to 1 27)

Iran_(Islamic_Republic_of),Prevalence,85 ~ 90,1 31(1 18 to 1 44)

Iran_(Islamic_Republic_of),Prevalence,90 ~ 95,1 44(1 24 to 1 64)

Iran_(Islamic_Republic_of),Prevalence,20 ~ 25,-0 08(-0 2 to 0 04)

Iran_(Islamic_Republic_of),Prevalence,25 ~ 30,-0 06(-0 14 to 0 02)

Iran_(Islamic_Republic_of),Prevalence,30 ~ 35,-0 02(-0 08 to 0 05)

Iran_(Islamic_Republic_of),Prevalence,35 ~ 40,0 03(-0 03 to 0 09)

Iran_(Islamic_Republic_of),Prevalence,40 ~ 45,0 1(0 04 to 0 15)

Iran_(Islamic_Republic_of),Prevalence,45 ~ 50,0 19(0 14 to 0 25)

Iran_(Islamic_Republic_of),Prevalence,50 ~ 55,0 32(0 27 to 0 37)

Iran_(Islamic_Republic_of),Prevalence,55 ~ 60,0 47(0 43 to 0 52)

Iran_(Islamic_Republic_of),Prevalence,60 ~ 65,0 63(0 59 to 0 68)

Iran_(Islamic_Republic_of),Prevalence,65 ~ 70,0 79(0 74 to 0 83)

Iran_(Islamic_Republic_of),Prevalence,70 ~ 75,0 94(0 89 to 0 98)

Iran_(Islamic_Republic_of),Prevalence,75 ~ 80,1 07(1 01 to 1 13)

Iran_(Islamic_Republic_of),Prevalence,80 ~ 85,1 18(1 08 to 1 27)

Iran_(Islamic_Republic_of),Prevalence,85 ~ 90,1 31(1 18 to 1 44)

Iran_(Islamic_Republic_of),Prevalence,90 ~ 95,1 44(1 24 to 1 64)

Iran_(Islamic_Republic_of),Prevalence,20 ~ 25,-0 08(-0 2 to 0 04)

Iran_(Islamic_Republic_of),Prevalence,25 ~ 30,-0 06(-0 14 to 0 02)

Iran_(Islamic_Republic_of),Prevalence,30 ~ 35,-0 02(-0 08 to 0 05)

Iran_(Islamic_Republic_of),Prevalence,35 ~ 40,0 03(-0 03 to 0 09)

Iran_(Islamic_Republic_of),Prevalence,40 ~ 45,0 1(0 04 to 0 15)

Iran_(Islamic_Republic_of),Prevalence,45 ~ 50,0 19(0 14 to 0 25)

Iran_(Islamic_Republic_of),Prevalence,50 ~ 55,0 32(0 27 to 0 37)

Iran_(Islamic_Republic_of),Prevalence,55 ~ 60,0 47(0 43 to 0 52)

Iran_(Islamic_Republic_of),Prevalence,60 ~ 65,0 63(0 59 to 0 68)

Iran_(Islamic_Republic_of),Prevalence,65 ~ 70,0 79(0 74 to 0 83)

Iran_(Islamic_Republic_of),Prevalence,70 ~ 75,0 94(0 89 to 0 98)

Iran_(Islamic_Republic_of),Prevalence,75 ~ 80,1 07(1 01 to 1 13)

Iran_(Islamic_Republic_of),Prevalence,80 ~ 85,1 18(1 08 to 1 27)

Iran_(Islamic_Republic_of),Prevalence,85 ~ 90,1 31(1 18 to 1 44)

Iran_(Islamic_Republic_of),Prevalence,90 ~ 95,1 44(1 24 to 1 64)

Iran_(Islamic_Republic_of),Prevalence,20 ~ 25,-0 08(-0 2 to 0 04)

Iran_(Islamic_Republic_of),Prevalence,25 ~ 30,-0 06(-0 14 to 0 02)

Iran_(Islamic_Republic_of),Prevalence,30 ~ 35,-0 02(-0 08 to 0 05)

Iran_(Islamic_Republic_of),Prevalence,35 ~ 40,0 03(-0 03 to 0 09)

Iran_(Islamic_Republic_of),Prevalence,40 ~ 45,0 1(0 04 to 0 15)

Iran_(Islamic_Republic_of),Prevalence,45 ~ 50,0 19(0 14 to 0 25)

Iran_(Islamic_Republic_of),Prevalence,50 ~ 55,0 32(0 27 to 0 37)

Iran_(Islamic_Republic_of),Prevalence,55 ~ 60,0 47(0 43 to 0 52)

Iran_(Islamic_Republic_of),Prevalence,60 ~ 65,0 63(0 59 to 0 68)

Iran_(Islamic_Republic_of),Prevalence,65 ~ 70,0 79(0 74 to 0 83)

Iran_(Islamic_Republic_of),Prevalence,70 ~ 75,0 94(0 89 to 0 98)

Iran_(Islamic_Republic_of),Prevalence,75 ~ 80,1 07(1 01 to 1 13)

Iran_(Islamic_Republic_of),Prevalence,80 ~ 85,1 18(1 08 to 1 27)

Iran_(Islamic_Republic_of),Prevalence,85 ~ 90,1 31(1 18 to 1 44)

Iran_(Islamic_Republic_of),Prevalence,90 ~ 95,1 44(1 24 to 1 64)

Iran_(Islamic_Republic_of),Deaths,20 ~ 25,-0 33(-1 3 to 0 66)

Iran_(Islamic_Republic_of),Deaths,25 ~ 30,-0 34(-1 01 to 0 33)

Iran_(Islamic_Republic_of),Deaths,30 ~ 35,-0 36(-0 87 to 0 16)

Iran_(Islamic_Republic_of),Deaths,35 ~ 40,-0 46(-0 88 to -0 03)

Iran_(Islamic_Republic_of),Deaths,40 ~ 45,-0 63(-0 99 to -0 26)

Iran_(Islamic_Republic_of),Deaths,45 ~ 50,-0 81(-1 12 to -0 5)

Iran_(Islamic_Republic_of),Deaths,50 ~ 55,-0 96(-1 21 to -0 71)

Iran_(Islamic_Republic_of),Deaths,55 ~ 60,-0 96(-1 16 to -0 75)

Iran_(Islamic_Republic_of),Deaths,60 ~ 65,-0 86(-1 03 to -0 69)

Iran_(Islamic_Republic_of),Deaths,65 ~ 70,-0 79(-0 93 to -0 64)

Iran_(Islamic_Republic_of),Deaths,70 ~ 75,-0 64(-0 78 to -0 5)

Iran_(Islamic_Republic_of),Deaths,75 ~ 80,-0 37(-0 51 to -0 23)

Iran_(Islamic_Republic_of),Deaths,80 ~ 85,-0 14(-0 33 to 0 06)

Iran_(Islamic_Republic_of),Deaths,85 ~ 90,-0 05(-0 3 to 0 19)

Iran_(Islamic_Republic_of),Deaths,90 ~ 95,0 12(-0 23 to 0 47)

Iran_(Islamic_Republic_of),Deaths,20 ~ 25,-0 33(-1 3 to 0 66)

Iran_(Islamic_Republic_of),Deaths,25 ~ 30,-0 34(-1 01 to 0 33)

Iran_(Islamic_Republic_of),Deaths,30 ~ 35,-0 36(-0 87 to 0 16)

Iran_(Islamic_Republic_of),Deaths,35 ~ 40,-0 46(-0 88 to -0 03)

Iran_(Islamic_Republic_of),Deaths,40 ~ 45,-0 63(-0 99 to -0 26)

Iran_(Islamic_Republic_of),Deaths,45 ~ 50,-0 81(-1 12 to -0 5)

Iran_(Islamic_Republic_of),Deaths,50 ~ 55,-0 96(-1 21 to -0 71)

Iran_(Islamic_Republic_of),Deaths,55 ~ 60,-0 96(-1 16 to -0 75)

Iran_(Islamic_Republic_of),Deaths,60 ~ 65,-0 86(-1 03 to -0 69)

Iran_(Islamic_Republic_of),Deaths,65 ~ 70,-0 79(-0 93 to -0 64)

Iran_(Islamic_Republic_of),Deaths,70 ~ 75,-0 64(-0 78 to -0 5)

Iran_(Islamic_Republic_of),Deaths,75 ~ 80,-0 37(-0 51 to -0 23)

Iran_(Islamic_Republic_of),Deaths,80 ~ 85,-0 14(-0 33 to 0 06)

Iran_(Islamic_Republic_of),Deaths,85 ~ 90,-0 05(-0 3 to 0 19)

Iran_(Islamic_Republic_of),Deaths,90 ~ 95,0 12(-0 23 to 0 47)

Iran_(Islamic_Republic_of),Deaths,20 ~ 25,-0 33(-1 3 to 0 66)

Iran_(Islamic_Republic_of),Deaths,25 ~ 30,-0 34(-1 01 to 0 33)

Iran_(Islamic_Republic_of),Deaths,30 ~ 35,-0 36(-0 87 to 0 16)

Iran_(Islamic_Republic_of),Deaths,35 ~ 40,-0 46(-0 88 to -0 03)

Iran_(Islamic_Republic_of),Deaths,40 ~ 45,-0 63(-0 99 to -0 26)

Iran_(Islamic_Republic_of),Deaths,45 ~ 50,-0 81(-1 12 to -0 5)

Iran_(Islamic_Republic_of),Deaths,50 ~ 55,-0 96(-1 21 to -0 71)

Iran_(Islamic_Republic_of),Deaths,55 ~ 60,-0 96(-1 16 to -0 75)

Iran_(Islamic_Republic_of),Deaths,60 ~ 65,-0 86(-1 03 to -0 69)

Iran_(Islamic_Republic_of),Deaths,65 ~ 70,-0 79(-0 93 to -0 64)

Iran_(Islamic_Republic_of),Deaths,70 ~ 75,-0 64(-0 78 to -0 5)

Iran_(Islamic_Republic_of),Deaths,75 ~ 80,-0 37(-0 51 to -0 23)

Iran_(Islamic_Republic_of),Deaths,80 ~ 85,-0 14(-0 33 to 0 06)

Iran_(Islamic_Republic_of),Deaths,85 ~ 90,-0 05(-0 3 to 0 19)

Iran_(Islamic_Republic_of),Deaths,90 ~ 95,0 12(-0 23 to 0 47)

Iran_(Islamic_Republic_of),Deaths,20 ~ 25,-0 33(-1 3 to 0 66)

Iran_(Islamic_Republic_of),Deaths,25 ~ 30,-0 34(-1 01 to 0 33)

Iran_(Islamic_Republic_of),Deaths,30 ~ 35,-0 36(-0 87 to 0 16)

Iran_(Islamic_Republic_of),Deaths,35 ~ 40,-0 46(-0 88 to -0 03)

Iran_(Islamic_Republic_of),Deaths,40 ~ 45,-0 63(-0 99 to -0 26)

Iran_(Islamic_Republic_of),Deaths,45 ~ 50,-0 81(-1 12 to -0 5)

Iran_(Islamic_Republic_of),Deaths,50 ~ 55,-0 96(-1 21 to -0 71)

Iran_(Islamic_Republic_of),Deaths,55 ~ 60,-0 96(-1 16 to -0 75)

Iran_(Islamic_Republic_of),Deaths,60 ~ 65,-0 86(-1 03 to -0 69)

Iran_(Islamic_Republic_of),Deaths,65 ~ 70,-0 79(-0 93 to -0 64)

Iran_(Islamic_Republic_of),Deaths,70 ~ 75,-0 64(-0 78 to -0 5)

Iran_(Islamic_Republic_of),Deaths,75 ~ 80,-0 37(-0 51 to -0 23)

Iran_(Islamic_Republic_of),Deaths,80 ~ 85,-0 14(-0 33 to 0 06)

Iran_(Islamic_Republic_of),Deaths,85 ~ 90,-0 05(-0 3 to 0 19)

Iran_(Islamic_Republic_of),Deaths,90 ~ 95,0 12(-0 23 to 0 47)

Iran_(Islamic_Republic_of),DALYs,20 ~ 25,-0 15(-0 46 to 0 16)

Iran_(Islamic_Republic_of),DALYs,25 ~ 30,-0 15(-0 37 to 0 06)

Iran_(Islamic_Republic_of),DALYs,30 ~ 35,-0 15(-0 33 to 0 02)

Iran_(Islamic_Republic_of),DALYs,35 ~ 40,-0 21(-0 36 to -0 05)

Iran_(Islamic_Republic_of),DALYs,40 ~ 45,-0 3(-0 44 to -0 16)

Iran_(Islamic_Republic_of),DALYs,45 ~ 50,-0 41(-0 54 to -0 28)

Iran_(Islamic_Republic_of),DALYs,50 ~ 55,-0 51(-0 63 to -0 39)

Iran_(Islamic_Republic_of),DALYs,55 ~ 60,-0 51(-0 62 to -0 41)

Iran_(Islamic_Republic_of),DALYs,60 ~ 65,-0 45(-0 55 to -0 36)

Iran_(Islamic_Republic_of),DALYs,65 ~ 70,-0 39(-0 48 to -0 3)

Iran_(Islamic_Republic_of),DALYs,70 ~ 75,-0 3(-0 4 to -0 21)

Iran_(Islamic_Republic_of),DALYs,75 ~ 80,-0 12(-0 23 to -0 01)

Iran_(Islamic_Republic_of),DALYs,80 ~ 85,0 07(-0 11 to 0 24)

Iran_(Islamic_Republic_of),DALYs,85 ~ 90,0 18(-0 06 to 0 42)

Iran_(Islamic_Republic_of),DALYs,90 ~ 95,0 34(-0 01 to 0 7)

Iran_(Islamic_Republic_of),DALYs,20 ~ 25,-0 15(-0 46 to 0 16)

Iran_(Islamic_Republic_of),DALYs,25 ~ 30,-0 15(-0 37 to 0 06)

Iran_(Islamic_Republic_of),DALYs,30 ~ 35,-0 15(-0 33 to 0 02)

Iran_(Islamic_Republic_of),DALYs,35 ~ 40,-0 21(-0 36 to -0 05)

Iran_(Islamic_Republic_of),DALYs,40 ~ 45,-0 3(-0 44 to -0 16)

Iran_(Islamic_Republic_of),DALYs,45 ~ 50,-0 41(-0 54 to -0 28)

Iran_(Islamic_Republic_of),DALYs,50 ~ 55,-0 51(-0 63 to -0 39)

Iran_(Islamic_Republic_of),DALYs,55 ~ 60,-0 51(-0 62 to -0 41)

Iran_(Islamic_Republic_of),DALYs,60 ~ 65,-0 45(-0 55 to -0 36)

Iran_(Islamic_Republic_of),DALYs,65 ~ 70,-0 39(-0 48 to -0 3)

Iran_(Islamic_Republic_of),DALYs,70 ~ 75,-0 3(-0 4 to -0 21)

Iran_(Islamic_Republic_of),DALYs,75 ~ 80,-0 12(-0 23 to -0 01)

Iran_(Islamic_Republic_of),DALYs,80 ~ 85,0 07(-0 11 to 0 24)

Iran_(Islamic_Republic_of),DALYs,85 ~ 90,0 18(-0 06 to 0 42)

Iran_(Islamic_Republic_of),DALYs,90 ~ 95,0 34(-0 01 to 0 7)

Iran_(Islamic_Republic_of),DALYs,20 ~ 25,-0 15(-0 46 to 0 16)

Iran_(Islamic_Republic_of),DALYs,25 ~ 30,-0 15(-0 37 to 0 06)

Iran_(Islamic_Republic_of),DALYs,30 ~ 35,-0 15(-0 33 to 0 02)

Iran_(Islamic_Republic_of),DALYs,35 ~ 40,-0 21(-0 36 to -0 05)

Iran_(Islamic_Republic_of),DALYs,40 ~ 45,-0 3(-0 44 to -0 16)

Iran_(Islamic_Republic_of),DALYs,45 ~ 50,-0 41(-0 54 to -0 28)

Iran_(Islamic_Republic_of),DALYs,50 ~ 55,-0 51(-0 63 to -0 39)

Iran_(Islamic_Republic_of),DALYs,55 ~ 60,-0 51(-0 62 to -0 41)

Iran_(Islamic_Republic_of),DALYs,60 ~ 65,-0 45(-0 55 to -0 36)

Iran_(Islamic_Republic_of),DALYs,65 ~ 70,-0 39(-0 48 to -0 3)

Iran_(Islamic_Republic_of),DALYs,70 ~ 75,-0 3(-0 4 to -0 21)

Iran_(Islamic_Republic_of),DALYs,75 ~ 80,-0 12(-0 23 to -0 01)

Iran_(Islamic_Republic_of),DALYs,80 ~ 85,0 07(-0 11 to 0 24)

Iran_(Islamic_Republic_of),DALYs,85 ~ 90,0 18(-0 06 to 0 42)

Iran_(Islamic_Republic_of),DALYs,90 ~ 95,0 34(-0 01 to 0 7)

Iran_(Islamic_Republic_of),DALYs,20 ~ 25,-0 15(-0 46 to 0 16)

Iran_(Islamic_Republic_of),DALYs,25 ~ 30,-0 15(-0 37 to 0 06)

Iran_(Islamic_Republic_of),DALYs,30 ~ 35,-0 15(-0 33 to 0 02)

Iran_(Islamic_Republic_of),DALYs,35 ~ 40,-0 21(-0 36 to -0 05)

Iran_(Islamic_Republic_of),DALYs,40 ~ 45,-0 3(-0 44 to -0 16)

Iran_(Islamic_Republic_of),DALYs,45 ~ 50,-0 41(-0 54 to -0 28)

Iran_(Islamic_Republic_of),DALYs,50 ~ 55,-0 51(-0 63 to -0 39)

Iran_(Islamic_Republic_of),DALYs,55 ~ 60,-0 51(-0 62 to -0 41)

Iran_(Islamic_Republic_of),DALYs,60 ~ 65,-0 45(-0 55 to -0 36)

Iran_(Islamic_Republic_of),DALYs,65 ~ 70,-0 39(-0 48 to -0 3)

Iran_(Islamic_Republic_of),DALYs,70 ~ 75,-0 3(-0 4 to -0 21)

Iran_(Islamic_Republic_of),DALYs,75 ~ 80,-0 12(-0 23 to -0 01)

Iran_(Islamic_Republic_of),DALYs,80 ~ 85,0 07(-0 11 to 0 24)

Iran_(Islamic_Republic_of),DALYs,85 ~ 90,0 18(-0 06 to 0 42)

Iran_(Islamic_Republic_of),DALYs,90 ~ 95,0 34(-0 01 to 0 7)

Mauritania,Prevalence,20 ~ 25,-0 41(-0 58 to -0 25)

Mauritania,Prevalence,25 ~ 30,-0 36(-0 49 to -0 24)

Mauritania,Prevalence,30 ~ 35,-0 3(-0 41 to -0 19)

Mauritania,Prevalence,35 ~ 40,-0 24(-0 34 to -0 13)

Mauritania,Prevalence,40 ~ 45,-0 16(-0 26 to -0 07)

Mauritania,Prevalence,45 ~ 50,-0 08(-0 18 to 0 01)

Mauritania,Prevalence,50 ~ 55,0 01(-0 08 to 0 1)

Mauritania,Prevalence,55 ~ 60,0 11(0 02 to 0 2)

Mauritania,Prevalence,60 ~ 65,0 22(0 13 to 0 31)

Mauritania,Prevalence,65 ~ 70,0 34(0 25 to 0 43)

Mauritania,Prevalence,70 ~ 75,0 49(0 39 to 0 58)

Mauritania,Prevalence,75 ~ 80,0 65(0 54 to 0 76)

Mauritania,Prevalence,80 ~ 85,0 83(0 68 to 0 98)

Mauritania,Prevalence,85 ~ 90,1 03(0 79 to 1 26)

Mauritania,Prevalence,90 ~ 95,1 25(0 79 to 1 71)

Mauritania,Prevalence,20 ~ 25,-0 41(-0 58 to -0 25)

Mauritania,Prevalence,25 ~ 30,-0 36(-0 49 to -0 24)

Mauritania,Prevalence,30 ~ 35,-0 3(-0 41 to -0 19)

Mauritania,Prevalence,35 ~ 40,-0 24(-0 34 to -0 13)

Mauritania,Prevalence,40 ~ 45,-0 16(-0 26 to -0 07)

Mauritania,Prevalence,45 ~ 50,-0 08(-0 18 to 0 01)

Mauritania,Prevalence,50 ~ 55,0 01(-0 08 to 0 1)

Mauritania,Prevalence,55 ~ 60,0 11(0 02 to 0 2)

Mauritania,Prevalence,60 ~ 65,0 22(0 13 to 0 31)

Mauritania,Prevalence,65 ~ 70,0 34(0 25 to 0 43)

Mauritania,Prevalence,70 ~ 75,0 49(0 39 to 0 58)

Mauritania,Prevalence,75 ~ 80,0 65(0 54 to 0 76)

Mauritania,Prevalence,80 ~ 85,0 83(0 68 to 0 98)

Mauritania,Prevalence,85 ~ 90,1 03(0 79 to 1 26)

Mauritania,Prevalence,90 ~ 95,1 25(0 79 to 1 71)

Mauritania,Prevalence,20 ~ 25,-0 41(-0 58 to -0 25)

Mauritania,Prevalence,25 ~ 30,-0 36(-0 49 to -0 24)

Mauritania,Prevalence,30 ~ 35,-0 3(-0 41 to -0 19)

Mauritania,Prevalence,35 ~ 40,-0 24(-0 34 to -0 13)

Mauritania,Prevalence,40 ~ 45,-0 16(-0 26 to -0 07)

Mauritania,Prevalence,45 ~ 50,-0 08(-0 18 to 0 01)

Mauritania,Prevalence,50 ~ 55,0 01(-0 08 to 0 1)

Mauritania,Prevalence,55 ~ 60,0 11(0 02 to 0 2)

Mauritania,Prevalence,60 ~ 65,0 22(0 13 to 0 31)

Mauritania,Prevalence,65 ~ 70,0 34(0 25 to 0 43)

Mauritania,Prevalence,70 ~ 75,0 49(0 39 to 0 58)

Mauritania,Prevalence,75 ~ 80,0 65(0 54 to 0 76)

Mauritania,Prevalence,80 ~ 85,0 83(0 68 to 0 98)

Mauritania,Prevalence,85 ~ 90,1 03(0 79 to 1 26)

Mauritania,Prevalence,90 ~ 95,1 25(0 79 to 1 71)

Mauritania,Prevalence,20 ~ 25,-0 41(-0 58 to -0 25)

Mauritania,Prevalence,25 ~ 30,-0 36(-0 49 to -0 24)

Mauritania,Prevalence,30 ~ 35,-0 3(-0 41 to -0 19)

Mauritania,Prevalence,35 ~ 40,-0 24(-0 34 to -0 13)

Mauritania,Prevalence,40 ~ 45,-0 16(-0 26 to -0 07)

Mauritania,Prevalence,45 ~ 50,-0 08(-0 18 to 0 01)

Mauritania,Prevalence,50 ~ 55,0 01(-0 08 to 0 1)

Mauritania,Prevalence,55 ~ 60,0 11(0 02 to 0 2)

Mauritania,Prevalence,60 ~ 65,0 22(0 13 to 0 31)

Mauritania,Prevalence,65 ~ 70,0 34(0 25 to 0 43)

Mauritania,Prevalence,70 ~ 75,0 49(0 39 to 0 58)

Mauritania,Prevalence,75 ~ 80,0 65(0 54 to 0 76)

Mauritania,Prevalence,80 ~ 85,0 83(0 68 to 0 98)

Mauritania,Prevalence,85 ~ 90,1 03(0 79 to 1 26)

Mauritania,Prevalence,90 ~ 95,1 25(0 79 to 1 71)

Mauritania,Deaths,20 ~ 25,-1 73(-8 54 to 5 58)

Mauritania,Deaths,25 ~ 30,-1 69(-5 79 to 2 59)

Mauritania,Deaths,30 ~ 35,-1 62(-4 88 to 1 74)

Mauritania,Deaths,35 ~ 40,-1 64(-4 4 to 1 21)

Mauritania,Deaths,40 ~ 45,-1 74(-3 99 to 0 57)

Mauritania,Deaths,45 ~ 50,-1 85(-3 57 to -0 1)

Mauritania,Deaths,50 ~ 55,-1 96(-3 3 to -0 6)

Mauritania,Deaths,55 ~ 60,-2(-3 07 to -0 92)

Mauritania,Deaths,60 ~ 65,-1 93(-2 79 to -1 07)

Mauritania,Deaths,65 ~ 70,-1 86(-2 57 to -1 14)

Mauritania,Deaths,70 ~ 75,-1 75(-2 39 to -1 11)

Mauritania,Deaths,75 ~ 80,-1 49(-2 13 to -0 85)

Mauritania,Deaths,80 ~ 85,-1 2(-1 95 to -0 44)

Mauritania,Deaths,85 ~ 90,-0 9(-1 92 to 0 12)

Mauritania,Deaths,90 ~ 95,-0 53(-2 28 to 1 25)

Mauritania,Deaths,20 ~ 25,-1 73(-8 54 to 5 58)

Mauritania,Deaths,25 ~ 30,-1 69(-5 79 to 2 59)

Mauritania,Deaths,30 ~ 35,-1 62(-4 88 to 1 74)

Mauritania,Deaths,35 ~ 40,-1 64(-4 4 to 1 21)

Mauritania,Deaths,40 ~ 45,-1 74(-3 99 to 0 57)

Mauritania,Deaths,45 ~ 50,-1 85(-3 57 to -0 1)

Mauritania,Deaths,50 ~ 55,-1 96(-3 3 to -0 6)

Mauritania,Deaths,55 ~ 60,-2(-3 07 to -0 92)

Mauritania,Deaths,60 ~ 65,-1 93(-2 79 to -1 07)

Mauritania,Deaths,65 ~ 70,-1 86(-2 57 to -1 14)

Mauritania,Deaths,70 ~ 75,-1 75(-2 39 to -1 11)

Mauritania,Deaths,75 ~ 80,-1 49(-2 13 to -0 85)

Mauritania,Deaths,80 ~ 85,-1 2(-1 95 to -0 44)

Mauritania,Deaths,85 ~ 90,-0 9(-1 92 to 0 12)

Mauritania,Deaths,90 ~ 95,-0 53(-2 28 to 1 25)

Mauritania,Deaths,20 ~ 25,-1 73(-8 54 to 5 58)

Mauritania,Deaths,25 ~ 30,-1 69(-5 79 to 2 59)

Mauritania,Deaths,30 ~ 35,-1 62(-4 88 to 1 74)

Mauritania,Deaths,35 ~ 40,-1 64(-4 4 to 1 21)

Mauritania,Deaths,40 ~ 45,-1 74(-3 99 to 0 57)

Mauritania,Deaths,45 ~ 50,-1 85(-3 57 to -0 1)

Mauritania,Deaths,50 ~ 55,-1 96(-3 3 to -0 6)

Mauritania,Deaths,55 ~ 60,-2(-3 07 to -0 92)

Mauritania,Deaths,60 ~ 65,-1 93(-2 79 to -1 07)

Mauritania,Deaths,65 ~ 70,-1 86(-2 57 to -1 14)

Mauritania,Deaths,70 ~ 75,-1 75(-2 39 to -1 11)

Mauritania,Deaths,75 ~ 80,-1 49(-2 13 to -0 85)

Mauritania,Deaths,80 ~ 85,-1 2(-1 95 to -0 44)

Mauritania,Deaths,85 ~ 90,-0 9(-1 92 to 0 12)

Mauritania,Deaths,90 ~ 95,-0 53(-2 28 to 1 25)

Mauritania,Deaths,20 ~ 25,-1 73(-8 54 to 5 58)

Mauritania,Deaths,25 ~ 30,-1 69(-5 79 to 2 59)

Mauritania,Deaths,30 ~ 35,-1 62(-4 88 to 1 74)

Mauritania,Deaths,35 ~ 40,-1 64(-4 4 to 1 21)

Mauritania,Deaths,40 ~ 45,-1 74(-3 99 to 0 57)

Mauritania,Deaths,45 ~ 50,-1 85(-3 57 to -0 1)

Mauritania,Deaths,50 ~ 55,-1 96(-3 3 to -0 6)

Mauritania,Deaths,55 ~ 60,-2(-3 07 to -0 92)

Mauritania,Deaths,60 ~ 65,-1 93(-2 79 to -1 07)

Mauritania,Deaths,65 ~ 70,-1 86(-2 57 to -1 14)

Mauritania,Deaths,70 ~ 75,-1 75(-2 39 to -1 11)

Mauritania,Deaths,75 ~ 80,-1 49(-2 13 to -0 85)

Mauritania,Deaths,80 ~ 85,-1 2(-1 95 to -0 44)

Mauritania,Deaths,85 ~ 90,-0 9(-1 92 to 0 12)

Mauritania,Deaths,90 ~ 95,-0 53(-2 28 to 1 25)

Mauritania,DALYs,20 ~ 25,-0 98(-1 41 to -0 54)

Mauritania,DALYs,25 ~ 30,-0 92(-1 24 to -0 6)

Mauritania,DALYs,30 ~ 35,-0 87(-1 15 to -0 6)

Mauritania,DALYs,35 ~ 40,-0 87(-1 12 to -0 63)

Mauritania,DALYs,40 ~ 45,-0 95(-1 17 to -0 73)

Mauritania,DALYs,45 ~ 50,-1 09(-1 28 to -0 89)

Mauritania,DALYs,50 ~ 55,-1 23(-1 41 to -1 06)

Mauritania,DALYs,55 ~ 60,-1 33(-1 48 to -1 17)

Mauritania,DALYs,60 ~ 65,-1 36(-1 5 to -1 22)

Mauritania,DALYs,65 ~ 70,-1 38(-1 51 to -1 25)

Mauritania,DALYs,70 ~ 75,-1 35(-1 48 to -1 22)

Mauritania,DALYs,75 ~ 80,-1 17(-1 32 to -1 02)

Mauritania,DALYs,80 ~ 85,-0 94(-1 14 to -0 75)

Mauritania,DALYs,85 ~ 90,-0 71(-1 to -0 41)

Mauritania,DALYs,90 ~ 95,-0 38(-0 92 to 0 17)

Mauritania,DALYs,20 ~ 25,-0 98(-1 41 to -0 54)

Mauritania,DALYs,25 ~ 30,-0 92(-1 24 to -0 6)

Mauritania,DALYs,30 ~ 35,-0 87(-1 15 to -0 6)

Mauritania,DALYs,35 ~ 40,-0 87(-1 12 to -0 63)

Mauritania,DALYs,40 ~ 45,-0 95(-1 17 to -0 73)

Mauritania,DALYs,45 ~ 50,-1 09(-1 28 to -0 89)

Mauritania,DALYs,50 ~ 55,-1 23(-1 41 to -1 06)

Mauritania,DALYs,55 ~ 60,-1 33(-1 48 to -1 17)

Mauritania,DALYs,60 ~ 65,-1 36(-1 5 to -1 22)

Mauritania,DALYs,65 ~ 70,-1 38(-1 51 to -1 25)

Mauritania,DALYs,70 ~ 75,-1 35(-1 48 to -1 22)

Mauritania,DALYs,75 ~ 80,-1 17(-1 32 to -1 02)

Mauritania,DALYs,80 ~ 85,-0 94(-1 14 to -0 75)

Mauritania,DALYs,85 ~ 90,-0 71(-1 to -0 41)

Mauritania,DALYs,90 ~ 95,-0 38(-0 92 to 0 17)

Mauritania,DALYs,20 ~ 25,-0 98(-1 41 to -0 54)

Mauritania,DALYs,25 ~ 30,-0 92(-1 24 to -0 6)

Mauritania,DALYs,30 ~ 35,-0 87(-1 15 to -0 6)

Mauritania,DALYs,35 ~ 40,-0 87(-1 12 to -0 63)

Mauritania,DALYs,40 ~ 45,-0 95(-1 17 to -0 73)

Mauritania,DALYs,45 ~ 50,-1 09(-1 28 to -0 89)

Mauritania,DALYs,50 ~ 55,-1 23(-1 41 to -1 06)

Mauritania,DALYs,55 ~ 60,-1 33(-1 48 to -1 17)

Mauritania,DALYs,60 ~ 65,-1 36(-1 5 to -1 22)

Mauritania,DALYs,65 ~ 70,-1 38(-1 51 to -1 25)

Mauritania,DALYs,70 ~ 75,-1 35(-1 48 to -1 22)

Mauritania,DALYs,75 ~ 80,-1 17(-1 32 to -1 02)

Mauritania,DALYs,80 ~ 85,-0 94(-1 14 to -0 75)

Mauritania,DALYs,85 ~ 90,-0 71(-1 to -0 41)

Mauritania,DALYs,90 ~ 95,-0 38(-0 92 to 0 17)

Mauritania,DALYs,20 ~ 25,-0 98(-1 41 to -0 54)

Mauritania,DALYs,25 ~ 30,-0 92(-1 24 to -0 6)

Mauritania,DALYs,30 ~ 35,-0 87(-1 15 to -0 6)

Mauritania,DALYs,35 ~ 40,-0 87(-1 12 to -0 63)

Mauritania,DALYs,40 ~ 45,-0 95(-1 17 to -0 73)

Mauritania,DALYs,45 ~ 50,-1 09(-1 28 to -0 89)

Mauritania,DALYs,50 ~ 55,-1 23(-1 41 to -1 06)

Mauritania,DALYs,55 ~ 60,-1 33(-1 48 to -1 17)

Mauritania,DALYs,60 ~ 65,-1 36(-1 5 to -1 22)

Mauritania,DALYs,65 ~ 70,-1 38(-1 51 to -1 25)

Mauritania,DALYs,70 ~ 75,-1 35(-1 48 to -1 22)

Mauritania,DALYs,75 ~ 80,-1 17(-1 32 to -1 02)

Mauritania,DALYs,80 ~ 85,-0 94(-1 14 to -0 75)

Mauritania,DALYs,85 ~ 90,-0 71(-1 to -0 41)

Mauritania,DALYs,90 ~ 95,-0 38(-0 92 to 0 17)

Nigeria,Prevalence,20 ~ 25,-0 23(-0 3 to -0 17)

Nigeria,Prevalence,25 ~ 30,-0 2(-0 25 to -0 14)

Nigeria,Prevalence,30 ~ 35,-0 14(-0 19 to -0 1)

Nigeria,Prevalence,35 ~ 40,-0 07(-0 11 to -0 03)

Nigeria,Prevalence,40 ~ 45,0 03(-0 01 to 0 07)

Nigeria,Prevalence,45 ~ 50,0 13(0 09 to 0 17)

Nigeria,Prevalence,50 ~ 55,0 23(0 2 to 0 27)

Nigeria,Prevalence,55 ~ 60,0 3(0 27 to 0 34)

Nigeria,Prevalence,60 ~ 65,0 36(0 32 to 0 39)

Nigeria,Prevalence,65 ~ 70,0 47(0 43 to 0 51)

Nigeria,Prevalence,70 ~ 75,0 63(0 59 to 0 67)

Nigeria,Prevalence,75 ~ 80,0 8(0 75 to 0 85)

Nigeria,Prevalence,80 ~ 85,0 99(0 92 to 1 05)

Nigeria,Prevalence,85 ~ 90,1 16(1 06 to 1 26)

Nigeria,Prevalence,90 ~ 95,1 26(1 07 to 1 46)

Nigeria,Prevalence,20 ~ 25,-0 23(-0 3 to -0 17)

Nigeria,Prevalence,25 ~ 30,-0 2(-0 25 to -0 14)

Nigeria,Prevalence,30 ~ 35,-0 14(-0 19 to -0 1)

Nigeria,Prevalence,35 ~ 40,-0 07(-0 11 to -0 03)

Nigeria,Prevalence,40 ~ 45,0 03(-0 01 to 0 07)

Nigeria,Prevalence,45 ~ 50,0 13(0 09 to 0 17)

Nigeria,Prevalence,50 ~ 55,0 23(0 2 to 0 27)

Nigeria,Prevalence,55 ~ 60,0 3(0 27 to 0 34)

Nigeria,Prevalence,60 ~ 65,0 36(0 32 to 0 39)

Nigeria,Prevalence,65 ~ 70,0 47(0 43 to 0 51)

Nigeria,Prevalence,70 ~ 75,0 63(0 59 to 0 67)

Nigeria,Prevalence,75 ~ 80,0 8(0 75 to 0 85)

Nigeria,Prevalence,80 ~ 85,0 99(0 92 to 1 05)

Nigeria,Prevalence,85 ~ 90,1 16(1 06 to 1 26)

Nigeria,Prevalence,90 ~ 95,1 26(1 07 to 1 46)

Nigeria,Prevalence,20 ~ 25,-0 23(-0 3 to -0 17)

Nigeria,Prevalence,25 ~ 30,-0 2(-0 25 to -0 14)

Nigeria,Prevalence,30 ~ 35,-0 14(-0 19 to -0 1)

Nigeria,Prevalence,35 ~ 40,-0 07(-0 11 to -0 03)

Nigeria,Prevalence,40 ~ 45,0 03(-0 01 to 0 07)

Nigeria,Prevalence,45 ~ 50,0 13(0 09 to 0 17)

Nigeria,Prevalence,50 ~ 55,0 23(0 2 to 0 27)

Nigeria,Prevalence,55 ~ 60,0 3(0 27 to 0 34)

Nigeria,Prevalence,60 ~ 65,0 36(0 32 to 0 39)

Nigeria,Prevalence,65 ~ 70,0 47(0 43 to 0 51)

Nigeria,Prevalence,70 ~ 75,0 63(0 59 to 0 67)

Nigeria,Prevalence,75 ~ 80,0 8(0 75 to 0 85)

Nigeria,Prevalence,80 ~ 85,0 99(0 92 to 1 05)

Nigeria,Prevalence,85 ~ 90,1 16(1 06 to 1 26)

Nigeria,Prevalence,90 ~ 95,1 26(1 07 to 1 46)

Nigeria,Prevalence,20 ~ 25,-0 23(-0 3 to -0 17)

Nigeria,Prevalence,25 ~ 30,-0 2(-0 25 to -0 14)

Nigeria,Prevalence,30 ~ 35,-0 14(-0 19 to -0 1)

Nigeria,Prevalence,35 ~ 40,-0 07(-0 11 to -0 03)

Nigeria,Prevalence,40 ~ 45,0 03(-0 01 to 0 07)

Nigeria,Prevalence,45 ~ 50,0 13(0 09 to 0 17)

Nigeria,Prevalence,50 ~ 55,0 23(0 2 to 0 27)

Nigeria,Prevalence,55 ~ 60,0 3(0 27 to 0 34)

Nigeria,Prevalence,60 ~ 65,0 36(0 32 to 0 39)

Nigeria,Prevalence,65 ~ 70,0 47(0 43 to 0 51)

Nigeria,Prevalence,70 ~ 75,0 63(0 59 to 0 67)

Nigeria,Prevalence,75 ~ 80,0 8(0 75 to 0 85)

Nigeria,Prevalence,80 ~ 85,0 99(0 92 to 1 05)

Nigeria,Prevalence,85 ~ 90,1 16(1 06 to 1 26)

Nigeria,Prevalence,90 ~ 95,1 26(1 07 to 1 46)

Nigeria,Deaths,20 ~ 25,-0 27(-1 52 to 0 99)

Nigeria,Deaths,25 ~ 30,-0 44(-1 16 to 0 29)

Nigeria,Deaths,30 ~ 35,-0 7(-1 26 to -0 15)

Nigeria,Deaths,35 ~ 40,-0 99(-1 45 to -0 54)

Nigeria,Deaths,40 ~ 45,-1 29(-1 65 to -0 93)

Nigeria,Deaths,45 ~ 50,-1 47(-1 74 to -1 21)

Nigeria,Deaths,50 ~ 55,-1 53(-1 73 to -1 32)

Nigeria,Deaths,55 ~ 60,-1 56(-1 72 to -1 39)

Nigeria,Deaths,60 ~ 65,-1 55(-1 68 to -1 41)

Nigeria,Deaths,65 ~ 70,-1 38(-1 5 to -1 27)

Nigeria,Deaths,70 ~ 75,-1 13(-1 23 to -1 03)

Nigeria,Deaths,75 ~ 80,-0 84(-0 94 to -0 73)

Nigeria,Deaths,80 ~ 85,-0 53(-0 65 to -0 4)

Nigeria,Deaths,85 ~ 90,-0 25(-0 42 to -0 08)

Nigeria,Deaths,90 ~ 95,-0 08(-0 38 to 0 22)

Nigeria,Deaths,20 ~ 25,-0 27(-1 52 to 0 99)

Nigeria,Deaths,25 ~ 30,-0 44(-1 16 to 0 29)

Nigeria,Deaths,30 ~ 35,-0 7(-1 26 to -0 15)

Nigeria,Deaths,35 ~ 40,-0 99(-1 45 to -0 54)

Nigeria,Deaths,40 ~ 45,-1 29(-1 65 to -0 93)

Nigeria,Deaths,45 ~ 50,-1 47(-1 74 to -1 21)

Nigeria,Deaths,50 ~ 55,-1 53(-1 73 to -1 32)

Nigeria,Deaths,55 ~ 60,-1 56(-1 72 to -1 39)

Nigeria,Deaths,60 ~ 65,-1 55(-1 68 to -1 41)

Nigeria,Deaths,65 ~ 70,-1 38(-1 5 to -1 27)

Nigeria,Deaths,70 ~ 75,-1 13(-1 23 to -1 03)

Nigeria,Deaths,75 ~ 80,-0 84(-0 94 to -0 73)

Nigeria,Deaths,80 ~ 85,-0 53(-0 65 to -0 4)

Nigeria,Deaths,85 ~ 90,-0 25(-0 42 to -0 08)

Nigeria,Deaths,90 ~ 95,-0 08(-0 38 to 0 22)

Nigeria,Deaths,20 ~ 25,-0 27(-1 52 to 0 99)

Nigeria,Deaths,25 ~ 30,-0 44(-1 16 to 0 29)

Nigeria,Deaths,30 ~ 35,-0 7(-1 26 to -0 15)

Nigeria,Deaths,35 ~ 40,-0 99(-1 45 to -0 54)

Nigeria,Deaths,40 ~ 45,-1 29(-1 65 to -0 93)

Nigeria,Deaths,45 ~ 50,-1 47(-1 74 to -1 21)

Nigeria,Deaths,50 ~ 55,-1 53(-1 73 to -1 32)

Nigeria,Deaths,55 ~ 60,-1 56(-1 72 to -1 39)

Nigeria,Deaths,60 ~ 65,-1 55(-1 68 to -1 41)

Nigeria,Deaths,65 ~ 70,-1 38(-1 5 to -1 27)

Nigeria,Deaths,70 ~ 75,-1 13(-1 23 to -1 03)

Nigeria,Deaths,75 ~ 80,-0 84(-0 94 to -0 73)

Nigeria,Deaths,80 ~ 85,-0 53(-0 65 to -0 4)

Nigeria,Deaths,85 ~ 90,-0 25(-0 42 to -0 08)

Nigeria,Deaths,90 ~ 95,-0 08(-0 38 to 0 22)

Nigeria,Deaths,20 ~ 25,-0 27(-1 52 to 0 99)

Nigeria,Deaths,25 ~ 30,-0 44(-1 16 to 0 29)

Nigeria,Deaths,30 ~ 35,-0 7(-1 26 to -0 15)

Nigeria,Deaths,35 ~ 40,-0 99(-1 45 to -0 54)

Nigeria,Deaths,40 ~ 45,-1 29(-1 65 to -0 93)

Nigeria,Deaths,45 ~ 50,-1 47(-1 74 to -1 21)

Nigeria,Deaths,50 ~ 55,-1 53(-1 73 to -1 32)

Nigeria,Deaths,55 ~ 60,-1 56(-1 72 to -1 39)

Nigeria,Deaths,60 ~ 65,-1 55(-1 68 to -1 41)

Nigeria,Deaths,65 ~ 70,-1 38(-1 5 to -1 27)

Nigeria,Deaths,70 ~ 75,-1 13(-1 23 to -1 03)

Nigeria,Deaths,75 ~ 80,-0 84(-0 94 to -0 73)

Nigeria,Deaths,80 ~ 85,-0 53(-0 65 to -0 4)

Nigeria,Deaths,85 ~ 90,-0 25(-0 42 to -0 08)

Nigeria,Deaths,90 ~ 95,-0 08(-0 38 to 0 22)

Nigeria,DALYs,20 ~ 25,-0 14(-0 28 to 0 01)

Nigeria,DALYs,25 ~ 30,-0 19(-0 3 to -0 08)

Nigeria,DALYs,30 ~ 35,-0 28(-0 37 to -0 18)

Nigeria,DALYs,35 ~ 40,-0 39(-0 47 to -0 3)

Nigeria,DALYs,40 ~ 45,-0 53(-0 61 to -0 46)

Nigeria,DALYs,45 ~ 50,-0 67(-0 74 to -0 6)

Nigeria,DALYs,50 ~ 55,-0 77(-0 83 to -0 71)

Nigeria,DALYs,55 ~ 60,-0 87(-0 92 to -0 81)

Nigeria,DALYs,60 ~ 65,-0 95(-1 to -0 91)

Nigeria,DALYs,65 ~ 70,-0 9(-0 95 to -0 86)

Nigeria,DALYs,70 ~ 75,-0 75(-0 8 to -0 7)

Nigeria,DALYs,75 ~ 80,-0 54(-0 6 to -0 48)

Nigeria,DALYs,80 ~ 85,-0 29(-0 36 to -0 22)

Nigeria,DALYs,85 ~ 90,-0 06(-0 17 to 0 06)

Nigeria,DALYs,90 ~ 95,0 09(-0 12 to 0 31)

Nigeria,DALYs,20 ~ 25,-0 14(-0 28 to 0 01)

Nigeria,DALYs,25 ~ 30,-0 19(-0 3 to -0 08)

Nigeria,DALYs,30 ~ 35,-0 28(-0 37 to -0 18)

Nigeria,DALYs,35 ~ 40,-0 39(-0 47 to -0 3)

Nigeria,DALYs,40 ~ 45,-0 53(-0 61 to -0 46)

Nigeria,DALYs,45 ~ 50,-0 67(-0 74 to -0 6)

Nigeria,DALYs,50 ~ 55,-0 77(-0 83 to -0 71)

Nigeria,DALYs,55 ~ 60,-0 87(-0 92 to -0 81)

Nigeria,DALYs,60 ~ 65,-0 95(-1 to -0 91)

Nigeria,DALYs,65 ~ 70,-0 9(-0 95 to -0 86)

Nigeria,DALYs,70 ~ 75,-0 75(-0 8 to -0 7)

Nigeria,DALYs,75 ~ 80,-0 54(-0 6 to -0 48)

Nigeria,DALYs,80 ~ 85,-0 29(-0 36 to -0 22)

Nigeria,DALYs,85 ~ 90,-0 06(-0 17 to 0 06)

Nigeria,DALYs,90 ~ 95,0 09(-0 12 to 0 31)

Nigeria,DALYs,20 ~ 25,-0 14(-0 28 to 0 01)

Nigeria,DALYs,25 ~ 30,-0 19(-0 3 to -0 08)

Nigeria,DALYs,30 ~ 35,-0 28(-0 37 to -0 18)

Nigeria,DALYs,35 ~ 40,-0 39(-0 47 to -0 3)

Nigeria,DALYs,40 ~ 45,-0 53(-0 61 to -0 46)

Nigeria,DALYs,45 ~ 50,-0 67(-0 74 to -0 6)

Nigeria,DALYs,50 ~ 55,-0 77(-0 83 to -0 71)

Nigeria,DALYs,55 ~ 60,-0 87(-0 92 to -0 81)

Nigeria,DALYs,60 ~ 65,-0 95(-1 to -0 91)

Nigeria,DALYs,65 ~ 70,-0 9(-0 95 to -0 86)

Nigeria,DALYs,70 ~ 75,-0 75(-0 8 to -0 7)

Nigeria,DALYs,75 ~ 80,-0 54(-0 6 to -0 48)

Nigeria,DALYs,80 ~ 85,-0 29(-0 36 to -0 22)

Nigeria,DALYs,85 ~ 90,-0 06(-0 17 to 0 06)

Nigeria,DALYs,90 ~ 95,0 09(-0 12 to 0 31)

Nigeria,DALYs,20 ~ 25,-0 14(-0 28 to 0 01)

Nigeria,DALYs,25 ~ 30,-0 19(-0 3 to -0 08)

Nigeria,DALYs,30 ~ 35,-0 28(-0 37 to -0 18)

Nigeria,DALYs,35 ~ 40,-0 39(-0 47 to -0 3)

Nigeria,DALYs,40 ~ 45,-0 53(-0 61 to -0 46)

Nigeria,DALYs,45 ~ 50,-0 67(-0 74 to -0 6)

Nigeria,DALYs,50 ~ 55,-0 77(-0 83 to -0 71)

Nigeria,DALYs,55 ~ 60,-0 87(-0 92 to -0 81)

Nigeria,DALYs,60 ~ 65,-0 95(-1 to -0 91)

Nigeria,DALYs,65 ~ 70,-0 9(-0 95 to -0 86)

Nigeria,DALYs,70 ~ 75,-0 75(-0 8 to -0 7)

Nigeria,DALYs,75 ~ 80,-0 54(-0 6 to -0 48)

Nigeria,DALYs,80 ~ 85,-0 29(-0 36 to -0 22)

Nigeria,DALYs,85 ~ 90,-0 06(-0 17 to 0 06)

Nigeria,DALYs,90 ~ 95,0 09(-0 12 to 0 31)

Finland,Prevalence,20 ~ 25,-0 23(-0 54 to 0 08)

Finland,Prevalence,25 ~ 30,-0 23(-0 44 to -0 02)

Finland,Prevalence,30 ~ 35,-0 27(-0 44 to -0 11)

Finland,Prevalence,35 ~ 40,-0 36(-0 5 to -0 23)

Finland,Prevalence,40 ~ 45,-0 48(-0 6 to -0 37)

Finland,Prevalence,45 ~ 50,-0 6(-0 69 to -0 5)

Finland,Prevalence,50 ~ 55,-0 64(-0 72 to -0 57)

Finland,Prevalence,55 ~ 60,-0 6(-0 66 to -0 53)

Finland,Prevalence,60 ~ 65,-0 48(-0 54 to -0 42)

Finland,Prevalence,65 ~ 70,-0 32(-0 37 to -0 27)

Finland,Prevalence,70 ~ 75,-0 1(-0 15 to -0 05)

Finland,Prevalence,75 ~ 80,0 13(0 08 to 0 18)

Finland,Prevalence,80 ~ 85,0 35(0 3 to 0 41)

Finland,Prevalence,85 ~ 90,0 57(0 49 to 0 65)

Finland,Prevalence,90 ~ 95,0 76(0 62 to 0 9)

Finland,Prevalence,20 ~ 25,-0 23(-0 54 to 0 08)

Finland,Prevalence,25 ~ 30,-0 23(-0 44 to -0 02)

Finland,Prevalence,30 ~ 35,-0 27(-0 44 to -0 11)

Finland,Prevalence,35 ~ 40,-0 36(-0 5 to -0 23)

Finland,Prevalence,40 ~ 45,-0 48(-0 6 to -0 37)

Finland,Prevalence,45 ~ 50,-0 6(-0 69 to -0 5)

Finland,Prevalence,50 ~ 55,-0 64(-0 72 to -0 57)

Finland,Prevalence,55 ~ 60,-0 6(-0 66 to -0 53)

Finland,Prevalence,60 ~ 65,-0 48(-0 54 to -0 42)

Finland,Prevalence,65 ~ 70,-0 32(-0 37 to -0 27)

Finland,Prevalence,70 ~ 75,-0 1(-0 15 to -0 05)

Finland,Prevalence,75 ~ 80,0 13(0 08 to 0 18)

Finland,Prevalence,80 ~ 85,0 35(0 3 to 0 41)

Finland,Prevalence,85 ~ 90,0 57(0 49 to 0 65)

Finland,Prevalence,90 ~ 95,0 76(0 62 to 0 9)

Finland,Prevalence,20 ~ 25,-0 23(-0 54 to 0 08)

Finland,Prevalence,25 ~ 30,-0 23(-0 44 to -0 02)

Finland,Prevalence,30 ~ 35,-0 27(-0 44 to -0 11)

Finland,Prevalence,35 ~ 40,-0 36(-0 5 to -0 23)

Finland,Prevalence,40 ~ 45,-0 48(-0 6 to -0 37)

Finland,Prevalence,45 ~ 50,-0 6(-0 69 to -0 5)

Finland,Prevalence,50 ~ 55,-0 64(-0 72 to -0 57)

Finland,Prevalence,55 ~ 60,-0 6(-0 66 to -0 53)

Finland,Prevalence,60 ~ 65,-0 48(-0 54 to -0 42)

Finland,Prevalence,65 ~ 70,-0 32(-0 37 to -0 27)

Finland,Prevalence,70 ~ 75,-0 1(-0 15 to -0 05)

Finland,Prevalence,75 ~ 80,0 13(0 08 to 0 18)

Finland,Prevalence,80 ~ 85,0 35(0 3 to 0 41)

Finland,Prevalence,85 ~ 90,0 57(0 49 to 0 65)

Finland,Prevalence,90 ~ 95,0 76(0 62 to 0 9)

Finland,Prevalence,20 ~ 25,-0 23(-0 54 to 0 08)

Finland,Prevalence,25 ~ 30,-0 23(-0 44 to -0 02)

Finland,Prevalence,30 ~ 35,-0 27(-0 44 to -0 11)

Finland,Prevalence,35 ~ 40,-0 36(-0 5 to -0 23)

Finland,Prevalence,40 ~ 45,-0 48(-0 6 to -0 37)

Finland,Prevalence,45 ~ 50,-0 6(-0 69 to -0 5)

Finland,Prevalence,50 ~ 55,-0 64(-0 72 to -0 57)

Finland,Prevalence,55 ~ 60,-0 6(-0 66 to -0 53)

Finland,Prevalence,60 ~ 65,-0 48(-0 54 to -0 42)

Finland,Prevalence,65 ~ 70,-0 32(-0 37 to -0 27)

Finland,Prevalence,70 ~ 75,-0 1(-0 15 to -0 05)

Finland,Prevalence,75 ~ 80,0 13(0 08 to 0 18)

Finland,Prevalence,80 ~ 85,0 35(0 3 to 0 41)

Finland,Prevalence,85 ~ 90,0 57(0 49 to 0 65)

Finland,Prevalence,90 ~ 95,0 76(0 62 to 0 9)

Finland,Deaths,20 ~ 25,-1 04(-11 95 to 11 21)

Finland,Deaths,25 ~ 30,-1 51(-9 24 to 6 87)

Finland,Deaths,30 ~ 35,-1 8(-7 59 to 4 35)

Finland,Deaths,35 ~ 40,-2 14(-6 36 to 2 27)

Finland,Deaths,40 ~ 45,-2 51(-5 47 to 0 55)

Finland,Deaths,45 ~ 50,-2 49(-4 55 to -0 39)

Finland,Deaths,50 ~ 55,-2 25(-3 56 to -0 92)

Finland,Deaths,55 ~ 60,-1 86(-2 68 to -1 03)

Finland,Deaths,60 ~ 65,-1 69(-2 24 to -1 13)

Finland,Deaths,65 ~ 70,-1 48(-1 88 to -1 08)

Finland,Deaths,70 ~ 75,-1 18(-1 49 to -0 86)

Finland,Deaths,75 ~ 80,-0 94(-1 24 to -0 64)

Finland,Deaths,80 ~ 85,-0 36(-0 68 to -0 04)

Finland,Deaths,85 ~ 90,0 32(-0 12 to 0 76)

Finland,Deaths,90 ~ 95,0 64(-0 11 to 1 4)

Finland,Deaths,20 ~ 25,-1 04(-11 95 to 11 21)

Finland,Deaths,25 ~ 30,-1 51(-9 24 to 6 87)

Finland,Deaths,30 ~ 35,-1 8(-7 59 to 4 35)

Finland,Deaths,35 ~ 40,-2 14(-6 36 to 2 27)

Finland,Deaths,40 ~ 45,-2 51(-5 47 to 0 55)

Finland,Deaths,45 ~ 50,-2 49(-4 55 to -0 39)

Finland,Deaths,50 ~ 55,-2 25(-3 56 to -0 92)

Finland,Deaths,55 ~ 60,-1 86(-2 68 to -1 03)

Finland,Deaths,60 ~ 65,-1 69(-2 24 to -1 13)

Finland,Deaths,65 ~ 70,-1 48(-1 88 to -1 08)

Finland,Deaths,70 ~ 75,-1 18(-1 49 to -0 86)

Finland,Deaths,75 ~ 80,-0 94(-1 24 to -0 64)

Finland,Deaths,80 ~ 85,-0 36(-0 68 to -0 04)

Finland,Deaths,85 ~ 90,0 32(-0 12 to 0 76)

Finland,Deaths,90 ~ 95,0 64(-0 11 to 1 4)

Finland,Deaths,20 ~ 25,-1 04(-11 95 to 11 21)

Finland,Deaths,25 ~ 30,-1 51(-9 24 to 6 87)

Finland,Deaths,30 ~ 35,-1 8(-7 59 to 4 35)

Finland,Deaths,35 ~ 40,-2 14(-6 36 to 2 27)

Finland,Deaths,40 ~ 45,-2 51(-5 47 to 0 55)

Finland,Deaths,45 ~ 50,-2 49(-4 55 to -0 39)

Finland,Deaths,50 ~ 55,-2 25(-3 56 to -0 92)

Finland,Deaths,55 ~ 60,-1 86(-2 68 to -1 03)

Finland,Deaths,60 ~ 65,-1 69(-2 24 to -1 13)

Finland,Deaths,65 ~ 70,-1 48(-1 88 to -1 08)

Finland,Deaths,70 ~ 75,-1 18(-1 49 to -0 86)

Finland,Deaths,75 ~ 80,-0 94(-1 24 to -0 64)

Finland,Deaths,80 ~ 85,-0 36(-0 68 to -0 04)

Finland,Deaths,85 ~ 90,0 32(-0 12 to 0 76)

Finland,Deaths,90 ~ 95,0 64(-0 11 to 1 4)

Finland,Deaths,20 ~ 25,-1 04(-11 95 to 11 21)

Finland,Deaths,25 ~ 30,-1 51(-9 24 to 6 87)

Finland,Deaths,30 ~ 35,-1 8(-7 59 to 4 35)

Finland,Deaths,35 ~ 40,-2 14(-6 36 to 2 27)

Finland,Deaths,40 ~ 45,-2 51(-5 47 to 0 55)

Finland,Deaths,45 ~ 50,-2 49(-4 55 to -0 39)

Finland,Deaths,50 ~ 55,-2 25(-3 56 to -0 92)

Finland,Deaths,55 ~ 60,-1 86(-2 68 to -1 03)

Finland,Deaths,60 ~ 65,-1 69(-2 24 to -1 13)

Finland,Deaths,65 ~ 70,-1 48(-1 88 to -1 08)

Finland,Deaths,70 ~ 75,-1 18(-1 49 to -0 86)

Finland,Deaths,75 ~ 80,-0 94(-1 24 to -0 64)

Finland,Deaths,80 ~ 85,-0 36(-0 68 to -0 04)

Finland,Deaths,85 ~ 90,0 32(-0 12 to 0 76)

Finland,Deaths,90 ~ 95,0 64(-0 11 to 1 4)

Finland,DALYs,20 ~ 25,-0 4(-1 23 to 0 45)

Finland,DALYs,25 ~ 30,-0 5(-1 08 to 0 08)

Finland,DALYs,30 ~ 35,-0 62(-1 07 to -0 17)

Finland,DALYs,35 ~ 40,-0 89(-1 25 to -0 52)

Finland,DALYs,40 ~ 45,-1 23(-1 52 to -0 94)

Finland,DALYs,45 ~ 50,-1 39(-1 62 to -1 16)

Finland,DALYs,50 ~ 55,-1 44(-1 61 to -1 26)

Finland,DALYs,55 ~ 60,-1 32(-1 45 to -1 19)

Finland,DALYs,60 ~ 65,-1 28(-1 38 to -1 17)

Finland,DALYs,65 ~ 70,-1 17(-1 26 to -1 09)

Finland,DALYs,70 ~ 75,-0 92(-1 to -0 85)

Finland,DALYs,75 ~ 80,-0 67(-0 75 to -0 59)

Finland,DALYs,80 ~ 85,-0 19(-0 29 to -0 1)

Finland,DALYs,85 ~ 90,0 37(0 22 to 0 51)

Finland,DALYs,90 ~ 95,0 68(0 42 to 0 94)

Finland,DALYs,20 ~ 25,-0 4(-1 23 to 0 45)

Finland,DALYs,25 ~ 30,-0 5(-1 08 to 0 08)

Finland,DALYs,30 ~ 35,-0 62(-1 07 to -0 17)

Finland,DALYs,35 ~ 40,-0 89(-1 25 to -0 52)

Finland,DALYs,40 ~ 45,-1 23(-1 52 to -0 94)

Finland,DALYs,45 ~ 50,-1 39(-1 62 to -1 16)

Finland,DALYs,50 ~ 55,-1 44(-1 61 to -1 26)

Finland,DALYs,55 ~ 60,-1 32(-1 45 to -1 19)

Finland,DALYs,60 ~ 65,-1 28(-1 38 to -1 17)

Finland,DALYs,65 ~ 70,-1 17(-1 26 to -1 09)

Finland,DALYs,70 ~ 75,-0 92(-1 to -0 85)

Finland,DALYs,75 ~ 80,-0 67(-0 75 to -0 59)

Finland,DALYs,80 ~ 85,-0 19(-0 29 to -0 1)

Finland,DALYs,85 ~ 90,0 37(0 22 to 0 51)

Finland,DALYs,90 ~ 95,0 68(0 42 to 0 94)

Finland,DALYs,20 ~ 25,-0 4(-1 23 to 0 45)

Finland,DALYs,25 ~ 30,-0 5(-1 08 to 0 08)

Finland,DALYs,30 ~ 35,-0 62(-1 07 to -0 17)

Finland,DALYs,35 ~ 40,-0 89(-1 25 to -0 52)

Finland,DALYs,40 ~ 45,-1 23(-1 52 to -0 94)

Finland,DALYs,45 ~ 50,-1 39(-1 62 to -1 16)

Finland,DALYs,50 ~ 55,-1 44(-1 61 to -1 26)

Finland,DALYs,55 ~ 60,-1 32(-1 45 to -1 19)

Finland,DALYs,60 ~ 65,-1 28(-1 38 to -1 17)

Finland,DALYs,65 ~ 70,-1 17(-1 26 to -1 09)

Finland,DALYs,70 ~ 75,-0 92(-1 to -0 85)

Finland,DALYs,75 ~ 80,-0 67(-0 75 to -0 59)

Finland,DALYs,80 ~ 85,-0 19(-0 29 to -0 1)

Finland,DALYs,85 ~ 90,0 37(0 22 to 0 51)

Finland,DALYs,90 ~ 95,0 68(0 42 to 0 94)

Finland,DALYs,20 ~ 25,-0 4(-1 23 to 0 45)

Finland,DALYs,25 ~ 30,-0 5(-1 08 to 0 08)

Finland,DALYs,30 ~ 35,-0 62(-1 07 to -0 17)

Finland,DALYs,35 ~ 40,-0 89(-1 25 to -0 52)

Finland,DALYs,40 ~ 45,-1 23(-1 52 to -0 94)

Finland,DALYs,45 ~ 50,-1 39(-1 62 to -1 16)

Finland,DALYs,50 ~ 55,-1 44(-1 61 to -1 26)

Finland,DALYs,55 ~ 60,-1 32(-1 45 to -1 19)

Finland,DALYs,60 ~ 65,-1 28(-1 38 to -1 17)

Finland,DALYs,65 ~ 70,-1 17(-1 26 to -1 09)

Finland,DALYs,70 ~ 75,-0 92(-1 to -0 85)

Finland,DALYs,75 ~ 80,-0 67(-0 75 to -0 59)

Finland,DALYs,80 ~ 85,-0 19(-0 29 to -0 1)

Finland,DALYs,85 ~ 90,0 37(0 22 to 0 51)

Finland,DALYs,90 ~ 95,0 68(0 42 to 0 94)

Jordan,Prevalence,20 ~ 25,-0 36(-0 45 to -0 26)

Jordan,Prevalence,25 ~ 30,-0 36(-0 43 to -0 28)

Jordan,Prevalence,30 ~ 35,-0 34(-0 41 to -0 28)

Jordan,Prevalence,35 ~ 40,-0 33(-0 39 to -0 27)

Jordan,Prevalence,40 ~ 45,-0 32(-0 38 to -0 26)

Jordan,Prevalence,45 ~ 50,-0 31(-0 37 to -0 26)

Jordan,Prevalence,50 ~ 55,-0 28(-0 33 to -0 23)

Jordan,Prevalence,55 ~ 60,-0 21(-0 26 to -0 16)

Jordan,Prevalence,60 ~ 65,-0 09(-0 14 to -0 04)

Jordan,Prevalence,65 ~ 70,0 07(0 02 to 0 13)

Jordan,Prevalence,70 ~ 75,0 27(0 2 to 0 33)

Jordan,Prevalence,75 ~ 80,0 48(0 4 to 0 55)

Jordan,Prevalence,80 ~ 85,0 7(0 6 to 0 8)

Jordan,Prevalence,85 ~ 90,0 92(0 78 to 1 06)

Jordan,Prevalence,90 ~ 95,1 14(0 88 to 1 4)

Jordan,Prevalence,20 ~ 25,-0 36(-0 45 to -0 26)

Jordan,Prevalence,25 ~ 30,-0 36(-0 43 to -0 28)

Jordan,Prevalence,30 ~ 35,-0 34(-0 41 to -0 28)

Jordan,Prevalence,35 ~ 40,-0 33(-0 39 to -0 27)

Jordan,Prevalence,40 ~ 45,-0 32(-0 38 to -0 26)

Jordan,Prevalence,45 ~ 50,-0 31(-0 37 to -0 26)

Jordan,Prevalence,50 ~ 55,-0 28(-0 33 to -0 23)

Jordan,Prevalence,55 ~ 60,-0 21(-0 26 to -0 16)

Jordan,Prevalence,60 ~ 65,-0 09(-0 14 to -0 04)

Jordan,Prevalence,65 ~ 70,0 07(0 02 to 0 13)

Jordan,Prevalence,70 ~ 75,0 27(0 2 to 0 33)

Jordan,Prevalence,75 ~ 80,0 48(0 4 to 0 55)

Jordan,Prevalence,80 ~ 85,0 7(0 6 to 0 8)

Jordan,Prevalence,85 ~ 90,0 92(0 78 to 1 06)

Jordan,Prevalence,90 ~ 95,1 14(0 88 to 1 4)

Jordan,Prevalence,20 ~ 25,-0 36(-0 45 to -0 26)

Jordan,Prevalence,25 ~ 30,-0 36(-0 43 to -0 28)

Jordan,Prevalence,30 ~ 35,-0 34(-0 41 to -0 28)

Jordan,Prevalence,35 ~ 40,-0 33(-0 39 to -0 27)

Jordan,Prevalence,40 ~ 45,-0 32(-0 38 to -0 26)

Jordan,Prevalence,45 ~ 50,-0 31(-0 37 to -0 26)

Jordan,Prevalence,50 ~ 55,-0 28(-0 33 to -0 23)

Jordan,Prevalence,55 ~ 60,-0 21(-0 26 to -0 16)

Jordan,Prevalence,60 ~ 65,-0 09(-0 14 to -0 04)

Jordan,Prevalence,65 ~ 70,0 07(0 02 to 0 13)

Jordan,Prevalence,70 ~ 75,0 27(0 2 to 0 33)

Jordan,Prevalence,75 ~ 80,0 48(0 4 to 0 55)

Jordan,Prevalence,80 ~ 85,0 7(0 6 to 0 8)

Jordan,Prevalence,85 ~ 90,0 92(0 78 to 1 06)

Jordan,Prevalence,90 ~ 95,1 14(0 88 to 1 4)

Jordan,Prevalence,20 ~ 25,-0 36(-0 45 to -0 26)

Jordan,Prevalence,25 ~ 30,-0 36(-0 43 to -0 28)

Jordan,Prevalence,30 ~ 35,-0 34(-0 41 to -0 28)

Jordan,Prevalence,35 ~ 40,-0 33(-0 39 to -0 27)

Jordan,Prevalence,40 ~ 45,-0 32(-0 38 to -0 26)

Jordan,Prevalence,45 ~ 50,-0 31(-0 37 to -0 26)

Jordan,Prevalence,50 ~ 55,-0 28(-0 33 to -0 23)

Jordan,Prevalence,55 ~ 60,-0 21(-0 26 to -0 16)

Jordan,Prevalence,60 ~ 65,-0 09(-0 14 to -0 04)

Jordan,Prevalence,65 ~ 70,0 07(0 02 to 0 13)

Jordan,Prevalence,70 ~ 75,0 27(0 2 to 0 33)

Jordan,Prevalence,75 ~ 80,0 48(0 4 to 0 55)

Jordan,Prevalence,80 ~ 85,0 7(0 6 to 0 8)

Jordan,Prevalence,85 ~ 90,0 92(0 78 to 1 06)

Jordan,Prevalence,90 ~ 95,1 14(0 88 to 1 4)

Jordan,Deaths,20 ~ 25,-2 94(-6 4 to 0 65)

Jordan,Deaths,25 ~ 30,-2 77(-5 3 to -0 18)

Jordan,Deaths,30 ~ 35,-2 78(-4 88 to -0 64)

Jordan,Deaths,35 ~ 40,-2 85(-4 63 to -1 04)

Jordan,Deaths,40 ~ 45,-2 96(-4 46 to -1 44)

Jordan,Deaths,45 ~ 50,-3 16(-4 4 to -1 91)

Jordan,Deaths,50 ~ 55,-3 4(-4 4 to -2 39)

Jordan,Deaths,55 ~ 60,-3 56(-4 39 to -2 72)

Jordan,Deaths,60 ~ 65,-3 62(-4 32 to -2 91)

Jordan,Deaths,65 ~ 70,-3 48(-4 11 to -2 85)

Jordan,Deaths,70 ~ 75,-3 14(-3 74 to -2 55)

Jordan,Deaths,75 ~ 80,-2 77(-3 4 to -2 13)

Jordan,Deaths,80 ~ 85,-2 4(-3 13 to -1 66)

Jordan,Deaths,85 ~ 90,-2 02(-2 97 to -1 06)

Jordan,Deaths,90 ~ 95,-1 67(-3 28 to -0 03)

Jordan,Deaths,20 ~ 25,-2 94(-6 4 to 0 65)

Jordan,Deaths,25 ~ 30,-2 77(-5 3 to -0 18)

Jordan,Deaths,30 ~ 35,-2 78(-4 88 to -0 64)

Jordan,Deaths,35 ~ 40,-2 85(-4 63 to -1 04)

Jordan,Deaths,40 ~ 45,-2 96(-4 46 to -1 44)

Jordan,Deaths,45 ~ 50,-3 16(-4 4 to -1 91)

Jordan,Deaths,50 ~ 55,-3 4(-4 4 to -2 39)

Jordan,Deaths,55 ~ 60,-3 56(-4 39 to -2 72)

Jordan,Deaths,60 ~ 65,-3 62(-4 32 to -2 91)

Jordan,Deaths,65 ~ 70,-3 48(-4 11 to -2 85)

Jordan,Deaths,70 ~ 75,-3 14(-3 74 to -2 55)

Jordan,Deaths,75 ~ 80,-2 77(-3 4 to -2 13)

Jordan,Deaths,80 ~ 85,-2 4(-3 13 to -1 66)

Jordan,Deaths,85 ~ 90,-2 02(-2 97 to -1 06)

Jordan,Deaths,90 ~ 95,-1 67(-3 28 to -0 03)

Jordan,Deaths,20 ~ 25,-2 94(-6 4 to 0 65)

Jordan,Deaths,25 ~ 30,-2 77(-5 3 to -0 18)

Jordan,Deaths,30 ~ 35,-2 78(-4 88 to -0 64)

Jordan,Deaths,35 ~ 40,-2 85(-4 63 to -1 04)

Jordan,Deaths,40 ~ 45,-2 96(-4 46 to -1 44)

Jordan,Deaths,45 ~ 50,-3 16(-4 4 to -1 91)

Jordan,Deaths,50 ~ 55,-3 4(-4 4 to -2 39)

Jordan,Deaths,55 ~ 60,-3 56(-4 39 to -2 72)

Jordan,Deaths,60 ~ 65,-3 62(-4 32 to -2 91)

Jordan,Deaths,65 ~ 70,-3 48(-4 11 to -2 85)

Jordan,Deaths,70 ~ 75,-3 14(-3 74 to -2 55)

Jordan,Deaths,75 ~ 80,-2 77(-3 4 to -2 13)

Jordan,Deaths,80 ~ 85,-2 4(-3 13 to -1 66)

Jordan,Deaths,85 ~ 90,-2 02(-2 97 to -1 06)

Jordan,Deaths,90 ~ 95,-1 67(-3 28 to -0 03)

Jordan,Deaths,20 ~ 25,-2 94(-6 4 to 0 65)

Jordan,Deaths,25 ~ 30,-2 77(-5 3 to -0 18)

Jordan,Deaths,30 ~ 35,-2 78(-4 88 to -0 64)

Jordan,Deaths,35 ~ 40,-2 85(-4 63 to -1 04)

Jordan,Deaths,40 ~ 45,-2 96(-4 46 to -1 44)

Jordan,Deaths,45 ~ 50,-3 16(-4 4 to -1 91)

Jordan,Deaths,50 ~ 55,-3 4(-4 4 to -2 39)

Jordan,Deaths,55 ~ 60,-3 56(-4 39 to -2 72)

Jordan,Deaths,60 ~ 65,-3 62(-4 32 to -2 91)

Jordan,Deaths,65 ~ 70,-3 48(-4 11 to -2 85)

Jordan,Deaths,70 ~ 75,-3 14(-3 74 to -2 55)

Jordan,Deaths,75 ~ 80,-2 77(-3 4 to -2 13)

Jordan,Deaths,80 ~ 85,-2 4(-3 13 to -1 66)

Jordan,Deaths,85 ~ 90,-2 02(-2 97 to -1 06)

Jordan,Deaths,90 ~ 95,-1 67(-3 28 to -0 03)

Jordan,DALYs,20 ~ 25,-1 4(-1 68 to -1 11)

Jordan,DALYs,25 ~ 30,-1 36(-1 58 to -1 14)

Jordan,DALYs,30 ~ 35,-1 39(-1 59 to -1 2)

Jordan,DALYs,35 ~ 40,-1 47(-1 65 to -1 3)

Jordan,DALYs,40 ~ 45,-1 6(-1 76 to -1 44)

Jordan,DALYs,45 ~ 50,-1 81(-1 96 to -1 67)

Jordan,DALYs,50 ~ 55,-2 07(-2 2 to -1 94)

Jordan,DALYs,55 ~ 60,-2 29(-2 41 to -2 17)

Jordan,DALYs,60 ~ 65,-2 44(-2 55 to -2 33)

Jordan,DALYs,65 ~ 70,-2 43(-2 54 to -2 32)

Jordan,DALYs,70 ~ 75,-2 24(-2 36 to -2 12)

Jordan,DALYs,75 ~ 80,-1 97(-2 12 to -1 82)

Jordan,DALYs,80 ~ 85,-1 67(-1 86 to -1 47)

Jordan,DALYs,85 ~ 90,-1 34(-1 61 to -1 07)

Jordan,DALYs,90 ~ 95,-1 03(-1 52 to -0 53)

Jordan,DALYs,20 ~ 25,-1 4(-1 68 to -1 11)

Jordan,DALYs,25 ~ 30,-1 36(-1 58 to -1 14)

Jordan,DALYs,30 ~ 35,-1 39(-1 59 to -1 2)

Jordan,DALYs,35 ~ 40,-1 47(-1 65 to -1 3)

Jordan,DALYs,40 ~ 45,-1 6(-1 76 to -1 44)

Jordan,DALYs,45 ~ 50,-1 81(-1 96 to -1 67)

Jordan,DALYs,50 ~ 55,-2 07(-2 2 to -1 94)

Jordan,DALYs,55 ~ 60,-2 29(-2 41 to -2 17)

Jordan,DALYs,60 ~ 65,-2 44(-2 55 to -2 33)

Jordan,DALYs,65 ~ 70,-2 43(-2 54 to -2 32)

Jordan,DALYs,70 ~ 75,-2 24(-2 36 to -2 12)

Jordan,DALYs,75 ~ 80,-1 97(-2 12 to -1 82)

Jordan,DALYs,80 ~ 85,-1 67(-1 86 to -1 47)

Jordan,DALYs,85 ~ 90,-1 34(-1 61 to -1 07)

Jordan,DALYs,90 ~ 95,-1 03(-1 52 to -0 53)

Jordan,DALYs,20 ~ 25,-1 4(-1 68 to -1 11)

Jordan,DALYs,25 ~ 30,-1 36(-1 58 to -1 14)

Jordan,DALYs,30 ~ 35,-1 39(-1 59 to -1 2)

Jordan,DALYs,35 ~ 40,-1 47(-1 65 to -1 3)

Jordan,DALYs,40 ~ 45,-1 6(-1 76 to -1 44)

Jordan,DALYs,45 ~ 50,-1 81(-1 96 to -1 67)

Jordan,DALYs,50 ~ 55,-2 07(-2 2 to -1 94)

Jordan,DALYs,55 ~ 60,-2 29(-2 41 to -2 17)

Jordan,DALYs,60 ~ 65,-2 44(-2 55 to -2 33)

Jordan,DALYs,65 ~ 70,-2 43(-2 54 to -2 32)

Jordan,DALYs,70 ~ 75,-2 24(-2 36 to -2 12)

Jordan,DALYs,75 ~ 80,-1 97(-2 12 to -1 82)

Jordan,DALYs,80 ~ 85,-1 67(-1 86 to -1 47)

Jordan,DALYs,85 ~ 90,-1 34(-1 61 to -1 07)

Jordan,DALYs,90 ~ 95,-1 03(-1 52 to -0 53)

Jordan,DALYs,20 ~ 25,-1 4(-1 68 to -1 11)

Jordan,DALYs,25 ~ 30,-1 36(-1 58 to -1 14)

Jordan,DALYs,30 ~ 35,-1 39(-1 59 to -1 2)

Jordan,DALYs,35 ~ 40,-1 47(-1 65 to -1 3)

Jordan,DALYs,40 ~ 45,-1 6(-1 76 to -1 44)

Jordan,DALYs,45 ~ 50,-1 81(-1 96 to -1 67)

Jordan,DALYs,50 ~ 55,-2 07(-2 2 to -1 94)

Jordan,DALYs,55 ~ 60,-2 29(-2 41 to -2 17)

Jordan,DALYs,60 ~ 65,-2 44(-2 55 to -2 33)

Jordan,DALYs,65 ~ 70,-2 43(-2 54 to -2 32)

Jordan,DALYs,70 ~ 75,-2 24(-2 36 to -2 12)

Jordan,DALYs,75 ~ 80,-1 97(-2 12 to -1 82)

Jordan,DALYs,80 ~ 85,-1 67(-1 86 to -1 47)

Jordan,DALYs,85 ~ 90,-1 34(-1 61 to -1 07)

Jordan,DALYs,90 ~ 95,-1 03(-1 52 to -0 53)

Lebanon,Prevalence,20 ~ 25,0 26(0 13 to 0 4)

Lebanon,Prevalence,25 ~ 30,0 22(0 13 to 0 31)

Lebanon,Prevalence,30 ~ 35,0 23(0 15 to 0 31)

Lebanon,Prevalence,35 ~ 40,0 27(0 2 to 0 34)

Lebanon,Prevalence,40 ~ 45,0 34(0 28 to 0 41)

Lebanon,Prevalence,45 ~ 50,0 45(0 39 to 0 51)

Lebanon,Prevalence,50 ~ 55,0 57(0 52 to 0 62)

Lebanon,Prevalence,55 ~ 60,0 69(0 65 to 0 74)

Lebanon,Prevalence,60 ~ 65,0 82(0 77 to 0 86)

Lebanon,Prevalence,65 ~ 70,0 95(0 91 to 1)

Lebanon,Prevalence,70 ~ 75,1 1(1 05 to 1 14)

Lebanon,Prevalence,75 ~ 80,1 24(1 18 to 1 29)

Lebanon,Prevalence,80 ~ 85,1 37(1 31 to 1 44)

Lebanon,Prevalence,85 ~ 90,1 51(1 41 to 1 6)

Lebanon,Prevalence,90 ~ 95,1 62(1 44 to 1 79)

Lebanon,Prevalence,20 ~ 25,0 26(0 13 to 0 4)

Lebanon,Prevalence,25 ~ 30,0 22(0 13 to 0 31)

Lebanon,Prevalence,30 ~ 35,0 23(0 15 to 0 31)

Lebanon,Prevalence,35 ~ 40,0 27(0 2 to 0 34)

Lebanon,Prevalence,40 ~ 45,0 34(0 28 to 0 41)

Lebanon,Prevalence,45 ~ 50,0 45(0 39 to 0 51)

Lebanon,Prevalence,50 ~ 55,0 57(0 52 to 0 62)

Lebanon,Prevalence,55 ~ 60,0 69(0 65 to 0 74)

Lebanon,Prevalence,60 ~ 65,0 82(0 77 to 0 86)

Lebanon,Prevalence,65 ~ 70,0 95(0 91 to 1)

Lebanon,Prevalence,70 ~ 75,1 1(1 05 to 1 14)

Lebanon,Prevalence,75 ~ 80,1 24(1 18 to 1 29)

Lebanon,Prevalence,80 ~ 85,1 37(1 31 to 1 44)

Lebanon,Prevalence,85 ~ 90,1 51(1 41 to 1 6)

Lebanon,Prevalence,90 ~ 95,1 62(1 44 to 1 79)

Lebanon,Prevalence,20 ~ 25,0 26(0 13 to 0 4)

Lebanon,Prevalence,25 ~ 30,0 22(0 13 to 0 31)

Lebanon,Prevalence,30 ~ 35,0 23(0 15 to 0 31)

Lebanon,Prevalence,35 ~ 40,0 27(0 2 to 0 34)

Lebanon,Prevalence,40 ~ 45,0 34(0 28 to 0 41)

Lebanon,Prevalence,45 ~ 50,0 45(0 39 to 0 51)

Lebanon,Prevalence,50 ~ 55,0 57(0 52 to 0 62)

Lebanon,Prevalence,55 ~ 60,0 69(0 65 to 0 74)

Lebanon,Prevalence,60 ~ 65,0 82(0 77 to 0 86)

Lebanon,Prevalence,65 ~ 70,0 95(0 91 to 1)

Lebanon,Prevalence,70 ~ 75,1 1(1 05 to 1 14)

Lebanon,Prevalence,75 ~ 80,1 24(1 18 to 1 29)

Lebanon,Prevalence,80 ~ 85,1 37(1 31 to 1 44)

Lebanon,Prevalence,85 ~ 90,1 51(1 41 to 1 6)

Lebanon,Prevalence,90 ~ 95,1 62(1 44 to 1 79)

Lebanon,Prevalence,20 ~ 25,0 26(0 13 to 0 4)

Lebanon,Prevalence,25 ~ 30,0 22(0 13 to 0 31)

Lebanon,Prevalence,30 ~ 35,0 23(0 15 to 0 31)

Lebanon,Prevalence,35 ~ 40,0 27(0 2 to 0 34)

Lebanon,Prevalence,40 ~ 45,0 34(0 28 to 0 41)

Lebanon,Prevalence,45 ~ 50,0 45(0 39 to 0 51)

Lebanon,Prevalence,50 ~ 55,0 57(0 52 to 0 62)

Lebanon,Prevalence,55 ~ 60,0 69(0 65 to 0 74)

Lebanon,Prevalence,60 ~ 65,0 82(0 77 to 0 86)

Lebanon,Prevalence,65 ~ 70,0 95(0 91 to 1)

Lebanon,Prevalence,70 ~ 75,1 1(1 05 to 1 14)

Lebanon,Prevalence,75 ~ 80,1 24(1 18 to 1 29)

Lebanon,Prevalence,80 ~ 85,1 37(1 31 to 1 44)

Lebanon,Prevalence,85 ~ 90,1 51(1 41 to 1 6)

Lebanon,Prevalence,90 ~ 95,1 62(1 44 to 1 79)

Lebanon,Deaths,20 ~ 25,-2 19(-6 53 to 2 36)

Lebanon,Deaths,25 ~ 30,-2 59(-5 39 to 0 29)

Lebanon,Deaths,30 ~ 35,-2 84(-5 06 to -0 56)

Lebanon,Deaths,35 ~ 40,-2 86(-4 73 to -0 95)

Lebanon,Deaths,40 ~ 45,-2 68(-4 23 to -1 11)

Lebanon,Deaths,45 ~ 50,-2 53(-3 78 to -1 27)

Lebanon,Deaths,50 ~ 55,-2 44(-3 42 to -1 45)

Lebanon,Deaths,55 ~ 60,-2 39(-3 15 to -1 62)

Lebanon,Deaths,60 ~ 65,-2 32(-2 92 to -1 72)

Lebanon,Deaths,65 ~ 70,-2 14(-2 63 to -1 66)

Lebanon,Deaths,70 ~ 75,-1 84(-2 25 to -1 43)

Lebanon,Deaths,75 ~ 80,-1 42(-1 81 to -1 03)

Lebanon,Deaths,80 ~ 85,-0 99(-1 4 to -0 57)

Lebanon,Deaths,85 ~ 90,-0 53(-1 05 to -0 01)

Lebanon,Deaths,90 ~ 95,-0 01(-0 91 to 0 9)

Lebanon,Deaths,20 ~ 25,-2 19(-6 53 to 2 36)

Lebanon,Deaths,25 ~ 30,-2 59(-5 39 to 0 29)

Lebanon,Deaths,30 ~ 35,-2 84(-5 06 to -0 56)

Lebanon,Deaths,35 ~ 40,-2 86(-4 73 to -0 95)

Lebanon,Deaths,40 ~ 45,-2 68(-4 23 to -1 11)

Lebanon,Deaths,45 ~ 50,-2 53(-3 78 to -1 27)

Lebanon,Deaths,50 ~ 55,-2 44(-3 42 to -1 45)

Lebanon,Deaths,55 ~ 60,-2 39(-3 15 to -1 62)

Lebanon,Deaths,60 ~ 65,-2 32(-2 92 to -1 72)

Lebanon,Deaths,65 ~ 70,-2 14(-2 63 to -1 66)

Lebanon,Deaths,70 ~ 75,-1 84(-2 25 to -1 43)

Lebanon,Deaths,75 ~ 80,-1 42(-1 81 to -1 03)

Lebanon,Deaths,80 ~ 85,-0 99(-1 4 to -0 57)

Lebanon,Deaths,85 ~ 90,-0 53(-1 05 to -0 01)

Lebanon,Deaths,90 ~ 95,-0 01(-0 91 to 0 9)

Lebanon,Deaths,20 ~ 25,-2 19(-6 53 to 2 36)

Lebanon,Deaths,25 ~ 30,-2 59(-5 39 to 0 29)

Lebanon,Deaths,30 ~ 35,-2 84(-5 06 to -0 56)

Lebanon,Deaths,35 ~ 40,-2 86(-4 73 to -0 95)

Lebanon,Deaths,40 ~ 45,-2 68(-4 23 to -1 11)

Lebanon,Deaths,45 ~ 50,-2 53(-3 78 to -1 27)

Lebanon,Deaths,50 ~ 55,-2 44(-3 42 to -1 45)

Lebanon,Deaths,55 ~ 60,-2 39(-3 15 to -1 62)

Lebanon,Deaths,60 ~ 65,-2 32(-2 92 to -1 72)

Lebanon,Deaths,65 ~ 70,-2 14(-2 63 to -1 66)

Lebanon,Deaths,70 ~ 75,-1 84(-2 25 to -1 43)

Lebanon,Deaths,75 ~ 80,-1 42(-1 81 to -1 03)

Lebanon,Deaths,80 ~ 85,-0 99(-1 4 to -0 57)

Lebanon,Deaths,85 ~ 90,-0 53(-1 05 to -0 01)

Lebanon,Deaths,90 ~ 95,-0 01(-0 91 to 0 9)

Lebanon,Deaths,20 ~ 25,-2 19(-6 53 to 2 36)

Lebanon,Deaths,25 ~ 30,-2 59(-5 39 to 0 29)

Lebanon,Deaths,30 ~ 35,-2 84(-5 06 to -0 56)

Lebanon,Deaths,35 ~ 40,-2 86(-4 73 to -0 95)

Lebanon,Deaths,40 ~ 45,-2 68(-4 23 to -1 11)

Lebanon,Deaths,45 ~ 50,-2 53(-3 78 to -1 27)

Lebanon,Deaths,50 ~ 55,-2 44(-3 42 to -1 45)

Lebanon,Deaths,55 ~ 60,-2 39(-3 15 to -1 62)

Lebanon,Deaths,60 ~ 65,-2 32(-2 92 to -1 72)

Lebanon,Deaths,65 ~ 70,-2 14(-2 63 to -1 66)

Lebanon,Deaths,70 ~ 75,-1 84(-2 25 to -1 43)

Lebanon,Deaths,75 ~ 80,-1 42(-1 81 to -1 03)

Lebanon,Deaths,80 ~ 85,-0 99(-1 4 to -0 57)

Lebanon,Deaths,85 ~ 90,-0 53(-1 05 to -0 01)

Lebanon,Deaths,90 ~ 95,-0 01(-0 91 to 0 9)

Lebanon,DALYs,20 ~ 25,-0 97(-1 33 to -0 6)

Lebanon,DALYs,25 ~ 30,-1 18(-1 42 to -0 93)

Lebanon,DALYs,30 ~ 35,-1 32(-1 53 to -1 12)

Lebanon,DALYs,35 ~ 40,-1 39(-1 57 to -1 2)

Lebanon,DALYs,40 ~ 45,-1 34(-1 51 to -1 18)

Lebanon,DALYs,45 ~ 50,-1 3(-1 44 to -1 16)

Lebanon,DALYs,50 ~ 55,-1 3(-1 42 to -1 18)

Lebanon,DALYs,55 ~ 60,-1 35(-1 45 to -1 24)

Lebanon,DALYs,60 ~ 65,-1 4(-1 49 to -1 3)

Lebanon,DALYs,65 ~ 70,-1 35(-1 43 to -1 27)

Lebanon,DALYs,70 ~ 75,-1 19(-1 27 to -1 11)

Lebanon,DALYs,75 ~ 80,-0 89(-0 98 to -0 81)

Lebanon,DALYs,80 ~ 85,-0 55(-0 66 to -0 44)

Lebanon,DALYs,85 ~ 90,-0 16(-0 31 to -0 01)

Lebanon,DALYs,90 ~ 95,0 29(0 01 to 0 57)

Lebanon,DALYs,20 ~ 25,-0 97(-1 33 to -0 6)

Lebanon,DALYs,25 ~ 30,-1 18(-1 42 to -0 93)

Lebanon,DALYs,30 ~ 35,-1 32(-1 53 to -1 12)

Lebanon,DALYs,35 ~ 40,-1 39(-1 57 to -1 2)

Lebanon,DALYs,40 ~ 45,-1 34(-1 51 to -1 18)

Lebanon,DALYs,45 ~ 50,-1 3(-1 44 to -1 16)

Lebanon,DALYs,50 ~ 55,-1 3(-1 42 to -1 18)

Lebanon,DALYs,55 ~ 60,-1 35(-1 45 to -1 24)

Lebanon,DALYs,60 ~ 65,-1 4(-1 49 to -1 3)

Lebanon,DALYs,65 ~ 70,-1 35(-1 43 to -1 27)

Lebanon,DALYs,70 ~ 75,-1 19(-1 27 to -1 11)

Lebanon,DALYs,75 ~ 80,-0 89(-0 98 to -0 81)

Lebanon,DALYs,80 ~ 85,-0 55(-0 66 to -0 44)

Lebanon,DALYs,85 ~ 90,-0 16(-0 31 to -0 01)

Lebanon,DALYs,90 ~ 95,0 29(0 01 to 0 57)

Lebanon,DALYs,20 ~ 25,-0 97(-1 33 to -0 6)

Lebanon,DALYs,25 ~ 30,-1 18(-1 42 to -0 93)

Lebanon,DALYs,30 ~ 35,-1 32(-1 53 to -1 12)

Lebanon,DALYs,35 ~ 40,-1 39(-1 57 to -1 2)

Lebanon,DALYs,40 ~ 45,-1 34(-1 51 to -1 18)

Lebanon,DALYs,45 ~ 50,-1 3(-1 44 to -1 16)

Lebanon,DALYs,50 ~ 55,-1 3(-1 42 to -1 18)

Lebanon,DALYs,55 ~ 60,-1 35(-1 45 to -1 24)

Lebanon,DALYs,60 ~ 65,-1 4(-1 49 to -1 3)

Lebanon,DALYs,65 ~ 70,-1 35(-1 43 to -1 27)

Lebanon,DALYs,70 ~ 75,-1 19(-1 27 to -1 11)

Lebanon,DALYs,75 ~ 80,-0 89(-0 98 to -0 81)

Lebanon,DALYs,80 ~ 85,-0 55(-0 66 to -0 44)

Lebanon,DALYs,85 ~ 90,-0 16(-0 31 to -0 01)

Lebanon,DALYs,90 ~ 95,0 29(0 01 to 0 57)

Lebanon,DALYs,20 ~ 25,-0 97(-1 33 to -0 6)

Lebanon,DALYs,25 ~ 30,-1 18(-1 42 to -0 93)

Lebanon,DALYs,30 ~ 35,-1 32(-1 53 to -1 12)

Lebanon,DALYs,35 ~ 40,-1 39(-1 57 to -1 2)

Lebanon,DALYs,40 ~ 45,-1 34(-1 51 to -1 18)

Lebanon,DALYs,45 ~ 50,-1 3(-1 44 to -1 16)

Lebanon,DALYs,50 ~ 55,-1 3(-1 42 to -1 18)

Lebanon,DALYs,55 ~ 60,-1 35(-1 45 to -1 24)

Lebanon,DALYs,60 ~ 65,-1 4(-1 49 to -1 3)

Lebanon,DALYs,65 ~ 70,-1 35(-1 43 to -1 27)

Lebanon,DALYs,70 ~ 75,-1 19(-1 27 to -1 11)

Lebanon,DALYs,75 ~ 80,-0 89(-0 98 to -0 81)

Lebanon,DALYs,80 ~ 85,-0 55(-0 66 to -0 44)

Lebanon,DALYs,85 ~ 90,-0 16(-0 31 to -0 01)

Lebanon,DALYs,90 ~ 95,0 29(0 01 to 0 57)

Germany,Prevalence,20 ~ 25,-0 38(-0 66 to -0 1)

Germany,Prevalence,25 ~ 30,-0 37(-0 56 to -0 18)

Germany,Prevalence,30 ~ 35,-0 35(-0 5 to -0 2)

Germany,Prevalence,35 ~ 40,-0 34(-0 46 to -0 22)

Germany,Prevalence,40 ~ 45,-0 33(-0 43 to -0 23)

Germany,Prevalence,45 ~ 50,-0 32(-0 4 to -0 23)

Germany,Prevalence,50 ~ 55,-0 28(-0 35 to -0 22)

Germany,Prevalence,55 ~ 60,-0 22(-0 27 to -0 16)

Germany,Prevalence,60 ~ 65,-0 13(-0 17 to -0 08)

Germany,Prevalence,65 ~ 70,-0 03(-0 08 to 0 02)

Germany,Prevalence,70 ~ 75,0 06(0 02 to 0 1)

Germany,Prevalence,75 ~ 80,0 13(0 08 to 0 17)

Germany,Prevalence,80 ~ 85,0 19(0 15 to 0 24)

Germany,Prevalence,85 ~ 90,0 26(0 2 to 0 32)

Germany,Prevalence,90 ~ 95,0 34(0 23 to 0 44)

Germany,Prevalence,20 ~ 25,-0 38(-0 66 to -0 1)

Germany,Prevalence,25 ~ 30,-0 37(-0 56 to -0 18)

Germany,Prevalence,30 ~ 35,-0 35(-0 5 to -0 2)

Germany,Prevalence,35 ~ 40,-0 34(-0 46 to -0 22)

Germany,Prevalence,40 ~ 45,-0 33(-0 43 to -0 23)

Germany,Prevalence,45 ~ 50,-0 32(-0 4 to -0 23)

Germany,Prevalence,50 ~ 55,-0 28(-0 35 to -0 22)

Germany,Prevalence,55 ~ 60,-0 22(-0 27 to -0 16)

Germany,Prevalence,60 ~ 65,-0 13(-0 17 to -0 08)

Germany,Prevalence,65 ~ 70,-0 03(-0 08 to 0 02)

Germany,Prevalence,70 ~ 75,0 06(0 02 to 0 1)

Germany,Prevalence,75 ~ 80,0 13(0 08 to 0 17)

Germany,Prevalence,80 ~ 85,0 19(0 15 to 0 24)

Germany,Prevalence,85 ~ 90,0 26(0 2 to 0 32)

Germany,Prevalence,90 ~ 95,0 34(0 23 to 0 44)

Germany,Prevalence,20 ~ 25,-0 38(-0 66 to -0 1)

Germany,Prevalence,25 ~ 30,-0 37(-0 56 to -0 18)

Germany,Prevalence,30 ~ 35,-0 35(-0 5 to -0 2)

Germany,Prevalence,35 ~ 40,-0 34(-0 46 to -0 22)

Germany,Prevalence,40 ~ 45,-0 33(-0 43 to -0 23)

Germany,Prevalence,45 ~ 50,-0 32(-0 4 to -0 23)

Germany,Prevalence,50 ~ 55,-0 28(-0 35 to -0 22)

Germany,Prevalence,55 ~ 60,-0 22(-0 27 to -0 16)

Germany,Prevalence,60 ~ 65,-0 13(-0 17 to -0 08)

Germany,Prevalence,65 ~ 70,-0 03(-0 08 to 0 02)

Germany,Prevalence,70 ~ 75,0 06(0 02 to 0 1)

Germany,Prevalence,75 ~ 80,0 13(0 08 to 0 17)

Germany,Prevalence,80 ~ 85,0 19(0 15 to 0 24)

Germany,Prevalence,85 ~ 90,0 26(0 2 to 0 32)

Germany,Prevalence,90 ~ 95,0 34(0 23 to 0 44)

Germany,Prevalence,20 ~ 25,-0 38(-0 66 to -0 1)

Germany,Prevalence,25 ~ 30,-0 37(-0 56 to -0 18)

Germany,Prevalence,30 ~ 35,-0 35(-0 5 to -0 2)

Germany,Prevalence,35 ~ 40,-0 34(-0 46 to -0 22)

Germany,Prevalence,40 ~ 45,-0 33(-0 43 to -0 23)

Germany,Prevalence,45 ~ 50,-0 32(-0 4 to -0 23)

Germany,Prevalence,50 ~ 55,-0 28(-0 35 to -0 22)

Germany,Prevalence,55 ~ 60,-0 22(-0 27 to -0 16)

Germany,Prevalence,60 ~ 65,-0 13(-0 17 to -0 08)

Germany,Prevalence,65 ~ 70,-0 03(-0 08 to 0 02)

Germany,Prevalence,70 ~ 75,0 06(0 02 to 0 1)

Germany,Prevalence,75 ~ 80,0 13(0 08 to 0 17)

Germany,Prevalence,80 ~ 85,0 19(0 15 to 0 24)

Germany,Prevalence,85 ~ 90,0 26(0 2 to 0 32)

Germany,Prevalence,90 ~ 95,0 34(0 23 to 0 44)

Germany,Deaths,20 ~ 25,-2 14(-5 35 to 1 18)

Germany,Deaths,25 ~ 30,-2 04(-4 19 to 0 15)

Germany,Deaths,30 ~ 35,-1 64(-3 07 to -0 18)

Germany,Deaths,35 ~ 40,-1 38(-2 38 to -0 37)

Germany,Deaths,40 ~ 45,-0 86(-1 5 to -0 21)

Germany,Deaths,45 ~ 50,-0 29(-0 69 to 0 12)

Germany,Deaths,50 ~ 55,0 46(0 23 to 0 69)

Germany,Deaths,55 ~ 60,0 91(0 76 to 1 06)

Germany,Deaths,60 ~ 65,0 67(0 56 to 0 78)

Germany,Deaths,65 ~ 70,0 29(0 2 to 0 37)

Germany,Deaths,70 ~ 75,-0 02(-0 09 to 0 05)

Germany,Deaths,75 ~ 80,-0 54(-0 61 to -0 47)

Germany,Deaths,80 ~ 85,-0 78(-0 84 to -0 72)

Germany,Deaths,85 ~ 90,-0 55(-0 63 to -0 47)

Germany,Deaths,90 ~ 95,-0 12(-0 26 to 0 02)

Germany,Deaths,20 ~ 25,-2 14(-5 35 to 1 18)

Germany,Deaths,25 ~ 30,-2 04(-4 19 to 0 15)

Germany,Deaths,30 ~ 35,-1 64(-3 07 to -0 18)

Germany,Deaths,35 ~ 40,-1 38(-2 38 to -0 37)

Germany,Deaths,40 ~ 45,-0 86(-1 5 to -0 21)

Germany,Deaths,45 ~ 50,-0 29(-0 69 to 0 12)

Germany,Deaths,50 ~ 55,0 46(0 23 to 0 69)

Germany,Deaths,55 ~ 60,0 91(0 76 to 1 06)

Germany,Deaths,60 ~ 65,0 67(0 56 to 0 78)

Germany,Deaths,65 ~ 70,0 29(0 2 to 0 37)

Germany,Deaths,70 ~ 75,-0 02(-0 09 to 0 05)

Germany,Deaths,75 ~ 80,-0 54(-0 61 to -0 47)

Germany,Deaths,80 ~ 85,-0 78(-0 84 to -0 72)

Germany,Deaths,85 ~ 90,-0 55(-0 63 to -0 47)

Germany,Deaths,90 ~ 95,-0 12(-0 26 to 0 02)

Germany,Deaths,20 ~ 25,-2 14(-5 35 to 1 18)

Germany,Deaths,25 ~ 30,-2 04(-4 19 to 0 15)

Germany,Deaths,30 ~ 35,-1 64(-3 07 to -0 18)

Germany,Deaths,35 ~ 40,-1 38(-2 38 to -0 37)

Germany,Deaths,40 ~ 45,-0 86(-1 5 to -0 21)

Germany,Deaths,45 ~ 50,-0 29(-0 69 to 0 12)

Germany,Deaths,50 ~ 55,0 46(0 23 to 0 69)

Germany,Deaths,55 ~ 60,0 91(0 76 to 1 06)

Germany,Deaths,60 ~ 65,0 67(0 56 to 0 78)

Germany,Deaths,65 ~ 70,0 29(0 2 to 0 37)

Germany,Deaths,70 ~ 75,-0 02(-0 09 to 0 05)

Germany,Deaths,75 ~ 80,-0 54(-0 61 to -0 47)

Germany,Deaths,80 ~ 85,-0 78(-0 84 to -0 72)

Germany,Deaths,85 ~ 90,-0 55(-0 63 to -0 47)

Germany,Deaths,90 ~ 95,-0 12(-0 26 to 0 02)

Germany,Deaths,20 ~ 25,-2 14(-5 35 to 1 18)

Germany,Deaths,25 ~ 30,-2 04(-4 19 to 0 15)

Germany,Deaths,30 ~ 35,-1 64(-3 07 to -0 18)

Germany,Deaths,35 ~ 40,-1 38(-2 38 to -0 37)

Germany,Deaths,40 ~ 45,-0 86(-1 5 to -0 21)

Germany,Deaths,45 ~ 50,-0 29(-0 69 to 0 12)

Germany,Deaths,50 ~ 55,0 46(0 23 to 0 69)

Germany,Deaths,55 ~ 60,0 91(0 76 to 1 06)

Germany,Deaths,60 ~ 65,0 67(0 56 to 0 78)

Germany,Deaths,65 ~ 70,0 29(0 2 to 0 37)

Germany,Deaths,70 ~ 75,-0 02(-0 09 to 0 05)

Germany,Deaths,75 ~ 80,-0 54(-0 61 to -0 47)

Germany,Deaths,80 ~ 85,-0 78(-0 84 to -0 72)

Germany,Deaths,85 ~ 90,-0 55(-0 63 to -0 47)

Germany,Deaths,90 ~ 95,-0 12(-0 26 to 0 02)

Germany,DALYs,20 ~ 25,-0 83(-1 66 to 0)

Germany,DALYs,25 ~ 30,-0 87(-1 43 to -0 31)

Germany,DALYs,30 ~ 35,-0 84(-1 26 to -0 42)

Germany,DALYs,35 ~ 40,-0 81(-1 15 to -0 48)

Germany,DALYs,40 ~ 45,-0 6(-0 86 to -0 34)

Germany,DALYs,45 ~ 50,-0 29(-0 48 to -0 1)

Germany,DALYs,50 ~ 55,0 22(0 09 to 0 35)

Germany,DALYs,55 ~ 60,0 58(0 48 to 0 67)

Germany,DALYs,60 ~ 65,0 46(0 38 to 0 54)

Germany,DALYs,65 ~ 70,0 21(0 14 to 0 28)

Germany,DALYs,70 ~ 75,-0 02(-0 08 to 0 05)

Germany,DALYs,75 ~ 80,-0 41(-0 47 to -0 34)

Germany,DALYs,80 ~ 85,-0 59(-0 66 to -0 51)

Germany,DALYs,85 ~ 90,-0 4(-0 5 to -0 31)

Germany,DALYs,90 ~ 95,-0 05(-0 23 to 0 12)

Germany,DALYs,20 ~ 25,-0 83(-1 66 to 0)

Germany,DALYs,25 ~ 30,-0 87(-1 43 to -0 31)

Germany,DALYs,30 ~ 35,-0 84(-1 26 to -0 42)

Germany,DALYs,35 ~ 40,-0 81(-1 15 to -0 48)

Germany,DALYs,40 ~ 45,-0 6(-0 86 to -0 34)

Germany,DALYs,45 ~ 50,-0 29(-0 48 to -0 1)

Germany,DALYs,50 ~ 55,0 22(0 09 to 0 35)

Germany,DALYs,55 ~ 60,0 58(0 48 to 0 67)

Germany,DALYs,60 ~ 65,0 46(0 38 to 0 54)

Germany,DALYs,65 ~ 70,0 21(0 14 to 0 28)

Germany,DALYs,70 ~ 75,-0 02(-0 08 to 0 05)

Germany,DALYs,75 ~ 80,-0 41(-0 47 to -0 34)

Germany,DALYs,80 ~ 85,-0 59(-0 66 to -0 51)

Germany,DALYs,85 ~ 90,-0 4(-0 5 to -0 31)

Germany,DALYs,90 ~ 95,-0 05(-0 23 to 0 12)

Germany,DALYs,20 ~ 25,-0 83(-1 66 to 0)

Germany,DALYs,25 ~ 30,-0 87(-1 43 to -0 31)

Germany,DALYs,30 ~ 35,-0 84(-1 26 to -0 42)

Germany,DALYs,35 ~ 40,-0 81(-1 15 to -0 48)

Germany,DALYs,40 ~ 45,-0 6(-0 86 to -0 34)

Germany,DALYs,45 ~ 50,-0 29(-0 48 to -0 1)

Germany,DALYs,50 ~ 55,0 22(0 09 to 0 35)

Germany,DALYs,55 ~ 60,0 58(0 48 to 0 67)

Germany,DALYs,60 ~ 65,0 46(0 38 to 0 54)

Germany,DALYs,65 ~ 70,0 21(0 14 to 0 28)

Germany,DALYs,70 ~ 75,-0 02(-0 08 to 0 05)

Germany,DALYs,75 ~ 80,-0 41(-0 47 to -0 34)

Germany,DALYs,80 ~ 85,-0 59(-0 66 to -0 51)

Germany,DALYs,85 ~ 90,-0 4(-0 5 to -0 31)

Germany,DALYs,90 ~ 95,-0 05(-0 23 to 0 12)

Germany,DALYs,20 ~ 25,-0 83(-1 66 to 0)

Germany,DALYs,25 ~ 30,-0 87(-1 43 to -0 31)

Germany,DALYs,30 ~ 35,-0 84(-1 26 to -0 42)

Germany,DALYs,35 ~ 40,-0 81(-1 15 to -0 48)

Germany,DALYs,40 ~ 45,-0 6(-0 86 to -0 34)

Germany,DALYs,45 ~ 50,-0 29(-0 48 to -0 1)

Germany,DALYs,50 ~ 55,0 22(0 09 to 0 35)

Germany,DALYs,55 ~ 60,0 58(0 48 to 0 67)

Germany,DALYs,60 ~ 65,0 46(0 38 to 0 54)

Germany,DALYs,65 ~ 70,0 21(0 14 to 0 28)

Germany,DALYs,70 ~ 75,-0 02(-0 08 to 0 05)

Germany,DALYs,75 ~ 80,-0 41(-0 47 to -0 34)

Germany,DALYs,80 ~ 85,-0 59(-0 66 to -0 51)

Germany,DALYs,85 ~ 90,-0 4(-0 5 to -0 31)

Germany,DALYs,90 ~ 95,-0 05(-0 23 to 0 12)

Senegal,Prevalence,20 ~ 25,-0 18(-0 26 to -0 1)

Senegal,Prevalence,25 ~ 30,-0 16(-0 22 to -0 1)

Senegal,Prevalence,30 ~ 35,-0 12(-0 17 to -0 06)

Senegal,Prevalence,35 ~ 40,-0 06(-0 11 to -0 01)

Senegal,Prevalence,40 ~ 45,0 01(-0 04 to 0 06)

Senegal,Prevalence,45 ~ 50,0 09(0 04 to 0 13)

Senegal,Prevalence,50 ~ 55,0 17(0 13 to 0 22)

Senegal,Prevalence,55 ~ 60,0 26(0 21 to 0 3)

Senegal,Prevalence,60 ~ 65,0 33(0 29 to 0 38)

Senegal,Prevalence,65 ~ 70,0 4(0 36 to 0 45)

Senegal,Prevalence,70 ~ 75,0 47(0 42 to 0 52)

Senegal,Prevalence,75 ~ 80,0 54(0 48 to 0 6)

Senegal,Prevalence,80 ~ 85,0 62(0 54 to 0 7)

Senegal,Prevalence,85 ~ 90,0 73(0 61 to 0 85)

Senegal,Prevalence,90 ~ 95,0 86(0 63 to 1 09)

Senegal,Prevalence,20 ~ 25,-0 18(-0 26 to -0 1)

Senegal,Prevalence,25 ~ 30,-0 16(-0 22 to -0 1)

Senegal,Prevalence,30 ~ 35,-0 12(-0 17 to -0 06)

Senegal,Prevalence,35 ~ 40,-0 06(-0 11 to -0 01)

Senegal,Prevalence,40 ~ 45,0 01(-0 04 to 0 06)

Senegal,Prevalence,45 ~ 50,0 09(0 04 to 0 13)

Senegal,Prevalence,50 ~ 55,0 17(0 13 to 0 22)

Senegal,Prevalence,55 ~ 60,0 26(0 21 to 0 3)

Senegal,Prevalence,60 ~ 65,0 33(0 29 to 0 38)

Senegal,Prevalence,65 ~ 70,0 4(0 36 to 0 45)

Senegal,Prevalence,70 ~ 75,0 47(0 42 to 0 52)

Senegal,Prevalence,75 ~ 80,0 54(0 48 to 0 6)

Senegal,Prevalence,80 ~ 85,0 62(0 54 to 0 7)

Senegal,Prevalence,85 ~ 90,0 73(0 61 to 0 85)

Senegal,Prevalence,90 ~ 95,0 86(0 63 to 1 09)

Senegal,Prevalence,20 ~ 25,-0 18(-0 26 to -0 1)

Senegal,Prevalence,25 ~ 30,-0 16(-0 22 to -0 1)

Senegal,Prevalence,30 ~ 35,-0 12(-0 17 to -0 06)

Senegal,Prevalence,35 ~ 40,-0 06(-0 11 to -0 01)

Senegal,Prevalence,40 ~ 45,0 01(-0 04 to 0 06)

Senegal,Prevalence,45 ~ 50,0 09(0 04 to 0 13)

Senegal,Prevalence,50 ~ 55,0 17(0 13 to 0 22)

Senegal,Prevalence,55 ~ 60,0 26(0 21 to 0 3)

Senegal,Prevalence,60 ~ 65,0 33(0 29 to 0 38)

Senegal,Prevalence,65 ~ 70,0 4(0 36 to 0 45)

Senegal,Prevalence,70 ~ 75,0 47(0 42 to 0 52)

Senegal,Prevalence,75 ~ 80,0 54(0 48 to 0 6)

Senegal,Prevalence,80 ~ 85,0 62(0 54 to 0 7)

Senegal,Prevalence,85 ~ 90,0 73(0 61 to 0 85)

Senegal,Prevalence,90 ~ 95,0 86(0 63 to 1 09)

Senegal,Prevalence,20 ~ 25,-0 18(-0 26 to -0 1)

Senegal,Prevalence,25 ~ 30,-0 16(-0 22 to -0 1)

Senegal,Prevalence,30 ~ 35,-0 12(-0 17 to -0 06)

Senegal,Prevalence,35 ~ 40,-0 06(-0 11 to -0 01)

Senegal,Prevalence,40 ~ 45,0 01(-0 04 to 0 06)

Senegal,Prevalence,45 ~ 50,0 09(0 04 to 0 13)

Senegal,Prevalence,50 ~ 55,0 17(0 13 to 0 22)

Senegal,Prevalence,55 ~ 60,0 26(0 21 to 0 3)

Senegal,Prevalence,60 ~ 65,0 33(0 29 to 0 38)

Senegal,Prevalence,65 ~ 70,0 4(0 36 to 0 45)

Senegal,Prevalence,70 ~ 75,0 47(0 42 to 0 52)

Senegal,Prevalence,75 ~ 80,0 54(0 48 to 0 6)

Senegal,Prevalence,80 ~ 85,0 62(0 54 to 0 7)

Senegal,Prevalence,85 ~ 90,0 73(0 61 to 0 85)

Senegal,Prevalence,90 ~ 95,0 86(0 63 to 1 09)

Senegal,Deaths,20 ~ 25,-0 27(-3 16 to 2 7)

Senegal,Deaths,25 ~ 30,-0 25(-1 98 to 1 51)

Senegal,Deaths,30 ~ 35,-0 23(-1 62 to 1 19)

Senegal,Deaths,35 ~ 40,-0 27(-1 46 to 0 95)

Senegal,Deaths,40 ~ 45,-0 38(-1 36 to 0 61)

Senegal,Deaths,45 ~ 50,-0 51(-1 27 to 0 26)

Senegal,Deaths,50 ~ 55,-0 58(-1 19 to 0 04)

Senegal,Deaths,55 ~ 60,-0 6(-1 09 to -0 1)

Senegal,Deaths,60 ~ 65,-0 59(-1 to -0 18)

Senegal,Deaths,65 ~ 70,-0 57(-0 92 to -0 22)

Senegal,Deaths,70 ~ 75,-0 54(-0 86 to -0 22)

Senegal,Deaths,75 ~ 80,-0 46(-0 79 to -0 12)

Senegal,Deaths,80 ~ 85,-0 36(-0 76 to 0 05)

Senegal,Deaths,85 ~ 90,-0 22(-0 76 to 0 33)

Senegal,Deaths,90 ~ 95,0(-0 92 to 0 93)

Senegal,Deaths,20 ~ 25,-0 27(-3 16 to 2 7)

Senegal,Deaths,25 ~ 30,-0 25(-1 98 to 1 51)

Senegal,Deaths,30 ~ 35,-0 23(-1 62 to 1 19)

Senegal,Deaths,35 ~ 40,-0 27(-1 46 to 0 95)

Senegal,Deaths,40 ~ 45,-0 38(-1 36 to 0 61)

Senegal,Deaths,45 ~ 50,-0 51(-1 27 to 0 26)

Senegal,Deaths,50 ~ 55,-0 58(-1 19 to 0 04)

Senegal,Deaths,55 ~ 60,-0 6(-1 09 to -0 1)

Senegal,Deaths,60 ~ 65,-0 59(-1 to -0 18)

Senegal,Deaths,65 ~ 70,-0 57(-0 92 to -0 22)

Senegal,Deaths,70 ~ 75,-0 54(-0 86 to -0 22)

Senegal,Deaths,75 ~ 80,-0 46(-0 79 to -0 12)

Senegal,Deaths,80 ~ 85,-0 36(-0 76 to 0 05)

Senegal,Deaths,85 ~ 90,-0 22(-0 76 to 0 33)

Senegal,Deaths,90 ~ 95,0(-0 92 to 0 93)

Senegal,Deaths,20 ~ 25,-0 27(-3 16 to 2 7)

Senegal,Deaths,25 ~ 30,-0 25(-1 98 to 1 51)

Senegal,Deaths,30 ~ 35,-0 23(-1 62 to 1 19)

Senegal,Deaths,35 ~ 40,-0 27(-1 46 to 0 95)

Senegal,Deaths,40 ~ 45,-0 38(-1 36 to 0 61)

Senegal,Deaths,45 ~ 50,-0 51(-1 27 to 0 26)

Senegal,Deaths,50 ~ 55,-0 58(-1 19 to 0 04)

Senegal,Deaths,55 ~ 60,-0 6(-1 09 to -0 1)

Senegal,Deaths,60 ~ 65,-0 59(-1 to -0 18)

Senegal,Deaths,65 ~ 70,-0 57(-0 92 to -0 22)

Senegal,Deaths,70 ~ 75,-0 54(-0 86 to -0 22)

Senegal,Deaths,75 ~ 80,-0 46(-0 79 to -0 12)

Senegal,Deaths,80 ~ 85,-0 36(-0 76 to 0 05)

Senegal,Deaths,85 ~ 90,-0 22(-0 76 to 0 33)

Senegal,Deaths,90 ~ 95,0(-0 92 to 0 93)

Senegal,Deaths,20 ~ 25,-0 27(-3 16 to 2 7)

Senegal,Deaths,25 ~ 30,-0 25(-1 98 to 1 51)

Senegal,Deaths,30 ~ 35,-0 23(-1 62 to 1 19)

Senegal,Deaths,35 ~ 40,-0 27(-1 46 to 0 95)

Senegal,Deaths,40 ~ 45,-0 38(-1 36 to 0 61)

Senegal,Deaths,45 ~ 50,-0 51(-1 27 to 0 26)

Senegal,Deaths,50 ~ 55,-0 58(-1 19 to 0 04)

Senegal,Deaths,55 ~ 60,-0 6(-1 09 to -0 1)

Senegal,Deaths,60 ~ 65,-0 59(-1 to -0 18)

Senegal,Deaths,65 ~ 70,-0 57(-0 92 to -0 22)

Senegal,Deaths,70 ~ 75,-0 54(-0 86 to -0 22)

Senegal,Deaths,75 ~ 80,-0 46(-0 79 to -0 12)

Senegal,Deaths,80 ~ 85,-0 36(-0 76 to 0 05)

Senegal,Deaths,85 ~ 90,-0 22(-0 76 to 0 33)

Senegal,Deaths,90 ~ 95,0(-0 92 to 0 93)

Senegal,DALYs,20 ~ 25,-0 29(-0 54 to -0 04)

Senegal,DALYs,25 ~ 30,-0 24(-0 42 to -0 06)

Senegal,DALYs,30 ~ 35,-0 19(-0 35 to -0 04)

Senegal,DALYs,35 ~ 40,-0 17(-0 31 to -0 03)

Senegal,DALYs,40 ~ 45,-0 2(-0 33 to -0 07)

Senegal,DALYs,45 ~ 50,-0 26(-0 38 to -0 15)

Senegal,DALYs,50 ~ 55,-0 31(-0 41 to -0 21)

Senegal,DALYs,55 ~ 60,-0 32(-0 41 to -0 23)

Senegal,DALYs,60 ~ 65,-0 33(-0 41 to -0 25)

Senegal,DALYs,65 ~ 70,-0 35(-0 42 to -0 27)

Senegal,DALYs,70 ~ 75,-0 37(-0 45 to -0 29)

Senegal,DALYs,75 ~ 80,-0 33(-0 43 to -0 23)

Senegal,DALYs,80 ~ 85,-0 26(-0 39 to -0 13)

Senegal,DALYs,85 ~ 90,-0 14(-0 34 to 0 05)

Senegal,DALYs,90 ~ 95,0 05(-0 3 to 0 4)

Senegal,DALYs,20 ~ 25,-0 29(-0 54 to -0 04)

Senegal,DALYs,25 ~ 30,-0 24(-0 42 to -0 06)

Senegal,DALYs,30 ~ 35,-0 19(-0 35 to -0 04)

Senegal,DALYs,35 ~ 40,-0 17(-0 31 to -0 03)

Senegal,DALYs,40 ~ 45,-0 2(-0 33 to -0 07)

Senegal,DALYs,45 ~ 50,-0 26(-0 38 to -0 15)

Senegal,DALYs,50 ~ 55,-0 31(-0 41 to -0 21)

Senegal,DALYs,55 ~ 60,-0 32(-0 41 to -0 23)

Senegal,DALYs,60 ~ 65,-0 33(-0 41 to -0 25)

Senegal,DALYs,65 ~ 70,-0 35(-0 42 to -0 27)

Senegal,DALYs,70 ~ 75,-0 37(-0 45 to -0 29)

Senegal,DALYs,75 ~ 80,-0 33(-0 43 to -0 23)

Senegal,DALYs,80 ~ 85,-0 26(-0 39 to -0 13)

Senegal,DALYs,85 ~ 90,-0 14(-0 34 to 0 05)

Senegal,DALYs,90 ~ 95,0 05(-0 3 to 0 4)

Senegal,DALYs,20 ~ 25,-0 29(-0 54 to -0 04)

Senegal,DALYs,25 ~ 30,-0 24(-0 42 to -0 06)

Senegal,DALYs,30 ~ 35,-0 19(-0 35 to -0 04)

Senegal,DALYs,35 ~ 40,-0 17(-0 31 to -0 03)

Senegal,DALYs,40 ~ 45,-0 2(-0 33 to -0 07)

Senegal,DALYs,45 ~ 50,-0 26(-0 38 to -0 15)

Senegal,DALYs,50 ~ 55,-0 31(-0 41 to -0 21)

Senegal,DALYs,55 ~ 60,-0 32(-0 41 to -0 23)

Senegal,DALYs,60 ~ 65,-0 33(-0 41 to -0 25)

Senegal,DALYs,65 ~ 70,-0 35(-0 42 to -0 27)

Senegal,DALYs,70 ~ 75,-0 37(-0 45 to -0 29)

Senegal,DALYs,75 ~ 80,-0 33(-0 43 to -0 23)

Senegal,DALYs,80 ~ 85,-0 26(-0 39 to -0 13)

Senegal,DALYs,85 ~ 90,-0 14(-0 34 to 0 05)

Senegal,DALYs,90 ~ 95,0 05(-0 3 to 0 4)

Senegal,DALYs,20 ~ 25,-0 29(-0 54 to -0 04)

Senegal,DALYs,25 ~ 30,-0 24(-0 42 to -0 06)

Senegal,DALYs,30 ~ 35,-0 19(-0 35 to -0 04)

Senegal,DALYs,35 ~ 40,-0 17(-0 31 to -0 03)

Senegal,DALYs,40 ~ 45,-0 2(-0 33 to -0 07)

Senegal,DALYs,45 ~ 50,-0 26(-0 38 to -0 15)

Senegal,DALYs,50 ~ 55,-0 31(-0 41 to -0 21)

Senegal,DALYs,55 ~ 60,-0 32(-0 41 to -0 23)

Senegal,DALYs,60 ~ 65,-0 33(-0 41 to -0 25)

Senegal,DALYs,65 ~ 70,-0 35(-0 42 to -0 27)

Senegal,DALYs,70 ~ 75,-0 37(-0 45 to -0 29)

Senegal,DALYs,75 ~ 80,-0 33(-0 43 to -0 23)

Senegal,DALYs,80 ~ 85,-0 26(-0 39 to -0 13)

Senegal,DALYs,85 ~ 90,-0 14(-0 34 to 0 05)

Senegal,DALYs,90 ~ 95,0 05(-0 3 to 0 4)

Togo,Prevalence,20 ~ 25,-0 15(-0 25 to -0 04)

Togo,Prevalence,25 ~ 30,-0 12(-0 2 to -0 03)

Togo,Prevalence,30 ~ 35,-0 08(-0 16 to -0 01)

Togo,Prevalence,35 ~ 40,-0 03(-0 1 to 0 04)

Togo,Prevalence,40 ~ 45,0 03(-0 03 to 0 1)

Togo,Prevalence,45 ~ 50,0 12(0 05 to 0 18)

Togo,Prevalence,50 ~ 55,0 2(0 13 to 0 26)

Togo,Prevalence,55 ~ 60,0 26(0 2 to 0 33)

Togo,Prevalence,60 ~ 65,0 33(0 27 to 0 4)

Togo,Prevalence,65 ~ 70,0 39(0 32 to 0 46)

Togo,Prevalence,70 ~ 75,0 44(0 35 to 0 52)

Togo,Prevalence,75 ~ 80,0 48(0 37 to 0 58)

Togo,Prevalence,80 ~ 85,0 52(0 38 to 0 66)

Togo,Prevalence,85 ~ 90,0 58(0 36 to 0 8)

Togo,Prevalence,90 ~ 95,0 66(0 21 to 1 1)

Togo,Prevalence,20 ~ 25,-0 15(-0 25 to -0 04)

Togo,Prevalence,25 ~ 30,-0 12(-0 2 to -0 03)

Togo,Prevalence,30 ~ 35,-0 08(-0 16 to -0 01)

Togo,Prevalence,35 ~ 40,-0 03(-0 1 to 0 04)

Togo,Prevalence,40 ~ 45,0 03(-0 03 to 0 1)

Togo,Prevalence,45 ~ 50,0 12(0 05 to 0 18)

Togo,Prevalence,50 ~ 55,0 2(0 13 to 0 26)

Togo,Prevalence,55 ~ 60,0 26(0 2 to 0 33)

Togo,Prevalence,60 ~ 65,0 33(0 27 to 0 4)

Togo,Prevalence,65 ~ 70,0 39(0 32 to 0 46)

Togo,Prevalence,70 ~ 75,0 44(0 35 to 0 52)

Togo,Prevalence,75 ~ 80,0 48(0 37 to 0 58)

Togo,Prevalence,80 ~ 85,0 52(0 38 to 0 66)

Togo,Prevalence,85 ~ 90,0 58(0 36 to 0 8)

Togo,Prevalence,90 ~ 95,0 66(0 21 to 1 1)

Togo,Prevalence,20 ~ 25,-0 15(-0 25 to -0 04)

Togo,Prevalence,25 ~ 30,-0 12(-0 2 to -0 03)

Togo,Prevalence,30 ~ 35,-0 08(-0 16 to -0 01)

Togo,Prevalence,35 ~ 40,-0 03(-0 1 to 0 04)

Togo,Prevalence,40 ~ 45,0 03(-0 03 to 0 1)

Togo,Prevalence,45 ~ 50,0 12(0 05 to 0 18)

Togo,Prevalence,50 ~ 55,0 2(0 13 to 0 26)

Togo,Prevalence,55 ~ 60,0 26(0 2 to 0 33)

Togo,Prevalence,60 ~ 65,0 33(0 27 to 0 4)

Togo,Prevalence,65 ~ 70,0 39(0 32 to 0 46)

Togo,Prevalence,70 ~ 75,0 44(0 35 to 0 52)

Togo,Prevalence,75 ~ 80,0 48(0 37 to 0 58)

Togo,Prevalence,80 ~ 85,0 52(0 38 to 0 66)

Togo,Prevalence,85 ~ 90,0 58(0 36 to 0 8)

Togo,Prevalence,90 ~ 95,0 66(0 21 to 1 1)

Togo,Prevalence,20 ~ 25,-0 15(-0 25 to -0 04)

Togo,Prevalence,25 ~ 30,-0 12(-0 2 to -0 03)

Togo,Prevalence,30 ~ 35,-0 08(-0 16 to -0 01)

Togo,Prevalence,35 ~ 40,-0 03(-0 1 to 0 04)

Togo,Prevalence,40 ~ 45,0 03(-0 03 to 0 1)

Togo,Prevalence,45 ~ 50,0 12(0 05 to 0 18)

Togo,Prevalence,50 ~ 55,0 2(0 13 to 0 26)

Togo,Prevalence,55 ~ 60,0 26(0 2 to 0 33)

Togo,Prevalence,60 ~ 65,0 33(0 27 to 0 4)

Togo,Prevalence,65 ~ 70,0 39(0 32 to 0 46)

Togo,Prevalence,70 ~ 75,0 44(0 35 to 0 52)

Togo,Prevalence,75 ~ 80,0 48(0 37 to 0 58)

Togo,Prevalence,80 ~ 85,0 52(0 38 to 0 66)

Togo,Prevalence,85 ~ 90,0 58(0 36 to 0 8)

Togo,Prevalence,90 ~ 95,0 66(0 21 to 1 1)

Togo,Deaths,20 ~ 25,0 13(-3 51 to 3 91)

Togo,Deaths,25 ~ 30,0 04(-2 1 to 2 23)

Togo,Deaths,30 ~ 35,-0 08(-1 76 to 1 64)

Togo,Deaths,35 ~ 40,-0 12(-1 55 to 1 33)

Togo,Deaths,40 ~ 45,-0 16(-1 32 to 1 03)

Togo,Deaths,45 ~ 50,-0 16(-1 09 to 0 77)

Togo,Deaths,50 ~ 55,-0 2(-0 97 to 0 57)

Togo,Deaths,55 ~ 60,-0 27(-0 93 to 0 39)

Togo,Deaths,60 ~ 65,-0 35(-0 92 to 0 22)

Togo,Deaths,65 ~ 70,-0 45(-0 96 to 0 07)

Togo,Deaths,70 ~ 75,-0 53(-1 03 to -0 03)

Togo,Deaths,75 ~ 80,-0 55(-1 1 to -0 01)

Togo,Deaths,80 ~ 85,-0 52(-1 2 to 0 16)

Togo,Deaths,85 ~ 90,-0 53(-1 47 to 0 42)

Togo,Deaths,90 ~ 95,-0 52(-2 23 to 1 22)

Togo,Deaths,20 ~ 25,0 13(-3 51 to 3 91)

Togo,Deaths,25 ~ 30,0 04(-2 1 to 2 23)

Togo,Deaths,30 ~ 35,-0 08(-1 76 to 1 64)

Togo,Deaths,35 ~ 40,-0 12(-1 55 to 1 33)

Togo,Deaths,40 ~ 45,-0 16(-1 32 to 1 03)

Togo,Deaths,45 ~ 50,-0 16(-1 09 to 0 77)

Togo,Deaths,50 ~ 55,-0 2(-0 97 to 0 57)

Togo,Deaths,55 ~ 60,-0 27(-0 93 to 0 39)

Togo,Deaths,60 ~ 65,-0 35(-0 92 to 0 22)

Togo,Deaths,65 ~ 70,-0 45(-0 96 to 0 07)

Togo,Deaths,70 ~ 75,-0 53(-1 03 to -0 03)

Togo,Deaths,75 ~ 80,-0 55(-1 1 to -0 01)

Togo,Deaths,80 ~ 85,-0 52(-1 2 to 0 16)

Togo,Deaths,85 ~ 90,-0 53(-1 47 to 0 42)

Togo,Deaths,90 ~ 95,-0 52(-2 23 to 1 22)

Togo,Deaths,20 ~ 25,0 13(-3 51 to 3 91)

Togo,Deaths,25 ~ 30,0 04(-2 1 to 2 23)

Togo,Deaths,30 ~ 35,-0 08(-1 76 to 1 64)

Togo,Deaths,35 ~ 40,-0 12(-1 55 to 1 33)

Togo,Deaths,40 ~ 45,-0 16(-1 32 to 1 03)

Togo,Deaths,45 ~ 50,-0 16(-1 09 to 0 77)

Togo,Deaths,50 ~ 55,-0 2(-0 97 to 0 57)

Togo,Deaths,55 ~ 60,-0 27(-0 93 to 0 39)

Togo,Deaths,60 ~ 65,-0 35(-0 92 to 0 22)

Togo,Deaths,65 ~ 70,-0 45(-0 96 to 0 07)

Togo,Deaths,70 ~ 75,-0 53(-1 03 to -0 03)

Togo,Deaths,75 ~ 80,-0 55(-1 1 to -0 01)

Togo,Deaths,80 ~ 85,-0 52(-1 2 to 0 16)

Togo,Deaths,85 ~ 90,-0 53(-1 47 to 0 42)

Togo,Deaths,90 ~ 95,-0 52(-2 23 to 1 22)

Togo,Deaths,20 ~ 25,0 13(-3 51 to 3 91)

Togo,Deaths,25 ~ 30,0 04(-2 1 to 2 23)

Togo,Deaths,30 ~ 35,-0 08(-1 76 to 1 64)

Togo,Deaths,35 ~ 40,-0 12(-1 55 to 1 33)

Togo,Deaths,40 ~ 45,-0 16(-1 32 to 1 03)

Togo,Deaths,45 ~ 50,-0 16(-1 09 to 0 77)

Togo,Deaths,50 ~ 55,-0 2(-0 97 to 0 57)

Togo,Deaths,55 ~ 60,-0 27(-0 93 to 0 39)

Togo,Deaths,60 ~ 65,-0 35(-0 92 to 0 22)

Togo,Deaths,65 ~ 70,-0 45(-0 96 to 0 07)

Togo,Deaths,70 ~ 75,-0 53(-1 03 to -0 03)

Togo,Deaths,75 ~ 80,-0 55(-1 1 to -0 01)

Togo,Deaths,80 ~ 85,-0 52(-1 2 to 0 16)

Togo,Deaths,85 ~ 90,-0 53(-1 47 to 0 42)

Togo,Deaths,90 ~ 95,-0 52(-2 23 to 1 22)

Togo,DALYs,20 ~ 25,0 06(-0 21 to 0 33)

Togo,DALYs,25 ~ 30,0 01(-0 18 to 0 2)

Togo,DALYs,30 ~ 35,-0 05(-0 21 to 0 11)

Togo,DALYs,35 ~ 40,-0 05(-0 2 to 0 1)

Togo,DALYs,40 ~ 45,-0 05(-0 18 to 0 08)

Togo,DALYs,45 ~ 50,-0 03(-0 15 to 0 09)

Togo,DALYs,50 ~ 55,-0 05(-0 16 to 0 05)

Togo,DALYs,55 ~ 60,-0 11(-0 21 to -0 01)

Togo,DALYs,60 ~ 65,-0 18(-0 28 to -0 08)

Togo,DALYs,65 ~ 70,-0 29(-0 38 to -0 19)

Togo,DALYs,70 ~ 75,-0 39(-0 49 to -0 28)

Togo,DALYs,75 ~ 80,-0 42(-0 55 to -0 29)

Togo,DALYs,80 ~ 85,-0 39(-0 57 to -0 22)

Togo,DALYs,85 ~ 90,-0 4(-0 68 to -0 13)

Togo,DALYs,90 ~ 95,-0 39(-0 92 to 0 15)

Togo,DALYs,20 ~ 25,0 06(-0 21 to 0 33)

Togo,DALYs,25 ~ 30,0 01(-0 18 to 0 2)

Togo,DALYs,30 ~ 35,-0 05(-0 21 to 0 11)

Togo,DALYs,35 ~ 40,-0 05(-0 2 to 0 1)

Togo,DALYs,40 ~ 45,-0 05(-0 18 to 0 08)

Togo,DALYs,45 ~ 50,-0 03(-0 15 to 0 09)

Togo,DALYs,50 ~ 55,-0 05(-0 16 to 0 05)

Togo,DALYs,55 ~ 60,-0 11(-0 21 to -0 01)

Togo,DALYs,60 ~ 65,-0 18(-0 28 to -0 08)

Togo,DALYs,65 ~ 70,-0 29(-0 38 to -0 19)

Togo,DALYs,70 ~ 75,-0 39(-0 49 to -0 28)

Togo,DALYs,75 ~ 80,-0 42(-0 55 to -0 29)

Togo,DALYs,80 ~ 85,-0 39(-0 57 to -0 22)

Togo,DALYs,85 ~ 90,-0 4(-0 68 to -0 13)

Togo,DALYs,90 ~ 95,-0 39(-0 92 to 0 15)

Togo,DALYs,20 ~ 25,0 06(-0 21 to 0 33)

Togo,DALYs,25 ~ 30,0 01(-0 18 to 0 2)

Togo,DALYs,30 ~ 35,-0 05(-0 21 to 0 11)

Togo,DALYs,35 ~ 40,-0 05(-0 2 to 0 1)

Togo,DALYs,40 ~ 45,-0 05(-0 18 to 0 08)

Togo,DALYs,45 ~ 50,-0 03(-0 15 to 0 09)

Togo,DALYs,50 ~ 55,-0 05(-0 16 to 0 05)

Togo,DALYs,55 ~ 60,-0 11(-0 21 to -0 01)

Togo,DALYs,60 ~ 65,-0 18(-0 28 to -0 08)

Togo,DALYs,65 ~ 70,-0 29(-0 38 to -0 19)

Togo,DALYs,70 ~ 75,-0 39(-0 49 to -0 28)

Togo,DALYs,75 ~ 80,-0 42(-0 55 to -0 29)

Togo,DALYs,80 ~ 85,-0 39(-0 57 to -0 22)

Togo,DALYs,85 ~ 90,-0 4(-0 68 to -0 13)

Togo,DALYs,90 ~ 95,-0 39(-0 92 to 0 15)

Togo,DALYs,20 ~ 25,0 06(-0 21 to 0 33)

Togo,DALYs,25 ~ 30,0 01(-0 18 to 0 2)

Togo,DALYs,30 ~ 35,-0 05(-0 21 to 0 11)

Togo,DALYs,35 ~ 40,-0 05(-0 2 to 0 1)

Togo,DALYs,40 ~ 45,-0 05(-0 18 to 0 08)

Togo,DALYs,45 ~ 50,-0 03(-0 15 to 0 09)

Togo,DALYs,50 ~ 55,-0 05(-0 16 to 0 05)

Togo,DALYs,55 ~ 60,-0 11(-0 21 to -0 01)

Togo,DALYs,60 ~ 65,-0 18(-0 28 to -0 08)

Togo,DALYs,65 ~ 70,-0 29(-0 38 to -0 19)

Togo,DALYs,70 ~ 75,-0 39(-0 49 to -0 28)

Togo,DALYs,75 ~ 80,-0 42(-0 55 to -0 29)

Togo,DALYs,80 ~ 85,-0 39(-0 57 to -0 22)

Togo,DALYs,85 ~ 90,-0 4(-0 68 to -0 13)

Togo,DALYs,90 ~ 95,-0 39(-0 92 to 0 15)

Morocco,Prevalence,20 ~ 25,-0 02(-0 11 to 0 07)

Morocco,Prevalence,25 ~ 30,0 01(-0 05 to 0 08)

Morocco,Prevalence,30 ~ 35,0 09(0 03 to 0 14)

Morocco,Prevalence,35 ~ 40,0 19(0 14 to 0 24)

Morocco,Prevalence,40 ~ 45,0 33(0 29 to 0 38)

Morocco,Prevalence,45 ~ 50,0 49(0 45 to 0 53)

Morocco,Prevalence,50 ~ 55,0 64(0 61 to 0 68)

Morocco,Prevalence,55 ~ 60,0 78(0 74 to 0 81)

Morocco,Prevalence,60 ~ 65,0 91(0 87 to 0 94)

Morocco,Prevalence,65 ~ 70,1 04(1 01 to 1 08)

Morocco,Prevalence,70 ~ 75,1 17(1 13 to 1 21)

Morocco,Prevalence,75 ~ 80,1 29(1 25 to 1 34)

Morocco,Prevalence,80 ~ 85,1 42(1 36 to 1 47)

Morocco,Prevalence,85 ~ 90,1 55(1 47 to 1 62)

Morocco,Prevalence,90 ~ 95,1 66(1 52 to 1 81)

Morocco,Prevalence,20 ~ 25,-0 02(-0 11 to 0 07)

Morocco,Prevalence,25 ~ 30,0 01(-0 05 to 0 08)

Morocco,Prevalence,30 ~ 35,0 09(0 03 to 0 14)

Morocco,Prevalence,35 ~ 40,0 19(0 14 to 0 24)

Morocco,Prevalence,40 ~ 45,0 33(0 29 to 0 38)

Morocco,Prevalence,45 ~ 50,0 49(0 45 to 0 53)

Morocco,Prevalence,50 ~ 55,0 64(0 61 to 0 68)

Morocco,Prevalence,55 ~ 60,0 78(0 74 to 0 81)

Morocco,Prevalence,60 ~ 65,0 91(0 87 to 0 94)

Morocco,Prevalence,65 ~ 70,1 04(1 01 to 1 08)

Morocco,Prevalence,70 ~ 75,1 17(1 13 to 1 21)

Morocco,Prevalence,75 ~ 80,1 29(1 25 to 1 34)

Morocco,Prevalence,80 ~ 85,1 42(1 36 to 1 47)

Morocco,Prevalence,85 ~ 90,1 55(1 47 to 1 62)

Morocco,Prevalence,90 ~ 95,1 66(1 52 to 1 81)

Morocco,Prevalence,20 ~ 25,-0 02(-0 11 to 0 07)

Morocco,Prevalence,25 ~ 30,0 01(-0 05 to 0 08)

Morocco,Prevalence,30 ~ 35,0 09(0 03 to 0 14)

Morocco,Prevalence,35 ~ 40,0 19(0 14 to 0 24)

Morocco,Prevalence,40 ~ 45,0 33(0 29 to 0 38)

Morocco,Prevalence,45 ~ 50,0 49(0 45 to 0 53)

Morocco,Prevalence,50 ~ 55,0 64(0 61 to 0 68)

Morocco,Prevalence,55 ~ 60,0 78(0 74 to 0 81)

Morocco,Prevalence,60 ~ 65,0 91(0 87 to 0 94)

Morocco,Prevalence,65 ~ 70,1 04(1 01 to 1 08)

Morocco,Prevalence,70 ~ 75,1 17(1 13 to 1 21)

Morocco,Prevalence,75 ~ 80,1 29(1 25 to 1 34)

Morocco,Prevalence,80 ~ 85,1 42(1 36 to 1 47)

Morocco,Prevalence,85 ~ 90,1 55(1 47 to 1 62)

Morocco,Prevalence,90 ~ 95,1 66(1 52 to 1 81)

Morocco,Prevalence,20 ~ 25,-0 02(-0 11 to 0 07)

Morocco,Prevalence,25 ~ 30,0 01(-0 05 to 0 08)

Morocco,Prevalence,30 ~ 35,0 09(0 03 to 0 14)

Morocco,Prevalence,35 ~ 40,0 19(0 14 to 0 24)

Morocco,Prevalence,40 ~ 45,0 33(0 29 to 0 38)

Morocco,Prevalence,45 ~ 50,0 49(0 45 to 0 53)

Morocco,Prevalence,50 ~ 55,0 64(0 61 to 0 68)

Morocco,Prevalence,55 ~ 60,0 78(0 74 to 0 81)

Morocco,Prevalence,60 ~ 65,0 91(0 87 to 0 94)

Morocco,Prevalence,65 ~ 70,1 04(1 01 to 1 08)

Morocco,Prevalence,70 ~ 75,1 17(1 13 to 1 21)

Morocco,Prevalence,75 ~ 80,1 29(1 25 to 1 34)

Morocco,Prevalence,80 ~ 85,1 42(1 36 to 1 47)

Morocco,Prevalence,85 ~ 90,1 55(1 47 to 1 62)

Morocco,Prevalence,90 ~ 95,1 66(1 52 to 1 81)

Morocco,Deaths,20 ~ 25,-2 09(-3 58 to -0 58)

Morocco,Deaths,25 ~ 30,-1 92(-2 96 to -0 88)

Morocco,Deaths,30 ~ 35,-1 52(-2 31 to -0 73)

Morocco,Deaths,35 ~ 40,-1 08(-1 71 to -0 44)

Morocco,Deaths,40 ~ 45,-0 64(-1 15 to -0 13)

Morocco,Deaths,45 ~ 50,-0 29(-0 69 to 0 12)

Morocco,Deaths,50 ~ 55,-0 02(-0 34 to 0 3)

Morocco,Deaths,55 ~ 60,0 17(-0 09 to 0 43)

Morocco,Deaths,60 ~ 65,0 29(0 07 to 0 51)

Morocco,Deaths,65 ~ 70,0 37(0 18 to 0 56)

Morocco,Deaths,70 ~ 75,0 41(0 23 to 0 59)

Morocco,Deaths,75 ~ 80,0 46(0 28 to 0 63)

Morocco,Deaths,80 ~ 85,0 53(0 34 to 0 71)

Morocco,Deaths,85 ~ 90,0 68(0 43 to 0 93)

Morocco,Deaths,90 ~ 95,0 9(0 46 to 1 34)

Morocco,Deaths,20 ~ 25,-2 09(-3 58 to -0 58)

Morocco,Deaths,25 ~ 30,-1 92(-2 96 to -0 88)

Morocco,Deaths,30 ~ 35,-1 52(-2 31 to -0 73)

Morocco,Deaths,35 ~ 40,-1 08(-1 71 to -0 44)

Morocco,Deaths,40 ~ 45,-0 64(-1 15 to -0 13)

Morocco,Deaths,45 ~ 50,-0 29(-0 69 to 0 12)

Morocco,Deaths,50 ~ 55,-0 02(-0 34 to 0 3)

Morocco,Deaths,55 ~ 60,0 17(-0 09 to 0 43)

Morocco,Deaths,60 ~ 65,0 29(0 07 to 0 51)

Morocco,Deaths,65 ~ 70,0 37(0 18 to 0 56)

Morocco,Deaths,70 ~ 75,0 41(0 23 to 0 59)

Morocco,Deaths,75 ~ 80,0 46(0 28 to 0 63)

Morocco,Deaths,80 ~ 85,0 53(0 34 to 0 71)

Morocco,Deaths,85 ~ 90,0 68(0 43 to 0 93)

Morocco,Deaths,90 ~ 95,0 9(0 46 to 1 34)

Morocco,Deaths,20 ~ 25,-2 09(-3 58 to -0 58)

Morocco,Deaths,25 ~ 30,-1 92(-2 96 to -0 88)

Morocco,Deaths,30 ~ 35,-1 52(-2 31 to -0 73)

Morocco,Deaths,35 ~ 40,-1 08(-1 71 to -0 44)

Morocco,Deaths,40 ~ 45,-0 64(-1 15 to -0 13)

Morocco,Deaths,45 ~ 50,-0 29(-0 69 to 0 12)

Morocco,Deaths,50 ~ 55,-0 02(-0 34 to 0 3)

Morocco,Deaths,55 ~ 60,0 17(-0 09 to 0 43)

Morocco,Deaths,60 ~ 65,0 29(0 07 to 0 51)

Morocco,Deaths,65 ~ 70,0 37(0 18 to 0 56)

Morocco,Deaths,70 ~ 75,0 41(0 23 to 0 59)

Morocco,Deaths,75 ~ 80,0 46(0 28 to 0 63)

Morocco,Deaths,80 ~ 85,0 53(0 34 to 0 71)

Morocco,Deaths,85 ~ 90,0 68(0 43 to 0 93)

Morocco,Deaths,90 ~ 95,0 9(0 46 to 1 34)

Morocco,Deaths,20 ~ 25,-2 09(-3 58 to -0 58)

Morocco,Deaths,25 ~ 30,-1 92(-2 96 to -0 88)

Morocco,Deaths,30 ~ 35,-1 52(-2 31 to -0 73)

Morocco,Deaths,35 ~ 40,-1 08(-1 71 to -0 44)

Morocco,Deaths,40 ~ 45,-0 64(-1 15 to -0 13)

Morocco,Deaths,45 ~ 50,-0 29(-0 69 to 0 12)

Morocco,Deaths,50 ~ 55,-0 02(-0 34 to 0 3)

Morocco,Deaths,55 ~ 60,0 17(-0 09 to 0 43)

Morocco,Deaths,60 ~ 65,0 29(0 07 to 0 51)

Morocco,Deaths,65 ~ 70,0 37(0 18 to 0 56)

Morocco,Deaths,70 ~ 75,0 41(0 23 to 0 59)

Morocco,Deaths,75 ~ 80,0 46(0 28 to 0 63)

Morocco,Deaths,80 ~ 85,0 53(0 34 to 0 71)

Morocco,Deaths,85 ~ 90,0 68(0 43 to 0 93)

Morocco,Deaths,90 ~ 95,0 9(0 46 to 1 34)

Morocco,DALYs,20 ~ 25,-1 14(-1 36 to -0 91)

Morocco,DALYs,25 ~ 30,-1 04(-1 21 to -0 88)

Morocco,DALYs,30 ~ 35,-0 82(-0 95 to -0 69)

Morocco,DALYs,35 ~ 40,-0 56(-0 67 to -0 44)

Morocco,DALYs,40 ~ 45,-0 28(-0 38 to -0 18)

Morocco,DALYs,45 ~ 50,-0 02(-0 11 to 0 06)

Morocco,DALYs,50 ~ 55,0 18(0 1 to 0 25)

Morocco,DALYs,55 ~ 60,0 33(0 26 to 0 4)

Morocco,DALYs,60 ~ 65,0 44(0 37 to 0 5)

Morocco,DALYs,65 ~ 70,0 52(0 46 to 0 58)

Morocco,DALYs,70 ~ 75,0 57(0 5 to 0 63)

Morocco,DALYs,75 ~ 80,0 61(0 54 to 0 68)

Morocco,DALYs,80 ~ 85,0 67(0 59 to 0 75)

Morocco,DALYs,85 ~ 90,0 8(0 68 to 0 93)

Morocco,DALYs,90 ~ 95,0 99(0 76 to 1 21)

Morocco,DALYs,20 ~ 25,-1 14(-1 36 to -0 91)

Morocco,DALYs,25 ~ 30,-1 04(-1 21 to -0 88)

Morocco,DALYs,30 ~ 35,-0 82(-0 95 to -0 69)

Morocco,DALYs,35 ~ 40,-0 56(-0 67 to -0 44)

Morocco,DALYs,40 ~ 45,-0 28(-0 38 to -0 18)

Morocco,DALYs,45 ~ 50,-0 02(-0 11 to 0 06)

Morocco,DALYs,50 ~ 55,0 18(0 1 to 0 25)

Morocco,DALYs,55 ~ 60,0 33(0 26 to 0 4)

Morocco,DALYs,60 ~ 65,0 44(0 37 to 0 5)

Morocco,DALYs,65 ~ 70,0 52(0 46 to 0 58)

Morocco,DALYs,70 ~ 75,0 57(0 5 to 0 63)

Morocco,DALYs,75 ~ 80,0 61(0 54 to 0 68)

Morocco,DALYs,80 ~ 85,0 67(0 59 to 0 75)

Morocco,DALYs,85 ~ 90,0 8(0 68 to 0 93)

Morocco,DALYs,90 ~ 95,0 99(0 76 to 1 21)

Morocco,DALYs,20 ~ 25,-1 14(-1 36 to -0 91)

Morocco,DALYs,25 ~ 30,-1 04(-1 21 to -0 88)

Morocco,DALYs,30 ~ 35,-0 82(-0 95 to -0 69)

Morocco,DALYs,35 ~ 40,-0 56(-0 67 to -0 44)

Morocco,DALYs,40 ~ 45,-0 28(-0 38 to -0 18)

Morocco,DALYs,45 ~ 50,-0 02(-0 11 to 0 06)

Morocco,DALYs,50 ~ 55,0 18(0 1 to 0 25)

Morocco,DALYs,55 ~ 60,0 33(0 26 to 0 4)

Morocco,DALYs,60 ~ 65,0 44(0 37 to 0 5)

Morocco,DALYs,65 ~ 70,0 52(0 46 to 0 58)

Morocco,DALYs,70 ~ 75,0 57(0 5 to 0 63)

Morocco,DALYs,75 ~ 80,0 61(0 54 to 0 68)

Morocco,DALYs,80 ~ 85,0 67(0 59 to 0 75)

Morocco,DALYs,85 ~ 90,0 8(0 68 to 0 93)

Morocco,DALYs,90 ~ 95,0 99(0 76 to 1 21)

Morocco,DALYs,20 ~ 25,-1 14(-1 36 to -0 91)

Morocco,DALYs,25 ~ 30,-1 04(-1 21 to -0 88)

Morocco,DALYs,30 ~ 35,-0 82(-0 95 to -0 69)

Morocco,DALYs,35 ~ 40,-0 56(-0 67 to -0 44)

Morocco,DALYs,40 ~ 45,-0 28(-0 38 to -0 18)

Morocco,DALYs,45 ~ 50,-0 02(-0 11 to 0 06)

Morocco,DALYs,50 ~ 55,0 18(0 1 to 0 25)

Morocco,DALYs,55 ~ 60,0 33(0 26 to 0 4)

Morocco,DALYs,60 ~ 65,0 44(0 37 to 0 5)

Morocco,DALYs,65 ~ 70,0 52(0 46 to 0 58)

Morocco,DALYs,70 ~ 75,0 57(0 5 to 0 63)

Morocco,DALYs,75 ~ 80,0 61(0 54 to 0 68)

Morocco,DALYs,80 ~ 85,0 67(0 59 to 0 75)

Morocco,DALYs,85 ~ 90,0 8(0 68 to 0 93)

Morocco,DALYs,90 ~ 95,0 99(0 76 to 1 21)

Oman,Prevalence,20 ~ 25,-0 55(-0 72 to -0 39)

Oman,Prevalence,25 ~ 30,-0 55(-0 66 to -0 44)

Oman,Prevalence,30 ~ 35,-0 51(-0 6 to -0 42)

Oman,Prevalence,35 ~ 40,-0 47(-0 56 to -0 39)

Oman,Prevalence,40 ~ 45,-0 43(-0 51 to -0 35)

Oman,Prevalence,45 ~ 50,-0 4(-0 48 to -0 32)

Oman,Prevalence,50 ~ 55,-0 33(-0 4 to -0 25)

Oman,Prevalence,55 ~ 60,-0 18(-0 26 to -0 1)

Oman,Prevalence,60 ~ 65,0 03(-0 05 to 0 11)

Oman,Prevalence,65 ~ 70,0 27(0 19 to 0 36)

Oman,Prevalence,70 ~ 75,0 54(0 44 to 0 63)

Oman,Prevalence,75 ~ 80,0 78(0 68 to 0 89)

Oman,Prevalence,80 ~ 85,1(0 86 to 1 13)

Oman,Prevalence,85 ~ 90,1 18(0 97 to 1 38)

Oman,Prevalence,90 ~ 95,1 33(0 95 to 1 7)

Oman,Prevalence,20 ~ 25,-0 55(-0 72 to -0 39)

Oman,Prevalence,25 ~ 30,-0 55(-0 66 to -0 44)

Oman,Prevalence,30 ~ 35,-0 51(-0 6 to -0 42)

Oman,Prevalence,35 ~ 40,-0 47(-0 56 to -0 39)

Oman,Prevalence,40 ~ 45,-0 43(-0 51 to -0 35)

Oman,Prevalence,45 ~ 50,-0 4(-0 48 to -0 32)

Oman,Prevalence,50 ~ 55,-0 33(-0 4 to -0 25)

Oman,Prevalence,55 ~ 60,-0 18(-0 26 to -0 1)

Oman,Prevalence,60 ~ 65,0 03(-0 05 to 0 11)

Oman,Prevalence,65 ~ 70,0 27(0 19 to 0 36)

Oman,Prevalence,70 ~ 75,0 54(0 44 to 0 63)

Oman,Prevalence,75 ~ 80,0 78(0 68 to 0 89)

Oman,Prevalence,80 ~ 85,1(0 86 to 1 13)

Oman,Prevalence,85 ~ 90,1 18(0 97 to 1 38)

Oman,Prevalence,90 ~ 95,1 33(0 95 to 1 7)

Oman,Prevalence,20 ~ 25,-0 55(-0 72 to -0 39)

Oman,Prevalence,25 ~ 30,-0 55(-0 66 to -0 44)

Oman,Prevalence,30 ~ 35,-0 51(-0 6 to -0 42)

Oman,Prevalence,35 ~ 40,-0 47(-0 56 to -0 39)

Oman,Prevalence,40 ~ 45,-0 43(-0 51 to -0 35)

Oman,Prevalence,45 ~ 50,-0 4(-0 48 to -0 32)

Oman,Prevalence,50 ~ 55,-0 33(-0 4 to -0 25)

Oman,Prevalence,55 ~ 60,-0 18(-0 26 to -0 1)

Oman,Prevalence,60 ~ 65,0 03(-0 05 to 0 11)

Oman,Prevalence,65 ~ 70,0 27(0 19 to 0 36)

Oman,Prevalence,70 ~ 75,0 54(0 44 to 0 63)

Oman,Prevalence,75 ~ 80,0 78(0 68 to 0 89)

Oman,Prevalence,80 ~ 85,1(0 86 to 1 13)

Oman,Prevalence,85 ~ 90,1 18(0 97 to 1 38)

Oman,Prevalence,90 ~ 95,1 33(0 95 to 1 7)

Oman,Prevalence,20 ~ 25,-0 55(-0 72 to -0 39)

Oman,Prevalence,25 ~ 30,-0 55(-0 66 to -0 44)

Oman,Prevalence,30 ~ 35,-0 51(-0 6 to -0 42)

Oman,Prevalence,35 ~ 40,-0 47(-0 56 to -0 39)

Oman,Prevalence,40 ~ 45,-0 43(-0 51 to -0 35)

Oman,Prevalence,45 ~ 50,-0 4(-0 48 to -0 32)

Oman,Prevalence,50 ~ 55,-0 33(-0 4 to -0 25)

Oman,Prevalence,55 ~ 60,-0 18(-0 26 to -0 1)

Oman,Prevalence,60 ~ 65,0 03(-0 05 to 0 11)

Oman,Prevalence,65 ~ 70,0 27(0 19 to 0 36)

Oman,Prevalence,70 ~ 75,0 54(0 44 to 0 63)

Oman,Prevalence,75 ~ 80,0 78(0 68 to 0 89)

Oman,Prevalence,80 ~ 85,1(0 86 to 1 13)

Oman,Prevalence,85 ~ 90,1 18(0 97 to 1 38)

Oman,Prevalence,90 ~ 95,1 33(0 95 to 1 7)

Oman,Deaths,20 ~ 25,-3 66(-10 17 to 3 33)

Oman,Deaths,25 ~ 30,-3 16(-7 17 to 1 02)

Oman,Deaths,30 ~ 35,-3 17(-6 34 to 0 1)

Oman,Deaths,35 ~ 40,-3 03(-5 69 to -0 29)

Oman,Deaths,40 ~ 45,-3 05(-5 34 to -0 7)

Oman,Deaths,45 ~ 50,-3 09(-5 05 to -1 09)

Oman,Deaths,50 ~ 55,-2 74(-4 37 to -1 09)

Oman,Deaths,55 ~ 60,-2 61(-3 97 to -1 23)

Oman,Deaths,60 ~ 65,-2 07(-3 2 to -0 93)

Oman,Deaths,65 ~ 70,-1 39(-2 41 to -0 37)

Oman,Deaths,70 ~ 75,-0 67(-1 62 to 0 28)

Oman,Deaths,75 ~ 80,0 01(-0 96 to 0 99)

Oman,Deaths,80 ~ 85,0 56(-0 62 to 1 74)

Oman,Deaths,85 ~ 90,1 08(-0 61 to 2 79)

Oman,Deaths,90 ~ 95,1 04(-2 02 to 4 2)

Oman,Deaths,20 ~ 25,-3 66(-10 17 to 3 33)

Oman,Deaths,25 ~ 30,-3 16(-7 17 to 1 02)

Oman,Deaths,30 ~ 35,-3 17(-6 34 to 0 1)

Oman,Deaths,35 ~ 40,-3 03(-5 69 to -0 29)

Oman,Deaths,40 ~ 45,-3 05(-5 34 to -0 7)

Oman,Deaths,45 ~ 50,-3 09(-5 05 to -1 09)

Oman,Deaths,50 ~ 55,-2 74(-4 37 to -1 09)

Oman,Deaths,55 ~ 60,-2 61(-3 97 to -1 23)

Oman,Deaths,60 ~ 65,-2 07(-3 2 to -0 93)

Oman,Deaths,65 ~ 70,-1 39(-2 41 to -0 37)

Oman,Deaths,70 ~ 75,-0 67(-1 62 to 0 28)

Oman,Deaths,75 ~ 80,0 01(-0 96 to 0 99)

Oman,Deaths,80 ~ 85,0 56(-0 62 to 1 74)

Oman,Deaths,85 ~ 90,1 08(-0 61 to 2 79)

Oman,Deaths,90 ~ 95,1 04(-2 02 to 4 2)

Oman,Deaths,20 ~ 25,-3 66(-10 17 to 3 33)

Oman,Deaths,25 ~ 30,-3 16(-7 17 to 1 02)

Oman,Deaths,30 ~ 35,-3 17(-6 34 to 0 1)

Oman,Deaths,35 ~ 40,-3 03(-5 69 to -0 29)

Oman,Deaths,40 ~ 45,-3 05(-5 34 to -0 7)

Oman,Deaths,45 ~ 50,-3 09(-5 05 to -1 09)

Oman,Deaths,50 ~ 55,-2 74(-4 37 to -1 09)

Oman,Deaths,55 ~ 60,-2 61(-3 97 to -1 23)

Oman,Deaths,60 ~ 65,-2 07(-3 2 to -0 93)

Oman,Deaths,65 ~ 70,-1 39(-2 41 to -0 37)

Oman,Deaths,70 ~ 75,-0 67(-1 62 to 0 28)

Oman,Deaths,75 ~ 80,0 01(-0 96 to 0 99)

Oman,Deaths,80 ~ 85,0 56(-0 62 to 1 74)

Oman,Deaths,85 ~ 90,1 08(-0 61 to 2 79)

Oman,Deaths,90 ~ 95,1 04(-2 02 to 4 2)

Oman,Deaths,20 ~ 25,-3 66(-10 17 to 3 33)

Oman,Deaths,25 ~ 30,-3 16(-7 17 to 1 02)

Oman,Deaths,30 ~ 35,-3 17(-6 34 to 0 1)

Oman,Deaths,35 ~ 40,-3 03(-5 69 to -0 29)

Oman,Deaths,40 ~ 45,-3 05(-5 34 to -0 7)

Oman,Deaths,45 ~ 50,-3 09(-5 05 to -1 09)

Oman,Deaths,50 ~ 55,-2 74(-4 37 to -1 09)

Oman,Deaths,55 ~ 60,-2 61(-3 97 to -1 23)

Oman,Deaths,60 ~ 65,-2 07(-3 2 to -0 93)

Oman,Deaths,65 ~ 70,-1 39(-2 41 to -0 37)

Oman,Deaths,70 ~ 75,-0 67(-1 62 to 0 28)

Oman,Deaths,75 ~ 80,0 01(-0 96 to 0 99)

Oman,Deaths,80 ~ 85,0 56(-0 62 to 1 74)

Oman,Deaths,85 ~ 90,1 08(-0 61 to 2 79)

Oman,Deaths,90 ~ 95,1 04(-2 02 to 4 2)

Oman,DALYs,20 ~ 25,-1 68(-2 26 to -1 09)

Oman,DALYs,25 ~ 30,-1 52(-1 9 to -1 13)

Oman,DALYs,30 ~ 35,-1 53(-1 85 to -1 22)

Oman,DALYs,35 ~ 40,-1 52(-1 8 to -1 23)

Oman,DALYs,40 ~ 45,-1 59(-1 86 to -1 33)

Oman,DALYs,45 ~ 50,-1 71(-1 96 to -1 46)

Oman,DALYs,50 ~ 55,-1 62(-1 86 to -1 39)

Oman,DALYs,55 ~ 60,-1 59(-1 81 to -1 37)

Oman,DALYs,60 ~ 65,-1 29(-1 5 to -1 09)

Oman,DALYs,65 ~ 70,-0 87(-1 08 to -0 66)

Oman,DALYs,70 ~ 75,-0 39(-0 6 to -0 17)

Oman,DALYs,75 ~ 80,0 08(-0 17 to 0 33)

Oman,DALYs,80 ~ 85,0 49(0 16 to 0 83)

Oman,DALYs,85 ~ 90,0 87(0 35 to 1 39)

Oman,DALYs,90 ~ 95,0 93(-0 07 to 1 93)

Oman,DALYs,20 ~ 25,-1 68(-2 26 to -1 09)

Oman,DALYs,25 ~ 30,-1 52(-1 9 to -1 13)

Oman,DALYs,30 ~ 35,-1 53(-1 85 to -1 22)

Oman,DALYs,35 ~ 40,-1 52(-1 8 to -1 23)

Oman,DALYs,40 ~ 45,-1 59(-1 86 to -1 33)

Oman,DALYs,45 ~ 50,-1 71(-1 96 to -1 46)

Oman,DALYs,50 ~ 55,-1 62(-1 86 to -1 39)

Oman,DALYs,55 ~ 60,-1 59(-1 81 to -1 37)

Oman,DALYs,60 ~ 65,-1 29(-1 5 to -1 09)

Oman,DALYs,65 ~ 70,-0 87(-1 08 to -0 66)

Oman,DALYs,70 ~ 75,-0 39(-0 6 to -0 17)

Oman,DALYs,75 ~ 80,0 08(-0 17 to 0 33)

Oman,DALYs,80 ~ 85,0 49(0 16 to 0 83)

Oman,DALYs,85 ~ 90,0 87(0 35 to 1 39)

Oman,DALYs,90 ~ 95,0 93(-0 07 to 1 93)

Oman,DALYs,20 ~ 25,-1 68(-2 26 to -1 09)

Oman,DALYs,25 ~ 30,-1 52(-1 9 to -1 13)

Oman,DALYs,30 ~ 35,-1 53(-1 85 to -1 22)

Oman,DALYs,35 ~ 40,-1 52(-1 8 to -1 23)

Oman,DALYs,40 ~ 45,-1 59(-1 86 to -1 33)

Oman,DALYs,45 ~ 50,-1 71(-1 96 to -1 46)

Oman,DALYs,50 ~ 55,-1 62(-1 86 to -1 39)

Oman,DALYs,55 ~ 60,-1 59(-1 81 to -1 37)

Oman,DALYs,60 ~ 65,-1 29(-1 5 to -1 09)

Oman,DALYs,65 ~ 70,-0 87(-1 08 to -0 66)

Oman,DALYs,70 ~ 75,-0 39(-0 6 to -0 17)

Oman,DALYs,75 ~ 80,0 08(-0 17 to 0 33)

Oman,DALYs,80 ~ 85,0 49(0 16 to 0 83)

Oman,DALYs,85 ~ 90,0 87(0 35 to 1 39)

Oman,DALYs,90 ~ 95,0 93(-0 07 to 1 93)

Oman,DALYs,20 ~ 25,-1 68(-2 26 to -1 09)

Oman,DALYs,25 ~ 30,-1 52(-1 9 to -1 13)

Oman,DALYs,30 ~ 35,-1 53(-1 85 to -1 22)

Oman,DALYs,35 ~ 40,-1 52(-1 8 to -1 23)

Oman,DALYs,40 ~ 45,-1 59(-1 86 to -1 33)

Oman,DALYs,45 ~ 50,-1 71(-1 96 to -1 46)

Oman,DALYs,50 ~ 55,-1 62(-1 86 to -1 39)

Oman,DALYs,55 ~ 60,-1 59(-1 81 to -1 37)

Oman,DALYs,60 ~ 65,-1 29(-1 5 to -1 09)

Oman,DALYs,65 ~ 70,-0 87(-1 08 to -0 66)

Oman,DALYs,70 ~ 75,-0 39(-0 6 to -0 17)

Oman,DALYs,75 ~ 80,0 08(-0 17 to 0 33)

Oman,DALYs,80 ~ 85,0 49(0 16 to 0 83)

Oman,DALYs,85 ~ 90,0 87(0 35 to 1 39)

Oman,DALYs,90 ~ 95,0 93(-0 07 to 1 93)

Sudan,Prevalence,20 ~ 25,-0 32(-0 41 to -0 23)

Sudan,Prevalence,25 ~ 30,-0 27(-0 34 to -0 21)

Sudan,Prevalence,30 ~ 35,-0 25(-0 31 to -0 19)

Sudan,Prevalence,35 ~ 40,-0 25(-0 3 to -0 19)

Sudan,Prevalence,40 ~ 45,-0 27(-0 32 to -0 22)

Sudan,Prevalence,45 ~ 50,-0 3(-0 35 to -0 25)

Sudan,Prevalence,50 ~ 55,-0 3(-0 34 to -0 25)

Sudan,Prevalence,55 ~ 60,-0 25(-0 29 to -0 2)

Sudan,Prevalence,60 ~ 65,-0 15(-0 19 to -0 11)

Sudan,Prevalence,65 ~ 70,-0 01(-0 06 to 0 03)

Sudan,Prevalence,70 ~ 75,0 14(0 09 to 0 18)

Sudan,Prevalence,75 ~ 80,0 29(0 24 to 0 34)

Sudan,Prevalence,80 ~ 85,0 43(0 37 to 0 5)

Sudan,Prevalence,85 ~ 90,0 59(0 48 to 0 7)

Sudan,Prevalence,90 ~ 95,0 76(0 54 to 0 98)

Sudan,Prevalence,20 ~ 25,-0 32(-0 41 to -0 23)

Sudan,Prevalence,25 ~ 30,-0 27(-0 34 to -0 21)

Sudan,Prevalence,30 ~ 35,-0 25(-0 31 to -0 19)

Sudan,Prevalence,35 ~ 40,-0 25(-0 3 to -0 19)

Sudan,Prevalence,40 ~ 45,-0 27(-0 32 to -0 22)

Sudan,Prevalence,45 ~ 50,-0 3(-0 35 to -0 25)

Sudan,Prevalence,50 ~ 55,-0 3(-0 34 to -0 25)

Sudan,Prevalence,55 ~ 60,-0 25(-0 29 to -0 2)

Sudan,Prevalence,60 ~ 65,-0 15(-0 19 to -0 11)

Sudan,Prevalence,65 ~ 70,-0 01(-0 06 to 0 03)

Sudan,Prevalence,70 ~ 75,0 14(0 09 to 0 18)

Sudan,Prevalence,75 ~ 80,0 29(0 24 to 0 34)

Sudan,Prevalence,80 ~ 85,0 43(0 37 to 0 5)

Sudan,Prevalence,85 ~ 90,0 59(0 48 to 0 7)

Sudan,Prevalence,90 ~ 95,0 76(0 54 to 0 98)

Sudan,Prevalence,20 ~ 25,-0 32(-0 41 to -0 23)

Sudan,Prevalence,25 ~ 30,-0 27(-0 34 to -0 21)

Sudan,Prevalence,30 ~ 35,-0 25(-0 31 to -0 19)

Sudan,Prevalence,35 ~ 40,-0 25(-0 3 to -0 19)

Sudan,Prevalence,40 ~ 45,-0 27(-0 32 to -0 22)

Sudan,Prevalence,45 ~ 50,-0 3(-0 35 to -0 25)

Sudan,Prevalence,50 ~ 55,-0 3(-0 34 to -0 25)

Sudan,Prevalence,55 ~ 60,-0 25(-0 29 to -0 2)

Sudan,Prevalence,60 ~ 65,-0 15(-0 19 to -0 11)

Sudan,Prevalence,65 ~ 70,-0 01(-0 06 to 0 03)

Sudan,Prevalence,70 ~ 75,0 14(0 09 to 0 18)

Sudan,Prevalence,75 ~ 80,0 29(0 24 to 0 34)

Sudan,Prevalence,80 ~ 85,0 43(0 37 to 0 5)

Sudan,Prevalence,85 ~ 90,0 59(0 48 to 0 7)

Sudan,Prevalence,90 ~ 95,0 76(0 54 to 0 98)

Sudan,Prevalence,20 ~ 25,-0 32(-0 41 to -0 23)

Sudan,Prevalence,25 ~ 30,-0 27(-0 34 to -0 21)

Sudan,Prevalence,30 ~ 35,-0 25(-0 31 to -0 19)

Sudan,Prevalence,35 ~ 40,-0 25(-0 3 to -0 19)

Sudan,Prevalence,40 ~ 45,-0 27(-0 32 to -0 22)

Sudan,Prevalence,45 ~ 50,-0 3(-0 35 to -0 25)

Sudan,Prevalence,50 ~ 55,-0 3(-0 34 to -0 25)

Sudan,Prevalence,55 ~ 60,-0 25(-0 29 to -0 2)

Sudan,Prevalence,60 ~ 65,-0 15(-0 19 to -0 11)

Sudan,Prevalence,65 ~ 70,-0 01(-0 06 to 0 03)

Sudan,Prevalence,70 ~ 75,0 14(0 09 to 0 18)

Sudan,Prevalence,75 ~ 80,0 29(0 24 to 0 34)

Sudan,Prevalence,80 ~ 85,0 43(0 37 to 0 5)

Sudan,Prevalence,85 ~ 90,0 59(0 48 to 0 7)

Sudan,Prevalence,90 ~ 95,0 76(0 54 to 0 98)

Sudan,Deaths,20 ~ 25,-1 52(-2 52 to -0 52)

Sudan,Deaths,25 ~ 30,-1 51(-2 25 to -0 77)

Sudan,Deaths,30 ~ 35,-1 51(-2 12 to -0 9)

Sudan,Deaths,35 ~ 40,-1 56(-2 08 to -1 03)

Sudan,Deaths,40 ~ 45,-1 62(-2 07 to -1 17)

Sudan,Deaths,45 ~ 50,-1 67(-2 04 to -1 29)

Sudan,Deaths,50 ~ 55,-1 67(-1 98 to -1 35)

Sudan,Deaths,55 ~ 60,-1 6(-1 87 to -1 32)

Sudan,Deaths,60 ~ 65,-1 5(-1 74 to -1 26)

Sudan,Deaths,65 ~ 70,-1 38(-1 59 to -1 17)

Sudan,Deaths,70 ~ 75,-1 21(-1 4 to -1 02)

Sudan,Deaths,75 ~ 80,-1 02(-1 2 to -0 83)

Sudan,Deaths,80 ~ 85,-0 83(-1 06 to -0 61)

Sudan,Deaths,85 ~ 90,-0 67(-0 99 to -0 35)

Sudan,Deaths,90 ~ 95,-0 48(-1 08 to 0 11)

Sudan,Deaths,20 ~ 25,-1 52(-2 52 to -0 52)

Sudan,Deaths,25 ~ 30,-1 51(-2 25 to -0 77)

Sudan,Deaths,30 ~ 35,-1 51(-2 12 to -0 9)

Sudan,Deaths,35 ~ 40,-1 56(-2 08 to -1 03)

Sudan,Deaths,40 ~ 45,-1 62(-2 07 to -1 17)

Sudan,Deaths,45 ~ 50,-1 67(-2 04 to -1 29)

Sudan,Deaths,50 ~ 55,-1 67(-1 98 to -1 35)

Sudan,Deaths,55 ~ 60,-1 6(-1 87 to -1 32)

Sudan,Deaths,60 ~ 65,-1 5(-1 74 to -1 26)

Sudan,Deaths,65 ~ 70,-1 38(-1 59 to -1 17)

Sudan,Deaths,70 ~ 75,-1 21(-1 4 to -1 02)

Sudan,Deaths,75 ~ 80,-1 02(-1 2 to -0 83)

Sudan,Deaths,80 ~ 85,-0 83(-1 06 to -0 61)

Sudan,Deaths,85 ~ 90,-0 67(-0 99 to -0 35)

Sudan,Deaths,90 ~ 95,-0 48(-1 08 to 0 11)

Sudan,Deaths,20 ~ 25,-1 52(-2 52 to -0 52)

Sudan,Deaths,25 ~ 30,-1 51(-2 25 to -0 77)

Sudan,Deaths,30 ~ 35,-1 51(-2 12 to -0 9)

Sudan,Deaths,35 ~ 40,-1 56(-2 08 to -1 03)

Sudan,Deaths,40 ~ 45,-1 62(-2 07 to -1 17)

Sudan,Deaths,45 ~ 50,-1 67(-2 04 to -1 29)

Sudan,Deaths,50 ~ 55,-1 67(-1 98 to -1 35)

Sudan,Deaths,55 ~ 60,-1 6(-1 87 to -1 32)

Sudan,Deaths,60 ~ 65,-1 5(-1 74 to -1 26)

Sudan,Deaths,65 ~ 70,-1 38(-1 59 to -1 17)

Sudan,Deaths,70 ~ 75,-1 21(-1 4 to -1 02)

Sudan,Deaths,75 ~ 80,-1 02(-1 2 to -0 83)

Sudan,Deaths,80 ~ 85,-0 83(-1 06 to -0 61)

Sudan,Deaths,85 ~ 90,-0 67(-0 99 to -0 35)

Sudan,Deaths,90 ~ 95,-0 48(-1 08 to 0 11)

Sudan,Deaths,20 ~ 25,-1 52(-2 52 to -0 52)

Sudan,Deaths,25 ~ 30,-1 51(-2 25 to -0 77)

Sudan,Deaths,30 ~ 35,-1 51(-2 12 to -0 9)

Sudan,Deaths,35 ~ 40,-1 56(-2 08 to -1 03)

Sudan,Deaths,40 ~ 45,-1 62(-2 07 to -1 17)

Sudan,Deaths,45 ~ 50,-1 67(-2 04 to -1 29)

Sudan,Deaths,50 ~ 55,-1 67(-1 98 to -1 35)

Sudan,Deaths,55 ~ 60,-1 6(-1 87 to -1 32)

Sudan,Deaths,60 ~ 65,-1 5(-1 74 to -1 26)

Sudan,Deaths,65 ~ 70,-1 38(-1 59 to -1 17)

Sudan,Deaths,70 ~ 75,-1 21(-1 4 to -1 02)

Sudan,Deaths,75 ~ 80,-1 02(-1 2 to -0 83)

Sudan,Deaths,80 ~ 85,-0 83(-1 06 to -0 61)

Sudan,Deaths,85 ~ 90,-0 67(-0 99 to -0 35)

Sudan,Deaths,90 ~ 95,-0 48(-1 08 to 0 11)

Sudan,DALYs,20 ~ 25,-1 06(-1 25 to -0 86)

Sudan,DALYs,25 ~ 30,-1 05(-1 2 to -0 91)

Sudan,DALYs,30 ~ 35,-1 07(-1 2 to -0 94)

Sudan,DALYs,35 ~ 40,-1 12(-1 24 to -1 01)

Sudan,DALYs,40 ~ 45,-1 2(-1 31 to -1 1)

Sudan,DALYs,45 ~ 50,-1 27(-1 37 to -1 18)

Sudan,DALYs,50 ~ 55,-1 31(-1 39 to -1 22)

Sudan,DALYs,55 ~ 60,-1 28(-1 36 to -1 2)

Sudan,DALYs,60 ~ 65,-1 22(-1 29 to -1 14)

Sudan,DALYs,65 ~ 70,-1 12(-1 19 to -1 05)

Sudan,DALYs,70 ~ 75,-0 98(-1 06 to -0 91)

Sudan,DALYs,75 ~ 80,-0 83(-0 91 to -0 75)

Sudan,DALYs,80 ~ 85,-0 67(-0 78 to -0 56)

Sudan,DALYs,85 ~ 90,-0 52(-0 7 to -0 34)

Sudan,DALYs,90 ~ 95,-0 34(-0 69 to 0 01)

Sudan,DALYs,20 ~ 25,-1 06(-1 25 to -0 86)

Sudan,DALYs,25 ~ 30,-1 05(-1 2 to -0 91)

Sudan,DALYs,30 ~ 35,-1 07(-1 2 to -0 94)

Sudan,DALYs,35 ~ 40,-1 12(-1 24 to -1 01)

Sudan,DALYs,40 ~ 45,-1 2(-1 31 to -1 1)

Sudan,DALYs,45 ~ 50,-1 27(-1 37 to -1 18)

Sudan,DALYs,50 ~ 55,-1 31(-1 39 to -1 22)

Sudan,DALYs,55 ~ 60,-1 28(-1 36 to -1 2)

Sudan,DALYs,60 ~ 65,-1 22(-1 29 to -1 14)

Sudan,DALYs,65 ~ 70,-1 12(-1 19 to -1 05)

Sudan,DALYs,70 ~ 75,-0 98(-1 06 to -0 91)

Sudan,DALYs,75 ~ 80,-0 83(-0 91 to -0 75)

Sudan,DALYs,80 ~ 85,-0 67(-0 78 to -0 56)

Sudan,DALYs,85 ~ 90,-0 52(-0 7 to -0 34)

Sudan,DALYs,90 ~ 95,-0 34(-0 69 to 0 01)

Sudan,DALYs,20 ~ 25,-1 06(-1 25 to -0 86)

Sudan,DALYs,25 ~ 30,-1 05(-1 2 to -0 91)

Sudan,DALYs,30 ~ 35,-1 07(-1 2 to -0 94)

Sudan,DALYs,35 ~ 40,-1 12(-1 24 to -1 01)

Sudan,DALYs,40 ~ 45,-1 2(-1 31 to -1 1)

Sudan,DALYs,45 ~ 50,-1 27(-1 37 to -1 18)

Sudan,DALYs,50 ~ 55,-1 31(-1 39 to -1 22)

Sudan,DALYs,55 ~ 60,-1 28(-1 36 to -1 2)

Sudan,DALYs,60 ~ 65,-1 22(-1 29 to -1 14)

Sudan,DALYs,65 ~ 70,-1 12(-1 19 to -1 05)

Sudan,DALYs,70 ~ 75,-0 98(-1 06 to -0 91)

Sudan,DALYs,75 ~ 80,-0 83(-0 91 to -0 75)

Sudan,DALYs,80 ~ 85,-0 67(-0 78 to -0 56)

Sudan,DALYs,85 ~ 90,-0 52(-0 7 to -0 34)

Sudan,DALYs,90 ~ 95,-0 34(-0 69 to 0 01)

Sudan,DALYs,20 ~ 25,-1 06(-1 25 to -0 86)

Sudan,DALYs,25 ~ 30,-1 05(-1 2 to -0 91)

Sudan,DALYs,30 ~ 35,-1 07(-1 2 to -0 94)

Sudan,DALYs,35 ~ 40,-1 12(-1 24 to -1 01)

Sudan,DALYs,40 ~ 45,-1 2(-1 31 to -1 1)

Sudan,DALYs,45 ~ 50,-1 27(-1 37 to -1 18)

Sudan,DALYs,50 ~ 55,-1 31(-1 39 to -1 22)

Sudan,DALYs,55 ~ 60,-1 28(-1 36 to -1 2)

Sudan,DALYs,60 ~ 65,-1 22(-1 29 to -1 14)

Sudan,DALYs,65 ~ 70,-1 12(-1 19 to -1 05)

Sudan,DALYs,70 ~ 75,-0 98(-1 06 to -0 91)

Sudan,DALYs,75 ~ 80,-0 83(-0 91 to -0 75)

Sudan,DALYs,80 ~ 85,-0 67(-0 78 to -0 56)

Sudan,DALYs,85 ~ 90,-0 52(-0 7 to -0 34)

Sudan,DALYs,90 ~ 95,-0 34(-0 69 to 0 01)

Egypt,Prevalence,20 ~ 25,0 08(0 03 to 0 14)

Egypt,Prevalence,25 ~ 30,0 1(0 06 to 0 14)

Egypt,Prevalence,30 ~ 35,0 14(0 11 to 0 18)

Egypt,Prevalence,35 ~ 40,0 2(0 17 to 0 23)

Egypt,Prevalence,40 ~ 45,0 29(0 26 to 0 32)

Egypt,Prevalence,45 ~ 50,0 39(0 36 to 0 42)

Egypt,Prevalence,50 ~ 55,0 5(0 47 to 0 52)

Egypt,Prevalence,55 ~ 60,0 6(0 58 to 0 62)

Egypt,Prevalence,60 ~ 65,0 71(0 68 to 0 73)

Egypt,Prevalence,65 ~ 70,0 84(0 81 to 0 86)

Egypt,Prevalence,70 ~ 75,0 98(0 95 to 1 01)

Egypt,Prevalence,75 ~ 80,1 13(1 09 to 1 16)

Egypt,Prevalence,80 ~ 85,1 28(1 23 to 1 32)

Egypt,Prevalence,85 ~ 90,1 41(1 34 to 1 49)

Egypt,Prevalence,90 ~ 95,1 55(1 4 to 1 7)

Egypt,Prevalence,20 ~ 25,0 08(0 03 to 0 14)

Egypt,Prevalence,25 ~ 30,0 1(0 06 to 0 14)

Egypt,Prevalence,30 ~ 35,0 14(0 11 to 0 18)

Egypt,Prevalence,35 ~ 40,0 2(0 17 to 0 23)

Egypt,Prevalence,40 ~ 45,0 29(0 26 to 0 32)

Egypt,Prevalence,45 ~ 50,0 39(0 36 to 0 42)

Egypt,Prevalence,50 ~ 55,0 5(0 47 to 0 52)

Egypt,Prevalence,55 ~ 60,0 6(0 58 to 0 62)

Egypt,Prevalence,60 ~ 65,0 71(0 68 to 0 73)

Egypt,Prevalence,65 ~ 70,0 84(0 81 to 0 86)

Egypt,Prevalence,70 ~ 75,0 98(0 95 to 1 01)

Egypt,Prevalence,75 ~ 80,1 13(1 09 to 1 16)

Egypt,Prevalence,80 ~ 85,1 28(1 23 to 1 32)

Egypt,Prevalence,85 ~ 90,1 41(1 34 to 1 49)

Egypt,Prevalence,90 ~ 95,1 55(1 4 to 1 7)

Egypt,Prevalence,20 ~ 25,0 08(0 03 to 0 14)

Egypt,Prevalence,25 ~ 30,0 1(0 06 to 0 14)

Egypt,Prevalence,30 ~ 35,0 14(0 11 to 0 18)

Egypt,Prevalence,35 ~ 40,0 2(0 17 to 0 23)

Egypt,Prevalence,40 ~ 45,0 29(0 26 to 0 32)

Egypt,Prevalence,45 ~ 50,0 39(0 36 to 0 42)

Egypt,Prevalence,50 ~ 55,0 5(0 47 to 0 52)

Egypt,Prevalence,55 ~ 60,0 6(0 58 to 0 62)

Egypt,Prevalence,60 ~ 65,0 71(0 68 to 0 73)

Egypt,Prevalence,65 ~ 70,0 84(0 81 to 0 86)

Egypt,Prevalence,70 ~ 75,0 98(0 95 to 1 01)

Egypt,Prevalence,75 ~ 80,1 13(1 09 to 1 16)

Egypt,Prevalence,80 ~ 85,1 28(1 23 to 1 32)

Egypt,Prevalence,85 ~ 90,1 41(1 34 to 1 49)

Egypt,Prevalence,90 ~ 95,1 55(1 4 to 1 7)

Egypt,Prevalence,20 ~ 25,0 08(0 03 to 0 14)

Egypt,Prevalence,25 ~ 30,0 1(0 06 to 0 14)

Egypt,Prevalence,30 ~ 35,0 14(0 11 to 0 18)

Egypt,Prevalence,35 ~ 40,0 2(0 17 to 0 23)

Egypt,Prevalence,40 ~ 45,0 29(0 26 to 0 32)

Egypt,Prevalence,45 ~ 50,0 39(0 36 to 0 42)

Egypt,Prevalence,50 ~ 55,0 5(0 47 to 0 52)

Egypt,Prevalence,55 ~ 60,0 6(0 58 to 0 62)

Egypt,Prevalence,60 ~ 65,0 71(0 68 to 0 73)

Egypt,Prevalence,65 ~ 70,0 84(0 81 to 0 86)

Egypt,Prevalence,70 ~ 75,0 98(0 95 to 1 01)

Egypt,Prevalence,75 ~ 80,1 13(1 09 to 1 16)

Egypt,Prevalence,80 ~ 85,1 28(1 23 to 1 32)

Egypt,Prevalence,85 ~ 90,1 41(1 34 to 1 49)

Egypt,Prevalence,90 ~ 95,1 55(1 4 to 1 7)

Egypt,Deaths,20 ~ 25,-1 95(-3 03 to -0 87)

Egypt,Deaths,25 ~ 30,-2 16(-2 93 to -1 38)

Egypt,Deaths,30 ~ 35,-2 47(-3 08 to -1 85)

Egypt,Deaths,35 ~ 40,-2 99(-3 5 to -2 48)

Egypt,Deaths,40 ~ 45,-3 34(-3 77 to -2 91)

Egypt,Deaths,45 ~ 50,-3 36(-3 71 to -3 02)

Egypt,Deaths,50 ~ 55,-3 22(-3 5 to -2 95)

Egypt,Deaths,55 ~ 60,-3 03(-3 24 to -2 81)

Egypt,Deaths,60 ~ 65,-2 96(-3 14 to -2 78)

Egypt,Deaths,65 ~ 70,-2 84(-3 to -2 68)

Egypt,Deaths,70 ~ 75,-2 53(-2 68 to -2 37)

Egypt,Deaths,75 ~ 80,-2 01(-2 17 to -1 84)

Egypt,Deaths,80 ~ 85,-1 27(-1 47 to -1 07)

Egypt,Deaths,85 ~ 90,-1(-1 27 to -0 72)

Egypt,Deaths,90 ~ 95,-1 08(-1 62 to -0 54)

Egypt,Deaths,20 ~ 25,-1 95(-3 03 to -0 87)

Egypt,Deaths,25 ~ 30,-2 16(-2 93 to -1 38)

Egypt,Deaths,30 ~ 35,-2 47(-3 08 to -1 85)

Egypt,Deaths,35 ~ 40,-2 99(-3 5 to -2 48)

Egypt,Deaths,40 ~ 45,-3 34(-3 77 to -2 91)

Egypt,Deaths,45 ~ 50,-3 36(-3 71 to -3 02)

Egypt,Deaths,50 ~ 55,-3 22(-3 5 to -2 95)

Egypt,Deaths,55 ~ 60,-3 03(-3 24 to -2 81)

Egypt,Deaths,60 ~ 65,-2 96(-3 14 to -2 78)

Egypt,Deaths,65 ~ 70,-2 84(-3 to -2 68)

Egypt,Deaths,70 ~ 75,-2 53(-2 68 to -2 37)

Egypt,Deaths,75 ~ 80,-2 01(-2 17 to -1 84)

Egypt,Deaths,80 ~ 85,-1 27(-1 47 to -1 07)

Egypt,Deaths,85 ~ 90,-1(-1 27 to -0 72)

Egypt,Deaths,90 ~ 95,-1 08(-1 62 to -0 54)

Egypt,Deaths,20 ~ 25,-1 95(-3 03 to -0 87)

Egypt,Deaths,25 ~ 30,-2 16(-2 93 to -1 38)

Egypt,Deaths,30 ~ 35,-2 47(-3 08 to -1 85)

Egypt,Deaths,35 ~ 40,-2 99(-3 5 to -2 48)

Egypt,Deaths,40 ~ 45,-3 34(-3 77 to -2 91)

Egypt,Deaths,45 ~ 50,-3 36(-3 71 to -3 02)

Egypt,Deaths,50 ~ 55,-3 22(-3 5 to -2 95)

Egypt,Deaths,55 ~ 60,-3 03(-3 24 to -2 81)

Egypt,Deaths,60 ~ 65,-2 96(-3 14 to -2 78)

Egypt,Deaths,65 ~ 70,-2 84(-3 to -2 68)

Egypt,Deaths,70 ~ 75,-2 53(-2 68 to -2 37)

Egypt,Deaths,75 ~ 80,-2 01(-2 17 to -1 84)

Egypt,Deaths,80 ~ 85,-1 27(-1 47 to -1 07)

Egypt,Deaths,85 ~ 90,-1(-1 27 to -0 72)

Egypt,Deaths,90 ~ 95,-1 08(-1 62 to -0 54)

Egypt,Deaths,20 ~ 25,-1 95(-3 03 to -0 87)

Egypt,Deaths,25 ~ 30,-2 16(-2 93 to -1 38)

Egypt,Deaths,30 ~ 35,-2 47(-3 08 to -1 85)

Egypt,Deaths,35 ~ 40,-2 99(-3 5 to -2 48)

Egypt,Deaths,40 ~ 45,-3 34(-3 77 to -2 91)

Egypt,Deaths,45 ~ 50,-3 36(-3 71 to -3 02)

Egypt,Deaths,50 ~ 55,-3 22(-3 5 to -2 95)

Egypt,Deaths,55 ~ 60,-3 03(-3 24 to -2 81)

Egypt,Deaths,60 ~ 65,-2 96(-3 14 to -2 78)

Egypt,Deaths,65 ~ 70,-2 84(-3 to -2 68)

Egypt,Deaths,70 ~ 75,-2 53(-2 68 to -2 37)

Egypt,Deaths,75 ~ 80,-2 01(-2 17 to -1 84)

Egypt,Deaths,80 ~ 85,-1 27(-1 47 to -1 07)

Egypt,Deaths,85 ~ 90,-1(-1 27 to -0 72)

Egypt,Deaths,90 ~ 95,-1 08(-1 62 to -0 54)

Egypt,DALYs,20 ~ 25,-1 23(-1 67 to -0 79)

Egypt,DALYs,25 ~ 30,-1 35(-1 67 to -1 02)

Egypt,DALYs,30 ~ 35,-1 56(-1 83 to -1 29)

Egypt,DALYs,35 ~ 40,-1 93(-2 17 to -1 69)

Egypt,DALYs,40 ~ 45,-2 23(-2 44 to -2 02)

Egypt,DALYs,45 ~ 50,-2 3(-2 48 to -2 11)

Egypt,DALYs,50 ~ 55,-2 26(-2 42 to -2 1)

Egypt,DALYs,55 ~ 60,-2 2(-2 34 to -2 06)

Egypt,DALYs,60 ~ 65,-2 26(-2 39 to -2 14)

Egypt,DALYs,65 ~ 70,-2 25(-2 38 to -2 13)

Egypt,DALYs,70 ~ 75,-2 05(-2 19 to -1 92)

Egypt,DALYs,75 ~ 80,-1 65(-1 82 to -1 49)

Egypt,DALYs,80 ~ 85,-1 03(-1 25 to -0 8)

Egypt,DALYs,85 ~ 90,-0 77(-1 1 to -0 43)

Egypt,DALYs,90 ~ 95,-0 82(-1 53 to -0 1)

Egypt,DALYs,20 ~ 25,-1 23(-1 67 to -0 79)

Egypt,DALYs,25 ~ 30,-1 35(-1 67 to -1 02)

Egypt,DALYs,30 ~ 35,-1 56(-1 83 to -1 29)

Egypt,DALYs,35 ~ 40,-1 93(-2 17 to -1 69)

Egypt,DALYs,40 ~ 45,-2 23(-2 44 to -2 02)

Egypt,DALYs,45 ~ 50,-2 3(-2 48 to -2 11)

Egypt,DALYs,50 ~ 55,-2 26(-2 42 to -2 1)

Egypt,DALYs,55 ~ 60,-2 2(-2 34 to -2 06)

Egypt,DALYs,60 ~ 65,-2 26(-2 39 to -2 14)

Egypt,DALYs,65 ~ 70,-2 25(-2 38 to -2 13)

Egypt,DALYs,70 ~ 75,-2 05(-2 19 to -1 92)

Egypt,DALYs,75 ~ 80,-1 65(-1 82 to -1 49)

Egypt,DALYs,80 ~ 85,-1 03(-1 25 to -0 8)

Egypt,DALYs,85 ~ 90,-0 77(-1 1 to -0 43)

Egypt,DALYs,90 ~ 95,-0 82(-1 53 to -0 1)

Egypt,DALYs,20 ~ 25,-1 23(-1 67 to -0 79)

Egypt,DALYs,25 ~ 30,-1 35(-1 67 to -1 02)

Egypt,DALYs,30 ~ 35,-1 56(-1 83 to -1 29)

Egypt,DALYs,35 ~ 40,-1 93(-2 17 to -1 69)

Egypt,DALYs,40 ~ 45,-2 23(-2 44 to -2 02)

Egypt,DALYs,45 ~ 50,-2 3(-2 48 to -2 11)

Egypt,DALYs,50 ~ 55,-2 26(-2 42 to -2 1)

Egypt,DALYs,55 ~ 60,-2 2(-2 34 to -2 06)

Egypt,DALYs,60 ~ 65,-2 26(-2 39 to -2 14)

Egypt,DALYs,65 ~ 70,-2 25(-2 38 to -2 13)

Egypt,DALYs,70 ~ 75,-2 05(-2 19 to -1 92)

Egypt,DALYs,75 ~ 80,-1 65(-1 82 to -1 49)

Egypt,DALYs,80 ~ 85,-1 03(-1 25 to -0 8)

Egypt,DALYs,85 ~ 90,-0 77(-1 1 to -0 43)

Egypt,DALYs,90 ~ 95,-0 82(-1 53 to -0 1)

Egypt,DALYs,20 ~ 25,-1 23(-1 67 to -0 79)

Egypt,DALYs,25 ~ 30,-1 35(-1 67 to -1 02)

Egypt,DALYs,30 ~ 35,-1 56(-1 83 to -1 29)

Egypt,DALYs,35 ~ 40,-1 93(-2 17 to -1 69)

Egypt,DALYs,40 ~ 45,-2 23(-2 44 to -2 02)

Egypt,DALYs,45 ~ 50,-2 3(-2 48 to -2 11)

Egypt,DALYs,50 ~ 55,-2 26(-2 42 to -2 1)

Egypt,DALYs,55 ~ 60,-2 2(-2 34 to -2 06)

Egypt,DALYs,60 ~ 65,-2 26(-2 39 to -2 14)

Egypt,DALYs,65 ~ 70,-2 25(-2 38 to -2 13)

Egypt,DALYs,70 ~ 75,-2 05(-2 19 to -1 92)

Egypt,DALYs,75 ~ 80,-1 65(-1 82 to -1 49)

Egypt,DALYs,80 ~ 85,-1 03(-1 25 to -0 8)

Egypt,DALYs,85 ~ 90,-0 77(-1 1 to -0 43)

Egypt,DALYs,90 ~ 95,-0 82(-1 53 to -0 1)

Niger,Prevalence,20 ~ 25,-0 01(-0 08 to 0 06)

Niger,Prevalence,25 ~ 30,0 02(-0 04 to 0 07)

Niger,Prevalence,30 ~ 35,0 05(0 to 0 1)

Niger,Prevalence,35 ~ 40,0 11(0 06 to 0 16)

Niger,Prevalence,40 ~ 45,0 19(0 15 to 0 24)

Niger,Prevalence,45 ~ 50,0 29(0 24 to 0 33)

Niger,Prevalence,50 ~ 55,0 39(0 35 to 0 43)

Niger,Prevalence,55 ~ 60,0 49(0 45 to 0 53)

Niger,Prevalence,60 ~ 65,0 59(0 55 to 0 63)

Niger,Prevalence,65 ~ 70,0 68(0 64 to 0 73)

Niger,Prevalence,70 ~ 75,0 78(0 73 to 0 83)

Niger,Prevalence,75 ~ 80,0 89(0 82 to 0 96)

Niger,Prevalence,80 ~ 85,1 02(0 92 to 1 11)

Niger,Prevalence,85 ~ 90,1 22(1 06 to 1 39)

Niger,Prevalence,90 ~ 95,1 51(1 16 to 1 86)

Niger,Prevalence,20 ~ 25,-0 01(-0 08 to 0 06)

Niger,Prevalence,25 ~ 30,0 02(-0 04 to 0 07)

Niger,Prevalence,30 ~ 35,0 05(0 to 0 1)

Niger,Prevalence,35 ~ 40,0 11(0 06 to 0 16)

Niger,Prevalence,40 ~ 45,0 19(0 15 to 0 24)

Niger,Prevalence,45 ~ 50,0 29(0 24 to 0 33)

Niger,Prevalence,50 ~ 55,0 39(0 35 to 0 43)

Niger,Prevalence,55 ~ 60,0 49(0 45 to 0 53)

Niger,Prevalence,60 ~ 65,0 59(0 55 to 0 63)

Niger,Prevalence,65 ~ 70,0 68(0 64 to 0 73)

Niger,Prevalence,70 ~ 75,0 78(0 73 to 0 83)

Niger,Prevalence,75 ~ 80,0 89(0 82 to 0 96)

Niger,Prevalence,80 ~ 85,1 02(0 92 to 1 11)

Niger,Prevalence,85 ~ 90,1 22(1 06 to 1 39)

Niger,Prevalence,90 ~ 95,1 51(1 16 to 1 86)

Niger,Prevalence,20 ~ 25,-0 01(-0 08 to 0 06)

Niger,Prevalence,25 ~ 30,0 02(-0 04 to 0 07)

Niger,Prevalence,30 ~ 35,0 05(0 to 0 1)

Niger,Prevalence,35 ~ 40,0 11(0 06 to 0 16)

Niger,Prevalence,40 ~ 45,0 19(0 15 to 0 24)

Niger,Prevalence,45 ~ 50,0 29(0 24 to 0 33)

Niger,Prevalence,50 ~ 55,0 39(0 35 to 0 43)

Niger,Prevalence,55 ~ 60,0 49(0 45 to 0 53)

Niger,Prevalence,60 ~ 65,0 59(0 55 to 0 63)

Niger,Prevalence,65 ~ 70,0 68(0 64 to 0 73)

Niger,Prevalence,70 ~ 75,0 78(0 73 to 0 83)

Niger,Prevalence,75 ~ 80,0 89(0 82 to 0 96)

Niger,Prevalence,80 ~ 85,1 02(0 92 to 1 11)

Niger,Prevalence,85 ~ 90,1 22(1 06 to 1 39)

Niger,Prevalence,90 ~ 95,1 51(1 16 to 1 86)

Niger,Prevalence,20 ~ 25,-0 01(-0 08 to 0 06)

Niger,Prevalence,25 ~ 30,0 02(-0 04 to 0 07)

Niger,Prevalence,30 ~ 35,0 05(0 to 0 1)

Niger,Prevalence,35 ~ 40,0 11(0 06 to 0 16)

Niger,Prevalence,40 ~ 45,0 19(0 15 to 0 24)

Niger,Prevalence,45 ~ 50,0 29(0 24 to 0 33)

Niger,Prevalence,50 ~ 55,0 39(0 35 to 0 43)

Niger,Prevalence,55 ~ 60,0 49(0 45 to 0 53)

Niger,Prevalence,60 ~ 65,0 59(0 55 to 0 63)

Niger,Prevalence,65 ~ 70,0 68(0 64 to 0 73)

Niger,Prevalence,70 ~ 75,0 78(0 73 to 0 83)

Niger,Prevalence,75 ~ 80,0 89(0 82 to 0 96)

Niger,Prevalence,80 ~ 85,1 02(0 92 to 1 11)

Niger,Prevalence,85 ~ 90,1 22(1 06 to 1 39)

Niger,Prevalence,90 ~ 95,1 51(1 16 to 1 86)

Niger,Deaths,20 ~ 25,-0 12(-2 78 to 2 61)

Niger,Deaths,25 ~ 30,-0 24(-1 85 to 1 41)

Niger,Deaths,30 ~ 35,-0 46(-1 78 to 0 87)

Niger,Deaths,35 ~ 40,-0 61(-1 74 to 0 54)

Niger,Deaths,40 ~ 45,-0 74(-1 66 to 0 2)

Niger,Deaths,45 ~ 50,-0 84(-1 56 to -0 11)

Niger,Deaths,50 ~ 55,-0 86(-1 43 to -0 29)

Niger,Deaths,55 ~ 60,-0 85(-1 32 to -0 39)

Niger,Deaths,60 ~ 65,-0 87(-1 26 to -0 48)

Niger,Deaths,65 ~ 70,-0 84(-1 18 to -0 5)

Niger,Deaths,70 ~ 75,-0 72(-1 05 to -0 39)

Niger,Deaths,75 ~ 80,-0 57(-0 94 to -0 21)

Niger,Deaths,80 ~ 85,-0 39(-0 85 to 0 08)

Niger,Deaths,85 ~ 90,-0 09(-0 77 to 0 59)

Niger,Deaths,90 ~ 95,0 39(-0 95 to 1 75)

Niger,Deaths,20 ~ 25,-0 12(-2 78 to 2 61)

Niger,Deaths,25 ~ 30,-0 24(-1 85 to 1 41)

Niger,Deaths,30 ~ 35,-0 46(-1 78 to 0 87)

Niger,Deaths,35 ~ 40,-0 61(-1 74 to 0 54)

Niger,Deaths,40 ~ 45,-0 74(-1 66 to 0 2)

Niger,Deaths,45 ~ 50,-0 84(-1 56 to -0 11)

Niger,Deaths,50 ~ 55,-0 86(-1 43 to -0 29)

Niger,Deaths,55 ~ 60,-0 85(-1 32 to -0 39)

Niger,Deaths,60 ~ 65,-0 87(-1 26 to -0 48)

Niger,Deaths,65 ~ 70,-0 84(-1 18 to -0 5)

Niger,Deaths,70 ~ 75,-0 72(-1 05 to -0 39)

Niger,Deaths,75 ~ 80,-0 57(-0 94 to -0 21)

Niger,Deaths,80 ~ 85,-0 39(-0 85 to 0 08)

Niger,Deaths,85 ~ 90,-0 09(-0 77 to 0 59)

Niger,Deaths,90 ~ 95,0 39(-0 95 to 1 75)

Niger,Deaths,20 ~ 25,-0 12(-2 78 to 2 61)

Niger,Deaths,25 ~ 30,-0 24(-1 85 to 1 41)

Niger,Deaths,30 ~ 35,-0 46(-1 78 to 0 87)

Niger,Deaths,35 ~ 40,-0 61(-1 74 to 0 54)

Niger,Deaths,40 ~ 45,-0 74(-1 66 to 0 2)

Niger,Deaths,45 ~ 50,-0 84(-1 56 to -0 11)

Niger,Deaths,50 ~ 55,-0 86(-1 43 to -0 29)

Niger,Deaths,55 ~ 60,-0 85(-1 32 to -0 39)

Niger,Deaths,60 ~ 65,-0 87(-1 26 to -0 48)

Niger,Deaths,65 ~ 70,-0 84(-1 18 to -0 5)

Niger,Deaths,70 ~ 75,-0 72(-1 05 to -0 39)

Niger,Deaths,75 ~ 80,-0 57(-0 94 to -0 21)

Niger,Deaths,80 ~ 85,-0 39(-0 85 to 0 08)

Niger,Deaths,85 ~ 90,-0 09(-0 77 to 0 59)

Niger,Deaths,90 ~ 95,0 39(-0 95 to 1 75)

Niger,Deaths,20 ~ 25,-0 12(-2 78 to 2 61)

Niger,Deaths,25 ~ 30,-0 24(-1 85 to 1 41)

Niger,Deaths,30 ~ 35,-0 46(-1 78 to 0 87)

Niger,Deaths,35 ~ 40,-0 61(-1 74 to 0 54)

Niger,Deaths,40 ~ 45,-0 74(-1 66 to 0 2)

Niger,Deaths,45 ~ 50,-0 84(-1 56 to -0 11)

Niger,Deaths,50 ~ 55,-0 86(-1 43 to -0 29)

Niger,Deaths,55 ~ 60,-0 85(-1 32 to -0 39)

Niger,Deaths,60 ~ 65,-0 87(-1 26 to -0 48)

Niger,Deaths,65 ~ 70,-0 84(-1 18 to -0 5)

Niger,Deaths,70 ~ 75,-0 72(-1 05 to -0 39)

Niger,Deaths,75 ~ 80,-0 57(-0 94 to -0 21)

Niger,Deaths,80 ~ 85,-0 39(-0 85 to 0 08)

Niger,Deaths,85 ~ 90,-0 09(-0 77 to 0 59)

Niger,Deaths,90 ~ 95,0 39(-0 95 to 1 75)

Niger,DALYs,20 ~ 25,-0 11(-0 28 to 0 07)

Niger,DALYs,25 ~ 30,-0 13(-0 26 to 0 01)

Niger,DALYs,30 ~ 35,-0 2(-0 31 to -0 08)

Niger,DALYs,35 ~ 40,-0 25(-0 35 to -0 14)

Niger,DALYs,40 ~ 45,-0 31(-0 41 to -0 21)

Niger,DALYs,45 ~ 50,-0 39(-0 47 to -0 3)

Niger,DALYs,50 ~ 55,-0 44(-0 51 to -0 36)

Niger,DALYs,55 ~ 60,-0 47(-0 53 to -0 4)

Niger,DALYs,60 ~ 65,-0 52(-0 58 to -0 45)

Niger,DALYs,65 ~ 70,-0 52(-0 59 to -0 46)

Niger,DALYs,70 ~ 75,-0 47(-0 54 to -0 4)

Niger,DALYs,75 ~ 80,-0 35(-0 44 to -0 27)

Niger,DALYs,80 ~ 85,-0 2(-0 32 to -0 07)

Niger,DALYs,85 ~ 90,0 05(-0 15 to 0 25)

Niger,DALYs,90 ~ 95,0 47(0 05 to 0 89)

Niger,DALYs,20 ~ 25,-0 11(-0 28 to 0 07)

Niger,DALYs,25 ~ 30,-0 13(-0 26 to 0 01)

Niger,DALYs,30 ~ 35,-0 2(-0 31 to -0 08)

Niger,DALYs,35 ~ 40,-0 25(-0 35 to -0 14)

Niger,DALYs,40 ~ 45,-0 31(-0 41 to -0 21)

Niger,DALYs,45 ~ 50,-0 39(-0 47 to -0 3)

Niger,DALYs,50 ~ 55,-0 44(-0 51 to -0 36)

Niger,DALYs,55 ~ 60,-0 47(-0 53 to -0 4)

Niger,DALYs,60 ~ 65,-0 52(-0 58 to -0 45)

Niger,DALYs,65 ~ 70,-0 52(-0 59 to -0 46)

Niger,DALYs,70 ~ 75,-0 47(-0 54 to -0 4)

Niger,DALYs,75 ~ 80,-0 35(-0 44 to -0 27)

Niger,DALYs,80 ~ 85,-0 2(-0 32 to -0 07)

Niger,DALYs,85 ~ 90,0 05(-0 15 to 0 25)

Niger,DALYs,90 ~ 95,0 47(0 05 to 0 89)

Niger,DALYs,20 ~ 25,-0 11(-0 28 to 0 07)

Niger,DALYs,25 ~ 30,-0 13(-0 26 to 0 01)

Niger,DALYs,30 ~ 35,-0 2(-0 31 to -0 08)

Niger,DALYs,35 ~ 40,-0 25(-0 35 to -0 14)

Niger,DALYs,40 ~ 45,-0 31(-0 41 to -0 21)

Niger,DALYs,45 ~ 50,-0 39(-0 47 to -0 3)

Niger,DALYs,50 ~ 55,-0 44(-0 51 to -0 36)

Niger,DALYs,55 ~ 60,-0 47(-0 53 to -0 4)

Niger,DALYs,60 ~ 65,-0 52(-0 58 to -0 45)

Niger,DALYs,65 ~ 70,-0 52(-0 59 to -0 46)

Niger,DALYs,70 ~ 75,-0 47(-0 54 to -0 4)

Niger,DALYs,75 ~ 80,-0 35(-0 44 to -0 27)

Niger,DALYs,80 ~ 85,-0 2(-0 32 to -0 07)

Niger,DALYs,85 ~ 90,0 05(-0 15 to 0 25)

Niger,DALYs,90 ~ 95,0 47(0 05 to 0 89)

Niger,DALYs,20 ~ 25,-0 11(-0 28 to 0 07)

Niger,DALYs,25 ~ 30,-0 13(-0 26 to 0 01)

Niger,DALYs,30 ~ 35,-0 2(-0 31 to -0 08)

Niger,DALYs,35 ~ 40,-0 25(-0 35 to -0 14)

Niger,DALYs,40 ~ 45,-0 31(-0 41 to -0 21)

Niger,DALYs,45 ~ 50,-0 39(-0 47 to -0 3)

Niger,DALYs,50 ~ 55,-0 44(-0 51 to -0 36)

Niger,DALYs,55 ~ 60,-0 47(-0 53 to -0 4)

Niger,DALYs,60 ~ 65,-0 52(-0 58 to -0 45)

Niger,DALYs,65 ~ 70,-0 52(-0 59 to -0 46)

Niger,DALYs,70 ~ 75,-0 47(-0 54 to -0 4)

Niger,DALYs,75 ~ 80,-0 35(-0 44 to -0 27)

Niger,DALYs,80 ~ 85,-0 2(-0 32 to -0 07)

Niger,DALYs,85 ~ 90,0 05(-0 15 to 0 25)

Niger,DALYs,90 ~ 95,0 47(0 05 to 0 89)

New_Zealand,Prevalence,20 ~ 25,-0 92(-1 18 to -0 65)

New_Zealand,Prevalence,25 ~ 30,-0 9(-1 08 to -0 71)

New_Zealand,Prevalence,30 ~ 35,-0 85(-0 99 to -0 7)

New_Zealand,Prevalence,35 ~ 40,-0 8(-0 92 to -0 68)

New_Zealand,Prevalence,40 ~ 45,-0 78(-0 88 to -0 68)

New_Zealand,Prevalence,45 ~ 50,-0 8(-0 88 to -0 72)

New_Zealand,Prevalence,50 ~ 55,-0 83(-0 9 to -0 77)

New_Zealand,Prevalence,55 ~ 60,-0 83(-0 89 to -0 78)

New_Zealand,Prevalence,60 ~ 65,-0 8(-0 84 to -0 75)

New_Zealand,Prevalence,65 ~ 70,-0 72(-0 76 to -0 68)

New_Zealand,Prevalence,70 ~ 75,-0 6(-0 63 to -0 56)

New_Zealand,Prevalence,75 ~ 80,-0 42(-0 46 to -0 38)

New_Zealand,Prevalence,80 ~ 85,-0 23(-0 27 to -0 18)

New_Zealand,Prevalence,85 ~ 90,-0 04(-0 11 to 0 03)

New_Zealand,Prevalence,90 ~ 95,0 11(-0 02 to 0 23)

New_Zealand,Prevalence,20 ~ 25,-0 92(-1 18 to -0 65)

New_Zealand,Prevalence,25 ~ 30,-0 9(-1 08 to -0 71)

New_Zealand,Prevalence,30 ~ 35,-0 85(-0 99 to -0 7)

New_Zealand,Prevalence,35 ~ 40,-0 8(-0 92 to -0 68)

New_Zealand,Prevalence,40 ~ 45,-0 78(-0 88 to -0 68)

New_Zealand,Prevalence,45 ~ 50,-0 8(-0 88 to -0 72)

New_Zealand,Prevalence,50 ~ 55,-0 83(-0 9 to -0 77)

New_Zealand,Prevalence,55 ~ 60,-0 83(-0 89 to -0 78)

New_Zealand,Prevalence,60 ~ 65,-0 8(-0 84 to -0 75)

New_Zealand,Prevalence,65 ~ 70,-0 72(-0 76 to -0 68)

New_Zealand,Prevalence,70 ~ 75,-0 6(-0 63 to -0 56)

New_Zealand,Prevalence,75 ~ 80,-0 42(-0 46 to -0 38)

New_Zealand,Prevalence,80 ~ 85,-0 23(-0 27 to -0 18)

New_Zealand,Prevalence,85 ~ 90,-0 04(-0 11 to 0 03)

New_Zealand,Prevalence,90 ~ 95,0 11(-0 02 to 0 23)

New_Zealand,Prevalence,20 ~ 25,-0 92(-1 18 to -0 65)

New_Zealand,Prevalence,25 ~ 30,-0 9(-1 08 to -0 71)

New_Zealand,Prevalence,30 ~ 35,-0 85(-0 99 to -0 7)

New_Zealand,Prevalence,35 ~ 40,-0 8(-0 92 to -0 68)

New_Zealand,Prevalence,40 ~ 45,-0 78(-0 88 to -0 68)

New_Zealand,Prevalence,45 ~ 50,-0 8(-0 88 to -0 72)

New_Zealand,Prevalence,50 ~ 55,-0 83(-0 9 to -0 77)

New_Zealand,Prevalence,55 ~ 60,-0 83(-0 89 to -0 78)

New_Zealand,Prevalence,60 ~ 65,-0 8(-0 84 to -0 75)

New_Zealand,Prevalence,65 ~ 70,-0 72(-0 76 to -0 68)

New_Zealand,Prevalence,70 ~ 75,-0 6(-0 63 to -0 56)

New_Zealand,Prevalence,75 ~ 80,-0 42(-0 46 to -0 38)

New_Zealand,Prevalence,80 ~ 85,-0 23(-0 27 to -0 18)

New_Zealand,Prevalence,85 ~ 90,-0 04(-0 11 to 0 03)

New_Zealand,Prevalence,90 ~ 95,0 11(-0 02 to 0 23)

New_Zealand,Prevalence,20 ~ 25,-0 92(-1 18 to -0 65)

New_Zealand,Prevalence,25 ~ 30,-0 9(-1 08 to -0 71)

New_Zealand,Prevalence,30 ~ 35,-0 85(-0 99 to -0 7)

New_Zealand,Prevalence,35 ~ 40,-0 8(-0 92 to -0 68)

New_Zealand,Prevalence,40 ~ 45,-0 78(-0 88 to -0 68)

New_Zealand,Prevalence,45 ~ 50,-0 8(-0 88 to -0 72)

New_Zealand,Prevalence,50 ~ 55,-0 83(-0 9 to -0 77)

New_Zealand,Prevalence,55 ~ 60,-0 83(-0 89 to -0 78)

New_Zealand,Prevalence,60 ~ 65,-0 8(-0 84 to -0 75)

New_Zealand,Prevalence,65 ~ 70,-0 72(-0 76 to -0 68)

New_Zealand,Prevalence,70 ~ 75,-0 6(-0 63 to -0 56)

New_Zealand,Prevalence,75 ~ 80,-0 42(-0 46 to -0 38)

New_Zealand,Prevalence,80 ~ 85,-0 23(-0 27 to -0 18)

New_Zealand,Prevalence,85 ~ 90,-0 04(-0 11 to 0 03)

New_Zealand,Prevalence,90 ~ 95,0 11(-0 02 to 0 23)

New_Zealand,Deaths,20 ~ 25,-2 89(-14 87 to 10 78)

New_Zealand,Deaths,25 ~ 30,-2 59(-11 03 to 6 65)

New_Zealand,Deaths,30 ~ 35,-2 31(-8 11 to 3 86)

New_Zealand,Deaths,35 ~ 40,-1 89(-5 58 to 1 94)

New_Zealand,Deaths,40 ~ 45,-1 69(-4 05 to 0 73)

New_Zealand,Deaths,45 ~ 50,-1 57(-3 02 to -0 09)

New_Zealand,Deaths,50 ~ 55,-1 6(-2 51 to -0 69)

New_Zealand,Deaths,55 ~ 60,-1 9(-2 51 to -1 29)

New_Zealand,Deaths,60 ~ 65,-2 33(-2 77 to -1 89)

New_Zealand,Deaths,65 ~ 70,-2 53(-2 87 to -2 19)

New_Zealand,Deaths,70 ~ 75,-2 4(-2 68 to -2 11)

New_Zealand,Deaths,75 ~ 80,-2 02(-2 27 to -1 76)

New_Zealand,Deaths,80 ~ 85,-1 5(-1 76 to -1 25)

New_Zealand,Deaths,85 ~ 90,-0 88(-1 21 to -0 54)

New_Zealand,Deaths,90 ~ 95,-0 27(-0 84 to 0 3)

New_Zealand,Deaths,20 ~ 25,-2 89(-14 87 to 10 78)

New_Zealand,Deaths,25 ~ 30,-2 59(-11 03 to 6 65)

New_Zealand,Deaths,30 ~ 35,-2 31(-8 11 to 3 86)

New_Zealand,Deaths,35 ~ 40,-1 89(-5 58 to 1 94)

New_Zealand,Deaths,40 ~ 45,-1 69(-4 05 to 0 73)

New_Zealand,Deaths,45 ~ 50,-1 57(-3 02 to -0 09)

New_Zealand,Deaths,50 ~ 55,-1 6(-2 51 to -0 69)

New_Zealand,Deaths,55 ~ 60,-1 9(-2 51 to -1 29)

New_Zealand,Deaths,60 ~ 65,-2 33(-2 77 to -1 89)

New_Zealand,Deaths,65 ~ 70,-2 53(-2 87 to -2 19)

New_Zealand,Deaths,70 ~ 75,-2 4(-2 68 to -2 11)

New_Zealand,Deaths,75 ~ 80,-2 02(-2 27 to -1 76)

New_Zealand,Deaths,80 ~ 85,-1 5(-1 76 to -1 25)

New_Zealand,Deaths,85 ~ 90,-0 88(-1 21 to -0 54)

New_Zealand,Deaths,90 ~ 95,-0 27(-0 84 to 0 3)

New_Zealand,Deaths,20 ~ 25,-2 89(-14 87 to 10 78)

New_Zealand,Deaths,25 ~ 30,-2 59(-11 03 to 6 65)

New_Zealand,Deaths,30 ~ 35,-2 31(-8 11 to 3 86)

New_Zealand,Deaths,35 ~ 40,-1 89(-5 58 to 1 94)

New_Zealand,Deaths,40 ~ 45,-1 69(-4 05 to 0 73)

New_Zealand,Deaths,45 ~ 50,-1 57(-3 02 to -0 09)

New_Zealand,Deaths,50 ~ 55,-1 6(-2 51 to -0 69)

New_Zealand,Deaths,55 ~ 60,-1 9(-2 51 to -1 29)

New_Zealand,Deaths,60 ~ 65,-2 33(-2 77 to -1 89)

New_Zealand,Deaths,65 ~ 70,-2 53(-2 87 to -2 19)

New_Zealand,Deaths,70 ~ 75,-2 4(-2 68 to -2 11)

New_Zealand,Deaths,75 ~ 80,-2 02(-2 27 to -1 76)

New_Zealand,Deaths,80 ~ 85,-1 5(-1 76 to -1 25)

New_Zealand,Deaths,85 ~ 90,-0 88(-1 21 to -0 54)

New_Zealand,Deaths,90 ~ 95,-0 27(-0 84 to 0 3)

New_Zealand,Deaths,20 ~ 25,-2 89(-14 87 to 10 78)

New_Zealand,Deaths,25 ~ 30,-2 59(-11 03 to 6 65)

New_Zealand,Deaths,30 ~ 35,-2 31(-8 11 to 3 86)

New_Zealand,Deaths,35 ~ 40,-1 89(-5 58 to 1 94)

New_Zealand,Deaths,40 ~ 45,-1 69(-4 05 to 0 73)

New_Zealand,Deaths,45 ~ 50,-1 57(-3 02 to -0 09)

New_Zealand,Deaths,50 ~ 55,-1 6(-2 51 to -0 69)

New_Zealand,Deaths,55 ~ 60,-1 9(-2 51 to -1 29)

New_Zealand,Deaths,60 ~ 65,-2 33(-2 77 to -1 89)

New_Zealand,Deaths,65 ~ 70,-2 53(-2 87 to -2 19)

New_Zealand,Deaths,70 ~ 75,-2 4(-2 68 to -2 11)

New_Zealand,Deaths,75 ~ 80,-2 02(-2 27 to -1 76)

New_Zealand,Deaths,80 ~ 85,-1 5(-1 76 to -1 25)

New_Zealand,Deaths,85 ~ 90,-0 88(-1 21 to -0 54)

New_Zealand,Deaths,90 ~ 95,-0 27(-0 84 to 0 3)

New_Zealand,DALYs,20 ~ 25,-1 58(-2 85 to -0 3)

New_Zealand,DALYs,25 ~ 30,-1 53(-2 42 to -0 64)

New_Zealand,DALYs,30 ~ 35,-1 44(-2 11 to -0 77)

New_Zealand,DALYs,35 ~ 40,-1 31(-1 81 to -0 81)

New_Zealand,DALYs,40 ~ 45,-1 26(-1 63 to -0 89)

New_Zealand,DALYs,45 ~ 50,-1 29(-1 55 to -1 03)

New_Zealand,DALYs,50 ~ 55,-1 39(-1 58 to -1 21)

New_Zealand,DALYs,55 ~ 60,-1 68(-1 81 to -1 54)

New_Zealand,DALYs,60 ~ 65,-2 06(-2 17 to -1 95)

New_Zealand,DALYs,65 ~ 70,-2 25(-2 34 to -2 16)

New_Zealand,DALYs,70 ~ 75,-2 15(-2 23 to -2 07)

New_Zealand,DALYs,75 ~ 80,-1 83(-1 92 to -1 75)

New_Zealand,DALYs,80 ~ 85,-1 39(-1 49 to -1 29)

New_Zealand,DALYs,85 ~ 90,-0 82(-0 96 to -0 67)

New_Zealand,DALYs,90 ~ 95,-0 26(-0 52 to 0)

New_Zealand,DALYs,20 ~ 25,-1 58(-2 85 to -0 3)

New_Zealand,DALYs,25 ~ 30,-1 53(-2 42 to -0 64)

New_Zealand,DALYs,30 ~ 35,-1 44(-2 11 to -0 77)

New_Zealand,DALYs,35 ~ 40,-1 31(-1 81 to -0 81)

New_Zealand,DALYs,40 ~ 45,-1 26(-1 63 to -0 89)

New_Zealand,DALYs,45 ~ 50,-1 29(-1 55 to -1 03)

New_Zealand,DALYs,50 ~ 55,-1 39(-1 58 to -1 21)

New_Zealand,DALYs,55 ~ 60,-1 68(-1 81 to -1 54)

New_Zealand,DALYs,60 ~ 65,-2 06(-2 17 to -1 95)

New_Zealand,DALYs,65 ~ 70,-2 25(-2 34 to -2 16)

New_Zealand,DALYs,70 ~ 75,-2 15(-2 23 to -2 07)

New_Zealand,DALYs,75 ~ 80,-1 83(-1 92 to -1 75)

New_Zealand,DALYs,80 ~ 85,-1 39(-1 49 to -1 29)

New_Zealand,DALYs,85 ~ 90,-0 82(-0 96 to -0 67)

New_Zealand,DALYs,90 ~ 95,-0 26(-0 52 to 0)

New_Zealand,DALYs,20 ~ 25,-1 58(-2 85 to -0 3)

New_Zealand,DALYs,25 ~ 30,-1 53(-2 42 to -0 64)

New_Zealand,DALYs,30 ~ 35,-1 44(-2 11 to -0 77)

New_Zealand,DALYs,35 ~ 40,-1 31(-1 81 to -0 81)

New_Zealand,DALYs,40 ~ 45,-1 26(-1 63 to -0 89)

New_Zealand,DALYs,45 ~ 50,-1 29(-1 55 to -1 03)

New_Zealand,DALYs,50 ~ 55,-1 39(-1 58 to -1 21)

New_Zealand,DALYs,55 ~ 60,-1 68(-1 81 to -1 54)

New_Zealand,DALYs,60 ~ 65,-2 06(-2 17 to -1 95)

New_Zealand,DALYs,65 ~ 70,-2 25(-2 34 to -2 16)

New_Zealand,DALYs,70 ~ 75,-2 15(-2 23 to -2 07)

New_Zealand,DALYs,75 ~ 80,-1 83(-1 92 to -1 75)

New_Zealand,DALYs,80 ~ 85,-1 39(-1 49 to -1 29)

New_Zealand,DALYs,85 ~ 90,-0 82(-0 96 to -0 67)

New_Zealand,DALYs,90 ~ 95,-0 26(-0 52 to 0)

New_Zealand,DALYs,20 ~ 25,-1 58(-2 85 to -0 3)

New_Zealand,DALYs,25 ~ 30,-1 53(-2 42 to -0 64)

New_Zealand,DALYs,30 ~ 35,-1 44(-2 11 to -0 77)

New_Zealand,DALYs,35 ~ 40,-1 31(-1 81 to -0 81)

New_Zealand,DALYs,40 ~ 45,-1 26(-1 63 to -0 89)

New_Zealand,DALYs,45 ~ 50,-1 29(-1 55 to -1 03)

New_Zealand,DALYs,50 ~ 55,-1 39(-1 58 to -1 21)

New_Zealand,DALYs,55 ~ 60,-1 68(-1 81 to -1 54)

New_Zealand,DALYs,60 ~ 65,-2 06(-2 17 to -1 95)

New_Zealand,DALYs,65 ~ 70,-2 25(-2 34 to -2 16)

New_Zealand,DALYs,70 ~ 75,-2 15(-2 23 to -2 07)

New_Zealand,DALYs,75 ~ 80,-1 83(-1 92 to -1 75)

New_Zealand,DALYs,80 ~ 85,-1 39(-1 49 to -1 29)

New_Zealand,DALYs,85 ~ 90,-0 82(-0 96 to -0 67)

New_Zealand,DALYs,90 ~ 95,-0 26(-0 52 to 0)

Andorra,Prevalence,20 ~ 25,-0 09(-1 28 to 1 12)

Andorra,Prevalence,25 ~ 30,-0 09(-0 9 to 0 73)

Andorra,Prevalence,30 ~ 35,-0 12(-0 74 to 0 49)

Andorra,Prevalence,35 ~ 40,-0 19(-0 68 to 0 3)

Andorra,Prevalence,40 ~ 45,-0 28(-0 68 to 0 12)

Andorra,Prevalence,45 ~ 50,-0 36(-0 7 to -0 03)

Andorra,Prevalence,50 ~ 55,-0 41(-0 7 to -0 12)

Andorra,Prevalence,55 ~ 60,-0 42(-0 69 to -0 16)

Andorra,Prevalence,60 ~ 65,-0 42(-0 66 to -0 18)

Andorra,Prevalence,65 ~ 70,-0 4(-0 63 to -0 17)

Andorra,Prevalence,70 ~ 75,-0 36(-0 58 to -0 13)

Andorra,Prevalence,75 ~ 80,-0 29(-0 53 to -0 06)

Andorra,Prevalence,80 ~ 85,-0 2(-0 47 to 0 07)

Andorra,Prevalence,85 ~ 90,-0 05(-0 43 to 0 33)

Andorra,Prevalence,90 ~ 95,0 09(-0 7 to 0 88)

Andorra,Prevalence,20 ~ 25,-0 09(-1 28 to 1 12)

Andorra,Prevalence,25 ~ 30,-0 09(-0 9 to 0 73)

Andorra,Prevalence,30 ~ 35,-0 12(-0 74 to 0 49)

Andorra,Prevalence,35 ~ 40,-0 19(-0 68 to 0 3)

Andorra,Prevalence,40 ~ 45,-0 28(-0 68 to 0 12)

Andorra,Prevalence,45 ~ 50,-0 36(-0 7 to -0 03)

Andorra,Prevalence,50 ~ 55,-0 41(-0 7 to -0 12)

Andorra,Prevalence,55 ~ 60,-0 42(-0 69 to -0 16)

Andorra,Prevalence,60 ~ 65,-0 42(-0 66 to -0 18)

Andorra,Prevalence,65 ~ 70,-0 4(-0 63 to -0 17)

Andorra,Prevalence,70 ~ 75,-0 36(-0 58 to -0 13)

Andorra,Prevalence,75 ~ 80,-0 29(-0 53 to -0 06)

Andorra,Prevalence,80 ~ 85,-0 2(-0 47 to 0 07)

Andorra,Prevalence,85 ~ 90,-0 05(-0 43 to 0 33)

Andorra,Prevalence,90 ~ 95,0 09(-0 7 to 0 88)

Andorra,Prevalence,20 ~ 25,-0 09(-1 28 to 1 12)

Andorra,Prevalence,25 ~ 30,-0 09(-0 9 to 0 73)

Andorra,Prevalence,30 ~ 35,-0 12(-0 74 to 0 49)

Andorra,Prevalence,35 ~ 40,-0 19(-0 68 to 0 3)

Andorra,Prevalence,40 ~ 45,-0 28(-0 68 to 0 12)

Andorra,Prevalence,45 ~ 50,-0 36(-0 7 to -0 03)

Andorra,Prevalence,50 ~ 55,-0 41(-0 7 to -0 12)

Andorra,Prevalence,55 ~ 60,-0 42(-0 69 to -0 16)

Andorra,Prevalence,60 ~ 65,-0 42(-0 66 to -0 18)

Andorra,Prevalence,65 ~ 70,-0 4(-0 63 to -0 17)

Andorra,Prevalence,70 ~ 75,-0 36(-0 58 to -0 13)

Andorra,Prevalence,75 ~ 80,-0 29(-0 53 to -0 06)

Andorra,Prevalence,80 ~ 85,-0 2(-0 47 to 0 07)

Andorra,Prevalence,85 ~ 90,-0 05(-0 43 to 0 33)

Andorra,Prevalence,90 ~ 95,0 09(-0 7 to 0 88)

Andorra,Prevalence,20 ~ 25,-0 09(-1 28 to 1 12)

Andorra,Prevalence,25 ~ 30,-0 09(-0 9 to 0 73)

Andorra,Prevalence,30 ~ 35,-0 12(-0 74 to 0 49)

Andorra,Prevalence,35 ~ 40,-0 19(-0 68 to 0 3)

Andorra,Prevalence,40 ~ 45,-0 28(-0 68 to 0 12)

Andorra,Prevalence,45 ~ 50,-0 36(-0 7 to -0 03)

Andorra,Prevalence,50 ~ 55,-0 41(-0 7 to -0 12)

Andorra,Prevalence,55 ~ 60,-0 42(-0 69 to -0 16)

Andorra,Prevalence,60 ~ 65,-0 42(-0 66 to -0 18)

Andorra,Prevalence,65 ~ 70,-0 4(-0 63 to -0 17)

Andorra,Prevalence,70 ~ 75,-0 36(-0 58 to -0 13)

Andorra,Prevalence,75 ~ 80,-0 29(-0 53 to -0 06)

Andorra,Prevalence,80 ~ 85,-0 2(-0 47 to 0 07)

Andorra,Prevalence,85 ~ 90,-0 05(-0 43 to 0 33)

Andorra,Prevalence,90 ~ 95,0 09(-0 7 to 0 88)

Andorra,Deaths,20 ~ 25,-1 33(-56 23 to 122 42)

Andorra,Deaths,25 ~ 30,-1 49(-42 33 to 68 26)

Andorra,Deaths,30 ~ 35,-1 62(-32 35 to 43 08)

Andorra,Deaths,35 ~ 40,-1 8(-23 58 to 26 18)

Andorra,Deaths,40 ~ 45,-2 1(-16 68 to 15 03)

Andorra,Deaths,45 ~ 50,-2 36(-12 01 to 8 34)

Andorra,Deaths,50 ~ 55,-2 43(-9 03 to 4 65)

Andorra,Deaths,55 ~ 60,-2 42(-7 19 to 2 6)

Andorra,Deaths,60 ~ 65,-2 17(-5 83 to 1 63)

Andorra,Deaths,65 ~ 70,-1 95(-4 87 to 1 05)

Andorra,Deaths,70 ~ 75,-1 84(-4 24 to 0 63)

Andorra,Deaths,75 ~ 80,-1 93(-4 04 to 0 24)

Andorra,Deaths,80 ~ 85,-1 95(-3 99 to 0 13)

Andorra,Deaths,85 ~ 90,-1 63(-4 08 to 0 88)

Andorra,Deaths,90 ~ 95,-1 31(-5 98 to 3 59)

Andorra,Deaths,20 ~ 25,-1 33(-56 23 to 122 42)

Andorra,Deaths,25 ~ 30,-1 49(-42 33 to 68 26)

Andorra,Deaths,30 ~ 35,-1 62(-32 35 to 43 08)

Andorra,Deaths,35 ~ 40,-1 8(-23 58 to 26 18)

Andorra,Deaths,40 ~ 45,-2 1(-16 68 to 15 03)

Andorra,Deaths,45 ~ 50,-2 36(-12 01 to 8 34)

Andorra,Deaths,50 ~ 55,-2 43(-9 03 to 4 65)

Andorra,Deaths,55 ~ 60,-2 42(-7 19 to 2 6)

Andorra,Deaths,60 ~ 65,-2 17(-5 83 to 1 63)

Andorra,Deaths,65 ~ 70,-1 95(-4 87 to 1 05)

Andorra,Deaths,70 ~ 75,-1 84(-4 24 to 0 63)

Andorra,Deaths,75 ~ 80,-1 93(-4 04 to 0 24)

Andorra,Deaths,80 ~ 85,-1 95(-3 99 to 0 13)

Andorra,Deaths,85 ~ 90,-1 63(-4 08 to 0 88)

Andorra,Deaths,90 ~ 95,-1 31(-5 98 to 3 59)

Andorra,Deaths,20 ~ 25,-1 33(-56 23 to 122 42)

Andorra,Deaths,25 ~ 30,-1 49(-42 33 to 68 26)

Andorra,Deaths,30 ~ 35,-1 62(-32 35 to 43 08)

Andorra,Deaths,35 ~ 40,-1 8(-23 58 to 26 18)

Andorra,Deaths,40 ~ 45,-2 1(-16 68 to 15 03)

Andorra,Deaths,45 ~ 50,-2 36(-12 01 to 8 34)

Andorra,Deaths,50 ~ 55,-2 43(-9 03 to 4 65)

Andorra,Deaths,55 ~ 60,-2 42(-7 19 to 2 6)

Andorra,Deaths,60 ~ 65,-2 17(-5 83 to 1 63)

Andorra,Deaths,65 ~ 70,-1 95(-4 87 to 1 05)

Andorra,Deaths,70 ~ 75,-1 84(-4 24 to 0 63)

Andorra,Deaths,75 ~ 80,-1 93(-4 04 to 0 24)

Andorra,Deaths,80 ~ 85,-1 95(-3 99 to 0 13)

Andorra,Deaths,85 ~ 90,-1 63(-4 08 to 0 88)

Andorra,Deaths,90 ~ 95,-1 31(-5 98 to 3 59)

Andorra,Deaths,20 ~ 25,-1 33(-56 23 to 122 42)

Andorra,Deaths,25 ~ 30,-1 49(-42 33 to 68 26)

Andorra,Deaths,30 ~ 35,-1 62(-32 35 to 43 08)

Andorra,Deaths,35 ~ 40,-1 8(-23 58 to 26 18)

Andorra,Deaths,40 ~ 45,-2 1(-16 68 to 15 03)

Andorra,Deaths,45 ~ 50,-2 36(-12 01 to 8 34)

Andorra,Deaths,50 ~ 55,-2 43(-9 03 to 4 65)

Andorra,Deaths,55 ~ 60,-2 42(-7 19 to 2 6)

Andorra,Deaths,60 ~ 65,-2 17(-5 83 to 1 63)

Andorra,Deaths,65 ~ 70,-1 95(-4 87 to 1 05)

Andorra,Deaths,70 ~ 75,-1 84(-4 24 to 0 63)

Andorra,Deaths,75 ~ 80,-1 93(-4 04 to 0 24)

Andorra,Deaths,80 ~ 85,-1 95(-3 99 to 0 13)

Andorra,Deaths,85 ~ 90,-1 63(-4 08 to 0 88)

Andorra,Deaths,90 ~ 95,-1 31(-5 98 to 3 59)

Andorra,DALYs,20 ~ 25,-0 43(-5 36 to 4 75)

Andorra,DALYs,25 ~ 30,-0 53(-3 88 to 2 94)

Andorra,DALYs,30 ~ 35,-0 64(-3 14 to 1 93)

Andorra,DALYs,35 ~ 40,-0 86(-2 77 to 1 08)

Andorra,DALYs,40 ~ 45,-1 21(-2 65 to 0 25)

Andorra,DALYs,45 ~ 50,-1 53(-2 62 to -0 43)

Andorra,DALYs,50 ~ 55,-1 69(-2 55 to -0 83)

Andorra,DALYs,55 ~ 60,-1 78(-2 48 to -1 08)

Andorra,DALYs,60 ~ 65,-1 69(-2 28 to -1 09)

Andorra,DALYs,65 ~ 70,-1 59(-2 11 to -1 07)

Andorra,DALYs,70 ~ 75,-1 55(-2 03 to -1 07)

Andorra,DALYs,75 ~ 80,-1 64(-2 12 to -1 16)

Andorra,DALYs,80 ~ 85,-1 68(-2 21 to -1 15)

Andorra,DALYs,85 ~ 90,-1 42(-2 14 to -0 7)

Andorra,DALYs,90 ~ 95,-1 15(-2 62 to 0 34)

Andorra,DALYs,20 ~ 25,-0 43(-5 36 to 4 75)

Andorra,DALYs,25 ~ 30,-0 53(-3 88 to 2 94)

Andorra,DALYs,30 ~ 35,-0 64(-3 14 to 1 93)

Andorra,DALYs,35 ~ 40,-0 86(-2 77 to 1 08)

Andorra,DALYs,40 ~ 45,-1 21(-2 65 to 0 25)

Andorra,DALYs,45 ~ 50,-1 53(-2 62 to -0 43)

Andorra,DALYs,50 ~ 55,-1 69(-2 55 to -0 83)

Andorra,DALYs,55 ~ 60,-1 78(-2 48 to -1 08)

Andorra,DALYs,60 ~ 65,-1 69(-2 28 to -1 09)

Andorra,DALYs,65 ~ 70,-1 59(-2 11 to -1 07)

Andorra,DALYs,70 ~ 75,-1 55(-2 03 to -1 07)

Andorra,DALYs,75 ~ 80,-1 64(-2 12 to -1 16)

Andorra,DALYs,80 ~ 85,-1 68(-2 21 to -1 15)

Andorra,DALYs,85 ~ 90,-1 42(-2 14 to -0 7)

Andorra,DALYs,90 ~ 95,-1 15(-2 62 to 0 34)

Andorra,DALYs,20 ~ 25,-0 43(-5 36 to 4 75)

Andorra,DALYs,25 ~ 30,-0 53(-3 88 to 2 94)

Andorra,DALYs,30 ~ 35,-0 64(-3 14 to 1 93)

Andorra,DALYs,35 ~ 40,-0 86(-2 77 to 1 08)

Andorra,DALYs,40 ~ 45,-1 21(-2 65 to 0 25)

Andorra,DALYs,45 ~ 50,-1 53(-2 62 to -0 43)

Andorra,DALYs,50 ~ 55,-1 69(-2 55 to -0 83)

Andorra,DALYs,55 ~ 60,-1 78(-2 48 to -1 08)

Andorra,DALYs,60 ~ 65,-1 69(-2 28 to -1 09)

Andorra,DALYs,65 ~ 70,-1 59(-2 11 to -1 07)

Andorra,DALYs,70 ~ 75,-1 55(-2 03 to -1 07)

Andorra,DALYs,75 ~ 80,-1 64(-2 12 to -1 16)

Andorra,DALYs,80 ~ 85,-1 68(-2 21 to -1 15)

Andorra,DALYs,85 ~ 90,-1 42(-2 14 to -0 7)

Andorra,DALYs,90 ~ 95,-1 15(-2 62 to 0 34)

Andorra,DALYs,20 ~ 25,-0 43(-5 36 to 4 75)

Andorra,DALYs,25 ~ 30,-0 53(-3 88 to 2 94)

Andorra,DALYs,30 ~ 35,-0 64(-3 14 to 1 93)

Andorra,DALYs,35 ~ 40,-0 86(-2 77 to 1 08)

Andorra,DALYs,40 ~ 45,-1 21(-2 65 to 0 25)

Andorra,DALYs,45 ~ 50,-1 53(-2 62 to -0 43)

Andorra,DALYs,50 ~ 55,-1 69(-2 55 to -0 83)

Andorra,DALYs,55 ~ 60,-1 78(-2 48 to -1 08)

Andorra,DALYs,60 ~ 65,-1 69(-2 28 to -1 09)

Andorra,DALYs,65 ~ 70,-1 59(-2 11 to -1 07)

Andorra,DALYs,70 ~ 75,-1 55(-2 03 to -1 07)

Andorra,DALYs,75 ~ 80,-1 64(-2 12 to -1 16)

Andorra,DALYs,80 ~ 85,-1 68(-2 21 to -1 15)

Andorra,DALYs,85 ~ 90,-1 42(-2 14 to -0 7)

Andorra,DALYs,90 ~ 95,-1 15(-2 62 to 0 34)

Democratic_People's_Republic_of_Korea,Prevalence,20 ~ 25,-0 4(-0 56 to -0 24)

Democratic_People's_Republic_of_Korea,Prevalence,25 ~ 30,-0 43(-0 54 to -0 32)

Democratic_People's_Republic_of_Korea,Prevalence,30 ~ 35,-0 47(-0 56 to -0 39)

Democratic_People's_Republic_of_Korea,Prevalence,35 ~ 40,-0 52(-0 6 to -0 44)

Democratic_People's_Republic_of_Korea,Prevalence,40 ~ 45,-0 58(-0 64 to -0 51)

Democratic_People's_Republic_of_Korea,Prevalence,45 ~ 50,-0 63(-0 69 to -0 58)

Democratic_People's_Republic_of_Korea,Prevalence,50 ~ 55,-0 66(-0 7 to -0 61)

Democratic_People's_Republic_of_Korea,Prevalence,55 ~ 60,-0 65(-0 69 to -0 61)

Democratic_People's_Republic_of_Korea,Prevalence,60 ~ 65,-0 64(-0 68 to -0 6)

Democratic_People's_Republic_of_Korea,Prevalence,65 ~ 70,-0 63(-0 67 to -0 59)

Democratic_People's_Republic_of_Korea,Prevalence,70 ~ 75,-0 57(-0 61 to -0 53)

Democratic_People's_Republic_of_Korea,Prevalence,75 ~ 80,-0 46(-0 51 to -0 42)

Democratic_People's_Republic_of_Korea,Prevalence,80 ~ 85,-0 29(-0 36 to -0 23)

Democratic_People's_Republic_of_Korea,Prevalence,85 ~ 90,-0 1(-0 2 to 0 01)

Democratic_People's_Republic_of_Korea,Prevalence,90 ~ 95,0 1(-0 1 to 0 3)

Democratic_People's_Republic_of_Korea,Prevalence,20 ~ 25,-0 4(-0 56 to -0 24)

Democratic_People's_Republic_of_Korea,Prevalence,25 ~ 30,-0 43(-0 54 to -0 32)

Democratic_People's_Republic_of_Korea,Prevalence,30 ~ 35,-0 47(-0 56 to -0 39)

Democratic_People's_Republic_of_Korea,Prevalence,35 ~ 40,-0 52(-0 6 to -0 44)

Democratic_People's_Republic_of_Korea,Prevalence,40 ~ 45,-0 58(-0 64 to -0 51)

Democratic_People's_Republic_of_Korea,Prevalence,45 ~ 50,-0 63(-0 69 to -0 58)

Democratic_People's_Republic_of_Korea,Prevalence,50 ~ 55,-0 66(-0 7 to -0 61)

Democratic_People's_Republic_of_Korea,Prevalence,55 ~ 60,-0 65(-0 69 to -0 61)

Democratic_People's_Republic_of_Korea,Prevalence,60 ~ 65,-0 64(-0 68 to -0 6)

Democratic_People's_Republic_of_Korea,Prevalence,65 ~ 70,-0 63(-0 67 to -0 59)

Democratic_People's_Republic_of_Korea,Prevalence,70 ~ 75,-0 57(-0 61 to -0 53)

Democratic_People's_Republic_of_Korea,Prevalence,75 ~ 80,-0 46(-0 51 to -0 42)

Democratic_People's_Republic_of_Korea,Prevalence,80 ~ 85,-0 29(-0 36 to -0 23)

Democratic_People's_Republic_of_Korea,Prevalence,85 ~ 90,-0 1(-0 2 to 0 01)

Democratic_People's_Republic_of_Korea,Prevalence,90 ~ 95,0 1(-0 1 to 0 3)

Democratic_People's_Republic_of_Korea,Prevalence,20 ~ 25,-0 4(-0 56 to -0 24)

Democratic_People's_Republic_of_Korea,Prevalence,25 ~ 30,-0 43(-0 54 to -0 32)

Democratic_People's_Republic_of_Korea,Prevalence,30 ~ 35,-0 47(-0 56 to -0 39)

Democratic_People's_Republic_of_Korea,Prevalence,35 ~ 40,-0 52(-0 6 to -0 44)

Democratic_People's_Republic_of_Korea,Prevalence,40 ~ 45,-0 58(-0 64 to -0 51)

Democratic_People's_Republic_of_Korea,Prevalence,45 ~ 50,-0 63(-0 69 to -0 58)

Democratic_People's_Republic_of_Korea,Prevalence,50 ~ 55,-0 66(-0 7 to -0 61)

Democratic_People's_Republic_of_Korea,Prevalence,55 ~ 60,-0 65(-0 69 to -0 61)

Democratic_People's_Republic_of_Korea,Prevalence,60 ~ 65,-0 64(-0 68 to -0 6)

Democratic_People's_Republic_of_Korea,Prevalence,65 ~ 70,-0 63(-0 67 to -0 59)

Democratic_People's_Republic_of_Korea,Prevalence,70 ~ 75,-0 57(-0 61 to -0 53)

Democratic_People's_Republic_of_Korea,Prevalence,75 ~ 80,-0 46(-0 51 to -0 42)

Democratic_People's_Republic_of_Korea,Prevalence,80 ~ 85,-0 29(-0 36 to -0 23)

Democratic_People's_Republic_of_Korea,Prevalence,85 ~ 90,-0 1(-0 2 to 0 01)

Democratic_People's_Republic_of_Korea,Prevalence,90 ~ 95,0 1(-0 1 to 0 3)

Democratic_People's_Republic_of_Korea,Prevalence,20 ~ 25,-0 4(-0 56 to -0 24)

Democratic_People's_Republic_of_Korea,Prevalence,25 ~ 30,-0 43(-0 54 to -0 32)

Democratic_People's_Republic_of_Korea,Prevalence,30 ~ 35,-0 47(-0 56 to -0 39)

Democratic_People's_Republic_of_Korea,Prevalence,35 ~ 40,-0 52(-0 6 to -0 44)

Democratic_People's_Republic_of_Korea,Prevalence,40 ~ 45,-0 58(-0 64 to -0 51)

Democratic_People's_Republic_of_Korea,Prevalence,45 ~ 50,-0 63(-0 69 to -0 58)

Democratic_People's_Republic_of_Korea,Prevalence,50 ~ 55,-0 66(-0 7 to -0 61)

Democratic_People's_Republic_of_Korea,Prevalence,55 ~ 60,-0 65(-0 69 to -0 61)

Democratic_People's_Republic_of_Korea,Prevalence,60 ~ 65,-0 64(-0 68 to -0 6)

Democratic_People's_Republic_of_Korea,Prevalence,65 ~ 70,-0 63(-0 67 to -0 59)

Democratic_People's_Republic_of_Korea,Prevalence,70 ~ 75,-0 57(-0 61 to -0 53)

Democratic_People's_Republic_of_Korea,Prevalence,75 ~ 80,-0 46(-0 51 to -0 42)

Democratic_People's_Republic_of_Korea,Prevalence,80 ~ 85,-0 29(-0 36 to -0 23)

Democratic_People's_Republic_of_Korea,Prevalence,85 ~ 90,-0 1(-0 2 to 0 01)

Democratic_People's_Republic_of_Korea,Prevalence,90 ~ 95,0 1(-0 1 to 0 3)

Democratic_People's_Republic_of_Korea,Deaths,20 ~ 25,-0 69(-2 24 to 0 88)

Democratic_People's_Republic_of_Korea,Deaths,25 ~ 30,-0 67(-1 66 to 0 33)

Democratic_People's_Republic_of_Korea,Deaths,30 ~ 35,-0 68(-1 37 to 0 02)

Democratic_People's_Republic_of_Korea,Deaths,35 ~ 40,-0 72(-1 23 to -0 21)

Democratic_People's_Republic_of_Korea,Deaths,40 ~ 45,-0 8(-1 16 to -0 44)

Democratic_People's_Republic_of_Korea,Deaths,45 ~ 50,-0 91(-1 15 to -0 66)

Democratic_People's_Republic_of_Korea,Deaths,50 ~ 55,-1 06(-1 22 to -0 89)

Democratic_People's_Republic_of_Korea,Deaths,55 ~ 60,-1 09(-1 22 to -0 97)

Democratic_People's_Republic_of_Korea,Deaths,60 ~ 65,-1 13(-1 23 to -1 03)

Democratic_People's_Republic_of_Korea,Deaths,65 ~ 70,-1 16(-1 25 to -1 08)

Democratic_People's_Republic_of_Korea,Deaths,70 ~ 75,-1 2(-1 28 to -1 13)

Democratic_People's_Republic_of_Korea,Deaths,75 ~ 80,-1 27(-1 34 to -1 21)

Democratic_People's_Republic_of_Korea,Deaths,80 ~ 85,-1 37(-1 44 to -1 29)

Democratic_People's_Republic_of_Korea,Deaths,85 ~ 90,-1 45(-1 55 to -1 35)

Democratic_People's_Republic_of_Korea,Deaths,90 ~ 95,-1 36(-1 53 to -1 19)

Democratic_People's_Republic_of_Korea,Deaths,20 ~ 25,-0 69(-2 24 to 0 88)

Democratic_People's_Republic_of_Korea,Deaths,25 ~ 30,-0 67(-1 66 to 0 33)

Democratic_People's_Republic_of_Korea,Deaths,30 ~ 35,-0 68(-1 37 to 0 02)

Democratic_People's_Republic_of_Korea,Deaths,35 ~ 40,-0 72(-1 23 to -0 21)

Democratic_People's_Republic_of_Korea,Deaths,40 ~ 45,-0 8(-1 16 to -0 44)

Democratic_People's_Republic_of_Korea,Deaths,45 ~ 50,-0 91(-1 15 to -0 66)

Democratic_People's_Republic_of_Korea,Deaths,50 ~ 55,-1 06(-1 22 to -0 89)

Democratic_People's_Republic_of_Korea,Deaths,55 ~ 60,-1 09(-1 22 to -0 97)

Democratic_People's_Republic_of_Korea,Deaths,60 ~ 65,-1 13(-1 23 to -1 03)

Democratic_People's_Republic_of_Korea,Deaths,65 ~ 70,-1 16(-1 25 to -1 08)

Democratic_People's_Republic_of_Korea,Deaths,70 ~ 75,-1 2(-1 28 to -1 13)

Democratic_People's_Republic_of_Korea,Deaths,75 ~ 80,-1 27(-1 34 to -1 21)

Democratic_People's_Republic_of_Korea,Deaths,80 ~ 85,-1 37(-1 44 to -1 29)

Democratic_People's_Republic_of_Korea,Deaths,85 ~ 90,-1 45(-1 55 to -1 35)

Democratic_People's_Republic_of_Korea,Deaths,90 ~ 95,-1 36(-1 53 to -1 19)

Democratic_People's_Republic_of_Korea,Deaths,20 ~ 25,-0 69(-2 24 to 0 88)

Democratic_People's_Republic_of_Korea,Deaths,25 ~ 30,-0 67(-1 66 to 0 33)

Democratic_People's_Republic_of_Korea,Deaths,30 ~ 35,-0 68(-1 37 to 0 02)

Democratic_People's_Republic_of_Korea,Deaths,35 ~ 40,-0 72(-1 23 to -0 21)

Democratic_People's_Republic_of_Korea,Deaths,40 ~ 45,-0 8(-1 16 to -0 44)

Democratic_People's_Republic_of_Korea,Deaths,45 ~ 50,-0 91(-1 15 to -0 66)

Democratic_People's_Republic_of_Korea,Deaths,50 ~ 55,-1 06(-1 22 to -0 89)

Democratic_People's_Republic_of_Korea,Deaths,55 ~ 60,-1 09(-1 22 to -0 97)

Democratic_People's_Republic_of_Korea,Deaths,60 ~ 65,-1 13(-1 23 to -1 03)

Democratic_People's_Republic_of_Korea,Deaths,65 ~ 70,-1 16(-1 25 to -1 08)

Democratic_People's_Republic_of_Korea,Deaths,70 ~ 75,-1 2(-1 28 to -1 13)

Democratic_People's_Republic_of_Korea,Deaths,75 ~ 80,-1 27(-1 34 to -1 21)

Democratic_People's_Republic_of_Korea,Deaths,80 ~ 85,-1 37(-1 44 to -1 29)

Democratic_People's_Republic_of_Korea,Deaths,85 ~ 90,-1 45(-1 55 to -1 35)

Democratic_People's_Republic_of_Korea,Deaths,90 ~ 95,-1 36(-1 53 to -1 19)

Democratic_People's_Republic_of_Korea,Deaths,20 ~ 25,-0 69(-2 24 to 0 88)

Democratic_People's_Republic_of_Korea,Deaths,25 ~ 30,-0 67(-1 66 to 0 33)

Democratic_People's_Republic_of_Korea,Deaths,30 ~ 35,-0 68(-1 37 to 0 02)

Democratic_People's_Republic_of_Korea,Deaths,35 ~ 40,-0 72(-1 23 to -0 21)

Democratic_People's_Republic_of_Korea,Deaths,40 ~ 45,-0 8(-1 16 to -0 44)

Democratic_People's_Republic_of_Korea,Deaths,45 ~ 50,-0 91(-1 15 to -0 66)

Democratic_People's_Republic_of_Korea,Deaths,50 ~ 55,-1 06(-1 22 to -0 89)

Democratic_People's_Republic_of_Korea,Deaths,55 ~ 60,-1 09(-1 22 to -0 97)

Democratic_People's_Republic_of_Korea,Deaths,60 ~ 65,-1 13(-1 23 to -1 03)

Democratic_People's_Republic_of_Korea,Deaths,65 ~ 70,-1 16(-1 25 to -1 08)

Democratic_People's_Republic_of_Korea,Deaths,70 ~ 75,-1 2(-1 28 to -1 13)

Democratic_People's_Republic_of_Korea,Deaths,75 ~ 80,-1 27(-1 34 to -1 21)

Democratic_People's_Republic_of_Korea,Deaths,80 ~ 85,-1 37(-1 44 to -1 29)

Democratic_People's_Republic_of_Korea,Deaths,85 ~ 90,-1 45(-1 55 to -1 35)

Democratic_People's_Republic_of_Korea,Deaths,90 ~ 95,-1 36(-1 53 to -1 19)

Democratic_People's_Republic_of_Korea,DALYs,20 ~ 25,-0 55(-0 97 to -0 13)

Democratic_People's_Republic_of_Korea,DALYs,25 ~ 30,-0 58(-0 86 to -0 3)

Democratic_People's_Republic_of_Korea,DALYs,30 ~ 35,-0 63(-0 84 to -0 42)

Democratic_People's_Republic_of_Korea,DALYs,35 ~ 40,-0 7(-0 86 to -0 53)

Democratic_People's_Republic_of_Korea,DALYs,40 ~ 45,-0 78(-0 91 to -0 65)

Democratic_People's_Republic_of_Korea,DALYs,45 ~ 50,-0 88(-0 98 to -0 78)

Democratic_People's_Republic_of_Korea,DALYs,50 ~ 55,-1 01(-1 09 to -0 94)

Democratic_People's_Republic_of_Korea,DALYs,55 ~ 60,-1 06(-1 12 to -0 99)

Democratic_People's_Republic_of_Korea,DALYs,60 ~ 65,-1 09(-1 15 to -1 04)

Democratic_People's_Republic_of_Korea,DALYs,65 ~ 70,-1 13(-1 17 to -1 08)

Democratic_People's_Republic_of_Korea,DALYs,70 ~ 75,-1 17(-1 21 to -1 12)

Democratic_People's_Republic_of_Korea,DALYs,75 ~ 80,-1 23(-1 28 to -1 18)

Democratic_People's_Republic_of_Korea,DALYs,80 ~ 85,-1 3(-1 37 to -1 24)

Democratic_People's_Republic_of_Korea,DALYs,85 ~ 90,-1 37(-1 46 to -1 28)

Democratic_People's_Republic_of_Korea,DALYs,90 ~ 95,-1 28(-1 45 to -1 12)

Democratic_People's_Republic_of_Korea,DALYs,20 ~ 25,-0 55(-0 97 to -0 13)

Democratic_People's_Republic_of_Korea,DALYs,25 ~ 30,-0 58(-0 86 to -0 3)

Democratic_People's_Republic_of_Korea,DALYs,30 ~ 35,-0 63(-0 84 to -0 42)

Democratic_People's_Republic_of_Korea,DALYs,35 ~ 40,-0 7(-0 86 to -0 53)

Democratic_People's_Republic_of_Korea,DALYs,40 ~ 45,-0 78(-0 91 to -0 65)

Democratic_People's_Republic_of_Korea,DALYs,45 ~ 50,-0 88(-0 98 to -0 78)

Democratic_People's_Republic_of_Korea,DALYs,50 ~ 55,-1 01(-1 09 to -0 94)

Democratic_People's_Republic_of_Korea,DALYs,55 ~ 60,-1 06(-1 12 to -0 99)

Democratic_People's_Republic_of_Korea,DALYs,60 ~ 65,-1 09(-1 15 to -1 04)

Democratic_People's_Republic_of_Korea,DALYs,65 ~ 70,-1 13(-1 17 to -1 08)

Democratic_People's_Republic_of_Korea,DALYs,70 ~ 75,-1 17(-1 21 to -1 12)

Democratic_People's_Republic_of_Korea,DALYs,75 ~ 80,-1 23(-1 28 to -1 18)

Democratic_People's_Republic_of_Korea,DALYs,80 ~ 85,-1 3(-1 37 to -1 24)

Democratic_People's_Republic_of_Korea,DALYs,85 ~ 90,-1 37(-1 46 to -1 28)

Democratic_People's_Republic_of_Korea,DALYs,90 ~ 95,-1 28(-1 45 to -1 12)

Democratic_People's_Republic_of_Korea,DALYs,20 ~ 25,-0 55(-0 97 to -0 13)

Democratic_People's_Republic_of_Korea,DALYs,25 ~ 30,-0 58(-0 86 to -0 3)

Democratic_People's_Republic_of_Korea,DALYs,30 ~ 35,-0 63(-0 84 to -0 42)

Democratic_People's_Republic_of_Korea,DALYs,35 ~ 40,-0 7(-0 86 to -0 53)

Democratic_People's_Republic_of_Korea,DALYs,40 ~ 45,-0 78(-0 91 to -0 65)

Democratic_People's_Republic_of_Korea,DALYs,45 ~ 50,-0 88(-0 98 to -0 78)

Democratic_People's_Republic_of_Korea,DALYs,50 ~ 55,-1 01(-1 09 to -0 94)

Democratic_People's_Republic_of_Korea,DALYs,55 ~ 60,-1 06(-1 12 to -0 99)

Democratic_People's_Republic_of_Korea,DALYs,60 ~ 65,-1 09(-1 15 to -1 04)

Democratic_People's_Republic_of_Korea,DALYs,65 ~ 70,-1 13(-1 17 to -1 08)

Democratic_People's_Republic_of_Korea,DALYs,70 ~ 75,-1 17(-1 21 to -1 12)

Democratic_People's_Republic_of_Korea,DALYs,75 ~ 80,-1 23(-1 28 to -1 18)

Democratic_People's_Republic_of_Korea,DALYs,80 ~ 85,-1 3(-1 37 to -1 24)

Democratic_People's_Republic_of_Korea,DALYs,85 ~ 90,-1 37(-1 46 to -1 28)

Democratic_People's_Republic_of_Korea,DALYs,90 ~ 95,-1 28(-1 45 to -1 12)

Democratic_People's_Republic_of_Korea,DALYs,20 ~ 25,-0 55(-0 97 to -0 13)

Democratic_People's_Republic_of_Korea,DALYs,25 ~ 30,-0 58(-0 86 to -0 3)

Democratic_People's_Republic_of_Korea,DALYs,30 ~ 35,-0 63(-0 84 to -0 42)

Democratic_People's_Republic_of_Korea,DALYs,35 ~ 40,-0 7(-0 86 to -0 53)

Democratic_People's_Republic_of_Korea,DALYs,40 ~ 45,-0 78(-0 91 to -0 65)

Democratic_People's_Republic_of_Korea,DALYs,45 ~ 50,-0 88(-0 98 to -0 78)

Democratic_People's_Republic_of_Korea,DALYs,50 ~ 55,-1 01(-1 09 to -0 94)

Democratic_People's_Republic_of_Korea,DALYs,55 ~ 60,-1 06(-1 12 to -0 99)

Democratic_People's_Republic_of_Korea,DALYs,60 ~ 65,-1 09(-1 15 to -1 04)

Democratic_People's_Republic_of_Korea,DALYs,65 ~ 70,-1 13(-1 17 to -1 08)

Democratic_People's_Republic_of_Korea,DALYs,70 ~ 75,-1 17(-1 21 to -1 12)

Democratic_People's_Republic_of_Korea,DALYs,75 ~ 80,-1 23(-1 28 to -1 18)

Democratic_People's_Republic_of_Korea,DALYs,80 ~ 85,-1 3(-1 37 to -1 24)

Democratic_People's_Republic_of_Korea,DALYs,85 ~ 90,-1 37(-1 46 to -1 28)

Democratic_People's_Republic_of_Korea,DALYs,90 ~ 95,-1 28(-1 45 to -1 12)

Iceland,Prevalence,20 ~ 25,-0 05(-0 57 to 0 47)

Iceland,Prevalence,25 ~ 30,-0 14(-0 5 to 0 22)

Iceland,Prevalence,30 ~ 35,-0 27(-0 56 to 0 02)

Iceland,Prevalence,35 ~ 40,-0 4(-0 65 to -0 15)

Iceland,Prevalence,40 ~ 45,-0 55(-0 76 to -0 33)

Iceland,Prevalence,45 ~ 50,-0 67(-0 85 to -0 49)

Iceland,Prevalence,50 ~ 55,-0 73(-0 88 to -0 58)

Iceland,Prevalence,55 ~ 60,-0 74(-0 87 to -0 61)

Iceland,Prevalence,60 ~ 65,-0 74(-0 85 to -0 62)

Iceland,Prevalence,65 ~ 70,-0 71(-0 82 to -0 6)

Iceland,Prevalence,70 ~ 75,-0 65(-0 75 to -0 54)

Iceland,Prevalence,75 ~ 80,-0 54(-0 65 to -0 43)

Iceland,Prevalence,80 ~ 85,-0 41(-0 53 to -0 29)

Iceland,Prevalence,85 ~ 90,-0 28(-0 43 to -0 12)

Iceland,Prevalence,90 ~ 95,-0 18(-0 44 to 0 07)

Iceland,Prevalence,20 ~ 25,-0 05(-0 57 to 0 47)

Iceland,Prevalence,25 ~ 30,-0 14(-0 5 to 0 22)

Iceland,Prevalence,30 ~ 35,-0 27(-0 56 to 0 02)

Iceland,Prevalence,35 ~ 40,-0 4(-0 65 to -0 15)

Iceland,Prevalence,40 ~ 45,-0 55(-0 76 to -0 33)

Iceland,Prevalence,45 ~ 50,-0 67(-0 85 to -0 49)

Iceland,Prevalence,50 ~ 55,-0 73(-0 88 to -0 58)

Iceland,Prevalence,55 ~ 60,-0 74(-0 87 to -0 61)

Iceland,Prevalence,60 ~ 65,-0 74(-0 85 to -0 62)

Iceland,Prevalence,65 ~ 70,-0 71(-0 82 to -0 6)

Iceland,Prevalence,70 ~ 75,-0 65(-0 75 to -0 54)

Iceland,Prevalence,75 ~ 80,-0 54(-0 65 to -0 43)

Iceland,Prevalence,80 ~ 85,-0 41(-0 53 to -0 29)

Iceland,Prevalence,85 ~ 90,-0 28(-0 43 to -0 12)

Iceland,Prevalence,90 ~ 95,-0 18(-0 44 to 0 07)

Iceland,Prevalence,20 ~ 25,-0 05(-0 57 to 0 47)

Iceland,Prevalence,25 ~ 30,-0 14(-0 5 to 0 22)

Iceland,Prevalence,30 ~ 35,-0 27(-0 56 to 0 02)

Iceland,Prevalence,35 ~ 40,-0 4(-0 65 to -0 15)

Iceland,Prevalence,40 ~ 45,-0 55(-0 76 to -0 33)

Iceland,Prevalence,45 ~ 50,-0 67(-0 85 to -0 49)

Iceland,Prevalence,50 ~ 55,-0 73(-0 88 to -0 58)

Iceland,Prevalence,55 ~ 60,-0 74(-0 87 to -0 61)

Iceland,Prevalence,60 ~ 65,-0 74(-0 85 to -0 62)

Iceland,Prevalence,65 ~ 70,-0 71(-0 82 to -0 6)

Iceland,Prevalence,70 ~ 75,-0 65(-0 75 to -0 54)

Iceland,Prevalence,75 ~ 80,-0 54(-0 65 to -0 43)

Iceland,Prevalence,80 ~ 85,-0 41(-0 53 to -0 29)

Iceland,Prevalence,85 ~ 90,-0 28(-0 43 to -0 12)

Iceland,Prevalence,90 ~ 95,-0 18(-0 44 to 0 07)

Iceland,Prevalence,20 ~ 25,-0 05(-0 57 to 0 47)

Iceland,Prevalence,25 ~ 30,-0 14(-0 5 to 0 22)

Iceland,Prevalence,30 ~ 35,-0 27(-0 56 to 0 02)

Iceland,Prevalence,35 ~ 40,-0 4(-0 65 to -0 15)

Iceland,Prevalence,40 ~ 45,-0 55(-0 76 to -0 33)

Iceland,Prevalence,45 ~ 50,-0 67(-0 85 to -0 49)

Iceland,Prevalence,50 ~ 55,-0 73(-0 88 to -0 58)

Iceland,Prevalence,55 ~ 60,-0 74(-0 87 to -0 61)

Iceland,Prevalence,60 ~ 65,-0 74(-0 85 to -0 62)

Iceland,Prevalence,65 ~ 70,-0 71(-0 82 to -0 6)

Iceland,Prevalence,70 ~ 75,-0 65(-0 75 to -0 54)

Iceland,Prevalence,75 ~ 80,-0 54(-0 65 to -0 43)

Iceland,Prevalence,80 ~ 85,-0 41(-0 53 to -0 29)

Iceland,Prevalence,85 ~ 90,-0 28(-0 43 to -0 12)

Iceland,Prevalence,90 ~ 95,-0 18(-0 44 to 0 07)

Iceland,Deaths,20 ~ 25,0 53(-27 07 to 38 59)

Iceland,Deaths,25 ~ 30,1 05(-18 61 to 25 46)

Iceland,Deaths,30 ~ 35,0 73(-15 29 to 19 77)

Iceland,Deaths,35 ~ 40,0 52(-11 76 to 14 52)

Iceland,Deaths,40 ~ 45,-0 02(-8 91 to 9 74)

Iceland,Deaths,45 ~ 50,-1 08(-7 69 to 6 01)

Iceland,Deaths,50 ~ 55,-1 43(-6 08 to 3 45)

Iceland,Deaths,55 ~ 60,-1 44(-4 57 to 1 78)

Iceland,Deaths,60 ~ 65,-1 41(-3 66 to 0 89)

Iceland,Deaths,65 ~ 70,-1 29(-3 02 to 0 47)

Iceland,Deaths,70 ~ 75,-1 07(-2 46 to 0 33)

Iceland,Deaths,75 ~ 80,-0 67(-1 86 to 0 53)

Iceland,Deaths,80 ~ 85,-0 34(-1 49 to 0 83)

Iceland,Deaths,85 ~ 90,0 15(-1 17 to 1 49)

Iceland,Deaths,90 ~ 95,0 69(-1 42 to 2 84)

Iceland,Deaths,20 ~ 25,0 53(-27 07 to 38 59)

Iceland,Deaths,25 ~ 30,1 05(-18 61 to 25 46)

Iceland,Deaths,30 ~ 35,0 73(-15 29 to 19 77)

Iceland,Deaths,35 ~ 40,0 52(-11 76 to 14 52)

Iceland,Deaths,40 ~ 45,-0 02(-8 91 to 9 74)

Iceland,Deaths,45 ~ 50,-1 08(-7 69 to 6 01)

Iceland,Deaths,50 ~ 55,-1 43(-6 08 to 3 45)

Iceland,Deaths,55 ~ 60,-1 44(-4 57 to 1 78)

Iceland,Deaths,60 ~ 65,-1 41(-3 66 to 0 89)

Iceland,Deaths,65 ~ 70,-1 29(-3 02 to 0 47)

Iceland,Deaths,70 ~ 75,-1 07(-2 46 to 0 33)

Iceland,Deaths,75 ~ 80,-0 67(-1 86 to 0 53)

Iceland,Deaths,80 ~ 85,-0 34(-1 49 to 0 83)

Iceland,Deaths,85 ~ 90,0 15(-1 17 to 1 49)

Iceland,Deaths,90 ~ 95,0 69(-1 42 to 2 84)

Iceland,Deaths,20 ~ 25,0 53(-27 07 to 38 59)

Iceland,Deaths,25 ~ 30,1 05(-18 61 to 25 46)

Iceland,Deaths,30 ~ 35,0 73(-15 29 to 19 77)

Iceland,Deaths,35 ~ 40,0 52(-11 76 to 14 52)

Iceland,Deaths,40 ~ 45,-0 02(-8 91 to 9 74)

Iceland,Deaths,45 ~ 50,-1 08(-7 69 to 6 01)

Iceland,Deaths,50 ~ 55,-1 43(-6 08 to 3 45)

Iceland,Deaths,55 ~ 60,-1 44(-4 57 to 1 78)

Iceland,Deaths,60 ~ 65,-1 41(-3 66 to 0 89)

Iceland,Deaths,65 ~ 70,-1 29(-3 02 to 0 47)

Iceland,Deaths,70 ~ 75,-1 07(-2 46 to 0 33)

Iceland,Deaths,75 ~ 80,-0 67(-1 86 to 0 53)

Iceland,Deaths,80 ~ 85,-0 34(-1 49 to 0 83)

Iceland,Deaths,85 ~ 90,0 15(-1 17 to 1 49)

Iceland,Deaths,90 ~ 95,0 69(-1 42 to 2 84)

Iceland,Deaths,20 ~ 25,0 53(-27 07 to 38 59)

Iceland,Deaths,25 ~ 30,1 05(-18 61 to 25 46)

Iceland,Deaths,30 ~ 35,0 73(-15 29 to 19 77)

Iceland,Deaths,35 ~ 40,0 52(-11 76 to 14 52)

Iceland,Deaths,40 ~ 45,-0 02(-8 91 to 9 74)

Iceland,Deaths,45 ~ 50,-1 08(-7 69 to 6 01)

Iceland,Deaths,50 ~ 55,-1 43(-6 08 to 3 45)

Iceland,Deaths,55 ~ 60,-1 44(-4 57 to 1 78)

Iceland,Deaths,60 ~ 65,-1 41(-3 66 to 0 89)

Iceland,Deaths,65 ~ 70,-1 29(-3 02 to 0 47)

Iceland,Deaths,70 ~ 75,-1 07(-2 46 to 0 33)

Iceland,Deaths,75 ~ 80,-0 67(-1 86 to 0 53)

Iceland,Deaths,80 ~ 85,-0 34(-1 49 to 0 83)

Iceland,Deaths,85 ~ 90,0 15(-1 17 to 1 49)

Iceland,Deaths,90 ~ 95,0 69(-1 42 to 2 84)

Iceland,DALYs,20 ~ 25,0 12(-1 88 to 2 16)

Iceland,DALYs,25 ~ 30,0 17(-1 21 to 1 57)

Iceland,DALYs,30 ~ 35,0(-1 14 to 1 14)

Iceland,DALYs,35 ~ 40,-0 15(-1 09 to 0 8)

Iceland,DALYs,40 ~ 45,-0 43(-1 2 to 0 35)

Iceland,DALYs,45 ~ 50,-0 94(-1 57 to -0 31)

Iceland,DALYs,50 ~ 55,-1 16(-1 66 to -0 66)

Iceland,DALYs,55 ~ 60,-1 23(-1 62 to -0 83)

Iceland,DALYs,60 ~ 65,-1 22(-1 55 to -0 9)

Iceland,DALYs,65 ~ 70,-1 14(-1 42 to -0 86)

Iceland,DALYs,70 ~ 75,-0 99(-1 25 to -0 74)

Iceland,DALYs,75 ~ 80,-0 68(-0 93 to -0 43)

Iceland,DALYs,80 ~ 85,-0 39(-0 67 to -0 11)

Iceland,DALYs,85 ~ 90,-0 01(-0 37 to 0 35)

Iceland,DALYs,90 ~ 95,0 4(-0 19 to 1)

Iceland,DALYs,20 ~ 25,0 12(-1 88 to 2 16)

Iceland,DALYs,25 ~ 30,0 17(-1 21 to 1 57)

Iceland,DALYs,30 ~ 35,0(-1 14 to 1 14)

Iceland,DALYs,35 ~ 40,-0 15(-1 09 to 0 8)

Iceland,DALYs,40 ~ 45,-0 43(-1 2 to 0 35)

Iceland,DALYs,45 ~ 50,-0 94(-1 57 to -0 31)

Iceland,DALYs,50 ~ 55,-1 16(-1 66 to -0 66)

Iceland,DALYs,55 ~ 60,-1 23(-1 62 to -0 83)

Iceland,DALYs,60 ~ 65,-1 22(-1 55 to -0 9)

Iceland,DALYs,65 ~ 70,-1 14(-1 42 to -0 86)

Iceland,DALYs,70 ~ 75,-0 99(-1 25 to -0 74)

Iceland,DALYs,75 ~ 80,-0 68(-0 93 to -0 43)

Iceland,DALYs,80 ~ 85,-0 39(-0 67 to -0 11)

Iceland,DALYs,85 ~ 90,-0 01(-0 37 to 0 35)

Iceland,DALYs,90 ~ 95,0 4(-0 19 to 1)

Iceland,DALYs,20 ~ 25,0 12(-1 88 to 2 16)

Iceland,DALYs,25 ~ 30,0 17(-1 21 to 1 57)

Iceland,DALYs,30 ~ 35,0(-1 14 to 1 14)

Iceland,DALYs,35 ~ 40,-0 15(-1 09 to 0 8)

Iceland,DALYs,40 ~ 45,-0 43(-1 2 to 0 35)

Iceland,DALYs,45 ~ 50,-0 94(-1 57 to -0 31)

Iceland,DALYs,50 ~ 55,-1 16(-1 66 to -0 66)

Iceland,DALYs,55 ~ 60,-1 23(-1 62 to -0 83)

Iceland,DALYs,60 ~ 65,-1 22(-1 55 to -0 9)

Iceland,DALYs,65 ~ 70,-1 14(-1 42 to -0 86)

Iceland,DALYs,70 ~ 75,-0 99(-1 25 to -0 74)

Iceland,DALYs,75 ~ 80,-0 68(-0 93 to -0 43)

Iceland,DALYs,80 ~ 85,-0 39(-0 67 to -0 11)

Iceland,DALYs,85 ~ 90,-0 01(-0 37 to 0 35)

Iceland,DALYs,90 ~ 95,0 4(-0 19 to 1)

Iceland,DALYs,20 ~ 25,0 12(-1 88 to 2 16)

Iceland,DALYs,25 ~ 30,0 17(-1 21 to 1 57)

Iceland,DALYs,30 ~ 35,0(-1 14 to 1 14)

Iceland,DALYs,35 ~ 40,-0 15(-1 09 to 0 8)

Iceland,DALYs,40 ~ 45,-0 43(-1 2 to 0 35)

Iceland,DALYs,45 ~ 50,-0 94(-1 57 to -0 31)

Iceland,DALYs,50 ~ 55,-1 16(-1 66 to -0 66)

Iceland,DALYs,55 ~ 60,-1 23(-1 62 to -0 83)

Iceland,DALYs,60 ~ 65,-1 22(-1 55 to -0 9)

Iceland,DALYs,65 ~ 70,-1 14(-1 42 to -0 86)

Iceland,DALYs,70 ~ 75,-0 99(-1 25 to -0 74)

Iceland,DALYs,75 ~ 80,-0 68(-0 93 to -0 43)

Iceland,DALYs,80 ~ 85,-0 39(-0 67 to -0 11)

Iceland,DALYs,85 ~ 90,-0 01(-0 37 to 0 35)

Iceland,DALYs,90 ~ 95,0 4(-0 19 to 1)

Israel,Prevalence,20 ~ 25,-0 18(-0 29 to -0 07)

Israel,Prevalence,25 ~ 30,-0 19(-0 27 to -0 1)

Israel,Prevalence,30 ~ 35,-0 22(-0 28 to -0 15)

Israel,Prevalence,35 ~ 40,-0 27(-0 33 to -0 21)

Israel,Prevalence,40 ~ 45,-0 35(-0 4 to -0 3)

Israel,Prevalence,45 ~ 50,-0 43(-0 47 to -0 39)

Israel,Prevalence,50 ~ 55,-0 48(-0 52 to -0 44)

Israel,Prevalence,55 ~ 60,-0 49(-0 53 to -0 46)

Israel,Prevalence,60 ~ 65,-0 48(-0 51 to -0 45)

Israel,Prevalence,65 ~ 70,-0 44(-0 47 to -0 41)

Israel,Prevalence,70 ~ 75,-0 4(-0 43 to -0 37)

Israel,Prevalence,75 ~ 80,-0 34(-0 37 to -0 31)

Israel,Prevalence,80 ~ 85,-0 27(-0 3 to -0 23)

Israel,Prevalence,85 ~ 90,-0 16(-0 21 to -0 12)

Israel,Prevalence,90 ~ 95,-0 02(-0 11 to 0 07)

Israel,Prevalence,20 ~ 25,-0 18(-0 29 to -0 07)

Israel,Prevalence,25 ~ 30,-0 19(-0 27 to -0 1)

Israel,Prevalence,30 ~ 35,-0 22(-0 28 to -0 15)

Israel,Prevalence,35 ~ 40,-0 27(-0 33 to -0 21)

Israel,Prevalence,40 ~ 45,-0 35(-0 4 to -0 3)

Israel,Prevalence,45 ~ 50,-0 43(-0 47 to -0 39)

Israel,Prevalence,50 ~ 55,-0 48(-0 52 to -0 44)

Israel,Prevalence,55 ~ 60,-0 49(-0 53 to -0 46)

Israel,Prevalence,60 ~ 65,-0 48(-0 51 to -0 45)

Israel,Prevalence,65 ~ 70,-0 44(-0 47 to -0 41)

Israel,Prevalence,70 ~ 75,-0 4(-0 43 to -0 37)

Israel,Prevalence,75 ~ 80,-0 34(-0 37 to -0 31)

Israel,Prevalence,80 ~ 85,-0 27(-0 3 to -0 23)

Israel,Prevalence,85 ~ 90,-0 16(-0 21 to -0 12)

Israel,Prevalence,90 ~ 95,-0 02(-0 11 to 0 07)

Israel,Prevalence,20 ~ 25,-0 18(-0 29 to -0 07)

Israel,Prevalence,25 ~ 30,-0 19(-0 27 to -0 1)

Israel,Prevalence,30 ~ 35,-0 22(-0 28 to -0 15)

Israel,Prevalence,35 ~ 40,-0 27(-0 33 to -0 21)

Israel,Prevalence,40 ~ 45,-0 35(-0 4 to -0 3)

Israel,Prevalence,45 ~ 50,-0 43(-0 47 to -0 39)

Israel,Prevalence,50 ~ 55,-0 48(-0 52 to -0 44)

Israel,Prevalence,55 ~ 60,-0 49(-0 53 to -0 46)

Israel,Prevalence,60 ~ 65,-0 48(-0 51 to -0 45)

Israel,Prevalence,65 ~ 70,-0 44(-0 47 to -0 41)

Israel,Prevalence,70 ~ 75,-0 4(-0 43 to -0 37)

Israel,Prevalence,75 ~ 80,-0 34(-0 37 to -0 31)

Israel,Prevalence,80 ~ 85,-0 27(-0 3 to -0 23)

Israel,Prevalence,85 ~ 90,-0 16(-0 21 to -0 12)

Israel,Prevalence,90 ~ 95,-0 02(-0 11 to 0 07)

Israel,Prevalence,20 ~ 25,-0 18(-0 29 to -0 07)

Israel,Prevalence,25 ~ 30,-0 19(-0 27 to -0 1)

Israel,Prevalence,30 ~ 35,-0 22(-0 28 to -0 15)

Israel,Prevalence,35 ~ 40,-0 27(-0 33 to -0 21)

Israel,Prevalence,40 ~ 45,-0 35(-0 4 to -0 3)

Israel,Prevalence,45 ~ 50,-0 43(-0 47 to -0 39)

Israel,Prevalence,50 ~ 55,-0 48(-0 52 to -0 44)

Israel,Prevalence,55 ~ 60,-0 49(-0 53 to -0 46)

Israel,Prevalence,60 ~ 65,-0 48(-0 51 to -0 45)

Israel,Prevalence,65 ~ 70,-0 44(-0 47 to -0 41)

Israel,Prevalence,70 ~ 75,-0 4(-0 43 to -0 37)

Israel,Prevalence,75 ~ 80,-0 34(-0 37 to -0 31)

Israel,Prevalence,80 ~ 85,-0 27(-0 3 to -0 23)

Israel,Prevalence,85 ~ 90,-0 16(-0 21 to -0 12)

Israel,Prevalence,90 ~ 95,-0 02(-0 11 to 0 07)

Israel,Deaths,20 ~ 25,-0 56(-8 93 to 8 58)

Israel,Deaths,25 ~ 30,-0 96(-7 16 to 5 65)

Israel,Deaths,30 ~ 35,-1 28(-5 94 to 3 6)

Israel,Deaths,35 ~ 40,-1 42(-4 86 to 2 14)

Israel,Deaths,40 ~ 45,-1 35(-3 75 to 1 11)

Israel,Deaths,45 ~ 50,-1 07(-2 69 to 0 57)

Israel,Deaths,50 ~ 55,-0 87(-1 93 to 0 21)

Israel,Deaths,55 ~ 60,-0 78(-1 51 to -0 06)

Israel,Deaths,60 ~ 65,-1 12(-1 64 to -0 6)

Israel,Deaths,65 ~ 70,-1 56(-1 96 to -1 15)

Israel,Deaths,70 ~ 75,-1 73(-2 07 to -1 39)

Israel,Deaths,75 ~ 80,-1 9(-2 2 to -1 59)

Israel,Deaths,80 ~ 85,-1 84(-2 12 to -1 56)

Israel,Deaths,85 ~ 90,-2 04(-2 37 to -1 71)

Israel,Deaths,90 ~ 95,-2 12(-2 69 to -1 55)

Israel,Deaths,20 ~ 25,-0 56(-8 93 to 8 58)

Israel,Deaths,25 ~ 30,-0 96(-7 16 to 5 65)

Israel,Deaths,30 ~ 35,-1 28(-5 94 to 3 6)

Israel,Deaths,35 ~ 40,-1 42(-4 86 to 2 14)

Israel,Deaths,40 ~ 45,-1 35(-3 75 to 1 11)

Israel,Deaths,45 ~ 50,-1 07(-2 69 to 0 57)

Israel,Deaths,50 ~ 55,-0 87(-1 93 to 0 21)

Israel,Deaths,55 ~ 60,-0 78(-1 51 to -0 06)

Israel,Deaths,60 ~ 65,-1 12(-1 64 to -0 6)

Israel,Deaths,65 ~ 70,-1 56(-1 96 to -1 15)

Israel,Deaths,70 ~ 75,-1 73(-2 07 to -1 39)

Israel,Deaths,75 ~ 80,-1 9(-2 2 to -1 59)

Israel,Deaths,80 ~ 85,-1 84(-2 12 to -1 56)

Israel,Deaths,85 ~ 90,-2 04(-2 37 to -1 71)

Israel,Deaths,90 ~ 95,-2 12(-2 69 to -1 55)

Israel,Deaths,20 ~ 25,-0 56(-8 93 to 8 58)

Israel,Deaths,25 ~ 30,-0 96(-7 16 to 5 65)

Israel,Deaths,30 ~ 35,-1 28(-5 94 to 3 6)

Israel,Deaths,35 ~ 40,-1 42(-4 86 to 2 14)

Israel,Deaths,40 ~ 45,-1 35(-3 75 to 1 11)

Israel,Deaths,45 ~ 50,-1 07(-2 69 to 0 57)

Israel,Deaths,50 ~ 55,-0 87(-1 93 to 0 21)

Israel,Deaths,55 ~ 60,-0 78(-1 51 to -0 06)

Israel,Deaths,60 ~ 65,-1 12(-1 64 to -0 6)

Israel,Deaths,65 ~ 70,-1 56(-1 96 to -1 15)

Israel,Deaths,70 ~ 75,-1 73(-2 07 to -1 39)

Israel,Deaths,75 ~ 80,-1 9(-2 2 to -1 59)

Israel,Deaths,80 ~ 85,-1 84(-2 12 to -1 56)

Israel,Deaths,85 ~ 90,-2 04(-2 37 to -1 71)

Israel,Deaths,90 ~ 95,-2 12(-2 69 to -1 55)

Israel,Deaths,20 ~ 25,-0 56(-8 93 to 8 58)

Israel,Deaths,25 ~ 30,-0 96(-7 16 to 5 65)

Israel,Deaths,30 ~ 35,-1 28(-5 94 to 3 6)

Israel,Deaths,35 ~ 40,-1 42(-4 86 to 2 14)

Israel,Deaths,40 ~ 45,-1 35(-3 75 to 1 11)

Israel,Deaths,45 ~ 50,-1 07(-2 69 to 0 57)

Israel,Deaths,50 ~ 55,-0 87(-1 93 to 0 21)

Israel,Deaths,55 ~ 60,-0 78(-1 51 to -0 06)

Israel,Deaths,60 ~ 65,-1 12(-1 64 to -0 6)

Israel,Deaths,65 ~ 70,-1 56(-1 96 to -1 15)

Israel,Deaths,70 ~ 75,-1 73(-2 07 to -1 39)

Israel,Deaths,75 ~ 80,-1 9(-2 2 to -1 59)

Israel,Deaths,80 ~ 85,-1 84(-2 12 to -1 56)

Israel,Deaths,85 ~ 90,-2 04(-2 37 to -1 71)

Israel,Deaths,90 ~ 95,-2 12(-2 69 to -1 55)

Israel,DALYs,20 ~ 25,-0 15(-0 84 to 0 55)

Israel,DALYs,25 ~ 30,-0 32(-0 84 to 0 19)

Israel,DALYs,30 ~ 35,-0 53(-0 94 to -0 11)

Israel,DALYs,35 ~ 40,-0 61(-0 96 to -0 27)

Israel,DALYs,40 ~ 45,-0 6(-0 89 to -0 32)

Israel,DALYs,45 ~ 50,-0 54(-0 77 to -0 31)

Israel,DALYs,50 ~ 55,-0 6(-0 79 to -0 42)

Israel,DALYs,55 ~ 60,-0 67(-0 82 to -0 52)

Israel,DALYs,60 ~ 65,-0 95(-1 07 to -0 83)

Israel,DALYs,65 ~ 70,-1 25(-1 35 to -1 14)

Israel,DALYs,70 ~ 75,-1 39(-1 48 to -1 29)

Israel,DALYs,75 ~ 80,-1 53(-1 63 to -1 43)

Israel,DALYs,80 ~ 85,-1 49(-1 6 to -1 39)

Israel,DALYs,85 ~ 90,-1 65(-1 79 to -1 52)

Israel,DALYs,90 ~ 95,-1 73(-1 98 to -1 48)

Israel,DALYs,20 ~ 25,-0 15(-0 84 to 0 55)

Israel,DALYs,25 ~ 30,-0 32(-0 84 to 0 19)

Israel,DALYs,30 ~ 35,-0 53(-0 94 to -0 11)

Israel,DALYs,35 ~ 40,-0 61(-0 96 to -0 27)

Israel,DALYs,40 ~ 45,-0 6(-0 89 to -0 32)

Israel,DALYs,45 ~ 50,-0 54(-0 77 to -0 31)

Israel,DALYs,50 ~ 55,-0 6(-0 79 to -0 42)

Israel,DALYs,55 ~ 60,-0 67(-0 82 to -0 52)

Israel,DALYs,60 ~ 65,-0 95(-1 07 to -0 83)

Israel,DALYs,65 ~ 70,-1 25(-1 35 to -1 14)

Israel,DALYs,70 ~ 75,-1 39(-1 48 to -1 29)

Israel,DALYs,75 ~ 80,-1 53(-1 63 to -1 43)

Israel,DALYs,80 ~ 85,-1 49(-1 6 to -1 39)

Israel,DALYs,85 ~ 90,-1 65(-1 79 to -1 52)

Israel,DALYs,90 ~ 95,-1 73(-1 98 to -1 48)

Israel,DALYs,20 ~ 25,-0 15(-0 84 to 0 55)

Israel,DALYs,25 ~ 30,-0 32(-0 84 to 0 19)

Israel,DALYs,30 ~ 35,-0 53(-0 94 to -0 11)

Israel,DALYs,35 ~ 40,-0 61(-0 96 to -0 27)

Israel,DALYs,40 ~ 45,-0 6(-0 89 to -0 32)

Israel,DALYs,45 ~ 50,-0 54(-0 77 to -0 31)

Israel,DALYs,50 ~ 55,-0 6(-0 79 to -0 42)

Israel,DALYs,55 ~ 60,-0 67(-0 82 to -0 52)

Israel,DALYs,60 ~ 65,-0 95(-1 07 to -0 83)

Israel,DALYs,65 ~ 70,-1 25(-1 35 to -1 14)

Israel,DALYs,70 ~ 75,-1 39(-1 48 to -1 29)

Israel,DALYs,75 ~ 80,-1 53(-1 63 to -1 43)

Israel,DALYs,80 ~ 85,-1 49(-1 6 to -1 39)

Israel,DALYs,85 ~ 90,-1 65(-1 79 to -1 52)

Israel,DALYs,90 ~ 95,-1 73(-1 98 to -1 48)

Israel,DALYs,20 ~ 25,-0 15(-0 84 to 0 55)

Israel,DALYs,25 ~ 30,-0 32(-0 84 to 0 19)

Israel,DALYs,30 ~ 35,-0 53(-0 94 to -0 11)

Israel,DALYs,35 ~ 40,-0 61(-0 96 to -0 27)

Israel,DALYs,40 ~ 45,-0 6(-0 89 to -0 32)

Israel,DALYs,45 ~ 50,-0 54(-0 77 to -0 31)

Israel,DALYs,50 ~ 55,-0 6(-0 79 to -0 42)

Israel,DALYs,55 ~ 60,-0 67(-0 82 to -0 52)

Israel,DALYs,60 ~ 65,-0 95(-1 07 to -0 83)

Israel,DALYs,65 ~ 70,-1 25(-1 35 to -1 14)

Israel,DALYs,70 ~ 75,-1 39(-1 48 to -1 29)

Israel,DALYs,75 ~ 80,-1 53(-1 63 to -1 43)

Israel,DALYs,80 ~ 85,-1 49(-1 6 to -1 39)

Israel,DALYs,85 ~ 90,-1 65(-1 79 to -1 52)

Israel,DALYs,90 ~ 95,-1 73(-1 98 to -1 48)

Sao_Tome_and_Principe,Prevalence,20 ~ 25,0 05(-0 57 to 0 67)

Sao_Tome_and_Principe,Prevalence,25 ~ 30,0 06(-0 42 to 0 55)

Sao_Tome_and_Principe,Prevalence,30 ~ 35,0 09(-0 34 to 0 51)

Sao_Tome_and_Principe,Prevalence,35 ~ 40,0 12(-0 27 to 0 52)

Sao_Tome_and_Principe,Prevalence,40 ~ 45,0 17(-0 21 to 0 54)

Sao_Tome_and_Principe,Prevalence,45 ~ 50,0 22(-0 13 to 0 58)

Sao_Tome_and_Principe,Prevalence,50 ~ 55,0 29(-0 04 to 0 62)

Sao_Tome_and_Principe,Prevalence,55 ~ 60,0 36(0 05 to 0 67)

Sao_Tome_and_Principe,Prevalence,60 ~ 65,0 44(0 13 to 0 74)

Sao_Tome_and_Principe,Prevalence,65 ~ 70,0 51(0 2 to 0 83)

Sao_Tome_and_Principe,Prevalence,70 ~ 75,0 57(0 23 to 0 92)

Sao_Tome_and_Principe,Prevalence,75 ~ 80,0 62(0 21 to 1 02)

Sao_Tome_and_Principe,Prevalence,80 ~ 85,0 72(0 21 to 1 23)

Sao_Tome_and_Principe,Prevalence,85 ~ 90,0 99(0 24 to 1 75)

Sao_Tome_and_Principe,Prevalence,90 ~ 95,1 52(-0 02 to 3 09)

Sao_Tome_and_Principe,Prevalence,20 ~ 25,0 05(-0 57 to 0 67)

Sao_Tome_and_Principe,Prevalence,25 ~ 30,0 06(-0 42 to 0 55)

Sao_Tome_and_Principe,Prevalence,30 ~ 35,0 09(-0 34 to 0 51)

Sao_Tome_and_Principe,Prevalence,35 ~ 40,0 12(-0 27 to 0 52)

Sao_Tome_and_Principe,Prevalence,40 ~ 45,0 17(-0 21 to 0 54)

Sao_Tome_and_Principe,Prevalence,45 ~ 50,0 22(-0 13 to 0 58)

Sao_Tome_and_Principe,Prevalence,50 ~ 55,0 29(-0 04 to 0 62)

Sao_Tome_and_Principe,Prevalence,55 ~ 60,0 36(0 05 to 0 67)

Sao_Tome_and_Principe,Prevalence,60 ~ 65,0 44(0 13 to 0 74)

Sao_Tome_and_Principe,Prevalence,65 ~ 70,0 51(0 2 to 0 83)

Sao_Tome_and_Principe,Prevalence,70 ~ 75,0 57(0 23 to 0 92)

Sao_Tome_and_Principe,Prevalence,75 ~ 80,0 62(0 21 to 1 02)

Sao_Tome_and_Principe,Prevalence,80 ~ 85,0 72(0 21 to 1 23)

Sao_Tome_and_Principe,Prevalence,85 ~ 90,0 99(0 24 to 1 75)

Sao_Tome_and_Principe,Prevalence,90 ~ 95,1 52(-0 02 to 3 09)

Sao_Tome_and_Principe,Prevalence,20 ~ 25,0 05(-0 57 to 0 67)

Sao_Tome_and_Principe,Prevalence,25 ~ 30,0 06(-0 42 to 0 55)

Sao_Tome_and_Principe,Prevalence,30 ~ 35,0 09(-0 34 to 0 51)

Sao_Tome_and_Principe,Prevalence,35 ~ 40,0 12(-0 27 to 0 52)

Sao_Tome_and_Principe,Prevalence,40 ~ 45,0 17(-0 21 to 0 54)

Sao_Tome_and_Principe,Prevalence,45 ~ 50,0 22(-0 13 to 0 58)

Sao_Tome_and_Principe,Prevalence,50 ~ 55,0 29(-0 04 to 0 62)

Sao_Tome_and_Principe,Prevalence,55 ~ 60,0 36(0 05 to 0 67)

Sao_Tome_and_Principe,Prevalence,60 ~ 65,0 44(0 13 to 0 74)

Sao_Tome_and_Principe,Prevalence,65 ~ 70,0 51(0 2 to 0 83)

Sao_Tome_and_Principe,Prevalence,70 ~ 75,0 57(0 23 to 0 92)

Sao_Tome_and_Principe,Prevalence,75 ~ 80,0 62(0 21 to 1 02)

Sao_Tome_and_Principe,Prevalence,80 ~ 85,0 72(0 21 to 1 23)

Sao_Tome_and_Principe,Prevalence,85 ~ 90,0 99(0 24 to 1 75)

Sao_Tome_and_Principe,Prevalence,90 ~ 95,1 52(-0 02 to 3 09)

Sao_Tome_and_Principe,Prevalence,20 ~ 25,0 05(-0 57 to 0 67)

Sao_Tome_and_Principe,Prevalence,25 ~ 30,0 06(-0 42 to 0 55)

Sao_Tome_and_Principe,Prevalence,30 ~ 35,0 09(-0 34 to 0 51)

Sao_Tome_and_Principe,Prevalence,35 ~ 40,0 12(-0 27 to 0 52)

Sao_Tome_and_Principe,Prevalence,40 ~ 45,0 17(-0 21 to 0 54)

Sao_Tome_and_Principe,Prevalence,45 ~ 50,0 22(-0 13 to 0 58)

Sao_Tome_and_Principe,Prevalence,50 ~ 55,0 29(-0 04 to 0 62)

Sao_Tome_and_Principe,Prevalence,55 ~ 60,0 36(0 05 to 0 67)

Sao_Tome_and_Principe,Prevalence,60 ~ 65,0 44(0 13 to 0 74)

Sao_Tome_and_Principe,Prevalence,65 ~ 70,0 51(0 2 to 0 83)

Sao_Tome_and_Principe,Prevalence,70 ~ 75,0 57(0 23 to 0 92)

Sao_Tome_and_Principe,Prevalence,75 ~ 80,0 62(0 21 to 1 02)

Sao_Tome_and_Principe,Prevalence,80 ~ 85,0 72(0 21 to 1 23)

Sao_Tome_and_Principe,Prevalence,85 ~ 90,0 99(0 24 to 1 75)

Sao_Tome_and_Principe,Prevalence,90 ~ 95,1 52(-0 02 to 3 09)

Sao_Tome_and_Principe,Deaths,20 ~ 25,-1 41(-16 23 to 16 04)

Sao_Tome_and_Principe,Deaths,25 ~ 30,-1 53(-10 52 to 8 37)

Sao_Tome_and_Principe,Deaths,30 ~ 35,-1 41(-8 6 to 6 35)

Sao_Tome_and_Principe,Deaths,35 ~ 40,-1 08(-7 22 to 5 46)

Sao_Tome_and_Principe,Deaths,40 ~ 45,-0 78(-5 88 to 4 59)

Sao_Tome_and_Principe,Deaths,45 ~ 50,-0 63(-4 67 to 3 59)

Sao_Tome_and_Principe,Deaths,50 ~ 55,-0 62(-3 88 to 2 75)

Sao_Tome_and_Principe,Deaths,55 ~ 60,-0 61(-3 25 to 2 1)

Sao_Tome_and_Principe,Deaths,60 ~ 65,-0 63(-2 76 to 1 55)

Sao_Tome_and_Principe,Deaths,65 ~ 70,-0 58(-2 37 to 1 25)

Sao_Tome_and_Principe,Deaths,70 ~ 75,-0 39(-2 04 to 1 3)

Sao_Tome_and_Principe,Deaths,75 ~ 80,-0 25(-1 92 to 1 45)

Sao_Tome_and_Principe,Deaths,80 ~ 85,-0 07(-1 96 to 1 85)

Sao_Tome_and_Principe,Deaths,85 ~ 90,-0 08(-2 5 to 2 41)

Sao_Tome_and_Principe,Deaths,90 ~ 95,0 16(-4 21 to 4 73)

Sao_Tome_and_Principe,Deaths,20 ~ 25,-1 41(-16 23 to 16 04)

Sao_Tome_and_Principe,Deaths,25 ~ 30,-1 53(-10 52 to 8 37)

Sao_Tome_and_Principe,Deaths,30 ~ 35,-1 41(-8 6 to 6 35)

Sao_Tome_and_Principe,Deaths,35 ~ 40,-1 08(-7 22 to 5 46)

Sao_Tome_and_Principe,Deaths,40 ~ 45,-0 78(-5 88 to 4 59)

Sao_Tome_and_Principe,Deaths,45 ~ 50,-0 63(-4 67 to 3 59)

Sao_Tome_and_Principe,Deaths,50 ~ 55,-0 62(-3 88 to 2 75)

Sao_Tome_and_Principe,Deaths,55 ~ 60,-0 61(-3 25 to 2 1)

Sao_Tome_and_Principe,Deaths,60 ~ 65,-0 63(-2 76 to 1 55)

Sao_Tome_and_Principe,Deaths,65 ~ 70,-0 58(-2 37 to 1 25)

Sao_Tome_and_Principe,Deaths,70 ~ 75,-0 39(-2 04 to 1 3)

Sao_Tome_and_Principe,Deaths,75 ~ 80,-0 25(-1 92 to 1 45)

Sao_Tome_and_Principe,Deaths,80 ~ 85,-0 07(-1 96 to 1 85)

Sao_Tome_and_Principe,Deaths,85 ~ 90,-0 08(-2 5 to 2 41)

Sao_Tome_and_Principe,Deaths,90 ~ 95,0 16(-4 21 to 4 73)

Sao_Tome_and_Principe,Deaths,20 ~ 25,-1 41(-16 23 to 16 04)

Sao_Tome_and_Principe,Deaths,25 ~ 30,-1 53(-10 52 to 8 37)

Sao_Tome_and_Principe,Deaths,30 ~ 35,-1 41(-8 6 to 6 35)

Sao_Tome_and_Principe,Deaths,35 ~ 40,-1 08(-7 22 to 5 46)

Sao_Tome_and_Principe,Deaths,40 ~ 45,-0 78(-5 88 to 4 59)

Sao_Tome_and_Principe,Deaths,45 ~ 50,-0 63(-4 67 to 3 59)

Sao_Tome_and_Principe,Deaths,50 ~ 55,-0 62(-3 88 to 2 75)

Sao_Tome_and_Principe,Deaths,55 ~ 60,-0 61(-3 25 to 2 1)

Sao_Tome_and_Principe,Deaths,60 ~ 65,-0 63(-2 76 to 1 55)

Sao_Tome_and_Principe,Deaths,65 ~ 70,-0 58(-2 37 to 1 25)

Sao_Tome_and_Principe,Deaths,70 ~ 75,-0 39(-2 04 to 1 3)

Sao_Tome_and_Principe,Deaths,75 ~ 80,-0 25(-1 92 to 1 45)

Sao_Tome_and_Principe,Deaths,80 ~ 85,-0 07(-1 96 to 1 85)

Sao_Tome_and_Principe,Deaths,85 ~ 90,-0 08(-2 5 to 2 41)

Sao_Tome_and_Principe,Deaths,90 ~ 95,0 16(-4 21 to 4 73)

Sao_Tome_and_Principe,Deaths,20 ~ 25,-1 41(-16 23 to 16 04)

Sao_Tome_and_Principe,Deaths,25 ~ 30,-1 53(-10 52 to 8 37)

Sao_Tome_and_Principe,Deaths,30 ~ 35,-1 41(-8 6 to 6 35)

Sao_Tome_and_Principe,Deaths,35 ~ 40,-1 08(-7 22 to 5 46)

Sao_Tome_and_Principe,Deaths,40 ~ 45,-0 78(-5 88 to 4 59)

Sao_Tome_and_Principe,Deaths,45 ~ 50,-0 63(-4 67 to 3 59)

Sao_Tome_and_Principe,Deaths,50 ~ 55,-0 62(-3 88 to 2 75)

Sao_Tome_and_Principe,Deaths,55 ~ 60,-0 61(-3 25 to 2 1)

Sao_Tome_and_Principe,Deaths,60 ~ 65,-0 63(-2 76 to 1 55)

Sao_Tome_and_Principe,Deaths,65 ~ 70,-0 58(-2 37 to 1 25)

Sao_Tome_and_Principe,Deaths,70 ~ 75,-0 39(-2 04 to 1 3)

Sao_Tome_and_Principe,Deaths,75 ~ 80,-0 25(-1 92 to 1 45)

Sao_Tome_and_Principe,Deaths,80 ~ 85,-0 07(-1 96 to 1 85)

Sao_Tome_and_Principe,Deaths,85 ~ 90,-0 08(-2 5 to 2 41)

Sao_Tome_and_Principe,Deaths,90 ~ 95,0 16(-4 21 to 4 73)

Sao_Tome_and_Principe,DALYs,20 ~ 25,-0 67(-2 03 to 0 72)

Sao_Tome_and_Principe,DALYs,25 ~ 30,-0 83(-1 78 to 0 12)

Sao_Tome_and_Principe,DALYs,30 ~ 35,-0 8(-1 59 to 0)

Sao_Tome_and_Principe,DALYs,35 ~ 40,-0 62(-1 34 to 0 09)

Sao_Tome_and_Principe,DALYs,40 ~ 45,-0 46(-1 09 to 0 18)

Sao_Tome_and_Principe,DALYs,45 ~ 50,-0 38(-0 94 to 0 18)

Sao_Tome_and_Principe,DALYs,50 ~ 55,-0 4(-0 88 to 0 08)

Sao_Tome_and_Principe,DALYs,55 ~ 60,-0 41(-0 83 to 0 01)

Sao_Tome_and_Principe,DALYs,60 ~ 65,-0 44(-0 81 to -0 06)

Sao_Tome_and_Principe,DALYs,65 ~ 70,-0 43(-0 77 to -0 08)

Sao_Tome_and_Principe,DALYs,70 ~ 75,-0 27(-0 63 to 0 09)

Sao_Tome_and_Principe,DALYs,75 ~ 80,-0 19(-0 6 to 0 22)

Sao_Tome_and_Principe,DALYs,80 ~ 85,-0 06(-0 57 to 0 46)

Sao_Tome_and_Principe,DALYs,85 ~ 90,-0 08(-0 8 to 0 65)

Sao_Tome_and_Principe,DALYs,90 ~ 95,0 15(-1 26 to 1 58)

Sao_Tome_and_Principe,DALYs,20 ~ 25,-0 67(-2 03 to 0 72)

Sao_Tome_and_Principe,DALYs,25 ~ 30,-0 83(-1 78 to 0 12)

Sao_Tome_and_Principe,DALYs,30 ~ 35,-0 8(-1 59 to 0)

Sao_Tome_and_Principe,DALYs,35 ~ 40,-0 62(-1 34 to 0 09)

Sao_Tome_and_Principe,DALYs,40 ~ 45,-0 46(-1 09 to 0 18)

Sao_Tome_and_Principe,DALYs,45 ~ 50,-0 38(-0 94 to 0 18)

Sao_Tome_and_Principe,DALYs,50 ~ 55,-0 4(-0 88 to 0 08)

Sao_Tome_and_Principe,DALYs,55 ~ 60,-0 41(-0 83 to 0 01)

Sao_Tome_and_Principe,DALYs,60 ~ 65,-0 44(-0 81 to -0 06)

Sao_Tome_and_Principe,DALYs,65 ~ 70,-0 43(-0 77 to -0 08)

Sao_Tome_and_Principe,DALYs,70 ~ 75,-0 27(-0 63 to 0 09)

Sao_Tome_and_Principe,DALYs,75 ~ 80,-0 19(-0 6 to 0 22)

Sao_Tome_and_Principe,DALYs,80 ~ 85,-0 06(-0 57 to 0 46)

Sao_Tome_and_Principe,DALYs,85 ~ 90,-0 08(-0 8 to 0 65)

Sao_Tome_and_Principe,DALYs,90 ~ 95,0 15(-1 26 to 1 58)

Sao_Tome_and_Principe,DALYs,20 ~ 25,-0 67(-2 03 to 0 72)

Sao_Tome_and_Principe,DALYs,25 ~ 30,-0 83(-1 78 to 0 12)

Sao_Tome_and_Principe,DALYs,30 ~ 35,-0 8(-1 59 to 0)

Sao_Tome_and_Principe,DALYs,35 ~ 40,-0 62(-1 34 to 0 09)

Sao_Tome_and_Principe,DALYs,40 ~ 45,-0 46(-1 09 to 0 18)

Sao_Tome_and_Principe,DALYs,45 ~ 50,-0 38(-0 94 to 0 18)

Sao_Tome_and_Principe,DALYs,50 ~ 55,-0 4(-0 88 to 0 08)

Sao_Tome_and_Principe,DALYs,55 ~ 60,-0 41(-0 83 to 0 01)

Sao_Tome_and_Principe,DALYs,60 ~ 65,-0 44(-0 81 to -0 06)

Sao_Tome_and_Principe,DALYs,65 ~ 70,-0 43(-0 77 to -0 08)

Sao_Tome_and_Principe,DALYs,70 ~ 75,-0 27(-0 63 to 0 09)

Sao_Tome_and_Principe,DALYs,75 ~ 80,-0 19(-0 6 to 0 22)

Sao_Tome_and_Principe,DALYs,80 ~ 85,-0 06(-0 57 to 0 46)

Sao_Tome_and_Principe,DALYs,85 ~ 90,-0 08(-0 8 to 0 65)

Sao_Tome_and_Principe,DALYs,90 ~ 95,0 15(-1 26 to 1 58)

Sao_Tome_and_Principe,DALYs,20 ~ 25,-0 67(-2 03 to 0 72)

Sao_Tome_and_Principe,DALYs,25 ~ 30,-0 83(-1 78 to 0 12)

Sao_Tome_and_Principe,DALYs,30 ~ 35,-0 8(-1 59 to 0)

Sao_Tome_and_Principe,DALYs,35 ~ 40,-0 62(-1 34 to 0 09)

Sao_Tome_and_Principe,DALYs,40 ~ 45,-0 46(-1 09 to 0 18)

Sao_Tome_and_Principe,DALYs,45 ~ 50,-0 38(-0 94 to 0 18)

Sao_Tome_and_Principe,DALYs,50 ~ 55,-0 4(-0 88 to 0 08)

Sao_Tome_and_Principe,DALYs,55 ~ 60,-0 41(-0 83 to 0 01)

Sao_Tome_and_Principe,DALYs,60 ~ 65,-0 44(-0 81 to -0 06)

Sao_Tome_and_Principe,DALYs,65 ~ 70,-0 43(-0 77 to -0 08)

Sao_Tome_and_Principe,DALYs,70 ~ 75,-0 27(-0 63 to 0 09)

Sao_Tome_and_Principe,DALYs,75 ~ 80,-0 19(-0 6 to 0 22)

Sao_Tome_and_Principe,DALYs,80 ~ 85,-0 06(-0 57 to 0 46)

Sao_Tome_and_Principe,DALYs,85 ~ 90,-0 08(-0 8 to 0 65)

Sao_Tome_and_Principe,DALYs,90 ~ 95,0 15(-1 26 to 1 58)

China,Prevalence,20 ~ 25,-0 87(-1 17 to -0 57)

China,Prevalence,25 ~ 30,-0 88(-1 07 to -0 68)

China,Prevalence,30 ~ 35,-0 87(-1 01 to -0 72)

China,Prevalence,35 ~ 40,-0 87(-0 99 to -0 74)

China,Prevalence,40 ~ 45,-0 9(-1 to -0 79)

China,Prevalence,45 ~ 50,-0 94(-1 03 to -0 85)

China,Prevalence,50 ~ 55,-0 95(-1 02 to -0 87)

China,Prevalence,55 ~ 60,-0 86(-0 93 to -0 79)

China,Prevalence,60 ~ 65,-0 71(-0 77 to -0 64)

China,Prevalence,65 ~ 70,-0 52(-0 58 to -0 46)

China,Prevalence,70 ~ 75,-0 3(-0 36 to -0 23)

China,Prevalence,75 ~ 80,-0 04(-0 12 to 0 03)

China,Prevalence,80 ~ 85,0 23(0 12 to 0 33)

China,Prevalence,85 ~ 90,0 52(0 34 to 0 71)

China,Prevalence,90 ~ 95,0 89(0 43 to 1 36)

China,Prevalence,20 ~ 25,-0 87(-1 17 to -0 57)

China,Prevalence,25 ~ 30,-0 88(-1 07 to -0 68)

China,Prevalence,30 ~ 35,-0 87(-1 01 to -0 72)

China,Prevalence,35 ~ 40,-0 87(-0 99 to -0 74)

China,Prevalence,40 ~ 45,-0 9(-1 to -0 79)

China,Prevalence,45 ~ 50,-0 94(-1 03 to -0 85)

China,Prevalence,50 ~ 55,-0 95(-1 02 to -0 87)

China,Prevalence,55 ~ 60,-0 86(-0 93 to -0 79)

China,Prevalence,60 ~ 65,-0 71(-0 77 to -0 64)

China,Prevalence,65 ~ 70,-0 52(-0 58 to -0 46)

China,Prevalence,70 ~ 75,-0 3(-0 36 to -0 23)

China,Prevalence,75 ~ 80,-0 04(-0 12 to 0 03)

China,Prevalence,80 ~ 85,0 23(0 12 to 0 33)

China,Prevalence,85 ~ 90,0 52(0 34 to 0 71)

China,Prevalence,90 ~ 95,0 89(0 43 to 1 36)

China,Prevalence,20 ~ 25,-0 87(-1 17 to -0 57)

China,Prevalence,25 ~ 30,-0 88(-1 07 to -0 68)

China,Prevalence,30 ~ 35,-0 87(-1 01 to -0 72)

China,Prevalence,35 ~ 40,-0 87(-0 99 to -0 74)

China,Prevalence,40 ~ 45,-0 9(-1 to -0 79)

China,Prevalence,45 ~ 50,-0 94(-1 03 to -0 85)

China,Prevalence,50 ~ 55,-0 95(-1 02 to -0 87)

China,Prevalence,55 ~ 60,-0 86(-0 93 to -0 79)

China,Prevalence,60 ~ 65,-0 71(-0 77 to -0 64)

China,Prevalence,65 ~ 70,-0 52(-0 58 to -0 46)

China,Prevalence,70 ~ 75,-0 3(-0 36 to -0 23)

China,Prevalence,75 ~ 80,-0 04(-0 12 to 0 03)

China,Prevalence,80 ~ 85,0 23(0 12 to 0 33)

China,Prevalence,85 ~ 90,0 52(0 34 to 0 71)

China,Prevalence,90 ~ 95,0 89(0 43 to 1 36)

China,Prevalence,20 ~ 25,-0 87(-1 17 to -0 57)

China,Prevalence,25 ~ 30,-0 88(-1 07 to -0 68)

China,Prevalence,30 ~ 35,-0 87(-1 01 to -0 72)

China,Prevalence,35 ~ 40,-0 87(-0 99 to -0 74)

China,Prevalence,40 ~ 45,-0 9(-1 to -0 79)

China,Prevalence,45 ~ 50,-0 94(-1 03 to -0 85)

China,Prevalence,50 ~ 55,-0 95(-1 02 to -0 87)

China,Prevalence,55 ~ 60,-0 86(-0 93 to -0 79)

China,Prevalence,60 ~ 65,-0 71(-0 77 to -0 64)

China,Prevalence,65 ~ 70,-0 52(-0 58 to -0 46)

China,Prevalence,70 ~ 75,-0 3(-0 36 to -0 23)

China,Prevalence,75 ~ 80,-0 04(-0 12 to 0 03)

China,Prevalence,80 ~ 85,0 23(0 12 to 0 33)

China,Prevalence,85 ~ 90,0 52(0 34 to 0 71)

China,Prevalence,90 ~ 95,0 89(0 43 to 1 36)

China,Deaths,20 ~ 25,-4 9(-7 8 to -1 9)

China,Deaths,25 ~ 30,-5 11(-6 9 to -3 28)

China,Deaths,30 ~ 35,-5 19(-6 31 to -4 04)

China,Deaths,35 ~ 40,-5 31(-6 14 to -4 47)

China,Deaths,40 ~ 45,-5 45(-6 05 to -4 84)

China,Deaths,45 ~ 50,-5 62(-6 03 to -5 21)

China,Deaths,50 ~ 55,-5 75(-6 02 to -5 47)

China,Deaths,55 ~ 60,-5 71(-5 92 to -5 51)

China,Deaths,60 ~ 65,-5 53(-5 69 to -5 37)

China,Deaths,65 ~ 70,-5 1(-5 21 to -4 98)

China,Deaths,70 ~ 75,-4 69(-4 78 to -4 59)

China,Deaths,75 ~ 80,-4 3(-4 39 to -4 22)

China,Deaths,80 ~ 85,-3 9(-3 98 to -3 81)

China,Deaths,85 ~ 90,-3 53(-3 64 to -3 42)

China,Deaths,90 ~ 95,-3 29(-3 51 to -3 08)

China,Deaths,20 ~ 25,-4 9(-7 8 to -1 9)

China,Deaths,25 ~ 30,-5 11(-6 9 to -3 28)

China,Deaths,30 ~ 35,-5 19(-6 31 to -4 04)

China,Deaths,35 ~ 40,-5 31(-6 14 to -4 47)

China,Deaths,40 ~ 45,-5 45(-6 05 to -4 84)

China,Deaths,45 ~ 50,-5 62(-6 03 to -5 21)

China,Deaths,50 ~ 55,-5 75(-6 02 to -5 47)

China,Deaths,55 ~ 60,-5 71(-5 92 to -5 51)

China,Deaths,60 ~ 65,-5 53(-5 69 to -5 37)

China,Deaths,65 ~ 70,-5 1(-5 21 to -4 98)

China,Deaths,70 ~ 75,-4 69(-4 78 to -4 59)

China,Deaths,75 ~ 80,-4 3(-4 39 to -4 22)

China,Deaths,80 ~ 85,-3 9(-3 98 to -3 81)

China,Deaths,85 ~ 90,-3 53(-3 64 to -3 42)

China,Deaths,90 ~ 95,-3 29(-3 51 to -3 08)

China,Deaths,20 ~ 25,-4 9(-7 8 to -1 9)

China,Deaths,25 ~ 30,-5 11(-6 9 to -3 28)

China,Deaths,30 ~ 35,-5 19(-6 31 to -4 04)

China,Deaths,35 ~ 40,-5 31(-6 14 to -4 47)

China,Deaths,40 ~ 45,-5 45(-6 05 to -4 84)

China,Deaths,45 ~ 50,-5 62(-6 03 to -5 21)

China,Deaths,50 ~ 55,-5 75(-6 02 to -5 47)

China,Deaths,55 ~ 60,-5 71(-5 92 to -5 51)

China,Deaths,60 ~ 65,-5 53(-5 69 to -5 37)

China,Deaths,65 ~ 70,-5 1(-5 21 to -4 98)

China,Deaths,70 ~ 75,-4 69(-4 78 to -4 59)

China,Deaths,75 ~ 80,-4 3(-4 39 to -4 22)

China,Deaths,80 ~ 85,-3 9(-3 98 to -3 81)

China,Deaths,85 ~ 90,-3 53(-3 64 to -3 42)

China,Deaths,90 ~ 95,-3 29(-3 51 to -3 08)

China,Deaths,20 ~ 25,-4 9(-7 8 to -1 9)

China,Deaths,25 ~ 30,-5 11(-6 9 to -3 28)

China,Deaths,30 ~ 35,-5 19(-6 31 to -4 04)

China,Deaths,35 ~ 40,-5 31(-6 14 to -4 47)

China,Deaths,40 ~ 45,-5 45(-6 05 to -4 84)

China,Deaths,45 ~ 50,-5 62(-6 03 to -5 21)

China,Deaths,50 ~ 55,-5 75(-6 02 to -5 47)

China,Deaths,55 ~ 60,-5 71(-5 92 to -5 51)

China,Deaths,60 ~ 65,-5 53(-5 69 to -5 37)

China,Deaths,65 ~ 70,-5 1(-5 21 to -4 98)

China,Deaths,70 ~ 75,-4 69(-4 78 to -4 59)

China,Deaths,75 ~ 80,-4 3(-4 39 to -4 22)

China,Deaths,80 ~ 85,-3 9(-3 98 to -3 81)

China,Deaths,85 ~ 90,-3 53(-3 64 to -3 42)

China,Deaths,90 ~ 95,-3 29(-3 51 to -3 08)

China,DALYs,20 ~ 25,-2 95(-3 76 to -2 13)

China,DALYs,25 ~ 30,-3 1(-3 61 to -2 58)

China,DALYs,30 ~ 35,-3 3(-3 66 to -2 94)

China,DALYs,35 ~ 40,-3 63(-3 92 to -3 34)

China,DALYs,40 ~ 45,-4 03(-4 27 to -3 8)

China,DALYs,45 ~ 50,-4 4(-4 57 to -4 22)

China,DALYs,50 ~ 55,-4 7(-4 83 to -4 56)

China,DALYs,55 ~ 60,-4 84(-4 95 to -4 73)

China,DALYs,60 ~ 65,-4 84(-4 93 to -4 75)

China,DALYs,65 ~ 70,-4 58(-4 65 to -4 5)

China,DALYs,70 ~ 75,-4 29(-4 36 to -4 23)

China,DALYs,75 ~ 80,-3 99(-4 06 to -3 92)

China,DALYs,80 ~ 85,-3 64(-3 73 to -3 56)

China,DALYs,85 ~ 90,-3 31(-3 43 to -3 19)

China,DALYs,90 ~ 95,-3 11(-3 35 to -2 86)

China,DALYs,20 ~ 25,-2 95(-3 76 to -2 13)

China,DALYs,25 ~ 30,-3 1(-3 61 to -2 58)

China,DALYs,30 ~ 35,-3 3(-3 66 to -2 94)

China,DALYs,35 ~ 40,-3 63(-3 92 to -3 34)

China,DALYs,40 ~ 45,-4 03(-4 27 to -3 8)

China,DALYs,45 ~ 50,-4 4(-4 57 to -4 22)

China,DALYs,50 ~ 55,-4 7(-4 83 to -4 56)

China,DALYs,55 ~ 60,-4 84(-4 95 to -4 73)

China,DALYs,60 ~ 65,-4 84(-4 93 to -4 75)

China,DALYs,65 ~ 70,-4 58(-4 65 to -4 5)

China,DALYs,70 ~ 75,-4 29(-4 36 to -4 23)

China,DALYs,75 ~ 80,-3 99(-4 06 to -3 92)

China,DALYs,80 ~ 85,-3 64(-3 73 to -3 56)

China,DALYs,85 ~ 90,-3 31(-3 43 to -3 19)

China,DALYs,90 ~ 95,-3 11(-3 35 to -2 86)

China,DALYs,20 ~ 25,-2 95(-3 76 to -2 13)

China,DALYs,25 ~ 30,-3 1(-3 61 to -2 58)

China,DALYs,30 ~ 35,-3 3(-3 66 to -2 94)

China,DALYs,35 ~ 40,-3 63(-3 92 to -3 34)

China,DALYs,40 ~ 45,-4 03(-4 27 to -3 8)

China,DALYs,45 ~ 50,-4 4(-4 57 to -4 22)

China,DALYs,50 ~ 55,-4 7(-4 83 to -4 56)

China,DALYs,55 ~ 60,-4 84(-4 95 to -4 73)

China,DALYs,60 ~ 65,-4 84(-4 93 to -4 75)

China,DALYs,65 ~ 70,-4 58(-4 65 to -4 5)

China,DALYs,70 ~ 75,-4 29(-4 36 to -4 23)

China,DALYs,75 ~ 80,-3 99(-4 06 to -3 92)

China,DALYs,80 ~ 85,-3 64(-3 73 to -3 56)

China,DALYs,85 ~ 90,-3 31(-3 43 to -3 19)

China,DALYs,90 ~ 95,-3 11(-3 35 to -2 86)

China,DALYs,20 ~ 25,-2 95(-3 76 to -2 13)

China,DALYs,25 ~ 30,-3 1(-3 61 to -2 58)

China,DALYs,30 ~ 35,-3 3(-3 66 to -2 94)

China,DALYs,35 ~ 40,-3 63(-3 92 to -3 34)

China,DALYs,40 ~ 45,-4 03(-4 27 to -3 8)

China,DALYs,45 ~ 50,-4 4(-4 57 to -4 22)

China,DALYs,50 ~ 55,-4 7(-4 83 to -4 56)

China,DALYs,55 ~ 60,-4 84(-4 95 to -4 73)

China,DALYs,60 ~ 65,-4 84(-4 93 to -4 75)

China,DALYs,65 ~ 70,-4 58(-4 65 to -4 5)

China,DALYs,70 ~ 75,-4 29(-4 36 to -4 23)

China,DALYs,75 ~ 80,-3 99(-4 06 to -3 92)

China,DALYs,80 ~ 85,-3 64(-3 73 to -3 56)

China,DALYs,85 ~ 90,-3 31(-3 43 to -3 19)

China,DALYs,90 ~ 95,-3 11(-3 35 to -2 86)

Iraq,Prevalence,20 ~ 25,-0 23(-0 32 to -0 14)

Iraq,Prevalence,25 ~ 30,-0 21(-0 28 to -0 14)

Iraq,Prevalence,30 ~ 35,-0 17(-0 23 to -0 11)

Iraq,Prevalence,35 ~ 40,-0 11(-0 17 to -0 06)

Iraq,Prevalence,40 ~ 45,-0 04(-0 09 to 0 01)

Iraq,Prevalence,45 ~ 50,0 05(0 01 to 0 1)

Iraq,Prevalence,50 ~ 55,0 17(0 13 to 0 22)

Iraq,Prevalence,55 ~ 60,0 32(0 27 to 0 36)

Iraq,Prevalence,60 ~ 65,0 48(0 43 to 0 53)

Iraq,Prevalence,65 ~ 70,0 66(0 61 to 0 71)

Iraq,Prevalence,70 ~ 75,0 85(0 8 to 0 91)

Iraq,Prevalence,75 ~ 80,1 03(0 97 to 1 09)

Iraq,Prevalence,80 ~ 85,1 19(1 11 to 1 26)

Iraq,Prevalence,85 ~ 90,1 32(1 22 to 1 41)

Iraq,Prevalence,90 ~ 95,1 41(1 26 to 1 57)

Iraq,Prevalence,20 ~ 25,-0 23(-0 32 to -0 14)

Iraq,Prevalence,25 ~ 30,-0 21(-0 28 to -0 14)

Iraq,Prevalence,30 ~ 35,-0 17(-0 23 to -0 11)

Iraq,Prevalence,35 ~ 40,-0 11(-0 17 to -0 06)

Iraq,Prevalence,40 ~ 45,-0 04(-0 09 to 0 01)

Iraq,Prevalence,45 ~ 50,0 05(0 01 to 0 1)

Iraq,Prevalence,50 ~ 55,0 17(0 13 to 0 22)

Iraq,Prevalence,55 ~ 60,0 32(0 27 to 0 36)

Iraq,Prevalence,60 ~ 65,0 48(0 43 to 0 53)

Iraq,Prevalence,65 ~ 70,0 66(0 61 to 0 71)

Iraq,Prevalence,70 ~ 75,0 85(0 8 to 0 91)

Iraq,Prevalence,75 ~ 80,1 03(0 97 to 1 09)

Iraq,Prevalence,80 ~ 85,1 19(1 11 to 1 26)

Iraq,Prevalence,85 ~ 90,1 32(1 22 to 1 41)

Iraq,Prevalence,90 ~ 95,1 41(1 26 to 1 57)

Iraq,Prevalence,20 ~ 25,-0 23(-0 32 to -0 14)

Iraq,Prevalence,25 ~ 30,-0 21(-0 28 to -0 14)

Iraq,Prevalence,30 ~ 35,-0 17(-0 23 to -0 11)

Iraq,Prevalence,35 ~ 40,-0 11(-0 17 to -0 06)

Iraq,Prevalence,40 ~ 45,-0 04(-0 09 to 0 01)

Iraq,Prevalence,45 ~ 50,0 05(0 01 to 0 1)

Iraq,Prevalence,50 ~ 55,0 17(0 13 to 0 22)

Iraq,Prevalence,55 ~ 60,0 32(0 27 to 0 36)

Iraq,Prevalence,60 ~ 65,0 48(0 43 to 0 53)

Iraq,Prevalence,65 ~ 70,0 66(0 61 to 0 71)

Iraq,Prevalence,70 ~ 75,0 85(0 8 to 0 91)

Iraq,Prevalence,75 ~ 80,1 03(0 97 to 1 09)

Iraq,Prevalence,80 ~ 85,1 19(1 11 to 1 26)

Iraq,Prevalence,85 ~ 90,1 32(1 22 to 1 41)

Iraq,Prevalence,90 ~ 95,1 41(1 26 to 1 57)

Iraq,Prevalence,20 ~ 25,-0 23(-0 32 to -0 14)

Iraq,Prevalence,25 ~ 30,-0 21(-0 28 to -0 14)

Iraq,Prevalence,30 ~ 35,-0 17(-0 23 to -0 11)

Iraq,Prevalence,35 ~ 40,-0 11(-0 17 to -0 06)

Iraq,Prevalence,40 ~ 45,-0 04(-0 09 to 0 01)

Iraq,Prevalence,45 ~ 50,0 05(0 01 to 0 1)

Iraq,Prevalence,50 ~ 55,0 17(0 13 to 0 22)

Iraq,Prevalence,55 ~ 60,0 32(0 27 to 0 36)

Iraq,Prevalence,60 ~ 65,0 48(0 43 to 0 53)

Iraq,Prevalence,65 ~ 70,0 66(0 61 to 0 71)

Iraq,Prevalence,70 ~ 75,0 85(0 8 to 0 91)

Iraq,Prevalence,75 ~ 80,1 03(0 97 to 1 09)

Iraq,Prevalence,80 ~ 85,1 19(1 11 to 1 26)

Iraq,Prevalence,85 ~ 90,1 32(1 22 to 1 41)

Iraq,Prevalence,90 ~ 95,1 41(1 26 to 1 57)

Iraq,Deaths,20 ~ 25,-3 53(-5 48 to -1 53)

Iraq,Deaths,25 ~ 30,-2 99(-4 41 to -1 56)

Iraq,Deaths,30 ~ 35,-2 63(-3 77 to -1 48)

Iraq,Deaths,35 ~ 40,-2 36(-3 29 to -1 41)

Iraq,Deaths,40 ~ 45,-2 18(-2 93 to -1 42)

Iraq,Deaths,45 ~ 50,-2 13(-2 74 to -1 52)

Iraq,Deaths,50 ~ 55,-1 91(-2 41 to -1 4)

Iraq,Deaths,55 ~ 60,-1 63(-2 07 to -1 2)

Iraq,Deaths,60 ~ 65,-1 37(-1 75 to -1)

Iraq,Deaths,65 ~ 70,-1(-1 34 to -0 66)

Iraq,Deaths,70 ~ 75,-0 66(-0 98 to -0 34)

Iraq,Deaths,75 ~ 80,-0 34(-0 67 to -0 02)

Iraq,Deaths,80 ~ 85,-0 06(-0 43 to 0 31)

Iraq,Deaths,85 ~ 90,0 27(-0 2 to 0 75)

Iraq,Deaths,90 ~ 95,0 56(-0 19 to 1 3)

Iraq,Deaths,20 ~ 25,-3 53(-5 48 to -1 53)

Iraq,Deaths,25 ~ 30,-2 99(-4 41 to -1 56)

Iraq,Deaths,30 ~ 35,-2 63(-3 77 to -1 48)

Iraq,Deaths,35 ~ 40,-2 36(-3 29 to -1 41)

Iraq,Deaths,40 ~ 45,-2 18(-2 93 to -1 42)

Iraq,Deaths,45 ~ 50,-2 13(-2 74 to -1 52)

Iraq,Deaths,50 ~ 55,-1 91(-2 41 to -1 4)

Iraq,Deaths,55 ~ 60,-1 63(-2 07 to -1 2)

Iraq,Deaths,60 ~ 65,-1 37(-1 75 to -1)

Iraq,Deaths,65 ~ 70,-1(-1 34 to -0 66)

Iraq,Deaths,70 ~ 75,-0 66(-0 98 to -0 34)

Iraq,Deaths,75 ~ 80,-0 34(-0 67 to -0 02)

Iraq,Deaths,80 ~ 85,-0 06(-0 43 to 0 31)

Iraq,Deaths,85 ~ 90,0 27(-0 2 to 0 75)

Iraq,Deaths,90 ~ 95,0 56(-0 19 to 1 3)

Iraq,Deaths,20 ~ 25,-3 53(-5 48 to -1 53)

Iraq,Deaths,25 ~ 30,-2 99(-4 41 to -1 56)

Iraq,Deaths,30 ~ 35,-2 63(-3 77 to -1 48)

Iraq,Deaths,35 ~ 40,-2 36(-3 29 to -1 41)

Iraq,Deaths,40 ~ 45,-2 18(-2 93 to -1 42)

Iraq,Deaths,45 ~ 50,-2 13(-2 74 to -1 52)

Iraq,Deaths,50 ~ 55,-1 91(-2 41 to -1 4)

Iraq,Deaths,55 ~ 60,-1 63(-2 07 to -1 2)

Iraq,Deaths,60 ~ 65,-1 37(-1 75 to -1)

Iraq,Deaths,65 ~ 70,-1(-1 34 to -0 66)

Iraq,Deaths,70 ~ 75,-0 66(-0 98 to -0 34)

Iraq,Deaths,75 ~ 80,-0 34(-0 67 to -0 02)

Iraq,Deaths,80 ~ 85,-0 06(-0 43 to 0 31)

Iraq,Deaths,85 ~ 90,0 27(-0 2 to 0 75)

Iraq,Deaths,90 ~ 95,0 56(-0 19 to 1 3)

Iraq,Deaths,20 ~ 25,-3 53(-5 48 to -1 53)

Iraq,Deaths,25 ~ 30,-2 99(-4 41 to -1 56)

Iraq,Deaths,30 ~ 35,-2 63(-3 77 to -1 48)

Iraq,Deaths,35 ~ 40,-2 36(-3 29 to -1 41)

Iraq,Deaths,40 ~ 45,-2 18(-2 93 to -1 42)

Iraq,Deaths,45 ~ 50,-2 13(-2 74 to -1 52)

Iraq,Deaths,50 ~ 55,-1 91(-2 41 to -1 4)

Iraq,Deaths,55 ~ 60,-1 63(-2 07 to -1 2)

Iraq,Deaths,60 ~ 65,-1 37(-1 75 to -1)

Iraq,Deaths,65 ~ 70,-1(-1 34 to -0 66)

Iraq,Deaths,70 ~ 75,-0 66(-0 98 to -0 34)

Iraq,Deaths,75 ~ 80,-0 34(-0 67 to -0 02)

Iraq,Deaths,80 ~ 85,-0 06(-0 43 to 0 31)

Iraq,Deaths,85 ~ 90,0 27(-0 2 to 0 75)

Iraq,Deaths,90 ~ 95,0 56(-0 19 to 1 3)

Iraq,DALYs,20 ~ 25,-1 55(-1 93 to -1 16)

Iraq,DALYs,25 ~ 30,-1 32(-1 61 to -1 03)

Iraq,DALYs,30 ~ 35,-1 18(-1 44 to -0 93)

Iraq,DALYs,35 ~ 40,-1 11(-1 34 to -0 88)

Iraq,DALYs,40 ~ 45,-1 08(-1 28 to -0 88)

Iraq,DALYs,45 ~ 50,-1 1(-1 28 to -0 92)

Iraq,DALYs,50 ~ 55,-1 02(-1 18 to -0 85)

Iraq,DALYs,55 ~ 60,-0 87(-1 03 to -0 71)

Iraq,DALYs,60 ~ 65,-0 71(-0 86 to -0 56)

Iraq,DALYs,65 ~ 70,-0 47(-0 62 to -0 32)

Iraq,DALYs,70 ~ 75,-0 24(-0 4 to -0 09)

Iraq,DALYs,75 ~ 80,-0 02(-0 2 to 0 17)

Iraq,DALYs,80 ~ 85,0 21(-0 02 to 0 44)

Iraq,DALYs,85 ~ 90,0 5(0 18 to 0 82)

Iraq,DALYs,90 ~ 95,0 74(0 22 to 1 26)

Iraq,DALYs,20 ~ 25,-1 55(-1 93 to -1 16)

Iraq,DALYs,25 ~ 30,-1 32(-1 61 to -1 03)

Iraq,DALYs,30 ~ 35,-1 18(-1 44 to -0 93)

Iraq,DALYs,35 ~ 40,-1 11(-1 34 to -0 88)

Iraq,DALYs,40 ~ 45,-1 08(-1 28 to -0 88)

Iraq,DALYs,45 ~ 50,-1 1(-1 28 to -0 92)

Iraq,DALYs,50 ~ 55,-1 02(-1 18 to -0 85)

Iraq,DALYs,55 ~ 60,-0 87(-1 03 to -0 71)

Iraq,DALYs,60 ~ 65,-0 71(-0 86 to -0 56)

Iraq,DALYs,65 ~ 70,-0 47(-0 62 to -0 32)

Iraq,DALYs,70 ~ 75,-0 24(-0 4 to -0 09)

Iraq,DALYs,75 ~ 80,-0 02(-0 2 to 0 17)

Iraq,DALYs,80 ~ 85,0 21(-0 02 to 0 44)

Iraq,DALYs,85 ~ 90,0 5(0 18 to 0 82)

Iraq,DALYs,90 ~ 95,0 74(0 22 to 1 26)

Iraq,DALYs,20 ~ 25,-1 55(-1 93 to -1 16)

Iraq,DALYs,25 ~ 30,-1 32(-1 61 to -1 03)

Iraq,DALYs,30 ~ 35,-1 18(-1 44 to -0 93)

Iraq,DALYs,35 ~ 40,-1 11(-1 34 to -0 88)

Iraq,DALYs,40 ~ 45,-1 08(-1 28 to -0 88)

Iraq,DALYs,45 ~ 50,-1 1(-1 28 to -0 92)

Iraq,DALYs,50 ~ 55,-1 02(-1 18 to -0 85)

Iraq,DALYs,55 ~ 60,-0 87(-1 03 to -0 71)

Iraq,DALYs,60 ~ 65,-0 71(-0 86 to -0 56)

Iraq,DALYs,65 ~ 70,-0 47(-0 62 to -0 32)

Iraq,DALYs,70 ~ 75,-0 24(-0 4 to -0 09)

Iraq,DALYs,75 ~ 80,-0 02(-0 2 to 0 17)

Iraq,DALYs,80 ~ 85,0 21(-0 02 to 0 44)

Iraq,DALYs,85 ~ 90,0 5(0 18 to 0 82)

Iraq,DALYs,90 ~ 95,0 74(0 22 to 1 26)

Iraq,DALYs,20 ~ 25,-1 55(-1 93 to -1 16)

Iraq,DALYs,25 ~ 30,-1 32(-1 61 to -1 03)

Iraq,DALYs,30 ~ 35,-1 18(-1 44 to -0 93)

Iraq,DALYs,35 ~ 40,-1 11(-1 34 to -0 88)

Iraq,DALYs,40 ~ 45,-1 08(-1 28 to -0 88)

Iraq,DALYs,45 ~ 50,-1 1(-1 28 to -0 92)

Iraq,DALYs,50 ~ 55,-1 02(-1 18 to -0 85)

Iraq,DALYs,55 ~ 60,-0 87(-1 03 to -0 71)

Iraq,DALYs,60 ~ 65,-0 71(-0 86 to -0 56)

Iraq,DALYs,65 ~ 70,-0 47(-0 62 to -0 32)

Iraq,DALYs,70 ~ 75,-0 24(-0 4 to -0 09)

Iraq,DALYs,75 ~ 80,-0 02(-0 2 to 0 17)

Iraq,DALYs,80 ~ 85,0 21(-0 02 to 0 44)

Iraq,DALYs,85 ~ 90,0 5(0 18 to 0 82)

Iraq,DALYs,90 ~ 95,0 74(0 22 to 1 26)

Kuwait,Prevalence,20 ~ 25,0 05(-0 12 to 0 22)

Kuwait,Prevalence,25 ~ 30,0 07(-0 04 to 0 19)

Kuwait,Prevalence,30 ~ 35,0 08(-0 01 to 0 18)

Kuwait,Prevalence,35 ~ 40,0 07(-0 01 to 0 15)

Kuwait,Prevalence,40 ~ 45,0 07(-0 01 to 0 15)

Kuwait,Prevalence,45 ~ 50,0 09(0 01 to 0 18)

Kuwait,Prevalence,50 ~ 55,0 15(0 07 to 0 23)

Kuwait,Prevalence,55 ~ 60,0 24(0 15 to 0 32)

Kuwait,Prevalence,60 ~ 65,0 38(0 3 to 0 46)

Kuwait,Prevalence,65 ~ 70,0 54(0 46 to 0 63)

Kuwait,Prevalence,70 ~ 75,0 74(0 64 to 0 84)

Kuwait,Prevalence,75 ~ 80,0 95(0 84 to 1 07)

Kuwait,Prevalence,80 ~ 85,1 15(1 to 1 3)

Kuwait,Prevalence,85 ~ 90,1 35(1 15 to 1 56)

Kuwait,Prevalence,90 ~ 95,1 5(1 15 to 1 85)

Kuwait,Prevalence,20 ~ 25,0 05(-0 12 to 0 22)

Kuwait,Prevalence,25 ~ 30,0 07(-0 04 to 0 19)

Kuwait,Prevalence,30 ~ 35,0 08(-0 01 to 0 18)

Kuwait,Prevalence,35 ~ 40,0 07(-0 01 to 0 15)

Kuwait,Prevalence,40 ~ 45,0 07(-0 01 to 0 15)

Kuwait,Prevalence,45 ~ 50,0 09(0 01 to 0 18)

Kuwait,Prevalence,50 ~ 55,0 15(0 07 to 0 23)

Kuwait,Prevalence,55 ~ 60,0 24(0 15 to 0 32)

Kuwait,Prevalence,60 ~ 65,0 38(0 3 to 0 46)

Kuwait,Prevalence,65 ~ 70,0 54(0 46 to 0 63)

Kuwait,Prevalence,70 ~ 75,0 74(0 64 to 0 84)

Kuwait,Prevalence,75 ~ 80,0 95(0 84 to 1 07)

Kuwait,Prevalence,80 ~ 85,1 15(1 to 1 3)

Kuwait,Prevalence,85 ~ 90,1 35(1 15 to 1 56)

Kuwait,Prevalence,90 ~ 95,1 5(1 15 to 1 85)

Kuwait,Prevalence,20 ~ 25,0 05(-0 12 to 0 22)

Kuwait,Prevalence,25 ~ 30,0 07(-0 04 to 0 19)

Kuwait,Prevalence,30 ~ 35,0 08(-0 01 to 0 18)

Kuwait,Prevalence,35 ~ 40,0 07(-0 01 to 0 15)

Kuwait,Prevalence,40 ~ 45,0 07(-0 01 to 0 15)

Kuwait,Prevalence,45 ~ 50,0 09(0 01 to 0 18)

Kuwait,Prevalence,50 ~ 55,0 15(0 07 to 0 23)

Kuwait,Prevalence,55 ~ 60,0 24(0 15 to 0 32)

Kuwait,Prevalence,60 ~ 65,0 38(0 3 to 0 46)

Kuwait,Prevalence,65 ~ 70,0 54(0 46 to 0 63)

Kuwait,Prevalence,70 ~ 75,0 74(0 64 to 0 84)

Kuwait,Prevalence,75 ~ 80,0 95(0 84 to 1 07)

Kuwait,Prevalence,80 ~ 85,1 15(1 to 1 3)

Kuwait,Prevalence,85 ~ 90,1 35(1 15 to 1 56)

Kuwait,Prevalence,90 ~ 95,1 5(1 15 to 1 85)

Kuwait,Prevalence,20 ~ 25,0 05(-0 12 to 0 22)

Kuwait,Prevalence,25 ~ 30,0 07(-0 04 to 0 19)

Kuwait,Prevalence,30 ~ 35,0 08(-0 01 to 0 18)

Kuwait,Prevalence,35 ~ 40,0 07(-0 01 to 0 15)

Kuwait,Prevalence,40 ~ 45,0 07(-0 01 to 0 15)

Kuwait,Prevalence,45 ~ 50,0 09(0 01 to 0 18)

Kuwait,Prevalence,50 ~ 55,0 15(0 07 to 0 23)

Kuwait,Prevalence,55 ~ 60,0 24(0 15 to 0 32)

Kuwait,Prevalence,60 ~ 65,0 38(0 3 to 0 46)

Kuwait,Prevalence,65 ~ 70,0 54(0 46 to 0 63)

Kuwait,Prevalence,70 ~ 75,0 74(0 64 to 0 84)

Kuwait,Prevalence,75 ~ 80,0 95(0 84 to 1 07)

Kuwait,Prevalence,80 ~ 85,1 15(1 to 1 3)

Kuwait,Prevalence,85 ~ 90,1 35(1 15 to 1 56)

Kuwait,Prevalence,90 ~ 95,1 5(1 15 to 1 85)

Kuwait,Deaths,20 ~ 25,-4 32(-18 23 to 11 97)

Kuwait,Deaths,25 ~ 30,-4 43(-13 61 to 5 72)

Kuwait,Deaths,30 ~ 35,-4 4(-11 14 to 2 85)

Kuwait,Deaths,35 ~ 40,-4 24(-9 57 to 1 4)

Kuwait,Deaths,40 ~ 45,-4 15(-8 58 to 0 48)

Kuwait,Deaths,45 ~ 50,-4 14(-7 9 to -0 23)

Kuwait,Deaths,50 ~ 55,-4 19(-7 37 to -0 9)

Kuwait,Deaths,55 ~ 60,-4 23(-6 96 to -1 43)

Kuwait,Deaths,60 ~ 65,-4(-6 34 to -1 6)

Kuwait,Deaths,65 ~ 70,-3 61(-5 7 to -1 47)

Kuwait,Deaths,70 ~ 75,-3 14(-5 07 to -1 17)

Kuwait,Deaths,75 ~ 80,-2 61(-4 46 to -0 72)

Kuwait,Deaths,80 ~ 85,-2 14(-4 04 to -0 21)

Kuwait,Deaths,85 ~ 90,-1 75(-4 04 to 0 59)

Kuwait,Deaths,90 ~ 95,-1 46(-5 23 to 2 46)

Kuwait,Deaths,20 ~ 25,-4 32(-18 23 to 11 97)

Kuwait,Deaths,25 ~ 30,-4 43(-13 61 to 5 72)

Kuwait,Deaths,30 ~ 35,-4 4(-11 14 to 2 85)

Kuwait,Deaths,35 ~ 40,-4 24(-9 57 to 1 4)

Kuwait,Deaths,40 ~ 45,-4 15(-8 58 to 0 48)

Kuwait,Deaths,45 ~ 50,-4 14(-7 9 to -0 23)

Kuwait,Deaths,50 ~ 55,-4 19(-7 37 to -0 9)

Kuwait,Deaths,55 ~ 60,-4 23(-6 96 to -1 43)

Kuwait,Deaths,60 ~ 65,-4(-6 34 to -1 6)

Kuwait,Deaths,65 ~ 70,-3 61(-5 7 to -1 47)

Kuwait,Deaths,70 ~ 75,-3 14(-5 07 to -1 17)

Kuwait,Deaths,75 ~ 80,-2 61(-4 46 to -0 72)

Kuwait,Deaths,80 ~ 85,-2 14(-4 04 to -0 21)

Kuwait,Deaths,85 ~ 90,-1 75(-4 04 to 0 59)

Kuwait,Deaths,90 ~ 95,-1 46(-5 23 to 2 46)

Kuwait,Deaths,20 ~ 25,-4 32(-18 23 to 11 97)

Kuwait,Deaths,25 ~ 30,-4 43(-13 61 to 5 72)

Kuwait,Deaths,30 ~ 35,-4 4(-11 14 to 2 85)

Kuwait,Deaths,35 ~ 40,-4 24(-9 57 to 1 4)

Kuwait,Deaths,40 ~ 45,-4 15(-8 58 to 0 48)

Kuwait,Deaths,45 ~ 50,-4 14(-7 9 to -0 23)

Kuwait,Deaths,50 ~ 55,-4 19(-7 37 to -0 9)

Kuwait,Deaths,55 ~ 60,-4 23(-6 96 to -1 43)

Kuwait,Deaths,60 ~ 65,-4(-6 34 to -1 6)

Kuwait,Deaths,65 ~ 70,-3 61(-5 7 to -1 47)

Kuwait,Deaths,70 ~ 75,-3 14(-5 07 to -1 17)

Kuwait,Deaths,75 ~ 80,-2 61(-4 46 to -0 72)

Kuwait,Deaths,80 ~ 85,-2 14(-4 04 to -0 21)

Kuwait,Deaths,85 ~ 90,-1 75(-4 04 to 0 59)

Kuwait,Deaths,90 ~ 95,-1 46(-5 23 to 2 46)

Kuwait,Deaths,20 ~ 25,-4 32(-18 23 to 11 97)

Kuwait,Deaths,25 ~ 30,-4 43(-13 61 to 5 72)

Kuwait,Deaths,30 ~ 35,-4 4(-11 14 to 2 85)

Kuwait,Deaths,35 ~ 40,-4 24(-9 57 to 1 4)

Kuwait,Deaths,40 ~ 45,-4 15(-8 58 to 0 48)

Kuwait,Deaths,45 ~ 50,-4 14(-7 9 to -0 23)

Kuwait,Deaths,50 ~ 55,-4 19(-7 37 to -0 9)

Kuwait,Deaths,55 ~ 60,-4 23(-6 96 to -1 43)

Kuwait,Deaths,60 ~ 65,-4(-6 34 to -1 6)

Kuwait,Deaths,65 ~ 70,-3 61(-5 7 to -1 47)

Kuwait,Deaths,70 ~ 75,-3 14(-5 07 to -1 17)

Kuwait,Deaths,75 ~ 80,-2 61(-4 46 to -0 72)

Kuwait,Deaths,80 ~ 85,-2 14(-4 04 to -0 21)

Kuwait,Deaths,85 ~ 90,-1 75(-4 04 to 0 59)

Kuwait,Deaths,90 ~ 95,-1 46(-5 23 to 2 46)

Kuwait,DALYs,20 ~ 25,-0 4(-1 02 to 0 22)

Kuwait,DALYs,25 ~ 30,-0 39(-0 81 to 0 03)

Kuwait,DALYs,30 ~ 35,-0 42(-0 75 to -0 08)

Kuwait,DALYs,35 ~ 40,-0 5(-0 8 to -0 2)

Kuwait,DALYs,40 ~ 45,-0 63(-0 91 to -0 34)

Kuwait,DALYs,45 ~ 50,-0 77(-1 05 to -0 49)

Kuwait,DALYs,50 ~ 55,-0 94(-1 22 to -0 67)

Kuwait,DALYs,55 ~ 60,-1 13(-1 4 to -0 86)

Kuwait,DALYs,60 ~ 65,-1 18(-1 44 to -0 92)

Kuwait,DALYs,65 ~ 70,-1 12(-1 39 to -0 85)

Kuwait,DALYs,70 ~ 75,-0 99(-1 28 to -0 7)

Kuwait,DALYs,75 ~ 80,-0 75(-1 08 to -0 42)

Kuwait,DALYs,80 ~ 85,-0 51(-0 92 to -0 1)

Kuwait,DALYs,85 ~ 90,-0 24(-0 8 to 0 31)

Kuwait,DALYs,90 ~ 95,-0 01(-0 97 to 0 97)

Kuwait,DALYs,20 ~ 25,-0 4(-1 02 to 0 22)

Kuwait,DALYs,25 ~ 30,-0 39(-0 81 to 0 03)

Kuwait,DALYs,30 ~ 35,-0 42(-0 75 to -0 08)

Kuwait,DALYs,35 ~ 40,-0 5(-0 8 to -0 2)

Kuwait,DALYs,40 ~ 45,-0 63(-0 91 to -0 34)

Kuwait,DALYs,45 ~ 50,-0 77(-1 05 to -0 49)

Kuwait,DALYs,50 ~ 55,-0 94(-1 22 to -0 67)

Kuwait,DALYs,55 ~ 60,-1 13(-1 4 to -0 86)

Kuwait,DALYs,60 ~ 65,-1 18(-1 44 to -0 92)

Kuwait,DALYs,65 ~ 70,-1 12(-1 39 to -0 85)

Kuwait,DALYs,70 ~ 75,-0 99(-1 28 to -0 7)

Kuwait,DALYs,75 ~ 80,-0 75(-1 08 to -0 42)

Kuwait,DALYs,80 ~ 85,-0 51(-0 92 to -0 1)

Kuwait,DALYs,85 ~ 90,-0 24(-0 8 to 0 31)

Kuwait,DALYs,90 ~ 95,-0 01(-0 97 to 0 97)

Kuwait,DALYs,20 ~ 25,-0 4(-1 02 to 0 22)

Kuwait,DALYs,25 ~ 30,-0 39(-0 81 to 0 03)

Kuwait,DALYs,30 ~ 35,-0 42(-0 75 to -0 08)

Kuwait,DALYs,35 ~ 40,-0 5(-0 8 to -0 2)

Kuwait,DALYs,40 ~ 45,-0 63(-0 91 to -0 34)

Kuwait,DALYs,45 ~ 50,-0 77(-1 05 to -0 49)

Kuwait,DALYs,50 ~ 55,-0 94(-1 22 to -0 67)

Kuwait,DALYs,55 ~ 60,-1 13(-1 4 to -0 86)

Kuwait,DALYs,60 ~ 65,-1 18(-1 44 to -0 92)

Kuwait,DALYs,65 ~ 70,-1 12(-1 39 to -0 85)

Kuwait,DALYs,70 ~ 75,-0 99(-1 28 to -0 7)

Kuwait,DALYs,75 ~ 80,-0 75(-1 08 to -0 42)

Kuwait,DALYs,80 ~ 85,-0 51(-0 92 to -0 1)

Kuwait,DALYs,85 ~ 90,-0 24(-0 8 to 0 31)

Kuwait,DALYs,90 ~ 95,-0 01(-0 97 to 0 97)

Kuwait,DALYs,20 ~ 25,-0 4(-1 02 to 0 22)

Kuwait,DALYs,25 ~ 30,-0 39(-0 81 to 0 03)

Kuwait,DALYs,30 ~ 35,-0 42(-0 75 to -0 08)

Kuwait,DALYs,35 ~ 40,-0 5(-0 8 to -0 2)

Kuwait,DALYs,40 ~ 45,-0 63(-0 91 to -0 34)

Kuwait,DALYs,45 ~ 50,-0 77(-1 05 to -0 49)

Kuwait,DALYs,50 ~ 55,-0 94(-1 22 to -0 67)

Kuwait,DALYs,55 ~ 60,-1 13(-1 4 to -0 86)

Kuwait,DALYs,60 ~ 65,-1 18(-1 44 to -0 92)

Kuwait,DALYs,65 ~ 70,-1 12(-1 39 to -0 85)

Kuwait,DALYs,70 ~ 75,-0 99(-1 28 to -0 7)

Kuwait,DALYs,75 ~ 80,-0 75(-1 08 to -0 42)

Kuwait,DALYs,80 ~ 85,-0 51(-0 92 to -0 1)

Kuwait,DALYs,85 ~ 90,-0 24(-0 8 to 0 31)

Kuwait,DALYs,90 ~ 95,-0 01(-0 97 to 0 97)

Saudi_Arabia,Prevalence,20 ~ 25,0 57(0 51 to 0 62)

Saudi_Arabia,Prevalence,25 ~ 30,0 57(0 53 to 0 61)

Saudi_Arabia,Prevalence,30 ~ 35,0 58(0 54 to 0 61)

Saudi_Arabia,Prevalence,35 ~ 40,0 58(0 55 to 0 61)

Saudi_Arabia,Prevalence,40 ~ 45,0 6(0 57 to 0 63)

Saudi_Arabia,Prevalence,45 ~ 50,0 65(0 62 to 0 67)

Saudi_Arabia,Prevalence,50 ~ 55,0 7(0 67 to 0 73)

Saudi_Arabia,Prevalence,55 ~ 60,0 76(0 73 to 0 79)

Saudi_Arabia,Prevalence,60 ~ 65,0 86(0 83 to 0 89)

Saudi_Arabia,Prevalence,65 ~ 70,0 97(0 94 to 1 01)

Saudi_Arabia,Prevalence,70 ~ 75,1 11(1 08 to 1 15)

Saudi_Arabia,Prevalence,75 ~ 80,1 27(1 23 to 1 32)

Saudi_Arabia,Prevalence,80 ~ 85,1 45(1 4 to 1 51)

Saudi_Arabia,Prevalence,85 ~ 90,1 64(1 56 to 1 73)

Saudi_Arabia,Prevalence,90 ~ 95,1 83(1 68 to 1 98)

Saudi_Arabia,Prevalence,20 ~ 25,0 57(0 51 to 0 62)

Saudi_Arabia,Prevalence,25 ~ 30,0 57(0 53 to 0 61)

Saudi_Arabia,Prevalence,30 ~ 35,0 58(0 54 to 0 61)

Saudi_Arabia,Prevalence,35 ~ 40,0 58(0 55 to 0 61)

Saudi_Arabia,Prevalence,40 ~ 45,0 6(0 57 to 0 63)

Saudi_Arabia,Prevalence,45 ~ 50,0 65(0 62 to 0 67)

Saudi_Arabia,Prevalence,50 ~ 55,0 7(0 67 to 0 73)

Saudi_Arabia,Prevalence,55 ~ 60,0 76(0 73 to 0 79)

Saudi_Arabia,Prevalence,60 ~ 65,0 86(0 83 to 0 89)

Saudi_Arabia,Prevalence,65 ~ 70,0 97(0 94 to 1 01)

Saudi_Arabia,Prevalence,70 ~ 75,1 11(1 08 to 1 15)

Saudi_Arabia,Prevalence,75 ~ 80,1 27(1 23 to 1 32)

Saudi_Arabia,Prevalence,80 ~ 85,1 45(1 4 to 1 51)

Saudi_Arabia,Prevalence,85 ~ 90,1 64(1 56 to 1 73)

Saudi_Arabia,Prevalence,90 ~ 95,1 83(1 68 to 1 98)

Saudi_Arabia,Prevalence,20 ~ 25,0 57(0 51 to 0 62)

Saudi_Arabia,Prevalence,25 ~ 30,0 57(0 53 to 0 61)

Saudi_Arabia,Prevalence,30 ~ 35,0 58(0 54 to 0 61)

Saudi_Arabia,Prevalence,35 ~ 40,0 58(0 55 to 0 61)

Saudi_Arabia,Prevalence,40 ~ 45,0 6(0 57 to 0 63)

Saudi_Arabia,Prevalence,45 ~ 50,0 65(0 62 to 0 67)

Saudi_Arabia,Prevalence,50 ~ 55,0 7(0 67 to 0 73)

Saudi_Arabia,Prevalence,55 ~ 60,0 76(0 73 to 0 79)

Saudi_Arabia,Prevalence,60 ~ 65,0 86(0 83 to 0 89)

Saudi_Arabia,Prevalence,65 ~ 70,0 97(0 94 to 1 01)

Saudi_Arabia,Prevalence,70 ~ 75,1 11(1 08 to 1 15)

Saudi_Arabia,Prevalence,75 ~ 80,1 27(1 23 to 1 32)

Saudi_Arabia,Prevalence,80 ~ 85,1 45(1 4 to 1 51)

Saudi_Arabia,Prevalence,85 ~ 90,1 64(1 56 to 1 73)

Saudi_Arabia,Prevalence,90 ~ 95,1 83(1 68 to 1 98)

Saudi_Arabia,Prevalence,20 ~ 25,0 57(0 51 to 0 62)

Saudi_Arabia,Prevalence,25 ~ 30,0 57(0 53 to 0 61)

Saudi_Arabia,Prevalence,30 ~ 35,0 58(0 54 to 0 61)

Saudi_Arabia,Prevalence,35 ~ 40,0 58(0 55 to 0 61)

Saudi_Arabia,Prevalence,40 ~ 45,0 6(0 57 to 0 63)

Saudi_Arabia,Prevalence,45 ~ 50,0 65(0 62 to 0 67)

Saudi_Arabia,Prevalence,50 ~ 55,0 7(0 67 to 0 73)

Saudi_Arabia,Prevalence,55 ~ 60,0 76(0 73 to 0 79)

Saudi_Arabia,Prevalence,60 ~ 65,0 86(0 83 to 0 89)

Saudi_Arabia,Prevalence,65 ~ 70,0 97(0 94 to 1 01)

Saudi_Arabia,Prevalence,70 ~ 75,1 11(1 08 to 1 15)

Saudi_Arabia,Prevalence,75 ~ 80,1 27(1 23 to 1 32)

Saudi_Arabia,Prevalence,80 ~ 85,1 45(1 4 to 1 51)

Saudi_Arabia,Prevalence,85 ~ 90,1 64(1 56 to 1 73)

Saudi_Arabia,Prevalence,90 ~ 95,1 83(1 68 to 1 98)

Saudi_Arabia,Deaths,20 ~ 25,0 19(-1 2 to 1 6)

Saudi_Arabia,Deaths,25 ~ 30,0 53(-0 38 to 1 45)

Saudi_Arabia,Deaths,30 ~ 35,0 63(-0 07 to 1 34)

Saudi_Arabia,Deaths,35 ~ 40,0 54(-0 04 to 1 11)

Saudi_Arabia,Deaths,40 ~ 45,0 27(-0 21 to 0 76)

Saudi_Arabia,Deaths,45 ~ 50,-0 09(-0 51 to 0 33)

Saudi_Arabia,Deaths,50 ~ 55,-0 51(-0 88 to -0 15)

Saudi_Arabia,Deaths,55 ~ 60,-0 94(-1 26 to -0 61)

Saudi_Arabia,Deaths,60 ~ 65,-1 33(-1 61 to -1 04)

Saudi_Arabia,Deaths,65 ~ 70,-1 59(-1 85 to -1 32)

Saudi_Arabia,Deaths,70 ~ 75,-1 77(-2 03 to -1 51)

Saudi_Arabia,Deaths,75 ~ 80,-1 8(-2 07 to -1 54)

Saudi_Arabia,Deaths,80 ~ 85,-1 71(-2 01 to -1 41)

Saudi_Arabia,Deaths,85 ~ 90,-1 43(-1 82 to -1 04)

Saudi_Arabia,Deaths,90 ~ 95,-1 05(-1 7 to -0 4)

Saudi_Arabia,Deaths,20 ~ 25,0 19(-1 2 to 1 6)

Saudi_Arabia,Deaths,25 ~ 30,0 53(-0 38 to 1 45)

Saudi_Arabia,Deaths,30 ~ 35,0 63(-0 07 to 1 34)

Saudi_Arabia,Deaths,35 ~ 40,0 54(-0 04 to 1 11)

Saudi_Arabia,Deaths,40 ~ 45,0 27(-0 21 to 0 76)

Saudi_Arabia,Deaths,45 ~ 50,-0 09(-0 51 to 0 33)

Saudi_Arabia,Deaths,50 ~ 55,-0 51(-0 88 to -0 15)

Saudi_Arabia,Deaths,55 ~ 60,-0 94(-1 26 to -0 61)

Saudi_Arabia,Deaths,60 ~ 65,-1 33(-1 61 to -1 04)

Saudi_Arabia,Deaths,65 ~ 70,-1 59(-1 85 to -1 32)

Saudi_Arabia,Deaths,70 ~ 75,-1 77(-2 03 to -1 51)

Saudi_Arabia,Deaths,75 ~ 80,-1 8(-2 07 to -1 54)

Saudi_Arabia,Deaths,80 ~ 85,-1 71(-2 01 to -1 41)

Saudi_Arabia,Deaths,85 ~ 90,-1 43(-1 82 to -1 04)

Saudi_Arabia,Deaths,90 ~ 95,-1 05(-1 7 to -0 4)

Saudi_Arabia,Deaths,20 ~ 25,0 19(-1 2 to 1 6)

Saudi_Arabia,Deaths,25 ~ 30,0 53(-0 38 to 1 45)

Saudi_Arabia,Deaths,30 ~ 35,0 63(-0 07 to 1 34)

Saudi_Arabia,Deaths,35 ~ 40,0 54(-0 04 to 1 11)

Saudi_Arabia,Deaths,40 ~ 45,0 27(-0 21 to 0 76)

Saudi_Arabia,Deaths,45 ~ 50,-0 09(-0 51 to 0 33)

Saudi_Arabia,Deaths,50 ~ 55,-0 51(-0 88 to -0 15)

Saudi_Arabia,Deaths,55 ~ 60,-0 94(-1 26 to -0 61)

Saudi_Arabia,Deaths,60 ~ 65,-1 33(-1 61 to -1 04)

Saudi_Arabia,Deaths,65 ~ 70,-1 59(-1 85 to -1 32)

Saudi_Arabia,Deaths,70 ~ 75,-1 77(-2 03 to -1 51)

Saudi_Arabia,Deaths,75 ~ 80,-1 8(-2 07 to -1 54)

Saudi_Arabia,Deaths,80 ~ 85,-1 71(-2 01 to -1 41)

Saudi_Arabia,Deaths,85 ~ 90,-1 43(-1 82 to -1 04)

Saudi_Arabia,Deaths,90 ~ 95,-1 05(-1 7 to -0 4)

Saudi_Arabia,Deaths,20 ~ 25,0 19(-1 2 to 1 6)

Saudi_Arabia,Deaths,25 ~ 30,0 53(-0 38 to 1 45)

Saudi_Arabia,Deaths,30 ~ 35,0 63(-0 07 to 1 34)

Saudi_Arabia,Deaths,35 ~ 40,0 54(-0 04 to 1 11)

Saudi_Arabia,Deaths,40 ~ 45,0 27(-0 21 to 0 76)

Saudi_Arabia,Deaths,45 ~ 50,-0 09(-0 51 to 0 33)

Saudi_Arabia,Deaths,50 ~ 55,-0 51(-0 88 to -0 15)

Saudi_Arabia,Deaths,55 ~ 60,-0 94(-1 26 to -0 61)

Saudi_Arabia,Deaths,60 ~ 65,-1 33(-1 61 to -1 04)

Saudi_Arabia,Deaths,65 ~ 70,-1 59(-1 85 to -1 32)

Saudi_Arabia,Deaths,70 ~ 75,-1 77(-2 03 to -1 51)

Saudi_Arabia,Deaths,75 ~ 80,-1 8(-2 07 to -1 54)

Saudi_Arabia,Deaths,80 ~ 85,-1 71(-2 01 to -1 41)

Saudi_Arabia,Deaths,85 ~ 90,-1 43(-1 82 to -1 04)

Saudi_Arabia,Deaths,90 ~ 95,-1 05(-1 7 to -0 4)

Saudi_Arabia,DALYs,20 ~ 25,0 48(0 03 to 0 93)

Saudi_Arabia,DALYs,25 ~ 30,0 67(0 35 to 0 98)

Saudi_Arabia,DALYs,30 ~ 35,0 73(0 47 to 0 98)

Saudi_Arabia,DALYs,35 ~ 40,0 66(0 43 to 0 88)

Saudi_Arabia,DALYs,40 ~ 45,0 47(0 26 to 0 67)

Saudi_Arabia,DALYs,45 ~ 50,0 2(0 01 to 0 38)

Saudi_Arabia,DALYs,50 ~ 55,-0 15(-0 32 to 0 03)

Saudi_Arabia,DALYs,55 ~ 60,-0 51(-0 68 to -0 34)

Saudi_Arabia,DALYs,60 ~ 65,-0 87(-1 03 to -0 71)

Saudi_Arabia,DALYs,65 ~ 70,-1 16(-1 33 to -1)

Saudi_Arabia,DALYs,70 ~ 75,-1 38(-1 56 to -1 21)

Saudi_Arabia,DALYs,75 ~ 80,-1 45(-1 65 to -1 24)

Saudi_Arabia,DALYs,80 ~ 85,-1 39(-1 64 to -1 13)

Saudi_Arabia,DALYs,85 ~ 90,-1 12(-1 49 to -0 74)

Saudi_Arabia,DALYs,90 ~ 95,-0 72(-1 38 to -0 06)

Saudi_Arabia,DALYs,20 ~ 25,0 48(0 03 to 0 93)

Saudi_Arabia,DALYs,25 ~ 30,0 67(0 35 to 0 98)

Saudi_Arabia,DALYs,30 ~ 35,0 73(0 47 to 0 98)

Saudi_Arabia,DALYs,35 ~ 40,0 66(0 43 to 0 88)

Saudi_Arabia,DALYs,40 ~ 45,0 47(0 26 to 0 67)

Saudi_Arabia,DALYs,45 ~ 50,0 2(0 01 to 0 38)

Saudi_Arabia,DALYs,50 ~ 55,-0 15(-0 32 to 0 03)

Saudi_Arabia,DALYs,55 ~ 60,-0 51(-0 68 to -0 34)

Saudi_Arabia,DALYs,60 ~ 65,-0 87(-1 03 to -0 71)

Saudi_Arabia,DALYs,65 ~ 70,-1 16(-1 33 to -1)

Saudi_Arabia,DALYs,70 ~ 75,-1 38(-1 56 to -1 21)

Saudi_Arabia,DALYs,75 ~ 80,-1 45(-1 65 to -1 24)

Saudi_Arabia,DALYs,80 ~ 85,-1 39(-1 64 to -1 13)

Saudi_Arabia,DALYs,85 ~ 90,-1 12(-1 49 to -0 74)

Saudi_Arabia,DALYs,90 ~ 95,-0 72(-1 38 to -0 06)

Saudi_Arabia,DALYs,20 ~ 25,0 48(0 03 to 0 93)

Saudi_Arabia,DALYs,25 ~ 30,0 67(0 35 to 0 98)

Saudi_Arabia,DALYs,30 ~ 35,0 73(0 47 to 0 98)

Saudi_Arabia,DALYs,35 ~ 40,0 66(0 43 to 0 88)

Saudi_Arabia,DALYs,40 ~ 45,0 47(0 26 to 0 67)

Saudi_Arabia,DALYs,45 ~ 50,0 2(0 01 to 0 38)

Saudi_Arabia,DALYs,50 ~ 55,-0 15(-0 32 to 0 03)

Saudi_Arabia,DALYs,55 ~ 60,-0 51(-0 68 to -0 34)

Saudi_Arabia,DALYs,60 ~ 65,-0 87(-1 03 to -0 71)

Saudi_Arabia,DALYs,65 ~ 70,-1 16(-1 33 to -1)

Saudi_Arabia,DALYs,70 ~ 75,-1 38(-1 56 to -1 21)

Saudi_Arabia,DALYs,75 ~ 80,-1 45(-1 65 to -1 24)

Saudi_Arabia,DALYs,80 ~ 85,-1 39(-1 64 to -1 13)

Saudi_Arabia,DALYs,85 ~ 90,-1 12(-1 49 to -0 74)

Saudi_Arabia,DALYs,90 ~ 95,-0 72(-1 38 to -0 06)

Saudi_Arabia,DALYs,20 ~ 25,0 48(0 03 to 0 93)

Saudi_Arabia,DALYs,25 ~ 30,0 67(0 35 to 0 98)

Saudi_Arabia,DALYs,30 ~ 35,0 73(0 47 to 0 98)

Saudi_Arabia,DALYs,35 ~ 40,0 66(0 43 to 0 88)

Saudi_Arabia,DALYs,40 ~ 45,0 47(0 26 to 0 67)

Saudi_Arabia,DALYs,45 ~ 50,0 2(0 01 to 0 38)

Saudi_Arabia,DALYs,50 ~ 55,-0 15(-0 32 to 0 03)

Saudi_Arabia,DALYs,55 ~ 60,-0 51(-0 68 to -0 34)

Saudi_Arabia,DALYs,60 ~ 65,-0 87(-1 03 to -0 71)

Saudi_Arabia,DALYs,65 ~ 70,-1 16(-1 33 to -1)

Saudi_Arabia,DALYs,70 ~ 75,-1 38(-1 56 to -1 21)

Saudi_Arabia,DALYs,75 ~ 80,-1 45(-1 65 to -1 24)

Saudi_Arabia,DALYs,80 ~ 85,-1 39(-1 64 to -1 13)

Saudi_Arabia,DALYs,85 ~ 90,-1 12(-1 49 to -0 74)

Saudi_Arabia,DALYs,90 ~ 95,-0 72(-1 38 to -0 06)

Sierra_Leone,Prevalence,20 ~ 25,-0 09(-0 2 to 0 01)

Sierra_Leone,Prevalence,25 ~ 30,-0 05(-0 14 to 0 03)

Sierra_Leone,Prevalence,30 ~ 35,-0 02(-0 09 to 0 06)

Sierra_Leone,Prevalence,35 ~ 40,0 02(-0 04 to 0 09)

Sierra_Leone,Prevalence,40 ~ 45,0 07(0 01 to 0 14)

Sierra_Leone,Prevalence,45 ~ 50,0 12(0 06 to 0 19)

Sierra_Leone,Prevalence,50 ~ 55,0 18(0 12 to 0 24)

Sierra_Leone,Prevalence,55 ~ 60,0 25(0 19 to 0 3)

Sierra_Leone,Prevalence,60 ~ 65,0 3(0 24 to 0 36)

Sierra_Leone,Prevalence,65 ~ 70,0 36(0 3 to 0 42)

Sierra_Leone,Prevalence,70 ~ 75,0 42(0 36 to 0 49)

Sierra_Leone,Prevalence,75 ~ 80,0 49(0 41 to 0 56)

Sierra_Leone,Prevalence,80 ~ 85,0 57(0 47 to 0 66)

Sierra_Leone,Prevalence,85 ~ 90,0 68(0 52 to 0 84)

Sierra_Leone,Prevalence,90 ~ 95,0 83(0 54 to 1 13)

Sierra_Leone,Prevalence,20 ~ 25,-0 09(-0 2 to 0 01)

Sierra_Leone,Prevalence,25 ~ 30,-0 05(-0 14 to 0 03)

Sierra_Leone,Prevalence,30 ~ 35,-0 02(-0 09 to 0 06)

Sierra_Leone,Prevalence,35 ~ 40,0 02(-0 04 to 0 09)

Sierra_Leone,Prevalence,40 ~ 45,0 07(0 01 to 0 14)

Sierra_Leone,Prevalence,45 ~ 50,0 12(0 06 to 0 19)

Sierra_Leone,Prevalence,50 ~ 55,0 18(0 12 to 0 24)

Sierra_Leone,Prevalence,55 ~ 60,0 25(0 19 to 0 3)

Sierra_Leone,Prevalence,60 ~ 65,0 3(0 24 to 0 36)

Sierra_Leone,Prevalence,65 ~ 70,0 36(0 3 to 0 42)

Sierra_Leone,Prevalence,70 ~ 75,0 42(0 36 to 0 49)

Sierra_Leone,Prevalence,75 ~ 80,0 49(0 41 to 0 56)

Sierra_Leone,Prevalence,80 ~ 85,0 57(0 47 to 0 66)

Sierra_Leone,Prevalence,85 ~ 90,0 68(0 52 to 0 84)

Sierra_Leone,Prevalence,90 ~ 95,0 83(0 54 to 1 13)

Sierra_Leone,Prevalence,20 ~ 25,-0 09(-0 2 to 0 01)

Sierra_Leone,Prevalence,25 ~ 30,-0 05(-0 14 to 0 03)

Sierra_Leone,Prevalence,30 ~ 35,-0 02(-0 09 to 0 06)

Sierra_Leone,Prevalence,35 ~ 40,0 02(-0 04 to 0 09)

Sierra_Leone,Prevalence,40 ~ 45,0 07(0 01 to 0 14)

Sierra_Leone,Prevalence,45 ~ 50,0 12(0 06 to 0 19)

Sierra_Leone,Prevalence,50 ~ 55,0 18(0 12 to 0 24)

Sierra_Leone,Prevalence,55 ~ 60,0 25(0 19 to 0 3)

Sierra_Leone,Prevalence,60 ~ 65,0 3(0 24 to 0 36)

Sierra_Leone,Prevalence,65 ~ 70,0 36(0 3 to 0 42)

Sierra_Leone,Prevalence,70 ~ 75,0 42(0 36 to 0 49)

Sierra_Leone,Prevalence,75 ~ 80,0 49(0 41 to 0 56)

Sierra_Leone,Prevalence,80 ~ 85,0 57(0 47 to 0 66)

Sierra_Leone,Prevalence,85 ~ 90,0 68(0 52 to 0 84)

Sierra_Leone,Prevalence,90 ~ 95,0 83(0 54 to 1 13)

Sierra_Leone,Prevalence,20 ~ 25,-0 09(-0 2 to 0 01)

Sierra_Leone,Prevalence,25 ~ 30,-0 05(-0 14 to 0 03)

Sierra_Leone,Prevalence,30 ~ 35,-0 02(-0 09 to 0 06)

Sierra_Leone,Prevalence,35 ~ 40,0 02(-0 04 to 0 09)

Sierra_Leone,Prevalence,40 ~ 45,0 07(0 01 to 0 14)

Sierra_Leone,Prevalence,45 ~ 50,0 12(0 06 to 0 19)

Sierra_Leone,Prevalence,50 ~ 55,0 18(0 12 to 0 24)

Sierra_Leone,Prevalence,55 ~ 60,0 25(0 19 to 0 3)

Sierra_Leone,Prevalence,60 ~ 65,0 3(0 24 to 0 36)

Sierra_Leone,Prevalence,65 ~ 70,0 36(0 3 to 0 42)

Sierra_Leone,Prevalence,70 ~ 75,0 42(0 36 to 0 49)

Sierra_Leone,Prevalence,75 ~ 80,0 49(0 41 to 0 56)

Sierra_Leone,Prevalence,80 ~ 85,0 57(0 47 to 0 66)

Sierra_Leone,Prevalence,85 ~ 90,0 68(0 52 to 0 84)

Sierra_Leone,Prevalence,90 ~ 95,0 83(0 54 to 1 13)

Sierra_Leone,Deaths,20 ~ 25,1 13(-2 69 to 5 1)

Sierra_Leone,Deaths,25 ~ 30,0 97(-1 36 to 3 35)

Sierra_Leone,Deaths,30 ~ 35,0 68(-1 21 to 2 6)

Sierra_Leone,Deaths,35 ~ 40,0 35(-1 27 to 1 99)

Sierra_Leone,Deaths,40 ~ 45,0 07(-1 26 to 1 42)

Sierra_Leone,Deaths,45 ~ 50,-0 16(-1 21 to 0 9)

Sierra_Leone,Deaths,50 ~ 55,-0 29(-1 14 to 0 56)

Sierra_Leone,Deaths,55 ~ 60,-0 37(-1 06 to 0 33)

Sierra_Leone,Deaths,60 ~ 65,-0 44(-1 01 to 0 13)

Sierra_Leone,Deaths,65 ~ 70,-0 55(-1 03 to -0 07)

Sierra_Leone,Deaths,70 ~ 75,-0 66(-1 09 to -0 24)

Sierra_Leone,Deaths,75 ~ 80,-0 68(-1 1 to -0 25)

Sierra_Leone,Deaths,80 ~ 85,-0 69(-1 18 to -0 19)

Sierra_Leone,Deaths,85 ~ 90,-0 65(-1 35 to 0 06)

Sierra_Leone,Deaths,90 ~ 95,-0 56(-1 73 to 0 62)

Sierra_Leone,Deaths,20 ~ 25,1 13(-2 69 to 5 1)

Sierra_Leone,Deaths,25 ~ 30,0 97(-1 36 to 3 35)

Sierra_Leone,Deaths,30 ~ 35,0 68(-1 21 to 2 6)

Sierra_Leone,Deaths,35 ~ 40,0 35(-1 27 to 1 99)

Sierra_Leone,Deaths,40 ~ 45,0 07(-1 26 to 1 42)

Sierra_Leone,Deaths,45 ~ 50,-0 16(-1 21 to 0 9)

Sierra_Leone,Deaths,50 ~ 55,-0 29(-1 14 to 0 56)

Sierra_Leone,Deaths,55 ~ 60,-0 37(-1 06 to 0 33)

Sierra_Leone,Deaths,60 ~ 65,-0 44(-1 01 to 0 13)

Sierra_Leone,Deaths,65 ~ 70,-0 55(-1 03 to -0 07)

Sierra_Leone,Deaths,70 ~ 75,-0 66(-1 09 to -0 24)

Sierra_Leone,Deaths,75 ~ 80,-0 68(-1 1 to -0 25)

Sierra_Leone,Deaths,80 ~ 85,-0 69(-1 18 to -0 19)

Sierra_Leone,Deaths,85 ~ 90,-0 65(-1 35 to 0 06)

Sierra_Leone,Deaths,90 ~ 95,-0 56(-1 73 to 0 62)

Sierra_Leone,Deaths,20 ~ 25,1 13(-2 69 to 5 1)

Sierra_Leone,Deaths,25 ~ 30,0 97(-1 36 to 3 35)

Sierra_Leone,Deaths,30 ~ 35,0 68(-1 21 to 2 6)

Sierra_Leone,Deaths,35 ~ 40,0 35(-1 27 to 1 99)

Sierra_Leone,Deaths,40 ~ 45,0 07(-1 26 to 1 42)

Sierra_Leone,Deaths,45 ~ 50,-0 16(-1 21 to 0 9)

Sierra_Leone,Deaths,50 ~ 55,-0 29(-1 14 to 0 56)

Sierra_Leone,Deaths,55 ~ 60,-0 37(-1 06 to 0 33)

Sierra_Leone,Deaths,60 ~ 65,-0 44(-1 01 to 0 13)

Sierra_Leone,Deaths,65 ~ 70,-0 55(-1 03 to -0 07)

Sierra_Leone,Deaths,70 ~ 75,-0 66(-1 09 to -0 24)

Sierra_Leone,Deaths,75 ~ 80,-0 68(-1 1 to -0 25)

Sierra_Leone,Deaths,80 ~ 85,-0 69(-1 18 to -0 19)

Sierra_Leone,Deaths,85 ~ 90,-0 65(-1 35 to 0 06)

Sierra_Leone,Deaths,90 ~ 95,-0 56(-1 73 to 0 62)

Sierra_Leone,Deaths,20 ~ 25,1 13(-2 69 to 5 1)

Sierra_Leone,Deaths,25 ~ 30,0 97(-1 36 to 3 35)

Sierra_Leone,Deaths,30 ~ 35,0 68(-1 21 to 2 6)

Sierra_Leone,Deaths,35 ~ 40,0 35(-1 27 to 1 99)

Sierra_Leone,Deaths,40 ~ 45,0 07(-1 26 to 1 42)

Sierra_Leone,Deaths,45 ~ 50,-0 16(-1 21 to 0 9)

Sierra_Leone,Deaths,50 ~ 55,-0 29(-1 14 to 0 56)

Sierra_Leone,Deaths,55 ~ 60,-0 37(-1 06 to 0 33)

Sierra_Leone,Deaths,60 ~ 65,-0 44(-1 01 to 0 13)

Sierra_Leone,Deaths,65 ~ 70,-0 55(-1 03 to -0 07)

Sierra_Leone,Deaths,70 ~ 75,-0 66(-1 09 to -0 24)

Sierra_Leone,Deaths,75 ~ 80,-0 68(-1 1 to -0 25)

Sierra_Leone,Deaths,80 ~ 85,-0 69(-1 18 to -0 19)

Sierra_Leone,Deaths,85 ~ 90,-0 65(-1 35 to 0 06)

Sierra_Leone,Deaths,90 ~ 95,-0 56(-1 73 to 0 62)

Sierra_Leone,DALYs,20 ~ 25,0 44(0 17 to 0 71)

Sierra_Leone,DALYs,25 ~ 30,0 43(0 24 to 0 63)

Sierra_Leone,DALYs,30 ~ 35,0 32(0 15 to 0 49)

Sierra_Leone,DALYs,35 ~ 40,0 18(0 02 to 0 34)

Sierra_Leone,DALYs,40 ~ 45,0 07(-0 07 to 0 21)

Sierra_Leone,DALYs,45 ~ 50,-0 03(-0 16 to 0 09)

Sierra_Leone,DALYs,50 ~ 55,-0 12(-0 23 to 0)

Sierra_Leone,DALYs,55 ~ 60,-0 18(-0 29 to -0 08)

Sierra_Leone,DALYs,60 ~ 65,-0 26(-0 35 to -0 17)

Sierra_Leone,DALYs,65 ~ 70,-0 36(-0 45 to -0 28)

Sierra_Leone,DALYs,70 ~ 75,-0 48(-0 56 to -0 39)

Sierra_Leone,DALYs,75 ~ 80,-0 5(-0 6 to -0 4)

Sierra_Leone,DALYs,80 ~ 85,-0 5(-0 63 to -0 38)

Sierra_Leone,DALYs,85 ~ 90,-0 47(-0 67 to -0 27)

Sierra_Leone,DALYs,90 ~ 95,-0 39(-0 75 to -0 02)

Sierra_Leone,DALYs,20 ~ 25,0 44(0 17 to 0 71)

Sierra_Leone,DALYs,25 ~ 30,0 43(0 24 to 0 63)

Sierra_Leone,DALYs,30 ~ 35,0 32(0 15 to 0 49)

Sierra_Leone,DALYs,35 ~ 40,0 18(0 02 to 0 34)

Sierra_Leone,DALYs,40 ~ 45,0 07(-0 07 to 0 21)

Sierra_Leone,DALYs,45 ~ 50,-0 03(-0 16 to 0 09)

Sierra_Leone,DALYs,50 ~ 55,-0 12(-0 23 to 0)

Sierra_Leone,DALYs,55 ~ 60,-0 18(-0 29 to -0 08)

Sierra_Leone,DALYs,60 ~ 65,-0 26(-0 35 to -0 17)

Sierra_Leone,DALYs,65 ~ 70,-0 36(-0 45 to -0 28)

Sierra_Leone,DALYs,70 ~ 75,-0 48(-0 56 to -0 39)

Sierra_Leone,DALYs,75 ~ 80,-0 5(-0 6 to -0 4)

Sierra_Leone,DALYs,80 ~ 85,-0 5(-0 63 to -0 38)

Sierra_Leone,DALYs,85 ~ 90,-0 47(-0 67 to -0 27)

Sierra_Leone,DALYs,90 ~ 95,-0 39(-0 75 to -0 02)

Sierra_Leone,DALYs,20 ~ 25,0 44(0 17 to 0 71)

Sierra_Leone,DALYs,25 ~ 30,0 43(0 24 to 0 63)

Sierra_Leone,DALYs,30 ~ 35,0 32(0 15 to 0 49)

Sierra_Leone,DALYs,35 ~ 40,0 18(0 02 to 0 34)

Sierra_Leone,DALYs,40 ~ 45,0 07(-0 07 to 0 21)

Sierra_Leone,DALYs,45 ~ 50,-0 03(-0 16 to 0 09)

Sierra_Leone,DALYs,50 ~ 55,-0 12(-0 23 to 0)

Sierra_Leone,DALYs,55 ~ 60,-0 18(-0 29 to -0 08)

Sierra_Leone,DALYs,60 ~ 65,-0 26(-0 35 to -0 17)

Sierra_Leone,DALYs,65 ~ 70,-0 36(-0 45 to -0 28)

Sierra_Leone,DALYs,70 ~ 75,-0 48(-0 56 to -0 39)

Sierra_Leone,DALYs,75 ~ 80,-0 5(-0 6 to -0 4)

Sierra_Leone,DALYs,80 ~ 85,-0 5(-0 63 to -0 38)

Sierra_Leone,DALYs,85 ~ 90,-0 47(-0 67 to -0 27)

Sierra_Leone,DALYs,90 ~ 95,-0 39(-0 75 to -0 02)

Sierra_Leone,DALYs,20 ~ 25,0 44(0 17 to 0 71)

Sierra_Leone,DALYs,25 ~ 30,0 43(0 24 to 0 63)

Sierra_Leone,DALYs,30 ~ 35,0 32(0 15 to 0 49)

Sierra_Leone,DALYs,35 ~ 40,0 18(0 02 to 0 34)

Sierra_Leone,DALYs,40 ~ 45,0 07(-0 07 to 0 21)

Sierra_Leone,DALYs,45 ~ 50,-0 03(-0 16 to 0 09)

Sierra_Leone,DALYs,50 ~ 55,-0 12(-0 23 to 0)

Sierra_Leone,DALYs,55 ~ 60,-0 18(-0 29 to -0 08)

Sierra_Leone,DALYs,60 ~ 65,-0 26(-0 35 to -0 17)

Sierra_Leone,DALYs,65 ~ 70,-0 36(-0 45 to -0 28)

Sierra_Leone,DALYs,70 ~ 75,-0 48(-0 56 to -0 39)

Sierra_Leone,DALYs,75 ~ 80,-0 5(-0 6 to -0 4)

Sierra_Leone,DALYs,80 ~ 85,-0 5(-0 63 to -0 38)

Sierra_Leone,DALYs,85 ~ 90,-0 47(-0 67 to -0 27)

Sierra_Leone,DALYs,90 ~ 95,-0 39(-0 75 to -0 02)

Tunisia,Prevalence,20 ~ 25,-0 03(-0 14 to 0 08)

Tunisia,Prevalence,25 ~ 30,-0 02(-0 1 to 0 05)

Tunisia,Prevalence,30 ~ 35,0 04(-0 03 to 0 1)

Tunisia,Prevalence,35 ~ 40,0 14(0 09 to 0 2)

Tunisia,Prevalence,40 ~ 45,0 28(0 23 to 0 33)

Tunisia,Prevalence,45 ~ 50,0 43(0 38 to 0 47)

Tunisia,Prevalence,50 ~ 55,0 54(0 5 to 0 58)

Tunisia,Prevalence,55 ~ 60,0 62(0 58 to 0 66)

Tunisia,Prevalence,60 ~ 65,0 68(0 64 to 0 71)

Tunisia,Prevalence,65 ~ 70,0 73(0 69 to 0 76)

Tunisia,Prevalence,70 ~ 75,0 78(0 74 to 0 81)

Tunisia,Prevalence,75 ~ 80,0 82(0 78 to 0 86)

Tunisia,Prevalence,80 ~ 85,0 87(0 82 to 0 92)

Tunisia,Prevalence,85 ~ 90,0 94(0 86 to 1 02)

Tunisia,Prevalence,90 ~ 95,1 02(0 83 to 1 21)

Tunisia,Prevalence,20 ~ 25,-0 03(-0 14 to 0 08)

Tunisia,Prevalence,25 ~ 30,-0 02(-0 1 to 0 05)

Tunisia,Prevalence,30 ~ 35,0 04(-0 03 to 0 1)

Tunisia,Prevalence,35 ~ 40,0 14(0 09 to 0 2)

Tunisia,Prevalence,40 ~ 45,0 28(0 23 to 0 33)

Tunisia,Prevalence,45 ~ 50,0 43(0 38 to 0 47)

Tunisia,Prevalence,50 ~ 55,0 54(0 5 to 0 58)

Tunisia,Prevalence,55 ~ 60,0 62(0 58 to 0 66)

Tunisia,Prevalence,60 ~ 65,0 68(0 64 to 0 71)

Tunisia,Prevalence,65 ~ 70,0 73(0 69 to 0 76)

Tunisia,Prevalence,70 ~ 75,0 78(0 74 to 0 81)

Tunisia,Prevalence,75 ~ 80,0 82(0 78 to 0 86)

Tunisia,Prevalence,80 ~ 85,0 87(0 82 to 0 92)

Tunisia,Prevalence,85 ~ 90,0 94(0 86 to 1 02)

Tunisia,Prevalence,90 ~ 95,1 02(0 83 to 1 21)

Tunisia,Prevalence,20 ~ 25,-0 03(-0 14 to 0 08)

Tunisia,Prevalence,25 ~ 30,-0 02(-0 1 to 0 05)

Tunisia,Prevalence,30 ~ 35,0 04(-0 03 to 0 1)

Tunisia,Prevalence,35 ~ 40,0 14(0 09 to 0 2)

Tunisia,Prevalence,40 ~ 45,0 28(0 23 to 0 33)

Tunisia,Prevalence,45 ~ 50,0 43(0 38 to 0 47)

Tunisia,Prevalence,50 ~ 55,0 54(0 5 to 0 58)

Tunisia,Prevalence,55 ~ 60,0 62(0 58 to 0 66)

Tunisia,Prevalence,60 ~ 65,0 68(0 64 to 0 71)

Tunisia,Prevalence,65 ~ 70,0 73(0 69 to 0 76)

Tunisia,Prevalence,70 ~ 75,0 78(0 74 to 0 81)

Tunisia,Prevalence,75 ~ 80,0 82(0 78 to 0 86)

Tunisia,Prevalence,80 ~ 85,0 87(0 82 to 0 92)

Tunisia,Prevalence,85 ~ 90,0 94(0 86 to 1 02)

Tunisia,Prevalence,90 ~ 95,1 02(0 83 to 1 21)

Tunisia,Prevalence,20 ~ 25,-0 03(-0 14 to 0 08)

Tunisia,Prevalence,25 ~ 30,-0 02(-0 1 to 0 05)

Tunisia,Prevalence,30 ~ 35,0 04(-0 03 to 0 1)

Tunisia,Prevalence,35 ~ 40,0 14(0 09 to 0 2)

Tunisia,Prevalence,40 ~ 45,0 28(0 23 to 0 33)

Tunisia,Prevalence,45 ~ 50,0 43(0 38 to 0 47)

Tunisia,Prevalence,50 ~ 55,0 54(0 5 to 0 58)

Tunisia,Prevalence,55 ~ 60,0 62(0 58 to 0 66)

Tunisia,Prevalence,60 ~ 65,0 68(0 64 to 0 71)

Tunisia,Prevalence,65 ~ 70,0 73(0 69 to 0 76)

Tunisia,Prevalence,70 ~ 75,0 78(0 74 to 0 81)

Tunisia,Prevalence,75 ~ 80,0 82(0 78 to 0 86)

Tunisia,Prevalence,80 ~ 85,0 87(0 82 to 0 92)

Tunisia,Prevalence,85 ~ 90,0 94(0 86 to 1 02)

Tunisia,Prevalence,90 ~ 95,1 02(0 83 to 1 21)

Tunisia,Deaths,20 ~ 25,-0 78(-3 93 to 2 49)

Tunisia,Deaths,25 ~ 30,-0 8(-2 89 to 1 32)

Tunisia,Deaths,30 ~ 35,-0 75(-2 28 to 0 8)

Tunisia,Deaths,35 ~ 40,-0 67(-1 89 to 0 57)

Tunisia,Deaths,40 ~ 45,-0 65(-1 65 to 0 36)

Tunisia,Deaths,45 ~ 50,-0 65(-1 47 to 0 18)

Tunisia,Deaths,50 ~ 55,-0 63(-1 29 to 0 03)

Tunisia,Deaths,55 ~ 60,-0 64(-1 16 to -0 12)

Tunisia,Deaths,60 ~ 65,-0 67(-1 09 to -0 25)

Tunisia,Deaths,65 ~ 70,-0 69(-1 05 to -0 32)

Tunisia,Deaths,70 ~ 75,-0 63(-0 95 to -0 3)

Tunisia,Deaths,75 ~ 80,-0 5(-0 8 to -0 19)

Tunisia,Deaths,80 ~ 85,-0 38(-0 7 to -0 07)

Tunisia,Deaths,85 ~ 90,-0 26(-0 69 to 0 18)

Tunisia,Deaths,90 ~ 95,0 03(-0 91 to 0 99)

Tunisia,Deaths,20 ~ 25,-0 78(-3 93 to 2 49)

Tunisia,Deaths,25 ~ 30,-0 8(-2 89 to 1 32)

Tunisia,Deaths,30 ~ 35,-0 75(-2 28 to 0 8)

Tunisia,Deaths,35 ~ 40,-0 67(-1 89 to 0 57)

Tunisia,Deaths,40 ~ 45,-0 65(-1 65 to 0 36)

Tunisia,Deaths,45 ~ 50,-0 65(-1 47 to 0 18)

Tunisia,Deaths,50 ~ 55,-0 63(-1 29 to 0 03)

Tunisia,Deaths,55 ~ 60,-0 64(-1 16 to -0 12)

Tunisia,Deaths,60 ~ 65,-0 67(-1 09 to -0 25)

Tunisia,Deaths,65 ~ 70,-0 69(-1 05 to -0 32)

Tunisia,Deaths,70 ~ 75,-0 63(-0 95 to -0 3)

Tunisia,Deaths,75 ~ 80,-0 5(-0 8 to -0 19)

Tunisia,Deaths,80 ~ 85,-0 38(-0 7 to -0 07)

Tunisia,Deaths,85 ~ 90,-0 26(-0 69 to 0 18)

Tunisia,Deaths,90 ~ 95,0 03(-0 91 to 0 99)

Tunisia,Deaths,20 ~ 25,-0 78(-3 93 to 2 49)

Tunisia,Deaths,25 ~ 30,-0 8(-2 89 to 1 32)

Tunisia,Deaths,30 ~ 35,-0 75(-2 28 to 0 8)

Tunisia,Deaths,35 ~ 40,-0 67(-1 89 to 0 57)

Tunisia,Deaths,40 ~ 45,-0 65(-1 65 to 0 36)

Tunisia,Deaths,45 ~ 50,-0 65(-1 47 to 0 18)

Tunisia,Deaths,50 ~ 55,-0 63(-1 29 to 0 03)

Tunisia,Deaths,55 ~ 60,-0 64(-1 16 to -0 12)

Tunisia,Deaths,60 ~ 65,-0 67(-1 09 to -0 25)

Tunisia,Deaths,65 ~ 70,-0 69(-1 05 to -0 32)

Tunisia,Deaths,70 ~ 75,-0 63(-0 95 to -0 3)

Tunisia,Deaths,75 ~ 80,-0 5(-0 8 to -0 19)

Tunisia,Deaths,80 ~ 85,-0 38(-0 7 to -0 07)

Tunisia,Deaths,85 ~ 90,-0 26(-0 69 to 0 18)

Tunisia,Deaths,90 ~ 95,0 03(-0 91 to 0 99)

Tunisia,Deaths,20 ~ 25,-0 78(-3 93 to 2 49)

Tunisia,Deaths,25 ~ 30,-0 8(-2 89 to 1 32)

Tunisia,Deaths,30 ~ 35,-0 75(-2 28 to 0 8)

Tunisia,Deaths,35 ~ 40,-0 67(-1 89 to 0 57)

Tunisia,Deaths,40 ~ 45,-0 65(-1 65 to 0 36)

Tunisia,Deaths,45 ~ 50,-0 65(-1 47 to 0 18)

Tunisia,Deaths,50 ~ 55,-0 63(-1 29 to 0 03)

Tunisia,Deaths,55 ~ 60,-0 64(-1 16 to -0 12)

Tunisia,Deaths,60 ~ 65,-0 67(-1 09 to -0 25)

Tunisia,Deaths,65 ~ 70,-0 69(-1 05 to -0 32)

Tunisia,Deaths,70 ~ 75,-0 63(-0 95 to -0 3)

Tunisia,Deaths,75 ~ 80,-0 5(-0 8 to -0 19)

Tunisia,Deaths,80 ~ 85,-0 38(-0 7 to -0 07)

Tunisia,Deaths,85 ~ 90,-0 26(-0 69 to 0 18)

Tunisia,Deaths,90 ~ 95,0 03(-0 91 to 0 99)

Tunisia,DALYs,20 ~ 25,-0 33(-0 59 to -0 07)

Tunisia,DALYs,25 ~ 30,-0 35(-0 53 to -0 18)

Tunisia,DALYs,30 ~ 35,-0 31(-0 45 to -0 18)

Tunisia,DALYs,35 ~ 40,-0 24(-0 36 to -0 12)

Tunisia,DALYs,40 ~ 45,-0 18(-0 29 to -0 08)

Tunisia,DALYs,45 ~ 50,-0 13(-0 22 to -0 04)

Tunisia,DALYs,50 ~ 55,-0 11(-0 19 to -0 03)

Tunisia,DALYs,55 ~ 60,-0 13(-0 2 to -0 06)

Tunisia,DALYs,60 ~ 65,-0 19(-0 25 to -0 12)

Tunisia,DALYs,65 ~ 70,-0 22(-0 28 to -0 16)

Tunisia,DALYs,70 ~ 75,-0 21(-0 27 to -0 15)

Tunisia,DALYs,75 ~ 80,-0 15(-0 21 to -0 08)

Tunisia,DALYs,80 ~ 85,-0 09(-0 17 to -0 01)

Tunisia,DALYs,85 ~ 90,-0 02(-0 14 to 0 1)

Tunisia,DALYs,90 ~ 95,0 2(-0 08 to 0 49)

Tunisia,DALYs,20 ~ 25,-0 33(-0 59 to -0 07)

Tunisia,DALYs,25 ~ 30,-0 35(-0 53 to -0 18)

Tunisia,DALYs,30 ~ 35,-0 31(-0 45 to -0 18)

Tunisia,DALYs,35 ~ 40,-0 24(-0 36 to -0 12)

Tunisia,DALYs,40 ~ 45,-0 18(-0 29 to -0 08)

Tunisia,DALYs,45 ~ 50,-0 13(-0 22 to -0 04)

Tunisia,DALYs,50 ~ 55,-0 11(-0 19 to -0 03)

Tunisia,DALYs,55 ~ 60,-0 13(-0 2 to -0 06)

Tunisia,DALYs,60 ~ 65,-0 19(-0 25 to -0 12)

Tunisia,DALYs,65 ~ 70,-0 22(-0 28 to -0 16)

Tunisia,DALYs,70 ~ 75,-0 21(-0 27 to -0 15)

Tunisia,DALYs,75 ~ 80,-0 15(-0 21 to -0 08)

Tunisia,DALYs,80 ~ 85,-0 09(-0 17 to -0 01)

Tunisia,DALYs,85 ~ 90,-0 02(-0 14 to 0 1)

Tunisia,DALYs,90 ~ 95,0 2(-0 08 to 0 49)

Tunisia,DALYs,20 ~ 25,-0 33(-0 59 to -0 07)

Tunisia,DALYs,25 ~ 30,-0 35(-0 53 to -0 18)

Tunisia,DALYs,30 ~ 35,-0 31(-0 45 to -0 18)

Tunisia,DALYs,35 ~ 40,-0 24(-0 36 to -0 12)

Tunisia,DALYs,40 ~ 45,-0 18(-0 29 to -0 08)

Tunisia,DALYs,45 ~ 50,-0 13(-0 22 to -0 04)

Tunisia,DALYs,50 ~ 55,-0 11(-0 19 to -0 03)

Tunisia,DALYs,55 ~ 60,-0 13(-0 2 to -0 06)

Tunisia,DALYs,60 ~ 65,-0 19(-0 25 to -0 12)

Tunisia,DALYs,65 ~ 70,-0 22(-0 28 to -0 16)

Tunisia,DALYs,70 ~ 75,-0 21(-0 27 to -0 15)

Tunisia,DALYs,75 ~ 80,-0 15(-0 21 to -0 08)

Tunisia,DALYs,80 ~ 85,-0 09(-0 17 to -0 01)

Tunisia,DALYs,85 ~ 90,-0 02(-0 14 to 0 1)

Tunisia,DALYs,90 ~ 95,0 2(-0 08 to 0 49)

Tunisia,DALYs,20 ~ 25,-0 33(-0 59 to -0 07)

Tunisia,DALYs,25 ~ 30,-0 35(-0 53 to -0 18)

Tunisia,DALYs,30 ~ 35,-0 31(-0 45 to -0 18)

Tunisia,DALYs,35 ~ 40,-0 24(-0 36 to -0 12)

Tunisia,DALYs,40 ~ 45,-0 18(-0 29 to -0 08)

Tunisia,DALYs,45 ~ 50,-0 13(-0 22 to -0 04)

Tunisia,DALYs,50 ~ 55,-0 11(-0 19 to -0 03)

Tunisia,DALYs,55 ~ 60,-0 13(-0 2 to -0 06)

Tunisia,DALYs,60 ~ 65,-0 19(-0 25 to -0 12)

Tunisia,DALYs,65 ~ 70,-0 22(-0 28 to -0 16)

Tunisia,DALYs,70 ~ 75,-0 21(-0 27 to -0 15)

Tunisia,DALYs,75 ~ 80,-0 15(-0 21 to -0 08)

Tunisia,DALYs,80 ~ 85,-0 09(-0 17 to -0 01)

Tunisia,DALYs,85 ~ 90,-0 02(-0 14 to 0 1)

Tunisia,DALYs,90 ~ 95,0 2(-0 08 to 0 49)

Belgium,Prevalence,20 ~ 25,-0 17(-0 33 to -0 01)

Belgium,Prevalence,25 ~ 30,-0 18(-0 29 to -0 07)

Belgium,Prevalence,30 ~ 35,-0 2(-0 28 to -0 11)

Belgium,Prevalence,35 ~ 40,-0 22(-0 29 to -0 15)

Belgium,Prevalence,40 ~ 45,-0 24(-0 3 to -0 18)

Belgium,Prevalence,45 ~ 50,-0 25(-0 3 to -0 2)

Belgium,Prevalence,50 ~ 55,-0 23(-0 27 to -0 19)

Belgium,Prevalence,55 ~ 60,-0 2(-0 23 to -0 16)

Belgium,Prevalence,60 ~ 65,-0 17(-0 2 to -0 14)

Belgium,Prevalence,65 ~ 70,-0 15(-0 17 to -0 12)

Belgium,Prevalence,70 ~ 75,-0 11(-0 14 to -0 09)

Belgium,Prevalence,75 ~ 80,-0 03(-0 05 to 0)

Belgium,Prevalence,80 ~ 85,0 11(0 08 to 0 14)

Belgium,Prevalence,85 ~ 90,0 27(0 23 to 0 31)

Belgium,Prevalence,90 ~ 95,0 42(0 36 to 0 48)

Belgium,Prevalence,20 ~ 25,-0 17(-0 33 to -0 01)

Belgium,Prevalence,25 ~ 30,-0 18(-0 29 to -0 07)

Belgium,Prevalence,30 ~ 35,-0 2(-0 28 to -0 11)

Belgium,Prevalence,35 ~ 40,-0 22(-0 29 to -0 15)

Belgium,Prevalence,40 ~ 45,-0 24(-0 3 to -0 18)

Belgium,Prevalence,45 ~ 50,-0 25(-0 3 to -0 2)

Belgium,Prevalence,50 ~ 55,-0 23(-0 27 to -0 19)

Belgium,Prevalence,55 ~ 60,-0 2(-0 23 to -0 16)

Belgium,Prevalence,60 ~ 65,-0 17(-0 2 to -0 14)

Belgium,Prevalence,65 ~ 70,-0 15(-0 17 to -0 12)

Belgium,Prevalence,70 ~ 75,-0 11(-0 14 to -0 09)

Belgium,Prevalence,75 ~ 80,-0 03(-0 05 to 0)

Belgium,Prevalence,80 ~ 85,0 11(0 08 to 0 14)

Belgium,Prevalence,85 ~ 90,0 27(0 23 to 0 31)

Belgium,Prevalence,90 ~ 95,0 42(0 36 to 0 48)

Belgium,Prevalence,20 ~ 25,-0 17(-0 33 to -0 01)

Belgium,Prevalence,25 ~ 30,-0 18(-0 29 to -0 07)

Belgium,Prevalence,30 ~ 35,-0 2(-0 28 to -0 11)

Belgium,Prevalence,35 ~ 40,-0 22(-0 29 to -0 15)

Belgium,Prevalence,40 ~ 45,-0 24(-0 3 to -0 18)

Belgium,Prevalence,45 ~ 50,-0 25(-0 3 to -0 2)

Belgium,Prevalence,50 ~ 55,-0 23(-0 27 to -0 19)

Belgium,Prevalence,55 ~ 60,-0 2(-0 23 to -0 16)

Belgium,Prevalence,60 ~ 65,-0 17(-0 2 to -0 14)

Belgium,Prevalence,65 ~ 70,-0 15(-0 17 to -0 12)

Belgium,Prevalence,70 ~ 75,-0 11(-0 14 to -0 09)

Belgium,Prevalence,75 ~ 80,-0 03(-0 05 to 0)

Belgium,Prevalence,80 ~ 85,0 11(0 08 to 0 14)

Belgium,Prevalence,85 ~ 90,0 27(0 23 to 0 31)

Belgium,Prevalence,90 ~ 95,0 42(0 36 to 0 48)

Belgium,Prevalence,20 ~ 25,-0 17(-0 33 to -0 01)

Belgium,Prevalence,25 ~ 30,-0 18(-0 29 to -0 07)

Belgium,Prevalence,30 ~ 35,-0 2(-0 28 to -0 11)

Belgium,Prevalence,35 ~ 40,-0 22(-0 29 to -0 15)

Belgium,Prevalence,40 ~ 45,-0 24(-0 3 to -0 18)

Belgium,Prevalence,45 ~ 50,-0 25(-0 3 to -0 2)

Belgium,Prevalence,50 ~ 55,-0 23(-0 27 to -0 19)

Belgium,Prevalence,55 ~ 60,-0 2(-0 23 to -0 16)

Belgium,Prevalence,60 ~ 65,-0 17(-0 2 to -0 14)

Belgium,Prevalence,65 ~ 70,-0 15(-0 17 to -0 12)

Belgium,Prevalence,70 ~ 75,-0 11(-0 14 to -0 09)

Belgium,Prevalence,75 ~ 80,-0 03(-0 05 to 0)

Belgium,Prevalence,80 ~ 85,0 11(0 08 to 0 14)

Belgium,Prevalence,85 ~ 90,0 27(0 23 to 0 31)

Belgium,Prevalence,90 ~ 95,0 42(0 36 to 0 48)

Belgium,Deaths,20 ~ 25,-1 81(-6 89 to 3 55)

Belgium,Deaths,25 ~ 30,-1 66(-5 11 to 1 92)

Belgium,Deaths,30 ~ 35,-1 63(-4 18 to 0 99)

Belgium,Deaths,35 ~ 40,-1 72(-3 57 to 0 17)

Belgium,Deaths,40 ~ 45,-1 75(-3 02 to -0 46)

Belgium,Deaths,45 ~ 50,-1 61(-2 44 to -0 77)

Belgium,Deaths,50 ~ 55,-1 37(-1 91 to -0 84)

Belgium,Deaths,55 ~ 60,-1 22(-1 57 to -0 86)

Belgium,Deaths,60 ~ 65,-1 26(-1 51 to -1 01)

Belgium,Deaths,65 ~ 70,-1 51(-1 69 to -1 32)

Belgium,Deaths,70 ~ 75,-1 75(-1 9 to -1 59)

Belgium,Deaths,75 ~ 80,-2 03(-2 17 to -1 89)

Belgium,Deaths,80 ~ 85,-1 97(-2 1 to -1 83)

Belgium,Deaths,85 ~ 90,-1 63(-1 78 to -1 47)

Belgium,Deaths,90 ~ 95,-1 22(-1 47 to -0 97)

Belgium,Deaths,20 ~ 25,-1 81(-6 89 to 3 55)

Belgium,Deaths,25 ~ 30,-1 66(-5 11 to 1 92)

Belgium,Deaths,30 ~ 35,-1 63(-4 18 to 0 99)

Belgium,Deaths,35 ~ 40,-1 72(-3 57 to 0 17)

Belgium,Deaths,40 ~ 45,-1 75(-3 02 to -0 46)

Belgium,Deaths,45 ~ 50,-1 61(-2 44 to -0 77)

Belgium,Deaths,50 ~ 55,-1 37(-1 91 to -0 84)

Belgium,Deaths,55 ~ 60,-1 22(-1 57 to -0 86)

Belgium,Deaths,60 ~ 65,-1 26(-1 51 to -1 01)

Belgium,Deaths,65 ~ 70,-1 51(-1 69 to -1 32)

Belgium,Deaths,70 ~ 75,-1 75(-1 9 to -1 59)

Belgium,Deaths,75 ~ 80,-2 03(-2 17 to -1 89)

Belgium,Deaths,80 ~ 85,-1 97(-2 1 to -1 83)

Belgium,Deaths,85 ~ 90,-1 63(-1 78 to -1 47)

Belgium,Deaths,90 ~ 95,-1 22(-1 47 to -0 97)

Belgium,Deaths,20 ~ 25,-1 81(-6 89 to 3 55)

Belgium,Deaths,25 ~ 30,-1 66(-5 11 to 1 92)

Belgium,Deaths,30 ~ 35,-1 63(-4 18 to 0 99)

Belgium,Deaths,35 ~ 40,-1 72(-3 57 to 0 17)

Belgium,Deaths,40 ~ 45,-1 75(-3 02 to -0 46)

Belgium,Deaths,45 ~ 50,-1 61(-2 44 to -0 77)

Belgium,Deaths,50 ~ 55,-1 37(-1 91 to -0 84)

Belgium,Deaths,55 ~ 60,-1 22(-1 57 to -0 86)

Belgium,Deaths,60 ~ 65,-1 26(-1 51 to -1 01)

Belgium,Deaths,65 ~ 70,-1 51(-1 69 to -1 32)

Belgium,Deaths,70 ~ 75,-1 75(-1 9 to -1 59)

Belgium,Deaths,75 ~ 80,-2 03(-2 17 to -1 89)

Belgium,Deaths,80 ~ 85,-1 97(-2 1 to -1 83)

Belgium,Deaths,85 ~ 90,-1 63(-1 78 to -1 47)

Belgium,Deaths,90 ~ 95,-1 22(-1 47 to -0 97)

Belgium,Deaths,20 ~ 25,-1 81(-6 89 to 3 55)

Belgium,Deaths,25 ~ 30,-1 66(-5 11 to 1 92)

Belgium,Deaths,30 ~ 35,-1 63(-4 18 to 0 99)

Belgium,Deaths,35 ~ 40,-1 72(-3 57 to 0 17)

Belgium,Deaths,40 ~ 45,-1 75(-3 02 to -0 46)

Belgium,Deaths,45 ~ 50,-1 61(-2 44 to -0 77)

Belgium,Deaths,50 ~ 55,-1 37(-1 91 to -0 84)

Belgium,Deaths,55 ~ 60,-1 22(-1 57 to -0 86)

Belgium,Deaths,60 ~ 65,-1 26(-1 51 to -1 01)

Belgium,Deaths,65 ~ 70,-1 51(-1 69 to -1 32)

Belgium,Deaths,70 ~ 75,-1 75(-1 9 to -1 59)

Belgium,Deaths,75 ~ 80,-2 03(-2 17 to -1 89)

Belgium,Deaths,80 ~ 85,-1 97(-2 1 to -1 83)

Belgium,Deaths,85 ~ 90,-1 63(-1 78 to -1 47)

Belgium,Deaths,90 ~ 95,-1 22(-1 47 to -0 97)

Belgium,DALYs,20 ~ 25,-0 8(-1 8 to 0 2)

Belgium,DALYs,25 ~ 30,-0 75(-1 43 to -0 07)

Belgium,DALYs,30 ~ 35,-0 8(-1 33 to -0 28)

Belgium,DALYs,35 ~ 40,-0 98(-1 39 to -0 56)

Belgium,DALYs,40 ~ 45,-1 1(-1 42 to -0 78)

Belgium,DALYs,45 ~ 50,-1 1(-1 33 to -0 86)

Belgium,DALYs,50 ~ 55,-0 99(-1 16 to -0 82)

Belgium,DALYs,55 ~ 60,-0 93(-1 06 to -0 8)

Belgium,DALYs,60 ~ 65,-1 03(-1 13 to -0 92)

Belgium,DALYs,65 ~ 70,-1 28(-1 36 to -1 19)

Belgium,DALYs,70 ~ 75,-1 53(-1 61 to -1 45)

Belgium,DALYs,75 ~ 80,-1 76(-1 84 to -1 68)

Belgium,DALYs,80 ~ 85,-1 7(-1 79 to -1 61)

Belgium,DALYs,85 ~ 90,-1 37(-1 49 to -1 26)

Belgium,DALYs,90 ~ 95,-0 99(-1 18 to -0 8)

Belgium,DALYs,20 ~ 25,-0 8(-1 8 to 0 2)

Belgium,DALYs,25 ~ 30,-0 75(-1 43 to -0 07)

Belgium,DALYs,30 ~ 35,-0 8(-1 33 to -0 28)

Belgium,DALYs,35 ~ 40,-0 98(-1 39 to -0 56)

Belgium,DALYs,40 ~ 45,-1 1(-1 42 to -0 78)

Belgium,DALYs,45 ~ 50,-1 1(-1 33 to -0 86)

Belgium,DALYs,50 ~ 55,-0 99(-1 16 to -0 82)

Belgium,DALYs,55 ~ 60,-0 93(-1 06 to -0 8)

Belgium,DALYs,60 ~ 65,-1 03(-1 13 to -0 92)

Belgium,DALYs,65 ~ 70,-1 28(-1 36 to -1 19)

Belgium,DALYs,70 ~ 75,-1 53(-1 61 to -1 45)

Belgium,DALYs,75 ~ 80,-1 76(-1 84 to -1 68)

Belgium,DALYs,80 ~ 85,-1 7(-1 79 to -1 61)

Belgium,DALYs,85 ~ 90,-1 37(-1 49 to -1 26)

Belgium,DALYs,90 ~ 95,-0 99(-1 18 to -0 8)

Belgium,DALYs,20 ~ 25,-0 8(-1 8 to 0 2)

Belgium,DALYs,25 ~ 30,-0 75(-1 43 to -0 07)

Belgium,DALYs,30 ~ 35,-0 8(-1 33 to -0 28)

Belgium,DALYs,35 ~ 40,-0 98(-1 39 to -0 56)

Belgium,DALYs,40 ~ 45,-1 1(-1 42 to -0 78)

Belgium,DALYs,45 ~ 50,-1 1(-1 33 to -0 86)

Belgium,DALYs,50 ~ 55,-0 99(-1 16 to -0 82)

Belgium,DALYs,55 ~ 60,-0 93(-1 06 to -0 8)

Belgium,DALYs,60 ~ 65,-1 03(-1 13 to -0 92)

Belgium,DALYs,65 ~ 70,-1 28(-1 36 to -1 19)

Belgium,DALYs,70 ~ 75,-1 53(-1 61 to -1 45)

Belgium,DALYs,75 ~ 80,-1 76(-1 84 to -1 68)

Belgium,DALYs,80 ~ 85,-1 7(-1 79 to -1 61)

Belgium,DALYs,85 ~ 90,-1 37(-1 49 to -1 26)

Belgium,DALYs,90 ~ 95,-0 99(-1 18 to -0 8)

Belgium,DALYs,20 ~ 25,-0 8(-1 8 to 0 2)

Belgium,DALYs,25 ~ 30,-0 75(-1 43 to -0 07)

Belgium,DALYs,30 ~ 35,-0 8(-1 33 to -0 28)

Belgium,DALYs,35 ~ 40,-0 98(-1 39 to -0 56)

Belgium,DALYs,40 ~ 45,-1 1(-1 42 to -0 78)

Belgium,DALYs,45 ~ 50,-1 1(-1 33 to -0 86)

Belgium,DALYs,50 ~ 55,-0 99(-1 16 to -0 82)

Belgium,DALYs,55 ~ 60,-0 93(-1 06 to -0 8)

Belgium,DALYs,60 ~ 65,-1 03(-1 13 to -0 92)

Belgium,DALYs,65 ~ 70,-1 28(-1 36 to -1 19)

Belgium,DALYs,70 ~ 75,-1 53(-1 61 to -1 45)

Belgium,DALYs,75 ~ 80,-1 76(-1 84 to -1 68)

Belgium,DALYs,80 ~ 85,-1 7(-1 79 to -1 61)

Belgium,DALYs,85 ~ 90,-1 37(-1 49 to -1 26)

Belgium,DALYs,90 ~ 95,-0 99(-1 18 to -0 8)

Luxembourg,Prevalence,20 ~ 25,-0 13(-0 55 to 0 31)

Luxembourg,Prevalence,25 ~ 30,-0 13(-0 42 to 0 16)

Luxembourg,Prevalence,30 ~ 35,-0 19(-0 42 to 0 04)

Luxembourg,Prevalence,35 ~ 40,-0 28(-0 47 to -0 09)

Luxembourg,Prevalence,40 ~ 45,-0 4(-0 56 to -0 23)

Luxembourg,Prevalence,45 ~ 50,-0 5(-0 64 to -0 36)

Luxembourg,Prevalence,50 ~ 55,-0 55(-0 67 to -0 43)

Luxembourg,Prevalence,55 ~ 60,-0 53(-0 63 to -0 43)

Luxembourg,Prevalence,60 ~ 65,-0 48(-0 57 to -0 39)

Luxembourg,Prevalence,65 ~ 70,-0 39(-0 48 to -0 3)

Luxembourg,Prevalence,70 ~ 75,-0 28(-0 36 to -0 19)

Luxembourg,Prevalence,75 ~ 80,-0 15(-0 24 to -0 06)

Luxembourg,Prevalence,80 ~ 85,0(-0 09 to 0 1)

Luxembourg,Prevalence,85 ~ 90,0 16(0 03 to 0 29)

Luxembourg,Prevalence,90 ~ 95,0 28(0 04 to 0 52)

Luxembourg,Prevalence,20 ~ 25,-0 13(-0 55 to 0 31)

Luxembourg,Prevalence,25 ~ 30,-0 13(-0 42 to 0 16)

Luxembourg,Prevalence,30 ~ 35,-0 19(-0 42 to 0 04)

Luxembourg,Prevalence,35 ~ 40,-0 28(-0 47 to -0 09)

Luxembourg,Prevalence,40 ~ 45,-0 4(-0 56 to -0 23)

Luxembourg,Prevalence,45 ~ 50,-0 5(-0 64 to -0 36)

Luxembourg,Prevalence,50 ~ 55,-0 55(-0 67 to -0 43)

Luxembourg,Prevalence,55 ~ 60,-0 53(-0 63 to -0 43)

Luxembourg,Prevalence,60 ~ 65,-0 48(-0 57 to -0 39)

Luxembourg,Prevalence,65 ~ 70,-0 39(-0 48 to -0 3)

Luxembourg,Prevalence,70 ~ 75,-0 28(-0 36 to -0 19)

Luxembourg,Prevalence,75 ~ 80,-0 15(-0 24 to -0 06)

Luxembourg,Prevalence,80 ~ 85,0(-0 09 to 0 1)

Luxembourg,Prevalence,85 ~ 90,0 16(0 03 to 0 29)

Luxembourg,Prevalence,90 ~ 95,0 28(0 04 to 0 52)

Luxembourg,Prevalence,20 ~ 25,-0 13(-0 55 to 0 31)

Luxembourg,Prevalence,25 ~ 30,-0 13(-0 42 to 0 16)

Luxembourg,Prevalence,30 ~ 35,-0 19(-0 42 to 0 04)

Luxembourg,Prevalence,35 ~ 40,-0 28(-0 47 to -0 09)

Luxembourg,Prevalence,40 ~ 45,-0 4(-0 56 to -0 23)

Luxembourg,Prevalence,45 ~ 50,-0 5(-0 64 to -0 36)

Luxembourg,Prevalence,50 ~ 55,-0 55(-0 67 to -0 43)

Luxembourg,Prevalence,55 ~ 60,-0 53(-0 63 to -0 43)

Luxembourg,Prevalence,60 ~ 65,-0 48(-0 57 to -0 39)

Luxembourg,Prevalence,65 ~ 70,-0 39(-0 48 to -0 3)

Luxembourg,Prevalence,70 ~ 75,-0 28(-0 36 to -0 19)

Luxembourg,Prevalence,75 ~ 80,-0 15(-0 24 to -0 06)

Luxembourg,Prevalence,80 ~ 85,0(-0 09 to 0 1)

Luxembourg,Prevalence,85 ~ 90,0 16(0 03 to 0 29)

Luxembourg,Prevalence,90 ~ 95,0 28(0 04 to 0 52)

Luxembourg,Prevalence,20 ~ 25,-0 13(-0 55 to 0 31)

Luxembourg,Prevalence,25 ~ 30,-0 13(-0 42 to 0 16)

Luxembourg,Prevalence,30 ~ 35,-0 19(-0 42 to 0 04)

Luxembourg,Prevalence,35 ~ 40,-0 28(-0 47 to -0 09)

Luxembourg,Prevalence,40 ~ 45,-0 4(-0 56 to -0 23)

Luxembourg,Prevalence,45 ~ 50,-0 5(-0 64 to -0 36)

Luxembourg,Prevalence,50 ~ 55,-0 55(-0 67 to -0 43)

Luxembourg,Prevalence,55 ~ 60,-0 53(-0 63 to -0 43)

Luxembourg,Prevalence,60 ~ 65,-0 48(-0 57 to -0 39)

Luxembourg,Prevalence,65 ~ 70,-0 39(-0 48 to -0 3)

Luxembourg,Prevalence,70 ~ 75,-0 28(-0 36 to -0 19)

Luxembourg,Prevalence,75 ~ 80,-0 15(-0 24 to -0 06)

Luxembourg,Prevalence,80 ~ 85,0(-0 09 to 0 1)

Luxembourg,Prevalence,85 ~ 90,0 16(0 03 to 0 29)

Luxembourg,Prevalence,90 ~ 95,0 28(0 04 to 0 52)

Luxembourg,Deaths,20 ~ 25,-2 96(-28 92 to 32 48)

Luxembourg,Deaths,25 ~ 30,-3 17(-22 03 to 20 26)

Luxembourg,Deaths,30 ~ 35,-2 92(-16 85 to 13 36)

Luxembourg,Deaths,35 ~ 40,-2 78(-12 8 to 8 39)

Luxembourg,Deaths,40 ~ 45,-2 82(-9 82 to 4 73)

Luxembourg,Deaths,45 ~ 50,-2 73(-7 32 to 2 08)

Luxembourg,Deaths,50 ~ 55,-2 54(-5 53 to 0 53)

Luxembourg,Deaths,55 ~ 60,-2 22(-4 23 to -0 16)

Luxembourg,Deaths,60 ~ 65,-2 07(-3 53 to -0 59)

Luxembourg,Deaths,65 ~ 70,-1 78(-2 9 to -0 65)

Luxembourg,Deaths,70 ~ 75,-1 51(-2 43 to -0 58)

Luxembourg,Deaths,75 ~ 80,-1 26(-2 07 to -0 45)

Luxembourg,Deaths,80 ~ 85,-0 83(-1 61 to -0 05)

Luxembourg,Deaths,85 ~ 90,-0 22(-1 17 to 0 73)

Luxembourg,Deaths,90 ~ 95,0 33(-1 3 to 1 98)

Luxembourg,Deaths,20 ~ 25,-2 96(-28 92 to 32 48)

Luxembourg,Deaths,25 ~ 30,-3 17(-22 03 to 20 26)

Luxembourg,Deaths,30 ~ 35,-2 92(-16 85 to 13 36)

Luxembourg,Deaths,35 ~ 40,-2 78(-12 8 to 8 39)

Luxembourg,Deaths,40 ~ 45,-2 82(-9 82 to 4 73)

Luxembourg,Deaths,45 ~ 50,-2 73(-7 32 to 2 08)

Luxembourg,Deaths,50 ~ 55,-2 54(-5 53 to 0 53)

Luxembourg,Deaths,55 ~ 60,-2 22(-4 23 to -0 16)

Luxembourg,Deaths,60 ~ 65,-2 07(-3 53 to -0 59)

Luxembourg,Deaths,65 ~ 70,-1 78(-2 9 to -0 65)

Luxembourg,Deaths,70 ~ 75,-1 51(-2 43 to -0 58)

Luxembourg,Deaths,75 ~ 80,-1 26(-2 07 to -0 45)

Luxembourg,Deaths,80 ~ 85,-0 83(-1 61 to -0 05)

Luxembourg,Deaths,85 ~ 90,-0 22(-1 17 to 0 73)

Luxembourg,Deaths,90 ~ 95,0 33(-1 3 to 1 98)

Luxembourg,Deaths,20 ~ 25,-2 96(-28 92 to 32 48)

Luxembourg,Deaths,25 ~ 30,-3 17(-22 03 to 20 26)

Luxembourg,Deaths,30 ~ 35,-2 92(-16 85 to 13 36)

Luxembourg,Deaths,35 ~ 40,-2 78(-12 8 to 8 39)

Luxembourg,Deaths,40 ~ 45,-2 82(-9 82 to 4 73)

Luxembourg,Deaths,45 ~ 50,-2 73(-7 32 to 2 08)

Luxembourg,Deaths,50 ~ 55,-2 54(-5 53 to 0 53)

Luxembourg,Deaths,55 ~ 60,-2 22(-4 23 to -0 16)

Luxembourg,Deaths,60 ~ 65,-2 07(-3 53 to -0 59)

Luxembourg,Deaths,65 ~ 70,-1 78(-2 9 to -0 65)

Luxembourg,Deaths,70 ~ 75,-1 51(-2 43 to -0 58)

Luxembourg,Deaths,75 ~ 80,-1 26(-2 07 to -0 45)

Luxembourg,Deaths,80 ~ 85,-0 83(-1 61 to -0 05)

Luxembourg,Deaths,85 ~ 90,-0 22(-1 17 to 0 73)

Luxembourg,Deaths,90 ~ 95,0 33(-1 3 to 1 98)

Luxembourg,Deaths,20 ~ 25,-2 96(-28 92 to 32 48)

Luxembourg,Deaths,25 ~ 30,-3 17(-22 03 to 20 26)

Luxembourg,Deaths,30 ~ 35,-2 92(-16 85 to 13 36)

Luxembourg,Deaths,35 ~ 40,-2 78(-12 8 to 8 39)

Luxembourg,Deaths,40 ~ 45,-2 82(-9 82 to 4 73)

Luxembourg,Deaths,45 ~ 50,-2 73(-7 32 to 2 08)

Luxembourg,Deaths,50 ~ 55,-2 54(-5 53 to 0 53)

Luxembourg,Deaths,55 ~ 60,-2 22(-4 23 to -0 16)

Luxembourg,Deaths,60 ~ 65,-2 07(-3 53 to -0 59)

Luxembourg,Deaths,65 ~ 70,-1 78(-2 9 to -0 65)

Luxembourg,Deaths,70 ~ 75,-1 51(-2 43 to -0 58)

Luxembourg,Deaths,75 ~ 80,-1 26(-2 07 to -0 45)

Luxembourg,Deaths,80 ~ 85,-0 83(-1 61 to -0 05)

Luxembourg,Deaths,85 ~ 90,-0 22(-1 17 to 0 73)

Luxembourg,Deaths,90 ~ 95,0 33(-1 3 to 1 98)

Luxembourg,DALYs,20 ~ 25,-0 89(-2 69 to 0 94)

Luxembourg,DALYs,25 ~ 30,-0 98(-2 22 to 0 27)

Luxembourg,DALYs,30 ~ 35,-1 03(-1 98 to -0 06)

Luxembourg,DALYs,35 ~ 40,-1 2(-1 97 to -0 42)

Luxembourg,DALYs,40 ~ 45,-1 46(-2 08 to -0 84)

Luxembourg,DALYs,45 ~ 50,-1 67(-2 15 to -1 19)

Luxembourg,DALYs,50 ~ 55,-1 72(-2 09 to -1 36)

Luxembourg,DALYs,55 ~ 60,-1 63(-1 91 to -1 35)

Luxembourg,DALYs,60 ~ 65,-1 58(-1 81 to -1 35)

Luxembourg,DALYs,65 ~ 70,-1 4(-1 6 to -1 2)

Luxembourg,DALYs,70 ~ 75,-1 24(-1 43 to -1 06)

Luxembourg,DALYs,75 ~ 80,-1 06(-1 24 to -0 88)

Luxembourg,DALYs,80 ~ 85,-0 72(-0 92 to -0 52)

Luxembourg,DALYs,85 ~ 90,-0 24(-0 51 to 0 03)

Luxembourg,DALYs,90 ~ 95,0 21(-0 29 to 0 7)

Luxembourg,DALYs,20 ~ 25,-0 89(-2 69 to 0 94)

Luxembourg,DALYs,25 ~ 30,-0 98(-2 22 to 0 27)

Luxembourg,DALYs,30 ~ 35,-1 03(-1 98 to -0 06)

Luxembourg,DALYs,35 ~ 40,-1 2(-1 97 to -0 42)

Luxembourg,DALYs,40 ~ 45,-1 46(-2 08 to -0 84)

Luxembourg,DALYs,45 ~ 50,-1 67(-2 15 to -1 19)

Luxembourg,DALYs,50 ~ 55,-1 72(-2 09 to -1 36)

Luxembourg,DALYs,55 ~ 60,-1 63(-1 91 to -1 35)

Luxembourg,DALYs,60 ~ 65,-1 58(-1 81 to -1 35)

Luxembourg,DALYs,65 ~ 70,-1 4(-1 6 to -1 2)

Luxembourg,DALYs,70 ~ 75,-1 24(-1 43 to -1 06)

Luxembourg,DALYs,75 ~ 80,-1 06(-1 24 to -0 88)

Luxembourg,DALYs,80 ~ 85,-0 72(-0 92 to -0 52)

Luxembourg,DALYs,85 ~ 90,-0 24(-0 51 to 0 03)

Luxembourg,DALYs,90 ~ 95,0 21(-0 29 to 0 7)

Luxembourg,DALYs,20 ~ 25,-0 89(-2 69 to 0 94)

Luxembourg,DALYs,25 ~ 30,-0 98(-2 22 to 0 27)

Luxembourg,DALYs,30 ~ 35,-1 03(-1 98 to -0 06)

Luxembourg,DALYs,35 ~ 40,-1 2(-1 97 to -0 42)

Luxembourg,DALYs,40 ~ 45,-1 46(-2 08 to -0 84)

Luxembourg,DALYs,45 ~ 50,-1 67(-2 15 to -1 19)

Luxembourg,DALYs,50 ~ 55,-1 72(-2 09 to -1 36)

Luxembourg,DALYs,55 ~ 60,-1 63(-1 91 to -1 35)

Luxembourg,DALYs,60 ~ 65,-1 58(-1 81 to -1 35)

Luxembourg,DALYs,65 ~ 70,-1 4(-1 6 to -1 2)

Luxembourg,DALYs,70 ~ 75,-1 24(-1 43 to -1 06)

Luxembourg,DALYs,75 ~ 80,-1 06(-1 24 to -0 88)

Luxembourg,DALYs,80 ~ 85,-0 72(-0 92 to -0 52)

Luxembourg,DALYs,85 ~ 90,-0 24(-0 51 to 0 03)

Luxembourg,DALYs,90 ~ 95,0 21(-0 29 to 0 7)

Luxembourg,DALYs,20 ~ 25,-0 89(-2 69 to 0 94)

Luxembourg,DALYs,25 ~ 30,-0 98(-2 22 to 0 27)

Luxembourg,DALYs,30 ~ 35,-1 03(-1 98 to -0 06)

Luxembourg,DALYs,35 ~ 40,-1 2(-1 97 to -0 42)

Luxembourg,DALYs,40 ~ 45,-1 46(-2 08 to -0 84)

Luxembourg,DALYs,45 ~ 50,-1 67(-2 15 to -1 19)

Luxembourg,DALYs,50 ~ 55,-1 72(-2 09 to -1 36)

Luxembourg,DALYs,55 ~ 60,-1 63(-1 91 to -1 35)

Luxembourg,DALYs,60 ~ 65,-1 58(-1 81 to -1 35)

Luxembourg,DALYs,65 ~ 70,-1 4(-1 6 to -1 2)

Luxembourg,DALYs,70 ~ 75,-1 24(-1 43 to -1 06)

Luxembourg,DALYs,75 ~ 80,-1 06(-1 24 to -0 88)

Luxembourg,DALYs,80 ~ 85,-0 72(-0 92 to -0 52)

Luxembourg,DALYs,85 ~ 90,-0 24(-0 51 to 0 03)

Luxembourg,DALYs,90 ~ 95,0 21(-0 29 to 0 7)

Libya,Prevalence,20 ~ 25,0 06(-0 06 to 0 17)

Libya,Prevalence,25 ~ 30,0 06(-0 02 to 0 15)

Libya,Prevalence,30 ~ 35,0 09(0 02 to 0 16)

Libya,Prevalence,35 ~ 40,0 14(0 08 to 0 2)

Libya,Prevalence,40 ~ 45,0 21(0 15 to 0 27)

Libya,Prevalence,45 ~ 50,0 29(0 23 to 0 34)

Libya,Prevalence,50 ~ 55,0 37(0 32 to 0 42)

Libya,Prevalence,55 ~ 60,0 47(0 42 to 0 52)

Libya,Prevalence,60 ~ 65,0 6(0 55 to 0 65)

Libya,Prevalence,65 ~ 70,0 74(0 68 to 0 79)

Libya,Prevalence,70 ~ 75,0 89(0 84 to 0 95)

Libya,Prevalence,75 ~ 80,1 06(0 99 to 1 12)

Libya,Prevalence,80 ~ 85,1 21(1 14 to 1 29)

Libya,Prevalence,85 ~ 90,1 35(1 25 to 1 46)

Libya,Prevalence,90 ~ 95,1 47(1 3 to 1 64)

Libya,Prevalence,20 ~ 25,0 06(-0 06 to 0 17)

Libya,Prevalence,25 ~ 30,0 06(-0 02 to 0 15)

Libya,Prevalence,30 ~ 35,0 09(0 02 to 0 16)

Libya,Prevalence,35 ~ 40,0 14(0 08 to 0 2)

Libya,Prevalence,40 ~ 45,0 21(0 15 to 0 27)

Libya,Prevalence,45 ~ 50,0 29(0 23 to 0 34)

Libya,Prevalence,50 ~ 55,0 37(0 32 to 0 42)

Libya,Prevalence,55 ~ 60,0 47(0 42 to 0 52)

Libya,Prevalence,60 ~ 65,0 6(0 55 to 0 65)

Libya,Prevalence,65 ~ 70,0 74(0 68 to 0 79)

Libya,Prevalence,70 ~ 75,0 89(0 84 to 0 95)

Libya,Prevalence,75 ~ 80,1 06(0 99 to 1 12)

Libya,Prevalence,80 ~ 85,1 21(1 14 to 1 29)

Libya,Prevalence,85 ~ 90,1 35(1 25 to 1 46)

Libya,Prevalence,90 ~ 95,1 47(1 3 to 1 64)

Libya,Prevalence,20 ~ 25,0 06(-0 06 to 0 17)

Libya,Prevalence,25 ~ 30,0 06(-0 02 to 0 15)

Libya,Prevalence,30 ~ 35,0 09(0 02 to 0 16)

Libya,Prevalence,35 ~ 40,0 14(0 08 to 0 2)

Libya,Prevalence,40 ~ 45,0 21(0 15 to 0 27)

Libya,Prevalence,45 ~ 50,0 29(0 23 to 0 34)

Libya,Prevalence,50 ~ 55,0 37(0 32 to 0 42)

Libya,Prevalence,55 ~ 60,0 47(0 42 to 0 52)

Libya,Prevalence,60 ~ 65,0 6(0 55 to 0 65)

Libya,Prevalence,65 ~ 70,0 74(0 68 to 0 79)

Libya,Prevalence,70 ~ 75,0 89(0 84 to 0 95)

Libya,Prevalence,75 ~ 80,1 06(0 99 to 1 12)

Libya,Prevalence,80 ~ 85,1 21(1 14 to 1 29)

Libya,Prevalence,85 ~ 90,1 35(1 25 to 1 46)

Libya,Prevalence,90 ~ 95,1 47(1 3 to 1 64)

Libya,Prevalence,20 ~ 25,0 06(-0 06 to 0 17)

Libya,Prevalence,25 ~ 30,0 06(-0 02 to 0 15)

Libya,Prevalence,30 ~ 35,0 09(0 02 to 0 16)

Libya,Prevalence,35 ~ 40,0 14(0 08 to 0 2)

Libya,Prevalence,40 ~ 45,0 21(0 15 to 0 27)

Libya,Prevalence,45 ~ 50,0 29(0 23 to 0 34)

Libya,Prevalence,50 ~ 55,0 37(0 32 to 0 42)

Libya,Prevalence,55 ~ 60,0 47(0 42 to 0 52)

Libya,Prevalence,60 ~ 65,0 6(0 55 to 0 65)

Libya,Prevalence,65 ~ 70,0 74(0 68 to 0 79)

Libya,Prevalence,70 ~ 75,0 89(0 84 to 0 95)

Libya,Prevalence,75 ~ 80,1 06(0 99 to 1 12)

Libya,Prevalence,80 ~ 85,1 21(1 14 to 1 29)

Libya,Prevalence,85 ~ 90,1 35(1 25 to 1 46)

Libya,Prevalence,90 ~ 95,1 47(1 3 to 1 64)

Libya,Deaths,20 ~ 25,0 41(-2 63 to 3 55)

Libya,Deaths,25 ~ 30,0 41(-1 72 to 2 59)

Libya,Deaths,30 ~ 35,0 43(-1 21 to 2 09)

Libya,Deaths,35 ~ 40,0 42(-0 92 to 1 77)

Libya,Deaths,40 ~ 45,0 42(-0 72 to 1 58)

Libya,Deaths,45 ~ 50,0 4(-0 57 to 1 39)

Libya,Deaths,50 ~ 55,0 35(-0 49 to 1 19)

Libya,Deaths,55 ~ 60,0 3(-0 42 to 1 03)

Libya,Deaths,60 ~ 65,0 21(-0 41 to 0 84)

Libya,Deaths,65 ~ 70,0 04(-0 53 to 0 61)

Libya,Deaths,70 ~ 75,0 03(-0 5 to 0 56)

Libya,Deaths,75 ~ 80,0 33(-0 19 to 0 85)

Libya,Deaths,80 ~ 85,0 83(0 26 to 1 42)

Libya,Deaths,85 ~ 90,1 12(0 4 to 1 84)

Libya,Deaths,90 ~ 95,1 14(0 05 to 2 24)

Libya,Deaths,20 ~ 25,0 41(-2 63 to 3 55)

Libya,Deaths,25 ~ 30,0 41(-1 72 to 2 59)

Libya,Deaths,30 ~ 35,0 43(-1 21 to 2 09)

Libya,Deaths,35 ~ 40,0 42(-0 92 to 1 77)

Libya,Deaths,40 ~ 45,0 42(-0 72 to 1 58)

Libya,Deaths,45 ~ 50,0 4(-0 57 to 1 39)

Libya,Deaths,50 ~ 55,0 35(-0 49 to 1 19)

Libya,Deaths,55 ~ 60,0 3(-0 42 to 1 03)

Libya,Deaths,60 ~ 65,0 21(-0 41 to 0 84)

Libya,Deaths,65 ~ 70,0 04(-0 53 to 0 61)

Libya,Deaths,70 ~ 75,0 03(-0 5 to 0 56)

Libya,Deaths,75 ~ 80,0 33(-0 19 to 0 85)

Libya,Deaths,80 ~ 85,0 83(0 26 to 1 42)

Libya,Deaths,85 ~ 90,1 12(0 4 to 1 84)

Libya,Deaths,90 ~ 95,1 14(0 05 to 2 24)

Libya,Deaths,20 ~ 25,0 41(-2 63 to 3 55)

Libya,Deaths,25 ~ 30,0 41(-1 72 to 2 59)

Libya,Deaths,30 ~ 35,0 43(-1 21 to 2 09)

Libya,Deaths,35 ~ 40,0 42(-0 92 to 1 77)

Libya,Deaths,40 ~ 45,0 42(-0 72 to 1 58)

Libya,Deaths,45 ~ 50,0 4(-0 57 to 1 39)

Libya,Deaths,50 ~ 55,0 35(-0 49 to 1 19)

Libya,Deaths,55 ~ 60,0 3(-0 42 to 1 03)

Libya,Deaths,60 ~ 65,0 21(-0 41 to 0 84)

Libya,Deaths,65 ~ 70,0 04(-0 53 to 0 61)

Libya,Deaths,70 ~ 75,0 03(-0 5 to 0 56)

Libya,Deaths,75 ~ 80,0 33(-0 19 to 0 85)

Libya,Deaths,80 ~ 85,0 83(0 26 to 1 42)

Libya,Deaths,85 ~ 90,1 12(0 4 to 1 84)

Libya,Deaths,90 ~ 95,1 14(0 05 to 2 24)

Libya,Deaths,20 ~ 25,0 41(-2 63 to 3 55)

Libya,Deaths,25 ~ 30,0 41(-1 72 to 2 59)

Libya,Deaths,30 ~ 35,0 43(-1 21 to 2 09)

Libya,Deaths,35 ~ 40,0 42(-0 92 to 1 77)

Libya,Deaths,40 ~ 45,0 42(-0 72 to 1 58)

Libya,Deaths,45 ~ 50,0 4(-0 57 to 1 39)

Libya,Deaths,50 ~ 55,0 35(-0 49 to 1 19)

Libya,Deaths,55 ~ 60,0 3(-0 42 to 1 03)

Libya,Deaths,60 ~ 65,0 21(-0 41 to 0 84)

Libya,Deaths,65 ~ 70,0 04(-0 53 to 0 61)

Libya,Deaths,70 ~ 75,0 03(-0 5 to 0 56)

Libya,Deaths,75 ~ 80,0 33(-0 19 to 0 85)

Libya,Deaths,80 ~ 85,0 83(0 26 to 1 42)

Libya,Deaths,85 ~ 90,1 12(0 4 to 1 84)

Libya,Deaths,90 ~ 95,1 14(0 05 to 2 24)

Libya,DALYs,20 ~ 25,0 32(-0 09 to 0 72)

Libya,DALYs,25 ~ 30,0 3(0 01 to 0 59)

Libya,DALYs,30 ~ 35,0 31(0 07 to 0 55)

Libya,DALYs,35 ~ 40,0 33(0 12 to 0 54)

Libya,DALYs,40 ~ 45,0 36(0 17 to 0 55)

Libya,DALYs,45 ~ 50,0 39(0 22 to 0 57)

Libya,DALYs,50 ~ 55,0 38(0 22 to 0 54)

Libya,DALYs,55 ~ 60,0 37(0 22 to 0 52)

Libya,DALYs,60 ~ 65,0 32(0 18 to 0 47)

Libya,DALYs,65 ~ 70,0 2(0 06 to 0 35)

Libya,DALYs,70 ~ 75,0 22(0 07 to 0 37)

Libya,DALYs,75 ~ 80,0 46(0 29 to 0 63)

Libya,DALYs,80 ~ 85,0 87(0 66 to 1 07)

Libya,DALYs,85 ~ 90,1 11(0 83 to 1 39)

Libya,DALYs,90 ~ 95,1 13(0 68 to 1 57)

Libya,DALYs,20 ~ 25,0 32(-0 09 to 0 72)

Libya,DALYs,25 ~ 30,0 3(0 01 to 0 59)

Libya,DALYs,30 ~ 35,0 31(0 07 to 0 55)

Libya,DALYs,35 ~ 40,0 33(0 12 to 0 54)

Libya,DALYs,40 ~ 45,0 36(0 17 to 0 55)

Libya,DALYs,45 ~ 50,0 39(0 22 to 0 57)

Libya,DALYs,50 ~ 55,0 38(0 22 to 0 54)

Libya,DALYs,55 ~ 60,0 37(0 22 to 0 52)

Libya,DALYs,60 ~ 65,0 32(0 18 to 0 47)

Libya,DALYs,65 ~ 70,0 2(0 06 to 0 35)

Libya,DALYs,70 ~ 75,0 22(0 07 to 0 37)

Libya,DALYs,75 ~ 80,0 46(0 29 to 0 63)

Libya,DALYs,80 ~ 85,0 87(0 66 to 1 07)

Libya,DALYs,85 ~ 90,1 11(0 83 to 1 39)

Libya,DALYs,90 ~ 95,1 13(0 68 to 1 57)

Libya,DALYs,20 ~ 25,0 32(-0 09 to 0 72)

Libya,DALYs,25 ~ 30,0 3(0 01 to 0 59)

Libya,DALYs,30 ~ 35,0 31(0 07 to 0 55)

Libya,DALYs,35 ~ 40,0 33(0 12 to 0 54)

Libya,DALYs,40 ~ 45,0 36(0 17 to 0 55)

Libya,DALYs,45 ~ 50,0 39(0 22 to 0 57)

Libya,DALYs,50 ~ 55,0 38(0 22 to 0 54)

Libya,DALYs,55 ~ 60,0 37(0 22 to 0 52)

Libya,DALYs,60 ~ 65,0 32(0 18 to 0 47)

Libya,DALYs,65 ~ 70,0 2(0 06 to 0 35)

Libya,DALYs,70 ~ 75,0 22(0 07 to 0 37)

Libya,DALYs,75 ~ 80,0 46(0 29 to 0 63)

Libya,DALYs,80 ~ 85,0 87(0 66 to 1 07)

Libya,DALYs,85 ~ 90,1 11(0 83 to 1 39)

Libya,DALYs,90 ~ 95,1 13(0 68 to 1 57)

Libya,DALYs,20 ~ 25,0 32(-0 09 to 0 72)

Libya,DALYs,25 ~ 30,0 3(0 01 to 0 59)

Libya,DALYs,30 ~ 35,0 31(0 07 to 0 55)

Libya,DALYs,35 ~ 40,0 33(0 12 to 0 54)

Libya,DALYs,40 ~ 45,0 36(0 17 to 0 55)

Libya,DALYs,45 ~ 50,0 39(0 22 to 0 57)

Libya,DALYs,50 ~ 55,0 38(0 22 to 0 54)

Libya,DALYs,55 ~ 60,0 37(0 22 to 0 52)

Libya,DALYs,60 ~ 65,0 32(0 18 to 0 47)

Libya,DALYs,65 ~ 70,0 2(0 06 to 0 35)

Libya,DALYs,70 ~ 75,0 22(0 07 to 0 37)

Libya,DALYs,75 ~ 80,0 46(0 29 to 0 63)

Libya,DALYs,80 ~ 85,0 87(0 66 to 1 07)

Libya,DALYs,85 ~ 90,1 11(0 83 to 1 39)

Libya,DALYs,90 ~ 95,1 13(0 68 to 1 57)

Denmark,Prevalence,20 ~ 25,-0 78(-1 18 to -0 37)

Denmark,Prevalence,25 ~ 30,-0 69(-0 96 to -0 41)

Denmark,Prevalence,30 ~ 35,-0 63(-0 85 to -0 4)

Denmark,Prevalence,35 ~ 40,-0 62(-0 81 to -0 43)

Denmark,Prevalence,40 ~ 45,-0 64(-0 79 to -0 49)

Denmark,Prevalence,45 ~ 50,-0 66(-0 78 to -0 54)

Denmark,Prevalence,50 ~ 55,-0 63(-0 72 to -0 53)

Denmark,Prevalence,55 ~ 60,-0 54(-0 63 to -0 46)

Denmark,Prevalence,60 ~ 65,-0 44(-0 52 to -0 37)

Denmark,Prevalence,65 ~ 70,-0 35(-0 42 to -0 28)

Denmark,Prevalence,70 ~ 75,-0 26(-0 32 to -0 19)

Denmark,Prevalence,75 ~ 80,-0 16(-0 23 to -0 1)

Denmark,Prevalence,80 ~ 85,-0 06(-0 13 to 0 01)

Denmark,Prevalence,85 ~ 90,0 03(-0 06 to 0 13)

Denmark,Prevalence,90 ~ 95,0 11(-0 05 to 0 27)

Denmark,Prevalence,20 ~ 25,-0 78(-1 18 to -0 37)

Denmark,Prevalence,25 ~ 30,-0 69(-0 96 to -0 41)

Denmark,Prevalence,30 ~ 35,-0 63(-0 85 to -0 4)

Denmark,Prevalence,35 ~ 40,-0 62(-0 81 to -0 43)

Denmark,Prevalence,40 ~ 45,-0 64(-0 79 to -0 49)

Denmark,Prevalence,45 ~ 50,-0 66(-0 78 to -0 54)

Denmark,Prevalence,50 ~ 55,-0 63(-0 72 to -0 53)

Denmark,Prevalence,55 ~ 60,-0 54(-0 63 to -0 46)

Denmark,Prevalence,60 ~ 65,-0 44(-0 52 to -0 37)

Denmark,Prevalence,65 ~ 70,-0 35(-0 42 to -0 28)

Denmark,Prevalence,70 ~ 75,-0 26(-0 32 to -0 19)

Denmark,Prevalence,75 ~ 80,-0 16(-0 23 to -0 1)

Denmark,Prevalence,80 ~ 85,-0 06(-0 13 to 0 01)

Denmark,Prevalence,85 ~ 90,0 03(-0 06 to 0 13)

Denmark,Prevalence,90 ~ 95,0 11(-0 05 to 0 27)

Denmark,Prevalence,20 ~ 25,-0 78(-1 18 to -0 37)

Denmark,Prevalence,25 ~ 30,-0 69(-0 96 to -0 41)

Denmark,Prevalence,30 ~ 35,-0 63(-0 85 to -0 4)

Denmark,Prevalence,35 ~ 40,-0 62(-0 81 to -0 43)

Denmark,Prevalence,40 ~ 45,-0 64(-0 79 to -0 49)

Denmark,Prevalence,45 ~ 50,-0 66(-0 78 to -0 54)

Denmark,Prevalence,50 ~ 55,-0 63(-0 72 to -0 53)

Denmark,Prevalence,55 ~ 60,-0 54(-0 63 to -0 46)

Denmark,Prevalence,60 ~ 65,-0 44(-0 52 to -0 37)

Denmark,Prevalence,65 ~ 70,-0 35(-0 42 to -0 28)

Denmark,Prevalence,70 ~ 75,-0 26(-0 32 to -0 19)

Denmark,Prevalence,75 ~ 80,-0 16(-0 23 to -0 1)

Denmark,Prevalence,80 ~ 85,-0 06(-0 13 to 0 01)

Denmark,Prevalence,85 ~ 90,0 03(-0 06 to 0 13)

Denmark,Prevalence,90 ~ 95,0 11(-0 05 to 0 27)

Denmark,Prevalence,20 ~ 25,-0 78(-1 18 to -0 37)

Denmark,Prevalence,25 ~ 30,-0 69(-0 96 to -0 41)

Denmark,Prevalence,30 ~ 35,-0 63(-0 85 to -0 4)

Denmark,Prevalence,35 ~ 40,-0 62(-0 81 to -0 43)

Denmark,Prevalence,40 ~ 45,-0 64(-0 79 to -0 49)

Denmark,Prevalence,45 ~ 50,-0 66(-0 78 to -0 54)

Denmark,Prevalence,50 ~ 55,-0 63(-0 72 to -0 53)

Denmark,Prevalence,55 ~ 60,-0 54(-0 63 to -0 46)

Denmark,Prevalence,60 ~ 65,-0 44(-0 52 to -0 37)

Denmark,Prevalence,65 ~ 70,-0 35(-0 42 to -0 28)

Denmark,Prevalence,70 ~ 75,-0 26(-0 32 to -0 19)

Denmark,Prevalence,75 ~ 80,-0 16(-0 23 to -0 1)

Denmark,Prevalence,80 ~ 85,-0 06(-0 13 to 0 01)

Denmark,Prevalence,85 ~ 90,0 03(-0 06 to 0 13)

Denmark,Prevalence,90 ~ 95,0 11(-0 05 to 0 27)

Denmark,Deaths,20 ~ 25,-0 94(-6 79 to 5 29)

Denmark,Deaths,25 ~ 30,-1 44(-5 7 to 3 01)

Denmark,Deaths,30 ~ 35,-1 8(-5 12 to 1 64)

Denmark,Deaths,35 ~ 40,-1 95(-4 41 to 0 58)

Denmark,Deaths,40 ~ 45,-1 93(-3 61 to -0 21)

Denmark,Deaths,45 ~ 50,-1 78(-2 86 to -0 69)

Denmark,Deaths,50 ~ 55,-1 7(-2 37 to -1 02)

Denmark,Deaths,55 ~ 60,-1 94(-2 37 to -1 51)

Denmark,Deaths,60 ~ 65,-2 3(-2 61 to -2)

Denmark,Deaths,65 ~ 70,-2 42(-2 64 to -2 19)

Denmark,Deaths,70 ~ 75,-2 01(-2 19 to -1 83)

Denmark,Deaths,75 ~ 80,-0 75(-0 92 to -0 59)

Denmark,Deaths,80 ~ 85,0 95(0 77 to 1 13)

Denmark,Deaths,85 ~ 90,2 49(2 25 to 2 74)

Denmark,Deaths,90 ~ 95,3 03(2 62 to 3 44)

Denmark,Deaths,20 ~ 25,-0 94(-6 79 to 5 29)

Denmark,Deaths,25 ~ 30,-1 44(-5 7 to 3 01)

Denmark,Deaths,30 ~ 35,-1 8(-5 12 to 1 64)

Denmark,Deaths,35 ~ 40,-1 95(-4 41 to 0 58)

Denmark,Deaths,40 ~ 45,-1 93(-3 61 to -0 21)

Denmark,Deaths,45 ~ 50,-1 78(-2 86 to -0 69)

Denmark,Deaths,50 ~ 55,-1 7(-2 37 to -1 02)

Denmark,Deaths,55 ~ 60,-1 94(-2 37 to -1 51)

Denmark,Deaths,60 ~ 65,-2 3(-2 61 to -2)

Denmark,Deaths,65 ~ 70,-2 42(-2 64 to -2 19)

Denmark,Deaths,70 ~ 75,-2 01(-2 19 to -1 83)

Denmark,Deaths,75 ~ 80,-0 75(-0 92 to -0 59)

Denmark,Deaths,80 ~ 85,0 95(0 77 to 1 13)

Denmark,Deaths,85 ~ 90,2 49(2 25 to 2 74)

Denmark,Deaths,90 ~ 95,3 03(2 62 to 3 44)

Denmark,Deaths,20 ~ 25,-0 94(-6 79 to 5 29)

Denmark,Deaths,25 ~ 30,-1 44(-5 7 to 3 01)

Denmark,Deaths,30 ~ 35,-1 8(-5 12 to 1 64)

Denmark,Deaths,35 ~ 40,-1 95(-4 41 to 0 58)

Denmark,Deaths,40 ~ 45,-1 93(-3 61 to -0 21)

Denmark,Deaths,45 ~ 50,-1 78(-2 86 to -0 69)

Denmark,Deaths,50 ~ 55,-1 7(-2 37 to -1 02)

Denmark,Deaths,55 ~ 60,-1 94(-2 37 to -1 51)

Denmark,Deaths,60 ~ 65,-2 3(-2 61 to -2)

Denmark,Deaths,65 ~ 70,-2 42(-2 64 to -2 19)

Denmark,Deaths,70 ~ 75,-2 01(-2 19 to -1 83)

Denmark,Deaths,75 ~ 80,-0 75(-0 92 to -0 59)

Denmark,Deaths,80 ~ 85,0 95(0 77 to 1 13)

Denmark,Deaths,85 ~ 90,2 49(2 25 to 2 74)

Denmark,Deaths,90 ~ 95,3 03(2 62 to 3 44)

Denmark,Deaths,20 ~ 25,-0 94(-6 79 to 5 29)

Denmark,Deaths,25 ~ 30,-1 44(-5 7 to 3 01)

Denmark,Deaths,30 ~ 35,-1 8(-5 12 to 1 64)

Denmark,Deaths,35 ~ 40,-1 95(-4 41 to 0 58)

Denmark,Deaths,40 ~ 45,-1 93(-3 61 to -0 21)

Denmark,Deaths,45 ~ 50,-1 78(-2 86 to -0 69)

Denmark,Deaths,50 ~ 55,-1 7(-2 37 to -1 02)

Denmark,Deaths,55 ~ 60,-1 94(-2 37 to -1 51)

Denmark,Deaths,60 ~ 65,-2 3(-2 61 to -2)

Denmark,Deaths,65 ~ 70,-2 42(-2 64 to -2 19)

Denmark,Deaths,70 ~ 75,-2 01(-2 19 to -1 83)

Denmark,Deaths,75 ~ 80,-0 75(-0 92 to -0 59)

Denmark,Deaths,80 ~ 85,0 95(0 77 to 1 13)

Denmark,Deaths,85 ~ 90,2 49(2 25 to 2 74)

Denmark,Deaths,90 ~ 95,3 03(2 62 to 3 44)

Denmark,DALYs,20 ~ 25,-0 81(-2 57 to 0 99)

Denmark,DALYs,25 ~ 30,-1(-2 26 to 0 28)

Denmark,DALYs,30 ~ 35,-1 16(-2 18 to -0 14)

Denmark,DALYs,35 ~ 40,-1 3(-2 12 to -0 48)

Denmark,DALYs,40 ~ 45,-1 32(-1 94 to -0 7)

Denmark,DALYs,45 ~ 50,-1 29(-1 73 to -0 84)

Denmark,DALYs,50 ~ 55,-1 33(-1 64 to -1 01)

Denmark,DALYs,55 ~ 60,-1 61(-1 84 to -1 38)

Denmark,DALYs,60 ~ 65,-1 98(-2 16 to -1 79)

Denmark,DALYs,65 ~ 70,-2 09(-2 24 to -1 94)

Denmark,DALYs,70 ~ 75,-1 76(-1 9 to -1 63)

Denmark,DALYs,75 ~ 80,-0 7(-0 84 to -0 57)

Denmark,DALYs,80 ~ 85,0 76(0 59 to 0 93)

Denmark,DALYs,85 ~ 90,2 09(1 84 to 2 34)

Denmark,DALYs,90 ~ 95,2 54(2 1 to 2 98)

Denmark,DALYs,20 ~ 25,-0 81(-2 57 to 0 99)

Denmark,DALYs,25 ~ 30,-1(-2 26 to 0 28)

Denmark,DALYs,30 ~ 35,-1 16(-2 18 to -0 14)

Denmark,DALYs,35 ~ 40,-1 3(-2 12 to -0 48)

Denmark,DALYs,40 ~ 45,-1 32(-1 94 to -0 7)

Denmark,DALYs,45 ~ 50,-1 29(-1 73 to -0 84)

Denmark,DALYs,50 ~ 55,-1 33(-1 64 to -1 01)

Denmark,DALYs,55 ~ 60,-1 61(-1 84 to -1 38)

Denmark,DALYs,60 ~ 65,-1 98(-2 16 to -1 79)

Denmark,DALYs,65 ~ 70,-2 09(-2 24 to -1 94)

Denmark,DALYs,70 ~ 75,-1 76(-1 9 to -1 63)

Denmark,DALYs,75 ~ 80,-0 7(-0 84 to -0 57)

Denmark,DALYs,80 ~ 85,0 76(0 59 to 0 93)

Denmark,DALYs,85 ~ 90,2 09(1 84 to 2 34)

Denmark,DALYs,90 ~ 95,2 54(2 1 to 2 98)

Denmark,DALYs,20 ~ 25,-0 81(-2 57 to 0 99)

Denmark,DALYs,25 ~ 30,-1(-2 26 to 0 28)

Denmark,DALYs,30 ~ 35,-1 16(-2 18 to -0 14)

Denmark,DALYs,35 ~ 40,-1 3(-2 12 to -0 48)

Denmark,DALYs,40 ~ 45,-1 32(-1 94 to -0 7)

Denmark,DALYs,45 ~ 50,-1 29(-1 73 to -0 84)

Denmark,DALYs,50 ~ 55,-1 33(-1 64 to -1 01)

Denmark,DALYs,55 ~ 60,-1 61(-1 84 to -1 38)

Denmark,DALYs,60 ~ 65,-1 98(-2 16 to -1 79)

Denmark,DALYs,65 ~ 70,-2 09(-2 24 to -1 94)

Denmark,DALYs,70 ~ 75,-1 76(-1 9 to -1 63)

Denmark,DALYs,75 ~ 80,-0 7(-0 84 to -0 57)

Denmark,DALYs,80 ~ 85,0 76(0 59 to 0 93)

Denmark,DALYs,85 ~ 90,2 09(1 84 to 2 34)

Denmark,DALYs,90 ~ 95,2 54(2 1 to 2 98)

Denmark,DALYs,20 ~ 25,-0 81(-2 57 to 0 99)

Denmark,DALYs,25 ~ 30,-1(-2 26 to 0 28)

Denmark,DALYs,30 ~ 35,-1 16(-2 18 to -0 14)

Denmark,DALYs,35 ~ 40,-1 3(-2 12 to -0 48)

Denmark,DALYs,40 ~ 45,-1 32(-1 94 to -0 7)

Denmark,DALYs,45 ~ 50,-1 29(-1 73 to -0 84)

Denmark,DALYs,50 ~ 55,-1 33(-1 64 to -1 01)

Denmark,DALYs,55 ~ 60,-1 61(-1 84 to -1 38)

Denmark,DALYs,60 ~ 65,-1 98(-2 16 to -1 79)

Denmark,DALYs,65 ~ 70,-2 09(-2 24 to -1 94)

Denmark,DALYs,70 ~ 75,-1 76(-1 9 to -1 63)

Denmark,DALYs,75 ~ 80,-0 7(-0 84 to -0 57)

Denmark,DALYs,80 ~ 85,0 76(0 59 to 0 93)

Denmark,DALYs,85 ~ 90,2 09(1 84 to 2 34)

Denmark,DALYs,90 ~ 95,2 54(2 1 to 2 98)

Taiwan_(Province_of_China),Prevalence,20 ~ 25,-0 38(-0 55 to -0 21)

Taiwan_(Province_of_China),Prevalence,25 ~ 30,-0 41(-0 52 to -0 29)

Taiwan_(Province_of_China),Prevalence,30 ~ 35,-0 45(-0 54 to -0 36)

Taiwan_(Province_of_China),Prevalence,35 ~ 40,-0 52(-0 59 to -0 45)

Taiwan_(Province_of_China),Prevalence,40 ~ 45,-0 62(-0 68 to -0 56)

Taiwan_(Province_of_China),Prevalence,45 ~ 50,-0 75(-0 8 to -0 69)

Taiwan_(Province_of_China),Prevalence,50 ~ 55,-0 86(-0 91 to -0 82)

Taiwan_(Province_of_China),Prevalence,55 ~ 60,-0 96(-1 to -0 92)

Taiwan_(Province_of_China),Prevalence,60 ~ 65,-1 05(-1 09 to -1 01)

Taiwan_(Province_of_China),Prevalence,65 ~ 70,-1 1(-1 13 to -1 06)

Taiwan_(Province_of_China),Prevalence,70 ~ 75,-1 07(-1 11 to -1 04)

Taiwan_(Province_of_China),Prevalence,75 ~ 80,-0 98(-1 02 to -0 95)

Taiwan_(Province_of_China),Prevalence,80 ~ 85,-0 84(-0 88 to -0 79)

Taiwan_(Province_of_China),Prevalence,85 ~ 90,-0 64(-0 71 to -0 57)

Taiwan_(Province_of_China),Prevalence,90 ~ 95,-0 43(-0 56 to -0 3)

Taiwan_(Province_of_China),Prevalence,20 ~ 25,-0 38(-0 55 to -0 21)

Taiwan_(Province_of_China),Prevalence,25 ~ 30,-0 41(-0 52 to -0 29)

Taiwan_(Province_of_China),Prevalence,30 ~ 35,-0 45(-0 54 to -0 36)

Taiwan_(Province_of_China),Prevalence,35 ~ 40,-0 52(-0 59 to -0 45)

Taiwan_(Province_of_China),Prevalence,40 ~ 45,-0 62(-0 68 to -0 56)

Taiwan_(Province_of_China),Prevalence,45 ~ 50,-0 75(-0 8 to -0 69)

Taiwan_(Province_of_China),Prevalence,50 ~ 55,-0 86(-0 91 to -0 82)

Taiwan_(Province_of_China),Prevalence,55 ~ 60,-0 96(-1 to -0 92)

Taiwan_(Province_of_China),Prevalence,60 ~ 65,-1 05(-1 09 to -1 01)

Taiwan_(Province_of_China),Prevalence,65 ~ 70,-1 1(-1 13 to -1 06)

Taiwan_(Province_of_China),Prevalence,70 ~ 75,-1 07(-1 11 to -1 04)

Taiwan_(Province_of_China),Prevalence,75 ~ 80,-0 98(-1 02 to -0 95)

Taiwan_(Province_of_China),Prevalence,80 ~ 85,-0 84(-0 88 to -0 79)

Taiwan_(Province_of_China),Prevalence,85 ~ 90,-0 64(-0 71 to -0 57)

Taiwan_(Province_of_China),Prevalence,90 ~ 95,-0 43(-0 56 to -0 3)

Taiwan_(Province_of_China),Prevalence,20 ~ 25,-0 38(-0 55 to -0 21)

Taiwan_(Province_of_China),Prevalence,25 ~ 30,-0 41(-0 52 to -0 29)

Taiwan_(Province_of_China),Prevalence,30 ~ 35,-0 45(-0 54 to -0 36)

Taiwan_(Province_of_China),Prevalence,35 ~ 40,-0 52(-0 59 to -0 45)

Taiwan_(Province_of_China),Prevalence,40 ~ 45,-0 62(-0 68 to -0 56)

Taiwan_(Province_of_China),Prevalence,45 ~ 50,-0 75(-0 8 to -0 69)

Taiwan_(Province_of_China),Prevalence,50 ~ 55,-0 86(-0 91 to -0 82)

Taiwan_(Province_of_China),Prevalence,55 ~ 60,-0 96(-1 to -0 92)

Taiwan_(Province_of_China),Prevalence,60 ~ 65,-1 05(-1 09 to -1 01)

Taiwan_(Province_of_China),Prevalence,65 ~ 70,-1 1(-1 13 to -1 06)

Taiwan_(Province_of_China),Prevalence,70 ~ 75,-1 07(-1 11 to -1 04)

Taiwan_(Province_of_China),Prevalence,75 ~ 80,-0 98(-1 02 to -0 95)

Taiwan_(Province_of_China),Prevalence,80 ~ 85,-0 84(-0 88 to -0 79)

Taiwan_(Province_of_China),Prevalence,85 ~ 90,-0 64(-0 71 to -0 57)

Taiwan_(Province_of_China),Prevalence,90 ~ 95,-0 43(-0 56 to -0 3)

Taiwan_(Province_of_China),Prevalence,20 ~ 25,-0 38(-0 55 to -0 21)

Taiwan_(Province_of_China),Prevalence,25 ~ 30,-0 41(-0 52 to -0 29)

Taiwan_(Province_of_China),Prevalence,30 ~ 35,-0 45(-0 54 to -0 36)

Taiwan_(Province_of_China),Prevalence,35 ~ 40,-0 52(-0 59 to -0 45)

Taiwan_(Province_of_China),Prevalence,40 ~ 45,-0 62(-0 68 to -0 56)

Taiwan_(Province_of_China),Prevalence,45 ~ 50,-0 75(-0 8 to -0 69)

Taiwan_(Province_of_China),Prevalence,50 ~ 55,-0 86(-0 91 to -0 82)

Taiwan_(Province_of_China),Prevalence,55 ~ 60,-0 96(-1 to -0 92)

Taiwan_(Province_of_China),Prevalence,60 ~ 65,-1 05(-1 09 to -1 01)

Taiwan_(Province_of_China),Prevalence,65 ~ 70,-1 1(-1 13 to -1 06)

Taiwan_(Province_of_China),Prevalence,70 ~ 75,-1 07(-1 11 to -1 04)

Taiwan_(Province_of_China),Prevalence,75 ~ 80,-0 98(-1 02 to -0 95)

Taiwan_(Province_of_China),Prevalence,80 ~ 85,-0 84(-0 88 to -0 79)

Taiwan_(Province_of_China),Prevalence,85 ~ 90,-0 64(-0 71 to -0 57)

Taiwan_(Province_of_China),Prevalence,90 ~ 95,-0 43(-0 56 to -0 3)

Taiwan_(Province_of_China),Deaths,20 ~ 25,-3 95(-10 05 to 2 58)

Taiwan_(Province_of_China),Deaths,25 ~ 30,-4 18(-8 14 to -0 05)

Taiwan_(Province_of_China),Deaths,30 ~ 35,-3 76(-6 44 to -1)

Taiwan_(Province_of_China),Deaths,35 ~ 40,-2 9(-4 63 to -1 14)

Taiwan_(Province_of_China),Deaths,40 ~ 45,-2 31(-3 49 to -1 11)

Taiwan_(Province_of_China),Deaths,45 ~ 50,-2 09(-2 96 to -1 22)

Taiwan_(Province_of_China),Deaths,50 ~ 55,-2 01(-2 61 to -1 41)

Taiwan_(Province_of_China),Deaths,55 ~ 60,-2 41(-2 83 to -1 99)

Taiwan_(Province_of_China),Deaths,60 ~ 65,-2 97(-3 27 to -2 68)

Taiwan_(Province_of_China),Deaths,65 ~ 70,-3 24(-3 46 to -3 01)

Taiwan_(Province_of_China),Deaths,70 ~ 75,-3 13(-3 31 to -2 94)

Taiwan_(Province_of_China),Deaths,75 ~ 80,-2 68(-2 83 to -2 52)

Taiwan_(Province_of_China),Deaths,80 ~ 85,-2 14(-2 29 to -2)

Taiwan_(Province_of_China),Deaths,85 ~ 90,-1 35(-1 53 to -1 16)

Taiwan_(Province_of_China),Deaths,90 ~ 95,-0 48(-0 83 to -0 12)

Taiwan_(Province_of_China),Deaths,20 ~ 25,-3 95(-10 05 to 2 58)

Taiwan_(Province_of_China),Deaths,25 ~ 30,-4 18(-8 14 to -0 05)

Taiwan_(Province_of_China),Deaths,30 ~ 35,-3 76(-6 44 to -1)

Taiwan_(Province_of_China),Deaths,35 ~ 40,-2 9(-4 63 to -1 14)

Taiwan_(Province_of_China),Deaths,40 ~ 45,-2 31(-3 49 to -1 11)

Taiwan_(Province_of_China),Deaths,45 ~ 50,-2 09(-2 96 to -1 22)

Taiwan_(Province_of_China),Deaths,50 ~ 55,-2 01(-2 61 to -1 41)

Taiwan_(Province_of_China),Deaths,55 ~ 60,-2 41(-2 83 to -1 99)

Taiwan_(Province_of_China),Deaths,60 ~ 65,-2 97(-3 27 to -2 68)

Taiwan_(Province_of_China),Deaths,65 ~ 70,-3 24(-3 46 to -3 01)

Taiwan_(Province_of_China),Deaths,70 ~ 75,-3 13(-3 31 to -2 94)

Taiwan_(Province_of_China),Deaths,75 ~ 80,-2 68(-2 83 to -2 52)

Taiwan_(Province_of_China),Deaths,80 ~ 85,-2 14(-2 29 to -2)

Taiwan_(Province_of_China),Deaths,85 ~ 90,-1 35(-1 53 to -1 16)

Taiwan_(Province_of_China),Deaths,90 ~ 95,-0 48(-0 83 to -0 12)

Taiwan_(Province_of_China),Deaths,20 ~ 25,-3 95(-10 05 to 2 58)

Taiwan_(Province_of_China),Deaths,25 ~ 30,-4 18(-8 14 to -0 05)

Taiwan_(Province_of_China),Deaths,30 ~ 35,-3 76(-6 44 to -1)

Taiwan_(Province_of_China),Deaths,35 ~ 40,-2 9(-4 63 to -1 14)

Taiwan_(Province_of_China),Deaths,40 ~ 45,-2 31(-3 49 to -1 11)

Taiwan_(Province_of_China),Deaths,45 ~ 50,-2 09(-2 96 to -1 22)

Taiwan_(Province_of_China),Deaths,50 ~ 55,-2 01(-2 61 to -1 41)

Taiwan_(Province_of_China),Deaths,55 ~ 60,-2 41(-2 83 to -1 99)

Taiwan_(Province_of_China),Deaths,60 ~ 65,-2 97(-3 27 to -2 68)

Taiwan_(Province_of_China),Deaths,65 ~ 70,-3 24(-3 46 to -3 01)

Taiwan_(Province_of_China),Deaths,70 ~ 75,-3 13(-3 31 to -2 94)

Taiwan_(Province_of_China),Deaths,75 ~ 80,-2 68(-2 83 to -2 52)

Taiwan_(Province_of_China),Deaths,80 ~ 85,-2 14(-2 29 to -2)

Taiwan_(Province_of_China),Deaths,85 ~ 90,-1 35(-1 53 to -1 16)

Taiwan_(Province_of_China),Deaths,90 ~ 95,-0 48(-0 83 to -0 12)

Taiwan_(Province_of_China),Deaths,20 ~ 25,-3 95(-10 05 to 2 58)

Taiwan_(Province_of_China),Deaths,25 ~ 30,-4 18(-8 14 to -0 05)

Taiwan_(Province_of_China),Deaths,30 ~ 35,-3 76(-6 44 to -1)

Taiwan_(Province_of_China),Deaths,35 ~ 40,-2 9(-4 63 to -1 14)

Taiwan_(Province_of_China),Deaths,40 ~ 45,-2 31(-3 49 to -1 11)

Taiwan_(Province_of_China),Deaths,45 ~ 50,-2 09(-2 96 to -1 22)

Taiwan_(Province_of_China),Deaths,50 ~ 55,-2 01(-2 61 to -1 41)

Taiwan_(Province_of_China),Deaths,55 ~ 60,-2 41(-2 83 to -1 99)

Taiwan_(Province_of_China),Deaths,60 ~ 65,-2 97(-3 27 to -2 68)

Taiwan_(Province_of_China),Deaths,65 ~ 70,-3 24(-3 46 to -3 01)

Taiwan_(Province_of_China),Deaths,70 ~ 75,-3 13(-3 31 to -2 94)

Taiwan_(Province_of_China),Deaths,75 ~ 80,-2 68(-2 83 to -2 52)

Taiwan_(Province_of_China),Deaths,80 ~ 85,-2 14(-2 29 to -2)

Taiwan_(Province_of_China),Deaths,85 ~ 90,-1 35(-1 53 to -1 16)

Taiwan_(Province_of_China),Deaths,90 ~ 95,-0 48(-0 83 to -0 12)

Taiwan_(Province_of_China),DALYs,20 ~ 25,-1 07(-1 88 to -0 26)

Taiwan_(Province_of_China),DALYs,25 ~ 30,-1 2(-1 74 to -0 65)

Taiwan_(Province_of_China),DALYs,30 ~ 35,-1 25(-1 67 to -0 83)

Taiwan_(Province_of_China),DALYs,35 ~ 40,-1 18(-1 51 to -0 85)

Taiwan_(Province_of_China),DALYs,40 ~ 45,-1 14(-1 4 to -0 87)

Taiwan_(Province_of_China),DALYs,45 ~ 50,-1 26(-1 49 to -1 04)

Taiwan_(Province_of_China),DALYs,50 ~ 55,-1 44(-1 62 to -1 26)

Taiwan_(Province_of_China),DALYs,55 ~ 60,-1 8(-1 95 to -1 65)

Taiwan_(Province_of_China),DALYs,60 ~ 65,-2 25(-2 37 to -2 12)

Taiwan_(Province_of_China),DALYs,65 ~ 70,-2 49(-2 59 to -2 38)

Taiwan_(Province_of_China),DALYs,70 ~ 75,-2 49(-2 59 to -2 39)

Taiwan_(Province_of_China),DALYs,75 ~ 80,-2 27(-2 37 to -2 18)

Taiwan_(Province_of_China),DALYs,80 ~ 85,-1 92(-2 02 to -1 81)

Taiwan_(Province_of_China),DALYs,85 ~ 90,-1 31(-1 47 to -1 16)

Taiwan_(Province_of_China),DALYs,90 ~ 95,-0 59(-0 9 to -0 28)

Taiwan_(Province_of_China),DALYs,20 ~ 25,-1 07(-1 88 to -0 26)

Taiwan_(Province_of_China),DALYs,25 ~ 30,-1 2(-1 74 to -0 65)

Taiwan_(Province_of_China),DALYs,30 ~ 35,-1 25(-1 67 to -0 83)

Taiwan_(Province_of_China),DALYs,35 ~ 40,-1 18(-1 51 to -0 85)

Taiwan_(Province_of_China),DALYs,40 ~ 45,-1 14(-1 4 to -0 87)

Taiwan_(Province_of_China),DALYs,45 ~ 50,-1 26(-1 49 to -1 04)

Taiwan_(Province_of_China),DALYs,50 ~ 55,-1 44(-1 62 to -1 26)

Taiwan_(Province_of_China),DALYs,55 ~ 60,-1 8(-1 95 to -1 65)

Taiwan_(Province_of_China),DALYs,60 ~ 65,-2 25(-2 37 to -2 12)

Taiwan_(Province_of_China),DALYs,65 ~ 70,-2 49(-2 59 to -2 38)

Taiwan_(Province_of_China),DALYs,70 ~ 75,-2 49(-2 59 to -2 39)

Taiwan_(Province_of_China),DALYs,75 ~ 80,-2 27(-2 37 to -2 18)

Taiwan_(Province_of_China),DALYs,80 ~ 85,-1 92(-2 02 to -1 81)

Taiwan_(Province_of_China),DALYs,85 ~ 90,-1 31(-1 47 to -1 16)

Taiwan_(Province_of_China),DALYs,90 ~ 95,-0 59(-0 9 to -0 28)

Taiwan_(Province_of_China),DALYs,20 ~ 25,-1 07(-1 88 to -0 26)

Taiwan_(Province_of_China),DALYs,25 ~ 30,-1 2(-1 74 to -0 65)

Taiwan_(Province_of_China),DALYs,30 ~ 35,-1 25(-1 67 to -0 83)

Taiwan_(Province_of_China),DALYs,35 ~ 40,-1 18(-1 51 to -0 85)

Taiwan_(Province_of_China),DALYs,40 ~ 45,-1 14(-1 4 to -0 87)

Taiwan_(Province_of_China),DALYs,45 ~ 50,-1 26(-1 49 to -1 04)

Taiwan_(Province_of_China),DALYs,50 ~ 55,-1 44(-1 62 to -1 26)

Taiwan_(Province_of_China),DALYs,55 ~ 60,-1 8(-1 95 to -1 65)

Taiwan_(Province_of_China),DALYs,60 ~ 65,-2 25(-2 37 to -2 12)

Taiwan_(Province_of_China),DALYs,65 ~ 70,-2 49(-2 59 to -2 38)

Taiwan_(Province_of_China),DALYs,70 ~ 75,-2 49(-2 59 to -2 39)

Taiwan_(Province_of_China),DALYs,75 ~ 80,-2 27(-2 37 to -2 18)

Taiwan_(Province_of_China),DALYs,80 ~ 85,-1 92(-2 02 to -1 81)

Taiwan_(Province_of_China),DALYs,85 ~ 90,-1 31(-1 47 to -1 16)

Taiwan_(Province_of_China),DALYs,90 ~ 95,-0 59(-0 9 to -0 28)

Taiwan_(Province_of_China),DALYs,20 ~ 25,-1 07(-1 88 to -0 26)

Taiwan_(Province_of_China),DALYs,25 ~ 30,-1 2(-1 74 to -0 65)

Taiwan_(Province_of_China),DALYs,30 ~ 35,-1 25(-1 67 to -0 83)

Taiwan_(Province_of_China),DALYs,35 ~ 40,-1 18(-1 51 to -0 85)

Taiwan_(Province_of_China),DALYs,40 ~ 45,-1 14(-1 4 to -0 87)

Taiwan_(Province_of_China),DALYs,45 ~ 50,-1 26(-1 49 to -1 04)

Taiwan_(Province_of_China),DALYs,50 ~ 55,-1 44(-1 62 to -1 26)

Taiwan_(Province_of_China),DALYs,55 ~ 60,-1 8(-1 95 to -1 65)

Taiwan_(Province_of_China),DALYs,60 ~ 65,-2 25(-2 37 to -2 12)

Taiwan_(Province_of_China),DALYs,65 ~ 70,-2 49(-2 59 to -2 38)

Taiwan_(Province_of_China),DALYs,70 ~ 75,-2 49(-2 59 to -2 39)

Taiwan_(Province_of_China),DALYs,75 ~ 80,-2 27(-2 37 to -2 18)

Taiwan_(Province_of_China),DALYs,80 ~ 85,-1 92(-2 02 to -1 81)

Taiwan_(Province_of_China),DALYs,85 ~ 90,-1 31(-1 47 to -1 16)

Taiwan_(Province_of_China),DALYs,90 ~ 95,-0 59(-0 9 to -0 28)

United_Arab_Emirates,Prevalence,20 ~ 25,-0 07(-0 2 to 0 06)

United_Arab_Emirates,Prevalence,25 ~ 30,-0 01(-0 1 to 0 08)

United_Arab_Emirates,Prevalence,30 ~ 35,0 05(0 to 0 11)

United_Arab_Emirates,Prevalence,35 ~ 40,0 05(0 to 0 1)

United_Arab_Emirates,Prevalence,40 ~ 45,0 05(0 to 0 1)

United_Arab_Emirates,Prevalence,45 ~ 50,0 08(0 03 to 0 14)

United_Arab_Emirates,Prevalence,50 ~ 55,0 1(0 04 to 0 16)

United_Arab_Emirates,Prevalence,55 ~ 60,0 16(0 09 to 0 23)

United_Arab_Emirates,Prevalence,60 ~ 65,0 24(0 15 to 0 33)

United_Arab_Emirates,Prevalence,65 ~ 70,0 36(0 26 to 0 46)

United_Arab_Emirates,Prevalence,70 ~ 75,0 47(0 34 to 0 6)

United_Arab_Emirates,Prevalence,75 ~ 80,0 59(0 43 to 0 75)

United_Arab_Emirates,Prevalence,80 ~ 85,0 71(0 52 to 0 91)

United_Arab_Emirates,Prevalence,85 ~ 90,0 86(0 52 to 1 19)

United_Arab_Emirates,Prevalence,90 ~ 95,1 05(0 33 to 1 77)

United_Arab_Emirates,Prevalence,20 ~ 25,-0 07(-0 2 to 0 06)

United_Arab_Emirates,Prevalence,25 ~ 30,-0 01(-0 1 to 0 08)

United_Arab_Emirates,Prevalence,30 ~ 35,0 05(0 to 0 11)

United_Arab_Emirates,Prevalence,35 ~ 40,0 05(0 to 0 1)

United_Arab_Emirates,Prevalence,40 ~ 45,0 05(0 to 0 1)

United_Arab_Emirates,Prevalence,45 ~ 50,0 08(0 03 to 0 14)

United_Arab_Emirates,Prevalence,50 ~ 55,0 1(0 04 to 0 16)

United_Arab_Emirates,Prevalence,55 ~ 60,0 16(0 09 to 0 23)

United_Arab_Emirates,Prevalence,60 ~ 65,0 24(0 15 to 0 33)

United_Arab_Emirates,Prevalence,65 ~ 70,0 36(0 26 to 0 46)

United_Arab_Emirates,Prevalence,70 ~ 75,0 47(0 34 to 0 6)

United_Arab_Emirates,Prevalence,75 ~ 80,0 59(0 43 to 0 75)

United_Arab_Emirates,Prevalence,80 ~ 85,0 71(0 52 to 0 91)

United_Arab_Emirates,Prevalence,85 ~ 90,0 86(0 52 to 1 19)

United_Arab_Emirates,Prevalence,90 ~ 95,1 05(0 33 to 1 77)

United_Arab_Emirates,Prevalence,20 ~ 25,-0 07(-0 2 to 0 06)

United_Arab_Emirates,Prevalence,25 ~ 30,-0 01(-0 1 to 0 08)

United_Arab_Emirates,Prevalence,30 ~ 35,0 05(0 to 0 11)

United_Arab_Emirates,Prevalence,35 ~ 40,0 05(0 to 0 1)

United_Arab_Emirates,Prevalence,40 ~ 45,0 05(0 to 0 1)

United_Arab_Emirates,Prevalence,45 ~ 50,0 08(0 03 to 0 14)

United_Arab_Emirates,Prevalence,50 ~ 55,0 1(0 04 to 0 16)

United_Arab_Emirates,Prevalence,55 ~ 60,0 16(0 09 to 0 23)

United_Arab_Emirates,Prevalence,60 ~ 65,0 24(0 15 to 0 33)

United_Arab_Emirates,Prevalence,65 ~ 70,0 36(0 26 to 0 46)

United_Arab_Emirates,Prevalence,70 ~ 75,0 47(0 34 to 0 6)

United_Arab_Emirates,Prevalence,75 ~ 80,0 59(0 43 to 0 75)

United_Arab_Emirates,Prevalence,80 ~ 85,0 71(0 52 to 0 91)

United_Arab_Emirates,Prevalence,85 ~ 90,0 86(0 52 to 1 19)

United_Arab_Emirates,Prevalence,90 ~ 95,1 05(0 33 to 1 77)

United_Arab_Emirates,Prevalence,20 ~ 25,-0 07(-0 2 to 0 06)

United_Arab_Emirates,Prevalence,25 ~ 30,-0 01(-0 1 to 0 08)

United_Arab_Emirates,Prevalence,30 ~ 35,0 05(0 to 0 11)

United_Arab_Emirates,Prevalence,35 ~ 40,0 05(0 to 0 1)

United_Arab_Emirates,Prevalence,40 ~ 45,0 05(0 to 0 1)

United_Arab_Emirates,Prevalence,45 ~ 50,0 08(0 03 to 0 14)

United_Arab_Emirates,Prevalence,50 ~ 55,0 1(0 04 to 0 16)

United_Arab_Emirates,Prevalence,55 ~ 60,0 16(0 09 to 0 23)

United_Arab_Emirates,Prevalence,60 ~ 65,0 24(0 15 to 0 33)

United_Arab_Emirates,Prevalence,65 ~ 70,0 36(0 26 to 0 46)

United_Arab_Emirates,Prevalence,70 ~ 75,0 47(0 34 to 0 6)

United_Arab_Emirates,Prevalence,75 ~ 80,0 59(0 43 to 0 75)

United_Arab_Emirates,Prevalence,80 ~ 85,0 71(0 52 to 0 91)

United_Arab_Emirates,Prevalence,85 ~ 90,0 86(0 52 to 1 19)

United_Arab_Emirates,Prevalence,90 ~ 95,1 05(0 33 to 1 77)

United_Arab_Emirates,Deaths,20 ~ 25,1 3(-1 94 to 4 64)

United_Arab_Emirates,Deaths,25 ~ 30,-0 43(-2 49 to 1 68)

United_Arab_Emirates,Deaths,30 ~ 35,-3 13(-4 56 to -1 68)

United_Arab_Emirates,Deaths,35 ~ 40,-3 99(-5 09 to -2 89)

United_Arab_Emirates,Deaths,40 ~ 45,-4 42(-5 33 to -3 49)

United_Arab_Emirates,Deaths,45 ~ 50,-4 79(-5 6 to -3 97)

United_Arab_Emirates,Deaths,50 ~ 55,-4 66(-5 42 to -3 88)

United_Arab_Emirates,Deaths,55 ~ 60,-4 14(-4 9 to -3 37)

United_Arab_Emirates,Deaths,60 ~ 65,-3 25(-4 02 to -2 47)

United_Arab_Emirates,Deaths,65 ~ 70,-2 12(-2 93 to -1 31)

United_Arab_Emirates,Deaths,70 ~ 75,-0 32(-1 24 to 0 62)

United_Arab_Emirates,Deaths,75 ~ 80,0 79(-0 27 to 1 86)

United_Arab_Emirates,Deaths,80 ~ 85,1 41(0 18 to 2 66)

United_Arab_Emirates,Deaths,85 ~ 90,1 77(-0 26 to 3 85)

United_Arab_Emirates,Deaths,90 ~ 95,1 96(-2 2 to 6 3)

United_Arab_Emirates,Deaths,20 ~ 25,1 3(-1 94 to 4 64)

United_Arab_Emirates,Deaths,25 ~ 30,-0 43(-2 49 to 1 68)

United_Arab_Emirates,Deaths,30 ~ 35,-3 13(-4 56 to -1 68)

United_Arab_Emirates,Deaths,35 ~ 40,-3 99(-5 09 to -2 89)

United_Arab_Emirates,Deaths,40 ~ 45,-4 42(-5 33 to -3 49)

United_Arab_Emirates,Deaths,45 ~ 50,-4 79(-5 6 to -3 97)

United_Arab_Emirates,Deaths,50 ~ 55,-4 66(-5 42 to -3 88)

United_Arab_Emirates,Deaths,55 ~ 60,-4 14(-4 9 to -3 37)

United_Arab_Emirates,Deaths,60 ~ 65,-3 25(-4 02 to -2 47)

United_Arab_Emirates,Deaths,65 ~ 70,-2 12(-2 93 to -1 31)

United_Arab_Emirates,Deaths,70 ~ 75,-0 32(-1 24 to 0 62)

United_Arab_Emirates,Deaths,75 ~ 80,0 79(-0 27 to 1 86)

United_Arab_Emirates,Deaths,80 ~ 85,1 41(0 18 to 2 66)

United_Arab_Emirates,Deaths,85 ~ 90,1 77(-0 26 to 3 85)

United_Arab_Emirates,Deaths,90 ~ 95,1 96(-2 2 to 6 3)

United_Arab_Emirates,Deaths,20 ~ 25,1 3(-1 94 to 4 64)

United_Arab_Emirates,Deaths,25 ~ 30,-0 43(-2 49 to 1 68)

United_Arab_Emirates,Deaths,30 ~ 35,-3 13(-4 56 to -1 68)

United_Arab_Emirates,Deaths,35 ~ 40,-3 99(-5 09 to -2 89)

United_Arab_Emirates,Deaths,40 ~ 45,-4 42(-5 33 to -3 49)

United_Arab_Emirates,Deaths,45 ~ 50,-4 79(-5 6 to -3 97)

United_Arab_Emirates,Deaths,50 ~ 55,-4 66(-5 42 to -3 88)

United_Arab_Emirates,Deaths,55 ~ 60,-4 14(-4 9 to -3 37)

United_Arab_Emirates,Deaths,60 ~ 65,-3 25(-4 02 to -2 47)

United_Arab_Emirates,Deaths,65 ~ 70,-2 12(-2 93 to -1 31)

United_Arab_Emirates,Deaths,70 ~ 75,-0 32(-1 24 to 0 62)

United_Arab_Emirates,Deaths,75 ~ 80,0 79(-0 27 to 1 86)

United_Arab_Emirates,Deaths,80 ~ 85,1 41(0 18 to 2 66)

United_Arab_Emirates,Deaths,85 ~ 90,1 77(-0 26 to 3 85)

United_Arab_Emirates,Deaths,90 ~ 95,1 96(-2 2 to 6 3)

United_Arab_Emirates,Deaths,20 ~ 25,1 3(-1 94 to 4 64)

United_Arab_Emirates,Deaths,25 ~ 30,-0 43(-2 49 to 1 68)

United_Arab_Emirates,Deaths,30 ~ 35,-3 13(-4 56 to -1 68)

United_Arab_Emirates,Deaths,35 ~ 40,-3 99(-5 09 to -2 89)

United_Arab_Emirates,Deaths,40 ~ 45,-4 42(-5 33 to -3 49)

United_Arab_Emirates,Deaths,45 ~ 50,-4 79(-5 6 to -3 97)

United_Arab_Emirates,Deaths,50 ~ 55,-4 66(-5 42 to -3 88)

United_Arab_Emirates,Deaths,55 ~ 60,-4 14(-4 9 to -3 37)

United_Arab_Emirates,Deaths,60 ~ 65,-3 25(-4 02 to -2 47)

United_Arab_Emirates,Deaths,65 ~ 70,-2 12(-2 93 to -1 31)

United_Arab_Emirates,Deaths,70 ~ 75,-0 32(-1 24 to 0 62)

United_Arab_Emirates,Deaths,75 ~ 80,0 79(-0 27 to 1 86)

United_Arab_Emirates,Deaths,80 ~ 85,1 41(0 18 to 2 66)

United_Arab_Emirates,Deaths,85 ~ 90,1 77(-0 26 to 3 85)

United_Arab_Emirates,Deaths,90 ~ 95,1 96(-2 2 to 6 3)

United_Arab_Emirates,DALYs,20 ~ 25,1 08(0 05 to 2 13)

United_Arab_Emirates,DALYs,25 ~ 30,0 12(-0 57 to 0 81)

United_Arab_Emirates,DALYs,30 ~ 35,-1 54(-2 01 to -1 07)

United_Arab_Emirates,DALYs,35 ~ 40,-2 14(-2 53 to -1 75)

United_Arab_Emirates,DALYs,40 ~ 45,-2 59(-2 95 to -2 24)

United_Arab_Emirates,DALYs,45 ~ 50,-3 07(-3 42 to -2 72)

United_Arab_Emirates,DALYs,50 ~ 55,-3 22(-3 58 to -2 86)

United_Arab_Emirates,DALYs,55 ~ 60,-3 05(-3 44 to -2 65)

United_Arab_Emirates,DALYs,60 ~ 65,-2 54(-2 98 to -2 1)

United_Arab_Emirates,DALYs,65 ~ 70,-1 7(-2 21 to -1 19)

United_Arab_Emirates,DALYs,70 ~ 75,-0 21(-0 87 to 0 45)

United_Arab_Emirates,DALYs,75 ~ 80,0 81(-0 01 to 1 64)

United_Arab_Emirates,DALYs,80 ~ 85,1 37(0 33 to 2 41)

United_Arab_Emirates,DALYs,85 ~ 90,1 64(-0 22 to 3 55)

United_Arab_Emirates,DALYs,90 ~ 95,1 74(-2 35 to 6 01)

United_Arab_Emirates,DALYs,20 ~ 25,1 08(0 05 to 2 13)

United_Arab_Emirates,DALYs,25 ~ 30,0 12(-0 57 to 0 81)

United_Arab_Emirates,DALYs,30 ~ 35,-1 54(-2 01 to -1 07)

United_Arab_Emirates,DALYs,35 ~ 40,-2 14(-2 53 to -1 75)

United_Arab_Emirates,DALYs,40 ~ 45,-2 59(-2 95 to -2 24)

United_Arab_Emirates,DALYs,45 ~ 50,-3 07(-3 42 to -2 72)

United_Arab_Emirates,DALYs,50 ~ 55,-3 22(-3 58 to -2 86)

United_Arab_Emirates,DALYs,55 ~ 60,-3 05(-3 44 to -2 65)

United_Arab_Emirates,DALYs,60 ~ 65,-2 54(-2 98 to -2 1)

United_Arab_Emirates,DALYs,65 ~ 70,-1 7(-2 21 to -1 19)

United_Arab_Emirates,DALYs,70 ~ 75,-0 21(-0 87 to 0 45)

United_Arab_Emirates,DALYs,75 ~ 80,0 81(-0 01 to 1 64)

United_Arab_Emirates,DALYs,80 ~ 85,1 37(0 33 to 2 41)

United_Arab_Emirates,DALYs,85 ~ 90,1 64(-0 22 to 3 55)

United_Arab_Emirates,DALYs,90 ~ 95,1 74(-2 35 to 6 01)

United_Arab_Emirates,DALYs,20 ~ 25,1 08(0 05 to 2 13)

United_Arab_Emirates,DALYs,25 ~ 30,0 12(-0 57 to 0 81)

United_Arab_Emirates,DALYs,30 ~ 35,-1 54(-2 01 to -1 07)

United_Arab_Emirates,DALYs,35 ~ 40,-2 14(-2 53 to -1 75)

United_Arab_Emirates,DALYs,40 ~ 45,-2 59(-2 95 to -2 24)

United_Arab_Emirates,DALYs,45 ~ 50,-3 07(-3 42 to -2 72)

United_Arab_Emirates,DALYs,50 ~ 55,-3 22(-3 58 to -2 86)

United_Arab_Emirates,DALYs,55 ~ 60,-3 05(-3 44 to -2 65)

United_Arab_Emirates,DALYs,60 ~ 65,-2 54(-2 98 to -2 1)

United_Arab_Emirates,DALYs,65 ~ 70,-1 7(-2 21 to -1 19)

United_Arab_Emirates,DALYs,70 ~ 75,-0 21(-0 87 to 0 45)

United_Arab_Emirates,DALYs,75 ~ 80,0 81(-0 01 to 1 64)

United_Arab_Emirates,DALYs,80 ~ 85,1 37(0 33 to 2 41)

United_Arab_Emirates,DALYs,85 ~ 90,1 64(-0 22 to 3 55)

United_Arab_Emirates,DALYs,90 ~ 95,1 74(-2 35 to 6 01)

United_Arab_Emirates,DALYs,20 ~ 25,1 08(0 05 to 2 13)

United_Arab_Emirates,DALYs,25 ~ 30,0 12(-0 57 to 0 81)

United_Arab_Emirates,DALYs,30 ~ 35,-1 54(-2 01 to -1 07)

United_Arab_Emirates,DALYs,35 ~ 40,-2 14(-2 53 to -1 75)

United_Arab_Emirates,DALYs,40 ~ 45,-2 59(-2 95 to -2 24)

United_Arab_Emirates,DALYs,45 ~ 50,-3 07(-3 42 to -2 72)

United_Arab_Emirates,DALYs,50 ~ 55,-3 22(-3 58 to -2 86)

United_Arab_Emirates,DALYs,55 ~ 60,-3 05(-3 44 to -2 65)

United_Arab_Emirates,DALYs,60 ~ 65,-2 54(-2 98 to -2 1)

United_Arab_Emirates,DALYs,65 ~ 70,-1 7(-2 21 to -1 19)

United_Arab_Emirates,DALYs,70 ~ 75,-0 21(-0 87 to 0 45)

United_Arab_Emirates,DALYs,75 ~ 80,0 81(-0 01 to 1 64)

United_Arab_Emirates,DALYs,80 ~ 85,1 37(0 33 to 2 41)

United_Arab_Emirates,DALYs,85 ~ 90,1 64(-0 22 to 3 55)

United_Arab_Emirates,DALYs,90 ~ 95,1 74(-2 35 to 6 01)

Indonesia,Prevalence,20 ~ 25,-0 27(-0 34 to -0 2)

Indonesia,Prevalence,25 ~ 30,-0 24(-0 29 to -0 18)

Indonesia,Prevalence,30 ~ 35,-0 2(-0 24 to -0 16)

Indonesia,Prevalence,35 ~ 40,-0 17(-0 21 to -0 13)

Indonesia,Prevalence,40 ~ 45,-0 14(-0 18 to -0 11)

Indonesia,Prevalence,45 ~ 50,-0 1(-0 13 to -0 07)

Indonesia,Prevalence,50 ~ 55,-0 03(-0 06 to 0)

Indonesia,Prevalence,55 ~ 60,0 07(0 04 to 0 09)

Indonesia,Prevalence,60 ~ 65,0 18(0 15 to 0 21)

Indonesia,Prevalence,65 ~ 70,0 31(0 28 to 0 33)

Indonesia,Prevalence,70 ~ 75,0 44(0 41 to 0 47)

Indonesia,Prevalence,75 ~ 80,0 57(0 54 to 0 61)

Indonesia,Prevalence,80 ~ 85,0 72(0 67 to 0 77)

Indonesia,Prevalence,85 ~ 90,0 88(0 81 to 0 95)

Indonesia,Prevalence,90 ~ 95,1 01(0 88 to 1 15)

Indonesia,Prevalence,20 ~ 25,-0 27(-0 34 to -0 2)

Indonesia,Prevalence,25 ~ 30,-0 24(-0 29 to -0 18)

Indonesia,Prevalence,30 ~ 35,-0 2(-0 24 to -0 16)

Indonesia,Prevalence,35 ~ 40,-0 17(-0 21 to -0 13)

Indonesia,Prevalence,40 ~ 45,-0 14(-0 18 to -0 11)

Indonesia,Prevalence,45 ~ 50,-0 1(-0 13 to -0 07)

Indonesia,Prevalence,50 ~ 55,-0 03(-0 06 to 0)

Indonesia,Prevalence,55 ~ 60,0 07(0 04 to 0 09)

Indonesia,Prevalence,60 ~ 65,0 18(0 15 to 0 21)

Indonesia,Prevalence,65 ~ 70,0 31(0 28 to 0 33)

Indonesia,Prevalence,70 ~ 75,0 44(0 41 to 0 47)

Indonesia,Prevalence,75 ~ 80,0 57(0 54 to 0 61)

Indonesia,Prevalence,80 ~ 85,0 72(0 67 to 0 77)

Indonesia,Prevalence,85 ~ 90,0 88(0 81 to 0 95)

Indonesia,Prevalence,90 ~ 95,1 01(0 88 to 1 15)

Indonesia,Prevalence,20 ~ 25,-0 27(-0 34 to -0 2)

Indonesia,Prevalence,25 ~ 30,-0 24(-0 29 to -0 18)

Indonesia,Prevalence,30 ~ 35,-0 2(-0 24 to -0 16)

Indonesia,Prevalence,35 ~ 40,-0 17(-0 21 to -0 13)

Indonesia,Prevalence,40 ~ 45,-0 14(-0 18 to -0 11)

Indonesia,Prevalence,45 ~ 50,-0 1(-0 13 to -0 07)

Indonesia,Prevalence,50 ~ 55,-0 03(-0 06 to 0)

Indonesia,Prevalence,55 ~ 60,0 07(0 04 to 0 09)

Indonesia,Prevalence,60 ~ 65,0 18(0 15 to 0 21)

Indonesia,Prevalence,65 ~ 70,0 31(0 28 to 0 33)

Indonesia,Prevalence,70 ~ 75,0 44(0 41 to 0 47)

Indonesia,Prevalence,75 ~ 80,0 57(0 54 to 0 61)

Indonesia,Prevalence,80 ~ 85,0 72(0 67 to 0 77)

Indonesia,Prevalence,85 ~ 90,0 88(0 81 to 0 95)

Indonesia,Prevalence,90 ~ 95,1 01(0 88 to 1 15)

Indonesia,Prevalence,20 ~ 25,-0 27(-0 34 to -0 2)

Indonesia,Prevalence,25 ~ 30,-0 24(-0 29 to -0 18)

Indonesia,Prevalence,30 ~ 35,-0 2(-0 24 to -0 16)

Indonesia,Prevalence,35 ~ 40,-0 17(-0 21 to -0 13)

Indonesia,Prevalence,40 ~ 45,-0 14(-0 18 to -0 11)

Indonesia,Prevalence,45 ~ 50,-0 1(-0 13 to -0 07)

Indonesia,Prevalence,50 ~ 55,-0 03(-0 06 to 0)

Indonesia,Prevalence,55 ~ 60,0 07(0 04 to 0 09)

Indonesia,Prevalence,60 ~ 65,0 18(0 15 to 0 21)

Indonesia,Prevalence,65 ~ 70,0 31(0 28 to 0 33)

Indonesia,Prevalence,70 ~ 75,0 44(0 41 to 0 47)

Indonesia,Prevalence,75 ~ 80,0 57(0 54 to 0 61)

Indonesia,Prevalence,80 ~ 85,0 72(0 67 to 0 77)

Indonesia,Prevalence,85 ~ 90,0 88(0 81 to 0 95)

Indonesia,Prevalence,90 ~ 95,1 01(0 88 to 1 15)

Indonesia,Deaths,20 ~ 25,-0 97(-1 34 to -0 59)

Indonesia,Deaths,25 ~ 30,-0 92(-1 19 to -0 65)

Indonesia,Deaths,30 ~ 35,-0 85(-1 06 to -0 63)

Indonesia,Deaths,35 ~ 40,-0 81(-0 98 to -0 63)

Indonesia,Deaths,40 ~ 45,-0 76(-0 9 to -0 61)

Indonesia,Deaths,45 ~ 50,-0 69(-0 8 to -0 57)

Indonesia,Deaths,50 ~ 55,-0 62(-0 71 to -0 53)

Indonesia,Deaths,55 ~ 60,-0 55(-0 62 to -0 48)

Indonesia,Deaths,60 ~ 65,-0 51(-0 57 to -0 45)

Indonesia,Deaths,65 ~ 70,-0 51(-0 56 to -0 46)

Indonesia,Deaths,70 ~ 75,-0 51(-0 56 to -0 46)

Indonesia,Deaths,75 ~ 80,-0 42(-0 47 to -0 37)

Indonesia,Deaths,80 ~ 85,-0 22(-0 28 to -0 16)

Indonesia,Deaths,85 ~ 90,0 15(0 07 to 0 23)

Indonesia,Deaths,90 ~ 95,0 57(0 42 to 0 72)

Indonesia,Deaths,20 ~ 25,-0 97(-1 34 to -0 59)

Indonesia,Deaths,25 ~ 30,-0 92(-1 19 to -0 65)

Indonesia,Deaths,30 ~ 35,-0 85(-1 06 to -0 63)

Indonesia,Deaths,35 ~ 40,-0 81(-0 98 to -0 63)

Indonesia,Deaths,40 ~ 45,-0 76(-0 9 to -0 61)

Indonesia,Deaths,45 ~ 50,-0 69(-0 8 to -0 57)

Indonesia,Deaths,50 ~ 55,-0 62(-0 71 to -0 53)

Indonesia,Deaths,55 ~ 60,-0 55(-0 62 to -0 48)

Indonesia,Deaths,60 ~ 65,-0 51(-0 57 to -0 45)

Indonesia,Deaths,65 ~ 70,-0 51(-0 56 to -0 46)

Indonesia,Deaths,70 ~ 75,-0 51(-0 56 to -0 46)

Indonesia,Deaths,75 ~ 80,-0 42(-0 47 to -0 37)

Indonesia,Deaths,80 ~ 85,-0 22(-0 28 to -0 16)

Indonesia,Deaths,85 ~ 90,0 15(0 07 to 0 23)

Indonesia,Deaths,90 ~ 95,0 57(0 42 to 0 72)

Indonesia,Deaths,20 ~ 25,-0 97(-1 34 to -0 59)

Indonesia,Deaths,25 ~ 30,-0 92(-1 19 to -0 65)

Indonesia,Deaths,30 ~ 35,-0 85(-1 06 to -0 63)

Indonesia,Deaths,35 ~ 40,-0 81(-0 98 to -0 63)

Indonesia,Deaths,40 ~ 45,-0 76(-0 9 to -0 61)

Indonesia,Deaths,45 ~ 50,-0 69(-0 8 to -0 57)

Indonesia,Deaths,50 ~ 55,-0 62(-0 71 to -0 53)

Indonesia,Deaths,55 ~ 60,-0 55(-0 62 to -0 48)

Indonesia,Deaths,60 ~ 65,-0 51(-0 57 to -0 45)

Indonesia,Deaths,65 ~ 70,-0 51(-0 56 to -0 46)

Indonesia,Deaths,70 ~ 75,-0 51(-0 56 to -0 46)

Indonesia,Deaths,75 ~ 80,-0 42(-0 47 to -0 37)

Indonesia,Deaths,80 ~ 85,-0 22(-0 28 to -0 16)

Indonesia,Deaths,85 ~ 90,0 15(0 07 to 0 23)

Indonesia,Deaths,90 ~ 95,0 57(0 42 to 0 72)

Indonesia,Deaths,20 ~ 25,-0 97(-1 34 to -0 59)

Indonesia,Deaths,25 ~ 30,-0 92(-1 19 to -0 65)

Indonesia,Deaths,30 ~ 35,-0 85(-1 06 to -0 63)

Indonesia,Deaths,35 ~ 40,-0 81(-0 98 to -0 63)

Indonesia,Deaths,40 ~ 45,-0 76(-0 9 to -0 61)

Indonesia,Deaths,45 ~ 50,-0 69(-0 8 to -0 57)

Indonesia,Deaths,50 ~ 55,-0 62(-0 71 to -0 53)

Indonesia,Deaths,55 ~ 60,-0 55(-0 62 to -0 48)

Indonesia,Deaths,60 ~ 65,-0 51(-0 57 to -0 45)

Indonesia,Deaths,65 ~ 70,-0 51(-0 56 to -0 46)

Indonesia,Deaths,70 ~ 75,-0 51(-0 56 to -0 46)

Indonesia,Deaths,75 ~ 80,-0 42(-0 47 to -0 37)

Indonesia,Deaths,80 ~ 85,-0 22(-0 28 to -0 16)

Indonesia,Deaths,85 ~ 90,0 15(0 07 to 0 23)

Indonesia,Deaths,90 ~ 95,0 57(0 42 to 0 72)

Indonesia,DALYs,20 ~ 25,-0 7(-0 86 to -0 54)

Indonesia,DALYs,25 ~ 30,-0 67(-0 79 to -0 55)

Indonesia,DALYs,30 ~ 35,-0 63(-0 72 to -0 53)

Indonesia,DALYs,35 ~ 40,-0 61(-0 69 to -0 52)

Indonesia,DALYs,40 ~ 45,-0 58(-0 66 to -0 51)

Indonesia,DALYs,45 ~ 50,-0 54(-0 6 to -0 48)

Indonesia,DALYs,50 ~ 55,-0 5(-0 55 to -0 45)

Indonesia,DALYs,55 ~ 60,-0 45(-0 5 to -0 4)

Indonesia,DALYs,60 ~ 65,-0 42(-0 46 to -0 38)

Indonesia,DALYs,65 ~ 70,-0 41(-0 45 to -0 37)

Indonesia,DALYs,70 ~ 75,-0 41(-0 45 to -0 36)

Indonesia,DALYs,75 ~ 80,-0 32(-0 37 to -0 27)

Indonesia,DALYs,80 ~ 85,-0 13(-0 19 to -0 06)

Indonesia,DALYs,85 ~ 90,0 23(0 13 to 0 33)

Indonesia,DALYs,90 ~ 95,0 62(0 43 to 0 82)

Indonesia,DALYs,20 ~ 25,-0 7(-0 86 to -0 54)

Indonesia,DALYs,25 ~ 30,-0 67(-0 79 to -0 55)

Indonesia,DALYs,30 ~ 35,-0 63(-0 72 to -0 53)

Indonesia,DALYs,35 ~ 40,-0 61(-0 69 to -0 52)

Indonesia,DALYs,40 ~ 45,-0 58(-0 66 to -0 51)

Indonesia,DALYs,45 ~ 50,-0 54(-0 6 to -0 48)

Indonesia,DALYs,50 ~ 55,-0 5(-0 55 to -0 45)

Indonesia,DALYs,55 ~ 60,-0 45(-0 5 to -0 4)

Indonesia,DALYs,60 ~ 65,-0 42(-0 46 to -0 38)

Indonesia,DALYs,65 ~ 70,-0 41(-0 45 to -0 37)

Indonesia,DALYs,70 ~ 75,-0 41(-0 45 to -0 36)

Indonesia,DALYs,75 ~ 80,-0 32(-0 37 to -0 27)

Indonesia,DALYs,80 ~ 85,-0 13(-0 19 to -0 06)

Indonesia,DALYs,85 ~ 90,0 23(0 13 to 0 33)

Indonesia,DALYs,90 ~ 95,0 62(0 43 to 0 82)

Indonesia,DALYs,20 ~ 25,-0 7(-0 86 to -0 54)

Indonesia,DALYs,25 ~ 30,-0 67(-0 79 to -0 55)

Indonesia,DALYs,30 ~ 35,-0 63(-0 72 to -0 53)

Indonesia,DALYs,35 ~ 40,-0 61(-0 69 to -0 52)

Indonesia,DALYs,40 ~ 45,-0 58(-0 66 to -0 51)

Indonesia,DALYs,45 ~ 50,-0 54(-0 6 to -0 48)

Indonesia,DALYs,50 ~ 55,-0 5(-0 55 to -0 45)

Indonesia,DALYs,55 ~ 60,-0 45(-0 5 to -0 4)

Indonesia,DALYs,60 ~ 65,-0 42(-0 46 to -0 38)

Indonesia,DALYs,65 ~ 70,-0 41(-0 45 to -0 37)

Indonesia,DALYs,70 ~ 75,-0 41(-0 45 to -0 36)

Indonesia,DALYs,75 ~ 80,-0 32(-0 37 to -0 27)

Indonesia,DALYs,80 ~ 85,-0 13(-0 19 to -0 06)

Indonesia,DALYs,85 ~ 90,0 23(0 13 to 0 33)

Indonesia,DALYs,90 ~ 95,0 62(0 43 to 0 82)

Indonesia,DALYs,20 ~ 25,-0 7(-0 86 to -0 54)

Indonesia,DALYs,25 ~ 30,-0 67(-0 79 to -0 55)

Indonesia,DALYs,30 ~ 35,-0 63(-0 72 to -0 53)

Indonesia,DALYs,35 ~ 40,-0 61(-0 69 to -0 52)

Indonesia,DALYs,40 ~ 45,-0 58(-0 66 to -0 51)

Indonesia,DALYs,45 ~ 50,-0 54(-0 6 to -0 48)

Indonesia,DALYs,50 ~ 55,-0 5(-0 55 to -0 45)

Indonesia,DALYs,55 ~ 60,-0 45(-0 5 to -0 4)

Indonesia,DALYs,60 ~ 65,-0 42(-0 46 to -0 38)

Indonesia,DALYs,65 ~ 70,-0 41(-0 45 to -0 37)

Indonesia,DALYs,70 ~ 75,-0 41(-0 45 to -0 36)

Indonesia,DALYs,75 ~ 80,-0 32(-0 37 to -0 27)

Indonesia,DALYs,80 ~ 85,-0 13(-0 19 to -0 06)

Indonesia,DALYs,85 ~ 90,0 23(0 13 to 0 33)

Indonesia,DALYs,90 ~ 95,0 62(0 43 to 0 82)

Malaysia,Prevalence,20 ~ 25,0 1(0 03 to 0 17)

Malaysia,Prevalence,25 ~ 30,0 1(0 05 to 0 14)

Malaysia,Prevalence,30 ~ 35,0 08(0 04 to 0 12)

Malaysia,Prevalence,35 ~ 40,0 06(0 02 to 0 09)

Malaysia,Prevalence,40 ~ 45,0 03(0 to 0 07)

Malaysia,Prevalence,45 ~ 50,0(-0 03 to 0 03)

Malaysia,Prevalence,50 ~ 55,-0 03(-0 06 to 0)

Malaysia,Prevalence,55 ~ 60,-0 05(-0 08 to -0 03)

Malaysia,Prevalence,60 ~ 65,-0 05(-0 08 to -0 03)

Malaysia,Prevalence,65 ~ 70,-0 03(-0 05 to 0)

Malaysia,Prevalence,70 ~ 75,0 01(-0 02 to 0 04)

Malaysia,Prevalence,75 ~ 80,0 07(0 04 to 0 11)

Malaysia,Prevalence,80 ~ 85,0 17(0 13 to 0 21)

Malaysia,Prevalence,85 ~ 90,0 31(0 26 to 0 36)

Malaysia,Prevalence,90 ~ 95,0 49(0 4 to 0 58)

Malaysia,Prevalence,20 ~ 25,0 1(0 03 to 0 17)

Malaysia,Prevalence,25 ~ 30,0 1(0 05 to 0 14)

Malaysia,Prevalence,30 ~ 35,0 08(0 04 to 0 12)

Malaysia,Prevalence,35 ~ 40,0 06(0 02 to 0 09)

Malaysia,Prevalence,40 ~ 45,0 03(0 to 0 07)

Malaysia,Prevalence,45 ~ 50,0(-0 03 to 0 03)

Malaysia,Prevalence,50 ~ 55,-0 03(-0 06 to 0)

Malaysia,Prevalence,55 ~ 60,-0 05(-0 08 to -0 03)

Malaysia,Prevalence,60 ~ 65,-0 05(-0 08 to -0 03)

Malaysia,Prevalence,65 ~ 70,-0 03(-0 05 to 0)

Malaysia,Prevalence,70 ~ 75,0 01(-0 02 to 0 04)

Malaysia,Prevalence,75 ~ 80,0 07(0 04 to 0 11)

Malaysia,Prevalence,80 ~ 85,0 17(0 13 to 0 21)

Malaysia,Prevalence,85 ~ 90,0 31(0 26 to 0 36)

Malaysia,Prevalence,90 ~ 95,0 49(0 4 to 0 58)

Malaysia,Prevalence,20 ~ 25,0 1(0 03 to 0 17)

Malaysia,Prevalence,25 ~ 30,0 1(0 05 to 0 14)

Malaysia,Prevalence,30 ~ 35,0 08(0 04 to 0 12)

Malaysia,Prevalence,35 ~ 40,0 06(0 02 to 0 09)

Malaysia,Prevalence,40 ~ 45,0 03(0 to 0 07)

Malaysia,Prevalence,45 ~ 50,0(-0 03 to 0 03)

Malaysia,Prevalence,50 ~ 55,-0 03(-0 06 to 0)

Malaysia,Prevalence,55 ~ 60,-0 05(-0 08 to -0 03)

Malaysia,Prevalence,60 ~ 65,-0 05(-0 08 to -0 03)

Malaysia,Prevalence,65 ~ 70,-0 03(-0 05 to 0)

Malaysia,Prevalence,70 ~ 75,0 01(-0 02 to 0 04)

Malaysia,Prevalence,75 ~ 80,0 07(0 04 to 0 11)

Malaysia,Prevalence,80 ~ 85,0 17(0 13 to 0 21)

Malaysia,Prevalence,85 ~ 90,0 31(0 26 to 0 36)

Malaysia,Prevalence,90 ~ 95,0 49(0 4 to 0 58)

Malaysia,Prevalence,20 ~ 25,0 1(0 03 to 0 17)

Malaysia,Prevalence,25 ~ 30,0 1(0 05 to 0 14)

Malaysia,Prevalence,30 ~ 35,0 08(0 04 to 0 12)

Malaysia,Prevalence,35 ~ 40,0 06(0 02 to 0 09)

Malaysia,Prevalence,40 ~ 45,0 03(0 to 0 07)

Malaysia,Prevalence,45 ~ 50,0(-0 03 to 0 03)

Malaysia,Prevalence,50 ~ 55,-0 03(-0 06 to 0)

Malaysia,Prevalence,55 ~ 60,-0 05(-0 08 to -0 03)

Malaysia,Prevalence,60 ~ 65,-0 05(-0 08 to -0 03)

Malaysia,Prevalence,65 ~ 70,-0 03(-0 05 to 0)

Malaysia,Prevalence,70 ~ 75,0 01(-0 02 to 0 04)

Malaysia,Prevalence,75 ~ 80,0 07(0 04 to 0 11)

Malaysia,Prevalence,80 ~ 85,0 17(0 13 to 0 21)

Malaysia,Prevalence,85 ~ 90,0 31(0 26 to 0 36)

Malaysia,Prevalence,90 ~ 95,0 49(0 4 to 0 58)

Malaysia,Deaths,20 ~ 25,-1 22(-3 03 to 0 61)

Malaysia,Deaths,25 ~ 30,-0 83(-2 05 to 0 41)

Malaysia,Deaths,30 ~ 35,-0 49(-1 43 to 0 46)

Malaysia,Deaths,35 ~ 40,-0 16(-0 91 to 0 59)

Malaysia,Deaths,40 ~ 45,-0 04(-0 64 to 0 56)

Malaysia,Deaths,45 ~ 50,-0 3(-0 77 to 0 18)

Malaysia,Deaths,50 ~ 55,-0 92(-1 27 to -0 56)

Malaysia,Deaths,55 ~ 60,-1 53(-1 8 to -1 26)

Malaysia,Deaths,60 ~ 65,-2 07(-2 28 to -1 86)

Malaysia,Deaths,65 ~ 70,-2 47(-2 64 to -2 29)

Malaysia,Deaths,70 ~ 75,-2 77(-2 93 to -2 62)

Malaysia,Deaths,75 ~ 80,-2 47(-2 63 to -2 32)

Malaysia,Deaths,80 ~ 85,-1 77(-1 95 to -1 59)

Malaysia,Deaths,85 ~ 90,-1(-1 24 to -0 77)

Malaysia,Deaths,90 ~ 95,-0 26(-0 66 to 0 14)

Malaysia,Deaths,20 ~ 25,-1 22(-3 03 to 0 61)

Malaysia,Deaths,25 ~ 30,-0 83(-2 05 to 0 41)

Malaysia,Deaths,30 ~ 35,-0 49(-1 43 to 0 46)

Malaysia,Deaths,35 ~ 40,-0 16(-0 91 to 0 59)

Malaysia,Deaths,40 ~ 45,-0 04(-0 64 to 0 56)

Malaysia,Deaths,45 ~ 50,-0 3(-0 77 to 0 18)

Malaysia,Deaths,50 ~ 55,-0 92(-1 27 to -0 56)

Malaysia,Deaths,55 ~ 60,-1 53(-1 8 to -1 26)

Malaysia,Deaths,60 ~ 65,-2 07(-2 28 to -1 86)

Malaysia,Deaths,65 ~ 70,-2 47(-2 64 to -2 29)

Malaysia,Deaths,70 ~ 75,-2 77(-2 93 to -2 62)

Malaysia,Deaths,75 ~ 80,-2 47(-2 63 to -2 32)

Malaysia,Deaths,80 ~ 85,-1 77(-1 95 to -1 59)

Malaysia,Deaths,85 ~ 90,-1(-1 24 to -0 77)

Malaysia,Deaths,90 ~ 95,-0 26(-0 66 to 0 14)

Malaysia,Deaths,20 ~ 25,-1 22(-3 03 to 0 61)

Malaysia,Deaths,25 ~ 30,-0 83(-2 05 to 0 41)

Malaysia,Deaths,30 ~ 35,-0 49(-1 43 to 0 46)

Malaysia,Deaths,35 ~ 40,-0 16(-0 91 to 0 59)

Malaysia,Deaths,40 ~ 45,-0 04(-0 64 to 0 56)

Malaysia,Deaths,45 ~ 50,-0 3(-0 77 to 0 18)

Malaysia,Deaths,50 ~ 55,-0 92(-1 27 to -0 56)

Malaysia,Deaths,55 ~ 60,-1 53(-1 8 to -1 26)

Malaysia,Deaths,60 ~ 65,-2 07(-2 28 to -1 86)

Malaysia,Deaths,65 ~ 70,-2 47(-2 64 to -2 29)

Malaysia,Deaths,70 ~ 75,-2 77(-2 93 to -2 62)

Malaysia,Deaths,75 ~ 80,-2 47(-2 63 to -2 32)

Malaysia,Deaths,80 ~ 85,-1 77(-1 95 to -1 59)

Malaysia,Deaths,85 ~ 90,-1(-1 24 to -0 77)

Malaysia,Deaths,90 ~ 95,-0 26(-0 66 to 0 14)

Malaysia,Deaths,20 ~ 25,-1 22(-3 03 to 0 61)

Malaysia,Deaths,25 ~ 30,-0 83(-2 05 to 0 41)

Malaysia,Deaths,30 ~ 35,-0 49(-1 43 to 0 46)

Malaysia,Deaths,35 ~ 40,-0 16(-0 91 to 0 59)

Malaysia,Deaths,40 ~ 45,-0 04(-0 64 to 0 56)

Malaysia,Deaths,45 ~ 50,-0 3(-0 77 to 0 18)

Malaysia,Deaths,50 ~ 55,-0 92(-1 27 to -0 56)

Malaysia,Deaths,55 ~ 60,-1 53(-1 8 to -1 26)

Malaysia,Deaths,60 ~ 65,-2 07(-2 28 to -1 86)

Malaysia,Deaths,65 ~ 70,-2 47(-2 64 to -2 29)

Malaysia,Deaths,70 ~ 75,-2 77(-2 93 to -2 62)

Malaysia,Deaths,75 ~ 80,-2 47(-2 63 to -2 32)

Malaysia,Deaths,80 ~ 85,-1 77(-1 95 to -1 59)

Malaysia,Deaths,85 ~ 90,-1(-1 24 to -0 77)

Malaysia,Deaths,90 ~ 95,-0 26(-0 66 to 0 14)

Malaysia,DALYs,20 ~ 25,-0 57(-1 1 to -0 04)

Malaysia,DALYs,25 ~ 30,-0 37(-0 75 to 0 01)

Malaysia,DALYs,30 ~ 35,-0 2(-0 51 to 0 11)

Malaysia,DALYs,35 ~ 40,-0 05(-0 31 to 0 21)

Malaysia,DALYs,40 ~ 45,0(-0 23 to 0 23)

Malaysia,DALYs,45 ~ 50,-0 19(-0 39 to 0)

Malaysia,DALYs,50 ~ 55,-0 69(-0 86 to -0 53)

Malaysia,DALYs,55 ~ 60,-1 22(-1 36 to -1 08)

Malaysia,DALYs,60 ~ 65,-1 73(-1 85 to -1 61)

Malaysia,DALYs,65 ~ 70,-2 12(-2 23 to -2)

Malaysia,DALYs,70 ~ 75,-2 43(-2 54 to -2 31)

Malaysia,DALYs,75 ~ 80,-2 17(-2 3 to -2 04)

Malaysia,DALYs,80 ~ 85,-1 51(-1 67 to -1 35)

Malaysia,DALYs,85 ~ 90,-0 81(-1 04 to -0 58)

Malaysia,DALYs,90 ~ 95,-0 12(-0 53 to 0 3)

Malaysia,DALYs,20 ~ 25,-0 57(-1 1 to -0 04)

Malaysia,DALYs,25 ~ 30,-0 37(-0 75 to 0 01)

Malaysia,DALYs,30 ~ 35,-0 2(-0 51 to 0 11)

Malaysia,DALYs,35 ~ 40,-0 05(-0 31 to 0 21)

Malaysia,DALYs,40 ~ 45,0(-0 23 to 0 23)

Malaysia,DALYs,45 ~ 50,-0 19(-0 39 to 0)

Malaysia,DALYs,50 ~ 55,-0 69(-0 86 to -0 53)

Malaysia,DALYs,55 ~ 60,-1 22(-1 36 to -1 08)

Malaysia,DALYs,60 ~ 65,-1 73(-1 85 to -1 61)

Malaysia,DALYs,65 ~ 70,-2 12(-2 23 to -2)

Malaysia,DALYs,70 ~ 75,-2 43(-2 54 to -2 31)

Malaysia,DALYs,75 ~ 80,-2 17(-2 3 to -2 04)

Malaysia,DALYs,80 ~ 85,-1 51(-1 67 to -1 35)

Malaysia,DALYs,85 ~ 90,-0 81(-1 04 to -0 58)

Malaysia,DALYs,90 ~ 95,-0 12(-0 53 to 0 3)

Malaysia,DALYs,20 ~ 25,-0 57(-1 1 to -0 04)

Malaysia,DALYs,25 ~ 30,-0 37(-0 75 to 0 01)

Malaysia,DALYs,30 ~ 35,-0 2(-0 51 to 0 11)

Malaysia,DALYs,35 ~ 40,-0 05(-0 31 to 0 21)

Malaysia,DALYs,40 ~ 45,0(-0 23 to 0 23)

Malaysia,DALYs,45 ~ 50,-0 19(-0 39 to 0)

Malaysia,DALYs,50 ~ 55,-0 69(-0 86 to -0 53)

Malaysia,DALYs,55 ~ 60,-1 22(-1 36 to -1 08)

Malaysia,DALYs,60 ~ 65,-1 73(-1 85 to -1 61)

Malaysia,DALYs,65 ~ 70,-2 12(-2 23 to -2)

Malaysia,DALYs,70 ~ 75,-2 43(-2 54 to -2 31)

Malaysia,DALYs,75 ~ 80,-2 17(-2 3 to -2 04)

Malaysia,DALYs,80 ~ 85,-1 51(-1 67 to -1 35)

Malaysia,DALYs,85 ~ 90,-0 81(-1 04 to -0 58)

Malaysia,DALYs,90 ~ 95,-0 12(-0 53 to 0 3)

Malaysia,DALYs,20 ~ 25,-0 57(-1 1 to -0 04)

Malaysia,DALYs,25 ~ 30,-0 37(-0 75 to 0 01)

Malaysia,DALYs,30 ~ 35,-0 2(-0 51 to 0 11)

Malaysia,DALYs,35 ~ 40,-0 05(-0 31 to 0 21)

Malaysia,DALYs,40 ~ 45,0(-0 23 to 0 23)

Malaysia,DALYs,45 ~ 50,-0 19(-0 39 to 0)

Malaysia,DALYs,50 ~ 55,-0 69(-0 86 to -0 53)

Malaysia,DALYs,55 ~ 60,-1 22(-1 36 to -1 08)

Malaysia,DALYs,60 ~ 65,-1 73(-1 85 to -1 61)

Malaysia,DALYs,65 ~ 70,-2 12(-2 23 to -2)

Malaysia,DALYs,70 ~ 75,-2 43(-2 54 to -2 31)

Malaysia,DALYs,75 ~ 80,-2 17(-2 3 to -2 04)

Malaysia,DALYs,80 ~ 85,-1 51(-1 67 to -1 35)

Malaysia,DALYs,85 ~ 90,-0 81(-1 04 to -0 58)

Malaysia,DALYs,90 ~ 95,-0 12(-0 53 to 0 3)

Bermuda,Prevalence,20 ~ 25,0 17(-1 72 to 2 1)

Bermuda,Prevalence,25 ~ 30,0 15(-1 12 to 1 44)

Bermuda,Prevalence,30 ~ 35,0 13(-0 82 to 1 09)

Bermuda,Prevalence,35 ~ 40,0 12(-0 64 to 0 88)

Bermuda,Prevalence,40 ~ 45,0 11(-0 51 to 0 74)

Bermuda,Prevalence,45 ~ 50,0 11(-0 41 to 0 64)

Bermuda,Prevalence,50 ~ 55,0 12(-0 31 to 0 56)

Bermuda,Prevalence,55 ~ 60,0 14(-0 25 to 0 52)

Bermuda,Prevalence,60 ~ 65,0 19(-0 16 to 0 53)

Bermuda,Prevalence,65 ~ 70,0 29(-0 04 to 0 62)

Bermuda,Prevalence,70 ~ 75,0 43(0 1 to 0 76)

Bermuda,Prevalence,75 ~ 80,0 6(0 24 to 0 96)

Bermuda,Prevalence,80 ~ 85,0 76(0 34 to 1 18)

Bermuda,Prevalence,85 ~ 90,0 89(0 31 to 1 47)

Bermuda,Prevalence,90 ~ 95,1 03(-0 11 to 2 17)

Bermuda,Prevalence,20 ~ 25,0 17(-1 72 to 2 1)

Bermuda,Prevalence,25 ~ 30,0 15(-1 12 to 1 44)

Bermuda,Prevalence,30 ~ 35,0 13(-0 82 to 1 09)

Bermuda,Prevalence,35 ~ 40,0 12(-0 64 to 0 88)

Bermuda,Prevalence,40 ~ 45,0 11(-0 51 to 0 74)

Bermuda,Prevalence,45 ~ 50,0 11(-0 41 to 0 64)

Bermuda,Prevalence,50 ~ 55,0 12(-0 31 to 0 56)

Bermuda,Prevalence,55 ~ 60,0 14(-0 25 to 0 52)

Bermuda,Prevalence,60 ~ 65,0 19(-0 16 to 0 53)

Bermuda,Prevalence,65 ~ 70,0 29(-0 04 to 0 62)

Bermuda,Prevalence,70 ~ 75,0 43(0 1 to 0 76)

Bermuda,Prevalence,75 ~ 80,0 6(0 24 to 0 96)

Bermuda,Prevalence,80 ~ 85,0 76(0 34 to 1 18)

Bermuda,Prevalence,85 ~ 90,0 89(0 31 to 1 47)

Bermuda,Prevalence,90 ~ 95,1 03(-0 11 to 2 17)

Bermuda,Prevalence,20 ~ 25,0 17(-1 72 to 2 1)

Bermuda,Prevalence,25 ~ 30,0 15(-1 12 to 1 44)

Bermuda,Prevalence,30 ~ 35,0 13(-0 82 to 1 09)

Bermuda,Prevalence,35 ~ 40,0 12(-0 64 to 0 88)

Bermuda,Prevalence,40 ~ 45,0 11(-0 51 to 0 74)

Bermuda,Prevalence,45 ~ 50,0 11(-0 41 to 0 64)

Bermuda,Prevalence,50 ~ 55,0 12(-0 31 to 0 56)

Bermuda,Prevalence,55 ~ 60,0 14(-0 25 to 0 52)

Bermuda,Prevalence,60 ~ 65,0 19(-0 16 to 0 53)

Bermuda,Prevalence,65 ~ 70,0 29(-0 04 to 0 62)

Bermuda,Prevalence,70 ~ 75,0 43(0 1 to 0 76)

Bermuda,Prevalence,75 ~ 80,0 6(0 24 to 0 96)

Bermuda,Prevalence,80 ~ 85,0 76(0 34 to 1 18)

Bermuda,Prevalence,85 ~ 90,0 89(0 31 to 1 47)

Bermuda,Prevalence,90 ~ 95,1 03(-0 11 to 2 17)

Bermuda,Prevalence,20 ~ 25,0 17(-1 72 to 2 1)

Bermuda,Prevalence,25 ~ 30,0 15(-1 12 to 1 44)

Bermuda,Prevalence,30 ~ 35,0 13(-0 82 to 1 09)

Bermuda,Prevalence,35 ~ 40,0 12(-0 64 to 0 88)

Bermuda,Prevalence,40 ~ 45,0 11(-0 51 to 0 74)

Bermuda,Prevalence,45 ~ 50,0 11(-0 41 to 0 64)

Bermuda,Prevalence,50 ~ 55,0 12(-0 31 to 0 56)

Bermuda,Prevalence,55 ~ 60,0 14(-0 25 to 0 52)

Bermuda,Prevalence,60 ~ 65,0 19(-0 16 to 0 53)

Bermuda,Prevalence,65 ~ 70,0 29(-0 04 to 0 62)

Bermuda,Prevalence,70 ~ 75,0 43(0 1 to 0 76)

Bermuda,Prevalence,75 ~ 80,0 6(0 24 to 0 96)

Bermuda,Prevalence,80 ~ 85,0 76(0 34 to 1 18)

Bermuda,Prevalence,85 ~ 90,0 89(0 31 to 1 47)

Bermuda,Prevalence,90 ~ 95,1 03(-0 11 to 2 17)

Bermuda,Deaths,20 ~ 25,0 15(-60 78 to 155 75)

Bermuda,Deaths,25 ~ 30,0 13(-45 42 to 83 69)

Bermuda,Deaths,30 ~ 35,-0 63(-38 8 to 61 36)

Bermuda,Deaths,35 ~ 40,-1 35(-32 58 to 44 35)

Bermuda,Deaths,40 ~ 45,-1 4(-24 1 to 28 1)

Bermuda,Deaths,45 ~ 50,-1 4(-16 89 to 16 99)

Bermuda,Deaths,50 ~ 55,-1 6(-12 24 to 10 33)

Bermuda,Deaths,55 ~ 60,-1 77(-9 26 to 6 34)

Bermuda,Deaths,60 ~ 65,-1 82(-7 37 to 4 07)

Bermuda,Deaths,65 ~ 70,-1 7(-6 21 to 3 02)

Bermuda,Deaths,70 ~ 75,-1 48(-5 36 to 2 55)

Bermuda,Deaths,75 ~ 80,-1 21(-4 72 to 2 44)

Bermuda,Deaths,80 ~ 85,-0 93(-4 43 to 2 71)

Bermuda,Deaths,85 ~ 90,-0 74(-4 99 to 3 7)

Bermuda,Deaths,90 ~ 95,-0 45(-8 13 to 7 87)

Bermuda,Deaths,20 ~ 25,0 15(-60 78 to 155 75)

Bermuda,Deaths,25 ~ 30,0 13(-45 42 to 83 69)

Bermuda,Deaths,30 ~ 35,-0 63(-38 8 to 61 36)

Bermuda,Deaths,35 ~ 40,-1 35(-32 58 to 44 35)

Bermuda,Deaths,40 ~ 45,-1 4(-24 1 to 28 1)

Bermuda,Deaths,45 ~ 50,-1 4(-16 89 to 16 99)

Bermuda,Deaths,50 ~ 55,-1 6(-12 24 to 10 33)

Bermuda,Deaths,55 ~ 60,-1 77(-9 26 to 6 34)

Bermuda,Deaths,60 ~ 65,-1 82(-7 37 to 4 07)

Bermuda,Deaths,65 ~ 70,-1 7(-6 21 to 3 02)

Bermuda,Deaths,70 ~ 75,-1 48(-5 36 to 2 55)

Bermuda,Deaths,75 ~ 80,-1 21(-4 72 to 2 44)

Bermuda,Deaths,80 ~ 85,-0 93(-4 43 to 2 71)

Bermuda,Deaths,85 ~ 90,-0 74(-4 99 to 3 7)

Bermuda,Deaths,90 ~ 95,-0 45(-8 13 to 7 87)

Bermuda,Deaths,20 ~ 25,0 15(-60 78 to 155 75)

Bermuda,Deaths,25 ~ 30,0 13(-45 42 to 83 69)

Bermuda,Deaths,30 ~ 35,-0 63(-38 8 to 61 36)

Bermuda,Deaths,35 ~ 40,-1 35(-32 58 to 44 35)

Bermuda,Deaths,40 ~ 45,-1 4(-24 1 to 28 1)

Bermuda,Deaths,45 ~ 50,-1 4(-16 89 to 16 99)

Bermuda,Deaths,50 ~ 55,-1 6(-12 24 to 10 33)

Bermuda,Deaths,55 ~ 60,-1 77(-9 26 to 6 34)

Bermuda,Deaths,60 ~ 65,-1 82(-7 37 to 4 07)

Bermuda,Deaths,65 ~ 70,-1 7(-6 21 to 3 02)

Bermuda,Deaths,70 ~ 75,-1 48(-5 36 to 2 55)

Bermuda,Deaths,75 ~ 80,-1 21(-4 72 to 2 44)

Bermuda,Deaths,80 ~ 85,-0 93(-4 43 to 2 71)

Bermuda,Deaths,85 ~ 90,-0 74(-4 99 to 3 7)

Bermuda,Deaths,90 ~ 95,-0 45(-8 13 to 7 87)

Bermuda,Deaths,20 ~ 25,0 15(-60 78 to 155 75)

Bermuda,Deaths,25 ~ 30,0 13(-45 42 to 83 69)

Bermuda,Deaths,30 ~ 35,-0 63(-38 8 to 61 36)

Bermuda,Deaths,35 ~ 40,-1 35(-32 58 to 44 35)

Bermuda,Deaths,40 ~ 45,-1 4(-24 1 to 28 1)

Bermuda,Deaths,45 ~ 50,-1 4(-16 89 to 16 99)

Bermuda,Deaths,50 ~ 55,-1 6(-12 24 to 10 33)

Bermuda,Deaths,55 ~ 60,-1 77(-9 26 to 6 34)

Bermuda,Deaths,60 ~ 65,-1 82(-7 37 to 4 07)

Bermuda,Deaths,65 ~ 70,-1 7(-6 21 to 3 02)

Bermuda,Deaths,70 ~ 75,-1 48(-5 36 to 2 55)

Bermuda,Deaths,75 ~ 80,-1 21(-4 72 to 2 44)

Bermuda,Deaths,80 ~ 85,-0 93(-4 43 to 2 71)

Bermuda,Deaths,85 ~ 90,-0 74(-4 99 to 3 7)

Bermuda,Deaths,90 ~ 95,-0 45(-8 13 to 7 87)

Bermuda,DALYs,20 ~ 25,0 07(-6 91 to 7 57)

Bermuda,DALYs,25 ~ 30,0 09(-4 63 to 5 04)

Bermuda,DALYs,30 ~ 35,-0 19(-3 86 to 3 62)

Bermuda,DALYs,35 ~ 40,-0 48(-3 41 to 2 53)

Bermuda,DALYs,40 ~ 45,-0 63(-2 93 to 1 73)

Bermuda,DALYs,45 ~ 50,-0 76(-2 53 to 1 05)

Bermuda,DALYs,50 ~ 55,-0 96(-2 33 to 0 43)

Bermuda,DALYs,55 ~ 60,-1 16(-2 25 to -0 06)

Bermuda,DALYs,60 ~ 65,-1 26(-2 17 to -0 35)

Bermuda,DALYs,65 ~ 70,-1 23(-2 04 to -0 41)

Bermuda,DALYs,70 ~ 75,-1 08(-1 86 to -0 3)

Bermuda,DALYs,75 ~ 80,-0 87(-1 68 to -0 07)

Bermuda,DALYs,80 ~ 85,-0 64(-1 56 to 0 29)

Bermuda,DALYs,85 ~ 90,-0 48(-1 72 to 0 78)

Bermuda,DALYs,90 ~ 95,-0 21(-2 61 to 2 26)

Bermuda,DALYs,20 ~ 25,0 07(-6 91 to 7 57)

Bermuda,DALYs,25 ~ 30,0 09(-4 63 to 5 04)

Bermuda,DALYs,30 ~ 35,-0 19(-3 86 to 3 62)

Bermuda,DALYs,35 ~ 40,-0 48(-3 41 to 2 53)

Bermuda,DALYs,40 ~ 45,-0 63(-2 93 to 1 73)

Bermuda,DALYs,45 ~ 50,-0 76(-2 53 to 1 05)

Bermuda,DALYs,50 ~ 55,-0 96(-2 33 to 0 43)

Bermuda,DALYs,55 ~ 60,-1 16(-2 25 to -0 06)

Bermuda,DALYs,60 ~ 65,-1 26(-2 17 to -0 35)

Bermuda,DALYs,65 ~ 70,-1 23(-2 04 to -0 41)

Bermuda,DALYs,70 ~ 75,-1 08(-1 86 to -0 3)

Bermuda,DALYs,75 ~ 80,-0 87(-1 68 to -0 07)

Bermuda,DALYs,80 ~ 85,-0 64(-1 56 to 0 29)

Bermuda,DALYs,85 ~ 90,-0 48(-1 72 to 0 78)

Bermuda,DALYs,90 ~ 95,-0 21(-2 61 to 2 26)

Bermuda,DALYs,20 ~ 25,0 07(-6 91 to 7 57)

Bermuda,DALYs,25 ~ 30,0 09(-4 63 to 5 04)

Bermuda,DALYs,30 ~ 35,-0 19(-3 86 to 3 62)

Bermuda,DALYs,35 ~ 40,-0 48(-3 41 to 2 53)

Bermuda,DALYs,40 ~ 45,-0 63(-2 93 to 1 73)

Bermuda,DALYs,45 ~ 50,-0 76(-2 53 to 1 05)

Bermuda,DALYs,50 ~ 55,-0 96(-2 33 to 0 43)

Bermuda,DALYs,55 ~ 60,-1 16(-2 25 to -0 06)

Bermuda,DALYs,60 ~ 65,-1 26(-2 17 to -0 35)

Bermuda,DALYs,65 ~ 70,-1 23(-2 04 to -0 41)

Bermuda,DALYs,70 ~ 75,-1 08(-1 86 to -0 3)

Bermuda,DALYs,75 ~ 80,-0 87(-1 68 to -0 07)

Bermuda,DALYs,80 ~ 85,-0 64(-1 56 to 0 29)

Bermuda,DALYs,85 ~ 90,-0 48(-1 72 to 0 78)

Bermuda,DALYs,90 ~ 95,-0 21(-2 61 to 2 26)

Bermuda,DALYs,20 ~ 25,0 07(-6 91 to 7 57)

Bermuda,DALYs,25 ~ 30,0 09(-4 63 to 5 04)

Bermuda,DALYs,30 ~ 35,-0 19(-3 86 to 3 62)

Bermuda,DALYs,35 ~ 40,-0 48(-3 41 to 2 53)

Bermuda,DALYs,40 ~ 45,-0 63(-2 93 to 1 73)

Bermuda,DALYs,45 ~ 50,-0 76(-2 53 to 1 05)

Bermuda,DALYs,50 ~ 55,-0 96(-2 33 to 0 43)

Bermuda,DALYs,55 ~ 60,-1 16(-2 25 to -0 06)

Bermuda,DALYs,60 ~ 65,-1 26(-2 17 to -0 35)

Bermuda,DALYs,65 ~ 70,-1 23(-2 04 to -0 41)

Bermuda,DALYs,70 ~ 75,-1 08(-1 86 to -0 3)

Bermuda,DALYs,75 ~ 80,-0 87(-1 68 to -0 07)

Bermuda,DALYs,80 ~ 85,-0 64(-1 56 to 0 29)

Bermuda,DALYs,85 ~ 90,-0 48(-1 72 to 0 78)

Bermuda,DALYs,90 ~ 95,-0 21(-2 61 to 2 26)

Netherlands,Prevalence,20 ~ 25,-0 65(-0 93 to -0 37)

Netherlands,Prevalence,25 ~ 30,-0 76(-0 95 to -0 57)

Netherlands,Prevalence,30 ~ 35,-0 79(-0 94 to -0 64)

Netherlands,Prevalence,35 ~ 40,-0 75(-0 87 to -0 62)

Netherlands,Prevalence,40 ~ 45,-0 66(-0 77 to -0 56)

Netherlands,Prevalence,45 ~ 50,-0 57(-0 65 to -0 48)

Netherlands,Prevalence,50 ~ 55,-0 46(-0 53 to -0 39)

Netherlands,Prevalence,55 ~ 60,-0 35(-0 41 to -0 29)

Netherlands,Prevalence,60 ~ 65,-0 24(-0 29 to -0 19)

Netherlands,Prevalence,65 ~ 70,-0 12(-0 17 to -0 08)

Netherlands,Prevalence,70 ~ 75,0 03(-0 02 to 0 07)

Netherlands,Prevalence,75 ~ 80,0 23(0 19 to 0 28)

Netherlands,Prevalence,80 ~ 85,0 49(0 43 to 0 54)

Netherlands,Prevalence,85 ~ 90,0 72(0 65 to 0 8)

Netherlands,Prevalence,90 ~ 95,0 91(0 77 to 1 04)

Netherlands,Prevalence,20 ~ 25,-0 65(-0 93 to -0 37)

Netherlands,Prevalence,25 ~ 30,-0 76(-0 95 to -0 57)

Netherlands,Prevalence,30 ~ 35,-0 79(-0 94 to -0 64)

Netherlands,Prevalence,35 ~ 40,-0 75(-0 87 to -0 62)

Netherlands,Prevalence,40 ~ 45,-0 66(-0 77 to -0 56)

Netherlands,Prevalence,45 ~ 50,-0 57(-0 65 to -0 48)

Netherlands,Prevalence,50 ~ 55,-0 46(-0 53 to -0 39)

Netherlands,Prevalence,55 ~ 60,-0 35(-0 41 to -0 29)

Netherlands,Prevalence,60 ~ 65,-0 24(-0 29 to -0 19)

Netherlands,Prevalence,65 ~ 70,-0 12(-0 17 to -0 08)

Netherlands,Prevalence,70 ~ 75,0 03(-0 02 to 0 07)

Netherlands,Prevalence,75 ~ 80,0 23(0 19 to 0 28)

Netherlands,Prevalence,80 ~ 85,0 49(0 43 to 0 54)

Netherlands,Prevalence,85 ~ 90,0 72(0 65 to 0 8)

Netherlands,Prevalence,90 ~ 95,0 91(0 77 to 1 04)

Netherlands,Prevalence,20 ~ 25,-0 65(-0 93 to -0 37)

Netherlands,Prevalence,25 ~ 30,-0 76(-0 95 to -0 57)

Netherlands,Prevalence,30 ~ 35,-0 79(-0 94 to -0 64)

Netherlands,Prevalence,35 ~ 40,-0 75(-0 87 to -0 62)

Netherlands,Prevalence,40 ~ 45,-0 66(-0 77 to -0 56)

Netherlands,Prevalence,45 ~ 50,-0 57(-0 65 to -0 48)

Netherlands,Prevalence,50 ~ 55,-0 46(-0 53 to -0 39)

Netherlands,Prevalence,55 ~ 60,-0 35(-0 41 to -0 29)

Netherlands,Prevalence,60 ~ 65,-0 24(-0 29 to -0 19)

Netherlands,Prevalence,65 ~ 70,-0 12(-0 17 to -0 08)

Netherlands,Prevalence,70 ~ 75,0 03(-0 02 to 0 07)

Netherlands,Prevalence,75 ~ 80,0 23(0 19 to 0 28)

Netherlands,Prevalence,80 ~ 85,0 49(0 43 to 0 54)

Netherlands,Prevalence,85 ~ 90,0 72(0 65 to 0 8)

Netherlands,Prevalence,90 ~ 95,0 91(0 77 to 1 04)

Netherlands,Prevalence,20 ~ 25,-0 65(-0 93 to -0 37)

Netherlands,Prevalence,25 ~ 30,-0 76(-0 95 to -0 57)

Netherlands,Prevalence,30 ~ 35,-0 79(-0 94 to -0 64)

Netherlands,Prevalence,35 ~ 40,-0 75(-0 87 to -0 62)

Netherlands,Prevalence,40 ~ 45,-0 66(-0 77 to -0 56)

Netherlands,Prevalence,45 ~ 50,-0 57(-0 65 to -0 48)

Netherlands,Prevalence,50 ~ 55,-0 46(-0 53 to -0 39)

Netherlands,Prevalence,55 ~ 60,-0 35(-0 41 to -0 29)

Netherlands,Prevalence,60 ~ 65,-0 24(-0 29 to -0 19)

Netherlands,Prevalence,65 ~ 70,-0 12(-0 17 to -0 08)

Netherlands,Prevalence,70 ~ 75,0 03(-0 02 to 0 07)

Netherlands,Prevalence,75 ~ 80,0 23(0 19 to 0 28)

Netherlands,Prevalence,80 ~ 85,0 49(0 43 to 0 54)

Netherlands,Prevalence,85 ~ 90,0 72(0 65 to 0 8)

Netherlands,Prevalence,90 ~ 95,0 91(0 77 to 1 04)

Netherlands,Deaths,20 ~ 25,-1 36(-5 34 to 2 79)

Netherlands,Deaths,25 ~ 30,-1 55(-4 4 to 1 38)

Netherlands,Deaths,30 ~ 35,-1 65(-3 81 to 0 57)

Netherlands,Deaths,35 ~ 40,-1 64(-3 31 to 0 05)

Netherlands,Deaths,40 ~ 45,-1 28(-2 46 to -0 08)

Netherlands,Deaths,45 ~ 50,-0 85(-1 62 to -0 09)

Netherlands,Deaths,50 ~ 55,-0 54(-1 03 to -0 05)

Netherlands,Deaths,55 ~ 60,-0 62(-0 96 to -0 28)

Netherlands,Deaths,60 ~ 65,-0 94(-1 18 to -0 7)

Netherlands,Deaths,65 ~ 70,-1 35(-1 54 to -1 17)

Netherlands,Deaths,70 ~ 75,-1 59(-1 74 to -1 45)

Netherlands,Deaths,75 ~ 80,-1 39(-1 52 to -1 27)

Netherlands,Deaths,80 ~ 85,-0 89(-1 01 to -0 77)

Netherlands,Deaths,85 ~ 90,-0 15(-0 3 to 0)

Netherlands,Deaths,90 ~ 95,0 39(0 15 to 0 63)

Netherlands,Deaths,20 ~ 25,-1 36(-5 34 to 2 79)

Netherlands,Deaths,25 ~ 30,-1 55(-4 4 to 1 38)

Netherlands,Deaths,30 ~ 35,-1 65(-3 81 to 0 57)

Netherlands,Deaths,35 ~ 40,-1 64(-3 31 to 0 05)

Netherlands,Deaths,40 ~ 45,-1 28(-2 46 to -0 08)

Netherlands,Deaths,45 ~ 50,-0 85(-1 62 to -0 09)

Netherlands,Deaths,50 ~ 55,-0 54(-1 03 to -0 05)

Netherlands,Deaths,55 ~ 60,-0 62(-0 96 to -0 28)

Netherlands,Deaths,60 ~ 65,-0 94(-1 18 to -0 7)

Netherlands,Deaths,65 ~ 70,-1 35(-1 54 to -1 17)

Netherlands,Deaths,70 ~ 75,-1 59(-1 74 to -1 45)

Netherlands,Deaths,75 ~ 80,-1 39(-1 52 to -1 27)

Netherlands,Deaths,80 ~ 85,-0 89(-1 01 to -0 77)

Netherlands,Deaths,85 ~ 90,-0 15(-0 3 to 0)

Netherlands,Deaths,90 ~ 95,0 39(0 15 to 0 63)

Netherlands,Deaths,20 ~ 25,-1 36(-5 34 to 2 79)

Netherlands,Deaths,25 ~ 30,-1 55(-4 4 to 1 38)

Netherlands,Deaths,30 ~ 35,-1 65(-3 81 to 0 57)

Netherlands,Deaths,35 ~ 40,-1 64(-3 31 to 0 05)

Netherlands,Deaths,40 ~ 45,-1 28(-2 46 to -0 08)

Netherlands,Deaths,45 ~ 50,-0 85(-1 62 to -0 09)

Netherlands,Deaths,50 ~ 55,-0 54(-1 03 to -0 05)

Netherlands,Deaths,55 ~ 60,-0 62(-0 96 to -0 28)

Netherlands,Deaths,60 ~ 65,-0 94(-1 18 to -0 7)

Netherlands,Deaths,65 ~ 70,-1 35(-1 54 to -1 17)

Netherlands,Deaths,70 ~ 75,-1 59(-1 74 to -1 45)

Netherlands,Deaths,75 ~ 80,-1 39(-1 52 to -1 27)

Netherlands,Deaths,80 ~ 85,-0 89(-1 01 to -0 77)

Netherlands,Deaths,85 ~ 90,-0 15(-0 3 to 0)

Netherlands,Deaths,90 ~ 95,0 39(0 15 to 0 63)

Netherlands,Deaths,20 ~ 25,-1 36(-5 34 to 2 79)

Netherlands,Deaths,25 ~ 30,-1 55(-4 4 to 1 38)

Netherlands,Deaths,30 ~ 35,-1 65(-3 81 to 0 57)

Netherlands,Deaths,35 ~ 40,-1 64(-3 31 to 0 05)

Netherlands,Deaths,40 ~ 45,-1 28(-2 46 to -0 08)

Netherlands,Deaths,45 ~ 50,-0 85(-1 62 to -0 09)

Netherlands,Deaths,50 ~ 55,-0 54(-1 03 to -0 05)

Netherlands,Deaths,55 ~ 60,-0 62(-0 96 to -0 28)

Netherlands,Deaths,60 ~ 65,-0 94(-1 18 to -0 7)

Netherlands,Deaths,65 ~ 70,-1 35(-1 54 to -1 17)

Netherlands,Deaths,70 ~ 75,-1 59(-1 74 to -1 45)

Netherlands,Deaths,75 ~ 80,-1 39(-1 52 to -1 27)

Netherlands,Deaths,80 ~ 85,-0 89(-1 01 to -0 77)

Netherlands,Deaths,85 ~ 90,-0 15(-0 3 to 0)

Netherlands,Deaths,90 ~ 95,0 39(0 15 to 0 63)

Netherlands,DALYs,20 ~ 25,-0 89(-2 31 to 0 54)

Netherlands,DALYs,25 ~ 30,-1 04(-2 03 to -0 03)

Netherlands,DALYs,30 ~ 35,-1 11(-1 9 to -0 32)

Netherlands,DALYs,35 ~ 40,-1 1(-1 74 to -0 46)

Netherlands,DALYs,40 ~ 45,-0 87(-1 37 to -0 37)

Netherlands,DALYs,45 ~ 50,-0 62(-0 99 to -0 25)

Netherlands,DALYs,50 ~ 55,-0 4(-0 68 to -0 13)

Netherlands,DALYs,55 ~ 60,-0 45(-0 66 to -0 23)

Netherlands,DALYs,60 ~ 65,-0 69(-0 86 to -0 52)

Netherlands,DALYs,65 ~ 70,-1 02(-1 17 to -0 88)

Netherlands,DALYs,70 ~ 75,-1 25(-1 38 to -1 12)

Netherlands,DALYs,75 ~ 80,-1 13(-1 25 to -1)

Netherlands,DALYs,80 ~ 85,-0 73(-0 87 to -0 59)

Netherlands,DALYs,85 ~ 90,-0 12(-0 3 to 0 07)

Netherlands,DALYs,90 ~ 95,0 36(0 04 to 0 69)

Netherlands,DALYs,20 ~ 25,-0 89(-2 31 to 0 54)

Netherlands,DALYs,25 ~ 30,-1 04(-2 03 to -0 03)

Netherlands,DALYs,30 ~ 35,-1 11(-1 9 to -0 32)

Netherlands,DALYs,35 ~ 40,-1 1(-1 74 to -0 46)

Netherlands,DALYs,40 ~ 45,-0 87(-1 37 to -0 37)

Netherlands,DALYs,45 ~ 50,-0 62(-0 99 to -0 25)

Netherlands,DALYs,50 ~ 55,-0 4(-0 68 to -0 13)

Netherlands,DALYs,55 ~ 60,-0 45(-0 66 to -0 23)

Netherlands,DALYs,60 ~ 65,-0 69(-0 86 to -0 52)

Netherlands,DALYs,65 ~ 70,-1 02(-1 17 to -0 88)

Netherlands,DALYs,70 ~ 75,-1 25(-1 38 to -1 12)

Netherlands,DALYs,75 ~ 80,-1 13(-1 25 to -1)

Netherlands,DALYs,80 ~ 85,-0 73(-0 87 to -0 59)

Netherlands,DALYs,85 ~ 90,-0 12(-0 3 to 0 07)

Netherlands,DALYs,90 ~ 95,0 36(0 04 to 0 69)

Netherlands,DALYs,20 ~ 25,-0 89(-2 31 to 0 54)

Netherlands,DALYs,25 ~ 30,-1 04(-2 03 to -0 03)

Netherlands,DALYs,30 ~ 35,-1 11(-1 9 to -0 32)

Netherlands,DALYs,35 ~ 40,-1 1(-1 74 to -0 46)

Netherlands,DALYs,40 ~ 45,-0 87(-1 37 to -0 37)

Netherlands,DALYs,45 ~ 50,-0 62(-0 99 to -0 25)

Netherlands,DALYs,50 ~ 55,-0 4(-0 68 to -0 13)

Netherlands,DALYs,55 ~ 60,-0 45(-0 66 to -0 23)

Netherlands,DALYs,60 ~ 65,-0 69(-0 86 to -0 52)

Netherlands,DALYs,65 ~ 70,-1 02(-1 17 to -0 88)

Netherlands,DALYs,70 ~ 75,-1 25(-1 38 to -1 12)

Netherlands,DALYs,75 ~ 80,-1 13(-1 25 to -1)

Netherlands,DALYs,80 ~ 85,-0 73(-0 87 to -0 59)

Netherlands,DALYs,85 ~ 90,-0 12(-0 3 to 0 07)

Netherlands,DALYs,90 ~ 95,0 36(0 04 to 0 69)

Netherlands,DALYs,20 ~ 25,-0 89(-2 31 to 0 54)

Netherlands,DALYs,25 ~ 30,-1 04(-2 03 to -0 03)

Netherlands,DALYs,30 ~ 35,-1 11(-1 9 to -0 32)

Netherlands,DALYs,35 ~ 40,-1 1(-1 74 to -0 46)

Netherlands,DALYs,40 ~ 45,-0 87(-1 37 to -0 37)

Netherlands,DALYs,45 ~ 50,-0 62(-0 99 to -0 25)

Netherlands,DALYs,50 ~ 55,-0 4(-0 68 to -0 13)

Netherlands,DALYs,55 ~ 60,-0 45(-0 66 to -0 23)

Netherlands,DALYs,60 ~ 65,-0 69(-0 86 to -0 52)

Netherlands,DALYs,65 ~ 70,-1 02(-1 17 to -0 88)

Netherlands,DALYs,70 ~ 75,-1 25(-1 38 to -1 12)

Netherlands,DALYs,75 ~ 80,-1 13(-1 25 to -1)

Netherlands,DALYs,80 ~ 85,-0 73(-0 87 to -0 59)

Netherlands,DALYs,85 ~ 90,-0 12(-0 3 to 0 07)

Netherlands,DALYs,90 ~ 95,0 36(0 04 to 0 69)

Palestine,Prevalence,20 ~ 25,-0 35(-0 49 to -0 21)

Palestine,Prevalence,25 ~ 30,-0 33(-0 44 to -0 22)

Palestine,Prevalence,30 ~ 35,-0 3(-0 4 to -0 2)

Palestine,Prevalence,35 ~ 40,-0 27(-0 36 to -0 17)

Palestine,Prevalence,40 ~ 45,-0 23(-0 32 to -0 15)

Palestine,Prevalence,45 ~ 50,-0 18(-0 26 to -0 1)

Palestine,Prevalence,50 ~ 55,-0 1(-0 17 to -0 02)

Palestine,Prevalence,55 ~ 60,0 01(-0 06 to 0 09)

Palestine,Prevalence,60 ~ 65,0 15(0 08 to 0 22)

Palestine,Prevalence,65 ~ 70,0 31(0 24 to 0 38)

Palestine,Prevalence,70 ~ 75,0 48(0 4 to 0 56)

Palestine,Prevalence,75 ~ 80,0 63(0 54 to 0 73)

Palestine,Prevalence,80 ~ 85,0 79(0 67 to 0 91)

Palestine,Prevalence,85 ~ 90,0 96(0 78 to 1 14)

Palestine,Prevalence,90 ~ 95,1 1(0 78 to 1 42)

Palestine,Prevalence,20 ~ 25,-0 35(-0 49 to -0 21)

Palestine,Prevalence,25 ~ 30,-0 33(-0 44 to -0 22)

Palestine,Prevalence,30 ~ 35,-0 3(-0 4 to -0 2)

Palestine,Prevalence,35 ~ 40,-0 27(-0 36 to -0 17)

Palestine,Prevalence,40 ~ 45,-0 23(-0 32 to -0 15)

Palestine,Prevalence,45 ~ 50,-0 18(-0 26 to -0 1)

Palestine,Prevalence,50 ~ 55,-0 1(-0 17 to -0 02)

Palestine,Prevalence,55 ~ 60,0 01(-0 06 to 0 09)

Palestine,Prevalence,60 ~ 65,0 15(0 08 to 0 22)

Palestine,Prevalence,65 ~ 70,0 31(0 24 to 0 38)

Palestine,Prevalence,70 ~ 75,0 48(0 4 to 0 56)

Palestine,Prevalence,75 ~ 80,0 63(0 54 to 0 73)

Palestine,Prevalence,80 ~ 85,0 79(0 67 to 0 91)

Palestine,Prevalence,85 ~ 90,0 96(0 78 to 1 14)

Palestine,Prevalence,90 ~ 95,1 1(0 78 to 1 42)

Palestine,Prevalence,20 ~ 25,-0 35(-0 49 to -0 21)

Palestine,Prevalence,25 ~ 30,-0 33(-0 44 to -0 22)

Palestine,Prevalence,30 ~ 35,-0 3(-0 4 to -0 2)

Palestine,Prevalence,35 ~ 40,-0 27(-0 36 to -0 17)

Palestine,Prevalence,40 ~ 45,-0 23(-0 32 to -0 15)

Palestine,Prevalence,45 ~ 50,-0 18(-0 26 to -0 1)

Palestine,Prevalence,50 ~ 55,-0 1(-0 17 to -0 02)

Palestine,Prevalence,55 ~ 60,0 01(-0 06 to 0 09)

Palestine,Prevalence,60 ~ 65,0 15(0 08 to 0 22)

Palestine,Prevalence,65 ~ 70,0 31(0 24 to 0 38)

Palestine,Prevalence,70 ~ 75,0 48(0 4 to 0 56)

Palestine,Prevalence,75 ~ 80,0 63(0 54 to 0 73)

Palestine,Prevalence,80 ~ 85,0 79(0 67 to 0 91)

Palestine,Prevalence,85 ~ 90,0 96(0 78 to 1 14)

Palestine,Prevalence,90 ~ 95,1 1(0 78 to 1 42)

Palestine,Prevalence,20 ~ 25,-0 35(-0 49 to -0 21)

Palestine,Prevalence,25 ~ 30,-0 33(-0 44 to -0 22)

Palestine,Prevalence,30 ~ 35,-0 3(-0 4 to -0 2)

Palestine,Prevalence,35 ~ 40,-0 27(-0 36 to -0 17)

Palestine,Prevalence,40 ~ 45,-0 23(-0 32 to -0 15)

Palestine,Prevalence,45 ~ 50,-0 18(-0 26 to -0 1)

Palestine,Prevalence,50 ~ 55,-0 1(-0 17 to -0 02)

Palestine,Prevalence,55 ~ 60,0 01(-0 06 to 0 09)

Palestine,Prevalence,60 ~ 65,0 15(0 08 to 0 22)

Palestine,Prevalence,65 ~ 70,0 31(0 24 to 0 38)

Palestine,Prevalence,70 ~ 75,0 48(0 4 to 0 56)

Palestine,Prevalence,75 ~ 80,0 63(0 54 to 0 73)

Palestine,Prevalence,80 ~ 85,0 79(0 67 to 0 91)

Palestine,Prevalence,85 ~ 90,0 96(0 78 to 1 14)

Palestine,Prevalence,90 ~ 95,1 1(0 78 to 1 42)

Palestine,Deaths,20 ~ 25,-1 58(-6 71 to 3 82)

Palestine,Deaths,25 ~ 30,-1 78(-5 61 to 2 21)

Palestine,Deaths,30 ~ 35,-2 1(-5 33 to 1 25)

Palestine,Deaths,35 ~ 40,-2 42(-5 24 to 0 49)

Palestine,Deaths,40 ~ 45,-2 44(-4 78 to -0 03)

Palestine,Deaths,45 ~ 50,-2 29(-4 17 to -0 37)

Palestine,Deaths,50 ~ 55,-2 11(-3 57 to -0 62)

Palestine,Deaths,55 ~ 60,-1 99(-3 15 to -0 83)

Palestine,Deaths,60 ~ 65,-1 95(-2 89 to -1)

Palestine,Deaths,65 ~ 70,-2(-2 81 to -1 19)

Palestine,Deaths,70 ~ 75,-2 11(-2 82 to -1 38)

Palestine,Deaths,75 ~ 80,-2 15(-2 84 to -1 45)

Palestine,Deaths,80 ~ 85,-2 03(-2 79 to -1 27)

Palestine,Deaths,85 ~ 90,-1 88(-2 84 to -0 91)

Palestine,Deaths,90 ~ 95,-1 69(-3 25 to -0 1)

Palestine,Deaths,20 ~ 25,-1 58(-6 71 to 3 82)

Palestine,Deaths,25 ~ 30,-1 78(-5 61 to 2 21)

Palestine,Deaths,30 ~ 35,-2 1(-5 33 to 1 25)

Palestine,Deaths,35 ~ 40,-2 42(-5 24 to 0 49)

Palestine,Deaths,40 ~ 45,-2 44(-4 78 to -0 03)

Palestine,Deaths,45 ~ 50,-2 29(-4 17 to -0 37)

Palestine,Deaths,50 ~ 55,-2 11(-3 57 to -0 62)

Palestine,Deaths,55 ~ 60,-1 99(-3 15 to -0 83)

Palestine,Deaths,60 ~ 65,-1 95(-2 89 to -1)

Palestine,Deaths,65 ~ 70,-2(-2 81 to -1 19)

Palestine,Deaths,70 ~ 75,-2 11(-2 82 to -1 38)

Palestine,Deaths,75 ~ 80,-2 15(-2 84 to -1 45)

Palestine,Deaths,80 ~ 85,-2 03(-2 79 to -1 27)

Palestine,Deaths,85 ~ 90,-1 88(-2 84 to -0 91)

Palestine,Deaths,90 ~ 95,-1 69(-3 25 to -0 1)

Palestine,Deaths,20 ~ 25,-1 58(-6 71 to 3 82)

Palestine,Deaths,25 ~ 30,-1 78(-5 61 to 2 21)

Palestine,Deaths,30 ~ 35,-2 1(-5 33 to 1 25)

Palestine,Deaths,35 ~ 40,-2 42(-5 24 to 0 49)

Palestine,Deaths,40 ~ 45,-2 44(-4 78 to -0 03)

Palestine,Deaths,45 ~ 50,-2 29(-4 17 to -0 37)

Palestine,Deaths,50 ~ 55,-2 11(-3 57 to -0 62)

Palestine,Deaths,55 ~ 60,-1 99(-3 15 to -0 83)

Palestine,Deaths,60 ~ 65,-1 95(-2 89 to -1)

Palestine,Deaths,65 ~ 70,-2(-2 81 to -1 19)

Palestine,Deaths,70 ~ 75,-2 11(-2 82 to -1 38)

Palestine,Deaths,75 ~ 80,-2 15(-2 84 to -1 45)

Palestine,Deaths,80 ~ 85,-2 03(-2 79 to -1 27)

Palestine,Deaths,85 ~ 90,-1 88(-2 84 to -0 91)

Palestine,Deaths,90 ~ 95,-1 69(-3 25 to -0 1)

Palestine,Deaths,20 ~ 25,-1 58(-6 71 to 3 82)

Palestine,Deaths,25 ~ 30,-1 78(-5 61 to 2 21)

Palestine,Deaths,30 ~ 35,-2 1(-5 33 to 1 25)

Palestine,Deaths,35 ~ 40,-2 42(-5 24 to 0 49)

Palestine,Deaths,40 ~ 45,-2 44(-4 78 to -0 03)

Palestine,Deaths,45 ~ 50,-2 29(-4 17 to -0 37)

Palestine,Deaths,50 ~ 55,-2 11(-3 57 to -0 62)

Palestine,Deaths,55 ~ 60,-1 99(-3 15 to -0 83)

Palestine,Deaths,60 ~ 65,-1 95(-2 89 to -1)

Palestine,Deaths,65 ~ 70,-2(-2 81 to -1 19)

Palestine,Deaths,70 ~ 75,-2 11(-2 82 to -1 38)

Palestine,Deaths,75 ~ 80,-2 15(-2 84 to -1 45)

Palestine,Deaths,80 ~ 85,-2 03(-2 79 to -1 27)

Palestine,Deaths,85 ~ 90,-1 88(-2 84 to -0 91)

Palestine,Deaths,90 ~ 95,-1 69(-3 25 to -0 1)

Palestine,DALYs,20 ~ 25,-0 74(-1 16 to -0 32)

Palestine,DALYs,25 ~ 30,-0 84(-1 16 to -0 51)

Palestine,DALYs,30 ~ 35,-1 02(-1 31 to -0 73)

Palestine,DALYs,35 ~ 40,-1 24(-1 5 to -0 97)

Palestine,DALYs,40 ~ 45,-1 34(-1 58 to -1 1)

Palestine,DALYs,45 ~ 50,-1 35(-1 57 to -1 14)

Palestine,DALYs,50 ~ 55,-1 35(-1 53 to -1 16)

Palestine,DALYs,55 ~ 60,-1 37(-1 54 to -1 21)

Palestine,DALYs,60 ~ 65,-1 41(-1 55 to -1 26)

Palestine,DALYs,65 ~ 70,-1 47(-1 61 to -1 33)

Palestine,DALYs,70 ~ 75,-1 56(-1 71 to -1 42)

Palestine,DALYs,75 ~ 80,-1 59(-1 75 to -1 43)

Palestine,DALYs,80 ~ 85,-1 5(-1 7 to -1 31)

Palestine,DALYs,85 ~ 90,-1 35(-1 62 to -1 07)

Palestine,DALYs,90 ~ 95,-1 17(-1 65 to -0 69)

Palestine,DALYs,20 ~ 25,-0 74(-1 16 to -0 32)

Palestine,DALYs,25 ~ 30,-0 84(-1 16 to -0 51)

Palestine,DALYs,30 ~ 35,-1 02(-1 31 to -0 73)

Palestine,DALYs,35 ~ 40,-1 24(-1 5 to -0 97)

Palestine,DALYs,40 ~ 45,-1 34(-1 58 to -1 1)

Palestine,DALYs,45 ~ 50,-1 35(-1 57 to -1 14)

Palestine,DALYs,50 ~ 55,-1 35(-1 53 to -1 16)

Palestine,DALYs,55 ~ 60,-1 37(-1 54 to -1 21)

Palestine,DALYs,60 ~ 65,-1 41(-1 55 to -1 26)

Palestine,DALYs,65 ~ 70,-1 47(-1 61 to -1 33)

Palestine,DALYs,70 ~ 75,-1 56(-1 71 to -1 42)

Palestine,DALYs,75 ~ 80,-1 59(-1 75 to -1 43)

Palestine,DALYs,80 ~ 85,-1 5(-1 7 to -1 31)

Palestine,DALYs,85 ~ 90,-1 35(-1 62 to -1 07)

Palestine,DALYs,90 ~ 95,-1 17(-1 65 to -0 69)

Palestine,DALYs,20 ~ 25,-0 74(-1 16 to -0 32)

Palestine,DALYs,25 ~ 30,-0 84(-1 16 to -0 51)

Palestine,DALYs,30 ~ 35,-1 02(-1 31 to -0 73)

Palestine,DALYs,35 ~ 40,-1 24(-1 5 to -0 97)

Palestine,DALYs,40 ~ 45,-1 34(-1 58 to -1 1)

Palestine,DALYs,45 ~ 50,-1 35(-1 57 to -1 14)

Palestine,DALYs,50 ~ 55,-1 35(-1 53 to -1 16)

Palestine,DALYs,55 ~ 60,-1 37(-1 54 to -1 21)

Palestine,DALYs,60 ~ 65,-1 41(-1 55 to -1 26)

Palestine,DALYs,65 ~ 70,-1 47(-1 61 to -1 33)

Palestine,DALYs,70 ~ 75,-1 56(-1 71 to -1 42)

Palestine,DALYs,75 ~ 80,-1 59(-1 75 to -1 43)

Palestine,DALYs,80 ~ 85,-1 5(-1 7 to -1 31)

Palestine,DALYs,85 ~ 90,-1 35(-1 62 to -1 07)

Palestine,DALYs,90 ~ 95,-1 17(-1 65 to -0 69)

Palestine,DALYs,20 ~ 25,-0 74(-1 16 to -0 32)

Palestine,DALYs,25 ~ 30,-0 84(-1 16 to -0 51)

Palestine,DALYs,30 ~ 35,-1 02(-1 31 to -0 73)

Palestine,DALYs,35 ~ 40,-1 24(-1 5 to -0 97)

Palestine,DALYs,40 ~ 45,-1 34(-1 58 to -1 1)

Palestine,DALYs,45 ~ 50,-1 35(-1 57 to -1 14)

Palestine,DALYs,50 ~ 55,-1 35(-1 53 to -1 16)

Palestine,DALYs,55 ~ 60,-1 37(-1 54 to -1 21)

Palestine,DALYs,60 ~ 65,-1 41(-1 55 to -1 26)

Palestine,DALYs,65 ~ 70,-1 47(-1 61 to -1 33)

Palestine,DALYs,70 ~ 75,-1 56(-1 71 to -1 42)

Palestine,DALYs,75 ~ 80,-1 59(-1 75 to -1 43)

Palestine,DALYs,80 ~ 85,-1 5(-1 7 to -1 31)

Palestine,DALYs,85 ~ 90,-1 35(-1 62 to -1 07)

Palestine,DALYs,90 ~ 95,-1 17(-1 65 to -0 69)

Cambodia,Prevalence,20 ~ 25,-0 38(-0 46 to -0 31)

Cambodia,Prevalence,25 ~ 30,-0 36(-0 42 to -0 31)

Cambodia,Prevalence,30 ~ 35,-0 33(-0 37 to -0 28)

Cambodia,Prevalence,35 ~ 40,-0 29(-0 33 to -0 25)

Cambodia,Prevalence,40 ~ 45,-0 26(-0 3 to -0 22)

Cambodia,Prevalence,45 ~ 50,-0 24(-0 28 to -0 21)

Cambodia,Prevalence,50 ~ 55,-0 22(-0 25 to -0 18)

Cambodia,Prevalence,55 ~ 60,-0 16(-0 2 to -0 13)

Cambodia,Prevalence,60 ~ 65,-0 09(-0 12 to -0 06)

Cambodia,Prevalence,65 ~ 70,0 02(-0 01 to 0 05)

Cambodia,Prevalence,70 ~ 75,0 17(0 13 to 0 2)

Cambodia,Prevalence,75 ~ 80,0 33(0 29 to 0 37)

Cambodia,Prevalence,80 ~ 85,0 49(0 43 to 0 54)

Cambodia,Prevalence,85 ~ 90,0 65(0 56 to 0 74)

Cambodia,Prevalence,90 ~ 95,0 78(0 6 to 0 96)

Cambodia,Prevalence,20 ~ 25,-0 38(-0 46 to -0 31)

Cambodia,Prevalence,25 ~ 30,-0 36(-0 42 to -0 31)

Cambodia,Prevalence,30 ~ 35,-0 33(-0 37 to -0 28)

Cambodia,Prevalence,35 ~ 40,-0 29(-0 33 to -0 25)

Cambodia,Prevalence,40 ~ 45,-0 26(-0 3 to -0 22)

Cambodia,Prevalence,45 ~ 50,-0 24(-0 28 to -0 21)

Cambodia,Prevalence,50 ~ 55,-0 22(-0 25 to -0 18)

Cambodia,Prevalence,55 ~ 60,-0 16(-0 2 to -0 13)

Cambodia,Prevalence,60 ~ 65,-0 09(-0 12 to -0 06)

Cambodia,Prevalence,65 ~ 70,0 02(-0 01 to 0 05)

Cambodia,Prevalence,70 ~ 75,0 17(0 13 to 0 2)

Cambodia,Prevalence,75 ~ 80,0 33(0 29 to 0 37)

Cambodia,Prevalence,80 ~ 85,0 49(0 43 to 0 54)

Cambodia,Prevalence,85 ~ 90,0 65(0 56 to 0 74)

Cambodia,Prevalence,90 ~ 95,0 78(0 6 to 0 96)

Cambodia,Prevalence,20 ~ 25,-0 38(-0 46 to -0 31)

Cambodia,Prevalence,25 ~ 30,-0 36(-0 42 to -0 31)

Cambodia,Prevalence,30 ~ 35,-0 33(-0 37 to -0 28)

Cambodia,Prevalence,35 ~ 40,-0 29(-0 33 to -0 25)

Cambodia,Prevalence,40 ~ 45,-0 26(-0 3 to -0 22)

Cambodia,Prevalence,45 ~ 50,-0 24(-0 28 to -0 21)

Cambodia,Prevalence,50 ~ 55,-0 22(-0 25 to -0 18)

Cambodia,Prevalence,55 ~ 60,-0 16(-0 2 to -0 13)

Cambodia,Prevalence,60 ~ 65,-0 09(-0 12 to -0 06)

Cambodia,Prevalence,65 ~ 70,0 02(-0 01 to 0 05)

Cambodia,Prevalence,70 ~ 75,0 17(0 13 to 0 2)

Cambodia,Prevalence,75 ~ 80,0 33(0 29 to 0 37)

Cambodia,Prevalence,80 ~ 85,0 49(0 43 to 0 54)

Cambodia,Prevalence,85 ~ 90,0 65(0 56 to 0 74)

Cambodia,Prevalence,90 ~ 95,0 78(0 6 to 0 96)

Cambodia,Prevalence,20 ~ 25,-0 38(-0 46 to -0 31)

Cambodia,Prevalence,25 ~ 30,-0 36(-0 42 to -0 31)

Cambodia,Prevalence,30 ~ 35,-0 33(-0 37 to -0 28)

Cambodia,Prevalence,35 ~ 40,-0 29(-0 33 to -0 25)

Cambodia,Prevalence,40 ~ 45,-0 26(-0 3 to -0 22)

Cambodia,Prevalence,45 ~ 50,-0 24(-0 28 to -0 21)

Cambodia,Prevalence,50 ~ 55,-0 22(-0 25 to -0 18)

Cambodia,Prevalence,55 ~ 60,-0 16(-0 2 to -0 13)

Cambodia,Prevalence,60 ~ 65,-0 09(-0 12 to -0 06)

Cambodia,Prevalence,65 ~ 70,0 02(-0 01 to 0 05)

Cambodia,Prevalence,70 ~ 75,0 17(0 13 to 0 2)

Cambodia,Prevalence,75 ~ 80,0 33(0 29 to 0 37)

Cambodia,Prevalence,80 ~ 85,0 49(0 43 to 0 54)

Cambodia,Prevalence,85 ~ 90,0 65(0 56 to 0 74)

Cambodia,Prevalence,90 ~ 95,0 78(0 6 to 0 96)

Cambodia,Deaths,20 ~ 25,-1 75(-3 6 to 0 14)

Cambodia,Deaths,25 ~ 30,-1 71(-3 02 to -0 39)

Cambodia,Deaths,30 ~ 35,-1 55(-2 6 to -0 49)

Cambodia,Deaths,35 ~ 40,-1 4(-2 26 to -0 53)

Cambodia,Deaths,40 ~ 45,-1 34(-2 05 to -0 63)

Cambodia,Deaths,45 ~ 50,-1 36(-1 91 to -0 8)

Cambodia,Deaths,50 ~ 55,-1 41(-1 82 to -0 99)

Cambodia,Deaths,55 ~ 60,-1 41(-1 73 to -1 09)

Cambodia,Deaths,60 ~ 65,-1 35(-1 61 to -1 1)

Cambodia,Deaths,65 ~ 70,-1 15(-1 37 to -0 93)

Cambodia,Deaths,70 ~ 75,-0 85(-1 05 to -0 64)

Cambodia,Deaths,75 ~ 80,-0 5(-0 72 to -0 28)

Cambodia,Deaths,80 ~ 85,-0 16(-0 42 to 0 1)

Cambodia,Deaths,85 ~ 90,0 17(-0 21 to 0 55)

Cambodia,Deaths,90 ~ 95,0 42(-0 3 to 1 15)

Cambodia,Deaths,20 ~ 25,-1 75(-3 6 to 0 14)

Cambodia,Deaths,25 ~ 30,-1 71(-3 02 to -0 39)

Cambodia,Deaths,30 ~ 35,-1 55(-2 6 to -0 49)

Cambodia,Deaths,35 ~ 40,-1 4(-2 26 to -0 53)

Cambodia,Deaths,40 ~ 45,-1 34(-2 05 to -0 63)

Cambodia,Deaths,45 ~ 50,-1 36(-1 91 to -0 8)

Cambodia,Deaths,50 ~ 55,-1 41(-1 82 to -0 99)

Cambodia,Deaths,55 ~ 60,-1 41(-1 73 to -1 09)

Cambodia,Deaths,60 ~ 65,-1 35(-1 61 to -1 1)

Cambodia,Deaths,65 ~ 70,-1 15(-1 37 to -0 93)

Cambodia,Deaths,70 ~ 75,-0 85(-1 05 to -0 64)

Cambodia,Deaths,75 ~ 80,-0 5(-0 72 to -0 28)

Cambodia,Deaths,80 ~ 85,-0 16(-0 42 to 0 1)

Cambodia,Deaths,85 ~ 90,0 17(-0 21 to 0 55)

Cambodia,Deaths,90 ~ 95,0 42(-0 3 to 1 15)

Cambodia,Deaths,20 ~ 25,-1 75(-3 6 to 0 14)

Cambodia,Deaths,25 ~ 30,-1 71(-3 02 to -0 39)

Cambodia,Deaths,30 ~ 35,-1 55(-2 6 to -0 49)

Cambodia,Deaths,35 ~ 40,-1 4(-2 26 to -0 53)

Cambodia,Deaths,40 ~ 45,-1 34(-2 05 to -0 63)

Cambodia,Deaths,45 ~ 50,-1 36(-1 91 to -0 8)

Cambodia,Deaths,50 ~ 55,-1 41(-1 82 to -0 99)

Cambodia,Deaths,55 ~ 60,-1 41(-1 73 to -1 09)

Cambodia,Deaths,60 ~ 65,-1 35(-1 61 to -1 1)

Cambodia,Deaths,65 ~ 70,-1 15(-1 37 to -0 93)

Cambodia,Deaths,70 ~ 75,-0 85(-1 05 to -0 64)

Cambodia,Deaths,75 ~ 80,-0 5(-0 72 to -0 28)

Cambodia,Deaths,80 ~ 85,-0 16(-0 42 to 0 1)

Cambodia,Deaths,85 ~ 90,0 17(-0 21 to 0 55)

Cambodia,Deaths,90 ~ 95,0 42(-0 3 to 1 15)

Cambodia,Deaths,20 ~ 25,-1 75(-3 6 to 0 14)

Cambodia,Deaths,25 ~ 30,-1 71(-3 02 to -0 39)

Cambodia,Deaths,30 ~ 35,-1 55(-2 6 to -0 49)

Cambodia,Deaths,35 ~ 40,-1 4(-2 26 to -0 53)

Cambodia,Deaths,40 ~ 45,-1 34(-2 05 to -0 63)

Cambodia,Deaths,45 ~ 50,-1 36(-1 91 to -0 8)

Cambodia,Deaths,50 ~ 55,-1 41(-1 82 to -0 99)

Cambodia,Deaths,55 ~ 60,-1 41(-1 73 to -1 09)

Cambodia,Deaths,60 ~ 65,-1 35(-1 61 to -1 1)

Cambodia,Deaths,65 ~ 70,-1 15(-1 37 to -0 93)

Cambodia,Deaths,70 ~ 75,-0 85(-1 05 to -0 64)

Cambodia,Deaths,75 ~ 80,-0 5(-0 72 to -0 28)

Cambodia,Deaths,80 ~ 85,-0 16(-0 42 to 0 1)

Cambodia,Deaths,85 ~ 90,0 17(-0 21 to 0 55)

Cambodia,Deaths,90 ~ 95,0 42(-0 3 to 1 15)

Cambodia,DALYs,20 ~ 25,-1 11(-1 31 to -0 9)

Cambodia,DALYs,25 ~ 30,-1 07(-1 22 to -0 92)

Cambodia,DALYs,30 ~ 35,-0 98(-1 11 to -0 86)

Cambodia,DALYs,35 ~ 40,-0 92(-1 03 to -0 81)

Cambodia,DALYs,40 ~ 45,-0 94(-1 04 to -0 84)

Cambodia,DALYs,45 ~ 50,-1 01(-1 1 to -0 93)

Cambodia,DALYs,50 ~ 55,-1 1(-1 17 to -1 03)

Cambodia,DALYs,55 ~ 60,-1 14(-1 19 to -1 08)

Cambodia,DALYs,60 ~ 65,-1 11(-1 17 to -1 06)

Cambodia,DALYs,65 ~ 70,-0 96(-1 01 to -0 91)

Cambodia,DALYs,70 ~ 75,-0 72(-0 78 to -0 67)

Cambodia,DALYs,75 ~ 80,-0 43(-0 5 to -0 37)

Cambodia,DALYs,80 ~ 85,-0 14(-0 22 to -0 05)

Cambodia,DALYs,85 ~ 90,0 16(0 03 to 0 3)

Cambodia,DALYs,90 ~ 95,0 4(0 13 to 0 68)

Cambodia,DALYs,20 ~ 25,-1 11(-1 31 to -0 9)

Cambodia,DALYs,25 ~ 30,-1 07(-1 22 to -0 92)

Cambodia,DALYs,30 ~ 35,-0 98(-1 11 to -0 86)

Cambodia,DALYs,35 ~ 40,-0 92(-1 03 to -0 81)

Cambodia,DALYs,40 ~ 45,-0 94(-1 04 to -0 84)

Cambodia,DALYs,45 ~ 50,-1 01(-1 1 to -0 93)

Cambodia,DALYs,50 ~ 55,-1 1(-1 17 to -1 03)

Cambodia,DALYs,55 ~ 60,-1 14(-1 19 to -1 08)

Cambodia,DALYs,60 ~ 65,-1 11(-1 17 to -1 06)

Cambodia,DALYs,65 ~ 70,-0 96(-1 01 to -0 91)

Cambodia,DALYs,70 ~ 75,-0 72(-0 78 to -0 67)

Cambodia,DALYs,75 ~ 80,-0 43(-0 5 to -0 37)

Cambodia,DALYs,80 ~ 85,-0 14(-0 22 to -0 05)

Cambodia,DALYs,85 ~ 90,0 16(0 03 to 0 3)

Cambodia,DALYs,90 ~ 95,0 4(0 13 to 0 68)

Cambodia,DALYs,20 ~ 25,-1 11(-1 31 to -0 9)

Cambodia,DALYs,25 ~ 30,-1 07(-1 22 to -0 92)

Cambodia,DALYs,30 ~ 35,-0 98(-1 11 to -0 86)

Cambodia,DALYs,35 ~ 40,-0 92(-1 03 to -0 81)

Cambodia,DALYs,40 ~ 45,-0 94(-1 04 to -0 84)

Cambodia,DALYs,45 ~ 50,-1 01(-1 1 to -0 93)

Cambodia,DALYs,50 ~ 55,-1 1(-1 17 to -1 03)

Cambodia,DALYs,55 ~ 60,-1 14(-1 19 to -1 08)

Cambodia,DALYs,60 ~ 65,-1 11(-1 17 to -1 06)

Cambodia,DALYs,65 ~ 70,-0 96(-1 01 to -0 91)

Cambodia,DALYs,70 ~ 75,-0 72(-0 78 to -0 67)

Cambodia,DALYs,75 ~ 80,-0 43(-0 5 to -0 37)

Cambodia,DALYs,80 ~ 85,-0 14(-0 22 to -0 05)

Cambodia,DALYs,85 ~ 90,0 16(0 03 to 0 3)

Cambodia,DALYs,90 ~ 95,0 4(0 13 to 0 68)

Cambodia,DALYs,20 ~ 25,-1 11(-1 31 to -0 9)

Cambodia,DALYs,25 ~ 30,-1 07(-1 22 to -0 92)

Cambodia,DALYs,30 ~ 35,-0 98(-1 11 to -0 86)

Cambodia,DALYs,35 ~ 40,-0 92(-1 03 to -0 81)

Cambodia,DALYs,40 ~ 45,-0 94(-1 04 to -0 84)

Cambodia,DALYs,45 ~ 50,-1 01(-1 1 to -0 93)

Cambodia,DALYs,50 ~ 55,-1 1(-1 17 to -1 03)

Cambodia,DALYs,55 ~ 60,-1 14(-1 19 to -1 08)

Cambodia,DALYs,60 ~ 65,-1 11(-1 17 to -1 06)

Cambodia,DALYs,65 ~ 70,-0 96(-1 01 to -0 91)

Cambodia,DALYs,70 ~ 75,-0 72(-0 78 to -0 67)

Cambodia,DALYs,75 ~ 80,-0 43(-0 5 to -0 37)

Cambodia,DALYs,80 ~ 85,-0 14(-0 22 to -0 05)

Cambodia,DALYs,85 ~ 90,0 16(0 03 to 0 3)

Cambodia,DALYs,90 ~ 95,0 4(0 13 to 0 68)

France,Prevalence,20 ~ 25,-0 19(-0 45 to 0 07)

France,Prevalence,25 ~ 30,-0 18(-0 37 to 0 01)

France,Prevalence,30 ~ 35,-0 17(-0 31 to -0 02)

France,Prevalence,35 ~ 40,-0 16(-0 28 to -0 03)

France,Prevalence,40 ~ 45,-0 14(-0 24 to -0 04)

France,Prevalence,45 ~ 50,-0 14(-0 22 to -0 05)

France,Prevalence,50 ~ 55,-0 14(-0 21 to -0 07)

France,Prevalence,55 ~ 60,-0 14(-0 2 to -0 08)

France,Prevalence,60 ~ 65,-0 14(-0 19 to -0 08)

France,Prevalence,65 ~ 70,-0 14(-0 19 to -0 09)

France,Prevalence,70 ~ 75,-0 13(-0 18 to -0 08)

France,Prevalence,75 ~ 80,-0 09(-0 14 to -0 04)

France,Prevalence,80 ~ 85,-0 02(-0 07 to 0 03)

France,Prevalence,85 ~ 90,0 11(0 05 to 0 17)

France,Prevalence,90 ~ 95,0 28(0 17 to 0 39)

France,Prevalence,20 ~ 25,-0 19(-0 45 to 0 07)

France,Prevalence,25 ~ 30,-0 18(-0 37 to 0 01)

France,Prevalence,30 ~ 35,-0 17(-0 31 to -0 02)

France,Prevalence,35 ~ 40,-0 16(-0 28 to -0 03)

France,Prevalence,40 ~ 45,-0 14(-0 24 to -0 04)

France,Prevalence,45 ~ 50,-0 14(-0 22 to -0 05)

France,Prevalence,50 ~ 55,-0 14(-0 21 to -0 07)

France,Prevalence,55 ~ 60,-0 14(-0 2 to -0 08)

France,Prevalence,60 ~ 65,-0 14(-0 19 to -0 08)

France,Prevalence,65 ~ 70,-0 14(-0 19 to -0 09)

France,Prevalence,70 ~ 75,-0 13(-0 18 to -0 08)

France,Prevalence,75 ~ 80,-0 09(-0 14 to -0 04)

France,Prevalence,80 ~ 85,-0 02(-0 07 to 0 03)

France,Prevalence,85 ~ 90,0 11(0 05 to 0 17)

France,Prevalence,90 ~ 95,0 28(0 17 to 0 39)

France,Prevalence,20 ~ 25,-0 19(-0 45 to 0 07)

France,Prevalence,25 ~ 30,-0 18(-0 37 to 0 01)

France,Prevalence,30 ~ 35,-0 17(-0 31 to -0 02)

France,Prevalence,35 ~ 40,-0 16(-0 28 to -0 03)

France,Prevalence,40 ~ 45,-0 14(-0 24 to -0 04)

France,Prevalence,45 ~ 50,-0 14(-0 22 to -0 05)

France,Prevalence,50 ~ 55,-0 14(-0 21 to -0 07)

France,Prevalence,55 ~ 60,-0 14(-0 2 to -0 08)

France,Prevalence,60 ~ 65,-0 14(-0 19 to -0 08)

France,Prevalence,65 ~ 70,-0 14(-0 19 to -0 09)

France,Prevalence,70 ~ 75,-0 13(-0 18 to -0 08)

France,Prevalence,75 ~ 80,-0 09(-0 14 to -0 04)

France,Prevalence,80 ~ 85,-0 02(-0 07 to 0 03)

France,Prevalence,85 ~ 90,0 11(0 05 to 0 17)

France,Prevalence,90 ~ 95,0 28(0 17 to 0 39)

France,Prevalence,20 ~ 25,-0 19(-0 45 to 0 07)

France,Prevalence,25 ~ 30,-0 18(-0 37 to 0 01)

France,Prevalence,30 ~ 35,-0 17(-0 31 to -0 02)

France,Prevalence,35 ~ 40,-0 16(-0 28 to -0 03)

France,Prevalence,40 ~ 45,-0 14(-0 24 to -0 04)

France,Prevalence,45 ~ 50,-0 14(-0 22 to -0 05)

France,Prevalence,50 ~ 55,-0 14(-0 21 to -0 07)

France,Prevalence,55 ~ 60,-0 14(-0 2 to -0 08)

France,Prevalence,60 ~ 65,-0 14(-0 19 to -0 08)

France,Prevalence,65 ~ 70,-0 14(-0 19 to -0 09)

France,Prevalence,70 ~ 75,-0 13(-0 18 to -0 08)

France,Prevalence,75 ~ 80,-0 09(-0 14 to -0 04)

France,Prevalence,80 ~ 85,-0 02(-0 07 to 0 03)

France,Prevalence,85 ~ 90,0 11(0 05 to 0 17)

France,Prevalence,90 ~ 95,0 28(0 17 to 0 39)

France,Deaths,20 ~ 25,-2 03(-6 18 to 2 3)

France,Deaths,25 ~ 30,-2 14(-5 08 to 0 89)

France,Deaths,30 ~ 35,-2 19(-4 35 to 0 01)

France,Deaths,35 ~ 40,-2 17(-3 7 to -0 62)

France,Deaths,40 ~ 45,-2 02(-3 07 to -0 97)

France,Deaths,45 ~ 50,-1 75(-2 44 to -1 06)

France,Deaths,50 ~ 55,-1 46(-1 92 to -1)

France,Deaths,55 ~ 60,-1 44(-1 76 to -1 12)

France,Deaths,60 ~ 65,-1 69(-1 91 to -1 46)

France,Deaths,65 ~ 70,-2 05(-2 23 to -1 88)

France,Deaths,70 ~ 75,-2 47(-2 61 to -2 33)

France,Deaths,75 ~ 80,-2 75(-2 87 to -2 62)

France,Deaths,80 ~ 85,-2 78(-2 88 to -2 67)

France,Deaths,85 ~ 90,-2 86(-2 97 to -2 76)

France,Deaths,90 ~ 95,-2 65(-2 8 to -2 49)

France,Deaths,20 ~ 25,-2 03(-6 18 to 2 3)

France,Deaths,25 ~ 30,-2 14(-5 08 to 0 89)

France,Deaths,30 ~ 35,-2 19(-4 35 to 0 01)

France,Deaths,35 ~ 40,-2 17(-3 7 to -0 62)

France,Deaths,40 ~ 45,-2 02(-3 07 to -0 97)

France,Deaths,45 ~ 50,-1 75(-2 44 to -1 06)

France,Deaths,50 ~ 55,-1 46(-1 92 to -1)

France,Deaths,55 ~ 60,-1 44(-1 76 to -1 12)

France,Deaths,60 ~ 65,-1 69(-1 91 to -1 46)

France,Deaths,65 ~ 70,-2 05(-2 23 to -1 88)

France,Deaths,70 ~ 75,-2 47(-2 61 to -2 33)

France,Deaths,75 ~ 80,-2 75(-2 87 to -2 62)

France,Deaths,80 ~ 85,-2 78(-2 88 to -2 67)

France,Deaths,85 ~ 90,-2 86(-2 97 to -2 76)

France,Deaths,90 ~ 95,-2 65(-2 8 to -2 49)

France,Deaths,20 ~ 25,-2 03(-6 18 to 2 3)

France,Deaths,25 ~ 30,-2 14(-5 08 to 0 89)

France,Deaths,30 ~ 35,-2 19(-4 35 to 0 01)

France,Deaths,35 ~ 40,-2 17(-3 7 to -0 62)

France,Deaths,40 ~ 45,-2 02(-3 07 to -0 97)

France,Deaths,45 ~ 50,-1 75(-2 44 to -1 06)

France,Deaths,50 ~ 55,-1 46(-1 92 to -1)

France,Deaths,55 ~ 60,-1 44(-1 76 to -1 12)

France,Deaths,60 ~ 65,-1 69(-1 91 to -1 46)

France,Deaths,65 ~ 70,-2 05(-2 23 to -1 88)

France,Deaths,70 ~ 75,-2 47(-2 61 to -2 33)

France,Deaths,75 ~ 80,-2 75(-2 87 to -2 62)

France,Deaths,80 ~ 85,-2 78(-2 88 to -2 67)

France,Deaths,85 ~ 90,-2 86(-2 97 to -2 76)

France,Deaths,90 ~ 95,-2 65(-2 8 to -2 49)

France,Deaths,20 ~ 25,-2 03(-6 18 to 2 3)

France,Deaths,25 ~ 30,-2 14(-5 08 to 0 89)

France,Deaths,30 ~ 35,-2 19(-4 35 to 0 01)

France,Deaths,35 ~ 40,-2 17(-3 7 to -0 62)

France,Deaths,40 ~ 45,-2 02(-3 07 to -0 97)

France,Deaths,45 ~ 50,-1 75(-2 44 to -1 06)

France,Deaths,50 ~ 55,-1 46(-1 92 to -1)

France,Deaths,55 ~ 60,-1 44(-1 76 to -1 12)

France,Deaths,60 ~ 65,-1 69(-1 91 to -1 46)

France,Deaths,65 ~ 70,-2 05(-2 23 to -1 88)

France,Deaths,70 ~ 75,-2 47(-2 61 to -2 33)

France,Deaths,75 ~ 80,-2 75(-2 87 to -2 62)

France,Deaths,80 ~ 85,-2 78(-2 88 to -2 67)

France,Deaths,85 ~ 90,-2 86(-2 97 to -2 76)

France,Deaths,90 ~ 95,-2 65(-2 8 to -2 49)

France,DALYs,20 ~ 25,-0 8(-2 16 to 0 58)

France,DALYs,25 ~ 30,-0 78(-1 75 to 0 2)

France,DALYs,30 ~ 35,-0 81(-1 55 to -0 05)

France,DALYs,35 ~ 40,-0 87(-1 46 to -0 27)

France,DALYs,40 ~ 45,-0 89(-1 36 to -0 41)

France,DALYs,45 ~ 50,-0 85(-1 21 to -0 48)

France,DALYs,50 ~ 55,-0 77(-1 05 to -0 49)

France,DALYs,55 ~ 60,-0 85(-1 07 to -0 62)

France,DALYs,60 ~ 65,-1 11(-1 29 to -0 93)

France,DALYs,65 ~ 70,-1 43(-1 58 to -1 28)

France,DALYs,70 ~ 75,-1 84(-1 98 to -1 7)

France,DALYs,75 ~ 80,-2 1(-2 24 to -1 97)

France,DALYs,80 ~ 85,-2 21(-2 34 to -2 08)

France,DALYs,85 ~ 90,-2 37(-2 51 to -2 22)

France,DALYs,90 ~ 95,-2 23(-2 46 to -1 99)

France,DALYs,20 ~ 25,-0 8(-2 16 to 0 58)

France,DALYs,25 ~ 30,-0 78(-1 75 to 0 2)

France,DALYs,30 ~ 35,-0 81(-1 55 to -0 05)

France,DALYs,35 ~ 40,-0 87(-1 46 to -0 27)

France,DALYs,40 ~ 45,-0 89(-1 36 to -0 41)

France,DALYs,45 ~ 50,-0 85(-1 21 to -0 48)

France,DALYs,50 ~ 55,-0 77(-1 05 to -0 49)

France,DALYs,55 ~ 60,-0 85(-1 07 to -0 62)

France,DALYs,60 ~ 65,-1 11(-1 29 to -0 93)

France,DALYs,65 ~ 70,-1 43(-1 58 to -1 28)

France,DALYs,70 ~ 75,-1 84(-1 98 to -1 7)

France,DALYs,75 ~ 80,-2 1(-2 24 to -1 97)

France,DALYs,80 ~ 85,-2 21(-2 34 to -2 08)

France,DALYs,85 ~ 90,-2 37(-2 51 to -2 22)

France,DALYs,90 ~ 95,-2 23(-2 46 to -1 99)

France,DALYs,20 ~ 25,-0 8(-2 16 to 0 58)

France,DALYs,25 ~ 30,-0 78(-1 75 to 0 2)

France,DALYs,30 ~ 35,-0 81(-1 55 to -0 05)

France,DALYs,35 ~ 40,-0 87(-1 46 to -0 27)

France,DALYs,40 ~ 45,-0 89(-1 36 to -0 41)

France,DALYs,45 ~ 50,-0 85(-1 21 to -0 48)

France,DALYs,50 ~ 55,-0 77(-1 05 to -0 49)

France,DALYs,55 ~ 60,-0 85(-1 07 to -0 62)

France,DALYs,60 ~ 65,-1 11(-1 29 to -0 93)

France,DALYs,65 ~ 70,-1 43(-1 58 to -1 28)

France,DALYs,70 ~ 75,-1 84(-1 98 to -1 7)

France,DALYs,75 ~ 80,-2 1(-2 24 to -1 97)

France,DALYs,80 ~ 85,-2 21(-2 34 to -2 08)

France,DALYs,85 ~ 90,-2 37(-2 51 to -2 22)

France,DALYs,90 ~ 95,-2 23(-2 46 to -1 99)

France,DALYs,20 ~ 25,-0 8(-2 16 to 0 58)

France,DALYs,25 ~ 30,-0 78(-1 75 to 0 2)

France,DALYs,30 ~ 35,-0 81(-1 55 to -0 05)

France,DALYs,35 ~ 40,-0 87(-1 46 to -0 27)

France,DALYs,40 ~ 45,-0 89(-1 36 to -0 41)

France,DALYs,45 ~ 50,-0 85(-1 21 to -0 48)

France,DALYs,50 ~ 55,-0 77(-1 05 to -0 49)

France,DALYs,55 ~ 60,-0 85(-1 07 to -0 62)

France,DALYs,60 ~ 65,-1 11(-1 29 to -0 93)

France,DALYs,65 ~ 70,-1 43(-1 58 to -1 28)

France,DALYs,70 ~ 75,-1 84(-1 98 to -1 7)

France,DALYs,75 ~ 80,-2 1(-2 24 to -1 97)

France,DALYs,80 ~ 85,-2 21(-2 34 to -2 08)

France,DALYs,85 ~ 90,-2 37(-2 51 to -2 22)

France,DALYs,90 ~ 95,-2 23(-2 46 to -1 99)

Myanmar,Prevalence,20 ~ 25,-0 33(-0 42 to -0 24)

Myanmar,Prevalence,25 ~ 30,-0 3(-0 36 to -0 23)

Myanmar,Prevalence,30 ~ 35,-0 29(-0 35 to -0 24)

Myanmar,Prevalence,35 ~ 40,-0 32(-0 36 to -0 27)

Myanmar,Prevalence,40 ~ 45,-0 36(-0 4 to -0 31)

Myanmar,Prevalence,45 ~ 50,-0 39(-0 43 to -0 35)

Myanmar,Prevalence,50 ~ 55,-0 38(-0 41 to -0 34)

Myanmar,Prevalence,55 ~ 60,-0 32(-0 36 to -0 29)

Myanmar,Prevalence,60 ~ 65,-0 23(-0 27 to -0 2)

Myanmar,Prevalence,65 ~ 70,-0 14(-0 17 to -0 1)

Myanmar,Prevalence,70 ~ 75,-0 04(-0 08 to -0 01)

Myanmar,Prevalence,75 ~ 80,0 03(-0 01 to 0 07)

Myanmar,Prevalence,80 ~ 85,0 11(0 05 to 0 16)

Myanmar,Prevalence,85 ~ 90,0 19(0 11 to 0 27)

Myanmar,Prevalence,90 ~ 95,0 28(0 11 to 0 45)

Myanmar,Prevalence,20 ~ 25,-0 33(-0 42 to -0 24)

Myanmar,Prevalence,25 ~ 30,-0 3(-0 36 to -0 23)

Myanmar,Prevalence,30 ~ 35,-0 29(-0 35 to -0 24)

Myanmar,Prevalence,35 ~ 40,-0 32(-0 36 to -0 27)

Myanmar,Prevalence,40 ~ 45,-0 36(-0 4 to -0 31)

Myanmar,Prevalence,45 ~ 50,-0 39(-0 43 to -0 35)

Myanmar,Prevalence,50 ~ 55,-0 38(-0 41 to -0 34)

Myanmar,Prevalence,55 ~ 60,-0 32(-0 36 to -0 29)

Myanmar,Prevalence,60 ~ 65,-0 23(-0 27 to -0 2)

Myanmar,Prevalence,65 ~ 70,-0 14(-0 17 to -0 1)

Myanmar,Prevalence,70 ~ 75,-0 04(-0 08 to -0 01)

Myanmar,Prevalence,75 ~ 80,0 03(-0 01 to 0 07)

Myanmar,Prevalence,80 ~ 85,0 11(0 05 to 0 16)

Myanmar,Prevalence,85 ~ 90,0 19(0 11 to 0 27)

Myanmar,Prevalence,90 ~ 95,0 28(0 11 to 0 45)

Myanmar,Prevalence,20 ~ 25,-0 33(-0 42 to -0 24)

Myanmar,Prevalence,25 ~ 30,-0 3(-0 36 to -0 23)

Myanmar,Prevalence,30 ~ 35,-0 29(-0 35 to -0 24)

Myanmar,Prevalence,35 ~ 40,-0 32(-0 36 to -0 27)

Myanmar,Prevalence,40 ~ 45,-0 36(-0 4 to -0 31)

Myanmar,Prevalence,45 ~ 50,-0 39(-0 43 to -0 35)

Myanmar,Prevalence,50 ~ 55,-0 38(-0 41 to -0 34)

Myanmar,Prevalence,55 ~ 60,-0 32(-0 36 to -0 29)

Myanmar,Prevalence,60 ~ 65,-0 23(-0 27 to -0 2)

Myanmar,Prevalence,65 ~ 70,-0 14(-0 17 to -0 1)

Myanmar,Prevalence,70 ~ 75,-0 04(-0 08 to -0 01)

Myanmar,Prevalence,75 ~ 80,0 03(-0 01 to 0 07)

Myanmar,Prevalence,80 ~ 85,0 11(0 05 to 0 16)

Myanmar,Prevalence,85 ~ 90,0 19(0 11 to 0 27)

Myanmar,Prevalence,90 ~ 95,0 28(0 11 to 0 45)

Myanmar,Prevalence,20 ~ 25,-0 33(-0 42 to -0 24)

Myanmar,Prevalence,25 ~ 30,-0 3(-0 36 to -0 23)

Myanmar,Prevalence,30 ~ 35,-0 29(-0 35 to -0 24)

Myanmar,Prevalence,35 ~ 40,-0 32(-0 36 to -0 27)

Myanmar,Prevalence,40 ~ 45,-0 36(-0 4 to -0 31)

Myanmar,Prevalence,45 ~ 50,-0 39(-0 43 to -0 35)

Myanmar,Prevalence,50 ~ 55,-0 38(-0 41 to -0 34)

Myanmar,Prevalence,55 ~ 60,-0 32(-0 36 to -0 29)

Myanmar,Prevalence,60 ~ 65,-0 23(-0 27 to -0 2)

Myanmar,Prevalence,65 ~ 70,-0 14(-0 17 to -0 1)

Myanmar,Prevalence,70 ~ 75,-0 04(-0 08 to -0 01)

Myanmar,Prevalence,75 ~ 80,0 03(-0 01 to 0 07)

Myanmar,Prevalence,80 ~ 85,0 11(0 05 to 0 16)

Myanmar,Prevalence,85 ~ 90,0 19(0 11 to 0 27)

Myanmar,Prevalence,90 ~ 95,0 28(0 11 to 0 45)

Myanmar,Deaths,20 ~ 25,-2 5(-3 16 to -1 84)

Myanmar,Deaths,25 ~ 30,-2 5(-2 97 to -2 03)

Myanmar,Deaths,30 ~ 35,-2 44(-2 82 to -2 07)

Myanmar,Deaths,35 ~ 40,-2 39(-2 68 to -2 09)

Myanmar,Deaths,40 ~ 45,-2 37(-2 61 to -2 14)

Myanmar,Deaths,45 ~ 50,-2 38(-2 56 to -2 19)

Myanmar,Deaths,50 ~ 55,-2 33(-2 46 to -2 2)

Myanmar,Deaths,55 ~ 60,-2 23(-2 33 to -2 13)

Myanmar,Deaths,60 ~ 65,-2 04(-2 12 to -1 96)

Myanmar,Deaths,65 ~ 70,-1 75(-1 81 to -1 68)

Myanmar,Deaths,70 ~ 75,-1 38(-1 44 to -1 32)

Myanmar,Deaths,75 ~ 80,-1 02(-1 08 to -0 96)

Myanmar,Deaths,80 ~ 85,-0 68(-0 76 to -0 61)

Myanmar,Deaths,85 ~ 90,-0 4(-0 5 to -0 29)

Myanmar,Deaths,90 ~ 95,-0 16(-0 36 to 0 04)

Myanmar,Deaths,20 ~ 25,-2 5(-3 16 to -1 84)

Myanmar,Deaths,25 ~ 30,-2 5(-2 97 to -2 03)

Myanmar,Deaths,30 ~ 35,-2 44(-2 82 to -2 07)

Myanmar,Deaths,35 ~ 40,-2 39(-2 68 to -2 09)

Myanmar,Deaths,40 ~ 45,-2 37(-2 61 to -2 14)

Myanmar,Deaths,45 ~ 50,-2 38(-2 56 to -2 19)

Myanmar,Deaths,50 ~ 55,-2 33(-2 46 to -2 2)

Myanmar,Deaths,55 ~ 60,-2 23(-2 33 to -2 13)

Myanmar,Deaths,60 ~ 65,-2 04(-2 12 to -1 96)

Myanmar,Deaths,65 ~ 70,-1 75(-1 81 to -1 68)

Myanmar,Deaths,70 ~ 75,-1 38(-1 44 to -1 32)

Myanmar,Deaths,75 ~ 80,-1 02(-1 08 to -0 96)

Myanmar,Deaths,80 ~ 85,-0 68(-0 76 to -0 61)

Myanmar,Deaths,85 ~ 90,-0 4(-0 5 to -0 29)

Myanmar,Deaths,90 ~ 95,-0 16(-0 36 to 0 04)

Myanmar,Deaths,20 ~ 25,-2 5(-3 16 to -1 84)

Myanmar,Deaths,25 ~ 30,-2 5(-2 97 to -2 03)

Myanmar,Deaths,30 ~ 35,-2 44(-2 82 to -2 07)

Myanmar,Deaths,35 ~ 40,-2 39(-2 68 to -2 09)

Myanmar,Deaths,40 ~ 45,-2 37(-2 61 to -2 14)

Myanmar,Deaths,45 ~ 50,-2 38(-2 56 to -2 19)

Myanmar,Deaths,50 ~ 55,-2 33(-2 46 to -2 2)

Myanmar,Deaths,55 ~ 60,-2 23(-2 33 to -2 13)

Myanmar,Deaths,60 ~ 65,-2 04(-2 12 to -1 96)

Myanmar,Deaths,65 ~ 70,-1 75(-1 81 to -1 68)

Myanmar,Deaths,70 ~ 75,-1 38(-1 44 to -1 32)

Myanmar,Deaths,75 ~ 80,-1 02(-1 08 to -0 96)

Myanmar,Deaths,80 ~ 85,-0 68(-0 76 to -0 61)

Myanmar,Deaths,85 ~ 90,-0 4(-0 5 to -0 29)

Myanmar,Deaths,90 ~ 95,-0 16(-0 36 to 0 04)

Myanmar,Deaths,20 ~ 25,-2 5(-3 16 to -1 84)

Myanmar,Deaths,25 ~ 30,-2 5(-2 97 to -2 03)

Myanmar,Deaths,30 ~ 35,-2 44(-2 82 to -2 07)

Myanmar,Deaths,35 ~ 40,-2 39(-2 68 to -2 09)

Myanmar,Deaths,40 ~ 45,-2 37(-2 61 to -2 14)

Myanmar,Deaths,45 ~ 50,-2 38(-2 56 to -2 19)

Myanmar,Deaths,50 ~ 55,-2 33(-2 46 to -2 2)

Myanmar,Deaths,55 ~ 60,-2 23(-2 33 to -2 13)

Myanmar,Deaths,60 ~ 65,-2 04(-2 12 to -1 96)

Myanmar,Deaths,65 ~ 70,-1 75(-1 81 to -1 68)

Myanmar,Deaths,70 ~ 75,-1 38(-1 44 to -1 32)

Myanmar,Deaths,75 ~ 80,-1 02(-1 08 to -0 96)

Myanmar,Deaths,80 ~ 85,-0 68(-0 76 to -0 61)

Myanmar,Deaths,85 ~ 90,-0 4(-0 5 to -0 29)

Myanmar,Deaths,90 ~ 95,-0 16(-0 36 to 0 04)

Myanmar,DALYs,20 ~ 25,-1 89(-2 18 to -1 61)

Myanmar,DALYs,25 ~ 30,-1 88(-2 09 to -1 67)

Myanmar,DALYs,30 ~ 35,-1 85(-2 02 to -1 68)

Myanmar,DALYs,35 ~ 40,-1 86(-2 to -1 71)

Myanmar,DALYs,40 ~ 45,-1 92(-2 04 to -1 79)

Myanmar,DALYs,45 ~ 50,-1 99(-2 09 to -1 88)

Myanmar,DALYs,50 ~ 55,-2 01(-2 1 to -1 93)

Myanmar,DALYs,55 ~ 60,-1 96(-2 03 to -1 9)

Myanmar,DALYs,60 ~ 65,-1 83(-1 89 to -1 78)

Myanmar,DALYs,65 ~ 70,-1 6(-1 66 to -1 55)

Myanmar,DALYs,70 ~ 75,-1 3(-1 36 to -1 25)

Myanmar,DALYs,75 ~ 80,-1(-1 06 to -0 94)

Myanmar,DALYs,80 ~ 85,-0 7(-0 78 to -0 61)

Myanmar,DALYs,85 ~ 90,-0 43(-0 56 to -0 29)

Myanmar,DALYs,90 ~ 95,-0 19(-0 46 to 0 08)

Myanmar,DALYs,20 ~ 25,-1 89(-2 18 to -1 61)

Myanmar,DALYs,25 ~ 30,-1 88(-2 09 to -1 67)

Myanmar,DALYs,30 ~ 35,-1 85(-2 02 to -1 68)

Myanmar,DALYs,35 ~ 40,-1 86(-2 to -1 71)

Myanmar,DALYs,40 ~ 45,-1 92(-2 04 to -1 79)

Myanmar,DALYs,45 ~ 50,-1 99(-2 09 to -1 88)

Myanmar,DALYs,50 ~ 55,-2 01(-2 1 to -1 93)

Myanmar,DALYs,55 ~ 60,-1 96(-2 03 to -1 9)

Myanmar,DALYs,60 ~ 65,-1 83(-1 89 to -1 78)

Myanmar,DALYs,65 ~ 70,-1 6(-1 66 to -1 55)

Myanmar,DALYs,70 ~ 75,-1 3(-1 36 to -1 25)

Myanmar,DALYs,75 ~ 80,-1(-1 06 to -0 94)

Myanmar,DALYs,80 ~ 85,-0 7(-0 78 to -0 61)

Myanmar,DALYs,85 ~ 90,-0 43(-0 56 to -0 29)

Myanmar,DALYs,90 ~ 95,-0 19(-0 46 to 0 08)

Myanmar,DALYs,20 ~ 25,-1 89(-2 18 to -1 61)

Myanmar,DALYs,25 ~ 30,-1 88(-2 09 to -1 67)

Myanmar,DALYs,30 ~ 35,-1 85(-2 02 to -1 68)

Myanmar,DALYs,35 ~ 40,-1 86(-2 to -1 71)

Myanmar,DALYs,40 ~ 45,-1 92(-2 04 to -1 79)

Myanmar,DALYs,45 ~ 50,-1 99(-2 09 to -1 88)

Myanmar,DALYs,50 ~ 55,-2 01(-2 1 to -1 93)

Myanmar,DALYs,55 ~ 60,-1 96(-2 03 to -1 9)

Myanmar,DALYs,60 ~ 65,-1 83(-1 89 to -1 78)

Myanmar,DALYs,65 ~ 70,-1 6(-1 66 to -1 55)

Myanmar,DALYs,70 ~ 75,-1 3(-1 36 to -1 25)

Myanmar,DALYs,75 ~ 80,-1(-1 06 to -0 94)

Myanmar,DALYs,80 ~ 85,-0 7(-0 78 to -0 61)

Myanmar,DALYs,85 ~ 90,-0 43(-0 56 to -0 29)

Myanmar,DALYs,90 ~ 95,-0 19(-0 46 to 0 08)

Myanmar,DALYs,20 ~ 25,-1 89(-2 18 to -1 61)

Myanmar,DALYs,25 ~ 30,-1 88(-2 09 to -1 67)

Myanmar,DALYs,30 ~ 35,-1 85(-2 02 to -1 68)

Myanmar,DALYs,35 ~ 40,-1 86(-2 to -1 71)

Myanmar,DALYs,40 ~ 45,-1 92(-2 04 to -1 79)

Myanmar,DALYs,45 ~ 50,-1 99(-2 09 to -1 88)

Myanmar,DALYs,50 ~ 55,-2 01(-2 1 to -1 93)

Myanmar,DALYs,55 ~ 60,-1 96(-2 03 to -1 9)

Myanmar,DALYs,60 ~ 65,-1 83(-1 89 to -1 78)

Myanmar,DALYs,65 ~ 70,-1 6(-1 66 to -1 55)

Myanmar,DALYs,70 ~ 75,-1 3(-1 36 to -1 25)

Myanmar,DALYs,75 ~ 80,-1(-1 06 to -0 94)

Myanmar,DALYs,80 ~ 85,-0 7(-0 78 to -0 61)

Myanmar,DALYs,85 ~ 90,-0 43(-0 56 to -0 29)

Myanmar,DALYs,90 ~ 95,-0 19(-0 46 to 0 08)

Afghanistan,Prevalence,20 ~ 25,-0 29(-0 37 to -0 2)

Afghanistan,Prevalence,25 ~ 30,-0 27(-0 33 to -0 2)

Afghanistan,Prevalence,30 ~ 35,-0 26(-0 32 to -0 19)

Afghanistan,Prevalence,35 ~ 40,-0 25(-0 31 to -0 19)

Afghanistan,Prevalence,40 ~ 45,-0 24(-0 3 to -0 19)

Afghanistan,Prevalence,45 ~ 50,-0 21(-0 26 to -0 16)

Afghanistan,Prevalence,50 ~ 55,-0 15(-0 19 to -0 1)

Afghanistan,Prevalence,55 ~ 60,-0 06(-0 1 to -0 01)

Afghanistan,Prevalence,60 ~ 65,0 05(0 01 to 0 1)

Afghanistan,Prevalence,65 ~ 70,0 18(0 13 to 0 22)

Afghanistan,Prevalence,70 ~ 75,0 3(0 26 to 0 35)

Afghanistan,Prevalence,75 ~ 80,0 43(0 37 to 0 48)

Afghanistan,Prevalence,80 ~ 85,0 57(0 5 to 0 64)

Afghanistan,Prevalence,85 ~ 90,0 71(0 6 to 0 82)

Afghanistan,Prevalence,90 ~ 95,0 83(0 6 to 1 06)

Afghanistan,Prevalence,20 ~ 25,-0 29(-0 37 to -0 2)

Afghanistan,Prevalence,25 ~ 30,-0 27(-0 33 to -0 2)

Afghanistan,Prevalence,30 ~ 35,-0 26(-0 32 to -0 19)

Afghanistan,Prevalence,35 ~ 40,-0 25(-0 31 to -0 19)

Afghanistan,Prevalence,40 ~ 45,-0 24(-0 3 to -0 19)

Afghanistan,Prevalence,45 ~ 50,-0 21(-0 26 to -0 16)

Afghanistan,Prevalence,50 ~ 55,-0 15(-0 19 to -0 1)

Afghanistan,Prevalence,55 ~ 60,-0 06(-0 1 to -0 01)

Afghanistan,Prevalence,60 ~ 65,0 05(0 01 to 0 1)

Afghanistan,Prevalence,65 ~ 70,0 18(0 13 to 0 22)

Afghanistan,Prevalence,70 ~ 75,0 3(0 26 to 0 35)

Afghanistan,Prevalence,75 ~ 80,0 43(0 37 to 0 48)

Afghanistan,Prevalence,80 ~ 85,0 57(0 5 to 0 64)

Afghanistan,Prevalence,85 ~ 90,0 71(0 6 to 0 82)

Afghanistan,Prevalence,90 ~ 95,0 83(0 6 to 1 06)

Afghanistan,Prevalence,20 ~ 25,-0 29(-0 37 to -0 2)

Afghanistan,Prevalence,25 ~ 30,-0 27(-0 33 to -0 2)

Afghanistan,Prevalence,30 ~ 35,-0 26(-0 32 to -0 19)

Afghanistan,Prevalence,35 ~ 40,-0 25(-0 31 to -0 19)

Afghanistan,Prevalence,40 ~ 45,-0 24(-0 3 to -0 19)

Afghanistan,Prevalence,45 ~ 50,-0 21(-0 26 to -0 16)

Afghanistan,Prevalence,50 ~ 55,-0 15(-0 19 to -0 1)

Afghanistan,Prevalence,55 ~ 60,-0 06(-0 1 to -0 01)

Afghanistan,Prevalence,60 ~ 65,0 05(0 01 to 0 1)

Afghanistan,Prevalence,65 ~ 70,0 18(0 13 to 0 22)

Afghanistan,Prevalence,70 ~ 75,0 3(0 26 to 0 35)

Afghanistan,Prevalence,75 ~ 80,0 43(0 37 to 0 48)

Afghanistan,Prevalence,80 ~ 85,0 57(0 5 to 0 64)

Afghanistan,Prevalence,85 ~ 90,0 71(0 6 to 0 82)

Afghanistan,Prevalence,90 ~ 95,0 83(0 6 to 1 06)

Afghanistan,Prevalence,20 ~ 25,-0 29(-0 37 to -0 2)

Afghanistan,Prevalence,25 ~ 30,-0 27(-0 33 to -0 2)

Afghanistan,Prevalence,30 ~ 35,-0 26(-0 32 to -0 19)

Afghanistan,Prevalence,35 ~ 40,-0 25(-0 31 to -0 19)

Afghanistan,Prevalence,40 ~ 45,-0 24(-0 3 to -0 19)

Afghanistan,Prevalence,45 ~ 50,-0 21(-0 26 to -0 16)

Afghanistan,Prevalence,50 ~ 55,-0 15(-0 19 to -0 1)

Afghanistan,Prevalence,55 ~ 60,-0 06(-0 1 to -0 01)

Afghanistan,Prevalence,60 ~ 65,0 05(0 01 to 0 1)

Afghanistan,Prevalence,65 ~ 70,0 18(0 13 to 0 22)

Afghanistan,Prevalence,70 ~ 75,0 3(0 26 to 0 35)

Afghanistan,Prevalence,75 ~ 80,0 43(0 37 to 0 48)

Afghanistan,Prevalence,80 ~ 85,0 57(0 5 to 0 64)

Afghanistan,Prevalence,85 ~ 90,0 71(0 6 to 0 82)

Afghanistan,Prevalence,90 ~ 95,0 83(0 6 to 1 06)

Afghanistan,Deaths,20 ~ 25,-1 22(-2 19 to -0 23)

Afghanistan,Deaths,25 ~ 30,-1 38(-2 13 to -0 62)

Afghanistan,Deaths,30 ~ 35,-1 6(-2 25 to -0 94)

Afghanistan,Deaths,35 ~ 40,-1 8(-2 36 to -1 25)

Afghanistan,Deaths,40 ~ 45,-1 86(-2 3 to -1 41)

Afghanistan,Deaths,45 ~ 50,-1 91(-2 24 to -1 57)

Afghanistan,Deaths,50 ~ 55,-1 79(-2 07 to -1 51)

Afghanistan,Deaths,55 ~ 60,-1 4(-1 65 to -1 14)

Afghanistan,Deaths,60 ~ 65,-1 01(-1 25 to -0 78)

Afghanistan,Deaths,65 ~ 70,-0 85(-1 04 to -0 66)

Afghanistan,Deaths,70 ~ 75,-0 82(-1 to -0 65)

Afghanistan,Deaths,75 ~ 80,-0 75(-0 93 to -0 56)

Afghanistan,Deaths,80 ~ 85,-0 67(-0 89 to -0 44)

Afghanistan,Deaths,85 ~ 90,-0 58(-0 91 to -0 26)

Afghanistan,Deaths,90 ~ 95,-0 51(-1 14 to 0 13)

Afghanistan,Deaths,20 ~ 25,-1 22(-2 19 to -0 23)

Afghanistan,Deaths,25 ~ 30,-1 38(-2 13 to -0 62)

Afghanistan,Deaths,30 ~ 35,-1 6(-2 25 to -0 94)

Afghanistan,Deaths,35 ~ 40,-1 8(-2 36 to -1 25)

Afghanistan,Deaths,40 ~ 45,-1 86(-2 3 to -1 41)

Afghanistan,Deaths,45 ~ 50,-1 91(-2 24 to -1 57)

Afghanistan,Deaths,50 ~ 55,-1 79(-2 07 to -1 51)

Afghanistan,Deaths,55 ~ 60,-1 4(-1 65 to -1 14)

Afghanistan,Deaths,60 ~ 65,-1 01(-1 25 to -0 78)

Afghanistan,Deaths,65 ~ 70,-0 85(-1 04 to -0 66)

Afghanistan,Deaths,70 ~ 75,-0 82(-1 to -0 65)

Afghanistan,Deaths,75 ~ 80,-0 75(-0 93 to -0 56)

Afghanistan,Deaths,80 ~ 85,-0 67(-0 89 to -0 44)

Afghanistan,Deaths,85 ~ 90,-0 58(-0 91 to -0 26)

Afghanistan,Deaths,90 ~ 95,-0 51(-1 14 to 0 13)

Afghanistan,Deaths,20 ~ 25,-1 22(-2 19 to -0 23)

Afghanistan,Deaths,25 ~ 30,-1 38(-2 13 to -0 62)

Afghanistan,Deaths,30 ~ 35,-1 6(-2 25 to -0 94)

Afghanistan,Deaths,35 ~ 40,-1 8(-2 36 to -1 25)

Afghanistan,Deaths,40 ~ 45,-1 86(-2 3 to -1 41)

Afghanistan,Deaths,45 ~ 50,-1 91(-2 24 to -1 57)

Afghanistan,Deaths,50 ~ 55,-1 79(-2 07 to -1 51)

Afghanistan,Deaths,55 ~ 60,-1 4(-1 65 to -1 14)

Afghanistan,Deaths,60 ~ 65,-1 01(-1 25 to -0 78)

Afghanistan,Deaths,65 ~ 70,-0 85(-1 04 to -0 66)

Afghanistan,Deaths,70 ~ 75,-0 82(-1 to -0 65)

Afghanistan,Deaths,75 ~ 80,-0 75(-0 93 to -0 56)

Afghanistan,Deaths,80 ~ 85,-0 67(-0 89 to -0 44)

Afghanistan,Deaths,85 ~ 90,-0 58(-0 91 to -0 26)

Afghanistan,Deaths,90 ~ 95,-0 51(-1 14 to 0 13)

Afghanistan,Deaths,20 ~ 25,-1 22(-2 19 to -0 23)

Afghanistan,Deaths,25 ~ 30,-1 38(-2 13 to -0 62)

Afghanistan,Deaths,30 ~ 35,-1 6(-2 25 to -0 94)

Afghanistan,Deaths,35 ~ 40,-1 8(-2 36 to -1 25)

Afghanistan,Deaths,40 ~ 45,-1 86(-2 3 to -1 41)

Afghanistan,Deaths,45 ~ 50,-1 91(-2 24 to -1 57)

Afghanistan,Deaths,50 ~ 55,-1 79(-2 07 to -1 51)

Afghanistan,Deaths,55 ~ 60,-1 4(-1 65 to -1 14)

Afghanistan,Deaths,60 ~ 65,-1 01(-1 25 to -0 78)

Afghanistan,Deaths,65 ~ 70,-0 85(-1 04 to -0 66)

Afghanistan,Deaths,70 ~ 75,-0 82(-1 to -0 65)

Afghanistan,Deaths,75 ~ 80,-0 75(-0 93 to -0 56)

Afghanistan,Deaths,80 ~ 85,-0 67(-0 89 to -0 44)

Afghanistan,Deaths,85 ~ 90,-0 58(-0 91 to -0 26)

Afghanistan,Deaths,90 ~ 95,-0 51(-1 14 to 0 13)

Afghanistan,DALYs,20 ~ 25,-0 91(-1 06 to -0 76)

Afghanistan,DALYs,25 ~ 30,-1 05(-1 17 to -0 92)

Afghanistan,DALYs,30 ~ 35,-1 26(-1 38 to -1 15)

Afghanistan,DALYs,35 ~ 40,-1 49(-1 59 to -1 39)

Afghanistan,DALYs,40 ~ 45,-1 58(-1 67 to -1 5)

Afghanistan,DALYs,45 ~ 50,-1 63(-1 7 to -1 56)

Afghanistan,DALYs,50 ~ 55,-1 55(-1 61 to -1 48)

Afghanistan,DALYs,55 ~ 60,-1 23(-1 29 to -1 17)

Afghanistan,DALYs,60 ~ 65,-0 91(-0 97 to -0 85)

Afghanistan,DALYs,65 ~ 70,-0 76(-0 81 to -0 71)

Afghanistan,DALYs,70 ~ 75,-0 73(-0 78 to -0 68)

Afghanistan,DALYs,75 ~ 80,-0 66(-0 73 to -0 6)

Afghanistan,DALYs,80 ~ 85,-0 58(-0 67 to -0 49)

Afghanistan,DALYs,85 ~ 90,-0 49(-0 63 to -0 35)

Afghanistan,DALYs,90 ~ 95,-0 41(-0 7 to -0 12)

Afghanistan,DALYs,20 ~ 25,-0 91(-1 06 to -0 76)

Afghanistan,DALYs,25 ~ 30,-1 05(-1 17 to -0 92)

Afghanistan,DALYs,30 ~ 35,-1 26(-1 38 to -1 15)

Afghanistan,DALYs,35 ~ 40,-1 49(-1 59 to -1 39)

Afghanistan,DALYs,40 ~ 45,-1 58(-1 67 to -1 5)

Afghanistan,DALYs,45 ~ 50,-1 63(-1 7 to -1 56)

Afghanistan,DALYs,50 ~ 55,-1 55(-1 61 to -1 48)

Afghanistan,DALYs,55 ~ 60,-1 23(-1 29 to -1 17)

Afghanistan,DALYs,60 ~ 65,-0 91(-0 97 to -0 85)

Afghanistan,DALYs,65 ~ 70,-0 76(-0 81 to -0 71)

Afghanistan,DALYs,70 ~ 75,-0 73(-0 78 to -0 68)

Afghanistan,DALYs,75 ~ 80,-0 66(-0 73 to -0 6)

Afghanistan,DALYs,80 ~ 85,-0 58(-0 67 to -0 49)

Afghanistan,DALYs,85 ~ 90,-0 49(-0 63 to -0 35)

Afghanistan,DALYs,90 ~ 95,-0 41(-0 7 to -0 12)

Afghanistan,DALYs,20 ~ 25,-0 91(-1 06 to -0 76)

Afghanistan,DALYs,25 ~ 30,-1 05(-1 17 to -0 92)

Afghanistan,DALYs,30 ~ 35,-1 26(-1 38 to -1 15)

Afghanistan,DALYs,35 ~ 40,-1 49(-1 59 to -1 39)

Afghanistan,DALYs,40 ~ 45,-1 58(-1 67 to -1 5)

Afghanistan,DALYs,45 ~ 50,-1 63(-1 7 to -1 56)

Afghanistan,DALYs,50 ~ 55,-1 55(-1 61 to -1 48)

Afghanistan,DALYs,55 ~ 60,-1 23(-1 29 to -1 17)

Afghanistan,DALYs,60 ~ 65,-0 91(-0 97 to -0 85)

Afghanistan,DALYs,65 ~ 70,-0 76(-0 81 to -0 71)

Afghanistan,DALYs,70 ~ 75,-0 73(-0 78 to -0 68)

Afghanistan,DALYs,75 ~ 80,-0 66(-0 73 to -0 6)

Afghanistan,DALYs,80 ~ 85,-0 58(-0 67 to -0 49)

Afghanistan,DALYs,85 ~ 90,-0 49(-0 63 to -0 35)

Afghanistan,DALYs,90 ~ 95,-0 41(-0 7 to -0 12)

Afghanistan,DALYs,20 ~ 25,-0 91(-1 06 to -0 76)

Afghanistan,DALYs,25 ~ 30,-1 05(-1 17 to -0 92)

Afghanistan,DALYs,30 ~ 35,-1 26(-1 38 to -1 15)

Afghanistan,DALYs,35 ~ 40,-1 49(-1 59 to -1 39)

Afghanistan,DALYs,40 ~ 45,-1 58(-1 67 to -1 5)

Afghanistan,DALYs,45 ~ 50,-1 63(-1 7 to -1 56)

Afghanistan,DALYs,50 ~ 55,-1 55(-1 61 to -1 48)

Afghanistan,DALYs,55 ~ 60,-1 23(-1 29 to -1 17)

Afghanistan,DALYs,60 ~ 65,-0 91(-0 97 to -0 85)

Afghanistan,DALYs,65 ~ 70,-0 76(-0 81 to -0 71)

Afghanistan,DALYs,70 ~ 75,-0 73(-0 78 to -0 68)

Afghanistan,DALYs,75 ~ 80,-0 66(-0 73 to -0 6)

Afghanistan,DALYs,80 ~ 85,-0 58(-0 67 to -0 49)

Afghanistan,DALYs,85 ~ 90,-0 49(-0 63 to -0 35)

Afghanistan,DALYs,90 ~ 95,-0 41(-0 7 to -0 12)

Bhutan,Prevalence,20 ~ 25,-0 68(-1 01 to -0 35)

Bhutan,Prevalence,25 ~ 30,-0 71(-0 94 to -0 47)

Bhutan,Prevalence,30 ~ 35,-0 71(-0 91 to -0 51)

Bhutan,Prevalence,35 ~ 40,-0 71(-0 89 to -0 53)

Bhutan,Prevalence,40 ~ 45,-0 7(-0 87 to -0 54)

Bhutan,Prevalence,45 ~ 50,-0 68(-0 83 to -0 53)

Bhutan,Prevalence,50 ~ 55,-0 61(-0 75 to -0 48)

Bhutan,Prevalence,55 ~ 60,-0 51(-0 63 to -0 39)

Bhutan,Prevalence,60 ~ 65,-0 37(-0 49 to -0 26)

Bhutan,Prevalence,65 ~ 70,-0 23(-0 34 to -0 12)

Bhutan,Prevalence,70 ~ 75,-0 09(-0 21 to 0 03)

Bhutan,Prevalence,75 ~ 80,0(-0 14 to 0 15)

Bhutan,Prevalence,80 ~ 85,0 07(-0 13 to 0 26)

Bhutan,Prevalence,85 ~ 90,0 09(-0 21 to 0 4)

Bhutan,Prevalence,90 ~ 95,0 11(-0 47 to 0 69)

Bhutan,Prevalence,20 ~ 25,-0 68(-1 01 to -0 35)

Bhutan,Prevalence,25 ~ 30,-0 71(-0 94 to -0 47)

Bhutan,Prevalence,30 ~ 35,-0 71(-0 91 to -0 51)

Bhutan,Prevalence,35 ~ 40,-0 71(-0 89 to -0 53)

Bhutan,Prevalence,40 ~ 45,-0 7(-0 87 to -0 54)

Bhutan,Prevalence,45 ~ 50,-0 68(-0 83 to -0 53)

Bhutan,Prevalence,50 ~ 55,-0 61(-0 75 to -0 48)

Bhutan,Prevalence,55 ~ 60,-0 51(-0 63 to -0 39)

Bhutan,Prevalence,60 ~ 65,-0 37(-0 49 to -0 26)

Bhutan,Prevalence,65 ~ 70,-0 23(-0 34 to -0 12)

Bhutan,Prevalence,70 ~ 75,-0 09(-0 21 to 0 03)

Bhutan,Prevalence,75 ~ 80,0(-0 14 to 0 15)

Bhutan,Prevalence,80 ~ 85,0 07(-0 13 to 0 26)

Bhutan,Prevalence,85 ~ 90,0 09(-0 21 to 0 4)

Bhutan,Prevalence,90 ~ 95,0 11(-0 47 to 0 69)

Bhutan,Prevalence,20 ~ 25,-0 68(-1 01 to -0 35)

Bhutan,Prevalence,25 ~ 30,-0 71(-0 94 to -0 47)

Bhutan,Prevalence,30 ~ 35,-0 71(-0 91 to -0 51)

Bhutan,Prevalence,35 ~ 40,-0 71(-0 89 to -0 53)

Bhutan,Prevalence,40 ~ 45,-0 7(-0 87 to -0 54)

Bhutan,Prevalence,45 ~ 50,-0 68(-0 83 to -0 53)

Bhutan,Prevalence,50 ~ 55,-0 61(-0 75 to -0 48)

Bhutan,Prevalence,55 ~ 60,-0 51(-0 63 to -0 39)

Bhutan,Prevalence,60 ~ 65,-0 37(-0 49 to -0 26)

Bhutan,Prevalence,65 ~ 70,-0 23(-0 34 to -0 12)

Bhutan,Prevalence,70 ~ 75,-0 09(-0 21 to 0 03)

Bhutan,Prevalence,75 ~ 80,0(-0 14 to 0 15)

Bhutan,Prevalence,80 ~ 85,0 07(-0 13 to 0 26)

Bhutan,Prevalence,85 ~ 90,0 09(-0 21 to 0 4)

Bhutan,Prevalence,90 ~ 95,0 11(-0 47 to 0 69)

Bhutan,Prevalence,20 ~ 25,-0 68(-1 01 to -0 35)

Bhutan,Prevalence,25 ~ 30,-0 71(-0 94 to -0 47)

Bhutan,Prevalence,30 ~ 35,-0 71(-0 91 to -0 51)

Bhutan,Prevalence,35 ~ 40,-0 71(-0 89 to -0 53)

Bhutan,Prevalence,40 ~ 45,-0 7(-0 87 to -0 54)

Bhutan,Prevalence,45 ~ 50,-0 68(-0 83 to -0 53)

Bhutan,Prevalence,50 ~ 55,-0 61(-0 75 to -0 48)

Bhutan,Prevalence,55 ~ 60,-0 51(-0 63 to -0 39)

Bhutan,Prevalence,60 ~ 65,-0 37(-0 49 to -0 26)

Bhutan,Prevalence,65 ~ 70,-0 23(-0 34 to -0 12)

Bhutan,Prevalence,70 ~ 75,-0 09(-0 21 to 0 03)

Bhutan,Prevalence,75 ~ 80,0(-0 14 to 0 15)

Bhutan,Prevalence,80 ~ 85,0 07(-0 13 to 0 26)

Bhutan,Prevalence,85 ~ 90,0 09(-0 21 to 0 4)

Bhutan,Prevalence,90 ~ 95,0 11(-0 47 to 0 69)

Bhutan,Deaths,20 ~ 25,-3 33(-13 61 to 8 17)

Bhutan,Deaths,25 ~ 30,-3 29(-10 01 to 3 94)

Bhutan,Deaths,30 ~ 35,-3 2(-8 13 to 2 01)

Bhutan,Deaths,35 ~ 40,-3 11(-6 87 to 0 81)

Bhutan,Deaths,40 ~ 45,-3 01(-5 84 to -0 1)

Bhutan,Deaths,45 ~ 50,-2 92(-5 03 to -0 77)

Bhutan,Deaths,50 ~ 55,-2 81(-4 33 to -1 26)

Bhutan,Deaths,55 ~ 60,-2 66(-3 78 to -1 52)

Bhutan,Deaths,60 ~ 65,-2 38(-3 24 to -1 52)

Bhutan,Deaths,65 ~ 70,-1 99(-2 68 to -1 28)

Bhutan,Deaths,70 ~ 75,-1 53(-2 14 to -0 91)

Bhutan,Deaths,75 ~ 80,-0 98(-1 6 to -0 35)

Bhutan,Deaths,80 ~ 85,-0 39(-1 14 to 0 37)

Bhutan,Deaths,85 ~ 90,0 14(-0 94 to 1 24)

Bhutan,Deaths,90 ~ 95,0 55(-1 5 to 2 64)

Bhutan,Deaths,20 ~ 25,-3 33(-13 61 to 8 17)

Bhutan,Deaths,25 ~ 30,-3 29(-10 01 to 3 94)

Bhutan,Deaths,30 ~ 35,-3 2(-8 13 to 2 01)

Bhutan,Deaths,35 ~ 40,-3 11(-6 87 to 0 81)

Bhutan,Deaths,40 ~ 45,-3 01(-5 84 to -0 1)

Bhutan,Deaths,45 ~ 50,-2 92(-5 03 to -0 77)

Bhutan,Deaths,50 ~ 55,-2 81(-4 33 to -1 26)

Bhutan,Deaths,55 ~ 60,-2 66(-3 78 to -1 52)

Bhutan,Deaths,60 ~ 65,-2 38(-3 24 to -1 52)

Bhutan,Deaths,65 ~ 70,-1 99(-2 68 to -1 28)

Bhutan,Deaths,70 ~ 75,-1 53(-2 14 to -0 91)

Bhutan,Deaths,75 ~ 80,-0 98(-1 6 to -0 35)

Bhutan,Deaths,80 ~ 85,-0 39(-1 14 to 0 37)

Bhutan,Deaths,85 ~ 90,0 14(-0 94 to 1 24)

Bhutan,Deaths,90 ~ 95,0 55(-1 5 to 2 64)

Bhutan,Deaths,20 ~ 25,-3 33(-13 61 to 8 17)

Bhutan,Deaths,25 ~ 30,-3 29(-10 01 to 3 94)

Bhutan,Deaths,30 ~ 35,-3 2(-8 13 to 2 01)

Bhutan,Deaths,35 ~ 40,-3 11(-6 87 to 0 81)

Bhutan,Deaths,40 ~ 45,-3 01(-5 84 to -0 1)

Bhutan,Deaths,45 ~ 50,-2 92(-5 03 to -0 77)

Bhutan,Deaths,50 ~ 55,-2 81(-4 33 to -1 26)

Bhutan,Deaths,55 ~ 60,-2 66(-3 78 to -1 52)

Bhutan,Deaths,60 ~ 65,-2 38(-3 24 to -1 52)

Bhutan,Deaths,65 ~ 70,-1 99(-2 68 to -1 28)

Bhutan,Deaths,70 ~ 75,-1 53(-2 14 to -0 91)

Bhutan,Deaths,75 ~ 80,-0 98(-1 6 to -0 35)

Bhutan,Deaths,80 ~ 85,-0 39(-1 14 to 0 37)

Bhutan,Deaths,85 ~ 90,0 14(-0 94 to 1 24)

Bhutan,Deaths,90 ~ 95,0 55(-1 5 to 2 64)

Bhutan,Deaths,20 ~ 25,-3 33(-13 61 to 8 17)

Bhutan,Deaths,25 ~ 30,-3 29(-10 01 to 3 94)

Bhutan,Deaths,30 ~ 35,-3 2(-8 13 to 2 01)

Bhutan,Deaths,35 ~ 40,-3 11(-6 87 to 0 81)

Bhutan,Deaths,40 ~ 45,-3 01(-5 84 to -0 1)

Bhutan,Deaths,45 ~ 50,-2 92(-5 03 to -0 77)

Bhutan,Deaths,50 ~ 55,-2 81(-4 33 to -1 26)

Bhutan,Deaths,55 ~ 60,-2 66(-3 78 to -1 52)

Bhutan,Deaths,60 ~ 65,-2 38(-3 24 to -1 52)

Bhutan,Deaths,65 ~ 70,-1 99(-2 68 to -1 28)

Bhutan,Deaths,70 ~ 75,-1 53(-2 14 to -0 91)

Bhutan,Deaths,75 ~ 80,-0 98(-1 6 to -0 35)

Bhutan,Deaths,80 ~ 85,-0 39(-1 14 to 0 37)

Bhutan,Deaths,85 ~ 90,0 14(-0 94 to 1 24)

Bhutan,Deaths,90 ~ 95,0 55(-1 5 to 2 64)

Bhutan,DALYs,20 ~ 25,-1 88(-2 67 to -1 09)

Bhutan,DALYs,25 ~ 30,-1 91(-2 46 to -1 36)

Bhutan,DALYs,30 ~ 35,-1 96(-2 4 to -1 52)

Bhutan,DALYs,35 ~ 40,-2 05(-2 42 to -1 68)

Bhutan,DALYs,40 ~ 45,-2 16(-2 47 to -1 85)

Bhutan,DALYs,45 ~ 50,-2 26(-2 51 to -2)

Bhutan,DALYs,50 ~ 55,-2 29(-2 49 to -2 08)

Bhutan,DALYs,55 ~ 60,-2 24(-2 41 to -2 07)

Bhutan,DALYs,60 ~ 65,-2 06(-2 21 to -1 92)

Bhutan,DALYs,65 ~ 70,-1 75(-1 88 to -1 62)

Bhutan,DALYs,70 ~ 75,-1 36(-1 49 to -1 23)

Bhutan,DALYs,75 ~ 80,-0 88(-1 03 to -0 73)

Bhutan,DALYs,80 ~ 85,-0 35(-0 55 to -0 14)

Bhutan,DALYs,85 ~ 90,0 13(-0 19 to 0 46)

Bhutan,DALYs,90 ~ 95,0 49(-0 16 to 1 14)

Bhutan,DALYs,20 ~ 25,-1 88(-2 67 to -1 09)

Bhutan,DALYs,25 ~ 30,-1 91(-2 46 to -1 36)

Bhutan,DALYs,30 ~ 35,-1 96(-2 4 to -1 52)

Bhutan,DALYs,35 ~ 40,-2 05(-2 42 to -1 68)

Bhutan,DALYs,40 ~ 45,-2 16(-2 47 to -1 85)

Bhutan,DALYs,45 ~ 50,-2 26(-2 51 to -2)

Bhutan,DALYs,50 ~ 55,-2 29(-2 49 to -2 08)

Bhutan,DALYs,55 ~ 60,-2 24(-2 41 to -2 07)

Bhutan,DALYs,60 ~ 65,-2 06(-2 21 to -1 92)

Bhutan,DALYs,65 ~ 70,-1 75(-1 88 to -1 62)

Bhutan,DALYs,70 ~ 75,-1 36(-1 49 to -1 23)

Bhutan,DALYs,75 ~ 80,-0 88(-1 03 to -0 73)

Bhutan,DALYs,80 ~ 85,-0 35(-0 55 to -0 14)

Bhutan,DALYs,85 ~ 90,0 13(-0 19 to 0 46)

Bhutan,DALYs,90 ~ 95,0 49(-0 16 to 1 14)

Bhutan,DALYs,20 ~ 25,-1 88(-2 67 to -1 09)

Bhutan,DALYs,25 ~ 30,-1 91(-2 46 to -1 36)

Bhutan,DALYs,30 ~ 35,-1 96(-2 4 to -1 52)

Bhutan,DALYs,35 ~ 40,-2 05(-2 42 to -1 68)

Bhutan,DALYs,40 ~ 45,-2 16(-2 47 to -1 85)

Bhutan,DALYs,45 ~ 50,-2 26(-2 51 to -2)

Bhutan,DALYs,50 ~ 55,-2 29(-2 49 to -2 08)

Bhutan,DALYs,55 ~ 60,-2 24(-2 41 to -2 07)

Bhutan,DALYs,60 ~ 65,-2 06(-2 21 to -1 92)

Bhutan,DALYs,65 ~ 70,-1 75(-1 88 to -1 62)

Bhutan,DALYs,70 ~ 75,-1 36(-1 49 to -1 23)

Bhutan,DALYs,75 ~ 80,-0 88(-1 03 to -0 73)

Bhutan,DALYs,80 ~ 85,-0 35(-0 55 to -0 14)

Bhutan,DALYs,85 ~ 90,0 13(-0 19 to 0 46)

Bhutan,DALYs,90 ~ 95,0 49(-0 16 to 1 14)

Bhutan,DALYs,20 ~ 25,-1 88(-2 67 to -1 09)

Bhutan,DALYs,25 ~ 30,-1 91(-2 46 to -1 36)

Bhutan,DALYs,30 ~ 35,-1 96(-2 4 to -1 52)

Bhutan,DALYs,35 ~ 40,-2 05(-2 42 to -1 68)

Bhutan,DALYs,40 ~ 45,-2 16(-2 47 to -1 85)

Bhutan,DALYs,45 ~ 50,-2 26(-2 51 to -2)

Bhutan,DALYs,50 ~ 55,-2 29(-2 49 to -2 08)

Bhutan,DALYs,55 ~ 60,-2 24(-2 41 to -2 07)

Bhutan,DALYs,60 ~ 65,-2 06(-2 21 to -1 92)

Bhutan,DALYs,65 ~ 70,-1 75(-1 88 to -1 62)

Bhutan,DALYs,70 ~ 75,-1 36(-1 49 to -1 23)

Bhutan,DALYs,75 ~ 80,-0 88(-1 03 to -0 73)

Bhutan,DALYs,80 ~ 85,-0 35(-0 55 to -0 14)

Bhutan,DALYs,85 ~ 90,0 13(-0 19 to 0 46)

Bhutan,DALYs,90 ~ 95,0 49(-0 16 to 1 14)

Greenland,Prevalence,20 ~ 25,-0 2(-2 08 to 1 71)

Greenland,Prevalence,25 ~ 30,-0 23(-1 5 to 1 06)

Greenland,Prevalence,30 ~ 35,-0 25(-1 25 to 0 75)

Greenland,Prevalence,35 ~ 40,-0 28(-1 1 to 0 56)

Greenland,Prevalence,40 ~ 45,-0 31(-0 99 to 0 38)

Greenland,Prevalence,45 ~ 50,-0 34(-0 87 to 0 2)

Greenland,Prevalence,50 ~ 55,-0 33(-0 73 to 0 06)

Greenland,Prevalence,55 ~ 60,-0 33(-0 66 to 0 01)

Greenland,Prevalence,60 ~ 65,-0 32(-0 64 to 0)

Greenland,Prevalence,65 ~ 70,-0 29(-0 62 to 0 03)

Greenland,Prevalence,70 ~ 75,-0 25(-0 61 to 0 11)

Greenland,Prevalence,75 ~ 80,-0 19(-0 62 to 0 25)

Greenland,Prevalence,80 ~ 85,-0 11(-0 71 to 0 49)

Greenland,Prevalence,85 ~ 90,-0 04(-1 01 to 0 94)

Greenland,Prevalence,90 ~ 95,0 03(-2 14 to 2 25)

Greenland,Prevalence,20 ~ 25,-0 2(-2 08 to 1 71)

Greenland,Prevalence,25 ~ 30,-0 23(-1 5 to 1 06)

Greenland,Prevalence,30 ~ 35,-0 25(-1 25 to 0 75)

Greenland,Prevalence,35 ~ 40,-0 28(-1 1 to 0 56)

Greenland,Prevalence,40 ~ 45,-0 31(-0 99 to 0 38)

Greenland,Prevalence,45 ~ 50,-0 34(-0 87 to 0 2)

Greenland,Prevalence,50 ~ 55,-0 33(-0 73 to 0 06)

Greenland,Prevalence,55 ~ 60,-0 33(-0 66 to 0 01)

Greenland,Prevalence,60 ~ 65,-0 32(-0 64 to 0)

Greenland,Prevalence,65 ~ 70,-0 29(-0 62 to 0 03)

Greenland,Prevalence,70 ~ 75,-0 25(-0 61 to 0 11)

Greenland,Prevalence,75 ~ 80,-0 19(-0 62 to 0 25)

Greenland,Prevalence,80 ~ 85,-0 11(-0 71 to 0 49)

Greenland,Prevalence,85 ~ 90,-0 04(-1 01 to 0 94)

Greenland,Prevalence,90 ~ 95,0 03(-2 14 to 2 25)

Greenland,Prevalence,20 ~ 25,-0 2(-2 08 to 1 71)

Greenland,Prevalence,25 ~ 30,-0 23(-1 5 to 1 06)

Greenland,Prevalence,30 ~ 35,-0 25(-1 25 to 0 75)

Greenland,Prevalence,35 ~ 40,-0 28(-1 1 to 0 56)

Greenland,Prevalence,40 ~ 45,-0 31(-0 99 to 0 38)

Greenland,Prevalence,45 ~ 50,-0 34(-0 87 to 0 2)

Greenland,Prevalence,50 ~ 55,-0 33(-0 73 to 0 06)

Greenland,Prevalence,55 ~ 60,-0 33(-0 66 to 0 01)

Greenland,Prevalence,60 ~ 65,-0 32(-0 64 to 0)

Greenland,Prevalence,65 ~ 70,-0 29(-0 62 to 0 03)

Greenland,Prevalence,70 ~ 75,-0 25(-0 61 to 0 11)

Greenland,Prevalence,75 ~ 80,-0 19(-0 62 to 0 25)

Greenland,Prevalence,80 ~ 85,-0 11(-0 71 to 0 49)

Greenland,Prevalence,85 ~ 90,-0 04(-1 01 to 0 94)

Greenland,Prevalence,90 ~ 95,0 03(-2 14 to 2 25)

Greenland,Prevalence,20 ~ 25,-0 2(-2 08 to 1 71)

Greenland,Prevalence,25 ~ 30,-0 23(-1 5 to 1 06)

Greenland,Prevalence,30 ~ 35,-0 25(-1 25 to 0 75)

Greenland,Prevalence,35 ~ 40,-0 28(-1 1 to 0 56)

Greenland,Prevalence,40 ~ 45,-0 31(-0 99 to 0 38)

Greenland,Prevalence,45 ~ 50,-0 34(-0 87 to 0 2)

Greenland,Prevalence,50 ~ 55,-0 33(-0 73 to 0 06)

Greenland,Prevalence,55 ~ 60,-0 33(-0 66 to 0 01)

Greenland,Prevalence,60 ~ 65,-0 32(-0 64 to 0)

Greenland,Prevalence,65 ~ 70,-0 29(-0 62 to 0 03)

Greenland,Prevalence,70 ~ 75,-0 25(-0 61 to 0 11)

Greenland,Prevalence,75 ~ 80,-0 19(-0 62 to 0 25)

Greenland,Prevalence,80 ~ 85,-0 11(-0 71 to 0 49)

Greenland,Prevalence,85 ~ 90,-0 04(-1 01 to 0 94)

Greenland,Prevalence,90 ~ 95,0 03(-2 14 to 2 25)

Greenland,Deaths,20 ~ 25,-1 12(-63 52 to 168 03)

Greenland,Deaths,25 ~ 30,-1 04(-47 91 to 88)

Greenland,Deaths,30 ~ 35,-1 04(-36 78 to 54 93)

Greenland,Deaths,35 ~ 40,-1 19(-25 17 to 30 49)

Greenland,Deaths,40 ~ 45,-1 46(-16 57 to 16 39)

Greenland,Deaths,45 ~ 50,-1 7(-10 57 to 8 04)

Greenland,Deaths,50 ~ 55,-1 87(-6 71 to 3 21)

Greenland,Deaths,55 ~ 60,-2 07(-5 39 to 1 37)

Greenland,Deaths,60 ~ 65,-2 24(-4 87 to 0 46)

Greenland,Deaths,65 ~ 70,-2 31(-4 62 to 0 06)

Greenland,Deaths,70 ~ 75,-2 38(-4 57 to -0 14)

Greenland,Deaths,75 ~ 80,-2 31(-4 6 to 0 04)

Greenland,Deaths,80 ~ 85,-2 15(-5 to 0 79)

Greenland,Deaths,85 ~ 90,-1 71(-5 9 to 2 68)

Greenland,Deaths,90 ~ 95,-0 91(-9 67 to 8 69)

Greenland,Deaths,20 ~ 25,-1 12(-63 52 to 168 03)

Greenland,Deaths,25 ~ 30,-1 04(-47 91 to 88)

Greenland,Deaths,30 ~ 35,-1 04(-36 78 to 54 93)

Greenland,Deaths,35 ~ 40,-1 19(-25 17 to 30 49)

Greenland,Deaths,40 ~ 45,-1 46(-16 57 to 16 39)

Greenland,Deaths,45 ~ 50,-1 7(-10 57 to 8 04)

Greenland,Deaths,50 ~ 55,-1 87(-6 71 to 3 21)

Greenland,Deaths,55 ~ 60,-2 07(-5 39 to 1 37)

Greenland,Deaths,60 ~ 65,-2 24(-4 87 to 0 46)

Greenland,Deaths,65 ~ 70,-2 31(-4 62 to 0 06)

Greenland,Deaths,70 ~ 75,-2 38(-4 57 to -0 14)

Greenland,Deaths,75 ~ 80,-2 31(-4 6 to 0 04)

Greenland,Deaths,80 ~ 85,-2 15(-5 to 0 79)

Greenland,Deaths,85 ~ 90,-1 71(-5 9 to 2 68)

Greenland,Deaths,90 ~ 95,-0 91(-9 67 to 8 69)

Greenland,Deaths,20 ~ 25,-1 12(-63 52 to 168 03)

Greenland,Deaths,25 ~ 30,-1 04(-47 91 to 88)

Greenland,Deaths,30 ~ 35,-1 04(-36 78 to 54 93)

Greenland,Deaths,35 ~ 40,-1 19(-25 17 to 30 49)

Greenland,Deaths,40 ~ 45,-1 46(-16 57 to 16 39)

Greenland,Deaths,45 ~ 50,-1 7(-10 57 to 8 04)

Greenland,Deaths,50 ~ 55,-1 87(-6 71 to 3 21)

Greenland,Deaths,55 ~ 60,-2 07(-5 39 to 1 37)

Greenland,Deaths,60 ~ 65,-2 24(-4 87 to 0 46)

Greenland,Deaths,65 ~ 70,-2 31(-4 62 to 0 06)

Greenland,Deaths,70 ~ 75,-2 38(-4 57 to -0 14)

Greenland,Deaths,75 ~ 80,-2 31(-4 6 to 0 04)

Greenland,Deaths,80 ~ 85,-2 15(-5 to 0 79)

Greenland,Deaths,85 ~ 90,-1 71(-5 9 to 2 68)

Greenland,Deaths,90 ~ 95,-0 91(-9 67 to 8 69)

Greenland,Deaths,20 ~ 25,-1 12(-63 52 to 168 03)

Greenland,Deaths,25 ~ 30,-1 04(-47 91 to 88)

Greenland,Deaths,30 ~ 35,-1 04(-36 78 to 54 93)

Greenland,Deaths,35 ~ 40,-1 19(-25 17 to 30 49)

Greenland,Deaths,40 ~ 45,-1 46(-16 57 to 16 39)

Greenland,Deaths,45 ~ 50,-1 7(-10 57 to 8 04)

Greenland,Deaths,50 ~ 55,-1 87(-6 71 to 3 21)

Greenland,Deaths,55 ~ 60,-2 07(-5 39 to 1 37)

Greenland,Deaths,60 ~ 65,-2 24(-4 87 to 0 46)

Greenland,Deaths,65 ~ 70,-2 31(-4 62 to 0 06)

Greenland,Deaths,70 ~ 75,-2 38(-4 57 to -0 14)

Greenland,Deaths,75 ~ 80,-2 31(-4 6 to 0 04)

Greenland,Deaths,80 ~ 85,-2 15(-5 to 0 79)

Greenland,Deaths,85 ~ 90,-1 71(-5 9 to 2 68)

Greenland,Deaths,90 ~ 95,-0 91(-9 67 to 8 69)

Greenland,DALYs,20 ~ 25,-0 48(-6 86 to 6 34)

Greenland,DALYs,25 ~ 30,-0 49(-4 81 to 4 02)

Greenland,DALYs,30 ~ 35,-0 57(-3 82 to 2 79)

Greenland,DALYs,35 ~ 40,-0 76(-3 21 to 1 74)

Greenland,DALYs,40 ~ 45,-1 04(-2 81 to 0 76)

Greenland,DALYs,45 ~ 50,-1 32(-2 49 to -0 13)

Greenland,DALYs,50 ~ 55,-1 52(-2 24 to -0 79)

Greenland,DALYs,55 ~ 60,-1 73(-2 27 to -1 19)

Greenland,DALYs,60 ~ 65,-1 94(-2 4 to -1 48)

Greenland,DALYs,65 ~ 70,-2 07(-2 52 to -1 62)

Greenland,DALYs,70 ~ 75,-2 19(-2 67 to -1 72)

Greenland,DALYs,75 ~ 80,-2 19(-2 74 to -1 62)

Greenland,DALYs,80 ~ 85,-2 05(-2 82 to -1 28)

Greenland,DALYs,85 ~ 90,-1 65(-2 91 to -0 38)

Greenland,DALYs,90 ~ 95,-0 93(-3 8 to 2 03)

Greenland,DALYs,20 ~ 25,-0 48(-6 86 to 6 34)

Greenland,DALYs,25 ~ 30,-0 49(-4 81 to 4 02)

Greenland,DALYs,30 ~ 35,-0 57(-3 82 to 2 79)

Greenland,DALYs,35 ~ 40,-0 76(-3 21 to 1 74)

Greenland,DALYs,40 ~ 45,-1 04(-2 81 to 0 76)

Greenland,DALYs,45 ~ 50,-1 32(-2 49 to -0 13)

Greenland,DALYs,50 ~ 55,-1 52(-2 24 to -0 79)

Greenland,DALYs,55 ~ 60,-1 73(-2 27 to -1 19)

Greenland,DALYs,60 ~ 65,-1 94(-2 4 to -1 48)

Greenland,DALYs,65 ~ 70,-2 07(-2 52 to -1 62)

Greenland,DALYs,70 ~ 75,-2 19(-2 67 to -1 72)

Greenland,DALYs,75 ~ 80,-2 19(-2 74 to -1 62)

Greenland,DALYs,80 ~ 85,-2 05(-2 82 to -1 28)

Greenland,DALYs,85 ~ 90,-1 65(-2 91 to -0 38)

Greenland,DALYs,90 ~ 95,-0 93(-3 8 to 2 03)

Greenland,DALYs,20 ~ 25,-0 48(-6 86 to 6 34)

Greenland,DALYs,25 ~ 30,-0 49(-4 81 to 4 02)

Greenland,DALYs,30 ~ 35,-0 57(-3 82 to 2 79)

Greenland,DALYs,35 ~ 40,-0 76(-3 21 to 1 74)

Greenland,DALYs,40 ~ 45,-1 04(-2 81 to 0 76)

Greenland,DALYs,45 ~ 50,-1 32(-2 49 to -0 13)

Greenland,DALYs,50 ~ 55,-1 52(-2 24 to -0 79)

Greenland,DALYs,55 ~ 60,-1 73(-2 27 to -1 19)

Greenland,DALYs,60 ~ 65,-1 94(-2 4 to -1 48)

Greenland,DALYs,65 ~ 70,-2 07(-2 52 to -1 62)

Greenland,DALYs,70 ~ 75,-2 19(-2 67 to -1 72)

Greenland,DALYs,75 ~ 80,-2 19(-2 74 to -1 62)

Greenland,DALYs,80 ~ 85,-2 05(-2 82 to -1 28)

Greenland,DALYs,85 ~ 90,-1 65(-2 91 to -0 38)

Greenland,DALYs,90 ~ 95,-0 93(-3 8 to 2 03)

Greenland,DALYs,20 ~ 25,-0 48(-6 86 to 6 34)

Greenland,DALYs,25 ~ 30,-0 49(-4 81 to 4 02)

Greenland,DALYs,30 ~ 35,-0 57(-3 82 to 2 79)

Greenland,DALYs,35 ~ 40,-0 76(-3 21 to 1 74)

Greenland,DALYs,40 ~ 45,-1 04(-2 81 to 0 76)

Greenland,DALYs,45 ~ 50,-1 32(-2 49 to -0 13)

Greenland,DALYs,50 ~ 55,-1 52(-2 24 to -0 79)

Greenland,DALYs,55 ~ 60,-1 73(-2 27 to -1 19)

Greenland,DALYs,60 ~ 65,-1 94(-2 4 to -1 48)

Greenland,DALYs,65 ~ 70,-2 07(-2 52 to -1 62)

Greenland,DALYs,70 ~ 75,-2 19(-2 67 to -1 72)

Greenland,DALYs,75 ~ 80,-2 19(-2 74 to -1 62)

Greenland,DALYs,80 ~ 85,-2 05(-2 82 to -1 28)

Greenland,DALYs,85 ~ 90,-1 65(-2 91 to -0 38)

Greenland,DALYs,90 ~ 95,-0 93(-3 8 to 2 03)

Greece,Prevalence,20 ~ 25,0 12(0 01 to 0 23)

Greece,Prevalence,25 ~ 30,0 11(0 04 to 0 19)

Greece,Prevalence,30 ~ 35,0 09(0 03 to 0 14)

Greece,Prevalence,35 ~ 40,0 04(0 to 0 09)

Greece,Prevalence,40 ~ 45,-0 01(-0 04 to 0 03)

Greece,Prevalence,45 ~ 50,-0 06(-0 09 to -0 03)

Greece,Prevalence,50 ~ 55,-0 09(-0 12 to -0 07)

Greece,Prevalence,55 ~ 60,-0 11(-0 13 to -0 09)

Greece,Prevalence,60 ~ 65,-0 11(-0 13 to -0 09)

Greece,Prevalence,65 ~ 70,-0 11(-0 12 to -0 09)

Greece,Prevalence,70 ~ 75,-0 08(-0 1 to -0 07)

Greece,Prevalence,75 ~ 80,-0 03(-0 05 to -0 02)

Greece,Prevalence,80 ~ 85,0 04(0 02 to 0 06)

Greece,Prevalence,85 ~ 90,0 14(0 11 to 0 16)

Greece,Prevalence,90 ~ 95,0 22(0 17 to 0 27)

Greece,Prevalence,20 ~ 25,0 12(0 01 to 0 23)

Greece,Prevalence,25 ~ 30,0 11(0 04 to 0 19)

Greece,Prevalence,30 ~ 35,0 09(0 03 to 0 14)

Greece,Prevalence,35 ~ 40,0 04(0 to 0 09)

Greece,Prevalence,40 ~ 45,-0 01(-0 04 to 0 03)

Greece,Prevalence,45 ~ 50,-0 06(-0 09 to -0 03)
[truncated: 1,385,756 more chars]
