# Supplementary material for: Global, regional, and national temporal trends in prevalence, deaths and disability-adjusted life years for chronic pulmonary disease, 1990–2021: an age-period-cohort analysis based on the global burden of disease study 2021
Source: Front Med (Lausanne). 2025 Mar 4;12:1554442. doi: 10.3389/fmed.2025.1554442 (PMC11913687; doi:10.3389/fmed.2025.1554442)
Supplement: Supplementary file 6 [file Table_4.docx]

**Table S4 Age effects on COPD related Prevalence, Deaths and DALYs in WCBA across SDI quintiles**

| **Region** | **measure** | **age** | **Age_effect_rate（95%UI）** |
| --- | --- | --- | --- |
| global | Prevalence | 20 ~ 25 | 483.46(476.53 to 490.48) |
| global | Prevalence | 25 ~ 30 | 653.83(645.82 to 661.93) |
| global | Prevalence | 30 ~ 35 | 830.89(821.85 to 840.04) |
| global | Prevalence | 35 ~ 40 | 1008.39(998.41 to 1018.47) |
| global | Prevalence | 40 ~ 45 | 1324.58(1313.04 to 1336.22) |
| global | Prevalence | 45 ~ 50 | 2198.54(2182.38 to 2214.82) |
| global | Prevalence | 50 ~ 55 | 3520.59(3498.18 to 3543.16) |
| global | Prevalence | 55 ~ 60 | 5021.69(4992.56 to 5050.98) |
| global | Prevalence | 60 ~ 65 | 6904.49(6866.89 to 6942.29) |
| global | Prevalence | 65 ~ 70 | 10075.47(10023.21 to 10128.01) |
| global | Prevalence | 70 ~ 75 | 14181.39(14107.8 to 14255.37) |
| global | Prevalence | 75 ~ 80 | 19039.43(18923.9 to 19155.66) |
| global | Prevalence | 80 ~ 85 | 24406.48(24246.19 to 24567.84) |
| global | Prevalence | 85 ~ 90 | 30584.92(30352.49 to 30819.13) |
| global | Prevalence | 90 ~ 95 | 36653.74(36283.22 to 37028.03) |
| global | Deaths | 20 ~ 25 | 1.1(0.99 to 1.23) |
| global | Deaths | 25 ~ 30 | 1.42(1.3 to 1.55) |
| global | Deaths | 30 ~ 35 | 2.13(1.98 to 2.28) |
| global | Deaths | 35 ~ 40 | 3.27(3.1 to 3.46) |
| global | Deaths | 40 ~ 45 | 5.99(5.75 to 6.24) |
| global | Deaths | 45 ~ 50 | 11.18(10.84 to 11.52) |
| global | Deaths | 50 ~ 55 | 23.73(23.21 to 24.26) |
| global | Deaths | 55 ~ 60 | 43.31(42.54 to 44.09) |
| global | Deaths | 60 ~ 65 | 86.65(85.4 to 87.92) |
| global | Deaths | 65 ~ 70 | 145.04(143.14 to 146.95) |
| global | Deaths | 70 ~ 75 | 255.49(252.36 to 258.66) |
| global | Deaths | 75 ~ 80 | 380.92(375.68 to 386.23) |
| global | Deaths | 80 ~ 85 | 573.7(565.67 to 581.85) |
| global | Deaths | 85 ~ 90 | 817.02(804.94 to 829.28) |
| global | Deaths | 90 ~ 95 | 967.56(950.81 to 984.61) |
| global | DALYs | 20 ~ 25 | 118.96(113.1 to 125.12) |
| global | DALYs | 25 ~ 30 | 149.46(143.2 to 155.99) |
| global | DALYs | 30 ~ 35 | 195.79(188.79 to 203.06) |
| global | DALYs | 35 ~ 40 | 255.67(247.79 to 263.81) |
| global | DALYs | 40 ~ 45 | 388.48(378.74 to 398.47) |
| global | DALYs | 45 ~ 50 | 647.73(634.52 to 661.21) |
| global | DALYs | 50 ~ 55 | 1162.51(1143.43 to 1181.91) |
| global | DALYs | 55 ~ 60 | 1806.23(1780.42 to 1832.42) |
| global | DALYs | 60 ~ 65 | 2989.79(2952.24 to 3027.83) |
| global | DALYs | 65 ~ 70 | 4234.43(4184.06 to 4285.41) |
| global | DALYs | 70 ~ 75 | 6097.77(6026.93 to 6169.44) |
| global | DALYs | 75 ~ 80 | 7341.59(7243.26 to 7441.26) |
| global | DALYs | 80 ~ 85 | 8680.66(8556.37 to 8806.75) |
| global | DALYs | 85 ~ 90 | 9844.04(9682.3 to 10008.48) |
| global | DALYs | 90 ~ 95 | 10234.26(10007.82 to 10465.84) |
| high_sdi | Prevalence | 20 ~ 25 | 390.28(381.2 to 399.59) |
| high_sdi | Prevalence | 25 ~ 30 | 519.76(509.88 to 529.83) |
| high_sdi | Prevalence | 30 ~ 35 | 669.56(658.82 to 680.47) |
| high_sdi | Prevalence | 35 ~ 40 | 835.11(823.49 to 846.89) |
| high_sdi | Prevalence | 40 ~ 45 | 1165.92(1152.43 to 1179.58) |
| high_sdi | Prevalence | 45 ~ 50 | 2113.06(2093.76 to 2132.54) |
| high_sdi | Prevalence | 50 ~ 55 | 3508.66(3482.05 to 3535.49) |
| high_sdi | Prevalence | 55 ~ 60 | 5067.8(5033.68 to 5102.16) |
| high_sdi | Prevalence | 60 ~ 65 | 7101.85(7057.84 to 7146.12) |
| high_sdi | Prevalence | 65 ~ 70 | 10652.83(10591.27 to 10714.75) |
| high_sdi | Prevalence | 70 ~ 75 | 15065.48(14980.05 to 15151.4) |
| high_sdi | Prevalence | 75 ~ 80 | 20274.77(20142.43 to 20407.98) |
| high_sdi | Prevalence | 80 ~ 85 | 26077.71(25899.13 to 26257.53) |
| high_sdi | Prevalence | 85 ~ 90 | 32408.98(32165.91 to 32653.89) |
| high_sdi | Prevalence | 90 ~ 95 | 38187.19(37838.37 to 38539.23) |
| high_sdi | Deaths | 20 ~ 25 | 0.14(0.1 to 0.19) |
| high_sdi | Deaths | 25 ~ 30 | 0.19(0.15 to 0.23) |
| high_sdi | Deaths | 30 ~ 35 | 0.3(0.25 to 0.36) |
| high_sdi | Deaths | 35 ~ 40 | 0.55(0.49 to 0.63) |
| high_sdi | Deaths | 40 ~ 45 | 1.28(1.18 to 1.39) |
| high_sdi | Deaths | 45 ~ 50 | 3.11(2.94 to 3.28) |
| high_sdi | Deaths | 50 ~ 55 | 7.75(7.47 to 8.04) |
| high_sdi | Deaths | 55 ~ 60 | 16.56(16.12 to 17.02) |
| high_sdi | Deaths | 60 ~ 65 | 34.99(34.25 to 35.75) |
| high_sdi | Deaths | 65 ~ 70 | 63.08(61.9 to 64.27) |
| high_sdi | Deaths | 70 ~ 75 | 113.1(111.12 to 115.11) |
| high_sdi | Deaths | 75 ~ 80 | 182.23(178.68 to 185.85) |
| high_sdi | Deaths | 80 ~ 85 | 272.91(267.55 to 278.38) |
| high_sdi | Deaths | 85 ~ 90 | 407.24(399.04 to 415.61) |
| high_sdi | Deaths | 90 ~ 95 | 547.36(535.44 to 559.54) |
| high_sdi | DALYs | 20 ~ 25 | 32.29(29.49 to 35.36) |
| high_sdi | DALYs | 25 ~ 30 | 41.45(38.49 to 44.64) |
| high_sdi | DALYs | 30 ~ 35 | 55.37(52.11 to 58.83) |
| high_sdi | DALYs | 35 ~ 40 | 76.06(72.34 to 79.96) |
| high_sdi | DALYs | 40 ~ 45 | 126.51(121.72 to 131.49) |
| high_sdi | DALYs | 45 ~ 50 | 254.2(247.02 to 261.6) |
| high_sdi | DALYs | 50 ~ 55 | 496.56(485.7 to 507.66) |
| high_sdi | DALYs | 55 ~ 60 | 844.13(828.76 to 859.78) |
| high_sdi | DALYs | 60 ~ 65 | 1418.94(1396.57 to 1441.67) |
| high_sdi | DALYs | 65 ~ 70 | 2150.14(2118.77 to 2181.98) |
| high_sdi | DALYs | 70 ~ 75 | 3130.86(3086.36 to 3175.99) |
| high_sdi | DALYs | 75 ~ 80 | 4048.52(3983 to 4115.12) |
| high_sdi | DALYs | 80 ~ 85 | 4801.14(4719.83 to 4883.85) |
| high_sdi | DALYs | 85 ~ 90 | 5692.18(5587.61 to 5798.7) |
| high_sdi | DALYs | 90 ~ 95 | 6614.43(6469.02 to 6763.12) |
| high-middle_sdi | Prevalence | 20 ~ 25 | 517.18(499.68 to 535.3) |
| high-middle_sdi | Prevalence | 25 ~ 30 | 696.46(676.77 to 716.71) |
| high-middle_sdi | Prevalence | 30 ~ 35 | 879.11(857.45 to 901.31) |
| high-middle_sdi | Prevalence | 35 ~ 40 | 1058.57(1035.18 to 1082.5) |
| high-middle_sdi | Prevalence | 40 ~ 45 | 1363.56(1337.29 to 1390.35) |
| high-middle_sdi | Prevalence | 45 ~ 50 | 2175.02(2139.76 to 2210.87) |
| high-middle_sdi | Prevalence | 50 ~ 55 | 3419.06(3371.48 to 3467.31) |
| high-middle_sdi | Prevalence | 55 ~ 60 | 4872.91(4811.6 to 4934.99) |
| high-middle_sdi | Prevalence | 60 ~ 65 | 6640.58(6562.21 to 6719.89) |
| high-middle_sdi | Prevalence | 65 ~ 70 | 9421.2(9314.77 to 9528.84) |
| high-middle_sdi | Prevalence | 70 ~ 75 | 13108.7(12960.72 to 13258.37) |
| high-middle_sdi | Prevalence | 75 ~ 80 | 17545.12(17316.3 to 17776.95) |
| high-middle_sdi | Prevalence | 80 ~ 85 | 22548.62(22233.63 to 22868.07) |
| high-middle_sdi | Prevalence | 85 ~ 90 | 28507.09(28047.32 to 28974.4) |
| high-middle_sdi | Prevalence | 90 ~ 95 | 34434.97(33685.2 to 35201.42) |
| high-middle_sdi | Deaths | 20 ~ 25 | 1.64(1.37 to 1.96) |
| high-middle_sdi | Deaths | 25 ~ 30 | 1.8(1.56 to 2.09) |
| high-middle_sdi | Deaths | 30 ~ 35 | 2.63(2.36 to 2.93) |
| high-middle_sdi | Deaths | 35 ~ 40 | 3.88(3.57 to 4.21) |
| high-middle_sdi | Deaths | 40 ~ 45 | 6.74(6.36 to 7.14) |
| high-middle_sdi | Deaths | 45 ~ 50 | 10.6(10.13 to 11.09) |
| high-middle_sdi | Deaths | 50 ~ 55 | 19.28(18.65 to 19.93) |
| high-middle_sdi | Deaths | 55 ~ 60 | 31.42(30.59 to 32.28) |
| high-middle_sdi | Deaths | 60 ~ 65 | 54.71(53.48 to 55.97) |
| high-middle_sdi | Deaths | 65 ~ 70 | 86.53(84.75 to 88.35) |
| high-middle_sdi | Deaths | 70 ~ 75 | 153.13(150.17 to 156.14) |
| high-middle_sdi | Deaths | 75 ~ 80 | 238.18(233.21 to 243.25) |
| high-middle_sdi | Deaths | 80 ~ 85 | 373.86(366.03 to 381.85) |
| high-middle_sdi | Deaths | 85 ~ 90 | 593.25(580.5 to 606.29) |
| high-middle_sdi | Deaths | 90 ~ 95 | 730.19(712.69 to 748.12) |
| high-middle_sdi | DALYs | 20 ~ 25 | 145.44(133.86 to 158.02) |
| high-middle_sdi | DALYs | 25 ~ 30 | 169.28(157.91 to 181.47) |
| high-middle_sdi | DALYs | 30 ~ 35 | 217.36(205.24 to 230.21) |
| high-middle_sdi | DALYs | 35 ~ 40 | 276.36(263.41 to 289.94) |
| high-middle_sdi | DALYs | 40 ~ 45 | 404.89(389.89 to 420.47) |
| high-middle_sdi | DALYs | 45 ~ 50 | 593.91(575.72 to 612.67) |
| high-middle_sdi | DALYs | 50 ~ 55 | 949.94(926.31 to 974.18) |
| high-middle_sdi | DALYs | 55 ~ 60 | 1355.25(1325.64 to 1385.52) |
| high-middle_sdi | DALYs | 60 ~ 65 | 1995.12(1955.79 to 2035.23) |
| high-middle_sdi | DALYs | 65 ~ 70 | 2686.4(2635.96 to 2737.81) |
| high-middle_sdi | DALYs | 70 ~ 75 | 3881.08(3810.45 to 3953.02) |
| high-middle_sdi | DALYs | 75 ~ 80 | 4861.41(4763.43 to 4961.41) |
| high-middle_sdi | DALYs | 80 ~ 85 | 5971.79(5846.42 to 6099.86) |
| high-middle_sdi | DALYs | 85 ~ 90 | 7484.33(7312.33 to 7660.36) |
| high-middle_sdi | DALYs | 90 ~ 95 | 8061.89(7825.68 to 8305.24) |
| middle_sdi | Prevalence | 20 ~ 25 | 502.18(491.29 to 513.31) |
| middle_sdi | Prevalence | 25 ~ 30 | 677.91(665.34 to 690.71) |
| middle_sdi | Prevalence | 30 ~ 35 | 859.04(844.8 to 873.53) |
| middle_sdi | Prevalence | 35 ~ 40 | 1038.46(1022.72 to 1054.44) |
| middle_sdi | Prevalence | 40 ~ 45 | 1338.38(1320.35 to 1356.66) |
| middle_sdi | Prevalence | 45 ~ 50 | 2137.79(2113.07 to 2162.8) |
| middle_sdi | Prevalence | 50 ~ 55 | 3408.55(3374.11 to 3443.35) |
| middle_sdi | Prevalence | 55 ~ 60 | 4889.19(4843.93 to 4934.87) |
| middle_sdi | Prevalence | 60 ~ 65 | 6732.44(6673.58 to 6791.82) |
| middle_sdi | Prevalence | 65 ~ 70 | 9802.82(9720.78 to 9885.55) |
| middle_sdi | Prevalence | 70 ~ 75 | 13937.52(13820.21 to 14055.83) |
| middle_sdi | Prevalence | 75 ~ 80 | 18827.63(18639.75 to 19017.41) |
| middle_sdi | Prevalence | 80 ~ 85 | 24025.11(23759.43 to 24293.75) |
| middle_sdi | Prevalence | 85 ~ 90 | 29761.06(29362.04 to 30165.5) |
| middle_sdi | Prevalence | 90 ~ 95 | 35331.37(34648.24 to 36027.95) |
| middle_sdi | Deaths | 20 ~ 25 | 1.95(1.71 to 2.22) |
| middle_sdi | Deaths | 25 ~ 30 | 2.34(2.1 to 2.6) |
| middle_sdi | Deaths | 30 ~ 35 | 3.43(3.15 to 3.72) |
| middle_sdi | Deaths | 35 ~ 40 | 4.91(4.6 to 5.25) |
| middle_sdi | Deaths | 40 ~ 45 | 8.37(7.97 to 8.79) |
| middle_sdi | Deaths | 45 ~ 50 | 13.63(13.12 to 14.16) |
| middle_sdi | Deaths | 50 ~ 55 | 27.87(27.11 to 28.66) |
| middle_sdi | Deaths | 55 ~ 60 | 47.02(45.96 to 48.11) |
| middle_sdi | Deaths | 60 ~ 65 | 90.67(88.98 to 92.4) |
| middle_sdi | Deaths | 65 ~ 70 | 150.04(147.5 to 152.62) |
| middle_sdi | Deaths | 70 ~ 75 | 276.52(272.17 to 280.95) |
| middle_sdi | Deaths | 75 ~ 80 | 418.38(411.06 to 425.84) |
| middle_sdi | Deaths | 80 ~ 85 | 646.7(635.19 to 658.42) |
| middle_sdi | Deaths | 85 ~ 90 | 989.25(970.74 to 1008.1) |
| middle_sdi | Deaths | 90 ~ 95 | 1165.42(1139.63 to 1191.8) |
| middle_sdi | DALYs | 20 ~ 25 | 176.52(165.69 to 188.07) |
| middle_sdi | DALYs | 25 ~ 30 | 212.95(201.83 to 224.7) |
| middle_sdi | DALYs | 30 ~ 35 | 276.74(264.42 to 289.63) |
| middle_sdi | DALYs | 35 ~ 40 | 348.63(335.2 to 362.59) |
| middle_sdi | DALYs | 40 ~ 45 | 506.13(490.23 to 522.54) |
| middle_sdi | DALYs | 45 ~ 50 | 758.38(738.39 to 778.91) |
| middle_sdi | DALYs | 50 ~ 55 | 1325.92(1297.76 to 1354.7) |
| middle_sdi | DALYs | 55 ~ 60 | 1942(1905.53 to 1979.17) |
| middle_sdi | DALYs | 60 ~ 65 | 3123.36(3071.29 to 3176.31) |
| middle_sdi | DALYs | 65 ~ 70 | 4372.88(4303.86 to 4443.02) |
| middle_sdi | DALYs | 70 ~ 75 | 6566.48(6466.15 to 6668.37) |
| middle_sdi | DALYs | 75 ~ 80 | 8013.29(7873.51 to 8155.56) |
| middle_sdi | DALYs | 80 ~ 85 | 9704.37(9523.78 to 9888.4) |
| middle_sdi | DALYs | 85 ~ 90 | 11733.36(11483.91 to 11988.22) |
| middle_sdi | DALYs | 90 ~ 95 | 12090.77(11736.08 to 12456.18) |
| low-middle_sdi | Prevalence | 20 ~ 25 | 494.12(490.45 to 497.83) |
| low-middle_sdi | Prevalence | 25 ~ 30 | 678.34(673.92 to 682.78) |
| low-middle_sdi | Prevalence | 30 ~ 35 | 868.6(863.48 to 873.76) |
| low-middle_sdi | Prevalence | 35 ~ 40 | 1055.35(1049.59 to 1061.15) |
| low-middle_sdi | Prevalence | 40 ~ 45 | 1407.85(1400.98 to 1414.75) |
| low-middle_sdi | Prevalence | 45 ~ 50 | 2431.34(2421.2 to 2441.52) |
| low-middle_sdi | Prevalence | 50 ~ 55 | 3895.68(3881.27 to 3910.15) |
| low-middle_sdi | Prevalence | 55 ~ 60 | 5445.85(5427.15 to 5464.61) |
| low-middle_sdi | Prevalence | 60 ~ 65 | 7387.59(7363.57 to 7411.68) |
| low-middle_sdi | Prevalence | 65 ~ 70 | 10888.69(10854.69 to 10922.81) |
| low-middle_sdi | Prevalence | 70 ~ 75 | 15334.93(15286.26 to 15383.76) |
| low-middle_sdi | Prevalence | 75 ~ 80 | 20416.91(20339.61 to 20494.5) |
| low-middle_sdi | Prevalence | 80 ~ 85 | 25697.44(25587.56 to 25807.8) |
| low-middle_sdi | Prevalence | 85 ~ 90 | 31939.43(31770.38 to 32109.39) |
| low-middle_sdi | Prevalence | 90 ~ 95 | 38823.24(38527.32 to 39121.44) |
| low-middle_sdi | Deaths | 20 ~ 25 | 0.99(0.78 to 1.25) |
| low-middle_sdi | Deaths | 25 ~ 30 | 1.42(1.18 to 1.72) |
| low-middle_sdi | Deaths | 30 ~ 35 | 2.24(1.93 to 2.6) |
| low-middle_sdi | Deaths | 35 ~ 40 | 3.75(3.34 to 4.23) |
| low-middle_sdi | Deaths | 40 ~ 45 | 7.48(6.85 to 8.17) |
| low-middle_sdi | Deaths | 45 ~ 50 | 16.42(15.41 to 17.51) |
| low-middle_sdi | Deaths | 50 ~ 55 | 39.17(37.4 to 41.04) |
| low-middle_sdi | Deaths | 55 ~ 60 | 79.23(76.36 to 82.2) |
| low-middle_sdi | Deaths | 60 ~ 65 | 173.28(168.27 to 178.43) |
| low-middle_sdi | Deaths | 65 ~ 70 | 304.77(296.78 to 312.98) |
| low-middle_sdi | Deaths | 70 ~ 75 | 543.9(530.37 to 557.78) |
| low-middle_sdi | Deaths | 75 ~ 80 | 830.8(806.69 to 855.62) |
| low-middle_sdi | Deaths | 80 ~ 85 | 1343.53(1302.92 to 1385.4) |
| low-middle_sdi | Deaths | 85 ~ 90 | 1786.75(1726.02 to 1849.61) |
| low-middle_sdi | Deaths | 90 ~ 95 | 2335.07(2234.36 to 2440.32) |
| low-middle_sdi | DALYs | 20 ~ 25 | 120.54(109.79 to 132.34) |
| low-middle_sdi | DALYs | 25 ~ 30 | 161.84(149.48 to 175.21) |
| low-middle_sdi | DALYs | 30 ~ 35 | 218.8(204.3 to 234.33) |
| low-middle_sdi | DALYs | 35 ~ 40 | 302.01(284.6 to 320.48) |
| low-middle_sdi | DALYs | 40 ~ 45 | 491.18(467.88 to 515.65) |
| low-middle_sdi | DALYs | 45 ~ 50 | 932.88(897.55 to 969.6) |
| low-middle_sdi | DALYs | 50 ~ 55 | 1846.55(1790.29 to 1904.58) |
| low-middle_sdi | DALYs | 55 ~ 60 | 3127.78(3045.4 to 3212.39) |
| low-middle_sdi | DALYs | 60 ~ 65 | 5632.68(5504.97 to 5763.35) |
| low-middle_sdi | DALYs | 65 ~ 70 | 8349.34(8169.14 to 8533.51) |
| low-middle_sdi | DALYs | 70 ~ 75 | 12200.7(11939.94 to 12467.16) |
| low-middle_sdi | DALYs | 75 ~ 80 | 14966.27(14582.28 to 15360.36) |
| low-middle_sdi | DALYs | 80 ~ 85 | 18866.54(18330.83 to 19417.91) |
| low-middle_sdi | DALYs | 85 ~ 90 | 20126.23(19404.65 to 20874.66) |
| low-middle_sdi | DALYs | 90 ~ 95 | 22800.99(21603.25 to 24065.14) |
| low_sdi | Prevalence | 20 ~ 25 | 489.75(482.36 to 497.24) |
| low_sdi | Prevalence | 25 ~ 30 | 666.31(657.35 to 675.4) |
| low_sdi | Prevalence | 30 ~ 35 | 853.97(843.39 to 864.69) |
| low_sdi | Prevalence | 35 ~ 40 | 1041.36(1029.26 to 1053.59) |
| low_sdi | Prevalence | 40 ~ 45 | 1362.01(1347.62 to 1376.56) |
| low_sdi | Prevalence | 45 ~ 50 | 2232.97(2212.13 to 2254.01) |
| low_sdi | Prevalence | 50 ~ 55 | 3511.63(3482.04 to 3541.46) |
| low_sdi | Prevalence | 55 ~ 60 | 4929.6(4890.67 to 4968.84) |
| low_sdi | Prevalence | 60 ~ 65 | 6669.62(6619.07 to 6720.56) |
| low_sdi | Prevalence | 65 ~ 70 | 9502.39(9432.18 to 9573.12) |
| low_sdi | Prevalence | 70 ~ 75 | 12863.09(12764.78 to 12962.16) |
| low_sdi | Prevalence | 75 ~ 80 | 16627.63(16473.86 to 16782.84) |
| low_sdi | Prevalence | 80 ~ 85 | 20458.17(20236.89 to 20681.87) |
| low_sdi | Prevalence | 85 ~ 90 | 24404.99(24055.57 to 24759.48) |
| low_sdi | Prevalence | 90 ~ 95 | 28177.97(27527.3 to 28844.03) |
| low_sdi | Deaths | 20 ~ 25 | 0.91(0.7 to 1.2) |
| low_sdi | Deaths | 25 ~ 30 | 1.38(1.11 to 1.72) |
| low_sdi | Deaths | 30 ~ 35 | 2.18(1.82 to 2.61) |
| low_sdi | Deaths | 35 ~ 40 | 3.39(2.92 to 3.94) |
| low_sdi | Deaths | 40 ~ 45 | 6.43(5.74 to 7.21) |
| low_sdi | Deaths | 45 ~ 50 | 15.43(14.22 to 16.74) |
| low_sdi | Deaths | 50 ~ 55 | 33.57(31.57 to 35.7) |
| low_sdi | Deaths | 55 ~ 60 | 67.18(63.95 to 70.57) |
| low_sdi | Deaths | 60 ~ 65 | 146.93(141.19 to 152.91) |
| low_sdi | Deaths | 65 ~ 70 | 263.66(254.32 to 273.34) |
| low_sdi | Deaths | 70 ~ 75 | 467.05(451.29 to 483.37) |
| low_sdi | Deaths | 75 ~ 80 | 681.42(654.39 to 709.57) |
| low_sdi | Deaths | 80 ~ 85 | 1106.98(1060.77 to 1155.2) |
| low_sdi | Deaths | 85 ~ 90 | 1534.12(1460.53 to 1611.42) |
| low_sdi | Deaths | 90 ~ 95 | 2003.78(1872.81 to 2143.9) |
| low_sdi | DALYs | 20 ~ 25 | 120.77(109.28 to 133.46) |
| low_sdi | DALYs | 25 ~ 30 | 164.8(151.16 to 179.67) |
| low_sdi | DALYs | 30 ~ 35 | 222.41(206.06 to 240.05) |
| low_sdi | DALYs | 35 ~ 40 | 292.49(273.2 to 313.14) |
| low_sdi | DALYs | 40 ~ 45 | 449.41(424.2 to 476.13) |
| low_sdi | DALYs | 45 ~ 50 | 885.66(845.85 to 927.35) |
| low_sdi | DALYs | 50 ~ 55 | 1620.68(1560.08 to 1683.63) |
| low_sdi | DALYs | 55 ~ 60 | 2711.83(2623.32 to 2803.33) |
| low_sdi | DALYs | 60 ~ 65 | 4860.73(4721.44 to 5004.13) |
| low_sdi | DALYs | 65 ~ 70 | 7306.07(7107.15 to 7510.57) |
| low_sdi | DALYs | 70 ~ 75 | 10568.38(10281.68 to 10863.08) |
| low_sdi | DALYs | 75 ~ 80 | 12426.07(12016.13 to 12849.99) |
| low_sdi | DALYs | 80 ~ 85 | 15725.38(15139.25 to 16334.2) |
| low_sdi | DALYs | 85 ~ 90 | 17395.37(16547.32 to 18286.89) |
| low_sdi | DALYs | 90 ~ 95 | 19639.25(18119.03 to 21287.01) |

**Table S5 Period effects on COPD related Prevalence, Deaths and DALYs in WCBA across SDI quintiles**

| Region | measure | Period | Period effect rate ratio （95%UI） |
| --- | --- | --- | --- |
| global | Prevalence | 1990 ~ 1995 | 1.02(1.01 to 1.03) |
| global | Prevalence | 1995 ~ 2000 | 1.01(1.01 to 1.02) |
| global | Prevalence | 2000 ~ 2005 | 1.01(1 to 1.01) |
| global | Prevalence | 2005 ~ 2010 | 1(1 to 1) |
| global | Prevalence | 2010 ~ 2015 | 0.99(0.98 to 0.99) |
| global | Prevalence | 2015 ~ 2020 | 0.98(0.97 to 0.98) |
| global | Prevalence | 2020 ~ 2025 | 0.97(0.97 to 0.98) |
| global | Deaths | 1990 ~ 1995 | 1.5(1.47 to 1.52) |
| global | Deaths | 1995 ~ 2000 | 1.34(1.32 to 1.36) |
| global | Deaths | 2000 ~ 2005 | 1.17(1.16 to 1.19) |
| global | Deaths | 2005 ~ 2010 | 1(1 to 1) |
| global | Deaths | 2010 ~ 2015 | 0.9(0.89 to 0.91) |
| global | Deaths | 2015 ~ 2020 | 0.83(0.82 to 0.84) |
| global | Deaths | 2020 ~ 2025 | 0.81(0.8 to 0.82) |
| global | DALYs | 1990 ~ 1995 | 1.36(1.34 to 1.38) |
| global | DALYs | 1995 ~ 2000 | 1.26(1.24 to 1.27) |
| global | DALYs | 2000 ~ 2005 | 1.13(1.12 to 1.15) |
| global | DALYs | 2005 ~ 2010 | 1(1 to 1) |
| global | DALYs | 2010 ~ 2015 | 0.92(0.91 to 0.93) |
| global | DALYs | 2015 ~ 2020 | 0.87(0.86 to 0.88) |
| global | DALYs | 2020 ~ 2025 | 0.85(0.84 to 0.86) |
| high_sdi | Prevalence | 1990 ~ 1995 | 1(1 to 1.01) |
| high_sdi | Prevalence | 1995 ~ 2000 | 0.99(0.99 to 1) |
| high_sdi | Prevalence | 2000 ~ 2005 | 1.01(1 to 1.01) |
| high_sdi | Prevalence | 2005 ~ 2010 | 1(1 to 1) |
| high_sdi | Prevalence | 2010 ~ 2015 | 0.98(0.97 to 0.98) |
| high_sdi | Prevalence | 2015 ~ 2020 | 0.96(0.95 to 0.96) |
| high_sdi | Prevalence | 2020 ~ 2025 | 0.94(0.94 to 0.95) |
| high_sdi | Deaths | 1990 ~ 1995 | 1.19(1.15 to 1.23) |
| high_sdi | Deaths | 1995 ~ 2000 | 1.13(1.1 to 1.15) |
| high_sdi | Deaths | 2000 ~ 2005 | 1.08(1.06 to 1.09) |
| high_sdi | Deaths | 2005 ~ 2010 | 1(1 to 1) |
| high_sdi | Deaths | 2010 ~ 2015 | 0.97(0.95 to 0.98) |
| high_sdi | Deaths | 2015 ~ 2020 | 0.96(0.94 to 0.98) |
| high_sdi | Deaths | 2020 ~ 2025 | 0.93(0.91 to 0.96) |
| high_sdi | DALYs | 1990 ~ 1995 | 1.11(1.09 to 1.13) |
| high_sdi | DALYs | 1995 ~ 2000 | 1.08(1.06 to 1.09) |
| high_sdi | DALYs | 2000 ~ 2005 | 1.05(1.04 to 1.07) |
| high_sdi | DALYs | 2005 ~ 2010 | 1(1 to 1) |
| high_sdi | DALYs | 2010 ~ 2015 | 0.97(0.96 to 0.98) |
| high_sdi | DALYs | 2015 ~ 2020 | 0.95(0.94 to 0.97) |
| high_sdi | DALYs | 2020 ~ 2025 | 0.92(0.91 to 0.94) |
| high-middle_sdi | Prevalence | 1990 ~ 1995 | 1.06(1.05 to 1.07) |
| high-middle_sdi | Prevalence | 1995 ~ 2000 | 1.04(1.03 to 1.05) |
| high-middle_sdi | Prevalence | 2000 ~ 2005 | 1.02(1.01 to 1.03) |
| high-middle_sdi | Prevalence | 2005 ~ 2010 | 1(1 to 1) |
| high-middle_sdi | Prevalence | 2010 ~ 2015 | 0.98(0.97 to 0.99) |
| high-middle_sdi | Prevalence | 2015 ~ 2020 | 0.96(0.95 to 0.97) |
| high-middle_sdi | Prevalence | 2020 ~ 2025 | 0.96(0.95 to 0.97) |
| high-middle_sdi | Deaths | 1990 ~ 1995 | 1.9(1.85 to 1.94) |
| high-middle_sdi | Deaths | 1995 ~ 2000 | 1.6(1.57 to 1.63) |
| high-middle_sdi | Deaths | 2000 ~ 2005 | 1.34(1.32 to 1.36) |
| high-middle_sdi | Deaths | 2005 ~ 2010 | 1(1 to 1) |
| high-middle_sdi | Deaths | 2010 ~ 2015 | 0.77(0.76 to 0.78) |
| high-middle_sdi | Deaths | 2015 ~ 2020 | 0.66(0.65 to 0.68) |
| high-middle_sdi | Deaths | 2020 ~ 2025 | 0.65(0.64 to 0.67) |
| high-middle_sdi | DALYs | 1990 ~ 1995 | 1.63(1.6 to 1.66) |
| high-middle_sdi | DALYs | 1995 ~ 2000 | 1.45(1.42 to 1.47) |
| high-middle_sdi | DALYs | 2000 ~ 2005 | 1.26(1.24 to 1.28) |
| high-middle_sdi | DALYs | 2005 ~ 2010 | 1(1 to 1) |
| high-middle_sdi | DALYs | 2010 ~ 2015 | 0.82(0.81 to 0.83) |
| high-middle_sdi | DALYs | 2015 ~ 2020 | 0.74(0.72 to 0.75) |
| high-middle_sdi | DALYs | 2020 ~ 2025 | 0.73(0.72 to 0.74) |
| middle_sdi | Prevalence | 1990 ~ 1995 | 1.04(1.03 to 1.05) |
| middle_sdi | Prevalence | 1995 ~ 2000 | 1.03(1.02 to 1.03) |
| middle_sdi | Prevalence | 2000 ~ 2005 | 1.01(1 to 1.02) |
| middle_sdi | Prevalence | 2005 ~ 2010 | 1(1 to 1) |
| middle_sdi | Prevalence | 2010 ~ 2015 | 0.99(0.99 to 1) |
| middle_sdi | Prevalence | 2015 ~ 2020 | 0.98(0.98 to 0.99) |
| middle_sdi | Prevalence | 2020 ~ 2025 | 0.98(0.98 to 0.99) |
| middle_sdi | Deaths | 1990 ~ 1995 | 1.87(1.83 to 1.91) |
| middle_sdi | Deaths | 1995 ~ 2000 | 1.57(1.54 to 1.59) |
| middle_sdi | Deaths | 2000 ~ 2005 | 1.28(1.27 to 1.3) |
| middle_sdi | Deaths | 2005 ~ 2010 | 1(1 to 1) |
| middle_sdi | Deaths | 2010 ~ 2015 | 0.85(0.83 to 0.86) |
| middle_sdi | Deaths | 2015 ~ 2020 | 0.74(0.73 to 0.75) |
| middle_sdi | Deaths | 2020 ~ 2025 | 0.71(0.7 to 0.72) |
| middle_sdi | DALYs | 1990 ~ 1995 | 1.65(1.62 to 1.67) |
| middle_sdi | DALYs | 1995 ~ 2000 | 1.44(1.41 to 1.46) |
| middle_sdi | DALYs | 2000 ~ 2005 | 1.22(1.2 to 1.24) |
| middle_sdi | DALYs | 2005 ~ 2010 | 1(1 to 1) |
| middle_sdi | DALYs | 2010 ~ 2015 | 0.87(0.86 to 0.88) |
| middle_sdi | DALYs | 2015 ~ 2020 | 0.78(0.77 to 0.8) |
| middle_sdi | DALYs | 2020 ~ 2025 | 0.76(0.75 to 0.78) |
| low-middle_sdi | Prevalence | 1990 ~ 1995 | 1(1 to 1) |
| low-middle_sdi | Prevalence | 1995 ~ 2000 | 1(0.99 to 1) |
| low-middle_sdi | Prevalence | 2000 ~ 2005 | 1(1 to 1) |
| low-middle_sdi | Prevalence | 2005 ~ 2010 | 1(1 to 1) |
| low-middle_sdi | Prevalence | 2010 ~ 2015 | 1(1 to 1) |
| low-middle_sdi | Prevalence | 2015 ~ 2020 | 1(0.99 to 1) |
| low-middle_sdi | Prevalence | 2020 ~ 2025 | 1(1 to 1) |
| low-middle_sdi | Deaths | 1990 ~ 1995 | 1.22(1.18 to 1.26) |
| low-middle_sdi | Deaths | 1995 ~ 2000 | 1.14(1.11 to 1.18) |
| low-middle_sdi | Deaths | 2000 ~ 2005 | 1.03(1 to 1.05) |
| low-middle_sdi | Deaths | 2005 ~ 2010 | 1(1 to 1) |
| low-middle_sdi | Deaths | 2010 ~ 2015 | 0.97(0.95 to 0.99) |
| low-middle_sdi | Deaths | 2015 ~ 2020 | 0.94(0.91 to 0.96) |
| low-middle_sdi | Deaths | 2020 ~ 2025 | 0.9(0.88 to 0.93) |
| low-middle_sdi | DALYs | 1990 ~ 1995 | 1.15(1.12 to 1.18) |
| low-middle_sdi | DALYs | 1995 ~ 2000 | 1.1(1.08 to 1.13) |
| low-middle_sdi | DALYs | 2000 ~ 2005 | 1.02(1 to 1.05) |
| low-middle_sdi | DALYs | 2005 ~ 2010 | 1(1 to 1) |
| low-middle_sdi | DALYs | 2010 ~ 2015 | 0.97(0.95 to 1) |
| low-middle_sdi | DALYs | 2015 ~ 2020 | 0.95(0.93 to 0.97) |
| low-middle_sdi | DALYs | 2020 ~ 2025 | 0.93(0.91 to 0.95) |
| low_sdi | Prevalence | 1990 ~ 1995 | 0.99(0.98 to 1) |
| low_sdi | Prevalence | 1995 ~ 2000 | 0.99(0.98 to 1) |
| low_sdi | Prevalence | 2000 ~ 2005 | 0.99(0.99 to 1) |
| low_sdi | Prevalence | 2005 ~ 2010 | 1(1 to 1) |
| low_sdi | Prevalence | 2010 ~ 2015 | 1(1 to 1.01) |
| low_sdi | Prevalence | 2015 ~ 2020 | 1.01(1 to 1.01) |
| low_sdi | Prevalence | 2020 ~ 2025 | 1.02(1.01 to 1.03) |
| low_sdi | Deaths | 1990 ~ 1995 | 1.29(1.23 to 1.35) |
| low_sdi | Deaths | 1995 ~ 2000 | 1.19(1.15 to 1.24) |
| low_sdi | Deaths | 2000 ~ 2005 | 1.08(1.04 to 1.12) |
| low_sdi | Deaths | 2005 ~ 2010 | 1(1 to 1) |
| low_sdi | Deaths | 2010 ~ 2015 | 1(0.97 to 1.04) |
| low_sdi | Deaths | 2015 ~ 2020 | 0.99(0.95 to 1.02) |
| low_sdi | Deaths | 2020 ~ 2025 | 0.93(0.9 to 0.97) |
| low_sdi | DALYs | 1990 ~ 1995 | 1.19(1.15 to 1.23) |
| low_sdi | DALYs | 1995 ~ 2000 | 1.14(1.11 to 1.18) |
| low_sdi | DALYs | 2000 ~ 2005 | 1.06(1.03 to 1.09) |
| low_sdi | DALYs | 2005 ~ 2010 | 1(1 to 1) |
| low_sdi | DALYs | 2010 ~ 2015 | 0.99(0.97 to 1.02) |
| low_sdi | DALYs | 2015 ~ 2020 | 0.98(0.95 to 1) |
| low_sdi | DALYs | 2020 ~ 2025 | 0.95(0.93 to 0.98) |

**Table S6 Birth cohort effects on COPD related Prevalence, Deaths and DALYs in WCBA across SDI quintiles**

| **Region** | **measure** | **Cohort** | **Cohort effect rate ratio（95%UI）** |
| --- | --- | --- | --- |
| global | Prevalence | 1900 | 0.9(0.87 to 0.93) |
| global | Prevalence | 1905 | 0.93(0.91 to 0.95) |
| global | Prevalence | 1910 | 0.96(0.95 to 0.97) |
| global | Prevalence | 1915 | 0.99(0.98 to 0.99) |
| global | Prevalence | 1920 | 1.01(1 to 1.01) |
| global | Prevalence | 1925 | 1.01(1 to 1.02) |
| global | Prevalence | 1930 | 1.01(1.01 to 1.02) |
| global | Prevalence | 1935 | 1.02(1.01 to 1.02) |
| global | Prevalence | 1940 | 1.02(1.01 to 1.02) |
| global | Prevalence | 1945 | 1.02(1.01 to 1.02) |
| global | Prevalence | 1950 | 1(1 to 1) |
| global | Prevalence | 1955 | 0.98(0.98 to 0.99) |
| global | Prevalence | 1960 | 0.96(0.96 to 0.97) |
| global | Prevalence | 1965 | 0.94(0.93 to 0.95) |
| global | Prevalence | 1970 | 0.92(0.91 to 0.93) |
| global | Prevalence | 1975 | 0.91(0.9 to 0.92) |
| global | Prevalence | 1980 | 0.9(0.89 to 0.91) |
| global | Prevalence | 1985 | 0.88(0.87 to 0.89) |
| global | Prevalence | 1990 | 0.86(0.85 to 0.88) |
| global | Prevalence | 1995 | 0.85(0.83 to 0.87) |
| global | Prevalence | 2000 | 0.85(0.82 to 0.87) |
| global | Deaths | 1900 | 1.9(1.81 to 1.99) |
| global | Deaths | 1905 | 1.97(1.92 to 2.02) |
| global | Deaths | 1910 | 1.96(1.93 to 2) |
| global | Deaths | 1915 | 2.04(2 to 2.07) |
| global | Deaths | 1920 | 1.92(1.89 to 1.95) |
| global | Deaths | 1925 | 1.73(1.71 to 1.76) |
| global | Deaths | 1930 | 1.61(1.58 to 1.63) |
| global | Deaths | 1935 | 1.47(1.45 to 1.49) |
| global | Deaths | 1940 | 1.3(1.28 to 1.31) |
| global | Deaths | 1945 | 1.16(1.15 to 1.18) |
| global | Deaths | 1950 | 1(1 to 1) |
| global | Deaths | 1955 | 0.88(0.87 to 0.9) |
| global | Deaths | 1960 | 0.78(0.76 to 0.8) |
| global | Deaths | 1965 | 0.71(0.69 to 0.73) |
| global | Deaths | 1970 | 0.61(0.59 to 0.64) |
| global | Deaths | 1975 | 0.54(0.52 to 0.58) |
| global | Deaths | 1980 | 0.48(0.44 to 0.52) |
| global | Deaths | 1985 | 0.42(0.38 to 0.46) |
| global | Deaths | 1990 | 0.37(0.33 to 0.43) |
| global | Deaths | 1995 | 0.34(0.28 to 0.41) |
| global | Deaths | 2000 | 0.3(0.22 to 0.4) |
| global | DALYs | 1900 | 1.72(1.61 to 1.85) |
| global | DALYs | 1905 | 1.79(1.73 to 1.86) |
| global | DALYs | 1910 | 1.79(1.75 to 1.83) |
| global | DALYs | 1915 | 1.85(1.82 to 1.89) |
| global | DALYs | 1920 | 1.76(1.73 to 1.79) |
| global | DALYs | 1925 | 1.61(1.58 to 1.63) |
| global | DALYs | 1930 | 1.5(1.48 to 1.52) |
| global | DALYs | 1935 | 1.39(1.37 to 1.41) |
| global | DALYs | 1940 | 1.25(1.23 to 1.26) |
| global | DALYs | 1945 | 1.14(1.12 to 1.15) |
| global | DALYs | 1950 | 1(1 to 1) |
| global | DALYs | 1955 | 0.91(0.89 to 0.92) |
| global | DALYs | 1960 | 0.82(0.81 to 0.83) |
| global | DALYs | 1965 | 0.76(0.75 to 0.78) |
| global | DALYs | 1970 | 0.69(0.67 to 0.71) |
| global | DALYs | 1975 | 0.65(0.62 to 0.67) |
| global | DALYs | 1980 | 0.6(0.58 to 0.63) |
| global | DALYs | 1985 | 0.57(0.54 to 0.59) |
| global | DALYs | 1990 | 0.53(0.5 to 0.56) |
| global | DALYs | 1995 | 0.5(0.47 to 0.55) |
| global | DALYs | 2000 | 0.47(0.42 to 0.53) |
| high_sdi | Prevalence | 1900 | 0.94(0.91 to 0.96) |
| high_sdi | Prevalence | 1905 | 0.96(0.95 to 0.98) |
| high_sdi | Prevalence | 1910 | 0.98(0.97 to 0.99) |
| high_sdi | Prevalence | 1915 | 1(0.99 to 1.01) |
| high_sdi | Prevalence | 1920 | 1.01(1.01 to 1.02) |
| high_sdi | Prevalence | 1925 | 1.01(1 to 1.01) |
| high_sdi | Prevalence | 1930 | 1(0.99 to 1) |
| high_sdi | Prevalence | 1935 | 0.99(0.99 to 1) |
| high_sdi | Prevalence | 1940 | 1(0.99 to 1) |
| high_sdi | Prevalence | 1945 | 1.01(1 to 1.02) |
| high_sdi | Prevalence | 1950 | 1(1 to 1) |
| high_sdi | Prevalence | 1955 | 1.01(1 to 1.01) |
| high_sdi | Prevalence | 1960 | 1(0.99 to 1.01) |
| high_sdi | Prevalence | 1965 | 0.97(0.96 to 0.98) |
| high_sdi | Prevalence | 1970 | 0.93(0.92 to 0.95) |
| high_sdi | Prevalence | 1975 | 0.9(0.89 to 0.91) |
| high_sdi | Prevalence | 1980 | 0.88(0.86 to 0.89) |
| high_sdi | Prevalence | 1985 | 0.85(0.83 to 0.87) |
| high_sdi | Prevalence | 1990 | 0.82(0.8 to 0.85) |
| high_sdi | Prevalence | 1995 | 0.79(0.76 to 0.82) |
| high_sdi | Prevalence | 2000 | 0.78(0.73 to 0.82) |
| high_sdi | Deaths | 1900 | 1.33(1.27 to 1.4) |
| high_sdi | Deaths | 1905 | 1.42(1.38 to 1.46) |
| high_sdi | Deaths | 1910 | 1.49(1.45 to 1.52) |
| high_sdi | Deaths | 1915 | 1.57(1.53 to 1.6) |
| high_sdi | Deaths | 1920 | 1.52(1.49 to 1.55) |
| high_sdi | Deaths | 1925 | 1.45(1.42 to 1.48) |
| high_sdi | Deaths | 1930 | 1.34(1.32 to 1.37) |
| high_sdi | Deaths | 1935 | 1.2(1.17 to 1.22) |
| high_sdi | Deaths | 1940 | 1.09(1.07 to 1.11) |
| high_sdi | Deaths | 1945 | 1.06(1.04 to 1.08) |
| high_sdi | Deaths | 1950 | 1(1 to 1) |
| high_sdi | Deaths | 1955 | 1.02(1 to 1.05) |
| high_sdi | Deaths | 1960 | 1.04(1 to 1.07) |
| high_sdi | Deaths | 1965 | 0.99(0.94 to 1.03) |
| high_sdi | Deaths | 1970 | 0.88(0.82 to 0.95) |
| high_sdi | Deaths | 1975 | 0.82(0.74 to 0.92) |
| high_sdi | Deaths | 1980 | 0.83(0.7 to 0.98) |
| high_sdi | Deaths | 1985 | 0.87(0.69 to 1.09) |
| high_sdi | Deaths | 1990 | 0.85(0.62 to 1.17) |
| high_sdi | Deaths | 1995 | 0.79(0.49 to 1.26) |
| high_sdi | Deaths | 2000 | 0.72(0.34 to 1.54) |
| high_sdi | DALYs | 1900 | 1.22(1.14 to 1.3) |
| high_sdi | DALYs | 1905 | 1.28(1.24 to 1.33) |
| high_sdi | DALYs | 1910 | 1.33(1.3 to 1.37) |
| high_sdi | DALYs | 1915 | 1.4(1.37 to 1.43) |
| high_sdi | DALYs | 1920 | 1.37(1.34 to 1.4) |
| high_sdi | DALYs | 1925 | 1.31(1.29 to 1.33) |
| high_sdi | DALYs | 1930 | 1.23(1.21 to 1.25) |
| high_sdi | DALYs | 1935 | 1.12(1.1 to 1.14) |
| high_sdi | DALYs | 1940 | 1.05(1.03 to 1.07) |
| high_sdi | DALYs | 1945 | 1.04(1.02 to 1.05) |
| high_sdi | DALYs | 1950 | 1(1 to 1) |
| high_sdi | DALYs | 1955 | 1.03(1.01 to 1.05) |
| high_sdi | DALYs | 1960 | 1.03(1.01 to 1.06) |
| high_sdi | DALYs | 1965 | 0.99(0.96 to 1.02) |
| high_sdi | DALYs | 1970 | 0.92(0.88 to 0.95) |
| high_sdi | DALYs | 1975 | 0.88(0.83 to 0.92) |
| high_sdi | DALYs | 1980 | 0.87(0.81 to 0.93) |
| high_sdi | DALYs | 1985 | 0.87(0.8 to 0.94) |
| high_sdi | DALYs | 1990 | 0.83(0.75 to 0.93) |
| high_sdi | DALYs | 1995 | 0.78(0.68 to 0.9) |
| high_sdi | DALYs | 2000 | 0.74(0.59 to 0.93) |
| high-middle_sdi | Prevalence | 1900 | 0.93(0.86 to 1.01) |
| high-middle_sdi | Prevalence | 1905 | 0.97(0.93 to 1) |
| high-middle_sdi | Prevalence | 1910 | 1.01(0.98 to 1.03) |
| high-middle_sdi | Prevalence | 1915 | 1.03(1.01 to 1.05) |
| high-middle_sdi | Prevalence | 1920 | 1.06(1.04 to 1.08) |
| high-middle_sdi | Prevalence | 1925 | 1.06(1.04 to 1.08) |
| high-middle_sdi | Prevalence | 1930 | 1.06(1.05 to 1.08) |
| high-middle_sdi | Prevalence | 1935 | 1.06(1.05 to 1.08) |
| high-middle_sdi | Prevalence | 1940 | 1.04(1.03 to 1.06) |
| high-middle_sdi | Prevalence | 1945 | 1.03(1.02 to 1.05) |
| high-middle_sdi | Prevalence | 1950 | 1(1 to 1) |
| high-middle_sdi | Prevalence | 1955 | 0.97(0.96 to 0.98) |
| high-middle_sdi | Prevalence | 1960 | 0.94(0.92 to 0.95) |
| high-middle_sdi | Prevalence | 1965 | 0.9(0.89 to 0.92) |
| high-middle_sdi | Prevalence | 1970 | 0.87(0.86 to 0.89) |
| high-middle_sdi | Prevalence | 1975 | 0.86(0.84 to 0.88) |
| high-middle_sdi | Prevalence | 1980 | 0.85(0.82 to 0.87) |
| high-middle_sdi | Prevalence | 1985 | 0.82(0.79 to 0.85) |
| high-middle_sdi | Prevalence | 1990 | 0.79(0.76 to 0.82) |
| high-middle_sdi | Prevalence | 1995 | 0.77(0.73 to 0.82) |
| high-middle_sdi | Prevalence | 2000 | 0.76(0.7 to 0.83) |
| high-middle_sdi | Deaths | 1900 | 3.77(3.58 to 3.97) |
| high-middle_sdi | Deaths | 1905 | 3.67(3.56 to 3.78) |
| high-middle_sdi | Deaths | 1910 | 3.55(3.46 to 3.64) |
| high-middle_sdi | Deaths | 1915 | 3.6(3.52 to 3.68) |
| high-middle_sdi | Deaths | 1920 | 3.28(3.21 to 3.35) |
| high-middle_sdi | Deaths | 1925 | 2.64(2.59 to 2.7) |
| high-middle_sdi | Deaths | 1930 | 2.26(2.22 to 2.31) |
| high-middle_sdi | Deaths | 1935 | 1.92(1.88 to 1.96) |
| high-middle_sdi | Deaths | 1940 | 1.53(1.5 to 1.56) |
| high-middle_sdi | Deaths | 1945 | 1.26(1.23 to 1.29) |
| high-middle_sdi | Deaths | 1950 | 1(1 to 1) |
| high-middle_sdi | Deaths | 1955 | 0.8(0.78 to 0.82) |
| high-middle_sdi | Deaths | 1960 | 0.62(0.6 to 0.64) |
| high-middle_sdi | Deaths | 1965 | 0.5(0.48 to 0.52) |
| high-middle_sdi | Deaths | 1970 | 0.4(0.37 to 0.42) |
| high-middle_sdi | Deaths | 1975 | 0.32(0.29 to 0.35) |
| high-middle_sdi | Deaths | 1980 | 0.27(0.24 to 0.31) |
| high-middle_sdi | Deaths | 1985 | 0.23(0.19 to 0.27) |
| high-middle_sdi | Deaths | 1990 | 0.19(0.15 to 0.24) |
| high-middle_sdi | Deaths | 1995 | 0.16(0.11 to 0.23) |
| high-middle_sdi | Deaths | 2000 | 0.13(0.07 to 0.25) |
| high-middle_sdi | DALYs | 1900 | 3.15(2.9 to 3.42) |
| high-middle_sdi | DALYs | 1905 | 3.08(2.96 to 3.21) |
| high-middle_sdi | DALYs | 1910 | 3(2.91 to 3.09) |
| high-middle_sdi | DALYs | 1915 | 3.03(2.95 to 3.1) |
| high-middle_sdi | DALYs | 1920 | 2.8(2.73 to 2.86) |
| high-middle_sdi | DALYs | 1925 | 2.29(2.24 to 2.34) |
| high-middle_sdi | DALYs | 1930 | 1.99(1.95 to 2.03) |
| high-middle_sdi | DALYs | 1935 | 1.72(1.69 to 1.76) |
| high-middle_sdi | DALYs | 1940 | 1.41(1.38 to 1.44) |
| high-middle_sdi | DALYs | 1945 | 1.21(1.18 to 1.23) |
| high-middle_sdi | DALYs | 1950 | 1(1 to 1) |
| high-middle_sdi | DALYs | 1955 | 0.84(0.82 to 0.86) |
| high-middle_sdi | DALYs | 1960 | 0.69(0.67 to 0.71) |
| high-middle_sdi | DALYs | 1965 | 0.6(0.58 to 0.62) |
| high-middle_sdi | DALYs | 1970 | 0.51(0.49 to 0.53) |
| high-middle_sdi | DALYs | 1975 | 0.45(0.42 to 0.47) |
| high-middle_sdi | DALYs | 1980 | 0.4(0.38 to 0.43) |
| high-middle_sdi | DALYs | 1985 | 0.38(0.35 to 0.41) |
| high-middle_sdi | DALYs | 1990 | 0.34(0.31 to 0.38) |
| high-middle_sdi | DALYs | 1995 | 0.31(0.26 to 0.36) |
| high-middle_sdi | DALYs | 2000 | 0.27(0.21 to 0.34) |
| middle_sdi | Prevalence | 1900 | 0.8(0.73 to 0.86) |
| middle_sdi | Prevalence | 1905 | 0.86(0.83 to 0.89) |
| middle_sdi | Prevalence | 1910 | 0.91(0.89 to 0.93) |
| middle_sdi | Prevalence | 1915 | 0.95(0.94 to 0.97) |
| middle_sdi | Prevalence | 1920 | 0.98(0.97 to 1) |
| middle_sdi | Prevalence | 1925 | 1.01(1 to 1.02) |
| middle_sdi | Prevalence | 1930 | 1.02(1.01 to 1.03) |
| middle_sdi | Prevalence | 1935 | 1.03(1.02 to 1.04) |
| middle_sdi | Prevalence | 1940 | 1.03(1.02 to 1.04) |
| middle_sdi | Prevalence | 1945 | 1.02(1.01 to 1.03) |
| middle_sdi | Prevalence | 1950 | 1(1 to 1) |
| middle_sdi | Prevalence | 1955 | 0.97(0.97 to 0.98) |
| middle_sdi | Prevalence | 1960 | 0.94(0.93 to 0.95) |
| middle_sdi | Prevalence | 1965 | 0.92(0.91 to 0.94) |
| middle_sdi | Prevalence | 1970 | 0.9(0.89 to 0.91) |
| middle_sdi | Prevalence | 1975 | 0.88(0.87 to 0.9) |
| middle_sdi | Prevalence | 1980 | 0.87(0.85 to 0.88) |
| middle_sdi | Prevalence | 1985 | 0.85(0.83 to 0.86) |
| middle_sdi | Prevalence | 1990 | 0.82(0.8 to 0.84) |
| middle_sdi | Prevalence | 1995 | 0.8(0.78 to 0.83) |
| middle_sdi | Prevalence | 2000 | 0.79(0.75 to 0.83) |
| middle_sdi | Deaths | 1900 | 3.27(3.08 to 3.47) |
| middle_sdi | Deaths | 1905 | 3.23(3.13 to 3.34) |
| middle_sdi | Deaths | 1910 | 3.19(3.12 to 3.27) |
| middle_sdi | Deaths | 1915 | 3.1(3.04 to 3.16) |
| middle_sdi | Deaths | 1920 | 2.76(2.71 to 2.81) |
| middle_sdi | Deaths | 1925 | 2.41(2.36 to 2.45) |
| middle_sdi | Deaths | 1930 | 2.09(2.05 to 2.13) |
| middle_sdi | Deaths | 1935 | 1.78(1.75 to 1.82) |
| middle_sdi | Deaths | 1940 | 1.49(1.46 to 1.52) |
| middle_sdi | Deaths | 1945 | 1.23(1.2 to 1.25) |
| middle_sdi | Deaths | 1950 | 1(1 to 1) |
| middle_sdi | Deaths | 1955 | 0.82(0.8 to 0.84) |
| middle_sdi | Deaths | 1960 | 0.66(0.64 to 0.68) |
| middle_sdi | Deaths | 1965 | 0.56(0.54 to 0.58) |
| middle_sdi | Deaths | 1970 | 0.47(0.44 to 0.49) |
| middle_sdi | Deaths | 1975 | 0.39(0.36 to 0.42) |
| middle_sdi | Deaths | 1980 | 0.32(0.29 to 0.35) |
| middle_sdi | Deaths | 1985 | 0.27(0.24 to 0.31) |
| middle_sdi | Deaths | 1990 | 0.23(0.19 to 0.27) |
| middle_sdi | Deaths | 1995 | 0.2(0.16 to 0.26) |
| middle_sdi | Deaths | 2000 | 0.17(0.12 to 0.25) |
| middle_sdi | DALYs | 1900 | 2.88(2.62 to 3.17) |
| middle_sdi | DALYs | 1905 | 2.87(2.74 to 3) |
| middle_sdi | DALYs | 1910 | 2.83(2.75 to 2.92) |
| middle_sdi | DALYs | 1915 | 2.76(2.69 to 2.83) |
| middle_sdi | DALYs | 1920 | 2.48(2.43 to 2.53) |
| middle_sdi | DALYs | 1925 | 2.18(2.14 to 2.22) |
| middle_sdi | DALYs | 1930 | 1.91(1.88 to 1.95) |
| middle_sdi | DALYs | 1935 | 1.66(1.63 to 1.69) |
| middle_sdi | DALYs | 1940 | 1.41(1.39 to 1.44) |
| middle_sdi | DALYs | 1945 | 1.19(1.17 to 1.21) |
| middle_sdi | DALYs | 1950 | 1(1 to 1) |
| middle_sdi | DALYs | 1955 | 0.85(0.83 to 0.86) |
| middle_sdi | DALYs | 1960 | 0.71(0.69 to 0.72) |
| middle_sdi | DALYs | 1965 | 0.63(0.62 to 0.65) |
| middle_sdi | DALYs | 1970 | 0.56(0.54 to 0.57) |
| middle_sdi | DALYs | 1975 | 0.49(0.47 to 0.51) |
| middle_sdi | DALYs | 1980 | 0.44(0.41 to 0.46) |
| middle_sdi | DALYs | 1985 | 0.4(0.38 to 0.43) |
| middle_sdi | DALYs | 1990 | 0.37(0.34 to 0.4) |
| middle_sdi | DALYs | 1995 | 0.34(0.3 to 0.38) |
| middle_sdi | DALYs | 2000 | 0.3(0.26 to 0.35) |
| low-middle_sdi | Prevalence | 1900 | 0.82(0.8 to 0.84) |
| low-middle_sdi | Prevalence | 1905 | 0.86(0.85 to 0.87) |
| low-middle_sdi | Prevalence | 1910 | 0.89(0.88 to 0.9) |
| low-middle_sdi | Prevalence | 1915 | 0.92(0.91 to 0.92) |
| low-middle_sdi | Prevalence | 1920 | 0.94(0.94 to 0.95) |
| low-middle_sdi | Prevalence | 1925 | 0.96(0.96 to 0.97) |
| low-middle_sdi | Prevalence | 1930 | 0.99(0.98 to 0.99) |
| low-middle_sdi | Prevalence | 1935 | 1(1 to 1.01) |
| low-middle_sdi | Prevalence | 1940 | 1.01(1 to 1.01) |
| low-middle_sdi | Prevalence | 1945 | 1.01(1 to 1.01) |
| low-middle_sdi | Prevalence | 1950 | 1(1 to 1) |
| low-middle_sdi | Prevalence | 1955 | 0.99(0.99 to 0.99) |
| low-middle_sdi | Prevalence | 1960 | 0.98(0.98 to 0.98) |
| low-middle_sdi | Prevalence | 1965 | 0.97(0.97 to 0.97) |
| low-middle_sdi | Prevalence | 1970 | 0.96(0.95 to 0.96) |
| low-middle_sdi | Prevalence | 1975 | 0.95(0.94 to 0.95) |
| low-middle_sdi | Prevalence | 1980 | 0.94(0.93 to 0.94) |
| low-middle_sdi | Prevalence | 1985 | 0.93(0.92 to 0.93) |
| low-middle_sdi | Prevalence | 1990 | 0.92(0.91 to 0.92) |
| low-middle_sdi | Prevalence | 1995 | 0.91(0.9 to 0.92) |
| low-middle_sdi | Prevalence | 2000 | 0.9(0.89 to 0.91) |
| low-middle_sdi | Deaths | 1900 | 0.92(0.8 to 1.06) |
| low-middle_sdi | Deaths | 1905 | 1(0.93 to 1.07) |
| low-middle_sdi | Deaths | 1910 | 1.04(0.99 to 1.09) |
| low-middle_sdi | Deaths | 1915 | 1.1(1.05 to 1.14) |
| low-middle_sdi | Deaths | 1920 | 1.15(1.11 to 1.19) |
| low-middle_sdi | Deaths | 1925 | 1.2(1.16 to 1.24) |
| low-middle_sdi | Deaths | 1930 | 1.2(1.16 to 1.24) |
| low-middle_sdi | Deaths | 1935 | 1.18(1.14 to 1.21) |
| low-middle_sdi | Deaths | 1940 | 1.1(1.07 to 1.14) |
| low-middle_sdi | Deaths | 1945 | 1.06(1.03 to 1.09) |
| low-middle_sdi | Deaths | 1950 | 1(1 to 1) |
| low-middle_sdi | Deaths | 1955 | 0.92(0.89 to 0.96) |
| low-middle_sdi | Deaths | 1960 | 0.87(0.83 to 0.91) |
| low-middle_sdi | Deaths | 1965 | 0.85(0.8 to 0.9) |
| low-middle_sdi | Deaths | 1970 | 0.76(0.7 to 0.82) |
| low-middle_sdi | Deaths | 1975 | 0.71(0.63 to 0.79) |
| low-middle_sdi | Deaths | 1980 | 0.65(0.56 to 0.76) |
| low-middle_sdi | Deaths | 1985 | 0.57(0.46 to 0.69) |
| low-middle_sdi | Deaths | 1990 | 0.51(0.39 to 0.67) |
| low-middle_sdi | Deaths | 1995 | 0.45(0.31 to 0.66) |
| low-middle_sdi | Deaths | 2000 | 0.42(0.23 to 0.74) |
| low-middle_sdi | DALYs | 1900 | 0.93(0.76 to 1.13) |
| low-middle_sdi | DALYs | 1905 | 0.99(0.91 to 1.09) |
| low-middle_sdi | DALYs | 1910 | 1.03(0.98 to 1.09) |
| low-middle_sdi | DALYs | 1915 | 1.09(1.04 to 1.13) |
| low-middle_sdi | DALYs | 1920 | 1.13(1.09 to 1.17) |
| low-middle_sdi | DALYs | 1925 | 1.18(1.14 to 1.21) |
| low-middle_sdi | DALYs | 1930 | 1.18(1.15 to 1.21) |
| low-middle_sdi | DALYs | 1935 | 1.16(1.13 to 1.19) |
| low-middle_sdi | DALYs | 1940 | 1.1(1.07 to 1.13) |
| low-middle_sdi | DALYs | 1945 | 1.06(1.04 to 1.09) |
| low-middle_sdi | DALYs | 1950 | 1(1 to 1) |
| low-middle_sdi | DALYs | 1955 | 0.94(0.91 to 0.96) |
| low-middle_sdi | DALYs | 1960 | 0.89(0.87 to 0.92) |
| low-middle_sdi | DALYs | 1965 | 0.87(0.84 to 0.9) |
| low-middle_sdi | DALYs | 1970 | 0.8(0.77 to 0.84) |
| low-middle_sdi | DALYs | 1975 | 0.77(0.72 to 0.81) |
| low-middle_sdi | DALYs | 1980 | 0.73(0.68 to 0.79) |
| low-middle_sdi | DALYs | 1985 | 0.68(0.62 to 0.74) |
| low-middle_sdi | DALYs | 1990 | 0.65(0.58 to 0.72) |
| low-middle_sdi | DALYs | 1995 | 0.61(0.53 to 0.7) |
| low-middle_sdi | DALYs | 2000 | 0.58(0.48 to 0.71) |
| low_sdi | Prevalence | 1900 | 0.77(0.71 to 0.84) |
| low_sdi | Prevalence | 1905 | 0.83(0.79 to 0.86) |
| low_sdi | Prevalence | 1910 | 0.86(0.84 to 0.88) |
| low_sdi | Prevalence | 1915 | 0.87(0.85 to 0.88) |
| low_sdi | Prevalence | 1920 | 0.89(0.88 to 0.9) |
| low_sdi | Prevalence | 1925 | 0.91(0.9 to 0.92) |
| low_sdi | Prevalence | 1930 | 0.94(0.93 to 0.95) |
| low_sdi | Prevalence | 1935 | 0.97(0.96 to 0.98) |
| low_sdi | Prevalence | 1940 | 0.98(0.97 to 0.99) |
| low_sdi | Prevalence | 1945 | 0.99(0.99 to 1) |
| low_sdi | Prevalence | 1950 | 1(1 to 1) |
| low_sdi | Prevalence | 1955 | 0.99(0.98 to 1) |
| low_sdi | Prevalence | 1960 | 0.98(0.97 to 0.99) |
| low_sdi | Prevalence | 1965 | 0.97(0.96 to 0.98) |
| low_sdi | Prevalence | 1970 | 0.96(0.95 to 0.97) |
| low_sdi | Prevalence | 1975 | 0.95(0.94 to 0.96) |
| low_sdi | Prevalence | 1980 | 0.94(0.93 to 0.95) |
| low_sdi | Prevalence | 1985 | 0.93(0.92 to 0.94) |
| low_sdi | Prevalence | 1990 | 0.92(0.9 to 0.93) |
| low_sdi | Prevalence | 1995 | 0.91(0.89 to 0.93) |
| low_sdi | Prevalence | 2000 | 0.9(0.88 to 0.92) |
| low_sdi | Deaths | 1900 | 0.94(0.75 to 1.18) |
| low_sdi | Deaths | 1905 | 1.03(0.93 to 1.15) |
| low_sdi | Deaths | 1910 | 1.07(0.99 to 1.14) |
| low_sdi | Deaths | 1915 | 1.07(1.01 to 1.14) |
| low_sdi | Deaths | 1920 | 1.12(1.07 to 1.18) |
| low_sdi | Deaths | 1925 | 1.18(1.12 to 1.23) |
| low_sdi | Deaths | 1930 | 1.19(1.14 to 1.24) |
| low_sdi | Deaths | 1935 | 1.2(1.15 to 1.25) |
| low_sdi | Deaths | 1940 | 1.14(1.09 to 1.18) |
| low_sdi | Deaths | 1945 | 1.08(1.03 to 1.12) |
| low_sdi | Deaths | 1950 | 1(1 to 1) |
| low_sdi | Deaths | 1955 | 0.9(0.86 to 0.94) |
| low_sdi | Deaths | 1960 | 0.83(0.78 to 0.88) |
| low_sdi | Deaths | 1965 | 0.77(0.71 to 0.83) |
| low_sdi | Deaths | 1970 | 0.69(0.62 to 0.76) |
| low_sdi | Deaths | 1975 | 0.65(0.57 to 0.75) |
| low_sdi | Deaths | 1980 | 0.61(0.51 to 0.73) |
| low_sdi | Deaths | 1985 | 0.57(0.45 to 0.72) |
| low_sdi | Deaths | 1990 | 0.54(0.4 to 0.72) |
| low_sdi | Deaths | 1995 | 0.5(0.34 to 0.73) |
| low_sdi | Deaths | 2000 | 0.47(0.27 to 0.83) |
| low_sdi | DALYs | 1900 | 0.94(0.7 to 1.26) |
| low_sdi | DALYs | 1905 | 1.03(0.9 to 1.17) |
| low_sdi | DALYs | 1910 | 1.05(0.98 to 1.14) |
| low_sdi | DALYs | 1915 | 1.06(1 to 1.12) |
| low_sdi | DALYs | 1920 | 1.1(1.05 to 1.14) |
| low_sdi | DALYs | 1925 | 1.14(1.1 to 1.18) |
| low_sdi | DALYs | 1930 | 1.16(1.12 to 1.2) |
| low_sdi | DALYs | 1935 | 1.17(1.13 to 1.21) |
| low_sdi | DALYs | 1940 | 1.12(1.09 to 1.16) |
| low_sdi | DALYs | 1945 | 1.07(1.04 to 1.11) |
| low_sdi | DALYs | 1950 | 1(1 to 1) |
| low_sdi | DALYs | 1955 | 0.91(0.88 to 0.95) |
| low_sdi | DALYs | 1960 | 0.86(0.83 to 0.9) |
| low_sdi | DALYs | 1965 | 0.81(0.78 to 0.85) |
| low_sdi | DALYs | 1970 | 0.75(0.71 to 0.8) |
| low_sdi | DALYs | 1975 | 0.73(0.68 to 0.78) |
| low_sdi | DALYs | 1980 | 0.7(0.65 to 0.76) |
| low_sdi | DALYs | 1985 | 0.68(0.62 to 0.74) |
| low_sdi | DALYs | 1990 | 0.65(0.59 to 0.73) |
| low_sdi | DALYs | 1995 | 0.62(0.55 to 0.71) |
| low_sdi | DALYs | 2000 | 0.6(0.5 to 0.73) |
