# Supplementary material for: Global, regional, and national temporal trends in prevalence, deaths and disability-adjusted life years for chronic pulmonary disease, 1990–2021: an age-period-cohort analysis based on the global burden of disease study 2021
Source: Front Med (Lausanne). 2025 Mar 4;12:1554442. doi: 10.3389/fmed.2025.1554442 (PMC11913687; doi:10.3389/fmed.2025.1554442)
Supplement: Supplementary file 7 [file Table_7.docx]

**Table S7** **Age effects on COPD related Prevalence, Deaths and DALYs across countries.**

Region, measure, age, Age effect rate(95%UI)

Australia,Prevalence,20~25,226.33(215.69to237.49)

Australia,Prevalence,25~30,290.76(279.52to302.44)

Australia,Prevalence,30~35,385.58(373.36to398.19)

Australia,Prevalence,35~40,530.92(517.03to545.18)

Australia,Prevalence,40~45,788.21(771.69to805.09)

Australia,Prevalence,45~50,1353.72(1331.48to1376.33)

Australia,Prevalence,50~55,2322.43(2291.55to2353.72)

Australia,Prevalence,55~60,3647.7(3606.03to3689.85)

Australia,Prevalence,60~65,5300.51(5245.92to5355.66)

Australia,Prevalence,65~70,7558.67(7485.85to7632.21)

Australia,Prevalence,70~75,10211.06(10114.69to10308.34)

Australia,Prevalence,75~80,12922.33(12782.05to13064.15)

Australia,Prevalence,80~85,15416.14(15239.78to15594.55)

Australia,Prevalence,85~90,17368.49(17148.74to17591.05)

Australia,Prevalence,90~95,18216.48(17928.07to18509.54)

Australia,Deaths,20~25,0.08(0.03to0.21)

Australia,Deaths,25~30,0.09(0.04to0.2)

Australia,Deaths,30~35,0.15(0.08to0.27)

Australia,Deaths,35~40,0.32(0.21to0.47)

Australia,Deaths,40~45,0.86(0.67to1.09)

Australia,Deaths,45~50,2.17(1.85to2.54)

Australia,Deaths,50~55,5.62(5.07to6.23)

Australia,Deaths,55~60,13.25(12.31to14.27)

Australia,Deaths,60~65,28.23(26.67to29.88)

Australia,Deaths,65~70,52.41(49.94to55)

Australia,Deaths,70~75,91.09(87.12to95.24)

Australia,Deaths,75~80,141.36(134.48to148.6)

Australia,Deaths,80~85,205.75(195.67to216.34)

Australia,Deaths,85~90,300.21(285.21to315.99)

Australia,Deaths,90~95,420.44(398.03to444.11)

Australia,DALYs,20~25,15.01(11.72to19.22)

Australia,DALYs,25~30,17.08(13.84to21.07)

Australia,DALYs,30~35,23.78(20.12to28.11)

Australia,DALYs,35~40,38.18(33.56to43.45)

Australia,DALYs,40~45,73.62(67.05to80.84)

Australia,DALYs,45~50,151.12(141.23to161.71)

Australia,DALYs,50~55,316.18(300.9to332.24)

Australia,DALYs,55~60,605.08(581.69to629.41)

Australia,DALYs,60~65,1051.15(1016.84to1086.62)

Australia,DALYs,65~70,1618.28(1570.43to1667.58)

Australia,DALYs,70~75,2288.36(2223.5to2355.12)

Australia,DALYs,75~80,2832.92(2741.38to2927.51)

Australia,DALYs,80~85,3231.06(3122.42to3343.48)

Australia,DALYs,85~90,3714(3579.81to3853.22)

Australia,DALYs,90~95,4434.37(4247.28to4629.71)

Austria,Prevalence,20~25,532.16(511.81to553.32)

Austria,Prevalence,25~30,743.41(720.7to766.83)

Austria,Prevalence,30~35,955.7(930.86to981.2)

Austria,Prevalence,35~40,1162.39(1135.55to1189.88)

Austria,Prevalence,40~45,1567.28(1536.65to1598.52)

Austria,Prevalence,45~50,2788.66(2745.44to2832.55)

Austria,Prevalence,50~55,4567.3(4508.32to4627.05)

Austria,Prevalence,55~60,6415.55(6340.61to6491.37)

Austria,Prevalence,60~65,8468.01(8374.74to8562.32)

Austria,Prevalence,65~70,11536.18(11414.28to11659.38)

Austria,Prevalence,70~75,15678.6(15514.43to15844.5)

Austria,Prevalence,75~80,21125.84(20879.86to21374.72)

Austria,Prevalence,80~85,28489.53(28146.76to28836.47)

Austria,Prevalence,85~90,38115.44(37620.95to38616.43)

Austria,Prevalence,90~95,47915.33(47180.93to48661.17)

Austria,Deaths,20~25,0.14(0.04to0.52)

Austria,Deaths,25~30,0.15(0.05to0.45)

Austria,Deaths,30~35,0.2(0.08to0.48)

Austria,Deaths,35~40,0.37(0.2to0.69)

Austria,Deaths,40~45,0.86(0.58to1.27)

Austria,Deaths,45~50,2.26(1.77to2.88)

Austria,Deaths,50~55,6.24(5.34to7.29)

Austria,Deaths,55~60,15.02(13.43to16.8)

Austria,Deaths,60~65,35.12(32.24to38.26)

Austria,Deaths,65~70,69.75(64.88to74.98)

Austria,Deaths,70~75,122.52(114.43to131.17)

Austria,Deaths,75~80,203.01(187.81to219.44)

Austria,Deaths,80~85,313.48(289.61to339.32)

Austria,Deaths,85~90,502.88(463.56to545.53)

Austria,Deaths,90~95,720.04(659.1to786.61)

Austria,DALYs,20~25,28.88(23.87to34.94)

Austria,DALYs,25~30,36.92(31.68to43.04)

Austria,DALYs,30~35,46.62(41to53)

Austria,DALYs,35~40,61.71(55.4to68.75)

Austria,DALYs,40~45,96.33(88.5to104.85)

Austria,DALYs,45~50,196.83(185.14to209.26)

Austria,DALYs,50~55,405.43(387.33to424.38)

Austria,DALYs,55~60,742.81(715.51to771.15)

Austria,DALYs,60~65,1325.6(1283.83to1368.72)

Austria,DALYs,65~70,2109.18(2048.7to2171.45)

Austria,DALYs,70~75,2998.79(2914.35to3085.68)

Austria,DALYs,75~80,3973.55(3845.25to4106.13)

Austria,DALYs,80~85,4923.66(4756.86to5096.3)

Austria,DALYs,85~90,6330.82(6098.9to6571.54)

Austria,DALYs,90~95,7883.4(7541to8241.34)

Cyprus,Prevalence,20~25,459.94(435.41to485.85)

Cyprus,Prevalence,25~30,631.57(603.53to660.92)

Cyprus,Prevalence,30~35,818.71(787.07to851.63)

Cyprus,Prevalence,35~40,1011.53(976.62to1047.69)

Cyprus,Prevalence,40~45,1312.56(1272.76to1353.6)

Cyprus,Prevalence,45~50,2049.04(1996.71to2102.75)

Cyprus,Prevalence,50~55,3261.05(3190.06to3333.62)

Cyprus,Prevalence,55~60,4767.39(4674.98to4861.62)

Cyprus,Prevalence,60~65,6600.2(6483.26to6719.25)

Cyprus,Prevalence,65~70,9280.15(9125.33to9437.6)

Cyprus,Prevalence,70~75,13111.7(12897.84to13329.1)

Cyprus,Prevalence,75~80,18342.71(17997.64to18694.41)

Cyprus,Prevalence,80~85,25314.25(24813.87to25824.72)

Cyprus,Prevalence,85~90,33855.74(33092.85to34636.23)

Cyprus,Prevalence,90~95,41769.56(40192.54to43408.46)

Cyprus,Deaths,20~25,0.27(0.01to6.69)

Cyprus,Deaths,25~30,0.28(0.02to3.92)

Cyprus,Deaths,30~35,0.35(0.04to2.96)

Cyprus,Deaths,35~40,0.52(0.1to2.65)

Cyprus,Deaths,40~45,1.04(0.34to3.21)

Cyprus,Deaths,45~50,2.44(1.16to5.1)

Cyprus,Deaths,50~55,5.96(3.65to9.74)

Cyprus,Deaths,55~60,12.81(8.98to18.3)

Cyprus,Deaths,60~65,26.37(20.02to34.74)

Cyprus,Deaths,65~70,46.14(36.4to58.49)

Cyprus,Deaths,70~75,86.11(69.25to107.08)

Cyprus,Deaths,75~80,148.51(116.66to189.06)

Cyprus,Deaths,80~85,327.58(257.88to416.12)

Cyprus,Deaths,85~90,649.9(510.38to827.56)

Cyprus,Deaths,90~95,1274.58(972.1to1671.16)

Cyprus,DALYs,20~25,37.26(24.74to56.11)

Cyprus,DALYs,25~30,46.55(33.18to65.32)

Cyprus,DALYs,30~35,58.16(43.54to77.7)

Cyprus,DALYs,35~40,73.09(56.97to93.77)

Cyprus,DALYs,40~45,106.2(86.5to130.4)

Cyprus,DALYs,45~50,190.69(162.84to223.31)

Cyprus,DALYs,50~55,360.88(319.48to407.65)

Cyprus,DALYs,55~60,623.35(564.15to688.75)

Cyprus,DALYs,60~65,1034.81(950.39to1126.74)

Cyprus,DALYs,65~70,1512.75(1399.35to1635.35)

Cyprus,DALYs,70~75,2294.25(2129.66to2471.56)

Cyprus,DALYs,75~80,3146.37(2893.04to3421.89)

Cyprus,DALYs,80~85,5225.21(4799.14to5689.1)

Cyprus,DALYs,85~90,8143.12(7446.34to8905.09)

Cyprus,DALYs,90~95,13608.36(12106.46to15296.59)

Iran_(Islamic_Republic_of),Prevalence,20~25,415.35(406.22to424.68)

Iran_(Islamic_Republic_of),Prevalence,25~30,560.44(549.45to571.65)

Iran_(Islamic_Republic_of),Prevalence,30~35,716.89(704.03to729.99)

Iran_(Islamic_Republic_of),Prevalence,35~40,882.44(867.67to897.46)

Iran_(Islamic_Republic_of),Prevalence,40~45,1121.46(1104.21to1138.99)

Iran_(Islamic_Republic_of),Prevalence,45~50,1612.47(1589.65to1635.61)

Iran_(Islamic_Republic_of),Prevalence,50~55,2424.86(2393.72to2456.4)

Iran_(Islamic_Republic_of),Prevalence,55~60,3551.04(3508.91to3593.69)

Iran_(Islamic_Republic_of),Prevalence,60~65,5022.05(4965.42to5079.33)

Iran_(Islamic_Republic_of),Prevalence,65~70,7017.19(6939.83to7095.4)

Iran_(Islamic_Republic_of),Prevalence,70~75,9999.35(9887.58to10112.39)

Iran_(Islamic_Republic_of),Prevalence,75~80,14654.24(14462.27to14848.76)

Iran_(Islamic_Republic_of),Prevalence,80~85,21844.4(21540.31to22152.77)

Iran_(Islamic_Republic_of),Prevalence,85~90,31797.78(31296.08to32307.53)

Iran_(Islamic_Republic_of),Prevalence,90~95,43395.08(42473.6to44336.56)

Iran_(Islamic_Republic_of),Deaths,20~25,0.68(0.58to0.81)

Iran_(Islamic_Republic_of),Deaths,25~30,0.88(0.76to1.01)

Iran_(Islamic_Republic_of),Deaths,30~35,1.16(1.02to1.32)

Iran_(Islamic_Republic_of),Deaths,35~40,1.55(1.39to1.74)

Iran_(Islamic_Republic_of),Deaths,40~45,2.54(2.31to2.78)

Iran_(Islamic_Republic_of),Deaths,45~50,4.46(4.13to4.82)

Iran_(Islamic_Republic_of),Deaths,50~55,8.98(8.44to9.55)

Iran_(Islamic_Republic_of),Deaths,55~60,15.57(14.78to16.41)

Iran_(Islamic_Republic_of),Deaths,60~65,29.01(27.74to30.34)

Iran_(Islamic_Republic_of),Deaths,65~70,47.3(45.38to49.3)

Iran_(Islamic_Republic_of),Deaths,70~75,83.68(80.42to87.08)

Iran_(Islamic_Republic_of),Deaths,75~80,158.97(152.11to166.14)

Iran_(Islamic_Republic_of),Deaths,80~85,270.88(258.98to283.33)

Iran_(Islamic_Republic_of),Deaths,85~90,439.87(419.45to461.29)

Iran_(Islamic_Republic_of),Deaths,90~95,649.2(614.15to686.26)

Iran_(Islamic_Republic_of),DALYs,20~25,73.09(69.03to77.4)

Iran_(Islamic_Republic_of),DALYs,25~30,91.66(87.08to96.49)

Iran_(Islamic_Republic_of),DALYs,30~35,114.1(108.89to119.55)

Iran_(Islamic_Republic_of),DALYs,35~40,139.57(133.67to145.74)

Iran_(Islamic_Republic_of),DALYs,40~45,192.97(185.66to200.56)

Iran_(Islamic_Republic_of),DALYs,45~50,293.53(283.57to303.83)

Iran_(Islamic_Republic_of),DALYs,50~55,494.22(479.69to509.18)

Iran_(Islamic_Republic_of),DALYs,55~60,743.23(723.51to763.49)

Iran_(Islamic_Republic_of),DALYs,60~65,1151.13(1122.98to1179.98)

Iran_(Islamic_Republic_of),DALYs,65~70,1584.3(1546.63to1622.89)

Iran_(Islamic_Republic_of),DALYs,70~75,2280.4(2225.88to2336.26)

Iran_(Islamic_Republic_of),DALYs,75~80,3423.13(3330.11to3518.76)

Iran_(Islamic_Republic_of),DALYs,80~85,4656.64(4522.36to4794.92)

Iran_(Islamic_Republic_of),DALYs,85~90,6134.01(5933.45to6341.36)

Iran_(Islamic_Republic_of),DALYs,90~95,7916.22(7571.04to8277.14)

Mauritania,Prevalence,20~25,417.14(401.23to433.68)

Mauritania,Prevalence,25~30,549.66(530.73to569.26)

Mauritania,Prevalence,30~35,702.46(680.04to725.63)

Mauritania,Prevalence,35~40,868.96(842.94to895.77)

Mauritania,Prevalence,40~45,1104.38(1074.25to1135.36)

Mauritania,Prevalence,45~50,1565.53(1525.96to1606.12)

Mauritania,Prevalence,50~55,2261.06(2207.65to2315.76)

Mauritania,Prevalence,55~60,3106.84(3037to3178.28)

Mauritania,Prevalence,60~65,4093.92(4004.63to4185.2)

Mauritania,Prevalence,65~70,5200.07(5087.21to5315.42)

Mauritania,Prevalence,70~75,6585.19(6436.21to6737.61)

Mauritania,Prevalence,75~80,8461.4(8235.15to8693.87)

Mauritania,Prevalence,80~85,11051.05(10720.47to11391.83)

Mauritania,Prevalence,85~90,14258.28(13739.51to14796.63)

Mauritania,Prevalence,90~95,17661.69(16710.71to18666.8)

Mauritania,Deaths,20~25,0.3(0.07to1.2)

Mauritania,Deaths,25~30,0.94(0.39to2.25)

Mauritania,Deaths,30~35,1.3(0.62to2.73)

Mauritania,Deaths,35~40,1.22(0.59to2.55)

Mauritania,Deaths,40~45,2.12(1.19to3.77)

Mauritania,Deaths,45~50,5.85(3.93to8.72)

Mauritania,Deaths,50~55,11.24(8.16to15.49)

Mauritania,Deaths,55~60,17.16(12.97to22.71)

Mauritania,Deaths,60~65,34.78(27.59to43.85)

Mauritania,Deaths,65~70,64.33(52.4to78.98)

Mauritania,Deaths,70~75,105.92(87.08to128.85)

Mauritania,Deaths,75~80,141.97(113.45to177.67)

Mauritania,Deaths,80~85,192.12(151.86to243.04)

Mauritania,Deaths,85~90,325.35(253.62to417.38)

Mauritania,Deaths,90~95,430.5(315.94to586.61)

Mauritania,DALYs,20~25,69.85(63.51to76.83)

Mauritania,DALYs,25~30,115.98(107.2to125.47)

Mauritania,DALYs,30~35,147.33(137.18to158.23)

Mauritania,DALYs,35~40,157.52(147.11to168.66)

Mauritania,DALYs,40~45,215.25(202.71to228.56)

Mauritania,DALYs,45~50,403.01(383.36to423.66)

Mauritania,DALYs,50~55,644.65(616.7to673.87)

Mauritania,DALYs,55~60,869.74(834.45to906.51)

Mauritania,DALYs,60~65,1393.98(1342.71to1447.2)

Mauritania,DALYs,65~70,2070.52(1998.66to2144.96)

Mauritania,DALYs,70~75,2766.05(2669.75to2865.82)

Mauritania,DALYs,75~80,3055.58(2930.01to3186.53)

Mauritania,DALYs,80~85,3351.01(3196.62to3512.84)

Mauritania,DALYs,85~90,4427.54(4189.82to4678.75)

Mauritania,DALYs,90~95,5087.75(4694.21to5514.29)

Nigeria,Prevalence,20~25,432.48(425.56to439.51)

Nigeria,Prevalence,25~30,583.09(574.71to591.6)

Nigeria,Prevalence,30~35,748.13(738.15to758.24)

Nigeria,Prevalence,35~40,921.17(909.57to932.92)

Nigeria,Prevalence,40~45,1168.09(1154.57to1181.77)

Nigeria,Prevalence,45~50,1697.03(1678.98to1715.28)

Nigeria,Prevalence,50~55,2577.57(2552.41to2602.97)

Nigeria,Prevalence,55~60,3725.44(3691.09to3760.1)

Nigeria,Prevalence,60~65,5092.84(5046.42to5139.69)

Nigeria,Prevalence,65~70,6697.48(6635.35to6760.2)

Nigeria,Prevalence,70~75,8634.18(8550.21to8718.97)

Nigeria,Prevalence,75~80,11029.38(10902.21to11158.04)

Nigeria,Prevalence,80~85,14001.81(13817.19to14188.89)

Nigeria,Prevalence,85~90,17220.5(16933.69to17512.17)

Nigeria,Prevalence,90~95,20089.2(19584.33to20607.09)

Nigeria,Deaths,20~25,0.13(0.1to0.17)

Nigeria,Deaths,25~30,0.49(0.42to0.57)

Nigeria,Deaths,30~35,0.83(0.73to0.94)

Nigeria,Deaths,35~40,0.97(0.86to1.09)

Nigeria,Deaths,40~45,1.84(1.68to2.01)

Nigeria,Deaths,45~50,5.42(5.11to5.76)

Nigeria,Deaths,50~55,10.31(9.83to10.82)

Nigeria,Deaths,55~60,17.32(16.62to18.04)

Nigeria,Deaths,60~65,37.04(35.77to38.34)

Nigeria,Deaths,65~70,69.13(66.98to71.35)

Nigeria,Deaths,70~75,128.07(124.25to132)

Nigeria,Deaths,75~80,175.08(169.14to181.23)

Nigeria,Deaths,80~85,242.51(233.83to251.52)

Nigeria,Deaths,85~90,373.23(358.68to388.36)

Nigeria,Deaths,90~95,488.32(464.57to513.29)

Nigeria,DALYs,20~25,57.73(55.89to59.62)

Nigeria,DALYs,25~30,92.79(90.29to95.37)

Nigeria,DALYs,30~35,125.41(122.36to128.54)

Nigeria,DALYs,35~40,146.81(143.43to150.27)

Nigeria,DALYs,40~45,205.2(201.01to209.48)

Nigeria,DALYs,45~50,391.78(385.15to398.53)

Nigeria,DALYs,50~55,632.36(622.94to641.92)

Nigeria,DALYs,55~60,922.73(910to935.62)

Nigeria,DALYs,60~65,1534.93(1515.37to1554.74)

Nigeria,DALYs,65~70,2298.99(2270.52to2327.82)

Nigeria,DALYs,70~75,3378.31(3336.25to3420.9)

Nigeria,DALYs,75~80,3796.7(3740.78to3853.46)

Nigeria,DALYs,80~85,4232.35(4161.32to4304.59)

Nigeria,DALYs,85~90,5142.73(5037.27to5250.4)

Nigeria,DALYs,90~95,5826.56(5651.78to6006.75)

Finland,Prevalence,20~25,444.99(421.91to469.32)

Finland,Prevalence,25~30,604.25(578.64to631)

Finland,Prevalence,30~35,778.15(750.23to807.12)

Finland,Prevalence,35~40,959.65(929.92to990.34)

Finland,Prevalence,40~45,1258.14(1225.98to1291.15)

Finland,Prevalence,45~50,2015.62(1974.56to2057.53)

Finland,Prevalence,50~55,3207.85(3153.63to3263.01)

Finland,Prevalence,55~60,4572.16(4503.61to4641.74)

Finland,Prevalence,60~65,6110.05(6025to6196.3)

Finland,Prevalence,65~70,8186.9(8078.49to8296.76)

Finland,Prevalence,70~75,10819.55(10679.12to10961.83)

Finland,Prevalence,75~80,14127.84(13915.5to14343.42)

Finland,Prevalence,80~85,18626.81(18333.49to18924.82)

Finland,Prevalence,85~90,25212.72(24782.69to25650.2)

Finland,Prevalence,90~95,33682.39(33003.12to34375.65)

Finland,Deaths,20~25,0.17(0.03to1.06)

Finland,Deaths,25~30,0.19(0.04to0.85)

Finland,Deaths,30~35,0.25(0.08to0.81)

Finland,Deaths,35~40,0.43(0.19to0.97)

Finland,Deaths,40~45,0.85(0.5to1.46)

Finland,Deaths,45~50,1.78(1.25to2.54)

Finland,Deaths,50~55,4.18(3.32to5.27)

Finland,Deaths,55~60,9.6(8.17to11.27)

Finland,Deaths,60~65,20.93(18.53to23.63)

Finland,Deaths,65~70,39.35(35.46to43.66)

Finland,Deaths,70~75,69.32(62.99to76.28)

Finland,Deaths,75~80,112.4(100.87to125.26)

Finland,Deaths,80~85,152.26(136.32to170.05)

Finland,Deaths,85~90,191.14(170.15to214.73)

Finland,Deaths,90~95,236.49(207.44to269.6)

Finland,DALYs,20~25,32.23(27.95to37.16)

Finland,DALYs,25~30,41(36.5to46.06)

Finland,DALYs,30~35,52.22(47.41to57.51)

Finland,DALYs,35~40,67.32(62.17to72.9)

Finland,DALYs,40~45,97.23(91.4to103.42)

Finland,DALYs,45~50,165.52(158to173.39)

Finland,DALYs,50~55,299.38(288.93to310.22)

Finland,DALYs,55~60,518.3(503.67to533.35)

Finland,DALYs,60~65,865.33(844.47to886.7)

Finland,DALYs,65~70,1308.11(1279.19to1337.69)

Finland,DALYs,70~75,1850.41(1811.09to1890.6)

Finland,DALYs,75~80,2396.96(2338.27to2457.12)

Finland,DALYs,80~85,2665.27(2596to2736.38)

Finland,DALYs,85~90,2871.29(2787.81to2957.26)

Finland,DALYs,90~95,3289.43(3171.64to3411.59)

Jordan,Prevalence,20~25,466.38(455.46to477.57)

Jordan,Prevalence,25~30,624.68(611.68to637.95)

Jordan,Prevalence,30~35,796.03(780.64to811.73)

Jordan,Prevalence,35~40,973.24(955.64to991.16)

Jordan,Prevalence,40~45,1250.54(1229.7to1271.73)

Jordan,Prevalence,45~50,1908.54(1879.75to1937.77)

Jordan,Prevalence,50~55,2983.91(2943.66to3024.71)

Jordan,Prevalence,55~60,4347.52(4293.42to4402.31)

Jordan,Prevalence,60~65,5944.76(5874.21to6016.16)

Jordan,Prevalence,65~70,7932.34(7841.47to8024.26)

Jordan,Prevalence,70~75,10676.61(10553.5to10801.16)

Jordan,Prevalence,75~80,14637.32(14425.99to14851.74)

Jordan,Prevalence,80~85,20102.91(19773.78to20437.52)

Jordan,Prevalence,85~90,27335.46(26772.31to27910.45)

Jordan,Prevalence,90~95,35816.09(34772.33to36891.17)

Jordan,Deaths,20~25,0.92(0.46to1.83)

Jordan,Deaths,25~30,1.11(0.61to2.03)

Jordan,Deaths,30~35,1.41(0.83to2.41)

Jordan,Deaths,35~40,1.76(1.09to2.82)

Jordan,Deaths,40~45,2.59(1.74to3.86)

Jordan,Deaths,45~50,4.11(2.94to5.74)

Jordan,Deaths,50~55,7.4(5.69to9.63)

Jordan,Deaths,55~60,11.8(9.46to14.71)

Jordan,Deaths,60~65,23.89(19.87to28.71)

Jordan,Deaths,65~70,36.01(30.36to42.7)

Jordan,Deaths,70~75,57.19(48.75to67.1)

Jordan,Deaths,75~80,72.2(59.63to87.43)

Jordan,Deaths,80~85,104.92(85.75to128.38)

Jordan,Deaths,85~90,173.5(139.42to215.91)

Jordan,Deaths,90~95,212.99(162.2to279.68)

Jordan,DALYs,20~25,85.29(79.83to91.11)

Jordan,DALYs,25~30,105.52(99.47to111.93)

Jordan,DALYs,30~35,129.4(122.52to136.68)

Jordan,DALYs,35~40,152.88(145.31to160.85)

Jordan,DALYs,40~45,199.53(190.59to208.89)

Jordan,DALYs,45~50,293.96(282.18to306.23)

Jordan,DALYs,50~55,463.45(447.35to480.13)

Jordan,DALYs,55~60,658.06(637.35to679.43)

Jordan,DALYs,60~65,1049.18(1019.14to1080.11)

Jordan,DALYs,65~70,1355.1(1317.51to1393.78)

Jordan,DALYs,70~75,1788.96(1740.01to1839.28)

Jordan,DALYs,75~80,1959.94(1893.34to2028.88)

Jordan,DALYs,80~85,2329.88(2240.57to2422.76)

Jordan,DALYs,85~90,3036.6(2895.27to3184.83)

Jordan,DALYs,90~95,3386.5(3162.24to3626.67)

Lebanon,Prevalence,20~25,399.58(388.82to410.63)

Lebanon,Prevalence,25~30,561.05(548.08to574.32)

Lebanon,Prevalence,30~35,737.65(722.21to753.42)

Lebanon,Prevalence,35~40,922.06(904.26to940.21)

Lebanon,Prevalence,40~45,1213.48(1192.5to1234.83)

Lebanon,Prevalence,45~50,1944.55(1915.64to1973.9)

Lebanon,Prevalence,50~55,3176.54(3135.51to3218.11)

Lebanon,Prevalence,55~60,4757.35(4702.18to4813.16)

Lebanon,Prevalence,60~65,6674.02(6602to6746.83)

Lebanon,Prevalence,65~70,9239.65(9144.08to9336.22)

Lebanon,Prevalence,70~75,12949.79(12816.24to13084.74)

Lebanon,Prevalence,75~80,18439.79(18219.68to18662.55)

Lebanon,Prevalence,80~85,26430.5(26094.02to26771.32)

Lebanon,Prevalence,85~90,37157.79(36626.16to37697.12)

Lebanon,Prevalence,90~95,49607.98(48706.09to50526.56)

Lebanon,Deaths,20~25,1.07(0.51to2.28)

Lebanon,Deaths,25~30,1.29(0.69to2.43)

Lebanon,Deaths,30~35,1.69(0.98to2.89)

Lebanon,Deaths,35~40,2.29(1.45to3.61)

Lebanon,Deaths,40~45,3.45(2.39to4.97)

Lebanon,Deaths,45~50,5.25(3.91to7.05)

Lebanon,Deaths,50~55,9.34(7.42to11.77)

Lebanon,Deaths,55~60,15.06(12.46to18.2)

Lebanon,Deaths,60~65,29.32(25.16to34.16)

Lebanon,Deaths,65~70,49.72(43.32to57.08)

Lebanon,Deaths,70~75,94.17(82.87to107.01)

Lebanon,Deaths,75~80,161.47(140.33to185.79)

Lebanon,Deaths,80~85,266.01(230.79to306.62)

Lebanon,Deaths,85~90,414.74(357.83to480.69)

Lebanon,Deaths,90~95,597(507.74to701.96)

Lebanon,DALYs,20~25,95.82(89.36to102.75)

Lebanon,DALYs,25~30,120.01(113.01to127.44)

Lebanon,DALYs,30~35,149.86(142to158.17)

Lebanon,DALYs,35~40,185.39(176.57to194.65)

Lebanon,DALYs,40~45,247.54(237.35to258.17)

Lebanon,DALYs,45~50,361.96(349.15to375.25)

Lebanon,DALYs,50~55,575.99(558.62to593.89)

Lebanon,DALYs,55~60,825.2(803.17to847.82)

Lebanon,DALYs,60~65,1289.29(1258.77to1320.54)

Lebanon,DALYs,65~70,1813.26(1772.49to1854.98)

Lebanon,DALYs,70~75,2739.19(2679.56to2800.14)

Lebanon,DALYs,75~80,3776.3(3684.19to3870.72)

Lebanon,DALYs,80~85,4956.26(4829.49to5086.36)

Lebanon,DALYs,85~90,6256.08(6079.66to6437.63)

Lebanon,DALYs,90~95,7882.94(7610.39to8165.25)

Germany,Prevalence,20~25,487.54(464.32to511.93)

Germany,Prevalence,25~30,666.2(641.12to692.26)

Germany,Prevalence,30~35,862.16(834.91to890.29)

Germany,Prevalence,35~40,1063.43(1034to1093.7)

Germany,Prevalence,40~45,1398.63(1365.78to1432.27)

Germany,Prevalence,45~50,2316.57(2272.31to2361.68)

Germany,Prevalence,50~55,3810.63(3750.71to3871.51)

Germany,Prevalence,55~60,5510.76(5433.59to5589.04)

Germany,Prevalence,60~65,7422.04(7324.19to7521.2)

Germany,Prevalence,65~70,10061.77(9934.62to10190.54)

Germany,Prevalence,70~75,13602.02(13433.34to13772.82)

Germany,Prevalence,75~80,18472.71(18218.15to18730.82)

Germany,Prevalence,80~85,25685.34(25324.06to26051.76)

Germany,Prevalence,85~90,35747.78(35205.28to36298.65)

Germany,Prevalence,90~95,47345.36(46490.95to48215.47)

Germany,Deaths,20~25,0.12(0.08to0.19)

Germany,Deaths,25~30,0.14(0.09to0.2)

Germany,Deaths,30~35,0.2(0.15to0.27)

Germany,Deaths,35~40,0.4(0.33to0.49)

Germany,Deaths,40~45,1.04(0.92to1.17)

Germany,Deaths,45~50,2.8(2.61to3.02)

Germany,Deaths,50~55,7.52(7.17to7.89)

Germany,Deaths,55~60,17.48(16.87to18.1)

Germany,Deaths,60~65,39.12(38.06to40.21)

Germany,Deaths,65~70,73.32(71.6to75.09)

Germany,Deaths,70~75,126.33(123.48to129.24)

Germany,Deaths,75~80,200.61(195.58to205.77)

Germany,Deaths,80~85,287.93(280.61to295.44)

Germany,Deaths,85~90,431.72(420.37to443.39)

Germany,Deaths,90~95,606.01(588.47to624.06)

Germany,DALYs,20~25,27.64(24.16to31.62)

Germany,DALYs,25~30,34.92(31.41to38.82)

Germany,DALYs,30~35,45.48(41.73to49.57)

Germany,DALYs,35~40,62.45(58.2to67.02)

Germany,DALYs,40~45,104.44(98.96to110.22)

Germany,DALYs,45~50,213.92(205.67to222.51)

Germany,DALYs,50~55,445.9(433.09to459.09)

Germany,DALYs,55~60,819.28(799.94to839.09)

Germany,DALYs,60~65,1442.89(1413.49to1472.9)

Germany,DALYs,65~70,2196.92(2155.66to2238.98)

Germany,DALYs,70~75,3068.38(3011.32to3126.53)

Germany,DALYs,75~80,3927(3844.56to4011.21)

Germany,DALYs,80~85,4609.91(4508.91to4713.17)

Germany,DALYs,85~90,5679.22(5543.09to5818.7)

Germany,DALYs,90~95,7044.64(6839.94to7255.48)

Senegal,Prevalence,20~25,440.13(431.86to448.56)

Senegal,Prevalence,25~30,591.89(581.84to602.12)

Senegal,Prevalence,30~35,760.56(748.53to772.78)

Senegal,Prevalence,35~40,936.16(922.15to950.38)

Senegal,Prevalence,40~45,1187.73(1171.34to1204.35)

Senegal,Prevalence,45~50,1722.34(1700.6to1744.36)

Senegal,Prevalence,50~55,2555.83(2526.33to2585.68)

Senegal,Prevalence,55~60,3566.26(3527.45to3605.5)

Senegal,Prevalence,60~65,4729.83(4679.66to4780.54)

Senegal,Prevalence,65~70,6017.36(5953.1to6082.32)

Senegal,Prevalence,70~75,7622.18(7536.94to7708.4)

Senegal,Prevalence,75~80,9738.98(9608.93to9870.79)

Senegal,Prevalence,80~85,12567.13(12375.93to12761.29)

Senegal,Prevalence,85~90,15799.47(15496.55to16108.32)

Senegal,Prevalence,90~95,18793(18246.28to19356.1)

Senegal,Deaths,20~25,0.26(0.14to0.48)

Senegal,Deaths,25~30,0.94(0.64to1.37)

Senegal,Deaths,30~35,1.32(0.95to1.84)

Senegal,Deaths,35~40,1.36(0.98to1.88)

Senegal,Deaths,40~45,2.6(2.01to3.37)

Senegal,Deaths,45~50,7.27(6.07to8.71)

Senegal,Deaths,50~55,13.89(12.01to16.07)

Senegal,Deaths,55~60,22.81(20.13to25.86)

Senegal,Deaths,60~65,47(42.35to52.17)

Senegal,Deaths,65~70,90.69(82.71to99.44)

Senegal,Deaths,70~75,159.56(146.09to174.26)

Senegal,Deaths,75~80,224.49(202.45to248.93)

Senegal,Deaths,80~85,316.75(283.76to353.58)

Senegal,Deaths,85~90,526.28(466.77to593.38)

Senegal,Deaths,90~95,705.93(605.4to823.15)

Senegal,DALYs,20~25,63.18(59.81to66.74)

Senegal,DALYs,25~30,118.09(112.91to123.51)

Senegal,DALYs,30~35,151.86(145.76to158.22)

Senegal,DALYs,35~40,165.01(158.6to171.69)

Senegal,DALYs,40~45,240.76(232.44to249.37)

Senegal,DALYs,45~50,477.4(463.84to491.36)

Senegal,DALYs,50~55,774.47(755.17to794.26)

Senegal,DALYs,55~60,1104.17(1078.81to1130.13)

Senegal,DALYs,60~65,1807.19(1769.45to1845.73)

Senegal,DALYs,65~70,2782.04(2727.09to2838.09)

Senegal,DALYs,70~75,3928.65(3850.57to4008.32)

Senegal,DALYs,75~80,4510.38(4402.1to4621.32)

Senegal,DALYs,80~85,5108.59(4968.24to5252.9)

Senegal,DALYs,85~90,6640.63(6418.92to6870)

Senegal,DALYs,90~95,7683.82(7303.12to8084.37)

Togo,Prevalence,20~25,456.54(444.66to468.74)

Togo,Prevalence,25~30,618.56(604.1to633.37)

Togo,Prevalence,30~35,795.42(778.22to813)

Togo,Prevalence,35~40,975.17(955.22to995.53)

Togo,Prevalence,40~45,1233.23(1209.93to1256.97)

Togo,Prevalence,45~50,1798.8(1767.38to1830.78)

Togo,Prevalence,50~55,2688.41(2644.76to2732.78)

Togo,Prevalence,55~60,3752.8(3694.65to3811.86)

Togo,Prevalence,60~65,4945.53(4870.2to5022.03)

Togo,Prevalence,65~70,6233.65(6137.06to6331.75)

Togo,Prevalence,70~75,7777.91(7647.52to7910.51)

Togo,Prevalence,75~80,9753.69(9548.79to9962.98)

Togo,Prevalence,80~85,12251.13(11937.73to12572.76)

Togo,Prevalence,85~90,15017.19(14495.74to15557.4)

Togo,Prevalence,90~95,17545.23(16559.32to18589.84)

Togo,Deaths,20~25,0.29(0.14to0.63)

Togo,Deaths,25~30,1.03(0.64to1.65)

Togo,Deaths,30~35,1.63(1.1to2.43)

Togo,Deaths,35~40,1.73(1.18to2.54)

Togo,Deaths,40~45,3.36(2.47to4.57)

Togo,Deaths,45~50,9.59(7.68to11.97)

Togo,Deaths,50~55,18.13(15.09to21.78)

Togo,Deaths,55~60,29.71(25.3to34.9)

Togo,Deaths,60~65,59.27(51.68to67.98)

Togo,Deaths,65~70,114.37(101.1to129.39)

Togo,Deaths,70~75,191.34(169.23to216.34)

Togo,Deaths,75~80,266.39(229.63to309.03)

Togo,Deaths,80~85,360.48(305.34to425.58)

Togo,Deaths,85~90,564.13(465.27to684.01)

Togo,Deaths,90~95,711.64(541.86to934.62)

Togo,DALYs,20~25,64.15(60.44to68.08)

Togo,DALYs,25~30,124.04(118.22to130.13)

Togo,DALYs,30~35,170.82(163.66to178.29)

Togo,DALYs,35~40,185.45(177.95to193.27)

Togo,DALYs,40~45,279.19(269.2to289.56)

Togo,DALYs,45~50,583.79(566.48to601.64)

Togo,DALYs,50~55,949.5(924.42to975.27)

Togo,DALYs,55~60,1355.55(1322.21to1389.72)

Togo,DALYs,60~65,2188.19(2138.59to2238.94)

Togo,DALYs,65~70,3391.81(3318.03to3467.24)

Togo,DALYs,70~75,4602.53(4497.55to4709.95)

Togo,DALYs,75~80,5217.65(5067.73to5372.01)

Togo,DALYs,80~85,5662.78(5462.73to5870.16)

Togo,DALYs,85~90,6973.15(6648.13to7314.07)

Togo,DALYs,90~95,7644.31(7070.37to8264.83)

Morocco,Prevalence,20~25,398.17(390.78to405.7)

Morocco,Prevalence,25~30,547.41(538.49to556.48)

Morocco,Prevalence,30~35,712.73(702.18to723.45)

Morocco,Prevalence,35~40,887.13(875.01to899.41)

Morocco,Prevalence,40~45,1166.35(1151.99to1180.88)

Morocco,Prevalence,45~50,1868.98(1848.77to1889.42)

Morocco,Prevalence,50~55,3043.45(3014.13to3073.04)

Morocco,Prevalence,55~60,4531.12(4491.01to4571.59)

Morocco,Prevalence,60~65,6295.15(6241.66to6349.09)

Morocco,Prevalence,65~70,8574.79(8502.22to8647.98)

Morocco,Prevalence,70~75,11758.9(11656.53to11862.16)

Morocco,Prevalence,75~80,16336.17(16169.3to16504.76)

Morocco,Prevalence,80~85,22944.25(22688.85to23202.53)

Morocco,Prevalence,85~90,31919.75(31496.45to32348.73)

Morocco,Prevalence,90~95,42626.81(41827.49to43441.4)

Morocco,Deaths,20~25,0.75(0.59to0.95)

Morocco,Deaths,25~30,0.92(0.75to1.13)

Morocco,Deaths,30~35,1.29(1.08to1.53)

Morocco,Deaths,35~40,1.81(1.56to2.1)

Morocco,Deaths,40~45,3.03(2.69to3.43)

Morocco,Deaths,45~50,5.46(4.95to6.03)

Morocco,Deaths,50~55,11.23(10.39to12.14)

Morocco,Deaths,55~60,20.3(19.01to21.67)

Morocco,Deaths,60~65,40.6(38.41to42.92)

Morocco,Deaths,65~70,72.79(69.2to76.57)

Morocco,Deaths,70~75,133.74(127.43to140.36)

Morocco,Deaths,75~80,230.04(217.65to243.15)

Morocco,Deaths,80~85,390.03(368.35to412.98)

Morocco,Deaths,85~90,630.56(592.85to670.67)

Morocco,Deaths,90~95,929.03(862.5to1000.71)

Morocco,DALYs,20~25,75.89(72.79to79.13)

Morocco,DALYs,25~30,93.99(90.59to97.51)

Morocco,DALYs,30~35,120.73(116.81to124.77)

Morocco,DALYs,35~40,152.52(148.05to157.13)

Morocco,DALYs,40~45,218.71(213.13to224.43)

Morocco,DALYs,45~50,351.62(343.89to359.53)

Morocco,DALYs,50~55,617.61(605.95to629.49)

Morocco,DALYs,55~60,958.86(942.59to975.41)

Morocco,DALYs,60~65,1557.36(1533.17to1581.94)

Morocco,DALYs,65~70,2289.16(2254.76to2324.08)

Morocco,DALYs,70~75,3379.35(3328.32to3431.16)

Morocco,DALYs,75~80,4645.47(4563.44to4728.96)

Morocco,DALYs,80~85,6213.64(6094.68to6334.91)

Morocco,DALYs,85~90,8065.27(7882.58to8252.2)

Morocco,DALYs,90~95,10346.29(10019.22to10684.03)

Oman,Prevalence,20~25,468.3(451.73to485.47)

Oman,Prevalence,25~30,605.33(587.14to624.09)

Oman,Prevalence,30~35,759.89(739.13to781.23)

Oman,Prevalence,35~40,927.48(903.59to951.99)

Oman,Prevalence,40~45,1205.49(1176.65to1235.03)

Oman,Prevalence,45~50,1907.03(1864.54to1950.49)

Oman,Prevalence,50~55,3103.13(3038.78to3168.85)

Oman,Prevalence,55~60,4659.05(4564.2to4755.86)

Oman,Prevalence,60~65,6487.23(6358.3to6618.78)

Oman,Prevalence,65~70,8858.25(8677.96to9042.28)

Oman,Prevalence,70~75,11998.97(11750.92to12252.24)

Oman,Prevalence,75~80,16373.47(15968.28to16788.94)

Oman,Prevalence,80~85,22281.44(21657.73to22923.11)

Oman,Prevalence,85~90,30071.35(29023.48to31157.04)

Oman,Prevalence,90~95,38860.7(37062.01to40746.69)

Oman,Deaths,20~25,1.02(0.34to3.06)

Oman,Deaths,25~30,0.97(0.39to2.41)

Oman,Deaths,30~35,1.05(0.47to2.35)

Oman,Deaths,35~40,1.31(0.64to2.66)

Oman,Deaths,40~45,2.06(1.13to3.73)

Oman,Deaths,45~50,3.73(2.28to6.12)

Oman,Deaths,50~55,7.55(5.05to11.3)

Oman,Deaths,55~60,14.21(10.03to20.15)

Oman,Deaths,60~65,29.94(22.34to40.11)

Oman,Deaths,65~70,57.74(44.22to75.39)

Oman,Deaths,70~75,92.87(71.66to120.36)

Oman,Deaths,75~80,141.72(105to191.29)

Oman,Deaths,80~85,207.35(150.73to285.25)

Oman,Deaths,85~90,301.69(210.96to431.45)

Oman,Deaths,90~95,337.95(213.19to535.74)

Oman,DALYs,20~25,85.67(76.32to96.16)

Oman,DALYs,25~30,93.53(84.72to103.24)

Oman,DALYs,30~35,107.72(98.48to117.83)

Oman,DALYs,35~40,127.53(117.3to138.64)

Oman,DALYs,40~45,171.6(159.11to185.07)

Oman,DALYs,45~50,275.71(257.52to295.19)

Oman,DALYs,50~55,474.61(446.5to504.49)

Oman,DALYs,55~60,757.5(715.1to802.41)

Oman,DALYs,60~65,1260.97(1195.99to1329.49)

Oman,DALYs,65~70,1956.95(1858.57to2060.54)

Oman,DALYs,70~75,2605.63(2472.64to2745.78)

Oman,DALYs,75~80,3261.62(3063.45to3472.6)

Oman,DALYs,80~85,3891.74(3622.78to4180.66)

Oman,DALYs,85~90,4602(4203.45to5038.33)

Oman,DALYs,90~95,4791.91(4204.91to5460.86)

Sudan,Prevalence,20~25,550.97(539.59to562.6)

Sudan,Prevalence,25~30,749.77(735.87to763.93)

Sudan,Prevalence,30~35,948.64(932.36to965.2)

Sudan,Prevalence,35~40,1137.96(1119.5to1156.71)

Sudan,Prevalence,40~45,1445.77(1424.21to1467.66)

Sudan,Prevalence,45~50,2284.15(2253.56to2315.16)

Sudan,Prevalence,50~55,3640.47(3596.13to3685.36)

Sudan,Prevalence,55~60,5180.89(5121.04to5241.43)

Sudan,Prevalence,60~65,6819.82(6742.67to6897.85)

Sudan,Prevalence,65~70,8869.01(8768.82to8970.36)

Sudan,Prevalence,70~75,11669.69(11535.04to11805.92)

Sudan,Prevalence,75~80,15483.68(15275.91to15694.28)

Sudan,Prevalence,80~85,20531.63(20227.61to20840.23)

Sudan,Prevalence,85~90,26718.02(26244.86to27199.71)

Sudan,Prevalence,90~95,33417.11(32595.58to34259.35)

Sudan,Deaths,20~25,1.72(1.4to2.1)

Sudan,Deaths,25~30,2.31(1.95to2.75)

Sudan,Deaths,30~35,3.15(2.71to3.66)

Sudan,Deaths,35~40,4.26(3.73to4.87)

Sudan,Deaths,40~45,6.48(5.79to7.26)

Sudan,Deaths,45~50,10.41(9.46to11.46)

Sudan,Deaths,50~55,19.66(18.16to21.29)

Sudan,Deaths,55~60,31.41(29.27to33.71)

Sudan,Deaths,60~65,56.14(52.76to59.74)

Sudan,Deaths,65~70,89.99(84.9to95.38)

Sudan,Deaths,70~75,147.56(139.51to156.06)

Sudan,Deaths,75~80,225.02(211.33to239.59)

Sudan,Deaths,80~85,330.17(309.27to352.47)

Sudan,Deaths,85~90,470.03(437.59to504.88)

Sudan,Deaths,90~95,625.6(573.14to682.87)

Sudan,DALYs,20~25,148.62(142.38to155.14)

Sudan,DALYs,25~30,191.17(184.01to198.61)

Sudan,DALYs,30~35,240.57(232.33to249.1)

Sudan,DALYs,35~40,294.9(285.54to304.56)

Sudan,DALYs,40~45,397.72(386.46to409.3)

Sudan,DALYs,45~50,588.94(574to604.27)

Sudan,DALYs,50~55,975.38(953.49to997.77)

Sudan,DALYs,55~60,1370.58(1341.64to1400.14)

Sudan,DALYs,60~65,2037.66(1997.23to2078.9)

Sudan,DALYs,65~70,2725.59(2672.3to2779.95)

Sudan,DALYs,70~75,3651.88(3579.95to3725.25)

Sudan,DALYs,75~80,4503.88(4401.56to4608.58)

Sudan,DALYs,80~85,5275.48(5142.26to5412.14)

Sudan,DALYs,85~90,6072.58(5886.77to6264.26)

Sudan,DALYs,90~95,7072.7(6769.73to7389.23)

Egypt,Prevalence,20~25,404.93(400.32to409.6)

Egypt,Prevalence,25~30,557.59(551.99to563.25)

Egypt,Prevalence,30~35,725.96(719.29to732.7)

Egypt,Prevalence,35~40,903.31(895.64to911.04)

Egypt,Prevalence,40~45,1192.23(1183.15to1201.39)

Egypt,Prevalence,45~50,1932.92(1920.01to1945.92)

Egypt,Prevalence,50~55,3182.14(3163.32to3201.06)

Egypt,Prevalence,55~60,4748.86(4722.89to4774.97)

Egypt,Prevalence,60~65,6590.43(6555.63to6625.42)

Egypt,Prevalence,65~70,8995.5(8947.49to9043.78)

Egypt,Prevalence,70~75,12417.26(12344.86to12490.09)

Egypt,Prevalence,75~80,17382.5(17256.22to17509.71)

Egypt,Prevalence,80~85,24458.54(24249.1to24669.79)

Egypt,Prevalence,85~90,33607.16(33228.57to33990.08)

Egypt,Prevalence,90~95,44332.69(43561.84to45117.19)

Egypt,Deaths,20~25,2.04(1.68to2.48)

Egypt,Deaths,25~30,2.46(2.08to2.91)

Egypt,Deaths,30~35,2.96(2.55to3.44)

Egypt,Deaths,35~40,4.03(3.56to4.56)

Egypt,Deaths,40~45,6(5.43to6.63)

Egypt,Deaths,45~50,10.2(9.43to11.04)

Egypt,Deaths,50~55,19.58(18.42to20.82)

Egypt,Deaths,55~60,29.01(27.46to30.64)

Egypt,Deaths,60~65,52.35(49.91to54.9)

Egypt,Deaths,65~70,75.79(72.4to79.33)

Egypt,Deaths,70~75,138.69(132.65to145.01)

Egypt,Deaths,75~80,209.45(198.98to220.47)

Egypt,Deaths,80~85,308.84(292.4to326.21)

Egypt,Deaths,85~90,470.59(442.28to500.72)

Egypt,Deaths,90~95,461.96(421.04to506.86)

Egypt,DALYs,20~25,155.8(142.8to169.99)

Egypt,DALYs,25~30,187.36(173.43to202.4)

Egypt,DALYs,30~35,219.98(204.95to236.12)

Egypt,DALYs,35~40,271.52(254.92to289.19)

Egypt,DALYs,40~45,362.58(343.48to382.74)

Egypt,DALYs,45~50,561.84(536.71to588.14)

Egypt,DALYs,50~55,949.26(913.31to986.62)

Egypt,DALYs,55~60,1275.14(1229.71to1322.24)

Egypt,DALYs,60~65,1929.16(1865.47to1995.03)

Egypt,DALYs,65~70,2391.93(2312.61to2473.98)

Egypt,DALYs,70~75,3541.68(3420.27to3667.4)

Egypt,DALYs,75~80,4359.44(4179.4to4547.23)

Egypt,DALYs,80~85,5142.92(4896.23to5402.04)

Egypt,DALYs,85~90,6252.73(5868.15to6662.5)

Egypt,DALYs,90~95,5613.84(5029.16to6266.5)

Niger,Prevalence,20~25,451.33(443.45to459.35)

Niger,Prevalence,25~30,617.54(607.83to627.4)

Niger,Prevalence,30~35,796.49(784.91to808.25)

Niger,Prevalence,35~40,978.3(964.94to991.85)

Niger,Prevalence,40~45,1247.15(1231.43to1263.07)

Niger,Prevalence,45~50,1871.59(1849.7to1893.73)

Niger,Prevalence,50~55,2887.16(2856.38to2918.27)

Niger,Prevalence,55~60,4118.27(4077.4to4159.56)

Niger,Prevalence,60~65,5512.21(5458.82to5566.11)

Niger,Prevalence,65~70,7074.3(7004.57to7144.73)

Niger,Prevalence,70~75,8999.84(8905.78to9094.89)

Niger,Prevalence,75~80,11549.46(11397.73to11703.21)

Niger,Prevalence,80~85,14891.19(14652to15134.28)

Niger,Prevalence,85~90,18781.52(18375.09to19196.94)

Niger,Prevalence,90~95,22644.5(21873.91to23442.23)

Niger,Deaths,20~25,0.25(0.14to0.45)

Niger,Deaths,25~30,0.94(0.65to1.37)

Niger,Deaths,30~35,1.44(1.06to1.96)

Niger,Deaths,35~40,1.53(1.14to2.06)

Niger,Deaths,40~45,2.78(2.19to3.53)

Niger,Deaths,45~50,8.02(6.78to9.5)

Niger,Deaths,50~55,16.2(14.17to18.51)

Niger,Deaths,55~60,26.16(23.32to29.34)

Niger,Deaths,60~65,54.16(49.19to59.63)

Niger,Deaths,65~70,104.77(96.22to114.08)

Niger,Deaths,70~75,178.33(164.23to193.64)

Niger,Deaths,75~80,251.56(227.65to277.98)

Niger,Deaths,80~85,347.79(311.31to388.54)

Niger,Deaths,85~90,592.04(522.41to670.95)

Niger,Deaths,90~95,777.59(656.62to920.85)

Niger,DALYs,20~25,67(64.25to69.86)

Niger,DALYs,25~30,123.36(119.18to127.68)

Niger,DALYs,30~35,164.28(159.27to169.46)

Niger,DALYs,35~40,181.61(176.32to187.05)

Niger,DALYs,40~45,258.87(252.16to265.76)

Niger,DALYs,45~50,523.91(512.58to535.48)

Niger,DALYs,50~55,892.34(875.64to909.36)

Niger,DALYs,55~60,1267.46(1245.73to1289.56)

Niger,DALYs,60~65,2085.48(2052.83to2118.65)

Niger,DALYs,65~70,3228.4(3180.39to3277.13)

Niger,DALYs,70~75,4449.5(4382to4518.04)

Niger,DALYs,75~80,5124.58(5026.65to5224.42)

Niger,DALYs,80~85,5686.85(5554.02to5822.87)

Niger,DALYs,85~90,7544.55(7318.78to7777.28)

Niger,DALYs,90~95,8618.97(8219.15to9038.25)

New_Zealand,Prevalence,20~25,234.73(223.75to246.25)

New_Zealand,Prevalence,25~30,308.76(296.74to321.27)

New_Zealand,Prevalence,30~35,411.02(397.82to424.66)

New_Zealand,Prevalence,35~40,556.58(541.59to571.99)

New_Zealand,Prevalence,40~45,802.81(785.16to820.84)

New_Zealand,Prevalence,45~50,1342.36(1318.8to1366.34)

New_Zealand,Prevalence,50~55,2271.49(2238.8to2304.66)

New_Zealand,Prevalence,55~60,3554.74(3510.52to3599.52)

New_Zealand,Prevalence,60~65,5196.28(5137.72to5255.5)

New_Zealand,Prevalence,65~70,7525.9(7446.42to7606.23)

New_Zealand,Prevalence,70~75,10567.28(10458.34to10677.35)

New_Zealand,Prevalence,75~80,14004.3(13838.06to14172.54)

New_Zealand,Prevalence,80~85,17529.05(17309.97to17750.9)

New_Zealand,Prevalence,85~90,20743.55(20456.8to21034.31)

New_Zealand,Prevalence,90~95,22809.81(22413.01to23213.63)

New_Zealand,Deaths,20~25,0.14(0.02to0.92)

New_Zealand,Deaths,25~30,0.11(0.02to0.72)

New_Zealand,Deaths,30~35,0.23(0.07to0.75)

New_Zealand,Deaths,35~40,0.6(0.3to1.22)

New_Zealand,Deaths,40~45,1.49(0.96to2.3)

New_Zealand,Deaths,45~50,3.56(2.68to4.73)

New_Zealand,Deaths,50~55,8.4(6.93to10.17)

New_Zealand,Deaths,55~60,18.68(16.24to21.49)

New_Zealand,Deaths,60~65,37.46(33.49to41.9)

New_Zealand,Deaths,65~70,67.42(61.15to74.32)

New_Zealand,Deaths,70~75,116.14(105.98to127.26)

New_Zealand,Deaths,75~80,180.99(163.56to200.28)

New_Zealand,Deaths,80~85,263.47(237.89to291.79)

New_Zealand,Deaths,85~90,369.47(332.62to410.4)

New_Zealand,Deaths,90~95,522.74(466.55to585.69)

New_Zealand,DALYs,20~25,20.7(16.73to25.62)

New_Zealand,DALYs,25~30,21.92(18.18to26.44)

New_Zealand,DALYs,30~35,32.54(28.21to37.54)

New_Zealand,DALYs,35~40,57.14(51.45to63.46)

New_Zealand,DALYs,40~45,107.02(99.24to115.41)

New_Zealand,DALYs,45~50,212.8(201.46to224.77)

New_Zealand,DALYs,50~55,422.2(405.26to439.84)

New_Zealand,DALYs,55~60,784.68(759.33to810.87)

New_Zealand,DALYs,60~65,1314.45(1277.66to1352.31)

New_Zealand,DALYs,65~70,1978.72(1927.86to2030.92)

New_Zealand,DALYs,70~75,2794.88(2725.15to2866.38)

New_Zealand,DALYs,75~80,3495.7(3397.29to3596.96)

New_Zealand,DALYs,80~85,4013.94(3895.97to4135.49)

New_Zealand,DALYs,85~90,4486.43(4342.77to4634.84)

New_Zealand,DALYs,90~95,5447.14(5239.22to5663.31)

Andorra,Prevalence,20~25,488.98(396.32to603.29)

Andorra,Prevalence,25~30,676.42(577.52to792.25)

Andorra,Prevalence,30~35,874.89(767.24to997.65)

Andorra,Prevalence,35~40,1074.68(956.57to1207.39)

Andorra,Prevalence,40~45,1417.04(1281.11to1567.39)

Andorra,Prevalence,45~50,2359.56(2169.44to2566.35)

Andorra,Prevalence,50~55,3863.84(3593.87to4154.09)

Andorra,Prevalence,55~60,5542.17(5188.4to5920.07)

Andorra,Prevalence,60~65,7412.32(6955.87to7898.73)

Andorra,Prevalence,65~70,10080.55(9475.93to10723.75)

Andorra,Prevalence,70~75,13743.57(12929.57to14608.81)

Andorra,Prevalence,75~80,18517.97(17270.94to19855.04)

Andorra,Prevalence,80~85,24824.67(23084.02to26696.58)

Andorra,Prevalence,85~90,32951.18(30499.92to35599.44)

Andorra,Prevalence,90~95,41003.07(37483.45to44853.17)

Andorra,Deaths,20~25,0.22(0to56593.44)

Andorra,Deaths,25~30,0.3(0to1977.94)

Andorra,Deaths,30~35,0.41(0to294.35)

Andorra,Deaths,35~40,0.75(0.01to74.03)

Andorra,Deaths,40~45,1.52(0.06to36.09)

Andorra,Deaths,45~50,3.53(0.42to29.52)

Andorra,Deaths,50~55,8.83(2.07to37.71)

Andorra,Deaths,55~60,17.34(5.63to53.43)

Andorra,Deaths,60~65,32.06(12.44to82.64)

Andorra,Deaths,65~70,56.23(24.24to130.44)

Andorra,Deaths,70~75,97.07(43.77to215.27)

Andorra,Deaths,75~80,162.92(68.06to390.01)

Andorra,Deaths,80~85,262.39(109.56to628.41)

Andorra,Deaths,85~90,416.73(172.6to1006.17)

Andorra,Deaths,90~95,568.47(224.66to1438.46)

Andorra,DALYs,20~25,38.57(16.61to89.56)

Andorra,DALYs,25~30,51.76(27.8to96.37)

Andorra,DALYs,30~35,66.1(40.04to109.14)

Andorra,DALYs,35~40,89.6(59.12to135.79)

Andorra,DALYs,40~45,134.93(96.56to188.54)

Andorra,DALYs,45~50,252.51(195.42to326.29)

Andorra,DALYs,50~55,498.26(407.69to608.94)

Andorra,DALYs,55~60,813.92(685.84to965.92)

Andorra,DALYs,60~65,1241.47(1062.05to1451.19)

Andorra,DALYs,65~70,1803.83(1557.23to2089.47)

Andorra,DALYs,70~75,2537.93(2197.15to2931.57)

Andorra,DALYs,75~80,3403.23(2896.43to3998.69)

Andorra,DALYs,80~85,4312.97(3651.53to5094.22)

Andorra,DALYs,85~90,5454.19(4580.17to6495.01)

Andorra,DALYs,90~95,6495.33(5324.09to7924.24)

Democratic_People's_Republic_of_Korea,Prevalence,20~25,569.66(553.24to586.57)

Democratic_People's_Republic_of_Korea,Prevalence,25~30,778.73(760.17to797.74)

Democratic_People's_Republic_of_Korea,Prevalence,30~35,984.92(964.38to1005.89)

Democratic_People's_Republic_of_Korea,Prevalence,35~40,1176.42(1154.21to1199.07)

Democratic_People's_Republic_of_Korea,Prevalence,40~45,1568.31(1542.54to1594.5)

Democratic_People's_Republic_of_Korea,Prevalence,45~50,2777.14(2739.44to2815.35)

Democratic_People's_Republic_of_Korea,Prevalence,50~55,4455.7(4403.71to4508.31)

Democratic_People's_Republic_of_Korea,Prevalence,55~60,6031.27(5964.97to6098.32)

Democratic_People's_Republic_of_Korea,Prevalence,60~65,7941.97(7858.87to8025.95)

Democratic_People's_Republic_of_Korea,Prevalence,65~70,11687.64(11568.84to11807.66)

Democratic_People's_Republic_of_Korea,Prevalence,70~75,15984.23(15820.71to16149.44)

Democratic_People's_Republic_of_Korea,Prevalence,75~80,20244.76(20005.7to20486.69)

Democratic_People's_Republic_of_Korea,Prevalence,80~85,24073.77(23753.29to24398.57)

Democratic_People's_Republic_of_Korea,Prevalence,85~90,28084.47(27603.98to28573.32)

Democratic_People's_Republic_of_Korea,Prevalence,90~95,30662.8(29799.19to31551.45)

Democratic_People's_Republic_of_Korea,Deaths,20~25,0.87(0.68to1.12)

Democratic_People's_Republic_of_Korea,Deaths,25~30,1.21(0.99to1.47)

Democratic_People's_Republic_of_Korea,Deaths,30~35,2.28(1.98to2.63)

Democratic_People's_Republic_of_Korea,Deaths,35~40,3.94(3.54to4.39)

Democratic_People's_Republic_of_Korea,Deaths,40~45,8.91(8.28to9.6)

Democratic_People's_Republic_of_Korea,Deaths,45~50,18.93(17.93to19.98)

Democratic_People's_Republic_of_Korea,Deaths,50~55,43.28(41.64to44.98)

Democratic_People's_Republic_of_Korea,Deaths,55~60,82.25(79.71to84.87)

Democratic_People's_Republic_of_Korea,Deaths,60~65,175.57(171.14to180.11)

Democratic_People's_Republic_of_Korea,Deaths,65~70,324.17(316.66to331.85)

Democratic_People's_Republic_of_Korea,Deaths,70~75,637.32(623.33to651.62)

Democratic_People's_Republic_of_Korea,Deaths,75~80,1047.16(1021.6to1073.37)

Democratic_People's_Republic_of_Korea,Deaths,80~85,1726.01(1682.56to1770.58)

Democratic_People's_Republic_of_Korea,Deaths,85~90,2725.23(2651.09to2801.45)

Democratic_People's_Republic_of_Korea,Deaths,90~95,3814.05(3685.58to3947.01)

Democratic_People's_Republic_of_Korea,DALYs,20~25,105.99(98.75to113.76)

Democratic_People's_Republic_of_Korea,DALYs,25~30,139.92(132.1to148.21)

Democratic_People's_Republic_of_Korea,DALYs,30~35,211.33(201.81to221.29)

Democratic_People's_Republic_of_Korea,DALYs,35~40,301.95(290.62to313.73)

Democratic_People's_Republic_of_Korea,DALYs,40~45,549.44(533.53to565.82)

Democratic_People's_Republic_of_Korea,DALYs,45~50,1027.02(1003.61to1050.98)

Democratic_People's_Republic_of_Korea,DALYs,50~55,1994.41(1958.84to2030.63)

Democratic_People's_Republic_of_Korea,DALYs,55~60,3215.61(3165.14to3266.9)

Democratic_People's_Republic_of_Korea,DALYs,60~65,5670.65(5592.74to5749.65)

Democratic_People's_Republic_of_Korea,DALYs,65~70,8783.68(8668.1to8900.8)

Democratic_People's_Republic_of_Korea,DALYs,70~75,13992.92(13812.64to14175.55)

Democratic_People's_Republic_of_Korea,DALYs,75~80,18360.07(18091.3to18632.83)

Democratic_People's_Republic_of_Korea,DALYs,80~85,23523.16(23150.44to23901.89)

Democratic_People's_Republic_of_Korea,DALYs,85~90,29340.49(28785.54to29906.14)

Democratic_People's_Republic_of_Korea,DALYs,90~95,35352.65(34373.29to36359.92)

Iceland,Prevalence,20~25,573.01(520.59to630.71)

Iceland,Prevalence,25~30,792.08(731.5to857.67)

Iceland,Prevalence,30~35,1008.68(941.4to1080.77)

Iceland,Prevalence,35~40,1214.37(1141.94to1291.39)

Iceland,Prevalence,40~45,1604.09(1522.06to1690.54)

Iceland,Prevalence,45~50,2751.25(2637.31to2870.11)

Iceland,Prevalence,50~55,4394.2(4239.09to4554.98)

Iceland,Prevalence,55~60,6088.78(5894.85to6289.09)

Iceland,Prevalence,60~65,8005.17(7766.54to8251.14)

Iceland,Prevalence,65~70,10980.27(10665.96to11303.83)

Iceland,Prevalence,70~75,15045.12(14616.61to15486.18)

Iceland,Prevalence,75~80,20159.45(19510.56to20829.92)

Iceland,Prevalence,80~85,26394.41(25508.69to27310.88)

Iceland,Prevalence,85~90,33976.39(32752.33to35246.21)

Iceland,Prevalence,90~95,41256.55(39496.67to43094.85)

Iceland,Deaths,20~25,0.14(0to31.08)

Iceland,Deaths,25~30,0.16(0to19.61)

Iceland,Deaths,30~35,0.22(0to12.07)

Iceland,Deaths,35~40,0.38(0.02to8.13)

Iceland,Deaths,40~45,0.9(0.11to7.2)

Iceland,Deaths,45~50,1.97(0.47to8.24)

Iceland,Deaths,50~55,4.68(1.76to12.5)

Iceland,Deaths,55~60,10.54(5.16to21.54)

Iceland,Deaths,60~65,21.55(12.28to37.82)

Iceland,Deaths,65~70,42(25.97to67.95)

Iceland,Deaths,70~75,83.39(53.42to130.17)

Iceland,Deaths,75~80,157.25(96.2to257.04)

Iceland,Deaths,80~85,274.54(167.86to449.01)

Iceland,Deaths,85~90,444.82(269.7to733.65)

Iceland,Deaths,90~95,675.72(399.2to1143.8)

Iceland,DALYs,20~25,38.53(26.88to55.23)

Iceland,DALYs,25~30,49.6(36.59to67.22)

Iceland,DALYs,30~35,63.23(48.64to82.18)

Iceland,DALYs,35~40,80.96(64.49to101.62)

Iceland,DALYs,40~45,124.16(103.29to149.23)

Iceland,DALYs,45~50,225.12(195.4to259.36)

Iceland,DALYs,50~55,405.67(362.47to454.02)

Iceland,DALYs,55~60,671.98(611.42to738.53)

Iceland,DALYs,60~65,1045.83(962.41to1136.47)

Iceland,DALYs,65~70,1608.06(1488.94to1736.72)

Iceland,DALYs,70~75,2468.81(2290.86to2660.57)

Iceland,DALYs,75~80,3570.54(3284.55to3881.42)

Iceland,DALYs,80~85,4778.72(4384.79to5208.04)

Iceland,DALYs,85~90,6114.43(5581.23to6698.57)

Iceland,DALYs,90~95,7896.53(7115.26to8763.6)

Israel,Prevalence,20~25,480.85(470.13to491.82)

Israel,Prevalence,25~30,657.26(644.7to670.06)

Israel,Prevalence,30~35,846.77(832.5to861.27)

Israel,Prevalence,35~40,1039.36(1023.74to1055.23)

Israel,Prevalence,40~45,1350.13(1332.53to1367.97)

Israel,Prevalence,45~50,2143.26(2119.82to2166.95)

Israel,Prevalence,50~55,3410.43(3378.48to3442.67)

Israel,Prevalence,55~60,4889.41(4848.61to4930.55)

Israel,Prevalence,60~65,6555.57(6505.07to6606.46)

Israel,Prevalence,65~70,8737.55(8673.49to8802.09)

Israel,Prevalence,70~75,11703.96(11619.24to11789.3)

Israel,Prevalence,75~80,15832.98(15698.62to15968.5)

Israel,Prevalence,80~85,21781.44(21590.18to21974.39)

Israel,Prevalence,85~90,29820.13(29537.99to30104.97)

Israel,Prevalence,90~95,37874.16(37444.57to38308.67)

Israel,Deaths,20~25,0.09(0.02to0.44)

Israel,Deaths,25~30,0.11(0.03to0.41)

Israel,Deaths,30~35,0.15(0.05to0.45)

Israel,Deaths,35~40,0.27(0.12to0.6)

Israel,Deaths,40~45,0.68(0.41to1.14)

Israel,Deaths,45~50,1.7(1.22to2.38)

Israel,Deaths,50~55,4.52(3.62to5.65)

Israel,Deaths,55~60,9.89(8.4to11.64)

Israel,Deaths,60~65,20.64(18.15to23.47)

Israel,Deaths,65~70,35.75(31.97to39.97)

Israel,Deaths,70~75,61.24(55.16to67.99)

Israel,Deaths,75~80,105.97(94.22to119.2)

Israel,Deaths,80~85,160.56(142.67to180.7)

Israel,Deaths,85~90,284.4(252.39to320.47)

Israel,Deaths,90~95,393.72(346.53to447.33)

Israel,DALYs,20~25,25.69(22.49to29.35)

Israel,DALYs,25~30,33.37(29.79to37.37)

Israel,DALYs,30~35,42.77(38.76to47.19)

Israel,DALYs,35~40,55.61(51.09to60.53)

Israel,DALYs,40~45,85.61(79.91to91.72)

Israel,DALYs,45~50,158.18(149.95to166.87)

Israel,DALYs,50~55,310.62(297.96to323.82)

Israel,DALYs,55~60,533.3(515.26to551.98)

Israel,DALYs,60~65,870.49(844.85to896.9)

Israel,DALYs,65~70,1238.52(1204.72to1273.27)

Israel,DALYs,70~75,1723.11(1677.13to1770.36)

Israel,DALYs,75~80,2366.49(2294to2441.27)

Israel,DALYs,80~85,2890.39(2798.7to2985.09)

Israel,DALYs,85~90,4017.58(3883.08to4156.74)

Israel,DALYs,90~95,4883.4(4692.41to5082.17)

Sao_Tome_and_Principe,Prevalence,20~25,474.5(409.54to549.76)

Sao_Tome_and_Principe,Prevalence,25~30,659.26(577.17to753.03)

Sao_Tome_and_Principe,Prevalence,30~35,848.33(749.27to960.49)

Sao_Tome_and_Principe,Prevalence,35~40,1031.98(916.84to1161.57)

Sao_Tome_and_Principe,Prevalence,40~45,1314.53(1178.35to1466.46)

Sao_Tome_and_Principe,Prevalence,45~50,2030.99(1838.89to2243.15)

Sao_Tome_and_Principe,Prevalence,50~55,3236.38(2956.82to3542.36)

Sao_Tome_and_Principe,Prevalence,55~60,4664.45(4287.97to5073.99)

Sao_Tome_and_Principe,Prevalence,60~65,6256.41(5767.51to6786.76)

Sao_Tome_and_Principe,Prevalence,65~70,8004.03(7375.36to8686.28)

Sao_Tome_and_Principe,Prevalence,70~75,10051.29(9224.47to10952.22)

Sao_Tome_and_Principe,Prevalence,75~80,12509.47(11316.24to13828.53)

Sao_Tome_and_Principe,Prevalence,80~85,15820.68(14150.16to17688.42)

Sao_Tome_and_Principe,Prevalence,85~90,19393.28(16866.28to22298.89)

Sao_Tome_and_Principe,Prevalence,90~95,22185.51(17883.92to27521.75)

Sao_Tome_and_Principe,Deaths,20~25,0.88(0.04to18.81)

Sao_Tome_and_Principe,Deaths,25~30,2.95(0.43to20.33)

Sao_Tome_and_Principe,Deaths,30~35,3.94(0.73to21.22)

Sao_Tome_and_Principe,Deaths,35~40,3.76(0.7to20.2)

Sao_Tome_and_Principe,Deaths,40~45,6.21(1.52to25.34)

Sao_Tome_and_Principe,Deaths,45~50,17.26(6.37to46.77)

Sao_Tome_and_Principe,Deaths,50~55,32.52(14.5to72.93)

Sao_Tome_and_Principe,Deaths,55~60,52.97(26.45to106.09)

Sao_Tome_and_Principe,Deaths,60~65,116.67(66.07to206.03)

Sao_Tome_and_Principe,Deaths,65~70,237.35(142.73to394.7)

Sao_Tome_and_Principe,Deaths,70~75,424.8(259.83to694.51)

Sao_Tome_and_Principe,Deaths,75~80,590.98(338.92to1030.52)

Sao_Tome_and_Principe,Deaths,80~85,790.73(440.74to1418.65)

Sao_Tome_and_Principe,Deaths,85~90,1278.9(679.47to2407.14)

Sao_Tome_and_Principe,Deaths,90~95,1593.16(710.58to3571.94)

Sao_Tome_and_Principe,DALYs,20~25,109.19(81.9to145.56)

Sao_Tome_and_Principe,DALYs,25~30,242.87(194.53to303.23)

Sao_Tome_and_Principe,DALYs,30~35,305.17(249.26to373.62)

Sao_Tome_and_Principe,DALYs,35~40,300.33(245.75to367.03)

Sao_Tome_and_Principe,DALYs,40~45,425.14(355.46to508.48)

Sao_Tome_and_Principe,DALYs,45~50,932.63(808.55to1075.74)

Sao_Tome_and_Principe,DALYs,50~55,1549.83(1369.26to1754.23)

Sao_Tome_and_Principe,DALYs,55~60,2219.79(1982.34to2485.68)

Sao_Tome_and_Principe,DALYs,60~65,3965.49(3587.03to4383.87)

Sao_Tome_and_Principe,DALYs,65~70,6552.07(5960.68to7202.13)

Sao_Tome_and_Principe,DALYs,70~75,9505.54(8643.19to10453.93)

Sao_Tome_and_Principe,DALYs,75~80,10669.22(9546.81to11923.59)

Sao_Tome_and_Principe,DALYs,80~85,11349.36(10017.02to12858.9)

Sao_Tome_and_Principe,DALYs,85~90,14442.4(12431.22to16778.95)

Sao_Tome_and_Principe,DALYs,90~95,15567.72(12433.02to19492.76)

China,Prevalence,20~25,585.45(559.02to613.13)

China,Prevalence,25~30,772.38(743.34to802.54)

China,Prevalence,30~35,957.59(925.86to990.41)

China,Prevalence,35~40,1131.38(1097.51to1166.29)

China,Prevalence,40~45,1438.83(1401.16to1477.51)

China,Prevalence,45~50,2298.96(2248.61to2350.44)

China,Prevalence,50~55,3631.1(3562.64to3700.88)

China,Prevalence,55~60,5126.13(5038.09to5215.71)

China,Prevalence,60~65,7001.87(6888.94to7116.64)

China,Prevalence,65~70,10338.37(10180.91to10498.26)

China,Prevalence,70~75,14715.54(14491.46to14943.09)

China,Prevalence,75~80,19548.68(19197.1to19906.71)

China,Prevalence,80~85,24144.71(23661.21to24638.08)

China,Prevalence,85~90,28550.49(27838.07to29281.15)

China,Prevalence,90~95,31467.27(30236.98to32747.6)

China,Deaths,20~25,3.43(2.52to4.67)

China,Deaths,25~30,3.49(2.7to4.5)

China,Deaths,30~35,5.42(4.5to6.53)

China,Deaths,35~40,7.56(6.57to8.71)

China,Deaths,40~45,12.58(11.38to13.9)

China,Deaths,45~50,17.38(16.05to18.82)

China,Deaths,50~55,32.98(31.15to34.91)

China,Deaths,55~60,51.5(49.15to53.96)

China,Deaths,60~65,90.61(87.15to94.21)

China,Deaths,65~70,145.26(140.23to150.48)

China,Deaths,70~75,277.38(268.52to286.53)

China,Deaths,75~80,423.33(408.63to438.56)

China,Deaths,80~85,678.17(654.44to702.77)

China,Deaths,85~90,1153.1(1111.43to1196.32)

China,Deaths,90~95,1467.19(1406.53to1530.45)

China,DALYs,20~25,274.93(247.66to305.2)

China,DALYs,25~30,300.38(275.18to327.88)

China,DALYs,30~35,397.74(370.43to427.06)

China,DALYs,35~40,491.07(462.8to521.07)

China,DALYs,40~45,706.14(674.32to739.46)

China,DALYs,45~50,934.15(898.6to971.1)

China,DALYs,50~55,1541.61(1495.03to1589.64)

China,DALYs,55~60,2117.06(2060.54to2175.13)

China,DALYs,60~65,3146.4(3071.23to3223.41)

China,DALYs,65~70,4280.98(4184.45to4379.74)

China,DALYs,70~75,6645.96(6503.4to6791.64)

China,DALYs,75~80,8174.3(7979.01to8374.38)

China,DALYs,80~85,10225.32(9969.28to10487.94)

China,DALYs,85~90,13671.22(13292.79to14060.43)

China,DALYs,90~95,15160.76(14601.27to15741.7)

Iraq,Prevalence,20~25,415.28(406.66to424.07)

Iraq,Prevalence,25~30,556.33(546.03to566.82)

Iraq,Prevalence,30~35,712.81(700.63to725.2)

Iraq,Prevalence,35~40,880.11(866.01to894.43)

Iraq,Prevalence,40~45,1135.75(1119.12to1152.62)

Iraq,Prevalence,45~50,1701.79(1679.2to1724.68)

Iraq,Prevalence,50~55,2597.88(2566.41to2629.74)

Iraq,Prevalence,55~60,3759.42(3716.75to3802.58)

Iraq,Prevalence,60~65,5197.06(5140.17to5254.59)

Iraq,Prevalence,65~70,7030.64(6954.28to7107.83)

Iraq,Prevalence,70~75,9651.11(9542.67to9760.79)

Iraq,Prevalence,75~80,13479.78(13295.61to13666.5)

Iraq,Prevalence,80~85,19044.84(18755.6to19338.55)

Iraq,Prevalence,85~90,26696.84(26219.28to27183.1)

Iraq,Prevalence,90~95,36180.04(35319.18to37061.89)

Iraq,Deaths,20~25,0.75(0.53to1.06)

Iraq,Deaths,25~30,0.78(0.57to1.06)

Iraq,Deaths,30~35,0.97(0.74to1.27)

Iraq,Deaths,35~40,1.38(1.09to1.73)

Iraq,Deaths,40~45,2.09(1.72to2.53)

Iraq,Deaths,45~50,4.16(3.58to4.83)

Iraq,Deaths,50~55,7.41(6.55to8.38)

Iraq,Deaths,55~60,10.81(9.67to12.08)

Iraq,Deaths,60~65,22.11(20.13to24.28)

Iraq,Deaths,65~70,34.61(31.69to37.79)

Iraq,Deaths,70~75,60.15(55.38to65.33)

Iraq,Deaths,75~80,90.68(82.19to100.05)

Iraq,Deaths,80~85,136.3(122.89to151.17)

Iraq,Deaths,85~90,196.34(175.21to220.01)

Iraq,Deaths,90~95,273.28(237.98to313.82)

Iraq,DALYs,20~25,72.58(66.83to78.82)

Iraq,DALYs,25~30,84.08(78.06to90.57)

Iraq,DALYs,30~35,102.01(95.28to109.22)

Iraq,DALYs,35~40,127.97(120.19to136.26)

Iraq,DALYs,40~45,169.85(160.57to179.65)

Iraq,DALYs,45~50,279.97(266.81to293.77)

Iraq,DALYs,50~55,438.92(420.53to458.1)

Iraq,DALYs,55~60,591.26(567.85to615.63)

Iraq,DALYs,60~65,954.01(919.23to990.1)

Iraq,DALYs,65~70,1269.22(1223.48to1316.66)

Iraq,DALYs,70~75,1789.87(1725.32to1856.84)

Iraq,DALYs,75~80,2243.97(2145.27to2347.21)

Iraq,DALYs,80~85,2771.52(2634.79to2915.35)

Iraq,DALYs,85~90,3343.79(3145.3to3554.81)

Iraq,DALYs,90~95,4145.28(3811.42to4508.38)

Kuwait,Prevalence,20~25,376.2(361.84to391.14)

Kuwait,Prevalence,25~30,504.06(488.49to520.13)

Kuwait,Prevalence,30~35,650.3(632.37to668.74)

Kuwait,Prevalence,35~40,813.3(792.33to834.83)

Kuwait,Prevalence,40~45,1058.67(1033.21to1084.76)

Kuwait,Prevalence,45~50,1567.41(1531.56to1604.09)

Kuwait,Prevalence,50~55,2387.64(2335.45to2441)

Kuwait,Prevalence,55~60,3515.06(3439.68to3592.1)

Kuwait,Prevalence,60~65,4997.64(4892.57to5104.96)

Kuwait,Prevalence,65~70,7019.28(6873.06to7168.61)

Kuwait,Prevalence,70~75,9938.79(9729.18to10152.92)

Kuwait,Prevalence,75~80,14269.52(13906.35to14642.17)

Kuwait,Prevalence,80~85,20672.24(20118.65to21241.07)

Kuwait,Prevalence,85~90,29264.59(28396.18to30159.55)

Kuwait,Prevalence,90~95,39595.41(38144.99to41100.98)

Kuwait,Deaths,20~25,0.29(0.03to3.33)

Kuwait,Deaths,25~30,0.31(0.05to1.96)

Kuwait,Deaths,30~35,0.33(0.07to1.57)

Kuwait,Deaths,35~40,0.35(0.09to1.45)

Kuwait,Deaths,40~45,0.54(0.17to1.74)

Kuwait,Deaths,45~50,0.89(0.33to2.42)

Kuwait,Deaths,50~55,1.52(0.64to3.61)

Kuwait,Deaths,55~60,2.53(1.17to5.45)

Kuwait,Deaths,60~65,4.77(2.43to9.36)

Kuwait,Deaths,65~70,7.18(3.78to13.64)

Kuwait,Deaths,70~75,13.31(7.36to24.06)

Kuwait,Deaths,75~80,20.17(10.42to39.03)

Kuwait,Deaths,80~85,33.87(17.5to65.56)

Kuwait,Deaths,85~90,54(27.38to106.49)

Kuwait,Deaths,90~95,67.02(31.72to141.63)

Kuwait,DALYs,20~25,36.39(31.73to41.75)

Kuwait,DALYs,25~30,46.64(41.76to52.09)

Kuwait,DALYs,30~35,57.77(52.35to63.74)

Kuwait,DALYs,35~40,69.78(63.63to76.51)

Kuwait,DALYs,40~45,91.21(83.76to99.32)

Kuwait,DALYs,45~50,134.74(124.35to146)

Kuwait,DALYs,50~55,203.83(188.88to219.97)

Kuwait,DALYs,55~60,298.58(277.33to321.47)

Kuwait,DALYs,60~65,438.46(408.26to470.89)

Kuwait,DALYs,65~70,589.82(549.37to633.24)

Kuwait,DALYs,70~75,853.17(795.05to915.55)

Kuwait,DALYs,75~80,1127.11(1037.03to1225.01)

Kuwait,DALYs,80~85,1540.34(1412to1680.35)

Kuwait,DALYs,85~90,2040.43(1854.16to2245.41)

Kuwait,DALYs,90~95,2464.61(2187.93to2776.27)

Saudi_Arabia,Prevalence,20~25,254.07(250.68to257.51)

Saudi_Arabia,Prevalence,25~30,358.08(353.95to362.25)

Saudi_Arabia,Prevalence,30~35,486.16(481.19to491.18)

Saudi_Arabia,Prevalence,35~40,640.69(634.7to646.74)

Saudi_Arabia,Prevalence,40~45,897.05(889.41to904.76)

Saudi_Arabia,Prevalence,45~50,1494.19(1482.59to1505.88)

Saudi_Arabia,Prevalence,50~55,2478.5(2460.59to2496.54)

Saudi_Arabia,Prevalence,55~60,3800.63(3774.55to3826.9)

Saudi_Arabia,Prevalence,60~65,5428.52(5391.39to5465.9)

Saudi_Arabia,Prevalence,65~70,7508.64(7456.18to7561.47)

Saudi_Arabia,Prevalence,70~75,10427.04(10348.51to10506.15)

Saudi_Arabia,Prevalence,75~80,14616.02(14476.59to14756.79)

Saudi_Arabia,Prevalence,80~85,20627.08(20400.98to20855.7)

Saudi_Arabia,Prevalence,85~90,29121.76(28733.05to29515.72)

Saudi_Arabia,Prevalence,90~95,39742.54(38993.06to40506.43)

Saudi_Arabia,Deaths,20~25,0.5(0.38to0.65)

Saudi_Arabia,Deaths,25~30,0.78(0.62to0.97)

Saudi_Arabia,Deaths,30~35,1.22(1.01to1.46)

Saudi_Arabia,Deaths,35~40,1.94(1.66to2.27)

Saudi_Arabia,Deaths,40~45,3.62(3.18to4.11)

Saudi_Arabia,Deaths,45~50,6.68(5.99to7.45)

Saudi_Arabia,Deaths,50~55,13.48(12.28to14.8)

Saudi_Arabia,Deaths,55~60,24.11(22.21to26.17)

Saudi_Arabia,Deaths,60~65,46.28(42.97to49.84)

Saudi_Arabia,Deaths,65~70,72.22(67.19to77.62)

Saudi_Arabia,Deaths,70~75,121.11(112.66to130.18)

Saudi_Arabia,Deaths,75~80,199.25(183.37to216.51)

Saudi_Arabia,Deaths,80~85,292.48(267.83to319.38)

Saudi_Arabia,Deaths,85~90,438.27(398.16to482.42)

Saudi_Arabia,Deaths,90~95,587.35(521.01to662.13)

Saudi_Arabia,DALYs,20~25,48.44(43.99to53.34)

Saudi_Arabia,DALYs,25~30,69.63(64.12to75.61)

Saudi_Arabia,DALYs,30~35,98.41(91.61to105.72)

Saudi_Arabia,DALYs,35~40,139.52(130.87to148.73)

Saudi_Arabia,DALYs,40~45,224.01(211.74to236.99)

Saudi_Arabia,DALYs,45~50,373.49(355.08to392.85)

Saudi_Arabia,DALYs,50~55,660.18(630.73to691)

Saudi_Arabia,DALYs,55~60,1034.24(991.39to1078.94)

Saudi_Arabia,DALYs,60~65,1666.14(1599.88to1735.14)

Saudi_Arabia,DALYs,65~70,2211.1(2121.36to2304.64)

Saudi_Arabia,DALYs,70~75,3048.46(2917.7to3185.09)

Saudi_Arabia,DALYs,75~80,4028.87(3820.54to4248.57)

Saudi_Arabia,DALYs,80~85,4723(4443.44to5020.15)

Saudi_Arabia,DALYs,85~90,5720.69(5313.15to6159.49)

Saudi_Arabia,DALYs,90~95,6787.08(6106.09to7544.01)

Sierra_Leone,Prevalence,20~25,454.17(442.36to466.29)

Sierra_Leone,Prevalence,25~30,616.26(601.96to630.89)

Sierra_Leone,Prevalence,30~35,793.65(776.67to811.01)

Sierra_Leone,Prevalence,35~40,975.45(955.64to995.67)

Sierra_Leone,Prevalence,40~45,1236.31(1213.12to1259.95)

Sierra_Leone,Prevalence,45~50,1807.16(1775.38to1839.5)

Sierra_Leone,Prevalence,50~55,2707.7(2663.21to2752.94)

Sierra_Leone,Prevalence,55~60,3786.32(3727.01to3846.58)

Sierra_Leone,Prevalence,60~65,4997.82(4921.57to5075.25)

Sierra_Leone,Prevalence,65~70,6317.96(6221.49to6415.93)

Sierra_Leone,Prevalence,70~75,7916.5(7791.6to8043.41)

Sierra_Leone,Prevalence,75~80,9964.3(9779.17to10152.94)

Sierra_Leone,Prevalence,80~85,12623.46(12355.14to12897.61)

Sierra_Leone,Prevalence,85~90,15564.6(15140.86to16000.21)

Sierra_Leone,Prevalence,90~95,18422.56(17653.65to19224.97)

Sierra_Leone,Deaths,20~25,0.2(0.08to0.47)

Sierra_Leone,Deaths,25~30,0.75(0.44to1.28)

Sierra_Leone,Deaths,30~35,1.17(0.74to1.83)

Sierra_Leone,Deaths,35~40,1.33(0.86to2.05)

Sierra_Leone,Deaths,40~45,2.49(1.75to3.53)

Sierra_Leone,Deaths,45~50,7.41(5.77to9.53)

Sierra_Leone,Deaths,50~55,14.95(12.2to18.32)

Sierra_Leone,Deaths,55~60,24.79(20.78to29.58)

Sierra_Leone,Deaths,60~65,50.67(43.71to58.74)

Sierra_Leone,Deaths,65~70,99.85(87.64to113.75)

Sierra_Leone,Deaths,70~75,172.86(152.44to196.02)

Sierra_Leone,Deaths,75~80,244.3(211.63to282)

Sierra_Leone,Deaths,80~85,329.28(282.57to383.71)

Sierra_Leone,Deaths,85~90,536.81(453.83to634.97)

Sierra_Leone,Deaths,90~95,682(548.16to848.52)

Sierra_Leone,DALYs,20~25,55.97(52.56to59.6)

Sierra_Leone,DALYs,25~30,106.81(101.51to112.39)

Sierra_Leone,DALYs,30~35,144.44(138to151.18)

Sierra_Leone,DALYs,35~40,164.54(157.52to171.88)

Sierra_Leone,DALYs,40~45,238.36(229.24to247.84)

Sierra_Leone,DALYs,45~50,490.64(474.84to506.98)

Sierra_Leone,DALYs,50~55,827.41(803.78to851.72)

Sierra_Leone,DALYs,55~60,1189.1(1157.58to1221.49)

Sierra_Leone,DALYs,60~65,1936.43(1889.63to1984.39)

Sierra_Leone,DALYs,65~70,3031.03(2962to3101.67)

Sierra_Leone,DALYs,70~75,4214.78(4118.13to4313.71)

Sierra_Leone,DALYs,75~80,4849.17(4718.33to4983.63)

Sierra_Leone,DALYs,80~85,5271.94(5108.57to5440.53)

Sierra_Leone,DALYs,85~90,6726.88(6470.4to6993.53)

Sierra_Leone,DALYs,90~95,7448.36(7019.69to7903.21)

Tunisia,Prevalence,20~25,459.11(449.93to468.49)

Tunisia,Prevalence,25~30,637.59(626.46to648.9)

Tunisia,Prevalence,30~35,822.25(809.35to835.36)

Tunisia,Prevalence,35~40,1007.34(992.85to1022.05)

Tunisia,Prevalence,40~45,1323.39(1306.23to1340.77)

Tunisia,Prevalence,45~50,2203.79(2178.92to2228.95)

Tunisia,Prevalence,50~55,3667.3(3631.21to3703.75)

Tunisia,Prevalence,55~60,5428.06(5379.35to5477.2)

Tunisia,Prevalence,60~65,7483.73(7420.23to7547.77)

Tunisia,Prevalence,65~70,10337.25(10252.54to10422.67)

Tunisia,Prevalence,70~75,14462.42(14342.38to14583.46)

Tunisia,Prevalence,75~80,20400.38(20199.57to20603.19)

Tunisia,Prevalence,80~85,28512.83(28209.21to28819.73)

Tunisia,Prevalence,85~90,38596.52(38118.02to39081.03)

Tunisia,Prevalence,90~95,49293.82(48456.22to50145.9)

Tunisia,Deaths,20~25,0.47(0.28to0.78)

Tunisia,Deaths,25~30,0.67(0.44to1.03)

Tunisia,Deaths,30~35,1.08(0.77to1.52)

Tunisia,Deaths,35~40,1.43(1.06to1.92)

Tunisia,Deaths,40~45,2.31(1.81to2.95)

Tunisia,Deaths,45~50,3.94(3.23to4.81)

Tunisia,Deaths,50~55,7.4(6.31to8.67)

Tunisia,Deaths,55~60,13.6(11.93to15.51)

Tunisia,Deaths,60~65,26.64(23.88to29.73)

Tunisia,Deaths,65~70,46.84(42.48to51.63)

Tunisia,Deaths,70~75,85.48(77.96to93.72)

Tunisia,Deaths,75~80,154.33(138.99to171.36)

Tunisia,Deaths,80~85,300.45(270.37to333.89)

Tunisia,Deaths,85~90,494.74(442.83to552.73)

Tunisia,Deaths,90~95,699.47(616.12to794.1)

Tunisia,DALYs,20~25,61.33(58.63to64.16)

Tunisia,DALYs,25~30,83.66(80.45to86.99)

Tunisia,DALYs,30~35,114.46(110.62to118.44)

Tunisia,DALYs,35~40,138.95(134.7to143.34)

Tunisia,DALYs,40~45,192.92(187.72to198.27)

Tunisia,DALYs,45~50,306.73(299.55to314.07)

Tunisia,DALYs,50~55,509.53(499.29to519.98)

Tunisia,DALYs,55~60,788.56(774.42to802.96)

Tunisia,DALYs,60~65,1223.79(1203.79to1244.13)

Tunisia,DALYs,65~70,1759.92(1732.62to1787.65)

Tunisia,DALYs,70~75,2574.4(2534.73to2614.7)

Tunisia,DALYs,75~80,3666.42(3600.58to3733.46)

Tunisia,DALYs,80~85,5443.46(5340.64to5548.27)

Tunisia,DALYs,85~90,7169.59(7015.89to7326.66)

Tunisia,DALYs,90~95,8854.7(8604.03to9112.67)

Belgium,Prevalence,20~25,415.36(404.04to426.99)

Belgium,Prevalence,25~30,576.1(563.49to588.99)

Belgium,Prevalence,30~35,756.24(742.37to770.38)

Belgium,Prevalence,35~40,945.62(930.56to960.92)

Belgium,Prevalence,40~45,1280.4(1263.36to1297.66)

Belgium,Prevalence,45~50,2207.58(2184.02to2231.4)

Belgium,Prevalence,50~55,3689.47(3656.82to3722.42)

Belgium,Prevalence,55~60,5345.6(5303.65to5387.88)

Belgium,Prevalence,60~65,7159.5(7106.95to7212.44)

Belgium,Prevalence,65~70,9628.07(9560.26to9696.36)

Belgium,Prevalence,70~75,12924.02(12834.08to13014.59)

Belgium,Prevalence,75~80,17242.17(17107.42to17377.99)

Belgium,Prevalence,80~85,23323.73(23136.43to23512.55)

Belgium,Prevalence,85~90,31818.38(31545.97to32093.14)

Belgium,Prevalence,90~95,41027.77(40611.95to41447.85)

Belgium,Deaths,20~25,0.36(0.17to0.78)

Belgium,Deaths,25~30,0.39(0.21to0.74)

Belgium,Deaths,30~35,0.5(0.3to0.83)

Belgium,Deaths,35~40,0.84(0.58to1.22)

Belgium,Deaths,40~45,1.86(1.46to2.37)

Belgium,Deaths,45~50,4.4(3.76to5.15)

Belgium,Deaths,50~55,10.74(9.67to11.93)

Belgium,Deaths,55~60,22.67(20.98to24.49)

Belgium,Deaths,60~65,47.9(45.07to50.9)

Belgium,Deaths,65~70,82.78(78.41to87.38)

Belgium,Deaths,70~75,137.52(130.6to144.8)

Belgium,Deaths,75~80,210.91(199.36to223.12)

Belgium,Deaths,80~85,299.95(283.44to317.41)

Belgium,Deaths,85~90,448.04(422.91to474.66)

Belgium,Deaths,90~95,627.19(589.53to667.26)

Belgium,DALYs,20~25,42.07(35.77to49.48)

Belgium,DALYs,25~30,50.69(44.37to57.92)

Belgium,DALYs,30~35,63.21(56.61to70.59)

Belgium,DALYs,35~40,85.69(78.31to93.77)

Belgium,DALYs,40~45,142.46(133.13to152.45)

Belgium,DALYs,45~50,281.22(267.65to295.48)

Belgium,DALYs,50~55,563.89(543.37to585.19)

Belgium,DALYs,55~60,983.31(954.06to1013.46)

Belgium,DALYs,60~65,1685.4(1642.34to1729.59)

Belgium,DALYs,65~70,2426.84(2368.72to2486.38)

Belgium,DALYs,70~75,3306.08(3228.12to3385.93)

Belgium,DALYs,75~80,4096.86(3989.43to4207.18)

Belgium,DALYs,80~85,4680.09(4552.86to4810.89)

Belgium,DALYs,85~90,5644.61(5480.73to5813.4)

Belgium,DALYs,90~95,6908.3(6673.22to7151.66)

Luxembourg,Prevalence,20~25,504.49(463.61to548.98)

Luxembourg,Prevalence,25~30,692.61(648.6to739.6)

Luxembourg,Prevalence,30~35,890.38(842.8to940.64)

Luxembourg,Prevalence,35~40,1088.33(1036.94to1142.27)

Luxembourg,Prevalence,40~45,1419.52(1361.7to1479.8)

Luxembourg,Prevalence,45~50,2308.12(2229.93to2389.05)

Luxembourg,Prevalence,50~55,3731.84(3624.42to3842.45)

Luxembourg,Prevalence,55~60,5356.43(5217.5to5499.06)

Luxembourg,Prevalence,60~65,7205.65(7029.97to7385.72)

Luxembourg,Prevalence,65~70,9820.85(9590.78to10056.43)

Luxembourg,Prevalence,70~75,13336.03(13027.89to13651.47)

Luxembourg,Prevalence,75~80,17956(17487.51to18437.03)

Luxembourg,Prevalence,80~85,24299.2(23643.79to24972.78)

Luxembourg,Prevalence,85~90,32851.87(31899.92to33832.23)

Luxembourg,Prevalence,90~95,41719.43(40268.47to43222.66)

Luxembourg,Deaths,20~25,0.3(0to41.49)

Luxembourg,Deaths,25~30,0.3(0.01to14.7)

Luxembourg,Deaths,30~35,0.38(0.02to7.42)

Luxembourg,Deaths,35~40,0.62(0.07to5.35)

Luxembourg,Deaths,40~45,1.23(0.28to5.31)

Luxembourg,Deaths,45~50,2.94(1.14to7.62)

Luxembourg,Deaths,50~55,7.02(3.73to13.21)

Luxembourg,Deaths,55~60,14.42(9.02to23.06)

Luxembourg,Deaths,60~65,30.35(21to43.86)

Luxembourg,Deaths,65~70,57.91(42.17to79.51)

Luxembourg,Deaths,70~75,101.01(75.11to135.84)

Luxembourg,Deaths,75~80,163.23(117.46to226.82)

Luxembourg,Deaths,80~85,251.97(181.13to350.52)

Luxembourg,Deaths,85~90,416.35(297.71to582.25)

Luxembourg,Deaths,90~95,644.11(450.65to920.63)

Luxembourg,DALYs,20~25,42.22(30.12to59.18)

Luxembourg,DALYs,25~30,52.15(40.08to67.85)

Luxembourg,DALYs,30~35,64.35(51.89to79.8)

Luxembourg,DALYs,35~40,82.55(68.92to98.88)

Luxembourg,DALYs,40~45,120.04(103.81to138.8)

Luxembourg,DALYs,45~50,222.86(199.65to248.77)

Luxembourg,DALYs,50~55,420.98(386.8to458.17)

Luxembourg,DALYs,55~60,703.6(655.78to754.91)

Luxembourg,DALYs,60~65,1175.85(1106.13to1249.97)

Luxembourg,DALYs,65~70,1834.1(1734.24to1939.71)

Luxembourg,DALYs,70~75,2603.78(2466.21to2749.03)

Luxembourg,DALYs,75~80,3376.79(3175.95to3590.32)

Luxembourg,DALYs,80~85,4158.61(3903.24to4430.68)

Luxembourg,DALYs,85~90,5472.7(5116.74to5853.42)

Luxembourg,DALYs,90~95,7260.21(6714.15to7850.68)

Libya,Prevalence,20~25,406.71(397.04to416.62)

Libya,Prevalence,25~30,561.2(549.34to573.32)

Libya,Prevalence,30~35,731.39(717.21to745.85)

Libya,Prevalence,35~40,908.53(892.02to925.34)

Libya,Prevalence,40~45,1182.4(1162.59to1202.55)

Libya,Prevalence,45~50,1837.77(1809.64to1866.35)

Libya,Prevalence,50~55,2908.68(2867.83to2950.12)

Libya,Prevalence,55~60,4291.78(4235.11to4349.21)

Libya,Prevalence,60~65,5965.07(5888.98to6042.14)

Libya,Prevalence,65~70,8098.64(7996to8202.6)

Libya,Prevalence,70~75,11088.5(10944.68to11234.21)

Libya,Prevalence,75~80,15395.02(15160.88to15632.79)

Libya,Prevalence,80~85,21538.25(21182.35to21900.13)

Libya,Prevalence,85~90,30016.34(29448.7to30594.92)

Libya,Prevalence,90~95,40287.09(39310.38to41288.07)

Libya,Deaths,20~25,0.47(0.26to0.84)

Libya,Deaths,25~30,0.67(0.41to1.09)

Libya,Deaths,30~35,1.15(0.77to1.71)

Libya,Deaths,35~40,1.77(1.25to2.5)

Libya,Deaths,40~45,2.9(2.14to3.93)

Libya,Deaths,45~50,5.47(4.23to7.07)

Libya,Deaths,50~55,10.05(8.08to12.48)

Libya,Deaths,55~60,17.52(14.5to21.18)

Libya,Deaths,60~65,38.36(32.68to45.02)

Libya,Deaths,65~70,65.47(56.44to75.94)

Libya,Deaths,70~75,111.67(96.7to128.96)

Libya,Deaths,75~80,198.87(168.85to234.22)

Libya,Deaths,80~85,314.27(265.17to372.46)

Libya,Deaths,85~90,537.19(449.17to642.44)

Libya,Deaths,90~95,757.96(617.36to930.59)

Libya,DALYs,20~25,57.15(52.63to62.06)

Libya,DALYs,25~30,76.66(71.29to82.44)

Libya,DALYs,30~35,111.33(104.37to118.74)

Libya,DALYs,35~40,149.4(140.83to158.49)

Libya,DALYs,40~45,211.86(200.78to223.56)

Libya,DALYs,45~50,348.53(332.18to365.69)

Libya,DALYs,50~55,563.46(539.68to588.28)

Libya,DALYs,55~60,849.68(816.46to884.26)

Libya,DALYs,60~65,1471.86(1419.16to1526.52)

Libya,DALYs,65~70,2083.36(2010.33to2159.04)

Libya,DALYs,70~75,2895.18(2791.99to3002.19)

Libya,DALYs,75~80,4090.56(3921.41to4267)

Libya,DALYs,80~85,5162.97(4929.79to5407.18)

Libya,DALYs,85~90,6996.75(6638.37to7374.47)

Libya,DALYs,90~95,8694.06(8120.78to9307.81)

Denmark,Prevalence,20~25,550.81(512.95to591.46)

Denmark,Prevalence,25~30,740.64(699.49to784.21)

Denmark,Prevalence,30~35,938.23(893.71to984.97)

Denmark,Prevalence,35~40,1132.05(1084.94to1181.21)

Denmark,Prevalence,40~45,1511.48(1459.38to1565.43)

Denmark,Prevalence,45~50,2672.09(2600.84to2745.28)

Denmark,Prevalence,50~55,4418.33(4320.73to4518.13)

Denmark,Prevalence,55~60,6183.3(6060.35to6308.75)

Denmark,Prevalence,60~65,8041.33(7890.71to8194.83)

Denmark,Prevalence,65~70,10722.84(10530.67to10918.52)

Denmark,Prevalence,70~75,14232.65(13984.41to14485.29)

Denmark,Prevalence,75~80,18457.04(18087.17to18834.47)

Denmark,Prevalence,80~85,23750.42(23247.03to24264.7)

Denmark,Prevalence,85~90,30716.59(30004.16to31445.94)

Denmark,Prevalence,90~95,38513.61(37442.66to39615.19)

Denmark,Deaths,20~25,0.37(0.13to1.06)

Denmark,Deaths,25~30,0.42(0.18to0.98)

Denmark,Deaths,30~35,0.57(0.29to1.1)

Denmark,Deaths,35~40,0.98(0.61to1.58)

Denmark,Deaths,40~45,2.05(1.5to2.81)

Denmark,Deaths,45~50,4.72(3.85to5.78)

Denmark,Deaths,50~55,11.81(10.35to13.47)

Denmark,Deaths,55~60,26.53(24.16to29.14)

Denmark,Deaths,60~65,55.07(51.14to59.29)

Denmark,Deaths,65~70,98.61(92.39to105.25)

Denmark,Deaths,70~75,172.14(161.95to182.96)

Denmark,Deaths,75~80,291.18(272.38to311.27)

Denmark,Deaths,80~85,432.33(404.14to462.49)

Denmark,Deaths,85~90,599.88(559.49to643.18)

Denmark,Deaths,90~95,794.66(736.11to857.88)

Denmark,DALYs,20~25,48.61(35.63to66.33)

Denmark,DALYs,25~30,58.34(45.31to75.11)

Denmark,DALYs,30~35,72.25(58.54to89.19)

Denmark,DALYs,35~40,97.83(82.42to116.12)

Denmark,DALYs,40~45,158.6(139.4to180.44)

Denmark,DALYs,45~50,310.58(283.24to340.55)

Denmark,DALYs,50~55,630.99(589.74to675.12)

Denmark,DALYs,55~60,1146.68(1086.27to1210.44)

Denmark,DALYs,60~65,1940.98(1852.73to2033.43)

Denmark,DALYs,65~70,2886.37(2764.59to3013.5)

Denmark,DALYs,70~75,4107.17(3939.8to4281.64)

Denmark,DALYs,75~80,5528.44(5277.88to5790.89)

Denmark,DALYs,80~85,6474.82(6167.93to6796.96)

Denmark,DALYs,85~90,7241.05(6865.69to7636.93)

Denmark,DALYs,90~95,8430.8(7901.65to8995.38)

Taiwan_(Province_of_China),Prevalence,20~25,335.57(326.32to345.08)

Taiwan_(Province_of_China),Prevalence,25~30,444.57(434.33to455.04)

Taiwan_(Province_of_China),Prevalence,30~35,570.1(558.82to581.6)

Taiwan_(Province_of_China),Prevalence,35~40,714.06(701.67to726.66)

Taiwan_(Province_of_China),Prevalence,40~45,935.79(921.79to950)

Taiwan_(Province_of_China),Prevalence,45~50,1369.22(1351.37to1387.3)

Taiwan_(Province_of_China),Prevalence,50~55,2017.56(1994.56to2040.83)

Taiwan_(Province_of_China),Prevalence,55~60,2878.01(2848.52to2907.8)

Taiwan_(Province_of_China),Prevalence,60~65,4047.23(4008.89to4085.94)

Taiwan_(Province_of_China),Prevalence,65~70,5692.86(5641.2to5744.99)

Taiwan_(Province_of_China),Prevalence,70~75,7954.77(7882.24to8027.98)

Taiwan_(Province_of_China),Prevalence,75~80,10865.39(10752.46to10979.52)

Taiwan_(Province_of_China),Prevalence,80~85,14612.53(14454.06to14772.73)

Taiwan_(Province_of_China),Prevalence,85~90,19230.73(19001.39to19462.84)

Taiwan_(Province_of_China),Prevalence,90~95,24034.8(23689.23to24385.41)

Taiwan_(Province_of_China),Deaths,20~25,0.15(0.07to0.32)

Taiwan_(Province_of_China),Deaths,25~30,0.2(0.11to0.36)

Taiwan_(Province_of_China),Deaths,30~35,0.33(0.22to0.51)

Taiwan_(Province_of_China),Deaths,35~40,0.52(0.38to0.71)

Taiwan_(Province_of_China),Deaths,40~45,0.92(0.73to1.16)

Taiwan_(Province_of_China),Deaths,45~50,1.6(1.34to1.91)

Taiwan_(Province_of_China),Deaths,50~55,3.07(2.69to3.51)

Taiwan_(Province_of_China),Deaths,55~60,5.7(5.14to6.33)

Taiwan_(Province_of_China),Deaths,60~65,13.23(12.23to14.31)

Taiwan_(Province_of_China),Deaths,65~70,26.01(24.32to27.83)

Taiwan_(Province_of_China),Deaths,70~75,56.65(53.21to60.31)

Taiwan_(Province_of_China),Deaths,75~80,109.74(102.56to117.43)

Taiwan_(Province_of_China),Deaths,80~85,209.09(195.49to223.64)

Taiwan_(Province_of_China),Deaths,85~90,363.73(339.78to389.36)

Taiwan_(Province_of_China),Deaths,90~95,513.29(478to551.18)

Taiwan_(Province_of_China),DALYs,20~25,38.16(33.59to43.36)

Taiwan_(Province_of_China),DALYs,25~30,49.17(44.27to54.62)

Taiwan_(Province_of_China),DALYs,30~35,65.2(59.77to71.12)

Taiwan_(Province_of_China),DALYs,35~40,83.57(77.6to90)

Taiwan_(Province_of_China),DALYs,40~45,116.15(109.18to123.56)

Taiwan_(Province_of_China),DALYs,45~50,172.91(164.05to182.25)

Taiwan_(Province_of_China),DALYs,50~55,269.01(257.3to281.25)

Taiwan_(Province_of_China),DALYs,55~60,404.72(389.44to420.6)

Taiwan_(Province_of_China),DALYs,60~65,683.8(661.39to706.98)

Taiwan_(Province_of_China),DALYs,65~70,1062.03(1029.79to1095.26)

Taiwan_(Province_of_China),DALYs,70~75,1763.53(1711.57to1817.08)

Taiwan_(Province_of_China),DALYs,75~80,2649.32(2563.49to2738.02)

Taiwan_(Province_of_China),DALYs,80~85,3871.66(3744.41to4003.24)

Taiwan_(Province_of_China),DALYs,85~90,5310.15(5126.56to5500.31)

Taiwan_(Province_of_China),DALYs,90~95,6544.15(6288.48to6810.22)

United_Arab_Emirates,Prevalence,20~25,522.55(509.61to535.81)

United_Arab_Emirates,Prevalence,25~30,734.13(719.51to749.05)

United_Arab_Emirates,Prevalence,30~35,942.23(925.49to959.28)

United_Arab_Emirates,Prevalence,35~40,1136.21(1117.52to1155.22)

United_Arab_Emirates,Prevalence,40~45,1513.83(1490.56to1537.47)

United_Arab_Emirates,Prevalence,45~50,2646.98(2608.72to2685.81)

United_Arab_Emirates,Prevalence,50~55,4212.41(4153.11to4272.57)

United_Arab_Emirates,Prevalence,55~60,5811.41(5727.74to5896.3)

United_Arab_Emirates,Prevalence,60~65,7495.2(7379.62to7612.59)

United_Arab_Emirates,Prevalence,65~70,9650.79(9489.28to9815.05)

United_Arab_Emirates,Prevalence,70~75,12789(12518.69to13065.14)

United_Arab_Emirates,Prevalence,75~80,17454.97(16927.2to17999.19)

United_Arab_Emirates,Prevalence,80~85,23838.81(22942.48to24770.15)

United_Arab_Emirates,Prevalence,85~90,31491.19(29843.32to33230.04)

United_Arab_Emirates,Prevalence,90~95,39932.95(36503.63to43684.44)

United_Arab_Emirates,Deaths,20~25,2.14(1.19to3.84)

United_Arab_Emirates,Deaths,25~30,3.1(2.03to4.72)

United_Arab_Emirates,Deaths,30~35,3.31(2.33to4.69)

United_Arab_Emirates,Deaths,35~40,4.77(3.56to6.41)

United_Arab_Emirates,Deaths,40~45,7.65(5.96to9.82)

United_Arab_Emirates,Deaths,45~50,13.53(10.92to16.78)

United_Arab_Emirates,Deaths,50~55,20.32(16.65to24.78)

United_Arab_Emirates,Deaths,55~60,31.03(25.54to37.71)

United_Arab_Emirates,Deaths,60~65,63.6(53.17to76.08)

United_Arab_Emirates,Deaths,65~70,83.13(68.63to100.7)

United_Arab_Emirates,Deaths,70~75,212.24(178.26to252.7)

United_Arab_Emirates,Deaths,75~80,304.23(241.06to383.97)

United_Arab_Emirates,Deaths,80~85,473.5(362.3to618.83)

United_Arab_Emirates,Deaths,85~90,732.54(523.95to1024.17)

United_Arab_Emirates,Deaths,90~95,1013.61(609.38to1686)

United_Arab_Emirates,DALYs,20~25,149.64(123.3to181.62)

United_Arab_Emirates,DALYs,25~30,205.67(177.02to238.96)

United_Arab_Emirates,DALYs,30~35,235.18(206.43to267.94)

United_Arab_Emirates,DALYs,35~40,301.69(268.23to339.31)

United_Arab_Emirates,DALYs,40~45,426.27(383.29to474.07)

United_Arab_Emirates,DALYs,45~50,711.41(645.7to783.81)

United_Arab_Emirates,DALYs,50~55,1015.91(925.29to1115.42)

United_Arab_Emirates,DALYs,55~60,1367.76(1243.08to1504.94)

United_Arab_Emirates,DALYs,60~65,2233.36(2031to2455.88)

United_Arab_Emirates,DALYs,65~70,2532.67(2275.07to2819.44)

United_Arab_Emirates,DALYs,70~75,5007.2(4493.75to5579.32)

United_Arab_Emirates,DALYs,75~80,5902.12(5060.8to6883.29)

United_Arab_Emirates,DALYs,80~85,7163.94(5881.64to8725.82)

United_Arab_Emirates,DALYs,85~90,8871.2(6695.14to11754.54)

United_Arab_Emirates,DALYs,90~95,10693.67(6589.27to17354.65)

Indonesia,Prevalence,20~25,480.28(473.28to487.38)

Indonesia,Prevalence,25~30,654.34(646.08to662.71)

Indonesia,Prevalence,30~35,831.66(822.19to841.24)

Indonesia,Prevalence,35~40,1008.16(997.54to1018.89)

Indonesia,Prevalence,40~45,1266.4(1254.32to1278.59)

Indonesia,Prevalence,45~50,1866.67(1850.63to1882.85)

Indonesia,Prevalence,50~55,2888.32(2865.97to2910.85)

Indonesia,Prevalence,55~60,4221.13(4190.75to4251.73)

Indonesia,Prevalence,60~65,5849.18(5808.72to5889.93)

Indonesia,Prevalence,65~70,8080(8024.87to8135.5)

Indonesia,Prevalence,70~75,11220.67(11141.32to11300.6)

Indonesia,Prevalence,75~80,15449.68(15317.73to15582.77)

Indonesia,Prevalence,80~85,21100.02(20895.06to21307)

Indonesia,Prevalence,85~90,27902.7(27561.12to28248.5)

Indonesia,Prevalence,90~95,34866.88(34218to35528.07)

Indonesia,Deaths,20~25,1.6(1.49to1.71)

Indonesia,Deaths,25~30,2.04(1.93to2.16)

Indonesia,Deaths,30~35,2.47(2.35to2.6)

Indonesia,Deaths,35~40,3.42(3.28to3.57)

Indonesia,Deaths,40~45,5.81(5.61to6.01)

Indonesia,Deaths,45~50,10.78(10.49to11.08)

Indonesia,Deaths,50~55,23.53(23.04to24.02)

Indonesia,Deaths,55~60,43.27(42.52to44.03)

Indonesia,Deaths,60~65,96.02(94.66to97.4)

Indonesia,Deaths,65~70,175.66(173.4to177.95)

Indonesia,Deaths,70~75,325.53(321.48to329.62)

Indonesia,Deaths,75~80,489.54(482.4to496.79)

Indonesia,Deaths,80~85,776.42(764.41to788.61)

Indonesia,Deaths,85~90,1176.8(1156.36to1197.6)

Indonesia,Deaths,90~95,1555.82(1519.98to1592.51)

Indonesia,DALYs,20~25,145.69(141.31to150.2)

Indonesia,DALYs,25~30,180.35(175.64to185.19)

Indonesia,DALYs,30~35,209.54(204.55to214.64)

Indonesia,DALYs,35~40,260.56(254.95to266.29)

Indonesia,DALYs,40~45,375.99(369.03to383.09)

Indonesia,DALYs,45~50,606.21(596.56to616.03)

Indonesia,DALYs,50~55,1116.63(1101.75to1131.71)

Indonesia,DALYs,55~60,1764.54(1743.56to1785.78)

Indonesia,DALYs,60~65,3203.46(3169.83to3237.44)

Indonesia,DALYs,65~70,4863.92(4814.95to4913.39)

Indonesia,DALYs,70~75,7334.62(7259.98to7410.02)

Indonesia,DALYs,75~80,8931.18(8820.64to9043.11)

Indonesia,DALYs,80~85,11168.02(11010.55to11327.75)

Indonesia,DALYs,85~90,13532.67(13291.89to13777.82)

Indonesia,DALYs,90~95,15622.09(15194.06to16062.17)

Malaysia,Prevalence,20~25,307.26(302.94to311.64)

Malaysia,Prevalence,25~30,429.39(424.19to434.66)

Malaysia,Prevalence,30~35,576.04(569.82to582.31)

Malaysia,Prevalence,35~40,740.87(733.6to748.2)

Malaysia,Prevalence,40~45,987.28(978.64to996)

Malaysia,Prevalence,45~50,1508.52(1496.81to1520.33)

Malaysia,Prevalence,50~55,2359(2342.79to2375.32)

Malaysia,Prevalence,55~60,3503.1(3481.02to3525.33)

Malaysia,Prevalence,60~65,4942.37(4912.88to4972.04)

Malaysia,Prevalence,65~70,6756.92(6717.31to6796.76)

Malaysia,Prevalence,70~75,9152.38(9096.79to9208.32)

Malaysia,Prevalence,75~80,12171.63(12081.8to12262.13)

Malaysia,Prevalence,80~85,16159.16(16025.02to16294.43)

Malaysia,Prevalence,85~90,20950.98(20741.3to21162.78)

Malaysia,Prevalence,90~95,26075.79(25719.57to26436.95)

Malaysia,Deaths,20~25,0.42(0.31to0.58)

Malaysia,Deaths,25~30,0.56(0.43to0.73)

Malaysia,Deaths,30~35,0.88(0.71to1.09)

Malaysia,Deaths,35~40,1.4(1.17to1.68)

Malaysia,Deaths,40~45,2.36(2.04to2.73)

Malaysia,Deaths,45~50,4.69(4.19to5.25)

Malaysia,Deaths,50~55,11.06(10.19to12.01)

Malaysia,Deaths,55~60,21.2(19.84to22.66)

Malaysia,Deaths,60~65,48.98(46.44to51.65)

Malaysia,Deaths,65~70,87.03(82.98to91.28)

Malaysia,Deaths,70~75,160.79(153.68to168.23)

Malaysia,Deaths,75~80,245.55(233.15to258.61)

Malaysia,Deaths,80~85,308.62(292.14to326.03)

Malaysia,Deaths,85~90,421.86(397.28to447.96)

Malaysia,Deaths,90~95,539.31(501.88to579.53)

Malaysia,DALYs,20~25,52.03(46.98to57.63)

Malaysia,DALYs,25~30,67.93(62.22to74.17)

Malaysia,DALYs,30~35,94.57(87.63to102.07)

Malaysia,DALYs,35~40,130(121.54to139.04)

Malaysia,DALYs,40~45,187.21(176.59to198.47)

Malaysia,DALYs,45~50,315.11(299.96to331.03)

Malaysia,DALYs,50~55,597.27(573.81to621.69)

Malaysia,DALYs,55~60,966.74(933.34to1001.34)

Malaysia,DALYs,60~65,1773.98(1720.38to1829.26)

Malaysia,DALYs,65~70,2607.89(2533.15to2684.83)

Malaysia,DALYs,70~75,3890.7(3779.84to4004.8)

Malaysia,DALYs,75~80,4811.1(4649.69to4978.11)

Malaysia,DALYs,80~85,4894.67(4708.78to5087.9)

Malaysia,DALYs,85~90,5399.11(5151.01to5659.15)

Malaysia,DALYs,90~95,6054.26(5676.8to6456.82)

Bermuda,Prevalence,20~25,236.05(172.68to322.68)

Bermuda,Prevalence,25~30,314.66(245.99to402.5)

Bermuda,Prevalence,30~35,417.82(341.84to510.69)

Bermuda,Prevalence,35~40,554.81(467.42to658.54)

Bermuda,Prevalence,40~45,768.53(664.66to888.63)

Bermuda,Prevalence,45~50,1175.64(1039.03to1330.22)

Bermuda,Prevalence,50~55,1847.28(1661.36to2054.02)

Bermuda,Prevalence,55~60,2817.7(2564.54to3095.84)

Bermuda,Prevalence,60~65,4099.15(3756.42to4473.14)

Bermuda,Prevalence,65~70,5762.38(5297.2to6268.41)

Bermuda,Prevalence,70~75,8083.24(7434.09to8789.08)

Bermuda,Prevalence,75~80,11307.24(10268.62to12450.92)

Bermuda,Prevalence,80~85,15721.41(14202.21to17403.11)

Bermuda,Prevalence,85~90,21215.95(18961.94to23737.89)

Bermuda,Prevalence,90~95,27468.27(23948.12to31505.84)

Bermuda,Deaths,20~25,0.14(0to301340)

Bermuda,Deaths,25~30,0.18(0to19460.08)

Bermuda,Deaths,30~35,0.18(0to3737.17)

Bermuda,Deaths,35~40,0.32(0to399.26)

Bermuda,Deaths,40~45,0.64(0to91.8)

Bermuda,Deaths,45~50,1.51(0.05to42.79)

Bermuda,Deaths,50~55,3.4(0.32to35.5)

Bermuda,Deaths,55~60,6.75(1.11to41.1)

Bermuda,Deaths,60~65,14.36(3.36to61.29)

Bermuda,Deaths,65~70,24.21(6.57to89.16)

Bermuda,Deaths,70~75,41.45(12.1to142.01)

Bermuda,Deaths,75~80,68.81(17.52to270.31)

Bermuda,Deaths,80~85,112.02(28.23to444.48)

Bermuda,Deaths,85~90,179.33(43.52to738.91)

Bermuda,Deaths,90~95,249.23(53.64to1157.99)

Bermuda,DALYs,20~25,20.36(6.37to65.05)

Bermuda,DALYs,25~30,25.62(10.13to64.8)

Bermuda,DALYs,30~35,29.74(13.73to64.42)

Bermuda,DALYs,35~40,41.76(22.21to78.53)

Bermuda,DALYs,40~45,63.19(38.04to104.97)

Bermuda,DALYs,45~50,112.77(75.77to167.82)

Bermuda,DALYs,50~55,202.51(147.47to278.08)

Bermuda,DALYs,55~60,334.63(255.73to437.87)

Bermuda,DALYs,60~65,572.12(451.88to724.35)

Bermuda,DALYs,65~70,808.55(646.55to1011.16)

Bermuda,DALYs,70~75,1131.55(907.98to1410.17)

Bermuda,DALYs,75~80,1511.49(1177.72to1939.84)

Bermuda,DALYs,80~85,1955.17(1507.11to2536.43)

Bermuda,DALYs,85~90,2506.9(1889.21to3326.55)

Bermuda,DALYs,90~95,3066.1(2183.13to4306.19)

Netherlands,Prevalence,20~25,470.69(448.96to493.46)

Netherlands,Prevalence,25~30,639.41(615.74to664)

Netherlands,Prevalence,30~35,810.21(784.84to836.4)

Netherlands,Prevalence,35~40,979.41(952.57to1007)

Netherlands,Prevalence,40~45,1301.68(1271.93to1332.12)

Netherlands,Prevalence,45~50,2241.64(2201.01to2283.02)

Netherlands,Prevalence,50~55,3757.41(3701.19to3814.5)

Netherlands,Prevalence,55~60,5406.92(5334.89to5479.92)

Netherlands,Prevalence,60~65,7186.11(7096.45to7276.9)

Netherlands,Prevalence,65~70,9623.12(9507.72to9739.93)

Netherlands,Prevalence,70~75,12854.29(12700.98to13009.45)

Netherlands,Prevalence,75~80,16948.02(16715.13to17184.16)

Netherlands,Prevalence,80~85,22240.19(21917.93to22567.19)

Netherlands,Prevalence,85~90,29082.82(28618.82to29554.34)

Netherlands,Prevalence,90~95,36004.59(35289.89to36733.77)

Netherlands,Deaths,20~25,0.32(0.17to0.61)

Netherlands,Deaths,25~30,0.34(0.2to0.59)

Netherlands,Deaths,30~35,0.45(0.29to0.69)

Netherlands,Deaths,35~40,0.72(0.52to0.99)

Netherlands,Deaths,40~45,1.47(1.18to1.84)

Netherlands,Deaths,45~50,3.45(2.98to4)

Netherlands,Deaths,50~55,7.87(7.1to8.73)

Netherlands,Deaths,55~60,16.47(15.25to17.78)

Netherlands,Deaths,60~65,35.63(33.58to37.8)

Netherlands,Deaths,65~70,68.74(65.36to72.29)

Netherlands,Deaths,70~75,132.24(126.18to138.6)

Netherlands,Deaths,75~80,235.86(223.89to248.46)

Netherlands,Deaths,80~85,375.86(356.7to396.06)

Netherlands,Deaths,85~90,610.2(578.56to643.58)

Netherlands,Deaths,90~95,904.84(854.92to957.68)

Netherlands,DALYs,20~25,44.2(34.91to55.96)

Netherlands,DALYs,25~30,52.52(43.3to63.71)

Netherlands,DALYs,30~35,64.78(55.1to76.15)

Netherlands,DALYs,35~40,84.34(73.7to96.51)

Netherlands,DALYs,40~45,133.07(119.84to147.76)

Netherlands,DALYs,45~50,259.21(239.88to280.1)

Netherlands,DALYs,50~55,494.23(465.49to524.75)

Netherlands,DALYs,55~60,843.04(802.36to885.78)

Netherlands,DALYs,60~65,1435.97(1376.37to1498.14)

Netherlands,DALYs,65~70,2242.52(2157.27to2331.14)

Netherlands,DALYs,70~75,3436.14(3309.22to3567.93)

Netherlands,DALYs,75~80,4825.11(4624.78to5034.12)

Netherlands,DALYs,80~85,6035.06(5775.01to6306.81)

Netherlands,DALYs,85~90,7750.92(7395.05to8123.92)

Netherlands,DALYs,90~95,9872.16(9342.78to10431.54)

Palestine,Prevalence,20~25,446.91(431.79to462.56)

Palestine,Prevalence,25~30,596.36(578.15to615.14)

Palestine,Prevalence,30~35,760.97(739.37to783.2)

Palestine,Prevalence,35~40,934.47(909.46to960.17)

Palestine,Prevalence,40~45,1203.72(1174.02to1234.17)

Palestine,Prevalence,45~50,1837.23(1796.4to1878.98)

Palestine,Prevalence,50~55,2883.55(2826.01to2942.27)

Palestine,Prevalence,55~60,4232.87(4154.6to4312.62)

Palestine,Prevalence,60~65,5837.26(5733.55to5942.84)

Palestine,Prevalence,65~70,7909.89(7770.97to8051.29)

Palestine,Prevalence,70~75,10784.03(10589.63to10982.01)

Palestine,Prevalence,75~80,14807.68(14489.5to15132.85)

Palestine,Prevalence,80~85,20280.53(19792.9to20780.18)

Palestine,Prevalence,85~90,27313.55(26510.47to28140.96)

Palestine,Prevalence,90~95,34845.68(33281.27to36483.63)

Palestine,Deaths,20~25,0.65(0.23to1.82)

Palestine,Deaths,25~30,0.79(0.32to1.96)

Palestine,Deaths,30~35,0.97(0.43to2.19)

Palestine,Deaths,35~40,1.31(0.64to2.69)

Palestine,Deaths,40~45,2.13(1.19to3.83)

Palestine,Deaths,45~50,3.85(2.41to6.13)

Palestine,Deaths,50~55,8.31(5.84to11.81)

Palestine,Deaths,55~60,15.26(11.42to20.38)

Palestine,Deaths,60~65,30.73(24.08to39.2)

Palestine,Deaths,65~70,50.97(40.68to63.87)

Palestine,Deaths,70~75,89.26(71.89to110.82)

Palestine,Deaths,75~80,146.12(114.72to186.11)

Palestine,Deaths,80~85,237.04(184.77to304.1)

Palestine,Deaths,85~90,374.99(287.01to489.94)

Palestine,Deaths,90~95,487.48(349.03to680.85)

Palestine,DALYs,20~25,70.97(64.59to77.99)

Palestine,DALYs,25~30,87.99(80.8to95.82)

Palestine,DALYs,30~35,106.83(98.72to115.6)

Palestine,DALYs,35~40,131.56(122.26to141.58)

Palestine,DALYs,40~45,179.59(168.23to191.71)

Palestine,DALYs,45~50,281.48(265.94to297.92)

Palestine,DALYs,50~55,494.56(471.44to518.81)

Palestine,DALYs,55~60,768.63(736.49to802.18)

Palestine,DALYs,60~65,1239.86(1192.43to1289.17)

Palestine,DALYs,65~70,1711.6(1647.72to1777.96)

Palestine,DALYs,70~75,2430.23(2339.22to2524.77)

Palestine,DALYs,75~80,3209.96(3071.29to3354.89)

Palestine,DALYs,80~85,4134.76(3939.86to4339.29)

Palestine,DALYs,85~90,5247.36(4954.75to5557.25)

Palestine,DALYs,90~95,6066.06(5570.8to6605.35)

Cambodia,Prevalence,20~25,494.24(486.59to502.01)

Cambodia,Prevalence,25~30,668.24(659.09to677.51)

Cambodia,Prevalence,30~35,850.24(839.6to861.02)

Cambodia,Prevalence,35~40,1030.97(1018.92to1043.16)

Cambodia,Prevalence,40~45,1312.21(1298.25to1326.32)

Cambodia,Prevalence,45~50,2008.87(1989.83to2028.08)

Cambodia,Prevalence,50~55,3145.27(3118.85to3171.92)

Cambodia,Prevalence,55~60,4520.99(4485.9to4556.35)

Cambodia,Prevalence,60~65,6096.73(6051.14to6142.67)

Cambodia,Prevalence,65~70,8179.43(8118.91to8240.4)

Cambodia,Prevalence,70~75,11110.21(11026.19to11194.86)

Cambodia,Prevalence,75~80,15079.61(14942.02to15218.46)

Cambodia,Prevalence,80~85,20135.96(19926.14to20347.99)

Cambodia,Prevalence,85~90,25708.52(25362.15to26059.63)

Cambodia,Prevalence,90~95,30826.57(30153.18to31515)

Cambodia,Deaths,20~25,1.17(0.84to1.64)

Cambodia,Deaths,25~30,1.49(1.12to1.98)

Cambodia,Deaths,30~35,1.86(1.45to2.4)

Cambodia,Deaths,35~40,2.66(2.15to3.29)

Cambodia,Deaths,40~45,4.76(4.02to5.63)

Cambodia,Deaths,45~50,9.08(7.97to10.35)

Cambodia,Deaths,50~55,20.57(18.69to22.65)

Cambodia,Deaths,55~60,38.96(36.04to42.12)

Cambodia,Deaths,60~65,82.11(77.01to87.55)

Cambodia,Deaths,65~70,144.68(136.5to153.35)

Cambodia,Deaths,70~75,252.37(238.75to266.76)

Cambodia,Deaths,75~80,398.04(373.12to424.63)

Cambodia,Deaths,80~85,650.17(607.4to695.95)

Cambodia,Deaths,85~90,1057.14(979.89to1140.48)

Cambodia,Deaths,90~95,1403.7(1266.78to1555.42)

Cambodia,DALYs,20~25,116.69(112.07to121.5)

Cambodia,DALYs,25~30,145.6(140.5to150.87)

Cambodia,DALYs,30~35,175.21(169.63to180.98)

Cambodia,DALYs,35~40,221.03(214.65to227.61)

Cambodia,DALYs,40~45,327.04(318.93to335.36)

Cambodia,DALYs,45~50,541.36(530.01to552.95)

Cambodia,DALYs,50~55,1018.52(1001.13to1036.21)

Cambodia,DALYs,55~60,1639.57(1615.06to1664.45)

Cambodia,DALYs,60~65,2822.27(2784.67to2860.38)

Cambodia,DALYs,65~70,4124.48(4071.83to4177.8)

Cambodia,DALYs,70~75,5869.66(5794.87to5945.42)

Cambodia,DALYs,75~80,7456.38(7342.38to7572.14)

Cambodia,DALYs,80~85,9563.81(9399.42to9731.08)

Cambodia,DALYs,85~90,12278.48(12014.44to12548.33)

Cambodia,DALYs,90~95,14087.18(13603.97to14587.56)

France,Prevalence,20~25,433.92(414.24to454.53)

France,Prevalence,25~30,595.05(572.74to618.22)

France,Prevalence,30~35,772.16(747.5to797.63)

France,Prevalence,35~40,957.69(930.97to985.18)

France,Prevalence,40~45,1252.61(1223.09to1282.84)

France,Prevalence,45~50,1968.62(1929.88to2008.13)

France,Prevalence,50~55,3085.34(3033.72to3137.83)

France,Prevalence,55~60,4420.39(4354.86to4486.91)

France,Prevalence,60~65,5952.8(5870.72to6036.02)

France,Prevalence,65~70,7917.67(7813.11to8023.64)

France,Prevalence,70~75,10627.75(10488.87to10768.47)

France,Prevalence,75~80,14489.32(14275.45to14706.39)

France,Prevalence,80~85,20413.77(20106.4to20725.85)

France,Prevalence,85~90,28938.99(28482.63to29402.67)

France,Prevalence,90~95,38577.7(37884.77to39283.3)

France,Deaths,20~25,0.15(0.08to0.28)

France,Deaths,25~30,0.17(0.1to0.28)

France,Deaths,30~35,0.21(0.14to0.32)

France,Deaths,35~40,0.39(0.29to0.52)

France,Deaths,40~45,0.83(0.68to1.01)

France,Deaths,45~50,1.83(1.6to2.09)

France,Deaths,50~55,4(3.64to4.4)

France,Deaths,55~60,7.39(6.87to7.95)

France,Deaths,60~65,14.68(13.86to15.55)

France,Deaths,65~70,23.69(22.51to24.93)

France,Deaths,70~75,40.26(38.38to42.24)

France,Deaths,75~80,64.15(60.87to67.61)

France,Deaths,80~85,105.26(99.93to110.88)

France,Deaths,85~90,188.61(179.08to198.65)

France,Deaths,90~95,286.1(271.22to301.79)

France,DALYs,20~25,23.46(18.63to29.54)

France,DALYs,25~30,29.38(24.27to35.57)

France,DALYs,30~35,36.32(30.93to42.65)

France,DALYs,35~40,49.1(43.01to56.06)

France,DALYs,40~45,75.25(67.85to83.46)

France,DALYs,45~50,134.57(124.24to145.76)

France,DALYs,50~55,241.84(226.93to257.72)

France,DALYs,55~60,378.63(358.77to399.57)

France,DALYs,60~65,607.74(580.09to636.7)

France,DALYs,65~70,830.42(794.98to867.44)

France,DALYs,70~75,1155.88(1107.68to1206.19)

France,DALYs,75~80,1495.41(1425.89to1568.31)

France,DALYs,80~85,1952.05(1860.15to2048.49)

France,DALYs,85~90,2765.32(2631.96to2905.43)

France,DALYs,90~95,3656.19(3463.64to3859.45)

Myanmar,Prevalence,20~25,583.88(573.45to594.5)

Myanmar,Prevalence,25~30,802.6(790.15to815.26)

Myanmar,Prevalence,30~35,1012.21(997.96to1026.66)

Myanmar,Prevalence,35~40,1203.89(1188.14to1219.85)

Myanmar,Prevalence,40~45,1549.44(1531.21to1567.9)

Myanmar,Prevalence,45~50,2576.2(2549.97to2602.7)

Myanmar,Prevalence,50~55,4145.02(4107.67to4182.72)

Myanmar,Prevalence,55~60,5767.52(5719.01to5816.45)

Myanmar,Prevalence,60~65,7563.23(7501.53to7625.44)

Myanmar,Prevalence,65~70,10486.2(10402.47to10570.6)

Myanmar,Prevalence,70~75,14550.18(14431.26to14670.08)

Myanmar,Prevalence,75~80,19418.45(19232.39to19606.32)

Myanmar,Prevalence,80~85,24568.49(24306.66to24833.13)

Myanmar,Prevalence,85~90,29900.07(29510.13to30295.17)

Myanmar,Prevalence,90~95,33712.28(33042.48to34395.65)

Myanmar,Deaths,20~25,4.07(3.66to4.53)

Myanmar,Deaths,25~30,5.04(4.6to5.51)

Myanmar,Deaths,30~35,5.92(5.47to6.4)

Myanmar,Deaths,35~40,7.72(7.22to8.25)

Myanmar,Deaths,40~45,12.44(11.79to13.12)

Myanmar,Deaths,45~50,22.08(21.18to23.03)

Myanmar,Deaths,50~55,51.43(49.9to53.01)

Myanmar,Deaths,55~60,98.32(95.98to100.73)

Myanmar,Deaths,60~65,202.56(198.53to206.68)

Myanmar,Deaths,65~70,351.87(345.46to358.39)

Myanmar,Deaths,70~75,598.09(587.62to608.74)

Myanmar,Deaths,75~80,898.63(880.88to916.75)

Myanmar,Deaths,80~85,1358.29(1330.52to1386.65)

Myanmar,Deaths,85~90,2001.58(1957.12to2047.05)

Myanmar,Deaths,90~95,2588.81(2516.05to2663.66)

Myanmar,DALYs,20~25,311.72(296.37to327.87)

Myanmar,DALYs,25~30,375.18(359.13to391.96)

Myanmar,DALYs,30~35,422.45(406.04to439.53)

Myanmar,DALYs,35~40,502.96(485.37to521.19)

Myanmar,DALYs,40~45,713.36(692.2to735.16)

Myanmar,DALYs,45~50,1147.44(1118.99to1176.61)

Myanmar,DALYs,50~55,2272.48(2227.75to2318.11)

Myanmar,DALYs,55~60,3722.04(3658.65to3786.53)

Myanmar,DALYs,60~65,6420.97(6323.5to6519.93)

Myanmar,DALYs,65~70,9358.02(9222.17to9495.88)

Myanmar,DALYs,70~75,13063.68(12874.06to13256.09)

Myanmar,DALYs,75~80,15768.76(15502.22to16039.89)

Myanmar,DALYs,80~85,18697.84(18350.64to19051.6)

Myanmar,DALYs,85~90,21890(21401.92to22389.21)

Myanmar,DALYs,90~95,24450.28(23643.41to25284.69)

Afghanistan,Prevalence,20~25,569.08(557.45to580.96)

Afghanistan,Prevalence,25~30,776.54(762.22to791.14)

Afghanistan,Prevalence,30~35,982.45(965.16to1000.05)

Afghanistan,Prevalence,35~40,1174.68(1154.82to1194.89)

Afghanistan,Prevalence,40~45,1520.48(1497.04to1544.29)

Afghanistan,Prevalence,45~50,2557.1(2522.86to2591.8)

Afghanistan,Prevalence,50~55,4166.19(4116.4to4216.59)

Afghanistan,Prevalence,55~60,5854.33(5788to5921.43)

Afghanistan,Prevalence,60~65,7552.94(7469.09to7637.73)

Afghanistan,Prevalence,65~70,9699.82(9593.46to9807.37)

Afghanistan,Prevalence,70~75,12631.23(12488.69to12775.4)

Afghanistan,Prevalence,75~80,16597.06(16379.43to16817.59)

Afghanistan,Prevalence,80~85,21548.52(21222.25to21879.81)

Afghanistan,Prevalence,85~90,27072.36(26531.01to27624.76)

Afghanistan,Prevalence,90~95,32407.38(31325.92to33526.18)

Afghanistan,Deaths,20~25,2.7(2.2to3.32)

Afghanistan,Deaths,25~30,3.68(3.08to4.39)

Afghanistan,Deaths,30~35,5.2(4.44to6.09)

Afghanistan,Deaths,35~40,7.62(6.63to8.75)

Afghanistan,Deaths,40~45,13.42(11.99to15.01)

Afghanistan,Deaths,45~50,24.59(22.5to26.87)

Afghanistan,Deaths,50~55,50.86(47.47to54.5)

Afghanistan,Deaths,55~60,88.16(83.11to93.52)

Afghanistan,Deaths,60~65,135.97(128.77to143.58)

Afghanistan,Deaths,65~70,202.41(192.16to213.21)

Afghanistan,Deaths,70~75,327.43(311.16to344.56)

Afghanistan,Deaths,75~80,483.64(456.93to511.92)

Afghanistan,Deaths,80~85,678.8(638.27to721.9)

Afghanistan,Deaths,85~90,969.01(901.38to1041.72)

Afghanistan,Deaths,90~95,1118.5(1003to1247.29)

Afghanistan,DALYs,20~25,219.91(212.36to227.73)

Afghanistan,DALYs,25~30,282.77(274.14to291.67)

Afghanistan,DALYs,30~35,365.26(354.83to375.99)

Afghanistan,DALYs,35~40,477.59(464.99to490.53)

Afghanistan,DALYs,40~45,735.15(718.57to752.12)

Afghanistan,DALYs,45~50,1216.61(1193.61to1240.06)

Afghanistan,DALYs,50~55,2199.74(2164.98to2235.06)

Afghanistan,DALYs,55~60,3316.8(3268.98to3365.32)

Afghanistan,DALYs,60~65,4390.3(4328.98to4452.49)

Afghanistan,DALYs,65~70,5506.97(5430.56to5584.46)

Afghanistan,DALYs,70~75,7305.89(7202.27to7411.01)

Afghanistan,DALYs,75~80,8717.6(8574.34to8863.26)

Afghanistan,DALYs,80~85,9714.5(9526.06to9906.67)

Afghanistan,DALYs,85~90,11073.45(10785.09to11369.52)

Afghanistan,DALYs,90~95,11216.59(10705.5to11752.07)

Bhutan,Prevalence,20~25,523.96(491.08to559.04)

Bhutan,Prevalence,25~30,703.48(663.8to745.54)

Bhutan,Prevalence,30~35,892.36(846.11to941.13)

Bhutan,Prevalence,35~40,1073.77(1021.35to1128.88)

Bhutan,Prevalence,40~45,1439.08(1375.83to1505.24)

Bhutan,Prevalence,45~50,2548.52(2453.93to2646.76)

Bhutan,Prevalence,50~55,4131(3995.81to4270.76)

Bhutan,Prevalence,55~60,5710.16(5536.26to5889.52)

Bhutan,Prevalence,60~65,7654.26(7431.57to7883.61)

Bhutan,Prevalence,65~70,11348.64(11033.55to11672.72)

Bhutan,Prevalence,70~75,16267.09(15815.55to16731.53)

Bhutan,Prevalence,75~80,21550.83(20849.97to22275.25)

Bhutan,Prevalence,80~85,26419.29(25474.32to27399.31)

Bhutan,Prevalence,85~90,31293.45(29938.89to32709.3)

Bhutan,Prevalence,90~95,36696.17(34381.47to39166.7)

Bhutan,Deaths,20~25,1.24(0.25to6.1)

Bhutan,Deaths,25~30,1.65(0.43to6.27)

Bhutan,Deaths,30~35,2.53(0.87to7.37)

Bhutan,Deaths,35~40,4.09(1.74to9.57)

Bhutan,Deaths,40~45,7.74(4.1to14.61)

Bhutan,Deaths,45~50,15.9(10.02to25.25)

Bhutan,Deaths,50~55,35.09(25.05to49.14)

Bhutan,Deaths,55~60,65.18(49.79to85.31)

Bhutan,Deaths,60~65,141.91(114.32to176.17)

Bhutan,Deaths,65~70,249.22(205.29to302.56)

Bhutan,Deaths,70~75,448.2(374to537.11)

Bhutan,Deaths,75~80,696.83(567.11to856.2)

Bhutan,Deaths,80~85,1213.3(985.16to1494.26)

Bhutan,Deaths,85~90,1689.32(1349.31to2115.02)

Bhutan,Deaths,90~95,2175.18(1652.08to2863.92)

Bhutan,DALYs,20~25,154.35(134.72to176.84)

Bhutan,DALYs,25~30,197.59(175.35to222.66)

Bhutan,DALYs,30~35,259.38(233.62to287.99)

Bhutan,DALYs,35~40,342.16(311.86to375.4)

Bhutan,DALYs,40~45,529.62(490.46to571.91)

Bhutan,DALYs,45~50,961.73(904.73to1022.33)

Bhutan,DALYs,50~55,1775.92(1690.15to1866.04)

Bhutan,DALYs,55~60,2767.2(2650.05to2889.53)

Bhutan,DALYs,60~65,4872.54(4692.01to5060.01)

Bhutan,DALYs,65~70,7203.16(6951.97to7463.44)

Bhutan,DALYs,70~75,10597.6(10237.97to10969.88)

Bhutan,DALYs,75~80,13245.18(12722.71to13789.1)

Bhutan,DALYs,80~85,17771.09(17031.95to18542.32)

Bhutan,DALYs,85~90,19780.06(18810.14to20799.99)

Bhutan,DALYs,90~95,22130.93(20586.67to23791.02)

Greenland,Prevalence,20~25,230.42(166.44to319)

Greenland,Prevalence,25~30,292.47(225.53to379.28)

Greenland,Prevalence,30~35,393.54(317.51to487.76)

Greenland,Prevalence,35~40,550.55(458.43to661.18)

Greenland,Prevalence,40~45,887.38(765.18to1029.11)

Greenland,Prevalence,45~50,1861.01(1662.74to2082.93)

Greenland,Prevalence,50~55,3446.89(3139.42to3784.48)

Greenland,Prevalence,55~60,5202.55(4777.25to5665.71)

Greenland,Prevalence,60~65,7524.4(6934.2to8164.84)

Greenland,Prevalence,65~70,12101.16(11177.37to13101.3)

Greenland,Prevalence,70~75,17382.66(16019.34to18862.01)

Greenland,Prevalence,75~80,22179.67(20106.73to24466.32)

Greenland,Prevalence,80~85,25217.77(22366.87to28432.05)

Greenland,Prevalence,85~90,26077.68(22107.99to30760.17)

Greenland,Prevalence,90~95,25081.22(19109.42to32919.24)

Greenland,Deaths,20~25,0.13(0to404128.03)

Greenland,Deaths,25~30,0.16(0to17447.34)

Greenland,Deaths,30~35,0.21(0to2496.87)

Greenland,Deaths,35~40,0.79(0.01to106.53)

Greenland,Deaths,40~45,2.81(0.2to40.04)

Greenland,Deaths,45~50,9.19(1.99to42.52)

Greenland,Deaths,50~55,25.33(9.12to70.35)

Greenland,Deaths,55~60,51.57(22.82to116.52)

Greenland,Deaths,60~65,102.92(50.9to208.1)

Greenland,Deaths,65~70,177.29(91.14to344.89)

Greenland,Deaths,70~75,295.71(154.53to565.87)

Greenland,Deaths,75~80,427.62(204.78to892.95)

Greenland,Deaths,80~85,524.61(232.2to1185.22)

Greenland,Deaths,85~90,628.01(239.15to1649.17)

Greenland,Deaths,90~95,732.21(198.26to2704.17)

Greenland,DALYs,20~25,24.32(8.4to70.42)

Greenland,DALYs,25~30,29.79(12.82to69.2)

Greenland,DALYs,30~35,38.38(19.21to76.69)

Greenland,DALYs,35~40,76.12(46.39to124.91)

Greenland,DALYs,40~45,185.5(133.67to257.43)

Greenland,DALYs,45~50,499.23(402.76to618.8)

Greenland,DALYs,50~55,1158.4(988.17to1357.96)

Greenland,DALYs,55~60,2020.99(1763.33to2316.3)

Greenland,DALYs,60~65,3414.59(3014.04to3868.38)

Greenland,DALYs,65~70,5019.69(4440.59to5674.32)

Greenland,DALYs,70~75,6905.95(6096.24to7823.21)

Greenland,DALYs,75~80,8069.21(6966.18to9346.9)

Greenland,DALYs,80~85,7858.93(6568.15to9403.38)

Greenland,DALYs,85~90,7459.12(5837.8to9530.73)

Greenland,DALYs,90~95,7458.85(5087.55to10935.41)

Greece,Prevalence,20~25,451.55(443.88to459.35)

Greece,Prevalence,25~30,628.48(619.73to637.35)

Greece,Prevalence,30~35,818.25(808.44to828.18)

Greece,Prevalence,35~40,1012.58(1001.89to1023.37)

Greece,Prevalence,40~45,1321.52(1309.51to1333.64)

Greece,Prevalence,45~50,2099.07(2083.3to2114.97)

Greece,Prevalence,50~55,3361.52(3340.19to3382.99)

Greece,Prevalence,55~60,4892.59(4865.19to4920.14)

Greece,Prevalence,60~65,6724.24(6689.32to6759.34)

Greece,Prevalence,65~70,9296.42(9250.21to9342.87)

Greece,Prevalence,70~75,13042.97(12979.87to13106.38)

Greece,Prevalence,75~80,18397.01(18298.63to18495.91)

Greece,Prevalence,80~85,26021.69(25880.13to26164.03)

Greece,Prevalence,85~90,35831.42(35624.02to36040.03)

Greece,Prevalence,90~95,45037.71(44722.45to45355.2)

Greece,Deaths,20~25,0.19(0.08to0.4)

Greece,Deaths,25~30,0.2(0.1to0.39)

Greece,Deaths,30~35,0.26(0.15to0.45)

Greece,Deaths,35~40,0.4(0.26to0.62)

Greece,Deaths,40~45,0.79(0.57to1.08)

Greece,Deaths,45~50,1.7(1.35to2.13)

Greece,Deaths,50~55,3.9(3.3to4.6)

Greece,Deaths,55~60,7.55(6.64to8.58)

Greece,Deaths,60~65,16.41(14.86to18.12)

Greece,Deaths,65~70,33.16(30.36to36.21)

Greece,Deaths,70~75,75.77(69.89to82.14)

Greece,Deaths,75~80,161.9(148.36to176.68)

Greece,Deaths,80~85,340.52(312.14to371.47)

Greece,Deaths,85~90,698.93(639.99to763.3)

Greece,Deaths,90~95,1303.67(1190.03to1428.16)

Greece,DALYs,20~25,35.13(29to42.54)

Greece,DALYs,25~30,44.84(38.22to52.61)

Greece,DALYs,30~35,57.49(50.11to65.96)

Greece,DALYs,35~40,74.93(66.58to84.32)

Greece,DALYs,40~45,110.63(100.33to121.98)

Greece,DALYs,45~50,193.84(179.34to209.51)

Greece,DALYs,50~55,351.7(330.27to374.51)

Greece,DALYs,55~60,558.93(529.91to589.54)

Greece,DALYs,60~65,902.41(861.2to945.59)

Greece,DALYs,65~70,1403.25(1342.58to1466.67)

Greece,DALYs,70~75,2344.74(2247.6to2446.08)

Greece,DALYs,75~80,3759.12(3589.06to3937.24)

Greece,DALYs,80~85,5919.86(5649.5to6203.15)

Greece,DALYs,85~90,9214.76(8777.28to9674.04)

Greece,DALYs,90~95,14092.35(13348.27to14877.91)

Sri_Lanka,Prevalence,20~25,378.58(373.47to383.77)

Sri_Lanka,Prevalence,25~30,516.57(510.61to522.59)

Sri_Lanka,Prevalence,30~35,674.21(667.37to681.11)

Sri_Lanka,Prevalence,35~40,843.11(835.42to850.88)

Sri_Lanka,Prevalence,40~45,1098.85(1090.09to1107.68)

Sri_Lanka,Prevalence,45~50,1673.89(1662.33to1685.52)

Sri_Lanka,Prevalence,50~55,2591.7(2576.04to2607.46)

Sri_Lanka,Prevalence,55~60,3754.72(3734.07to3775.49)

Sri_Lanka,Prevalence,60~65,5176.59(5149.69to5203.64)

Sri_Lanka,Prevalence,65~70,7018.67(6982.96to7054.56)

Sri_Lanka,Prevalence,70~75,9590.12(9540.39to9640.11)

Sri_Lanka,Prevalence,75~80,13140.82(13058.66to13223.5)

Sri_Lanka,Prevalence,80~85,18243.8(18118.13to18370.34)

Sri_Lanka,Prevalence,85~90,24563.16(24363.51to24764.44)

Sri_Lanka,Prevalence,90~95,31149.34(30801.58to31501.03)

Sri_Lanka,Deaths,20~25,2.28(1.62to3.21)

Sri_Lanka,Deaths,25~30,2.38(1.77to3.21)

Sri_Lanka,Deaths,30~35,2.8(2.18to3.6)

Sri_Lanka,Deaths,35~40,3.72(3.03to4.56)

Sri_Lanka,Deaths,40~45,5.59(4.77to6.55)

Sri_Lanka,Deaths,45~50,10.2(9.08to11.46)

Sri_Lanka,Deaths,50~55,20.88(19.21to22.71)

Sri_Lanka,Deaths,55~60,33.78(31.54to36.19)

Sri_Lanka,Deaths,60~65,79.05(74.95to83.37)

Sri_Lanka,Deaths,65~70,138.59(132.11to145.39)

Sri_Lanka,Deaths,70~75,262.27(250.71to274.36)

Sri_Lanka,Deaths,75~80,377.88(358.83to397.94)

Sri_Lanka,Deaths,80~85,644.74(611.61to679.66)

Sri_Lanka,Deaths,85~90,1013.78(958.74to1071.99)

Sri_Lanka,Deaths,90~95,1317.73(1233.69to1407.49)

Sri_Lanka,DALYs,20~25,168.19(144.71to195.48)

Sri_Lanka,DALYs,25~30,185.9(162.79to212.29)

Sri_Lanka,DALYs,30~35,216.72(192.98to243.39)

Sri_Lanka,DALYs,35~40,264.79(239.47to292.8)

Sri_Lanka,DALYs,40~45,354.13(325.8to384.93)

Sri_Lanka,DALYs,45~50,565.94(529.39to605.01)

Sri_Lanka,DALYs,50~55,987.99(936.94to1041.82)

Sri_Lanka,DALYs,55~60,1405.68(1341.44to1473)

Sri_Lanka,DALYs,60~65,2655.74(2552.53to2763.12)

Sri_Lanka,DALYs,65~70,3875.56(3732.53to4024.07)

Sri_Lanka,DALYs,70~75,5968.2(5752.24to6192.26)

Sri_Lanka,DALYs,75~80,6968.99(6671.39to7279.87)

Sri_Lanka,DALYs,80~85,9299.93(8875.06to9745.13)

Sri_Lanka,DALYs,85~90,11668.99(11057.79to12313.96)

Sri_Lanka,DALYs,90~95,13249.75(12314.7to14255.79)

Lao_People's_Democratic_Republic,Prevalence,20~25,571.45(558.41to584.81)

Lao_People's_Democratic_Republic,Prevalence,25~30,778.85(763.06to794.96)

Lao_People's_Democratic_Republic,Prevalence,30~35,983.01(964.81to1001.55)

Lao_People's_Democratic_Republic,Prevalence,35~40,1171.6(1151.22to1192.33)

Lao_People's_Democratic_Republic,Prevalence,40~45,1479.41(1455.85to1503.35)

Lao_People's_Democratic_Republic,Prevalence,45~50,2325.91(2293.21to2359.07)

Lao_People's_Democratic_Republic,Prevalence,50~55,3658.68(3612.88to3705.06)

Lao_People's_Democratic_Republic,Prevalence,55~60,5140.04(5079.58to5201.23)

Lao_People's_Democratic_Republic,Prevalence,60~65,6755.2(6677.51to6833.79)

Lao_People's_Democratic_Republic,Prevalence,65~70,8974.95(8871.83to9079.28)

Lao_People's_Democratic_Republic,Prevalence,70~75,12031.93(11889.46to12176.11)

Lao_People's_Democratic_Republic,Prevalence,75~80,15806.4(15585.91to16030.01)

Lao_People's_Democratic_Republic,Prevalence,80~85,20289.56(19969.22to20615.05)

Lao_People's_Democratic_Republic,Prevalence,85~90,25155.37(24652.07to25668.94)

Lao_People's_Democratic_Republic,Prevalence,90~95,29495.01(28578.11to30441.33)

Lao_People's_Democratic_Republic,Deaths,20~25,2.71(1.82to4.04)

Lao_People's_Democratic_Republic,Deaths,25~30,3.43(2.44to4.81)

Lao_People's_Democratic_Republic,Deaths,30~35,4.33(3.23to5.8)

Lao_People's_Democratic_Republic,Deaths,35~40,6.29(4.92to8.03)

Lao_People's_Democratic_Republic,Deaths,40~45,10.32(8.48to12.56)

Lao_People's_Democratic_Republic,Deaths,45~50,17.48(14.94to20.46)

Lao_People's_Democratic_Republic,Deaths,50~55,35.2(31.23to39.67)

Lao_People's_Democratic_Republic,Deaths,55~60,61.56(55.69to68.04)

Lao_People's_Democratic_Republic,Deaths,60~65,125.28(115.12to136.33)

Lao_People's_Democratic_Republic,Deaths,65~70,210.02(194.18to227.15)

Lao_People's_Democratic_Republic,Deaths,70~75,348.36(323.03to375.67)

Lao_People's_Democratic_Republic,Deaths,75~80,492.19(451.84to536.14)

Lao_People's_Democratic_Republic,Deaths,80~85,687.58(628.19to752.59)

Lao_People's_Democratic_Republic,Deaths,85~90,915.24(825.8to1014.36)

Lao_People's_Democratic_Republic,Deaths,90~95,1090.16(946.77to1255.26)

Lao_People's_Democratic_Republic,DALYs,20~25,223.35(210.88to236.54)

Lao_People's_Democratic_Republic,DALYs,25~30,275.53(261.94to289.82)

Lao_People's_Democratic_Republic,DALYs,30~35,329.75(315.11to345.06)

Lao_People's_Democratic_Republic,DALYs,35~40,423.24(406.44to440.73)

Lao_People's_Democratic_Republic,DALYs,40~45,603.32(582.72to624.66)

Lao_People's_Democratic_Republic,DALYs,45~50,927.16(900.12to955)

Lao_People's_Democratic_Republic,DALYs,50~55,1616.57(1577.46to1656.64)

Lao_People's_Democratic_Republic,DALYs,55~60,2444.31(2391.17to2498.62)

Lao_People's_Democratic_Republic,DALYs,60~65,4124.28(4043.18to4207.02)

Lao_People's_Democratic_Republic,DALYs,65~70,5784.14(5673.61to5896.82)

Lao_People's_Democratic_Republic,DALYs,70~75,7870.52(7719.48to8024.51)

Lao_People's_Democratic_Republic,DALYs,75~80,8993.23(8790.98to9200.14)

Lao_People's_Democratic_Republic,DALYs,80~85,9958.98(9704.68to10219.94)

Lao_People's_Democratic_Republic,DALYs,85~90,10632.92(10283.76to10993.94)

Lao_People's_Democratic_Republic,DALYs,90~95,11039.96(10462.86to11648.89)

Maldives,Prevalence,20~25,630.03(570.6to695.65)

Maldives,Prevalence,25~30,828.02(758.45to903.96)

Maldives,Prevalence,30~35,1015.41(936.86to1100.54)

Maldives,Prevalence,35~40,1189.56(1102.4to1283.62)

Maldives,Prevalence,40~45,1464.66(1364.06to1572.68)

Maldives,Prevalence,45~50,2158.6(2023.54to2302.68)

Maldives,Prevalence,50~55,3284.01(3097.24to3482.04)

Maldives,Prevalence,55~60,4655.51(4408.14to4916.75)

Maldives,Prevalence,60~65,6250.23(5930.8to6586.87)

Maldives,Prevalence,65~70,8398.85(7974.02to8846.32)

Maldives,Prevalence,70~75,11429.37(10845.76to12044.39)

Maldives,Prevalence,75~80,15563.72(14654.17to16529.73)

Maldives,Prevalence,80~85,20856.07(19554.82to22243.9)

Maldives,Prevalence,85~90,26921.52(24967.57to29028.39)

Maldives,Prevalence,90~95,32055.42(28779.65to35704.05)

Maldives,Deaths,20~25,7.53(1.19to47.56)

Maldives,Deaths,25~30,8.17(1.73to38.62)

Maldives,Deaths,30~35,9.04(2.38to34.37)

Maldives,Deaths,35~40,9.72(2.99to31.56)

Maldives,Deaths,40~45,10.56(3.71to30.04)

Maldives,Deaths,45~50,14.43(6.1to34.1)

Maldives,Deaths,50~55,23.14(11.56to46.3)

Maldives,Deaths,55~60,30.87(16.8to56.74)

Maldives,Deaths,60~65,56.88(33.9to95.42)

Maldives,Deaths,65~70,93.68(57.97to151.37)

Maldives,Deaths,70~75,151.04(95.73to238.3)

Maldives,Deaths,75~80,216.86(132.19to355.75)

Maldives,Deaths,80~85,301.38(182.18to498.55)

Maldives,Deaths,85~90,466.9(276.43to788.62)

Maldives,Deaths,90~95,624.65(342.29to1139.93)

Maldives,DALYs,20~25,439.15(360.88to534.4)

Maldives,DALYs,25~30,493.61(415.36to586.6)

Maldives,DALYs,30~35,540.89(462.7to632.29)

Maldives,DALYs,35~40,567.91(491.21to656.59)

Maldives,DALYs,40~45,604.83(529.22to691.24)

Maldives,DALYs,45~50,786.34(698.92to884.69)

Maldives,DALYs,50~55,1135.68(1024.15to1259.35)

Maldives,DALYs,55~60,1384.36(1257.41to1524.13)

Maldives,DALYs,60~65,2097.19(1921.39to2289.07)

Maldives,DALYs,65~70,2886.66(2652.73to3141.22)

Maldives,DALYs,70~75,3835.9(3530.19to4168.08)

Maldives,DALYs,75~80,4479.13(4083.21to4913.45)

Maldives,DALYs,80~85,4976.89(4510.96to5490.95)

Maldives,DALYs,85~90,6114.52(5475.9to6827.63)

Maldives,DALYs,90~95,7067.53(6099.59to8189.08)

Qatar,Prevalence,20~25,488.85(461.21to518.15)

Qatar,Prevalence,25~30,646.39(616.82to677.39)

Qatar,Prevalence,30~35,815(782.16to849.23)

Qatar,Prevalence,35~40,984.62(948.08to1022.58)

Qatar,Prevalence,40~45,1252.55(1209.35to1297.29)

Qatar,Prevalence,45~50,1883.95(1822.68to1947.28)

Qatar,Prevalence,50~55,2913.14(2822.8to3006.37)

Qatar,Prevalence,55~60,4270.2(4140.02to4404.48)

Qatar,Prevalence,60~65,6036.18(5844.66to6233.98)

Qatar,Prevalence,65~70,8551.54(8256.8to8856.8)

Qatar,Prevalence,70~75,12286.16(11803.36to12788.71)

Qatar,Prevalence,75~80,17743.76(16870.54to18662.18)

Qatar,Prevalence,80~85,25171.04(23806.51to26613.78)

Qatar,Prevalence,85~90,35153.97(32585.6to37924.79)

Qatar,Prevalence,90~95,45256.81(39559.51to51774.62)

Qatar,Deaths,20~25,0.9(0.12to6.44)

Qatar,Deaths,25~30,0.79(0.17to3.75)

Qatar,Deaths,30~35,0.83(0.22to3.06)

Qatar,Deaths,35~40,1.07(0.34to3.31)

Qatar,Deaths,40~45,1.57(0.6to4.09)

Qatar,Deaths,45~50,2.84(1.25to6.44)

Qatar,Deaths,50~55,4.98(2.46to10.1)

Qatar,Deaths,55~60,9.16(4.91to17.08)

Qatar,Deaths,60~65,19.96(11.35to35.09)

Qatar,Deaths,65~70,37.82(21.93to65.23)

Qatar,Deaths,70~75,76.47(44.73to130.73)

Qatar,Deaths,75~80,103.58(54.61to196.45)

Qatar,Deaths,80~85,139.62(71.78to271.59)

Qatar,Deaths,85~90,210.31(96.13to460.11)

Qatar,Deaths,90~95,259.95(80.37to840.8)

Qatar,DALYs,20~25,72.43(61to86)

Qatar,DALYs,25~30,82.15(71.51to94.38)

Qatar,DALYs,30~35,96.39(85.38to108.81)

Qatar,DALYs,35~40,115.68(103.58to129.2)

Qatar,DALYs,40~45,150.37(135.92to166.35)

Qatar,DALYs,45~50,233.11(212.33to255.92)

Qatar,DALYs,50~55,363.91(333.62to396.94)

Qatar,DALYs,55~60,556.89(512.55to605.07)

Qatar,DALYs,60~65,924.77(851.71to1004.09)

Qatar,DALYs,65~70,1416.71(1300.17to1543.71)

Qatar,DALYs,70~75,2284.96(2085.89to2503.03)

Qatar,DALYs,75~80,2638.9(2353.94to2958.36)

Qatar,DALYs,80~85,2971.73(2618.39to3372.74)

Qatar,DALYs,85~90,3696.05(3114.98to4385.52)

Qatar,DALYs,90~95,4115.14(3029.69to5589.48)

Portugal,Prevalence,20~25,614.2(572.23to659.26)

Portugal,Prevalence,25~30,830.33(783to880.52)

Portugal,Prevalence,30~35,1039.82(988.72to1093.57)

Portugal,Prevalence,35~40,1232.71(1179.19to1288.65)

Portugal,Prevalence,40~45,1559.39(1501.7to1619.31)

Portugal,Prevalence,45~50,2437.33(2363.31to2513.66)

Portugal,Prevalence,50~55,3694.69(3599.52to3792.39)

Portugal,Prevalence,55~60,5019.57(4903.81to5138.07)

Portugal,Prevalence,60~65,6499.51(6359.82to6642.26)

Portugal,Prevalence,65~70,8648.91(8471.36to8830.17)

Portugal,Prevalence,70~75,11587.79(11354.24to11826.14)

Portugal,Prevalence,75~80,15536.17(15189.04to15891.23)

Portugal,Prevalence,80~85,21065.18(20580.73to21561.04)

Portugal,Prevalence,85~90,28487.41(27786.14to29206.37)

Portugal,Prevalence,90~95,35910.18(34841.7to37011.42)

Portugal,Deaths,20~25,0.45(0.21to0.97)

Portugal,Deaths,25~30,0.48(0.25to0.92)

Portugal,Deaths,30~35,0.58(0.34to0.97)

Portugal,Deaths,35~40,0.95(0.65to1.38)

Portugal,Deaths,40~45,1.79(1.38to2.32)

Portugal,Deaths,45~50,3.35(2.78to4.04)

Portugal,Deaths,50~55,6.4(5.58to7.34)

Portugal,Deaths,55~60,10.95(9.83to12.19)

Portugal,Deaths,60~65,19.7(18.02to21.53)

Portugal,Deaths,65~70,32.43(29.95to35.12)

Portugal,Deaths,70~75,59.33(55.05to63.94)

Portugal,Deaths,75~80,107.36(99.16to116.23)

Portugal,Deaths,80~85,202.48(187.16to219.06)

Portugal,Deaths,85~90,376.71(348.07to407.71)

Portugal,Deaths,90~95,593.46(546.79to644.11)

Portugal,DALYs,20~25,56.08(49.82to63.13)

Portugal,DALYs,25~30,67.18(60.84to74.16)

Portugal,DALYs,30~35,79.4(73to86.36)

Portugal,DALYs,35~40,102.58(95.66to109.99)

Portugal,DALYs,40~45,149.76(141.71to158.26)

Portugal,DALYs,45~50,244.89(234.51to255.74)

Portugal,DALYs,50~55,397.79(384.1to411.97)

Portugal,DALYs,55~60,574.36(557.32to591.92)

Portugal,DALYs,60~65,837.24(814.96to860.12)

Portugal,DALYs,65~70,1142.33(1113.69to1171.7)

Portugal,DALYs,70~75,1665.1(1624.77to1706.42)

Portugal,DALYs,75~80,2363.56(2301.98to2426.79)

Portugal,DALYs,80~85,3429.47(3339.5to3521.87)

Portugal,DALYs,85~90,4989.19(4854.01to5128.15)

Portugal,DALYs,90~95,6763.93(6560.7to6973.46)

Nepal,Prevalence,20~25,502.02(494.33to509.83)

Nepal,Prevalence,25~30,687.52(678.32to696.85)

Nepal,Prevalence,30~35,877.89(867.2to888.7)

Nepal,Prevalence,35~40,1063.24(1051.26to1075.35)

Nepal,Prevalence,40~45,1549.63(1534.53to1564.88)

Nepal,Prevalence,45~50,3153.74(3129.05to3178.63)

Nepal,Prevalence,50~55,5040.49(5005.99to5075.24)

Nepal,Prevalence,55~60,6633.66(6591.27to6676.32)

Nepal,Prevalence,60~65,8804.02(8750.54to8857.84)

Nepal,Prevalence,65~70,13755.38(13676.48to13834.74)

Nepal,Prevalence,70~75,18958.36(18846.89to19070.49)

Nepal,Prevalence,75~80,23956.14(23783.76to24129.76)

Nepal,Prevalence,80~85,27665.46(27431.63to27901.28)

Nepal,Prevalence,85~90,30723.15(30381.51to31068.63)

Nepal,Prevalence,90~95,32842.62(32264.3to33431.32)

Nepal,Deaths,20~25,0.93(0.72to1.21)

Nepal,Deaths,25~30,1.22(0.99to1.51)

Nepal,Deaths,30~35,1.5(1.24to1.81)

Nepal,Deaths,35~40,3.63(3.19to4.13)

Nepal,Deaths,40~45,9.38(8.61to10.21)

Nepal,Deaths,45~50,27.45(26to28.98)

Nepal,Deaths,50~55,68.21(65.66to70.86)

Nepal,Deaths,55~60,130.28(126.39to134.29)

Nepal,Deaths,60~65,288.14(281.29to295.15)

Nepal,Deaths,65~70,507.31(496.5to518.35)

Nepal,Deaths,70~75,863.75(846.08to881.78)

Nepal,Deaths,75~80,1320.27(1288.48to1352.83)

Nepal,Deaths,80~85,2034.45(1982.78to2087.46)

Nepal,Deaths,85~90,2816.19(2735.31to2899.46)

Nepal,Deaths,90~95,3593.68(3458.54to3734.09)

Nepal,DALYs,20~25,125.86(118.38to133.81)

Nepal,DALYs,25~30,161.36(153.08to170.08)

Nepal,DALYs,30~35,192.51(183.51to201.95)

Nepal,DALYs,35~40,311.86(299.92to324.27)

Nepal,DALYs,40~45,616.14(598.29to634.52)

Nepal,DALYs,45~50,1514.42(1483to1546.52)

Nepal,DALYs,50~55,3128.54(3077.84to3180.07)

Nepal,DALYs,55~60,5041.27(4970.85to5112.69)

Nepal,DALYs,60~65,9215.52(9105.3to9327.08)

Nepal,DALYs,65~70,13737.95(13583.44to13894.21)

Nepal,DALYs,70~75,19229.52(19012.65to19448.86)

Nepal,DALYs,75~80,23552.89(23229.84to23880.43)

Nepal,DALYs,80~85,28259.15(27820.45to28704.77)

Nepal,DALYs,85~90,30983.85(30369.19to31610.95)

Nepal,DALYs,90~95,34104.41(33073.02to35167.97)

Ireland,Prevalence,20~25,457.34(436.96to478.68)

Ireland,Prevalence,25~30,622.37(599.25to646.38)

Ireland,Prevalence,30~35,802.68(777.22to828.98)

Ireland,Prevalence,35~40,989.17(961.58to1017.56)

Ireland,Prevalence,40~45,1299.18(1268.06to1331.05)

Ireland,Prevalence,45~50,2113.38(2071.69to2155.91)

Ireland,Prevalence,50~55,3468.79(3411.25to3527.29)

Ireland,Prevalence,55~60,5056.73(4981.94to5132.64)

Ireland,Prevalence,60~65,6860.42(6766.16to6955.99)

Ireland,Prevalence,65~70,9457.39(9334.26to9582.15)

Ireland,Prevalence,70~75,12994.61(12828.94to13162.42)

Ireland,Prevalence,75~80,17542.69(17286.52to17802.66)

Ireland,Prevalence,80~85,23494.88(23135.67to23859.66)

Ireland,Prevalence,85~90,31237.04(30715.73to31767.2)

Ireland,Prevalence,90~95,38977.03(38177.05to39793.77)

Ireland,Deaths,20~25,0.74(0.27to2.02)

Ireland,Deaths,25~30,0.73(0.3to1.75)

Ireland,Deaths,30~35,0.83(0.4to1.74)

Ireland,Deaths,35~40,1.18(0.66to2.1)

Ireland,Deaths,40~45,2.13(1.41to3.21)

Ireland,Deaths,45~50,4.14(3.1to5.54)

Ireland,Deaths,50~55,8.4(6.84to10.32)

Ireland,Deaths,55~60,16.2(13.9to18.9)

Ireland,Deaths,60~65,32.81(29.08to37)

Ireland,Deaths,65~70,58.4(52.61to64.82)

Ireland,Deaths,70~75,103.01(93.45to113.55)

Ireland,Deaths,75~80,175.32(157.81to194.77)

Ireland,Deaths,80~85,268.1(241.29to297.89)

Ireland,Deaths,85~90,404.89(363.75to450.69)

Ireland,Deaths,90~95,532.25(474.64to596.86)

Ireland,DALYs,20~25,65.84(50.86to85.22)

Ireland,DALYs,25~30,72.54(58.02to90.7)

Ireland,DALYs,30~35,84.27(69.59to102.06)

Ireland,DALYs,35~40,105.45(89.67to124)

Ireland,DALYs,40~45,155.24(136.36to176.74)

Ireland,DALYs,45~50,265.43(240.12to293.41)

Ireland,DALYs,50~55,464.48(429.42to502.42)

Ireland,DALYs,55~60,753.02(705.55to803.67)

Ireland,DALYs,60~65,1236.27(1168.97to1307.44)

Ireland,DALYs,65~70,1828.08(1736.82to1924.13)

Ireland,DALYs,70~75,2633.11(2506.65to2765.94)

Ireland,DALYs,75~80,3577.45(3388.69to3776.73)

Ireland,DALYs,80~85,4330.49(4095.28to4579.22)

Ireland,DALYs,85~90,5230.78(4928.51to5551.58)

Ireland,DALYs,90~95,6018.34(5610.4to6455.94)

Italy,Prevalence,20~25,405.79(395.42to416.44)

Italy,Prevalence,25~30,549.48(538.19to561)

Italy,Prevalence,30~35,712.54(700.15to725.15)

Italy,Prevalence,35~40,889.32(875.84to903)

Italy,Prevalence,40~45,1142.63(1127.86to1157.6)

Italy,Prevalence,45~50,1671.62(1653.43to1690.01)

Italy,Prevalence,50~55,2559.84(2536.41to2583.49)

Italy,Prevalence,55~60,3790.78(3760.42to3821.37)

Italy,Prevalence,60~65,5399(5359.71to5438.57)

Italy,Prevalence,65~70,7730.78(7678.08to7783.86)

Italy,Prevalence,70~75,11198.85(11125.46to11272.73)

Italy,Prevalence,75~80,16196.39(16078.73to16314.91)

Italy,Prevalence,80~85,23190.13(23018.78to23362.75)

Italy,Prevalence,85~90,32242.09(31991.48to32494.66)

Italy,Prevalence,90~95,41687.13(41316.74to42060.83)

Italy,Deaths,20~25,0.27(0.18to0.41)

Italy,Deaths,25~30,0.29(0.2to0.4)

Italy,Deaths,30~35,0.34(0.26to0.45)

Italy,Deaths,35~40,0.47(0.38to0.59)

Italy,Deaths,40~45,0.8(0.68to0.94)

Italy,Deaths,45~50,1.44(1.28to1.62)

Italy,Deaths,50~55,2.99(2.75to3.24)

Italy,Deaths,55~60,5.85(5.51to6.21)

Italy,Deaths,60~65,12.71(12.15to13.3)

Italy,Deaths,65~70,24.92(23.98to25.89)

Italy,Deaths,70~75,49.19(47.49to50.94)

Italy,Deaths,75~80,94.49(90.99to98.13)

Italy,Deaths,80~85,173.14(166.78to179.75)

Italy,Deaths,85~90,313.39(301.84to325.38)

Italy,Deaths,90~95,518.62(499.05to538.96)

Italy,DALYs,20~25,35.58(30.52to41.49)

Italy,DALYs,25~30,42.62(37.6to48.32)

Italy,DALYs,30~35,52.13(46.89to57.96)

Italy,DALYs,35~40,64.5(58.91to70.63)

Italy,DALYs,40~45,87.6(81.3to94.4)

Italy,DALYs,45~50,132.91(125.12to141.2)

Italy,DALYs,50~55,220.84(210.48to231.72)

Italy,DALYs,55~60,352.03(338.27to366.35)

Italy,DALYs,60~65,588.86(569.17to609.23)

Italy,DALYs,65~70,924.84(896.78to953.76)

Italy,DALYs,70~75,1450.9(1409.33to1493.7)

Italy,DALYs,75~80,2184.6(2116.36to2255.05)

Italy,DALYs,80~85,3121.87(3023.87to3223.06)

Italy,DALYs,85~90,4435.62(4292.74to4583.26)

Italy,DALYs,90~95,6253.92(6036.58to6479.08)

Timor-Leste,Prevalence,20~25,515.93(487.34to546.21)

Timor-Leste,Prevalence,25~30,699.1(664.97to734.99)

Timor-Leste,Prevalence,30~35,887.8(847.66to929.83)

Timor-Leste,Prevalence,35~40,1069(1023.6to1116.41)

Timor-Leste,Prevalence,40~45,1355.42(1303.85to1409.03)

Timor-Leste,Prevalence,45~50,2110.55(2039.65to2183.9)

Timor-Leste,Prevalence,50~55,3362.57(3260.16to3468.2)

Timor-Leste,Prevalence,55~60,4822.16(4687.57to4960.62)

Timor-Leste,Prevalence,60~65,6418.83(6256.16to6585.73)

Timor-Leste,Prevalence,65~70,8567.54(8354.51to8786)

Timor-Leste,Prevalence,70~75,11530.37(11233.62to11834.95)

Timor-Leste,Prevalence,75~80,15386.51(14897.29to15891.8)

Timor-Leste,Prevalence,80~85,20096.64(19343.33to20879.29)

Timor-Leste,Prevalence,85~90,25204.06(23990.91to26478.56)

Timor-Leste,Prevalence,90~95,29691.62(27430.41to32139.24)

Timor-Leste,Deaths,20~25,0.85(0.23to3.08)

Timor-Leste,Deaths,25~30,1.09(0.37to3.27)

Timor-Leste,Deaths,30~35,1.45(0.54to3.89)

Timor-Leste,Deaths,35~40,2.25(0.97to5.18)

Timor-Leste,Deaths,40~45,4.1(2.15to7.83)

Timor-Leste,Deaths,45~50,8.18(4.97to13.46)

Timor-Leste,Deaths,50~55,19.69(13.59to28.52)

Timor-Leste,Deaths,55~60,39.94(29.93to53.29)

Timor-Leste,Deaths,60~65,90.31(72.92to111.84)

Timor-Leste,Deaths,65~70,165.82(137.17to200.46)

Timor-Leste,Deaths,70~75,302.02(251.72to362.38)

Timor-Leste,Deaths,75~80,489.25(392.91to609.21)

Timor-Leste,Deaths,80~85,777(612.87to985.09)

Timor-Leste,Deaths,85~90,1169.5(892.84to1531.88)

Timor-Leste,Deaths,90~95,1553.82(1079.73to2236.07)

Timor-Leste,DALYs,20~25,98.82(87.03to112.2)

Timor-Leste,DALYs,25~30,124.22(111.17to138.81)

Timor-Leste,DALYs,30~35,154.13(139.14to170.74)

Timor-Leste,DALYs,35~40,202.39(184.44to222.08)

Timor-Leste,DALYs,40~45,301.5(278.8to326.04)

Timor-Leste,DALYs,45~50,513.98(481.43to548.72)

Timor-Leste,DALYs,50~55,1008.54(955.52to1064.49)

Timor-Leste,DALYs,55~60,1699.77(1623.32to1779.82)

Timor-Leste,DALYs,60~65,3080.89(2967.66to3198.45)

Timor-Leste,DALYs,65~70,4660.19(4498.91to4827.25)

Timor-Leste,DALYs,70~75,6879.55(6640.26to7127.45)

Timor-Leste,DALYs,75~80,8950.79(8566.4to9352.42)

Timor-Leste,DALYs,80~85,11118.2(10563.02to11702.56)

Timor-Leste,DALYs,85~90,13299.09(12456.33to14198.87)

Timor-Leste,DALYs,90~95,15327.34(13818.62to17000.78)

Syrian_Arab_Republic,Prevalence,20~25,477.18(470.65to483.8)

Syrian_Arab_Republic,Prevalence,25~30,658.57(650.48to666.75)

Syrian_Arab_Republic,Prevalence,30~35,843.54(834.03to853.16)

Syrian_Arab_Republic,Prevalence,35~40,1026.13(1015.37to1037.01)

Syrian_Arab_Republic,Prevalence,40~45,1310.66(1298.08to1323.37)

Syrian_Arab_Republic,Prevalence,45~50,2023.26(2005.93to2040.75)

Syrian_Arab_Republic,Prevalence,50~55,3212.72(3188.07to3237.56)

Syrian_Arab_Republic,Prevalence,55~60,4707.59(4674.11to4741.31)

Syrian_Arab_Republic,Prevalence,60~65,6460.33(6415.97to6505)

Syrian_Arab_Republic,Prevalence,65~70,8743.96(8683.8to8804.53)

Syrian_Arab_Republic,Prevalence,70~75,12054.26(11968.69to12140.43)

Syrian_Arab_Republic,Prevalence,75~80,16961.95(16818.03to17107.1)

Syrian_Arab_Republic,Prevalence,80~85,24156.37(23927.78to24387.15)

Syrian_Arab_Republic,Prevalence,85~90,33554.83(33151.03to33963.55)

Syrian_Arab_Republic,Prevalence,90~95,43933.27(42986.83to44900.55)

Syrian_Arab_Republic,Deaths,20~25,0.99(0.75to1.32)

Syrian_Arab_Republic,Deaths,25~30,1.41(1.1to1.81)

Syrian_Arab_Republic,Deaths,30~35,1.7(1.35to2.14)

Syrian_Arab_Republic,Deaths,35~40,2.13(1.73to2.62)

Syrian_Arab_Republic,Deaths,40~45,3.35(2.81to3.99)

Syrian_Arab_Republic,Deaths,45~50,6.23(5.4to7.19)

Syrian_Arab_Republic,Deaths,50~55,12.68(11.31to14.22)

Syrian_Arab_Republic,Deaths,55~60,22.41(20.33to24.7)

Syrian_Arab_Republic,Deaths,60~65,46.37(42.72to50.33)

Syrian_Arab_Republic,Deaths,65~70,73.25(67.81to79.13)

Syrian_Arab_Republic,Deaths,70~75,125.47(116.46to135.17)

Syrian_Arab_Republic,Deaths,75~80,200.14(183.62to218.14)

Syrian_Arab_Republic,Deaths,80~85,333.09(304.54to364.32)

Syrian_Arab_Republic,Deaths,85~90,646.34(586.44to712.36)

Syrian_Arab_Republic,Deaths,90~95,1072.42(939.16to1224.58)

Syrian_Arab_Republic,DALYs,20~25,99.55(94.69to104.65)

Syrian_Arab_Republic,DALYs,25~30,132.84(126.97to138.98)

Syrian_Arab_Republic,DALYs,30~35,154.66(148.27to161.33)

Syrian_Arab_Republic,DALYs,35~40,180.34(173.36to187.6)

Syrian_Arab_Republic,DALYs,40~45,245.46(236.94to254.28)

Syrian_Arab_Republic,DALYs,45~50,397.33(385.31to409.72)

Syrian_Arab_Republic,DALYs,50~55,687.52(669.56to705.96)

Syrian_Arab_Republic,DALYs,55~60,1043.21(1018.42to1068.61)

Syrian_Arab_Republic,DALYs,60~65,1737.06(1699.52to1775.44)

Syrian_Arab_Republic,DALYs,65~70,2312.48(2262.95to2363.08)

Syrian_Arab_Republic,DALYs,70~75,3234.3(3164.05to3306.11)

Syrian_Arab_Republic,DALYs,75~80,4189.67(4081.19to4301.04)

Syrian_Arab_Republic,DALYs,80~85,5518.88(5360.59to5681.84)

Syrian_Arab_Republic,DALYs,85~90,8322.07(8033.79to8620.7)

Syrian_Arab_Republic,DALYs,90~95,11779.14(11107.85to12490.99)

Monaco,Prevalence,20~25,471(326.79to678.85)

Monaco,Prevalence,25~30,646.98(487.86to858.01)

Monaco,Prevalence,30~35,836.41(664.55to1052.73)

Monaco,Prevalence,35~40,1028.76(847.58to1248.67)

Monaco,Prevalence,40~45,1334.18(1137.05to1565.48)

Monaco,Prevalence,45~50,2098.24(1847.41to2383.12)

Monaco,Prevalence,50~55,3334.14(3004.22to3700.29)

Monaco,Prevalence,55~60,4832.69(4412.82to5292.52)

Monaco,Prevalence,60~65,6566.57(6044.38to7133.88)

Monaco,Prevalence,65~70,8886.25(8215.29to9612.02)

Monaco,Prevalence,70~75,12081.7(11195.29to13038.3)

Monaco,Prevalence,75~80,16428.9(15087.56to17889.5)

Monaco,Prevalence,80~85,22568.72(20676.82to24633.73)

Monaco,Prevalence,85~90,30810.73(28093.94to33790.24)

Monaco,Prevalence,90~95,39725.45(35784.4to44100.54)

Monaco,Deaths,20~25,0.27(0to2502471.72)

Monaco,Deaths,25~30,0.29(0to189293.99)

Monaco,Deaths,30~35,0.35(0to17754.38)

Monaco,Deaths,35~40,0.54(0to1693.6)

Monaco,Deaths,40~45,1(0to278.9)

Monaco,Deaths,45~50,1.98(0.04to96.55)

Monaco,Deaths,50~55,4.43(0.32to61.92)

Monaco,Deaths,55~60,8.67(1.21to62.07)

Monaco,Deaths,60~65,17.32(3.72to80.58)

Monaco,Deaths,65~70,32.18(8.51to121.74)

Monaco,Deaths,70~75,60.06(17.58to205.19)

Monaco,Deaths,75~80,109.19(28.42to419.44)

Monaco,Deaths,80~85,189.27(49.23to727.62)

Monaco,Deaths,85~90,332.18(85.63to1288.62)

Monaco,Deaths,90~95,493.26(120.43to2020.27)

Monaco,DALYs,20~25,37.56(9.9to142.47)

Monaco,DALYs,25~30,44.78(15.26to131.46)

Monaco,DALYs,30~35,55(22.74to133.01)

Monaco,DALYs,35~40,70.97(34.5to145.98)

Monaco,DALYs,40~45,102.31(57.98to180.55)

Monaco,DALYs,45~50,171.29(111.09to264.11)

Monaco,DALYs,50~55,307.25(220.36to428.42)

Monaco,DALYs,55~60,492.26(373.57to648.68)

Monaco,DALYs,60~65,777.41(612.5to986.72)

Monaco,DALYs,65~70,1163.18(933.4to1449.52)

Monaco,DALYs,70~75,1718.73(1391.77to2122.51)

Monaco,DALYs,75~80,2448.5(1937.53to3094.22)

Monaco,DALYs,80~85,3319.31(2611.95to4218.25)

Monaco,DALYs,85~90,4582.66(3570.75to5881.32)

Monaco,DALYs,90~95,5908.94(4477.84to7797.43)

Niue,Prevalence,20~25,604.28(168.81to2163.06)

Niue,Prevalence,25~30,809.53(266.89to2455.49)

Niue,Prevalence,30~35,1001.28(370.81to2703.7)

Niue,Prevalence,35~40,1172.13(473.67to2900.51)

Niue,Prevalence,40~45,1407.4(642.87to3081.17)

Niue,Prevalence,45~50,1939.59(981.88to3831.42)

Niue,Prevalence,50~55,2797.36(1528.72to5118.83)

Niue,Prevalence,55~60,3862.44(2204.23to6768.1)

Niue,Prevalence,60~65,5174.52(3026.6to8846.78)

Niue,Prevalence,65~70,6925.22(4046.67to11851.4)

Niue,Prevalence,70~75,9319.61(5423.88to16013.47)

Niue,Prevalence,75~80,12309.87(6771.16to22379.17)

Niue,Prevalence,80~85,15954.34(8441.82to30152.36)

Niue,Prevalence,85~90,20271.93(9784.89to41998.54)

Niue,Prevalence,90~95,24427.69(9105.62to65532.31)

Niue,Deaths,20~25,3.01(0to1590525736.14)

Niue,Deaths,25~30,3.48(0to188061386.54)

Niue,Deaths,30~35,5.49(0to5560727.61)

Niue,Deaths,35~40,6.47(0to1059576.67)

Niue,Deaths,40~45,10.43(0to83591.85)

Niue,Deaths,45~50,16(0.01to20352.89)

Niue,Deaths,50~55,29.5(0.12to7457.4)

Niue,Deaths,55~60,43.19(0.33to5614.3)

Niue,Deaths,60~65,85.71(1.4to5253.68)

Niue,Deaths,65~70,145.45(3.03to6973.21)

Niue,Deaths,70~75,248.51(6.02to10253.09)

Niue,Deaths,75~80,404.28(7.63to21420.47)

Niue,Deaths,80~85,621.65(11.11to34774.91)

Niue,Deaths,85~90,924.95(13.75to62227.15)

Niue,Deaths,90~95,1345.58(11.48to157779.11)

Niue,DALYs,20~25,252.23(26.87to2367.39)

Niue,DALYs,25~30,286.77(38.27to2148.73)

Niue,DALYs,30~35,396.85(73.74to2135.73)

Niue,DALYs,35~40,434.93(94.69to1997.64)

Niue,DALYs,40~45,605.49(178.9to2049.25)

Niue,DALYs,45~50,832.87(296.33to2340.87)

Niue,DALYs,50~55,1333.66(560.02to3176.03)

Niue,DALYs,55~60,1728.56(771.67to3872)

Niue,DALYs,60~65,2850.9(1380.1to5889.16)

Niue,DALYs,65~70,4034.13(1981.86to8211.58)

Niue,DALYs,70~75,5629.54(2779.45to11402.15)

Niue,DALYs,75~80,7331.74(3396.59to15825.97)

Niue,DALYs,80~85,8885.55(3939.99to20038.91)

Niue,DALYs,85~90,10555.7(4215.86to26429.43)

Niue,DALYs,90~95,13266.12(4060.34to43343.62)

Sweden,Prevalence,20~25,593.58(568.07to620.25)

Sweden,Prevalence,25~30,816.77(788.22to846.36)

Sweden,Prevalence,30~35,1036.38(1005.26to1068.46)

Sweden,Prevalence,35~40,1244.52(1211.51to1278.42)

Sweden,Prevalence,40~45,1617.85(1581.86to1654.66)

Sweden,Prevalence,45~50,2713.82(2666.78to2761.69)

Sweden,Prevalence,50~55,4416.44(4353.52to4480.27)

Sweden,Prevalence,55~60,6252.47(6173.11to6332.84)

Sweden,Prevalence,60~65,8446.21(8347.13to8546.46)

Sweden,Prevalence,65~70,12265.53(12131.83to12400.69)

Sweden,Prevalence,70~75,17682.36(17497.16to17869.52)

Sweden,Prevalence,75~80,24464.95(24176.87to24756.47)

Sweden,Prevalence,80~85,32301.58(31905.67to32702.39)

Sweden,Prevalence,85~90,41648.16(41102.35to42201.22)

Sweden,Prevalence,90~95,50972.1(50198.01to51758.12)

Sweden,Deaths,20~25,0.08(0.02to0.39)

Sweden,Deaths,25~30,0.09(0.03to0.35)

Sweden,Deaths,30~35,0.14(0.05to0.4)

Sweden,Deaths,35~40,0.31(0.16to0.62)

Sweden,Deaths,40~45,0.59(0.37to0.94)

Sweden,Deaths,45~50,1.35(0.99to1.82)

Sweden,Deaths,50~55,3.63(3.01to4.38)

Sweden,Deaths,55~60,8.57(7.54to9.76)

Sweden,Deaths,60~65,20.24(18.4to22.26)

Sweden,Deaths,65~70,42.78(39.51to46.31)

Sweden,Deaths,70~75,84.62(78.63to91.06)

Sweden,Deaths,75~80,153.15(141.18to166.13)

Sweden,Deaths,80~85,231.52(213.15to251.48)

Sweden,Deaths,85~90,407.06(374.33to442.64)

Sweden,Deaths,90~95,624.63(571.86to682.27)

Sweden,DALYs,20~25,32.04(27.59to37.21)

Sweden,DALYs,25~30,42.51(37.64to48)

Sweden,DALYs,30~35,54.98(49.62to60.92)

Sweden,DALYs,35~40,71.97(65.98to78.5)

Sweden,DALYs,40~45,99.23(92.44to106.51)

Sweden,DALYs,45~50,176.94(167.89to186.48)

Sweden,DALYs,50~55,331.74(318.83to345.17)

Sweden,DALYs,55~60,560.57(542.63to579.09)

Sweden,DALYs,60~65,952.74(926.71to979.49)

Sweden,DALYs,65~70,1573.58(1534.84to1613.29)

Sweden,DALYs,70~75,2455.55(2397.63to2514.86)

Sweden,DALYs,75~80,3496.32(3404.03to3591.11)

Sweden,DALYs,80~85,4274.44(4156.66to4395.56)

Sweden,DALYs,85~90,5797.48(5628.84to5971.17)

Sweden,DALYs,90~95,7527.61(7278.87to7784.85)

American_Samoa,Prevalence,20~25,569.89(449.42to722.66)

American_Samoa,Prevalence,25~30,773.33(628.85to951.02)

American_Samoa,Prevalence,30~35,970.81(806.26to1168.94)

American_Samoa,Prevalence,35~40,1150.49(968.72to1366.35)

American_Samoa,Prevalence,40~45,1397.61(1198.19to1630.22)

American_Samoa,Prevalence,45~50,1958.17(1704.31to2249.84)

American_Samoa,Prevalence,50~55,2852.02(2512.7to3237.17)

American_Samoa,Prevalence,55~60,3973(3524.09to4479.09)

American_Samoa,Prevalence,60~65,5348.68(4751.48to6020.95)

American_Samoa,Prevalence,65~70,7223.59(6411.41to8138.64)

American_Samoa,Prevalence,70~75,9811.27(8680.64to11089.16)

American_Samoa,Prevalence,75~80,12980.19(11265.7to14955.59)

American_Samoa,Prevalence,80~85,16759.76(14318.29to19617.54)

American_Samoa,Prevalence,85~90,21068.45(17367.59to25557.93)

American_Samoa,Prevalence,90~95,25135.47(18751.05to33693.66)

American_Samoa,Deaths,20~25,2.4(0.06to91.12)

American_Samoa,Deaths,25~30,2.7(0.1to69.81)

American_Samoa,Deaths,30~35,4.99(0.43to57.65)

American_Samoa,Deaths,35~40,6.6(0.79to55.3)

American_Samoa,Deaths,40~45,10.24(1.84to57.06)

American_Samoa,Deaths,45~50,17.18(4.18to70.6)

American_Samoa,Deaths,50~55,30.16(9.37to97.13)

American_Samoa,Deaths,55~60,44.2(15.22to128.35)

American_Samoa,Deaths,60~65,89.9(35.71to226.32)

American_Samoa,Deaths,65~70,145.39(60.54to349.16)

American_Samoa,Deaths,70~75,240.33(103.35to558.86)

American_Samoa,Deaths,75~80,396.19(155.27to1010.91)

American_Samoa,Deaths,80~85,617.79(234.6to1626.88)

American_Samoa,Deaths,85~90,972.13(341.95to2763.72)

American_Samoa,Deaths,90~95,1455.93(407.1to5207)

American_Samoa,DALYs,20~25,208.08(138.03to313.69)

American_Samoa,DALYs,25~30,232.8(160.62to337.41)

American_Samoa,DALYs,30~35,364.42(268.54to494.54)

American_Samoa,DALYs,35~40,437.44(331.22to577.72)

American_Samoa,DALYs,40~45,595.09(469.59to754.15)

American_Samoa,DALYs,45~50,884.02(718.65to1087.45)

American_Samoa,DALYs,50~55,1361.63(1134.5to1634.23)

American_Samoa,DALYs,55~60,1772.44(1488.46to2110.6)

American_Samoa,DALYs,60~65,2988.43(2543.31to3511.46)

American_Samoa,DALYs,65~70,4058.53(3456.79to4765.01)

American_Samoa,DALYs,70~75,5505.11(4682.56to6472.15)

American_Samoa,DALYs,75~80,7246.79(6025.08to8716.23)

American_Samoa,DALYs,80~85,8874.53(7244.09to10871.96)

American_Samoa,DALYs,85~90,11061.89(8680.58to14096.47)

American_Samoa,DALYs,90~95,14211.67(10064.47to20067.78)

Philippines,Prevalence,20~25,493.5(477.84to509.67)

Philippines,Prevalence,25~30,683.68(664.58to703.32)

Philippines,Prevalence,30~35,879.09(856.76to902.01)

Philippines,Prevalence,35~40,1071.59(1046.35to1097.43)

Philippines,Prevalence,40~45,1378.48(1349.15to1408.44)

Philippines,Prevalence,45~50,2165.24(2124.57to2206.69)

Philippines,Prevalence,50~55,3377.05(3320.41to3434.65)

Philippines,Prevalence,55~60,4755.93(4681.01to4832.04)

Philippines,Prevalence,60~65,6302.11(6205.91to6399.79)

Philippines,Prevalence,65~70,8419.96(8291.26to8550.66)

Philippines,Prevalence,70~75,11121.96(10944.57to11302.23)

Philippines,Prevalence,75~80,14492.65(14216.64to14774.02)

Philippines,Prevalence,80~85,18487.49(18096.93to18886.47)

Philippines,Prevalence,85~90,22559.83(21985.44to23149.22)

Philippines,Prevalence,90~95,25405.02(24485.64to26358.91)

Philippines,Deaths,20~25,0.4(0.34to0.48)

Philippines,Deaths,25~30,0.66(0.57to0.76)

Philippines,Deaths,30~35,1.07(0.95to1.2)

Philippines,Deaths,35~40,1.85(1.69to2.04)

Philippines,Deaths,40~45,3.44(3.19to3.71)

Philippines,Deaths,45~50,6.9(6.5to7.32)

Philippines,Deaths,50~55,15.6(14.92to16.31)

Philippines,Deaths,55~60,31.89(30.77to33.06)

Philippines,Deaths,60~65,72.34(70.25to74.49)

Philippines,Deaths,65~70,135.29(131.73to138.94)

Philippines,Deaths,70~75,244.65(238.41to251.06)

Philippines,Deaths,75~80,381.26(369.79to393.08)

Philippines,Deaths,80~85,551.37(533.83to569.49)

Philippines,Deaths,85~90,754.63(727.96to782.29)

Philippines,Deaths,90~95,956.28(914.69to999.76)

Philippines,DALYs,20~25,73.49(69.09to78.18)

Philippines,DALYs,25~30,105.85(100.4to111.59)

Philippines,DALYs,30~35,145.46(138.81to152.43)

Philippines,DALYs,35~40,200.53(192.38to209.03)

Philippines,DALYs,40~45,297.46(286.95to308.35)

Philippines,DALYs,45~50,506.89(491.7to522.54)

Philippines,DALYs,50~55,924.14(900.93to947.94)

Philippines,DALYs,55~60,1524.36(1490.48to1559.01)

Philippines,DALYs,60~65,2679.25(2626.07to2733.49)

Philippines,DALYs,65~70,4059.01(3980.97to4138.59)

Philippines,DALYs,70~75,5871.04(5755.81to5988.59)

Philippines,DALYs,75~80,7324.46(7150.16to7503)

Philippines,DALYs,80~85,8423.55(8197.18to8656.16)

Philippines,DALYs,85~90,9312.08(9002.31to9632.5)

Philippines,DALYs,90~95,10290.25(9806.82to10797.5)

Thailand,Prevalence,20~25,594.75(574.92to615.26)

Thailand,Prevalence,25~30,794.67(772.37to817.61)

Thailand,Prevalence,30~35,985.58(961.36to1010.41)

Thailand,Prevalence,35~40,1156.06(1130.44to1182.27)

Thailand,Prevalence,40~45,1408.41(1380.75to1436.62)

Thailand,Prevalence,45~50,2019.88(1985.04to2055.34)

Thailand,Prevalence,50~55,2917.87(2872.83to2963.61)

Thailand,Prevalence,55~60,3909.88(3853.88to3966.7)

Thailand,Prevalence,60~65,5019.8(4950.71to5089.86)

Thailand,Prevalence,65~70,6515(6425.97to6605.27)

Thailand,Prevalence,70~75,8523.99(8405.89to8643.75)

Thailand,Prevalence,75~80,11004.4(10825.42to11186.35)

Thailand,Prevalence,80~85,13937.07(13691.21to14187.34)

Thailand,Prevalence,85~90,17416.17(17062.54to17777.13)

Thailand,Prevalence,90~95,21435.77(20885.8to22000.23)

Thailand,Deaths,20~25,3.06(2.11to4.43)

Thailand,Deaths,25~30,4.32(3.25to5.74)

Thailand,Deaths,30~35,6.22(4.98to7.76)

Thailand,Deaths,35~40,6.87(5.69to8.31)

Thailand,Deaths,40~45,8.46(7.24to9.89)

Thailand,Deaths,45~50,10.97(9.64to12.5)

Thailand,Deaths,50~55,16.79(15.11to18.66)

Thailand,Deaths,55~60,23.36(21.32to25.59)

Thailand,Deaths,60~65,40.47(37.39to43.8)

Thailand,Deaths,65~70,59.55(55.25to64.19)

Thailand,Deaths,70~75,92.39(86.07to99.16)

Thailand,Deaths,75~80,130.45(120.72to140.96)

Thailand,Deaths,80~85,180.95(167.25to195.77)

Thailand,Deaths,85~90,202.23(185.91to219.99)

Thailand,Deaths,90~95,243.59(221.69to267.65)

Thailand,DALYs,20~25,230.24(188.61to281.06)

Thailand,DALYs,25~30,302.79(257.21to356.45)

Thailand,DALYs,30~35,395.27(344.84to453.07)

Thailand,DALYs,35~40,424.2(375.45to479.27)

Thailand,DALYs,40~45,492.27(443to547.03)

Thailand,DALYs,45~50,616.58(562.24to676.16)

Thailand,DALYs,50~55,861.02(794.75to932.83)

Thailand,DALYs,55~60,1081.5(1004.62to1164.27)

Thailand,DALYs,60~65,1558.32(1455.54to1668.37)

Thailand,DALYs,65~70,1947.68(1820.73to2083.48)

Thailand,DALYs,70~75,2488.77(2329.05to2659.45)

Thailand,DALYs,75~80,2852.07(2646.04to3074.15)

Thailand,DALYs,80~85,3154.83(2912.22to3417.64)

Thailand,DALYs,85~90,2923.29(2661.24to3211.15)

Thailand,DALYs,90~95,3090.77(2745.77to3479.11)

Turkey,Prevalence,20~25,573.31(555.74to591.44)

Turkey,Prevalence,25~30,798.74(777.67to820.38)

Turkey,Prevalence,30~35,1018.83(994.83to1043.4)

Turkey,Prevalence,35~40,1226.95(1200.39to1254.11)

Turkey,Prevalence,40~45,1679.49(1647.51to1712.08)

Turkey,Prevalence,45~50,3110.02(3061.38to3159.45)

Turkey,Prevalence,50~55,4994.83(4926.43to5064.18)

Turkey,Prevalence,55~60,6849.63(6763.64to6936.72)

Turkey,Prevalence,60~65,8944.99(8836.77to9054.53)

Turkey,Prevalence,65~70,12289.88(12145.01to12436.49)

Turkey,Prevalence,70~75,16951.79(16750.85to17155.14)

Turkey,Prevalence,75~80,22929.06(22605.36to23257.38)

Turkey,Prevalence,80~85,30282.9(29812.32to30760.91)

Turkey,Prevalence,85~90,39228.1(38516.01to39953.35)

Turkey,Prevalence,90~95,48003.43(46843.38to49192.22)

Turkey,Deaths,20~25,1.41(1.04to1.91)

Turkey,Deaths,25~30,1.61(1.24to2.1)

Turkey,Deaths,30~35,1.88(1.49to2.36)

Turkey,Deaths,35~40,2.58(2.13to3.12)

Turkey,Deaths,40~45,4.36(3.76to5.05)

Turkey,Deaths,45~50,8.61(7.73to9.59)

Turkey,Deaths,50~55,18.54(17.12to20.07)

Turkey,Deaths,55~60,33.64(31.58to35.85)

Turkey,Deaths,60~65,65.52(62.2to69.02)

Turkey,Deaths,65~70,117.93(112.61to123.5)

Turkey,Deaths,70~75,211.19(202.21to220.58)

Turkey,Deaths,75~80,367.31(349.66to385.86)

Turkey,Deaths,80~85,573.6(545.29to603.39)

Turkey,Deaths,85~90,823.36(780.04to869.08)

Turkey,Deaths,90~95,951.04(891.78to1014.25)

Turkey,DALYs,20~25,127.32(112.9to143.57)

Turkey,DALYs,25~30,151.46(136.31to168.29)

Turkey,DALYs,30~35,174.66(158.96to191.91)

Turkey,DALYs,35~40,213.19(196.02to231.86)

Turkey,DALYs,40~45,309.32(288.35to331.82)

Turkey,DALYs,45~50,555.84(525.74to587.66)

Turkey,DALYs,50~55,1001.58(957.02to1048.21)

Turkey,DALYs,55~60,1532.71(1473.3to1594.52)

Turkey,DALYs,60~65,2435.65(2351.3to2523.03)

Turkey,DALYs,65~70,3625.82(3507.64to3747.98)

Turkey,DALYs,70~75,5256.56(5087.92to5430.79)

Turkey,DALYs,75~80,7261.07(6991.63to7540.89)

Turkey,DALYs,80~85,8964.31(8605.44to9338.13)

Turkey,DALYs,85~90,10387.5(9907.31to10890.97)

Turkey,DALYs,90~95,10627.16(9959.25to11339.87)

Yemen,Prevalence,20~25,555.45(543.88to567.27)

Yemen,Prevalence,25~30,753.99(739.93to768.32)

Yemen,Prevalence,30~35,952.58(936.26to969.19)

Yemen,Prevalence,35~40,1140.25(1121.83to1158.97)

Yemen,Prevalence,40~45,1458.28(1436.36to1480.54)

Yemen,Prevalence,45~50,2354.61(2322.84to2386.81)

Yemen,Prevalence,50~55,3786.02(3740.25to3832.34)

Yemen,Prevalence,55~60,5371.99(5311.4to5433.26)

Yemen,Prevalence,60~65,7037.86(6961.21to7115.36)

Yemen,Prevalence,65~70,9115.05(9016.44to9214.75)

Yemen,Prevalence,70~75,11966.9(11832.15to12103.18)

Yemen,Prevalence,75~80,15873.2(15658.3to16091.06)

Yemen,Prevalence,80~85,21065.85(20740.2to21396.61)

Yemen,Prevalence,85~90,27401.29(26869.74to27943.36)

Yemen,Prevalence,90~95,34064.59(33074.04to35084.81)

Yemen,Deaths,20~25,0.91(0.67to1.23)

Yemen,Deaths,25~30,1.51(1.19to1.92)

Yemen,Deaths,30~35,2.23(1.83to2.72)

Yemen,Deaths,35~40,3.52(2.98to4.16)

Yemen,Deaths,40~45,6.39(5.57to7.32)

Yemen,Deaths,45~50,11.33(10.12to12.7)

Yemen,Deaths,50~55,21.9(19.98to24.01)

Yemen,Deaths,55~60,35.92(33.17to38.89)

Yemen,Deaths,60~65,65.8(61.43to70.49)

Yemen,Deaths,65~70,106.13(99.56to113.13)

Yemen,Deaths,70~75,173.97(163.53to185.08)

Yemen,Deaths,75~80,271.86(252.99to292.13)

Yemen,Deaths,80~85,410.64(380.33to443.37)

Yemen,Deaths,85~90,582.88(534.03to636.21)

Yemen,Deaths,90~95,771.76(686.7to867.36)

Yemen,DALYs,20~25,98.5(92.64to104.73)

Yemen,DALYs,25~30,143.5(136.18to151.2)

Yemen,DALYs,30~35,189.46(180.86to198.47)

Yemen,DALYs,35~40,256.71(246.23to267.65)

Yemen,DALYs,40~45,393.64(379.52to408.29)

Yemen,DALYs,45~50,631.4(611.39to652.08)

Yemen,DALYs,50~55,1068.98(1039.56to1099.23)

Yemen,DALYs,55~60,1532.9(1494.17to1572.64)

Yemen,DALYs,60~65,2331.75(2277.3to2387.52)

Yemen,DALYs,65~70,3138.04(3066.4to3211.35)

Yemen,DALYs,70~75,4208.01(4110to4308.34)

Yemen,DALYs,75~80,5301.1(5153.15to5453.3)

Yemen,DALYs,80~85,6364.88(6160.99to6575.51)

Yemen,DALYs,85~90,7307.44(7007.42to7620.31)

Yemen,DALYs,90~95,8445.39(7916.01to9010.17)

Palau,Prevalence,20~25,559.17(382.59to817.23)

Palau,Prevalence,25~30,776.98(566.38to1065.89)

Palau,Prevalence,30~35,987.39(750.04to1299.84)

Palau,Prevalence,35~40,1181.46(919.01to1518.86)

Palau,Prevalence,40~45,1440.75(1150.45to1804.3)

Palau,Prevalence,45~50,2015.43(1647.4to2465.67)

Palau,Prevalence,50~55,2923.23(2429.99to3516.6)

Palau,Prevalence,55~60,4021.44(3374.96to4791.76)

Palau,Prevalence,60~65,5352.93(4510.8to6352.28)

Palau,Prevalence,65~70,7134.19(5984.52to8504.71)

Palau,Prevalence,70~75,9565.87(7960.74to11494.64)

Palau,Prevalence,75~80,12554.44(10075.52to15643.26)

Palau,Prevalence,80~85,16101.27(12540.52to20673.05)

Palau,Prevalence,85~90,19999.55(14691.35to27225.68)

Palau,Prevalence,90~95,23435.8(14176.29to38743.32)

Palau,Deaths,20~25,3.97(0.04to356.11)

Palau,Deaths,25~30,3.94(0.07to230.81)

Palau,Deaths,30~35,6.74(0.3to153.46)

Palau,Deaths,35~40,7.68(0.45to130.01)

Palau,Deaths,40~45,12.11(1.21to121.39)

Palau,Deaths,45~50,19.93(3to132.57)

Palau,Deaths,50~55,36.55(7.56to176.69)

Palau,Deaths,55~60,51.32(11.98to219.82)

Palau,Deaths,60~65,109.99(32.87to368.08)

Palau,Deaths,65~70,183.8(58.12to581.2)

Palau,Deaths,70~75,317.7(104.03to970.2)

Palau,Deaths,75~80,545.86(152.27to1956.73)

Palau,Deaths,80~85,927.66(247.1to3482.63)

Palau,Deaths,85~90,1720.48(424.22to6977.68)

Palau,Deaths,90~95,3031.44(548.7to16748)

Palau,DALYs,20~25,306.09(178.3to525.47)

Palau,DALYs,25~30,303.09(185.21to495.99)

Palau,DALYs,30~35,458.69(306.1to687.35)

Palau,DALYs,35~40,490.72(336.62to715.38)

Palau,DALYs,40~45,681.12(492.98to941.04)

Palau,DALYs,45~50,999.79(754.85to1324.21)

Palau,DALYs,50~55,1605.35(1252.93to2056.91)

Palau,DALYs,55~60,2011.73(1583.42to2555.89)

Palau,DALYs,60~65,3577.86(2889.48to4430.25)

Palau,DALYs,65~70,5018.25(4048.45to6220.36)

Palau,DALYs,70~75,7079.04(5685.48to8814.19)

Palau,DALYs,75~80,9659.22(7456.22to12513.11)

Palau,DALYs,80~85,12729.96(9552.51to16964.33)

Palau,DALYs,85~90,18469.55(13250to25745.23)

Palau,DALYs,90~95,27721.16(17286.86to44453.56)

Angola,Prevalence,20~25,456.6(447.76to465.6)

Angola,Prevalence,25~30,581.15(571.07to591.42)

Angola,Prevalence,30~35,726.01(714.39to737.82)

Angola,Prevalence,35~40,887.72(874.47to901.18)

Angola,Prevalence,40~45,1134.16(1118.7to1149.83)

Angola,Prevalence,45~50,1681.23(1660.43to1702.29)

Angola,Prevalence,50~55,2583.27(2554.23to2612.65)

Angola,Prevalence,55~60,3746.13(3706.41to3786.27)

Angola,Prevalence,60~65,5038.4(4985.99to5091.37)

Angola,Prevalence,65~70,6486.78(6418.08to6556.22)

Angola,Prevalence,70~75,8237.94(8144.28to8332.67)

Angola,Prevalence,75~80,10394.65(10248.8to10542.59)

Angola,Prevalence,80~85,13084.18(12861.68to13310.52)

Angola,Prevalence,85~90,15856.45(15481.81to16240.16)

Angola,Prevalence,90~95,18148(17422.99to18903.17)

Angola,Deaths,20~25,0.91(0.62to1.33)

Angola,Deaths,25~30,0.99(0.71to1.4)

Angola,Deaths,30~35,1.74(1.33to2.27)

Angola,Deaths,35~40,2.28(1.81to2.87)

Angola,Deaths,40~45,4.95(4.18to5.86)

Angola,Deaths,45~50,12.44(11.04to14.02)

Angola,Deaths,50~55,20.06(18.15to22.17)

Angola,Deaths,55~60,38.75(35.66to42.1)

Angola,Deaths,60~65,71.06(66.07to76.43)

Angola,Deaths,65~70,121.91(113.89to130.5)

Angola,Deaths,70~75,190.1(177.75to203.31)

Angola,Deaths,75~80,235.62(217.51to255.23)

Angola,Deaths,80~85,326.53(298.98to356.62)

Angola,Deaths,85~90,395.47(354.18to441.58)

Angola,Deaths,90~95,552.2(472.13to645.84)

Angola,DALYs,20~25,112.12(107.39to117.06)

Angola,DALYs,25~30,129.08(124.19to134.17)

Angola,DALYs,30~35,178.88(172.92to185.05)

Angola,DALYs,35~40,215.76(209.18to222.55)

Angola,DALYs,40~45,349.05(340.15to358.17)

Angola,DALYs,45~50,689.1(674.91to703.6)

Angola,DALYs,50~55,1008.68(990.17to1027.53)

Angola,DALYs,55~60,1645.84(1618.79to1673.33)

Angola,DALYs,60~65,2521.68(2482.92to2561.04)

Angola,DALYs,65~70,3578.5(3524.47to3633.35)

Angola,DALYs,70~75,4587.81(4515.79to4660.97)

Angola,DALYs,75~80,4697.25(4605.19to4791.14)

Angola,DALYs,80~85,5195.27(5071.7to5321.85)

Angola,DALYs,85~90,5141.81(4965.51to5324.37)

Angola,DALYs,90~95,6118.7(5779.36to6477.96)

Congo,Prevalence,20~25,382.39(369.13to396.12)

Congo,Prevalence,25~30,502.26(486.69to518.33)

Congo,Prevalence,30~35,645.99(627.59to664.92)

Congo,Prevalence,35~40,808.74(787.22to830.85)

Congo,Prevalence,40~45,1055.22(1029.49to1081.6)

Congo,Prevalence,45~50,1594.38(1558.96to1630.61)

Congo,Prevalence,50~55,2490.08(2439.55to2541.66)

Congo,Prevalence,55~60,3670.48(3600.8to3741.51)

Congo,Prevalence,60~65,5022.27(4929.88to5116.39)

Congo,Prevalence,65~70,6581.44(6460.51to6704.64)

Congo,Prevalence,70~75,8511.66(8347.35to8679.21)

Congo,Prevalence,75~80,10987.94(10732.61to11249.34)

Congo,Prevalence,80~85,14174.24(13785.35to14574.1)

Congo,Prevalence,85~90,17582.67(16918.66to18272.75)

Congo,Prevalence,90~95,20285.01(18895.23to21777.01)

Congo,Deaths,20~25,1.05(0.51to2.17)

Congo,Deaths,25~30,1.24(0.66to2.33)

Congo,Deaths,30~35,2.33(1.45to3.74)

Congo,Deaths,35~40,3.14(2.08to4.73)

Congo,Deaths,40~45,6.7(4.94to9.07)

Congo,Deaths,45~50,16.68(13.39to20.77)

Congo,Deaths,50~55,26.3(21.78to31.75)

Congo,Deaths,55~60,49.32(42.15to57.7)

Congo,Deaths,60~65,87.63(76.35to100.57)

Congo,Deaths,65~70,145.9(128.37to165.81)

Congo,Deaths,70~75,228.29(201.3to258.9)

Congo,Deaths,75~80,300.75(260.24to347.57)

Congo,Deaths,80~85,415.47(354.88to486.42)

Congo,Deaths,85~90,546.51(451.21to661.95)

Congo,Deaths,90~95,783.62(590.35to1040.17)

Congo,DALYs,20~25,116.59(108.83to124.92)

Congo,DALYs,25~30,139.1(130.85to147.87)

Congo,DALYs,30~35,206.51(196.04to217.54)

Congo,DALYs,35~40,255.08(243.24to267.49)

Congo,DALYs,40~45,426.53(410.05to443.67)

Congo,DALYs,45~50,864.4(837.43to892.24)

Congo,DALYs,50~55,1242.48(1207.25to1278.75)

Congo,DALYs,55~60,1998.65(1947.93to2050.69)

Congo,DALYs,60~65,3003.52(2932.56to3076.2)

Congo,DALYs,65~70,4169.6(4073.7to4267.76)

Congo,DALYs,70~75,5364.85(5237.9to5494.88)

Congo,DALYs,75~80,5789.96(5625.71to5959)

Congo,DALYs,80~85,6386.07(6170.57to6609.09)

Congo,DALYs,85~90,6783.72(6463.56to7119.73)

Congo,DALYs,90~95,8294.59(7627.4to9020.14)

Kiribati,Prevalence,20~25,557.05(467.8to663.32)

Kiribati,Prevalence,25~30,770.36(660.47to898.53)

Kiribati,Prevalence,30~35,975.85(848.21to1122.7)

Kiribati,Prevalence,35~40,1169.13(1024.79to1333.79)

Kiribati,Prevalence,40~45,1454.26(1288.84to1640.91)

Kiribati,Prevalence,45~50,2177.01(1952.4to2427.45)

Kiribati,Prevalence,50~55,3373.91(3056.73to3724)

Kiribati,Prevalence,55~60,4713(4293to5174.09)

Kiribati,Prevalence,60~65,6221.25(5675.08to6819.98)

Kiribati,Prevalence,65~70,8414.33(7653.72to9250.53)

Kiribati,Prevalence,70~75,11514.34(10418.26to12725.73)

Kiribati,Prevalence,75~80,15168.71(13482.24to17066.14)

Kiribati,Prevalence,80~85,19339.72(16824.73to22230.66)

Kiribati,Prevalence,85~90,24194.79(20038.7to29212.88)

Kiribati,Prevalence,90~95,28867.7(20796.09to40072.14)

Kiribati,Deaths,20~25,5.66(0.93to34.4)

Kiribati,Deaths,25~30,6.17(1.19to31.88)

Kiribati,Deaths,30~35,9.59(2.45to37.48)

Kiribati,Deaths,35~40,11.78(3.43to40.53)

Kiribati,Deaths,40~45,19.72(7.14to54.43)

Kiribati,Deaths,45~50,29.82(12.39to71.75)

Kiribati,Deaths,50~55,55.46(26.78to114.83)

Kiribati,Deaths,55~60,81.35(41.99to157.61)

Kiribati,Deaths,60~65,160.27(89.99to285.43)

Kiribati,Deaths,65~70,265.4(151.78to464.07)

Kiribati,Deaths,70~75,428.34(245.22to748.18)

Kiribati,Deaths,75~80,697.15(374.44to1297.98)

Kiribati,Deaths,80~85,1101.26(567.45to2137.23)

Kiribati,Deaths,85~90,1698.96(788.81to3659.24)

Kiribati,Deaths,90~95,2550.52(875.8to7427.68)

Kiribati,DALYs,20~25,420.69(336.21to526.4)

Kiribati,DALYs,25~30,446.12(362.55to548.95)

Kiribati,DALYs,30~35,625.7(521.89to750.16)

Kiribati,DALYs,35~40,709.27(598.23to840.92)

Kiribati,DALYs,40~45,1047.24(904.11to1213.02)

Kiribati,DALYs,45~50,1441.32(1261.7to1646.52)

Kiribati,DALYs,50~55,2366.02(2104.87to2659.57)

Kiribati,DALYs,55~60,3074.42(2750.35to3436.69)

Kiribati,DALYs,60~65,5095.96(4598.2to5647.61)

Kiribati,DALYs,65~70,7090.53(6388.34to7869.9)

Kiribati,DALYs,70~75,9420.57(8446.17to10507.38)

Kiribati,DALYs,75~80,12275.38(10819.79to13926.81)

Kiribati,DALYs,80~85,15206.37(13137.01to17601.7)

Kiribati,DALYs,85~90,18599.99(15325.3to22574.4)

Kiribati,DALYs,90~95,23956.03(17444.12to32898.85)

Micronesia_(Federated_States_of),Prevalence,20~25,569.63(483.91to670.53)

Micronesia_(Federated_States_of),Prevalence,25~30,794.95(686.89to920.01)

Micronesia_(Federated_States_of),Prevalence,30~35,1009.85(883.3to1154.52)

Micronesia_(Federated_States_of),Prevalence,35~40,1207.87(1065.12to1369.76)

Micronesia_(Federated_States_of),Prevalence,40~45,1496.54(1337.69to1674.26)

Micronesia_(Federated_States_of),Prevalence,45~50,2211.92(1999.38to2447.06)

Micronesia_(Federated_States_of),Prevalence,50~55,3348.7(3048.74to3678.18)

Micronesia_(Federated_States_of),Prevalence,55~60,4606.14(4217to5031.2)

Micronesia_(Federated_States_of),Prevalence,60~65,6003.76(5508.01to6544.14)

Micronesia_(Federated_States_of),Prevalence,65~70,8004.96(7306.42to8770.28)

Micronesia_(Federated_States_of),Prevalence,70~75,10762.3(9764.8to11861.68)

Micronesia_(Federated_States_of),Prevalence,75~80,14030.01(12524.62to15716.35)

Micronesia_(Federated_States_of),Prevalence,80~85,17657.68(15557.08to20041.91)

Micronesia_(Federated_States_of),Prevalence,85~90,21584.29(18424.91to25285.41)

Micronesia_(Federated_States_of),Prevalence,90~95,24556.62(19027.74to31692.01)

Micronesia_(Federated_States_of),Deaths,20~25,5.22(0.77to35.15)

Micronesia_(Federated_States_of),Deaths,25~30,6.22(1.13to34.37)

Micronesia_(Federated_States_of),Deaths,30~35,10.09(2.54to40.15)

Micronesia_(Federated_States_of),Deaths,35~40,12.1(3.49to42.01)

Micronesia_(Federated_States_of),Deaths,40~45,20.1(7.59to53.22)

Micronesia_(Federated_States_of),Deaths,45~50,29.47(12.61to68.88)

Micronesia_(Federated_States_of),Deaths,50~55,52.38(25.25to108.66)

Micronesia_(Federated_States_of),Deaths,55~60,72.97(37.51to141.93)

Micronesia_(Federated_States_of),Deaths,60~65,137.65(77.28to245.18)

Micronesia_(Federated_States_of),Deaths,65~70,220.87(124.98to390.31)

Micronesia_(Federated_States_of),Deaths,70~75,344.76(194.78to610.22)

Micronesia_(Federated_States_of),Deaths,75~80,526.79(280.05to990.91)

Micronesia_(Federated_States_of),Deaths,80~85,796.69(413.81to1533.83)

Micronesia_(Federated_States_of),Deaths,85~90,1196.63(582.29to2459.12)

Micronesia_(Federated_States_of),Deaths,90~95,1778.1(708.31to4463.63)

Micronesia_(Federated_States_of),DALYs,20~25,396.22(315.01to498.37)

Micronesia_(Federated_States_of),DALYs,25~30,456.73(370.09to563.66)

Micronesia_(Federated_States_of),DALYs,30~35,660.59(551.29to791.55)

Micronesia_(Federated_States_of),DALYs,35~40,733.11(618.92to868.37)

Micronesia_(Federated_States_of),DALYs,40~45,1071.79(932.16to1232.34)

Micronesia_(Federated_States_of),DALYs,45~50,1433.58(1261.88to1628.65)

Micronesia_(Federated_States_of),DALYs,50~55,2251.83(2005.16to2528.85)

Micronesia_(Federated_States_of),DALYs,55~60,2786.33(2494.11to3112.78)

Micronesia_(Federated_States_of),DALYs,60~65,4428.77(4000.58to4902.78)

Micronesia_(Federated_States_of),DALYs,65~70,5973.83(5376.28to6637.79)

Micronesia_(Federated_States_of),DALYs,70~75,7680.31(6871.92to8583.79)

Micronesia_(Federated_States_of),DALYs,75~80,9409.11(8275.62to10697.86)

Micronesia_(Federated_States_of),DALYs,80~85,11173.2(9689.26to12884.41)

Micronesia_(Federated_States_of),DALYs,85~90,13324.77(11189.19to15867.95)

Micronesia_(Federated_States_of),DALYs,90~95,16962.67(13071.73to22011.78)

Cook_Islands,Prevalence,20~25,526.27(346.96to798.25)

Cook_Islands,Prevalence,25~30,695.82(484.55to999.2)

Cook_Islands,Prevalence,30~35,867.06(626.79to1199.42)

Cook_Islands,Prevalence,35~40,1030.23(765.02to1387.39)

Cook_Islands,Prevalence,40~45,1258.87(970.01to1633.76)

Cook_Islands,Prevalence,45~50,1746.77(1388.7to2197.16)

Cook_Islands,Prevalence,50~55,2509.22(2057.16to3060.6)

Cook_Islands,Prevalence,55~60,3492.13(2910.63to4189.8)

Cook_Islands,Prevalence,60~65,4752.66(3995.97to5652.64)

Cook_Islands,Prevalence,65~70,6458.02(5449.56to7653.1)

Cook_Islands,Prevalence,70~75,8835.61(7443.71to10487.78)

Cook_Islands,Prevalence,75~80,11981.63(9869.6to14545.61)

Cook_Islands,Prevalence,80~85,16131.08(13083.1to19889.16)

Cook_Islands,Prevalence,85~90,21226.75(16690.75to26995.49)

Cook_Islands,Prevalence,90~95,26685.35(19210.48to37068.72)

Cook_Islands,Deaths,20~25,1.47(0to11516)

Cook_Islands,Deaths,25~30,1.59(0to4908.02)

Cook_Islands,Deaths,30~35,2.48(0to1386.14)

Cook_Islands,Deaths,35~40,3.37(0.02to677.52)

Cook_Islands,Deaths,40~45,5.35(0.09to311.32)

Cook_Islands,Deaths,45~50,8.38(0.32to219.56)

Cook_Islands,Deaths,50~55,14.78(1.22to179.2)

Cook_Islands,Deaths,55~60,20.92(2.36to185.22)

Cook_Islands,Deaths,60~65,40.32(6.45to252.23)

Cook_Islands,Deaths,65~70,64.81(11.87to353.83)

Cook_Islands,Deaths,70~75,107.76(21.18to548.27)

Cook_Islands,Deaths,75~80,172.4(29.54to1006.22)

Cook_Islands,Deaths,80~85,269(44.8to1615.09)

Cook_Islands,Deaths,85~90,414.2(64.25to2670.08)

Cook_Islands,Deaths,90~95,646.21(81.19to5143.61)

Cook_Islands,DALYs,20~25,141.57(57.19to350.45)

Cook_Islands,DALYs,25~30,158.96(70.52to358.33)

Cook_Islands,DALYs,30~35,213.16(106.36to427.22)

Cook_Islands,DALYs,35~40,259.38(139.44to482.48)

Cook_Islands,DALYs,40~45,351.64(210.73to586.77)

Cook_Islands,DALYs,45~50,491.3(315.97to763.92)

Cook_Islands,DALYs,50~55,750.19(520.25to1081.73)

Cook_Islands,DALYs,55~60,957.13(682.61to1342.05)

Cook_Islands,DALYs,60~65,1515.1(1116.46to2056.08)

Cook_Islands,DALYs,65~70,2043.73(1520.34to2747.3)

Cook_Islands,DALYs,70~75,2791.6(2080.12to3746.43)

Cook_Islands,DALYs,75~80,3589.78(2589.48to4976.51)

Cook_Islands,DALYs,80~85,4444.21(3138.49to6293.15)

Cook_Islands,DALYs,85~90,5473.19(3707.18to8080.5)

Cook_Islands,DALYs,90~95,7306.91(4455.45to11983.28)

Malta,Prevalence,20~25,474.9(433.4to520.37)

Malta,Prevalence,25~30,644.4(596.98to695.58)

Malta,Prevalence,30~35,826.3(774.22to881.89)

Malta,Prevalence,35~40,1012.15(956.3to1071.26)

Malta,Prevalence,40~45,1298.98(1238.41to1362.51)

Malta,Prevalence,45~50,1981.59(1905.88to2060.31)

Malta,Prevalence,50~55,3054.69(2955.83to3156.86)

Malta,Prevalence,55~60,4338.47(4214.66to4465.92)

Malta,Prevalence,60~65,5856.99(5703.17to6014.96)

Malta,Prevalence,65~70,7922.52(7724.94to8125.14)

Malta,Prevalence,70~75,10639.32(10380.53to10904.55)

Malta,Prevalence,75~80,14178.52(13774.38to14594.51)

Malta,Prevalence,80~85,19036.61(18462.88to19628.17)

Malta,Prevalence,85~90,25683.41(24836.48to26559.22)

Malta,Prevalence,90~95,33148.86(31818.84to34534.48)

Malta,Deaths,20~25,0.18(0to58.78)

Malta,Deaths,25~30,0.2(0to23.15)

Malta,Deaths,30~35,0.23(0to13.87)

Malta,Deaths,35~40,0.36(0.02to7.62)

Malta,Deaths,40~45,0.65(0.08to5.64)

Malta,Deaths,45~50,1.54(0.39to6.04)

Malta,Deaths,50~55,3.73(1.54to9.05)

Malta,Deaths,55~60,7.54(3.99to14.24)

Malta,Deaths,60~65,16(9.88to25.91)

Malta,Deaths,65~70,30.29(20.09to45.66)

Malta,Deaths,70~75,54.09(37.09to78.89)

Malta,Deaths,75~80,83.89(55to127.94)

Malta,Deaths,80~85,127.71(83.52to195.27)

Malta,Deaths,85~90,169.12(109.1to262.16)

Malta,Deaths,90~95,192.15(117.9to313.17)

Malta,DALYs,20~25,32.01(21.94to46.69)

Malta,DALYs,25~30,40.65(29.65to55.73)

Malta,DALYs,30~35,49.67(37.92to65.06)

Malta,DALYs,35~40,62.72(49.92to78.82)

Malta,DALYs,40~45,86.42(71.8to104.02)

Malta,DALYs,45~50,148.79(129.33to171.18)

Malta,DALYs,50~55,267.6(240.15to298.18)

Malta,DALYs,55~60,429.56(393.14to469.34)

Malta,DALYs,60~65,701.71(650.67to756.75)

Malta,DALYs,65~70,1072.19(1000.87to1148.58)

Malta,DALYs,70~75,1544.51(1446.23to1649.46)

Malta,DALYs,75~80,1948.32(1806.72to2101.03)

Malta,DALYs,80~85,2378(2198.12to2572.59)

Malta,DALYs,85~90,2628.36(2411.08to2865.23)

Malta,DALYs,90~95,2757.23(2476.39to3069.92)

Norway,Prevalence,20~25,444.18(432.53to456.14)

Norway,Prevalence,25~30,616.68(603.53to630.11)

Norway,Prevalence,30~35,800.88(786.29to815.74)

Norway,Prevalence,35~40,990.4(974.53to1006.52)

Norway,Prevalence,40~45,1304.09(1286.31to1322.1)

Norway,Prevalence,45~50,2147.69(2123.97to2171.67)

Norway,Prevalence,50~55,3610.56(3577.41to3644.01)

Norway,Prevalence,55~60,5381.34(5337.7to5425.33)

Norway,Prevalence,60~65,7535.64(7479.54to7592.17)

Norway,Prevalence,65~70,10938.83(10862.43to11015.78)

Norway,Prevalence,70~75,15868.91(15761.36to15977.19)

Norway,Prevalence,75~80,22275.25(22102.67to22449.18)

Norway,Prevalence,80~85,30274.89(30031.06to30520.69)

Norway,Prevalence,85~90,40302.56(39955.81to40652.31)

Norway,Prevalence,90~95,51251.24(50738.4to51769.25)

Norway,Deaths,20~25,0.06(0.01to0.49)

Norway,Deaths,25~30,0.07(0.01to0.39)

Norway,Deaths,30~35,0.13(0.04to0.45)

Norway,Deaths,35~40,0.41(0.2to0.84)

Norway,Deaths,40~45,0.82(0.49to1.35)

Norway,Deaths,45~50,2.24(1.63to3.08)

Norway,Deaths,50~55,6.46(5.3to7.88)

Norway,Deaths,55~60,15.27(13.27to17.57)

Norway,Deaths,60~65,38.89(35.12to43.05)

Norway,Deaths,65~70,81.79(75.08to89.1)

Norway,Deaths,70~75,165.55(153.04to179.08)

Norway,Deaths,75~80,312.48(285.74to341.73)

Norway,Deaths,80~85,504.42(460.56to552.45)

Norway,Deaths,85~90,832.31(758.01to913.89)

Norway,Deaths,90~95,1130.37(1020.65to1251.88)

Norway,DALYs,20~25,20(16.96to23.58)

Norway,DALYs,25~30,26.89(23.53to30.72)

Norway,DALYs,30~35,37.59(33.68to41.94)

Norway,DALYs,35~40,58.73(53.78to64.13)

Norway,DALYs,40~45,88.1(82.05to94.6)

Norway,DALYs,45~50,177.97(168.93to187.5)

Norway,DALYs,50~55,384.53(370.12to399.49)

Norway,DALYs,55~60,717.32(695.81to739.49)

Norway,DALYs,60~65,1408.1(1373.57to1443.51)

Norway,DALYs,65~70,2401.62(2348.41to2456.03)

Norway,DALYs,70~75,3896.31(3813.64to3980.77)

Norway,DALYs,75~80,5786.15(5645.03to5930.79)

Norway,DALYs,80~85,7365.12(7177.01to7558.17)

Norway,DALYs,85~90,9626.76(9362.92to9898.04)

Norway,DALYs,90~95,11499.74(11124.68to11887.44)

Saint_Kitts_and_Nevis,Prevalence,20~25,194.78(137.41to276.09)

Saint_Kitts_and_Nevis,Prevalence,25~30,262.09(194.21to353.68)

Saint_Kitts_and_Nevis,Prevalence,30~35,353.29(272.7to457.7)

Saint_Kitts_and_Nevis,Prevalence,35~40,479.58(381.77to602.47)

Saint_Kitts_and_Nevis,Prevalence,40~45,685.42(561.11to837.28)

Saint_Kitts_and_Nevis,Prevalence,45~50,1092.26(916.73to1301.4)

Saint_Kitts_and_Nevis,Prevalence,50~55,1785.88(1531.1to2083.06)

Saint_Kitts_and_Nevis,Prevalence,55~60,2808.6(2447.8to3222.59)

Saint_Kitts_and_Nevis,Prevalence,60~65,4138.51(3646.07to4697.44)

Saint_Kitts_and_Nevis,Prevalence,65~70,5816.67(5135.96to6587.61)

Saint_Kitts_and_Nevis,Prevalence,70~75,8052.63(7065.33to9177.89)

Saint_Kitts_and_Nevis,Prevalence,75~80,11106.81(9547.21to12921.19)

Saint_Kitts_and_Nevis,Prevalence,80~85,15182.51(12909.79to17855.34)

Saint_Kitts_and_Nevis,Prevalence,85~90,20256.6(16806.27to24415.28)

Saint_Kitts_and_Nevis,Prevalence,90~95,26083.83(20247.52to33602.43)

Saint_Kitts_and_Nevis,Deaths,20~25,0.14(0to91342.3)

Saint_Kitts_and_Nevis,Deaths,25~30,0.15(0to14222.67)

Saint_Kitts_and_Nevis,Deaths,30~35,0.2(0to3322.15)

Saint_Kitts_and_Nevis,Deaths,35~40,0.45(0to317.13)

Saint_Kitts_and_Nevis,Deaths,40~45,0.96(0.01to111.07)

Saint_Kitts_and_Nevis,Deaths,45~50,2.28(0.08to67.9)

Saint_Kitts_and_Nevis,Deaths,50~55,5.57(0.45to68.34)

Saint_Kitts_and_Nevis,Deaths,55~60,13.01(1.97to86.03)

Saint_Kitts_and_Nevis,Deaths,60~65,31.01(7.09to135.67)

Saint_Kitts_and_Nevis,Deaths,65~70,58.64(15.87to216.64)

Saint_Kitts_and_Nevis,Deaths,70~75,111.36(31.58to392.73)

Saint_Kitts_and_Nevis,Deaths,75~80,187.76(44.23to797.04)

Saint_Kitts_and_Nevis,Deaths,80~85,299.2(68.09to1314.73)

Saint_Kitts_and_Nevis,Deaths,85~90,498.01(104.31to2377.73)

Saint_Kitts_and_Nevis,Deaths,90~95,642.09(97.06to4247.78)

Saint_Kitts_and_Nevis,DALYs,20~25,18.72(5.83to60.14)

Saint_Kitts_and_Nevis,DALYs,25~30,21.51(7.86to58.84)

Saint_Kitts_and_Nevis,DALYs,30~35,27.05(11.35to64.47)

Saint_Kitts_and_Nevis,DALYs,35~40,44.37(22.18to88.73)

Saint_Kitts_and_Nevis,DALYs,40~45,75.08(42.71to131.98)

Saint_Kitts_and_Nevis,DALYs,45~50,143.97(91.47to226.63)

Saint_Kitts_and_Nevis,DALYs,50~55,286.34(197.77to414.57)

Saint_Kitts_and_Nevis,DALYs,55~60,545.18(401.7to739.9)

Saint_Kitts_and_Nevis,DALYs,60~65,1048.75(809.59to1358.57)

Saint_Kitts_and_Nevis,DALYs,65~70,1631.05(1278.66to2080.55)

Saint_Kitts_and_Nevis,DALYs,70~75,2493.2(1948.18to3190.7)

Saint_Kitts_and_Nevis,DALYs,75~80,3368.06(2522.45to4497.13)

Saint_Kitts_and_Nevis,DALYs,80~85,4257.33(3118.8to5811.48)

Saint_Kitts_and_Nevis,DALYs,85~90,5652.99(3958.51to8072.81)

Saint_Kitts_and_Nevis,DALYs,90~95,6455.68(3933.39to10595.4)

United_Kingdom,Prevalence,20~25,487.07(476.76to497.61)

United_Kingdom,Prevalence,25~30,679.55(667.98to691.33)

United_Kingdom,Prevalence,30~35,878.43(865.64to891.4)

United_Kingdom,Prevalence,35~40,1077.68(1063.76to1091.79)

United_Kingdom,Prevalence,40~45,1465.78(1449.83to1481.91)

United_Kingdom,Prevalence,45~50,2633.66(2611.18to2656.34)

United_Kingdom,Prevalence,50~55,4397.18(4366.1to4428.48)

United_Kingdom,Prevalence,55~60,6292.35(6252.79to6332.16)

United_Kingdom,Prevalence,60~65,8685.64(8635.26to8736.32)

United_Kingdom,Prevalence,65~70,13071.37(13001.06to13142.07)

United_Kingdom,Prevalence,70~75,19101.86(19002.96to19201.28)

United_Kingdom,Prevalence,75~80,26383.47(26227.82to26540.03)

United_Kingdom,Prevalence,80~85,34566.27(34353.79to34780.07)

United_Kingdom,Prevalence,85~90,44410.21(44115.42to44706.97)

United_Kingdom,Prevalence,90~95,54402.51(53970.66to54837.81)

United_Kingdom,Deaths,20~25,0.1(0.04to0.27)

United_Kingdom,Deaths,25~30,0.15(0.07to0.3)

United_Kingdom,Deaths,30~35,0.23(0.13to0.4)

United_Kingdom,Deaths,35~40,0.47(0.32to0.69)

United_Kingdom,Deaths,40~45,1.2(0.94to1.53)

United_Kingdom,Deaths,45~50,3.06(2.61to3.58)

United_Kingdom,Deaths,50~55,8.87(8.04to9.79)

United_Kingdom,Deaths,55~60,20.02(18.67to21.46)

United_Kingdom,Deaths,60~65,46.88(44.47to49.42)

United_Kingdom,Deaths,65~70,88.58(84.65to92.7)

United_Kingdom,Deaths,70~75,157.07(150.53to163.89)

United_Kingdom,Deaths,75~80,255.55(243.85to267.8)

United_Kingdom,Deaths,80~85,385.68(367.95to404.28)

United_Kingdom,Deaths,85~90,569.52(542.74to597.63)

United_Kingdom,Deaths,90~95,801.17(760.76to843.73)

United_Kingdom,DALYs,20~25,29.2(23.49to36.3)

United_Kingdom,DALYs,25~30,40.09(33.75to47.62)

United_Kingdom,DALYs,30~35,53.31(46.2to61.5)

United_Kingdom,DALYs,35~40,73.46(65.15to82.82)

United_Kingdom,DALYs,40~45,123.01(112.12to134.95)

United_Kingdom,DALYs,45~50,249.04(232.82to266.39)

United_Kingdom,DALYs,50~55,532.88(507.17to559.9)

United_Kingdom,DALYs,55~60,944.13(907.39to982.35)

United_Kingdom,DALYs,60~65,1729.08(1672.33to1787.76)

United_Kingdom,DALYs,65~70,2713.8(2632.51to2797.61)

United_Kingdom,DALYs,70~75,3948.21(3834.06to4065.77)

United_Kingdom,DALYs,75~80,5165.47(4997.99to5338.56)

United_Kingdom,DALYs,80~85,6168.98(5961.48to6383.7)

United_Kingdom,DALYs,85~90,7285.59(7021.86to7559.21)

United_Kingdom,DALYs,90~95,8853.31(8475.78to9247.67)

Argentina,Prevalence,20~25,291.43(281.44to301.77)

Argentina,Prevalence,25~30,377.65(366.4to389.24)

Argentina,Prevalence,30~35,488.98(476.12to502.19)

Argentina,Prevalence,35~40,633.28(618.55to648.36)

Argentina,Prevalence,40~45,860.03(842.84to877.58)

Argentina,Prevalence,45~50,1329.01(1306.36to1352.04)

Argentina,Prevalence,50~55,2122.34(2091.41to2153.72)

Argentina,Prevalence,55~60,3256.52(3214.67to3298.91)

Argentina,Prevalence,60~65,4724.57(4668.57to4781.24)

Argentina,Prevalence,65~70,6651.83(6576.35to6728.18)

Argentina,Prevalence,70~75,9192.48(9088.44to9297.72)

Argentina,Prevalence,75~80,12344.81(12185.22to12506.49)

Argentina,Prevalence,80~85,16176.52(15954.81to16401.3)

Argentina,Prevalence,85~90,20268.4(19958.59to20583.02)

Argentina,Prevalence,90~95,23730.58(23267.58to24202.79)

Argentina,Deaths,20~25,1.22(0.98to1.53)

Argentina,Deaths,25~30,1.35(1.11to1.63)

Argentina,Deaths,30~35,1.73(1.47to2.03)

Argentina,Deaths,35~40,2.44(2.14to2.79)

Argentina,Deaths,40~45,4.02(3.64to4.44)

Argentina,Deaths,45~50,7.43(6.89to8.01)

Argentina,Deaths,50~55,15.36(14.52to16.24)

Argentina,Deaths,55~60,28.44(27.2to29.74)

Argentina,Deaths,60~65,55.7(53.69to57.79)

Argentina,Deaths,65~70,93.59(90.52to96.75)

Argentina,Deaths,70~75,159.96(154.92to165.16)

Argentina,Deaths,75~80,256.16(247.17to265.48)

Argentina,Deaths,80~85,425.76(410.62to441.45)

Argentina,Deaths,85~90,687.98(662.73to714.19)

Argentina,Deaths,90~95,1095.22(1051.46to1140.8)

Argentina,DALYs,20~25,94.04(83.6to105.8)

Argentina,DALYs,25~30,100.79(90.8to111.88)

Argentina,DALYs,30~35,121.13(110.57to132.7)

Argentina,DALYs,35~40,155.82(144.13to168.47)

Argentina,DALYs,40~45,226.94(213.08to241.71)

Argentina,DALYs,45~50,371.58(353.21to390.9)

Argentina,DALYs,50~55,668.43(641.79to696.18)

Argentina,DALYs,55~60,1075.28(1038.49to1113.38)

Argentina,DALYs,60~65,1779.63(1726.03to1834.89)

Argentina,DALYs,65~70,2508.8(2436.94to2582.78)

Argentina,DALYs,70~75,3508.65(3408.3to3611.95)

Argentina,DALYs,75~80,4498.78(4352.23to4650.27)

Argentina,DALYs,80~85,5846.06(5645.29to6053.98)

Argentina,DALYs,85~90,7493.59(7211.63to7786.57)

Argentina,DALYs,90~95,10269.16(9804.78to10755.54)

Guam,Prevalence,20~25,408.96(351.17to476.25)

Guam,Prevalence,25~30,541.76(475.15to617.71)

Guam,Prevalence,30~35,690.33(614.42to775.63)

Guam,Prevalence,35~40,848.23(763.57to942.27)

Guam,Prevalence,40~45,1070.64(975.09to1175.54)

Guam,Prevalence,45~50,1505.49(1385.74to1635.59)

Guam,Prevalence,50~55,2171.55(2014.99to2340.26)

Guam,Prevalence,55~60,3043.84(2840.49to3261.75)

Guam,Prevalence,60~65,4200.85(3934.39to4485.36)

Guam,Prevalence,65~70,5767.54(5409.13to6149.68)

Guam,Prevalence,70~75,7925.24(7423.13to8461.31)

Guam,Prevalence,75~80,10757.17(9946.34to11634.09)

Guam,Prevalence,80~85,14431.48(13272.51to15691.67)

Guam,Prevalence,85~90,19150.69(17473.77to20988.53)

Guam,Prevalence,90~95,24409.75(21964.52to27127.2)

Guam,Deaths,20~25,0.71(0.02to20.42)

Guam,Deaths,25~30,0.99(0.06to15.69)

Guam,Deaths,30~35,1.62(0.18to14.54)

Guam,Deaths,35~40,2.12(0.32to14.04)

Guam,Deaths,40~45,3.6(0.82to15.82)

Guam,Deaths,45~50,7.01(2.17to22.6)

Guam,Deaths,50~55,13.85(5.62to34.09)

Guam,Deaths,55~60,19.79(8.69to45.09)

Guam,Deaths,60~65,34.67(17.07to70.41)

Guam,Deaths,65~70,62.09(33.17to116.22)

Guam,Deaths,70~75,99.6(53.47to185.53)

Guam,Deaths,75~80,145.79(71.93to295.5)

Guam,Deaths,80~85,166.61(79.36to349.77)

Guam,Deaths,85~90,166.78(73.67to377.58)

Guam,Deaths,90~95,164.31(64.13to420.97)

Guam,DALYs,20~25,80.76(57.72to113.01)

Guam,DALYs,25~30,105.44(79.06to140.62)

Guam,DALYs,30~35,148.23(115.97to189.48)

Guam,DALYs,35~40,178.29(142.93to222.4)

Guam,DALYs,40~45,254.9(211.52to307.17)

Guam,DALYs,45~50,416.07(354.86to487.84)

Guam,DALYs,50~55,693(606.48to791.86)

Guam,DALYs,55~60,889.42(783.19to1010.07)

Guam,DALYs,60~65,1308.02(1163.75to1470.18)

Guam,DALYs,65~70,1929.64(1728.77to2153.85)

Guam,DALYs,70~75,2566.95(2291.3to2875.77)

Guam,DALYs,75~80,3103.5(2714.14to3548.73)

Guam,DALYs,80~85,3021.81(2602.93to3508.09)

Guam,DALYs,85~90,2727.83(2285.29to3256.06)

Guam,DALYs,90~95,2596.02(2085.21to3231.97)

Viet_Nam,Prevalence,20~25,381.5(377.57to385.47)

Viet_Nam,Prevalence,25~30,528.41(523.71to533.15)

Viet_Nam,Prevalence,30~35,694.04(688.52to699.61)

Viet_Nam,Prevalence,35~40,869.91(863.54to876.33)

Viet_Nam,Prevalence,40~45,1151.64(1143.98to1159.36)

Viet_Nam,Prevalence,45~50,1850.99(1840.05to1861.99)

Viet_Nam,Prevalence,50~55,2988.4(2972.69to3004.19)

Viet_Nam,Prevalence,55~60,4366.29(4345.17to4387.51)

Viet_Nam,Prevalence,60~65,6035.26(6007.43to6063.21)

Viet_Nam,Prevalence,65~70,8404.97(8367.12to8442.98)

Viet_Nam,Prevalence,70~75,11796.98(11742.83to11851.38)

Viet_Nam,Prevalence,75~80,16391.87(16305.47to16478.72)

Viet_Nam,Prevalence,80~85,22189.26(22063.47to22315.76)

Viet_Nam,Prevalence,85~90,28788.55(28598.78to28979.58)

Viet_Nam,Prevalence,90~95,35136.59(34814.74to35461.42)

Viet_Nam,Deaths,20~25,0.56(0.47to0.66)

Viet_Nam,Deaths,25~30,0.69(0.6to0.79)

Viet_Nam,Deaths,30~35,0.94(0.83to1.06)

Viet_Nam,Deaths,35~40,1.51(1.37to1.67)

Viet_Nam,Deaths,40~45,3.12(2.91to3.36)

Viet_Nam,Deaths,45~50,7.04(6.68to7.42)

Viet_Nam,Deaths,50~55,16.98(16.34to17.65)

Viet_Nam,Deaths,55~60,31.92(30.93to32.93)

Viet_Nam,Deaths,60~65,71.18(69.41to73)

Viet_Nam,Deaths,65~70,141.45(138.33to144.64)

Viet_Nam,Deaths,70~75,262.25(256.71to267.91)

Viet_Nam,Deaths,75~80,441.5(431.17to452.08)

Viet_Nam,Deaths,80~85,731.88(714.44to749.74)

Viet_Nam,Deaths,85~90,1132.03(1103.63to1161.17)

Viet_Nam,Deaths,90~95,1379.17(1338.16to1421.44)

Viet_Nam,DALYs,20~25,62.55(58.66to66.69)

Viet_Nam,DALYs,25~30,78.37(74.17to82.8)

Viet_Nam,DALYs,30~35,100.58(95.84to105.55)

Viet_Nam,DALYs,35~40,136.87(131.19to142.79)

Viet_Nam,DALYs,40~45,222.7(214.97to230.71)

Viet_Nam,DALYs,45~50,417.78(405.88to430.02)

Viet_Nam,DALYs,50~55,831.5(812.3to851.15)

Viet_Nam,DALYs,55~60,1334.7(1307.8to1362.16)

Viet_Nam,DALYs,60~65,2418.74(2376.11to2462.13)

Viet_Nam,DALYs,65~70,3943.38(3878.52to4009.33)

Viet_Nam,DALYs,70~75,5947.29(5849.83to6046.37)

Viet_Nam,DALYs,75~80,8023.57(7874.94to8175.01)

Viet_Nam,DALYs,80~85,10447.2(10242.2to10656.29)

Viet_Nam,DALYs,85~90,12880.71(12593.76to13174.2)

Viet_Nam,DALYs,90~95,13793.94(13370.8to14230.46)

Samoa,Prevalence,20~25,594.21(524.5to673.19)

Samoa,Prevalence,25~30,824.33(737.57to921.3)

Samoa,Prevalence,30~35,1043.22(941.65to1155.74)

Samoa,Prevalence,35~40,1241.66(1127.55to1367.31)

Samoa,Prevalence,40~45,1526.09(1397.84to1666.1)

Samoa,Prevalence,45~50,2236.09(2067.2to2418.78)

Samoa,Prevalence,50~55,3384.75(3155.22to3630.99)

Samoa,Prevalence,55~60,4700.16(4402.2to5018.28)

Samoa,Prevalence,60~65,6215.26(5830.06to6625.92)

Samoa,Prevalence,65~70,8418.28(7899.25to8971.42)

Samoa,Prevalence,70~75,11518.11(10794.77to12289.92)

Samoa,Prevalence,75~80,15271.47(14184.93to16441.23)

Samoa,Prevalence,80~85,19583.67(18055.37to21241.33)

Samoa,Prevalence,85~90,24492.75(22211.45to27008.35)

Samoa,Prevalence,90~95,28727.23(24856.25to33201.05)

Samoa,Deaths,20~25,2.61(0.46to14.72)

Samoa,Deaths,25~30,3.05(0.63to14.76)

Samoa,Deaths,30~35,5.07(1.39to18.48)

Samoa,Deaths,35~40,7.13(2.3to22.12)

Samoa,Deaths,40~45,12.22(4.91to30.41)

Samoa,Deaths,45~50,20.53(9.62to43.84)

Samoa,Deaths,50~55,39.22(21.34to72.09)

Samoa,Deaths,55~60,60.1(35.22to102.57)

Samoa,Deaths,60~65,123.02(78.26to193.39)

Samoa,Deaths,65~70,218.32(143.97to331.08)

Samoa,Deaths,70~75,368.79(246.25to552.32)

Samoa,Deaths,75~80,612.37(393.01to954.16)

Samoa,Deaths,80~85,921.18(582.35to1457.15)

Samoa,Deaths,85~90,1418.94(868.11to2319.28)

Samoa,Deaths,90~95,2127.68(1181.74to3830.84)

Samoa,DALYs,20~25,223.5(182.76to273.32)

Samoa,DALYs,25~30,257.36(213.78to309.81)

Samoa,DALYs,30~35,374.66(318.61to440.56)

Samoa,DALYs,35~40,471.7(406.47to547.41)

Samoa,DALYs,40~45,699.38(615.93to794.14)

Samoa,DALYs,45~50,1049.19(938.24to1173.27)

Samoa,DALYs,50~55,1747.71(1587.81to1923.72)

Samoa,DALYs,55~60,2358.38(2157.6to2577.84)

Samoa,DALYs,60~65,4007.1(3697.05to4343.16)

Samoa,DALYs,65~70,5921.48(5478.6to6400.17)

Samoa,DALYs,70~75,8204.01(7585.66to8872.77)

Samoa,DALYs,75~80,10884.61(9964.95to11889.15)

Samoa,DALYs,80~85,12889.47(11700.18to14199.65)

Samoa,DALYs,85~90,15755.12(14056.66to17658.8)

Samoa,DALYs,90~95,20280.65(17311.5to23759.06)

Bangladesh,Prevalence,20~25,541.29(532.47to550.26)

Bangladesh,Prevalence,25~30,745.55(734.96to756.29)

Bangladesh,Prevalence,30~35,949.07(936.78to961.53)

Bangladesh,Prevalence,35~40,1140.91(1127.06to1154.94)

Bangladesh,Prevalence,40~45,1546.75(1529.93to1563.75)

Bangladesh,Prevalence,45~50,2814.57(2788.71to2840.67)

Bangladesh,Prevalence,50~55,4566.07(4529.02to4603.43)

Bangladesh,Prevalence,55~60,6213.59(6166.64to6260.89)

Bangladesh,Prevalence,60~65,8182.42(8123.57to8241.7)

Bangladesh,Prevalence,65~70,11926.11(11844.36to12008.44)

Bangladesh,Prevalence,70~75,17091.84(16975.57to17208.91)

Bangladesh,Prevalence,75~80,23021.39(22833.43to23210.9)

Bangladesh,Prevalence,80~85,29417.35(29145.95to29691.27)

Bangladesh,Prevalence,85~90,36732.64(36306.66to37163.61)

Bangladesh,Prevalence,90~95,44854.75(44073.93to45649.4)

Bangladesh,Deaths,20~25,1.03(0.7to1.52)

Bangladesh,Deaths,25~30,1.9(1.44to2.52)

Bangladesh,Deaths,30~35,2.82(2.25to3.55)

Bangladesh,Deaths,35~40,4.25(3.53to5.13)

Bangladesh,Deaths,40~45,7.82(6.79to9.01)

Bangladesh,Deaths,45~50,17.33(15.66to19.18)

Bangladesh,Deaths,50~55,44.12(41.11to47.35)

Bangladesh,Deaths,55~60,76.29(72.04to80.79)

Bangladesh,Deaths,60~65,166.56(159.01to174.47)

Bangladesh,Deaths,65~70,226.53(216.76to236.75)

Bangladesh,Deaths,70~75,352.36(338.08to367.25)

Bangladesh,Deaths,75~80,450.42(429.08to472.82)

Bangladesh,Deaths,80~85,825.87(786.18to867.56)

Bangladesh,Deaths,85~90,967.37(914.02to1023.84)

Bangladesh,Deaths,90~95,1066.4(986.44to1152.85)

Bangladesh,DALYs,20~25,133.66(115.05to155.28)

Bangladesh,DALYs,25~30,200.75(177.59to226.94)

Bangladesh,DALYs,30~35,263.45(236.65to293.28)

Bangladesh,DALYs,35~40,339.91(308.82to374.12)

Bangladesh,DALYs,40~45,521.15(481.29to564.3)

Bangladesh,DALYs,45~50,1003.65(942.56to1068.7)

Bangladesh,DALYs,50~55,2080.07(1980.35to2184.82)

Bangladesh,DALYs,55~60,3087.57(2957.68to3223.16)

Bangladesh,DALYs,60~65,5514.24(5311.18to5725.07)

Bangladesh,DALYs,65~70,6536.58(6298.68to6783.46)

Bangladesh,DALYs,70~75,8502.87(8199.77to8817.16)

Bangladesh,DALYs,75~80,8950.12(8564.67to9352.91)

Bangladesh,DALYs,80~85,12603.34(12021.04to13213.85)

Bangladesh,DALYs,85~90,12211.81(11480.68to12989.51)

Bangladesh,DALYs,90~95,12039.98(10901.44to13297.43)

Fiji,Prevalence,20~25,529.32(496.9to563.86)

Fiji,Prevalence,25~30,683(645.99to722.13)

Fiji,Prevalence,30~35,840.27(799.01to883.67)

Fiji,Prevalence,35~40,995.62(950.37to1043.02)

Fiji,Prevalence,40~45,1219.9(1170.33to1271.57)

Fiji,Prevalence,45~50,1699.49(1637.14to1764.22)

Fiji,Prevalence,50~55,2447.14(2365.41to2531.7)

Fiji,Prevalence,55~60,3396.73(3289.91to3507.01)

Fiji,Prevalence,60~65,4578.54(4437.64to4723.92)

Fiji,Prevalence,65~70,6076.38(5886.61to6272.28)

Fiji,Prevalence,70~75,8082.51(7817.58to8356.4)

Fiji,Prevalence,75~80,10619(10204.59to11050.23)

Fiji,Prevalence,80~85,13793.72(13171.67to14445.14)

Fiji,Prevalence,85~90,17563.59(16519.39to18673.79)

Fiji,Prevalence,90~95,21285.3(19090.49to23732.45)

Fiji,Deaths,20~25,2.77(1.05to7.33)

Fiji,Deaths,25~30,2.15(0.81to5.72)

Fiji,Deaths,30~35,3.1(1.39to6.92)

Fiji,Deaths,35~40,3.57(1.74to7.32)

Fiji,Deaths,40~45,6.36(3.69to10.97)

Fiji,Deaths,45~50,11.19(7.28to17.21)

Fiji,Deaths,50~55,25.31(18.35to34.9)

Fiji,Deaths,55~60,33.76(25.09to45.42)

Fiji,Deaths,60~65,77.24(60.36to98.86)

Fiji,Deaths,65~70,122.05(96.4to154.53)

Fiji,Deaths,70~75,192.34(152.45to242.66)

Fiji,Deaths,75~80,297.87(228.66to388.02)

Fiji,Deaths,80~85,455.19(343.91to602.48)

Fiji,Deaths,85~90,798.68(584.57to1091.22)

Fiji,Deaths,90~95,1067.32(682.61to1668.84)

Fiji,DALYs,20~25,224.99(201.41to251.33)

Fiji,DALYs,25~30,192.31(172.63to214.23)

Fiji,DALYs,30~35,247.2(225.03to271.55)

Fiji,DALYs,35~40,269.13(246.72to293.58)

Fiji,DALYs,40~45,397.9(370.37to427.47)

Fiji,DALYs,45~50,608.12(572.06to646.45)

Fiji,DALYs,50~55,1145.45(1089.52to1204.25)

Fiji,DALYs,55~60,1379.57(1314.55to1447.81)

Fiji,DALYs,60~65,2565.86(2457.62to2678.86)

Fiji,DALYs,65~70,3408.05(3264.04to3558.42)

Fiji,DALYs,70~75,4423.38(4230.26to4625.32)

Fiji,DALYs,75~80,5511.26(5228.16to5809.69)

Fiji,DALYs,80~85,6634.76(6244.71to7049.18)

Fiji,DALYs,85~90,9175.31(8503.51to9900.19)

Fiji,DALYs,90~95,10697.53(9393.93to12182.04)

Equatorial_Guinea,Prevalence,20~25,435.82(402.75to471.6)

Equatorial_Guinea,Prevalence,25~30,558.58(520.06to599.95)

Equatorial_Guinea,Prevalence,30~35,702.61(657.46to750.87)

Equatorial_Guinea,Prevalence,35~40,863.48(811.14to919.19)

Equatorial_Guinea,Prevalence,40~45,1107.87(1045.82to1173.59)

Equatorial_Guinea,Prevalence,45~50,1645.27(1561.24to1733.83)

Equatorial_Guinea,Prevalence,50~55,2534.05(2416.28to2657.56)

Equatorial_Guinea,Prevalence,55~60,3688.64(3527.64to3856.99)

Equatorial_Guinea,Prevalence,60~65,5007.76(4795.94to5228.95)

Equatorial_Guinea,Prevalence,65~70,6534.27(6259.64to6820.94)

Equatorial_Guinea,Prevalence,70~75,8441.57(8068.36to8832.04)

Equatorial_Guinea,Prevalence,75~80,10882.45(10311.83to11484.66)

Equatorial_Guinea,Prevalence,80~85,13961.46(13117.68to14859.52)

Equatorial_Guinea,Prevalence,85~90,17312.88(15950.58to18791.54)

Equatorial_Guinea,Prevalence,90~95,20251.84(17722.4to23142.3)

Equatorial_Guinea,Deaths,20~25,1.09(0.17to7.09)

Equatorial_Guinea,Deaths,25~30,1.16(0.22to6.26)

Equatorial_Guinea,Deaths,30~35,2(0.52to7.68)

Equatorial_Guinea,Deaths,35~40,2.45(0.74to8.08)

Equatorial_Guinea,Deaths,40~45,4.9(2to11.97)

Equatorial_Guinea,Deaths,45~50,11.63(6.15to22)

Equatorial_Guinea,Deaths,50~55,17.96(10.49to30.74)

Equatorial_Guinea,Deaths,55~60,32.78(20.93to51.34)

Equatorial_Guinea,Deaths,60~65,56.02(37.71to83.24)

Equatorial_Guinea,Deaths,65~70,92.74(64.45to133.45)

Equatorial_Guinea,Deaths,70~75,138.67(97.16to197.93)

Equatorial_Guinea,Deaths,75~80,171.67(114.4to257.61)

Equatorial_Guinea,Deaths,80~85,235.63(153.13to362.57)

Equatorial_Guinea,Deaths,85~90,288.95(174.68to477.98)

Equatorial_Guinea,Deaths,90~95,408(209.77to793.54)

Equatorial_Guinea,DALYs,20~25,128.01(108.28to151.33)

Equatorial_Guinea,DALYs,25~30,145.12(124.68to168.89)

Equatorial_Guinea,DALYs,30~35,197.24(172.32to225.77)

Equatorial_Guinea,DALYs,35~40,229.06(201.99to259.77)

Equatorial_Guinea,DALYs,40~45,349.12(313.66to388.58)

Equatorial_Guinea,DALYs,45~50,652.99(598.44to712.51)

Equatorial_Guinea,DALYs,50~55,927.51(857.41to1003.33)

Equatorial_Guinea,DALYs,55~60,1443.05(1344.42to1548.92)

Equatorial_Guinea,DALYs,60~65,2083.69(1950.22to2226.29)

Equatorial_Guinea,DALYs,65~70,2866.57(2689.72to3055.06)

Equatorial_Guinea,DALYs,70~75,3545.22(3321.24to3784.31)

Equatorial_Guinea,DALYs,75~80,3647.67(3375.23to3942.1)

Equatorial_Guinea,DALYs,80~85,4010.79(3667.5to4386.22)

Equatorial_Guinea,DALYs,85~90,4031.58(3575.91to4545.31)

Equatorial_Guinea,DALYs,90~95,4844.79(4025.28to5831.15)

Spain,Prevalence,20~25,536.16(515.81to557.31)

Spain,Prevalence,25~30,736.7(714.01to760.12)

Spain,Prevalence,30~35,939.75(914.95to965.23)

Spain,Prevalence,35~40,1136.67(1110.04to1163.94)

Spain,Prevalence,40~45,1482.61(1452.72to1513.12)

Spain,Prevalence,45~50,2441.9(2401.43to2483.05)

Spain,Prevalence,50~55,3888.58(3833.86to3944.08)

Spain,Prevalence,55~60,5478.88(5410.11to5548.53)

Spain,Prevalence,60~65,7346.74(7261.2to7433.29)

Spain,Prevalence,65~70,10201.33(10088.36to10315.57)

Spain,Prevalence,70~75,14119.56(13966.43to14274.37)

Spain,Prevalence,75~80,19216.68(18984.83to19451.36)

Spain,Prevalence,80~85,26030.55(25708.07to26357.08)

Spain,Prevalence,85~90,34915.68(34459.05to35378.35)

Spain,Prevalence,90~95,43241.17(42578.18to43914.48)

Spain,Deaths,20~25,0.42(0.28to0.62)

Spain,Deaths,25~30,0.48(0.35to0.65)

Spain,Deaths,30~35,0.58(0.45to0.74)

Spain,Deaths,35~40,0.8(0.66to0.98)

Spain,Deaths,40~45,1.43(1.24to1.65)

Spain,Deaths,45~50,2.96(2.68to3.27)

Spain,Deaths,50~55,6.27(5.84to6.72)

Spain,Deaths,55~60,11.73(11.13to12.37)

Spain,Deaths,60~65,23.87(22.9to24.88)

Spain,Deaths,65~70,43.12(41.59to44.7)

Spain,Deaths,70~75,81.01(78.33to83.79)

Spain,Deaths,75~80,145.97(140.8to151.32)

Spain,Deaths,80~85,264.17(254.91to273.76)

Spain,Deaths,85~90,482.37(465.44to499.92)

Spain,Deaths,90~95,808.84(779.8to838.96)

Spain,DALYs,20~25,46.61(40.5to53.62)

Spain,DALYs,25~30,57.46(51.26to64.4)

Spain,DALYs,30~35,69.06(62.71to76.06)

Spain,DALYs,35~40,84.56(77.84to91.86)

Spain,DALYs,40~45,121.1(113.26to129.48)

Spain,DALYs,45~50,213.78(203.01to225.12)

Spain,DALYs,50~55,377.65(362.55to393.39)

Spain,DALYs,55~60,589.09(569.3to609.56)

Spain,DALYs,60~65,958.3(930.42to987.01)

Spain,DALYs,65~70,1430.31(1391.96to1469.71)

Spain,DALYs,70~75,2161.95(2106.25to2219.13)

Spain,DALYs,75~80,3078.07(2992.17to3166.43)

Spain,DALYs,80~85,4315.09(4193.91to4439.77)

Spain,DALYs,85~90,6186.75(6009.17to6369.57)

Spain,DALYs,90~95,8872.66(8599.02to9155.02)

Tonga,Prevalence,20~25,571.57(478.14to683.25)

Tonga,Prevalence,25~30,765.66(651.67to899.58)

Tonga,Prevalence,30~35,955.41(822.22to1110.18)

Tonga,Prevalence,35~40,1129.68(981.17to1300.66)

Tonga,Prevalence,40~45,1367.88(1204.65to1553.23)

Tonga,Prevalence,45~50,1897.34(1692.67to2126.75)

Tonga,Prevalence,50~55,2747.78(2481.56to3042.56)

Tonga,Prevalence,55~60,3831.71(3484.47to4213.56)

Tonga,Prevalence,60~65,5210.49(4755.67to5708.81)

Tonga,Prevalence,65~70,7172.83(6557.51to7845.9)

Tonga,Prevalence,70~75,9796.98(8947.82to10726.71)

Tonga,Prevalence,75~80,12936.44(11682.5to14324.97)

Tonga,Prevalence,80~85,16544.25(14832.64to18453.38)

Tonga,Prevalence,85~90,20716.91(18287.16to23469.49)

Tonga,Prevalence,90~95,24845.69(21073.28to29293.42)

Tonga,Deaths,20~25,1.16(0.04to34.54)

Tonga,Deaths,25~30,1.53(0.08to30.71)

Tonga,Deaths,30~35,3.36(0.36to30.88)

Tonga,Deaths,35~40,4.61(0.67to31.91)

Tonga,Deaths,40~45,9.06(2.11to38.86)

Tonga,Deaths,45~50,14.19(4.18to48.22)

Tonga,Deaths,50~55,26.04(9.82to69.07)

Tonga,Deaths,55~60,39.36(16.71to92.72)

Tonga,Deaths,60~65,78.29(38.01to161.27)

Tonga,Deaths,65~70,148.75(77.26to286.43)

Tonga,Deaths,70~75,276.88(147.77to518.79)

Tonga,Deaths,75~80,468.41(236.01to929.67)

Tonga,Deaths,80~85,723.7(359.82to1455.57)

Tonga,Deaths,85~90,1057.15(506.81to2205.08)

Tonga,Deaths,90~95,1497.88(651.93to3441.54)

Tonga,DALYs,20~25,123.98(87.03to176.63)

Tonga,DALYs,25~30,156.99(114.03to216.14)

Tonga,DALYs,30~35,267.84(205.15to349.69)

Tonga,DALYs,35~40,329.96(258.44to421.27)

Tonga,DALYs,40~45,535.91(439.05to654.14)

Tonga,DALYs,45~50,752.21(630.28to897.73)

Tonga,DALYs,50~55,1198.25(1029.86to1394.18)

Tonga,DALYs,55~60,1601.52(1391.38to1843.4)

Tonga,DALYs,60~65,2643.12(2327.74to3001.23)

Tonga,DALYs,65~70,4138.01(3666.77to4669.8)

Tonga,DALYs,70~75,6245.75(5539.41to7042.16)

Tonga,DALYs,75~80,8412.35(7358.43to9617.23)

Tonga,DALYs,80~85,10207.76(8849.36to11774.66)

Tonga,DALYs,85~90,11917.45(10130to14020.29)

Tonga,DALYs,90~95,14585.37(11837.47to17971.17)

Tokelau,Prevalence,20~25,610.45(139.14to2678.14)

Tokelau,Prevalence,25~30,818.13(221.65to3019.83)

Tokelau,Prevalence,30~35,1012.33(307.06to3337.52)

Tokelau,Prevalence,35~40,1184.07(391.66to3579.64)

Tokelau,Prevalence,40~45,1417.86(538.01to3736.59)

Tokelau,Prevalence,45~50,1962.32(834.85to4612.45)

Tokelau,Prevalence,50~55,2836.83(1325.59to6070.97)

Tokelau,Prevalence,55~60,3896.33(1923.25to7893.63)

Tokelau,Prevalence,60~65,5174.23(2698.75to9920.4)

Tokelau,Prevalence,65~70,6894(3669.82to12950.85)

Tokelau,Prevalence,70~75,9220.09(4837.37to17573.58)

Tokelau,Prevalence,75~80,12119.72(5944.18to24711.13)

Tokelau,Prevalence,80~85,15619.71(7291.45to33460.45)

Tokelau,Prevalence,85~90,19603.56(8157.76to47108.43)

Tokelau,Prevalence,90~95,23305.99(7093.84to76569.17)

Tokelau,Deaths,20~25,2.35(0to38471584501.8)

Tokelau,Deaths,25~30,2.76(0to6721757683.04)

Tokelau,Deaths,30~35,4.18(0to222360296.37)

Tokelau,Deaths,35~40,5.21(0to36683547.26)

Tokelau,Deaths,40~45,8.28(0to1557881.08)

Tokelau,Deaths,45~50,13.38(0to241765.31)

Tokelau,Deaths,50~55,24.71(0.01to52488.45)

Tokelau,Deaths,55~60,36.66(0.05to27614.16)

Tokelau,Deaths,60~65,76.94(0.43to13735.54)

Tokelau,Deaths,65~70,132.08(1.14to15275.9)

Tokelau,Deaths,70~75,225.98(2.25to22704.49)

Tokelau,Deaths,75~80,360.06(2.6to49882.85)

Tokelau,Deaths,80~85,548.96(3.67to82051.25)

Tokelau,Deaths,85~90,844.46(4.59to155395.56)

Tokelau,Deaths,90~95,1238.92(3.55to432806.19)

Tokelau,DALYs,20~25,207.49(14.85to2899.25)

Tokelau,DALYs,25~30,238.58(20.8to2736.16)

Tokelau,DALYs,30~35,321.85(38.2to2711.57)

Tokelau,DALYs,35~40,367.89(51.52to2626.92)

Tokelau,DALYs,40~45,504.51(100.38to2535.64)

Tokelau,DALYs,45~50,721.98(180.34to2890.41)

Tokelau,DALYs,50~55,1160.29(360.41to3735.37)

Tokelau,DALYs,55~60,1515.96(515.99to4453.81)

Tokelau,DALYs,60~65,2615.63(1050.36to6513.51)

Tokelau,DALYs,65~70,3729.62(1561.58to8907.71)

Tokelau,DALYs,70~75,5215.37(2180.73to12472.93)

Tokelau,DALYs,75~80,6656.35(2564.58to17276.47)

Tokelau,DALYs,80~85,7972.39(2899.12to21923.54)

Tokelau,DALYs,85~90,9755.41(3138.37to30324.03)

Tokelau,DALYs,90~95,12304.17(2884.91to52477.32)

United_States_Virgin_Islands,Prevalence,20~25,203.41(157.17to263.27)

United_States_Virgin_Islands,Prevalence,25~30,272.15(219to338.19)

United_States_Virgin_Islands,Prevalence,30~35,364.5(304.88to435.78)

United_States_Virgin_Islands,Prevalence,35~40,492.45(423.83to572.17)

United_States_Virgin_Islands,Prevalence,40~45,698.15(617.76to789.01)

United_States_Virgin_Islands,Prevalence,45~50,1091.25(987.68to1205.68)

United_States_Virgin_Islands,Prevalence,50~55,1748.52(1608.17to1901.12)

United_States_Virgin_Islands,Prevalence,55~60,2718.21(2526.32to2924.68)

United_States_Virgin_Islands,Prevalence,60~65,4014.14(3750.98to4295.75)

United_States_Virgin_Islands,Prevalence,65~70,5671.59(5312.04to6055.48)

United_States_Virgin_Islands,Prevalence,70~75,7952.4(7447.81to8491.17)

United_States_Virgin_Islands,Prevalence,75~80,11021.59(10183.66to11928.46)

United_States_Virgin_Islands,Prevalence,80~85,15054.01(13790.73to16433.01)

United_States_Virgin_Islands,Prevalence,85~90,20226.92(18223.22to22450.92)

United_States_Virgin_Islands,Prevalence,90~95,26541.02(23082.68to30517.49)

United_States_Virgin_Islands,Deaths,20~25,0.26(0to1409.3)

United_States_Virgin_Islands,Deaths,25~30,0.35(0to423.69)

United_States_Virgin_Islands,Deaths,30~35,0.38(0to172.28)

United_States_Virgin_Islands,Deaths,35~40,0.64(0.01to55.86)

United_States_Virgin_Islands,Deaths,40~45,1.04(0.04to28.88)

United_States_Virgin_Islands,Deaths,45~50,2.31(0.24to22.19)

United_States_Virgin_Islands,Deaths,50~55,4.2(0.81to21.92)

United_States_Virgin_Islands,Deaths,55~60,8.42(2.33to30.42)

United_States_Virgin_Islands,Deaths,60~65,17.13(5.97to49.16)

United_States_Virgin_Islands,Deaths,65~70,23.89(8.87to64.33)

United_States_Virgin_Islands,Deaths,70~75,40.73(15.87to104.49)

United_States_Virgin_Islands,Deaths,75~80,68.31(23.57to197.95)

United_States_Virgin_Islands,Deaths,80~85,104.67(35.06to312.49)

United_States_Virgin_Islands,Deaths,85~90,161.78(51.04to512.79)

United_States_Virgin_Islands,Deaths,90~95,249.48(68.56to907.79)

United_States_Virgin_Islands,DALYs,20~25,25.83(11.36to58.73)

United_States_Virgin_Islands,DALYs,25~30,32.79(16.32to65.9)

United_States_Virgin_Islands,DALYs,30~35,37.47(20.57to68.25)

United_States_Virgin_Islands,DALYs,35~40,54.3(33.62to87.69)

United_States_Virgin_Islands,DALYs,40~45,77.64(52.96to113.82)

United_States_Virgin_Islands,DALYs,45~50,140.79(105.34to188.15)

United_States_Virgin_Islands,DALYs,50~55,225.69(178.9to284.73)

United_States_Virgin_Islands,DALYs,55~60,380.87(312.95to463.54)

United_States_Virgin_Islands,DALYs,60~65,640.21(538.1to761.7)

United_States_Virgin_Islands,DALYs,65~70,782.68(659.87to928.34)

United_States_Virgin_Islands,DALYs,70~75,1092.31(922.16to1293.87)

United_States_Virgin_Islands,DALYs,75~80,1466.7(1203.68to1787.19)

United_States_Virgin_Islands,DALYs,80~85,1799.28(1451.18to2230.87)

United_States_Virgin_Islands,DALYs,85~90,2232.3(1741.88to2860.79)

United_States_Virgin_Islands,DALYs,90~95,2968.09(2169.1to4061.39)

Uruguay,Prevalence,20~25,231.62(222.26to241.37)

Uruguay,Prevalence,25~30,312.5(301.65to323.74)

Uruguay,Prevalence,30~35,422.74(410.06to435.82)

Uruguay,Prevalence,35~40,571.75(556.83to587.06)

Uruguay,Prevalence,40~45,807.36(789.44to825.68)

Uruguay,Prevalence,45~50,1292.9(1268.96to1317.3)

Uruguay,Prevalence,50~55,2096.88(2064.2to2130.09)

Uruguay,Prevalence,55~60,3207.37(3163.24to3252.12)

Uruguay,Prevalence,60~65,4587.46(4529.25to4646.42)

Uruguay,Prevalence,65~70,6422.7(6344.77to6501.6)

Uruguay,Prevalence,70~75,8723.86(8618.67to8830.32)

Uruguay,Prevalence,75~80,11522.16(11366.98to11679.46)

Uruguay,Prevalence,80~85,14939.61(14730.02to15152.19)

Uruguay,Prevalence,85~90,18785.19(18497.74to19077.1)

Uruguay,Prevalence,90~95,22389.75(21971.1to22816.38)

Uruguay,Deaths,20~25,0.64(0.29to1.38)

Uruguay,Deaths,25~30,0.81(0.42to1.57)

Uruguay,Deaths,30~35,1.16(0.68to2.01)

Uruguay,Deaths,35~40,1.87(1.21to2.88)

Uruguay,Deaths,40~45,3.5(2.54to4.84)

Uruguay,Deaths,45~50,7.52(5.98to9.46)

Uruguay,Deaths,50~55,17.18(14.59to20.24)

Uruguay,Deaths,55~60,34.32(30.18to39.01)

Uruguay,Deaths,60~65,65.88(59.27to73.22)

Uruguay,Deaths,65~70,115.67(105.28to127.08)

Uruguay,Deaths,70~75,193.63(176.85to211.99)

Uruguay,Deaths,75~80,296.35(268.08to327.61)

Uruguay,Deaths,80~85,459.55(415.27to508.55)

Uruguay,Deaths,85~90,701.89(632.2to779.26)

Uruguay,Deaths,90~95,1199.19(1072.37to1341.01)

Uruguay,DALYs,20~25,52.49(46.51to59.23)

Uruguay,DALYs,25~30,63.45(57.07to70.55)

Uruguay,DALYs,30~35,84.19(76.92to92.15)

Uruguay,DALYs,35~40,121.14(112.29to130.69)

Uruguay,DALYs,40~45,198.52(186.92to210.83)

Uruguay,DALYs,45~50,371.35(354.62to388.87)

Uruguay,DALYs,50~55,733.73(707.99to760.42)

Uruguay,DALYs,55~60,1268.02(1230.51to1306.66)

Uruguay,DALYs,60~65,2068.9(2015.01to2124.23)

Uruguay,DALYs,65~70,3044.15(2970.05to3120.09)

Uruguay,DALYs,70~75,4179.05(4077.69to4282.93)

Uruguay,DALYs,75~80,5130.32(4989.99to5274.61)

Uruguay,DALYs,80~85,6249.15(6070.01to6433.57)

Uruguay,DALYs,85~90,7595.72(7357.93to7841.19)

Uruguay,DALYs,90~95,11105.98(10702.45to11524.72)

Canada,Prevalence,20~25,234.5(213.19to257.93)

Canada,Prevalence,25~30,293.05(271.19to316.68)

Canada,Prevalence,30~35,388.94(365.56to413.81)

Canada,Prevalence,35~40,539.65(513.35to567.29)

Canada,Prevalence,40~45,824.16(792.5to857.09)

Canada,Prevalence,45~50,1559.66(1514.43to1606.24)

Canada,Prevalence,50~55,2830.31(2765.21to2896.95)

Canada,Prevalence,55~60,4452.95(4365.13to4542.52)

Canada,Prevalence,60~65,6722.94(6604.45to6843.56)

Canada,Prevalence,65~70,10823.99(10648.31to11002.57)

Canada,Prevalence,70~75,15844.63(15591.91to16101.45)

Canada,Prevalence,75~80,20578.74(20198.36to20966.28)

Canada,Prevalence,80~85,24133.33(23657.83to24618.38)

Canada,Prevalence,85~90,26137.56(25559.8to26728.38)

Canada,Prevalence,90~95,26455.29(25707.77to27224.54)

Canada,Deaths,20~25,0.05(0.02to0.12)

Canada,Deaths,25~30,0.07(0.04to0.14)

Canada,Deaths,30~35,0.13(0.08to0.21)

Canada,Deaths,35~40,0.26(0.18to0.36)

Canada,Deaths,40~45,0.65(0.53to0.8)

Canada,Deaths,45~50,1.74(1.53to1.98)

Canada,Deaths,50~55,4.84(4.45to5.27)

Canada,Deaths,55~60,11.76(11.09to12.48)

Canada,Deaths,60~65,27.55(26.35to28.81)

Canada,Deaths,65~70,55.06(53.04to57.16)

Canada,Deaths,70~75,102.76(99.21to106.44)

Canada,Deaths,75~80,174.05(167.35to181.02)

Canada,Deaths,80~85,263.51(253.29to274.15)

Canada,Deaths,85~90,411.46(395.24to428.36)

Canada,Deaths,90~95,575.2(551.06to600.39)

Canada,DALYs,20~25,13.95(12.32to15.79)

Canada,DALYs,25~30,17.37(15.7to19.23)

Canada,DALYs,30~35,24.29(22.43to26.29)

Canada,DALYs,35~40,37.52(35.29to39.88)

Canada,DALYs,40~45,68.19(65.21to71.3)

Canada,DALYs,45~50,145.84(141.32to150.51)

Canada,DALYs,50~55,315.58(308.38to322.94)

Canada,DALYs,55~60,601.39(590.49to612.49)

Canada,DALYs,60~65,1110.09(1093.26to1127.19)

Canada,DALYs,65~70,1844.92(1819.85to1870.34)

Canada,DALYs,70~75,2795.61(2758.56to2833.16)

Canada,DALYs,75~80,3737.21(3680.81to3794.48)

Canada,DALYs,80~85,4398.42(4329.09to4468.85)

Canada,DALYs,85~90,5309.71(5220.12to5400.84)

Canada,DALYs,90~95,6290.91(6166.69to6417.64)

Switzerland,Prevalence,20~25,560.67(538.93to583.27)

Switzerland,Prevalence,25~30,765.7(742.14to790)

Switzerland,Prevalence,30~35,971.41(946.19to997.3)

Switzerland,Prevalence,35~40,1169.84(1143.04to1197.27)

Switzerland,Prevalence,40~45,1502.69(1473.29to1532.67)

Switzerland,Prevalence,45~50,2408.9(2370.51to2447.91)

Switzerland,Prevalence,50~55,3848.27(3796.65to3900.6)

Switzerland,Prevalence,55~60,5476.14(5410.12to5542.97)

Switzerland,Prevalence,60~65,7290.59(7207.63to7374.49)

Switzerland,Prevalence,65~70,9820.83(9713.27to9929.57)

Switzerland,Prevalence,70~75,13217.83(13076.05to13361.14)

Switzerland,Prevalence,75~80,17665.03(17453.25to17879.38)

Switzerland,Prevalence,80~85,23712.15(23418.52to24009.45)

Switzerland,Prevalence,85~90,31686.17(31270.78to32107.07)

Switzerland,Prevalence,90~95,40136.36(39532.01to40749.95)

Switzerland,Deaths,20~25,0.16(0.04to0.67)

Switzerland,Deaths,25~30,0.16(0.05to0.53)

Switzerland,Deaths,30~35,0.2(0.08to0.52)

Switzerland,Deaths,35~40,0.33(0.16to0.66)

Switzerland,Deaths,40~45,0.69(0.43to1.1)

Switzerland,Deaths,45~50,1.69(1.26to2.28)

Switzerland,Deaths,50~55,4.23(3.48to5.13)

Switzerland,Deaths,55~60,9.17(7.97to10.55)

Switzerland,Deaths,60~65,20.87(18.74to23.23)

Switzerland,Deaths,65~70,37.43(34.05to41.13)

Switzerland,Deaths,70~75,65.05(59.56to71.04)

Switzerland,Deaths,75~80,104.42(94.81to115)

Switzerland,Deaths,80~85,156.16(141.77to172.02)

Switzerland,Deaths,85~90,232.03(210.35to255.94)

Switzerland,Deaths,90~95,328.33(296.15to364.02)

Switzerland,DALYs,20~25,34.99(30.16to40.6)

Switzerland,DALYs,25~30,44.21(39.27to49.76)

Switzerland,DALYs,30~35,54.72(49.59to60.38)

Switzerland,DALYs,35~40,68.36(62.85to74.36)

Switzerland,DALYs,40~45,96.74(90.36to103.57)

Switzerland,DALYs,45~50,173.84(165.06to183.09)

Switzerland,DALYs,50~55,321.55(308.91to334.7)

Switzerland,DALYs,55~60,534.22(516.64to552.4)

Switzerland,DALYs,60~65,908.03(882.05to934.76)

Switzerland,DALYs,65~70,1328.11(1292.62to1364.58)

Switzerland,DALYs,70~75,1867.57(1819.28to1917.15)

Switzerland,DALYs,75~80,2413.66(2344.32to2485.05)

Switzerland,DALYs,80~85,2908.31(2822.13to2997.12)

Switzerland,DALYs,85~90,3525.12(3414.88to3638.92)

Switzerland,DALYs,90~95,4347.41(4193.14to4507.35)

India,Prevalence,20~25,502.8(499.11to506.52)

India,Prevalence,25~30,695.08(690.66to699.53)

India,Prevalence,30~35,889.1(884.03to894.19)

India,Prevalence,35~40,1077.96(1072.31to1083.64)

India,Prevalence,40~45,1473.88(1467.04to1480.75)

India,Prevalence,45~50,2697.22(2686.72to2707.76)

India,Prevalence,50~55,4383(4367.89to4398.17)

India,Prevalence,55~60,6055.39(6036.09to6074.75)

India,Prevalence,60~65,8216.1(8191.62to8240.66)

India,Prevalence,65~70,12564.05(12528.53to12599.67)

India,Prevalence,70~75,17935.62(17884.15to17987.24)

India,Prevalence,75~80,23836.83(23754.36to23919.59)

India,Prevalence,80~85,29343.99(29228.13to29460.32)

India,Prevalence,85~90,35650.96(35473.55to35829.26)

India,Prevalence,90~95,42363.04(42048.36to42680.07)

India,Deaths,20~25,0.81(0.53to1.24)

India,Deaths,25~30,1.13(0.8to1.59)

India,Deaths,30~35,1.98(1.54to2.55)

India,Deaths,35~40,3.76(3.13to4.53)

India,Deaths,40~45,8(7to9.15)

India,Deaths,45~50,18.69(17to20.54)

India,Deaths,50~55,46.43(43.4to49.67)

India,Deaths,55~60,96.55(91.59to101.78)

India,Deaths,60~65,213.82(205.22to222.78)

India,Deaths,65~70,384.23(370.34to398.64)

India,Deaths,70~75,686.81(663.27to711.18)

India,Deaths,75~80,1062.89(1020.04to1107.55)

India,Deaths,80~85,1747.02(1673.47to1823.79)

India,Deaths,85~90,2326.23(2215.56to2442.43)

India,Deaths,90~95,3103.21(2913.92to3304.79)

India,DALYs,20~25,121.97(105.75to140.66)

India,DALYs,25~30,161.24(142.98to181.83)

India,DALYs,30~35,223.8(202.26to247.63)

India,DALYs,35~40,324.01(297.52to352.85)

India,DALYs,40~45,546.95(510.67to585.81)

India,DALYs,45~50,1094.93(1038.1to1154.88)

India,DALYs,50~55,2228.91(2136.38to2325.45)

India,DALYs,55~60,3842.67(3706.17to3984.2)

India,DALYs,60~65,6976.69(6766.55to7193.36)

India,DALYs,65~70,10571.63(10271.56to10880.46)

India,DALYs,70~75,15483.35(15048.06to15931.23)

India,DALYs,75~80,19225.79(18571.54to19903.08)

India,DALYs,80~85,24603.76(23675.76to25568.14)

India,DALYs,85~90,26288.58(25029.26to27611.25)

India,DALYs,90~95,30337.9(28182.04to32658.67)

Pakistan,Prevalence,20~25,489.88(483.51to496.34)

Pakistan,Prevalence,25~30,670.39(662.65to678.23)

Pakistan,Prevalence,30~35,859.28(850.14to868.52)

Pakistan,Prevalence,35~40,1045.88(1035.46to1056.39)

Pakistan,Prevalence,40~45,1355.53(1343.37to1367.8)

Pakistan,Prevalence,45~50,2206.44(2189.23to2223.78)

Pakistan,Prevalence,50~55,3616.94(3592.01to3642.03)

Pakistan,Prevalence,55~60,5181.75(5148.36to5215.35)

Pakistan,Prevalence,60~65,7042.34(6998.5to7086.45)

Pakistan,Prevalence,65~70,10320.8(10258.37to10383.62)

Pakistan,Prevalence,70~75,14993.19(14901.21to15085.75)

Pakistan,Prevalence,75~80,20334.33(20187.82to20481.91)

Pakistan,Prevalence,80~85,25626.78(25419.36to25835.9)

Pakistan,Prevalence,85~90,31474.08(31162.54to31788.74)

Pakistan,Prevalence,90~95,37748.09(37214.6to38289.22)

Pakistan,Deaths,20~25,0.63(0.56to0.7)

Pakistan,Deaths,25~30,1.2(1.1to1.31)

Pakistan,Deaths,30~35,2.03(1.89to2.18)

Pakistan,Deaths,35~40,3.44(3.25to3.64)

Pakistan,Deaths,40~45,6.66(6.38to6.95)

Pakistan,Deaths,45~50,14.27(13.82to14.72)

Pakistan,Deaths,50~55,33.24(32.48to34.02)

Pakistan,Deaths,55~60,65.45(64.24to66.69)

Pakistan,Deaths,60~65,145.88(143.65to148.14)

Pakistan,Deaths,65~70,260.72(257.08to264.41)

Pakistan,Deaths,70~75,476.88(470.49to483.35)

Pakistan,Deaths,75~80,743.12(731.83to754.59)

Pakistan,Deaths,80~85,1205.33(1186.41to1224.56)

Pakistan,Deaths,85~90,1609.73(1581.85to1638.11)

Pakistan,Deaths,90~95,2009.44(1965.97to2053.87)

Pakistan,DALYs,20~25,110(106.97to113.11)

Pakistan,DALYs,25~30,165.19(161.36to169.12)

Pakistan,DALYs,30~35,229.69(224.98to234.49)

Pakistan,DALYs,35~40,314.51(308.78to320.34)

Pakistan,DALYs,40~45,485.47(478.05to493.02)

Pakistan,DALYs,45~50,879.64(868.72to890.71)

Pakistan,DALYs,50~55,1696.98(1679.83to1714.31)

Pakistan,DALYs,55~60,2795(2770.14to2820.09)

Pakistan,DALYs,60~65,5025.04(4985.3to5065.09)

Pakistan,DALYs,65~70,7523.72(7466.59to7581.29)

Pakistan,DALYs,70~75,11243.82(11158.45to11329.84)

Pakistan,DALYs,75~80,14135.81(14010.38to14262.37)

Pakistan,DALYs,80~85,17874.02(17699.72to18050.04)

Pakistan,DALYs,85~90,19237.49(19006.12to19471.68)

Pakistan,DALYs,90~95,21038.38(20671.98to21411.27)

Nauru,Prevalence,20~25,548.35(325.14to924.8)

Nauru,Prevalence,25~30,759.08(477.2to1207.48)

Nauru,Prevalence,30~35,962.09(628.42to1472.95)

Nauru,Prevalence,35~40,1151.83(771.3to1720.1)

Nauru,Prevalence,40~45,1422.23(989.01to2045.21)

Nauru,Prevalence,45~50,2072.48(1486.53to2889.39)

Nauru,Prevalence,50~55,3138.85(2297.19to4288.88)

Nauru,Prevalence,55~60,4363.7(3211.6to5929.11)

Nauru,Prevalence,60~65,5752.54(4246.9to7791.98)

Nauru,Prevalence,65~70,7793.03(5737.64to10584.73)

Nauru,Prevalence,70~75,10592.85(7679.72to14611.02)

Nauru,Prevalence,75~80,13845.44(9430.91to20326.38)

Nauru,Prevalence,80~85,17332.59(10899.64to27562.26)

Nauru,Prevalence,85~90,20904.29(10899.35to40093.13)

Nauru,Prevalence,90~95,23550.13(7096.41to78153.33)

Nauru,Deaths,20~25,4.62(0.01to1578.48)

Nauru,Deaths,25~30,5.73(0.04to925.64)

Nauru,Deaths,30~35,9.56(0.16to573.38)

Nauru,Deaths,35~40,12.52(0.34to467.88)

Nauru,Deaths,40~45,22.3(1.26to394.35)

Nauru,Deaths,45~50,33.54(2.77to406.81)

Nauru,Deaths,50~55,61.74(7.39to515.57)

Nauru,Deaths,55~60,85(11.15to648.02)

Nauru,Deaths,60~65,164.9(27.81to977.67)

Nauru,Deaths,65~70,285.69(52.54to1553.51)

Nauru,Deaths,70~75,447.45(82.31to2432.46)

Nauru,Deaths,75~80,682.38(101.86to4571.15)

Nauru,Deaths,80~85,1007.46(126.17to8044.55)

Nauru,Deaths,85~90,1390.59(110.52to17496.55)

Nauru,Deaths,90~95,1884.29(39.43to90054.37)

Nauru,DALYs,20~25,353.32(174.54to715.23)

Nauru,DALYs,25~30,420.02(223.01to791.07)

Nauru,DALYs,30~35,625.21(363.4to1075.62)

Nauru,DALYs,35~40,748.2(453.58to1234.19)

Nauru,DALYs,40~45,1168.62(769.71to1774.26)

Nauru,DALYs,45~50,1593.56(1090.07to2329.59)

Nauru,DALYs,50~55,2592.8(1842.2to3649.24)

Nauru,DALYs,55~60,3176.77(2256.04to4473.28)

Nauru,DALYs,60~65,5207.71(3790.94to7153.96)

Nauru,DALYs,65~70,7556.92(5510.64to10363.04)

Nauru,DALYs,70~75,9759.63(7010.34to13587.13)

Nauru,DALYs,75~80,11938.45(8086.6to17625.04)

Nauru,DALYs,80~85,13849.61(8674.85to22111.24)

Nauru,DALYs,85~90,15200.55(7838.21to29478.27)

Nauru,DALYs,90~95,17663.74(5404.42to57731.97)

Marshall_Islands,Prevalence,20~25,561.28(442.83to711.43)

Marshall_Islands,Prevalence,25~30,781.5(631.66to966.89)

Marshall_Islands,Prevalence,30~35,993.54(816.46to1209.03)

Marshall_Islands,Prevalence,35~40,1189.55(989.1to1430.62)

Marshall_Islands,Prevalence,40~45,1455.42(1229.5to1722.85)

Marshall_Islands,Prevalence,45~50,2066.28(1769.11to2413.36)

Marshall_Islands,Prevalence,50~55,3058.69(2649.39to3531.22)

Marshall_Islands,Prevalence,55~60,4210.37(3668.72to4831.98)

Marshall_Islands,Prevalence,60~65,5522.64(4815.59to6333.51)

Marshall_Islands,Prevalence,65~70,7299.09(6339.63to8403.75)

Marshall_Islands,Prevalence,70~75,9700.87(8322.88to11307)

Marshall_Islands,Prevalence,75~80,12550.3(10361.6to15201.34)

Marshall_Islands,Prevalence,80~85,15701.33(12418.15to19852.54)

Marshall_Islands,Prevalence,85~90,18895.63(13678.46to26102.72)

Marshall_Islands,Prevalence,90~95,21225.42(12024.62to37466.35)

Marshall_Islands,Deaths,20~25,4.81(0.36to65.1)

Marshall_Islands,Deaths,25~30,5.76(0.55to60.39)

Marshall_Islands,Deaths,30~35,9.43(1.35to65.82)

Marshall_Islands,Deaths,35~40,11.51(1.97to67.36)

Marshall_Islands,Deaths,40~45,19.34(4.63to80.85)

Marshall_Islands,Deaths,45~50,30.02(8.7to103.53)

Marshall_Islands,Deaths,50~55,54.53(19.23to154.65)

Marshall_Islands,Deaths,55~60,77.67(29.61to203.77)

Marshall_Islands,Deaths,60~65,149.31(64.08to347.87)

Marshall_Islands,Deaths,65~70,240.91(106.73to543.76)

Marshall_Islands,Deaths,70~75,386.2(169.92to877.78)

Marshall_Islands,Deaths,75~80,613.81(233.76to1611.71)

Marshall_Islands,Deaths,80~85,921.34(317.35to2674.85)

Marshall_Islands,Deaths,85~90,1404.93(396.82to4974.12)

Marshall_Islands,Deaths,90~95,2063.38(335.58to12687.19)

Marshall_Islands,DALYs,20~25,364.98(265.36to501.99)

Marshall_Islands,DALYs,25~30,421.74(314.3to565.92)

Marshall_Islands,DALYs,30~35,616.63(476.73to797.58)

Marshall_Islands,DALYs,35~40,694.94(545.52to885.28)

Marshall_Islands,DALYs,40~45,1026.31(834.92to1261.57)

Marshall_Islands,DALYs,45~50,1434.86(1189.74to1730.48)

Marshall_Islands,DALYs,50~55,2297.05(1943.85to2714.42)

Marshall_Islands,DALYs,55~60,2905.87(2471.85to3416.09)

Marshall_Islands,DALYs,60~65,4715.46(4056.25to5481.79)

Marshall_Islands,DALYs,65~70,6396.88(5491.17to7451.97)

Marshall_Islands,DALYs,70~75,8434.75(7164.55to9930.13)

Marshall_Islands,DALYs,75~80,10714.88(8756.87to13110.71)

Marshall_Islands,DALYs,80~85,12611.1(9860.01to16129.78)

Marshall_Islands,DALYs,85~90,15174.92(10876.22to21172.63)

Marshall_Islands,DALYs,90~95,19032.36(10964.01to33038.17)

Papua_New_Guinea,Prevalence,20~25,576.98(565.17to589.04)

Papua_New_Guinea,Prevalence,25~30,801.77(787.35to816.45)

Papua_New_Guinea,Prevalence,30~35,1015.76(998.97to1032.84)

Papua_New_Guinea,Prevalence,35~40,1213.3(1194.41to1232.5)

Papua_New_Guinea,Prevalence,40~45,1578.11(1555.71to1600.84)

Papua_New_Guinea,Prevalence,45~50,2678.12(2644.67to2712)

Papua_New_Guinea,Prevalence,50~55,4291.31(4242.6to4340.58)

Papua_New_Guinea,Prevalence,55~60,5760.97(5698.4to5824.23)

Papua_New_Guinea,Prevalence,60~65,7440.39(7360.59to7521.07)

Papua_New_Guinea,Prevalence,65~70,10850.72(10735.47to10967.21)

Papua_New_Guinea,Prevalence,70~75,15049.34(14883.16to15217.38)

Papua_New_Guinea,Prevalence,75~80,19387.08(19125.9to19651.83)

Papua_New_Guinea,Prevalence,80~85,22818.08(22451.28to23190.88)

Papua_New_Guinea,Prevalence,85~90,26252.93(25689.08to26829.17)

Papua_New_Guinea,Prevalence,90~95,27750.87(26761.71to28776.58)

Papua_New_Guinea,Deaths,20~25,5.24(4.19to6.56)

Papua_New_Guinea,Deaths,25~30,5.42(4.42to6.63)

Papua_New_Guinea,Deaths,30~35,7.51(6.3to8.95)

Papua_New_Guinea,Deaths,35~40,9.53(8.14to11.16)

Papua_New_Guinea,Deaths,40~45,17.74(15.67to20.09)

Papua_New_Guinea,Deaths,45~50,33.53(30.36to37.04)

Papua_New_Guinea,Deaths,50~55,78(72.26to84.19)

Papua_New_Guinea,Deaths,55~60,143.22(134.26to152.77)

Papua_New_Guinea,Deaths,60~65,314.07(297.6to331.45)

Papua_New_Guinea,Deaths,65~70,584.64(556.54to614.16)

Papua_New_Guinea,Deaths,70~75,951.55(906.55to998.78)

Papua_New_Guinea,Deaths,75~80,1494.38(1411.83to1581.76)

Papua_New_Guinea,Deaths,80~85,2250.01(2116.76to2391.65)

Papua_New_Guinea,Deaths,85~90,3239.45(3018.69to3476.36)

Papua_New_Guinea,Deaths,90~95,4420.54(4016.89to4864.75)

Papua_New_Guinea,DALYs,20~25,397.86(387.16to408.85)

Papua_New_Guinea,DALYs,25~30,405.48(395.48to415.74)

Papua_New_Guinea,DALYs,30~35,515.31(503.86to527.03)

Papua_New_Guinea,DALYs,35~40,599.59(587.15to612.29)

Papua_New_Guinea,DALYs,40~45,968.81(951.91to986)

Papua_New_Guinea,DALYs,45~50,1643.57(1619.18to1668.34)

Papua_New_Guinea,DALYs,50~55,3294.94(3254.39to3335.98)

Papua_New_Guinea,DALYs,55~60,5215.59(5157.82to5274)

Papua_New_Guinea,DALYs,60~65,9606.66(9511.99to9702.27)

Papua_New_Guinea,DALYs,65~70,15008.75(14866.75to15152.11)

Papua_New_Guinea,DALYs,70~75,20103.2(19907.12to20301.2)

Papua_New_Guinea,DALYs,75~80,25306.14(25006.3to25609.58)

Papua_New_Guinea,DALYs,80~85,29814.06(29402.17to30231.73)

Papua_New_Guinea,DALYs,85~90,34022.32(33405.65to34650.38)

Papua_New_Guinea,DALYs,90~95,39955.09(38822.56to41120.66)

Comoros,Prevalence,20~25,427.05(392.86to464.22)

Comoros,Prevalence,25~30,561.96(521.45to605.61)

Comoros,Prevalence,30~35,716.35(668.46to767.67)

Comoros,Prevalence,35~40,881.81(826.31to941.03)

Comoros,Prevalence,40~45,1113.16(1049.01to1181.24)

Comoros,Prevalence,45~50,1575.25(1492.09to1663.03)

Comoros,Prevalence,50~55,2307.98(2196.21to2425.44)

Comoros,Prevalence,55~60,3255.52(3107.86to3410.21)

Comoros,Prevalence,60~65,4326.82(4137.1to4525.23)

Comoros,Prevalence,65~70,5529.95(5288.23to5782.72)

Comoros,Prevalence,70~75,7029.31(6711.35to7362.32)

Comoros,Prevalence,75~80,8860.26(8378.47to9369.75)

Comoros,Prevalence,80~85,10959.31(10272.18to11692.4)

Comoros,Prevalence,85~90,12646.47(11611.4to13773.81)

Comoros,Prevalence,90~95,13488.78(11726.4to15516.02)

Comoros,Deaths,20~25,0.85(0.13to5.68)

Comoros,Deaths,25~30,0.93(0.16to5.25)

Comoros,Deaths,30~35,1.47(0.35to6.21)

Comoros,Deaths,35~40,2.38(0.74to7.73)

Comoros,Deaths,40~45,3.4(1.25to9.29)

Comoros,Deaths,45~50,7.47(3.57to15.66)

Comoros,Deaths,50~55,13.07(7.18to23.78)

Comoros,Deaths,55~60,28.46(17.88to45.31)

Comoros,Deaths,60~65,55.75(37.78to82.28)

Comoros,Deaths,65~70,94.04(65.98to134.05)

Comoros,Deaths,70~75,159.61(113.97to223.53)

Comoros,Deaths,75~80,216.13(146.09to319.77)

Comoros,Deaths,80~85,319.63(211.82to482.31)

Comoros,Deaths,85~90,389.78(242.25to627.16)

Comoros,Deaths,90~95,525.64(282.27to978.84)

Comoros,DALYs,20~25,104.77(87.95to124.82)

Comoros,DALYs,25~30,122.1(104.16to143.13)

Comoros,DALYs,30~35,163.11(141.43to188.12)

Comoros,DALYs,35~40,217.71(191.51to247.51)

Comoros,DALYs,40~45,276.97(246.77to310.86)

Comoros,DALYs,45~50,474.88(431.18to523.02)

Comoros,DALYs,50~55,719.22(660.6to783.03)

Comoros,DALYs,55~60,1254.39(1165.75to1349.78)

Comoros,DALYs,60~65,2008.21(1879.52to2145.71)

Comoros,DALYs,65~70,2801.82(2629.37to2985.58)

Comoros,DALYs,70~75,3855.03(3618.75to4106.73)

Comoros,DALYs,75~80,4257(3944.38to4594.38)

Comoros,DALYs,80~85,4953.18(4543.61to5399.67)

Comoros,DALYs,85~90,4887.67(4359.97to5479.24)

Comoros,DALYs,90~95,5611.87(4711.51to6684.29)

Northern_Mariana_Islands,Prevalence,20~25,489.69(382.71to626.57)

Northern_Mariana_Islands,Prevalence,25~30,653.23(537.92to793.28)

Northern_Mariana_Islands,Prevalence,30~35,825.37(696.96to977.42)

Northern_Mariana_Islands,Prevalence,35~40,991.13(849.13to1156.88)

Northern_Mariana_Islands,Prevalence,40~45,1224.53(1067.68to1404.42)

Northern_Mariana_Islands,Prevalence,45~50,1728.33(1522.68to1961.77)

Northern_Mariana_Islands,Prevalence,50~55,2529.53(2248.79to2845.31)

Northern_Mariana_Islands,Prevalence,55~60,3586.81(3198.17to4022.68)

Northern_Mariana_Islands,Prevalence,60~65,4974.34(4429.79to5585.84)

Northern_Mariana_Islands,Prevalence,65~70,6876.83(6093.9to7760.35)

Northern_Mariana_Islands,Prevalence,70~75,9520.89(8369.58to10830.57)

Northern_Mariana_Islands,Prevalence,75~80,12980.66(11056.14to15240.19)

Northern_Mariana_Islands,Prevalence,80~85,17339.82(14472.71to20774.9)

Northern_Mariana_Islands,Prevalence,85~90,22562.45(17960.9to28342.91)

Northern_Mariana_Islands,Prevalence,90~95,27889.17(19816.88to39249.67)

Northern_Mariana_Islands,Deaths,20~25,1.53(0.02to123.72)

Northern_Mariana_Islands,Deaths,25~30,2.05(0.08to52.99)

Northern_Mariana_Islands,Deaths,30~35,3.46(0.31to38.55)

Northern_Mariana_Islands,Deaths,35~40,4.55(0.58to35.68)

Northern_Mariana_Islands,Deaths,40~45,7.72(1.54to38.59)

Northern_Mariana_Islands,Deaths,45~50,13.6(3.52to52.49)

Northern_Mariana_Islands,Deaths,50~55,26.24(8.51to80.88)

Northern_Mariana_Islands,Deaths,55~60,40.85(14.24to117.18)

Northern_Mariana_Islands,Deaths,60~65,82(32.35to207.87)

Northern_Mariana_Islands,Deaths,65~70,137.61(56.29to336.42)

Northern_Mariana_Islands,Deaths,70~75,251.17(107.05to589.28)

Northern_Mariana_Islands,Deaths,75~80,430.89(155.05to1197.48)

Northern_Mariana_Islands,Deaths,80~85,678.84(232.04to1985.98)

Northern_Mariana_Islands,Deaths,85~90,1037.63(310.99to3462.1)

Northern_Mariana_Islands,Deaths,90~95,1569.88(349.96to7042.24)

Northern_Mariana_Islands,DALYs,20~25,147.64(92.5to235.64)

Northern_Mariana_Islands,DALYs,25~30,187.36(130.48to269.03)

Northern_Mariana_Islands,DALYs,30~35,270.28(201.72to362.14)

Northern_Mariana_Islands,DALYs,35~40,323.26(248.54to420.45)

Northern_Mariana_Islands,DALYs,40~45,470.3(378.04to585.07)

Northern_Mariana_Islands,DALYs,45~50,722.42(594.2to878.31)

Northern_Mariana_Islands,DALYs,50~55,1207.6(1015.78to1435.65)

Northern_Mariana_Islands,DALYs,55~60,1639.65(1383.71to1942.93)

Northern_Mariana_Islands,DALYs,60~65,2748.07(2341.85to3224.74)

Northern_Mariana_Islands,DALYs,65~70,3829.57(3249.54to4513.13)

Northern_Mariana_Islands,DALYs,70~75,5725.21(4847.39to6761.99)

Northern_Mariana_Islands,DALYs,75~80,7791.17(6335.27to9581.65)

Northern_Mariana_Islands,DALYs,80~85,9729.89(7707.42to12283.07)

Northern_Mariana_Islands,DALYs,85~90,11886.98(8861.73to15945)

Northern_Mariana_Islands,DALYs,90~95,15548.47(10219.96to23655.16)

Armenia,Prevalence,20~25,472.73(456.66to489.38)

Armenia,Prevalence,25~30,628.81(610.45to647.73)

Armenia,Prevalence,30~35,793.29(773.03to814.08)

Armenia,Prevalence,35~40,965.83(943.67to988.5)

Armenia,Prevalence,40~45,1231.26(1206.17to1256.87)

Armenia,Prevalence,45~50,1856.04(1823.01to1889.66)

Armenia,Prevalence,50~55,2935.96(2891.42to2981.18)

Armenia,Prevalence,55~60,4383.1(4323.35to4443.67)

Armenia,Prevalence,60~65,6226.62(6148.3to6305.93)

Armenia,Prevalence,65~70,9016.47(8907.07to9127.22)

Armenia,Prevalence,70~75,13030.27(12872.4to13190.08)

Armenia,Prevalence,75~80,18389.95(18137.39to18646.03)

Armenia,Prevalence,80~85,24887.48(24531.75to25248.36)

Armenia,Prevalence,85~90,31464.89(30937.07to32001.73)

Armenia,Prevalence,90~95,36995.36(36111.71to37900.64)

Armenia,Deaths,20~25,3.11(1.52to6.39)

Armenia,Deaths,25~30,3.07(1.67to5.62)

Armenia,Deaths,30~35,3.6(2.23to5.83)

Armenia,Deaths,35~40,4.16(2.78to6.21)

Armenia,Deaths,40~45,5.69(4.14to7.83)

Armenia,Deaths,45~50,7.79(5.97to10.17)

Armenia,Deaths,50~55,12.61(10.29to15.45)

Armenia,Deaths,55~60,20.16(17.03to23.87)

Armenia,Deaths,60~65,33.18(28.68to38.38)

Armenia,Deaths,65~70,49.8(43.27to57.33)

Armenia,Deaths,70~75,73.07(63.53to84.03)

Armenia,Deaths,75~80,103.22(89.17to119.47)

Armenia,Deaths,80~85,148.95(128.54to172.6)

Armenia,Deaths,85~90,227.81(195.42to265.57)

Armenia,Deaths,90~95,285.14(238.69to340.62)

Armenia,DALYs,20~25,200.41(165.13to243.23)

Armenia,DALYs,25~30,208.7(176.37to246.97)

Armenia,DALYs,30~35,238.2(206.62to274.6)

Armenia,DALYs,35~40,260.94(230.21to295.76)

Armenia,DALYs,40~45,324.92(292.5to360.92)

Armenia,DALYs,45~50,420.24(383.1to460.98)

Armenia,DALYs,50~55,617.65(572.24to666.68)

Armenia,DALYs,55~60,875.76(818.61to936.91)

Armenia,DALYs,60~65,1241.35(1167.79to1319.53)

Armenia,DALYs,65~70,1608.91(1514.7to1708.98)

Armenia,DALYs,70~75,1998.44(1879.44to2124.97)

Armenia,DALYs,75~80,2332.82(2183.09to2492.81)

Armenia,DALYs,80~85,2695.83(2515.99to2888.52)

Armenia,DALYs,85~90,3271.5(3026.96to3535.8)

Armenia,DALYs,90~95,3569.82(3209.43to3970.68)

Eritrea,Prevalence,20~25,442.15(428.88to455.84)

Eritrea,Prevalence,25~30,587.98(572to604.41)

Eritrea,Prevalence,30~35,752.72(733.69to772.25)

Eritrea,Prevalence,35~40,923.77(901.66to946.41)

Eritrea,Prevalence,40~45,1167.36(1141.58to1193.71)

Eritrea,Prevalence,45~50,1687.25(1652.8to1722.42)

Eritrea,Prevalence,50~55,2511.89(2464.31to2560.39)

Eritrea,Prevalence,55~60,3534.59(3470.51to3599.86)

Eritrea,Prevalence,60~65,4607.58(4524.12to4692.58)

Eritrea,Prevalence,65~70,5740.11(5632.05to5850.24)

Eritrea,Prevalence,70~75,7065.57(6919.97to7214.23)

Eritrea,Prevalence,75~80,8638.61(8417.18to8865.88)

Eritrea,Prevalence,80~85,10279.05(9940.6to10629.02)

Eritrea,Prevalence,85~90,11384.91(10795.15to12006.88)

Eritrea,Prevalence,90~95,11661.79(10414.52to13058.45)

Eritrea,Deaths,20~25,1.24(0.71to2.16)

Eritrea,Deaths,25~30,1.3(0.77to2.17)

Eritrea,Deaths,30~35,2.47(1.65to3.69)

Eritrea,Deaths,35~40,4.01(2.87to5.6)

Eritrea,Deaths,40~45,6.69(5.09to8.8)

Eritrea,Deaths,45~50,15.79(12.88to19.36)

Eritrea,Deaths,50~55,25.42(21.36to30.25)

Eritrea,Deaths,55~60,52.19(45.19to60.27)

Eritrea,Deaths,60~65,93.07(81.75to105.95)

Eritrea,Deaths,65~70,145.82(128.66to165.28)

Eritrea,Deaths,70~75,235.04(207.62to266.09)

Eritrea,Deaths,75~80,305.86(263.77to354.68)

Eritrea,Deaths,80~85,448.41(379.56to529.75)

Eritrea,Deaths,85~90,547.93(436.99to687.03)

Eritrea,Deaths,90~95,652.77(434.67to980.29)

Eritrea,DALYs,20~25,131.58(124.38to139.2)

Eritrea,DALYs,25~30,147.85(140.38to155.71)

Eritrea,DALYs,30~35,222.3(212.49to232.55)

Eritrea,DALYs,35~40,305.61(293.49to318.23)

Eritrea,DALYs,40~45,435.34(420.13to451.09)

Eritrea,DALYs,45~50,835.42(811.25to860.31)

Eritrea,DALYs,50~55,1206.73(1175.04to1239.28)

Eritrea,DALYs,55~60,2075.77(2027.12to2125.59)

Eritrea,DALYs,60~65,3118.86(3049.33to3189.97)

Eritrea,DALYs,65~70,4089.38(3997.08to4183.81)

Eritrea,DALYs,70~75,5376.34(5249.34to5506.42)

Eritrea,DALYs,75~80,5690.73(5522.44to5864.16)

Eritrea,DALYs,80~85,6540.27(6298.17to6791.67)

Eritrea,DALYs,85~90,6405.25(6028.3to6805.77)

Eritrea,DALYs,90~95,6555.74(5780.04to7435.54)

Chile,Prevalence,20~25,234.5(227.6to241.6)

Chile,Prevalence,25~30,291(283.64to298.54)

Chile,Prevalence,30~35,368.05(359.96to376.33)

Chile,Prevalence,35~40,479.64(470.43to489.04)

Chile,Prevalence,40~45,659.91(649.1to670.9)

Chile,Prevalence,45~50,978.45(964.69to992.41)

Chile,Prevalence,50~55,1499.42(1481.27to1517.79)

Chile,Prevalence,55~60,2291.79(2267.14to2316.71)

Chile,Prevalence,60~65,3401.2(3367.68to3435.06)

Chile,Prevalence,65~70,4859.17(4813.53to4905.23)

Chile,Prevalence,70~75,6790.02(6727.58to6853.04)

Chile,Prevalence,75~80,9325.91(9226.92to9425.97)

Chile,Prevalence,80~85,12511.3(12371.57to12652.62)

Chile,Prevalence,85~90,16092.36(15896.12to16291.03)

Chile,Prevalence,90~95,19103.8(18813.65to19398.42)

Chile,Deaths,20~25,0.59(0.36to0.96)

Chile,Deaths,25~30,0.64(0.42to0.98)

Chile,Deaths,30~35,0.85(0.6to1.21)

Chile,Deaths,35~40,1.12(0.83to1.5)

Chile,Deaths,40~45,1.73(1.37to2.18)

Chile,Deaths,45~50,2.98(2.49to3.55)

Chile,Deaths,50~55,5.72(5.01to6.54)

Chile,Deaths,55~60,10.82(9.74to12.02)

Chile,Deaths,60~65,21.45(19.69to23.38)

Chile,Deaths,65~70,38.78(35.94to41.85)

Chile,Deaths,70~75,67.91(63.22to72.94)

Chile,Deaths,75~80,121.36(112.16to131.3)

Chile,Deaths,80~85,208.52(192.68to225.67)

Chile,Deaths,85~90,349.71(322.68to379.01)

Chile,Deaths,90~95,603.94(554.96to657.25)

Chile,DALYs,20~25,47.92(43.76to52.48)

Chile,DALYs,25~30,51.07(47.12to55.36)

Chile,DALYs,30~35,62.59(58.4to67.09)

Chile,DALYs,35~40,76.83(72.32to81.63)

Chile,DALYs,40~45,107.03(101.79to112.54)

Chile,DALYs,45~50,163.47(156.85to170.37)

Chile,DALYs,50~55,272.78(263.76to282.12)

Chile,DALYs,55~60,444.93(432.33to457.88)

Chile,DALYs,60~65,741.06(722.69to759.89)

Chile,DALYs,65~70,1116.71(1090.85to1143.18)

Chile,DALYs,70~75,1598.32(1562.29to1635.18)

Chile,DALYs,75~80,2268.82(2211.53to2327.6)

Chile,DALYs,80~85,3045.04(2965.58to3126.62)

Chile,DALYs,85~90,4034.74(3923.04to4149.61)

Chile,DALYs,90~95,5938.77(5752.85to6130.7)

Antigua_and_Barbuda,Prevalence,20~25,183.5(138.06to243.89)

Antigua_and_Barbuda,Prevalence,25~30,245.54(192.93to312.49)

Antigua_and_Barbuda,Prevalence,30~35,329.07(266.84to405.83)

Antigua_and_Barbuda,Prevalence,35~40,443.75(368.53to534.31)

Antigua_and_Barbuda,Prevalence,40~45,623.01(529.48to733.08)

Antigua_and_Barbuda,Prevalence,45~50,941.48(815.43to1087.02)

Antigua_and_Barbuda,Prevalence,50~55,1465.88(1289.2to1666.78)

Antigua_and_Barbuda,Prevalence,55~60,2264.45(2013.61to2546.53)

Antigua_and_Barbuda,Prevalence,60~65,3387.1(3037.28to3777.21)

Antigua_and_Barbuda,Prevalence,65~70,4889.84(4401.76to5432.03)

Antigua_and_Barbuda,Prevalence,70~75,7043.85(6334.94to7832.09)

Antigua_and_Barbuda,Prevalence,75~80,10116.19(8931.37to11458.18)

Antigua_and_Barbuda,Prevalence,80~85,14225.24(12441.49to16264.74)

Antigua_and_Barbuda,Prevalence,85~90,19497.39(16755.43to22688.06)

Antigua_and_Barbuda,Prevalence,90~95,26061.79(21413.38to31719.27)

Antigua_and_Barbuda,Deaths,20~25,0.04(0to858868.02)

Antigua_and_Barbuda,Deaths,25~30,0.04(0to230244.61)

Antigua_and_Barbuda,Deaths,30~35,0.06(0to16582.52)

Antigua_and_Barbuda,Deaths,35~40,0.12(0to810.59)

Antigua_and_Barbuda,Deaths,40~45,0.26(0to179.8)

Antigua_and_Barbuda,Deaths,45~50,0.67(0.01to70.71)

Antigua_and_Barbuda,Deaths,50~55,1.84(0.07to45.94)

Antigua_and_Barbuda,Deaths,55~60,5.16(0.5to53.42)

Antigua_and_Barbuda,Deaths,60~65,12.88(2.15to77.01)

Antigua_and_Barbuda,Deaths,65~70,23.4(4.81to113.87)

Antigua_and_Barbuda,Deaths,70~75,45.64(10.3to202.25)

Antigua_and_Barbuda,Deaths,75~80,86.48(15.52to481.77)

Antigua_and_Barbuda,Deaths,80~85,145.29(24.98to845.11)

Antigua_and_Barbuda,Deaths,85~90,241.46(38.46to1516.19)

Antigua_and_Barbuda,Deaths,90~95,376.34(47.82to2961.93)

Antigua_and_Barbuda,DALYs,20~25,10.44(3.35to32.51)

Antigua_and_Barbuda,DALYs,25~30,12.55(4.7to33.51)

Antigua_and_Barbuda,DALYs,30~35,16.68(7.2to38.68)

Antigua_and_Barbuda,DALYs,35~40,24.65(12.12to50.12)

Antigua_and_Barbuda,DALYs,40~45,37.61(20.59to68.7)

Antigua_and_Barbuda,DALYs,45~50,66.9(40.53to110.44)

Antigua_and_Barbuda,DALYs,50~55,128.04(84.96to192.96)

Antigua_and_Barbuda,DALYs,55~60,260.3(184.87to366.49)

Antigua_and_Barbuda,DALYs,60~65,501.37(374.6to671.05)

Antigua_and_Barbuda,DALYs,65~70,752.11(571.05to990.57)

Antigua_and_Barbuda,DALYs,70~75,1167.49(888.83to1533.51)

Antigua_and_Barbuda,DALYs,75~80,1740.16(1263.93to2395.81)

Antigua_and_Barbuda,DALYs,80~85,2318.38(1646.11to3265.2)

Antigua_and_Barbuda,DALYs,85~90,3078.26(2098.51to4515.42)

Antigua_and_Barbuda,DALYs,90~95,4146.75(2549.12to6745.69)

Central_African_Republic,Prevalence,20~25,424.59(411.6to437.99)

Central_African_Republic,Prevalence,25~30,574.12(558.4to590.28)

Central_African_Republic,Prevalence,30~35,745.49(726.65to764.82)

Central_African_Republic,Prevalence,35~40,925.83(903.74to948.45)

Central_African_Republic,Prevalence,40~45,1211.98(1185.53to1239.01)

Central_African_Republic,Prevalence,45~50,1952.43(1914.24to1991.39)

Central_African_Republic,Prevalence,50~55,3226.96(3169.81to3285.13)

Central_African_Republic,Prevalence,55~60,4745.16(4665.85to4825.82)

Central_African_Republic,Prevalence,60~65,6330.32(6225.45to6436.95)

Central_African_Republic,Prevalence,65~70,8289.3(8147.4to8433.67)

Central_African_Republic,Prevalence,70~75,10729.74(10528.88to10934.42)

Central_African_Republic,Prevalence,75~80,13715.42(13394.76to14043.77)

Central_African_Republic,Prevalence,80~85,17216.34(16704.88to17743.47)

Central_African_Republic,Prevalence,85~90,20413.41(19494.59to21375.53)

Central_African_Republic,Prevalence,90~95,22118.46(20182.16to24240.53)

Central_African_Republic,Deaths,20~25,0.72(0.38to1.37)

Central_African_Republic,Deaths,25~30,0.88(0.49to1.56)

Central_African_Republic,Deaths,30~35,1.97(1.29to3)

Central_African_Republic,Deaths,35~40,3.02(2.12to4.32)

Central_African_Republic,Deaths,40~45,8.1(6.33to10.38)

Central_African_Republic,Deaths,45~50,23.79(20.03to28.27)

Central_African_Republic,Deaths,50~55,38.76(33.42to44.96)

Central_African_Republic,Deaths,55~60,78.89(69.7to89.3)

Central_African_Republic,Deaths,60~65,143.69(128.57to160.6)

Central_African_Republic,Deaths,65~70,250.87(225.71to278.83)

Central_African_Republic,Deaths,70~75,396.89(356.43to441.95)

Central_African_Republic,Deaths,75~80,512.21(449.8to583.28)

Central_African_Republic,Deaths,80~85,715.23(615.18to831.55)

Central_African_Republic,Deaths,85~90,916.37(746.68to1124.63)

Central_African_Republic,Deaths,90~95,1210.25(856.62to1709.88)

Central_African_Republic,DALYs,20~25,93.93(88.42to99.79)

Central_African_Republic,DALYs,25~30,116.18(110.07to122.62)

Central_African_Republic,DALYs,30~35,189.99(181.54to198.83)

Central_African_Republic,DALYs,35~40,252.42(242.19to263.09)

Central_African_Republic,DALYs,40~45,502.8(486.54to519.6)

Central_African_Republic,DALYs,45~50,1200.68(1170.43to1231.7)

Central_African_Republic,DALYs,50~55,1780.76(1740.14to1822.33)

Central_African_Republic,DALYs,55~60,3085.01(3022.13to3149.2)

Central_African_Republic,DALYs,60~65,4744.7(4653.01to4838.19)

Central_African_Republic,DALYs,65~70,6892.68(6759.85to7028.12)

Central_African_Republic,DALYs,70~75,8972.68(8787.24to9162.04)

Central_African_Republic,DALYs,75~80,9469.25(9219.09to9726.2)

Central_African_Republic,DALYs,80~85,10492.47(10133.63to10864.02)

Central_African_Republic,DALYs,85~90,10808.53(10227.78to11422.25)

Central_African_Republic,DALYs,90~95,12187.73(10958.1to13555.35)

Puerto_Rico,Prevalence,20~25,247.67(238.81to256.85)

Puerto_Rico,Prevalence,25~30,341.62(331.17to352.39)

Puerto_Rico,Prevalence,30~35,465.89(453.64to478.46)

Puerto_Rico,Prevalence,35~40,623.76(609.53to638.33)

Puerto_Rico,Prevalence,40~45,867.94(851.26to884.94)

Puerto_Rico,Prevalence,45~50,1374.96(1353.06to1397.21)

Puerto_Rico,Prevalence,50~55,2213.61(2184.02to2243.6)

Puerto_Rico,Prevalence,55~60,3382.21(3342.56to3422.32)

Puerto_Rico,Prevalence,60~65,4890.93(4838.52to4943.91)

Puerto_Rico,Prevalence,65~70,6843.9(6773.87to6914.65)

Puerto_Rico,Prevalence,70~75,9606.95(9510.05to9704.83)

Puerto_Rico,Prevalence,75~80,13471.01(13314.59to13629.27)

Puerto_Rico,Prevalence,80~85,18648.67(18420.66to18879.51)

Puerto_Rico,Prevalence,85~90,24844.17(24514.3to25178.48)

Puerto_Rico,Prevalence,90~95,31547.48(31060.16to32042.45)

Puerto_Rico,Deaths,20~25,0.56(0.24to1.33)

Puerto_Rico,Deaths,25~30,0.7(0.33to1.47)

Puerto_Rico,Deaths,30~35,0.82(0.43to1.57)

Puerto_Rico,Deaths,35~40,1.4(0.85to2.3)

Puerto_Rico,Deaths,40~45,2.42(1.67to3.52)

Puerto_Rico,Deaths,45~50,4.47(3.39to5.91)

Puerto_Rico,Deaths,50~55,8.73(7.09to10.74)

Puerto_Rico,Deaths,55~60,15.25(12.93to17.99)

Puerto_Rico,Deaths,60~65,29.25(25.54to33.5)

Puerto_Rico,Deaths,65~70,46.98(41.51to53.17)

Puerto_Rico,Deaths,70~75,84.84(75.69to95.1)

Puerto_Rico,Deaths,75~80,142.2(125.26to161.43)

Puerto_Rico,Deaths,80~85,240.53(211.74to273.23)

Puerto_Rico,Deaths,85~90,400.14(351.61to455.37)

Puerto_Rico,Deaths,90~95,552.95(482.92to633.14)

Puerto_Rico,DALYs,20~25,47.19(41.32to53.89)

Puerto_Rico,DALYs,25~30,57.62(51.3to64.71)

Puerto_Rico,DALYs,30~35,66.94(60.39to74.2)

Puerto_Rico,DALYs,35~40,98.86(90.81to107.62)

Puerto_Rico,DALYs,40~45,149.68(139.83to160.23)

Puerto_Rico,DALYs,45~50,245.04(232.05to258.76)

Puerto_Rico,DALYs,50~55,417.08(399.11to435.87)

Puerto_Rico,DALYs,55~60,637.46(613.9to661.92)

Puerto_Rico,DALYs,60~65,1026.93(993.38to1061.62)

Puerto_Rico,DALYs,65~70,1396.94(1353.22to1442.07)

Puerto_Rico,DALYs,70~75,2056.85(1994.95to2120.66)

Puerto_Rico,DALYs,75~80,2766.72(2672.78to2863.96)

Puerto_Rico,DALYs,80~85,3668.13(3538.88to3802.09)

Puerto_Rico,DALYs,85~90,4837.84(4657.64to5025.01)

Puerto_Rico,DALYs,90~95,5845.09(5600.2to6100.68)

Georgia,Prevalence,20~25,390.41(375.56to405.85)

Georgia,Prevalence,25~30,538.97(521.62to556.9)

Georgia,Prevalence,30~35,701.93(682.22to722.21)

Georgia,Prevalence,35~40,877.36(855.27to900.03)

Georgia,Prevalence,40~45,1123.71(1098.81to1149.18)

Georgia,Prevalence,45~50,1626.16(1594.06to1658.9)

Georgia,Prevalence,50~55,2467.54(2425.29to2510.52)

Georgia,Prevalence,55~60,3677.41(3620.59to3735.12)

Georgia,Prevalence,60~65,5315(5239.44to5391.65)

Georgia,Prevalence,65~70,7582.83(7478.89to7688.21)

Georgia,Prevalence,70~75,10775.62(10626.19to10927.14)

Georgia,Prevalence,75~80,15087.47(14853.41to15325.23)

Georgia,Prevalence,80~85,20601.45(20268.98to20939.39)

Georgia,Prevalence,85~90,27091.32(26602.58to27589.05)

Georgia,Prevalence,90~95,34061.22(33325.45to34813.24)

Georgia,Deaths,20~25,0.23(0.08to0.64)

Georgia,Deaths,25~30,0.29(0.13to0.65)

Georgia,Deaths,30~35,0.38(0.2to0.74)

Georgia,Deaths,35~40,0.65(0.39to1.09)

Georgia,Deaths,40~45,1.15(0.76to1.73)

Georgia,Deaths,45~50,2.28(1.65to3.16)

Georgia,Deaths,50~55,4.74(3.72to6.05)

Georgia,Deaths,55~60,9.32(7.64to11.36)

Georgia,Deaths,60~65,20.09(17.13to23.56)

Georgia,Deaths,65~70,40.01(34.58to46.29)

Georgia,Deaths,70~75,83.4(72.54to95.89)

Georgia,Deaths,75~80,152.76(130.67to178.59)

Georgia,Deaths,80~85,239.71(204.05to281.6)

Georgia,Deaths,85~90,350.43(294.25to417.35)

Georgia,Deaths,90~95,416.57(338.91to512.02)

Georgia,DALYs,20~25,29.73(24.54to36.01)

Georgia,DALYs,25~30,38.43(32.74to45.11)

Georgia,DALYs,30~35,49.6(43.28to56.86)

Georgia,DALYs,35~40,69.31(61.64to77.93)

Georgia,DALYs,40~45,100.8(91.19to111.42)

Georgia,DALYs,45~50,166.11(152.37to181.09)

Georgia,DALYs,50~55,287.62(268.01to308.68)

Georgia,DALYs,55~60,473.24(444.77to503.53)

Georgia,DALYs,60~65,813.92(770.96to859.28)

Georgia,DALYs,65~70,1296.61(1230.61to1366.15)

Georgia,DALYs,70~75,2102.98(1996.18to2215.49)

Georgia,DALYs,75~80,3018.83(2845.03to3203.26)

Georgia,DALYs,80~85,3813.74(3580.56to4062.11)

Georgia,DALYs,85~90,4562.92(4238.51to4912.16)

Georgia,DALYs,90~95,5046.83(4586.3to5553.61)

Kyrgyzstan,Prevalence,20~25,672.68(647.54to698.8)

Kyrgyzstan,Prevalence,25~30,913.49(883.56to944.44)

Kyrgyzstan,Prevalence,30~35,1132.85(1099.08to1167.65)

Kyrgyzstan,Prevalence,35~40,1325.02(1287.99to1363.11)

Kyrgyzstan,Prevalence,40~45,1646.54(1604.67to1689.5)

Kyrgyzstan,Prevalence,45~50,2562.23(2504.21to2621.59)

Kyrgyzstan,Prevalence,50~55,4019.57(3938.54to4102.26)

Kyrgyzstan,Prevalence,55~60,5586.6(5480.65to5694.59)

Kyrgyzstan,Prevalence,60~65,7344.36(7209.88to7481.35)

Kyrgyzstan,Prevalence,65~70,10264.65(10079.13to10453.58)

Kyrgyzstan,Prevalence,70~75,14273.07(14008.65to14542.48)

Kyrgyzstan,Prevalence,75~80,19045.3(18644.91to19454.29)

Kyrgyzstan,Prevalence,80~85,24052.72(23516.32to24601.35)

Kyrgyzstan,Prevalence,85~90,28672.57(27915.06to29450.64)

Kyrgyzstan,Prevalence,90~95,31872.3(30694.71to33095.07)

Kyrgyzstan,Deaths,20~25,11.42(8.31to15.69)

Kyrgyzstan,Deaths,25~30,13.66(10.5to17.78)

Kyrgyzstan,Deaths,30~35,17.27(13.91to21.45)

Kyrgyzstan,Deaths,35~40,20.61(17.13to24.79)

Kyrgyzstan,Deaths,40~45,24.83(21.29to28.97)

Kyrgyzstan,Deaths,45~50,32.19(28.19to36.76)

Kyrgyzstan,Deaths,50~55,45.55(40.75to50.92)

Kyrgyzstan,Deaths,55~60,63.56(57.56to70.19)

Kyrgyzstan,Deaths,60~65,88.77(80.88to97.43)

Kyrgyzstan,Deaths,65~70,122.22(111.62to133.82)

Kyrgyzstan,Deaths,70~75,159.71(145.76to175)

Kyrgyzstan,Deaths,75~80,205.61(186.65to226.5)

Kyrgyzstan,Deaths,80~85,237.72(215.04to262.78)

Kyrgyzstan,Deaths,85~90,295.15(264.75to329.04)

Kyrgyzstan,Deaths,90~95,301.29(263.76to344.16)

Kyrgyzstan,DALYs,20~25,739.24(657.77to830.8)

Kyrgyzstan,DALYs,25~30,851.12(769.32to941.62)

Kyrgyzstan,DALYs,30~35,1003.97(919.69to1095.96)

Kyrgyzstan,DALYs,35~40,1110.76(1026.83to1201.54)

Kyrgyzstan,DALYs,40~45,1237.94(1156.63to1324.97)

Kyrgyzstan,DALYs,45~50,1493.4(1405.03to1587.33)

Kyrgyzstan,DALYs,50~55,1937.12(1834.99to2044.94)

Kyrgyzstan,DALYs,55~60,2405.8(2286.8to2530.99)

Kyrgyzstan,DALYs,60~65,2927.25(2785.74to3075.95)

Kyrgyzstan,DALYs,65~70,3447.65(3280.09to3623.78)

Kyrgyzstan,DALYs,70~75,3775.13(3583.53to3976.97)

Kyrgyzstan,DALYs,75~80,3957.83(3737.43to4191.23)

Kyrgyzstan,DALYs,80~85,3687.25(3462.62to3926.46)

Kyrgyzstan,DALYs,85~90,3677.3(3410.8to3964.62)

Kyrgyzstan,DALYs,90~95,3313.83(2977.75to3687.84)

Solomon_Islands,Prevalence,20~25,580.51(537.3to627.18)

Solomon_Islands,Prevalence,25~30,806.09(752.21to863.83)

Solomon_Islands,Prevalence,30~35,1022.83(959.21to1090.67)

Solomon_Islands,Prevalence,35~40,1224.69(1152.49to1301.4)

Solomon_Islands,Prevalence,40~45,1547.99(1463.79to1637.03)

Solomon_Islands,Prevalence,45~50,2450.47(2331.45to2575.56)

Solomon_Islands,Prevalence,50~55,3876.04(3705.86to4054.03)

Solomon_Islands,Prevalence,55~60,5290.95(5070.68to5520.79)

Solomon_Islands,Prevalence,60~65,6793.17(6515.53to7082.64)

Solomon_Islands,Prevalence,65~70,9315.16(8936.21to9710.17)

Solomon_Islands,Prevalence,70~75,12872.48(12332.7to13435.88)

Solomon_Islands,Prevalence,75~80,16950.08(16107.85to17836.36)

Solomon_Islands,Prevalence,80~85,21031.41(19807.07to22331.42)

Solomon_Islands,Prevalence,85~90,24971.64(22980.39to27135.44)

Solomon_Islands,Prevalence,90~95,27913.88(23916.1to32579.93)

Solomon_Islands,Deaths,20~25,1.39(0.35to5.49)

Solomon_Islands,Deaths,25~30,2.33(0.78to6.95)

Solomon_Islands,Deaths,30~35,4.01(1.65to9.79)

Solomon_Islands,Deaths,35~40,4.92(2.17to11.15)

Solomon_Islands,Deaths,40~45,10(5.29to18.9)

Solomon_Islands,Deaths,45~50,19.68(11.84to32.72)

Solomon_Islands,Deaths,50~55,41.13(27.41to61.73)

Solomon_Islands,Deaths,55~60,66.22(46.38to94.53)

Solomon_Islands,Deaths,60~65,128.36(94.49to174.37)

Solomon_Islands,Deaths,65~70,218.66(164.35to290.92)

Solomon_Islands,Deaths,70~75,353.32(266.56to468.32)

Solomon_Islands,Deaths,75~80,585.28(425.19to805.64)

Solomon_Islands,Deaths,80~85,931.1(662.87to1307.86)

Solomon_Islands,Deaths,85~90,1469.15(992.84to2173.97)

Solomon_Islands,Deaths,90~95,2059.01(1163.44to3643.96)

Solomon_Islands,DALYs,20~25,140.16(121to162.36)

Solomon_Islands,DALYs,25~30,209.1(184.55to236.92)

Solomon_Islands,DALYs,30~35,308.89(276.84to344.63)

Solomon_Islands,DALYs,35~40,351.8(317.14to390.25)

Solomon_Islands,DALYs,40~45,591.18(541.71to645.17)

Solomon_Islands,DALYs,45~50,1024.53(951.02to1103.72)

Solomon_Islands,DALYs,50~55,1853.95(1739.47to1975.95)

Solomon_Islands,DALYs,55~60,2603.28(2453.75to2761.93)

Solomon_Islands,DALYs,60~65,4199.88(3978.34to4433.76)

Solomon_Islands,DALYs,65~70,5988.21(5679.31to6313.92)

Solomon_Islands,DALYs,70~75,7980.08(7555.74to8428.24)

Solomon_Islands,DALYs,75~80,10551.62(9896to11250.67)

Solomon_Islands,DALYs,80~85,13113.7(12180.9to14117.93)

Solomon_Islands,DALYs,85~90,16310.23(14795.77to17979.71)

Solomon_Islands,DALYs,90~95,19589.29(16535.26to23207.39)

Vanuatu,Prevalence,20~25,577.65(514.11to649.04)

Vanuatu,Prevalence,25~30,803.85(725.16to891.08)

Vanuatu,Prevalence,30~35,1020.66(928.26to1122.25)

Vanuatu,Prevalence,35~40,1221.6(1117.32to1335.62)

Vanuatu,Prevalence,40~45,1525.33(1405.45to1655.43)

Vanuatu,Prevalence,45~50,2315.44(2151.82to2491.5)

Vanuatu,Prevalence,50~55,3582.82(3353.17to3828.2)

Vanuatu,Prevalence,55~60,4919.78(4620.73to5238.19)

Vanuatu,Prevalence,60~65,6359.28(5984.33to6757.72)

Vanuatu,Prevalence,65~70,8582.44(8074.89to9121.9)

Vanuatu,Prevalence,70~75,11684.53(10960.82to12456.04)

Vanuatu,Prevalence,75~80,15282.09(14156.22to16497.51)

Vanuatu,Prevalence,80~85,19008.34(17356.03to20817.96)

Vanuatu,Prevalence,85~90,22847.47(20180.38to25867.04)

Vanuatu,Prevalence,90~95,25504.86(20455.69to31800.35)

Vanuatu,Deaths,20~25,4.34(1.15to16.33)

Vanuatu,Deaths,25~30,5.48(1.74to17.24)

Vanuatu,Deaths,30~35,9.37(3.67to23.91)

Vanuatu,Deaths,35~40,11.6(4.97to27.1)

Vanuatu,Deaths,40~45,19.91(9.96to39.79)

Vanuatu,Deaths,45~50,30.79(16.93to56)

Vanuatu,Deaths,50~55,56.82(34.56to93.43)

Vanuatu,Deaths,55~60,82.51(52.49to129.69)

Vanuatu,Deaths,60~65,162.07(110.48to237.77)

Vanuatu,Deaths,65~70,271.06(189.37to387.98)

Vanuatu,Deaths,70~75,441.94(309.88to630.28)

Vanuatu,Deaths,75~80,705.58(469.74to1059.81)

Vanuatu,Deaths,80~85,1094.37(707.03to1693.9)

Vanuatu,Deaths,85~90,1652.54(994.69to2745.47)

Vanuatu,Deaths,90~95,2479.56(1225.63to5016.39)

Vanuatu,DALYs,20~25,340.85(290.26to400.26)

Vanuatu,DALYs,25~30,410.54(355.79to473.73)

Vanuatu,DALYs,30~35,621.28(548.71to703.45)

Vanuatu,DALYs,35~40,707.94(630.11to795.39)

Vanuatu,DALYs,40~45,1066.85(965.29to1179.1)

Vanuatu,DALYs,45~50,1498.42(1368.91to1640.19)

Vanuatu,DALYs,50~55,2437.62(2250.84to2639.89)

Vanuatu,DALYs,55~60,3124.57(2895.67to3371.57)

Vanuatu,DALYs,60~65,5140.58(4799.7to5505.67)

Vanuatu,DALYs,65~70,7218.35(6747.87to7721.64)

Vanuatu,DALYs,70~75,9676.54(9021.96to10378.61)

Vanuatu,DALYs,75~80,12379.07(11391.66to13452.06)

Vanuatu,DALYs,80~85,15060.25(13666.73to16595.86)

Vanuatu,DALYs,85~90,18038.77(15853.87to20524.77)

Vanuatu,DALYs,90~95,23185.63(18790.92to28608.16)

Kenya,Prevalence,20~25,402.25(391.85to412.91)

Kenya,Prevalence,25~30,535.76(523.17to548.66)

Kenya,Prevalence,30~35,689.23(674.08to704.72)

Kenya,Prevalence,35~40,857.34(839.43to875.62)

Kenya,Prevalence,40~45,1087.75(1066.68to1109.25)

Kenya,Prevalence,45~50,1529.18(1501.68to1557.18)

Kenya,Prevalence,50~55,2234.47(2197.25to2272.32)

Kenya,Prevalence,55~60,3185.09(3135.12to3235.86)

Kenya,Prevalence,60~65,4335.66(4270.32to4402)

Kenya,Prevalence,65~70,5718.44(5633.11to5805.06)

Kenya,Prevalence,70~75,7454.3(7337.04to7573.42)

Kenya,Prevalence,75~80,9522.85(9338.14to9711.21)

Kenya,Prevalence,80~85,11722.12(11455.5to11994.95)

Kenya,Prevalence,85~90,13430.9(13030.45to13843.66)

Kenya,Prevalence,90~95,14328.28(13652.92to15037.04)

Kenya,Deaths,20~25,0.41(0.31to0.54)

Kenya,Deaths,25~30,0.5(0.39to0.64)

Kenya,Deaths,30~35,0.96(0.79to1.17)

Kenya,Deaths,35~40,1.87(1.6to2.19)

Kenya,Deaths,40~45,3.21(2.81to3.66)

Kenya,Deaths,45~50,8.23(7.47to9.07)

Kenya,Deaths,50~55,15.23(14.07to16.48)

Kenya,Deaths,55~60,35.02(32.94to37.23)

Kenya,Deaths,60~65,72.71(69.12to76.49)

Kenya,Deaths,65~70,131.32(125.4to137.52)

Kenya,Deaths,70~75,252.12(241.09to263.66)

Kenya,Deaths,75~80,383.52(363.39to404.77)

Kenya,Deaths,80~85,662.91(626.17to701.81)

Kenya,Deaths,85~90,868.1(812.7to927.28)

Kenya,Deaths,90~95,1152.01(1056.31to1256.39)

Kenya,DALYs,20~25,66.19(62.83to69.74)

Kenya,DALYs,25~30,83.51(79.67to87.52)

Kenya,DALYs,30~35,121.97(116.95to127.21)

Kenya,DALYs,35~40,180.61(173.91to187.56)

Kenya,DALYs,40~45,256.76(248.18to265.63)

Kenya,DALYs,45~50,496.88(483.02to511.13)

Kenya,DALYs,50~55,790.13(770.77to809.98)

Kenya,DALYs,55~60,1469.63(1438.87to1501.05)

Kenya,DALYs,60~65,2503.45(2456.59to2551.2)

Kenya,DALYs,65~70,3727.67(3660.41to3796.17)

Kenya,DALYs,70~75,5735.13(5629.76to5842.46)

Kenya,DALYs,75~80,7002.1(6842.03to7165.91)

Kenya,DALYs,80~85,9340.44(9098.19to9589.14)

Kenya,DALYs,85~90,9791.98(9459.03to10136.65)

Kenya,DALYs,90~95,11119.1(10545.3to11724.13)

Malawi,Prevalence,20~25,345.11(338.65to351.69)

Malawi,Prevalence,25~30,469.93(461.96to478.04)

Malawi,Prevalence,30~35,618.03(608.18to628.05)

Malawi,Prevalence,35~40,784.65(772.72to796.76)

Malawi,Prevalence,40~45,1021.08(1006.73to1035.65)

Malawi,Prevalence,45~50,1491.4(1471.84to1511.22)

Malawi,Prevalence,50~55,2242.37(2214.99to2270.08)

Malawi,Prevalence,55~60,3229.02(3191.86to3266.62)

Malawi,Prevalence,60~65,4365.28(4317.06to4414.04)

Malawi,Prevalence,65~70,5659.34(5597.28to5722.08)

Malawi,Prevalence,70~75,7240.2(7156.14to7325.24)

Malawi,Prevalence,75~80,9122.05(8989.89to9256.14)

Malawi,Prevalence,80~85,11178.48(10982.66to11377.8)

Malawi,Prevalence,85~90,12880.44(12570.18to13198.36)

Malawi,Prevalence,90~95,14042.95(13474.16to14635.76)

Malawi,Deaths,20~25,0.53(0.35to0.82)

Malawi,Deaths,25~30,0.64(0.43to0.93)

Malawi,Deaths,30~35,1.17(0.85to1.6)

Malawi,Deaths,35~40,2.01(1.54to2.61)

Malawi,Deaths,40~45,3.64(2.94to4.52)

Malawi,Deaths,45~50,9.47(8.1to11.08)

Malawi,Deaths,50~55,16.66(14.63to18.97)

Malawi,Deaths,55~60,37.57(33.95to41.58)

Malawi,Deaths,60~65,72.98(67.02to79.48)

Malawi,Deaths,65~70,127.72(118.18to138.04)

Malawi,Deaths,70~75,216.65(200.86to233.68)

Malawi,Deaths,75~80,290.14(265.06to317.6)

Malawi,Deaths,80~85,432.86(392.48to477.41)

Malawi,Deaths,85~90,496.09(439.35to560.14)

Malawi,Deaths,90~95,596.49(498.75to713.39)

Malawi,DALYs,20~25,71.77(68.52to75.18)

Malawi,DALYs,25~30,89.27(85.61to93.09)

Malawi,DALYs,30~35,130.87(126.03to135.9)

Malawi,DALYs,35~40,184.62(178.36to191.1)

Malawi,DALYs,40~45,274.9(266.6to283.47)

Malawi,DALYs,45~50,547.18(533.51to561.19)

Malawi,DALYs,50~55,846.13(827.28to865.41)

Malawi,DALYs,55~60,1556.3(1526.5to1586.69)

Malawi,DALYs,60~65,2513.36(2469.81to2557.68)

Malawi,DALYs,65~70,3645.59(3584.99to3707.21)

Malawi,DALYs,70~75,5040.42(4954.82to5127.49)

Malawi,DALYs,75~80,5494.97(5378.26to5614.21)

Malawi,DALYs,80~85,6431.7(6269.75to6597.82)

Malawi,DALYs,85~90,6006.26(5790.3to6230.27)

Malawi,DALYs,90~95,6255.81(5878.49to6657.34)

Barbados,Prevalence,20~25,193.77(166.17to225.96)

Barbados,Prevalence,25~30,257.68(226.39to293.3)

Barbados,Prevalence,30~35,343.68(307.77to383.77)

Barbados,Prevalence,35~40,462.13(420.2to508.25)

Barbados,Prevalence,40~45,647.89(596.85to703.28)

Barbados,Prevalence,45~50,978.6(912.12to1049.93)

Barbados,Prevalence,50~55,1511.88(1421.78to1607.68)

Barbados,Prevalence,55~60,2306.38(2183.79to2435.84)

Barbados,Prevalence,60~65,3398.86(3233.82to3572.32)

Barbados,Prevalence,65~70,4837.85(4614.6to5071.9)

Barbados,Prevalence,70~75,6885.2(6571.74to7213.63)

Barbados,Prevalence,75~80,9799.28(9281.16to10346.32)

Barbados,Prevalence,80~85,13872.72(13103.57to14687.01)

Barbados,Prevalence,85~90,19264.49(18097.67to20506.54)

Barbados,Prevalence,90~95,25998.37(24064.42to28087.74)

Barbados,Deaths,20~25,0.07(0to195.86)

Barbados,Deaths,25~30,0.09(0to60.7)

Barbados,Deaths,30~35,0.12(0to25.86)

Barbados,Deaths,35~40,0.21(0to11.93)

Barbados,Deaths,40~45,0.4(0.02to7.99)

Barbados,Deaths,45~50,0.96(0.13to7.38)

Barbados,Deaths,50~55,2.24(0.53to9.54)

Barbados,Deaths,55~60,5.3(1.82to15.41)

Barbados,Deaths,60~65,12.94(5.77to29.02)

Barbados,Deaths,65~70,23.23(11.54to46.77)

Barbados,Deaths,70~75,44.01(22.94to84.43)

Barbados,Deaths,75~80,73.74(34.94to155.63)

Barbados,Deaths,80~85,123.63(58.16to262.78)

Barbados,Deaths,85~90,227.75(105.52to491.59)

Barbados,Deaths,90~95,371.86(162.12to852.97)

Barbados,DALYs,20~25,12.74(7.04to23.06)

Barbados,DALYs,25~30,16.48(10.01to27.14)

Barbados,DALYs,30~35,21.22(13.89to32.42)

Barbados,DALYs,35~40,29.93(21.01to42.64)

Barbados,DALYs,40~45,45.26(33.71to60.76)

Barbados,DALYs,45~50,80.28(63.54to101.44)

Barbados,DALYs,50~55,144.91(119.87to175.18)

Barbados,DALYs,55~60,265.8(227.12to311.07)

Barbados,DALYs,60~65,501.55(439.44to572.43)

Barbados,DALYs,65~70,743.73(658.02to840.61)

Barbados,DALYs,70~75,1129.07(1002.19to1272.02)

Barbados,DALYs,75~80,1525.99(1327.81to1753.74)

Barbados,DALYs,80~85,2025.62(1751.56to2342.55)

Barbados,DALYs,85~90,2925.57(2502.66to3419.96)

Barbados,DALYs,90~95,4114.21(3414.41to4957.43)

Cuba,Prevalence,20~25,307.23(298.37to316.36)

Cuba,Prevalence,25~30,414.93(405.09to425.01)

Cuba,Prevalence,30~35,551.6(540.32to563.11)

Cuba,Prevalence,35~40,715.87(702.91to729.07)

Cuba,Prevalence,40~45,984.65(969.32to1000.23)

Cuba,Prevalence,45~50,1639.97(1618.75to1661.46)

Cuba,Prevalence,50~55,2804.69(2774.02to2835.71)

Cuba,Prevalence,55~60,4380.65(4337.89to4423.83)

Cuba,Prevalence,60~65,6285.54(6227.8to6343.81)

Cuba,Prevalence,65~70,8800.63(8722.58to8879.37)

Cuba,Prevalence,70~75,12186.44(12078.25to12295.61)

Cuba,Prevalence,75~80,16638.68(16467.57to16811.56)

Cuba,Prevalence,80~85,22243.72(21999.17to22491)

Cuba,Prevalence,85~90,29002.02(28646.7to29361.74)

Cuba,Prevalence,90~95,36707.9(36162.88to37261.14)

Cuba,Deaths,20~25,0.36(0.2to0.63)

Cuba,Deaths,25~30,0.45(0.28to0.71)

Cuba,Deaths,30~35,0.59(0.41to0.87)

Cuba,Deaths,35~40,1.09(0.82to1.44)

Cuba,Deaths,40~45,2.1(1.71to2.57)

Cuba,Deaths,45~50,4.61(3.99to5.32)

Cuba,Deaths,50~55,10.56(9.51to11.72)

Cuba,Deaths,55~60,23.38(21.57to25.35)

Cuba,Deaths,60~65,51(47.76to54.45)

Cuba,Deaths,65~70,95.11(89.79to100.74)

Cuba,Deaths,70~75,164.46(155.51to173.94)

Cuba,Deaths,75~80,276.56(259.19to295.1)

Cuba,Deaths,80~85,425.34(397.51to455.13)

Cuba,Deaths,85~90,620.42(577.31to666.76)

Cuba,Deaths,90~95,859.48(792.78to931.79)

Cuba,DALYs,20~25,38.74(31.89to47.07)

Cuba,DALYs,25~30,47.98(40.89to56.3)

Cuba,DALYs,30~35,60.06(52.38to68.85)

Cuba,DALYs,35~40,89.82(80.32to100.44)

Cuba,DALYs,40~45,143.65(131.42to157.03)

Cuba,DALYs,45~50,268.15(250.42to287.14)

Cuba,DALYs,50~55,522.36(494.7to551.56)

Cuba,DALYs,55~60,967.55(924.45to1012.66)

Cuba,DALYs,60~65,1730.36(1662.51to1800.99)

Cuba,DALYs,65~70,2658.17(2560.95to2759.08)

Cuba,DALYs,70~75,3745.01(3606.1to3889.26)

Cuba,DALYs,75~80,5033.62(4814.01to5263.25)

Cuba,DALYs,80~85,6137.47(5845.35to6444.19)

Cuba,DALYs,85~90,7220.26(6827.76to7635.33)

Cuba,DALYs,90~95,8759.01(8171.22to9389.09)

San_Marino,Prevalence,20~25,454.02(317.19to649.86)

San_Marino,Prevalence,25~30,616.9(462.56to822.73)

San_Marino,Prevalence,30~35,794.8(622.56to1014.68)

San_Marino,Prevalence,35~40,979.45(790.59to1213.42)

San_Marino,Prevalence,40~45,1259.56(1048.77to1512.72)

San_Marino,Prevalence,45~50,1896.47(1626.71to2210.97)

San_Marino,Prevalence,50~55,2915.6(2558.89to3322.03)

San_Marino,Prevalence,55~60,4215.28(3754.19to4733.01)

San_Marino,Prevalence,60~65,5790.07(5203.04to6443.34)

San_Marino,Prevalence,65~70,7844.38(7087.58to8682)

San_Marino,Prevalence,70~75,10626.62(9623.54to11734.25)

San_Marino,Prevalence,75~80,14482.6(12957.97to16186.62)

San_Marino,Prevalence,80~85,20135.4(17976.3to22553.83)

San_Marino,Prevalence,85~90,27874.92(24773.44to31364.68)

San_Marino,Prevalence,90~95,36195.88(31803.16to41195.33)

San_Marino,Deaths,20~25,0.07(0to9422539048658.04)

San_Marino,Deaths,25~30,0.09(0to16228454361.81)

San_Marino,Deaths,30~35,0.11(0to88754928.11)

San_Marino,Deaths,35~40,0.19(0to619306.23)

San_Marino,Deaths,40~45,0.36(0to10987.7)

San_Marino,Deaths,45~50,0.85(0to705.9)

San_Marino,Deaths,50~55,2.19(0.03to172.45)

San_Marino,Deaths,55~60,4.71(0.2to109.24)

San_Marino,Deaths,60~65,11.68(1.18to116.04)

San_Marino,Deaths,65~70,21.1(2.93to152.16)

San_Marino,Deaths,70~75,39.31(5.91to261.33)

San_Marino,Deaths,75~80,69.02(8.97to531.15)

San_Marino,Deaths,80~85,113.66(14.85to869.73)

San_Marino,Deaths,85~90,184.18(23.82to1424.21)

San_Marino,Deaths,90~95,267.9(32.91to2180.73)

San_Marino,DALYs,20~25,23.82(4.85to116.88)

San_Marino,DALYs,25~30,31.95(8.95to114.03)

San_Marino,DALYs,30~35,40.77(14.05to118.35)

San_Marino,DALYs,35~40,51.41(20.64to128.06)

San_Marino,DALYs,40~45,70.01(32.87to149.13)

San_Marino,DALYs,45~50,115.69(63.52to210.69)

San_Marino,DALYs,50~55,205.09(127.57to329.7)

San_Marino,DALYs,55~60,333.67(224.35to496.25)

San_Marino,DALYs,60~65,580.01(414.23to812.14)

San_Marino,DALYs,65~70,843.74(618.56to1150.9)

San_Marino,DALYs,70~75,1236.36(911.34to1677.29)

San_Marino,DALYs,75~80,1714.18(1225.51to2397.7)

San_Marino,DALYs,80~85,2256.73(1604.01to3175.06)

San_Marino,DALYs,85~90,2960.88(2080.7to4213.4)

San_Marino,DALYs,90~95,3782.3(2585.03to5534.08)

Democratic_Republic_of_the_Congo,Prevalence,20~25,385.62(381.04to390.25)

Democratic_Republic_of_the_Congo,Prevalence,25~30,516.29(510.79to521.85)

Democratic_Republic_of_the_Congo,Prevalence,30~35,670.46(663.88to677.11)

Democratic_Republic_of_the_Congo,Prevalence,35~40,841.35(833.62to849.16)

Democratic_Republic_of_the_Congo,Prevalence,40~45,1106.76(1097.45to1116.15)

Democratic_Republic_of_the_Congo,Prevalence,45~50,1739.4(1726.22to1752.68)

Democratic_Republic_of_the_Congo,Prevalence,50~55,2839.7(2820.34to2859.19)

Democratic_Republic_of_the_Congo,Prevalence,55~60,4276.75(4249.64to4304.03)

Democratic_Republic_of_the_Congo,Prevalence,60~65,5910.96(5874.51to5947.63)

Democratic_Republic_of_the_Congo,Prevalence,65~70,7902.09(7852.77to7951.71)

Democratic_Republic_of_the_Congo,Prevalence,70~75,10441.33(10371.85to10511.27)

Democratic_Republic_of_the_Congo,Prevalence,75~80,13672.2(13562.61to13782.67)

Democratic_Republic_of_the_Congo,Prevalence,80~85,17746.37(17580.21to17914.1)

Democratic_Republic_of_the_Congo,Prevalence,85~90,22013.66(21739.7to22291.07)

Democratic_Republic_of_the_Congo,Prevalence,90~95,25431.12(24901.05to25972.49)

Democratic_Republic_of_the_Congo,Deaths,20~25,0.48(0.39to0.58)

Democratic_Republic_of_the_Congo,Deaths,25~30,0.59(0.49to0.7)

Democratic_Republic_of_the_Congo,Deaths,30~35,1.18(1.02to1.35)

Democratic_Republic_of_the_Congo,Deaths,35~40,1.78(1.58to2)

Democratic_Republic_of_the_Congo,Deaths,40~45,4.25(3.9to4.63)

Democratic_Republic_of_the_Congo,Deaths,45~50,12.25(11.53to13.01)

Democratic_Republic_of_the_Congo,Deaths,50~55,23.21(22.1to24.38)

Democratic_Republic_of_the_Congo,Deaths,55~60,50.26(48.32to52.28)

Democratic_Republic_of_the_Congo,Deaths,60~65,103.76(100.34to107.3)

Democratic_Republic_of_the_Congo,Deaths,65~70,196.62(190.65to202.77)

Democratic_Republic_of_the_Congo,Deaths,70~75,336.46(326.33to346.89)

Democratic_Republic_of_the_Congo,Deaths,75~80,460.25(443.98to477.11)

Democratic_Republic_of_the_Congo,Deaths,80~85,675.6(649.75to702.47)

Democratic_Republic_of_the_Congo,Deaths,85~90,896.39(855.42to939.31)

Democratic_Republic_of_the_Congo,Deaths,90~95,1300.25(1217.92to1388.14)

Democratic_Republic_of_the_Congo,DALYs,20~25,70.99(69.04to73.01)

Democratic_Republic_of_the_Congo,DALYs,25~30,88.74(86.55to90.98)

Democratic_Republic_of_the_Congo,DALYs,30~35,134.28(131.38to137.24)

Democratic_Republic_of_the_Congo,DALYs,35~40,176.22(172.76to179.75)

Democratic_Republic_of_the_Congo,DALYs,40~45,309.46(304.38to314.63)

Democratic_Republic_of_the_Congo,DALYs,45~50,689.96(680.98to699.07)

Democratic_Republic_of_the_Congo,DALYs,50~55,1153.43(1140.37to1166.64)

Democratic_Republic_of_the_Congo,DALYs,55~60,2085.05(2064.49to2105.81)

Democratic_Republic_of_the_Congo,DALYs,60~65,3553.95(3522.01to3586.18)

Democratic_Republic_of_the_Congo,DALYs,65~70,5537.92(5489.74to5586.53)

Democratic_Republic_of_the_Congo,DALYs,70~75,7732.13(7662.18to7802.72)

Democratic_Republic_of_the_Congo,DALYs,75~80,8643.48(8547.5to8740.55)

Democratic_Republic_of_the_Congo,DALYs,80~85,10070.13(9938.21to10203.81)

Democratic_Republic_of_the_Congo,DALYs,85~90,10812.11(10617.95to11009.83)

Democratic_Republic_of_the_Congo,DALYs,90~95,13351.09(12964.29to13749.44)

United_States_of_America,Prevalence,20~25,414.49(376to456.92)

United_States_of_America,Prevalence,25~30,523.26(483.03to566.85)

United_States_of_America,Prevalence,30~35,670.41(627.25to716.55)

United_States_of_America,Prevalence,35~40,841.65(795.13to890.89)

United_States_of_America,Prevalence,40~45,1291.8(1234.51to1351.74)

United_States_of_America,Prevalence,45~50,2794.58(2702.71to2889.57)

United_States_of_America,Prevalence,50~55,4785.58(4656.24to4918.51)

United_States_of_America,Prevalence,55~60,6815.6(6651.3to6983.95)

United_States_of_America,Prevalence,60~65,9742.42(9527.51to9962.17)

United_States_of_America,Prevalence,65~70,16024.81(15702.09to16354.16)

United_States_of_America,Prevalence,70~75,23330.76(22868.44to23802.43)

United_States_of_America,Prevalence,75~80,31268.68(30542.44to32012.2)

United_States_of_America,Prevalence,80~85,38002.39(37058.94to38969.86)

United_States_of_America,Prevalence,85~90,42742.96(41549.26to43970.96)

United_States_of_America,Prevalence,90~95,45250.36(43639.09to46921.12)

United_States_of_America,Deaths,20~25,0.09(0.06to0.13)

United_States_of_America,Deaths,25~30,0.13(0.09to0.18)

United_States_of_America,Deaths,30~35,0.24(0.19to0.31)

United_States_of_America,Deaths,35~40,0.55(0.47to0.65)

United_States_of_America,Deaths,40~45,1.56(1.42to1.72)

United_States_of_America,Deaths,45~50,4.5(4.24to4.78)

United_States_of_America,Deaths,50~55,12.45(11.97to12.96)

United_States_of_America,Deaths,55~60,28.24(27.43to29.08)

United_States_of_America,Deaths,60~65,60.01(58.63to61.41)

United_States_of_America,Deaths,65~70,109.81(107.6to112.07)

United_States_of_America,Deaths,70~75,195.42(191.7to199.21)

United_States_of_America,Deaths,75~80,313.45(306.68to320.38)

United_States_of_America,Deaths,80~85,453.84(443.82to464.09)

United_States_of_America,Deaths,85~90,661.71(646.54to677.24)

United_States_of_America,Deaths,90~95,942.09(918.38to966.41)

United_States_of_America,DALYs,20~25,33.74(30.59to37.23)

United_States_of_America,DALYs,25~30,43.02(39.7to46.61)

United_States_of_America,DALYs,30~35,59.11(55.41to63.07)

United_States_of_America,DALYs,35~40,86.53(82.15to91.14)

United_States_of_America,DALYs,40~45,163.56(157.46to169.91)

United_States_of_America,DALYs,45~50,386.2(376.06to396.62)

United_States_of_America,DALYs,50~55,807.66(791.56to824.08)

United_States_of_America,DALYs,55~60,1419.88(1396.43to1443.73)

United_States_of_America,DALYs,60~65,2405.28(2370.8to2440.27)

United_States_of_America,DALYs,65~70,3752.8(3702.98to3803.29)

United_States_of_America,DALYs,70~75,5455.84(5384.7to5527.92)

United_States_of_America,DALYs,75~80,7018.93(6912.47to7127.03)

United_States_of_America,DALYs,80~85,8023.46(7893.41to8155.66)

United_States_of_America,DALYs,85~90,9142.89(8977.81to9310.99)

United_States_of_America,DALYs,90~95,10872.58(10631.8to11118.82)

Tuvalu,Prevalence,20~25,587.78(348.26to992.04)

Tuvalu,Prevalence,25~30,815.38(518.28to1282.81)

Tuvalu,Prevalence,30~35,1032.61(686.44to1553.35)

Tuvalu,Prevalence,35~40,1231.25(845.28to1793.46)

Tuvalu,Prevalence,40~45,1504.29(1076.81to2101.48)

Tuvalu,Prevalence,45~50,2151.25(1601.18to2890.31)

Tuvalu,Prevalence,50~55,3191.42(2454.74to4149.2)

Tuvalu,Prevalence,55~60,4372.58(3422.46to5586.46)

Tuvalu,Prevalence,60~65,5696.05(4494.51to7218.8)

Tuvalu,Prevalence,65~70,7528.6(5955.12to9517.84)

Tuvalu,Prevalence,70~75,10031.61(7879.98to12770.75)

Tuvalu,Prevalence,75~80,12999.89(9817.01to17214.73)

Tuvalu,Prevalence,80~85,16395.84(11909.5to22572.18)

Tuvalu,Prevalence,85~90,20123.56(13379.95to30266.01)

Tuvalu,Prevalence,90~95,23210.79(11837.37to45511.86)

Tuvalu,Deaths,20~25,4.48(0to5183.94)

Tuvalu,Deaths,25~30,5.1(0.01to2418.81)

Tuvalu,Deaths,30~35,7.95(0.06to1089.19)

Tuvalu,Deaths,35~40,9.59(0.13to689.99)

Tuvalu,Deaths,40~45,15.69(0.58to425.56)

Tuvalu,Deaths,45~50,23.28(1.48to366.48)

Tuvalu,Deaths,50~55,42.08(4.68to378.69)

Tuvalu,Deaths,55~60,58.37(8.08to421.42)

Tuvalu,Deaths,60~65,110.03(20.4to593.56)

Tuvalu,Deaths,65~70,176.55(36.74to848.41)

Tuvalu,Deaths,70~75,273.92(58.47to1283.33)

Tuvalu,Deaths,75~80,419.39(76.52to2298.53)

Tuvalu,Deaths,80~85,618.91(103.74to3692.37)

Tuvalu,Deaths,85~90,910.72(124.55to6659.35)

Tuvalu,Deaths,90~95,1326.74(98.23to17919.02)

Tuvalu,DALYs,20~25,346.5(152.97to784.86)

Tuvalu,DALYs,25~30,388.49(187.3to805.81)

Tuvalu,DALYs,30~35,540.06(290.33to1004.59)

Tuvalu,DALYs,35~40,603.61(343.5to1060.7)

Tuvalu,DALYs,40~45,862.75(543.75to1368.9)

Tuvalu,DALYs,45~50,1163.35(774.38to1747.68)

Tuvalu,DALYs,50~55,1847.17(1304.72to2615.14)

Tuvalu,DALYs,55~60,2282.8(1642.72to3172.29)

Tuvalu,DALYs,60~65,3606.54(2676.55to4859.66)

Tuvalu,DALYs,65~70,4856.4(3633.77to6490.41)

Tuvalu,DALYs,70~75,6212.7(4615.42to8362.76)

Tuvalu,DALYs,75~80,7640.88(5441.92to10728.39)

Tuvalu,DALYs,80~85,8879.27(6052.31to13026.68)

Tuvalu,DALYs,85~90,10384.99(6417.83to16804.46)

Tuvalu,DALYs,90~95,12965.89(6169.51to27249.22)

Tajikistan,Prevalence,20~25,618.95(603.76to634.52)

Tajikistan,Prevalence,25~30,842.95(824.55to861.76)

Tajikistan,Prevalence,30~35,1051.17(1029.97to1072.8)

Tajikistan,Prevalence,35~40,1237.07(1213.33to1261.28)

Tajikistan,Prevalence,40~45,1522.89(1495.61to1550.67)

Tajikistan,Prevalence,45~50,2293.8(2256.18to2332.06)

Tajikistan,Prevalence,50~55,3591.82(3538.8to3645.65)

Tajikistan,Prevalence,55~60,5169.25(5098.07to5241.42)

Tajikistan,Prevalence,60~65,7014.43(6921.27to7108.84)

Tajikistan,Prevalence,65~70,9681.32(9553.03to9811.34)

Tajikistan,Prevalence,70~75,13268.55(13086.12to13453.53)

Tajikistan,Prevalence,75~80,17709.4(17425.98to17997.43)

Tajikistan,Prevalence,80~85,22601.71(22208.61to23001.78)

Tajikistan,Prevalence,85~90,27906.88(27319.59to28506.8)

Tajikistan,Prevalence,90~95,32859.82(31889.08to33860.1)

Tajikistan,Deaths,20~25,3.79(2.65to5.41)

Tajikistan,Deaths,25~30,4.49(3.3to6.1)

Tajikistan,Deaths,30~35,6.78(5.27to8.72)

Tajikistan,Deaths,35~40,7.55(6to9.51)

Tajikistan,Deaths,40~45,9.49(7.75to11.62)

Tajikistan,Deaths,45~50,13.86(11.64to16.52)

Tajikistan,Deaths,50~55,23.71(20.53to27.38)

Tajikistan,Deaths,55~60,38.94(34.44to44.02)

Tajikistan,Deaths,60~65,72.19(64.86to80.36)

Tajikistan,Deaths,65~70,118.65(107.21to131.32)

Tajikistan,Deaths,70~75,177.78(160.83to196.51)

Tajikistan,Deaths,75~80,263.03(235.5to293.78)

Tajikistan,Deaths,80~85,341.75(304.47to383.59)

Tajikistan,Deaths,85~90,441.26(388.46to501.25)

Tajikistan,Deaths,90~95,616.67(529.42to718.3)

Tajikistan,DALYs,20~25,278.83(246.75to315.08)

Tajikistan,DALYs,25~30,321.97(288.7to359.07)

Tajikistan,DALYs,30~35,435.52(395.9to479.1)

Tajikistan,DALYs,35~40,456.41(416.96to499.58)

Tajikistan,DALYs,40~45,526.51(484.89to571.69)

Tajikistan,DALYs,45~50,707.28(656.25to762.29)

Tajikistan,DALYs,50~55,1075.53(1006.99to1148.74)

Tajikistan,DALYs,55~60,1535.18(1446.36to1629.46)

Tajikistan,DALYs,60~65,2388.56(2260.71to2523.65)

Tajikistan,DALYs,65~70,3311.98(3136.94to3496.8)

Tajikistan,DALYs,70~75,4143.37(3917.76to4381.98)

Tajikistan,DALYs,75~80,4960.88(4653.17to5288.94)

Tajikistan,DALYs,80~85,5169.08(4813.19to5551.29)

Tajikistan,DALYs,85~90,5404.75(4949.51to5901.87)

Tajikistan,DALYs,90~95,6534.46(5794.86to7368.47)

Gabon,Prevalence,20~25,288.55(270.82to307.44)

Gabon,Prevalence,25~30,383.64(362.7to405.78)

Gabon,Prevalence,30~35,504.91(479.74to531.41)

Gabon,Prevalence,35~40,655.06(624.75to686.85)

Gabon,Prevalence,40~45,884.14(847.14to922.76)

Gabon,Prevalence,45~50,1341.78(1291.46to1394.06)

Gabon,Prevalence,50~55,2087.57(2017.05to2160.56)

Gabon,Prevalence,55~60,3100.57(3003.48to3200.79)

Gabon,Prevalence,60~65,4313.37(4183.31to4447.47)

Gabon,Prevalence,65~70,5739.02(5566.72to5916.65)

Gabon,Prevalence,70~75,7540.16(7300.1to7788.12)

Gabon,Prevalence,75~80,9910.88(9544.15to10291.7)

Gabon,Prevalence,80~85,13079.43(12535.75to13646.69)

Gabon,Prevalence,85~90,16720.83(15847.96to17641.78)

Gabon,Prevalence,90~95,20119.25(18473.75to21911.31)

Gabon,Deaths,20~25,0.62(0.13to2.97)

Gabon,Deaths,25~30,0.66(0.16to2.69)

Gabon,Deaths,30~35,1.41(0.52to3.84)

Gabon,Deaths,35~40,1.9(0.8to4.5)

Gabon,Deaths,40~45,4.39(2.39to8.04)

Gabon,Deaths,45~50,11.59(7.65to17.55)

Gabon,Deaths,50~55,17.2(12.07to24.52)

Gabon,Deaths,55~60,33.19(24.86to44.29)

Gabon,Deaths,60~65,60.04(46.62to77.32)

Gabon,Deaths,65~70,105.92(83.89to133.75)

Gabon,Deaths,70~75,170.12(134.99to214.39)

Gabon,Deaths,75~80,221.62(170.95to287.32)

Gabon,Deaths,80~85,322.2(245.46to422.94)

Gabon,Deaths,85~90,424.03(311.68to576.89)

Gabon,Deaths,90~95,613.11(411.89to912.63)

Gabon,DALYs,20~25,75.81(66.15to86.88)

Gabon,DALYs,25~30,88.91(78.75to100.38)

Gabon,DALYs,30~35,137.24(123.93to151.98)

Gabon,DALYs,35~40,172.01(156.77to188.73)

Gabon,DALYs,40~45,297.35(275.81to320.57)

Gabon,DALYs,45~50,618.62(583.31to656.07)

Gabon,DALYs,50~55,853.43(809.5to899.75)

Gabon,DALYs,55~60,1399.82(1336.24to1466.43)

Gabon,DALYs,60~65,2133.88(2043.62to2228.12)

Gabon,DALYs,65~70,3114.3(2987.09to3246.93)

Gabon,DALYs,70~75,4107.58(3933.85to4289)

Gabon,DALYs,75~80,4415.53(4198.2to4644.1)

Gabon,DALYs,80~85,5118.22(4835.41to5417.56)

Gabon,DALYs,85~90,5483.89(5099.4to5897.38)

Gabon,DALYs,90~95,6792.41(6086.72to7579.92)

Uzbekistan,Prevalence,20~25,626.29(604.83to648.52)

Uzbekistan,Prevalence,25~30,815.69(790.98to841.17)

Uzbekistan,Prevalence,30~35,996.06(968.51to1024.4)

Uzbekistan,Prevalence,35~40,1160.67(1130.54to1191.61)

Uzbekistan,Prevalence,40~45,1392.34(1358.79to1426.71)

Uzbekistan,Prevalence,45~50,1912.83(1870.33to1956.3)

Uzbekistan,Prevalence,50~55,2780.17(2723.9to2837.6)

Uzbekistan,Prevalence,55~60,3947.67(3873.42to4023.34)

Uzbekistan,Prevalence,60~65,5402.52(5304.42to5502.44)

Uzbekistan,Prevalence,65~70,7263.14(7131.56to7397.15)

Uzbekistan,Prevalence,70~75,9710.39(9524.7to9899.69)

Uzbekistan,Prevalence,75~80,12719.41(12445.13to12999.72)

Uzbekistan,Prevalence,80~85,16269.84(15888.41to16660.43)

Uzbekistan,Prevalence,85~90,20082.85(19527.52to20653.96)

Uzbekistan,Prevalence,90~95,23839.12(22950.38to24762.29)

Uzbekistan,Deaths,20~25,2.79(2.11to3.68)

Uzbekistan,Deaths,25~30,2.88(2.27to3.66)

Uzbekistan,Deaths,30~35,3.74(3.07to4.56)

Uzbekistan,Deaths,35~40,4.53(3.82to5.36)

Uzbekistan,Deaths,40~45,5.53(4.78to6.39)

Uzbekistan,Deaths,45~50,6.15(5.39to7.03)

Uzbekistan,Deaths,50~55,8.36(7.46to9.37)

Uzbekistan,Deaths,55~60,12.39(11.19to13.71)

Uzbekistan,Deaths,60~65,23.23(21.25to25.39)

Uzbekistan,Deaths,65~70,31.04(28.47to33.84)

Uzbekistan,Deaths,70~75,45.16(41.48to49.17)

Uzbekistan,Deaths,75~80,56.32(51.37to61.75)

Uzbekistan,Deaths,80~85,62.44(56.71to68.75)

Uzbekistan,Deaths,85~90,65.36(58.75to72.72)

Uzbekistan,Deaths,90~95,67.96(59.57to77.53)

Uzbekistan,DALYs,20~25,190.71(164.66to220.89)

Uzbekistan,DALYs,25~30,205.52(180.27to234.31)

Uzbekistan,DALYs,30~35,246.22(219.33to276.4)

Uzbekistan,DALYs,35~40,276.93(249.19to307.75)

Uzbekistan,DALYs,40~45,316.38(287.84to347.75)

Uzbekistan,DALYs,45~50,351.04(321.23to383.62)

Uzbekistan,DALYs,50~55,449.9(415.01to487.73)

Uzbekistan,DALYs,55~60,594.26(550.73to641.22)

Uzbekistan,DALYs,60~65,920.61(858.2to987.55)

Uzbekistan,DALYs,65~70,1070.44(997.69to1148.5)

Uzbekistan,DALYs,70~75,1299.79(1209.08to1397.3)

Uzbekistan,DALYs,75~80,1358.84(1253to1473.61)

Uzbekistan,DALYs,80~85,1272.56(1162.41to1393.15)

Uzbekistan,DALYs,85~90,1152.06(1030.47to1287.99)

Uzbekistan,DALYs,90~95,1099.86(939.96to1286.96)

Dominican_Republic,Prevalence,20~25,267.99(262.14to273.98)

Dominican_Republic,Prevalence,25~30,358.93(351.99to366.01)

Dominican_Republic,Prevalence,30~35,475.27(466.92to483.77)

Dominican_Republic,Prevalence,35~40,621.33(611.31to631.52)

Dominican_Republic,Prevalence,40~45,841.86(829.65to854.24)

Dominican_Republic,Prevalence,45~50,1275.87(1259.31to1292.65)

Dominican_Republic,Prevalence,50~55,1998.76(1975.61to2022.19)

Dominican_Republic,Prevalence,55~60,3035.21(3003.12to3067.64)

Dominican_Republic,Prevalence,60~65,4413.72(4369.8to4458.09)

Dominican_Republic,Prevalence,65~70,6176.62(6116.77to6237.06)

Dominican_Republic,Prevalence,70~75,8677.6(8592.49to8763.56)

Dominican_Republic,Prevalence,75~80,12136.72(11996.52to12278.55)

Dominican_Republic,Prevalence,80~85,17104.72(16894.63to17317.42)

Dominican_Republic,Prevalence,85~90,23262.33(22944.52to23584.55)

Dominican_Republic,Prevalence,90~95,30177.86(29663.12to30701.53)

Dominican_Republic,Deaths,20~25,0.14(0.07to0.29)

Dominican_Republic,Deaths,25~30,0.21(0.11to0.38)

Dominican_Republic,Deaths,30~35,0.28(0.17to0.49)

Dominican_Republic,Deaths,35~40,0.54(0.35to0.83)

Dominican_Republic,Deaths,40~45,1.09(0.77to1.52)

Dominican_Republic,Deaths,45~50,2.31(1.77to3.01)

Dominican_Republic,Deaths,50~55,5.43(4.45to6.64)

Dominican_Republic,Deaths,55~60,10.66(9.03to12.59)

Dominican_Republic,Deaths,60~65,25.64(22.46to29.27)

Dominican_Republic,Deaths,65~70,41.67(36.85to47.12)

Dominican_Republic,Deaths,70~75,79.15(70.42to88.97)

Dominican_Republic,Deaths,75~80,128.89(112.55to147.61)

Dominican_Republic,Deaths,80~85,219.09(190.74to251.65)

Dominican_Republic,Deaths,85~90,457.58(397.23to527.09)

Dominican_Republic,Deaths,90~95,633.74(542.03to740.97)

Dominican_Republic,DALYs,20~25,20.31(16.98to24.3)

Dominican_Republic,DALYs,25~30,27.34(23.4to31.94)

Dominican_Republic,DALYs,30~35,35.2(30.57to40.52)

Dominican_Republic,DALYs,35~40,53.34(47.15to60.36)

Dominican_Republic,DALYs,40~45,86.34(77.69to95.96)

Dominican_Republic,DALYs,45~50,151.42(138.41to165.65)

Dominican_Republic,DALYs,50~55,289.12(268.32to311.52)

Dominican_Republic,DALYs,55~60,476.59(446.05to509.22)

Dominican_Republic,DALYs,60~65,906.99(856.34to960.64)

Dominican_Republic,DALYs,65~70,1235.02(1167.63to1306.3)

Dominican_Republic,DALYs,70~75,1877.44(1775.19to1985.57)

Dominican_Republic,DALYs,75~80,2457.63(2299.92to2626.15)

Dominican_Republic,DALYs,80~85,3267.2(3044.55to3506.14)

Dominican_Republic,DALYs,85~90,5251.7(4868.94to5664.54)

Dominican_Republic,DALYs,90~95,6421.75(5849.03to7050.54)

Azerbaijan,Prevalence,20~25,575.36(555.24to596.2)

Azerbaijan,Prevalence,25~30,753.52(730.77to776.98)

Azerbaijan,Prevalence,30~35,928.03(902.95to953.8)

Azerbaijan,Prevalence,35~40,1097.58(1070.05to1125.82)

Azerbaijan,Prevalence,40~45,1344.26(1313.21to1376.04)

Azerbaijan,Prevalence,45~50,1916.86(1876.37to1958.23)

Azerbaijan,Prevalence,50~55,2900.17(2845.48to2955.9)

Azerbaijan,Prevalence,55~60,4250.58(4177.19to4325.26)

Azerbaijan,Prevalence,60~65,5965.07(5866.63to6065.16)

Azerbaijan,Prevalence,65~70,8334.51(8197.62to8473.68)

Azerbaijan,Prevalence,70~75,11619.14(11422.4to11819.27)

Azerbaijan,Prevalence,75~80,16081.32(15767.24to16401.65)

Azerbaijan,Prevalence,80~85,21762.14(21310.91to22222.92)

Azerbaijan,Prevalence,85~90,28057.41(27364.63to28767.74)

Azerbaijan,Prevalence,90~95,34031.5(32918.86to35181.75)

Azerbaijan,Deaths,20~25,3.11(2.11to4.6)

Azerbaijan,Deaths,25~30,2.91(2.07to4.11)

Azerbaijan,Deaths,30~35,3.24(2.42to4.34)

Azerbaijan,Deaths,35~40,3.65(2.82to4.73)

Azerbaijan,Deaths,40~45,5.01(4.04to6.22)

Azerbaijan,Deaths,45~50,7.37(6.14to8.85)

Azerbaijan,Deaths,50~55,12.46(10.76to14.43)

Azerbaijan,Deaths,55~60,20.44(18.09to23.1)

Azerbaijan,Deaths,60~65,35.88(32.22to39.96)

Azerbaijan,Deaths,65~70,59.46(53.75to65.77)

Azerbaijan,Deaths,70~75,91.8(83.12to101.38)

Azerbaijan,Deaths,75~80,129.53(116.04to144.6)

Azerbaijan,Deaths,80~85,166.26(148.41to186.26)

Azerbaijan,Deaths,85~90,213.69(188.49to242.24)

Azerbaijan,Deaths,90~95,229.82(196.61to268.65)

Azerbaijan,DALYs,20~25,214.58(199.88to230.36)

Azerbaijan,DALYs,25~30,212.09(199.04to226.01)

Azerbaijan,DALYs,30~35,229.7(217.06to243.07)

Azerbaijan,DALYs,35~40,245.56(233.06to258.72)

Azerbaijan,DALYs,40~45,302.34(288.78to316.54)

Azerbaijan,DALYs,45~50,405.63(389.21to422.74)

Azerbaijan,DALYs,50~55,606.86(585.5to629.01)

Azerbaijan,DALYs,55~60,872.31(844.87to900.64)

Azerbaijan,DALYs,60~65,1298.3(1260.42to1337.31)

Azerbaijan,DALYs,65~70,1806.12(1754.62to1859.12)

Azerbaijan,DALYs,70~75,2320.29(2252.5to2390.13)

Azerbaijan,DALYs,75~80,2692.51(2602.42to2785.72)

Azerbaijan,DALYs,80~85,2848.7(2745.05to2956.27)

Azerbaijan,DALYs,85~90,3022.33(2887.4to3163.56)

Azerbaijan,DALYs,90~95,2968.61(2782.96to3166.64)

Mozambique,Prevalence,20~25,395.02(389.06to401.07)

Mozambique,Prevalence,25~30,529.06(521.85to536.36)

Mozambique,Prevalence,30~35,684.55(675.88to693.32)

Mozambique,Prevalence,35~40,854.55(844.36to864.86)

Mozambique,Prevalence,40~45,1094.52(1082.57to1106.6)

Mozambique,Prevalence,45~50,1576.84(1560.96to1592.88)

Mozambique,Prevalence,50~55,2340.73(2318.95to2362.71)

Mozambique,Prevalence,55~60,3339.61(3310.06to3369.42)

Mozambique,Prevalence,60~65,4491.37(4452.4to4530.67)

Mozambique,Prevalence,65~70,5778.72(5727.98to5829.92)

Mozambique,Prevalence,70~75,7347.22(7278.13to7416.95)

Mozambique,Prevalence,75~80,9288.56(9181.32to9397.06)

Mozambique,Prevalence,80~85,11342.46(11185.45to11501.67)

Mozambique,Prevalence,85~90,12743.15(12501.08to12989.9)

Mozambique,Prevalence,90~95,13375.77(12951.9to13813.51)

Mozambique,Deaths,20~25,0.27(0.19to0.4)

Mozambique,Deaths,25~30,0.35(0.25to0.49)

Mozambique,Deaths,30~35,0.68(0.52to0.9)

Mozambique,Deaths,35~40,1.24(0.99to1.56)

Mozambique,Deaths,40~45,2.79(2.34to3.33)

Mozambique,Deaths,45~50,7.61(6.69to8.65)

Mozambique,Deaths,50~55,13.52(12.15to15.05)

Mozambique,Deaths,55~60,30.8(28.29to33.53)

Mozambique,Deaths,60~65,69(64.32to74.02)

Mozambique,Deaths,65~70,127.84(119.78to136.45)

Mozambique,Deaths,70~75,209.42(196.03to223.71)

Mozambique,Deaths,75~80,283.77(262.27to307.03)

Mozambique,Deaths,80~85,506.2(465.87to550.04)

Mozambique,Deaths,85~90,640.14(579.38to707.28)

Mozambique,Deaths,90~95,750.53(650.06to866.53)

Mozambique,DALYs,20~25,52.62(49.78to55.62)

Mozambique,DALYs,25~30,67.45(64.18to70.89)

Mozambique,DALYs,30~35,100.07(95.7to104.63)

Mozambique,DALYs,35~40,142.78(137.15to148.65)

Mozambique,DALYs,40~45,235.36(227.3to243.71)

Mozambique,DALYs,45~50,476.2(462.62to490.16)

Mozambique,DALYs,50~55,734.64(715.98to753.78)

Mozambique,DALYs,55~60,1343.39(1313.62to1373.83)

Mozambique,DALYs,60~65,2409.26(2361.33to2458.16)

Mozambique,DALYs,65~70,3643.01(3572.1to3715.32)

Mozambique,DALYs,70~75,4864.99(4764.5to4967.6)

Mozambique,DALYs,75~80,5379.63(5240.91to5522.02)

Mozambique,DALYs,80~85,7356.22(7139.13to7579.92)

Mozambique,DALYs,85~90,7485.14(7181.17to7801.97)

Mozambique,DALYs,90~95,7625.42(7114.76to8172.73)

Seychelles,Prevalence,20~25,374.04(304.56to459.37)

Seychelles,Prevalence,25~30,520.66(437.68to619.38)

Seychelles,Prevalence,30~35,687.69(590.56to800.81)

Seychelles,Prevalence,35~40,861(749.33to989.33)

Seychelles,Prevalence,40~45,1112.58(981.48to1261.18)

Seychelles,Prevalence,45~50,1660.38(1484.14to1857.56)

Seychelles,Prevalence,50~55,2516.28(2274.08to2784.27)

Seychelles,Prevalence,55~60,3604.25(3282.2to3957.9)

Seychelles,Prevalence,60~65,4938.09(4515.3to5400.47)

Seychelles,Prevalence,65~70,6667.23(6104.78to7281.5)

Seychelles,Prevalence,70~75,9097.69(8319.85to9948.25)

Seychelles,Prevalence,75~80,12452.05(11234.9to13801.07)

Seychelles,Prevalence,80~85,16908.15(15140.86to18881.73)

Seychelles,Prevalence,85~90,22579.79(19918.95to25596.07)

Seychelles,Prevalence,90~95,28587(24262.61to33682.12)

Seychelles,Deaths,20~25,0.83(0.01to126.11)

Seychelles,Deaths,25~30,1.13(0.02to62.83)

Seychelles,Deaths,30~35,1.8(0.08to39.3)

Seychelles,Deaths,35~40,2.92(0.26to32.77)

Seychelles,Deaths,40~45,4.92(0.71to34.22)

Seychelles,Deaths,45~50,7.91(1.62to38.56)

Seychelles,Deaths,50~55,14.62(4.08to52.34)

Seychelles,Deaths,55~60,25.37(8.85to72.71)

Seychelles,Deaths,60~65,49.08(20.21to119.16)

Seychelles,Deaths,65~70,81.18(36.37to181.2)

Seychelles,Deaths,70~75,135.8(61.96to297.64)

Seychelles,Deaths,75~80,225.38(95.94to529.49)

Seychelles,Deaths,80~85,313.15(130.29to752.67)

Seychelles,Deaths,85~90,467.37(186.6to1170.63)

Seychelles,Deaths,90~95,611.75(213.03to1756.76)

Seychelles,DALYs,20~25,88.23(54.85to141.95)

Seychelles,DALYs,25~30,116.61(78.48to173.26)

Seychelles,DALYs,30~35,162.39(116.66to226.05)

Seychelles,DALYs,35~40,223.22(167.85to296.87)

Seychelles,DALYs,40~45,321.46(251.51to410.87)

Seychelles,DALYs,45~50,469.59(379.22to581.5)

Seychelles,DALYs,50~55,751.13(623.61to904.74)

Seychelles,DALYs,55~60,1119.41(948.79to1320.71)

Seychelles,DALYs,60~65,1777.33(1529.75to2064.99)

Seychelles,DALYs,65~70,2458.88(2130.87to2837.38)

Seychelles,DALYs,70~75,3359.32(2905.6to3883.9)

Seychelles,DALYs,75~80,4461.29(3793.9to5246.08)

Seychelles,DALYs,80~85,4993.43(4191.04to5949.45)

Seychelles,DALYs,85~90,5985.29(4909.14to7297.33)

Seychelles,DALYs,90~95,6881.9(5307to8924.17)

Guyana,Prevalence,20~25,215.38(198.07to234.21)

Guyana,Prevalence,25~30,290.02(269.36to312.26)

Guyana,Prevalence,30~35,391.13(366to417.98)

Guyana,Prevalence,35~40,527.22(496.45to559.9)

Guyana,Prevalence,40~45,739.98(701.92to780.11)

Guyana,Prevalence,45~50,1151.18(1097.92to1207.02)

Guyana,Prevalence,50~55,1820.38(1744.04to1900.07)

Guyana,Prevalence,55~60,2747.53(2639.65to2859.82)

Guyana,Prevalence,60~65,3914.36(3766.08to4068.48)

Guyana,Prevalence,65~70,5331.2(5129.67to5540.65)

Guyana,Prevalence,70~75,7185.21(6899.16to7483.12)

Guyana,Prevalence,75~80,9558.08(9110.14to10028.03)

Guyana,Prevalence,80~85,12687.56(12027.91to13383.39)

Guyana,Prevalence,85~90,16692.09(15670.34to17780.46)

Guyana,Prevalence,90~95,21962.25(20156.72to23929.51)

Guyana,Deaths,20~25,0.07(0to0.99)

Guyana,Deaths,25~30,0.13(0.02to1.07)

Guyana,Deaths,30~35,0.2(0.03to1.26)

Guyana,Deaths,35~40,0.47(0.12to1.88)

Guyana,Deaths,40~45,1.06(0.37to3.05)

Guyana,Deaths,45~50,2.53(1.12to5.71)

Guyana,Deaths,50~55,6.58(3.54to12.24)

Guyana,Deaths,55~60,14.53(8.75to24.14)

Guyana,Deaths,60~65,31.85(20.72to48.95)

Guyana,Deaths,65~70,54.51(36.35to81.74)

Guyana,Deaths,70~75,97.79(65.1to146.91)

Guyana,Deaths,75~80,153.92(96.57to245.31)

Guyana,Deaths,80~85,214.84(130.74to353.03)

Guyana,Deaths,85~90,312.14(180.88to538.65)

Guyana,Deaths,90~95,442.83(226.21to866.87)

Guyana,DALYs,20~25,10.27(7.92to13.31)

Guyana,DALYs,25~30,16.4(13.18to20.41)

Guyana,DALYs,30~35,23.22(19.1to28.23)

Guyana,DALYs,35~40,41.63(35.32to49.07)

Guyana,DALYs,40~45,75.92(66.23to87.02)

Guyana,DALYs,45~50,149.68(133.53to167.79)

Guyana,DALYs,50~55,319.13(290.31to350.81)

Guyana,DALYs,55~60,592.47(545.17to643.87)

Guyana,DALYs,60~65,1073.6(995.8to1157.49)

Guyana,DALYs,65~70,1534.5(1424.18to1653.37)

Guyana,DALYs,70~75,2237.46(2068.88to2419.78)

Guyana,DALYs,75~80,2834.6(2583.5to3110.12)

Guyana,DALYs,80~85,3164.1(2845.27to3518.66)

Guyana,DALYs,85~90,3682.56(3236.27to4190.38)

Guyana,DALYs,90~95,4554.99(3801.28to5458.14)

Burundi,Prevalence,20~25,475.15(464.61to485.92)

Burundi,Prevalence,25~30,642.27(629.45to655.36)

Burundi,Prevalence,30~35,820.93(805.81to836.35)

Burundi,Prevalence,35~40,1000.33(982.92to1018.04)

Burundi,Prevalence,40~45,1261.33(1241to1281.99)

Burundi,Prevalence,45~50,1867.81(1839.93to1896.11)

Burundi,Prevalence,50~55,2878.67(2839.15to2918.74)

Burundi,Prevalence,55~60,4148.62(4094.66to4203.29)

Burundi,Prevalence,60~65,5510.31(5439.94to5581.59)

Burundi,Prevalence,65~70,7038.77(6947.44to7131.3)

Burundi,Prevalence,70~75,8942.23(8818.49to9067.7)

Burundi,Prevalence,75~80,11262.59(11074.24to11454.14)

Burundi,Prevalence,80~85,13731.62(13460.38to14008.33)

Burundi,Prevalence,85~90,15449.82(15035.16to15875.92)

Burundi,Prevalence,90~95,15922.89(15186.12to16695.42)

Burundi,Deaths,20~25,1.64(1.06to2.54)

Burundi,Deaths,25~30,1.78(1.19to2.64)

Burundi,Deaths,30~35,2.62(1.88to3.65)

Burundi,Deaths,35~40,3.97(3.01to5.23)

Burundi,Deaths,40~45,5.95(4.72to7.51)

Burundi,Deaths,45~50,13.53(11.38to16.08)

Burundi,Deaths,50~55,22.9(19.83to26.45)

Burundi,Deaths,55~60,48.11(42.82to54.05)

Burundi,Deaths,60~65,88.35(79.83to97.78)

Burundi,Deaths,65~70,146.62(133.52to161)

Burundi,Deaths,70~75,240.91(220.07to263.72)

Burundi,Deaths,75~80,313.62(282.37to348.33)

Burundi,Deaths,80~85,437.86(391.39to489.86)

Burundi,Deaths,85~90,499.84(436.69to572.14)

Burundi,Deaths,90~95,618.68(510.84to749.27)

Burundi,DALYs,20~25,163.83(156.88to171.08)

Burundi,DALYs,25~30,188.27(180.99to195.84)

Burundi,DALYs,30~35,244.41(235.95to253.18)

Burundi,DALYs,35~40,316.45(306.53to326.7)

Burundi,DALYs,40~45,415.91(404.2to427.96)

Burundi,DALYs,45~50,759.87(741.98to778.18)

Burundi,DALYs,50~55,1148.7(1124.44to1173.47)

Burundi,DALYs,55~60,1994.04(1956.89to2031.91)

Burundi,DALYs,60~65,3062.78(3009.81to3116.69)

Burundi,DALYs,65~70,4231.38(4160.29to4303.69)

Burundi,DALYs,70~75,5673.75(5576.82to5772.38)

Burundi,DALYs,75~80,6027.43(5902.92to6154.58)

Burundi,DALYs,80~85,6650.39(6490.62to6814.08)

Burundi,DALYs,85~90,6159.07(5954to6371.19)

Burundi,DALYs,90~95,6534.14(6177.76to6911.07)

Djibouti,Prevalence,20~25,346.13(320.05to374.32)

Djibouti,Prevalence,25~30,457.92(426.97to491.12)

Djibouti,Prevalence,30~35,592.52(555.67to631.82)

Djibouti,Prevalence,35~40,746.93(703.61to792.92)

Djibouti,Prevalence,40~45,966.33(914.75to1020.81)

Djibouti,Prevalence,45~50,1380.82(1312.79to1452.38)

Djibouti,Prevalence,50~55,2037.64(1944.42to2135.32)

Djibouti,Prevalence,55~60,2904.67(2778.62to3036.44)

Djibouti,Prevalence,60~65,3920.52(3753.7to4094.77)

Djibouti,Prevalence,65~70,5088.35(4868.08to5318.59)

Djibouti,Prevalence,70~75,6528.64(6225.91to6846.08)

Djibouti,Prevalence,75~80,8268.07(7767.23to8801.21)

Djibouti,Prevalence,80~85,10246.37(9478.65to11076.28)

Djibouti,Prevalence,85~90,11883.97(10625.15to13291.94)

Djibouti,Prevalence,90~95,12820.03(10507.04to15642.18)

Djibouti,Deaths,20~25,0.54(0.07to4.28)

Djibouti,Deaths,25~30,0.54(0.08to3.61)

Djibouti,Deaths,30~35,0.99(0.23to4.29)

Djibouti,Deaths,35~40,1.53(0.46to5.12)

Djibouti,Deaths,40~45,2.54(0.95to6.82)

Djibouti,Deaths,45~50,6.23(3.05to12.73)

Djibouti,Deaths,50~55,10.78(5.98to19.45)

Djibouti,Deaths,55~60,23.65(14.83to37.71)

Djibouti,Deaths,60~65,46.29(31.11to68.88)

Djibouti,Deaths,65~70,83.36(57.98to119.84)

Djibouti,Deaths,70~75,140.75(98.85to200.42)

Djibouti,Deaths,75~80,183.91(117.72to287.3)

Djibouti,Deaths,80~85,269.47(164.37to441.77)

Djibouti,Deaths,85~90,317.06(169.2to594.12)

Djibouti,Deaths,90~95,418.99(167.78to1046.3)

Djibouti,DALYs,20~25,72.79(61.1to86.72)

Djibouti,DALYs,25~30,85.23(72.81to99.77)

Djibouti,DALYs,30~35,119.76(104.28to137.53)

Djibouti,DALYs,35~40,157.53(139.07to178.44)

Djibouti,DALYs,40~45,218.22(195.34to243.79)

Djibouti,DALYs,45~50,396.95(361.79to435.54)

Djibouti,DALYs,50~55,601.92(554.15to653.81)

Djibouti,DALYs,55~60,1056.72(982.97to1135.99)

Djibouti,DALYs,60~65,1694.82(1585.76to1811.39)

Djibouti,DALYs,65~70,2502.29(2345.8to2669.21)

Djibouti,DALYs,70~75,3438.62(3217.2to3675.28)

Djibouti,DALYs,75~80,3696.09(3384.1to4036.84)

Djibouti,DALYs,80~85,4274.68(3837.65to4761.49)

Djibouti,DALYs,85~90,4106.95(3502.48to4815.75)

Djibouti,DALYs,90~95,4616.86(3531.56to6035.7)

Bahamas,Prevalence,20~25,206.07(180.36to235.44)

Bahamas,Prevalence,25~30,277.27(247.46to310.68)

Bahamas,Prevalence,30~35,372.83(337.58to411.74)

Bahamas,Prevalence,35~40,502.45(459.92to548.91)

Bahamas,Prevalence,40~45,705.15(652.38to762.19)

Bahamas,Prevalence,45~50,1080.46(1008.27to1157.83)

Bahamas,Prevalence,50~55,1682.12(1581.14to1789.55)

Bahamas,Prevalence,55~60,2526.28(2386.59to2674.15)

Bahamas,Prevalence,60~65,3620.34(3428.73to3822.66)

Bahamas,Prevalence,65~70,4999.73(4737.49to5276.48)

Bahamas,Prevalence,70~75,6849.27(6477.88to7241.96)

Bahamas,Prevalence,75~80,9412.07(8812.41to10052.53)

Bahamas,Prevalence,80~85,12896.02(12001.5to13857.21)

Bahamas,Prevalence,85~90,17331.03(15950.41to18831.16)

Bahamas,Prevalence,90~95,22633.34(20378.51to25137.68)

Bahamas,Deaths,20~25,0.15(0to11.83)

Bahamas,Deaths,25~30,0.19(0.01to6.97)

Bahamas,Deaths,30~35,0.28(0.01to5.76)

Bahamas,Deaths,35~40,0.52(0.05to5.36)

Bahamas,Deaths,40~45,1.04(0.17to6.27)

Bahamas,Deaths,45~50,2.2(0.55to8.7)

Bahamas,Deaths,50~55,4.93(1.72to14.15)

Bahamas,Deaths,55~60,9.51(4to22.62)

Bahamas,Deaths,60~65,20.47(9.89to42.36)

Bahamas,Deaths,65~70,36.77(19.09to70.81)

Bahamas,Deaths,70~75,63.11(33.37to119.34)

Bahamas,Deaths,75~80,97.47(46.21to205.58)

Bahamas,Deaths,80~85,152.67(70.22to331.93)

Bahamas,Deaths,85~90,232.69(101.01to536.02)

Bahamas,Deaths,90~95,334.23(128.6to868.71)

Bahamas,DALYs,20~25,18.69(12.27to28.45)

Bahamas,DALYs,25~30,23.39(16.37to33.43)

Bahamas,DALYs,30~35,31.28(22.92to42.68)

Bahamas,DALYs,35~40,47.84(36.77to62.24)

Bahamas,DALYs,40~45,78.09(62.64to97.35)

Bahamas,DALYs,45~50,136.92(113.81to164.73)

Bahamas,DALYs,50~55,253.01(216.61to295.53)

Bahamas,DALYs,55~60,414.49(361.36to475.43)

Bahamas,DALYs,60~65,727.6(642.89to823.47)

Bahamas,DALYs,65~70,1081.83(961.15to1217.67)

Bahamas,DALYs,70~75,1516.94(1344.61to1711.37)

Bahamas,DALYs,75~80,1896.39(1640.65to2192.01)

Bahamas,DALYs,80~85,2363.57(2015.52to2771.73)

Bahamas,DALYs,85~90,2904.93(2410.21to3501.2)

Bahamas,DALYs,90~95,3644.1(2870.36to4626.42)

Albania,Prevalence,20~25,405.71(392.16to419.73)

Albania,Prevalence,25~30,539.42(523.78to555.53)

Albania,Prevalence,30~35,693.26(675.62to711.36)

Albania,Prevalence,35~40,862.3(842.55to882.51)

Albania,Prevalence,40~45,1132.67(1110.09to1155.71)

Albania,Prevalence,45~50,1783.74(1753.7to1814.3)

Albania,Prevalence,50~55,2904.72(2863.72to2946.3)

Albania,Prevalence,55~60,4404.9(4350.07to4460.42)

Albania,Prevalence,60~65,6354.35(6280.72to6428.84)

Albania,Prevalence,65~70,9345.32(9242.14to9449.65)

Albania,Prevalence,70~75,13709.36(13561.01to13859.33)

Albania,Prevalence,75~80,19250.09(19008.9to19494.35)

Albania,Prevalence,80~85,25450.36(25104.9to25800.56)

Albania,Prevalence,85~90,31806.89(31293.1to32329.11)

Albania,Prevalence,90~95,37452.6(36594.58to38330.74)

Albania,Deaths,20~25,0.39(0.1to1.58)

Albania,Deaths,25~30,0.38(0.11to1.33)

Albania,Deaths,30~35,0.52(0.19to1.42)

Albania,Deaths,35~40,0.77(0.35to1.71)

Albania,Deaths,40~45,1.44(0.81to2.56)

Albania,Deaths,45~50,3(1.99to4.52)

Albania,Deaths,50~55,5.34(3.93to7.24)

Albania,Deaths,55~60,8.87(6.92to11.36)

Albania,Deaths,60~65,19.02(15.59to23.2)

Albania,Deaths,65~70,32.43(27.12to38.79)

Albania,Deaths,70~75,58.01(49.03to68.65)

Albania,Deaths,75~80,98.38(81.99to118.06)

Albania,Deaths,80~85,150.2(124.93to180.59)

Albania,Deaths,85~90,228.56(188.82to276.66)

Albania,Deaths,90~95,286.07(230.35to355.26)

Albania,DALYs,20~25,45.93(36.69to57.49)

Albania,DALYs,25~30,52.23(42.83to63.69)

Albania,DALYs,30~35,65.55(55.28to77.75)

Albania,DALYs,35~40,83.72(72.13to97.17)

Albania,DALYs,40~45,123.45(109.3to139.43)

Albania,DALYs,45~50,212.63(192.9to234.39)

Albania,DALYs,50~55,340.81(314.84to368.93)

Albania,DALYs,55~60,500.69(467.15to536.63)

Albania,DALYs,60~65,847.35(797.19to900.67)

Albania,DALYs,65~70,1217.9(1149.81to1290.02)

Albania,DALYs,70~75,1779.4(1682.69to1881.67)

Albania,DALYs,75~80,2420.9(2275.07to2576.07)

Albania,DALYs,80~85,2934.11(2748.21to3132.59)

Albania,DALYs,85~90,3530.04(3279.69to3799.49)

Albania,DALYs,90~95,3834.53(3477.45to4228.28)

South_Sudan,Prevalence,20~25,383.98(374.8to393.39)

South_Sudan,Prevalence,25~30,514.22(503.03to525.66)

South_Sudan,Prevalence,30~35,666.98(653.42to680.82)

South_Sudan,Prevalence,35~40,834.89(818.75to851.35)

South_Sudan,Prevalence,40~45,1072.48(1053.36to1091.95)

South_Sudan,Prevalence,45~50,1552.36(1526.46to1578.7)

South_Sudan,Prevalence,50~55,2332.8(2296.29to2369.88)

South_Sudan,Prevalence,55~60,3363.77(3313.12to3415.19)

South_Sudan,Prevalence,60~65,4552.12(4484.19to4621.09)

South_Sudan,Prevalence,65~70,5909.6(5819.58to6001.02)

South_Sudan,Prevalence,70~75,7571.05(7449.15to7694.94)

South_Sudan,Prevalence,75~80,9584.65(9403.09to9769.72)

South_Sudan,Prevalence,80~85,11769.92(11513.25to12032.3)

South_Sudan,Prevalence,85~90,13483.39(13109.73to13867.69)

South_Sudan,Prevalence,90~95,14196.96(13583.76to14837.84)

South_Sudan,Deaths,20~25,0.44(0.25to0.76)

South_Sudan,Deaths,25~30,0.53(0.32to0.88)

South_Sudan,Deaths,30~35,0.99(0.66to1.49)

South_Sudan,Deaths,35~40,1.77(1.27to2.46)

South_Sudan,Deaths,40~45,3.1(2.35to4.09)

South_Sudan,Deaths,45~50,8.24(6.73to10.08)

South_Sudan,Deaths,50~55,16.01(13.55to18.91)

South_Sudan,Deaths,55~60,37.85(33.1to43.28)

South_Sudan,Deaths,60~65,76.99(68.57to86.44)

South_Sudan,Deaths,65~70,144.9(130.4to161)

South_Sudan,Deaths,70~75,251.35(226.91to278.42)

South_Sudan,Deaths,75~80,338.47(301.21to380.34)

South_Sudan,Deaths,80~85,498.57(441.13to563.5)

South_Sudan,Deaths,85~90,579.7(503.62to667.28)

South_Sudan,Deaths,90~95,790.65(660.89to945.89)

South_Sudan,DALYs,20~25,69.19(65.79to72.76)

South_Sudan,DALYs,25~30,85.53(81.71to89.52)

South_Sudan,DALYs,30~35,123.98(119.06to129.11)

South_Sudan,DALYs,35~40,175.19(168.85to181.76)

South_Sudan,DALYs,40~45,252.69(244.5to261.14)

South_Sudan,DALYs,45~50,501.61(487.97to515.64)

South_Sudan,DALYs,50~55,828.06(807.89to848.73)

South_Sudan,DALYs,55~60,1576.3(1542.4to1610.94)

South_Sudan,DALYs,60~65,2637.01(2584.49to2690.6)

South_Sudan,DALYs,65~70,4067.26(3989.44to4146.6)

South_Sudan,DALYs,70~75,5733.94(5622.72to5847.37)

South_Sudan,DALYs,75~80,6273.07(6128.93to6420.61)

South_Sudan,DALYs,80~85,7258.02(7069.34to7451.75)

South_Sudan,DALYs,85~90,6837.7(6606.45to7077.06)

South_Sudan,DALYs,90~95,7958.05(7564.57to8371.99)

Kazakhstan,Prevalence,20~25,551.71(527.89to576.61)

Kazakhstan,Prevalence,25~30,762.3(734.24to791.43)

Kazakhstan,Prevalence,30~35,964.12(932.57to996.74)

Kazakhstan,Prevalence,35~40,1155.39(1120.65to1191.21)

Kazakhstan,Prevalence,40~45,1450.3(1411.25to1490.43)

Kazakhstan,Prevalence,45~50,2246.6(2192.79to2301.73)

Kazakhstan,Prevalence,50~55,3616.65(3540.95to3693.95)

Kazakhstan,Prevalence,55~60,5273.18(5171.06to5377.32)

Kazakhstan,Prevalence,60~65,7281.96(7147.38to7419.07)

Kazakhstan,Prevalence,65~70,10470.97(10281.93to10663.49)

Kazakhstan,Prevalence,70~75,14854.63(14580.73to15133.69)

Kazakhstan,Prevalence,75~80,20398.94(19957.7to20849.94)

Kazakhstan,Prevalence,80~85,26989.51(26354.02to27640.32)

Kazakhstan,Prevalence,85~90,34462.32(33438.71to35517.26)

Kazakhstan,Prevalence,90~95,41974.42(40167.26to43862.9)

Kazakhstan,Deaths,20~25,2.46(1.78to3.39)

Kazakhstan,Deaths,25~30,2.91(2.23to3.8)

Kazakhstan,Deaths,30~35,4.14(3.36to5.11)

Kazakhstan,Deaths,35~40,6.09(5.15to7.2)

Kazakhstan,Deaths,40~45,9.23(8.07to10.56)

Kazakhstan,Deaths,45~50,15.98(14.34to17.81)

Kazakhstan,Deaths,50~55,29.55(27.12to32.19)

Kazakhstan,Deaths,55~60,58.49(54.48to62.81)

Kazakhstan,Deaths,60~65,113.84(107.19to120.89)

Kazakhstan,Deaths,65~70,189.64(179.44to200.41)

Kazakhstan,Deaths,70~75,304.96(288.92to321.89)

Kazakhstan,Deaths,75~80,422.7(396.66to450.44)

Kazakhstan,Deaths,80~85,559.64(522.96to598.9)

Kazakhstan,Deaths,85~90,761.89(703.89to824.68)

Kazakhstan,Deaths,90~95,1025.62(923.02to1139.62)

Kazakhstan,DALYs,20~25,187.92(152.79to231.13)

Kazakhstan,DALYs,25~30,217.43(182.27to259.36)

Kazakhstan,DALYs,30~35,281.18(242.4to326.15)

Kazakhstan,DALYs,35~40,369.91(325.93to419.84)

Kazakhstan,DALYs,40~45,502.02(451.09to558.7)

Kazakhstan,DALYs,45~50,782.4(714.21to857.1)

Kazakhstan,DALYs,50~55,1289.52(1193.38to1393.4)

Kazakhstan,DALYs,55~60,2192.57(2046.46to2349.11)

Kazakhstan,DALYs,60~65,3618.04(3401.85to3847.96)

Kazakhstan,DALYs,65~70,5076.79(4784.79to5386.61)

Kazakhstan,DALYs,70~75,6756.75(6358.77to7179.64)

Kazakhstan,DALYs,75~80,7597.79(7054.33to8183.11)

Kazakhstan,DALYs,80~85,8081.65(7422.53to8799.3)

Kazakhstan,DALYs,85~90,8836.69(7890.09to9896.85)

Kazakhstan,DALYs,90~95,10293.32(8666.41to12225.64)

Mongolia,Prevalence,20~25,545.35(526.81to564.54)

Mongolia,Prevalence,25~30,723.13(701.35to745.6)

Mongolia,Prevalence,30~35,899.94(875.05to925.54)

Mongolia,Prevalence,35~40,1068.32(1040.33to1097.06)

Mongolia,Prevalence,40~45,1308.58(1276.68to1341.27)

Mongolia,Prevalence,45~50,1869.92(1827.79to1913.02)

Mongolia,Prevalence,50~55,2846.03(2787.39to2905.91)

Mongolia,Prevalence,55~60,4185.1(4104.39to4267.4)

Mongolia,Prevalence,60~65,5880.45(5769.54to5993.5)

Mongolia,Prevalence,65~70,8168.45(8014.5to8325.35)

Mongolia,Prevalence,70~75,11285.81(11066.94to11509)

Mongolia,Prevalence,75~80,15570.94(15220.91to15929.03)

Mongolia,Prevalence,80~85,21130.96(20613.28to21661.63)

Mongolia,Prevalence,85~90,27640.82(26817.36to28489.57)

Mongolia,Prevalence,90~95,33245.32(31619.74to34954.48)

Mongolia,Deaths,20~25,2.63(1.31to5.27)

Mongolia,Deaths,25~30,2.85(1.57to5.2)

Mongolia,Deaths,30~35,3.72(2.24to6.15)

Mongolia,Deaths,35~40,4.98(3.22to7.71)

Mongolia,Deaths,40~45,6.96(4.81to10.08)

Mongolia,Deaths,45~50,10.18(7.42to13.97)

Mongolia,Deaths,50~55,16.74(12.84to21.84)

Mongolia,Deaths,55~60,25.67(20.28to32.49)

Mongolia,Deaths,60~65,47.47(38.49to58.55)

Mongolia,Deaths,65~70,76.76(62.93to93.62)

Mongolia,Deaths,70~75,103.17(84.55to125.9)

Mongolia,Deaths,75~80,153.3(123.36to190.52)

Mongolia,Deaths,80~85,214.87(171.7to268.88)

Mongolia,Deaths,85~90,297.94(232.83to381.26)

Mongolia,Deaths,90~95,353.02(251.4to495.74)

Mongolia,DALYs,20~25,192.57(176.28to210.37)

Mongolia,DALYs,25~30,210.52(194.61to227.73)

Mongolia,DALYs,30~35,254.34(237.15to272.78)

Mongolia,DALYs,35~40,307.92(288.8to328.31)

Mongolia,DALYs,40~45,386.49(365.04to409.2)

Mongolia,DALYs,45~50,517.69(491.69to545.05)

Mongolia,DALYs,50~55,765.19(730.77to801.23)

Mongolia,DALYs,55~60,1049.71(1005.53to1095.83)

Mongolia,DALYs,60~65,1640.71(1575.75to1708.34)

Mongolia,DALYs,65~70,2242.87(2155.27to2334.03)

Mongolia,DALYs,70~75,2540.91(2437.3to2648.92)

Mongolia,DALYs,75~80,3059.82(2919to3207.43)

Mongolia,DALYs,80~85,3450.36(3276.64to3633.3)

Mongolia,DALYs,85~90,3868.85(3631.12to4122.15)

Mongolia,DALYs,90~95,4002.69(3604.32to4445.08)

Bulgaria,Prevalence,20~25,424.46(414.13to435.05)

Bulgaria,Prevalence,25~30,580.24(568.54to592.19)

Bulgaria,Prevalence,30~35,757(744.02to770.21)

Bulgaria,Prevalence,35~40,942.85(928.8to957.11)

Bulgaria,Prevalence,40~45,1262.06(1246.48to1277.83)

Bulgaria,Prevalence,45~50,2133.46(2112.49to2154.64)

Bulgaria,Prevalence,50~55,3533.01(3504.36to3561.9)

Bulgaria,Prevalence,55~60,5150.35(5113.68to5187.27)

Bulgaria,Prevalence,60~65,6986.6(6940.69to7032.81)

Bulgaria,Prevalence,65~70,9574.21(9514.24to9634.56)

Bulgaria,Prevalence,70~75,13183.1(13101.87to13264.82)

Bulgaria,Prevalence,75~80,17668.53(17545.03to17792.9)

Bulgaria,Prevalence,80~85,22858.61(22687.89to23030.61)

Bulgaria,Prevalence,85~90,28180.83(27935.29to28428.53)

Bulgaria,Prevalence,90~95,33145.28(32669.84to33627.63)

Bulgaria,Deaths,20~25,0.37(0.16to0.87)

Bulgaria,Deaths,25~30,0.45(0.22to0.92)

Bulgaria,Deaths,30~35,0.74(0.45to1.24)

Bulgaria,Deaths,35~40,1.42(1to2.01)

Bulgaria,Deaths,40~45,3.2(2.56to3.99)

Bulgaria,Deaths,45~50,7.39(6.38to8.55)

Bulgaria,Deaths,50~55,14.03(12.58to15.65)

Bulgaria,Deaths,55~60,23.55(21.57to25.72)

Bulgaria,Deaths,60~65,41.76(38.75to45)

Bulgaria,Deaths,65~70,57.86(53.95to62.05)

Bulgaria,Deaths,70~75,82.96(77.54to88.77)

Bulgaria,Deaths,75~80,114.56(106.36to123.39)

Bulgaria,Deaths,80~85,160.62(148.88to173.28)

Bulgaria,Deaths,85~90,259.8(239.75to281.53)

Bulgaria,Deaths,90~95,357.72(321.94to397.47)

Bulgaria,DALYs,20~25,49.95(43.66to57.14)

Bulgaria,DALYs,25~30,62.67(56.03to70.09)

Bulgaria,DALYs,30~35,85.31(77.93to93.39)

Bulgaria,DALYs,35~40,123.9(115.31to133.13)

Bulgaria,DALYs,40~45,213.67(202.8to225.12)

Bulgaria,DALYs,45~50,416.68(401.08to432.88)

Bulgaria,DALYs,50~55,699.15(677.98to720.99)

Bulgaria,DALYs,55~60,1023.56(996.72to1051.11)

Bulgaria,DALYs,60~65,1520.04(1483.98to1556.98)

Bulgaria,DALYs,65~70,1825.82(1783.73to1868.9)

Bulgaria,DALYs,70~75,2213.01(2162.06to2265.17)

Bulgaria,DALYs,75~80,2535.13(2469.09to2602.94)

Bulgaria,DALYs,80~85,2867(2786.71to2949.61)

Bulgaria,DALYs,85~90,3649.82(3531.97to3771.59)

Bulgaria,DALYs,90~95,4321.38(4100.59to4554.07)

United_Republic_of_Tanzania,Prevalence,20~25,385.65(379.84to391.54)

United_Republic_of_Tanzania,Prevalence,25~30,515.67(508.63to522.8)

United_Republic_of_Tanzania,Prevalence,30~35,666.7(658.21to675.3)

United_Republic_of_Tanzania,Prevalence,35~40,833.85(823.8to844.01)

United_Republic_of_Tanzania,Prevalence,40~45,1068.95(1057.03to1081.01)

United_Republic_of_Tanzania,Prevalence,45~50,1528.87(1513.08to1544.82)

United_Republic_of_Tanzania,Prevalence,50~55,2253.79(2232.29to2275.51)

United_Republic_of_Tanzania,Prevalence,55~60,3202.43(3173.54to3231.6)

United_Republic_of_Tanzania,Prevalence,60~65,4301.78(4264.25to4339.65)

United_Republic_of_Tanzania,Prevalence,65~70,5578.95(5530.39to5627.93)

United_Republic_of_Tanzania,Prevalence,70~75,7159.83(7094.47to7225.8)

United_Republic_of_Tanzania,Prevalence,75~80,9098.13(8998.7to9198.67)

United_Republic_of_Tanzania,Prevalence,80~85,11267.28(11125.5to11410.88)

United_Republic_of_Tanzania,Prevalence,85~90,13163.92(12948.37to13383.05)

United_Republic_of_Tanzania,Prevalence,90~95,14507.39(14129.06to14895.84)

United_Republic_of_Tanzania,Deaths,20~25,0.51(0.38to0.67)

United_Republic_of_Tanzania,Deaths,25~30,0.61(0.47to0.79)

United_Republic_of_Tanzania,Deaths,30~35,1.06(0.86to1.3)

United_Republic_of_Tanzania,Deaths,35~40,1.76(1.48to2.09)

United_Republic_of_Tanzania,Deaths,40~45,2.8(2.42to3.23)

United_Republic_of_Tanzania,Deaths,45~50,6.61(5.94to7.35)

United_Republic_of_Tanzania,Deaths,50~55,12.18(11.18to13.27)

United_Republic_of_Tanzania,Deaths,55~60,26.65(24.9to28.52)

United_Republic_of_Tanzania,Deaths,60~65,48.59(45.83to51.51)

United_Republic_of_Tanzania,Deaths,65~70,84.05(79.66to88.69)

United_Republic_of_Tanzania,Deaths,70~75,142.57(135.36to150.17)

United_Republic_of_Tanzania,Deaths,75~80,198.68(187.18to210.88)

United_Republic_of_Tanzania,Deaths,80~85,299.64(281.5to318.95)

United_Republic_of_Tanzania,Deaths,85~90,349.97(325.47to376.31)

United_Republic_of_Tanzania,Deaths,90~95,451.1(409.86to496.5)

United_Republic_of_Tanzania,DALYs,20~25,75.14(72.31to78.09)

United_Republic_of_Tanzania,DALYs,25~30,94.12(90.92to97.44)

United_Republic_of_Tanzania,DALYs,30~35,130.68(126.67to134.82)

United_Republic_of_Tanzania,DALYs,35~40,177.38(172.41to182.5)

United_Republic_of_Tanzania,DALYs,40~45,240.66(234.57to246.91)

United_Republic_of_Tanzania,DALYs,45~50,429.13(419.94to438.53)

United_Republic_of_Tanzania,DALYs,50~55,677.19(664.4to690.22)

United_Republic_of_Tanzania,DALYs,55~60,1189.42(1169.83to1209.34)

United_Republic_of_Tanzania,DALYs,60~65,1803.9(1776.42to1831.8)

United_Republic_of_Tanzania,DALYs,65~70,2571.08(2533.18to2609.54)

United_Republic_of_Tanzania,DALYs,70~75,3535.32(3482.49to3588.95)

United_Republic_of_Tanzania,DALYs,75~80,4014.48(3943.06to4087.2)

United_Republic_of_Tanzania,DALYs,80~85,4759.92(4664.57to4857.22)

United_Republic_of_Tanzania,DALYs,85~90,4558.64(4437.61to4682.96)

United_Republic_of_Tanzania,DALYs,90~95,5044.82(4839.19to5259.19)

Belize,Prevalence,20~25,260.87(228.72to297.52)

Belize,Prevalence,25~30,355.89(316.67to399.97)

Belize,Prevalence,30~35,478.96(430.87to532.41)

Belize,Prevalence,35~40,632.77(574.33to697.16)

Belize,Prevalence,40~45,870.87(797.84to950.58)

Belize,Prevalence,45~50,1374.54(1271.39to1486.06)

Belize,Prevalence,50~55,2227.82(2077.92to2388.54)

Belize,Prevalence,55~60,3418.39(3208.54to3641.97)

Belize,Prevalence,60~65,4899.4(4615.16to5201.15)

Belize,Prevalence,65~70,6728.86(6346.59to7134.15)

Belize,Prevalence,70~75,9180.94(8648.28to9746.41)

Belize,Prevalence,75~80,12367.17(11511.04to13286.98)

Belize,Prevalence,80~85,16490.42(15258.84to17821.41)

Belize,Prevalence,85~90,21497.08(19666.2to23498.42)

Belize,Prevalence,90~95,27611.12(24671.17to30901.41)

Belize,Deaths,20~25,0.29(0.01to6.36)

Belize,Deaths,25~30,0.45(0.03to6.57)

Belize,Deaths,30~35,0.5(0.04to5.94)

Belize,Deaths,35~40,0.91(0.12to6.87)

Belize,Deaths,40~45,1.71(0.33to8.81)

Belize,Deaths,45~50,4.08(1.18to14.14)

Belize,Deaths,50~55,9.14(3.51to23.83)

Belize,Deaths,55~60,19.7(9.07to42.76)

Belize,Deaths,60~65,43.73(23.16to82.56)

Belize,Deaths,65~70,74.14(41.68to131.87)

Belize,Deaths,70~75,143.01(82.35to248.33)

Belize,Deaths,75~80,228.99(119.06to440.44)

Belize,Deaths,80~85,372.44(188.7to735.1)

Belize,Deaths,85~90,541.17(259.71to1127.69)

Belize,Deaths,90~95,799.46(347.69to1838.23)

Belize,DALYs,20~25,28.48(20.18to40.2)

Belize,DALYs,25~30,40.93(30.15to55.58)

Belize,DALYs,30~35,45.73(34.39to60.8)

Belize,DALYs,35~40,70.57(55to90.56)

Belize,DALYs,40~45,113.77(91.71to141.14)

Belize,DALYs,45~50,225.44(188.73to269.3)

Belize,DALYs,50~55,431.49(372.28to500.12)

Belize,DALYs,55~60,787.03(692.68to894.24)

Belize,DALYs,60~65,1448.32(1294.37to1620.57)

Belize,DALYs,65~70,2055.54(1845.39to2289.62)

Belize,DALYs,70~75,3202.67(2874.17to3568.71)

Belize,DALYs,75~80,4106.83(3601.67to4682.84)

Belize,DALYs,80~85,5236.79(4541.05to6039.14)

Belize,DALYs,85~90,6098.27(5161.62to7204.9)

Belize,DALYs,90~95,7814.3(6325.24to9653.91)

Jamaica,Prevalence,20~25,296.39(284.58to308.68)

Jamaica,Prevalence,25~30,394.83(380.99to409.17)

Jamaica,Prevalence,30~35,521.06(504.62to538.03)

Jamaica,Prevalence,35~40,676.71(657.32to696.68)

Jamaica,Prevalence,40~45,915.49(892.07to939.52)

Jamaica,Prevalence,45~50,1421.72(1389.9to1454.27)

Jamaica,Prevalence,50~55,2267.26(2222.6to2312.81)

Jamaica,Prevalence,55~60,3409.77(3348.82to3471.82)

Jamaica,Prevalence,60~65,4780.26(4700.12to4861.76)

Jamaica,Prevalence,65~70,6534.67(6427.36to6643.76)

Jamaica,Prevalence,70~75,8818.05(8672.05to8966.5)

Jamaica,Prevalence,75~80,11660.72(11439.15to11886.59)

Jamaica,Prevalence,80~85,15288.56(14981.02to15602.42)

Jamaica,Prevalence,85~90,19770.37(19336.77to20213.7)

Jamaica,Prevalence,90~95,25327.42(24684.95to25986.61)

Jamaica,Deaths,20~25,0.13(0.03to0.59)

Jamaica,Deaths,25~30,0.19(0.05to0.68)

Jamaica,Deaths,30~35,0.29(0.1to0.85)

Jamaica,Deaths,35~40,0.6(0.27to1.34)

Jamaica,Deaths,40~45,1.24(0.68to2.28)

Jamaica,Deaths,45~50,2.72(1.74to4.25)

Jamaica,Deaths,50~55,6.67(4.82to9.22)

Jamaica,Deaths,55~60,15.77(12.33to20.18)

Jamaica,Deaths,60~65,34.13(27.96to41.67)

Jamaica,Deaths,65~70,57.47(47.83to69.06)

Jamaica,Deaths,70~75,112.94(95.16to134.03)

Jamaica,Deaths,75~80,169.16(139.25to205.49)

Jamaica,Deaths,80~85,234.71(191.98to286.95)

Jamaica,Deaths,85~90,306.6(247.9to379.22)

Jamaica,Deaths,90~95,397.39(314.8to501.64)

Jamaica,DALYs,20~25,20.39(17.83to23.33)

Jamaica,DALYs,25~30,27.24(24.23to30.64)

Jamaica,DALYs,30~35,36.84(33.2to40.88)

Jamaica,DALYs,35~40,58.02(53.16to63.33)

Jamaica,DALYs,40~45,95.18(88.44to102.43)

Jamaica,DALYs,45~50,171.45(161.5to182.01)

Jamaica,DALYs,50~55,340.52(324.61to357.2)

Jamaica,DALYs,55~60,652.43(626.85to679.04)

Jamaica,DALYs,60~65,1153.99(1114.31to1195.09)

Jamaica,DALYs,65~70,1621(1567to1676.85)

Jamaica,DALYs,70~75,2551.65(2469.06to2637.02)

Jamaica,DALYs,75~80,3082.89(2967.31to3202.97)

Jamaica,DALYs,80~85,3424.48(3285.92to3568.89)

Jamaica,DALYs,85~90,3663.49(3495.97to3839.04)

Jamaica,DALYs,90~95,4224.3(3993.8to4468.11)

Dominica,Prevalence,20~25,234.03(175.75to311.62)

Dominica,Prevalence,25~30,309.07(241.16to396.12)

Dominica,Prevalence,30~35,408.18(328.59to507.04)

Dominica,Prevalence,35~40,540.41(445.4to655.69)

Dominica,Prevalence,40~45,748.56(633.1to885.07)

Dominica,Prevalence,45~50,1150.91(994.75to1331.59)

Dominica,Prevalence,50~55,1832.32(1612.11to2082.62)

Dominica,Prevalence,55~60,2825.33(2517.24to3171.13)

Dominica,Prevalence,60~65,4104.94(3684.73to4573.07)

Dominica,Prevalence,65~70,5693.08(5126.52to6322.26)

Dominica,Prevalence,70~75,7786.75(7002to8659.45)

Dominica,Prevalence,75~80,10516.29(9312.78to11875.33)

Dominica,Prevalence,80~85,14142.75(12415.69to16110.04)

Dominica,Prevalence,85~90,18629.44(16051.83to21620.96)

Dominica,Prevalence,90~95,23809.96(19578.47to28955.99)

Dominica,Deaths,20~25,0.11(0to3374.25)

Dominica,Deaths,25~30,0.13(0to1594.42)

Dominica,Deaths,30~35,0.19(0to541.25)

Dominica,Deaths,35~40,0.43(0to124.56)

Dominica,Deaths,40~45,1.05(0.02to50.69)

Dominica,Deaths,45~50,2.56(0.17to38.45)

Dominica,Deaths,50~55,6.61(0.97to45.11)

Dominica,Deaths,55~60,15.37(3.59to65.92)

Dominica,Deaths,60~65,35.51(11.21to112.51)

Dominica,Deaths,65~70,65.23(23.25to183.01)

Dominica,Deaths,70~75,122.84(46.12to327.19)

Dominica,Deaths,75~80,200.09(66.42to602.77)

Dominica,Deaths,80~85,313.96(101.77to968.6)

Dominica,Deaths,85~90,475.1(144.51to1561.95)

Dominica,Deaths,90~95,630.99(157.24to2532.19)

Dominica,DALYs,20~25,16.98(6.66to43.25)

Dominica,DALYs,25~30,20.4(8.85to47.02)

Dominica,DALYs,30~35,26.74(12.92to55.33)

Dominica,DALYs,35~40,43.96(24.22to79.8)

Dominica,DALYs,40~45,79.95(50.09to127.6)

Dominica,DALYs,45~50,154.97(107.43to223.56)

Dominica,DALYs,50~55,323.17(242.12to431.35)

Dominica,DALYs,55~60,621.57(489.25to789.68)

Dominica,DALYs,60~65,1176.67(958.34to1444.75)

Dominica,DALYs,65~70,1788.83(1473.49to2171.65)

Dominica,DALYs,70~75,2723.74(2248.78to3299.01)

Dominica,DALYs,75~80,3556.8(2853.66to4433.21)

Dominica,DALYs,80~85,4409.31(3482.79to5582.31)

Dominica,DALYs,85~90,5352.16(4085.06to7012.29)

Dominica,DALYs,90~95,6247.51(4361.29to8949.5)

Saint_Vincent_and_the_Grenadines,Prevalence,20~25,199.54(158.61to251.03)

Saint_Vincent_and_the_Grenadines,Prevalence,25~30,264.87(216.22to324.46)

Saint_Vincent_and_the_Grenadines,Prevalence,30~35,353.11(294.56to423.31)

Saint_Vincent_and_the_Grenadines,Prevalence,35~40,474.6(403.38to558.4)

Saint_Vincent_and_the_Grenadines,Prevalence,40~45,667.68(578.69to770.37)

Saint_Vincent_and_the_Grenadines,Prevalence,45~50,1024.04(901.72to1162.96)

Saint_Vincent_and_the_Grenadines,Prevalence,50~55,1593.19(1423.23to1783.45)

Saint_Vincent_and_the_Grenadines,Prevalence,55~60,2410.18(2177.16to2668.15)

Saint_Vincent_and_the_Grenadines,Prevalence,60~65,3492.01(3176.66to3838.67)

Saint_Vincent_and_the_Grenadines,Prevalence,65~70,4885.81(4460.08to5352.18)

Saint_Vincent_and_the_Grenadines,Prevalence,70~75,6849.65(6253.01to7503.21)

Saint_Vincent_and_the_Grenadines,Prevalence,75~80,9593.17(8635.07to10657.59)

Saint_Vincent_and_the_Grenadines,Prevalence,80~85,13189.35(11794.9to14748.66)

Saint_Vincent_and_the_Grenadines,Prevalence,85~90,17727.7(15602.57to20142.29)

Saint_Vincent_and_the_Grenadines,Prevalence,90~95,22814.23(19116.08to27227.8)

Saint_Vincent_and_the_Grenadines,Deaths,20~25,0.05(0to1273.93)

Saint_Vincent_and_the_Grenadines,Deaths,25~30,0.09(0to313.91)

Saint_Vincent_and_the_Grenadines,Deaths,30~35,0.13(0to105.97)

Saint_Vincent_and_the_Grenadines,Deaths,35~40,0.27(0to44.94)

Saint_Vincent_and_the_Grenadines,Deaths,40~45,0.63(0.01to27.36)

Saint_Vincent_and_the_Grenadines,Deaths,45~50,1.58(0.1to25.18)

Saint_Vincent_and_the_Grenadines,Deaths,50~55,3.64(0.46to29.07)

Saint_Vincent_and_the_Grenadines,Deaths,55~60,7.6(1.44to40.14)

Saint_Vincent_and_the_Grenadines,Deaths,60~65,17.48(4.62to66.13)

Saint_Vincent_and_the_Grenadines,Deaths,65~70,33.79(10.62to107.52)

Saint_Vincent_and_the_Grenadines,Deaths,70~75,61.84(20.6to185.62)

Saint_Vincent_and_the_Grenadines,Deaths,75~80,115.58(32.86to406.54)

Saint_Vincent_and_the_Grenadines,Deaths,80~85,190.43(52.18to694.96)

Saint_Vincent_and_the_Grenadines,Deaths,85~90,369.44(95.82to1424.4)

Saint_Vincent_and_the_Grenadines,Deaths,90~95,567.92(120.09to2685.76)

Saint_Vincent_and_the_Grenadines,DALYs,20~25,10.12(4.43to23.13)

Saint_Vincent_and_the_Grenadines,DALYs,25~30,14.38(7.05to29.37)

Saint_Vincent_and_the_Grenadines,DALYs,30~35,19.68(10.55to36.68)

Saint_Vincent_and_the_Grenadines,DALYs,35~40,30.9(18.17to52.56)

Saint_Vincent_and_the_Grenadines,DALYs,40~45,53.66(34.5to83.47)

Saint_Vincent_and_the_Grenadines,DALYs,45~50,104.88(72.97to150.74)

Saint_Vincent_and_the_Grenadines,DALYs,50~55,197.51(146.42to266.41)

Saint_Vincent_and_the_Grenadines,DALYs,55~60,342.5(264.27to443.89)

Saint_Vincent_and_the_Grenadines,DALYs,60~65,631.11(503.44to791.16)

Saint_Vincent_and_the_Grenadines,DALYs,65~70,997.81(809.22to1230.36)

Saint_Vincent_and_the_Grenadines,DALYs,70~75,1476.9(1199.54to1818.39)

Saint_Vincent_and_the_Grenadines,DALYs,75~80,2175.81(1709.68to2769.02)

Saint_Vincent_and_the_Grenadines,DALYs,80~85,2823.7(2182.11to3653.94)

Saint_Vincent_and_the_Grenadines,DALYs,85~90,4247.27(3190.22to5654.57)

Saint_Vincent_and_the_Grenadines,DALYs,90~95,5631(3851.52to8232.65)

Zambia,Prevalence,20~25,385.13(377.45to392.96)

Zambia,Prevalence,25~30,515.36(505.98to524.92)

Zambia,Prevalence,30~35,668.63(657.2to680.27)

Zambia,Prevalence,35~40,836.84(823.17to850.72)

Zambia,Prevalence,40~45,1075.13(1058.84to1091.67)

Zambia,Prevalence,45~50,1561.35(1539.33to1583.69)

Zambia,Prevalence,50~55,2334.5(2303.74to2365.67)

Zambia,Prevalence,55~60,3332.52(3290.56to3375.01)

Zambia,Prevalence,60~65,4448.5(4393.9to4503.79)

Zambia,Prevalence,65~70,5700.02(5629.84to5771.08)

Zambia,Prevalence,70~75,7244.55(7151.64to7338.66)

Zambia,Prevalence,75~80,9100.49(8959.41to9243.79)

Zambia,Prevalence,80~85,11174.02(10966.98to11384.96)

Zambia,Prevalence,85~90,12806.44(12474.92to13146.76)

Zambia,Prevalence,90~95,13632.35(13002.58to14292.62)

Zambia,Deaths,20~25,0.59(0.38to0.91)

Zambia,Deaths,25~30,0.69(0.46to1.02)

Zambia,Deaths,30~35,1.29(0.93to1.78)

Zambia,Deaths,35~40,2.22(1.69to2.91)

Zambia,Deaths,40~45,3.89(3.11to4.87)

Zambia,Deaths,45~50,9.84(8.34to11.61)

Zambia,Deaths,50~55,16.92(14.72to19.45)

Zambia,Deaths,55~60,37.07(33.14to41.46)

Zambia,Deaths,60~65,71.28(64.76to78.45)

Zambia,Deaths,65~70,123.75(113.29to135.17)

Zambia,Deaths,70~75,220.22(202.34to239.67)

Zambia,Deaths,75~80,295.67(267.84to326.4)

Zambia,Deaths,80~85,432.69(388.84to481.5)

Zambia,Deaths,85~90,490.69(429.49to560.61)

Zambia,Deaths,90~95,637.11(522.83to776.38)

Zambia,DALYs,20~25,78.58(74.81to82.54)

Zambia,DALYs,25~30,95.82(91.61to100.22)

Zambia,DALYs,30~35,141.64(136.03to147.47)

Zambia,DALYs,35~40,199.25(192.03to206.74)

Zambia,DALYs,40~45,290.19(280.8to299.91)

Zambia,DALYs,45~50,568.09(552.7to583.9)

Zambia,DALYs,50~55,863.43(842.35to885.04)

Zambia,DALYs,55~60,1550.27(1516.95to1584.32)

Zambia,DALYs,60~65,2473.05(2424.27to2522.81)

Zambia,DALYs,65~70,3547.8(3479.86to3617.07)

Zambia,DALYs,70~75,5098.71(5000.74to5198.59)

Zambia,DALYs,75~80,5577.37(5447.61to5710.23)

Zambia,DALYs,80~85,6436.48(6260.53to6617.38)

Zambia,DALYs,85~90,5951.6(5716.31to6196.57)

Zambia,DALYs,90~95,6587.9(6142.24to7065.9)

Ethiopia,Prevalence,20~25,532.97(517.14to549.29)

Ethiopia,Prevalence,25~30,706.82(687.55to726.63)

Ethiopia,Prevalence,30~35,887.8(865.13to911.07)

Ethiopia,Prevalence,35~40,1066.94(1041.25to1093.27)

Ethiopia,Prevalence,40~45,1317.35(1288.15to1347.22)

Ethiopia,Prevalence,45~50,1872.67(1834.03to1912.13)

Ethiopia,Prevalence,50~55,2778.17(2724.79to2832.59)

Ethiopia,Prevalence,55~60,3909.02(3837.41to3981.97)

Ethiopia,Prevalence,60~65,5154.14(5061.95to5248)

Ethiopia,Prevalence,65~70,6572.1(6454.42to6691.92)

Ethiopia,Prevalence,70~75,8235.86(8082.28to8392.35)

Ethiopia,Prevalence,75~80,10032.25(9809.34to10260.23)

Ethiopia,Prevalence,80~85,11713.64(11405to12030.63)

Ethiopia,Prevalence,85~90,12789.6(12324.25to13272.52)

Ethiopia,Prevalence,90~95,13015.09(12179.21to13908.33)

Ethiopia,Deaths,20~25,1.27(1.06to1.52)

Ethiopia,Deaths,25~30,1.41(1.19to1.66)

Ethiopia,Deaths,30~35,2.14(1.87to2.45)

Ethiopia,Deaths,35~40,3.32(2.98to3.71)

Ethiopia,Deaths,40~45,5.01(4.59to5.48)

Ethiopia,Deaths,45~50,11.23(10.53to11.98)

Ethiopia,Deaths,50~55,17.58(16.63to18.57)

Ethiopia,Deaths,55~60,35.31(33.77to36.92)

Ethiopia,Deaths,60~65,62.8(60.4to65.3)

Ethiopia,Deaths,65~70,100.14(96.58to103.83)

Ethiopia,Deaths,70~75,159.64(154.21to165.26)

Ethiopia,Deaths,75~80,192.21(184.71to200.01)

Ethiopia,Deaths,80~85,269.81(258.61to281.49)

Ethiopia,Deaths,85~90,297.23(282.24to313.01)

Ethiopia,Deaths,90~95,407.35(378.94to437.9)

Ethiopia,DALYs,20~25,150.23(141.43to159.57)

Ethiopia,DALYs,25~30,178.12(168.5to188.28)

Ethiopia,DALYs,30~35,229.08(217.83to240.91)

Ethiopia,DALYs,35~40,292.79(279.83to306.34)

Ethiopia,DALYs,40~45,377.75(362.97to393.12)

Ethiopia,DALYs,45~50,657.55(635.93to679.91)

Ethiopia,DALYs,50~55,931.18(903.19to960.05)

Ethiopia,DALYs,55~60,1535.9(1494.79to1578.15)

Ethiopia,DALYs,60~65,2278(2221.03to2336.43)

Ethiopia,DALYs,65~70,3028.02(2954.5to3103.37)

Ethiopia,DALYs,70~75,3934.58(3838.76to4032.79)

Ethiopia,DALYs,75~80,3908.6(3794.78to4025.84)

Ethiopia,DALYs,80~85,4316.89(4171.31to4467.54)

Ethiopia,DALYs,85~90,3883.87(3698.68to4078.33)

Ethiopia,DALYs,90~95,4473.23(4127.45to4847.97)

Madagascar,Prevalence,20~25,466.04(459.32to472.86)

Madagascar,Prevalence,25~30,639.62(631.32to648.03)

Madagascar,Prevalence,30~35,820.53(810.64to830.54)

Madagascar,Prevalence,35~40,998.76(987.34to1010.31)

Madagascar,Prevalence,40~45,1245.48(1232.23to1258.87)

Madagascar,Prevalence,45~50,1784.56(1766.94to1802.36)

Madagascar,Prevalence,50~55,2686.71(2662.22to2711.42)

Madagascar,Prevalence,55~60,3875.9(3842.41to3909.67)

Madagascar,Prevalence,60~65,5238.99(5194.39to5283.98)

Madagascar,Prevalence,65~70,6825.5(6765.93to6885.59)

Madagascar,Prevalence,70~75,8895.17(8811.54to8979.58)

Madagascar,Prevalence,75~80,11648.72(11513.96to11785.06)

Madagascar,Prevalence,80~85,15013.63(14807.7to15222.42)

Madagascar,Prevalence,85~90,17962.67(17632.53to18298.99)

Madagascar,Prevalence,90~95,19395.39(18799.03to20010.68)

Madagascar,Deaths,20~25,1.96(1.58to2.42)

Madagascar,Deaths,25~30,2.41(1.99to2.92)

Madagascar,Deaths,30~35,3.35(2.83to3.96)

Madagascar,Deaths,35~40,4.68(4.03to5.44)

Madagascar,Deaths,40~45,4.98(4.3to5.76)

Madagascar,Deaths,45~50,10.49(9.36to11.76)

Madagascar,Deaths,50~55,21.3(19.46to23.33)

Madagascar,Deaths,55~60,49.86(46.42to53.54)

Madagascar,Deaths,60~65,105.8(99.61to112.38)

Madagascar,Deaths,65~70,193.53(183.04to204.63)

Madagascar,Deaths,70~75,349.66(331.16to369.18)

Madagascar,Deaths,75~80,518.06(485.96to552.28)

Madagascar,Deaths,80~85,800.14(747.32to856.7)

Madagascar,Deaths,85~90,1135.92(1049.91to1228.98)

Madagascar,Deaths,90~95,1489.43(1337.6to1658.49)

Madagascar,DALYs,20~25,176.41(172.2to180.73)

Madagascar,DALYs,25~30,213.79(209.15to218.54)

Madagascar,DALYs,30~35,273.33(267.87to278.9)

Madagascar,DALYs,35~40,343.6(337.22to350.1)

Madagascar,DALYs,40~45,361.53(355.01to368.17)

Madagascar,DALYs,45~50,622.52(612.84to632.36)

Madagascar,DALYs,50~55,1066.35(1052.02to1080.88)

Madagascar,DALYs,55~60,2029.22(2005.68to2053.03)

Madagascar,DALYs,60~65,3543.99(3506.65to3581.72)

Madagascar,DALYs,65~70,5356.03(5301.03to5411.6)

Madagascar,DALYs,70~75,7860.49(7777.83to7944.02)

Madagascar,DALYs,75~80,9392.99(9271.79to9515.79)

Madagascar,DALYs,80~85,11396.16(11225.55to11569.37)

Madagascar,DALYs,85~90,12860.73(12608.09to13118.44)

Madagascar,DALYs,90~95,14490.03(14039.88to14954.6)

Czechia,Prevalence,20~25,370.06(359.51to380.92)

Czechia,Prevalence,25~30,503.34(491.46to515.5)

Czechia,Prevalence,30~35,659.87(646.55to673.46)

Czechia,Prevalence,35~40,834.68(820.12to849.5)

Czechia,Prevalence,40~45,1131.15(1114.77to1147.77)

Czechia,Prevalence,45~50,1900.79(1878.72to1923.11)

Czechia,Prevalence,50~55,3215.66(3184.8to3246.81)

Czechia,Prevalence,55~60,4895.45(4854.47to4936.77)

Czechia,Prevalence,60~65,6931.83(6878.86to6985.21)

Czechia,Prevalence,65~70,9891.82(9820.79to9963.37)

Czechia,Prevalence,70~75,14017.14(13918.54to14116.45)

Czechia,Prevalence,75~80,19277.2(19118.33to19437.39)

Czechia,Prevalence,80~85,25531.45(25304.79to25760.14)

Czechia,Prevalence,85~90,32578.32(32249.31to32910.69)

Czechia,Prevalence,90~95,40030.63(39496.73to40571.75)

Czechia,Deaths,20~25,0.27(0.11to0.71)

Czechia,Deaths,25~30,0.28(0.13to0.64)

Czechia,Deaths,30~35,0.43(0.23to0.78)

Czechia,Deaths,35~40,0.76(0.51to1.15)

Czechia,Deaths,40~45,1.71(1.33to2.2)

Czechia,Deaths,45~50,4.34(3.7to5.1)

Czechia,Deaths,50~55,9.36(8.34to10.5)

Czechia,Deaths,55~60,19.24(17.67to20.95)

Czechia,Deaths,60~65,40.21(37.62to42.98)

Czechia,Deaths,65~70,69.86(65.93to74.02)

Czechia,Deaths,70~75,119.15(112.8to125.85)

Czechia,Deaths,75~80,191.56(179.52to204.41)

Czechia,Deaths,80~85,296.27(276.87to317.03)

Czechia,Deaths,85~90,447.5(416.11to481.27)

Czechia,Deaths,90~95,576.78(527.41to630.77)

Czechia,DALYs,20~25,41.23(35.07to48.48)

Czechia,DALYs,25~30,49.89(43.55to57.15)

Czechia,DALYs,30~35,64.76(57.89to72.44)

Czechia,DALYs,35~40,86.27(78.82to94.42)

Czechia,DALYs,40~45,138.21(129.24to147.8)

Czechia,DALYs,45~50,277.56(264.35to291.43)

Czechia,DALYs,50~55,510.65(491.47to530.58)

Czechia,DALYs,55~60,873.25(846.29to901.07)

Czechia,DALYs,60~65,1475.63(1436.52to1515.81)

Czechia,DALYs,65~70,2133.24(2081.12to2186.66)

Czechia,DALYs,70~75,2969(2897.25to3042.54)

Czechia,DALYs,75~80,3848.93(3736.85to3964.36)

Czechia,DALYs,80~85,4724.71(4573.47to4880.95)

Czechia,DALYs,85~90,5716.87(5504.22to5937.74)

Czechia,DALYs,90~95,6532.57(6195.53to6887.95)

North_Macedonia,Prevalence,20~25,437.11(420.4to454.48)

North_Macedonia,Prevalence,25~30,586.59(567.75to606.06)

North_Macedonia,Prevalence,30~35,753.22(732.27to774.77)

North_Macedonia,Prevalence,35~40,931.61(908.69to955.1)

North_Macedonia,Prevalence,40~45,1236.41(1210.46to1262.92)

North_Macedonia,Prevalence,45~50,2047.57(2012.15to2083.6)

North_Macedonia,Prevalence,50~55,3411.04(3361.73to3461.08)

North_Macedonia,Prevalence,55~60,5090.26(5025.17to5156.19)

North_Macedonia,Prevalence,60~65,7137.31(7052.68to7222.96)

North_Macedonia,Prevalence,65~70,10322.11(10205.92to10439.61)

North_Macedonia,Prevalence,70~75,14947.46(14780.64to15116.15)

North_Macedonia,Prevalence,75~80,20873.04(20599.58to21150.12)

North_Macedonia,Prevalence,80~85,27552.55(27146.18to27965.01)

North_Macedonia,Prevalence,85~90,34534.59(33837.53to35246.02)

North_Macedonia,Prevalence,90~95,40581.48(38645.93to42613.96)

North_Macedonia,Deaths,20~25,0.29(0.05to1.79)

North_Macedonia,Deaths,25~30,0.34(0.08to1.52)

North_Macedonia,Deaths,30~35,0.5(0.16to1.57)

North_Macedonia,Deaths,35~40,0.93(0.42to2.08)

North_Macedonia,Deaths,40~45,2.01(1.18to3.42)

North_Macedonia,Deaths,45~50,4.83(3.4to6.86)

North_Macedonia,Deaths,50~55,9.7(7.49to12.54)

North_Macedonia,Deaths,55~60,17.9(14.63to21.9)

North_Macedonia,Deaths,60~65,35.32(29.93to41.67)

North_Macedonia,Deaths,65~70,59.34(51.04to69)

North_Macedonia,Deaths,70~75,100.17(86.7to115.73)

North_Macedonia,Deaths,75~80,173.09(147.56to203.04)

North_Macedonia,Deaths,80~85,299.84(254.46to353.31)

North_Macedonia,Deaths,85~90,585.42(489.94to699.51)

North_Macedonia,Deaths,90~95,819.14(606to1107.24)

North_Macedonia,DALYs,20~25,43.39(37.53to50.17)

North_Macedonia,DALYs,25~30,53.83(47.67to60.8)

North_Macedonia,DALYs,30~35,69.85(63.1to77.32)

North_Macedonia,DALYs,35~40,96.72(88.96to105.17)

North_Macedonia,DALYs,40~45,155.56(145.82to165.95)

North_Macedonia,DALYs,45~50,302.99(288.63to318.06)

North_Macedonia,DALYs,50~55,528.72(508.55to549.69)

North_Macedonia,DALYs,55~60,834.11(806.98to862.16)

North_Macedonia,DALYs,60~65,1349.03(1310.09to1389.14)

North_Macedonia,DALYs,65~70,1912.11(1859.5to1966.21)

North_Macedonia,DALYs,70~75,2665.03(2592.22to2739.88)

North_Macedonia,DALYs,75~80,3673.91(3559.71to3791.77)

North_Macedonia,DALYs,80~85,4945.72(4778.46to5118.85)

North_Macedonia,DALYs,85~90,7432.29(7125.63to7752.14)

North_Macedonia,DALYs,90~95,8981.2(8187.22to9852.18)

Turkmenistan,Prevalence,20~25,606.74(578.2to636.7)

Turkmenistan,Prevalence,25~30,789.16(756.25to823.5)

Turkmenistan,Prevalence,30~35,966.23(929.31to1004.61)

Turkmenistan,Prevalence,35~40,1132.52(1091.81to1174.74)

Turkmenistan,Prevalence,40~45,1359.44(1314.33to1406.09)

Turkmenistan,Prevalence,45~50,1828.47(1772.07to1886.66)

Turkmenistan,Prevalence,50~55,2565.72(2492.84to2640.73)

Turkmenistan,Prevalence,55~60,3522.24(3428.07to3619)

Turkmenistan,Prevalence,60~65,4674.04(4552.38to4798.94)

Turkmenistan,Prevalence,65~70,6076.84(5918.06to6239.88)

Turkmenistan,Prevalence,70~75,7816.18(7600.96to8037.5)

Turkmenistan,Prevalence,75~80,9978.43(9666.19to10300.75)

Turkmenistan,Prevalence,80~85,12600.55(12169.3to13047.08)

Turkmenistan,Prevalence,85~90,15525.88(14903.02to16174.78)

Turkmenistan,Prevalence,90~95,18504.39(17551.43to19509.08)

Turkmenistan,Deaths,20~25,2.53(1.54to4.15)

Turkmenistan,Deaths,25~30,2.98(1.93to4.6)

Turkmenistan,Deaths,30~35,3.83(2.62to5.62)

Turkmenistan,Deaths,35~40,5.3(3.81to7.38)

Turkmenistan,Deaths,40~45,7.4(5.59to9.81)

Turkmenistan,Deaths,45~50,10(7.77to12.89)

Turkmenistan,Deaths,50~55,13.42(10.67to16.88)

Turkmenistan,Deaths,55~60,19.62(15.99to24.06)

Turkmenistan,Deaths,60~65,28.13(23.22to34.08)

Turkmenistan,Deaths,65~70,38.19(31.68to46.03)

Turkmenistan,Deaths,70~75,49.12(40.73to59.25)

Turkmenistan,Deaths,75~80,56.91(46.29to69.97)

Turkmenistan,Deaths,80~85,69.65(56.17to86.35)

Turkmenistan,Deaths,85~90,74.25(58.48to94.26)

Turkmenistan,Deaths,90~95,79.61(59.85to105.9)

Turkmenistan,DALYs,20~25,190.86(168.08to216.73)

Turkmenistan,DALYs,25~30,218.43(194.82to244.91)

Turkmenistan,DALYs,30~35,261.01(235.07to289.82)

Turkmenistan,DALYs,35~40,325.59(295.89to358.27)

Turkmenistan,DALYs,40~45,408.49(375.34to444.58)

Turkmenistan,DALYs,45~50,507.91(469.29to549.7)

Turkmenistan,DALYs,50~55,629.05(583.65to677.99)

Turkmenistan,DALYs,55~60,821.54(765.97to881.13)

Turkmenistan,DALYs,60~65,1030.7(962.63to1103.59)

Turkmenistan,DALYs,65~70,1195.14(1115.42to1280.56)

Turkmenistan,DALYs,70~75,1291.26(1200.83to1388.5)

Turkmenistan,DALYs,75~80,1249.91(1149.72to1358.83)

Turkmenistan,DALYs,80~85,1253.73(1142.2to1376.16)

Turkmenistan,DALYs,85~90,1130.19(1007.44to1267.89)

Turkmenistan,DALYs,90~95,1089.28(933.61to1270.9)

Trinidad_and_Tobago,Prevalence,20~25,264.95(249.54to281.3)

Trinidad_and_Tobago,Prevalence,25~30,352.17(334.45to370.84)

Trinidad_and_Tobago,Prevalence,30~35,464.43(443.9to485.9)

Trinidad_and_Tobago,Prevalence,35~40,607.12(583.21to632.02)

Trinidad_and_Tobago,Prevalence,40~45,823.08(794.67to852.5)

Trinidad_and_Tobago,Prevalence,45~50,1229.93(1192.75to1268.27)

Trinidad_and_Tobago,Prevalence,50~55,1865.7(1815.92to1916.83)

Trinidad_and_Tobago,Prevalence,55~60,2756.18(2689.42to2824.6)

Trinidad_and_Tobago,Prevalence,60~65,3901.66(3813.21to3992.17)

Trinidad_and_Tobago,Prevalence,65~70,5323.34(5206.42to5442.9)

Trinidad_and_Tobago,Prevalence,70~75,7220.82(7058.75to7386.61)

Trinidad_and_Tobago,Prevalence,75~80,9742.82(9481.61to10011.23)

Trinidad_and_Tobago,Prevalence,80~85,13031.39(12651.66to13422.51)

Trinidad_and_Tobago,Prevalence,85~90,17100.94(16531.35to17690.16)

Trinidad_and_Tobago,Prevalence,90~95,21949.67(21027.56to22912.2)

Trinidad_and_Tobago,Deaths,20~25,0.15(0.02to1.34)

Trinidad_and_Tobago,Deaths,25~30,0.22(0.04to1.32)

Trinidad_and_Tobago,Deaths,30~35,0.29(0.06to1.32)

Trinidad_and_Tobago,Deaths,35~40,0.58(0.19to1.84)

Trinidad_and_Tobago,Deaths,40~45,1.08(0.46to2.56)

Trinidad_and_Tobago,Deaths,45~50,2.53(1.37to4.67)

Trinidad_and_Tobago,Deaths,50~55,5.7(3.61to8.99)

Trinidad_and_Tobago,Deaths,55~60,11.59(8.09to16.6)

Trinidad_and_Tobago,Deaths,60~65,24.74(18.48to33.1)

Trinidad_and_Tobago,Deaths,65~70,40.44(31.08to52.61)

Trinidad_and_Tobago,Deaths,70~75,69.02(53.57to88.92)

Trinidad_and_Tobago,Deaths,75~80,104.44(77.64to140.49)

Trinidad_and_Tobago,Deaths,80~85,150.57(110.78to204.66)

Trinidad_and_Tobago,Deaths,85~90,222.46(160.54to308.28)

Trinidad_and_Tobago,Deaths,90~95,275.56(188.93to401.9)

Trinidad_and_Tobago,DALYs,20~25,21.2(17.39to25.85)

Trinidad_and_Tobago,DALYs,25~30,28.15(23.77to33.34)

Trinidad_and_Tobago,DALYs,30~35,35.3(30.44to40.92)

Trinidad_and_Tobago,DALYs,35~40,55.05(48.62to62.32)

Trinidad_and_Tobago,DALYs,40~45,84.02(75.82to93.1)

Trinidad_and_Tobago,DALYs,45~50,155.9(143.64to169.21)

Trinidad_and_Tobago,DALYs,50~55,288.15(269.49to308.11)

Trinidad_and_Tobago,DALYs,55~60,490.45(463.07to519.45)

Trinidad_and_Tobago,DALYs,60~65,858.49(816.44to902.71)

Trinidad_and_Tobago,DALYs,65~70,1179.11(1123.95to1236.97)

Trinidad_and_Tobago,DALYs,70~75,1645.02(1567.4to1726.48)

Trinidad_and_Tobago,DALYs,75~80,2018.22(1904.1to2139.18)

Trinidad_and_Tobago,DALYs,80~85,2334.95(2190.71to2488.68)

Trinidad_and_Tobago,DALYs,85~90,2778.22(2580.62to2990.96)

Trinidad_and_Tobago,DALYs,90~95,3067.03(2786.93to3375.27)

Bolivia_(Plurinational_State_of),Prevalence,20~25,371.79(363.68to380.07)

Bolivia_(Plurinational_State_of),Prevalence,25~30,469.44(460.26to478.81)

Bolivia_(Plurinational_State_of),Prevalence,30~35,590.56(580.03to601.27)

Bolivia_(Plurinational_State_of),Prevalence,35~40,741.08(729.02to753.34)

Bolivia_(Plurinational_State_of),Prevalence,40~45,973.99(959.84to988.33)

Bolivia_(Plurinational_State_of),Prevalence,45~50,1466.56(1447.75to1485.61)

Bolivia_(Plurinational_State_of),Prevalence,50~55,2322.1(2295.89to2348.61)

Bolivia_(Plurinational_State_of),Prevalence,55~60,3556.18(3520.22to3592.51)

Bolivia_(Plurinational_State_of),Prevalence,60~65,5185.33(5136.83to5234.29)

Bolivia_(Plurinational_State_of),Prevalence,65~70,7426.03(7359.63to7493.04)

Bolivia_(Plurinational_State_of),Prevalence,70~75,10608.59(10513.7to10704.32)

Bolivia_(Plurinational_State_of),Prevalence,75~80,14994.5(14836.48to15154.2)

Bolivia_(Plurinational_State_of),Prevalence,80~85,20563.53(20324.92to20804.94)

Bolivia_(Plurinational_State_of),Prevalence,85~90,26699.08(26327.54to27075.85)

Bolivia_(Plurinational_State_of),Prevalence,90~95,32495.69(31843.18to33161.58)

Bolivia_(Plurinational_State_of),Deaths,20~25,1.46(0.91to2.33)

Bolivia_(Plurinational_State_of),Deaths,25~30,1.36(0.87to2.11)

Bolivia_(Plurinational_State_of),Deaths,30~35,1.31(0.86to1.99)

Bolivia_(Plurinational_State_of),Deaths,35~40,1.74(1.22to2.49)

Bolivia_(Plurinational_State_of),Deaths,40~45,2.74(2.06to3.65)

Bolivia_(Plurinational_State_of),Deaths,45~50,4.46(3.53to5.62)

Bolivia_(Plurinational_State_of),Deaths,50~55,7.99(6.64to9.6)

Bolivia_(Plurinational_State_of),Deaths,55~60,12.88(11.04to15.01)

Bolivia_(Plurinational_State_of),Deaths,60~65,30.83(27.44to34.64)

Bolivia_(Plurinational_State_of),Deaths,65~70,57.1(51.56to63.23)

Bolivia_(Plurinational_State_of),Deaths,70~75,120.52(109.75to132.34)

Bolivia_(Plurinational_State_of),Deaths,75~80,216.75(195.03to240.89)

Bolivia_(Plurinational_State_of),Deaths,80~85,411.36(369.48to457.99)

Bolivia_(Plurinational_State_of),Deaths,85~90,682.79(609.75to764.59)

Bolivia_(Plurinational_State_of),Deaths,90~95,1010.73(887.9to1150.56)

Bolivia_(Plurinational_State_of),DALYs,20~25,107.64(102.03to113.57)

Bolivia_(Plurinational_State_of),DALYs,25~30,103.13(98.05to108.47)

Bolivia_(Plurinational_State_of),DALYs,30~35,102.13(97.33to107.15)

Bolivia_(Plurinational_State_of),DALYs,35~40,124.35(119.1to129.83)

Bolivia_(Plurinational_State_of),DALYs,40~45,171.44(165.24to177.88)

Bolivia_(Plurinational_State_of),DALYs,45~50,251.26(243.38to259.39)

Bolivia_(Plurinational_State_of),DALYs,50~55,396.75(386.13to407.67)

Bolivia_(Plurinational_State_of),DALYs,55~60,568.18(554.63to582.05)

Bolivia_(Plurinational_State_of),DALYs,60~65,1084.72(1063.05to1106.82)

Bolivia_(Plurinational_State_of),DALYs,65~70,1663.66(1632.74to1695.17)

Bolivia_(Plurinational_State_of),DALYs,70~75,2811.7(2761.67to2862.63)

Bolivia_(Plurinational_State_of),DALYs,75~80,4037.72(3955.27to4121.89)

Bolivia_(Plurinational_State_of),DALYs,80~85,5956.31(5827.96to6087.48)

Bolivia_(Plurinational_State_of),DALYs,85~90,7830.4(7639.18to8026.41)

Bolivia_(Plurinational_State_of),DALYs,90~95,9993.87(9673.04to10325.33)

Grenada,Prevalence,20~25,222.6(177.39to279.33)

Grenada,Prevalence,25~30,297.12(243.18to363.01)

Grenada,Prevalence,30~35,396.89(330.24to476.99)

Grenada,Prevalence,35~40,531.43(450.19to627.32)

Grenada,Prevalence,40~45,744.03(643.86to859.79)

Grenada,Prevalence,45~50,1156.24(1023.05to1306.76)

Grenada,Prevalence,50~55,1844.55(1665.61to2042.72)

Grenada,Prevalence,55~60,2862.4(2613.44to3135.07)

Grenada,Prevalence,60~65,4220.09(3865.4to4607.33)

Grenada,Prevalence,65~70,5929.5(5432.86to6471.53)

Grenada,Prevalence,70~75,8096.68(7406.55to8851.12)

Grenada,Prevalence,75~80,10757.2(9677.44to11957.45)

Grenada,Prevalence,80~85,13872.34(12302.36to15642.68)

Grenada,Prevalence,85~90,17731.65(15393.16to20425.4)

Grenada,Prevalence,90~95,22780.67(19128.12to27130.69)

Grenada,Deaths,20~25,0.12(0to263.8)

Grenada,Deaths,25~30,0.17(0to125.68)

Grenada,Deaths,30~35,0.27(0to74.33)

Grenada,Deaths,35~40,0.56(0.01to37.17)

Grenada,Deaths,40~45,1.08(0.05to24.82)

Grenada,Deaths,45~50,2.1(0.19to22.88)

Grenada,Deaths,50~55,4.86(0.9to26.18)

Grenada,Deaths,55~60,10.87(2.91to40.59)

Grenada,Deaths,60~65,25.81(8.84to75.32)

Grenada,Deaths,65~70,50.83(19.08to135.38)

Grenada,Deaths,70~75,103.33(40.58to263.15)

Grenada,Deaths,75~80,184.08(64.28to527.21)

Grenada,Deaths,80~85,226.56(72.97to703.41)

Grenada,Deaths,85~90,283.32(80.81to993.31)

Grenada,Deaths,90~95,323.48(74.26to1409.11)

Grenada,DALYs,20~25,16.89(8.2to34.83)

Grenada,DALYs,25~30,22.43(11.86to42.41)

Grenada,DALYs,30~35,30.61(17.25to54.32)

Grenada,DALYs,35~40,50.23(31.11to81.09)

Grenada,DALYs,40~45,80.86(54.62to119.69)

Grenada,DALYs,45~50,135.85(98.52to187.32)

Grenada,DALYs,50~55,258.64(201.69to331.67)

Grenada,DALYs,55~60,475.64(385.28to587.18)

Grenada,DALYs,60~65,903.67(750.22to1088.51)

Grenada,DALYs,65~70,1443.75(1205.91to1728.48)

Grenada,DALYs,70~75,2345.34(1960.9to2805.16)

Grenada,DALYs,75~80,3346.02(2723.89to4110.25)

Grenada,DALYs,80~85,3372.24(2656.83to4280.3)

Grenada,DALYs,85~90,3498.63(2608.97to4691.67)

Grenada,DALYs,90~95,3652.54(2493.11to5351.16)

Botswana,Prevalence,20~25,423.83(405.47to443.02)

Botswana,Prevalence,25~30,569.55(547.6to592.37)

Botswana,Prevalence,30~35,734.63(708.61to761.6)

Botswana,Prevalence,35~40,910.5(880.24to941.8)

Botswana,Prevalence,40~45,1192.97(1156.6to1230.49)

Botswana,Prevalence,45~50,1906.43(1854.29to1960.03)

Botswana,Prevalence,50~55,3084.05(3007.79to3162.24)

Botswana,Prevalence,55~60,4473.74(4370.79to4579.12)

Botswana,Prevalence,60~65,5986.5(5853.34to6122.69)

Botswana,Prevalence,65~70,7895.87(7722.06to8073.6)

Botswana,Prevalence,70~75,10153.76(9917.91to10395.22)

Botswana,Prevalence,75~80,12623.72(12268.01to12989.74)

Botswana,Prevalence,80~85,15622.94(15112.27to16150.86)

Botswana,Prevalence,85~90,18809.5(18008.2to19646.46)

Botswana,Prevalence,90~95,22174.41(20673.86to23783.88)

Botswana,Deaths,20~25,0.86(0.27to2.79)

Botswana,Deaths,25~30,1.23(0.48to3.15)

Botswana,Deaths,30~35,1.84(0.85to3.96)

Botswana,Deaths,35~40,3.32(1.83to6.01)

Botswana,Deaths,40~45,7.08(4.61to10.88)

Botswana,Deaths,45~50,14.54(10.54to20.04)

Botswana,Deaths,50~55,27.95(21.74to35.93)

Botswana,Deaths,55~60,52.93(43.15to64.93)

Botswana,Deaths,60~65,80.56(67.08to96.75)

Botswana,Deaths,65~70,145.74(123.42to172.1)

Botswana,Deaths,70~75,276.54(236.11to323.88)

Botswana,Deaths,75~80,359.63(299.94to431.21)

Botswana,Deaths,80~85,450.9(370.72to548.43)

Botswana,Deaths,85~90,528.56(419.32to666.24)

Botswana,Deaths,90~95,602.47(432.73to838.79)

Botswana,DALYs,20~25,114.21(103.83to125.62)

Botswana,DALYs,25~30,151.56(139.63to164.5)

Botswana,DALYs,30~35,199.62(185.61to214.69)

Botswana,DALYs,35~40,281.17(263.74to299.75)

Botswana,DALYs,40~45,460.73(436.92to485.83)

Botswana,DALYs,45~50,812.73(777.87to849.16)

Botswana,DALYs,50~55,1372.2(1322.01to1424.29)

Botswana,DALYs,55~60,2214.52(2143to2288.44)

Botswana,DALYs,60~65,2921.15(2831.6to3013.52)

Botswana,DALYs,65~70,4341.61(4214.25to4472.81)

Botswana,DALYs,70~75,6612.29(6419.22to6811.16)

Botswana,DALYs,75~80,7022.95(6777.93to7276.83)

Botswana,DALYs,80~85,7083.01(6793.85to7384.47)

Botswana,DALYs,85~90,6804.91(6431.21to7200.32)

Botswana,DALYs,90~95,6850.8(6237.05to7524.95)

Namibia,Prevalence,20~25,408.68(391.15to426.99)

Namibia,Prevalence,25~30,549.47(528.31to571.47)

Namibia,Prevalence,30~35,711.76(686.44to738.02)

Namibia,Prevalence,35~40,887.95(858.17to918.76)

Namibia,Prevalence,40~45,1175(1139.07to1212.06)

Namibia,Prevalence,45~50,1909.56(1857.8to1962.77)

Namibia,Prevalence,50~55,3133.39(3057.61to3211.06)

Namibia,Prevalence,55~60,4577.9(4474.83to4683.35)

Namibia,Prevalence,60~65,6139.24(6006.54to6274.86)

Namibia,Prevalence,65~70,8111.26(7938.58to8287.71)

Namibia,Prevalence,70~75,10480.21(10247.33to10718.39)

Namibia,Prevalence,75~80,13123.72(12771.77to13485.37)

Namibia,Prevalence,80~85,16288.17(15775.06to16817.97)

Namibia,Prevalence,85~90,19583.25(18752.66to20450.62)

Namibia,Prevalence,90~95,22896.48(21255.27to24664.43)

Namibia,Deaths,20~25,0.56(0.19to1.63)

Namibia,Deaths,25~30,0.86(0.36to2.05)

Namibia,Deaths,30~35,1.43(0.71to2.91)

Namibia,Deaths,35~40,2.84(1.65to4.88)

Namibia,Deaths,40~45,7.05(4.81to10.35)

Namibia,Deaths,45~50,16.2(12.21to21.49)

Namibia,Deaths,50~55,34.15(27.52to42.38)

Namibia,Deaths,55~60,68.53(57.53to81.63)

Namibia,Deaths,60~65,108.73(92.99to127.13)

Namibia,Deaths,65~70,210.49(183.17to241.89)

Namibia,Deaths,70~75,408.05(357.57to465.64)

Namibia,Deaths,75~80,554.09(475.29to645.96)

Namibia,Deaths,80~85,735.62(622.51to869.29)

Namibia,Deaths,85~90,938.44(767.7to1147.16)

Namibia,Deaths,90~95,1112.3(819.32to1510.05)

Namibia,DALYs,20~25,84.86(77.51to92.91)

Namibia,DALYs,25~30,116.44(107.63to125.97)

Namibia,DALYs,30~35,162.52(151.59to174.24)

Namibia,DALYs,35~40,245.74(231.26to261.12)

Namibia,DALYs,40~45,458.18(436.24to481.23)

Namibia,DALYs,45~50,887.54(853.01to923.46)

Namibia,DALYs,50~55,1617.81(1565.54to1671.83)

Namibia,DALYs,55~60,2755.88(2677.76to2836.28)

Namibia,DALYs,60~65,3760.53(3659.56to3864.3)

Namibia,DALYs,65~70,5954.81(5804.5to6109.02)

Namibia,DALYs,70~75,9280.04(9047.84to9518.2)

Namibia,DALYs,75~80,10219.07(9910.8to10536.93)

Namibia,DALYs,80~85,10811.45(10425.29to11211.91)

Namibia,DALYs,85~90,11137.79(10589.65to11714.29)

Namibia,DALYs,90~95,11558.13(10566.2to12643.17)

Mauritius,Prevalence,20~25,376.25(356.34to397.28)

Mauritius,Prevalence,25~30,521.72(498.41to546.11)

Mauritius,Prevalence,30~35,685.97(659.15to713.88)

Mauritius,Prevalence,35~40,858.13(827.93to889.44)

Mauritius,Prevalence,40~45,1097.58(1063.61to1132.64)

Mauritius,Prevalence,45~50,1576.4(1532.98to1621.04)

Mauritius,Prevalence,50~55,2286.16(2229.53to2344.24)

Mauritius,Prevalence,55~60,3190.95(3117.88to3265.73)

Mauritius,Prevalence,60~65,4334.7(4240.37to4431.14)

Mauritius,Prevalence,65~70,5797.37(5673.06to5924.4)

Mauritius,Prevalence,70~75,7854.71(7681.91to8031.41)

Mauritius,Prevalence,75~80,10716.27(10435.35to11004.75)

Mauritius,Prevalence,80~85,14746.74(14326.73to15179.07)

Mauritius,Prevalence,85~90,19846.17(19208.56to20504.94)

Mauritius,Prevalence,90~95,25675.04(24638.81to26754.84)

Mauritius,Deaths,20~25,0.58(0.09to3.87)

Mauritius,Deaths,25~30,0.77(0.17to3.42)

Mauritius,Deaths,30~35,1.13(0.36to3.55)

Mauritius,Deaths,35~40,1.82(0.77to4.3)

Mauritius,Deaths,40~45,2.74(1.4to5.35)

Mauritius,Deaths,45~50,4.35(2.56to7.38)

Mauritius,Deaths,50~55,7.89(5.24to11.86)

Mauritius,Deaths,55~60,12.43(8.84to17.47)

Mauritius,Deaths,60~65,27.82(21.25to36.42)

Mauritius,Deaths,65~70,44.8(34.97to57.39)

Mauritius,Deaths,70~75,76.18(60.13to96.51)

Mauritius,Deaths,75~80,114.45(87.73to149.32)

Mauritius,Deaths,80~85,202.02(154.6to263.98)

Mauritius,Deaths,85~90,264.25(199.28to350.41)

Mauritius,Deaths,90~95,357.87(261.36to490.03)

Mauritius,DALYs,20~25,68.72(57.45to82.21)

Mauritius,DALYs,25~30,91.02(78.48to105.57)

Mauritius,DALYs,30~35,120.33(106.2to136.34)

Mauritius,DALYs,35~40,161.53(145.26to179.63)

Mauritius,DALYs,40~45,213.42(195.1to233.46)

Mauritius,DALYs,45~50,305.73(283.1to330.16)

Mauritius,DALYs,50~55,471.98(441.98to504.02)

Mauritius,DALYs,55~60,654.71(617.1to694.62)

Mauritius,DALYs,60~65,1130.75(1073.63to1190.9)

Mauritius,DALYs,65~70,1530.69(1455.6to1609.66)

Mauritius,DALYs,70~75,2122.36(2018.13to2231.97)

Mauritius,DALYs,75~80,2604.72(2456.53to2761.85)

Mauritius,DALYs,80~85,3573.71(3359.4to3801.7)

Mauritius,DALYs,85~90,3842.64(3579.64to4124.95)

Mauritius,DALYs,90~95,4562.5(4175.59to4985.25)

Poland,Prevalence,20~25,461.32(443.39to479.97)

Poland,Prevalence,25~30,618.03(597.75to639)

Poland,Prevalence,30~35,788.9(766.76to811.67)

Poland,Prevalence,35~40,968.67(945.14to992.79)

Poland,Prevalence,40~45,1262.67(1236.82to1289.07)

Poland,Prevalence,45~50,2021.55(1987.27to2056.42)

Poland,Prevalence,50~55,3306.57(3259.71to3354.09)

Poland,Prevalence,55~60,4870.98(4810.36to4932.36)

Poland,Prevalence,60~65,6704.09(6627.22to6781.86)

Poland,Prevalence,65~70,9314.47(9211.8to9418.27)

Poland,Prevalence,70~75,12679.2(12538.86to12821.11)

Poland,Prevalence,75~80,16541.15(16328.56to16756.5)

Poland,Prevalence,80~85,20729.92(20447.32to21016.42)

Poland,Prevalence,85~90,25264.29(24873.2to25661.53)

Poland,Prevalence,90~95,29809.45(29191.4to30440.58)

Poland,Deaths,20~25,0.23(0.13to0.42)

Poland,Deaths,25~30,0.23(0.14to0.38)

Poland,Deaths,30~35,0.37(0.26to0.53)

Poland,Deaths,35~40,0.68(0.53to0.86)

Poland,Deaths,40~45,1.54(1.33to1.78)

Poland,Deaths,45~50,3.94(3.6to4.32)

Poland,Deaths,50~55,8.59(8.06to9.15)

Poland,Deaths,55~60,16.75(15.98to17.57)

Poland,Deaths,60~65,33.56(32.29to34.87)

Poland,Deaths,65~70,53.59(51.74to55.5)

Poland,Deaths,70~75,80.46(77.77to83.24)

Poland,Deaths,75~80,109.4(105.34to113.62)

Poland,Deaths,80~85,131.33(126.3to136.56)

Poland,Deaths,85~90,161.53(154.91to168.45)

Poland,Deaths,90~95,181.58(172.45to191.18)

Poland,DALYs,20~25,44.51(39.75to49.84)

Poland,DALYs,25~30,54.89(49.88to60.4)

Poland,DALYs,30~35,70.54(65.2to76.33)

Poland,DALYs,35~40,91.8(86.07to97.9)

Poland,DALYs,40~45,140.67(133.9to147.79)

Poland,DALYs,45~50,271.5(261.7to281.66)

Poland,DALYs,50~55,491.75(477.84to506.06)

Poland,DALYs,55~60,797.63(778.98to816.72)

Poland,DALYs,60~65,1294.71(1268.16to1321.82)

Poland,DALYs,65~70,1754.21(1719.83to1789.27)

Poland,DALYs,70~75,2207.04(2163.56to2251.4)

Poland,DALYs,75~80,2483.99(2427.53to2541.76)

Poland,DALYs,80~85,2485.52(2424.24to2548.34)

Poland,DALYs,85~90,2551.68(2478.36to2627.17)

Poland,DALYs,90~95,2601.74(2497.17to2710.69)

Rwanda,Prevalence,20~25,494.32(484.44to504.4)

Rwanda,Prevalence,25~30,662.46(650.47to674.66)

Rwanda,Prevalence,30~35,841(826.91to855.33)

Rwanda,Prevalence,35~40,1018.72(1002.59to1035.12)

Rwanda,Prevalence,40~45,1275.72(1256.93to1294.8)

Rwanda,Prevalence,45~50,1857.87(1832.4to1883.69)

Rwanda,Prevalence,50~55,2813.5(2778.25to2849.2)

Rwanda,Prevalence,55~60,4012.41(3965.14to4060.24)

Rwanda,Prevalence,60~65,5318.32(5257.42to5379.92)

Rwanda,Prevalence,65~70,6806.88(6728.56to6886.12)

Rwanda,Prevalence,70~75,8657.85(8551.26to8765.77)

Rwanda,Prevalence,75~80,10966(10799.75to11134.82)

Rwanda,Prevalence,80~85,13502.23(13258.95to13749.97)

Rwanda,Prevalence,85~90,15432.36(15049.59to15824.86)

Rwanda,Prevalence,90~95,16323.65(15619.37to17059.69)

Rwanda,Deaths,20~25,3.2(2.22to4.6)

Rwanda,Deaths,25~30,3.29(2.36to4.58)

Rwanda,Deaths,30~35,4.63(3.53to6.08)

Rwanda,Deaths,35~40,6.47(5.16to8.13)

Rwanda,Deaths,40~45,8.14(6.66to9.93)

Rwanda,Deaths,45~50,16.41(14.11to19.09)

Rwanda,Deaths,50~55,25.72(22.65to29.22)

Rwanda,Deaths,55~60,49.1(44.23to54.5)

Rwanda,Deaths,60~65,81.97(74.76to89.88)

Rwanda,Deaths,65~70,123.49(113.37to134.52)

Rwanda,Deaths,70~75,192.82(177.52to209.44)

Rwanda,Deaths,75~80,243.63(221.25to268.28)

Rwanda,Deaths,80~85,340.24(306.95to377.14)

Rwanda,Deaths,85~90,389.07(343.57to440.59)

Rwanda,Deaths,90~95,465.41(389.09to556.7)

Rwanda,DALYs,20~25,260.46(250.53to270.77)

Rwanda,DALYs,25~30,287.74(277.68to298.15)

Rwanda,DALYs,30~35,362.53(351.24to374.18)

Rwanda,DALYs,35~40,448.37(435.73to461.37)

Rwanda,DALYs,40~45,526.94(513.36to540.89)

Rwanda,DALYs,45~50,886.19(866.88to905.93)

Rwanda,DALYs,50~55,1259(1234.24to1284.27)

Rwanda,DALYs,55~60,2026.26(1990.81to2062.35)

Rwanda,DALYs,60~65,2872.44(2825.51to2920.16)

Rwanda,DALYs,65~70,3651.96(3593.85to3711.01)

Rwanda,DALYs,70~75,4682.34(4606.44to4759.5)

Rwanda,DALYs,75~80,4845.75(4749.94to4943.5)

Rwanda,DALYs,80~85,5351.36(5228.13to5477.5)

Rwanda,DALYs,85~90,4969.82(4810.07to5134.88)

Rwanda,DALYs,90~95,5124.05(4849.62to5414.01)

Haiti,Prevalence,20~25,368.46(360.71to376.38)

Haiti,Prevalence,25~30,485.56(476.45to494.85)

Haiti,Prevalence,30~35,630.75(619.97to641.71)

Haiti,Prevalence,35~40,799.57(786.98to812.36)

Haiti,Prevalence,40~45,1063.37(1048.28to1078.69)

Haiti,Prevalence,45~50,1689.77(1668.45to1711.36)

Haiti,Prevalence,50~55,2770.48(2739.47to2801.84)

Haiti,Prevalence,55~60,4154.84(4112.06to4198.07)

Haiti,Prevalence,60~65,5709.27(5652.61to5766.5)

Haiti,Prevalence,65~70,7605.1(7530.02to7680.94)

Haiti,Prevalence,70~75,10002.97(9898.74to10108.29)

Haiti,Prevalence,75~80,12997.9(12835.58to13162.28)

Haiti,Prevalence,80~85,16556(16315.94to16799.6)

Haiti,Prevalence,85~90,20431.62(20043.88to20826.86)

Haiti,Prevalence,90~95,24197.84(23450.92to24968.55)

Haiti,Deaths,20~25,0.52(0.32to0.84)

Haiti,Deaths,25~30,0.76(0.51to1.14)

Haiti,Deaths,30~35,0.98(0.69to1.41)

Haiti,Deaths,35~40,1.91(1.44to2.52)

Haiti,Deaths,40~45,3.84(3.09to4.75)

Haiti,Deaths,45~50,7.86(6.65to9.29)

Haiti,Deaths,50~55,18.36(16.19to20.82)

Haiti,Deaths,55~60,37.82(34.19to41.84)

Haiti,Deaths,60~65,80.27(73.78to87.33)

Haiti,Deaths,65~70,138.27(127.9to149.49)

Haiti,Deaths,70~75,237.33(219.78to256.28)

Haiti,Deaths,75~80,359.21(328.6to392.66)

Haiti,Deaths,80~85,519.3(471.72to571.67)

Haiti,Deaths,85~90,742.56(664.03to830.38)

Haiti,Deaths,90~95,966.73(825.17to1132.57)

Haiti,DALYs,20~25,50.85(48.25to53.59)

Haiti,DALYs,25~30,68.07(65.03to71.24)

Haiti,DALYs,30~35,83.03(79.63to86.57)

Haiti,DALYs,35~40,133.48(128.85to138.27)

Haiti,DALYs,40~45,226.34(219.81to233.07)

Haiti,DALYs,45~50,405.1(395.32to415.13)

Haiti,DALYs,50~55,809.98(794.02to826.26)

Haiti,DALYs,55~60,1427.21(1402.96to1451.88)

Haiti,DALYs,60~65,2535.99(2497.52to2575.05)

Haiti,DALYs,65~70,3650.93(3597.2to3705.46)

Haiti,DALYs,70~75,5127.86(5050.16to5206.75)

Haiti,DALYs,75~80,6235.56(6122.01to6351.21)

Haiti,DALYs,80~85,7106.86(6955.42to7261.59)

Haiti,DALYs,85~90,8100.37(7872.44to8334.9)

Haiti,DALYs,90~95,9138.89(8718.06to9580.04)

Serbia,Prevalence,20~25,409.66(401.14to418.35)

Serbia,Prevalence,25~30,556.5(546.76to566.4)

Serbia,Prevalence,30~35,723.77(712.88to734.83)

Serbia,Prevalence,35~40,904.51(892.64to916.53)

Serbia,Prevalence,40~45,1219.6(1206.04to1233.31)

Serbia,Prevalence,45~50,2088.74(2069.85to2107.81)

Serbia,Prevalence,50~55,3565.38(3538.98to3591.98)

Serbia,Prevalence,55~60,5334.19(5299.87to5368.72)

Serbia,Prevalence,60~65,7444.81(7400.73to7489.16)

Serbia,Prevalence,65~70,10645.58(10585.2to10706.3)

Serbia,Prevalence,70~75,15241.74(15155.66to15328.31)

Serbia,Prevalence,75~80,21139.01(21003.58to21275.31)

Serbia,Prevalence,80~85,27786.38(27591.73to27982.4)

Serbia,Prevalence,85~90,34678.37(34382.09to34977.2)

Serbia,Prevalence,90~95,41338.37(40846.65to41836.01)

Serbia,Deaths,20~25,0.31(0.13to0.72)

Serbia,Deaths,25~30,0.39(0.2to0.78)

Serbia,Deaths,30~35,0.62(0.38to1.04)

Serbia,Deaths,35~40,1.16(0.82to1.65)

Serbia,Deaths,40~45,2.61(2.08to3.26)

Serbia,Deaths,45~50,6.41(5.53to7.43)

Serbia,Deaths,50~55,13.32(11.97to14.81)

Serbia,Deaths,55~60,25.53(23.57to27.65)

Serbia,Deaths,60~65,50.27(47.1to53.65)

Serbia,Deaths,65~70,80.94(76.21to85.97)

Serbia,Deaths,70~75,128.67(121.38to136.39)

Serbia,Deaths,75~80,214.49(201.26to228.59)

Serbia,Deaths,80~85,341.71(320.12to364.75)

Serbia,Deaths,85~90,384.91(357.57to414.34)

Serbia,Deaths,90~95,389.15(353.26to428.7)

Serbia,DALYs,20~25,45.12(35.81to56.86)

Serbia,DALYs,25~30,57.3(47.21to69.55)

Serbia,DALYs,30~35,77.26(65.9to90.58)

Serbia,DALYs,35~40,109.17(96.1to124.01)

Serbia,DALYs,40~45,184.14(167.33to202.63)

Serbia,DALYs,45~50,373.72(348.25to401.06)

Serbia,DALYs,50~55,673.09(636.7to711.57)

Serbia,DALYs,55~60,1096.25(1047.51to1147.26)

Serbia,DALYs,60~65,1785.08(1715.27to1857.74)

Serbia,DALYs,65~70,2439.11(2347.19to2534.63)

Serbia,DALYs,70~75,3218.17(3096.64to3344.47)

Serbia,DALYs,75~80,4288.03(4106.75to4477.32)

Serbia,DALYs,80~85,5407.71(5161.3to5665.89)

Serbia,DALYs,85~90,5133.13(4838.55to5445.65)

Serbia,DALYs,90~95,4741.6(4333.88to5187.67)

Bosnia_and_Herzegovina,Prevalence,20~25,426.05(413.62to438.85)

Bosnia_and_Herzegovina,Prevalence,25~30,572.89(558.97to587.15)

Bosnia_and_Herzegovina,Prevalence,30~35,738.47(723.07to754.19)

Bosnia_and_Herzegovina,Prevalence,35~40,917.18(900.54to934.13)

Bosnia_and_Herzegovina,Prevalence,40~45,1221.88(1203.2to1240.85)

Bosnia_and_Herzegovina,Prevalence,45~50,2022.73(1997.08to2048.71)

Bosnia_and_Herzegovina,Prevalence,50~55,3374.57(3339.15to3410.37)

Bosnia_and_Herzegovina,Prevalence,55~60,5048.24(5001.43to5095.49)

Bosnia_and_Herzegovina,Prevalence,60~65,7075.22(7013.98to7136.99)

Bosnia_and_Herzegovina,Prevalence,65~70,10186.46(10102.06to10271.57)

Bosnia_and_Herzegovina,Prevalence,70~75,14559.51(14438.87to14681.16)

Bosnia_and_Herzegovina,Prevalence,75~80,19915.13(19726.01to20106.07)

Bosnia_and_Herzegovina,Prevalence,80~85,25755.49(25494.42to26019.24)

Bosnia_and_Herzegovina,Prevalence,85~90,31811.19(31429.21to32197.81)

Bosnia_and_Herzegovina,Prevalence,90~95,37690.1(37055.07to38336.03)

Bosnia_and_Herzegovina,Deaths,20~25,0.66(0.23to1.89)

Bosnia_and_Herzegovina,Deaths,25~30,0.63(0.26to1.56)

Bosnia_and_Herzegovina,Deaths,30~35,0.83(0.41to1.68)

Bosnia_and_Herzegovina,Deaths,35~40,1.32(0.79to2.21)

Bosnia_and_Herzegovina,Deaths,40~45,2.57(1.81to3.64)

Bosnia_and_Herzegovina,Deaths,45~50,5.58(4.38to7.11)

Bosnia_and_Herzegovina,Deaths,50~55,10.09(8.42to12.09)

Bosnia_and_Herzegovina,Deaths,55~60,17.99(15.61to20.73)

Bosnia_and_Herzegovina,Deaths,60~65,36.19(32.18to40.71)

Bosnia_and_Herzegovina,Deaths,65~70,58.16(52.16to64.85)

Bosnia_and_Herzegovina,Deaths,70~75,98.65(88.86to109.52)

Bosnia_and_Herzegovina,Deaths,75~80,153.21(136.69to171.72)

Bosnia_and_Herzegovina,Deaths,80~85,183.75(163.3to206.76)

Bosnia_and_Herzegovina,Deaths,85~90,215.55(189.43to245.28)

Bosnia_and_Herzegovina,Deaths,90~95,238.54(202.66to280.77)

Bosnia_and_Herzegovina,DALYs,20~25,65.25(55.91to76.15)

Bosnia_and_Herzegovina,DALYs,25~30,72.43(63.5to82.63)

Bosnia_and_Herzegovina,DALYs,30~35,89.05(79.78to99.4)

Bosnia_and_Herzegovina,DALYs,35~40,117.3(107.21to128.33)

Bosnia_and_Herzegovina,DALYs,40~45,181.81(169.69to194.8)

Bosnia_and_Herzegovina,DALYs,45~50,335.43(318.05to353.77)

Bosnia_and_Herzegovina,DALYs,50~55,544.08(521.11to568.07)

Bosnia_and_Herzegovina,DALYs,55~60,835.79(805.62to867.1)

Bosnia_and_Herzegovina,DALYs,60~65,1376.92(1332.59to1422.73)

Bosnia_and_Herzegovina,DALYs,65~70,1875.61(1817.57to1935.51)

Bosnia_and_Herzegovina,DALYs,70~75,2612.84(2532.44to2695.79)

Bosnia_and_Herzegovina,DALYs,75~80,3283.92(3170.61to3401.29)

Bosnia_and_Herzegovina,DALYs,80~85,3276.94(3154.22to3404.45)

Bosnia_and_Herzegovina,DALYs,85~90,3211.38(3065.88to3363.78)

Bosnia_and_Herzegovina,DALYs,90~95,3187.65(2978.11to3411.94)

Peru,Prevalence,20~25,287.61(283.8to291.46)

Peru,Prevalence,25~30,371.6(367.23to376.02)

Peru,Prevalence,30~35,479.38(474.29to484.54)

Peru,Prevalence,35~40,620.57(614.57to626.62)

Peru,Prevalence,40~45,836.02(828.85to843.26)

Peru,Prevalence,45~50,1242.46(1233to1251.99)

Peru,Prevalence,50~55,1911.22(1898.36to1924.17)

Peru,Prevalence,55~60,2887.55(2869.97to2905.25)

Peru,Prevalence,60~65,4223.42(4199.42to4247.57)

Peru,Prevalence,65~70,6032.62(5999.73to6065.69)

Peru,Prevalence,70~75,8641.61(8594.47to8689)

Peru,Prevalence,75~80,12364.69(12286.46to12443.41)

Peru,Prevalence,80~85,17451.47(17334.58to17569.15)

Peru,Prevalence,85~90,23919.68(23743.77to24096.9)

Peru,Prevalence,90~95,31404.36(31126.57to31684.64)

Peru,Deaths,20~25,0.65(0.44to0.95)

Peru,Deaths,25~30,0.62(0.43to0.9)

Peru,Deaths,30~35,0.6(0.42to0.84)

Peru,Deaths,35~40,0.76(0.57to1.03)

Peru,Deaths,40~45,1.16(0.91to1.48)

Peru,Deaths,45~50,1.73(1.41to2.12)

Peru,Deaths,50~55,2.85(2.42to3.37)

Peru,Deaths,55~60,4.25(3.67to4.91)

Peru,Deaths,60~65,11.04(9.9to12.31)

Peru,Deaths,65~70,19.05(17.28to20.99)

Peru,Deaths,70~75,40.21(36.88to43.85)

Peru,Deaths,75~80,73.47(66.61to81.05)

Peru,Deaths,80~85,146.75(133.07to161.84)

Peru,Deaths,85~90,252.76(228.73to279.31)

Peru,Deaths,90~95,406.77(366.34to451.67)

Peru,DALYs,20~25,50.81(47.53to54.3)

Peru,DALYs,25~30,52.85(49.67to56.23)

Peru,DALYs,30~35,55.16(52.03to58.48)

Peru,DALYs,35~40,66.92(63.48to70.55)

Peru,DALYs,40~45,89.98(85.94to94.21)

Peru,DALYs,45~50,124.94(119.98to130.11)

Peru,DALYs,50~55,185.23(178.75to191.94)

Peru,DALYs,55~60,253.92(245.78to262.34)

Peru,DALYs,60~65,476.94(463.83to490.42)

Peru,DALYs,65~70,683.7(665.86to702.02)

Peru,DALYs,70~75,1120.69(1093to1149.09)

Peru,DALYs,75~80,1620.65(1574.74to1667.89)

Peru,DALYs,80~85,2473.31(2401.51to2547.26)

Peru,DALYs,85~90,3371.39(3267.15to3478.97)

Peru,DALYs,90~95,4668.26(4504.93to4837.51)

Saint_Lucia,Prevalence,20~25,259.79(220.33to306.31)

Saint_Lucia,Prevalence,25~30,357.81(310.01to412.98)

Saint_Lucia,Prevalence,30~35,485.82(427.63to551.92)

Saint_Lucia,Prevalence,35~40,644.13(574.46to722.24)

Saint_Lucia,Prevalence,40~45,889.16(803.98to983.35)

Saint_Lucia,Prevalence,45~50,1412.31(1294.3to1541.09)

Saint_Lucia,Prevalence,50~55,2281.77(2114.36to2462.44)

Saint_Lucia,Prevalence,55~60,3456.99(3226.83to3703.56)

Saint_Lucia,Prevalence,60~65,4877.67(4573.22to5202.4)

Saint_Lucia,Prevalence,65~70,6691.14(6287.22to7121.02)

Saint_Lucia,Prevalence,70~75,9214.67(8659.22to9805.75)

Saint_Lucia,Prevalence,75~80,12533.62(11653.92to13479.72)

Saint_Lucia,Prevalence,80~85,17086.63(15813.79to18461.92)

Saint_Lucia,Prevalence,85~90,22397.91(20535.79to24428.87)

Saint_Lucia,Prevalence,90~95,27646.23(24698.48to30945.8)

Saint_Lucia,Deaths,20~25,0.26(0to18.34)

Saint_Lucia,Deaths,25~30,0.41(0.01to12.61)

Saint_Lucia,Deaths,30~35,0.6(0.03to11.43)

Saint_Lucia,Deaths,35~40,1.11(0.11to10.89)

Saint_Lucia,Deaths,40~45,2.3(0.41to12.92)

Saint_Lucia,Deaths,45~50,5.31(1.44to19.53)

Saint_Lucia,Deaths,50~55,10.56(3.79to29.38)

Saint_Lucia,Deaths,55~60,21.47(9.47to48.7)

Saint_Lucia,Deaths,60~65,43.59(21.99to86.41)

Saint_Lucia,Deaths,65~70,67.09(36to125.02)

Saint_Lucia,Deaths,70~75,126.73(71.04to226.06)

Saint_Lucia,Deaths,75~80,190.77(97.69to372.51)

Saint_Lucia,Deaths,80~85,303.38(153.24to600.62)

Saint_Lucia,Deaths,85~90,502.99(247.82to1020.91)

Saint_Lucia,Deaths,90~95,813.55(372.95to1774.66)

Saint_Lucia,DALYs,20~25,26.16(16.71to40.95)

Saint_Lucia,DALYs,25~30,38.11(26.11to55.63)

Saint_Lucia,DALYs,30~35,51.26(36.6to71.79)

Saint_Lucia,DALYs,35~40,81.25(61.25to107.78)

Saint_Lucia,DALYs,40~45,140.77(111.65to177.48)

Saint_Lucia,DALYs,45~50,277.21(229.51to334.83)

Saint_Lucia,DALYs,50~55,485.11(414.06to568.34)

Saint_Lucia,DALYs,55~60,847.27(739.56to970.67)

Saint_Lucia,DALYs,60~65,1447.9(1282.22to1634.99)

Saint_Lucia,DALYs,65~70,1890.72(1682.5to2124.72)

Saint_Lucia,DALYs,70~75,2893.26(2582.81to3241.02)

Saint_Lucia,DALYs,75~80,3531.12(3088.33to4037.38)

Saint_Lucia,DALYs,80~85,4431.07(3842.3to5110.06)

Saint_Lucia,DALYs,85~90,5823.08(4975.58to6814.93)

Saint_Lucia,DALYs,90~95,8059.5(6653.52to9762.59)

Croatia,Prevalence,20~25,373.82(362.95to385.02)

Croatia,Prevalence,25~30,503.61(491.48to516.03)

Croatia,Prevalence,30~35,655.68(642.32to669.33)

Croatia,Prevalence,35~40,827.31(812.86to842.01)

Croatia,Prevalence,40~45,1114.17(1097.85to1130.73)

Croatia,Prevalence,45~50,1834.24(1812.15to1856.6)

Croatia,Prevalence,50~55,3075.1(3044.86to3105.64)

Croatia,Prevalence,55~60,4705.13(4664.89to4745.72)

Croatia,Prevalence,60~65,6776.64(6723.75to6829.94)

Croatia,Prevalence,65~70,9953.15(9879.91to10026.94)

Croatia,Prevalence,70~75,14549.26(14444.6to14654.68)

Croatia,Prevalence,75~80,20457.82(20291.36to20625.64)

Croatia,Prevalence,80~85,27329.45(27095.11to27565.81)

Croatia,Prevalence,85~90,34640.04(34299.95to34983.51)

Croatia,Prevalence,90~95,41859.48(41286.07to42440.85)

Croatia,Deaths,20~25,0.29(0.07to1.21)

Croatia,Deaths,25~30,0.31(0.1to1.03)

Croatia,Deaths,30~35,0.45(0.19to1.1)

Croatia,Deaths,35~40,0.8(0.44to1.47)

Croatia,Deaths,40~45,1.67(1.12to2.48)

Croatia,Deaths,45~50,3.83(2.93to5)

Croatia,Deaths,50~55,7.4(6.11to8.97)

Croatia,Deaths,55~60,13.97(12.07to16.17)

Croatia,Deaths,60~65,28.16(25.06to31.63)

Croatia,Deaths,65~70,51.75(46.69to57.36)

Croatia,Deaths,70~75,96.8(87.86to106.64)

Croatia,Deaths,75~80,172.92(155.5to192.28)

Croatia,Deaths,80~85,297.3(267.14to330.87)

Croatia,Deaths,85~90,481.82(430.84to538.84)

Croatia,Deaths,90~95,697.42(613.33to793.03)

Croatia,DALYs,20~25,39.99(34.95to45.75)

Croatia,DALYs,25~30,48.65(43.48to54.43)

Croatia,DALYs,30~35,62.35(56.87to68.37)

Croatia,DALYs,35~40,85.25(79.2to91.76)

Croatia,DALYs,40~45,133.8(126.47to141.56)

Croatia,DALYs,45~50,250.51(239.99to261.49)

Croatia,DALYs,50~55,426.16(411.97to440.83)

Croatia,DALYs,55~60,682.58(663.55to702.16)

Croatia,DALYs,60~65,1117.63(1090.55to1145.39)

Croatia,DALYs,65~70,1702.06(1663.7to1741.29)

Croatia,DALYs,70~75,2573.83(2517.32to2631.6)

Croatia,DALYs,75~80,3638.38(3549.22to3729.78)

Croatia,DALYs,80~85,4871.91(4747.72to4999.34)

Croatia,DALYs,85~90,6224.95(6047.76to6407.33)

Croatia,DALYs,90~95,7737.84(7444.38to8042.86)

Colombia,Prevalence,20~25,409.22(393.9to425.14)

Colombia,Prevalence,25~30,538.25(520.76to556.32)

Colombia,Prevalence,30~35,694.97(674.92to715.6)

Colombia,Prevalence,35~40,872.63(849.99to895.88)

Colombia,Prevalence,40~45,1161.75(1135.19to1188.93)

Colombia,Prevalence,45~50,1873.63(1837.25to1910.73)

Colombia,Prevalence,50~55,3068.33(3017.3to3120.22)

Colombia,Prevalence,55~60,4562.12(4493.52to4631.77)

Colombia,Prevalence,60~65,6328.3(6238.4to6419.49)

Colombia,Prevalence,65~70,8824.18(8703.28to8946.75)

Colombia,Prevalence,70~75,12522.15(12352.18to12694.45)

Colombia,Prevalence,75~80,17756.57(17475.97to18041.67)

Colombia,Prevalence,80~85,24857.25(24439.4to25282.25)

Colombia,Prevalence,85~90,33809.25(33182.4to34447.95)

Colombia,Prevalence,90~95,42996.14(42050.26to43963.3)

Colombia,Deaths,20~25,0.99(0.71to1.38)

Colombia,Deaths,25~30,1.1(0.83to1.47)

Colombia,Deaths,30~35,1.29(1.01to1.66)

Colombia,Deaths,35~40,1.65(1.34to2.02)

Colombia,Deaths,40~45,2.68(2.28to3.14)

Colombia,Deaths,45~50,5(4.44to5.63)

Colombia,Deaths,50~55,10.69(9.82to11.64)

Colombia,Deaths,55~60,19.8(18.51to21.19)

Colombia,Deaths,60~65,42.32(40.09to44.68)

Colombia,Deaths,65~70,72.32(68.86to75.95)

Colombia,Deaths,70~75,131.95(126.19to137.97)

Colombia,Deaths,75~80,215.99(205.37to227.16)

Colombia,Deaths,80~85,367.35(349.12to386.52)

Colombia,Deaths,85~90,573.34(544.04to604.22)

Colombia,Deaths,90~95,854.67(808.33to903.67)

Colombia,DALYs,20~25,82.58(70.93to96.14)

Colombia,DALYs,25~30,93.22(81.53to106.58)

Colombia,DALYs,30~35,107.6(95.5to121.22)

Colombia,DALYs,35~40,128.48(115.63to142.75)

Colombia,DALYs,40~45,180.96(165.65to197.67)

Colombia,DALYs,45~50,297.84(277.38to319.81)

Colombia,DALYs,50~55,539.15(509.55to570.45)

Colombia,DALYs,55~60,854(813.49to896.52)

Colombia,DALYs,60~65,1484.44(1422.69to1548.87)

Colombia,DALYs,65~70,2128.01(2043.81to2215.67)

Colombia,DALYs,70~75,3169.7(3050.23to3293.85)

Colombia,DALYs,75~80,4179.14(3998.42to4368.03)

Colombia,DALYs,80~85,5584.94(5333.22to5848.54)

Colombia,DALYs,85~90,6967.73(6627.36to7325.58)

Colombia,DALYs,90~95,8992.32(8490to9524.36)

Somalia,Prevalence,20~25,462.14(454.01to470.41)

Somalia,Prevalence,25~30,637.21(627.05to647.54)

Somalia,Prevalence,30~35,821.8(809.67to834.1)

Somalia,Prevalence,35~40,1005.12(991.25to1019.17)

Somalia,Prevalence,40~45,1286.53(1270.36to1302.91)

Somalia,Prevalence,45~50,2002.1(1979.21to2025.25)

Somalia,Prevalence,50~55,3208.32(3174.87to3242.12)

Somalia,Prevalence,55~60,4663.27(4617.27to4709.73)

Somalia,Prevalence,60~65,6190.38(6129.69to6251.68)

Somalia,Prevalence,65~70,7981.62(7900.1to8063.99)

Somalia,Prevalence,70~75,10219.86(10102.2to10338.89)

Somalia,Prevalence,75~80,12877.57(12680.48to13077.72)

Somalia,Prevalence,80~85,15584.37(15262.08to15913.46)

Somalia,Prevalence,85~90,17379.52(16799.03to17980.07)

Somalia,Prevalence,90~95,17642.01(16439.63to18932.32)

Somalia,Deaths,20~25,0.93(0.64to1.33)

Somalia,Deaths,25~30,1.03(0.73to1.44)

Somalia,Deaths,30~35,1.97(1.5to2.57)

Somalia,Deaths,35~40,3.37(2.71to4.19)

Somalia,Deaths,40~45,6.01(5.06to7.15)

Somalia,Deaths,45~50,15.4(13.59to17.44)

Somalia,Deaths,50~55,28.47(25.7to31.53)

Somalia,Deaths,55~60,62.59(57.65to67.95)

Somalia,Deaths,60~65,117.56(109.33to126.41)

Somalia,Deaths,65~70,195.3(182.18to209.37)

Somalia,Deaths,70~75,317.4(295.65to340.74)

Somalia,Deaths,75~80,404.6(369.62to442.89)

Somalia,Deaths,80~85,556.54(499.47to620.14)

Somalia,Deaths,85~90,619.91(529.56to725.68)

Somalia,Deaths,90~95,718.29(541.07to953.57)

Somalia,DALYs,20~25,113.01(109.14to117.03)

Somalia,DALYs,25~30,135.73(131.45to140.15)

Somalia,DALYs,30~35,201.66(196.06to207.42)

Somalia,DALYs,35~40,281.77(274.82to288.88)

Somalia,DALYs,40~45,416.52(407.63to425.6)

Somalia,DALYs,45~50,851.16(836.47to866.12)

Somalia,DALYs,50~55,1389.97(1368.84to1411.43)

Somalia,DALYs,55~60,2532.89(2499.26to2566.96)

Somalia,DALYs,60~65,3978.03(3928.48to4028.21)

Somalia,DALYs,65~70,5517.29(5447.95to5587.52)

Somalia,DALYs,70~75,7351.27(7250.98to7452.95)

Somalia,DALYs,75~80,7685.49(7543.41to7830.26)

Somalia,DALYs,80~85,8378.89(8169.71to8593.43)

Somalia,DALYs,85~90,7597.87(7278.13to7931.66)

Somalia,DALYs,90~95,7572.91(6941.49to8261.77)

Uganda,Prevalence,20~25,449.11(443.31to455)

Uganda,Prevalence,25~30,599.64(592.55to606.81)

Uganda,Prevalence,30~35,768.08(759.53to776.73)

Uganda,Prevalence,35~40,942.41(932.31to952.61)

Uganda,Prevalence,40~45,1189.07(1177.21to1201.06)

Uganda,Prevalence,45~50,1716.68(1700.78to1732.73)

Uganda,Prevalence,50~55,2570.32(2548.3to2592.54)

Uganda,Prevalence,55~60,3656.14(3626.55to3685.97)

Uganda,Prevalence,60~65,4867.44(4829.09to4906.09)

Uganda,Prevalence,65~70,6249.83(6200.49to6299.56)

Uganda,Prevalence,70~75,7926.47(7860.73to7992.76)

Uganda,Prevalence,75~80,9885.07(9786.35to9984.78)

Uganda,Prevalence,80~85,11906.64(11767.57to12047.36)

Uganda,Prevalence,85~90,13262.52(13055.41to13472.92)

Uganda,Prevalence,90~95,13658.82(13310.6to14016.15)

Uganda,Deaths,20~25,0.91(0.68to1.2)

Uganda,Deaths,25~30,0.99(0.76to1.29)

Uganda,Deaths,30~35,1.77(1.43to2.19)

Uganda,Deaths,35~40,2.77(2.3to3.33)

Uganda,Deaths,40~45,4.4(3.77to5.14)

Uganda,Deaths,45~50,10.41(9.28to11.67)

Uganda,Deaths,50~55,17.72(16.12to19.48)

Uganda,Deaths,55~60,37.11(34.41to40.03)

Uganda,Deaths,60~65,69.33(64.94to74.01)

Uganda,Deaths,65~70,118.58(111.65to125.94)

Uganda,Deaths,70~75,192.78(181.82to204.41)

Uganda,Deaths,75~80,253.86(237.31to271.56)

Uganda,Deaths,80~85,359.82(335.01to386.46)

Uganda,Deaths,85~90,398.12(365.79to433.3)

Uganda,Deaths,90~95,476.46(424.58to534.69)

Uganda,DALYs,20~25,108.81(103.18to114.75)

Uganda,DALYs,25~30,128.7(122.56to135.15)

Uganda,DALYs,30~35,182.56(174.67to190.8)

Uganda,DALYs,35~40,241.7(232to251.81)

Uganda,DALYs,40~45,328.25(316.31to340.63)

Uganda,DALYs,45~50,606.24(587.8to625.26)

Uganda,DALYs,50~55,915.57(890.75to941.09)

Uganda,DALYs,55~60,1578.72(1541.18to1617.18)

Uganda,DALYs,60~65,2454.31(2400.32to2509.52)

Uganda,DALYs,65~70,3480.13(3406.08to3555.79)

Uganda,DALYs,70~75,4624.48(4524.41to4726.75)

Uganda,DALYs,75~80,4972.04(4843.32to5104.17)

Uganda,DALYs,80~85,5556.68(5391.79to5726.61)

Uganda,DALYs,85~90,5011.04(4808.8to5221.79)

Uganda,DALYs,90~95,5148.05(4822.36to5495.73)

Eswatini,Prevalence,20~25,407.92(383.51to433.88)

Eswatini,Prevalence,25~30,553.09(522.95to584.96)

Eswatini,Prevalence,30~35,720.32(683.76to758.84)

Eswatini,Prevalence,35~40,900.15(857.02to945.45)

Eswatini,Prevalence,40~45,1185.93(1134.24to1239.97)

Eswatini,Prevalence,45~50,1902.81(1828.95to1979.65)

Eswatini,Prevalence,50~55,3073.34(2966to3184.57)

Eswatini,Prevalence,55~60,4408.64(4262.56to4559.72)

Eswatini,Prevalence,60~65,5796.62(5609.18to5990.33)

Eswatini,Prevalence,65~70,7485.86(7243.51to7736.32)

Eswatini,Prevalence,70~75,9423.64(9096.6to9762.44)

Eswatini,Prevalence,75~80,11522.98(11023.96to12044.59)

Eswatini,Prevalence,80~85,14023.15(13261.65to14828.37)

Eswatini,Prevalence,85~90,16566.38(15249.54to17996.94)

Eswatini,Prevalence,90~95,19079.83(16361.27to22250.1)

Eswatini,Deaths,20~25,0.59(0.18to1.96)

Eswatini,Deaths,25~30,0.96(0.35to2.61)

Eswatini,Deaths,30~35,1.72(0.76to3.89)

Eswatini,Deaths,35~40,3.66(1.95to6.88)

Eswatini,Deaths,40~45,9.73(6.21to15.25)

Eswatini,Deaths,45~50,23.78(17.05to33.16)

Eswatini,Deaths,50~55,48.89(37.74to63.35)

Eswatini,Deaths,55~60,95.76(77.35to118.55)

Eswatini,Deaths,60~65,145.65(119.41to177.65)

Eswatini,Deaths,65~70,270.21(224.68to324.96)

Eswatini,Deaths,70~75,502.33(418.3to603.24)

Eswatini,Deaths,75~80,668.16(537.28to830.92)

Eswatini,Deaths,80~85,848.43(659.21to1091.97)

Eswatini,Deaths,85~90,1006.18(718.53to1408.97)

Eswatini,Deaths,90~95,1147.1(648.66to2028.57)

Eswatini,DALYs,20~25,76.21(67.14to86.51)

Eswatini,DALYs,25~30,111.43(99.71to124.51)

Eswatini,DALYs,30~35,167.21(151.54to184.49)

Eswatini,DALYs,35~40,278.68(256.09to303.27)

Eswatini,DALYs,40~45,576.29(538.78to616.41)

Eswatini,DALYs,45~50,1197.61(1134.58to1264.13)

Eswatini,DALYs,50~55,2157.83(2063.05to2256.97)

Eswatini,DALYs,55~60,3639.78(3498.57to3786.69)

Eswatini,DALYs,60~65,4790.65(4608.44to4980.06)

Eswatini,DALYs,65~70,7351.3(7078.25to7634.88)

Eswatini,DALYs,70~75,11059.43(10633.39to11502.54)

Eswatini,DALYs,75~80,11890.31(11317.56to12492.03)

Eswatini,DALYs,80~85,12000.47(11257.24to12792.77)

Eswatini,DALYs,85~90,11463.31(10376.48to12663.99)

Eswatini,DALYs,90~95,11381.99(9395.2to13788.93)

Slovenia,Prevalence,20~25,367.5(351.01to384.76)

Slovenia,Prevalence,25~30,491.42(473.3to510.24)

Slovenia,Prevalence,30~35,637.76(617.85to658.3)

Slovenia,Prevalence,35~40,805.57(783.95to827.78)

Slovenia,Prevalence,40~45,1078.4(1054.1to1103.25)

Slovenia,Prevalence,45~50,1716.3(1684.42to1748.77)

Slovenia,Prevalence,50~55,2783.82(2740.82to2827.49)

Slovenia,Prevalence,55~60,4194.12(4137.45to4251.55)

Slovenia,Prevalence,60~65,5994.67(5920.38to6069.89)

Slovenia,Prevalence,65~70,8608.66(8507.91to8710.6)

Slovenia,Prevalence,70~75,12232.11(12090.81to12375.07)

Slovenia,Prevalence,75~80,16727.51(16508.91to16949.02)

Slovenia,Prevalence,80~85,21809.01(21510.41to22111.76)

Slovenia,Prevalence,85~90,27110.39(26700.59to27526.49)

Slovenia,Prevalence,90~95,32411.76(31805.64to33029.44)

Slovenia,Deaths,20~25,0.65(0.08to5.17)

Slovenia,Deaths,25~30,0.58(0.1to3.45)

Slovenia,Deaths,30~35,0.74(0.19to2.8)

Slovenia,Deaths,35~40,1.16(0.47to2.88)

Slovenia,Deaths,40~45,2.24(1.24to4.02)

Slovenia,Deaths,45~50,4.4(2.95to6.56)

Slovenia,Deaths,50~55,7.53(5.58to10.15)

Slovenia,Deaths,55~60,11.91(9.37to15.14)

Slovenia,Deaths,60~65,20.59(16.86to25.15)

Slovenia,Deaths,65~70,32.02(26.68to38.43)

Slovenia,Deaths,70~75,51.84(43.55to61.71)

Slovenia,Deaths,75~80,81.61(67.75to98.31)

Slovenia,Deaths,80~85,119.75(99.35to144.32)

Slovenia,Deaths,85~90,143.07(118.01to173.46)

Slovenia,Deaths,90~95,173.69(140.86to214.19)

Slovenia,DALYs,20~25,59.39(45.73to77.12)

Slovenia,DALYs,25~30,67.14(53.95to83.56)

Slovenia,DALYs,30~35,82.08(68.61to98.21)

Slovenia,DALYs,35~40,106.19(91.99to122.57)

Slovenia,DALYs,40~45,159.42(143.05to177.67)

Slovenia,DALYs,45~50,270.26(248.69to293.71)

Slovenia,DALYs,50~55,420.42(392.94to449.82)

Slovenia,DALYs,55~60,597.57(563.64to633.53)

Slovenia,DALYs,60~65,875.91(831.35to922.85)

Slovenia,DALYs,65~70,1170.34(1113.94to1229.6)

Slovenia,DALYs,70~75,1569.75(1495.28to1647.92)

Slovenia,DALYs,75~80,1990.91(1887.63to2099.84)

Slovenia,DALYs,80~85,2327.93(2202.86to2460.11)

Slovenia,DALYs,85~90,2317.87(2181.34to2462.95)

Slovenia,DALYs,90~95,2479.51(2302.18to2670.51)

Belarus,Prevalence,20~25,539.71(506.8to574.75)

Belarus,Prevalence,25~30,725.15(688.47to763.78)

Belarus,Prevalence,30~35,916.13(876.62to957.41)

Belarus,Prevalence,35~40,1101.58(1059.57to1145.25)

Belarus,Prevalence,40~45,1401.32(1355.19to1449.02)

Belarus,Prevalence,45~50,2177.29(2116.62to2239.7)

Belarus,Prevalence,50~55,3351.83(3272.04to3433.56)

Belarus,Prevalence,55~60,4637.73(4538.06to4739.58)

Belarus,Prevalence,60~65,6055.37(5932.41to6180.88)

Belarus,Prevalence,65~70,7977.16(7817.69to8139.89)

Belarus,Prevalence,70~75,10379.27(10169.07to10593.81)

Belarus,Prevalence,75~80,13074.94(12775.41to13381.5)

Belarus,Prevalence,80~85,16134.03(15744to16533.72)

Belarus,Prevalence,85~90,19325.39(18786.33to19879.92)

Belarus,Prevalence,90~95,22258.52(21438.08to23110.36)

Belarus,Deaths,20~25,1.92(1.11to3.31)

Belarus,Deaths,25~30,2.45(1.63to3.7)

Belarus,Deaths,30~35,3.47(2.57to4.68)

Belarus,Deaths,35~40,4.74(3.79to5.92)

Belarus,Deaths,40~45,7.12(6.06to8.37)

Belarus,Deaths,45~50,11.56(10.25to13.03)

Belarus,Deaths,50~55,18.71(17.07to20.51)

Belarus,Deaths,55~60,26.1(24.1to28.27)

Belarus,Deaths,60~65,36.85(34.22to39.67)

Belarus,Deaths,65~70,42.04(39.07to45.23)

Belarus,Deaths,70~75,46.06(42.78to49.59)

Belarus,Deaths,75~80,48.2(44.54to52.17)

Belarus,Deaths,80~85,45.93(42.27to49.91)

Belarus,Deaths,85~90,39.11(35.65to42.91)

Belarus,Deaths,90~95,31.47(28.13to35.2)

Belarus,DALYs,20~25,150(129.32to173.99)

Belarus,DALYs,25~30,187.28(166.38to210.8)

Belarus,DALYs,30~35,239.53(218.05to263.13)

Belarus,DALYs,35~40,295.31(273.61to318.74)

Belarus,DALYs,40~45,395.47(372.56to419.78)

Belarus,DALYs,45~50,585.07(557.82to613.65)

Belarus,DALYs,50~55,851.34(818.68to885.29)

Belarus,DALYs,55~60,1066.16(1028.67to1105.03)

Belarus,DALYs,60~65,1327.89(1283.22to1374.11)

Belarus,DALYs,65~70,1351.02(1304.81to1398.86)

Belarus,DALYs,70~75,1297.88(1251.71to1345.75)

Belarus,DALYs,75~80,1170.03(1123.73to1218.24)

Belarus,DALYs,80~85,969.03(926.77to1013.22)

Belarus,DALYs,85~90,741.17(702.12to782.4)

Belarus,DALYs,90~95,580.42(539.27to624.71)

Suriname,Prevalence,20~25,267.94(242.85to295.62)

Suriname,Prevalence,25~30,357.42(328.21to389.24)

Suriname,Prevalence,30~35,474.24(439.61to511.6)

Suriname,Prevalence,35~40,623.54(582.25to667.75)

Suriname,Prevalence,40~45,854.12(804.09to907.27)

Suriname,Prevalence,45~50,1317.02(1249.71to1387.96)

Suriname,Prevalence,50~55,2085.18(1991.42to2183.35)

Suriname,Prevalence,55~60,3169.72(3039.55to3305.46)

Suriname,Prevalence,60~65,4530.4(4353.92to4714.03)

Suriname,Prevalence,65~70,6160.16(5923.74to6406)

Suriname,Prevalence,70~75,8252.96(7930.43to8588.61)

Suriname,Prevalence,75~80,10989.01(10487.93to11514.04)

Suriname,Prevalence,80~85,14407.53(13690.04to15162.63)

Suriname,Prevalence,85~90,18403.27(17326.01to19547.52)

Suriname,Prevalence,90~95,23265.38(21490.6to25186.72)

Suriname,Deaths,20~25,0.3(0.02to3.63)

Suriname,Deaths,25~30,0.37(0.04to3.19)

Suriname,Deaths,30~35,0.58(0.1to3.44)

Suriname,Deaths,35~40,1.04(0.25to4.28)

Suriname,Deaths,40~45,1.98(0.67to5.88)

Suriname,Deaths,45~50,4.08(1.78to9.35)

Suriname,Deaths,50~55,8.96(4.77to16.85)

Suriname,Deaths,55~60,18.42(11.04to30.73)

Suriname,Deaths,60~65,37.95(24.72to58.25)

Suriname,Deaths,65~70,64.3(43.31to95.48)

Suriname,Deaths,70~75,99.96(67.58to147.87)

Suriname,Deaths,75~80,150.13(96.5to233.56)

Suriname,Deaths,80~85,223.85(141.59to353.91)

Suriname,Deaths,85~90,316.68(192.77to520.23)

Suriname,Deaths,90~95,381.38(209.61to693.89)

Suriname,DALYs,20~25,30.83(23.65to40.18)

Suriname,DALYs,25~30,37.64(29.83to47.48)

Suriname,DALYs,30~35,52.49(42.89to64.25)

Suriname,DALYs,35~40,79.63(66.96to94.69)

Suriname,DALYs,40~45,128.78(111.55to148.67)

Suriname,DALYs,45~50,227.34(201.98to255.88)

Suriname,DALYs,50~55,423.89(384.58to467.21)

Suriname,DALYs,55~60,740.57(680.51to805.93)

Suriname,DALYs,60~65,1273.29(1180.48to1373.4)

Suriname,DALYs,65~70,1806.51(1678.95to1943.77)

Suriname,DALYs,70~75,2318.49(2149.9to2500.3)

Suriname,DALYs,75~80,2810.08(2574.07to3067.74)

Suriname,DALYs,80~85,3323.6(3019.2to3658.69)

Suriname,DALYs,85~90,3780.98(3372.32to4239.17)

Suriname,DALYs,90~95,4032.17(3444.31to4720.35)

Hungary,Prevalence,20~25,442.3(431.42to453.46)

Hungary,Prevalence,25~30,615.3(602.68to628.18)

Hungary,Prevalence,30~35,803.6(789.59to817.86)

Hungary,Prevalence,35~40,995.58(980.65to1010.74)

Hungary,Prevalence,40~45,1366.45(1349.45to1383.66)

Hungary,Prevalence,45~50,2498.47(2473.87to2523.32)

Hungary,Prevalence,50~55,4225.6(4191.08to4260.41)

Hungary,Prevalence,55~60,6081.84(6037.64to6126.36)

Hungary,Prevalence,60~65,8216.62(8161.16to8272.46)

Hungary,Prevalence,65~70,11531.5(11457.31to11606.16)

Hungary,Prevalence,70~75,16231.25(16127.14to16336.03)

Hungary,Prevalence,75~80,22093.65(21932.27to22256.21)

Hungary,Prevalence,80~85,28719.96(28495.65to28946.04)

Hungary,Prevalence,85~90,35683.2(35364.83to36004.44)

Hungary,Prevalence,90~95,42291.6(41788.13to42801.13)

Hungary,Deaths,20~25,0.32(0.16to0.65)

Hungary,Deaths,25~30,0.45(0.26to0.77)

Hungary,Deaths,30~35,0.88(0.62to1.25)

Hungary,Deaths,35~40,2.04(1.64to2.53)

Hungary,Deaths,40~45,5.09(4.44to5.83)

Hungary,Deaths,45~50,11.81(10.75to12.98)

Hungary,Deaths,50~55,24.46(22.77to26.27)

Hungary,Deaths,55~60,43.99(41.52to46.6)

Hungary,Deaths,60~65,79.12(75.33to83.09)

Hungary,Deaths,65~70,119.74(114.45to125.29)

Hungary,Deaths,70~75,185.75(177.62to194.27)

Hungary,Deaths,75~80,285.33(271.28to300.11)

Hungary,Deaths,80~85,431.42(409.59to454.42)

Hungary,Deaths,85~90,618.68(585.19to654.09)

Hungary,Deaths,90~95,726.71(678.35to778.52)

Hungary,DALYs,20~25,48.21(39.1to59.43)

Hungary,DALYs,25~30,63.46(53.53to75.24)

Hungary,DALYs,30~35,92.85(81.48to105.8)

Hungary,DALYs,35~40,155.78(141.59to171.39)

Hungary,DALYs,40~45,307.94(287.97to329.29)

Hungary,DALYs,45~50,627.8(596.86to660.34)

Hungary,DALYs,50~55,1140.21(1094.22to1188.13)

Hungary,DALYs,55~60,1763.54(1702.09to1827.2)

Hungary,DALYs,60~65,2659.84(2576.14to2746.27)

Hungary,DALYs,65~70,3421.45(3317.47to3528.69)

Hungary,DALYs,70~75,4417.66(4279.42to4560.37)

Hungary,DALYs,75~80,5495.54(5297.01to5701.53)

Hungary,DALYs,80~85,6588.63(6330.27to6857.53)

Hungary,DALYs,85~90,7597.01(7249.36to7961.33)

Hungary,DALYs,90~95,7937.48(7426.83to8483.25)

Montenegro,Prevalence,20~25,327.75(302.76to354.8)

Montenegro,Prevalence,25~30,441.39(412.59to472.19)

Montenegro,Prevalence,30~35,577.57(544.78to612.33)

Montenegro,Prevalence,35~40,737.91(701.02to776.75)

Montenegro,Prevalence,40~45,986.27(944.12to1030.3)

Montenegro,Prevalence,45~50,1511.88(1456.68to1569.17)

Montenegro,Prevalence,50~55,2397.2(2323.15to2473.6)

Montenegro,Prevalence,55~60,3663.59(3564.24to3765.72)

Montenegro,Prevalence,60~65,5384.73(5250.33to5522.57)

Montenegro,Prevalence,65~70,7814.86(7628.96to8005.29)

Montenegro,Prevalence,70~75,11263.11(10994.56to11538.23)

Montenegro,Prevalence,75~80,15866.24(15429.77to16315.06)

Montenegro,Prevalence,80~85,21530.36(20894.1to22186)

Montenegro,Prevalence,85~90,27944.41(26957.28to28967.69)

Montenegro,Prevalence,90~95,34778.3(33079.13to36564.76)

Montenegro,Deaths,20~25,0.09(0to35.6)

Montenegro,Deaths,25~30,0.11(0to15.79)

Montenegro,Deaths,30~35,0.17(0to6.8)

Montenegro,Deaths,35~40,0.28(0.02to4.24)

Montenegro,Deaths,40~45,0.59(0.1to3.67)

Montenegro,Deaths,45~50,1.4(0.41to4.74)

Montenegro,Deaths,50~55,2.86(1.2to6.82)

Montenegro,Deaths,55~60,5.38(2.74to10.57)

Montenegro,Deaths,60~65,11.07(6.41to19.13)

Montenegro,Deaths,65~70,18.76(11.42to30.83)

Montenegro,Deaths,70~75,32.43(20.07to52.4)

Montenegro,Deaths,75~80,52.71(30.74to90.38)

Montenegro,Deaths,80~85,81.59(46.74to142.42)

Montenegro,Deaths,85~90,124.78(68.01to228.95)

Montenegro,Deaths,90~95,158.65(74.09to339.72)

Montenegro,DALYs,20~25,23.56(17.14to32.38)

Montenegro,DALYs,25~30,30.36(23.18to39.78)

Montenegro,DALYs,30~35,39.54(31.43to49.74)

Montenegro,DALYs,35~40,51.73(42.52to62.92)

Montenegro,DALYs,40~45,75.67(64.52to88.74)

Montenegro,DALYs,45~50,130.29(114.68to148.01)

Montenegro,DALYs,50~55,219.01(197.52to242.85)

Montenegro,DALYs,55~60,347.81(318.39to379.94)

Montenegro,DALYs,60~65,568.46(525.55to614.87)

Montenegro,DALYs,65~70,813.71(755.09to876.88)

Montenegro,DALYs,70~75,1152.2(1068.79to1242.11)

Montenegro,DALYs,75~80,1532.22(1404.79to1671.21)

Montenegro,DALYs,80~85,1937.78(1763.84to2128.87)

Montenegro,DALYs,85~90,2388.76(2132.55to2675.74)

Montenegro,DALYs,90~95,2712.36(2301.84to3196.1)

El_Salvador,Prevalence,20~25,307.49(298.83to316.4)

El_Salvador,Prevalence,25~30,403.45(393.29to413.87)

El_Salvador,Prevalence,30~35,524.81(512.76to537.14)

El_Salvador,Prevalence,35~40,674.19(660.02to688.66)

El_Salvador,Prevalence,40~45,902.24(885.45to919.34)

El_Salvador,Prevalence,45~50,1362.16(1339.82to1384.88)

El_Salvador,Prevalence,50~55,2120.85(2090.25to2151.91)

El_Salvador,Prevalence,55~60,3189.73(3148.11to3231.91)

El_Salvador,Prevalence,60~65,4601.73(4545.62to4658.53)

El_Salvador,Prevalence,65~70,6514(6437.96to6590.94)

El_Salvador,Prevalence,70~75,9416.08(9307.74to9525.68)

El_Salvador,Prevalence,75~80,13862.84(13681.23to14046.87)

El_Salvador,Prevalence,80~85,20559.58(20279.46to20843.57)

El_Salvador,Prevalence,85~90,29697.15(29265.29to30135.38)

El_Salvador,Prevalence,90~95,40513.89(39838to41201.24)

El_Salvador,Deaths,20~25,0.38(0.17to0.84)

El_Salvador,Deaths,25~30,0.49(0.25to0.97)

El_Salvador,Deaths,30~35,0.72(0.4to1.3)

El_Salvador,Deaths,35~40,1.1(0.67to1.79)

El_Salvador,Deaths,40~45,1.88(1.27to2.78)

El_Salvador,Deaths,45~50,3.49(2.57to4.75)

El_Salvador,Deaths,50~55,6.98(5.51to8.85)

El_Salvador,Deaths,55~60,12.59(10.37to15.29)

El_Salvador,Deaths,60~65,25.52(21.76to29.93)

El_Salvador,Deaths,65~70,43.12(37.36to49.77)

El_Salvador,Deaths,70~75,86.26(75.61to98.41)

El_Salvador,Deaths,75~80,150.03(129.63to173.65)

El_Salvador,Deaths,80~85,255.59(220.69to296)

El_Salvador,Deaths,85~90,423.64(364.99to491.7)

El_Salvador,Deaths,90~95,627.98(537.2to734.11)

El_Salvador,DALYs,20~25,40.73(37.6to44.12)

El_Salvador,DALYs,25~30,50.15(46.68to53.87)

El_Salvador,DALYs,30~35,66.33(62.19to70.74)

El_Salvador,DALYs,35~40,89.17(84.2to94.43)

El_Salvador,DALYs,40~45,130.7(124.48to137.23)

El_Salvador,DALYs,45~50,210.55(202.01to219.44)

El_Salvador,DALYs,50~55,358.86(346.61to371.54)

El_Salvador,DALYs,55~60,557.4(540.64to574.68)

El_Salvador,DALYs,60~65,930.63(905.71to956.24)

El_Salvador,DALYs,65~70,1319.13(1285.69to1353.45)

El_Salvador,DALYs,70~75,2117.59(2066.15to2170.3)

El_Salvador,DALYs,75~80,2960.85(2879.92to3044.06)

El_Salvador,DALYs,80~85,3990.12(3877.37to4106.15)

El_Salvador,DALYs,85~90,5291.98(5133.98to5454.85)

El_Salvador,DALYs,90~95,6868.47(6638.19to7106.73)

Burkina_Faso,Prevalence,20~25,435.3(427.65to443.09)

Burkina_Faso,Prevalence,25~30,582.67(573.41to592.08)

Burkina_Faso,Prevalence,30~35,748.31(737.3to759.49)

Burkina_Faso,Prevalence,35~40,923.42(910.61to936.41)

Burkina_Faso,Prevalence,40~45,1172.8(1157.82to1187.97)

Burkina_Faso,Prevalence,45~50,1688.39(1668.59to1708.42)

Burkina_Faso,Prevalence,50~55,2478.32(2451.6to2505.33)

Burkina_Faso,Prevalence,55~60,3428.73(3393.86to3463.96)

Burkina_Faso,Prevalence,60~65,4504.62(4459.95to4549.74)

Burkina_Faso,Prevalence,65~70,5704.53(5647.6to5762.04)

Burkina_Faso,Prevalence,70~75,7208.5(7133.54to7284.25)

Burkina_Faso,Prevalence,75~80,9196.58(9081.94to9312.67)

Burkina_Faso,Prevalence,80~85,11745.76(11575.52to11918.51)

Burkina_Faso,Prevalence,85~90,14540.88(14267.98to14818.99)

Burkina_Faso,Prevalence,90~95,17084.17(16583.72to17599.73)

Burkina_Faso,Deaths,20~25,0.15(0.07to0.3)

Burkina_Faso,Deaths,25~30,0.53(0.34to0.83)

Burkina_Faso,Deaths,30~35,0.88(0.61to1.26)

Burkina_Faso,Deaths,35~40,1.01(0.71to1.42)

Burkina_Faso,Deaths,40~45,1.84(1.39to2.43)

Burkina_Faso,Deaths,45~50,5.72(4.74to6.89)

Burkina_Faso,Deaths,50~55,11.26(9.71to13.06)

Burkina_Faso,Deaths,55~60,19.12(16.85to21.69)

Burkina_Faso,Deaths,60~65,38.32(34.47to42.6)

Burkina_Faso,Deaths,65~70,70.6(64.19to77.65)

Burkina_Faso,Deaths,70~75,119.14(108.6to130.71)

Burkina_Faso,Deaths,75~80,170.78(153.46to190.06)

Burkina_Faso,Deaths,80~85,244.93(218.43to274.65)

Burkina_Faso,Deaths,85~90,397.24(349.88to451.01)

Burkina_Faso,Deaths,90~95,524.55(444.26to619.36)

Burkina_Faso,DALYs,20~25,54.34(51.97to56.81)

Burkina_Faso,DALYs,25~30,92.25(88.86to95.76)

Burkina_Faso,DALYs,30~35,125.37(121.22to129.65)

Burkina_Faso,DALYs,35~40,145.87(141.3to150.58)

Burkina_Faso,DALYs,40~45,204.65(198.9to210.57)

Burkina_Faso,DALYs,45~50,408.7(399.28to418.33)

Burkina_Faso,DALYs,50~55,667.42(653.99to681.12)

Burkina_Faso,DALYs,55~60,967.11(949.25to985.3)

Burkina_Faso,DALYs,60~65,1533.91(1507.98to1560.29)

Burkina_Faso,DALYs,65~70,2261.86(2225.22to2299.1)

Burkina_Faso,DALYs,70~75,3074.06(3023.3to3125.68)

Burkina_Faso,DALYs,75~80,3596.41(3525.41to3668.84)

Burkina_Faso,DALYs,80~85,4135.76(4041.06to4232.68)

Burkina_Faso,DALYs,85~90,5255.61(5105.18to5410.48)

Burkina_Faso,DALYs,90~95,6014.75(5752.59to6288.85)

Honduras,Prevalence,20~25,392.66(383.53to402.02)

Honduras,Prevalence,25~30,520.42(509.53to531.54)

Honduras,Prevalence,30~35,671.23(658.35to684.37)

Honduras,Prevalence,35~40,838.2(823.34to853.34)

Honduras,Prevalence,40~45,1107.7(1089.96to1125.74)

Honduras,Prevalence,45~50,1800.09(1774.88to1825.67)

Honduras,Prevalence,50~55,3043.05(3005.87to3080.69)

Honduras,Prevalence,55~60,4653.7(4602.4to4705.57)

Honduras,Prevalence,60~65,6597.17(6528.85to6666.2)

Honduras,Prevalence,65~70,9471.54(9377.8to9566.22)

Honduras,Prevalence,70~75,13825.58(13688.95to13963.58)

Honduras,Prevalence,75~80,20053.84(19818.75to20291.71)

Honduras,Prevalence,80~85,28500.24(28132.99to28872.28)

Honduras,Prevalence,85~90,39045.09(38441.54to39658.12)

Honduras,Prevalence,90~95,49183.78(48040.85to50353.91)

Honduras,Deaths,20~25,1.38(0.91to2.1)

Honduras,Deaths,25~30,1.61(1.11to2.33)

Honduras,Deaths,30~35,2.07(1.5to2.87)

Honduras,Deaths,35~40,2.86(2.17to3.78)

Honduras,Deaths,40~45,5.24(4.22to6.51)

Honduras,Deaths,45~50,9.63(8.08to11.47)

Honduras,Deaths,50~55,21.19(18.54to24.22)

Honduras,Deaths,55~60,43.59(39.18to48.51)

Honduras,Deaths,60~65,94.97(87.17to103.47)

Honduras,Deaths,65~70,173.42(160.7to187.14)

Honduras,Deaths,70~75,349.27(325to375.34)

Honduras,Deaths,75~80,610.56(560.78to664.75)

Honduras,Deaths,80~85,1046.86(956.91to1145.27)

Honduras,Deaths,85~90,2122.86(1928.21to2337.15)

Honduras,Deaths,90~95,3798.56(3384.85to4262.83)

Honduras,DALYs,20~25,116.04(100.62to133.83)

Honduras,DALYs,25~30,130.88(114.95to149.03)

Honduras,DALYs,30~35,156.98(139.53to176.61)

Honduras,DALYs,35~40,195.82(176.19to217.64)

Honduras,DALYs,40~45,307.92(281.85to336.4)

Honduras,DALYs,45~50,504.43(467.8to543.93)

Honduras,DALYs,50~55,957.75(900.19to1018.99)

Honduras,DALYs,55~60,1675.87(1589.25to1767.21)

Honduras,DALYs,60~65,3025.53(2890.14to3167.27)

Honduras,DALYs,65~70,4584.75(4391.43to4786.58)

Honduras,DALYs,70~75,7466.17(7154.96to7790.92)

Honduras,DALYs,75~80,10434.46(9913.65to10982.63)

Honduras,DALYs,80~85,14019.55(13246.05to14838.21)

Honduras,DALYs,85~90,22295.21(20876.39to23810.46)

Honduras,DALYs,90~95,34253.32(31255.88to37538.2)

Lesotho,Prevalence,20~25,432.28(414.1to451.25)

Lesotho,Prevalence,25~30,597.12(574.59to620.53)

Lesotho,Prevalence,30~35,778.54(751.53to806.52)

Lesotho,Prevalence,35~40,965.22(934.04to997.44)

Lesotho,Prevalence,40~45,1282.5(1245.65to1320.43)

Lesotho,Prevalence,45~50,2168.03(2115.1to2222.29)

Lesotho,Prevalence,50~55,3628.78(3552.03to3707.2)

Lesotho,Prevalence,55~60,5238.72(5135.25to5344.27)

Lesotho,Prevalence,60~65,6912.87(6779.05to7049.34)

Lesotho,Prevalence,65~70,9129.1(8951.77to9309.95)

Lesotho,Prevalence,70~75,11781.88(11538to12030.91)

Lesotho,Prevalence,75~80,14630.2(14260.78to15009.2)

Lesotho,Prevalence,80~85,17854.99(17312.11to18414.88)

Lesotho,Prevalence,85~90,20940.02(20069.14to21848.69)

Lesotho,Prevalence,90~95,23537.16(21887.61to25311.02)

Lesotho,Deaths,20~25,0.28(0.1to0.8)

Lesotho,Deaths,25~30,0.49(0.21to1.16)

Lesotho,Deaths,30~35,0.94(0.47to1.89)

Lesotho,Deaths,35~40,2.25(1.34to3.78)

Lesotho,Deaths,40~45,6.83(4.83to9.68)

Lesotho,Deaths,45~50,18.88(14.86to23.98)

Lesotho,Deaths,50~55,44.37(37.27to52.81)

Lesotho,Deaths,55~60,98.19(85.64to112.58)

Lesotho,Deaths,60~65,163.94(144.81to185.59)

Lesotho,Deaths,65~70,333.05(297.48to372.86)

Lesotho,Deaths,70~75,673.46(602.52to752.76)

Lesotho,Deaths,75~80,971.62(851.29to1108.97)

Lesotho,Deaths,80~85,1345.85(1161.55to1559.4)

Lesotho,Deaths,85~90,1755.41(1464.01to2104.81)

Lesotho,Deaths,90~95,2177.79(1651.31to2872.13)

Lesotho,DALYs,20~25,53.5(47.28to60.54)

Lesotho,DALYs,25~30,80.47(72.26to89.61)

Lesotho,DALYs,30~35,122.51(111.41to134.71)

Lesotho,DALYs,35~40,209.01(192.84to226.53)

Lesotho,DALYs,40~45,454.37(427.07to483.4)

Lesotho,DALYs,45~50,1026.49(979.25to1076.02)

Lesotho,DALYs,50~55,2060.31(1985.22to2138.23)

Lesotho,DALYs,55~60,3831.45(3712.83to3953.86)

Lesotho,DALYs,60~65,5459.62(5296.87to5627.37)

Lesotho,DALYs,65~70,9046.5(8786.61to9314.07)

Lesotho,DALYs,70~75,14632.76(14200.05to15078.66)

Lesotho,DALYs,75~80,16941.15(16319.22to17586.79)

Lesotho,DALYs,80~85,18527.84(17695.62to19399.21)

Lesotho,DALYs,85~90,19342.22(18122.66to20643.86)

Lesotho,DALYs,90~95,20753.86(18522.7to23253.77)

Cabo_Verde,Prevalence,20~25,335.83(300.45to375.37)

Cabo_Verde,Prevalence,25~30,436.4(394.63to482.6)

Cabo_Verde,Prevalence,30~35,555.84(506.73to609.71)

Cabo_Verde,Prevalence,35~40,692.32(634.09to755.9)

Cabo_Verde,Prevalence,40~45,878.83(809.95to953.56)

Cabo_Verde,Prevalence,45~50,1187.06(1099.46to1281.65)

Cabo_Verde,Prevalence,50~55,1615.66(1502.28to1737.6)

Cabo_Verde,Prevalence,55~60,2132.45(1990.88to2284.09)

Cabo_Verde,Prevalence,60~65,2755.02(2581.52to2940.19)

Cabo_Verde,Prevalence,65~70,3469.47(3252.11to3701.36)

Cabo_Verde,Prevalence,70~75,4344.98(4060.9to4648.94)

Cabo_Verde,Prevalence,75~80,5520.19(5115.08to5957.4)

Cabo_Verde,Prevalence,80~85,7086.45(6544.32to7673.48)

Cabo_Verde,Prevalence,85~90,9040.67(8270.94to9882.03)

Cabo_Verde,Prevalence,90~95,11291.01(10102.61to12619.2)

Cabo_Verde,Deaths,20~25,0.59(0.03to11.54)

Cabo_Verde,Deaths,25~30,2.08(0.39to11.18)

Cabo_Verde,Deaths,30~35,2.44(0.57to10.39)

Cabo_Verde,Deaths,35~40,2.11(0.48to9.23)

Cabo_Verde,Deaths,40~45,3.21(0.91to11.27)

Cabo_Verde,Deaths,45~50,7.15(2.73to18.75)

Cabo_Verde,Deaths,50~55,9.45(3.95to22.6)

Cabo_Verde,Deaths,55~60,13.72(6.47to29.11)

Cabo_Verde,Deaths,60~65,22.13(11.49to42.61)

Cabo_Verde,Deaths,65~70,44.82(25.32to79.33)

Cabo_Verde,Deaths,70~75,76.99(44.82to132.25)

Cabo_Verde,Deaths,75~80,104.62(57.65to189.85)

Cabo_Verde,Deaths,80~85,137.9(75.67to251.3)

Cabo_Verde,Deaths,85~90,217.09(117.91to399.7)

Cabo_Verde,Deaths,90~95,272.61(140.26to529.85)

Cabo_Verde,DALYs,20~25,91.5(72.08to116.15)

Cabo_Verde,DALYs,25~30,171.52(142.62to206.28)

Cabo_Verde,DALYs,30~35,198.31(167.74to234.45)

Cabo_Verde,DALYs,35~40,192.08(162.71to226.75)

Cabo_Verde,DALYs,40~45,252.02(216.87to292.87)

Cabo_Verde,DALYs,45~50,431.69(378.74to492.04)

Cabo_Verde,DALYs,50~55,533.82(471.47to604.42)

Cabo_Verde,DALYs,55~60,682.96(608.92to766.02)

Cabo_Verde,DALYs,60~65,926.27(832.78to1030.26)

Cabo_Verde,DALYs,65~70,1465.31(1327.51to1617.41)

Cabo_Verde,DALYs,70~75,2019.63(1831.5to2227.08)

Cabo_Verde,DALYs,75~80,2237.67(2004.45to2498.02)

Cabo_Verde,DALYs,80~85,2381.09(2122.37to2671.34)

Cabo_Verde,DALYs,85~90,2957(2612.31to3347.18)

Cabo_Verde,DALYs,90~95,3237.56(2784.15to3764.8)

Ecuador,Prevalence,20~25,318.01(309.14to327.13)

Ecuador,Prevalence,25~30,403.97(393.92to414.28)

Ecuador,Prevalence,30~35,511.43(499.88to523.25)

Ecuador,Prevalence,35~40,649.58(636.2to663.25)

Ecuador,Prevalence,40~45,859.72(843.94to875.79)

Ecuador,Prevalence,45~50,1252.37(1231.94to1273.15)

Ecuador,Prevalence,50~55,1893.74(1866.3to1921.57)

Ecuador,Prevalence,55~60,2841.21(2804.05to2878.87)

Ecuador,Prevalence,60~65,4165.63(4114.99to4216.9)

Ecuador,Prevalence,65~70,6013.52(5944.18to6083.67)

Ecuador,Prevalence,70~75,8864.28(8763.8to8965.91)

Ecuador,Prevalence,75~80,13270.98(13095.72to13448.58)

Ecuador,Prevalence,80~85,19703.3(19430.21to19980.24)

Ecuador,Prevalence,85~90,27919.69(27494.04to28351.93)

Ecuador,Prevalence,90~95,36198.04(35514.5to36894.74)

Ecuador,Deaths,20~25,1.11(0.73to1.7)

Ecuador,Deaths,25~30,1.2(0.82to1.76)

Ecuador,Deaths,30~35,1.1(0.76to1.58)

Ecuador,Deaths,35~40,1.31(0.95to1.82)

Ecuador,Deaths,40~45,1.82(1.38to2.41)

Ecuador,Deaths,45~50,2.63(2.07to3.34)

Ecuador,Deaths,50~55,4.23(3.48to5.14)

Ecuador,Deaths,55~60,6.11(5.15to7.24)

Ecuador,Deaths,60~65,13.5(11.8to15.44)

Ecuador,Deaths,65~70,23.42(20.79to26.38)

Ecuador,Deaths,70~75,52.58(47.15to58.63)

Ecuador,Deaths,75~80,100.85(89.42to113.74)

Ecuador,Deaths,80~85,210.34(186.58to237.11)

Ecuador,Deaths,85~90,440.87(390.67to497.52)

Ecuador,Deaths,90~95,864.98(763.58to979.86)

Ecuador,DALYs,20~25,79.89(71.33to89.48)

Ecuador,DALYs,25~30,85.51(77.07to94.87)

Ecuador,DALYs,30~35,81.58(73.84to90.13)

Ecuador,DALYs,35~40,93.39(85.2to102.37)

Ecuador,DALYs,40~45,119.48(110.15to129.6)

Ecuador,DALYs,45~50,161.24(149.87to173.46)

Ecuador,DALYs,50~55,234.3(219.77to249.8)

Ecuador,DALYs,55~60,312.98(295.21to331.82)

Ecuador,DALYs,60~65,548.72(521.47to577.4)

Ecuador,DALYs,65~70,798.5(761.13to837.7)

Ecuador,DALYs,70~75,1403.79(1340.93to1469.59)

Ecuador,DALYs,75~80,2144.94(2037.33to2258.24)

Ecuador,DALYs,80~85,3452.62(3276.59to3638.11)

Ecuador,DALYs,85~90,5631.61(5333.71to5946.16)

Ecuador,DALYs,90~95,9367.21(8826.79to9940.71)

Latvia,Prevalence,20~25,433.44(413.35to454.51)

Latvia,Prevalence,25~30,577.6(555.04to601.08)

Latvia,Prevalence,30~35,738(713.26to763.59)

Latvia,Prevalence,35~40,909.22(882.27to937)

Latvia,Prevalence,40~45,1164.06(1134.59to1194.29)

Latvia,Prevalence,45~50,1711.45(1674.48to1749.24)

Latvia,Prevalence,50~55,2570.94(2523.26to2619.52)

Latvia,Prevalence,55~60,3654.07(3592.83to3716.35)

Latvia,Prevalence,60~65,4944.71(4866.75to5023.92)

Latvia,Prevalence,65~70,6575.85(6475.26to6678.01)

Latvia,Prevalence,70~75,8715.44(8582.68to8850.26)

Latvia,Prevalence,75~80,11506.55(11311.63to11704.82)

Latvia,Prevalence,80~85,15160.84(14891.17to15435.38)

Latvia,Prevalence,85~90,19592.06(19200.95to19991.15)

Latvia,Prevalence,90~95,24444.63(23821.65to25083.91)

Latvia,Deaths,20~25,0.16(0.01to2.24)

Latvia,Deaths,25~30,0.23(0.03to1.62)

Latvia,Deaths,30~35,0.44(0.12to1.6)

Latvia,Deaths,35~40,0.75(0.3to1.86)

Latvia,Deaths,40~45,1.49(0.81to2.75)

Latvia,Deaths,45~50,2.87(1.85to4.45)

Latvia,Deaths,50~55,6.59(4.85to8.96)

Latvia,Deaths,55~60,12.47(9.81to15.84)

Latvia,Deaths,60~65,23.85(19.43to29.27)

Latvia,Deaths,65~70,34.36(28.39to41.59)

Latvia,Deaths,70~75,48.09(39.85to58.04)

Latvia,Deaths,75~80,63.06(51.25to77.59)

Latvia,Deaths,80~85,74.54(60.04to92.56)

Latvia,Deaths,85~90,81.49(64.1to103.59)

Latvia,Deaths,90~95,97.92(72.84to131.64)

Latvia,DALYs,20~25,33.45(28.38to39.43)

Latvia,DALYs,25~30,43.83(38.35to50.11)

Latvia,DALYs,30~35,60.24(54.12to67.05)

Latvia,DALYs,35~40,80.34(73.53to87.77)

Latvia,DALYs,40~45,120.34(112.33to128.91)

Latvia,DALYs,45~50,193.2(182.8to204.19)

Latvia,DALYs,50~55,354.26(339.1to370.09)

Latvia,DALYs,55~60,560.8(540.24to582.14)

Latvia,DALYs,60~65,883.06(853.57to913.57)

Latvia,DALYs,65~70,1094.35(1058.95to1130.93)

Latvia,DALYs,70~75,1302.89(1260.06to1347.17)

Latvia,DALYs,75~80,1450.04(1396.29to1505.87)

Latvia,DALYs,80~85,1496.46(1435.98to1559.49)

Latvia,DALYs,85~90,1495(1423.1to1570.55)

Latvia,DALYs,90~95,1665.93(1557.63to1781.76)

Republic_of_Moldova,Prevalence,20~25,611.14(584.86to638.6)

Republic_of_Moldova,Prevalence,25~30,814.54(785.2to844.98)

Republic_of_Moldova,Prevalence,30~35,1013.96(982.63to1046.28)

Republic_of_Moldova,Prevalence,35~40,1195.78(1162.92to1229.57)

Republic_of_Moldova,Prevalence,40~45,1492.7(1457.27to1528.98)

Republic_of_Moldova,Prevalence,45~50,2290.69(2244.02to2338.32)

Republic_of_Moldova,Prevalence,50~55,3489.29(3427.86to3551.82)

Republic_of_Moldova,Prevalence,55~60,4797.3(4720.12to4875.74)

Republic_of_Moldova,Prevalence,60~65,6208.97(6113.32to6306.12)

Republic_of_Moldova,Prevalence,65~70,8037.53(7915.44to8161.49)

Republic_of_Moldova,Prevalence,70~75,10355.43(10192.54to10520.93)

Republic_of_Moldova,Prevalence,75~80,12997.74(12767.56to13232.07)

Republic_of_Moldova,Prevalence,80~85,15898.48(15594.37to16208.53)

Republic_of_Moldova,Prevalence,85~90,18906.29(18478.06to19344.44)

Republic_of_Moldova,Prevalence,90~95,21859.02(21194.33to22544.55)

Republic_of_Moldova,Deaths,20~25,1.47(0.64to3.4)

Republic_of_Moldova,Deaths,25~30,1.8(0.93to3.49)

Republic_of_Moldova,Deaths,30~35,2.77(1.72to4.46)

Republic_of_Moldova,Deaths,35~40,4.39(3.13to6.17)

Republic_of_Moldova,Deaths,40~45,7.77(6.15to9.82)

Republic_of_Moldova,Deaths,45~50,13.06(10.94to15.61)

Republic_of_Moldova,Deaths,50~55,21.13(18.38to24.31)

Republic_of_Moldova,Deaths,55~60,30.86(27.4to34.75)

Republic_of_Moldova,Deaths,60~65,46.85(42.09to52.14)

Republic_of_Moldova,Deaths,65~70,59.63(53.69to66.23)

Republic_of_Moldova,Deaths,70~75,74.41(66.94to82.7)

Republic_of_Moldova,Deaths,75~80,94.14(84.12to105.35)

Republic_of_Moldova,Deaths,80~85,108.72(96.65to122.3)

Republic_of_Moldova,Deaths,85~90,102.25(89.52to116.77)

Republic_of_Moldova,Deaths,90~95,83.86(70.4to99.88)

Republic_of_Moldova,DALYs,20~25,133.82(109.75to163.17)

Republic_of_Moldova,DALYs,25~30,161.21(136.89to189.84)

Republic_of_Moldova,DALYs,30~35,213.65(187.72to243.16)

Republic_of_Moldova,DALYs,35~40,286.68(258.63to317.78)

Republic_of_Moldova,DALYs,40~45,432.09(399.96to466.8)

Republic_of_Moldova,DALYs,45~50,655.87(615.68to698.67)

Republic_of_Moldova,DALYs,50~55,945.45(896.36to997.23)

Republic_of_Moldova,DALYs,55~60,1221.53(1164.3to1281.58)

Republic_of_Moldova,DALYs,60~65,1603.85(1533.05to1677.91)

Republic_of_Moldova,DALYs,65~70,1774.25(1695.22to1856.96)

Republic_of_Moldova,DALYs,70~75,1889.44(1801.57to1981.6)

Republic_of_Moldova,DALYs,75~80,1978.57(1877to2085.63)

Republic_of_Moldova,DALYs,80~85,1876.21(1769.67to1989.16)

Republic_of_Moldova,DALYs,85~90,1512.64(1405.26to1628.24)

Republic_of_Moldova,DALYs,90~95,1180.05(1056.21to1318.42)

Nicaragua,Prevalence,20~25,292.27(282.79to302.08)

Nicaragua,Prevalence,25~30,385.22(373.96to396.81)

Nicaragua,Prevalence,30~35,503.98(490.5to517.84)

Nicaragua,Prevalence,35~40,652.84(636.78to669.31)

Nicaragua,Prevalence,40~45,889.95(870.28to910.07)

Nicaragua,Prevalence,45~50,1403.54(1376.06to1431.57)

Nicaragua,Prevalence,50~55,2303.17(2263.51to2343.53)

Nicaragua,Prevalence,55~60,3623.27(3567.17to3680.26)

Nicaragua,Prevalence,60~65,5439.4(5361.05to5518.89)

Nicaragua,Prevalence,65~70,8132.31(8020.45to8245.72)

Nicaragua,Prevalence,70~75,12401.87(12233.47to12572.59)

Nicaragua,Prevalence,75~80,18714.15(18416.46to19016.66)

Nicaragua,Prevalence,80~85,27305.43(26846.23to27772.48)

Nicaragua,Prevalence,85~90,38122.63(37413.03to38845.69)

Nicaragua,Prevalence,90~95,49657.18(48494.22to50848.04)

Nicaragua,Deaths,20~25,0.25(0.1to0.66)

Nicaragua,Deaths,25~30,0.35(0.15to0.8)

Nicaragua,Deaths,30~35,0.53(0.26to1.06)

Nicaragua,Deaths,35~40,0.88(0.49to1.56)

Nicaragua,Deaths,40~45,1.47(0.92to2.35)

Nicaragua,Deaths,45~50,2.93(2.03to4.21)

Nicaragua,Deaths,50~55,6.12(4.61to8.12)

Nicaragua,Deaths,55~60,12.56(10.03to15.71)

Nicaragua,Deaths,60~65,28.03(23.4to33.57)

Nicaragua,Deaths,65~70,53.02(45.12to62.29)

Nicaragua,Deaths,70~75,106.02(91.09to123.39)

Nicaragua,Deaths,75~80,188.85(158.79to224.59)

Nicaragua,Deaths,80~85,331.94(278.04to396.3)

Nicaragua,Deaths,85~90,595.18(495.04to715.57)

Nicaragua,Deaths,90~95,1010.77(828to1233.9)

Nicaragua,DALYs,20~25,31.39(27.93to35.28)

Nicaragua,DALYs,25~30,40.63(36.58to45.14)

Nicaragua,DALYs,30~35,54.75(49.85to60.13)

Nicaragua,DALYs,35~40,76.97(70.8to83.67)

Nicaragua,DALYs,40~45,111.38(103.59to119.74)

Nicaragua,DALYs,45~50,189.06(177.79to201.03)

Nicaragua,DALYs,50~55,334.87(317.97to352.67)

Nicaragua,DALYs,55~60,575.65(550.6to601.83)

Nicaragua,DALYs,60~65,1036.08(996.56to1077.15)

Nicaragua,DALYs,65~70,1616.44(1558.06to1677)

Nicaragua,DALYs,70~75,2603.44(2511.04to2699.24)

Nicaragua,DALYs,75~80,3723.31(3569.96to3883.24)

Nicaragua,DALYs,80~85,5147.5(4923.71to5381.46)

Nicaragua,DALYs,85~90,7289.79(6943.15to7653.73)

Nicaragua,DALYs,90~95,10571.14(9974.41to11203.58)

Venezuela_(Bolivarian_Republic_of),Prevalence,20~25,250.69(246.87to254.58)

Venezuela_(Bolivarian_Republic_of),Prevalence,25~30,345.51(340.95to350.13)

Venezuela_(Bolivarian_Republic_of),Prevalence,30~35,469.79(464.32to475.32)

Venezuela_(Bolivarian_Republic_of),Prevalence,35~40,626.58(620.05to633.19)

Venezuela_(Bolivarian_Republic_of),Prevalence,40~45,878.73(870.68to886.85)

Venezuela_(Bolivarian_Republic_of),Prevalence,45~50,1444.64(1433.15to1456.23)

Venezuela_(Bolivarian_Republic_of),Prevalence,50~55,2412.43(2395.76to2429.21)

Venezuela_(Bolivarian_Republic_of),Prevalence,55~60,3735.3(3712.04to3758.7)

Venezuela_(Bolivarian_Republic_of),Prevalence,60~65,5436.37(5404.9to5468.02)

Venezuela_(Bolivarian_Republic_of),Prevalence,65~70,7751.31(7707.83to7795.03)

Venezuela_(Bolivarian_Republic_of),Prevalence,70~75,11203(11139.15to11267.21)

Venezuela_(Bolivarian_Republic_of),Prevalence,75~80,16406.36(16294.51to16518.97)

Venezuela_(Bolivarian_Republic_of),Prevalence,80~85,24056.33(23880.48to24233.48)

Venezuela_(Bolivarian_Republic_of),Prevalence,85~90,34334.68(34055.99to34615.65)

Venezuela_(Bolivarian_Republic_of),Prevalence,90~95,46568.11(46122.07to47018.47)

Venezuela_(Bolivarian_Republic_of),Deaths,20~25,0.33(0.23to0.49)

Venezuela_(Bolivarian_Republic_of),Deaths,25~30,0.41(0.29to0.56)

Venezuela_(Bolivarian_Republic_of),Deaths,30~35,0.55(0.41to0.72)

Venezuela_(Bolivarian_Republic_of),Deaths,35~40,0.84(0.67to1.06)

Venezuela_(Bolivarian_Republic_of),Deaths,40~45,1.6(1.35to1.91)

Venezuela_(Bolivarian_Republic_of),Deaths,45~50,3.66(3.21to4.16)

Venezuela_(Bolivarian_Republic_of),Deaths,50~55,8.51(7.74to9.37)

Venezuela_(Bolivarian_Republic_of),Deaths,55~60,17.04(15.78to18.41)

Venezuela_(Bolivarian_Republic_of),Deaths,60~65,34.77(32.63to37.05)

Venezuela_(Bolivarian_Republic_of),Deaths,65~70,62.51(59.07to66.15)

Venezuela_(Bolivarian_Republic_of),Deaths,70~75,121.15(114.94to127.69)

Venezuela_(Bolivarian_Republic_of),Deaths,75~80,204.17(191.81to217.34)

Venezuela_(Bolivarian_Republic_of),Deaths,80~85,352.86(330.86to376.32)

Venezuela_(Bolivarian_Republic_of),Deaths,85~90,566.66(529.53to606.38)

Venezuela_(Bolivarian_Republic_of),Deaths,90~95,799.26(741.88to861.08)

Venezuela_(Bolivarian_Republic_of),DALYs,20~25,34.46(31.7to37.46)

Venezuela_(Bolivarian_Republic_of),DALYs,25~30,42.07(39.1to45.27)

Venezuela_(Bolivarian_Republic_of),DALYs,30~35,53.79(50.42to57.39)

Venezuela_(Bolivarian_Republic_of),DALYs,35~40,73.5(69.45to77.78)

Venezuela_(Bolivarian_Republic_of),DALYs,40~45,116.4(111.03to122.03)

Venezuela_(Bolivarian_Republic_of),DALYs,45~50,220.42(212.16to228.99)

Venezuela_(Bolivarian_Republic_of),DALYs,50~55,428.88(415.77to442.41)

Venezuela_(Bolivarian_Republic_of),DALYs,55~60,728.95(709.65to748.78)

Venezuela_(Bolivarian_Republic_of),DALYs,60~65,1231.99(1202.97to1261.7)

Venezuela_(Bolivarian_Republic_of),DALYs,65~70,1843.06(1801.97to1885.08)

Venezuela_(Bolivarian_Republic_of),DALYs,70~75,2889.37(2826.23to2953.92)

Venezuela_(Bolivarian_Republic_of),DALYs,75~80,3927.97(3824.28to4034.47)

Venezuela_(Bolivarian_Republic_of),DALYs,80~85,5355.97(5204.41to5511.94)

Venezuela_(Bolivarian_Republic_of),DALYs,85~90,6912.96(6694.05to7139.02)

Venezuela_(Bolivarian_Republic_of),DALYs,90~95,8593.72(8266.86to8933.51)

South_Africa,Prevalence,20~25,512.21(498.32to526.48)

South_Africa,Prevalence,25~30,694.42(677.96to711.27)

South_Africa,Prevalence,30~35,879.97(860.93to899.45)

South_Africa,Prevalence,35~40,1062.31(1040.92to1084.14)

South_Africa,Prevalence,40~45,1352.81(1328.11to1377.96)

South_Africa,Prevalence,45~50,2103.22(2069.32to2137.68)

South_Africa,Prevalence,50~55,3330.84(3283.38to3379)

South_Africa,Prevalence,55~60,4788.73(4725.43to4852.88)

South_Africa,Prevalence,60~65,6438.33(6356.92to6520.79)

South_Africa,Prevalence,65~70,8659.18(8549.92to8769.83)

South_Africa,Prevalence,70~75,11488.1(11338.44to11639.75)

South_Africa,Prevalence,75~80,15015.45(14784.13to15250.39)

South_Africa,Prevalence,80~85,19368.17(19042.54to19699.36)

South_Africa,Prevalence,85~90,24137.07(23650.33to24633.82)

South_Africa,Prevalence,90~95,28650.34(27790.39to29536.9)

South_Africa,Deaths,20~25,0.76(0.56to1.02)

South_Africa,Deaths,25~30,1.39(1.13to1.73)

South_Africa,Deaths,30~35,2.27(1.91to2.69)

South_Africa,Deaths,35~40,3.21(2.77to3.71)

South_Africa,Deaths,40~45,5.51(4.9to6.18)

South_Africa,Deaths,45~50,10.5(9.59to11.51)

South_Africa,Deaths,50~55,22.47(20.93to24.11)

South_Africa,Deaths,55~60,46.98(44.4to49.7)

South_Africa,Deaths,60~65,72.71(69.09to76.52)

South_Africa,Deaths,65~70,140.86(134.49to147.54)

South_Africa,Deaths,70~75,261.34(249.89to273.32)

South_Africa,Deaths,75~80,362.66(344.21to382.1)

South_Africa,Deaths,80~85,595.77(564.62to628.63)

South_Africa,Deaths,85~90,830.29(782.71to880.77)

South_Africa,Deaths,90~95,1079.72(1001.59to1163.94)

South_Africa,DALYs,20~25,113.15(98.89to129.46)

South_Africa,DALYs,25~30,167.74(150.18to187.35)

South_Africa,DALYs,30~35,229.62(208.44to252.96)

South_Africa,DALYs,35~40,287.54(263.44to313.84)

South_Africa,DALYs,40~45,411.98(382.06to444.23)

South_Africa,DALYs,45~50,679.47(637.22to724.53)

South_Africa,DALYs,50~55,1214.36(1150.39to1281.88)

South_Africa,DALYs,55~60,2080.18(1984.82to2180.13)

South_Africa,DALYs,60~65,2764.18(2642.93to2890.99)

South_Africa,DALYs,65~70,4301.51(4119.56to4491.49)

South_Africa,DALYs,70~75,6366.64(6094.14to6651.32)

South_Africa,DALYs,75~80,7228.59(6857.14to7620.17)

South_Africa,DALYs,80~85,9257.99(8741.64to9804.85)

South_Africa,DALYs,85~90,10494.94(9787.29to11253.76)

South_Africa,DALYs,90~95,11992.8(10804.91to13311.28)

Romania,Prevalence,20~25,442.18(432.4to452.18)

Romania,Prevalence,25~30,597.65(586.33to609.18)

Romania,Prevalence,30~35,772.66(760.18to785.35)

Romania,Prevalence,35~40,954.86(941.36to968.55)

Romania,Prevalence,40~45,1270.38(1255.37to1285.57)

Romania,Prevalence,45~50,2128.62(2108to2149.45)

Romania,Prevalence,50~55,3475.97(3447.94to3504.23)

Romania,Prevalence,55~60,5011.99(4976.07to5048.17)

Romania,Prevalence,60~65,6771.04(6726.22to6816.17)

Romania,Prevalence,65~70,9315.51(9256.44to9374.95)

Romania,Prevalence,70~75,12808.46(12727.25to12890.18)

Romania,Prevalence,75~80,17034.52(16912.5to17157.43)

Romania,Prevalence,80~85,21693.49(21528.95to21859.3)

Romania,Prevalence,85~90,26404.19(26171.32to26639.13)

Romania,Prevalence,90~95,30775.17(30391.86to31163.32)

Romania,Deaths,20~25,0.4(0.26to0.62)

Romania,Deaths,25~30,0.55(0.39to0.77)

Romania,Deaths,30~35,0.94(0.74to1.21)

Romania,Deaths,35~40,1.96(1.66to2.32)

Romania,Deaths,40~45,4.44(3.97to4.95)

Romania,Deaths,45~50,9.56(8.84to10.34)

Romania,Deaths,50~55,17.36(16.33to18.45)

Romania,Deaths,55~60,27.8(26.42to29.25)

Romania,Deaths,60~65,45.04(43.08to47.1)

Romania,Deaths,65~70,62.62(60.05to65.31)

Romania,Deaths,70~75,89.55(85.96to93.29)

Romania,Deaths,75~80,124.55(119.16to130.19)

Romania,Deaths,80~85,162.62(155.44to170.13)

Romania,Deaths,85~90,211.83(201.84to222.31)

Romania,Deaths,90~95,230.41(217.16to244.48)

Romania,DALYs,20~25,52.1(46to59.01)

Romania,DALYs,25~30,67.28(60.55to74.76)

Romania,DALYs,30~35,95(87.37to103.31)

Romania,DALYs,35~40,151.14(141.59to161.33)

Romania,DALYs,40~45,272.85(260.15to286.17)

Romania,DALYs,45~50,512.89(494.26to532.23)

Romania,DALYs,50~55,828.27(803.26to854.05)

Romania,DALYs,55~60,1161.73(1130.46to1193.85)

Romania,DALYs,60~65,1606.48(1566.69to1647.28)

Romania,DALYs,65~70,1931.48(1884.95to1979.17)

Romania,DALYs,70~75,2332.09(2275.36to2390.24)

Romania,DALYs,75~80,2666.5(2594.22to2740.8)

Romania,DALYs,80~85,2821.79(2739.91to2906.13)

Romania,DALYs,85~90,2992.68(2891.2to3097.73)

Romania,DALYs,90~95,2921.51(2780.43to3069.74)

Slovakia,Prevalence,20~25,351.09(341.92to360.51)

Slovakia,Prevalence,25~30,474.17(463.75to484.81)

Slovakia,Prevalence,30~35,621.15(609.45to633.08)

Slovakia,Prevalence,35~40,789.94(777to803.09)

Slovakia,Prevalence,40~45,1063.65(1048.9to1078.62)

Slovakia,Prevalence,45~50,1713.04(1693.2to1733.11)

Slovakia,Prevalence,50~55,2797.77(2770.32to2825.49)

Slovakia,Prevalence,55~60,4213.3(4176.78to4250.13)

Slovakia,Prevalence,60~65,5980.55(5932.78to6028.7)

Slovakia,Prevalence,65~70,8369.59(8305.65to8434.03)

Slovakia,Prevalence,70~75,11642.94(11553.51to11733.06)

Slovakia,Prevalence,75~80,15828.36(15687.02to15970.97)

Slovakia,Prevalence,80~85,20909.93(20709.27to21112.54)

Slovakia,Prevalence,85~90,26730.85(26436.59to27028.39)

Slovakia,Prevalence,90~95,33149.27(32667.6to33638.03)

Slovakia,Deaths,20~25,0.15(0.03to0.71)

Slovakia,Deaths,25~30,0.18(0.05to0.65)

Slovakia,Deaths,30~35,0.32(0.13to0.78)

Slovakia,Deaths,35~40,0.66(0.36to1.19)

Slovakia,Deaths,40~45,1.51(1.03to2.21)

Slovakia,Deaths,45~50,3.52(2.72to4.54)

Slovakia,Deaths,50~55,7.4(6.14to8.92)

Slovakia,Deaths,55~60,12.99(11.18to15.09)

Slovakia,Deaths,60~65,24.96(22.06to28.25)

Slovakia,Deaths,65~70,38.84(34.64to43.55)

Slovakia,Deaths,70~75,62.02(55.52to69.29)

Slovakia,Deaths,75~80,95.03(83.91to107.61)

Slovakia,Deaths,80~85,139.91(123.08to159.04)

Slovakia,Deaths,85~90,187.6(163.42to215.36)

Slovakia,Deaths,90~95,226.73(191.7to268.16)

Slovakia,DALYs,20~25,30.72(27.83to33.91)

Slovakia,DALYs,25~30,39(35.9to42.36)

Slovakia,DALYs,30~35,52.74(49.27to56.46)

Slovakia,DALYs,35~40,75.75(71.7to80.02)

Slovakia,DALYs,40~45,123.57(118.49to128.87)

Slovakia,DALYs,45~50,231.03(223.7to238.6)

Slovakia,DALYs,50~55,412.1(401.54to422.93)

Slovakia,DALYs,55~60,628.03(614.11to642.27)

Slovakia,DALYs,60~65,992.63(973.02to1012.63)

Slovakia,DALYs,65~70,1319.14(1293.95to1344.83)

Slovakia,DALYs,70~75,1748.46(1714.77to1782.81)

Slovakia,DALYs,75~80,2181.3(2132.66to2231.05)

Slovakia,DALYs,80~85,2589.78(2527.03to2654.1)

Slovakia,DALYs,85~90,2884.21(2802.19to2968.63)

Slovakia,DALYs,90~95,3157.04(3033.49to3285.63)

Ukraine,Prevalence,20~25,582.33(550.38to616.13)

Ukraine,Prevalence,25~30,788.27(752.14to826.14)

Ukraine,Prevalence,30~35,988.95(949.91to1029.6)

Ukraine,Prevalence,35~40,1174.32(1133.22to1216.91)

Ukraine,Prevalence,40~45,1442.73(1398.95to1487.89)

Ukraine,Prevalence,45~50,2092.62(2038.45to2148.24)

Ukraine,Prevalence,50~55,3157.41(3088.52to3227.84)

Ukraine,Prevalence,55~60,4427.71(4340to4517.19)

Ukraine,Prevalence,60~65,5829.38(5720.74to5940.09)

Ukraine,Prevalence,65~70,7672.08(7531.91to7814.87)

Ukraine,Prevalence,70~75,10006.82(9822.26to10194.85)

Ukraine,Prevalence,75~80,12644.27(12385.08to12908.9)

Ukraine,Prevalence,80~85,15524.07(15186.87to15868.75)

Ukraine,Prevalence,85~90,18338.82(17866.06to18824.08)

Ukraine,Prevalence,90~95,20786.88(20022.45to21580.49)

Ukraine,Deaths,20~25,2.64(1.68to4.15)

Ukraine,Deaths,25~30,3.46(2.46to4.86)

Ukraine,Deaths,30~35,5.36(4.23to6.8)

Ukraine,Deaths,35~40,6.82(5.68to8.18)

Ukraine,Deaths,40~45,10.52(9.24to11.96)

Ukraine,Deaths,45~50,15.15(13.74to16.71)

Ukraine,Deaths,50~55,21.36(19.77to23.06)

Ukraine,Deaths,55~60,30.4(28.4to32.54)

Ukraine,Deaths,60~65,39.33(36.89to41.94)

Ukraine,Deaths,65~70,44.99(42.23to47.94)

Ukraine,Deaths,70~75,48.47(45.45to51.68)

Ukraine,Deaths,75~80,50.99(47.64to54.58)

Ukraine,Deaths,80~85,54.46(50.73to58.46)

Ukraine,Deaths,85~90,58.53(54.13to63.29)

Ukraine,Deaths,90~95,59.49(53.92to65.65)

Ukraine,DALYs,20~25,206.18(168.56to252.19)

Ukraine,DALYs,25~30,259.32(220.71to304.69)

Ukraine,DALYs,30~35,350.9(309.79to397.47)

Ukraine,DALYs,35~40,409.04(369.02to453.39)

Ukraine,DALYs,40~45,558.18(515.9to603.93)

Ukraine,DALYs,45~50,742.29(696.6to790.97)

Ukraine,DALYs,50~55,963.01(912.96to1015.81)

Ukraine,DALYs,55~60,1232.94(1173.42to1295.48)

Ukraine,DALYs,60~65,1419.7(1353.3to1489.37)

Ukraine,DALYs,65~70,1431.9(1363.93to1503.26)

Ukraine,DALYs,70~75,1338.84(1272.53to1408.61)

Ukraine,DALYs,75~80,1203.77(1138.18to1273.13)

Ukraine,DALYs,80~85,1080.75(1016.37to1149.21)

Ukraine,DALYs,85~90,977.86(907.48to1053.7)

Ukraine,DALYs,90~95,901.78(808.4to1005.95)

Côte_d'Ivoire,Prevalence,20~25,446.41(438.95to454.01)

Côte_d'Ivoire,Prevalence,25~30,603.25(594.17to612.47)

Côte_d'Ivoire,Prevalence,30~35,776.27(765.37to787.34)

Côte_d'Ivoire,Prevalence,35~40,953.89(941.08to966.86)

Côte_d'Ivoire,Prevalence,40~45,1207.42(1192.3to1222.73)

Côte_d'Ivoire,Prevalence,45~50,1751.12(1730.7to1771.79)

Côte_d'Ivoire,Prevalence,50~55,2601.03(2572.61to2629.77)

Côte_d'Ivoire,Prevalence,55~60,3616.62(3578.61to3655.02)

Côte_d'Ivoire,Prevalence,60~65,4773.91(4724.25to4824.1)

Côte_d'Ivoire,Prevalence,65~70,6033.08(5968.32to6098.55)

Côte_d'Ivoire,Prevalence,70~75,7586.31(7498.59to7675.06)

Côte_d'Ivoire,Prevalence,75~80,9582.35(9446.29to9720.37)

Côte_d'Ivoire,Prevalence,80~85,12162.05(11956.41to12371.23)

Côte_d'Ivoire,Prevalence,85~90,14995.96(14658.36to15341.34)

Côte_d'Ivoire,Prevalence,90~95,17564.4(16924.64to18228.35)

Côte_d'Ivoire,Deaths,20~25,0.31(0.2to0.48)

Côte_d'Ivoire,Deaths,25~30,1.09(0.83to1.43)

Côte_d'Ivoire,Deaths,30~35,1.67(1.33to2.11)

Côte_d'Ivoire,Deaths,35~40,1.77(1.42to2.22)

Côte_d'Ivoire,Deaths,40~45,3.85(3.24to4.57)

Côte_d'Ivoire,Deaths,45~50,10.07(8.87to11.43)

Côte_d'Ivoire,Deaths,50~55,18.26(16.42to20.3)

Côte_d'Ivoire,Deaths,55~60,28.5(25.93to31.32)

Côte_d'Ivoire,Deaths,60~65,53.91(49.64to58.55)

Côte_d'Ivoire,Deaths,65~70,96.2(89.14to103.81)

Côte_d'Ivoire,Deaths,70~75,164.81(152.91to177.64)

Côte_d'Ivoire,Deaths,75~80,223.67(204.91to244.15)

Côte_d'Ivoire,Deaths,80~85,294.19(267.18to323.93)

Côte_d'Ivoire,Deaths,85~90,443.99(397.6to495.78)

Côte_d'Ivoire,Deaths,90~95,547.68(469.53to638.83)

Côte_d'Ivoire,DALYs,20~25,69.61(66.77to72.56)

Côte_d'Ivoire,DALYs,25~30,129.88(125.56to134.35)

Côte_d'Ivoire,DALYs,30~35,175.03(169.75to180.48)

Côte_d'Ivoire,DALYs,35~40,191.97(186.35to197.77)

Côte_d'Ivoire,DALYs,40~45,302.45(294.68to310.42)

Côte_d'Ivoire,DALYs,45~50,595.88(583.07to608.97)

Côte_d'Ivoire,DALYs,50~55,940.78(922.54to959.38)

Côte_d'Ivoire,DALYs,55~60,1295.01(1271.28to1319.19)

Côte_d'Ivoire,DALYs,60~65,2006.75(1972.28to2041.82)

Côte_d'Ivoire,DALYs,65~70,2917.49(2868.38to2967.45)

Côte_d'Ivoire,DALYs,70~75,4035.73(3965.32to4107.38)

Côte_d'Ivoire,DALYs,75~80,4478.42(4382.98to4575.94)

Côte_d'Ivoire,DALYs,80~85,4757.81(4635.73to4883.11)

Côte_d'Ivoire,DALYs,85~90,5694.77(5504.86to5891.24)

Côte_d'Ivoire,DALYs,90~95,6127.26(5798.9to6474.22)

Ghana,Prevalence,20~25,416.93(411.48to422.46)

Ghana,Prevalence,25~30,568.78(562.13to575.51)

Ghana,Prevalence,30~35,739.21(731.24to747.26)

Ghana,Prevalence,35~40,916.89(907.62to926.25)

Ghana,Prevalence,40~45,1167.68(1156.85to1178.6)

Ghana,Prevalence,45~50,1691.6(1677.16to1706.16)

Ghana,Prevalence,50~55,2508.92(2489.05to2528.94)

Ghana,Prevalence,55~60,3508.46(3482.08to3535.03)

Ghana,Prevalence,60~65,4710.34(4676.11to4744.81)

Ghana,Prevalence,65~70,6116.04(6071.24to6161.16)

Ghana,Prevalence,70~75,7949.27(7887.21to8011.81)

Ghana,Prevalence,75~80,10357.32(10258.77to10456.81)

Ghana,Prevalence,80~85,13436.58(13286.65to13588.21)

Ghana,Prevalence,85~90,16942.69(16694.04to17195.05)

Ghana,Prevalence,90~95,20204.88(19727.19to20694.13)

Ghana,Deaths,20~25,0.17(0.11to0.28)

Ghana,Deaths,25~30,0.6(0.44to0.82)

Ghana,Deaths,30~35,0.92(0.71to1.2)

Ghana,Deaths,35~40,0.96(0.75to1.23)

Ghana,Deaths,40~45,1.86(1.52to2.28)

Ghana,Deaths,45~50,4.76(4.11to5.52)

Ghana,Deaths,50~55,9.14(8.09to10.33)

Ghana,Deaths,55~60,13.92(12.5to15.5)

Ghana,Deaths,60~65,28.6(26.13to31.31)

Ghana,Deaths,65~70,58.81(54.23to63.77)

Ghana,Deaths,70~75,104.77(96.72to113.5)

Ghana,Deaths,75~80,153.63(139.76to168.88)

Ghana,Deaths,80~85,218.31(196.83to242.14)

Ghana,Deaths,85~90,347.29(308.42to391.06)

Ghana,Deaths,90~95,445.91(378.06to525.96)

Ghana,DALYs,20~25,48.78(46.81to50.82)

Ghana,DALYs,25~30,87.92(84.98to90.96)

Ghana,DALYs,30~35,119.44(115.83to123.16)

Ghana,DALYs,35~40,133.79(129.94to137.74)

Ghana,DALYs,40~45,194.83(189.84to199.96)

Ghana,DALYs,45~50,358.7(350.86to366.71)

Ghana,DALYs,50~55,579.03(567.71to590.58)

Ghana,DALYs,55~60,787.65(773.28to802.3)

Ghana,DALYs,60~65,1255.87(1234.77to1277.32)

Ghana,DALYs,65~70,1987.81(1955.67to2020.47)

Ghana,DALYs,70~75,2821.58(2774.14to2869.83)

Ghana,DALYs,75~80,3390.06(3320.21to3461.37)

Ghana,DALYs,80~85,3916.72(3820.97to4014.88)

Ghana,DALYs,85~90,4913.54(4757.42to5074.79)

Ghana,DALYs,90~95,5526.13(5243.23to5824.29)

Zimbabwe,Prevalence,20~25,359.92(350.05to370.07)

Zimbabwe,Prevalence,25~30,482.82(470.7to495.26)

Zimbabwe,Prevalence,30~35,627.49(612.71to642.62)

Zimbabwe,Prevalence,35~40,792.65(774.95to810.76)

Zimbabwe,Prevalence,40~45,1043.88(1022.45to1065.76)

Zimbabwe,Prevalence,45~50,1589.92(1560.07to1620.35)

Zimbabwe,Prevalence,50~55,2473.84(2431.55to2516.87)

Zimbabwe,Prevalence,55~60,3628.81(3570.57to3688)

Zimbabwe,Prevalence,60~65,5010.57(4933.17to5089.18)

Zimbabwe,Prevalence,65~70,6656.91(6555.3to6760.09)

Zimbabwe,Prevalence,70~75,8693.75(8552.79to8837.05)

Zimbabwe,Prevalence,75~80,11177.29(10957.7to11401.28)

Zimbabwe,Prevalence,80~85,14002.66(13675.66to14337.48)

Zimbabwe,Prevalence,85~90,16959.17(16421.92to17514)

Zimbabwe,Prevalence,90~95,19930.5(18865.31to21055.83)

Zimbabwe,Deaths,20~25,0.14(0.08to0.26)

Zimbabwe,Deaths,25~30,0.24(0.14to0.39)

Zimbabwe,Deaths,30~35,0.41(0.27to0.62)

Zimbabwe,Deaths,35~40,0.89(0.64to1.24)

Zimbabwe,Deaths,40~45,2.47(1.94to3.14)

Zimbabwe,Deaths,45~50,5.99(5to7.18)

Zimbabwe,Deaths,50~55,13.07(11.38to15.01)

Zimbabwe,Deaths,55~60,27.02(24.16to30.22)

Zimbabwe,Deaths,60~65,50.15(45.49to55.3)

Zimbabwe,Deaths,65~70,109.94(100.91to119.77)

Zimbabwe,Deaths,70~75,240.84(221.79to261.53)

Zimbabwe,Deaths,75~80,407.07(370.15to447.68)

Zimbabwe,Deaths,80~85,589.2(531.58to653.05)

Zimbabwe,Deaths,85~90,762.23(673.93to862.1)

Zimbabwe,Deaths,90~95,866.61(717.84to1046.22)

Zimbabwe,DALYs,20~25,40.64(37.72to43.8)

Zimbabwe,DALYs,25~30,57.48(53.77to61.46)

Zimbabwe,DALYs,30~35,80.6(75.82to85.67)

Zimbabwe,DALYs,35~40,121.8(115.27to128.71)

Zimbabwe,DALYs,40~45,220.65(210.51to231.28)
[truncated: 813,463 more chars]
